# A single gene mutation underpins metabolic adaptation and acquisition of filamentous competence in the emerging fungal pathogen *Candida auris*

**DOI:** 10.1371/journal.ppat.1012362

**Published:** 2024-07-08

**Authors:** Yuchen Deng, Ming Xu, Shuaihu Li, Jian Bing, Qiushi Zheng, Guanghua Huang, Wanqing Liao, Weihua Pan, Li Tao

**Affiliations:** 1 State Key Laboratory of Genetic Engineering, School of Life Sciences, Department of Infectious Diseases, Huashan Hospital, Fudan University, Shanghai, China; 2 Department of Dermatology, Shanghai Key Laboratory of Molecular Medical Mycology, Second Affiliated Hospital of Naval Medical University, Shanghai, China; University of Michigan, UNITED STATES OF AMERICA

## Abstract

Filamentous cell growth is a vital property of fungal pathogens. The mechanisms of filamentation in the emerging multidrug-resistant fungal pathogen *Candida auris* are poorly understood. Here, we show that exposure of *C*. *auris* to glycerol triggers a rod-like filamentation-competent (RL-FC) phenotype, which forms elongated filamentous cells after a prolonged culture period. Whole-genome sequencing analysis reveals that all RL-FC isolates harbor a mutation in the C_2_H_2_ zinc finger transcription factor-encoding gene *GFC1* (Gfc1 variants). Deletion of *GFC1* leads to an RL-FC phenotype similar to that observed in Gfc1 variants. We further demonstrate that *GFC1* mutation causes enhanced fatty acid β-oxidation metabolism and thereby promotes RL-FC/filamentous growth. This regulation is achieved through a Multiple Carbon source Utilizer (Mcu1)-dependent mechanism. Interestingly, both the evolved RL-FC isolates and the *gfc1*Δ mutant exhibit an enhanced ability to colonize the skin. Our results reveal that glycerol-mediated *GFC1* mutations are beneficial during *C*. *auris* skin colonization and infection.

## Author summary

*Candida auris* is an emerging multidrug-resistant fungal pathogen that has rapidly spread worldwide in the past decade. This fungus can manifest filamentous morphology under certain conditions, such as passage through a mammalian host. Here, we demonstrate that exposure of *C*. *auris* to glycerol triggers a rod-like filamentation-competent (RL-FC) phenotype, resulting in elongated filamentous cells post a prolonged cultivation. Whole-genome sequencing analysis indicates that mutation of a C_2_H_2_ zinc finger transcription factor-encoding gene *GFC1* is responsible for the generation of RL-FC/filamentous phenotype and enhances ability of skin colonization. We further substantiate that Multiple Carbon source Utilizer (Mcu1)-mediated fatty acid β-oxidation metabolism contributes to Gfc1-suppressed filamentous growth. Our findings link a single gene mutation to metabolic adaptation and acquisition of filamentous competence in *C*. *auris*, shedding light on how adaptive traits emerge in pathogens to enhance colonization and infection of the host.

## Introduction

*Candida auris* is a recently emerging fungal pathogen, first isolated in Japan in 2009 from the ear discharge of a female patient [1,2]. In the past decade, infections with *C*. *auris* have become a global health threat and have attracted considerable attention. Based on data from the Centers for Disease Control and Prevention (CDC) (https://www.cdc.gov), *C*. *auris* has been isolated in over 40 countries across six continents [2]. As of 2021, 3270 clinical cases and 7413 screening cases of *C*. *auris* were reported in the United States alone [3]. CDC has continued to see an increase in case counts for 2022. From Jan to Dec, 2022, 2377 clinical cases were reported [4]. More importantly, the predilection of this fungus for long-term skin colonization and its environmental persistence has led to rapid and widespread transmission within and between healthcare facilities, thereby posing an imminent threat to patients [2,5,6].

Morphological plasticity is a common strategy adopted by pathogenic fungi to adapt to diverse host environments and to cause infections [7–11]. *Cryptococcus neoformans* and *Histoplasma capsulatum* are capable of altering their cellular forms to a filamentous phenotype in response to a changing environment [7–9]. *Saccharomyces cerevisiae* can alternate between unicellular yeast and multicellular pseudohyphal cell forms [10,11]. Phenotypic transitions have been considered a prominent feature of pathogenic *Candida* spp. Yeast-filament transition and white-opaque switching in *C*. *albicans*, *C*. *tropicalis*, and *C*. *dubliniensis* are the best examples and have been well investigated [12–18]. Different cell types differ not only in cellular morphologies, but also in biological and pathological features [14,19–24]. For example, filamentous cells are critical for host adherence, tissue invasion, and tissue damage, and are essential for initiating systemic infections, whereas yeast cells facilitate biofilm formation and are easily disseminated through the bloodstream [19].

In *C*. *albicans*, the yeast-filament transition is regulated by a variety of host environmental factors, such as physiological temperature (37°C), neutral pH, elevated levels of CO_2_, N-acetylglucosamine (GlcNAc) and serum [13,25–28]. In contrast, *C*. *auris* fails to develop filaments when exposed to these environmental signals [29]. It has long been thought that *C*. *auris* was unable to undergo filamentation [30,31]. Interestingly, we recently reported that unlike other *Candida* species, *C*. *auris* underwent filamentous growth after passage through a mammalian body [32]. Low temperature (<25°C) was found to facilitate filamentous growth, suggesting that filamentation of *C*. *auris* may occur at low-temperature niches, such as on the host skin where the temperature is lower than that inside the host body. Another study reported that treatment of *C*. *auris* cells with genotoxins that induce DNA damage or replication inhibition resulted in pseudohyphal-like cell formation [33]. Loss of function of heat shock protein 90 (Hsp90) or deletion of the DNA damage-induced long non-coding RNA DINOR resulted in a polarized filamentous growth morphology [34,35]. Taken together, these findings suggest that host environment stresses and DNA stability may be critical factors for *C*. *auris* filamentous growth. However, the detailed regulatory mechanisms are largely unknown.

A striking feature unique to *C*. *auris* is its rarity of isolating from the gastrointestinal tract, but efficiently colonizes the skin surface, a phenomenon that has been considered to be associated with its metabolic predilection [36,37]. One study reported that *C*. *auris* survived on human skin for several weeks, whereas *C*. *albicans* completely lost cell viability within one week [38]. Growth in synthetic sweat medium that supplemented human sweat fatty acids allowed *C*. *auris* to form a multilayer biofilm with a cellular burden 10-times greater than that formed by *C*. *albicans*. This finding indicated that high salinity and fatty acids may confer *C*. *auris* a metabolic advantage over *C*. *albicans*.

Adaptive evolution is a common biological process of pathogenic microorganisms contributing to improve host fitness and enhance pathogenicity. As a rapidly evolved human pathogen, *C*. *auris* exhibits a high degree of genetic and genomic heterogeneity among a wide range of clinical isolates [39]. *In vitro* experimental assays have demonstrated that environmental stresses drive the occurrence of genetic mutations in *C*. *auris* [2,40,41]. Our *in vivo* results revealed that passage through a mammalian body triggered filamentous growth of *C*. *auris*. In the present study, we set out to explore the evolutionary mechanism of filamentation in *C*. *auris* [32]. Through a carbon source screening assay, we found that exposure of *C*. *auris* to glycerol triggered the generation of a novel RL-FC phenotype. WGS and Sanger sequencing analysis indicated that all evolved RL-FC isolates carried a mutation in the *GFC1* open reading frame (ORF) region, which led to filamentous growth and increased skin fitness. Deletion of *GFC1* resulted in an RL-FC phenotype similar to that observed in Gfc1 variants. Furthermore, we demonstrated that Gfc1 mutation caused enhanced fatty acid β-oxidation metabolism and thereby promoted RL-FC/filamentous growth and skin colonization. This was achieved through the metabolic changes mediated by Mcu1 and by the regulation of two regulators Ume6 and Hgc1. Therefore, our results reveal that glycerol-induced *GFC1* mutations may be beneficial during *C*. *auris* skin colonization and infection. Considering that glycerol is widely used in our daily life, we propose that it could lead to genetic heterogeneity of this fungus, promoting adaptive evolution and thereby enhancing host colonization and transmission.

## Results

### Glycerol induces a rod-like phenotype and filamentation in *C*. *auris*

**Fig 1:**
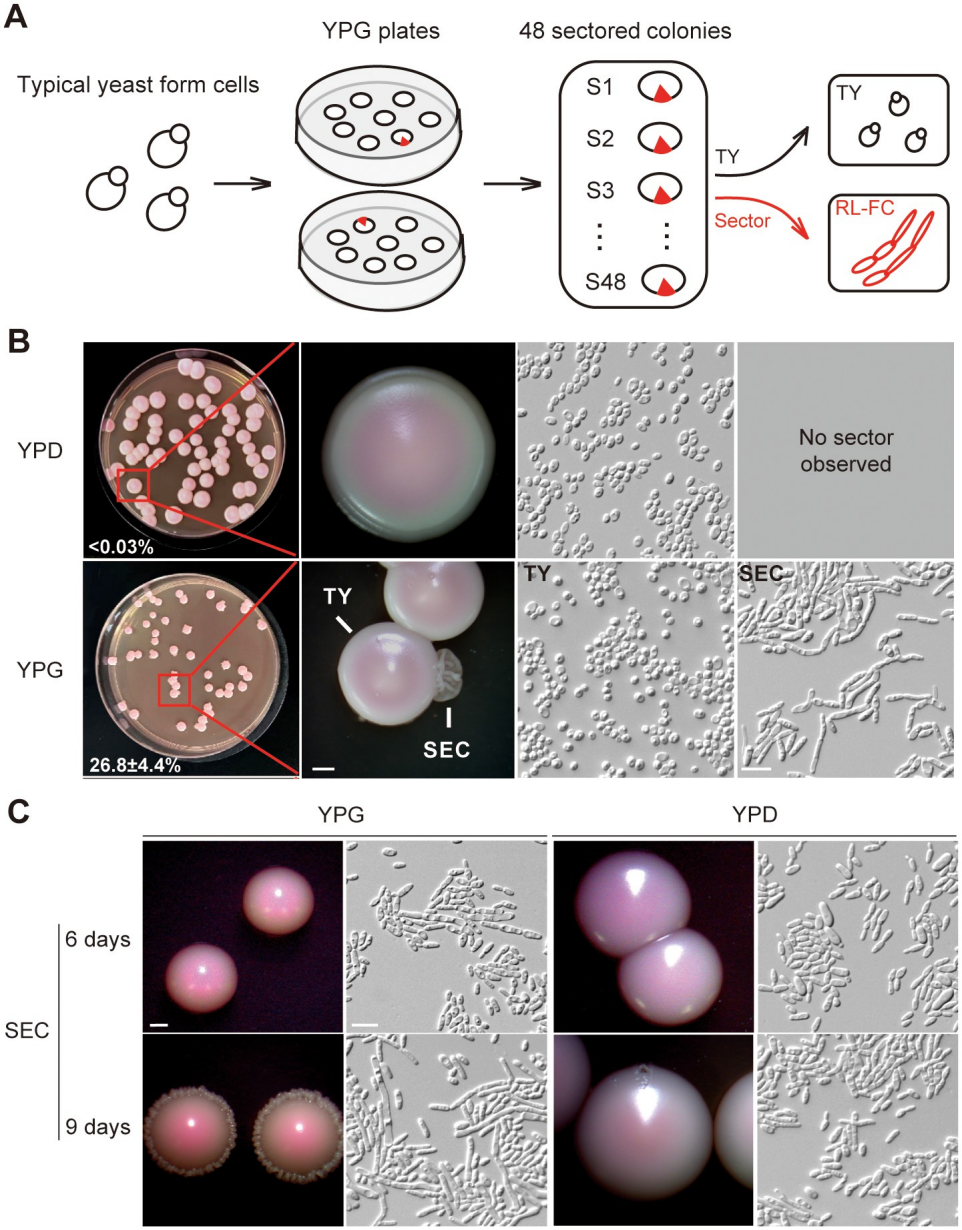
**Glycerol induces rod-like phenotype and filamentation in *C*. *auris*** (A) Schematic representation of the screening strategy. *C*. *auris* typical yeast cells were plated on YPG medium and grown at 25°C for 11 days. A total of 48 wrinkled sectored colonies that contain rod-like/elongated cells were observed. (B) Data for a YPG medium plate containing the wrinkled sectored colonies. Percentage of sectored colonies are indicated. Colony and cellular morphologies of TY and rod-like/elongated cells were shown. No sectored colonies were observed on YPD medium. TY: Typical yeast; SEC: Filament-sector; RL-FC: Rod-like filamentation-competent. Scale bar for colonies, 1 mm; Scale bar for cells, 10 μm. (C) Colony and cellular morphologies of filament-sector replated on YPG and YPD media for 6 or 9 days of growth at 25°C. Scale bar for colonies, 1 mm; Scale bar for cells, 10 μm. SEC: Filament-sector. The strain used was BJCA001 (WT).

We previously found that *C*. *auris* can undergo filamentous growth after passage through a mammalian body [32]. The filamentous cells exhibited significantly different expression patterns of carbon metabolism-related genes compared with the yeast cells. Therefore, we first designed a carbon source screening experiment to examine the effects of carbon sources on *C*. *auris* filament formation. The *C*. *auris* strain we used was the previously reported “typical yeast” (TY) cell form (BJCA001) that is considered unable to form filaments *in vitro* [32] (Fig 1A). We cultured *C*. *auris* cells on YP media (1% yeast extract, 2% peptone, w/v) containing different carbon sources (2%, w/v). As shown in Fig 1B, when the TY cells were cultured on YPD medium (1% yeast extract, 2% peptone, 2% glucose, w/v), they exclusively grew in the round yeast form and formed smooth colonies, which was consistent with our previous report. However, when the TY cells were grown on YP plus glycerol medium, several highly wrinkled sectored colonies (26.8±4.4%, a total of 48 individual sectored colonies) containing rod-like/elongated cells were observed (Fig 1A and 1B). No wrinkled or sectored colonies (<0.03%) were observed on media containing sucrose, GlcNAc, mannitol, pyruvate, citric acid, or acetic acid as a sole carbon source (S1 Fig). Hereafter, we referred to YP plus glycerol medium as YPG (1% yeast extract, 2% peptone, 2% glycerol, w/v). These results indicate that glycerol exclusively drives the morphological transition of *C*. *auris* from typical yeast cells to rod-like/elongated cells, which results in the formation of wrinkled colonies.

To test whether the glycerol-induced phenotype was heritable or not, we grew *C*. *auris* cells derived from the sectored colonies on either YPG or YPD media and cultured them at 25°C for 6 or 9 days. We found that on YPD medium at 25°C, the sector-derived cells always maintained their rod-like phenotype. On YPG medium, the cells exhibited a rod-like phenotype after 6 days of incubation at 25°C, while around 30% of rod-like cells converted to elongated filamentous cells after further incubation for three additional days. Most of the colonies grown on YPG medium exhibited wrinkled edges after prolonged incubation (Fig 1C). All the cells derived from glycerol-induced sectors were morphologically indistinguishable from the original round TY cells. These results indicate that the glycerol-induced rod-like phenotype and filament formation are heritably maintained. Thus, hereafter we referred to this phenotype as the “rod-like filamentation-competent (RL-FC)” phenotype.

### Glycerol-induced *GFC1* variants confer enhanced filamentation competence and skin fitness to *C*. *auris*

**Fig 2:**
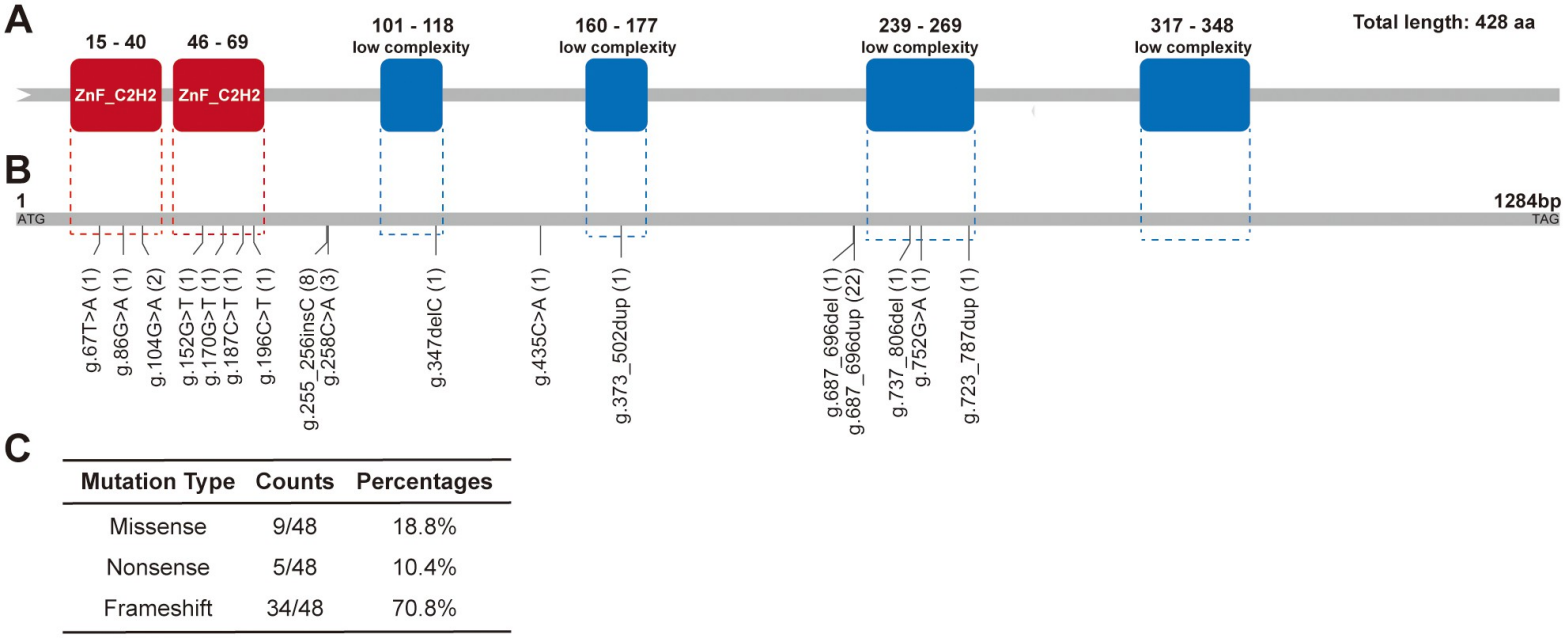
All evolved RL-FC mutants carry a mutation in the transcription factor-encoding gene *GFC1*. We obtained 48 independently-evolved RL-FC isolates from YPG medium and performed whole-genome sequencing (WGS) and Sanger sequencing analysis. Missense, nonsense, or frameshift mutations were identified in all evolved isolates. aa: Amino acids. (A) Schematic diagram of *C*. *aruis* Gfc1 protein. The protein is 428 amino acids long and comprised of two ZnF-C_2_H_2_ domains and four low complexity domains, which were denoted by red and blue boxes, respectively. ZnF-C_2_H_2_, C_2_H_2_ zinc-finger domain (15–40 aa and 46–69 aa). Low compositional complexity (101–118 aa, 160–177 aa, 239–269 aa, and 317–348 aa). Domains of Gfc1 protein were estimated using the SMART database. (B) Schematic diagram of mutation sites in *GFC1* gene ORF sequence. The numbers and short lines indicate individual mutation sites. g.347delC, g.687_696del, and g.737_806del represent deletion mutations. Point mutations include g.67T>A, g.86G>A, g.104G>A, g.152G>T, g.170G>T, g.187C>T, g.196C>T, g.258C>A, g.435C>A, and g.752G>A. g.255_256insC, g.373_502dup, g.687_696dup, and g.723_787dup represent insert mutations. The dashed boxes indicate the positions of two ZnF-C2H2 and four low complexity domains. Details for mutations are described in S1 Table. (C) Numbers and percentages of mutation types (Missense, nonsense, or frameshift) of Gfc1 identified in 48 evolved RL-FC isolates. Details for mutations are described in S1 Table.

We first sequenced the genomes of 21 evolved RL-FC isolates derived from individual glycerol-induced sectors using whole genome sequencing (WGS). To identify mutations that likely resulted in the heritable RL-FC phenotype, non-synonymous differences relative to the reference sequences from each isolate were determined. Interestingly, all 21 individual isolates exhibited alternative mutations in the ORF of the B9J08_003985 gene (Figs 2, S2, and S1 Table), suggesting that exposure of *C*. *auris* to glycerol resulted in a high frequency of mutations in the B9J08_003985 ORF. Sanger sequencing was used to verify mutations identified by WGS. For the other 27 RL-FC isolates, we also performed Sanger sequencing to determine the sequences of the B9J08_003985 ORF region (48 RL-FC isolates). As expected, all 27 isolates harbored a mutation in B9J08_003985, which encodes a C_2_H_2_ zinc finger regulator as shown in Fig 2A. Due to the glycerol-induced RL-FC phenotype and the phenotypic characterization of deletion mutants (described below), we heretofore referred to B9J08_003985 as Glycerol-induced Filamentation-Competent factor (*GFC1*).

Further investigation indicated that among 48 evolved RL-FC isolates, 9 harbored missense mutations (18.8%), 5 harbored nonsense mutations (10.4%), and 34 harbored frameshift mutations (70.8%) (Fig 2C). In total, eight RL-FC isolates harbored mutations in ZnF-C_2_H_2_ domains, whereas five RL-FC isolates harbored mutations in low complexity domains. Details for mutations that occurred in the *GFC1* ORF of all 48 isolates are described in S1 Table. Since all the 48 isolates exhibited both *GFC1* mutations and rod-like filamentous phenotypes, we assumed that Gfc1 might function in suppressing filamentous growth of *C*. *auris*.

Unlike *C*. *albicans* or other pathogenic *Candida* species, which prefer to colonize the human gut, *C*. *auris* predominantly colonizes the skin [2,5]. We further investigated the impact of Gfc1 variants on skin colonization using a newborn mouse skin infection model. Based on the types of *GFC1* mutations, six representative isolates V3 (152G>T), V4 (687_696dupTCGCACCGCT), V5 (86G>A), V8 (347delC), V10 (723_787dupGGGGTCTCTAGCTCCCGCCGGAGCCTCTTGGAGCTTAGGGTCAGGGTCAGGGCCAGGGTCAGGCT), and V23 (255_256insC) were selected for use (S1 Table). Scanning electron microscope (SEM) assays showed that all six Gfc1 variant-containing isolates exhibited the RL-FC phenotype and filamentous growth on skin surfaces, whereas the WT strain maintained a round yeast cell phenotype (S3A Fig). As expected, the fungal burdens of the six Gfc1 variant-containing isolates on the mouse skin were much higher than those of the WT strain, indicating that glycerol-induced Gfc1 variants confer *C*. *auris* with an increased ability to colonize the host skin (S3B Fig).

### Deleting the *GFC1* ORF results in the RL-FC/filamentous phenotype and enhances skin colonization

**Fig 3:**
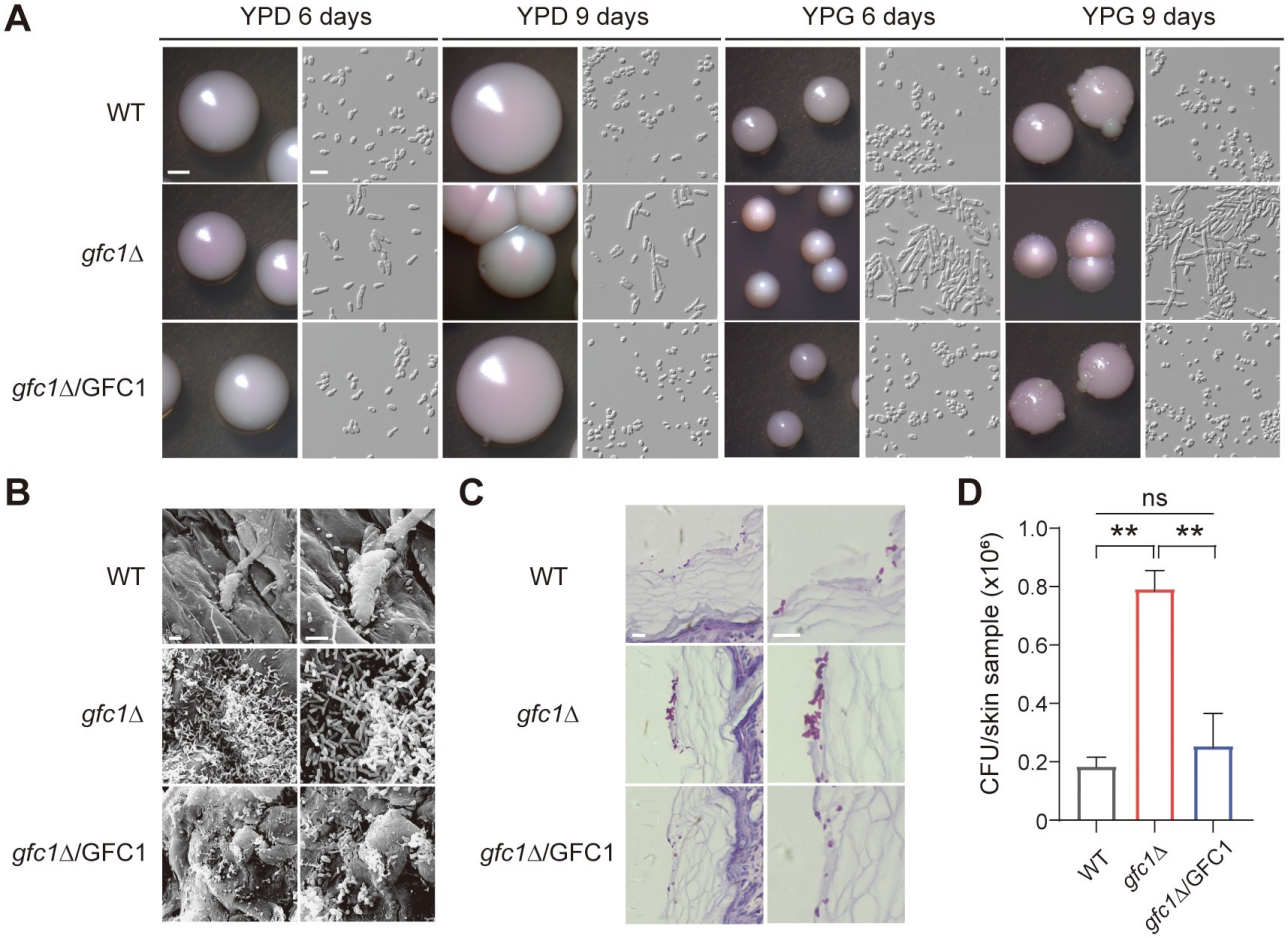
Deletion of *GFC1* in *C*. *auris* results in the RL-FC/filamentous phenotype and increased colonization ability on the mouse skin. WT, BJCA001. (A) Colony and cellular morphologies of the WT, *gfc1*Δ, and *gfc1*Δ/GFC strains on YPG and YPD media for 6 and 9 days of growth at 25°C. Scale bar for colonies, 1 mm; Scale bar for cells, 10 μm. (B) SEM images of the infected skin samples. 2 × 10^6^ cells of the WT, *gfc1*Δ, and *gfc1*Δ/GFC1 strains in 2 μL PBS were spotted on the dorsal back skin of new born mice. After 3 days of infection, the infected skin areas were excised, gently washed with 1 × PBS, and fixed with 2.5% glutaraldehyde for SEM assays. Scale bar, 10 μm. (C) Histopathological assays. Cell inoculation method was the same as described in panel B. After 3 days infection, the infected skin areas were excised and stained with periodic acid-Schiff (PAS) and then used for microscopy assays. Scale bar, 10 μm. (D) Fungal burdens on skin. Cell inoculation method was the same as described in panel B. After 3 days infection, the infected skin areas were excised, homogenized and then plated onto YPD agar for CFU assays. The experiment was repeated three times. For each time, three skin samples were used for each strain. The result of a representative experiment is shown. Error bars denote SD. ns, no significant difference. **P < 0.01 (Student’s *t*-test, two tailed).

To confirm the function of *GFC1* in *C*. *auris*, we first constructed a *GFC1* deletion (*gfc1*Δ) mutant using plasmid pSFS2a [42], then complemented back the *GFC1* cassette at the native locus. Consistent with glycerol-induced Gfc1 variants, *gfc1*Δ mutant exhibited the RL-FC phenotype on both YPD and YPG media, and formed wrinkled colonies containing a minority of filaments after prolonged incubation on YPG medium (Fig 3A). In contrast, the WT and *GFC1* complemented strain consistently maintained their round yeast cell phenotype. Although after 9 days of growth some “blebs” occurred on the surfaces of the WT and *GFC1* complemented strain colonies, no RL-FC/filamentous cells were observed. An incubation period of at least 11 days would be necessary for RL-FC/filamentous sectors formation. These results demonstrate that deletion of *GFC1* results in an RL-FC phenotype similar to that of *GFC1* variants and confers *C*. *auris* the ability to undergo filamentous growth, suggesting that Gfc1 functions as a negative regulator of filamentous growth in *C*. *auris*.

To investigate the impacts of Gfc1 disruption on colonization, we performed skin colonization and infection experiments with the *gfc1*Δ mutant, WT and complemented strains using the newborn mouse skin infection model. As expected, *gfc1*Δ mutant exhibited the RL-FC phenotype and an increased skin fungal burden, while the WT and complemented strains did not (Fig 3B–3D). These results were consistent with the findings of the *GFC1* variant-containing isolates. Taken together, glycerol could exclusively drive the loss of function of Gfc1, which thereby generates RL-FC phenotype and enhances colonization of the host skin.

### Inactivation of *GFC1* leads to increased fatty acid β-oxidation metabolism

**Fig 4:**
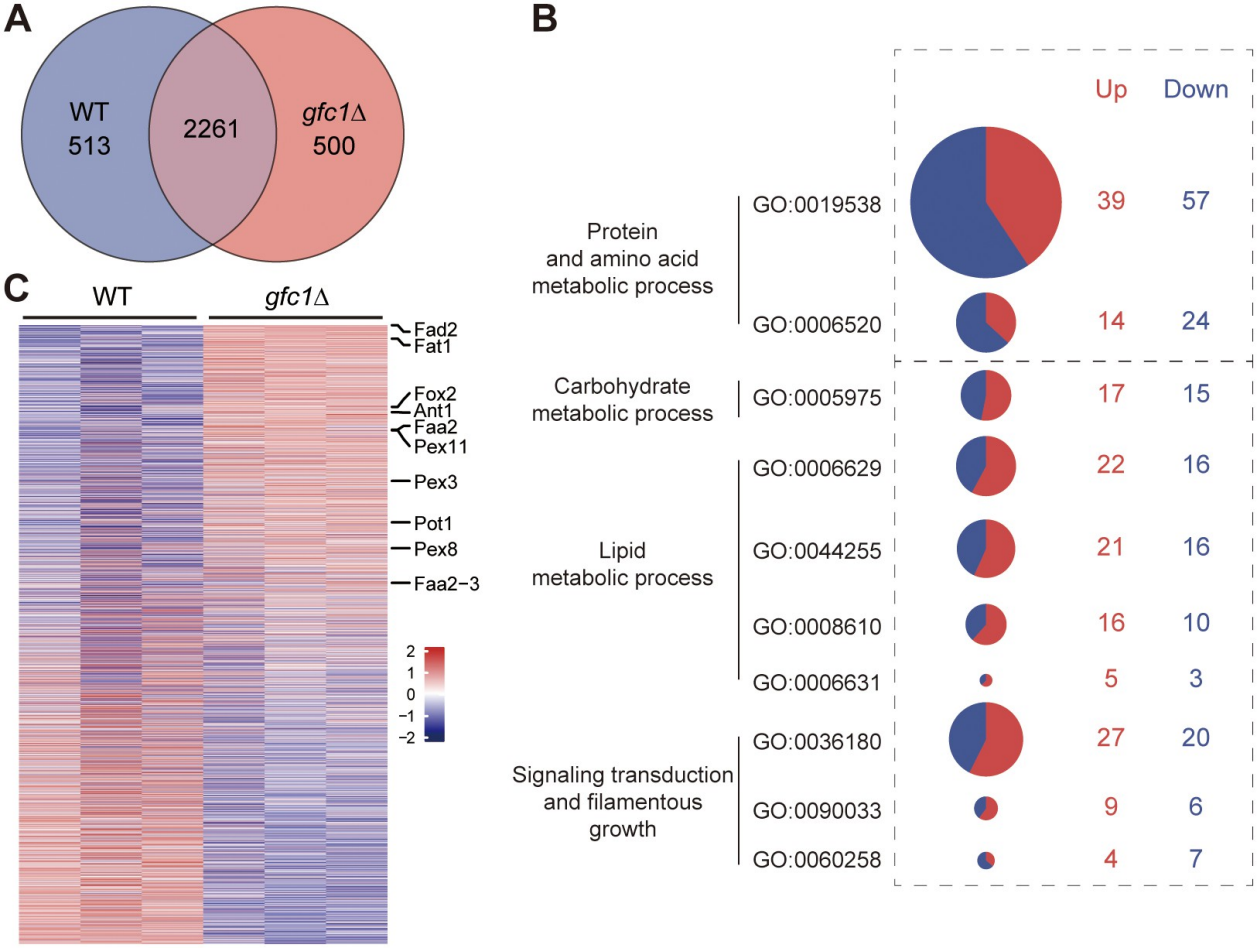
Protein expression profiles of the WT and *gfc1*Δ mutant strains grown on YPG medium. *C*. *auris* cells were grown on YPG medium at 25°C for 6 days, and then collected and lysed for proteomic analysis. WT, BJCA001. Detailed protein expression data were shown in S1 Dataset. (A) Venn diagram showing the differentially expressed proteins in *gfc1*Δ mutant. (B) The Gene Ontology (GO) analysis of the differentially expressed proteins in *gfc1*Δ mutant. Circle size is proportional to the number of differentially expressed proteins. Proteins categories (based on the GO analysis; S1 Dataset). (C) The heatmap shows changes in the expression of proteins in *gfc1*Δ mutant relative to WT cells. The pheatmap R package (version 1.0.12; https://cran.r-project.org/web/ packages/pheatmap/index.html) was used to plot expression levels of differentially expressed proteins. Proteins involved in fatty acid β-oxidation mechanism are indicated. Colors represent the relative expression levels of proteins (*gfc1*Δ/WT). The three columns for each strain represent three experiments were performed with 3 biological replicates.

To further investigate the regulatory mechanism of Gfc1 on *C*. *auris* filamentation, we performed a proteomic comparative analysis of the WT strain and *gfc1*Δ mutant grown on YPG medium (Fig 4A and S1 Dataset). In total, 1013 proteins exhibited ≥1.5 fold changes. Among them, 513 proteins were downregulated and 500 were upregulated in *gfc1*Δ mutant compared with those in the WT strain. We further performed gene ontology (GO) analysis to investigate the functional properties of the differentially expressed proteins. As expected, a subset of signal transduction and filamentation-related proteins were upregulated in the *gfc1*Δ mutant. Interestingly, a number of carbohydrate metabolism- and lipid metabolism-related proteins were also upregulated (Fig 4B), and most of them were related to fatty acid β-oxidation metabolism process, including Fad2, Fat1, Fox2, Ant1, Faa2, Pex3, Pex8, Pex11, Faa2-3, and Pot1 (Fig 4C). Consistently, high transcriptional levels of these genes were confirmed by qRT-PCR analysis (S4A Fig). The finding was consistent with our previous study in which the filamentous phenotype of *C*. *auris* isolated after passage through the mammalian body, exhibited remarkedly increased expression of fatty acid β-oxidation metabolism-related genes [32]. Taken together, this suggests that fatty acid β-oxidation plays a role in the regulation of filamentous growth in *C*. *auris*. Since the fatty acid β-oxidation metabolism is highly associated with mitochondrial oxidative respiration and ATP synthesis, we further determined whether deletion of *GFC1* also affected the mitochondrial basal oxygen consumption rate (OCR) and intracellular ATP content. As shown in S4B and S4C Fig, both the OCR and ATP levels were significantly increased in the *gfc1*Δ mutant compared to the WT and complemented strains. As expected, the metabolic activity of the *gfc1*Δ mutant was shown to be enhanced, as determined by using an XTT assay (S4D Fig). Taken together, the loss of function of Gfc1 increases fatty acid β-oxidation metabolism, which in turn enhances mitochondrial oxidative respiration and intracellular ATP production.

### Dysfunction of Gfc1 results in accumulation of oleic acid and linoleic acid

**Fig 5:**
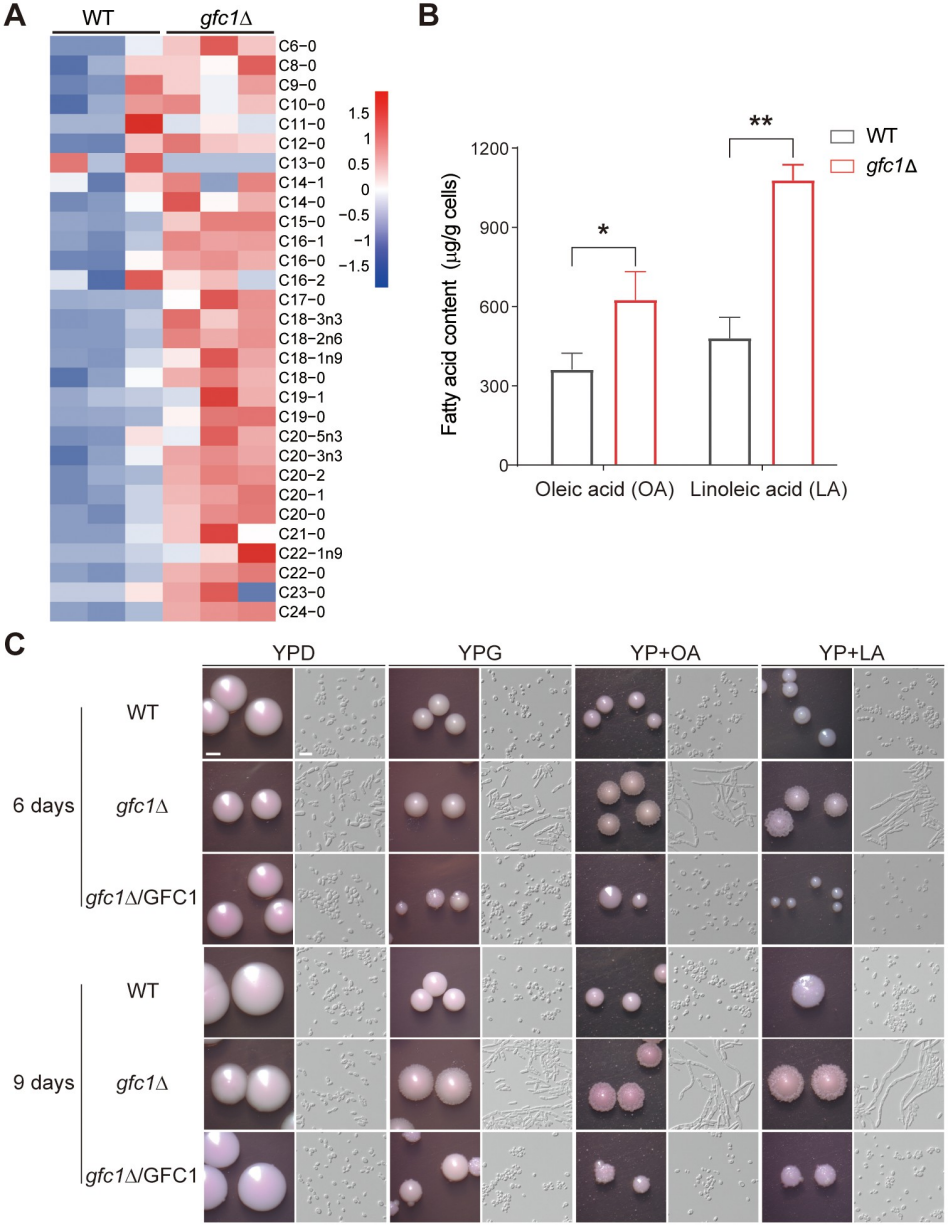
Deletion of *GFC1* promotes RL-FC cell formation and filamentation via accumulation of oleic acid (OA) and linoleic acid (LA). (A) Fatty acid profiles and relative content in the WT and *gfc1*Δ mutant strains after cultured in YPG at 25°C for 6 days. Cellular fatty acids were detected by MetWare based on the Agilent 7890B-7000D GC-MS/MS platform. The pheatmap R package (version 1.0.12; https://cran.r-project.org/web/packages/pheatmap/index.html) was used to plot different levels of fatty acids. Detailed fatty acid data were shown in S2 Dataset. (B) OA and LA content (μg/g cells) of the WT and *gfc1*Δ mutant strains grown in YPG at 25°C for 6 days. Error bars denote SD. *P < 0.05, **P < 0.01 (Student’s *t*-test, two tailed). (C) Colony and cellular morphologies of the WT, *gfc1*Δ and *gfc1*Δ/GFC1 strains in the presence of OA or LA. *C*. *auris* cells were grown on YPD, YPG, or YP containing OA (0.03%) or LA (0.03%), and incubated at 25°C for 6 or 9 days. Scale bar for colonies, 1 mm; Scale bar for cells, 10 μm. WT, BJCA001.

Fatty acids are defining components of fungal cell membranes and are therefore crucial for cell viability and cellular regulation [43]. We predicted that the induction of fatty acid β-oxidation metabolism in *C*. *auris gfc1*Δ cells would result from an increase in the levels of fatty acids. Given that, we investigated the fatty acid profile of the WT and *gfc1*Δ mutant strains. As shown in Fig 5A and S2 Dataset, compared with the WT strain, fatty acids accumulated in *gfc1*Δ cells. The two most abundant fatty acids of *Candida* lipids, oleic acid (OA) and linoleic acid (LA) [44], increased by ~30% and ~50%, respectively (Fig 5B). Several studies have indicated that the biosynthesis of fatty acids often resulted in changes in the adherence and virulence of *C*. *albicans* [44–48]. In addition, the composition of the fatty acids varies with the different morphological forms of *C*. *albicans*. Higher levels of OA and LA in the hyphal form of *C*. *albicans* were proposed, and OA was identified to play an inductive role in cell filamentation [44,45]. Therefore, we were also interested in determining whether exogenously supplied OA or LA promoted filamentous growth in *C*. *auris*. As shown in Fig 5C, in the presence of OA or LA, the *gfc1*Δ mutant underwent obvious filamentous growth and formed rough colonies with wrinkled edges as early as 6 days after incubation. Almost all *gfc1*Δ cells converted to elongated filaments by day 9 after further incubation. In contrast, the WT and *GFC1* complemented strains consistently maintained their round yeast cell phenotype and formed smooth colonies. These results demonstrated that OA and LA promoted filamentous growth in the *gfc1*Δ mutant, and exerted a more pronounced effect than that of glycerol. Taken together, disruption of *GFC1* in *C*. *auris* results in an increased fatty acid content (principally OA and LA), which in turn promotes filamentous growth.

### Mcu1 is required for Gfc1-suppressed filamentous growth

**Fig 6:**
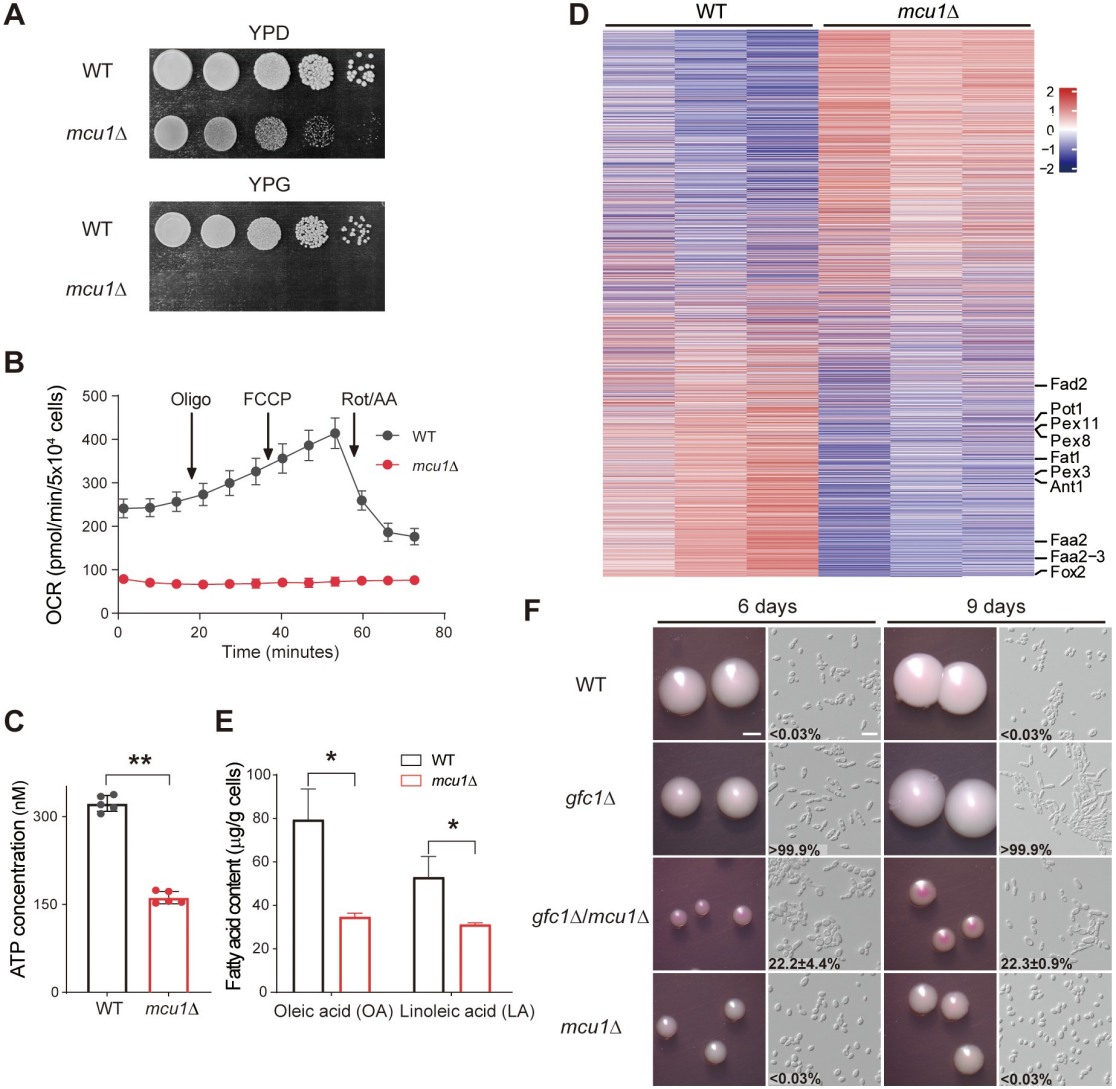
Role of the Mcu1 protein in Gfc1-suppressed filamentous growth in *C*. *auris*. For B, C and E, data are shown as the mean ± SD of three independent experiments. Error bars denote SD. For C and E, *P < 0.05, **P < 0.01 (Student’s *t*-test, two tailed). WT, BJCA001. (A) Growth of the WT, and *mcu1*Δ mutant strains on YPD and YPG media. The WT and *mcu1*Δ mutant strains were adjusted to 2.5 × 10^8^ cells/mL, and then 10-fold serial dilutions of cells were spotted onto YPD and YPG, respectively. Cells were cultured at 25°C for 3 days. (B) OCR in the WT and *mcu1*Δ mutant strains were measured by a Seahorse XFe96 analyser. *C*. *auris* cells were grown on YPD medium at 25°C for 6 days. Oligo (1.5 μM), oligomycin; FCCP (2 μM), Carbonyl cyanide 4-(trifluoromethoxy) phenylhydrazone; Rot/AA (0.5 μM), Rotenone /antimycin A. (C) Intracellular ATP content in the WT and *mcu1*Δ mutant strains after cultured on YPD medium at 25°C for 6 days. (D) The heatmap shows changes in the expression of proteins in *mcu1*Δ mutant relative to the WT cells. The pheatmap R package (version 1.0.12; https://cran.r-project.org/web/ packages/pheatmap/index.html) was used to plot expression levels of differentially expressed proteins. Proteins involved in fatty acid β-oxidation mechanism are indicated. Colors represent the relative expression levels of proteins (*mcu1*Δ/WT). The three columns for each strain represent three experiments were performed with 3 biological replicates. (E) OA and LA contents (μg/g cells) of the WT and *mcu1*Δ mutant strains grown on YPD medium at 25°C for 6 days. (F) Colony and cellular morphologies of the WT, *gfc1*Δ, *gfc1*Δ/*mcu1*Δ and *mcu1*Δ mutant strains on 1.5% YPG (YP plus 1.5% glycerol and 0.5% glucose) medium for 6 or 9 days of growth at 25°C. Percentage of RL-FC cells are indicated. Approximately 300–500 cells were examined for each culture. Scale bar for colonies, 1 mm; Scale bar for cells, 10 μm.

In *C*. *albicans*, the mitochondria protein Mcu1 (Multiple Carbon source Utilizer 1), which underpins carbon and respiratory metabolism, also plays crucial roles in filamentation and virulence [49]. Deletion of *MCU1* results in the inability of *C*. *albicans* to utilize glycerol as a sole carbon source. Thus, we investigated the effects of the Mcu1 homolog in *C*. *auris* on Gfc1-suppressed filamentation in the presence of glycerol. Consistently, dysfunction of Mcu1 in *C*. *auris* led to growth defects on YPG medium (Fig 6A), and greatly reduced the OCR and ATP production in YPD medium, suggesting a conserved role of Mcu1 in carbon source utilization and metabolism between the two *Candida* species (Fig 6B and 6C). A subsequent proteomic comparative analysis of *C*. *auris mcu1*Δ mutant grown on YPD medium indicated that compared with the WT strain, the aforementioned fatty acid β-oxidation metabolism-related proteins were significantly downregulated in the *mcu1*Δ mutant, indicating a crucial role of Mcu1 in fatty acid β-oxidation metabolism (Fig 6D). Fatty acid profile analysis showed that the content of two most abundant fatty acids OA and LA was significantly reduced in the *mcu1*Δ mutant, implying the potential roles of Mcu1 in OA and LA synthesis and filamentous regulation in *C*. *auris* (Fig 6E). To further test the role of Mcu1 in Gfc1-suppressed filamentation, we deleted the *MCU1* gene in the *gfc1*Δ background. Since dysfunction of Mcu1 caused growth defect of *C*. *auris* on YPG (YP plus 2% glycerol) medium, we cultured the *gfc1*Δ/*mcu1*Δ mutant on 1.5% YPG medium (YP plus 1.5% glycerol and 0.5% glucose). As expected, the *gfc1*Δ mutant exhibited the RL-FC phenotype on both YPD and 1.5% YPG media, while the *gfc1*Δ/*mcu1*Δ mutant only generated the RL-FC phenotype in 22.2±4.4% cells after 6 days of incubation, and 22.3±0.9% of cells after 9 days of incubation. These results indicated that deleting the *MCU1* gene in *gfc1*Δ mutant partly rescued the yeast phenotype, suggesting that Mcu1-mediated OA and LA metabolism was required for filamentous growth of the *gfc1*Δ mutant (Fig 6F). Taken together, the loss of function of Gfc1 increases OA and LA metabolism through Mcu1, which in turn triggers filamentous growth of *C*. *auris*.

### Filament-specific G1 cyclin-related protein Hgc1 and the transcription factor Ume6 are required for Gfc1-suppressed filamentous growth

To further characterize the regulatory mechanism controlling the development of the RL-FC/filamentous phenotype, we examined the transcriptional expression levels of a subset of filament-related regulators by performing qRT-PCR analysis. Interestingly, *C*. *auris* homologs of the cyclin-related protein Hgc1 and its key transcriptional interactor Ume6 were found to be greatly upregulated in the *gfc1*Δ mutant compared with the WT strain (S5A Fig) [50–52]. As expected, the expression levels of the two genes were remarkably downregulated in *C*. *auris mcu1*Δ mutant. Hgc1 is a hypha-specific G1 cyclin-related protein that is essential for hyphal morphogenesis in *C*. *albicans* [52]. Ume6 is a zinc DNA-binding transcription factor, and plays an inductive role in hyphal elongation in *C*. *albicans* [50, 51]. Consistently, deletion of either *UME6* or *HGC1* in *gfc1*Δ mutant completely restored the yeast growth form, even on YPG medium, after a prolonged incubation period (S5B Fig). Taken together, loss of function of Gfc1 in *C*. *auris* results in filamentous growth via the activating function of the two filament regulators Ume6 and Hgc1.

## Discussion

**Fig 7:**
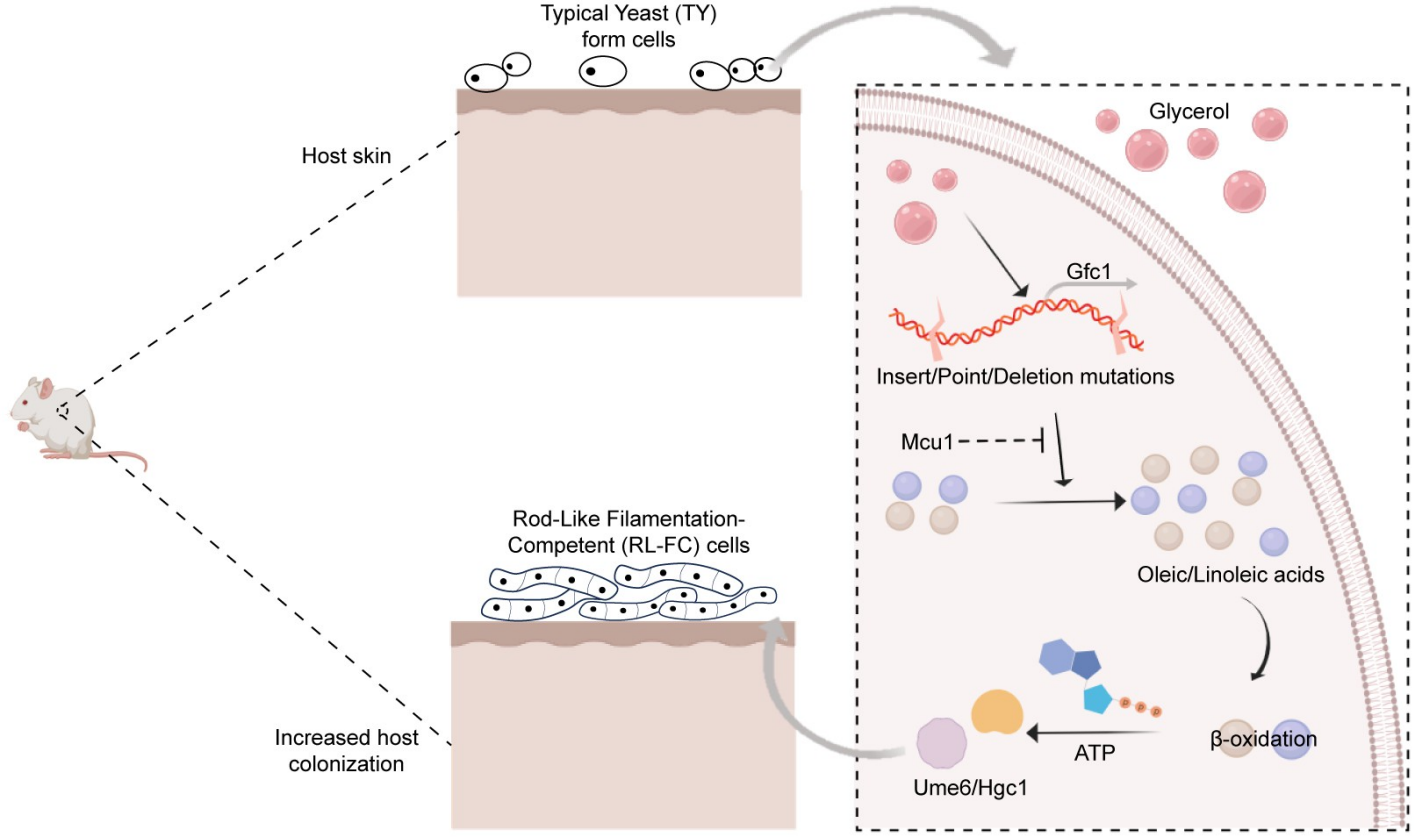
Schematic model of adaptive evolution of *C*. *auris* caused by glycerol.

Morphological plasticity is a common strategy adopted by pathogenic fungi to rapidly adapt to host environment, and to cause infections. As a rapidly emerging threat worldwide, *C*. *auris* exhibits many unique characteristics in the initiation and progression of filamentation. In this study, we reported that exposure of *C*. *auris* to glycerol resulted in recurrent mutations in the *GFC1* ORF region, which thereby led to novel RL-FC phenotype formation and filamentous growth in *C*. *auris*. Mcu1-mediated fatty acid β-oxidation metabolism and two cell cycle-related factors Ume6 and Hgc1 were subsequently identified to play critical roles during this process. Both glycerol-induced Gfc1 variants and deletion of *GFC1* resulted in an increased colonization ability in a mouse skin infection model (Fig 7). Glycerol is a compound liquid widely used in the cosmetics, pharmaceutical, and household industries. It is a common ingredient in skin care products, humectant, cough medicines, gel capsules, toothpaste, soaps, textiles and so on. Given that, the selection for *GFC1* mutations by glycerol exposure may occur frequently and is important for *C*. *auris* colonization, infection and transmission.

Adaptive evolution to the natural or host environment is found to occur frequently in pathogenic fungi. Many environmental cues, such as temperature fluctuations, nutrient alterations, and antifungal stresses, have been demonstrated to drive the evolution of fungal characteristics [53]. It has been indicated that the evolution of *C*. *auris* as a human pathogen may have resulted from climate change, specifically global warming [2,54]. Its unique property, salinity tolerance, has been considered to confer this fungus the capacity to undergo a morphological transition as an adaptive evolution mechanism [2,29,55]. In addition, the widespread use of antifungal drugs has led to the evolution of antifungal resistance in *C*. *auris* [2,54]. Our previous study revealed that passage through a mammalian body triggered filamentous growth of *C*. *auris* [32]. Here, we set out to explore the evolutionary mechanism and found that long-term cultivation of *C*. *auris* in the presence of glycerol resulted in an evolved RL-FC phenotype and filamentous growth, which enhanced its ability to colonize host skin (Figs 1 and 3). Long-term persistence and survival on biotic and abiotic surfaces are hallmark characteristics of *C*. *auris* which may contribute to its intrahospital transmission [2,5,6]. Therefore, our findings shed light on a novel adaptive evolutionary mechanism of *C*. *auris* in enhancing host colonization, infection, and transmission.

Adaptive evolution often results in loss-of-function mutations and changes in cellular physiology. A study of *C*. *albicans* under evolutionary pressure by serial passage in a mouse GI tract identified recurrent mutations in the *FLO8* gene, which contributed to highly competitive fitness in the host GI tract [56]. Collections of *Candida lusitaniae* isolates from three individuals with cystic fibrosis and persistent lung infections showed acquired mutations in the *MRS4* gene, which encodes a mitochondrial iron transporter [57]. Recently, several studies highlight the critical roles of environmental stresses in driving genetic or genomic heterogeneity of *C*. *auris*. After a series of passages through increasing concentrations of fluconazole, a fluconazole-susceptible isolate of *C*. *auris* acquired one extra copy of chromosome V, which harbors several drug resistance-related genes, and thereby conferred the fungus with fluconazole resistance [40]. A similar study recently reported that upon exposure to fluconazole, mutations of the transcription factor TAC1B arose rapidly, which contributed to clinical fluconazole resistance in *C*. *auris* [58]. In addition, *C*. *auris* can undergo a ploidy shift between haploid and diploid forms under certain stressful conditions [59]. To explore the genetic or genomic mutations following the adaptive evolution of *C*. *auris* in response to glycerol, we performed a WGS analysis of the evolved RL-FC isolates. We found that all RL-FC isolates harbored a mutation within the ORF region of the *GFC1* gene, including missense, nonsense, or frameshift mutations (Fig 2 and S1 Table), implying that the presence of glycerol accumulates *GFC1* mutations. Although the mechanism underlying this phenomenon remains unclear, one possible explanation could be that different mutations in the *GFC1* gene confer *C*. *auris* cells a high selection advantage in the presence of glycerol, such as an advantage in glycerol metabolism or an increased ability to colonize the host (Figs 3, S3 and S6).

It has been demonstrated that carbon compounds are not only a nutrient source, but also act as signal effectors that cause alternative cellular responses [60]. Glycerol is a common carbon source for fungal growth and often triggers a morphological transition in *Candida* spp. Our recent studies reported that glycerol was able to induce filamentous growth in *C*. *haemulonii*, a close relative of *C*. *auris* in the Metschnikowiaceae clade [61–63]. We demonstrated that the presence of glycerol or low temperatures favored the filamentous phenotype in *C*. *haemulonii*, which was consistent with our findings here. One study demonstrated that glycerol promoted pseudohyphal growth of *C*. *parapsilosis* at 37°C through a different signaling pathway compared with that induced by glucose at 30°C [64]. Moreover, glycerol has been demonstrated to promote biofilm development of *C*. *albicans*, and the authors emphasize the pivotal role that glycerol can play, is not only metabolic but also regulatory impact [65]. Here, we found that exposure to glycerol caused the accumulation of *GFC1* gene mutations, which in turn triggered a novel RL-FC phenotype as well as filamentous growth of *C*. *auris* (Figs 1, 2 and S1 Table). The role of glycerol in promoting filamentous growth of *C*. *auris* via accumulating genetic mutations was reported here for the first time. However, the underlying mechanism needs to be further explored.

Many studies have reported that lipid or fatty acid β-oxidation metabolism in pathogenic fungi is highly linked with their morphogenesis or pathogenesis [66]. Catabolism occurs via the β-oxidation pathway, in which fatty acids are first esterified to the corresponding acyl coenzyme A (CoA) and then oxidized to acetyl-CoA, which finally enters the TCA cycle [66]. In *Ustilago maydis*, the ability to utilize host lipids is highly related to its pathogenic development [67]. β-Oxidation metabolism associated with the cAMP signaling pathway and the Ras/MAPK pathway was demonstrated to promote filamentous growth and pathogenesis in *U*. *maydis*. In *Blastomyces dermatitidis*, lipid droplets were found to be actively metabolized during the phase transition from yeast to mold, and lipid metabolism contributed to filamentous growth [68]. Many studies have reported that the altered composition of fatty acids often resulted in changes in adherence and virulence of *Candida* species [44]. Moreover, the preference of *C*. *auris* for colonizing human skin has been considered to be associated with its metabolic predilection. Our previous study demonstrated that the filamentous phenotype obtained after passage through the mammalian body exhibited upregulated expression of fatty acid β-oxidation metabolism-related genes [32]. In this study, we investigated protein expression levels of the *gfc1*Δ mutant by performing a proteomic comparative analysis. Consistently, increased expression of fatty acid β-oxidation metabolism-related proteins was observed in the *gfc1*Δ mutant compared to the WT strain (Fig 4). Taken together, filament development in *C*. *auris* requires the enhanced activity of fatty acid β-oxidation metabolism and Gfc1 plays an essential role during this process. However, the underlying mechanisms that generate this effect need to be further explored.

Here, we found that Gfc1 is a C_2_H_2_ zinc finger regulator that controls the generation of RL-FC phenotype in *C*. *auris*. The closest homologs of Gfc1 in *C*. *albicans* is Bcr1, a key transcription factor that governs morphological switching in both white and opaque cells [69–71]. In white cells, Bcr1 is required for biofilm development *in vitro* and *in vivo* but is not required for filamentous growth [71]. In opaque cells, Bcr1 is a central regulator that suppresses filamentation in a cAMP signaling-dependent manner. A subset of genes, including the filament regulator Ume6 and G1 cyclin-related protein Hgc1, are repressed by Bcr1 during opaque cell filamentation [70,71]. Consistently, Gfc1 of *C*. *auris* plays a negative role in the regulation of filamentous growth in *C*. *auris*, and the expression of *UME6* and *HGC1* genes was also repressed by Gfc1. These findings indicate that Gfc1 in *C*. *auris* appears to have similar functions to Bcr1 in *C*. *albicans* opaque cells, but not to Bcr1 in *C*. *albicans* white cells [70,71]. Notably, *C*. *auris* cells share more biological features with *C*. *albicans* opaque cells, including oxidative metabolism preference and skin colonization advantage [19,72].

In summary, we uncovered a novel RL-FC phenotype and a unique evolutionary strategy involving in a filamentous regulatory mechanism in *C*. *auris*. Exposure of *C*. *auris* to glycerol caused recurrent mutations in the *GFC1* gene, which resulted in metabolism changes and filamentous growth, and thereby enhanced skin colonization. Considering that *C*. *auris* predominantly colonizes human skin and glycerol is widely used in our daily life, we suggest that presence of glycerol may enhance *C*. *auris* colonization or infection, perhaps for adaptation to the host environment, as well as for rapid and widespread transmission. Our study therefore not only sheds light on the biology and pathogenicity of *C*. *auris* but also provides important information for prevention and control of fungal infections.

## Material and methods

### Ethics statement

All animal experiments were conducted in compliance with the guidelines and regulations set forth by the Animal Care and Use Committee of Fudan University (2021JS004). The present study was approved by the Committee.

### Strains and growth condition

Yeast strains used in the study are listed in S1 Table. *C*. *auris* strains were stored at -80°C in 25% glycerol (Sinopharm Chemical Reagent Co., Ltd. Cat. No. 56-81-5). To revive the strains from the frozen stocks, *C*. *auris* cells were scratched with a sterile tip and streaked onto yeast extract-peptone-dextrose (YPD) medium plates (10 g/L yeast extract from Angel Company, Hubei, China; 20 g/L peptone from Oxoid Ltd. Company. Hants, UK; 20 g/L glucose and 20 g/L Agar from Sangon Biotech, Shanghai, China) and incubated at 30°C. YPD medium supplemented with 5 μg/mL phloxine B (Sigma-Aldrich) were used for routine growth of *C*. *auris*.

For the screening assay of carbon sources, YP medium with 5 μg/mL phloxine B plus different carbon sources (Sigma-Aldrich) including glycerol (20 mL/L), glucose (20 g/L), GlcNAc (20 g/L), mannitol (20 g/L), pyruvate (20 g/L), citric acid (20 g/L), and acetic acid (20 g/L) were used. For the morphological assays, YPOA medium (10 g/L yeast extract, 20 g/L peptone, 0.3 mL/L oleic acid) and YPLA medium (10 g/L yeast extract, 20 g/L peptone, 0.3 mL/L linoleic acid) supplemented with 5 μg/mL phloxine B were used.

### Plasmid construction

The primers used for PCR amplification in this study are listed in S2 Table. To construct the deletion plasmids pSFS2a-ko-au*ARG4* and pSFS2a-ko-au*GFC1*, approximately 1000 bp fragments of the 5’-UTR and 3’-UTR of *ARG4* or *GFC1* were amplified from *C*. *auris* BJCA001 genome and subcloned into the *Apa*I*/Xho*I and *Sac*II/*Sac*I sites of pSFS2a plasmid [73], respectively. To construct the deletion plasmid pSFS2a-ko-au*MCU1*, around 1000bp fragments of the 5’-UTR and 3’-UTR of *MCU1* were amplified and sequentially introduced into the *Not*I/*Sac*II and *Kpn*I/*Xho*I sites of pSFS2a. The reconstituted plasmid pBlueScript-*GFC1p*-*GFC1* was created based on the pBlueScript plasmid [74]. The *ARG4* cassette, along with the 5’-UTR plus ORF region of *GFC1*, and 3’-UTR were amplified from *C*. *auris* BJCA001 genome and sequentially inserted into the *Xba*I/*Not*I, *Spe*I/*Eco*RI and *Xba*I/*Spe*I sites of the pBluescript II KS (+) plasmid [74].

### Construction of *C*. *auris* mutant strains

To construct the deletion mutant of *ARG4* (FDYC0194), the *Apa*I/*Sac*I linearized plasmid pSFS2a-ko-au*ARG4* was transformed into strain BJCA001 to replace the *ARG4* allele. The resulting strain was then cultured in 5% YPM (10 g/L yeast extract, 20 g/L peptone, 50 g/L maltose) medium for FLP-mediated excision of the *SAT1* flipper cassette. The deletion mutant of *GFC1* (FDYC0231) was constructed using a similar strategy. The *Apa*I/*Sac*I linearized plasmid pSFS2a-ko-au*GFC1* was transformed into strain FDYC0231 to replace the *GFC1* allele. The resulting strain was then cultured on 5% YPM medium for FLP-mediated excision of the *SAT1* flipper cassette. To generate the *GFC1* reconstituted strain FDYC0747, the plasmid pBlueScript-*GFC1p*-*GFC1* was linearized with *Spe*I and transformed into strain FDYC0231. To construct the deletion mutant of *MCU1* (FDYC0582), the *Kpn*I linearized plasmid pSFS2a-ko-au*MCU1* was transformed into strain FDYC0194 to replace the *MCU1* allele. The *gfc1*Δ/*mcu1*Δ double mutant FDYC0952 was constructed by transforming the FDYC0231 strain with the *Kpn*I linearized plasmid pSFS2a-ko-au*MCU1*. To delete *UME6* and *HGC1* in *gfc1*Δ (FDYC0231) mutant, fusion PCR reactions were performed [75]. 5’- and 3’-flank fragments of *UME6* or *HGC1*, as well as the selectable marker gene *ARG4* were amplified from *C*. *auris* BJCA001 genome. Fusion PCR assays were performed using the 5’- and 3’-flank fragments and *ARG4* as templates. *gfc1*Δ (FDYC0231) strain was transformed with the fusion PCR products comprising the Cau*ARG4* cassette flanked by 5’- and 3’- flanking fragments of the target gene.

### Whole genome sequencing and analysis

Single colonies of selected RL-FC isolates were inoculated into YPG medium and grown at 25°C for 24 h. Genomic DNA was extracted using the TIANamp Yeast DNA Kit (TianGen Biotech, Beijing, China). In brief, approximately 5×10^7^ cells were suspended in 600 μL of sorbitol buffer containing 50 U Lyticase (Cat. No. RT410) and incubated at 37°C for 30 min. Following centrifugation at 13, 000 × g for 5 min, 200 μL of buffer GA, 20 μL of Proteinase K solution, and 220 μL buffer GB were added. The samples were then incubated at 70°C for 10 min. After two rounds of washing, genomic DNA was collected and stored at -20°C. Whole genome sequencing was conducted by Berry Genomics Co., Beijing, China. Sequencing libraries were generated using a NEBNext Ultra DNA library prep kit for Illumina (NEB, USA). The DNA samples were sonicated to obtain fragments of approximately 300 bp in size. The fragmented DNA was end-polished, and PCR amplification was performed using full-length adaptor for Illumina sequencing. Purified PCR products were analyzed for size distribution using an Agilent 2100 Bioanalyzer. Each sample was then subjected to sequencing on the Illumina NovaSeq platform, generating 2 × 150 bp reads with a minimum coverage of 450 × SNP and INDEL analyses were performed as described in our previous publication [40]. Briefly, clean reads were mapped to the genomic assembly of *C*. *auris* strain B11221 (NCBI accession number: GCF_002775015.1) using BWA mem 0.7.17 software with default settings [75]. SAMTools v1.361 [76], Picard Tools v1.56 (http://picard.source-forge.net), and GATK v2.7.2 (https://gatk.broadinstitute.org/hc/en-us) were employed for variation analyses [77]. Finally, Sanger sequencing was used to confirm identified variations.

### Virulence in a cutaneous mouse model

The experimental protocol followed in this study was adapted from the method described by Kvaal et al [78], with some modification. Newborn BALB/c mice aged between 3 to 5 days were used for cutaneous infection experiments. A stencil with a surface area of 8 mm^2^ was used to mark the specific area of the skin for colonization. The marked skin area was disinfected using 75% ethanol. *C*. *auris* cells were initially grown overnight in YPD liquid medium at 30°C and then harvested and suspended in PBS. A suspension containing 2×10^6^ *C*. *auris* cells in 2 μL was spotted onto the marked area of the dorsal back skin. To ensure complete coverage of the marked area with *C*. *auris* cells, the inoculum was thoroughly spread using a sterile pipette tip. After the skin surface dried, a small sterilized glossy paper was affixed on the inoculated spot with First Aid waterproof tape. At the end of the experimental period, each mouse was euthanized using CO_2_ anesthesia. The filter paper was carefully removed, and the marked skin area was excised for further analysis.

### Histopathological assay

The infected skin tissues of the mice were fixed with 10% (w/v) buffered formalin. After fixation, the tissues were washed, dehydrated, and embedded in paraffin wax. The embedded samples were sliced into sections with a thickness of around 4 μm using a microtome (MICROM International GmbH, Germany). These sections were then subjected to staining with periodic acid-Schiff (PAS) for subsequent microscopy assays.

### Scanning electron microscopy (SEM)

The infected skin tissues were sectioned and fixed with 2.5% glutaraldehyde for 24 h. The samples were then dehydrated using gradually increasing concentrations of ethanol (50, 75, 90, and 100%) and tertiary butanol (50, 75, 90, and 100%), dried, and then coated with a thin layer of gold. The prepared samples were visualized and imaged using the Hitachi FlexSem1000 II Scanning Electron Microscope.

### Proteomic analysis

*C*. *auris* strains were cultured on YPD or YPG medium plates at 25°C for 6 days. Cells were harvested, and total protein was extracted for proteomic analysis following a previously described protocol [79]. The collected cells were rinsed twice with 1 × PBS, and then resuspended in 200 μL of lysis buffer (50 mM Tris-HCl, pH 8.0, 150 mM NaCl, 1% NP-40, 1% Na-deoxycholate, 0.1% (w/w) SDS, 1 mM EGTA, 1 mM EDTA, 1 mM PMSF) containing the protease inhibitor cocktail (Cat. No. 11873580001 Roche Diagnostics, Mannheim, Germany). *C*. *auris* cells were lysed using a bead beating instrument (40 s beating followed by 1 min cooling on ice for 5 cycles). The supernatant was collected and the protein concentration was determined by Bradford assays (Sigma-Aldrich). For LC-MS/MS analysis, total proteins were digested via FASP method using the FASP method with Nanosep 10k filters (Pall Life Science, USA) [80]. After three rounds of buffer displacement using 8 M urea in 25 mM NH_4_HCO_3_, proteins were reduced with 10 mM DTT and alkylated with 30 mM iodoacetamide. The filter was then washed once with 20% acetonitrile (ACN) followed by three washes with digestion buffer (30 mM NH_4_HCO_3_), and was overnight digested using trypsin (enzyme/protein (w/w) ratio as 1:50). The resulting solution was filtered, and the filter was washed twice with 15% ACN. All the filtrates were pooled and vacuum-dried. LC-MS/MS analysis was performed using an EASYnLC 1200 system (Thermo Fisher Scientific, USA) coupled with an Orbitrap Fusion mass spectrometer (Thermo Fisher Scientific, USA). A one-column configuration was utilized, employing a home-packed C 18 column (75 μm i.d. × 25 cm; ReproSil-Pur 120 C18-AQ, 1.9 μm (Dr. Maisch GmbH, Germany)) [81]. The mobile phases consisted of Solvent A (0.1% formic acid) and Solvent B (0.1% formic acid in 80% ACN). Peptides were eluted directly into the Orbitrap Fusion Lumos mass spectrometer. Data-dependent analysis was employed for MS scans, with MS1 scans acquired in the Orbitrap analyzer at a resolution of 60,000 (m/z range: 350–1600) and an auto maximum ion injection time. The cycle time between master scans was set to 3 seconds, and precursor ions were fragmented using HCD mode. Fragmented ions were then analyzed using the Orbitrap analyzer at a resolution of 15,000 and a normalized collision energy (NCE) of 30%. The raw data were processed using Proteome Discoverer software (version 2.4, Thermo Fisher Scientific) with an in-house Mascot search engine (version 2.7.0, Matrix Science). The data search was conducted against the *C*. *auris* protein database obtained from UniProt. Trypsin/P was chosen as the enzyme, allowing for up to two missed cleavages. The precursor mass tolerance was set at 10 ppm, and the fragment mass tolerance was set at 0.05 Da. Carbamidomethylation on cysteine was designated as a fixed modification, while N-acetylation at the protein N-terminal and oxidation on methionine were considered as variable modifications. The false discovery rate threshold was set to 0.05 for proteins and 0.01 for peptides. Three replicates were carried out. Differential expression analysis of proteins was performed using the DEP package (version 3.16) in R [82].

### RNA extraction and qRT-PCR

RNA extraction and quantitative real-time polymerase chain reaction (qRT-PCR) experiments were performed with slight modifications to a previously described protocol [40]. *C*. *auris* strains were cultured on YPG plates at 25°C for 9 days. Total RNA was extracted from the harvested cells using the GeneJET RNA Purification Kit (Thermo Fisher Scientific, Cat. No. K0731). Briefly, the collected cells were resuspended in 200 μL solution buffer containing 0.9 M sorbitol, 0.1 M EDTA (pH 7.5), 50 U Lyticase (Cat. No. RT410) and 10 μL DTT (1M). After incubation at 37°C for 30 min, cells were collected, resuspended in pre-cooling Lysis Buffer (40 seconds beating followed by 1 minute cooling on ice), and then lysed using a bead beating instrument. Total RNA was isolated using the GeneJET RNA Purification Kit. For qRT-PCR assays, 1 μg of total RNA per sample was used to synthesize cDNA with RevertAid H Minus Reverse Transcriptase (Thermo Scientific, Inc., Beijing, China). Quantification of transcripts for qRT-PCR was conducted on a Bio-Rad CFX96 real-time PCR detection system (Bio-Rad, Hercules, USA) using SYBR green master mix (QPS-201, TOYOBO, Osaka, Japan). The expression levels were normalized to the *C*. *auris ACT1* gene. Data were analyzed using Bio-Rad CFX Manager 3.1 for Bio-Rad Real-Time PCR system. Three biological replicates were employed for the analysis.

### Intracellular ATP quantification assays

To investigate the intracellular ATP content of *C*. *auris* strains, colonies grown on YPG plate for 6 days were collected and subjected to analysis using the BacTiter-Glo Microbial Cell Viability Assay (Promega). In brief, approximately 10^8^ CFU of *C*. *auris* in 100 μL was mixed with an equal volume of BacTiter-Glo luciferase reagent. The mixture was then incubated for 15 min at room temperature in the dark. To quantify the intracellular ATP content, a standard curve was established using serial tenfold dilution of ATP disodium salt (Solarbio, Cat. No.C0550), starting from a concentration of 1 μM. The ATP content of the samples was determined using Cytation 3 plate reader (BioTek Instruments Inc, USA) and the resulting values were then normalized to their corresponding CFU values.

### XTT assays

A 2,3-bis (2-methoxy-4-nitro-5-sulfophenyl)-2H-tetrazolium-5-carboxanilide (XTT) assay was conducted using XTT Cell Viability Kit (Cell signaling tech., Cat. No.9095). Piror to the experiment, the electron coupling solution and XTT Reagent were thaw. Then electron coupling solution was added to the XTT Reagent in a volume ratio of 1:50 to prepare the XTT detection solution. For the investigation of XTT, *C*. *auris* strains on YPG plate for 6 days were resuspended in 1x PBS. Approximately 3×10^7^ CFU in 150 μL was mixed with 50 μL of the XTT detection solution. The mixture was incubated for 10 min at room temperature in the dark. Subsequently, the optical density (OD) of the samples was measured at 450 nm using a microplate reader (BioTek Instruments Inc. USA).

### OCR assays

A Seahorse Xfe96 analyser (Agilent) was used to measure the oxygen consumption rate (OCR), following the described protocol [79]. Data were analyzed using the Wave 2.6. In brief, *C*. *auris* cells on YPG plate for 6 days-incubation were seeded at a density of 5×10^4^ CFU per well in poly-L-lysine (0.03%) precoated Seahorse XF96 cell culture microplates (Agilent). The plates were then centrifuged and incubated for 1 h at 30°C allowing the cells to adhere to the microtiter plates. The OCR was examined by sequential injections of oligomycin (Oligo, 1.5 μM), Carbonyl cyanide 4-(trifluoromethoxy) phenylhydrazone (FCCP, 2 μM) and rotenone/antimycin A (Rot/AA, 0.5 μM). These injections allowed the assessment of various aspects of mitochondrial function.

### Fatty acid profile analysis

*C*. *auris* strains were cultured on YPG plate at 25°C for 6 days. Samples were weighed 50 mg and then mixed with 150 μL of methanol, 200 μL of methyl tert-butyl ether and 50 μL of 36% phosphoric acid. The samples were vortexed for 3 min and then subjected to a freeze-thaw cycle by immersing them in liquid nitrogen for 2 min, followed by thawing on ice for 5 min. This freeze-thaw process was repeated twice. After centrifugation at 4°C, 100 μL of supernatant was obtained and evaporated using a nitrogen blower. Then, 300 μL of 15% boron trifluoride methanol solution was added, following by vortexing for 3 min. The mixture was kept in a 60°C oven for 30 min, cooled to room temperature, and 500 μL of hexane solution and 200 μL saturated sodium chloride solution were added accurately. After vortexing for 3 min, the mixture was centrifuged at 4°C, and 100 μL of hexane layer solution was collected for analysis. Gas chromatography (GC)-electron ionization (EI)-mass spectrometry (MS)/MS system (GC, Agilent 7890B; MS, 7000D System) was used for analysis. The following GC conditions were employed: GC column-DB-5MS capillary column (30 m × 0.25 mm × 0.25 μm, Agilent); carrier gas-high purity helium (purity >99.999%); heating procedure–initial temperature of 40°C (2 min), followed by an increase of 30°C/min up to 200°C (1 min), then an increase of 10°C/min up to 240°C (1 min), and a final increase of 5°C/min up to 285°C (3 min); flow rate—1.0 mL/min; inlet temperature—230°C; injection volume—1.0 μL. The Agilent 7890B-7000D EI-MS/MS system was used, with the following setting: temperature—230°C; ionization voltage—70eV; transmission line temperature—240°C; four-stage rod temperature—150°C; solvent delay—4 min; and scanning mode—SIM. Fatty acids and their metabolites were detected by MetWare based on the Agilent 7890B-7000D GC-MS/MS platform. The data gathered from metabolomics analysis were derived from the average of three independent samples for every condition. The normalized metabolomic data used in this study can be accessed in S2 Dataset.

### Statistical analysis

All values presented in this study are reported as means ± standard deviation (SD) unless specifically mentioned. All experiments were conducted in a minimum of three independent trials to ensure reproducibility. GraphPad Prism software (version 10.1.0) was utilized to generate figures and perform statistical analyses. Statistical significance was determined using two-tailed unpaired Student’s *t*-test, one-way ANOVA or two-way ANOVA, as indicated in the respective analyses. A P-value less than 0.05 was considered statistically significant, except for the proteomic analysis and fatty acid profile analysis, where a fold change greater than 1.5 was defined as statistically significant.

## Supporting information

S1 Fig Growth and morphology of *C*. *auris* on different media at 25°C for 11 days. Y (Yeast extract) P (Peptone) + different carbon source: Sucrose, N-acetylglucosamine GlcNAc, Mannitol, Pyruvate, Citric acid, Acetic acid. Scale bar for colonies, 1 mm; Scale bar for cells, 10 μm. The strain used was BJCA001.
(TIF)

S2 Fig Colony morphologies of 21 *GFC1* variants-containing isolates of *C*. *auris* identified by whole genome sequencing. 21 *C*. *auris* isolates were grown on YPG medium for 9 days at 25°C. Details for mutations are described in S1 Table. Scale bar: 1 mm.
(TIF)

S3 Fig Skin infection assays with glycerol-induced *GFC1* variants-containing isolates of *C*. *auris* using a newborn mouse model. Approximately 2 × 10^6^ *C*. *auris* cells of the WT and *GFC1* variants-containing isolates V3 (152G>T), V4 (687_696dupTCGCACCGCT), V5 (86G>A), V8 (347delC), V10 (723_787dupGGGGTCTCTAGCTCCCGCCGGAGCCTCTTGGAGCTTAGGGTCAGGGTCAGGGCCAGGGTCAGGCT), and V23 (255_256insC) in 2 μL PBS were spotted on the dorsal back skin of newborn mice. After the skin surface dried, a small sterilized glossy paper was affixed on the inoculated spot with medical tape. WT, BJCA001. (A) Scanning electron microscope (SEM) images of the infected skin samples. After 3 days of infection, the infected skin areas were excised, gently washed with 1 × PBS, and fixed with 2.5% glutaraldehyde for SEM assays. Scale bar, 10 μm. (B) Fungal burdens of the WT and *GFC1* variants-containing isolates on skin. After 3 days of infection, the infected skin areas were excised, homogenized and then plated onto YPD mdeia for CFU assays. The experiment was repeated three times. For each time, three skin samples were used for each strain. The result of a representative experiment is shown. Error bars denote the standard deviation (SD). *P < 0.05, **P < 0.01, (Student’s *t*-test, two tailed).
(TIF)

S4 Fig Deletion of *GFC1* leads to increased oxidative metabolisms and ATP production. *C*. *auris* cells were grown on YPG medium at 25°C for 6 days. Data are shown as the mean ± SD of three independent experiments. Error bars denote SD. For A, B and C, *P < 0.05, **P < 0.01 (Student’s *t*-test, two tailed). WT, BJCA001. (A) Relative expression levels of genes involved in fatty acid mechanism in the WT, *gfc1*Δ, and *gfc1*ΔGFC1 strains. Cells were collected and lysed for qRT-PCR analysis. The expression level of the WT strain for each gene was set as 1. ns not significant. (B) Oxygen consumption rate (OCR) in the WT, *gfc1*Δ, and *gfc1*Δ/GFC1 strains were measured by a Seahorse XFe96 analyser. Oligo (1.5 μM), oligomycin; FCCP (2 μM), Carbonyl cyanide 4-(trifluoromethoxy) phenylhydrazone; Rot/AA (0.5 μM), Rotenone/antimycin A. (C) Intracellular ATP content in the WT, *gfc1*Δ, and *gfc1*Δ/GFC1 strains cultured on YPG medium. (D) The metabolic activity detected by 2,3-bis (2-methoxy-4-nitro-5-sulfophenyl)-2H-tetrazolium-5-carboxanilide (XTT) assay. Three biological replicates were performed.
(TIF)

S5 Fig Ume6 and Hgc1 are associated with *GFC1*-suppressed filamentous growth in *C*. *auris*. WT, BJCA001. (A) Relative expression levels of *UME6* and *HGC1* in the WT, *gfc1*Δ, and *mcu1*Δ mutant strains. *C*. *auris* cells were grown on YPG medium at 25°C for 9 days, and then collected and lysed for qRT-PCR analysis. The expression level of the WT strain was set as 1. Error bars denote SD. *P < 0.05, (Student’s *t*-test, two tailed). (B) Colony and cellular morphologies of the WT, *gfc1*Δ, *gfc1*Δ/*ume6*Δ, and *gfc1*Δ/*hgc1*Δ mutant strains on YPG medium for 6 or 9 days of growth at 25°C. Scale bar for colonies, 1 mm; Scale bar for cells, 10 μm.
(TIF)

S6 Fig Competitive growth assays of *C*. *auris* WT and *GFC1* mutant strains. WT, BJCA001. *GFC1* mutant strains: *gfc1*Δ, V3, V4. A 50:50 mixture of the WT strain and *gfc1*Δ or evolved RL-FC isolates (V3 or V4) was inoculated into liquid YPG medium for growth at 25°C. The survival rates of the different strains were determined by CFU assays. The WT (yeast-form) and *GFC1* mutant (RL-FC form) cells could be easily distinguished by plating on phloxine B-containing YPG plates. Percentages of the WT and *GFC1* mutant (*gfc1*Δ, V3, or V4.) cells were calculated at different time points as indicated. (A) WT versus *gfc1*Δ; (B) WT versus V3; (C) WT versus V4. Three biological repeats were performed. Error bars denote SD.
(TIF)

S1 Table Strains used in this study. (DOCX)

S2 Table Primers used in this study. (DOCX)

S1 Dataset Protein expression profiles of *C*. *auris* WT, *gfc1*Δ, and *mcu1*Δ mutant strains grown on YPG or YPD medium for 6 days at 25°C. (PDF)

S2 Dataset Fatty acid profiles of *C*. *auris* WT, *gfc1*Δ, and *mcu1*Δ mutant strains grown on YPG or YPD medium for 6 days at 25°C. (PDF)

S3 Dataset Source data file. (XLSX)

## References

[1] Satoh K, Makimura K, Hasumi Y, Nishiyama Y, Uchida K, Yamaguchi H. *Candida auris* sp. nov., a novel ascomycetous yeast isolated from the external ear canal of an inpatient in a Japanese hospital. Microbiol Immunol. 2009;53(1):41–4. doi: 10.1111/j.1348-0421.2008.00083.x 19161556

[2] Du H, Bing J, Hu T, Ennis CL, Nobile CJ, Huang G. *Candida auris*: Epidemiology, biology, antifungal resistance, and virulence. PLoS Pathog. 2020;16(10):e1008921. doi: 10.1371/journal.ppat.1008921 33091071 PMC7581363

[3] Lyman M, Forsberg K, Sexton DJ, Chow NA, Lockhart SR, Jackson BR, et al. Worsening spread of *Candida auris* in the United States, 2019 to 2021. Ann Intern Med. 2023;176(4):489–95. doi: 10.7326/M22-3469 36940442 PMC11307313

[4] Nelson R. Emergence of resistant *Candida auris*. Lancet Microbe. 2023;4(6):e396. doi: 10.1016/S2666-5247(23)00143-X 37150182

[5] Uppuluri P. *Candida auris* biofilm colonization on skin niche conditions. mSphere. 2020;5(1): e00972–19. doi: 10.1128/mSphere.00972-19 31969480 PMC6977181

[6] Kean R, Sherry L, Townsend E, McKloud E, Short B, Akinbobola A, et al. Surface disinfection challenges for *Candida auris*: an in-vitro study. J Hosp Infect. 2018;98(4):433–6. doi: 10.1016/j.jhin.2017.11.015 29203448

[7] Wang P, Heitman J. Signal transduction cascades regulating mating, filamentation, and virulence in *Cryptococcus neoformans*. Curr Opin Microbiol. 1999;2:258–362. doi: 10.1016/S1369-5274(99)80063-0 10458985

[8] Conant NF. Cultural study of the life cycle of Histoplasma capsulatum darling 1906. J Bacteriol. 1941;41:563–79. doi: 10.1128/jb.41.5.563-579.1941 16560424 PMC374721

[9] Klein BS, Tebbets B. Dimorphism and virulence in fungi. Curr Opin Microbiol. 2007;10(4):314–9. doi: 10.1016/j.mib.2007.04.002 17719267 PMC3412142

[10] Pan X, Heitman J. Cyclic AMP-dependent protein kinase regulates pseudohyphal differentiation in Saccharomyces cerevisiae. Mol Cell Biol. 1999;19(7):4874–87. doi: 10.1128/mcb.19.7.4874 10373537 PMC84286

[11] Gancedo JM. Control of pseudohyphae formation in *Saccharomyces cerevisiae*. FEMS Microbiol Rev. 2001;25:107–23. doi: 10.1111/j.1574-6976.2001.tb00573.x 11152942

[12] Sudbery P, Gow N, Berman J. The distinct morphogenic states of *Candida albicans*. Trends Microbiol. 2004;12(7):317–24. doi: 10.1016/j.tim.2004.05.008 15223059

[13] Huang G. Regulation of phenotypic transitions in the fungal pathogen *Candida albicans*. Virulence. 2012;3(3):251–61. doi: 10.4161/viru.20010 22546903 PMC3442837

[14] Slutsky B, Staebell M, Anderson J, Risen L, Pfaller M, Soll DR. "White-opaque transition": a second high-frequency switching system in *Candida albicans*. J Bacteriol 1987;169:189–97. doi: 10.1128/jb.169.1.189-197.1987 3539914 PMC211752

[15] Zhang Q, Tao L, Guan G, Yue H, Liang W, Cao C, et al. Regulation of filamentation in the human fungal pathogen *Candida tropicalis*. Mol Microbiol. 2016;99(3):528–45. 26466925 10.1111/mmi.13247

[16] Porman AM, Alby K, Hirakawa MP, Bennett RJ. Discovery of a phenotypic switch regulating sexual mating in the opportunistic fungal pathogen *Candida tropicalis*. Proc Natl Acad Sci U S A. 2011;108(52):21158–63. doi: 10.1073/pnas.1112076109 22158989 PMC3248515

[17] Pujol C, Daniels KJ, Lockhart SR, Srikantha T, Radke JB, Geiger J, et al. The closely related species *Candida albicans* and *Candida dubliniensis* can mate. Eukaryot Cell. 2004;3(4):1015–27. doi: 10.1128/EC.3.4.1015-1027.2004 15302834 PMC500882

[18] Gilfillan GD SD, Haynes K, Parkinson T, Coleman DC, Gow NA. *Candida dubliniensis* phylogeny and putative virulence factors. Microbiology. 1998;144:829–38. doi: 10.1099/00221287-144-4-829 9579058

[19] Soll DR. Why does *Candida albicans* switch? FEMS Yeast Res. 2009;9(7):973–89. doi: 10.1111/j.1567-1364.2009.00562.x 19744246

[20] Whiteway M, Bachewich C. Morphogenesis in *Candida albicans*. Annu Rev Microbiol. 2007;61(1):529–53. doi: 10.1146/annurev.micro.61.080706.093341 17506678 PMC4452225

[21] Noble SM, Gianetti BA, Witchley JN. *Candida albicans* cell-type switching and functional plasticity in the mammalian host. Nat Rev Microbiol. 2017;15(2):96–108. doi: 10.1038/nrmicro.2016.157 27867199 PMC5957277

[22] Biswas S, Van Dijck P, Datta A. Environmental sensing and signal transduction pathways regulating morphopathogenic determinants of *Candida albicans*. Microbiol Mol Biol Rev. 2007;71(2):348–76. doi: 10.1128/MMBR.00009-06 17554048 PMC1899878

[23] Lohse MB, Johnson AD. White–opaque switching in *Candida albicans*. Curr Opin Microbiol. 2009;12(6):650–4. doi: 10.1016/j.mib.2009.09.010 19853498 PMC2812476

[24] Xie J, Tao L, Nobile CJ, Tong Y, Guan G, Sun Y, et al. White-opaque switching in natural MTLa/α isolates of *Candida albicans*: evolutionary implications for roles in host adaptation, pathogenesis, and sex. PLoS Biol. 2013;11(3):e1001525. doi: 10.1371/journal.pbio.1001525 23555196 PMC3608550

[25] Simonetti N, Strippoli V, Cassone A. Yeast-mycelial conversion induced by N-acetyl-D-glucosamine in *Candida albicans*. Nature. 1974;250:344–6. doi: 10.1038/250344a0 4605454

[26] Sims W. Effect of carbon dioxide on the growth and form of *Candida albicans*. J Med Microbiol. 1986;22:203–8. doi: 10.1099/00222615-22-3-203 3095550

[27] Ramon AM, Porta A, Fonzi WA. Effect of environmental pH on morphological development of *Candida albicans* is mediated via the PacC-related transcription factor encoded by PRR2. J Bacteriol. 1999;181:7524–30. doi: 10.1128/jb.181.24.7524-7530.1999 10601210 PMC94210

[28] Xu X, Lee RTH, Fang H, Wang Y, Li R, Zou H, et al. Bacterial peptidoglycan triggers *Candida albicans* hyphal growth by directly activating the adenylyl cyclase Cyr1p. Cell Host Microbe. 2008;4(1):28–39. doi: 10.1016/j.chom.2008.05.014 18621008

[29] Wang X, Bing J, Zheng Q, Zhang F, Liu J, Yue H, et al. The first isolate of Candida auris in China: clinical and biological aspects. Emerg Microbes Infect. 2018;7(1):1–9. doi: 10.1038/s41426-018-0095-0 29777096 PMC5959928

[30] Saris K, Meis JF, Voss A. Candida auris. Curr Opin Infect Dis. 2018;31(4):334–40. doi: 10.1097/QCO.0000000000000469 29878905

[31] Spivak ES, Hanson KE. Candida auris an emerging fungal pathogen. J Clin Microbiol. 2018;56(2):e01588–17. doi: 10.1128/JCM.01588-17 29167291 PMC5786713

[32] Yue H, Bing J, Zheng Q, Zhang Y, Hu T, Du H, et al. Filamentation in *Candida auris*, an emerging fungal pathogen of humans: passage through the mammalian body induces a heritable phenotypic switch. Emerg Microbes Infect. 2018;7(1):1–13. doi: 10.1038/s41426-018-0187-x 30482894 PMC6258701

[33] Bravo Ruiz G, Ross ZK, Gow NAR, Lorenz A, Mitchell AP. Pseudohyphal growth of the emerging pathogen *Candida auris* is triggered by genotoxic stress through the S phase checkpoint. mSphere. 2020;5(2):e00151–20. doi: 10.1128/mSphere.00151-20 32161147 PMC7067593

[34] Gao J, Chow EWL, Wang H, Xu X, Cai C, Song Y, et al. LncRNA DINOR is a virulence factor and global regulator of stress responses in *Candida auris*. Nat Microbiol. 2021;6(7):842–51. doi: 10.1038/s41564-021-00915-x 34083769

[35] Santana DJ, O’Meara TR. Forward and reverse genetic dissection of morphogenesis identifies filament-competent *Candida auris* strains. Nat Commun. 2021;12(1):7197. doi: 10.1038/s41467-021-27545-5 34893621 PMC8664941

[36] Lockhart SR, Etienne KA, Vallabhaneni S, Farooqi J, Chowdhary A, Govender NP, et al. Simultaneous emergence of multidrug-resistant *Candida auris* on 3 continents confirmed by whole-genome sequencing and epidemiological analyses. Clin Infect Dis. 2017;64(2):134–40. doi: 10.1093/cid/ciw691 27988485 PMC5215215

[37] Day AM, McNiff MM, da Silva Dantas A, Gow NAR, Quinn J. Hog1 regulates stress tolerance and virulence in the emerging fungal pathogen *Candida auris*. mSphere. 2018;3(5):e00506–18. doi: 10.1128/mSphere.00506-18 30355673 PMC6200985

[38] Horton MV, Johnson CJ, Kernien JF, Patel TD, Lam BC, Cheong JZA, et al. *Candida auris* forms high-burden biofilms in skin niche conditions and on porcine skin. mSphere. 2020;5(1):e00910–19. doi: 10.1128/mSphere.00910-19 31969479 PMC6977180

[39] Biswas C, Wang Q, van Hal SJ, Eyre DW, Hudson B, Halliday CL, et al. Genetic heterogeneity of Australian *Candida auris* isolates: insights from a nonoutbreak setting using whole-genome sequencing. Open Forum Infect Di. 2020;7(5):ofaa158. doi: 10.1093/ofid/ofaa158 32500091 PMC7255648

[40] Bing J, Hu T, Zheng Q, Muñoz JF, Cuomo CA, Huang G. Experimental evolution identifies adaptive aneuploidy as a mechanism of fluconazole resistance in *Candida auris*. Antimicrob Agents Chemother. 2020;65(1):e01466–20. doi: 10.1128/AAC.01466-20 33077664 PMC7927865

[41] Burrack LS, Todd RT, Soisangwan N, Wiederhold NP, Selmecki A. Genomic diversity across *Candida auris* clinical isolates shapes rapid development of antifungal resistance in vitro and in vivo. mBio. 2022;13(4):e0084222. doi: 10.1128/mbio.00842-22 35862787 PMC9426540

[42] Park Y, Morschhäuser J. Tetracycline-inducible gene expression and gene deletion in *Candida albicans*. Eukaryot Cell. 2005;4(8):1328–42. doi: 10.1128/EC.4.8.1328-1342.2005 16087738 PMC1214539

[43] Tehlivets O, Scheuringer K, Kohlwein SD. Fatty acid synthesis and elongation in yeast. Biochim Biophys Acta—Molecular and Cell Biology of Lipids. 2007;1771(3):255–70. doi: 10.1016/j.bbalip.2006.07.004 16950653

[44] Mishra P, Bolard J, Prasad R. Emerging role of lipids of *Candida aibicans*, a pathogenic dimorphic yeast. Biochim Biophys Acta. 1992;1127:1–14. doi: 10.1016/0005-2760(92)90194-z 1627629

[45] Krishnamurthy S, Plaine A, Albert J, Prasad T, Prasad R, Ernst JF. Dosage-dependent functions of fatty acid desaturase Ole1p in growth and morphogenesis of Candida albicans. Microbiology. 2004;150(6):1991–2003. doi: 10.1099/mic.0.27029-0 15184585

[46] Zhao XJ, McElhaney-Feser GE, Bowen WH, Cole MF, Broedel SE, Cihlar RL. Requirement for the *Candida albicans FAS2* gene for infection in a rat model of oropharyngeal candidiasis. Microbiology. 1996;142:2509–14. doi: 10.1099/00221287-142-9-2509 8828218

[47] Zhao XJ, McElhaney-Feser GE, Sheridan MJ, Broedel SE, Cihlar RL. Avirulence of *Candida albicans FAS2* mutants in a mouse model of systemic candidiasis. Infect Immun. 1997;65:829–32. doi: 10.1128/iai.65.2.829-832.1997 9009352 PMC176135

[48] Xu D, Sillaots S, Davison J, Hu W, Jiang B, Kauffman S, et al. Chemical genetic profiling and characterization of small-molecule compounds that affect the biosynthesis of unsaturated fatty acids in *Candida albicans*. J Biol Chem. 2009;284(29):19754–64. doi: 10.1074/jbc.M109.019877 19487691 PMC2740599

[49] Guan G, Wang H, Liang W, Cao C, Tao L, Naseem S, et al. The mitochondrial protein Mcu1 plays important roles in carbon source utilization, filamentation, and virulence in *Candida albicans*. Fungal Genet Biol. 2015;81:150–9. doi: 10.1016/j.fgb.2015.01.006 25626172

[50] Carlisle PL, Kadosh D. *Candida albicans Ume6*, a filament-specific transcriptional regulator, directs hyphal growth via a pathway involving Hgc1 cyclin-related protein. Eukaryot Cell. 2010;9(9):1320–8. doi: 10.1128/EC.00046-10 20656912 PMC2937344

[51] Banerjee M, Thompson DS, Lazzell A, Carlisle PL, Pierce C, Monteagudo C, et al. *UME6*, a novel filament-specific regulator of *Candida albicans* hyphal extension and virulence. Mol Biol Cell. 2008;19(4):1354–65. doi: 10.1091/mbc.E07-11-1110 18216277 PMC2291399

[52] Zheng X, Wang Y, Wang Y. Hgc1, a novel hypha-specific G1 cyclin-related protein regulates *Candida albicans* hyphal morphogenesis. EMBO J. 2004;23(8):1845–56. doi: 10.1038/sj.emboj.7600195 15071502 PMC394249

[53] Fisher MC, Henk DA, Briggs CJ, Brownstein JS, Madoff LC, McCraw SL, et al. Emerging fungal threats to animal, plant and ecosystem health. Nature. 2012;484(7393):186–94. doi: 10.1038/nature10947 22498624 PMC3821985

[54] Casadevall A, Kontoyiannis DP, Robert V, Kronstad JW. On the emergence of *Candida auris*: climate change, azoles, swamps, and birds. mBio. 2019;10(4):e01397–19. doi: 10.1128/mBio.01397-19 31337723 PMC6650554

[55] Welsh RM, Bentz ML, Shams A, Houston H, Lyons A, Rose LJ, et al. Survival, persistence, and isolation of the emerging multidrug-resistant pathogenic yeast *Candida auris* on a plastic health care surface. J Clin Microbiol. 2017;55(10):2996–3005. doi: 10.1128/JCM.00921-17 28747370 PMC5625385

[56] Tso GHW, Reales-Calderon JA, Tan ASM, Sem X, Le GTT, Tan TG, et al. Experimental evolution of a fungal pathogen into a gut symbiont. Science. 2018;362:589–95. doi: 10.1126/science.aat0537 30385579

[57] Murante D, Demers EG, Kurbessoian T, Ruzic M, Ashare A, Stajich JE, et al. Mrs4 loss of function in fungi during adaptation to the cystic fibrosis lung. mBio. 2023;14(4):e0117123. doi: 10.1128/mbio.01171-23 37432019 PMC10470810

[58] Rybak JM, Muñoz JF, Barker KS, Parker JE, Esquivel BD, Berkow EL, et al. Mutations in TAC1B: a novel genetic determinant of clinical fluconazole resistance in *Candida auris*. mBio. 2020;11(3):e00365–20. doi: 10.1128/mBio.00365-20 32398311 PMC7218281

[59] Fan S, Li C, Bing J, Huang G, Du H. Discovery of the diploid form of the emerging fungal pathogen *Candida auris*. ACS Infect Dis. 2020;6(10):2641–6. doi: 10.1021/acsinfecdis.0c00282 32902947

[60] Williams RB, Lorenz MC. Multiple alternative carbon pathways combine to promote *Candida albicans* stress resistance, immune interactions, and virulence. mBio. 2020;11(1):e03070–19. doi: 10.1128/mBio.03070-19 31937647 PMC6960290

[61] Lavarde V, Daniel F, Saez H, Arnold M, Faguer B. Peritonite mycosique a Torulopsis haemulonii. Bull Soc Fr Mycol Med. 1984;13:173–6.

[62] Chowdhary A, Sharma C, Duggal S, Agarwal K, Prakash A, Singh PK, et al. New clonal strain of *Candida auris*, Delhi, India. Emerg Infect Dis. 2013;19(10):1670–3. doi: 10.3201/eid1910.130393 24048006 PMC3810747

[63] Deng Y, Li S, Bing J, Liao W, Tao L. Phenotypic switching and filamentation in *Candida haemulonii* an emerging opportunistic pathogen of humans. Microbiol Spectr. 2021;9(3): e0077921. doi: 10.1128/Spectrum.00779-21 34878301 PMC8653834

[64] Rupert CB, Rusche LN. The pathogenic yeast *Candida parapsilosis* forms pseudohyphae through different signaling pathways depending on the available carbon source. mSphere. 2022;7(3):e0002922. doi: 10.1128/msphere.00029-22 35766504 PMC9241547

[65] Desai JV, Bruno VM, Ganguly S, Stamper RJ, Mitchell KF, Solis N, et al. Regulatory role of glycerol in *Candida albicans* biofilm formation. mBio. 2013;4(2):e00637–12. doi: 10.1128/mBio.00637-12 23572557 PMC3622937

[66] Chopra A, Khuller G. K. Lipid of pathogenic fungi. Prog Lipid Res. 1983;22:189–220. doi: 10.1016/0163-7827(83)90009-7 6356150

[67] Klose J, De Sá MM, Kronstad JW. Lipid-induced filamentous growth in *Ustilago maydis*. Mol Microbiol. 2004;52(3):823–35. doi: 10.1111/j.1365-2958.2004.04019.x 15101987

[68] Marty AJ, Broman AT, Zarnowski R, Dwyer TG, Bond LM, Lounes-Hadj Sahraoui A, et al. Fungal morphology, iron homeostasis, and lipid metabolism regulated by a GATA transcription factor in *Blastomyces dermatitidis*. PLoS Pathog. 2015;11(6):e1004959. doi: 10.1371/journal.ppat.1004959 26114571 PMC4482641

[69] Nobile CJ, Mitchell AP. Regulation of cell-surface genes and biofilm formation by the *C*. *albicans* transcription factor Bcr1p. Curr Biol. 2005;15(12):1150–5. doi: 10.1016/j.cub.2005.05.047 15964282

[70] Guan G, Xie J, Tao L, Nobile CJ, Sun Y, Cao C, et al. Bcr1 plays a central role in the regulation of opaque cell filamentation in *Candida albicans*. Mol Microbiol. 2013;89(4):732–50. doi: 10.1111/mmi.12310 23808664 PMC3758918

[71] Si H, Hernday AD, Hirakawa MP, Johnson AD, Bennett RJ. *Candida albicans* white and opaque cells undergo distinct programs of filamentous growth. PLoS Pathog. 2013;9(3): e1003210. doi: 10.1371/journal.ppat.1003210 23505370 PMC3591317

[72] Lan CY, Newport G, Murillo LA, Jones T, Scherer S, Davis RW, et al. Metabolic specialization associated with phenotypic switching in *Candida albicans*. Proc Natl Acad Sci U S A. 2002;99:14907–12. doi: 10.1073/pnas.232566499 12397174 PMC137518

[73] Noble SM, Johnson AD. Strains and strategies for large-scale gene deletion studies of the diploid human fungal pathogen *Candida albicans*. Eukaryotic Cell. 2005;4(2):298–309. doi: 10.1128/EC.4.2.298-309.2005 15701792 PMC549318

[74] Bing J, Guan Z, Zheng T, Zhang Z, Fan S, Ennis CL, et al. Clinical isolates of *Candida auris* with enhanced adherence and biofilm formation due to genomic amplification of *ALS4*. PLoS Pathog. 2023;19(3):e1011239. doi: 10.1371/journal.ppat.1011239 36913408 PMC10035925

[75] Li H, Durbin R. Fast and accurate short read alignment with Burrows–Wheeler transform. Bioinformatics. 2009;25(14):1754–60. doi: 10.1093/bioinformatics/btp324 19451168 PMC2705234

[76] Li H, Handsaker B, Wysoker A, Fennell T, Ruan J, Homer N, et al. The sequence alignment/map format and SAMtools. Bioinformatics. 2009;25(16):2078–9. doi: 10.1093/bioinformatics/btp352 19505943 PMC2723002

[77] DePristo MA, Banks E, Poplin R, Garimella KV, Maguire JR, Hartl C, et al. A framework for variation discovery and genotyping using next-generation DNA sequencing data. Nat Genet. 2011;43(5):491–8. doi: 10.1038/ng.806 21478889 PMC3083463

[78] Kvaal C, Lachke SA, Srikantha T, Daniels K, McCoy J, Soll DR. Misexpression of the opaque-phase-specific genePEP1 (SAP1) in the white phase of *Candida albicans* confers increased virulence in a mouse model of cutaneous infection. Infect Immun. 1999;67(12):6652–62. doi: 10.1128/iai.67.12.6652-6662.1999 10569787 PMC97079

[79] Li S, Zhao Y, Zhang Y, Zhang Y, Zhang Z, Tang C, et al. The δ subunit of F1Fo-ATP synthase is required for pathogenicity of *Candida albicans*. Nat Commun. 2021;12(1):6041. doi: 10.1038/s41467-021-26313-9 34654833 PMC8519961

[80] Wu Z, Huang J, Huang J, Li Q, Zhang X. Lys-C/Arg-C, a more specific and efficient digestion approach for proteomics studies. Anal Chem. 2018;90(16):9700–7. doi: 10.1021/acs.analchem.8b02448 30024741

[81] Kovalchuk SI, Jensen ON, Rogowska-Wrzesinska A. FlashPack: Fast and simple preparation of ultrahigh-performance capillary columns for LC-MS. Mol Cell Proteomics. 2019;18(2):383–90. doi: 10.1074/mcp.TIR118.000953 30373789 PMC6356079

[82] Zhang X, Smits AH, van Tilburg GBA, Ovaa H, Huber W, Vermeulen M. Proteome-wide identification of ubiquitin interactions using UbIA-MS. Nat Protoc. 2018;13(3):530–50. doi: 10.1038/nprot.2017.147 29446774

