# Supplementary figures and images for: A single gene mutation underpins metabolic adaptation and acquisition of filamentous competence in the emerging fungal pathogen Candida auris
Source: PLoS Pathog. 2024 Jul 8;20(7):e1012362. doi: 10.1371/journal.ppat.1012362 (PMC11257696; doi:10.1371/journal.ppat.1012362)

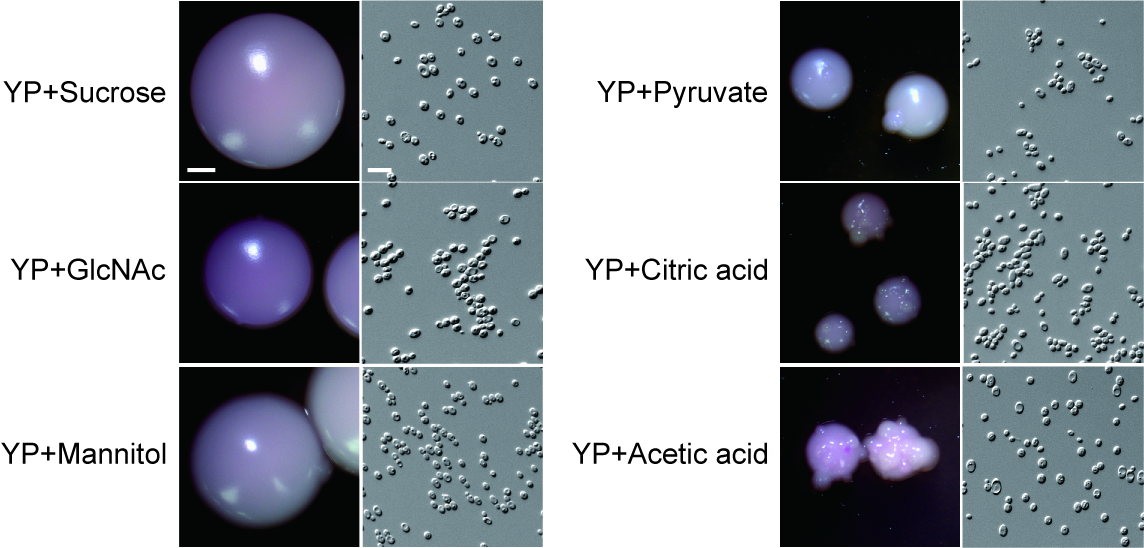

Supplement: S1 Fig — Y (Yeast extract) P (Peptone) + different carbon source: Sucrose, N-acetylglucosamine GlcNAc, Mannitol, Pyruvate, Citric acid, Acetic acid. Scale bar for colonies, 1 mm; Scale bar for cells, 10 μm. The strain used was BJCA001. (TIF) [file ppat.1012362.s001.tif]

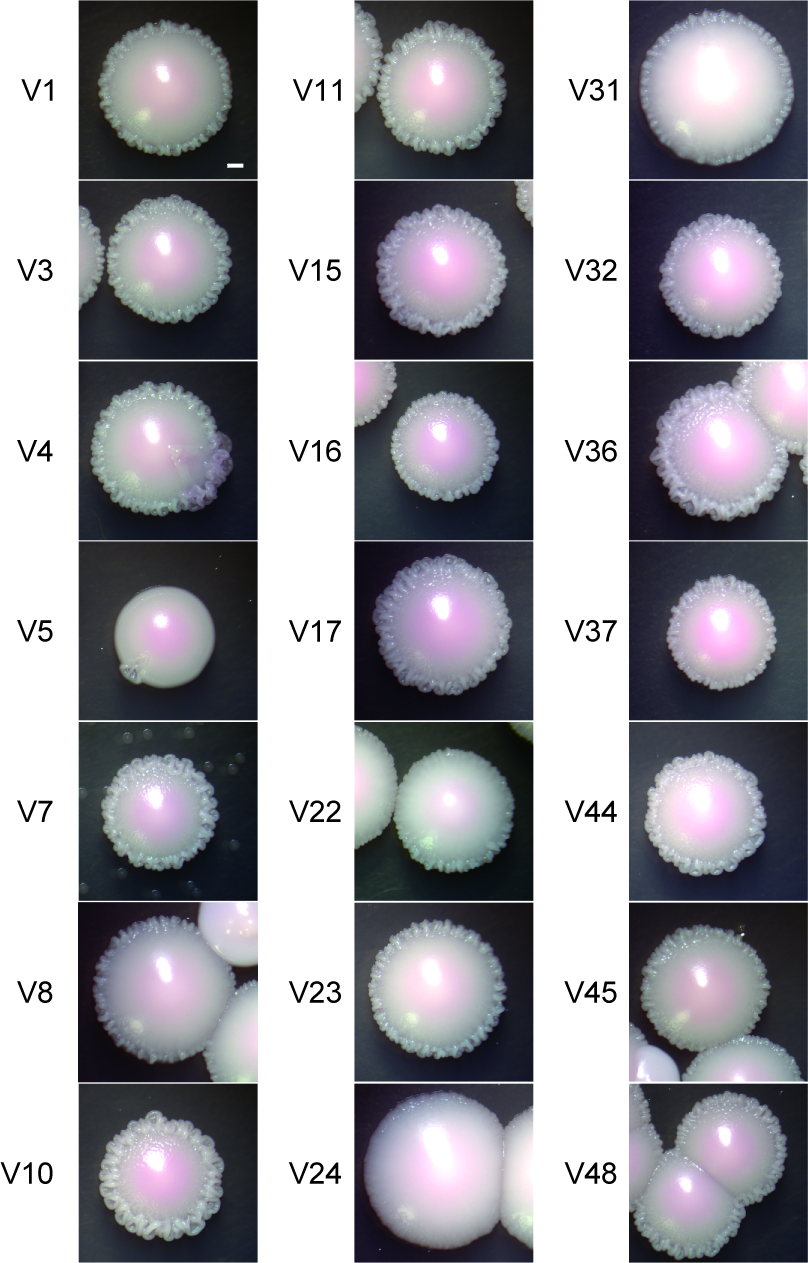

Supplement: S2 Fig — 21 C. auris isolates were grown on YPG medium for 9 days at 25°C. Details for mutations are described in S1 Table. Scale bar: 1 mm. (TIF) [file ppat.1012362.s002.tif]

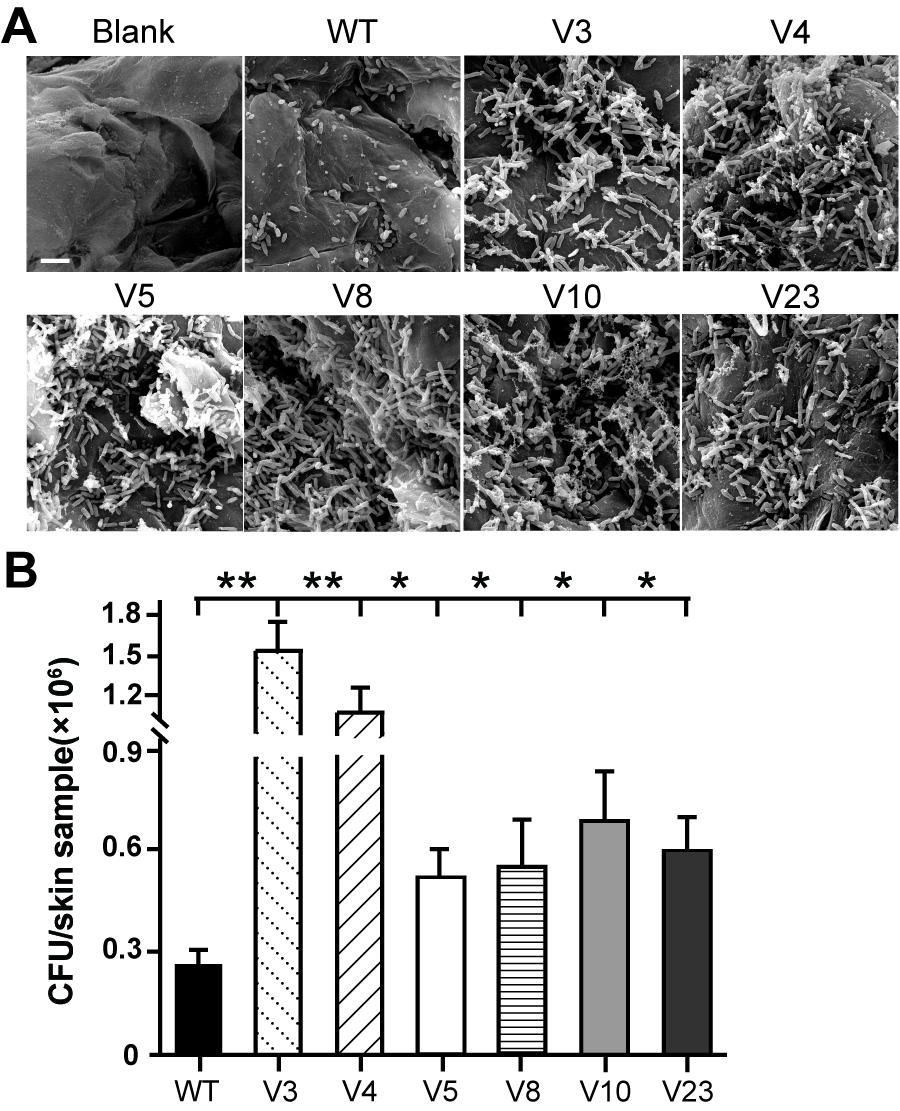

Supplement: S3 Fig — Approximately 2 × 10^6^ C. auris cells of the WT and GFC1 variants-containing isolates V3 (152G>T), V4 (687_696dupTCGCACCGCT), V5 (86G>A), V8 (347delC), V10 (723_787dupGGGGTCTCTAGCTCCCGCCGGAGCCTCTTGGAGCTTAGGGTCAGGGTCAGGGCCAGGGTCAGGCT), and V23 (255_256insC) in 2 μL PBS were spotted on the dorsal back skin of newborn mice. After the skin surface dried, a small sterilized glossy paper was affixed on the inoculated spot with medical tape. WT, BJCA001. (A) Scanning electron microscope (SEM) images of the infected skin samples. After 3 days of infection, the infected skin areas were excised, gently washed with 1 × PBS, and fixed with 2.5% glutaraldehyde for SEM assays. Scale bar, 10 μm. (B) Fungal burdens of the WT and GFC1 variants-containing isolates on skin. After 3 days of infection, the infected skin areas were excised, homogenized and then plated onto YPD mdeia for CFU assays. The experiment was repeated three times. For each time, three skin samples were used for each strain. The result of a representative experiment is shown. Error bars denote the standard deviation (SD). *P < 0.05, **P < 0.01, (Student’s t-test, two tailed). (TIF) [file ppat.1012362.s003.tif]

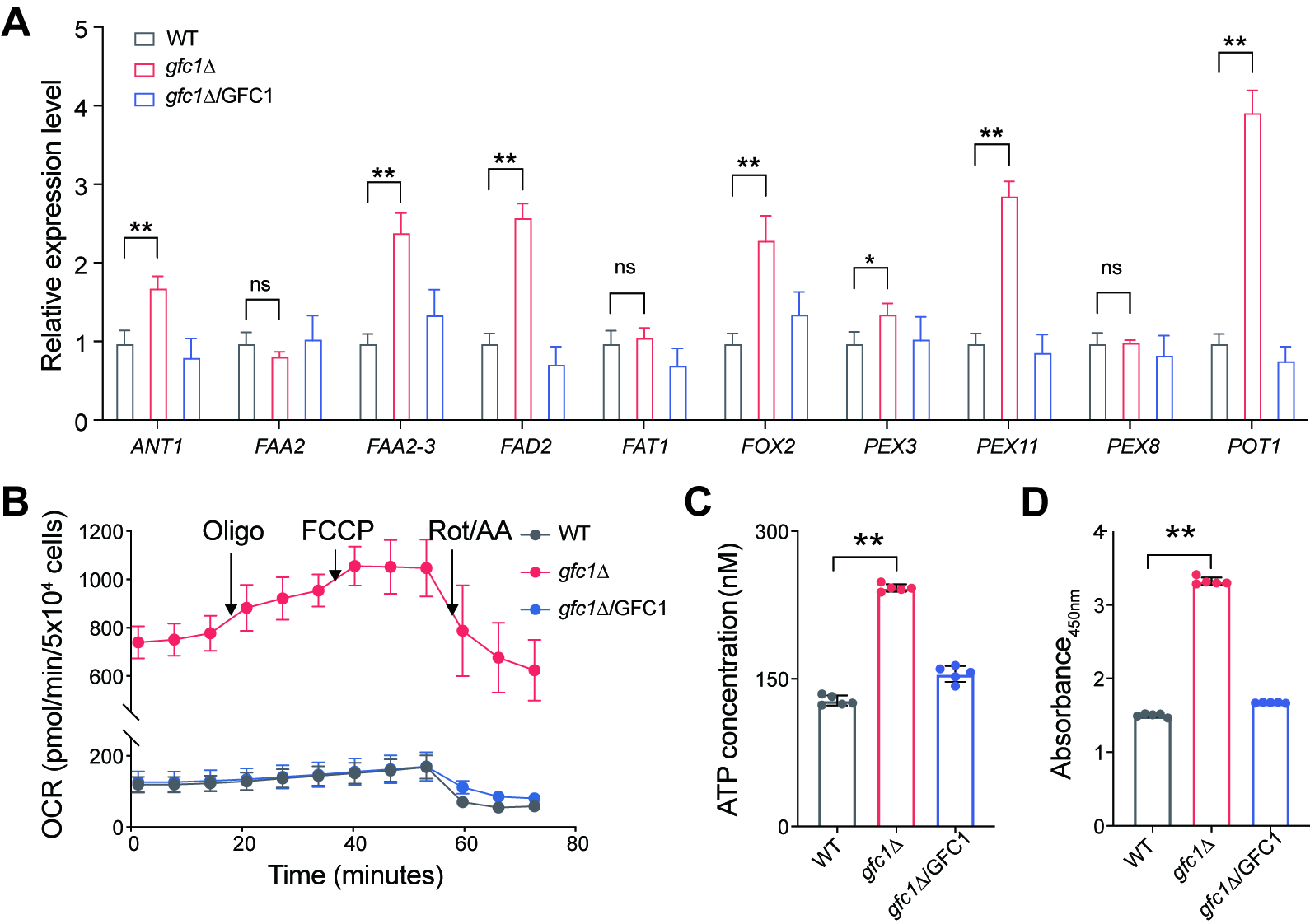

Supplement: S4 Fig — C. auris cells were grown on YPG medium at 25°C for 6 days. Data are shown as the mean ± SD of three independent experiments. Error bars denote SD. For A, B and C, *P < 0.05, **P < 0.01 (Student’s t-test, two tailed). WT, BJCA001. (A) Relative expression levels of genes involved in fatty acid mechanism in the WT, gfc1Δ, and gfc1ΔGFC1 strains. Cells were collected and lysed for qRT-PCR analysis. The expression level of the WT strain for each gene was set as 1. ns not significant. (B) Oxygen consumption rate (OCR) in the WT, gfc1Δ, and gfc1Δ/GFC1 strains were measured by a Seahorse XFe96 analyser. Oligo (1.5 μM), oligomycin; FCCP (2 μM), Carbonyl cyanide 4-(trifluoromethoxy) phenylhydrazone; Rot/AA (0.5 μM), Rotenone/antimycin A. (C) Intracellular ATP content in the WT, gfc1Δ, and gfc1Δ/GFC1 strains cultured on YPG medium. (D) The metabolic activity detected by 2,3-bis (2-methoxy-4-nitro-5-sulfophenyl)-2H-tetrazolium-5-carboxanilide (XTT) assay. Three biological replicates were performed. (TIF) [file ppat.1012362.s004.tif]

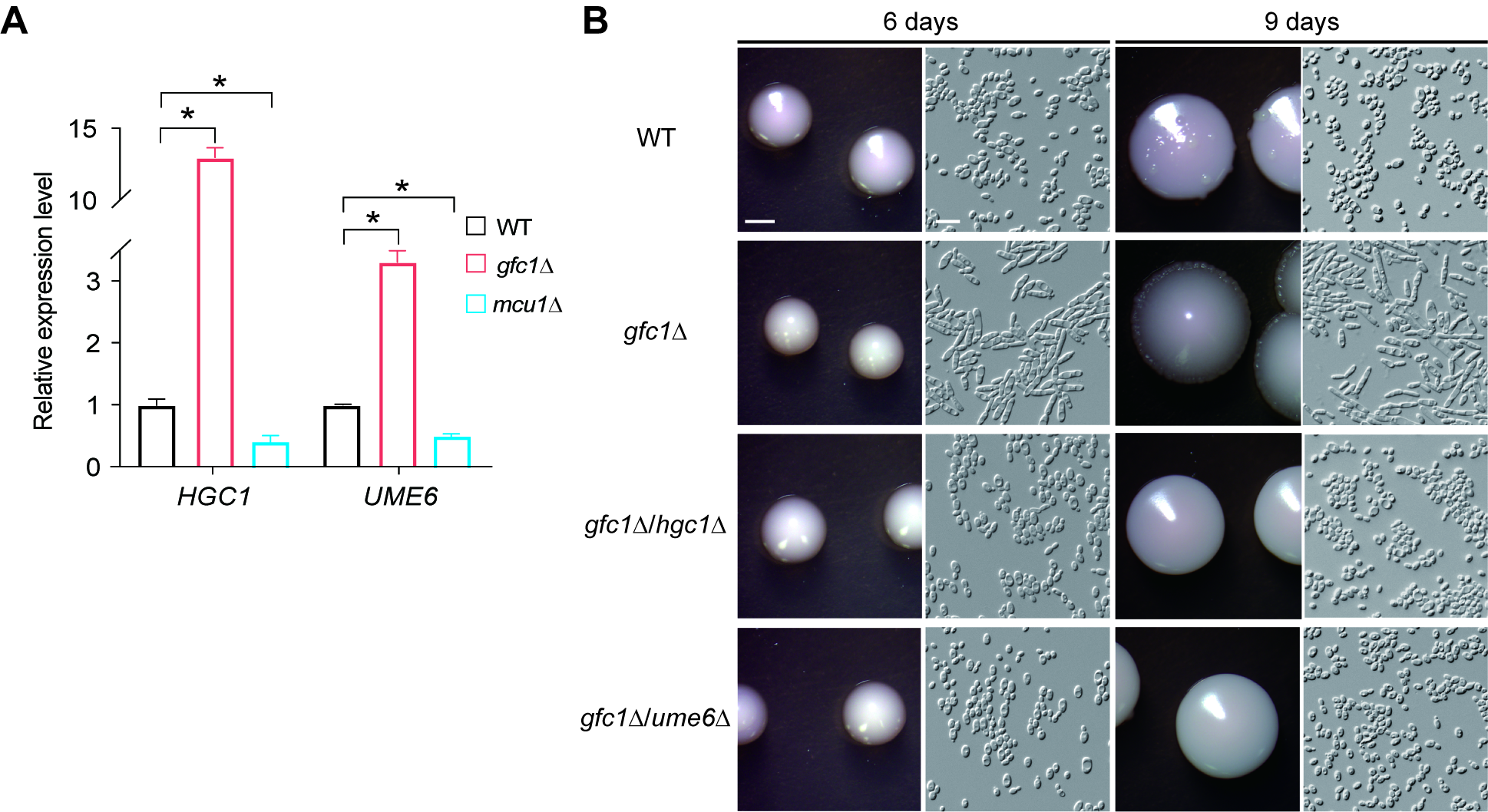

Supplement: S5 Fig — WT, BJCA001. (A) Relative expression levels of UME6 and HGC1 in the WT, gfc1Δ, and mcu1Δ mutant strains. C. auris cells were grown on YPG medium at 25°C for 9 days, and then collected and lysed for qRT-PCR analysis. The expression level of the WT strain was set as 1. Error bars denote SD. *P < 0.05, (Student’s t-test, two tailed). (B) Colony and cellular morphologies of the WT, gfc1Δ, gfc1Δ/ume6Δ, and gfc1Δ/hgc1Δ mutant strains on YPG medium for 6 or 9 days of growth at 25°C. Scale bar for colonies, 1 mm; Scale bar for cells, 10 μm. (TIF) [file ppat.1012362.s005.tif]

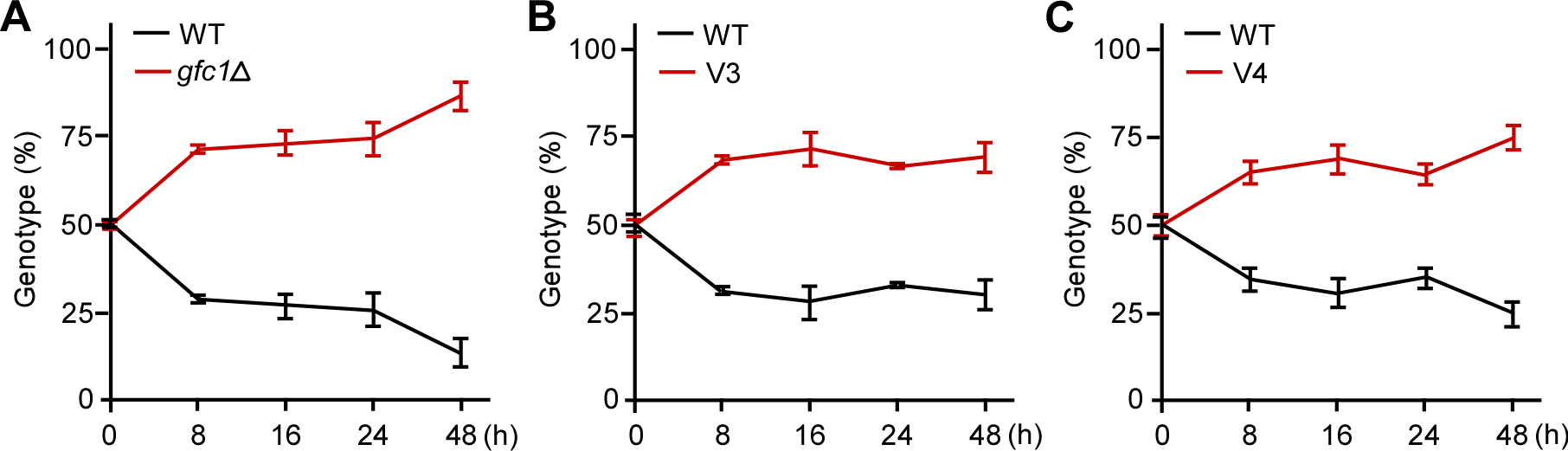

Supplement: S6 Fig — WT, BJCA001. GFC1 mutant strains: gfc1Δ, V3, V4. A 50:50 mixture of the WT strain and gfc1Δ or evolved RL-FC isolates (V3 or V4) was inoculated into liquid YPG medium for growth at 25°C. The survival rates of the different strains were determined by CFU assays. The WT (yeast-form) and GFC1 mutant (RL-FC form) cells could be easily distinguished by plating on phloxine B-containing YPG plates. Percentages of the WT and GFC1 mutant (gfc1Δ, V3, or V4.) cells were calculated at different time points as indicated. (A) WT versus gfc1Δ; (B) WT versus V3; (C) WT versus V4. Three biological repeats were performed. Error bars denote SD. (TIF) [file ppat.1012362.s006.tif]
