# Supplementary material for: A single gene mutation underpins metabolic adaptation and acquisition of filamentous competence in the emerging fungal pathogen Candida auris
Source: PLoS Pathog. 2024 Jul 8;20(7):e1012362. doi: 10.1371/journal.ppat.1012362 (PMC11257696; doi:10.1371/journal.ppat.1012362)
Supplement: S1 Table — (DOCX) [file ppat.1012362.s007.docx]

**S1 Table. Strains used in this study**

| Strain name | Parent strain | Genotype | Reference |
| --- | --- | --- | --- |
| FDYC0100 | BJCA001 | WT | [1] |
| FDYC0194 | BJCA001 | As BJCA001, but *arg4::FRT* | This study |
| FDYC0231 | FDYC0194 | As FDYC0194，but *gfc1::FRT* | This study |
| FDYC0747 | FDYC0231 | As FDYC0231, but *GFCp-GFC1-ARG4* | This study |
| FDYC0582 | FDYC0194 | As FDYC0194，but *mcu1::FRT-SAT1-FRT* | This study |
| FDYC0952 | FDYC0231 | As FDYC0231, but *mcu1::FRT-SAT1-FRT* | This study |
| FDYC0953 | FDYC0231 | As FDYC0231, but *hgc1::ARG4* | This study |
| FDYC0954 | FDYC0231 | As FDYC0231, but *ume6::ARG4* | This study |
| V1 | BJCA001 | As BJCA001, but g.687_696dupTCGCACCGCT, [p.P233fs] | This study |
| V2 | BJCA001 | As BJCA001, but g.687_696dupTCGCACCGCT, [p.P233fs] | This study |
| V3 | BJCA001 | As BJCA001, but g.152G>T, [p.C51F] | This study |
| V4 | BJCA001 | As BJCA001, but g.687_696dupTCGCACCGCT, [p.P233fs] | This study |
| V5 | BJCA001 | As BJCA001, but g.86G>A, [p.R29H] | This study |
| V6 | BJCA001 | As BJCA001, but g.687_696dupTCGCACCGCT, [p.P233fs] | This study |
| V7 | BJCA001 | As BJCA001, but g.687_696dupTCGCACCGCT, [p.P233fs] | This study |
| V8 | BJCA001 | As BJCA001, but g.347delC, [p.P116fs] | This study |
| V9 | BJCA001 | As BJCA001, but g.687_696dupTCGCACCGCT, [p.P233fs] | This study |
| V10 | BJCA001 | As BJCA001, but g.723_787dupGGGGTCTCTAGCTCCCGCCGGAGCCTCTTGGAGCTTAGGGTCAGGGTCAGGGCCAGGGTCAGGCT, [p.L265fs] | This study |
| V11 | BJCA001 | As BJCA001, but g.687_696dupTCGCACCGCT, [p.P233fs] | This study |
| V12 | BJCA001 | As BJCA001, but g.687_696dupTCGCACCGCT, [p.P233fs] | This study |
| V13 | BJCA001 | As BJCA001, but g.687_696dupTCGCACCGCT, [p.P233fs] | This study |
| V14 | BJCA001 | As BJCA001, but g.687_696dupTCGCACCGCT, [p.P233fs] | This study |
| V15 | BJCA001 | As BJCA001, but g.258C>A, [p.Y86*] | This study |
| V16 | BJCA001 | As BJCA001, but g.687_696delTCGCACCGCT, [p.R230fs] | This study |
| V17 | BJCA001 | As BJCA001, but g.104G>A, [p.R35H] | This study |
| V18 | BJCA001 | As BJCA001, but g.104G>A, [p.R35H] | This study |
| V19 | BJCA001 | As BJCA001, but g.687_696dupTCGCACCGCT, [p.P233fs] | This study |
| V20 | BJCA001 | As BJCA001, but g.687_696dupTCGCACCGCT, [p.P233fs] | This study |
| V21 | BJCA001 | As BJCA001, but g.687_696dupTCGCACCGCT, [p.P233fs] | This study |
| V22 | BJCA001 | As BJCA001, but g.687_696dupTCGCACCGCT, [p.P233fs] | This study |
| V23 | BJCA001 | As BJCA001, but g.255_256insC, [p.Y86fs] | This study |
| V24 | BJCA001 | As BJCA001, but g.752G>A, [p.W251*] | This study |
| V25 | BJCA001 | As BJCA001, but g.255_256insC, [p.Y86fs] | This study |
| V26 | BJCA001 | As BJCA001, but g.258C>A, [p.Y86*] | This study |
| V27 | BJCA001 | As BJCA001, but g.687_696dupTCGCACCGCT, [p.P233fs] | This study |
| V28 | BJCA001 | As BJCA001, but g.255_256insC, [p.Y86fs] | This study |
| V29 | BJCA001 | As BJCA001, but g.687_696dupTCGCACCGCT, [p.P233fs] | This study |
| V30 | BJCA001 | As BJCA001, but g.687_696dupTCGCACCGCT, [p.P233fs] | This study |
| V31 | BJCA001 | As BJCA001, but g.435C>A, [p.Y145*] | This study |
| V32 | BJCA001 | As BJCA001, but g.255_256insC, [p.Y86fs] | This study |
| V33 | BJCA001 | As BJCA001, but g.170G>T, [p.R57L] | This study |
| V34 | BJCA001 | As BJCA001, but g.258C>A, [p.Y86*] | This study |
| V35 | BJCA001 | As BJCA001, but g.196C>T, [p.Q66*] | This study |
| V36 | BJCA001 | As BJCA001, but g.255_256insC, [p.Y86fs] | This study |
| V37 | BJCA001 | As BJCA001, but g.687_696dupTCGCACCGCT, [p.P233fs] | This study |
| V38 | BJCA001 | As BJCA001, but g.255_256insC, [p.Y86fs] | This study |
| V39 | BJCA001 | As BJCA001, but g.687_696dupTCGCACCGCT, [p.P233fs] | This study |
| V40 | BJCA001 | As BJCA001, but g.67T>A, [p.C23S] | This study |
| V41 | BJCA001 | As BJCA001, but g.687_696dupTCGCACCGCT, [p.P233fs] | This study |
| V42 | BJCA001 | As BJCA001, but g.687_696dupTCGCACCGCT, [p.P233fs] | This study |
| V43 | BJCA001 | As BJCA001, but g.255_256insC, [p.Y86fs] | This study |
| V44 | BJCA001 | As BJCA001, but g.737_806delCCGCCGGAGCCTCTTGGAGCTTAGGGTCAGGGTCAGGGCCAGGGTCAGGCTCCGGCTTGGGCCCCAGCTC, [p.P246fs] | This study |
| V45 | BJCA001 | As BJCA001, but g.373_502dupACACTCACTCCTCACGGCAACAGGCCCATGGTGTCGCCGCCGTCCTCAGGCCTGCCCAACTACGGCACGACATCTGCCTACTACCCTCTGCCCTACGTGGGTAATGGGGCTCCCTCGAGCAGTGGGCCGG, [p.G168fs] | This study |
| V46 | BJCA001 | As BJCA001, but g.687_696dupTCGCACCGCT, [p.P233fs] | This study |
| V47 | BJCA001 | As BJCA001, but g.187C>T, [p.Q63*] | This study |
| V48 | BJCA001 | As BJCA001, but g.255_256insC, [p.Y86fs] | This study |

**Notes:** dup: duplicate sequences; ins: insert mutations; del: deletion mutations; “*”: nonsense mutations; fs: frameshift mutations.

[1] Wang, X., Bing, J., Zheng, Q., Zhang, F., Liu, J., Yue, H., Tao, L., Du, H., Wang, Y., Wang, H., and Huang, G. (2018). The first isolate of *Candida auris* in China: clinical and biological aspects. Emerg. Microbes Infec. *7*, 1-9. 10.1038/s41426-018-0095-0.
