# Supplementary material for: A single gene mutation underpins metabolic adaptation and acquisition of filamentous competence in the emerging fungal pathogen Candida auris
Source: PLoS Pathog. 2024 Jul 8;20(7):e1012362. doi: 10.1371/journal.ppat.1012362 (PMC11257696; doi:10.1371/journal.ppat.1012362)
Supplement: S2 Table — (DOCX) [file ppat.1012362.s008.docx]

**S2 Table. Primers used in this study**

| Primer number | Description | Sequence (5’ to 3’) | Purpose |
| --- | --- | --- | --- |
| LT1519 | *C. auris ARG4* 5'flank top with *Apa*I | TTATATGGGCCCAGTCTTTCCGTGCTGAAAAG | For *C.auris ARG4* KO |
| LT1520 | *C. auris ARG4* 5'flank bottom with *Xho*I | AATCATCTCGAGAGTTGGGTATTGGTATCTATCG |  |
| LT1521 | *C. auris ARG4* 3'flank top with *Sac*II | TAATATCCGCGGTTAGAGGTTGAGGAATGATG |  |
| LT1522 | *C.auris ARG4* 3'flank bottom with *Sac*I | ATATACGAGCTCAACTTTTTGTTGTGCTGCTC |  |
| LT1529 | *C.auris ARG4* ORF left | TAGCTACTGACATGAGACTTCAC | *C. auris ARG4* deletion confirmation |
| LT1530 | *C.auris ARG4* ORF right | AAGAGCTCAAGAGAATCAGG |  |
| LT1533 | *C.auris GFC1* 5'flank top with *Apa*I | TTATATGGGCCCTTGATACTGCGAGAAACACTAG | For *C.auris GFC1* KO |
| LT1534 | *C.auris GFC1* 5'flank bottom with *Sal*I | AATCATGTCGACTCCAGAGGGTTTATAAGGTTG |  |
| LT1535 | *C.auris GFC1* 3'flank top with *Sac*II | TAATATCCGCGGTCAACAGCTTGATCGAAAAG |  |
| LT1536 | *C.auris GFC1* 3'flank bottom with *Sac*I | ATATACGAGCTCACATGAGCGTAGACATATACG |  |
| LT1537 | *C.auris GFC1* ORF left | ATGCTCTCGAAAACTACGTC | *C. auris GFC1* deletion confirmation |
| LT1538 | *C.auris GFC1* ORF right | TCAACACCTGATTGAACGAC |  |
| LT1708 | *C.auris ARG4* cassette 5'F with *Xba*I | ATATACTCTAGAAGTCTTTCCGTGCTGAAAAG | Complemented *C. auris GFC1* gene |
| LT1709 | *C.auris ARG4* cassette 3'R with *Not*I | ATATACGCGGCCGCACAATGGATATAGGTGAGTGG |  |
| LT1737 | *C.auris GFC1* COMP 5' forward *Spe*I | ATATACACTAGTAATTTACTCGAGACCCTACC |  |
| LT1738 | *C.auris GFC1* COMP 5' reverse *EcoR*I | ATATACGAATTCAGAGAAGACAAACTCGTATCAC |  |
| LT1739 | *C.auris GFC1* COMP 3' forward *Xba*I | ATATACTCTAGACAGGTTGTACGATGCAACTG |  |
| LT1740 | *C.auris GFC1* COMP 3' reverse *Spe*I | ATATACACTAGTTGGTTCCATTTCAGTGTAAG |  |
| LT2364 | *C.auris MCU1* 5'flank top with *Kpn*I | TTATATGGTACCATTTCATCATCGGAAAGGAC | For *C.auris MCU1* KO |
| LT2365 | *C.auris MCU1* 5'flank bottom with *Sal*I *(Xho*I*)* | AATCATGTCGACAGCTTTAGGTGGAATCCAAC |  |
| LT2366 | *C.auris MCU1* 3'flank top with *Not*I | TAATATGCGGCCGCAGTAAATGGTGGTGGTTTTG |  |
| LT2367 | *C.auris MCU1* 3'flank bottom with *Sac*II | ATATACCCGCGGATAGCAAGCTATCAACAGATG |  |
| LT2336 | *C.auris MCU*1 ORF left | AGCTCCGTATAATCCTTATG | *C. auris MCU1* deletion confirmation |
| LT2337 | *C.auris MCU1* ORF right | AGCTCCGTATAATCCTTATG |  |
| DYC0173 | *C.auris ARG4* cassette 5' | AGTCTTTCCGTGCTGAAAAG | *ARG4* cassette for Fusion PCR |
| DYC0174 | *C.auris ARG4* cassette 3' | ACAATGGATATAGGTGAGTGG |  |
| DYC0179 | *C.auris UME6* 5'flank top | CGTCTACCCGCATAAACA | For *C.auris UME6* KO (Fusion PCR) |
| DYC0180 | *C.auris UME6* 5'flank bottom with *ARG4* cassette | CTTTTCAGCACGGAAAGACTCAGGACTTGCAAGTGACCTT |  |
| DYC0181 | *C.auris UME6* 3'flank top with *ARG4* cassette | CCACTCACCTATATCCATTGTCAGAGGCCACTGTTGAGG |  |
| DYC0182 | *C.auris UME6* 3'flank bottom | GAAGACCTTCTGCAGATGC |  |
| DYC0183 | *C.auris UME6* ORF left | CACTCTCTCACCACCATCC | *C. auris UME6* deletion confirmation |
| DYC0184 | C.auris *UME6* ORF right | ATCTCGAAGTGCCTGAAGC |  |
| DYC0186 | *C.auris HGC1* 5'flank top | TGTTGAGAGATAGGACTTAAGC | For *C.auris HGC1* KO (Fusion PCR) |
| DYC0187 | *C.auris HGC1* 5'flank bottom with *ARG4* cassette | CTTTTCAGCACGGAAAGACTAGGTAGAAAAACAAACACACC |  |
| DYC0188 | *C.auris HGC1* 3'flank top with *ARG4* cassette | CCACTCACCTATATCCATTGTAATCAGCATAGAGGACGACA |  |
| DYC0189 | *C.auris HGC1* 3'flank bottom | TCTGACTGAGCTCAAACTACC |  |
| DYC0190 | *C.auris HGC1* ORF left | ACAACCAACCATACATCAATG | *C. auris HGC1* deletion confirmation |
| DYC0191 | *C.auris HGC1* ORF right | TTCCTTATGTCGTCCTGTCT |  |
| LT3280 | *C.auris* *UME6* RT 5' F | TGTATGAGCAATCACCAGAG | q-RT-PCR |
| LT3281 | *C.auris* *UME6* RT 3' R | ATGAAGCTATGGTCAAGAGG |  |
| LT3282 | *C.auris* *HGC1* RT 5' F | AGACATTGCTACCTTGAAAG |  |
| LT3283 | *C.auris* *HGC1* RT 3' R | TTGTTTAGCGTAAGGAAAGC |  |
| LT1729 | *C.auris* *ACT1* RT 5' F | TTCGAAGACGTTTCAAGTTG |  |
| LT1730 | *C.auris* *ACT1* RT 3' R | AATCTCGATCACAAGGTAGC |  |
