# Supplementary material for: A single gene mutation underpins metabolic adaptation and acquisition of filamentous competence in the emerging fungal pathogen Candida auris
Source: PLoS Pathog. 2024 Jul 8;20(7):e1012362. doi: 10.1371/journal.ppat.1012362 (PMC11257696; doi:10.1371/journal.ppat.1012362)
Supplement: S1 Dataset — (PDF) [file ppat.1012362.s009.pdf]

Protein expression profiles of the *gfc1*Δ mutant grown on YPG medium

Accession	YPG_WT_1	YPG_WT_2	YPG_WT_3	YPG_*gfc1*Δ_1	YPG_*gfc1*Δ_2	YPG_*gfc1*Δ_3	YPG_*gfc1*Δ_vs_YPG_WT_p.val	YPG_*gfc1*Δ_vs_YPG_WT_p.adj	YPG_*gfc1*Δ_vs_YPG_WT_ratio	Protein_IDs	Gene_names	Description
PIS49566.1	22.99027448	29.88866759	24.02277217	30.02672254	30.23428056	30.17495978	0.012276673	0.15	4.51	PIS49566.1	FAD2	Delta-12 fatty acid desaturase, involved in production of linoleic acid, which is a major component of membranes
PIS51826.1	19.91082776	18.02930017	21.59920346	23.70286168	23.99959796	24.67275545	0.018714267	0.218	4.28	PIS51826.1	orf19.6012	Ortholog(s) have phosphatidylinositol-4,5-bisphosphate binding, phosphatidylserine binding activity and role in endocytosis, site selection
PIS58704.1	19.91082776	18.73104872	23.77962527	23.91850373	23.82355558	24.0546854	0.005652949	0.0637	3.13	PIS58704.1	HAP42	Predicted transcription factor; possibly an essential gene, disruptants not obtained by UAU1 method
PIS58890.1	22.29634645	25.64362472	25.80837268	27.8405967	27.75539014	27.49447798	0.00242197	0.0209	3.11	PIS58890.1	HGT18	Putative glucose transporter of the major facilitator superfamily; the C. albicans glucose transporter family comprises 20 members; 12 probable membrane-spanning segments; expressed in rich medium with 2% glucose
PIS51658.1	25.14976708	27.79827094	23.98611555	29.51235229	28.33804341	28.28054373	0.002018062	0.0166	3.07	PIS51658.1	orf19.2985	Protein of unknown function; Hap43-repressed gene
PIS52299.1	21.66963692	24.10090989	22.7455831	25.77926012	24.97859857	25.93849693	0.00126711	0.00774	2.73	PIS52299.1	orf19.7091	Protein of unknown function; induced by nitric oxide; Spider biofilm repressed
PIS58854.1	20.96154074	22.93497236	21.24748166	25.41953486	24.15578956	23.68255169	0.000238361	0.00024	2.7	PIS58854.1	orf19.5040	Ortholog(s) have phospholipid binding, single-stranded DNA binding, structural constituent of nuclear pore activity and role in mRNA export from nucleus in response to heat stress, nuclear pore organization
PIS58788.1	22.96473929	18.97021369	22.56641384	24.62122935	24.44612536	23.40274669	0.225468527	0.837	2.66	PIS58788.1	orf19.2325	Ortholog(s) have RNA polymerase III general transcription initiation factor activity and RNA polymerase III type 1 promoter sequence-specific DNA binding, more

PIS55823.1	25.47988347	23.17199712	24.97391917	25.42886185	28.1668694	27.91233718	0.131162152	0.731	2.63	PIS55823.1	orf19.1210	Ortholog(s) have L-arginine transmembrane transporter activity, L-aspartate transmembrane transporter activity and L-glutamate transmembrane transporter activity, more
PIS58836.1	23.82657225	25.03494572	24.23420765	26.94439731	26.6790777	27.10507904	0.093484059	0.641	2.54	PIS58836.1	HSL1	Probable protein kinase involved in determination of morphology during the cell cycle of both yeast-form and hyphal cells via regulation of Swe1p and Cdc28p; required for full virulence and kidney colonization in mouse systemic infection
PIS54646.1	24.05042906	24.54435276	21.78824348	24.9413923	27.12639701	25.94652095	0.008502489	0.105	2.54	PIS54646.1	orf19.201	Protein of unknown function; unmerged from orf19.202 in a revision of Assembly 21
PIS48277.1	25.52115692	28.50390351	24.54771257	28.72172439	28.58236851	28.83628027	0.040491735	0.381	2.52	PIS48277.1	FGR22	Putative phosphatidylinositol-specific phospholipase C (PI-PLC); predicted type 2 membrane protein; no S. cerevisiae ortholog; role in, and regulated by, filamentation, Hap43p; almost identical to orf19.5797
PIS54506.1	27.38183116	24.66901483	24.79559045	28.20779198	28.24822434	27.94123582	0.008435189	0.104	2.52	PIS54506.1	GAP4	High-affinity S-adenosylmethionine permease; required for SAM-induced morphogenesis; hyphal induced; regulated by Hap43, Gcn2 and Gcn4; colony morphology-related gene regulation by Ssnp
PIS49771.1	24.85707171	25.28244687	25.31782991	27.43052441	27.40490452	27.94043914	1.02E-05	8.40E-09	2.44	PIS49771.1	ARG3	Putative ornithine carbamoyltransferase; Gcn4-regulated; Hap43-induced; repressed in alkalinizing medium; rat catheter and Spider biofilm induced
PIS58122.1	25.90941699	26.92629902	25.52599869	26.94720421	29.3198631	29.31117899	0.021815	0.249	2.41	PIS58122.1	BPH1	Ortholog of S. cerevisiae Bph1; a putative ortholog of human Chediak-Higashi syndrome protein and murine beige gene implicated in disease syndromes involving defective lysosomal trafficking; mutant is viable
PIS56612.1	25.45323672	23.53586671	26.96501793	27.9044565	27.59378534	27.58559789	0.005405061	0.0603	2.38	PIS56612.1	SWR1	Component of the SWR1 complex, which has a role in exchanging histone variant H2AZ for histone H2A in chromatin; required for proper nucleosome positioning on WOR1 promoter
PIS51722.1	19.11132379	22.77395602	22.97770679	23.90946942	24.00723966	23.79824262	0.015971106	0.189	2.28	PIS51722.1	orf19.7224	Ortholog(s) have ubiquitin protein ligase activity and role in histone catabolic process
PIS51584.1	24.47567927	23.88987587	23.28661639	25.45215439	26.7332924	26.22758102	0.000132616	4.77E-05	2.25	PIS51584.1	orf19.5209	Ortholog(s) have role in cytoplasm to vacuole transport by the Cvt pathway, intra-Golgi vesicle-mediated transport and Golgi transport complex localization

PIS58278.1	17.14554342	23.62648657	24.43947271	23.78284095	23.65899336	24.52027432	0.129246552	0.727	2.25	PIS58278.1	RLM1	Transcription factor required for wild-type resistance to cell wall perturbation caused by caspofungin treatment; regulates caspofungin-induced transcription of SKO1
PIS49669.1	21.87709539	21.7709625	22.21443277	23.94560694	23.89124197	24.60048075	7.29E-05	1.15E-05	2.19	PIS49669.1	orf19.6667	Predicted histone deacetylase activity; Spider biofilm induced
PIS51798.1	26.11325916	25.98501478	25.22993722	27.75320828	27.982154	28.10487077	0.012231762	0.149	2.17	PIS51798.1	ATC1	Cell wall acid trehalase; catalyzes hydrolysis of the disaccharide trehalose; similar to S. cerevisiae vacuolar acid trehalase (Ath1p); Hap43p-repressed gene
PIS51387.1	25.7975176	25.56777025	26.01053442	28.45368897	27.59190096	27.77675919	0.000943271	0.00436	2.15	PIS51387.1	MRV2	Protein of unknown function; repressed by fluphenazine treatment or in an azole-resistant strain that overexpresses CDR1 and CDR2; Spider biofilm induced
PIS58368.1	28.69078854	29.07639061	29.08532131	31.32756031	31.125191	30.84794294	0.005557678	0.0624	2.15	PIS58368.1	orf19.804.1	Ortholog of S. cerevisiae : MIN8, C. dubliniensis CD36 : Cd36_02990, C. parapsilosis CDC317 : CPAR2_211020, C. auris B8441 : B9J08_000865 and Candida tenuis NRRL Y-1498 : CANTEDRAFT_116148
PIS56674.1	22.89802331	24.9948842	22.9254615	26.20313512	25.28164563	25.75450642	0.003749219	0.0361	2.14	PIS56674.1	NAG4	Putative transporter; fungal-specific; similar to Nag3p and to S. cerevisiae Ypr156Cp and Ygr138Cp; required for wild-type mouse virulence and wild-type cycloheximide resistance; gene cluster encodes enzymes of GlcNAc
PIS56699.1	26.90884821	28.82382001	26.50092816	29.3965247	29.55061883	29.72113845	0.049422104	0.44	2.14	PIS56699.1	orf19.1139	Protein of unknown funtion; S. cerevisiae ortholog Svl3 plays a role in endocytosis and is localized to the bud neck; Spider biofilm induced
PIS48502.1	26.48904043	23.56312622	25.3175277	26.74272226	27.6565681	27.3432042	0.011756458	0.143	2.12	PIS48502.1	VID27	Protein similar to S. cerevisiae Vid27p; transposon mutation affects filamentous growth; mutation confers hypersensitivity to toxic ergosterol analog; fungal-specific (no human or murine homolog)
PIS52269.1	26.0565394	28.18548446	26.02624753	28.25981136	29.23375631	29.05559927	0.066336928	0.535	2.09	PIS52269.1	orf19.4059	Ortholog(s) have role in arginine transmembrane transport, lysine transport, regulation of intracellular pH and cell division site, cell tip, cytoplasm, fungal-type vacuole, fungal-type vacuole membrane localization
PIS55445.1	25.55873021	26.40736763	26.9406147	28.33918279	28.36253511	28.48301984	0.083010428	0.606	2.09	PIS55445.1	orf19.4240	Ortholog(s) have role in GPI anchor biosynthetic process, intracellular manganese ion homeostasis and endoplasmic reticulum, fungal-type vacuole membrane localization

PIS51497.1	23.69816562	24.45898916	23.89792101	25.53629382	26.25816877	26.46390579	0.000331044	0.000504	2.07	PIS51497.1	PDR17	Fungal-specific protein (no human or murine homolog); role in sensitivity to fluconazole, specifically
PIS58430.1	24.11107835	23.85507107	23.86656304	26.31254636	25.82844281	25.75195218	8.91E-05	1.52E-05	2.02	PIS58430.1	AQY1	Aquaporin water channel; osmotic shock resistance, WT freeze tolerance; virulent in mice; flucytosine repressed; flow model/RPMI/Spider/rat catheter biofilm induced; required for RPMI biofilm formation; Bcr1-induced in a/a RPMI biofilms
PIS55670.1	22.42962543	24.66677556	23.98341911	25.49126071	25.48423079	26.1539767	0.012055659	0.147	2.02	PIS55670.1	orf19.1573	Ortholog(s) have fungal-type vacuole membrane, vacuole-mitochondrion membrane contact site localization
PIS52202.1	26.83019951	26.31470712	28.3843756	29.24604792	29.03950831	29.31570007	0.001805612	0.0142	2.02	PIS52202.1	orf19.6934	Ortholog(s) have role in protein insertion into mitochondrial inner membrane from matrix and mitochondrial inner membrane localization
PIS58171.1	24.91524614	26.95330392	24.87515152	27.45950854	27.46433909	27.4670176	0.04028251	0.38	1.88	PIS58171.1	SFL1	Transcription factor involved in negative regulation of morphogenesis, flocculation and virulence; induced in core caspofungin response; Spider biofilm induced
PIS49661.1	23.96330988	21.08754312	24.36986492	24.6954271	25.38142537	24.96680221	0.040438519	0.381	1.87	PIS49661.1	orf19.529	Ortholog of C. dubliniensis CD36 : Cd36_29750, C. parapsilosis CDC317 : CPAR2_205260, C. auris B8441 : B9J08_005543 and Candida tenuis NRRL Y-1498 : CANTEDRAFT_91782
PIS48659.1	26.51893512	24.26546737	24.16391244	27.15457193	26.87633884	26.45093582	0.020737226	0.239	1.84	PIS48659.1	CAN2	Basic amino acid permease; arginine metabolism; regulated by Nrg1/Tup1; caspofungin, flucytosine induced; colony morphology-related regulation by Ssn6; Hap43-repressed; rat catheter and Spider biofilm induced; promoter bound by Efg1
PIS52465.1	25.11241514	23.9359619	26.25201123	27.2131295	26.83557791	26.74714931	0.0355078	0.351	1.83	PIS52465.1	IPT1	Inositol phosphoryl transferase; catalyzes the synthesis of the most abundant sphingolipid, mannose-(inositol-P)2-ceramide, M(IP)2C, from MIPC; required for wild-type membrane localization of Cdr1; Spider biofilm induced
PIS56753.1	23.49933006	23.58598205	25.11603382	26.55203085	25.96167667	25.13913715	0.048473961	0.435	1.82	PIS56753.1	OPT6	Putative oligopeptide transporter; fungal-specific (no human or murine homolog); expression of OPT6, OPT7, or OPT8 does not suppress defect of mutant lacking Opt1p, Opt2p, and Opt3p; alleles are nonidentical
PIS51661.1	23.98334276	22.50134401	24.20810312	24.92623126	25.33715555	25.89199156	0.025778066	0.281	1.82	PIS51661.1	orf19.2982	Putative autophagy-related gene; upregulated during biofilm production

PIS58634.1	24.13180279	23.6794391	23.14730665	26.43624794	25.01223227	24.91845373	0.002378778	0.0204	1.8	PIS58634.1	AVT1	Putative vacuolar transporter; promoter bound by a1p and alpha2p by ChIP-chip analysis
PIS56701.1	21.45344516	25.86102334	24.74259953	25.60313352	25.84654822	25.98631228	0.134660829	0.737	1.79	PIS56701.1	AVT4	Putative vacuolar transporter of large neutral amino acids; induced by alpha pheromone in SpiderM medium
PIS51654.1	22.82629379	22.8111681	24.48622691	23.37362307	25.82677842	26.29829861	0.278500202	0.867	1.79	PIS51654.1	XOG1	Exo-1,3-beta-glucanase; 5 glycosyl hydrolase family member; affects sensitivity to chitin and glucan synthesis inhibitors; not required for yeast-to-hypha transition or for virulence in mice; Hap43-induced; Spider biofilm induced
PIS54865.1	24.49895187	22.16025432	24.88158389	24.73435111	27.36281582	24.67534685	0.470845383	0.92	1.74	PIS54865.1	orf19.3310	Protein of unknown function; Hap43-repressed; rat catheter and Spider biofilm induced
PIS58443.1	23.57538954	22.92280064	24.43545343	24.86926679	25.73399429	25.53279041	0.05377852	0.465	1.73	PIS58443.1	orf19.3673	Ortholog(s) have guanyl-nucleotide exchange factor activity and role in chromosome organization, endoplasmic reticulum to Golgi vesicle-mediated transport
PIS48694.1	24.9982627	24.19045643	25.82672744	27.50810044	26.41393508	26.26948105	0.027031881	0.29	1.73	PIS48694.1	VPS15	Protein involved in retrograde endosome-to-Golgi protein transport; required for normal virulence
PIS51060.1	24.02434767	22.71075981	24.50175957	24.51719535	25.02465422	26.85118307	0.025630058	0.28	1.72	PIS51060.1	orf19.1617	Protein similar to S. cerevisiae Ydr282cp; transposon mutation affects filamentous growth; Hap43p-repressed gene
PIS55686.1	25.4066839	23.34233049	27.17797761	28.39126206	26.74883821	25.94994275	0.236378631	0.844	1.72	PIS55686.1	orf19.2733	Putative subunit of phosphatidylinositol 3-kinase complexes I and II; transcription is activated in the presence of elevated CO2
PIS58305.1	27.82340602	28.77182938	27.36618125	29.2465852	30.02764538	29.85339865	0.044029418	0.406	1.72	PIS58305.1	orf19.3869	Protein of unknown function; regulated by Tsa1, Tsa1B in minimal media at 37 degrees C; shows colony morphology-related gene regulation by Ssn6; Spider biofilm induced
PIS58976.1	25.74734125	25.05682643	26.27743676	26.23000225	26.42752305	29.58224319	0.047466454	0.428	1.72	PIS58976.1	orf19.933	Predicted ubiquitin conjugating enzyme involved in DNA damage response; forms a complex with Mms2p

PIS55835.1	20.906784	24.32558785	24.6430636	25.22776384	24.67540986	25.0939515	0.196845343	0.814	1.71	PIS55835.1	orf19.3161	Ortholog(s) have ATPase activator activity, RNA binding activity
PIS49670.1	24.7209129	20.9431756	23.70022629	24.20736331	24.94463092	25.27181409	0.142485504	0.75	1.69	PIS49670.1	PTH1	Putative gluconate transport protein; antigenic during human oral infection; possibly an essential gene, disruptants not obtained by UAU1 method
PIS49797.1	27.37809524	27.69636449	27.3943611	29.26193421	28.89349785	29.34575572	0.001856673	0.0148	1.68	PIS49797.1	AGE2	Ortholog(s) have GTPase activator activity and role in endoplasmic reticulum to Golgi vesicle-mediated transport, intra-Golgi vesicle-mediated transport
PIS52425.1	28.33190777	29.32291648	27.6439569	29.49804159	30.25010535	30.54949811	0.04468859	0.411	1.67	PIS52425.1	orf19.4888	Has domain(s) with predicted RNA binding, nucleic acid binding activity
PIS48646.1	24.97997389	25.29401853	24.99469645	26.88947371	26.82003646	26.50939222	0.003271678	0.0306	1.65	PIS48646.1	CDC27	Putative ubiquitin-protein ligase; periodic mRNA expression, peak at cell-cycle S/G2 phase
PIS51473.1	26.03790388	25.52707621	27.0316753	27.63207866	27.96193806	27.96414975	0.006759585	0.0804	1.65	PIS51473.1	orf19.7235	Putative protein of unknown function; mutation confers hypersensitivity to amphotericin B
PIS58779.1	24.81522792	25.7163318	24.35721892	26.9708598	26.76749875	26.05575453	0.013178666	0.16	1.64	PIS58779.1	DCC1	Protein with a predicted role in sister chromatid cohesion and telomere length maintenance; cell-cycle regulated periodic mRNA expression
PIS58643.1	28.32385562	29.45076397	27.49770163	29.46604225	30.29888846	30.36782523	0.042125305	0.393	1.62	PIS58643.1	orf19.5984	Putative protein of unknown function; homozygous transposon insertion causes decreased colony wrinkling under filamentous growth-inducing conditions, but does not block true hyphal formation in liquid media
PIS51589.1	24.59405405	28.11317748	29.02226859	28.67970777	28.69634463	29.17202016	0.354905856	0.894	1.61	PIS51589.1	COX8	Putative cytochrome c oxidase; flucytosine induced; caspofungin repressed
PIS54500.1	25.73180061	24.83018464	25.10263108	26.91640646	27.0492902	26.47389629	0.015702854	0.187	1.59	PIS54500.1	orf19.1308	Predicted membrane transporter, member of the drug:proton antiporter (14 spanner) (DHA2) family, major facilitator superfamily (MFS)

PIS51594.1	23.72968892	24.00861422	21.32894083	24.35518123	24.52412822	24.96211566	0.331582848	0.887	1.59	PIS51594.1	orf19.194	Ortholog of C. dubliniensis CD36 : Cd36_19300, C. parapsilosis CDC317 : CPAR2_209720, C. auris B8441 : B9J08_003188 and Candida tenuis NRRL Y-1498 : CANTEDRAFT_114035
PIS58292.1	26.01772976	27.7429431	26.21329807	28.12177655	28.28304552	28.31412277	0.049518993	0.441	1.58	PIS58292.1	EST1	Telomerase subunit; allosteric activator of catalytic activity, but not required for catalytic activity; has TPR domain
PIS48387.1	27.38651952	27.86128689	27.76060314	28.97513485	29.48304135	29.26714124	0.029998866	0.312	1.57	PIS48387.1	CHS2	Chitin synthase; nonessential; required for wild-type chitin deposition in hyphae; transcript regulated during dimorphic transition; Chs1 and Chs2, but not Chs3, are inhibited by the protoberberine HWY-289; flow model biofilm repressed
PIS55484.1	25.21944829	27.78799854	25.75395537	27.47609517	28.02014305	27.98965377	0.090152104	0.631	1.57	PIS55484.1	PET100	Chaperone that facilitates the assembly of cytochrome c oxidase; plasma membrane protein
PIS48379.1	21.43065104	24.57034437	23.77214242	25.15054687	24.79257204	24.5259453	0.051713184	0.454	1.57	PIS48379.1	RRP8	Ribosomal protein; Hap43-induced; F-12/CO2 early biofilm and rat catheter biofilm induced
PIS54585.1	27.73150646	24.3782335	28.02335481	28.40148499	28.34405796	28.06578497	0.142945362	0.751	1.56	PIS54585.1	AYR1	Putative oxidoreductase; transcriptionally induced by interaction with macrophage; rat catheter biofilm repressed
PIS58174.1	24.11597051	24.72301779	25.42048106	26.84843869	26.18985359	25.9101808	0.010155693	0.125	1.56	PIS58174.1	orf19.3848	Ortholog of C. dubliniensis CD36 : Cd36_31420, C. parapsilosis CDC317 : CPAR2_205100, C. auris B8441 : B9J08_000664, Debaryomyces hansenii CBS767 : DEHA2G10648g and Pichia stipitis Pignal : PICST_32997
PIS49484.1	24.50464827	24.16405606	25.63358567	26.56487179	27.03846944	25.32217174	0.076895626	0.582	1.54	PIS49484.1	CBP1	Corticosteroid binding protein; transcription induced at late log-phase or upon adherence to polystyrene; not induced by corticosterone; contains a possible NAD/FAD binding region; regulated by Nrg1, Tup1; Spider biofilm induced
PIS58575.1	24.11883149	26.63336967	24.8301451	26.42928761	26.20361845	27.58246545	0.179533117	0.798	1.54	PIS58575.1	orf19.6855	Ortholog of C. dubliniensis CD36 : Cd36_04370, C. parapsilosis CDC317 : CPAR2_105410, C. auris B8441 : B9J08_000021 and Candida tenuis NRRL Y-1498 : CANTEDRAFT_114891
PIS49824.1	25.73441174	23.26324903	24.09934713	25.99060487	26.6543833	25.06473966	0.049831846	0.443	1.54	PIS49824.1	SGD1	Predicted small ribosomal subunit biogenesis protein; repressed in core stress response; transcript increases in populations of cells exposed to fluconazole over multiple generations; Spider biofilm induced

PIS58026.1	29.50852811	29.40496104	30.08051929	31.14814826	31.20943916	31.22569278	8.62E-05	1.46E-05	1.53	PIS58026.1	orf19.5777	Protein of unknown function; F-12/CO2 early biofilm induced
PIS51458.1	24.4380775	26.24283214	24.48815083	27.26504186	26.63785471	25.82416604	0.030538472	0.316	1.52	PIS51458.1	orf19.4529	Ortholog of Srp21, signal recognition particle subunit, functions in protein targeting to the endoplasmic reticulum membrane; predicted adhesin-like protein; mutants are viable
PIS56712.1	25.60919132	25.39838007	24.63468253	26.42884769	26.67931508	27.08073502	0.031033979	0.32	1.52	PIS56712.1	orf19.7673	Ortholog(s) have mRNA binding activity, role in mRNA splicing, via spliceosome and U1 snRNP, U2-type prespliceosome, U4/U6 x U5 tri-snRNP complex, U5 snRNP, commitment complex, cytosol, nucleus localization
PIS55756.1	28.47765773	27.49490824	27.57427127	29.12136766	29.42388485	29.48845795	0.009873687	0.121	1.5	PIS55756.1	FAT1	Predicted enzyme of sphingolipid biosynthesis; upregulated in biofilm
PIS51017.1	24.59940665	24.28463006	24.12561689	25.38494165	25.45478237	26.62694685	0.003245291	0.0304	1.49	PIS51017.1	AFP99	Agmatinase, involved in metabolism of agmatine; downregulated upon adherence to polystyrene; regulated by Gcn2p and Gcn4p
PIS48276.1	25.18938949	26.32946929	24.2049993	25.95460821	27.06965488	27.17817828	0.02166882	0.247	1.49	PIS48276.1	SHE9	Protein with similarity to S. cerevisiae She9p, which inhibits growth when overproduced; gene has possible growth-regulation element; fungal-specific (no human or murine homolog)
PIS52438.1	23.33614408	22.28481882	26.18484855	25.82057638	25.25523491	25.16897812	0.067805067	0.542	1.48	PIS52438.1	orf19.5276	Putative nuclear pore-associated protein; Hap43p-induced gene; induced upon low-level peroxide stress; possibly an essential gene, disruptants not obtained by UAU1 method
PIS48535.1	23.96630475	27.80069156	24.1080869	27.2766502	26.36537768	26.64412928	0.117435154	0.704	1.47	PIS48535.1	orf19.4373	Protein similar to S. cerevisiae Fmn1p, which is riboflavin kinase; predicted Kex2p substrate; Hap43p-repressed gene
PIS51890.1	21.13277631	21.36378332	23.57101509	23.79857243	23.44504202	23.21402807	0.180543276	0.799	1.46	PIS51890.1	orf19.2401	Ortholog(s) have protein-phosphatidylethanolamide deconjugating activity
PIS58020.1	27.47955672	27.14706304	26.6281668	29.10882662	28.42419751	28.10688547	0.015212261	0.182	1.46	PIS58020.1	VMA8	Putative vacuolar H+-ATPase subunit; regulated by Nrg1 and Mig1; transcript increases in populations of cells exposed to fluconazole over multiple generations; rat catheter and Spider biofilm repressed

PIS58627.1	24.07591552	21.69279424	24.3167685	24.89625655	24.84221446	24.68279242	0.091547002	0.635	1.45	PIS58627.1	orf19.21	Ortholog(s) have role in ethanol metabolic process and mitochondrial inner membrane localization
PIS54981.1	26.9519013	27.06178894	28.28757122	29.14523947	28.73478287	28.77717376	0.007279687	0.088	1.45	PIS54981.1	PUT1	Putative proline oxidase; regulated by Rim101p and Put3p; null mutant cannot grow on proline as a nitrogen source and shows reduced filamentation and reduced invasive growth both in vitro and in vivo; Spider biofilm induced
PIS56910.1	19.2405685	24.21492759	22.82173972	23.44273582	23.58821876	23.55192176	0.344322742	0.891	1.44	PIS56910.1	orf19.3202	Ortholog of C. dubliniensis CD36 : Cd36_51590, C. parapsilosis CDC317 : CPAR2_303610, C. auris B8441 : B9J08_001456 and Candida tenuis NRRL Y-1498 : CANTEDRAFT_135127
PIS48187.1	26.11271356	26.8988924	25.44016591	26.95680152	27.84467359	27.97351227	0.056029974	0.479	1.44	PIS48187.1	orf19.6506	Ortholog(s) have role in negative regulation of antisense RNA transcription, positive regulation of transcription by RNA polymerase II, regulation of DNA-templated DNA replication initiation, transcription elongation by RNA polymerase II
PIS51835.1	23.33203109	23.7404079	24.74796648	25.89170304	25.3628497	24.88997225	0.058673789	0.494	1.44	PIS51835.1	orf19.6581	Ortholog(s) have palmitoyltransferase activity, role in protein palmitoylation and endoplasmic reticulum localization
PIS58769.1	27.67558421	27.87070211	27.43929495	28.79717622	29.22154132	29.21276653	0.032981747	0.334	1.42	PIS58769.1	PEL1	Predicted enzyme of mitochondrial phospholipid biosynthesis; rat catheter and flow model biofilm induced
PIS58951.1	26.88739082	27.54410648	26.60247379	28.10190483	28.44595069	28.72744069	0.06478991	0.527	1.41	PIS58951.1	OXR1	Ortholog(s) have role in vacuolar acidification and fungal-type vacuole membrane, mitochondrion localization
PIS52073.1	29.89478468	30.56179977	29.73242664	31.39229076	31.37113295	31.61892371	0.042374449	0.395	1.4	PIS52073.1	GPD2	Surface protein similar to glycerol 3-P dehydrogenase; binds host Factor H, FHL-1, plasminogen; regulated by Ssn6, Nrg1, Efg1; induced by cell wall regeneration, macrophage/pseudohyphal growth, core stress response; Spider biofilm induced
PIS51467.1	23.80747727	24.95442901	24.47998182	26.21981525	25.84252048	25.34848315	0.091734911	0.636	1.39	PIS51467.1	HNM1	Putative choline/ethanolamine transporter; mutation confers hypersensitivity to toxic ergosterol analog; colony morphology-related gene regulation by Ssn6; clade-associated gene expression
PIS56659.1	22.74474544	23.31645439	23.05666805	24.51950535	24.68073967	24.08528446	0.300438744	0.876	1.39	PIS56659.1	orf19.1519	Protein of unknown function; Hap43-repressed gene

PIS58497.1	26.17953195	26.35375927	26.0975133	27.81571638	27.46116763	27.51267957	0.018587013	0.217	1.39	PIS58497.1	orf19.3325	Putative glycogen synthesis initiator; regulated by Efg1 and Efh1; Hog1-repressed; colony morphology-related gene regulation by Ssn6; induced by prostaglandins; flow model biofilm induced
PIS56927.1	24.79054605	24.14534755	23.85500739	26.05280633	25.27153714	25.63848526	0.116181309	0.701	1.39	PIS56927.1	orf19.4029	Ortholog(s) have unfolded protein binding activity, role in ribosomal large subunit assembly, ribosomal large subunit biogenesis and cytosol localization
PIS58654.1	24.57524044	24.22740809	25.77120054	27.47441575	25.44918312	25.83297055	0.060618293	0.505	1.39	PIS58654.1	PTP1	Phosphotyrosine-specific protein phosphatase; rat catheter biofilm induced
PIS55016.1	29.25887863	31.02156715	29.38325541	31.37569648	31.24342614	31.16433884	0.009794648	0.121	1.37	PIS55016.1	orf19.1183	Ortholog of S. cerevisiae : YNL115C, C. glabrata CBS138 : CAGL0L03938g, C. dubliniensis CD36 : Cd36_60200, C. parapsilosis CDC317 : CPAR2_603320 and C. auris B8441 : B9J08_002166
PIS51150.1	27.04210983	27.26791484	27.26325531	28.54202488	28.66516729	28.47094047	0.050382947	0.446	1.37	PIS51150.1	RFC3	Putative heteropentameric replication factor C subunit; transcription is induced upon filamentous growth
PIS48287.1	25.85445573	25.01133786	26.31073185	27.51325964	26.79129862	26.95622442	0.0630813	0.518	1.36	PIS48287.1	CHS4	Activator of Chs3p chitin synthase; required for wild-type wall chitin content, but not for hyphal growth; mutant resistant to Calcofluor white; prenylation and 2 transmembrane segments predicted; functional homolog of S. cerevisiae Chs4p
PIS51170.1	26.07942451	26.21179801	26.72131266	27.58493263	27.73737737	27.7689999	0.028480482	0.3	1.36	PIS51170.1	orf19.4839	GET complex subunit; expression downregulated in an ssr1 null mutant
PIS56754.1	24.80064593	24.91024393	24.97974174	25.83346513	26.37761373	26.56635754	0.003245196	0.0304	1.36	PIS56754.1	orf19.5536	Ortholog of S. cerevisiae : AMD2, C. dubliniensis CD36 : Cd36_62950, C. parapsilosis CDC317 : CPAR2_301880, Candida tenuis NRRL Y-1498 : CANTEDRAFT_123468 and Debaryomyces hansenii CBS767 : DEHA2E23342g
PIS52237.1	25.49292267	26.45267996	24.01332933	26.6667605	26.80944788	26.55016918	0.041969944	0.392	1.36	PIS52237.1	SNX4	Putative sorting nexin; induced during the mating process
PIS58906.1	27.1506015	26.84609105	27.10045735	28.42790385	28.26272868	28.45465615	0.005753425	0.065	1.35	PIS58906.1	NCE103	Carbonic anhydrase; converts of CO2 to bicarbonate; essential for virulence in host niches with limited CO2, normal white-opaque switch; Mnl1-induced in weak acid stress; Hap43-induced gene; F-12/CO2, rat catheter, Spider biofilm induced

PIS59004.1	23.77893497	21.81408271	23.34962269	25.05503712	23.9747475	23.92377933	0.178763904	0.797	1.34	PIS59004.1	MNL1	Transcription factor; induces transcripts of stress response genes via SLE (STRE-like) elements; required for adaptation to weak acid stress; activates a subset of the genes that are repressed by Nrg1
PIS52360.1	26.66984568	25.27014484	25.23559946	27.39963161	26.54128554	27.26366367	0.054047525	0.467	1.34	PIS52360.1	orf19.4017	Ortholog of C. dubliniensis CD36 : Cd36_54840, C. parapsilosis CDC317 : CPAR2_100270, C. auris B8441 : B9J08_003974 and Candida tenuis NRRL Y-1498 : cten_CGOB_00242
PIS58660.1	25.02850897	28.29413721	25.96966259	26.93774302	28.03350385	28.3262726	0.080609553	0.597	1.34	PIS58660.1	orf19.6374	Ortholog(s) have unfolded protein binding activity, role in mitochondrial proton-transporting ATP synthase complex assembly and mitochondrial inner membrane, mitochondrial membrane localization
PIS51182.1	25.07551391	26.18699583	25.244637	26.68596902	26.71530696	27.12059549	0.023516291	0.263	1.34	PIS51182.1	orf19.7578	Ortholog(s) have oligosaccharide binding activity and role in endoplasmic reticulum unfolded protein response, retrograde protein transport, ER to cytosol, ubiquitin-dependent ERAD pathway, ubiquitin-dependent glycoprotein ERAD pathway
PIS58398.1	25.51001148	25.54137435	25.28853246	27.00549905	26.94141595	26.38692012	0.024852255	0.274	1.33	PIS58398.1	orf19.2517	Predicted membrane transporter, member of the drug:proton antiporter (12 spanner) (DHA1) family, major facilitator superfamily (MFS)
PIS51785.1	28.28669999	29.20837238	28.45606041	30.01950427	29.92425618	29.98672947	0.148065888	0.759	1.33	PIS51785.1	orf19.5201	Ortholog(s) have structural constituent of ribosome activity and mitochondrial small ribosomal subunit localization
PIS48719.1	23.63440967	23.88527126	23.61963302	25.05381323	25.18745922	24.89005957	0.010807039	0.132	1.33	PIS48719.1	SAP3	Secreted aspartyl proteinase, acts in utilization of protein as nitrogen source; assessment of virulence role complicated by URA3 effects; regulated by growth phase; produced by opaque phase cells; alpha-pheromone repressed
PIS52055.1	25.21884534	25.97341295	23.02399141	25.06388159	25.83116607	27.27775973	0.270169392	0.863	1.32	PIS52055.1	orf19.3051	Protein of unknown function; S. pombe ortholog SPAC17A2.02c plays a role in resistance to cadmium; colony morphology-related gene regulation by Ssn6; Spider biofilm repressed
PIS50492.1	27.50193421	27.7105745	29.55470578	30.19283009	29.19738329	29.31925926	0.021582256	0.247	1.31	PIS50492.1	orf19.3977	Protein with a role in translation; flow model biofilm repressed
PIS58273.1	26.41722103	26.11447394	23.75460017	27.16927176	26.31212342	26.7495064	0.173684623	0.791	1.31	PIS58273.1	orf19.4656	Ortholog of C. dubliniensis CD36 : Cd36_41300, C. parapsilosis CDC317 : CPAR2_400390, C. auris B8441 : B9J08_000767 and Candida tenuis NRRL Y-1498 : CANTEDRAFT_94704

PIS49630.1	24.68321906	24.64473253	22.88439732	26.17631594	25.00968946	24.95121074	0.09652738	0.651	1.31	PIS49630.1	orf19.7291	Ortholog(s) have tRNA (adenine(58)-N1)-methyltransferase activity, role in tRNA methylation and nucleus, tRNA (m1A) methyltransferase complex localization
PIS51197.1	24.86368583	27.62849187	26.46712169	27.16389208	27.73036237	27.9313684	0.092711722	0.639	1.29	PIS51197.1	ADE8	Putative phosphoribosylglycinamide formyl-transferase, enzyme of amino acid biosynthesis pathway; upregulated in biofilm; S. cerevisiae ortholog is Gcn4p regulated; protein enriched in stationary phase yeast-form cultures
PIS58738.1	27.45999123	27.18030924	28.07692183	29.01241807	28.90380445	28.66295974	0.09594751	0.649	1.29	PIS58738.1	ARF3	Similar to but not orthologous to S. cerevisae Arf3; transcript filament induced; Tup1 regulated; rat catheter biofilm repressed (see Locus History Note for Assembly 19 correction)
PIS51375.1	24.37603039	25.95331264	25.2403431	26.32710713	26.55986637	26.53652717	0.19558704	0.813	1.28	PIS51375.1	CNT	CNT family H(+)/nucleoside symporter; transports adenosine, uridine, inosine, guanosine, tubercidin; variant alleles for high/low-affinity isoforms; S or G at residue 328 affects specificity; Spider, flow model biofilm induced
PIS50392.1	23.62949708	24.93212729	24.73718101	25.52537142	25.88092742	25.72000105	0.026543763	0.287	1.28	PIS50392.1	OMA1	Putative metalloendopeptidase of mitochondrial inner membrane, involved in TOR-mediated signaling
PIS51522.1	24.42176874	24.35864669	24.81337672	24.86639909	26.17807988	26.39361777	0.053157235	0.462	1.28	PIS51522.1	orf19.1285	Plasma membrane-localized protein of unknown function; Hap43p-repressed gene
PIS48448.1	27.38216517	28.18969861	25.48862905	27.70649386	28.41473861	28.73727582	0.213988565	0.828	1.27	PIS48448.1	ERB1	Protein with a predicted role in ribosomal large subunit biogenesis; mutation confers hypersensitivity to 5-fluorocytosine (5-FC), 5-fluorouracil (5-FU), and tubercidin (7-deazaadenosine); hyphal, macrophage repressed
PIS49848.1	22.21798241	21.47425429	21.87752784	24.18260609	23.38483016	21.8256679	0.096928558	0.652	1.27	PIS49848.1	orf19.1259	Ortholog(s) have ubiquitin protein ligase activity, role in histone catabolic process, regulation of transcription by RNA polymerase II and Lid2 complex localization
PIS48723.1	24.04899185	23.79778533	24.04392355	26.10224883	25.00982557	24.5742421	0.16112865	0.776	1.27	PIS48723.1	orf19.6563.1	Ortholog(s) have role in mitochondrial cytochrome c oxidase assembly, negative regulation of mitochondrial translation and mitochondrial inner membrane localization
PIS58491.1	28.62120563	29.37724158	28.83463146	29.77373462	30.09321784	30.73617718	0.010811094	0.132	1.26	PIS58491.1	BIO32	Putative class III aminotransferase with a predicted role in biotin biosynthesis; Spider biofilm induced

PIS56967.1	27.0402214	27.04090305	26.68143419	28.6008705	28.23856732	27.69595881	0.090075249	0.631	1.26	PIS56967.1	MNN12	Predicted alpha-1,3-mannosyltransferase activity with a role in protein glycosylation
PIS50386.1	29.67705431	29.1002125	29.59056566	30.69934896	30.7751338	30.638439	0.02485133	0.274	1.25	PIS50386.1	JEN2	Dicarboxylic acid transporter; regulated by glucose repression; induced by Rgt1; disruptants not obtained by UAU1 method; rat catheter and Spider biofilm induced
PIS58097.1	26.64254711	25.47522062	26.54789831	26.92714782	27.94716145	27.54630418	0.036785348	0.359	1.25	PIS58097.1	orf19.6393	Putative Arf3p GTPase activating protein; Hap43p-repressed gene; possibly an essential gene, disruptants not obtained by UAU1 method
PIS58827.1	24.3934615	24.4069341	22.85738272	26.54764483	23.95003166	24.90137185	0.069687136	0.551	1.25	PIS58827.1	UME1	Ortholog of C. dubliniensis CD36 : Cd36_73630, C. parapsilosis CDC317 : CPAR2_805330, C. auris B8441 : B9J08_000281 and Candida tenuis NRRL Y-1498 : CANTEDRAFT_119330
PIS52412.1	24.31416937	21.61857846	24.67676407	24.49776978	25.03893635	24.77898394	0.22030694	0.833	1.24	PIS52412.1	CLA4	Ste20p family Ser/Thr kinase required for wild-type filamentous growth, organ colonization and virulence in mouse systemic infection; role in chlamydospore formation; functional homolog of S. cerevisiae Cla4p; mutant caspofungin sensitive
PIS56786.1	27.96004213	28.26782612	26.3684247	27.92031955	29.14279168	29.22849741	0.12991428	0.729	1.23	PIS56786.1	GIS2	Translational activator for mRNAs with internal ribosome entry sites; induced in high iron; repressed by yeast-hypha switch; null exhibits sensitivity to sorbitol, 5-fluorocytosine, and cold temperatures; Spider biofilm repressed
PIS52248.1	27.11701109	22.56002832	27.52828511	26.41923161	25.99898023	28.46761051	0.333740049	0.888	1.23	PIS52248.1	orf19.1457	Putative RNA polymerase transcription factor TFIIH core component; possibly an essential gene, disruptants not obtained by UAU1 method
PIS48417.1	28.90572524	28.57646898	29.29070199	30.31970887	30.17093703	29.98599723	0.000607471	0.00192	1.23	PIS48417.1	orf19.1840	Tricalbin-family endoplasmic reticulum-plasma membrane tethering protein; required for transport of cell wall proteins; involved in stress responses, including sensitivity to caspofungin; Spider biofilm induced
PIS58803.1	26.47468104	26.24600488	26.97599907	27.70040349	27.72170254	27.95843165	0.016939622	0.199	1.23	PIS58803.1	orf19.2328	Ortholog of C. dubliniensis CD36 : Cd36_10220, C. parapsilosis CDC317 : CPAR2_210530, C. auris B8441 : B9J08_000256 and Candida tenuis NRRL Y-1498 : CANTEDRAFT_105553
PIS50348.1	24.94917843	27.35391882	20.04761967	24.57251838	25.52216359	25.90935573	0.605049118	0.937	1.22	PIS50348.1	MAM33	Putative mitochondrial acidic matrix protein; regulated by Ssn6p; protein present in exponential and stationary growth phase yeast cultures

PIS51163.1	29.35829882	29.0612712	29.53320572	30.45387838	30.57501796	30.57529987	0.003513719	0.0331	1.22	PIS51163.1	orf19.345	Succinate semialdehyde dehydrogenase; for utilization of gamma-aminobutyrate (GABA) as a nitrogen source; part of 4-aminobutyrate and glutamate degradation pathways; rat catheter biofilm induced
PIS48754.1	27.14678485	25.18439794	27.6451593	27.57753505	28.23526858	27.8327933	0.10642474	0.678	1.22	PIS48754.1	orf19.6905	Has domain(s) with predicted LPPG:FO 2-phospho-L-lactate transferase activity
PIS58692.1	28.81614004	27.14288523	28.72583272	29.76897661	29.15880524	29.37595244	0.014628488	0.175	1.21	PIS58692.1	COX13	Cytochrome c oxidase; flucytosine induced; repressed by nitric oxide
PIS50534.1	26.28854855	25.65001608	23.68977544	26.51923114	26.40421553	26.34434574	0.224990852	0.836	1.21	PIS50534.1	orf19.3919.1	Putative adhesin-like protein
PIS58420.1	23.70564842	24.03051672	23.26414413	25.21142496	23.79588771	25.63243687	0.025373942	0.278	1.21	PIS58420.1	PHR1	Cell surface glycosidase; may act on cell-wall beta-1,3-glucan prior to beta-1,6-glucan linkage; role in systemic, not vaginal virulence (neutral, not low pH); high pH or filamentation induced; Bcr1-repressed in RPMI a/a biofilm
PIS48712.1	27.66090207	25.98728366	28.21410702	28.78874518	28.52518638	28.14005222	0.042136741	0.393	1.2	PIS48712.1	DPP3	Protein similar to S. cerevisiae pyrophosphate phosphatase Dpp1; required for farnesol biosynthesis; repressed by 17-beta-estradiol, ethynyl estradiol; Spider biofilm induced
PIS54664.1	26.16857535	26.08559094	25.8011235	27.67105042	27.01425052	26.97228067	0.221047863	0.834	1.2	PIS54664.1	orf19.7566	High-specificity proline permease; transcript upregulated in clinical strains from HIV+ patients with oral candidiasis; alkaline upregulated by Rim101; rat catheter, Spider and flow model biofilm induced
PIS54916.1	23.92416401	25.29816114	25.37851807	24.82139005	26.76556573	26.62032765	0.150304923	0.762	1.2	PIS54916.1	SGE1	Putative multidrug resistance factor; induced in low iron; regulated by Sef1, Sfu1, and Hap43
PIS54764.1	22.68311449	24.74279903	25.45634059	25.7868606	24.60645516	26.07111111	0.078165762	0.587	1.19	PIS54764.1	HST7	MAP kinase kinase involved in mating and hyphal growth signal transduction pathways; phosphorylates Cek1p; wild-type virulence in mouse systemic infection; functional homolog of S. cerevisiae Ste7p; mutants are hypersensitive to caspofungin
PIS51245.1	24.13242768	24.45739783	26.13232624	25.93980444	26.20097845	26.15928705	0.10191624	0.666	1.19	PIS51245.1	NOT3	Transcriptional regulator; not required for buccal epithelial cell adherence or virulence in mouse systemic infection; null mutant colonies exhibit slightly decreased filamentation ratio; required for yeast adherence to silicone substrate

PIS55702.1	22.06210844	21.41512622	21.18456917	23.67442238	16.758415	27.80268891	0.533140273	0.929	1.19	PIS55702.1	orf19.3142	Ortholog of C. dubliniensis CD36 : Cd36_46140, C. parapsilosis CDC317 : CPAR2_501210, C. auris B8441 : B9J08_001807 and Candida tenuis NRRL Y-1498 : CANTEDRAFT_97195
PIS58837.1	28.11717749	28.19945344	28.55768883	29.40422999	29.67197952	29.35553074	0.061385601	0.509	1.19	PIS58837.1	orf19.399	Probable serine/threonine protein kinase; null mutant grows slowly, lacks hyphal development, has abnormal actin polarization, and is hypersensitive to a sphingolipid inhibitor; appears to act in a pathway with FPK1, PKH2, and PKH3
PIS56552.1	25.563049	25.41529251	25.05263257	26.80958899	26.36224745	26.43997774	0.00355284	0.0334	1.19	PIS56552.1	orf19.4324	Has domain(s) with predicted role in mitochondrial respiratory chain complex I assembly and membrane localization
PIS54723.1	24.48291474	26.0562911	26.30323454	26.98414564	26.50583058	26.93566622	0.024630007	0.272	1.19	PIS54723.1	orf19.6358	Predicted ubiquitin conjugating enzyme involved in DNA damage response; forms a complex with Ubc13p
PIS58665.1	25.89649328	22.77520564	26.32902365	26.23515867	26.69371801	25.62319565	0.272743394	0.864	1.18	PIS58665.1	orf19.6432	Ortholog(s) have ATPase activity, role in response to xenobiotic stimulus, ribosomal large subunit biogenesis and preribosome, large subunit precursor localization
PIS50550.1	23.83440401	24.34949778	24.96187299	26.38469374	25.12394561	25.18379114	0.038477183	0.37	1.18	PIS50550.1	RHR2	Glycerol 3-phosphatase; roles in osmotic tolerance, glycerol accumulation in response to salt; Spider/flow model biofilm induced; regulated by macrophage, stress, yeast-hyphal switch, pheromone, Gcn4, Hog1, Nrg1, Tup1
PIS58296.1	29.88069217	30.2051876	29.54621343	30.96167888	31.12097089	31.07905987	0.180440157	0.799	1.18	PIS58296.1	RVS167	SH3-domain- and BAR domain-containing protein involved in endocytosis; null mutant exhibits defects in hyphal growth, virulence, cell wall integrity, and actin patch localization; cosediments with phosphorylated Myo5p
PIS54507.1	23.31985767	25.33502864	21.26854733	25.06430147	24.01425579	24.38018608	0.143345548	0.751	1.18	PIS54507.1	TAF145	Protein similar to S. cerevisiae Taf145p, a component of RNA polymerase II transcription factor TFIID; flucytosine repressed; likely to be essential for growth, based on an insertional mutagenesis strategy
PIS51866.1	26.14658701	26.01934922	26.60916603	27.53092358	27.54352127	27.22323331	0.010677487	0.13	1.17	PIS51866.1	orf19.3762	Ortholog of C. dubliniensis CD36 : Cd36_11720, C. parapsilosis CDC317 : CPAR2_201890, C. auris B8441 : B9J08_003468 and Candida tenuis NRRL Y-1498 : CANTEDRAFT_91948
PIS51454.1	27.99204266	26.73753336	27.99955975	28.60072017	28.13655143	29.50696261	0.127075385	0.723	1.17	PIS51454.1	RRP15	Putative nucleolar protein; constituent of pre-60S ribosomal particles; Hap43-induced; repressed by prostaglandins

PIS58190.1	24.2006732	23.03879596	25.01240829	25.36426645	25.57837494	24.79969549	0.145313799	0.754	1.16	PIS58190.1	orf19.5175	Putative ubiquitin protein ligase with ubiquitin-protein transferase activity; null mutant is sensitive to proteotoxic stress and shows differential expression of other proteins likely involved in ER-associated protein catabolism
PIS51541.1	27.34384213	27.39622857	27.34299301	28.49168127	28.64620197	28.43966508	0.168918464	0.786	1.16	PIS51541.1	YAK1	Putative dual-specificity tyrosine-phosphorylation regulated kinase; involved in hyphal growth regulation and biofilm formation; appears to act either downstream or in parallel with the RAS/cAMP/PKA pathway
PIS58293.1	27.20303079	27.95262646	27.14728386	28.55663246	28.85794056	28.32336859	0.059751763	0.5	1.15	PIS58293.1	DES1	Putative delta-4 sphingolipid desaturase; planktonic growth-induced gene
PIS51188.1	31.42274029	31.15685726	31.3904515	32.51072738	32.23992951	32.66622865	0.003946424	0.0389	1.15	PIS51188.1	INO1	Inositol-1-phosphate synthase; antigenic in human; repressed by farnesol in biofilm or by caspofungin; upstream inositol/choline regulatory element; glycosylation predicted; rat catheter, flow model induced; Spider biofilm repressed
PIS58037.1	26.53787337	25.18255257	24.00241998	25.89307583	26.74499109	26.54648103	0.060294715	0.503	1.15	PIS58037.1	orf19.1195	Putative metalloendopeptidase; orthologs are involved in cellular iron ion homeostasis, protein processing involved in protein targeting to mitochondrion, protein stabilization, and mitochondrial matrix localization
PIS54892.1	26.23049766	25.71232868	27.33486433	26.56187951	28.00816414	28.15764993	0.035588149	0.352	1.15	PIS54892.1	orf19.4953	Putative ATPase; predicted role in ER-associated protein catabolism; induced during chlamydospore formation in both C. albicans and C. dubliniensis; rat catheter biofilm repressed
PIS54755.1	27.05203501	26.49783765	27.24529601	27.99254818	27.92660624	28.32903612	0.002414715	0.0208	1.15	PIS54755.1	orf19.5817	Ortholog(s) have guanyl-nucleotide exchange factor activity and role in cytoplasm to vacuole transport by the Cvt pathway, endoplasmic reticulum to Golgi vesicle-mediated transport, intra-Golgi vesicle-mediated transport, macroautophagy
PIS48437.1	23.44841592	23.52018316	23.58727189	24.88047901	24.67589966	24.44697052	0.004650024	0.0495	1.15	PIS48437.1	orf19.708	Ortholog(s) have gamma-tubulin binding activity
PIS50456.1	27.35828267	24.68283734	27.85121557	28.54302049	27.94633858	26.85136746	0.201643066	0.818	1.15	PIS50456.1	orf19.879	Ortholog(s) have myosin binding activity, role in intracellular mRNA localization, mating type switching and cytoplasm localization
PIS55086.1	28.79059154	28.32767316	29.14376516	29.62491149	29.77188109	30.28787553	0.01380875	0.167	1.14	PIS55086.1	orf19.3477	Putative pseudouridine synthase; predicted role in snRNA pseudouridine synthesis, tRNA pseudouridine synthesis; Spider biofilm induced

PIS55575.1	25.91856192	27.85867511	26.50883501	27.28149694	28.23732806	28.15536763	0.036390935	0.357	1.13	PIS55575.1	CPH2	Myc-bHLH transcription factor; promotes hyphal growth; directly regulates Tec1 to induce hypha-specific genes; probably homodimeric, phosphorylated; required for colonization of the mouse GI tract; rat catheter and Spider biofilm
PIS52111.1	22.81744514	23.42686985	23.65939988	24.15473933	24.46087312	24.66461927	0.035267664	0.35	1.13	PIS52111.1	LYS22	Homocitrate synthase, minor isoform; repressed by nitric oxide and by hypoxia; protein level decreases in stationary phase cultures; induced by ketoconazole, Spider biofilm induced; flow model biofilm repressed
PIS50352.1	24.3408981	20.80359424	23.06027239	24.58710778	23.39715413	23.6211418	0.324413099	0.885	1.13	PIS50352.1	orf19.173	C2H2 transcription factor; induced by Mnl1 under weak acid stress
PIS56619.1	25.99666545	24.80213641	26.0528263	27.03720113	26.59805321	26.60851485	0.050197746	0.445	1.13	PIS56619.1	orf19.7058	Ortholog of S. cerevisiae : RMD8, C. glabrata CBS138 : CAGL0C01969g, C. dubliniensis CD36 : Cd36_70050, C. parapsilosis CDC317 : CPAR2_301100 and C. auris B8441 : B9J08_001157
PIS49702.1	25.70751777	27.36614057	26.64504701	27.70939788	27.43903158	27.9419295	0.019884943	0.23	1.12	PIS49702.1	BEM2	Putative Rho1p GTPase activating protein (GAP); serum-induced transcript; Spider biofilm induced; flow model biofilm repressed
PIS58808.1	28.9621455	28.46276512	28.50113425	29.80716134	29.76480055	29.71852598	0.00212718	0.0178	1.12	PIS58808.1	ERG1	Squalene epoxidase, epoxidation of squalene to 2,3(S)-oxidosqualene; ergosterol biosynthesis; allylamine antifungal drug target; NADH reducing cofactor but S. cerevisiae Erg1 uses NADPH; flow model biofilm induced; Spider biofilm repressed
PIS54682.1	23.06170782	21.84815011	23.27479346	24.42926487	23.7578993	23.36258424	0.063406707	0.52	1.12	PIS54682.1	orf19.3226	Ortholog(s) have phosphatidylcholine binding, phosphatidylinositol binding, phosphatidylserine binding, sterol binding activity, role in intracellular sterol transport, sterol transport and fungal-type vacuole lumen localization
PIS55084.1	30.9056301	31.22600805	29.71017833	31.28046818	31.90831862	32.00518832	0.087694885	0.623	1.12	PIS55084.1	orf19.3475	Described as a Gag-related protein; hyphal induced; downregulation correlates with clinical development of fluconazole resistance; repressed by nitric oxide, 17-beta-estradiol, ethynyl estradiol
PIS51637.1	27.65632278	28.21705627	27.88165754	28.92880058	29.15130213	29.01006153	0.215249433	0.829	1.11	PIS51637.1	CNS1	Putative co-chaperone; Hap43p-induced gene; mutation confers hypersensitivity to radicicol
PIS55839.1	25.1712453	24.30340193	25.23090656	26.78291625	26.01378407	25.22644676	0.119964387	0.709	1.11	PIS55839.1	orf19.1404	Predicted tRNA dihydrouridine synthase; Spider biofilm induced

PIS49731.1	26.84581185	28.23870476	26.34291115	27.7689722	28.12547559	28.85552279	0.097567682	0.654	1.11	PIS49731.1	orf19.6788	Protein with a predicted role in cotranslational protein targeting to membrane; induced during chlamydospore formation in both C. albicans and C. dubliniensis
PIS51729.1	23.53716366	22.93386117	24.02158776	24.41104383	25.09388419	24.28838629	0.096520456	0.651	1.1	PIS51729.1	orf19.2043	Has domain(s) with predicted ATP binding, ATP-dependent FeS chaperone activity, iron-sulfur cluster binding activity and role in iron-sulfur cluster assembly
PIS58453.1	21.68656833	25.40785203	21.84951033	24.26956768	23.63238901	24.34853622	0.1570753	0.771	1.1	PIS58453.1	orf19.3686	Ortholog(s) have protein domain specific binding activity, role in mitochondrial proton-transporting ATP synthase complex assembly and mitochondrion localization
PIS56573.1	26.16318722	26.4108325	24.64495517	26.83263265	26.97098959	26.7221052	0.063066268	0.518	1.1	PIS56573.1	orf19.6246	Ortholog(s) have peptide alpha-N-acetyltransferase activity and role in N-terminal peptidyl-methionine acetylation, mitochondrion inheritance, regulation of actin cytoskeleton organization, ubiquitin-dependent ERAD pathway
PIS55546.1	30.36589658	31.07226555	30.31665818	31.57631017	31.58639908	31.87296001	0.009539272	0.118	1.09	PIS55546.1	ARO8	Aromatic transaminase of the Ehrlich fusel oil pathway of aromatic alcohol biosynthesis; Rim101 independent alkaline induction; protein abundance affected by URA3 expression in CAI-4 strain; Gcn4-regulated; stationary phase enriched
PIS56615.1	27.3334004	27.18415031	26.70635231	27.8740743	28.06553938	28.54548954	0.022027317	0.251	1.09	PIS56615.1	orf19.1864	Ortholog(s) have protein-macromolecule adaptor activity and role in proteasome-mediated ubiquitin-dependent protein catabolic process, rescue of stalled ribosome, ribosome-associated ubiquitin-dependent protein catabolic process
PIS55481.1	26.36120781	26.9992061	23.18260231	27.17555858	26.50646351	26.13669302	0.214593977	0.829	1.09	PIS55481.1	PTC1	Putative protein phosphatase of the Type 2C-related family (serine/threonine-specific), similar to S. cerevisiae Ptc1p; mutant shows virulence defect
PIS54715.1	29.42067732	29.11027768	28.82983663	30.18041338	30.1489698	30.26315815	0.009003499	0.111	1.08	PIS54715.1	MTR10	Putative importin; member of a family of fungal-specific nuclear importins; Mig1-regulated
PIS58358.1	29.86232274	28.57110105	29.81636495	31.16494778	30.16222366	30.13878913	0.401840795	0.906	1.07	PIS58358.1	HHO1	Putative histone H1; farnesol regulated; Hap43-induced; contains 5' UTR intron; Spider biofilm repressed
PIS49653.1	26.33402446	26.94021507	27.17739846	29.3099983	27.0704539	27.26783564	0.101037714	0.663	1.07	PIS49653.1	TRS33	Putative TRAPP complex subunit; constitutive expression independent of MTL or white-opaque status

PIS54492.1	20.96975516	18.19570398	20.74396913	22.67465928	21.75452416	18.69508802	0.403135928	0.907	1.07	PIS54492.1	TRY4	C2H2 transcription factor; fluconazole-repressed; induced in ssr1 mutant; required for yeast cell adherence to silicone substrate; Spider biofilm induced
PIS54821.1	23.05483873	22.63792525	22.74804097	24.89004432	23.36078467	23.36381141	0.276894224	0.866	1.06	PIS54821.1	CDC43	Beta subunit of heterodimeric protein geranylgeranyltransferase type I; GGTase I enzyme binds zinc, is Mg-dependent; Cdc42p is GGTase I substrate
PIS52013.1	29.63998653	29.54497289	29.81929588	30.80584602	30.70940251	30.65475417	0.002675471	0.0239	1.06	PIS52013.1	DCK1	Putative guanine nucleotide exchange factor; required for embedded filamentous growth; activates Rac1; has a DOCKER domain; similar to adjacent DCK2 and to S. cerevisiae Ylr422wp; regulated by Nrg1; Spider biofilm induced
PIS58119.1	22.83670497	22.96360745	22.75485668	24.66990967	23.59412753	23.47973111	0.426957045	0.912	1.06	PIS58119.1	orf19.6263	Predicted MFS membrane transporter; member of the monocarboxylate porter (MCP) family; Spider biofilm induced
PIS49805.1	26.70600189	25.38842104	25.99947904	26.790464	27.19375838	27.2607078	0.068975793	0.548	1.05	PIS49805.1	ECM15	Protein of unknown function; predicted role in cell wall organization; Hap43-repressed; caspofungin repressed; Spider biofilm induced; rat catheter biofilm repressed
PIS51783.1	25.4725293	25.43602633	25.39814601	26.01836835	26.95147663	26.48563619	0.252135887	0.853	1.05	PIS51783.1	NOP4	Putative nucleolar protein; Hap43-induced; mutation confers hypersensitivity to 5-fluorocytosine (5-FC), 5-fluorouracil (5-FU), and tubercidin (7-deazaadenosine); represses in core stress response
PIS58541.1	26.3235619	24.79773286	26.47562296	27.30771052	27.17051018	26.26815286	0.058366194	0.493	1.05	PIS58541.1	orf19.178	Has domain(s) with predicted role in peroxisome fission and peroxisomal membrane localization
PIS51749.1	25.68295	23.80830019	25.64543705	25.86734071	26.19801991	26.21128576	0.058779757	0.495	1.05	PIS51749.1	orf19.3806	Ortholog(s) have role in negative regulation of gluconeogenesis, proteasome-mediated ubiquitin-dependent protein catabolic process, traversing start control point of mitotic cell cycle and GID complex localization
PIS52046.1	25.89116023	23.81063117	27.0937056	26.58621665	26.63125526	26.72621036	0.114693794	0.698	1.05	PIS52046.1	ZRT2	Zinc transporter, essential for zinc uptake and acidic conditions tolerance; transcript induced by amphotericin B, interaction with macrophages; induced in oralpharyngeal candidiasis; Spider biofilm induced
PIS54847.1	27.02377083	26.01480219	24.98676547	27.06384543	27.20908847	26.87989348	0.038652786	0.371	1.04	PIS54847.1	BET4	Ortholog(s) have Rab geranylgeranyltransferase activity and role in endoplasmic reticulum to Golgi vesicle-mediated transport, protein geranylgeranylation, protein targeting to membrane

PIS51885.1	26.06202363	21.73969786	27.88213161	26.66034323	26.03763109	26.1166051	0.417201025	0.91	1.04	PIS51885.1	orf19.3260	Putative phosphopantothenoylcysteine decarboxylase, binds to protein phosphatase Ppz1p and regulates its activity
PIS58535.1	25.02362609	25.40849923	24.8103078	24.84737477	26.74028067	26.78206389	0.09494753	0.646	1.04	PIS58535.1	orf19.3698	Protein of unknown function that may function in RNA processing; filament induced
PIS51300.1	22.6776917	24.53549386	21.60201978	22.61285378	24.91209199	24.41690944	0.508788218	0.926	1.04	PIS51300.1	orf19.4715	Ortholog(s) have phosphatidylinositol-4,5-bisphosphate binding, tubulin binding activity
PIS49534.1	25.65745909	25.3812134	26.29659547	27.15314377	26.80245394	26.49485965	0.089266508	0.628	1.04	PIS49534.1	orf19.4728	Ortholog(s) have role in chromatin remodeling and Set3 complex localization
PIS54792.1	26.44292213	24.61155618	26.59934274	26.97830082	26.98296718	26.80709749	0.052036527	0.455	1.04	PIS54792.1	orf19.7102	Has domain(s) with predicted nucleic acid binding activity
PIS54862.1	22.85978299	23.96292078	23.43299281	24.74728904	24.12089988	24.49140837	0.179541388	0.798	1.03	PIS54862.1	orf19.3463	Putative GTPase; role in 60S ribosomal subunit biogenesis; Spider biofilm induced
PIS49666.1	26.6838907	27.47276655	22.10130751	26.17283004	26.3253646	26.85652997	0.464608827	0.919	1.03	PIS49666.1	SEC9	t-SNARE protein required for secretory vesicle-membrane fusion
PIS51221.1	27.11995155	25.03628424	26.94727636	27.51231876	27.29328277	27.36859824	0.051879718	0.455	1.02	PIS51221.1	FLU1	Multidrug efflux pump of the plasma membrane; MDR family member of the MFS (major facilitator superfamily) of transporters; involved in histatin 5 efflux; fungal-specific (no human/murine homolog)
PIS58540.1	23.34937453	24.88234798	24.87193786	25.10338981	25.62159429	25.44266534	0.063050594	0.518	1.02	PIS58540.1	orf19.1841	Protein of unknown function; Hap43-induced gene
PIS51202.1	25.84811797	24.24504379	25.47793291	26.86861991	26.0195322	25.73151071	0.047621126	0.429	1.02	PIS51202.1	orf19.5782	Ortholog(s) have phospholipase activity, role in cardiolipin metabolic process, phosphatidylethanolamine metabolic process, phospholipid metabolic process and mitochondrial matrix localization

PIS56905.1	23.83792359	25.53440788	24.45550597	24.74497659	25.81151655	26.3424171	0.112278758	0.692	1.02	PIS56905.1	RMT2	Minor protein arginine methyltransferases (PRMT) involved in methylation of arginine residues
PIS55805.1	26.25000337	25.47573232	25.55727787	27.24209913	27.20844956	25.85111602	0.081106511	0.599	1.01	PIS55805.1	orf19.2436	SR-like protein kinase involved in osmotic stress and polyamine resistance; expression analysis suggests roles in mRNA processing and mitochondrial function; Spider biofilm induced; null mutant shows resistance to hygromycin B
PIS50576.1	26.36932986	24.33659314	26.81967074	27.08603558	26.71542687	26.76907134	0.195045912	0.813	1.01	PIS50576.1	orf19.4264	Protein of unknown function; induced during chlamydospore formation in both C. albicans and C. dubliniensis; flow model biofilm induced
PIS58639.1	24.89507934	24.16892388	24.98765569	26.09913059	25.84746027	25.12065809	0.145609955	0.755	1.01	PIS58639.1	orf19.5491	Ortholog of C. dubliniensis CD36 : Cd36_20670, C. parapsilosis CDC317 : CPAR2_104720, C. auris B8441 : B9J08_000085 and Candida tenuis NRRL Y-1498 : CANTEDRAFT_103989
PIS56792.1	26.53427174	23.12852263	25.74214427	26.0382066	26.09372539	26.26847337	0.250929377	0.853	0.998	PIS56792.1	orf19.443	Ortholog(s) have RNA polymerase III activity, role in tRNA transcription by RNA polymerase III, transcription initiation at RNA polymerase III promoter and RNA polymerase III complex, chromatin localization
PIS49713.1	25.50387562	27.4807548	25.37439438	26.50807998	27.53402768	27.30811684	0.132046326	0.732	0.997	PIS49713.1	NOP15	Nucleolar ribosome biogenesis factor; hyphal-induced expression; Hap43-induced; rat catheter biofilm induced
PIS51687.1	26.59267401	25.44020518	26.42033252	27.26689443	27.12052655	27.05398588	0.179058303	0.797	0.996	PIS51687.1	orf19.7043	Ortholog(s) have role in protein targeting to mitochondrion
PIS51482.1	25.32207311	27.78152511	24.71868931	26.64219459	26.89508666	27.26972314	0.197268268	0.815	0.995	PIS51482.1	CTF5	Predicted component of the kinetochore sub-complex COMA; induced during the mating process; repressed by alpha pheromone in SpiderM medium
PIS56717.1	21.13085289	21.24677186	24.14493496	24.50200006	23.83111818	21.17414002	0.489867771	0.923	0.995	PIS56717.1	POP3	Putative RNase MRP and nuclear RNase P component; decreased repressed by prostaglandins; Spider biofilm induced
PIS56751.1	25.84526739	27.31949214	26.17138322	27.40894245	27.38081315	27.52961833	0.025858657	0.282	0.994	PIS56751.1	orf19.2050	Ortholog(s) have sterol esterase activity, role in cellular lipid metabolic process, sterol metabolic process and lipid droplet, membrane localization

PIS55705.1	25.03549401	24.87707643	25.6023546	26.3077304	26.22958071	25.96059826	0.052780743	0.46	0.994	PIS55705.1	orf19.2208	Ortholog(s) have role in negative regulation of translation, response to salt stress and P-body localization
PIS58859.1	27.03106646	26.35477486	26.96397214	27.92313808	27.77284111	27.63028952	0.016194249	0.191	0.992	PIS58859.1	orf19.2302	Ortholog(s) have calcium-release channel activity, enzyme regulator activity
PIS52203.1	26.28907633	26.35867074	25.9255759	27.38252363	27.19436235	26.95700405	0.133997553	0.736	0.987	PIS52203.1	orf19.6933	Ortholog(s) have peptidyl-prolyl cis-trans isomerase activity, protein phosphatase regulator activity, role in mitotic spindle organization, response to osmotic stress and protein phosphatase type 2A complex localization
PIS54628.1	24.19133709	24.5037502	24.46884335	25.9031931	25.3381476	24.88277627	0.156923455	0.771	0.987	PIS54628.1	orf19.7345	Ortholog(s) have ATP binding activity
PIS51435.1	26.34533123	25.50218259	26.05993667	26.80562994	27.07433609	26.98189177	0.011379953	0.139	0.985	PIS51435.1	BMT8	Putative beta-mannosyltransferase, member of a 9-gene family including characterized BMT genes with roles in beta-1,2-mannosylation of cell wall phosphopeptidomannan; transposon insertion in promoter region causes decreased colony wrinkling
PIS58151.1	26.51061171	26.71258773	25.24373683	26.98682097	26.71869701	27.71011679	0.238931132	0.845	0.983	PIS58151.1	orf19.3021	Putative protein of unknown function; Hap43-repressed gene; Spider biofilm induced
PIS58673.1	25.95887885	26.42289742	25.61408716	27.08693628	27.43258395	26.4244976	0.189874284	0.808	0.983	PIS58673.1	orf19.6419.1	Ortholog of C. parapsilosis CDC317 : CPAR2_205710, C. auris B8441 : B9J08_000119, Candida tenuis NRRL Y-1498 : CANTEDRAFT_117073 and Debaryomyces hansenii CBS767 : DEHA2A11462g
PIS51576.1	24.07472639	25.3616049	24.97186559	25.65950582	25.69386872	25.99738543	0.110938102	0.689	0.981	PIS51576.1	orf19.6062	Putative TIM23 translocase complex subunit; membrane-localized; Hap43-repressed
PIS50326.1	25.78468269	24.24994837	25.88996367	26.11008662	26.33838335	26.40319725	0.148087316	0.759	0.976	PIS50326.1	orf19.4922	Ortholog(s) have small GTPase binding activity and Golgi apparatus localization
PIS51225.1	26.45891653	27.24910277	26.45061641	27.24966404	28.00666892	27.82782043	0.277422365	0.866	0.975	PIS51225.1	orf19.2263	Ortholog of C. dubliniensis CD36 : Cd36_21360, C. parapsilosis CDC317 : CPAR2_406560, C. auris B8441 : B9J08_002799 and Candida tenuis NRRL Y-1498 : CANTEDRAFT_103408

PIS58723.1	24.84590687	23.71284034	24.93851302	26.18194358	25.27842611	24.9514519	0.047512336	0.429	0.972	PIS58723.1	ITR1	MFS inositol transporter; uptake of exogenous inositol; 12 transmembrane motifs; expressed in rich medium; fluconazole, caspofungin repressed; possibly essential (UAU1 method); flow model, rat catheter and Spider biofilm induced
PIS54845.1	26.71938221	26.74430842	26.62943811	27.56459517	27.72260847	27.71703496	0.152209813	0.764	0.97	PIS54845.1	KAP120	Putative karyopherin; mutation confers hypersensitivity to cytochalasin D
PIS56749.1	21.72255655	26.22113096	25.73912505	24.92711949	25.81952587	25.83985685	0.468896448	0.919	0.968	PIS56749.1	FGR15	Putative transcription factor with zinc finger DNA-binding motif; lacks an ortholog in S. cerevisiae; transposon mutation affects filamentous growth; caspofungin induced; mutation causes marginal increase in caspofungin sensitivity
PIS55076.1	27.3529437	27.04333055	27.07848508	28.09962948	28.0313994	28.24497891	0.189152335	0.807	0.967	PIS55076.1	CSO99	Protein of unknown function; Hap43-repressed gene; protein not conserved in S. cerevisiae
PIS58949.1	26.32356285	26.31918612	26.59403241	27.45673581	26.74327097	27.93747825	0.080008706	0.595	0.967	PIS58949.1	KRE5	UDP-glucose:glycoprotein glucosyltransferase; 1,6-beta-D-glucan biosynthesis, hyphal growth, virulence in mouse IV model; partially complements S. cerevisiae kre5 mutant defects; flow biofilm repressed, Bcr1-repressed in RPMI a/a biofilms
PIS56685.1	27.36317334	28.6211435	27.47459318	28.13683041	29.08814237	29.12027981	0.267479118	0.861	0.962	PIS56685.1	ECM17	Putative sulfite reductase beta subunit; role in cell wall biogenesis; regulated by Tsa1/Tsa1B in H2O2 stress; Gcn4-regulated; Tbf1-activated; Hap43-repressed; Spider, flow, F-12/CO2 model biofilm induced
PIS51439.1	24.41670288	26.79275439	26.45647916	26.85672993	26.9921301	26.70324457	0.14990933	0.761	0.962	PIS51439.1	orf19.4780	Predicted MFS family membrane transporter, member of the drug:proton antiporter (12 spanner) (DHA1) family; Spider biofilm induced
PIS54813.1	26.56063022	24.53882254	26.25657185	25.83996235	25.45512911	28.92755599	0.432195989	0.913	0.956	PIS54813.1	HLJ1	Putative HSP40 co-chaperone; repressed during the mating process
PIS51836.1	26.97123241	24.40181214	26.98555185	27.05482054	27.12575065	27.03415977	0.180116507	0.798	0.952	PIS51836.1	PRX1	Thioredoxin peroxidase; transcriptionally induced by interaction with macrophage; fluconazole induced; Fkh2p-downregulated; caspofungin repressed; protein present in exponential and stationary growth phase yeast cultures
PIS58524.1	26.86218605	27.40219854	26.69070479	28.0860805	28.00678233	27.71871541	0.173396971	0.791	0.952	PIS58524.1	PWP2	Putative 90S pre-ribosomal component; repressed in core stress response; repressed by prostaglandins; physically interacts with TAP-tagged Nop1; Hap43-induced

PIS54583.1	26.52861065	25.87423501	26.66108934	27.86915937	27.65213816	26.39264296	0.241972618	0.847	0.95	PIS54583.1	orf19.158	Ortholog of S. cerevisiae Apd1; required for normal localization of actin patches and normal tolerance of sodium ions and hydrogen peroxide; Hap43-induced; Spider biofilm induced
PIS56929.1	25.24799022	23.66561289	23.56430149	24.05864798	25.61915023	25.64257786	0.344829635	0.891	0.947	PIS56929.1	orf19.4031	Ortholog(s) have steryl-beta-glucosidase activity, role in ergosteryl 3-beta-D-glucoside catabolic process and cytosol localization
PIS51650.1	26.16711756	22.13871781	27.19786177	26.72241674	25.83746207	25.78556054	0.404054496	0.907	0.947	PIS51650.1	RIC1	Ortholog of S. cerevisiae Ric1 guanyl-nucleotide exchange factor; mutant is viable; rat catheter biofilm repressed
PIS56854.1	24.73730527	23.12576693	24.63811676	25.42538118	24.69733512	25.21734693	0.210743162	0.826	0.946	PIS56854.1	TFG1	Protein similar to S. cerevisiae Tfg1p, which is part of transcription factor TFIIF; transposon mutation affects filamentous growth; possibly an essential gene, disruptants not obtained by UAU1 method
PIS51365.1	25.99538355	26.88128729	26.43175537	26.221779	27.99404147	27.92790074	0.135674773	0.739	0.945	PIS51365.1	HIS3	Imidazoleglycerol-phosphate dehydratase, enzyme of histidine biosynthesis; functionally complements S. cerevisiae his3-1 mutation; hyphal-induced expression; regulated by Gcn2p and Gcn4p; fungal-specific (no human or murine homolog)
PIS55037.1	25.37683194	24.35511057	25.09510545	27.33491857	25.50070365	24.82482806	0.219055708	0.832	0.944	PIS55037.1	orf19.2073	Protein with a multidrug and toxin extrusion protein domain; induced by Mnl1 under weak acid stress
PIS55576.1	26.34986748	24.435798	26.41693231	27.39579985	26.0672186	26.54857269	0.104960886	0.674	0.936	PIS55576.1	orf19.6943	Predicted ORF from Assembly 19; removed from Assembly 20; subsequently reinstated in Assembly 21 based on comparative genome analysis
PIS55506.1	29.92351982	31.03516099	29.81461428	30.86056979	31.36855613	31.34414619	0.074636287	0.573	0.933	PIS55506.1	ADE5,7	Phosphoribosylamine-glycine ligase and phosphoribosylformylglycinamidine cyclo-ligase; interacts with Vps34p; required for hyphal growth and virulence; flucytosine induced; not induced in GCN response, in contrast to S. cerevisiae ortholog
PIS51438.1	27.55507241	27.58041094	27.81689458	28.95109088	28.43429661	28.36570718	0.216472756	0.83	0.933	PIS51438.1	orf19.4699	Putative phospholipase of patatin family; similar to S. cerevisiae Tgl3p; predicted Kex2p substrate
PIS48631.1	24.65199593	23.78896542	24.8570372	25.60294001	25.20394539	25.28402367	0.03725656	0.362	0.931	PIS48631.1	STE11	Protein similar to S. cerevisiae Ste11p; mutants are sensitive to growth on H2O2 medium

PIS48410.1	24.11203048	23.59248946	24.27049013	25.39045169	24.90031649	24.46709296	0.210003293	0.825	0.928	PIS48410.1	orf19.3517	Ortholog(s) have role in SREBP signaling pathway and Dsc E3 ubiquitin ligase complex, endoplasmic reticulum, fungal-type vacuole lumen localization
PIS52217.1	31.62175085	31.55308513	32.03880014	32.85480158	32.61243123	32.52697168	0.008395921	0.103	0.927	PIS52217.1	PTR22	Oligopeptide transporter involved in uptake of di-/tripeptides; regulated by Stp2 and Stp3; transcript induced upon phagocytosis by macrophage; repressed by Rim101 at pH 8; flow model biofilm induced
PIS48218.1	27.30946503	27.34190073	26.3139254	27.8923194	27.74914061	28.10140721	0.027908431	0.296	0.926	PIS48218.1	orf19.730	Ortholog(s) have GTPase activator activity and role in establishment or maintenance of actin cytoskeleton polarity, small GTPase mediated signal transduction
PIS51203.1	29.7321338	28.93462193	29.94632047	30.34780551	30.60919534	30.43017692	0.043331205	0.401	0.925	PIS51203.1	orf19.5686	Protein of unknown function; Spider biofilm induced
PIS48317.1	29.09449921	29.06095986	28.92771618	29.78470126	30.25747032	29.81284525	0.076917593	0.582	0.924	PIS48317.1	ERG7	2,3-epoxysqualene-lanosterol cyclase (lanosterol synthase), conversion of 2,3-oxidosqualene to lanosterol in sterol biosynthesis; fluconazole-induced; possibly essential, disruptants not obtained by UAU1 method; rat catheter biofilm induced
PIS58084.1	25.76175921	26.02302759	25.3808749	26.61640989	26.61848122	26.69789012	0.037492738	0.364	0.922	PIS58084.1	orf19.6135	Ortholog of C. dubliniensis CD36 : Cd36_32970, C. parapsilosis CDC317 : CPAR2_701710, C. auris B8441 : B9J08_000571 and Candida tenuis NRRL Y-1498 : CANTEDRAFT_106599
PIS51191.1	25.02320753	27.27905813	26.40355224	27.42361449	26.99927644	27.04356141	0.375440407	0.9	0.92	PIS51191.1	orf19.7603	Protein with a predicted role in cytochrome c oxidase assembly; rat catheter biofilm induced
PIS54534.1	27.28313802	25.5904691	26.47215555	27.11774852	27.41577838	27.56268378	0.170056047	0.787	0.917	PIS54534.1	orf19.4798	Component of a complex with Ypp1p and Stt4p that is required for phosphatidylinositol-4-phosphate, PI(4)P, in plasma membrane; required for invasive growth and cell wall organization
PIS50560.1	26.79180386	24.60310364	26.65972252	26.90578212	26.93674196	26.9544805	0.086748648	0.619	0.914	PIS50560.1	orf19.4262	Ortholog(s) have mRNA binding activity and role in establishment of mitochondrion localization, nuclear-transcribed mRNA catabolic process, deadenylation-dependent decay, protein-containing complex localization
PIS56637.1	25.35880903	25.80667767	24.91943189	25.71938557	27.3710552	25.73212889	0.095272893	0.647	0.913	PIS56637.1	orf19.2574	Ortholog(s) have AP-1 adaptor complex binding activity, role in clathrin-coated vesicle cargo loading and membrane localization

PIS55709.1	24.47403669	25.34216208	25.98991707	26.28765256	26.26386114	25.99068259	0.313805582	0.881	0.912	PIS55709.1	GPX3	Putative glutathione peroxidase involved in Cap1p-dependent oxidative stress response, required for Cap1p oxidation in response to H2O2; planktonic growth-induced
PIS55056.1	28.16997929	29.11427197	27.26271959	28.75463977	29.07706757	29.42716563	0.221570859	0.834	0.904	PIS55056.1	CLC1	Clathrin light chain; subunit of the major coat protein involved in intracellular protein transport and endocytosis; rat cathetr and Spider biofilm repressed
PIS58591.1	28.12663463	29.24600419	28.38405846	28.94092501	29.75169109	29.77483898	0.059563783	0.499	0.904	PIS58591.1	CYM1	Putative metalloprotease of the mitochondrial intermembrane space; rat catheter biofilm induced
PIS51107.1	24.13956368	20.9515798	24.08198397	24.75795033	20.58152306	26.54533247	0.547006523	0.931	0.904	PIS51107.1	EPL1	Subunit of the NuA4 histone acetyltransferase complex
PIS52470.1	25.66442373	25.63642346	24.75784541	25.70279712	26.58336149	26.47970751	0.459706332	0.918	0.902	PIS52470.1	orf19.2826	Ortholog of C. dubliniensis CD36 : Cd36_27680, C. parapsilosis CDC317 : CPAR2_801140, C. auris B8441 : B9J08_004087 and Candida tenuis NRRL Y-1498 : CANTEDRAFT_129982
PIS55749.1	23.68878388	23.03198673	23.54382502	24.49345712	24.11383703	24.36168232	0.041237945	0.387	0.901	PIS55749.1	orf19.3644	Protein of unknown function; Cyr1-repressed; rat catheter and Spider biofilm induced
PIS51947.1	28.14454694	27.81519256	27.38980097	28.54202033	28.66905634	28.8415481	0.026325181	0.285	0.901	PIS51947.1	orf19.998	Putative adapter protein; links synaptojanins Inp52 and Inp53 to the cortical actin cytoskeleton in S. cerevisiae; mutants are viable
PIS56871.1	24.5004487	25.80062365	24.94133472	26.72325544	26.26696132	24.94786895	0.152738213	0.765	0.899	PIS56871.1	orf19.2703	Specificity factor required for ubiquitination and sorting of specific cargo proteins at the multivesicular body; ortholog of S. cerevisiae Ear1; transcript regulated by iron; Ssr1-represssed; Spider biofilm induced
PIS56665.1	28.28707524	27.16976053	28.30799759	28.86281866	28.92562933	28.67155483	0.036572401	0.358	0.898	PIS56665.1	orf19.273	Protein involved in regulation of C14-methylated sterol biosynthesis; mutations increase azole sensitivity
PIS55018.1	27.29499904	26.95488979	27.8726017	28.76283405	27.94228271	28.10298656	0.162402253	0.778	0.895	PIS55018.1	MAL31	Putative high-affinity maltose transporter; transcript is upregulated in clinical isolates from HIV+ patients with oral candidiasis; alkaline induced; Spider biofilm induced

PIS49736.1	24.98885386	26.21125188	26.40328425	27.24276979	26.86258037	26.18429263	0.430837951	0.913	0.895	PIS49736.1	MET8	Putative bifunctional dehydrogenase and ferrochelatase with a predicted role in siroheme biosynthesis; regulated by Gcn2p and Gcn4p
PIS51858.1	27.4899017	29.27070254	27.60776554	27.46588993	29.7984778	29.77860157	0.188835624	0.807	0.892	PIS51858.1	SWI1	Protein involved in transcription regulation; ortholog of S. cerevisiae Swi1p, which is a subunit of the SWI/SNF chromatin remodeling complex; interacts with Snf2p; SWI/SNF complex is essential for hyphal growth and virulence
PIS51388.1	28.92505352	28.58479532	28.57003949	29.74921971	29.6400264	29.36354284	0.003562046	0.0335	0.891	PIS51388.1	YPT52	Rab-family GTPase involved in vacuolar trafficking, colocolizes with Vps1p and Ypt53p in late endosome
PIS50479.1	29.42099612	28.73666557	29.67522857	30.25410241	30.12080955	30.12911691	0.013804005	0.167	0.89	PIS50479.1	orf19.3983	Protein with a predicted transcription factor BTF3 domain; flow model biofilm induced
PIS52478.1	24.83688018	25.98001707	26.26024509	25.8713392	26.39750055	27.47520601	0.108284233	0.682	0.889	PIS52478.1	PAN6	Ortholog(s) have pantoate-beta-alanine ligase activity and role in pantothenate biosynthetic process
PIS48408.1	27.30941569	25.37775792	26.66421715	26.96879462	27.80054306	27.24473688	0.080964011	0.598	0.888	PIS48408.1	orf19.3516	Protein of unknown function; Hap43-repressed; Spider biofilm repressed
PIS50543.1	26.20805309	25.73664426	25.90146091	27.14710663	26.50269082	26.86093343	0.203378417	0.82	0.888	PIS50543.1	VPS20	ESCRT III complex protein; role in multivesicular body (MVB) trafficking; required for processing of Rim8; Bcr1-repressed in RPMI a/a biofilms
PIS56777.1	25.59849802	24.65327211	25.03866542	26.58064506	26.33495731	25.03713254	0.170455002	0.788	0.887	PIS56777.1	orf19.302	Ortholog(s) have guanyl-nucleotide exchange factor activity, role in endoplasmic reticulum to Golgi vesicle-mediated transport and TRAPPI protein complex, TRAPPII protein complex, TRAPPIII protein complex localization
PIS48520.1	27.66556997	25.57823903	26.97715517	28.37735488	27.86316665	26.63748446	0.388395315	0.903	0.886	PIS48520.1	orf19.3778	Protein with a predicted role in ribosome biogenesis; mutation confers hypersensitivity to 5-fluorocytosine (5-FC), 5-fluorouracil (5-FU); repressed in core stress response; repressed by prostaglandins; Hap43-induced
PIS48447.1	25.5670741	27.26844814	25.36380591	25.02411296	27.95359567	27.86725154	0.263411603	0.859	0.882	PIS48447.1	orf19.1049	Predicted NUDIX hydrolase domain; Hap43-induced

PIS51802.1	23.41975487	23.83924631	23.55787846	24.44743607	24.96858966	24.0394788	0.195454619	0.813	0.88	PIS51802.1	CCH1	Voltage-gated Ca2+ channel of the high affinity calcium uptake system; roles in thigmotropism, establishment of galvanotropism; transcript regulated by Nrg1 and Mig1; flow model biofilm repressed
PIS49524.1	25.16019746	23.40756685	25.36407862	25.33245269	26.1284622	25.1120472	0.145183629	0.754	0.88	PIS49524.1	orf19.4756	Ortholog of S. cerevisiae : YTP1, C. dubliniensis CD36 : Cd36_08490, C. parapsilosis CDC317 : CPAR2_801590, C. auris B8441 : B9J08_004547 and Candida tenuis NRRL Y-1498 : CANTEDRAFT_109732
PIS58600.1	25.29481857	26.05990341	23.81403409	25.15656539	26.92174467	25.72718412	0.177282614	0.795	0.879	PIS58600.1	orf19.7425	Ortholog(s) have uracil DNA N-glycosylase activity, role in DNA repair, base-excision repair and cytoplasm, mitochondrion, nucleus localization
PIS56654.1	27.85272216	26.43355728	27.10669626	27.99797624	28.27009283	27.76149348	0.199845015	0.817	0.879	PIS56654.1	SNF2	Protein involved in transcriptional regulation; ortholog of S. cerevisiae Snf2p, which is the catalytic subunit of the SWI/SNF chromatin remodeling complex; interacts with Swi1p; SWI/SNF complex is essential for hyphal growth and virulence
PIS58799.1	24.04690145	25.67877298	23.76003001	25.27753431	25.86832827	24.97510932	0.442393027	0.915	0.878	PIS58799.1	BIG1	Endoplasmic reticulum (ER) protein; ortholog of S. cerevisiae Big1; required for beta-1,6-glucan synthesis, filamentation, adhesion, and virulence; rat catheter biofilm repressed
PIS49641.1	26.40324937	25.35930759	26.17643565	27.12311886	26.79511947	26.65176815	0.102180589	0.667	0.877	PIS49641.1	DAO1	Putative D-amino acid oxidase; transcription is regulated upon yeast-hyphal switch
PIS51160.1	24.7764721	25.36810153	24.55925528	26.33401219	25.37514452	25.62692095	0.183748388	0.802	0.877	PIS51160.1	orf19.6329	Protein of unknown function; opaque-specific transcript; fluconazole-repressed; induced in cyr1 mutant and in oralpharyngeal candidasis; Spider biofilm induced
PIS58870.1	24.76744376	23.04688752	24.83279391	24.48034258	25.44371886	25.35146303	0.159698524	0.775	0.876	PIS58870.1	orf19.4748	Putative U2B'' component of the U2 snRNP, involved in splicing; contains an RNA recognition motif (RRM); ortholog of S. cerevisiae MSL1; Hap43p-induced gene
PIS55620.1	29.77709453	30.04554686	30.08637997	30.62217776	30.82884934	31.0765189	0.023886814	0.266	0.873	PIS55620.1	PXA2	Putative peroxisomal, half-size adrenoleukodystrophy protein (ALD or ALDp) subfamily ABC transporter; Gcn4p-regulated
PIS51255.1	29.1456702	28.68852916	29.09934611	29.87515974	29.86406472	29.79180377	0.007863722	0.0962	0.866	PIS51255.1	AYR2	Putative NADPH-dependent 1-acyl dihydroxyacetone phosphate reductase; shows colony morphology-related gene regulation by Ssn6p

PIS48461.1	27.35880517	24.30546027	27.14230135	27.21915321	27.49662394	26.68863521	0.207867748	0.824	0.866	PIS48461.1	orf19.1364	Ortholog of S. pombe Stm1 G-protein coupled receptor; PQ-loop domains; constitutive expression independent of MTL or white-opaque status; Hap43-repressed
PIS52115.1	25.90193582	25.94232504	26.61939898	28.04422782	26.61040326	26.40243093	0.108860787	0.684	0.864	PIS52115.1	orf19.4518	Protein kinase that appears to be phosphorylated by Sky1p and Sky2p and itself phosphorylates Hrk1p; has a putative role in stress response; mutants are viable
PIS49482.1	22.64041757	24.84865148	23.89955763	23.28866918	24.91068117	25.77193253	0.444013279	0.915	0.861	PIS49482.1	orf19.7321	Ortholog(s) have role in re-entry into mitotic cell cycle after pheromone arrest and endoplasmic reticulum localization
PIS58538.1	25.70799178	24.14933586	26.68293472	26.80936691	26.02402631	26.28546744	0.184731471	0.803	0.86	PIS58538.1	orf19.3697	Ortholog(s) have role in mitotic intra-S DNA damage checkpoint signaling, positive regulation of macroautophagy, re-entry into mitotic cell cycle after pheromone arrest and Golgi trans cisterna, endoplasmic reticulum localization
PIS48525.1	27.61083067	27.36878071	27.66668802	28.61693897	28.40807833	28.20156778	0.088846361	0.626	0.86	PIS48525.1	USO1	Ortholog(s) have role in Golgi vesicle docking, SNARE complex assembly, endoplasmic reticulum to Golgi vesicle-mediated transport and ER to Golgi transport vesicle membrane, Golgi membrane localization
PIS52042.1	24.12799057	24.34438452	23.81278062	25.10713465	25.06145194	24.69196455	0.172932664	0.79	0.858	PIS52042.1	orf19.1547	Ortholog(s) have double-stranded DNA binding, polynucleotide 3'-phosphatase activity and role in double-strand break repair
PIS58143.1	27.66798018	26.74334329	27.10776187	27.29273344	28.93473937	27.8508465	0.286389792	0.87	0.853	PIS58143.1	SEC10	Ortholog(s) have role in Golgi to plasma membrane transport, exocytosis and cell division site, cell tip, exocyst localization
PIS51353.1	22.39999984	25.64385202	24.96979353	24.06872322	25.53354279	25.96786784	0.243745867	0.848	0.852	PIS51353.1	ZCF14	Putative Zn(II)2Cys6 transcription factor; caspofungin induced
PIS48283.1	25.40438689	26.14784392	25.52018766	26.43087025	26.30957015	26.8859904	0.319503293	0.883	0.851	PIS48283.1	orf19.5821	Ortholog of S. cerevisiae : VRL1, C. dubliniensis CD36 : Cd36_17530, C. parapsilosis CDC317 : CPAR2_212430, C. auris B8441 : B9J08_004968 and Candida tenuis NRRL Y-1498 : CANTEDRAFT_128807
PIS48342.1	23.45971816	22.95880842	23.46406598	24.48526612	24.00680476	23.94105139	0.442201929	0.915	0.85	PIS48342.1	SMP3	Mannosyltransferase of glycosylphosphatidylinositol (GPI) biosynthesis; catalyzes mannosylation of Man3-GPI precursor; essential for viability; 8-9 transmembrane regions predicted; has HQEXRF motif; functional homolog of S. cerevisiae Smp3p

PIS48469.1	22.29748295	23.4676843	22.55751477	23.39274493	23.64233292	23.83440249	0.029225683	0.306	0.849	PIS48469.1	orf19.6180	Protein similar to Pichia anomala YDL054c and S. cerevisiae Ydl054cp; transmembrane regions predicted; Hap43p-repressed gene
PIS48794.1	25.73352977	25.52089477	26.59499899	26.74093934	26.98523413	26.67008442	0.165729067	0.782	0.849	PIS48794.1	orf19.684	Putative transcription factor with zinc finger DNA-binding motif; heterozygous null mutant exhibits hypersensitivity to parnafungin and cordycepin in the C. albicans fitness test
PIS50593.1	23.21452978	23.4483462	24.1682617	24.76841099	24.18950676	24.4132431	0.045536596	0.416	0.847	PIS50593.1	ZCF21	Predicted Zn(II)2Cys6 transcription factor; mutants display increased colonization of mouse kidneys; Spider biofilm induced
PIS52223.1	22.95566423	23.70438147	25.17800746	23.17503718	25.65018529	25.54093294	0.2349337	0.843	0.843	PIS52223.1	NAG1	Glucosamine-6-phosphate deaminase; required for normal hyphal growth and mouse virulence; converts glucosamine 6-P to fructose 6-P; reversible reaction in vitro; gene and protein is GlcNAc-induced; Spider biofilm induced
PIS58770.1	26.2904556	25.80757656	26.75417188	27.55275084	26.90582885	26.92349582	0.11101945	0.689	0.843	PIS58770.1	orf19.2128	Has domain(s) with predicted ATP binding, ATPase, GTP binding activity
PIS48348.1	22.69789479	27.41838018	23.24704241	25.96821532	24.8031402	25.11717031	0.410669504	0.908	0.842	PIS48348.1	orf19.1248	Putative RNA polymerase II subunit B44; heterozygous null mutant exhibits resistance to parnafungin in the C. albicans fitness test; Spider biofilm repressed
PIS56830.1	23.95971277	25.62861943	24.531519	26.82255366	25.80581368	24.00409218	0.262847445	0.859	0.838	PIS56830.1	orf19.4760	Putative protein-histidine N-methyltransferase; Spider biofilm induced
PIS58597.1	25.88317586	26.30633728	27.75924538	27.89783772	27.52644548	27.03410517	0.115277407	0.699	0.837	PIS58597.1	orf19.7403	Ortholog of S. cerevisiae : YML020W, C. glabrata CBS138 : CAGL0G07062g, C. dubliniensis CD36 : Cd36_86190, C. parapsilosis CDC317 : CPAR2_404740 and C. auris B8441 : B9J08_000043
PIS48560.1	24.956907	23.64941429	24.66030965	24.55819991	25.52982185	25.68102434	0.177727634	0.796	0.834	PIS48560.1	TOS4	Putative fork-head transcription factor; rat catheter and Spider biofilm repressed
PIS52052.1	28.93675336	28.24293619	29.06227103	29.61551679	29.60213982	29.52373315	0.019289857	0.224	0.833	PIS52052.1	MDL2	Putative mitochondrial, half-size MDR-subfamily ABC transporter

PIS48711.1	26.01904781	24.45039012	26.18762421	26.74813723	26.23444304	26.17154981	0.118927567	0.707	0.832	PIS48711.1	orf19.6912	Putative ethanolamine kinase
PIS58324.1	27.52532828	27.52701655	27.69133095	28.77407944	28.6721527	27.79016719	0.12556073	0.72	0.831	PIS58324.1	CAF16	ABC family protein, predicted not to be a transporter; Hap43, caspofungin repressed; rat catheter and Spider biofilm repressed
PIS52312.1	27.53823895	27.09190336	27.59659932	28.36276774	28.21243019	28.14304303	0.094163833	0.643	0.83	PIS52312.1	ATP19	Subunit k of the mitochondrial F1F0 ATP synthase; a large enzyme complex required for ATP synthesis; Spider biofilm repressed
PIS58841.1	24.11381856	23.80011945	24.63522339	25.55433634	24.71855487	24.76432234	0.67560013	0.944	0.829	PIS58841.1	orf19.1417	Ortholog of C. dubliniensis CD36 : Cd36_43970, C. parapsilosis CDC317 : CPAR2_401720, C. auris B8441 : B9J08_000295 and Candida tenuis NRRL Y-1498 : CANTEDRAFT_102588
PIS49745.1	27.25382038	26.33423559	26.97170555	27.90243339	27.52100562	27.62354868	0.161037251	0.776	0.829	PIS49745.1	orf19.4905	Putative MFS transporter; Hap43p-induced gene; also regulated by regulated by Sef1p and Sfu1p; repressed in a ssr1 null mutant
PIS58471.1	25.25982547	25.55121172	23.56564402	25.37685782	25.59464543	25.87930455	0.09330367	0.641	0.825	PIS58471.1	orf19.6170	Ortholog(s) have kinesin binding, microtubule binding, microtubule plus-end binding activity
PIS50447.1	28.50063918	26.94661663	27.24284395	28.09713443	29.03672575	28.03272906	0.226204411	0.837	0.825	PIS50447.1	orf19.863	Ortholog(s) have structural constituent of ribosome activity, role in mitochondrial cytochrome c oxidase assembly and mitochondrial large ribosomal subunit localization
PIS48267.1	27.89866238	29.15198543	27.46985081	28.58028752	29.29852048	29.1148451	0.270981798	0.863	0.824	PIS48267.1	orf19.2996	Ortholog of S. cerevisiae : ECM30, C. glabrata CBS138 : CAGL0M00924g, C. dubliniensis CD36 : Cd36_02850, C. parapsilosis CDC317 : CPAR2_110240 and C. auris B8441 : B9J08_004952
PIS56937.1	26.57589395	25.89531372	27.08960129	27.27681859	27.29103608	27.46454234	0.052623187	0.459	0.824	PIS56937.1	orf19.849	Ortholog(s) have enzyme activator activity, role in protein N-linked glycosylation, protein O-linked glycosylation and Golgi apparatus localization
PIS58898.1	26.21553388	24.69847668	26.28008992	26.8334328	26.57719567	26.24979356	0.370654665	0.899	0.822	PIS58898.1	SMC5	Protein similar to S. cerevisiae Smc5p, which is involved in DNA repair; transposon mutation affects filamentous growth

PIS51492.1	25.61970229	27.70266814	25.73387067	25.10778467	28.14962283	28.25935447	0.494742486	0.924	0.82	PIS51492.1	orf19.5828	Ortholog(s) have pre-mRNA branch point binding activity and role in mRNA branch site recognition, mRNA cis splicing, via spliceosome, spliceosomal complex assembly
PIS48528.1	27.21062285	26.93186173	26.69137973	27.83927807	27.89629143	27.55185032	0.349071728	0.893	0.818	PIS48528.1	orf19.6862	Hap43-induced gene; mutation confers hypersensitivity to 5-fluorocytosine (5-FC), 5-fluorouracil (5-FU), and tubercidin (7-deazaadenosine)
PIS55829.1	26.84775064	26.6529089	27.40621239	28.03931745	27.58201673	27.73140339	0.110356995	0.687	0.815	PIS55829.1	MNN2	Alpha-1,2-mannosyltransferase; similar to S. cerevisiae Mnn2; role in cell wall integrity, temperature sensitivity; iron utilization in low iron; Tn mutation affects filamentous growth; filament induced; increased chitin exposes beta-glucan
PIS51941.1	27.37948298	27.88560984	26.72158681	27.82345151	27.99543065	28.60049626	0.118143417	0.705	0.811	PIS51941.1	YWP1	Secreted yeast-cell wall protein; controls exposure of cell wall beta-glucan to host immune system; involved in adhesion and biofilm formation; growth phase, phosphate, Ssk1/Ssn6/Efg1/Efh1/Hap43 regulated
PIS58230.1	24.65370979	22.43626552	23.66405478	24.13641596	24.5231752	24.52387485	0.381713121	0.902	0.81	PIS58230.1	DBP7	Putative ATP-dependent DEAD-box RNA helicase; Hap43-induced; rat catheter biofilm induced
PIS52196.1	28.342084	27.99298186	28.48028262	29.89161328	28.68939637	28.66202519	0.036862099	0.36	0.809	PIS52196.1	orf19.1355	Putative protein of unknown function; stationary phase enriched protein
PIS50580.1	28.72834829	28.95674091	28.38470188	29.39460812	29.50925882	29.59348711	0.071574933	0.56	0.809	PIS50580.1	PCT1	Putative choline-phosphate cytidylyl transferase, antigenic during human oral infection
PIS49808.1	27.54110084	27.85286782	26.27732411	27.53347112	28.40391314	28.15743651	0.121439164	0.712	0.808	PIS49808.1	orf19.549	Ortholog(s) have structural constituent of ribosome activity and mitochondrial large ribosomal subunit localization
PIS55490.1	30.46549068	29.03371337	30.36064282	30.95546915	30.7199365	30.60422615	0.159000398	0.774	0.807	PIS55490.1	orf19.1152	Protein of unknown function; induced in core stress response; Gcn2 and Gcn4 regulated; flow model biofilm induced; Spider biofilm induced
PIS58684.1	26.38236111	25.2975232	24.84323302	27.11381361	26.15862966	25.66882584	0.449211784	0.916	0.806	PIS58684.1	ANP1	Putative mannosyltransferase of Golgi; member of Mnn9p family; similar to S. cerevisiae Anp1p; fungal-specific (no human or murine homolog)

PIS52038.1	28.45951999	28.31013402	27.64268955	28.98511507	28.79262299	29.05166823	0.033643346	0.339	0.806	PIS52038.1	ENT3	Putative epsin; induced during the mating process; transcript is upregulated in an RHE model of oral candidiasis
PIS58579.1	23.23966509	26.61356708	24.10927275	25.00543906	25.39796795	25.97463995	0.289070117	0.871	0.805	PIS58579.1	ESS1	Prolyl isomerase (parvulin class); essential; involved in yeast-hyphal switching, Cph1p pathway; has inflexible linker between WW and isomerase domains, unlike human homolog; functional homolog of S. cerevisiae Ess1p
PIS49782.1	25.83515145	17.40811773	25.12596176	25.33536266	23.90687489	21.54164632	0.658066252	0.942	0.805	PIS49782.1	RIT1	Putative initiator tRNA methionine ribosyltransferase; fungal-specific (no human or murine homolog)
PIS58499.1	26.47723981	26.99019261	25.67234683	25.89509743	27.82892169	27.82028794	0.228718618	0.839	0.802	PIS58499.1	TRM2	Putative tRNA methyltransferase; repressed by prostaglandins; Spider biofilm induced
PIS52124.1	22.80653803	23.93157165	23.31968295	24.13029839	24.32928978	23.99115057	0.6449948	0.941	0.798	PIS52124.1	CAK1	Monomeric CDK-activating kinase; functional homolog of S. cerevisiae Cak1p; phosphorylates cyclin-free human CDK2; lacks glycine loop motif; conserved lysine (K36) not required for activity; possibly essential gene (by UAU1 method)
PIS49576.1	27.12038483	27.75585328	26.30866924	27.90490206	27.89514536	27.77961391	0.071721454	0.56	0.798	PIS49576.1	orf19.4504	Has domain(s) with predicted oxidoreductase activity
PIS51363.1	26.26647819	26.66459052	25.5800627	27.04968545	27.2743186	26.57687854	0.128695544	0.726	0.797	PIS51363.1	orf19.7100	Putative vacuolar transporter of large neutral amino acids; possibly transcriptionally regulated upon hyphal formation; not the true Avt1 ortholog of S. cerevisiae
PIS54747.1	24.92057996	26.57863779	24.34344211	26.42543036	25.92264761	25.88220374	0.217216465	0.831	0.796	PIS54747.1	orf19.446.2	Protein with a NADH-ubiquinone oxidoreductase B18 subunit domain; gene has intron
PIS52022.1	27.57100882	27.41482134	27.35684379	28.68537844	28.23145581	27.80359357	0.443967558	0.915	0.793	PIS52022.1	PEX6	Ortholog(s) have ATPase activity, role in protein import into peroxisome matrix, receptor recycling, protein unfolding and cytosol, peroxisome localization
PIS58957.1	27.56855394	27.21035015	27.40363838	28.29381351	28.06459792	28.19729899	0.008216352	0.101	0.791	PIS58957.1	CDC24	GDP-GTP exchange factor for Cdc42p; phosphorylated; required for maintenance of hyphal growth; misexpression blocks hyphal growth and causes avirulence in a mouse model of systemic infection; antigenic during human oral infection

PIS49504.1	22.9833677	24.85822797	22.75424563	24.0633025	23.16138609	25.73810003	0.240262943	0.846	0.789	PIS49504.1	orf19.5680	Protein with predicted hydrolase domains; similar to a universal stress protein family protein; possibly essential, disruptants not obtained by UAU1 method; Spider biofilm repressed
PIS56609.1	25.1377607	25.22607607	26.50624506	27.05869305	26.28946032	25.88394037	0.625062687	0.939	0.787	PIS56609.1	orf19.1876	Ortholog(s) have mRNA binding activity and role in mRNA splice site recognition, mRNA splicing, via spliceosome, positive regulation of mRNA splicing, via spliceosome
PIS49792.1	24.70706456	24.17684985	24.67129283	24.96123076	25.39810747	25.55592041	0.328896932	0.886	0.787	PIS49792.1	TAC1	Zn(2)-Cys(6) transcriptional activator of drug-responsive genes (CDR1 and CDR2); binds DRE element; gene in zinc cluster region near MTL locus; resequencing indicates that TAC1 spans orf19.3188 and orf19.3189; Spider biofilm induced
PIS48234.1	26.05117288	24.94236102	25.68619298	26.87901269	25.74974852	26.40751242	0.248621319	0.851	0.786	PIS48234.1	orf19.7159	Putative protein of unknown function; Hap43p-repressed gene; ortholog of S. cerevisiae YMR185W
PIS48608.1	23.84970989	23.11039351	23.99499541	25.20878701	24.03649853	24.06026971	0.079712579	0.594	0.783	PIS48608.1	orf19.2301	Ortholog(s) have role in proteasome regulatory particle assembly and cytoplasm, cytosol, nucleus localization
PIS51279.1	25.64640447	26.17395973	26.10104157	26.87177586	26.72094465	26.67548477	0.077465257	0.585	0.782	PIS51279.1	orf19.5381	Ortholog(s) have mRNA binding activity and role in negative regulation of translation, nuclear-transcribed mRNA catabolic process, deadenylation-dependent decay, protein localization
PIS56962.1	29.10198128	28.63174633	29.10693133	29.73348311	29.69619315	29.75212826	0.058374042	0.493	0.78	PIS56962.1	LMO1	Protein involved together with Dck1p and Rac1p in invasive filamentous growth and cell wall integrity; acts upstream of MAP kinase Cek1p; has similarity to human ELMO1
PIS55017.1	28.30256727	27.60651246	27.78574997	28.65029142	28.75551602	28.61657575	0.175168122	0.793	0.776	PIS55017.1	orf19.1185	Ortholog(s) have ubiquitin protein ligase activity, ubiquitin-protein transferase activity
PIS48721.1	27.98550044	28.51771329	27.36222385	28.11566958	29.0423608	29.0353773	0.185694203	0.804	0.776	PIS48721.1	orf19.7041	Putative pre-tRNA processing protein; heterozygous null mutant exhibits hypersensitivity to parnafungin and cordycepin in the C. albicans fitness test
PIS51710.1	26.57089204	25.33281387	26.47745686	26.47503216	27.2351223	26.99109127	0.32765281	0.886	0.773	PIS51710.1	PEP8	Protein similar to S. cerevisiae Pep8p, which is involved in retrograde transport; transposon mutation affects filamentous growth

PIS58249.1	28.75334498	28.37165203	26.76768305	28.20612456	29.0336272	28.96534632	0.127702376	0.724	0.771	PIS58249.1	GDH3	NADP-glutamate dehydrogenase; Nrg1, Plc1 regulated; hypha, hypoxia, Efg1-repressed; Rim101-induced at pH 8; GlcNAc, ciclopirox, ketoconazole induced; exp and stationary phase protein; Spider biofilm repressed; rat catheter biofilm induced
PIS58649.1	25.97767799	26.5988117	26.5219578	27.18321249	27.07076076	27.15342232	0.062611204	0.516	0.77	PIS58649.1	orf19.1604	Zinc cluster DNA-binding transcription factor; positive regulator of filamentous growth; Spider biofilm induced
PIS58282.1	27.86494063	26.53519083	27.67790992	27.97123488	28.2140186	28.19594714	0.105328574	0.675	0.768	PIS58282.1	EHT1	Putative acyl-coenzymeA:ethanol O-acyltransferase; regulated by Sef1, Sfu1, and Hap43; induced by alpha pheromone in SpiderM medium; Spider biofilm induced; promoter bound by Ndt80
PIS52281.1	31.22919753	30.91830036	31.38260759	32.00838143	31.8965455	31.91953871	0.374430143	0.9	0.765	PIS52281.1	orf19.3813	Ortholog of C. dubliniensis CD36 : Cd36_44340, C. parapsilosis CDC317 : CPAR2_302240, Candida tenuis NRRL Y-1498 : CANTEDRAFT_105331 and Debaryomyces hansenii CBS767 : DEHA2E03454g
PIS52153.1	27.754855	27.62255769	27.18262956	27.96264616	28.40743799	28.48203582	0.024655896	0.273	0.764	PIS52153.1	orf19.4005	Putative dephospho-CoA kinase; protein likely to be essential for growth, based on an insertional mutagenesis strategy
PIS52333.1	25.7646062	24.76566576	25.07055456	26.31388606	26.22733485	25.33839007	0.055744837	0.477	0.76	PIS52333.1	RVS162	Protein containing a BAR domain, which is found in proteins involved in membrane curvature; null mutant does not display the endocytic, hyphal growth, virulence, or cell wall defects exhibited by mutants in related genes RVS161 and RVS167
PIS55628.1	24.98313193	25.84519578	24.99952802	25.67827347	26.20148893	26.21891565	0.202254871	0.819	0.757	PIS55628.1	CNB1	Regulatory subunit of calcineurin B (Ca[2+]-calmodulin-regulated S/T protein phosphatase); required for wild-type resistance to fluconazole or to SDS; micafungin is fungicidal to null mutant
PIS49559.1	30.56726269	30.29089264	30.65925441	31.37252247	31.17208291	31.24259425	0.075914263	0.578	0.757	PIS49559.1	orf19.2244	Similar to oxidoreductases and to S. cerevisiae Yjr096wp; Sfu1 repressed; induced by benomyl treatment, Ssr1; Hap43-repressed; flow model biofilm repressed
PIS50495.1	28.33550464	27.95094027	27.8263652	29.45380485	28.38607844	28.53173194	0.170015427	0.787	0.753	PIS50495.1	HAS1	Functional homolog of S. cerevisiae Has1p, which is a nucleolar protein of the DEAD-box ATP-dependent RNA helicase family that is involved in biogenesis of the ribosome, particularly the small (40S) subunit; caspofungin-downregulated
PIS51420.1	27.71440785	28.17572486	27.22053501	28.40240099	28.5265306	28.44190435	0.075440269	0.576	0.753	PIS51420.1	SEC5	Predicted exocyst component; ortholog of S. cerevisiae Sec5p; merged with orf19.75 in Assembly 21

PIS50597.1	24.27983855	24.15921041	24.36505513	25.37889165	24.57931406	25.09669433	0.131280734	0.731	0.75	PIS50597.1	MLH1	Putative mismatch repair protein; cell-cycle regulated periodic mRNA expression
PIS51714.1	27.3570266	27.84948469	27.41409467	28.48933719	28.26917535	28.11165719	0.09582163	0.649	0.75	PIS51714.1	orf19.2500	Mitochondrial protein involved in assembly of NADH ubiquinone oxidoreductase Complex I; required for biofilm formation, respiration and virulence
PIS51028.1	24.08157184	24.82263004	23.69578776	25.16873641	24.95654499	24.72086531	0.030375708	0.315	0.749	PIS51028.1	orf19.2939	Ortholog(s) have role in mitochondrial cytochrome c oxidase assembly, regulation of proline metabolic process and mitochondrial inner membrane, mitochondrion localization
PIS49786.1	24.44744428	24.2980845	24.86859075	25.59606622	25.15217341	25.10811319	0.063375289	0.52	0.747	PIS49786.1	orf19.3178	Ortholog(s) have RNA binding activity, role in mRNA 5'-splice site recognition, mRNA splicing, via spliceosome and U2-type prespliceosome localization
PIS49464.1	27.43346068	26.90627577	27.21094118	28.09659826	27.85974012	27.83451907	0.114722503	0.698	0.747	PIS49464.1	orf19.757	Ortholog of S. cerevisiae : YPR089W, C. glabrata CBS138 : CAGL0K08008g, C. dubliniensis CD36 : Cd36_04600, C. parapsilosis CDC317 : CPAR2_105630 and C. auris B8441 : B9J08_004487
PIS52375.1	26.3669254	24.64446689	25.82494715	25.64994421	26.67862792	26.74783379	0.176568029	0.794	0.747	PIS52375.1	RRN11	Putative RNA polymerase I subunit; rat catheter biofilm induced; Spider biofilm induced
PIS49820.1	30.08709586	30.59589502	30.16901494	30.89146428	31.07000493	31.12661311	0.028146397	0.298	0.745	PIS49820.1	VTC3	Putative polyphosphate synthetase; downregulated in core caspofungin response; Hog1p-induced; transcription is opaque-specific; fungal-specific (no human or murine homolog)
PIS58338.1	20.47069222	22.25916538	17.88153616	20.75316072	20.68093672	21.40810372	0.595952475	0.936	0.744	PIS58338.1	orf19.7085	Protein of unknown function; induced in core stress response; induced by cadmium stress via Hog1; oxidative stress-induced via Cap1; induced by Mnl1 under weak acid stress; macrophage-repressed; rat catheter and Spider biofilm induced
PIS55699.1	26.73302666	24.26525504	26.63134785	26.79072043	26.31256812	26.74785051	0.214201305	0.829	0.741	PIS55699.1	orf19.2890	Has domain(s) with predicted pyrimidine nucleotide-sugar transmembrane transporter activity, role in pyrimidine nucleotide-sugar transmembrane transport and Golgi membrane, membrane localization
PIS56613.1	26.38569367	27.29658185	25.18890499	26.7136002	26.72008069	27.65696307	0.241424148	0.847	0.74	PIS56613.1	VMA10	Subunit G of the V1 peripheral membrane domain of the vacuolar H+-ATPase (V-ATPase); involved in vacuolar acidification; rat catheter biofilm repressed

PIS55794.1	27.90271718	26.98041481	27.93691301	28.4673948	28.21600754	28.35299469	0.123763348	0.717	0.739	PIS55794.1 BRO1	Class E vacuolar protein sorting factor; role in transport from multivesicular body to vacuole; not involved in Rim101 pathway; macrophage and pseudohyphal-repressed; flow model biofilm induced
PIS51510.1	23.29136283	23.47157299	23.73927142	24.35028989	24.16863002	24.19854019	0.423095312	0.911	0.738	PIS51510.1 orf19.5486	Putative endoribonuclease; heterozygous null mutant exhibits hypersensitivity to parnafungin and cordycepin in the C. albicans fitness test
PIS55714.1	26.72380171	27.44132237	27.08032032	27.70509483	28.09716568	27.65153789	0.182453729	0.801	0.736	PIS55714.1 RTT101	Putative cullin subunit of E3 ubiquitin ligase complex, involved in response to DNA damage; induced by alpha pheromone in SpiderM medium
PIS56918.1	26.4775818	28.28067463	26.18906022	26.93995659	28.09667175	28.11513715	0.395186309	0.905	0.735	PIS56918.1 PHO84	High-affinity phosphate transporter; transcript regulated by white-opaque switch; Hog1, ciclopirox olamine or alkaline induced; caspofungin, stress repressed; required for normal TORC1 function
PIS55584.1	28.58182867	28.84223423	28.73526953	29.29633197	29.48424818	29.58064029	0.060178312	0.503	0.734	PIS55584.1 orf19.3352	Ortholog of C. dubliniensis CD36 : Cd36_01490, C. parapsilosis CDC317 : CPAR2_109390, C. auris B8441 : B9J08_001688 and Candida tenuis NRRL Y-1498 : CANTEDRAFT_97929
PIS50349.1	24.7915781	25.44542087	26.0093992	24.96263201	26.71401613	26.76505249	0.259680289	0.857	0.732	PIS50349.1 orf19.1646	Ortholog(s) have rRNA primary transcript binding activity
PIS54602.1	24.52658331	23.88193483	24.20977642	24.56679956	25.15305678	25.08916285	0.451180319	0.916	0.73	PIS54602.1 orf19.3438	Ortholog(s) have protein-folding chaperone binding activity and role in protein folding in endoplasmic reticulum, response to unfolded protein, ubiquitin-dependent ERAD pathway
PIS58312.1	27.24100847	26.63618661	27.85615997	28.20713901	27.92463668	27.77994362	0.08982957	0.63	0.726	PIS58312.1 orf19.3881	Ortholog of C. dubliniensis CD36 : Cd36_31790, C. parapsilosis CDC317 : CPAR2_204900, C. auris B8441 : B9J08_000806 and Candida tenuis NRRL Y-1498 : CANTEDRAFT_136864
PIS56831.1	28.32745282	28.80628271	28.05452343	29.14946771	29.12366492	29.09000137	0.114012267	0.696	0.725	PIS56831.1 MSN5	Predicted karyopherin involved in nuclear import and export of proteins; flow model biofilm induced; Spider biofilm induced
PIS52045.1	23.84816452	25.73273072	23.25969367	23.88487536	24.86215786	26.26945613	0.334945876	0.888	0.725	PIS52045.1 orf19.1544	Putative cis-golgi localized protein involved in ER to Golgi transport; Spider biofilm repressed

PIS58988.1	23.53909141	24.42213515	23.8237881	23.58956331	24.76045246	25.60408147	0.146758079	0.757	0.723	PIS58988.1	GAP6	Broad-specificity amino acid permease; Plc1, Gcn4 regulated; rat catheter biofilm induced
PIS51911.1	32.78403417	33.44277717	32.63269831	33.540318	33.59238161	33.89372374	0.142322503	0.75	0.722	PIS51911.1	CAR2	Ornithine aminotransferase; arginine metabolism; alkaline induced; mutant sensitivite to toxic ergosterol analog, to amphotericin B; exponential and stationary phase yeast; flow model biofilm induced; rat catheter, Spider biofilm repressed
PIS49845.1	30.14815726	30.28798911	30.23659649	30.94663099	30.69541816	31.19789326	0.061037545	0.508	0.722	PIS49845.1	orf19.1272	Protein of unknown function; may play a role in regulation of cell size; rat catheter biofilm repressed
PIS52346.1	26.59005456	27.77549946	26.13001204	27.46418889	27.83272595	27.35790856	0.36644479	0.898	0.72	PIS52346.1	SPA2	Protein involved in cell polarity, Spitzenkorper formation; required for mouse virulence; localizes to hyphal tip; cell-cycle regulated localization in yeast-form cells; functional domains conserved with S. cerevisiae ; Hap43p-induced gene
PIS56856.1	26.14369556	26.72939874	25.9848398	26.64942107	27.36189449	27.00373475	0.147091309	0.757	0.719	PIS56856.1	orf19.4610	Predicted metallocarboxypeptidase; role in proteolysis; rat catheter biofilm repressed
PIS51318.1	27.12406466	27.9917651	26.66751841	27.39253411	28.2646455	28.279241	0.322258325	0.884	0.718	PIS51318.1	FUN31	Putative PAS kinase involved in cell wall damage response; similar to S. cerevisiae Psk1p, a putative serine/threonine protein kinase; induced by Mnl1 under weak acid stress; rat catheter and Spider biofilm induced
PIS50317.1	27.23842583	26.7602498	26.57724511	27.99068823	27.80012182	26.93642785	0.151604268	0.764	0.717	PIS50317.1	NMD5	Karyopherin; carrier protein involved in nuclear import of proteins; repressed in core stress response; Hap43-induced; Spider biofilm induced
PIS58544.1	29.26632685	29.67479471	29.19496909	29.93687263	30.16010805	30.18641308	0.039075017	0.373	0.716	PIS58544.1	MRP20	Component of mitochondrial ribosome; decreased expression in hyphae compared to yeast-form cells
PIS51162.1	25.50874259	22.7970743	26.04457518	26.16700307	25.55101409	24.77033855	0.44572459	0.915	0.713	PIS51162.1	orf19.2131	Ortholog(s) have ubiquitin protein ligase activity, ubiquitin-protein transferase activity
PIS50605.1	26.38896431	28.10075507	23.45613369	25.46430484	26.97639238	27.64235543	0.451976216	0.917	0.712	PIS50605.1	orf19.913.2	Putative subunit 6 of the ubiquinol cytochrome-c reductase complex, a component of the mitochondrial inner membrane electron transport chain; null mutant is viable

PIS51451.1	27.21716982	26.56766383	27.0330071	27.79284849	27.75548743	27.40219203	0.113036407	0.694	0.711	PIS51451.1	TAZ1	Putative lyso-phosphatidylcholine acyltransferase, required for normal phospholipid content of mitochondrial membranes; rat catheter biofilm induced
PIS50533.1	27.95003182	26.65375977	27.09010908	27.8148189	27.93503786	28.05814649	0.164173353	0.78	0.705	PIS50533.1	orf19.3920	Ortholog(s) have protein disulfide isomerase activity, protein-disulfide reductase (glutathione) activity, protein-disulfide reductase activity and role in protein folding
PIS51846.1	25.98023996	25.96196224	25.28449188	27.13338253	26.04215201	26.16750654	0.235224765	0.843	0.705	PIS51846.1	PHO23	Ortholog(s) have methylated histone binding activity
PIS49854.1	23.849226	24.74197029	24.39684265	25.26136356	24.89253174	24.94687986	0.129960921	0.729	0.704	PIS49854.1	orf19.721	Ortholog(s) have polynucleotide 5'-hydroxyl-kinase activity
PIS58162.1	26.37464265	26.94277352	24.51567172	25.75589504	27.18543522	26.99997392	0.284237845	0.869	0.703	PIS58162.1	ARP8	Putative mitochondrial, half-size MDR-subfamily ABC transporter
PIS55564.1	27.9105409	28.20064903	28.17625449	28.62664322	28.84862943	28.91962239	0.164022988	0.78	0.702	PIS55564.1	BET2	Putative Type II geranylgeranyltransferase beta subunit; transcript regulated by Mig1
PIS58562.1	29.88296307	29.72784856	30.10306214	30.65923898	30.43405981	30.7254604	0.05279506	0.46	0.702	PIS58562.1	orf19.7077	Putative ferric reductase; induced by Mac1 under copper starvation; Plc1-regulated; Rim101-repressed
PIS56681.1	30.27031401	30.1810083	29.73187879	30.94153827	30.80105139	30.54294008	0.431726397	0.913	0.701	PIS56681.1	CSM3	Putative subunit of a replication fork-pausing checkpoint complex
PIS50431.1	26.91875115	26.38588282	25.19671167	26.22527319	27.04596088	27.33331671	0.253875374	0.854	0.701	PIS50431.1	orf19.2675	Ortholog(s) have mRNA binding activity, role in mRNA splicing, via spliceosome, spliceosomal complex assembly and U2 snRNP, U2-type prespliceosome localization
PIS48747.1	24.29220526	25.80124897	24.25282403	24.42475566	25.91890956	26.10680726	0.377391206	0.901	0.701	PIS48747.1	SSK1	Response regulator of two-component system; role in oxidative stress response, cell wall biosynthesis, virulence, hyphal growth on solid media; expressed in hyphae and yeast; peroxisomal targeting sequence (PTS1); Spider biofilm induced

PIS51416.1	28.77989256	28.43928487	28.39711386	29.27143575	29.14155207	29.30228267	0.05273764	0.459	0.7	PIS51416.1	FET33	Putative multicopper ferro-O2-oxidoreductase; repressed in core caspofungin response; fails to complement S. cerevisiae fet3 iron-related phenotypes; rat catheter and Spider biofilm induced
PIS52441.1	25.15725343	25.22013867	25.70280911	25.69331134	26.38363003	26.09843537	0.095479538	0.647	0.698	PIS52441.1	orf19.7361	Ortholog(s) have tRNA-intron endonuclease activity, role in tRNA-type intron splice site recognition and cleavage and mitochondrial outer membrane, tRNA-intron endonuclease complex localization
PIS49710.1	30.27967582	30.61371725	29.74123173	30.74014655	31.12273362	30.85826627	0.403856034	0.907	0.696	PIS49710.1	LAT1	Putative dihydrolipoamide acetyltransferase component (E2) of pyruvate dehydrogenase complex; sumoylation target; Spider biofilm repressed
PIS58276.1	25.130747	25.68076117	23.39987347	25.31906457	23.75448552	27.22701488	0.454014948	0.917	0.696	PIS58276.1	orf19.4659	Ortholog(s) have RNA binding activity, role in mRNA splicing, via spliceosome and U2 snRNP, U2-type prespliceosome localization
PIS51328.1	30.96359078	30.82407352	31.11182804	31.85735238	31.6447511	31.48516019	0.065568522	0.531	0.696	PIS51328.1	orf19.6828.1	Ortholog(s) have protein transmembrane transporter activity, protein-transporting ATPase activity, structural molecule activity
PIS51432.1	24.72043751	23.09007495	27.42113029	24.45594881	28.06652868	24.79321198	0.539761211	0.93	0.695	PIS51432.1	orf19.2857	Ortholog(s) have DNA helicase activity, DNA translocase activity, RNA polymerase II general transcription initiation factor activity
PIS58061.1	26.69185974	28.56391708	25.36485031	26.51619696	28.75036723	27.43962507	0.429938323	0.912	0.695	PIS58061.1	orf19.3453	Ortholog(s) have GTPase activator activity, role in establishment or maintenance of cell polarity, positive regulation of GTPase activity and cytoplasmic vesicle membrane, plasma membrane localization
PIS49541.1	27.2122267	25.62506934	26.46846327	27.83805493	27.11583033	26.43762033	0.415244331	0.909	0.695	PIS49541.1	orf19.698	Integral ER membrane protein; predicted role in maintenance of ER zinc homeostasis; Spider biofilm induced
PIS52322.1	30.45855077	30.87375547	29.4501167	30.50535918	31.22348756	31.12116043	0.183019318	0.801	0.689	PIS52322.1	SNT1	Ortholog of S. cerevisiae Snt1; an NAD-independent histone deacetylase; heterozygous transposon mutation affects filamentous growth; null mutants are viable
PIS48346.1	26.87245005	28.42099925	26.01102847	27.55369674	27.94053202	27.87574384	0.233396856	0.842	0.688	PIS48346.1	orf19.1246	Putative eisosome component role in proper eisosome assembly; upregulated in cyr1 null mutant

PIS48779.1	27.1462281	25.00894905	27.61739922	27.31495694	27.31497244	27.20667041	0.296951036	0.875	0.688	PIS48779.1	orf19.516	S. cerevisiae ortholog Rft1p has role in glycolipid translocation, protein N-linked glycosylation and localizes to endoplasmic reticulum membrane; regulated by Sef1p-, Sfu1p-, and Hap43p
PIS51964.1	27.12450244	26.97264832	27.62601723	28.16583185	27.90633651	27.71522194	0.405794167	0.907	0.688	PIS51964.1	orf19.6741	Putative plasma membrane protein; predicted role in cell wall integrity; regulated by Nrg1, Tup1; induced during chlamydospore formation in both C. albicans and C. dubliniensis
PIS49777.1	29.53481533	30.66528808	30.06390995	30.73625152	30.3353443	31.25302335	0.125105348	0.719	0.687	PIS49777.1	HPD1	3-hydroxypropionate dehydrogenase; involved in degradation of toxic propionyl-CoA; rat catheter and Spider biofilm induced
PIS51290.1	26.83326237	27.61218495	27.04435605	28.72110965	27.50274745	27.32463646	0.257770759	0.856	0.686	PIS51290.1	orf19.5395	Ortholog(s) have role in early endosome to late endosome transport, regulation of protein-containing complex assembly, vacuolar acidification and RAVE complex, cytoplasm localization
PIS55737.1	28.03273817	29.01643122	28.29131956	29.010968	29.1296857	29.2565718	0.072417123	0.563	0.686	PIS55737.1	RPT4	26S proteasome regulatory subunit; regulated by Gcn2p and Gcn4p; protein level decreases in stationary phase cultures
PIS52288.1	25.4786661	26.40407863	26.31036709	26.76158811	26.61678919	26.86220345	0.336300935	0.889	0.682	PIS52288.1	EMC9	Ortholog of S. cerevisiae Nnf2; possible role in chromosome segregation; mutants are viable, induced during the mating process
PIS51110.1	28.13929685	28.85735463	28.08368483	29.14852572	29.01821219	28.95512341	0.459354059	0.918	0.681	PIS51110.1	MKC1	MAP kinase; role in biofilm formation, contact-induced invasive filamentation, systemic virulence in mouse, cell wall structure/maintenance, caspofungin response; phosphorylated on surface contact, membrane perturbation, or cell wall stress
PIS51096.1	24.56509915	25.92420294	24.8768574	25.95296979	25.82630985	25.6243914	0.338451983	0.889	0.679	PIS51096.1	ALK2	N-Alkane inducible cytochrome P450
PIS51187.1	25.56263583	26.82139241	23.74456295	26.17977554	25.49248159	26.49249869	0.43596118	0.914	0.679	PIS51187.1	CHT3	Major chitinase; secreted; functional homolog of S. cerevisiae Cts1p; 4 N-glycosylation motifs; possible O-mannosylation; putative signal peptide; hyphal-repressed; farnesol upregulated in biofilm; regulated by Efg1p, Cyr1p, Ras1p
PIS55454.1	23.48582257	21.86415181	24.86945916	24.18744429	23.77319539	24.29404254	0.613272439	0.938	0.678	PIS55454.1	BTS1	Putative geranylgeranyl diphosphate synthase; repressed by benomyl treatment; Spider biofilm induced

PIS58067.1	26.91475357	24.71991299	27.30367151	26.99626404	26.88854862	27.08559593	0.352151725	0.894	0.677	PIS58067.1	orf19.688	Mitochondrial ribosomal protein of the small subunit; S. cerevisiae ortholog is essential for viability; Spider biofilm repressed
PIS58687.1	23.82159318	24.75736481	23.11685468	24.20021728	24.66596763	24.85853873	0.238572427	0.845	0.676	PIS58687.1	orf19.2112	snRNP U5 splicing factor component; involved in positioning the 3' splice site during the 2nd catalytic step of splicing; Spider biofilm induced
PIS58229.1	25.47229909	25.19757383	25.09642591	25.56224395	26.26126455	25.97098121	0.342632045	0.891	0.676	PIS58229.1	orf19.6901	Ortholog(s) have 5'-(N(7)-methyl 5'-triphosphoguanosine)-[mRNA] diphosphatase activity, RNA 7-methylguanosine cap binding, exoribonuclease activator activity, hydrolase activity and acting on acid anhydrides, more
PIS48313.1	31.47884347	31.54604216	30.88296275	31.67227226	32.2597717	32.00197402	0.446588087	0.916	0.675	PIS48313.1	CKA2	Catalytic alpha-subunit of protein kinase CK2; interaction with calcineurin pathway affects fluconazole sensitivity; synthetically lethal with CKA1; attenuated virulence in a mouse oropharyngeal candidiasis but not in a systemic mouse model
PIS52099.1	26.06340166	26.10367486	26.45025616	27.22255612	26.96642168	26.45105806	0.075076104	0.575	0.674	PIS52099.1	orf19.438	Has domain(s) with predicted membrane localization
PIS56801.1	24.86702021	27.30590138	25.28671548	26.63137807	26.26427816	26.58234586	0.331630355	0.887	0.673	PIS56801.1	orf19.7116	Protein of unknown function; transcript induced in RHE model of oral candidiasis; Spider biofilm repressed
PIS49714.1	24.55987148	24.22314243	26.61021689	26.37992506	24.45565815	26.57321666	0.373069691	0.899	0.672	PIS49714.1	orf19.1449	Protein of unknown function; induced in azole-resistant strain that overexpresses MDR1; protein present in exponential and stationary growth phase yeast cultures; Spider biofilm induced
PIS54958.1	26.16790653	25.15674707	27.00787307	26.98427387	26.46265897	26.90097132	0.351463486	0.893	0.672	PIS54958.1	orf19.7244	Putative fumarylacetoacetate hydrolase; induced by nitric oxide independent of Yhb1; regulated by Sef1, Sfu1, Hap43; flow model biofilm induced
PIS48465.1	24.95528677	24.64402712	24.82703454	25.51834495	25.57740468	25.34269906	0.224866965	0.836	0.671	PIS48465.1	orf19.6187	possible pseudouridine monophosphate glycosidase; overlaps orf19.6185 and orf19.618; has intron; Spider biofilm repressed
PIS58467.1	30.74173544	30.70546945	29.55144785	31.44330101	30.81428592	30.75214587	0.561600281	0.932	0.67	PIS58467.1	orf19.1144	Protein with SEL-1 like protein domain; early-stage flow model biofilm induced

PIS58732.1	26.95057344	27.59858523	24.60174859	26.98510635	27.21161372	26.96000023	0.346359103	0.892	0.669	PIS58732.1	CAS4	RAM cell wall integrity signaling network protein; cell separation, azole sensitivity; needed for hyphal growth; insertion mutation near 3' end of gene increases caspofungin sensitivity; pheromone/hyphal induced; flow biofilm repressed
PIS51489.1	26.72884961	27.04605095	27.13888934	27.40702437	27.94391155	27.56984484	0.190644885	0.809	0.669	PIS51489.1	orf19.5833	Protein involved in resistance to caspofungin and anidulafungin
PIS51983.1	25.83691128	25.56851231	25.53292362	26.74138439	26.23580795	25.96470199	0.662082516	0.942	0.668	PIS51983.1	DOT4	Protein similar to ubiquitin C-terminal hydrolase; localizes to cell surface of hyphal cells, but not yeast-form cells; repressed upon high-level peroxide; Hap43p-induced; rat catheter biofilm induced
PIS51101.1	27.72919268	28.3160922	28.08477939	28.43486831	28.7558357	28.94280742	0.203306138	0.82	0.668	PIS51101.1	orf19.1217	Ortholog(s) have ribosomal large subunit binding, ubiquitin protein ligase activity, ubiquitin-protein transferase activity
PIS52139.1	24.90465853	25.59237253	25.11879171	25.89503115	25.98974269	25.73004339	0.445697461	0.915	0.666	PIS52139.1	orf19.5921	Ortholog of C. dubliniensis CD36 : Cd36_84580, C. parapsilosis CDC317 : CPAR2_404290, C. auris B8441 : B9J08_003750 and Candida tenuis NRRL Y-1498 : CANTEDRAFT_115338
PIS55577.1	24.18171217	25.22203264	24.84834168	25.14676393	25.421161	25.67825947	0.215036842	0.829	0.665	PIS55577.1	ORC3	Protein similar to S. cerevisiae Orc3p, which is a component of the origin recognition complex involved in DNA replication; mutation confers hypersensitivity to toxic ergosterol analog; induced under hydroxyurea treatment
PIS48443.1	25.94165435	25.4723601	26.01628885	26.36808424	26.56487733	26.48959294	0.109102602	0.684	0.664	PIS48443.1	MAD2	Protein required for the spindle assembly checkpoint of the cell cycle; necessary for survival in host cell macrophages and for virulence in a mouse model of systemic candidiasis
PIS48294.1	27.1021078	27.11854221	27.62776933	28.47677784	27.82032168	27.53591008	0.142902047	0.751	0.662	PIS48294.1	RPP1	Putative ortholog of S. cerevisiae Rpp1; subunit of both RNase MRP and nuclear RNase P; rat catheter and Spider biofilm induced
PIS48665.1	26.99037028	27.4955997	26.36623034	27.1168858	27.86903472	27.84273683	0.231180048	0.84	0.659	PIS48665.1	orf19.504	Putative nuclear actin-related protein, component of INO80 complex, involved in chromatin remodeling
PIS58376.1	25.32852973	24.23465342	25.59724949	25.60199787	25.47567959	26.05672557	0.112415516	0.692	0.658	PIS58376.1	YTH1	Putative mRNA cleavage and polyadenylation specificity factor; transcription is regulated upon yeast-hyphal switch; decreased expression in hyphae compared to yeast-form cells; fluconazole or flucytosine induced

PIS56963.1	26.93124664	25.2603813	26.12439421	26.45725056	26.56810089	27.25875342	0.327253149	0.886	0.656	PIS56963.1	DRS1	Putative nucleolar DEAD-box protein; Hap43-induced; mutation confers hypersensitivity to 5-fluorouracil (5-FU), tubercidin (7-deazaadenosine); Tbf1-induced; repressed in core stress response
PIS48749.1	26.19009763	25.14027414	26.57498039	26.92887216	26.32410199	26.6188949	0.225792313	0.837	0.656	PIS48749.1	MODF	Has domain(s) with predicted ATP binding, ATPase activity
PIS51908.1	27.50044507	26.32256895	27.89038371	27.79778464	27.76071493	28.11683244	0.146051221	0.756	0.654	PIS51908.1	MET15	O-acetylhomoserine O-acetylserine sulfhydrylase; sulfur amino acid synthesis; immunogenic; Hog1, adherence-induced; brown color of mutant in Pb(2+) medium a visual selection; chlamydospore formation induced, F-12/CO2 biofilm induced
PIS52207.1	27.96947452	27.36456257	27.87219438	28.20915888	28.17320222	28.78294499	0.16337227	0.779	0.653	PIS52207.1	orf19.5406	Predicted plasma membrane associated protein phosphatase; required for normal filamentous growth; mRNA binds She3 and is localized to hyphal tips
PIS51272.1	32.88184782	32.60599408	33.04772398	33.43841093	33.21590584	33.8366246	0.114003416	0.696	0.652	PIS51272.1	FOX2	3-hydroxyacyl-CoA epimerase; fatty acid beta-oxidation; induced by phagocytosis; regulated by Mig1, by white-opaque switch, by DNA methylation; transcriptional activation by oleate requires Ctf1; rat catheter and Spider biofilm induced
PIS51138.1	26.48422578	24.50740615	27.06547384	27.13078498	26.91304941	25.96994653	0.433068159	0.913	0.652	PIS51138.1	orf19.5131	Ortholog of S. cerevisiae Gid7, a GID complex protein; involved in proteasome-dependent catabolite inactivation of fructose-1,6-bisphosphatase; Hap43-repressed gene
PIS51850.1	24.79398237	25.19239763	23.6575721	25.28089363	24.40097923	25.91820649	0.259302939	0.857	0.652	PIS51850.1	orf19.871	Ortholog(s) have GTPase activator activity, role in positive regulation of TORC1 signaling and FNIP-folliculin RagC/D GAP, cytoplasm, vacuolar membrane localization
PIS58065.1	27.12411136	26.12976153	27.30745123	28.20098724	27.23379773	27.07911824	0.374597425	0.9	0.651	PIS58065.1	orf19.3458	Ortholog(s) have role in late endosome to vacuole transport via multivesicular body sorting pathway and Vps55/Vps68 complex, fungal-type vacuole membrane localization
PIS55497.1	24.32083661	21.18814178	24.72359281	24.08674592	24.71524323	23.38392567	0.599926313	0.937	0.651	PIS55497.1	orf19.5049	Putative U3-containing 90S preribosome processome complex subunit; Hap43-induced gene; rat catheter and Spider biofilm induced; F-12/CO2 early biofilm induced
PIS56603.1	26.98005912	27.78014292	27.55211276	28.11143551	28.08207029	28.07163046	0.114865138	0.698	0.651	PIS56603.1	TAF4	Putative TFIID subunit; mutation confers hypersensitivity to toxic ergosterol analog

PIS51143.1	22.93611485	23.6621022	23.82635264	23.964567	24.17536638	24.23479913	0.200823043	0.818	0.65	PIS51143.1	FGR10	Putative asparaginase; lacks ortholog in S. cerevisiae; transposon mutation affects filamentous growth; Spider biofilm induced
PIS50452.1	23.81658873	26.65548358	26.77967628	26.52196864	26.09369876	26.58213182	0.400868265	0.906	0.649	PIS50452.1	orf19.874	Predicted mitochondrial i-AAA protease supercomplex; degrades misfolded mitochondrial proteins; Hap43-repressed gene
PIS51313.1	22.86481989	22.35965904	23.00946753	23.30100019	23.49346857	23.37500035	0.536961577	0.929	0.645	PIS51313.1	CAT8	Zn(II)2Cys6 transcription factor; similar to S. cerevisiae Cat8 but mutant phenotype suggests different target genes; mutant displays increased filamentous/invasive growth; flucytosine repressed; rat catheter biofilm induced
PIS51465.1	26.09714047	24.71995241	27.39628541	27.43353479	26.62192215	26.09083885	0.515106115	0.926	0.644	PIS51465.1	orf19.2001	Has domain(s) with predicted L-ascorbic acid binding, iron ion binding, oxidoreductase activity, acting on paired donors, with incorporation or reduction of molecular oxygen activity
PIS51805.1	25.79745862	25.67500387	24.29817911	25.85693792	25.83564912	26.00118899	0.218888523	0.832	0.641	PIS51805.1	orf19.3302	Putative type-1 protein phosphatase targeting subunit; transcript repressed by yeast-hyphal switch; transcript induced by Mnl1p under weak acid stress; flow model biofilm induced
PIS59031.1	26.20571606	25.16828197	26.21491698	25.89631562	26.88119081	26.73331142	0.278299772	0.866	0.641	PIS59031.1	orf19.4523	Ortholog(s) have 5-formyltetrahydrofolate cyclo-ligase activity and role in folic acid-containing compound biosynthetic process
PIS56799.1	29.56940766	29.32066026	29.86365551	30.30495523	29.94130166	30.43092676	0.26254263	0.859	0.641	PIS56799.1	orf19.7118	Ortholog(s) have nucleoside triphosphate adenylate kinase activity, role in nucleotide metabolic process and mitochondrial inner membrane, mitochondrial matrix localization
PIS48442.1	26.98546776	26.09295131	27.22959908	27.79220232	27.52635337	26.9090051	0.341678431	0.89	0.64	PIS48442.1	orf19.703	Ortholog(s) have 2',3'-cyclic-nucleotide 3'-phosphodiesterase activity and role in cyclic nucleotide metabolic process
PIS58401.1	24.13092183	23.66810003	24.24462287	24.21603568	24.93382119	24.80565011	0.309111146	0.879	0.637	PIS58401.1	orf19.7111	Putative mitochondrial outer membrane protein membrane fission effector; possibly an essential gene, disruptants not obtained by UAU1 method
PIS55512.1	26.08591452	26.50941446	26.37377372	26.77168335	27.18678922	26.91558004	0.176379258	0.794	0.635	PIS55512.1	orf19.4423	Putative glucosyltransferase; localized to the mitochondrial membrane

PIS58168.1	26.31291779	24.92235946	26.70183292	26.6196312	25.94455537	27.27857807	0.328329718	0.886	0.635	PIS58168.1	orf19.457	Ortholog(s) have K63-linked deubiquitinase activity, cysteine-type deubiquitinase activity
PIS55488.1	28.77657018	27.8583431	28.46251608	29.13155052	28.96255366	28.8987419	0.278306183	0.866	0.632	PIS55488.1	orf19.5728	Putative cytochrome P450; Spider biofilm induced
PIS55645.1	26.59484966	25.33593007	26.1784627	26.71568695	26.4750503	26.8113779	0.180561447	0.799	0.631	PIS55645.1	orf19.6732	Ortholog(s) have thiamine phosphate phosphatase activity and role in phosphate ion transport
PIS51741.1	26.74010983	25.90709078	27.82831375	27.67483711	27.64917357	27.04402652	0.178402169	0.796	0.631	PIS51741.1	RFC5	Putative heteropentameric replication factor C subunit; periodic mRNA expression, peak at cell-cycle G1/S phase
PIS55516.1	26.96307612	28.6148138	26.16250821	27.45200383	27.73289486	28.44835561	0.349229801	0.893	0.631	PIS55516.1	SKP1	Putative subunit D of kinetochore protein complex CBF3; regulated by Gcn4p; repressed in response to amino acid starvation (3-aminotriazole treatment)
PIS52462.1	27.82780375	26.94855472	27.84937423	28.1153302	28.17123566	28.22937081	0.316357527	0.882	0.63	PIS52462.1	orf19.2829	Ortholog(s) have role in protein transport
PIS58043.1	28.66857391	28.79712623	28.99030631	29.73047858	29.21246061	29.40386976	0.085259794	0.614	0.63	PIS58043.1	VPS17	Ortholog(s) have phosphatidylinositol-3-phosphate binding, protein carrier activity
PIS50614.1	23.08937231	25.69834192	23.49714031	25.52767442	24.94137511	23.69994958	0.557381947	0.932	0.628	PIS50614.1	orf19.6970	Ortholog of C. dubliniensis CD36 : Cd36_85310, C. parapsilosis CDC317 : CPAR2_807370, C. auris B8441 : B9J08_004442 and Candida tenuis NRRL Y-1498 : CANTEDRAFT_115544
PIS54582.1	22.95726688	24.61514048	24.65672907	24.41921953	24.68422621	25.00718246	0.239431841	0.846	0.627	PIS54582.1	MSS4	Phosphatidylinositol-4-phosphate 5-kinase; activity induced by phosphatidic acid (Pld1 product); macrophage/pseudohyphal-repressed; mRNA binds to She3, localized to yeast cell buds and hyphal tips; Hap43-induced; Spider biofilm induced
PIS49767.1	26.00192448	26.67705653	26.28190893	27.52432947	26.74544945	26.56804337	0.481178598	0.921	0.626	PIS49767.1	orf19.1359	Ortholog(s) have protein-containing complex binding activity, role in sporulation resulting in formation of a cellular spore, ubiquitin-dependent ERAD pathway and nucleus localization

PIS58308.1	22.96669245	23.99868831	24.50160055	24.23673067	24.98066703	24.12741924	0.3488092	0.893	0.626	PIS58308.1	orf19.3872	Protein of unknown function; oral infection induced; mutants have reduced capacity to damage oral epithelial cells; rat catheter biofilm repressed
PIS55751.1	32.1427801	32.21199223	32.42297098	32.97899723	33.00605632	32.66377345	0.086937962	0.62	0.624	PIS55751.1	CTR1	Copper transporter; transcribed in low copper; induced Mac1, Tye7, macrophage interaction, alkaline pH via Rim101; 17-beta-estradiol repressed; complements S. cerevisiae ctr1 ctr3 copper transport mutant; flow model/Spider biofilm induced
PIS52067.1	26.78335348	26.90378316	27.32747199	27.41320406	27.3836584	28.08403205	0.481434255	0.921	0.622	PIS52067.1	ANT1	Peroxisomal adenine nucleotide transporter; role in beta-oxidation of medium-chain fatty acid and peroxisome proliferation; rat catheter biofilm induced
PIS50309.1	28.63810408	27.77219954	28.47226711	29.22496646	28.81032907	28.71215571	0.15751086	0.772	0.622	PIS50309.1	orf19.7643	Ortholog(s) have 4-hydroxybenzoate octaprenyltransferase activity, role in ubiquinone biosynthetic process and mitochondrial inner membrane, mitochondrial membrane, mitochondrion localization
PIS52430.1	29.16592796	28.57401572	29.39631423	29.93747332	29.6803438	29.37790471	0.087898942	0.623	0.62	PIS52430.1	MCD4	Mannose-ethanolamine phosphotransferase, essential gene involved in GPI anchor biosynthesis
PIS52135.1	25.3422265	23.74227698	25.67929868	23.76965517	26.9808927	25.87242988	0.525106945	0.928	0.62	PIS52135.1	orf19.3758	Ortholog(s) have fungal-type vacuole membrane, membrane raft localization
PIS55033.1	27.19687108	26.95894263	25.70498389	26.89682445	27.22887866	27.59025651	0.326266274	0.885	0.618	PIS55033.1	CHT4	Chitinase; similar to S. cerevisiae sporulation-specific Cts2p; functionally complements A. gossypii cts2 mutant sporulation defect; homozygous null mutation causes no obvious defects; transcription decreases upon yeast-to-hyphal switch
PIS49488.1	27.4093502	26.64107911	27.93856322	28.66239909	27.57296349	27.60717546	0.246873463	0.85	0.618	PIS49488.1	orf19.7326	Ortholog(s) have protein-lysine N-methyltransferase activity and role in peptidyl-lysine dimethylation, peptidyl-lysine monomethylation
PIS48638.1	29.48107269	29.38448734	29.16214739	30.20540388	29.71438868	29.95824146	0.217318609	0.831	0.617	PIS48638.1	UTP4	Putative U3 snoRNA-associated protein; Hap43-induced; physically interacts with TAP-tagged Nop1; Spider biofilm induced
PIS58255.1	28.02735928	26.57522805	27.46406705	27.79258319	28.17100276	27.95087573	0.227141699	0.838	0.616	PIS58255.1	LRG1	GTPase activator (GAP) that negatively controls small GTPases Cdc42p and Ras1p, involved in signaling pathway that controls morphogenesis in response to environmental signals

PIS50427.1	24.14985112	24.67832112	24.65217177	25.50169788	25.04622062	24.78100239	0.205856096	0.822	0.616	PIS50427.1	orf19.3242	Putative TFIID and SAGA complex subunit; possibly an essential gene, disruptants not obtained by UAU1 method
PIS50494.1	27.62874363	26.61595742	27.97251022	28.25600366	27.50643429	28.3041156	0.416389566	0.91	0.616	PIS50494.1	orf19.3980	Ortholog(s) have ATPase activity, role in cytoplasmic translation, regulation of translation, rescue of stalled ribosome, ribosome disassembly and cytosolic ribosome localization
PIS52031.1	30.09912932	29.30238842	29.87019246	30.32020463	30.4413694	30.35466286	0.044942823	0.412	0.615	PIS52031.1	orf19.1564	Plasma membrane-localized protein of unknown function
PIS51655.1	23.64905713	23.29195284	23.42470145	23.89972872	23.86541554	24.44036659	0.259665108	0.857	0.613	PIS51655.1	orf19.2988	Predicted aminotransferase based on S. pombe ortholog SPBC660.12c; flow model biofilm induced
PIS49708.1	23.14674179	23.48414068	22.76431636	23.39229382	23.06992263	24.76621946	0.509937535	0.926	0.611	PIS49708.1	orf19.6532	Ortholog(s) have FAD transmembrane transporter activity, role in FAD transport and mitochondrion localization
PIS58605.1	25.72274996	25.88479414	25.70233737	25.73527462	26.13398694	27.26946444	0.292785078	0.873	0.61	PIS58605.1	MGM101	Putative mitochondrial genome maintenance protein; fungal-specific (no human or murine homolog); mutation confers hypersensitivity to tubercidin (7-deazaadenosine)
PIS49595.1	28.22413691	28.68143989	28.05509948	28.76988252	29.02391108	28.98777878	0.126909933	0.723	0.607	PIS49595.1	orf19.4537	Ortholog(s) have RNA polymerase II complex binding, RNA polymerase II complex recruiting activity, RNA polymerase II transcription regulatory region sequence-specific DNA binding activity
PIS58094.1	31.29041717	31.34506169	31.23768605	31.6217557	31.96352251	32.10810836	0.097877928	0.655	0.607	PIS58094.1	orf19.6143	Predicted long-chain-alcohol oxidase; Spider biofilm induced
PIS51333.1	26.48712981	25.893383	26.18518417	26.56186363	27.02779521	26.79522658	0.445232237	0.915	0.606	PIS51333.1	orf19.4726	Ortholog(s) have 1-phosphatidylinositol 4-kinase activator activity, calcium ion binding, enzyme activator activity
PIS54834.1	25.0703474	24.43807482	25.64660178	26.15330182	25.29158891	25.52658821	0.208667389	0.824	0.605	PIS54834.1	orf19.6315	Ortholog of C. dubliniensis CD36 : Cd36_30140, C. parapsilosis CDC317 : CPAR2_204040, C. auris B8441 : B9J08_001978 and Candida tenuis NRRL Y-1498 : CANTEDRAFT_114703

PIS51513.1	26.3414479	27.28490903	26.73061756	27.47920029	27.1858781	27.50495532	0.186738809	0.805	0.604	PIS51513.1	SER1	Putative 3-phosphoserine aminotransferase; predicted role in serine and glycine biosynthesis; protein present in exponential and stationary yeast growth phases; Spider biofilm repressed
PIS52436.1	24.43853422	24.67151538	24.3608464	24.41132257	25.36814539	25.50104361	0.174757709	0.792	0.603	PIS52436.1	orf19.4843	Putative iron/copper reductas; involved in iron homeostasis; rat catheter and Spider biofilm induced
PIS48575.1	27.57119807	28.37698107	26.68541523	27.57573058	28.41348076	28.45108608	0.372177923	0.899	0.602	PIS48575.1	orf19.5618	Ortholog(s) have SNARE binding, unfolded protein binding activity
PIS56885.1	27.63541221	26.79575258	27.80206264	28.43809163	27.8190432	27.77901885	0.283116248	0.869	0.601	PIS56885.1	orf19.5543	Has domain(s) with predicted role in intracellular protein transport, vesicle-mediated transport
PIS55629.1	29.64360324	28.95376874	29.97036496	30.22204527	29.9544297	30.19399521	0.165416001	0.782	0.601	PIS55629.1	orf19.6838	Putative protein of unknown function, transcript upregulated in clinical isolates from HIV+ patients with oral candidiasis; Spider biofilm induced
PIS58167.1	29.69246213	30.12515057	29.10828669	29.99861128	30.38216742	30.33574543	0.156478687	0.77	0.597	PIS58167.1	ADP1	Putative PDR-subfamily ABC transporter; similar to WHITE subfamily proteins; gene used for strain identification by multilocus sequence typing
PIS50573.1	27.87506491	27.60024023	27.14561146	27.82332446	28.0230715	28.56568513	0.185065774	0.803	0.597	PIS50573.1	orf19.3949	Ortholog(s) have ATP-dependent H3-H4 histone complex chaperone activity, ATP-dependent chromatin remodeler activity, ATPase, chromatin binding, histone binding activity
PIS55060.1	25.17415905	24.5081641	25.60383654	25.76321963	25.56094943	25.75212356	0.204813555	0.821	0.597	PIS55060.1	RPC53	Ortholog(s) have RNA polymerase III activity, role in tRNA transcription by RNA polymerase III and RNA polymerase III complex localization
PIS56730.1	23.93675596	24.48197678	24.19931789	25.11088269	24.64887749	24.64182158	0.356753013	0.895	0.595	PIS56730.1	RCA1	Protein involved in regulation of carbonic anhydrases; controls CO2 sensing; bZIP domain-containing transcription factor of the ATF/CREB family; null mutant displays slow growth, abnormal colony morphology and invasive growth
PIS55599.1	26.60379574	24.21905093	25.74545529	25.93208873	26.79122608	25.62746108	0.392231909	0.904	0.594	PIS55599.1	BUD5	Predicted GTP/GDP exchange factor for Rsr1; rat catheter biofilm induced

PIS52276.1	23.06722601	23.41603285	24.01169094	24.23757058	24.10737307	23.93220335	0.201866174	0.819	0.594	PIS52276.1	orf19.25	Ortholog(s) have tRNA (guanine) methyltransferase activity, tRNA (guanosine(9)-N1)-methyltransferase activity and role in mRNA methylation, tRNA N1-guanine methylation, tRNA methylation
PIS58838.1	23.15394939	23.43539253	22.36910981	23.35425908	23.62656469	23.75818919	0.380259277	0.901	0.594	PIS58838.1	orf19.398	Ortholog of C. dubliniensis CD36 : Cd36_08040, C. parapsilosis CDC317 : CPAR2_207180, C. auris B8441 : B9J08_000292 and Candida tenuis NRRL Y-1498 : CANTEDRAFT_114140
PIS58344.1	26.28930821	25.5502945	26.41075998	26.34037187	26.5475571	27.13515812	0.23053501	0.84	0.591	PIS58344.1	orf19.3076	Ortholog(s) have role in vesicle-mediated transport and Golgi membrane localization
PIS58706.1	25.12752086	25.50149009	24.09214978	25.60699106	25.98479929	24.89959103	0.33773713	0.889	0.59	PIS58706.1	orf19.1483	Ortholog(s) have copper ion binding activity, role in mitochondrial cytochrome c oxidase assembly and mitochondrial intermembrane space localization
PIS56586.1	27.64521787	28.39096949	27.52882049	28.06621828	28.54148044	28.72582974	0.127877785	0.725	0.59	PIS56586.1	orf19.5757	Ortholog(s) have FAD diphosphatase activity and role in flavin-containing compound metabolic process
PIS54786.1	32.11533601	32.60362327	31.84164526	32.48738812	32.8377583	33.00233295	0.069349475	0.549	0.589	PIS54786.1	HXK2	Hexokinase II; antigenic in humans; repressed by human neutrophils; Efg1-regulated; fluconazole-induced; gene regulation by Ssn6; present in exponential and stationary growth phase; flow model biofilm induced; Spider biofilm repressed
PIS54651.1	28.45136528	28.41470345	28.15605907	28.96202165	28.77702576	29.04976581	0.06520248	0.529	0.589	PIS54651.1	orf19.213	Subunit of the 19S regulatory particle lid of the proteasome
PIS48685.1	30.91648891	32.02063957	30.31067398	31.20926802	31.81578428	31.98761029	0.275997067	0.865	0.588	PIS48685.1	ANB1	Translation initiation factor eIF-5A; repressed in hyphae vs yeast cells; downregulated upon phagocytosis by murine macrophage; Hap43-induced; GlcNAc-induced protein; Spider biofilm repressed
PIS48404.1	25.94606505	26.23823244	24.7244093	26.86584667	25.83552175	25.97129711	0.409522948	0.908	0.588	PIS48404.1	orf19.6550	Mitochondrial outer membrane protein, component of vacuole and mitochondria patches (vCLAMPs); involved in mitophagy; mutants are defective in mitochondrial function and virulence; regulated by Sef1p, Sfu1p, and Hap43p
PIS50322.1	26.10178121	26.22186801	25.58439517	26.8377351	26.27811312	26.55416693	0.17942551	0.798	0.587	PIS50322.1	SEC2	Guanyl-nucleotide exchange factor for the small G-protein Sec4; delivery of post-Golgi secretory vesicles to sites of polarized growth; phosphorylation by Cdc28 needed for normal hyphal growth; Hap43-repressed; flow model biofilm induced

PIS55723.1	27.47009552	27.24544278	26.5750371	27.63108705	27.57825127	27.83878059	0.331301166	0.887	0.586	PIS55723.1	orf19.2446	Has domain(s) with predicted 2-dehydropantoate 2-reductase activity, oxidoreductase activity and role in pantothenate biosynthetic process
PIS48409.1	24.94597164	27.58256636	23.9500173	25.87720101	26.03724122	26.32220168	0.479544686	0.921	0.586	PIS48409.1	orf19.3515	Putative 3-hydroxyanthranilic acid dioxygenase, involved in NAD biosynthesis; Hap43p-repressed gene
PIS58683.1	23.92055485	23.25878205	24.33854413	23.86916634	25.6971445	23.69679614	0.404004786	0.907	0.582	PIS58683.1	RGA2	Putative GTPase-activating protein (GAP) for Rho-type GTPase Cdc42; involved in cell signaling pathways controlling cell polarity; induced by low-level peroxide stress; flow model biofilm induced
PIS48739.1	26.05704553	24.38234318	24.70438917	25.42746842	25.59573196	25.86396386	0.502547811	0.925	0.581	PIS48739.1	orf19.2149	Putative sulfate permease; S. cerevisiae ortholog YPR003C localizes to the endoplasmic reticulum; regulated by Sef1p-, Sfu1p-, and Hap43p
PIS55624.1	27.15520695	27.0455982	27.63173407	28.3003397	27.70294157	27.56591347	0.403897329	0.907	0.579	PIS55624.1	orf19.5941	Ortholog(s) have role in endoplasmic reticulum to Golgi vesicle-mediated transport and COPII-coated ER to Golgi transport vesicle, Golgi membrane, endoplasmic reticulum membrane localization
PIS55555.1	26.40177294	27.66693833	26.19422091	26.94227888	27.31298504	27.74550551	0.499212098	0.924	0.579	PIS55555.1	orf19.6606	Ortholog of C. parapsilosis CDC317 : CPAR2_201040, C. auris B8441 : B9J08_001657, Candida tenuis NRRL Y-1498 : CANTEDRAFT_112677 and Debaryomyces hansenii CBS767 : DEHA2E02420g
PIS55541.1	26.41578019	26.02744155	26.10831181	26.37966589	26.67732891	27.22769933	0.119568488	0.708	0.578	PIS55541.1	PDR6	Putative pleiotropic drug resistance regulatory protein 6; transcript regulated by Nrg1; induced by alpha pheromone in SpiderM medium
PIS58268.1	28.95027382	28.04201096	29.16137613	29.5746261	29.30409392	29.00463925	0.210182297	0.825	0.577	PIS58268.1	DPM3	Dolichol-phosphate mannose synthase subunit, essential for enzyme activity; flow model biofilm repressed
PIS58464.1	27.50724503	27.93358544	28.71776776	28.82396571	28.53722699	28.52488092	0.133692053	0.735	0.576	PIS58464.1	MNN11	Ortholog(s) have alpha-1,6-mannosyltransferase activity, role in protein N-linked glycosylation, protein glycosylation and Golgi apparatus, mannan polymerase complex localization
PIS54701.1	29.55955308	29.5572512	29.72012997	29.68919307	31.17545016	29.69575082	0.209520641	0.825	0.574	PIS54701.1	HGT7	Putative MFS glucose transporter; glucose, fluconazole, Snf3 induced, expressed at high glucose; 20 member C. albicans glucose transporter family; 12 TM regions predicted; flow model biofilm induced; Spider biofilm repressed

PIS58729.1	31.17511199	31.15996922	30.85958363	31.34891882	31.68245446	31.88645344	0.128873253	0.727	0.574	PIS58729.1	orf19.1709	Sterol carrier domain protein; alkaline downregulated; colony morphology-related gene regulation by Ssn6; Spider biofilm induced
PIS51674.1	30.31480774	30.28696043	30.37723072	31.23179602	30.63192554	30.83359802	0.049665146	0.442	0.573	PIS51674.1	LYS2	Heterodimeric alpha-aminoadipate reductase large subunit; lysine biosynthesis; predicted binding sites for AMP and alpha-aminoadipate; inhibited by lys or thialysine; regulated by Gcn2 and Gcn4; Spider biofilm induced, flow model repressed
PIS54843.1	32.38973202	32.38668983	32.22276568	32.89857728	32.90601783	32.91352343	0.031660478	0.325	0.573	PIS54843.1	UGP1	UTP-glucose-1-phosphaturidyl transferase; localizes to yeast, not hyphal cell surface; Hog1-repressed; stationary phase enriched; induced in oralpharyngeal candidasis; rat catheter biofilm repressed; Bcr1-repressed in RPMI a/a biofilms
PIS51758.1	24.7170251	25.71039754	24.20365159	25.39295933	25.62363252	25.32931319	0.217516374	0.831	0.572	PIS51758.1	ATG1	Putative protein serine/threonine kinase; predicted role in vesicle formation in autophagy and the cytoplasm-to-vacuole targeting (Cvt) pathway; Spider biofilm induced
PIS50366.1	25.99134968	26.67998987	26.26088857	26.22612366	27.2756745	27.14444565	0.220100009	0.833	0.571	PIS50366.1	orf19.168	Ortholog(s) have role in U4 snRNA 3'-end processing, exonucleolytic trimming to generate mature 3'-end of 5.8S rRNA from tricistronic rRNA transcript (SSU-rRNA, 5.8S rRNA and LSU-rRNA), more
PIS54577.1	26.65164874	26.66156549	25.16187877	25.79498213	27.18040167	27.21171512	0.399417101	0.906	0.571	PIS54577.1	orf19.1730	HMG-box protein; Spider biofilm repressed
PIS50335.1	30.37681736	30.48437529	30.433566	30.86657964	31.06615514	31.07350694	0.285538227	0.87	0.57	PIS50335.1	CHS3	Major chitin synthase of yeast and hyphae; synthesizes short-chitin fibrils; Chs4-activated; transcript induced at yeast-hyphal transition; Chs1 and Chs2, but not Chs3, are inhibited by the protoberberine HWY-289; Spider biofilm induced
PIS52274.1	25.16026405	26.19277528	26.3110818	26.70320402	26.6174099	26.05374987	0.418307065	0.91	0.57	PIS52274.1	orf19.3611	Protein of unknown function; Hap43-repressed gene; repressed by nitric oxide
PIS55703.1	26.01307747	26.27044261	26.29314428	26.70700123	26.92886245	26.64674453	0.12582299	0.721	0.569	PIS55703.1	orf19.2867	Ortholog(s) have cargo adaptor activity, phosphatidylinositol-3-phosphate binding activity
PIS51664.1	25.85486239	26.54215958	25.59281746	26.3084458	26.46081772	26.92339293	0.596296871	0.936	0.568	PIS51664.1	MIA40	Predicted component of the mitochondrial intermembrane space import machinery; Hap43p-repressed gene

PIS55492.1	28.28328175	27.8465888	28.49381628	29.14675063	28.76170672	28.41821383	0.141288044	0.748	0.568	PIS55492.1	VRG4	GDP-mannose transporter; essential; required for glycosylation, hyphal growth; functional homolog of S. cerevisiae Vrg4p, which imports GDP-mannose from cytoplasm to Golgi for protein and lipid mannosylation; no mammalian homolog
PIS58955.1	25.37435645	23.71131589	26.68320508	25.97321261	26.01583996	25.48236839	0.600652182	0.937	0.568	PIS58955.1	ZCF1	Zn(II)2Cys6 transcription factor; transcript regulated during hypha formation; 5'-UTR intron; mutants show decreased colonization of mouse kidneys; flow model biofilm induced; Spider biofilm induced
PIS52007.1	26.13939469	27.30458684	26.43274313	26.80187352	27.22385801	27.55267257	0.47516199	0.92	0.567	PIS52007.1	LTP1	Putative protein phosphatase of the PTP family (tyrosine-specific), similar to S. cerevisiae Ltp1p
PIS51417.1	28.58320191	27.75316038	27.63326925	28.54833364	28.65820576	28.45972221	0.312553756	0.881	0.566	PIS51417.1	orf19.3247	Putative ortholog of S. cerevisiae Laa1p; likely to be essential for growth, based on an insertional mutagenesis strategy
PIS51090.1	26.96032944	27.00951985	26.05597935	26.81878073	27.82860138	27.07343	0.47640552	0.921	0.565	PIS51090.1	orf19.4680	Possile protease; mutation confers hypersensitivity to toxic ergosterol analog
PIS48717.1	24.06764996	24.59982248	23.67558681	24.73673553	24.55804057	24.74306491	0.25725571	0.856	0.565	PIS48717.1	orf19.7034	Putative eIF4E-associated protein;, accelerates mRNA degradation by promoting decapping; Spider biofilm repressed
PIS55652.1	27.97861783	27.53543144	27.82885841	28.36213642	28.23064457	28.43702744	0.141860799	0.749	0.562	PIS55652.1	LYS4	Homoaconitase; regulated by Gcn4, Gcn2; induced in response to amino acid starvation (3-AT); induced by human whole blood or PMNs; Hap43-repressed; flow model and Spider biofilm repressed
PIS55570.1	29.51816351	29.38881391	28.34517666	29.35026244	29.91071809	29.67800463	0.389756048	0.904	0.562	PIS55570.1	NOP5	Ortholog of S. cerevisiae Nop58; involved in pre-rRNA process; Tn mutation affects filamentous growth; macrophage/pseudohyphal-induced; physically interacts with TAP-tagged Nop1; Spider biofilm repressed
PIS54480.1	25.59642754	26.28733185	25.7311177	26.72964428	26.24691498	26.32523057	0.406228122	0.907	0.562	PIS54480.1	orf19.7098	Putative protein of unknown function; Hap43-repressed; repressed by nitric oxide; Spider biofilm induced
PIS54955.1	31.6716468	31.94775276	30.8118543	31.7614836	32.16020714	32.19392784	0.138436155	0.743	0.561	PIS54955.1	MDG1	Ortholog(s) have role in pheromone-dependent signal transduction involved in conjugation with cellular fusion and eisosome, plasma membrane localization

PIS51970.1	24.72681185	20.24329152	22.11209833	26.88757454	20.90165162	20.97559552	0.740037128	0.948	0.561	PIS51970.1	orf19.5342	Ortholog(s) have role in NAD catabolic process and cytosol, extracellular region localization
PIS48492.1	31.03023863	30.8319436	30.76456508	31.68759612	31.47891086	31.14343723	0.409520824	0.908	0.561	PIS48492.1	RPT6	Putative ATPase of the 19S regulatory particle of the 26S proteasome; transcript regulated by Mig1; regulated by Gcn2 and Gcn4
PIS54691.1	27.22713131	28.21399755	26.83565293	27.71110704	27.98029617	28.26547077	0.325852386	0.885	0.56	PIS54691.1	orf19.2518	Ortholog(s) have ATPase activator activity, soluble NSF attachment protein activity, role in SNARE complex disassembly, autophagy, vacuole fusion, non-autophagic, vesicle fusion with Golgi apparatus and SNARE complex, cytosol localization
PIS58259.1	26.90120309	26.95585642	26.63402347	27.10261423	27.72390522	27.34178983	0.533473762	0.929	0.559	PIS58259.1	HDA1	Histone deacetylase; inducer of filamentation; conserved deacetylation motif; regulates white-to-opaque switch frequency but not opaque-to-white switch; greater expression in white cells than opaque cells; inhibited by trichostatin-A;
PIS55444.1	27.84617638	28.3831571	28.27007135	29.37096557	28.74971295	28.05676209	0.155476379	0.769	0.559	PIS55444.1	orf19.1124.2	Ortholog(s) have diphthine synthase activity and role in protein histidyl modification to diphthamide
PIS52062.1	30.14457298	30.76258299	29.94922954	30.72062234	30.90345237	30.90846736	0.265701607	0.86	0.559	PIS52062.1	orf19.3060	OPutative dolichyl-diphosphooligosaccharide-protein glycotransferase; role in protein N-linked glycosylation; Spider biofilm repressed
PIS51265.1	21.52922989	20.83364399	20.8815495	22.59460663	21.42148436	20.90203334	0.285814904	0.87	0.558	PIS51265.1	MSN4	Zinc finger transcription factor; similar to S. cerevisiae Msn4, but not a significant stress response regulator in C. albicans; partly complements STRE-activation defect of S. cerevisiae msn2 msn4 double mutant; flow model biofilm induced
PIS48240.1	23.46037476	24.89732748	25.73143234	25.57753552	25.21397478	24.97170765	0.413213312	0.909	0.558	PIS48240.1	orf19.6526	Ortholog(s) have nuclear periphery localization
PIS58503.1	26.69634195	25.14963398	26.82485481	27.20921807	26.76315608	26.36739812	0.37509808	0.9	0.556	PIS58503.1	orf19.1686	Has domain(s) with predicted kinase activity and role in carbohydrate metabolic process
PIS55553.1	24.80251947	25.56621681	25.63967783	26.39548021	25.66510899	25.61717565	0.161956556	0.777	0.556	PIS55553.1	orf19.7344	Ortholog(s) have DNA binding, chromatin binding, histone deacetylase activity and role in chromosome segregation, negative regulation of transcription by RNA polymerase II, regulatory ncRNA-mediated gene silencing

PIS49556.1	26.55364119	26.41493379	26.64829824	27.3420424	27.41400048	26.52680108	0.306023059	0.878	0.555	PIS49556.1	orf19.2246	Ortholog(s) have role in nuclear-transcribed mRNA catabolic process, non-stop decay
PIS51952.1	27.5547355	26.41431987	27.81351377	28.23635212	27.53946071	27.67132384	0.577722091	0.934	0.555	PIS51952.1	orf19.988	Glycerophosphocholine acyltransferase; synthesizes phosphatidylcholine (PC); oral infection upregulated gene; null mutant shows reduced ability to damage oral epithelial cells and decreased resistance to drugs targeting lipid synthesis
PIS49546.1	26.85242997	26.83420954	27.28378464	27.56519495	27.60628425	27.46061478	0.20692397	0.823	0.554	PIS49546.1	orf19.2472.1	Ortholog of C. dubliniensis CD36 : Cd36_61610, C. parapsilosis CDC317 : CPAR2_107530, C. auris B8441 : B9J08_004570 and Candida tenuis NRRL Y-1498 : CANTEDRAFT_115252
PIS58188.1	22.25500177	23.86743796	26.15054576	26.7462794	24.83163749	22.35757019	0.631421454	0.94	0.554	PIS58188.1	orf19.6025	Ortholog(s) have N-acetylglucosaminyldiphosphodolichol N-acetylglucosaminyltransferase activity, role in dolichol-linked oligosaccharide biosynthetic process and UDP-N-acetylglucosamine transferase complex,
PIS58304.1	25.25804173	24.51250472	25.33929141	24.88215212	25.89223705	25.99577681	0.211448061	0.826	0.553	PIS58304.1	DFI1	Cell-surface associated glycoprotein; promotes activation of Cek1 in a matrix-dependent manner; N-glycosylated; Spider biofilm induced
PIS56853.1	23.6209641	24.98257668	24.07266977	25.79940092	24.72095828	23.81601763	0.365489901	0.897	0.553	PIS56853.1	orf19.4583	Protein with a mitochondrial carrier protein domain; possibly an essential gene, disruptants not obtained by UAU1 method; Spider biofilm repressed
PIS58500.1	28.38856678	28.53076707	28.43679261	28.5505386	29.25554936	29.2074365	0.135229865	0.738	0.552	PIS58500.1	orf19.3329	Ortholog(s) have sphingosine-1-phosphate phosphatase activity, role in calcium-mediated signaling and endoplasmic reticulum localization
PIS59019.1	25.6601421	28.39256921	26.20014839	26.95673334	27.45949917	27.49066403	0.451829164	0.917	0.551	PIS59019.1	GCV3	Glycine decarboxylase, subunit H; protein level decrease in stationary phase cultures
PIS52089.1	27.62762518	28.47910943	27.75815345	28.25398936	28.77838987	28.48551076	0.446810381	0.916	0.551	PIS52089.1	KOG1	Putative TORC1 subunit; heterozygous null mutant displays sensitivity to rapamycin; likely to be essential for growth, based on an insertional mutagenesis strategy
PIS49619.1	26.88129601	26.5160139	27.01575491	27.51033884	27.42414507	27.12668483	0.255125118	0.855	0.549	PIS49619.1	HPC2	Ortholog(s) have DNA binding, nucleosome binding activity, role in chromatin organization, regulation of transcription by RNA polymerase II, transcription elongation by RNA polymerase II and HIR complex localization

PIS49761.1	26.48965655	26.56983773	26.16126493	26.51874032	27.26636906	27.08193409	0.277369006	0.866	0.549	PIS49761.1	orf19.216	Protein with a metallo-dependent phosphatase domain; ketoconazole-induced; upregulation correlates with clinical development of fluconazole resistance
PIS50608.1	27.06783145	27.77037485	27.59879721	28.36404417	27.97644897	27.73764808	0.523321239	0.928	0.547	PIS50608.1	HGT5	Putative glucose transporter of the major facilitator superfamily; the C. albicans glucose transporter family comprises 20 members; 12 probable membrane-spanning segments, extended N terminus; expressed in rich medium; Hap43p-
PIS49495.1	23.61446488	24.72712969	23.80016168	24.74746453	24.50389806	24.52179081	0.187685505	0.806	0.544	PIS49495.1	TRP99	Putative thioredoxin peroxidase/alkyl hydroperoxide reductase; induced in low iron; regulated by Gcn4; induced in response to amino acid starvation (3-AT treatment)
PIS48241.1	23.43572331	24.39018527	25.31526226	24.05068448	25.66705074	25.05115949	0.305440019	0.878	0.543	PIS48241.1	orf19.6525	S. cerevisiae ortholog Inp1 is a peripheral membrane protein of peroxisomes involved in peroxisomal inheritance; induced by Mnl1 under weak acid stress
PIS51048.1	28.39287064	27.91898155	28.07450072	28.7386679	28.73541579	28.5416967	0.462135804	0.918	0.543	PIS51048.1	orf19.6973	ATP-dependent LON protease family member; Hap43-repressed gene; regulated by Gcn2 and Gcn4; Spider biofilm induced
PIS52443.1	28.06419426	28.6024212	27.83866633	28.60564796	27.94875534	29.57725249	0.365169616	0.897	0.542	PIS52443.1	FAA2	Putative acyl CoA synthetase; expression regulated upon white-opaque switch; rat catheter biofilm induced; Spider biofilm induced
PIS48734.1	26.70753457	27.06738365	26.86318514	27.23429151	27.6046754	27.42533843	0.232505752	0.841	0.542	PIS48734.1	orf19.6199	Ortholog(s) have 5'-3' DNA helicase activity, role in postreplication repair and DNA helicase A complex, nuclear replisome localization
PIS58025.1	28.86006596	28.26243426	29.22689711	29.51941206	29.36235472	29.08994071	0.357402351	0.895	0.541	PIS58025.1	FEN12	Putative protein with a predicted role in the elongation of fatty acids; amphotericin B, caspofungin repressed
PIS58938.1	29.97240743	29.35324837	30.18662552	30.71090707	30.20234501	30.21812466	0.287019044	0.87	0.54	PIS58938.1	PEX11	Putative peroxisomal membrane protein; role in fatty acid oxidation; expression is Tac1-regulated; Hms1p-dependent induction by geldamycin; Spider biofilm induced
PIS50638.1	25.68867739	26.26501242	25.17131569	25.6769678	26.20700982	26.85947013	0.516891617	0.927	0.539	PIS50638.1	ECM38	Putative gamma-glutamyltransferase; alkaline upregulated; Spider biofilm induced; possibly an essential gene, disruptants not obtained by UAU1 method

PIS48557.1	28.60391862	27.10315378	28.76161632	28.41397959	28.98228907	28.68877601	0.487481063	0.922	0.539	PIS48557.1	NEP1	Ortholog(s) have rRNA (pseudouridine) methyltransferase activity
PIS51424.1	25.37911042	27.86004164	26.49483071	29.32278477	26.03101158	25.99624856	0.583129302	0.935	0.539	PIS51424.1	orf19.1082.1	Ortholog(s) have cytochrome-c oxidase activity and role in mitochondrial cytochrome c oxidase assembly, mitochondrial electron transport, cytochrome c to oxygen
PIS52336.1	22.23967683	21.34077448	22.30497462	24.39854412	25.25171586	17.85149199	0.764810091	0.95	0.539	PIS52336.1	TEC1	TEA/ATTS transcription factor; white cell pheromone response, hyphal gene regulation; required for Spider and RPMI biofilm formation; regulates BCR1; Cph2 regulated transcript; alkaline, rat catheter, Spider, flow model biofilm induced
PIS51681.1	28.62770611	28.12948987	28.69378078	29.16371493	29.04223582	28.85818883	0.210674311	0.826	0.538	PIS51681.1	MCM1	Transcription factor; regulator of hyphal growth; may act with Wor1p; canonical and non-canonical binding sites; MADS domain DNA-binding motif; similar to S. cerevisiae Mcm1p; greater expression in white than opaque cells; intron in 5'-UTR
PIS58941.1	25.86663944	26.19773352	26.5778353	26.97487037	26.65925207	26.62298135	0.397158126	0.905	0.538	PIS58941.1	orf19.1092	Dolichol-P-Man dependent alpha(1-3) mannosyltransferase; role in the synthesis of dolichol-linked oligosaccharide donor for N-linked glycosylation of proteins; rat catheter biofilm repressed
PIS48707.1	23.43034846	25.33813576	24.57030085	24.77268728	24.75474139	25.42439628	0.575178436	0.934	0.538	PIS48707.1	orf19.6917	Putative heat shock protein with a zinc finger motif; required for protein import into mitochondria in S. cerevisiae; Spider biofilm induced
PIS58795.1	25.67658787	24.65316788	25.61415406	25.59958505	26.02620632	25.92825961	0.490923737	0.923	0.537	PIS58795.1	orf19.2320	Putative serine/threonine-protein kinase; possibly an essential gene, disruptants not obtained by UAU1 method
PIS51228.1	27.32482491	27.89648609	27.59122151	27.77676608	28.46946268	28.17111964	0.191977911	0.81	0.535	PIS51228.1	orf19.2259	Ortholog(s) have structural constituent of ribosome activity and mitochondrial large ribosomal subunit localization
PIS55002.1	30.42774646	31.37181109	29.69984664	31.13878079	30.85758403	31.10656219	0.197423834	0.815	0.535	PIS55002.1	TMA19	Cell wall protein, ortholog of S. cerevisiae Tma19p (Ykl065cp)
PIS49723.1	23.69429098	23.3105139	26.50835309	25.77162827	24.90609972	24.43636601	0.751095382	0.949	0.534	PIS49723.1	orf19.6797	Ortholog of C. dubliniensis CD36 : Cd36_86960, C. parapsilosis CDC317 : CPAR2_808800, C. auris B8441 : B9J08_004750 and Candida tenuis NRRL Y-1498 : CANTEDRAFT_113621

PIS58423.1	24.99436602	24.55596232	24.41142007	24.8545749	26.11808824	24.58718984	0.680347506	0.944	0.533	PIS58423.1	NUP60	Ortholog of S. cerevisiae Nup60p; a subunit of the nuclear pore complex; mutants are viable
PIS48476.1	27.79763692	28.95034156	27.25572731	28.41270425	28.56846446	28.62297313	0.446761455	0.916	0.533	PIS48476.1	orf19.1267	Ortholog(s) have cytosol, plasma membrane localization
PIS51108.1	25.05998001	25.80547822	25.60273257	26.67655866	25.79888881	25.58742116	0.217027464	0.831	0.532	PIS51108.1	orf19.7527	Ortholog of C. dubliniensis CD36 : Cd36_25130, C. parapsilosis CDC317 : CPAR2_800100, C. auris B8441 : B9J08_002680 and Candida tenuis NRRL Y-1498 : CANTEDRAFT_107256
PIS58582.1	27.3274716	27.32080212	27.4967839	27.9849725	27.90165876	27.85132473	0.186945234	0.805	0.531	PIS58582.1	PAN3	Ortholog(s) have RNA binding, poly(A) binding activity, role in DNA repair, nuclear-transcribed mRNA poly(A) tail shortening, postreplication repair and PAN complex localization
PIS51282.1	24.8534644	24.60245169	24.63934365	26.0489876	24.92705053	24.711421	0.173574178	0.791	0.531	PIS51282.1	VPS8	Putative vacuolar sorting protein; downregulated upon adherence to polystyrene
PIS54953.1	25.95106991	24.65409111	26.09104939	26.44894104	25.63548595	26.20055682	0.195235581	0.813	0.53	PIS54953.1	AVT7	Ortholog of S. cerevisiae Avt7 transporter; repressed upon adherence to polystyrene; constitutive expression independent of MTL or white-opaque status; Spider biofilm induced
PIS50552.1	28.90652654	29.56188016	29.06039357	29.56236232	29.74627111	29.80894154	0.285737448	0.87	0.53	PIS50552.1	GUT1	Putative glycerol kinase; downregulated upon adherence to polystyrene; greater mRNA abundance observed in a cyr1 homozygous null mutant than in wild type
PIS58320.1	23.88035989	24.05607682	24.72880376	24.69340543	24.81188068	24.74969808	0.30724729	0.879	0.53	PIS58320.1	GYP8	Ortholog(s) have GTPase activator activity, role in vesicle-mediated transport and cytoplasm, peroxisome localization
PIS49615.1	22.223662	22.85037933	22.80348156	22.71261723	23.55194722	23.20205248	0.784635047	0.951	0.53	PIS49615.1	KNS1	Protein kinase involved in negative regulation of PolIII transcription; effector kinase of the TOR signaling pathway, phosphorylates Rpc53p to regulate ribosome and tRNA biosynthesis; Spider and flow model biofilm induced
PIS54621.1	25.13562402	26.16458913	25.31067112	26.29682371	25.58595144	26.31870767	0.346391861	0.892	0.53	PIS54621.1	orf19.1381	Ortholog of S. cerevisiae/S. pombe Lsb5; predicted role in actin cortical patch localization, actin filament organization, endocytosis; flow model biofilm induced; Spider biofilm repressed

PIS56783.1	31.4098041	31.15147565	31.53179094	31.93579453	31.86067193	31.88670354	0.065287774	0.53	0.53	PIS56783.1	orf19.338	Putative glycoside hydrolase; stationary phase enriched protein; Hog1p-downregulated; shows colony morphology-related gene regulation by Ssn6p
PIS58557.1	29.37219914	28.29349733	29.42614325	29.85790792	29.5405149	29.28286126	0.403576576	0.907	0.53	PIS58557.1	ZRT1	Putative zinc transporter; acts with Pra1 in sequestration of zinc from host tissues during infection; hyphal, macrophage-induced; alkaline induced upon adherence to polystyrene; induced in oralpharyngeal candidasis; Spider biofilm induced
PIS54685.1	25.68623291	25.87405472	26.65256776	27.15072138	26.25054076	26.39976476	0.458984861	0.918	0.529	PIS54685.1	FTH2	Putative iron permease involved in virulence and production of prostaglandin E2; mutants show decreased hyphal growth against C. elegans and abnormal biofilm metabolism and morphology
PIS56532.1	28.87964538	29.65558523	29.28197817	29.44916685	29.89613611	30.04816494	0.281976168	0.868	0.525	PIS56532.1	orf19.5515	Ortholog(s) have mRNA binding, ribosome binding activity
PIS54700.1	28.95457051	28.48679391	29.18997961	29.24211877	29.63246774	29.32456126	0.238236871	0.845	0.523	PIS54700.1	orf19.2262	Probable quinone oxidoreductase; involved in protection from oxidative stress; induced by quinones, benomyl treatment, nitric oxide, and Spider biofilm; null mutant shows increased susceptibility to killing by neutrophils
PIS48825.1	25.8028973	27.02025613	27.21571736	27.41195857	26.55247171	27.64278546	0.287007305	0.87	0.523	PIS48825.1	RAM2	Alpha subunit of heterodimeric protein geranylgeranyltransferase type I and farnesyltransferase; a-specific transcript; lovastatin, fluconazole regulated; GGTase I binds zinc, is Mg-dependent; Cdc42 substrate; rat catheter biofilm
PIS51601.1	24.46456046	26.81276656	25.65136929	25.6466581	26.63388893	26.21655827	0.569585337	0.933	0.523	PIS51601.1	REG1	Putative protein phosphatase regulatory subunit; Hap43-repressed gene; macrophage/pseudohyphal-induced; possibly regulated upon hyphal formation; flow model biofilm induced
PIS51811.1	26.25198218	25.74247309	25.99120668	26.37780866	26.36644459	26.80707731	0.417400617	0.91	0.522	PIS51811.1	orf19.6822	Ortholog(s) have cullin family protein binding, protein-macromolecule adaptor activity, ubiquitin conjugating enzyme binding, ubiquitin-like protein binding activity and role in protein neddylation
PIS58681.1	24.56362879	24.17750833	25.14153268	26.12250756	25.03132613	24.2921758	0.413565706	0.909	0.521	PIS58681.1	orf19.4676	Protein with homology to mitochondrial intermembrane space proteins; regulated by Sef1p-, Sfu1p-, and Hap43p
PIS50330.1	29.40160788	29.8519888	29.7794329	30.26773167	30.21200057	30.1160263	0.334320246	0.888	0.521	PIS50330.1	orf19.4931	Putative tRNA-Cys synthetase; induced by alpha pheromone in SpiderM medium; ribosomal subunits, translation factors, tRNA synthetases are downregulated upon phagocytosis by murine macrophage

PIS50636.1	29.35368358	29.58246099	30.15764145	30.33224729	30.09439279	30.23048699	0.17699402	0.795	0.521	PIS50636.1	RDI1	Putative rho GDP dissociation inhibitor; transposon mutation affects filamentous growth; farnesol, filament-induced; regulated by Nrg1, Tup1; protein levels low in stationary phase yeast
PIS52319.1	24.13104498	23.85384801	22.89557693	24.0730753	24.34268438	24.02680054	0.207172203	0.823	0.521	PIS52319.1	VPH2	Protein required for for proper vacuolar ATPase assembly and vacuolar functions
PIS49618.1	30.01094699	29.46324582	30.23138539	30.51591738	30.44241042	30.30949393	0.146699677	0.756	0.521	PIS49618.1	YBP1	Protein involved in response to oxidative stress, binds and stabilizes Cap1p transcription factor in response to H2O2; essential for macrophage killing
PIS51402.1	25.52759496	24.34709597	23.38819073	26.00662827	25.93946471	22.87627369	0.540521999	0.93	0.52	PIS51402.1	MTG1	Putative mitochondrial GTPase; likely essential for respiratory competence and in large ribosomal subunit assembly; mitochondrial translation; Spider biofilm induced
PIS52310.1	28.69825795	28.63253402	28.32711628	29.18096835	29.0747666	28.96057348	0.19507346	0.813	0.519	PIS52310.1	IRA2	GTPase-activating protein; negatively regulates RAS by converting it from the GTP- to the GDP-bound inactive form; Spider biofilm induced; flow model biofilm repressed
PIS50419.1	27.53445748	28.9836819	27.86516005	27.88560393	29.09348857	28.95688061	0.409325418	0.908	0.518	PIS50419.1	orf19.5747	Ortholog(s) have structural constituent of ribosome activity and mitochondrial small ribosomal subunit localization
PIS55556.1	25.06173236	26.47360614	25.24958386	25.82776093	26.15828104	26.34873535	0.316115272	0.882	0.517	PIS55556.1	AXL1	Putative endoprotease; induced by alpha factor; transcript is upregulated in an RHE model of oral candidiasis and in clinical isolates from HIV+ patients with oral candidiasis
PIS50604.1	30.13815144	31.2095368	29.71819529	30.46115468	30.97349032	31.1802439	0.215505754	0.83	0.516	PIS50604.1	SBP1	Similar to RNA binding proteins; downregulated upon adherence to polystyrene; stationary-phase enriched protein
PIS58242.1	26.44397239	25.90797176	25.41271832	26.20367719	26.33421351	26.77054851	0.44215946	0.915	0.515	PIS58242.1	orf19.5160	Regulatory subunit of PP2A-like protein phosphatase Sit4p, involved in cell wall maintenance, regulation of hyphal growth, and virulence
PIS58893.1	28.16819726	28.2418891	28.56418579	28.78272974	28.80036732	28.9321412	0.137075837	0.741	0.514	PIS58893.1	orf19.2036	Predicted dihydrodiol dehydrogenase; ortholog of S. pombe SPAC513.06c; flow model and rat catheter biofilm repressed

PIS50334.1	26.78916095	27.16228693	27.17555748	27.54463471	27.69481613	27.42167064	0.360433868	0.896	0.511	PIS50334.1	FAD3	Omega-3 fatty acid desaturase; production of alpha-linolenic acid, a major component of membranes; caspofungin induced; Plc1-regulated; colony morphology-related gene regulation by Ssn6; Spider biofilm induced, flow model biofilm repressed
PIS58053.1	25.46969155	25.97262684	26.27424782	27.00260091	26.15516254	26.0875598	0.430225789	0.912	0.51	PIS58053.1	orf19.7615	Protein involved in endoplasmic reticulum (ER) to Golgi vesicle-mediated transport; putative subunit of the transport protein particle (TRAPP) complex of the cis-Golgi; Spider biofilm induced
PIS48750.1	29.76240009	29.80784396	29.53199157	30.02025432	30.2967718	30.31338803	0.280851033	0.868	0.509	PIS48750.1	LCB2	Putative serine palmitoyltransferase component; mutation confers hypersensitivity to aureobasidin A
PIS55783.1	29.71011314	29.32760854	30.48624834	30.45204024	30.31369215	30.28207963	0.37448729	0.9	0.508	PIS55783.1	CRC1	Mitochondrial carnitine carrier protein
PIS56566.1	31.9486794	32.03009728	31.65738838	32.19175465	32.4909152	32.47474619	0.107769825	0.681	0.507	PIS56566.1	ECM4	Cytoplasmic glutathione S-transferase; regulated by Nrg1, Tup1; induced in core stress response, in cyr1 or ras1 mutant (yeast or hyphal cells); Tn mutation affects filamentous growth; stationary phase enriched; Spider biofilm induced
PIS52183.1	28.52522966	28.29755598	28.56853649	29.01840372	28.89333255	28.99985106	0.312738406	0.881	0.507	PIS52183.1	GYP7	Protein similar to S. cerevisiae Gyp7p (GTPase-activating protein for Ypt1p); caspofungin-induced
PIS51621.1	29.77539589	29.67795477	29.0798131	30.16702308	29.93670064	29.95013938	0.583376745	0.935	0.507	PIS51621.1	OFD1	Putative prolyl hydroxylase family member; regulates Ume6p stability in response to oxygen; inhibited by hypoxia; repressed by prostaglandins; Spider biofilm induced
PIS48796.1	24.9062554	25.61108132	24.26348546	24.63859837	25.66985454	25.99455917	0.697089599	0.945	0.507	PIS48796.1	orf19.686	Protein of unknown function; regulated by Nrg1
PIS54531.1	27.88525346	27.17801435	28.31589733	28.61522264	28.31637615	27.9655422	0.477685236	0.921	0.506	PIS54531.1	orf19.4795	Protein of unknown function; Sef1-, Sfu1-, and Hap43 regulated; Spider biofilm induced
PIS56605.1	28.67745405	27.89999071	28.86983955	28.54744698	28.58376268	29.83201238	0.210234665	0.825	0.505	PIS56605.1	LPT1	Lysophospholipid acyltransferase, involved in phospholipid remodeling; role in glycerophospholipid biosynthesis; rat catheter biofilm repressed

PIS58778.1	26.81026802	26.47629634	26.98030823	26.78273531	27.80078553	27.19952287	0.430740343	0.913	0.505	PIS58778.1	orf19.7082	S-adenosylmethionine transporter of the mitochondrial inner membrane; mitochondrial carrier family; predicted role in biotin biosynthesis and respiratory growth; Spider biofilm repressed
PIS51316.1	24.38682314	23.75668337	24.70244134	24.87595579	25.02568311	24.45627429	0.473457803	0.92	0.504	PIS51316.1	orf19.4904	Ortholog(s) have nucleosome binding activity, role in chromatin remodeling and Swr1 complex, cytoplasm, nuclear periphery localization
PIS49650.1	24.43293907	24.43578102	24.17057356	23.96292911	25.28302742	25.30683662	0.633039558	0.94	0.504	PIS49650.1	orf19.6492	Predicted protein serine/threonine kinase and/or protein tyrosine kinase; Spider biofilm induced
PIS55774.1	24.04915814	25.26457324	24.36784419	24.48905537	25.76559047	24.94025234	0.294613267	0.874	0.504	PIS55774.1	orf19.6751	Ortholog(s) have tRNA (cytidine(32)-2'-O)-methyltransferase activity, tRNA (guanine(34)-2'-O)-methyltransferase activity, tRNA 2'-O-methyltransferase activity
PIS54800.1	25.58640147	23.90763587	26.31159918	26.44822359	25.95284705	24.91800566	0.594080356	0.936	0.504	PIS54800.1	SUA71	Transcription factor TFIIB; required for transcription initiation and start site selection by RNA polymerase II; downregulated during planktonic growth, whereas related SUA72 is induced; induced by Tbf1; rat catheter, Spider biofilm induced
PIS55674.1	26.48664877	26.38211875	26.37217541	27.14950437	26.65421148	26.9454637	0.335054671	0.888	0.503	PIS55674.1	orf19.5457	Ortholog(s) have lipid droplet localization
PIS58486.1	27.81904369	29.25746245	27.56963336	28.3525287	29.01355726	28.78636865	0.347299256	0.892	0.502	PIS58486.1	orf19.7295	Ortholog(s) have protein phosphatase 1 binding activity, role in cortical actin cytoskeleton organization, positive regulation of clathrin-dependent endocytosis, protein secretion and actin cortical patch, nucleus localization
PIS51425.1	22.41155852	22.92953235	23.06717483	23.13970793	23.15649102	23.61376405	0.529313919	0.928	0.501	PIS51425.1	orf19.1083	Putative protein of unknown function; macrophage-induced gene
PIS49622.1	28.29275433	26.94707984	28.65257766	28.46830635	28.49462096	28.43099086	0.232438433	0.841	0.501	PIS49622.1	orf19.7194	Protein required for virulence in reconstituted human epithelium (RHE) model of ex vivo infection; decreased transcription is observed upon fluphenazine treatment; induced upon adherence to polystyrene
PIS51557.1	26.34020243	25.85816999	25.71603351	26.14182945	26.19775656	27.0768106	0.512127302	0.926	0.501	PIS51557.1	PRY1	Pry family pathogenesis-related protein; extracellular; opaque specific transcript; repressed by alpha pheromone in SpiderM medium; possibly essential, disruptants not obtained by UAU1 method; Spider biofilm induced

PIS51790.1	26.18393757	25.25167461	26.93341544	26.41809149	26.92394372	26.52742324	0.303026467	0.877	0.5	PIS51790.1	orf19.6225.1	Ortholog(s) have role in mitochondrial cytochrome c oxidase assembly
PIS50351.1	24.66492266	24.26703395	25.24144492	25.01466415	25.54390678	25.11036	0.355647699	0.895	0.499	PIS50351.1	RAD50	Putative DNA double-strand break repair factor; involved in response to oxidative stress and drug resistance; flow model biofilm repressed
PIS55505.1	22.47462429	24.17537472	22.99645698	23.71771608	23.90209581	23.51991994	0.472318273	0.92	0.498	PIS55505.1	GCS1	Gamma-glutamylcysteine synthetase; glutathione synthesis, required for virulence;induced in low iron, H2O2, Cd, or presence of human neutrophils; possibly adherence-induced; Spider and F-12/CO2 biofilm induced
PIS55446.1	26.82580011	26.57223261	27.36553729	27.01816259	27.26891901	27.96313608	0.349972717	0.893	0.496	PIS55446.1	orf19.6679	Ortholog(s) have cytoplasm, nucleus localization
PIS54644.1	25.30145703	24.47806908	26.47700765	25.77042441	25.50826658	26.46384798	0.631586242	0.94	0.495	PIS54644.1	STB3	Putative SIN3-binding protein 3 homolog; caspofungin induced; macrophage/pseudohyphal-repressed; rat catheter biofilm induced
PIS54671.1	29.77112822	29.04291577	29.48182016	30.08532626	29.88751686	29.80567435	0.1049926	0.674	0.494	PIS54671.1	orf19.4758	Putative reductase or dehydrogenase; Hap43-repressed gene; alkaline repressed
PIS54560.1	28.65536108	29.62763202	28.31455267	29.15962785	29.37087448	29.54788159	0.177455162	0.795	0.494	PIS54560.1	orf19.577	Predicted protein tyrosine phosphatase; rat catheter biofilm induced
PIS56537.1	29.96268481	30.57711472	29.62285593	30.45034722	30.33832821	30.85243465	0.439228288	0.914	0.493	PIS56537.1	GCV1	Putative T subunit of glycine decarboxylase; transcript negatively regulated by Sfu1; Spider biofilm repressed
PIS56758.1	27.86918408	26.86745613	27.91591606	28.03573034	28.2074847	27.88764516	0.208340895	0.824	0.493	PIS56758.1	orf19.969	Ortholog(s) have ribose phosphate diphosphokinase activity, role in 5-phosphoribose 1-diphosphate biosynthetic process, fungal-type cell wall organization and cytoplasm, ribose phosphate diphosphokinase complex localization
PIS58428.1	30.28201761	30.13763362	30.26997672	30.86787722	30.70976057	30.59041304	0.073241134	0.567	0.493	PIS58428.1	SKI3	Ortholog(s) have role in nuclear-transcribed mRNA catabolic process, 3'-5' exonucleolytic nonsense-mediated decay, nuclear-transcribed mRNA catabolic process, exonucleolytic and 3'-5', more

PIS56696.1	30.13108807	30.02671352	30.54330251	31.16938933	30.52232624	30.48429276	0.597121752	0.936	0.492	PIS56696.1	CAS1	Putative transcription factor with Ku70/Ku80 beta-barrel DNA-binding motif; involved in telomerase regulation and telomere protection; mutation causes marginal increase in caspofungin sensitivity
PIS49643.1	25.75575585	26.6168531	24.01391112	25.76203682	26.28208615	25.81739963	0.424541599	0.911	0.492	PIS49643.1	MRPL27	Putative 60S ribosomal protein L27, mitochondrial precursor
PIS50404.1	28.99506689	29.39587915	28.43377814	29.19805381	29.70467452	29.39808745	0.173093933	0.791	0.492	PIS50404.1	orf19.4825	Mitochondrial matrix protein; required for assembly/stability of the F1 sector of mitochondrial F1F0 ATP synthase at high temperature; rat catheter biofilm repressed
PIS50591.1	26.38150271	24.73373256	26.01899685	26.04189358	26.39181671	26.17447124	0.403316842	0.907	0.491	PIS50591.1	orf19.4191.1	Ortholog(s) have ubiquitin-ubiquitin ligase activity and role in free ubiquitin chain polymerization, protein polyubiquitination, ubiquitin-dependent ERAD pathway, ubiquitin-dependent protein catabolic process
PIS58653.1	29.23209624	30.07839714	28.97293458	30.28274821	29.56118308	29.90694977	0.235097924	0.843	0.489	PIS58653.1	orf19.590	Putative thiamine biosynthesis enzyme; decreased expression in an ssr1 null mutant; protein present in exponential and stationary growth phase yeast cultures
PIS59007.1	24.88989835	25.24626811	23.55298162	25.82079124	24.83293233	24.50084077	0.551906526	0.931	0.488	PIS59007.1	orf19.9	Ortholog of C. dubliniensis CD36 : Cd36_32700, C. parapsilosis CDC317 : CPAR2_204280, C. auris B8441 : B9J08_000471 and Candida tenuis NRRL Y-1498 : CANTEDRAFT_113831
PIS52088.1	25.13562402	25.61953794	25.50002471	24.85215157	26.44408438	26.42121689	0.415546527	0.909	0.487	PIS52088.1	MTM1	Ortholog(s) have pyridoxal phosphate binding activity, role in intracellular iron ion homeostasis, pyridoxal phosphate transport and mitochondrion localization
PIS52461.1	24.64522913	25.80510381	24.82311113	25.26573127	25.6799573	25.7879829	0.437102118	0.914	0.487	PIS52461.1	orf19.2828	Ortholog(s) have alpha-tubulin binding, microtubule binding activity, role in cytoplasmic microtubule organization, post-chaperonin tubulin folding pathway, protein folding and cytoplasm localization
PIS51648.1	29.32607925	29.68129712	29.22986213	30.01883257	29.77774867	29.9021638	0.15324966	0.766	0.487	PIS51648.1	TUB2	Beta-tubulin; functional homolog of ScTub2; overproduction makes S. cerevisiae inviable; has two introns; GlcNAc, hypha fluconazole-induced; slow growth, ectopic expression increases white-to opaque switch; rat catheter biofilm repressed
PIS50515.1	31.0371575	31.06053113	31.20954375	31.49407514	31.64162429	31.63131458	0.201030668	0.818	0.487	PIS50515.1	VTC4	Putative polyphosphate synthetase; decreased expression in hyphae compared to yeast-form cells; fungal-specific (no human or murine homolog); virulence-group-correlated expression

PIS49769.1	27.32213134	24.21313858	27.18580343	27.27965173	26.40503836	26.49275675	0.581630499	0.935	0.485	PIS49769.1	orf19.7375	Putative U1A component of the U1 snRNP, involved in splicing; contains two RNA recognition motifs (RRMs); ortholog of S. cerevisiae MUD1
PIS50336.1	28.49838303	28.65302662	28.55105063	28.7841973	29.09482862	29.27539624	0.088053514	0.624	0.484	PIS50336.1	MON2	Peripheral membrane protein; role in endocytosis and vacuole integrity; flow model and rat catheter biofilm repressed
PIS52193.1	23.96512582	23.56861092	23.45040705	23.95146481	24.1376213	24.34632232	0.136919746	0.741	0.484	PIS52193.1	orf19.1769	Ortholog(s) have G-protein beta/gamma-subunit complex binding, actin binding activity, role in positive regulation of transcription by RNA polymerase II, protein folding and cytoplasm localization
PIS56882.1	28.45874416	27.79815665	28.88704385	29.07190264	28.87237317	28.65138933	0.211632092	0.827	0.484	PIS56882.1	orf19.5628	Mitochondrial dicarboxylate transporter; possibly an essential gene, disruptants not obtained by UAU1 method
PIS54726.1	24.60964338	26.08977443	24.23962659	25.38506101	26.13916553	24.85371685	0.506465017	0.925	0.48	PIS54726.1	orf19.6355	Ortholog(s) have unfolded protein binding activity, role in ribosome biogenesis and nucleolus localization
PIS49474.1	28.092838	27.35135471	28.18825242	28.69263212	28.25294417	28.12645163	0.314516753	0.881	0.48	PIS49474.1	orf19.7307	Putative oxidoreductase; similar to S. cerevisiae Pga3p; possible Kex2p substrate
PIS58351.1	27.5286494	27.13356295	27.55594071	28.03405807	27.74003253	27.88343058	0.143680338	0.752	0.48	PIS58351.1	PDK2	Putative pyruvate dehydrogenase kinase; mutation confers hypersensitivity to amphotericin B
PIS55793.1	30.29068344	30.76132479	30.15532319	30.86536661	30.78665187	30.99056689	0.164437044	0.781	0.478	PIS55793.1	MLT1	Vacuolar membrane transporter; MRP subfamily of ABC family; may transport organic anions conjugated to glutathione, glucuronate, or sulfate; needed for virulence in mouse peritonitis; Spider biofilm induced; flow model biofilm repressed
PIS58864.1	30.58971799	30.40940086	30.31468176	30.95434832	30.97085737	30.81887942	0.107145501	0.68	0.477	PIS58864.1	HGT19	Putative MFS glucose/myo-inositol transporter; 20 member family; 12 transmembrane segments, extended N terminus; expressed in rich medium; Hap43, phagocytosis, rat catheter, Spider and flow model biofilm induced
PIS54493.1	26.12419513	26.9511373	26.96456722	27.45786984	26.92059273	27.08826631	0.650166322	0.941	0.476	PIS54493.1	orf19.349	Ortholog(s) have role in aerobic respiration, mRNA metabolic process

PIS51539.1	24.26519659	24.64054862	24.50231269	23.2436888	25.81917043	25.77182724	0.526311755	0.928	0.476	PIS51539.1	SNU114	Protein similar to S. cerevisiae Snu114p, which is an RNA helicase involved in pre-mRNA splicing; likely to be essential for growth, based on an insertional mutagenesis strategy
PIS54620.1	28.10309645	27.762207	28.36921953	28.58977724	28.58534008	28.48354078	0.13030472	0.729	0.475	PIS54620.1	orf19.1383	Protein of unknown function; induced by alpha pheromone in SpiderM medium
PIS51924.1	30.30796943	29.9792535	30.59425509	30.90081032	30.65499776	30.74393429	0.223165206	0.835	0.473	PIS51924.1	HIS5	Putative histidinol-phosphate aminotransferase; Gcn4p-regulated; protein present in exponential and stationary growth phase yeast cultures
PIS49758.1	25.91433313	27.44493512	27.18790392	26.80077243	27.59917793	27.56577511	0.518139225	0.927	0.473	PIS49758.1	orf19.223	Putative serine/threonine protein kinase; Hap43-repressed; induced by prostaglandins; appears to be a downstream target of Ypk1; flow model biofilm induced; Spider biofilm induced
PIS58378.1	25.12620464	23.52925336	25.73600899	25.41397869	25.52403373	24.87256375	0.598319244	0.936	0.473	PIS58378.1	SPO7	Putative regulatory subunit of Nem1-Spo7 phosphatase holoenzyme that regulates nuclear growth by controling phospholipid biosynthesis; induced by alpha pheromone in SpiderM medium
PIS58303.1	24.7073821	24.87723687	24.45938248	25.91450999	24.12652028	25.4192591	0.554604725	0.932	0.472	PIS58303.1	orf19.3432	Predicted membrane transporter, member of the drug:proton antiporter (12 spanner) (DHA1) family, major facilitator superfamily (MFS); induced by nitric oxide
PIS56802.1	26.5517932	26.50706237	27.56462068	27.78736782	27.22852354	27.00566328	0.325515446	0.885	0.466	PIS56802.1	SAC7	Putative GTPase activating protein (GAP) for Rho1; repressed upon adherence to polystyrene; macrophage/pseudohyphal-repressed; transcript is upregulated in RHE model of oral candidiasis and in clinical oral candidiasis
PIS54725.1	23.70287626	22.11490411	24.08459742	23.34165461	23.96097758	23.99486399	0.489863776	0.923	0.465	PIS54725.1	orf19.6356	Ortholog(s) have role in mRNA splicing, via spliceosome and U4/U6 snRNP, U4/U6 x U5 tri-snRNP complex localization
PIS51406.1	30.89952349	31.37985205	30.90781297	31.61559021	31.4704981	31.49191786	0.577712786	0.934	0.464	PIS51406.1	orf19.4395	Putative actin cytoskeleton component; protein present in exponential and stationary growth phase yeast cultures
PIS58529.1	25.61223954	25.54317469	25.56784768	25.72392245	26.24360812	26.14562197	0.508760122	0.926	0.463	PIS58529.1	orf19.6455	Protein of unknown function, transcript is upregulated in clinical isolates from HIV+ patients with oral candidiasis

PIS50412.1	28.49351247	28.82884332	28.35363076	29.10441044	28.72355721	29.23017629	0.153037822	0.766	0.461	PIS50412.1	DRG1	Member of the DRG family of GTP-binding proteins; involved in regulation of invasive filamentous growth
PIS48818.1	28.90559564	28.52185719	29.16505096	29.67479854	28.9438427	29.35642688	0.157831708	0.772	0.461	PIS48818.1	NUP188	Putative nuclear pore complex subunit; transcript regulated by Nrg1 and Mig1
PIS48253.1	28.71312579	28.46839875	27.96363879	29.13806426	28.51607158	28.87087066	0.193776685	0.812	0.46	PIS48253.1	LRO1	Acyltransferase that catalyzes diacylglycerol esterification of phospholipids; role in lipid storage, triglyceride biosynthesis; flow model biofilm repressed
PIS51596.1	24.56270638	24.01391641	25.3439295	24.94492184	25.15827902	25.19683677	0.379493241	0.901	0.46	PIS51596.1	orf19.28	Putative thiamine transmembrane transporter; Spider biofilm induced
PIS59009.1	27.108222	26.71819133	27.05627579	27.28504903	27.84891797	27.12511422	0.459968141	0.918	0.459	PIS59009.1	FLC2	Protein involved in heme uptake; putative FAD transporter, similar to S. cerevisiae Flc2p
PIS55834.1	30.19201279	33.0259379	29.60335163	29.70641257	31.87026006	32.62265214	0.695465863	0.945	0.459	PIS55834.1	orf19.4216	Putative heat shock protein; decreased expression in hyphae; transcription is increased in populations of cells exposed to fluconazole over multiple generations; overexpression increases resistance to farnesol and azoles
PIS48556.1	25.04891872	25.82443287	24.17139879	25.89038344	24.90652999	25.62351494	0.413366959	0.909	0.459	PIS48556.1	orf19.4542	Ortholog(s) have cyclin-dependent protein serine/threonine kinase activator activity, cyclin-dependent protein serine/threonine kinase regulator activity
PIS55024.1	28.12697143	28.40617367	28.76905958	28.80337888	28.68104758	29.19434016	0.251272897	0.853	0.459	PIS55024.1	orf19.6071	Ortholog(s) have peptide alpha-N-acetyltransferase activity and role in N-terminal peptidyl-methionine acetylation, cytoskeleton organization, mitochondrion inheritance, regulation of actin cytoskeleton organization
PIS48587.1	30.98201712	30.45675209	31.11212357	31.496724	31.24780698	31.17952525	0.296331178	0.874	0.458	PIS48587.1	orf19.2168.3	Ortholog(s) have role in ER-dependent peroxisome organization, endoplasmic reticulum inheritance, endoplasmic reticulum organization and endoplasmic reticulum tubular network membrane organization, more
PIS50397.1	27.27496874	26.77769372	26.78466426	27.42075027	27.54137075	27.24836659	0.269396756	0.862	0.458	PIS50397.1	orf19.3810	Ortholog(s) have methylenetetrahydrofolate dehydrogenase (NAD+) activity, role in folic acid-containing compound biosynthetic process, one-carbon metabolic process, purine nucleobase biosynthetic process and cytosol localization

PIS58366.1	33.3082545	33.72448324	33.7942379	33.97183588	33.85701703	34.37300719	0.327041365	0.886	0.458	PIS58366.1	UGA1	Putative GABA transaminase; transcription regulated by Mig1 and Tup1; stationary phase enriched protein; rat catheter and Spider biofilm induced
PIS48444.1	25.24154907	24.72408839	26.36944187	24.73157155	26.14525048	26.82876581	0.57699462	0.934	0.457	PIS48444.1	orf19.1041	Ortholog(s) have cyclin-dependent protein serine/threonine kinase activator activity, cyclin-dependent protein serine/threonine kinase regulator activity
PIS51748.1	28.05381768	27.23050575	28.08009535	27.97592036	28.47531967	28.28552334	0.288270629	0.871	0.457	PIS51748.1	orf19.3804	Protein of unknown function; filament induced
PIS58881.1	30.26974001	30.03468377	29.96868032	30.54364431	30.5560213	30.53924798	0.387725006	0.903	0.455	PIS58881.1	SEC3	Predicted subunit of the exocyst complex, involved in exocytosis; required for hyphal growth after the first septin ring formation; interacts with septins Cdc3p, Cdc10p, and Cdc11p; localizes to a crescent on the surface of the hyphal tip
PIS48633.1	24.88856597	25.82333831	24.56731106	25.17635493	26.19464577	25.27075022	0.40522667	0.907	0.454	PIS48633.1	ASR3	Adenylyl cyclase and stress responsive protein; induced in cyr1 or ras1 mutant; Spider biofilm induced
PIS55464.1	25.37051356	24.97227576	25.14919894	25.29413526	26.66343049	24.89770774	0.325047817	0.885	0.454	PIS55464.1	orf19.6528	Has domain(s) with predicted role in signal transduction and Ragulator complex localization
PIS55559.1	25.16420546	27.16479305	25.92103697	26.23756921	26.57812584	26.79541894	0.5866904	0.935	0.454	PIS55559.1	orf19.7341	Protein of unknown function; flow model biofilm induced; ketoconazole-repressed
PIS52234.1	29.42369297	28.45520798	29.6629983	29.5603309	29.50411408	29.8363185	0.292292261	0.873	0.453	PIS52234.1	orf19.1994	Ortholog(s) have phosphatidylinositol binding, phosphatidylinositol-3-phosphate binding activity, role in retrograde transport, endosome to Golgi and endosome localization
PIS51034.1	24.68197422	25.04329651	24.28507493	24.81966569	25.62026186	24.93015668	0.42151784	0.911	0.453	PIS51034.1	orf19.7624	Ortholog(s) have role in maturation of SSU-rRNA from tricistronic rRNA transcript (SSU-rRNA, 5.8S rRNA, LSU-rRNA), rRNA processing and nucleolus, small-subunit processome localization
PIS48773.1	30.18310132	30.29601526	30.36112066	31.06117587	30.63029357	30.50914327	0.351391867	0.893	0.453	PIS48773.1	PIM1	ATP-dependent Lon protease; role in degradation of misfolded proteins in mitochondria, biogenesis and maintenance of mitochondria; rat catheter biofilm induced

PIS49766.1	22.7426644	22.36454386	22.59654337	22.93457892	23.05664479	23.06985188	0.676487797	0.944	0.452	PIS49766.1	orf19.3606	Ortholog of S. cereviae Sna4 vacuolar outer membrane protein that plays a role in sensitivity to NA+; induced by Mnl1 under weak acid stress
PIS52208.1	26.47763	22.82921613	25.4043896	23.35357753	25.16857222	27.54326586	0.670334929	0.943	0.451	PIS52208.1	SOF1	Putative protein with a predicted role in 40S ribosomal subunit biogenesis; rat catheter biofilm induced
PIS54934.1	25.25268568	24.89379011	23.72517078	24.06997521	25.74432541	25.41103652	0.525332718	0.928	0.451	PIS54934.1	TSC11	Protein similar to S. cerevisiae Tsc11p which is involved in sphingolipid biosynthesis; transposon mutation affects filamentous growth
PIS52328.1	27.46726227	27.28569244	27.32950839	28.28797173	27.77561756	27.36879939	0.220654181	0.833	0.45	PIS52328.1	FEN1	Putative fatty acid elongase; predicted role in sphingolipid biosynthesis; possibly an essential gene, disruptants not obtained by UAU1 method; Spider and flow model biofilm induced
PIS55762.1	25.60989299	25.64342216	25.40141587	25.85530779	26.01740093	26.13279536	0.320940328	0.884	0.45	PIS55762.1	orf19.4192.1	Ortholog of C. dubliniensis CD36 : Cd36_60590, C. parapsilosis CDC317 : CPAR2_602880, C. auris B8441 : B9J08_001867 and Candida tenuis NRRL Y-1498 : CANTEDRAFT_115034
PIS58739.1	30.51264936	31.02058543	29.9918335	30.26003995	31.31723109	31.29214607	0.494237879	0.923	0.448	PIS58739.1	orf19.1687	Ortholog of S. cerevisiae Prp43, an RNA helicase in the DEAH-box family that functions in both RNA polymerase I and polymerase II transcript metabolism; Hap43-induced gene
PIS54936.1	24.68783776	27.3106967	24.91087705	25.77915711	26.17653663	26.29394405	0.703028077	0.946	0.447	PIS54936.1	ASF1	Protein similar to S. cerevisiae Asf1p, a chromatin assembly complex component; likely to be essential for growth, based on an insertional mutagenesis strategy
PIS54542.1	25.27622993	23.22601754	26.33087177	25.76474724	25.45148948	24.95913039	0.499428858	0.924	0.447	PIS54542.1	orf19.3798	Ortholog(s) have tRNA (guanine(46)-N7)-methyltransferase activity, role in tRNA (guanine-N7)-methylation and tRNA (m7G46) methyltransferase complex localization
PIS58251.1	24.15683425	25.06779908	24.61092978	25.73307367	24.48421771	24.9580183	0.397413082	0.905	0.447	PIS58251.1	orf19.4409	Ortholog(s) have nucleoside triphosphate diphosphatase activity, ribonucleoside triphosphate phosphatase activity and role in cellular response to phosphate starvation, nucleoside triphosphate metabolic process
PIS58783.1	29.50385697	30.07926526	30.25677026	30.47517888	30.32762623	30.37609578	0.496468492	0.924	0.446	PIS58783.1	SIS1	Putative Type II HSP40 co-chaperone; macrophage/pseudohyphal-repressed; heavy metal (cadmium) stress-induced; heterozygous null mutant displays sensitivity to virgineone; rat catheter biofilm induced

PIS51253.1	27.38771873	27.10943788	27.56044294	27.79970684	28.16171541	27.43061231	0.494012999	0.923	0.445	PIS51253.1	orf19.81	Ortholog(s) have role in exonucleolytic trimming to generate mature 3'-end of 5.8S rRNA from tricistronic rRNA transcript (SSU-rRNA, 5.8S rRNA and LSU-rRNA), more
PIS54873.1	24.00646061	25.45652029	23.76037824	24.64243403	25.21256304	24.69997494	0.319157702	0.883	0.444	PIS54873.1	DUT1	dUTP pyrophosphatase; cell-cycle regulated if expressed in S. cerevisiae; upstream MluI and SCB elements; 17-beta-estradiol, ethynyl estradiol, macrophage induced; decreased in stationary phase yeast; rat catheter, Spider biofilm repressed
PIS58197.1	27.64483441	26.52228918	27.26558798	26.64735247	28.18680791	27.93137385	0.546466038	0.931	0.444	PIS58197.1	orf19.2063	Ortholog of C. dubliniensis CD36 : Cd36_15600, C. parapsilosis CDC317 : CPAR2_213140, C. auris B8441 : B9J08_000687 and Debaryomyces hansenii CBS767 : DEHA2B02442g
PIS51701.1	25.38067004	26.78638104	26.02329059	26.98336407	26.05570461	26.48000377	0.365470958	0.897	0.443	PIS51701.1	PWP1	Putative rRNA processing protein; Hap43-induced; repressed in core stress response
PIS52390.1	29.00515144	29.24815176	27.94646352	28.78503973	29.31030891	29.42993661	0.321198699	0.884	0.442	PIS52390.1	orf19.5420	Ortholog(s) have structural constituent of ribosome activity, role in mitochondrial translation and mitochondrial large ribosomal subunit localization
PIS49817.1	24.94417827	25.13248218	24.86074563	25.09558874	25.64400589	25.52132976	0.26224353	0.859	0.441	PIS49817.1	BUR2	Protein with similarity to S. cerevisiae Bur2p, contains a cyclin domain; not required for wild-type hyphal growth, adherence to buccal epithelial cells, or virulence in mouse systemic infection
PIS58984.1	23.34846973	25.21111392	23.37753769	25.44787598	23.26110043	24.55109082	0.494077962	0.923	0.441	PIS58984.1	orf19.318	Ortholog(s) have role in protein maturation by [2Fe-2S] cluster transfer, protein maturation by [4Fe-4S] cluster transfer and mitochondrial matrix localization
PIS58405.1	26.14139245	25.53950676	26.59466846	27.39365887	26.1981558	26.00812636	0.507247747	0.925	0.441	PIS58405.1	orf19.6984	Ortholog of C. dubliniensis CD36 : Cd36_85460, C. parapsilosis CDC317 : CPAR2_405760, C. auris B8441 : B9J08_000903 and Candida tenuis NRRL Y-1498 : CANTEDRAFT_116052
PIS50338.1	25.1245488	23.20697914	25.53372153	24.64658121	25.21641306	25.32314157	0.400555546	0.906	0.44	PIS50338.1	HBR1	Essential protein involved in regulation of MTL gene expression; hemoglobin-regulated inhibitor of white-opaque switching, may affect survival in host; activator of MTLalpha1 and MTLalpha2; transcript activated by hemoglobin
PIS55729.1	27.51752426	27.7065698	27.83018198	28.03148073	28.16843296	28.17278158	0.554331528	0.932	0.439	PIS55729.1	TPO4	Putative sperimidine transporter; fungal-specific (no human or murine homolog); Spider biofilm induced; promoter bound by Tec1 and Ndt80; Bcr1-repressed in RPMI a/a biofilms

PIS52102.1	22.96633393	22.32568587	23.10971891	23.54585102	23.40056566	22.76909629	0.803506968	0.952	0.438	PIS52102.1	orf19.3470	Putative flavodoxin; similar to S. cerevisiae Tyw1, an iron-sulfur protein required for synthesis of wybutosine modified tRNA; predicted Kex2p substrate; Spider biofilm induced
PIS49611.1	21.73235005	23.40435286	22.07234854	25.80401825	18.8942532	23.82219905	0.771654641	0.95	0.437	PIS49611.1	COX19	Putative cytochrome c oxidase assembly protein; Plc1-regulated; rat catheter biofilm induced
PIS58440.1	30.48912546	29.83287735	30.52976958	30.86780541	30.85566132	30.44057271	0.206887879	0.823	0.437	PIS58440.1	HGT10	Glycerol permease involved in glycerol uptake; member of the major facilitator superfamily; induced by osmotic stress, at low glucose in rich media, during cell wall regeneration; 12 membrane spans; Hap43p-induced gene
PIS48216.1	24.3746968	25.02777031	24.3071769	24.74335196	24.94477292	25.33355774	0.337722095	0.889	0.437	PIS48216.1	orf19.1430	Ortholog of C. dubliniensis CD36 : Cd36_43870, C. parapsilosis CDC317 : CPAR2_402120, Candida tropicalis MYA-3404 : CTRG_05742 and Candida albicans WO-1 : CAWG_03407
PIS55639.1	26.68817304	26.45884893	26.05246956	27.05308461	26.68729541	26.77109445	0.192097332	0.81	0.437	PIS55639.1	orf19.1658	Protein involved in filamentous growth and maintenance of cell membrane integrity; null mutant has normal growth but shows sensitivity to sodium dodecyl sulfate and reduced filamentation under hyphae-inducing agents
PIS58794.1	24.88916098	25.18980504	25.26312236	25.7845905	25.44865106	25.42080363	0.329989663	0.887	0.437	PIS58794.1	orf19.2318.1	Ortholog(s) have inositol phosphoceramide synthase regulator activity, role in inositol phosphoceramide metabolic process and Golgi membrane, inositol phosphoceramide synthase complex localization
PIS55658.1	27.61146936	27.67706816	26.97782637	27.33783183	27.9310996	28.30848069	0.599236047	0.937	0.437	PIS55658.1	SEC65	Component of the protein-targeting Signal Recognition Particle (SRP); similar to mammalian Srp19; functional homolog of S. cerevisiae Sec65; Tbf1-induced
PIS55696.1	29.03493016	28.56242919	29.19086022	29.59380107	29.00189208	29.50136891	0.529932046	0.928	0.436	PIS55696.1	orf19.3136	Ortholog(s) have transcription coactivator activity and role in carbon catabolite regulation of transcription from RNA polymerase II promoter
PIS58685.1	23.9752657	23.10260821	25.09772447	25.23569577	24.2094352	24.03900683	0.524684691	0.928	0.436	PIS58685.1	SMC2	Protein similar to S. cerevisiae Smc2p, which is a component of the condensin complex involved in mitotic chromosome condensation; induced under hydroxyurea treatment
PIS51879.1	23.54258132	25.7806963	25.03731313	23.79194403	25.53863441	26.33359535	0.576501155	0.934	0.435	PIS51879.1	orf19.3266	Ortholog of C. dubliniensis CD36 : Cd36_25930, C. parapsilosis CDC317 : CPAR2_800910, C. auris B8441 : B9J08_003483 and Candida tenuis NRRL Y-1498 : CANTEDRAFT_132053

PIS56829.1	29.13716176	29.56306368	29.13394474	29.71924525	29.64174537	29.77895509	0.146016693	0.755	0.435	PIS56829.1	PFK26	Putative 6-phosphofructo-2-kinase; protein repressed during the mating process
PIS51767.1	24.33982928	24.2174564	22.65285956	24.55161278	23.78641978	24.17353264	0.351974467	0.894	0.434	PIS51767.1	orf19.1063	Ortholog(s) have alpha-1,6-mannosyltransferase activity, mannosyltransferase activity, role in GPI anchor biosynthetic process and endoplasmic reticulum, endoplasmic reticulum membrane, mannosyltransferase complex localization
PIS52131.1	28.45521947	29.22135392	28.75984661	29.06240608	29.43519083	29.23524432	0.389305043	0.903	0.432	PIS52131.1	orf19.5321	Ortholog(s) have methylenetetrahydrofolate reductase (NAD(P)H) activity and role in methionine biosynthetic process
PIS48716.1	26.53242992	26.60871867	27.70634423	27.79573656	27.4051288	26.93515011	0.364375118	0.897	0.43	PIS48716.1	RFC2	Putative heteropentameric replication factor C subunit; periodic mRNA expression, peak at cell-cycle G1/S phase
PIS55524.1	32.10955737	32.56250383	31.9463669	32.38829047	32.82286375	32.69357613	0.305133364	0.878	0.429	PIS55524.1	ILS1	Putative isoleucyl-tRNA synthetase, the target of drugs including the cyclic beta-amino acid icofungipen/PLD-118/BAY-10-8888 and mupirocin; protein present in exponential and stationary growth phase yeast cultures
PIS50391.1	22.15096302	23.39667291	24.98446121	22.83657451	24.21908581	24.76238708	0.801640314	0.952	0.429	PIS50391.1	orf19.3826	Predicted transmembrane protein with a role in cell wall polymer composition; Plc1-regulated; Spider biofilm induced
PIS58223.1	26.82439634	27.55644711	26.76162213	27.09890181	27.62579956	27.70552546	0.373577407	0.9	0.429	PIS58223.1	orf19.7012	Ortholog(s) have structural constituent of ribosome activity and mitochondrial small ribosomal subunit localization
PIS58726.1	28.28232314	28.01247324	28.11072282	28.55604455	28.58847148	28.54659089	0.16286821	0.779	0.429	PIS58726.1	PMC1	Vacuolar calcium P-type ATPase; transcript regulated by calcineurin and fluconazole; mutant shows increased resistance to fluconazole, lithium; increased sensitivity to calcium; Spider biofilm induced
PIS54599.1	32.53590996	32.34124257	32.70828132	33.05120007	32.93345234	32.88538957	0.416530775	0.91	0.428	PIS54599.1	LYS9	Saccharopine dehydrogenase; lysine biosynthesis; soluble protein in hyphae; amphotericin B repressed; Gcn4-regulated; colony morphology-related gene regulation by Ssn6; protein present in exponential and stationary growth phase yeast
PIS55771.1	30.00027998	29.35214799	29.39980241	29.76576487	30.21017215	30.06118106	0.689023058	0.945	0.428	PIS55771.1	orf19.6747	Ortholog(s) have acid phosphatase activity and role in intracellular sterol transport

PIS58128.1	29.68359285	29.69072092	29.65808879	29.99715949	30.19471233	30.12074252	0.137161544	0.741	0.427	PIS58128.1	GGA2	Protein involved in Golgi trafficking; rat catheter and Spider biofilm repressed
PIS49601.1	28.15941049	28.17445795	28.12931352	28.59340865	28.64168965	28.50435021	0.172346239	0.79	0.425	PIS49601.1	NSA2	Putative protein constituent of 66S pre-ribosomal particles; Hap43-induced; repressed by prostaglandins
PIS56647.1	27.01779455	27.33513001	26.09420924	26.79632532	27.28379069	27.64298574	0.447164955	0.916	0.425	PIS56647.1	orf19.1534	Ortholog of S. cerevisiae Zrt3, vacuolar membrane zinc transporter; predicted Kex2 substrate; induced in oralpharyngeal candidasis; flow model biofilm induced; Spider biofilm induced
PIS55528.1	23.84214748	24.57487872	23.80677659	24.0393941	24.63858839	24.82208428	0.37121281	0.899	0.425	PIS55528.1	orf19.5532	Protein of unknown function; Spider biofilm induced
PIS52334.1	24.94886524	25.432911	24.79345272	25.22638357	25.6320571	25.58775155	0.554846835	0.932	0.424	PIS52334.1	KIS2	Scaffold protein of Snf1p complex; similar to S. cerevisiae Gal83p and Sip2p; interacts with Snf4p; interaction with Snf1p complex is regulated by carbon source, decreased on ethanol; N-terminal myristoylation; Hog1p-downregulated
PIS49682.1	31.19160661	31.55627425	31.10440008	31.50760736	31.8222451	31.79382962	0.246810387	0.85	0.424	PIS49682.1	orf19.2664	Ortholog(s) have mRNA 3'-UTR binding activity, role in DNA damage response and P-body, nuclear periphery localization
PIS51205.1	28.65003819	28.67926185	28.54309639	29.18078284	29.07617484	28.88884375	0.309384289	0.879	0.424	PIS51205.1	orf19.5684	Ortholog(s) have structural constituent of ribosome activity and mitochondrial large ribosomal subunit localization
PIS51536.1	33.11036172	33.31762799	32.59765048	33.10957176	33.69571399	33.49358278	0.213804644	0.828	0.424	PIS51536.1	RPL17B	Ribosomal protein L17; mutation confers hypersensitivity to 5-FU, tubercidin; repressed upon phagocytosis by macrophage; Hap43-induced; Spider biofilm repressed
PIS54931.1	24.50641104	23.17946949	23.8831359	25.4234118	23.66139409	23.7545446	0.492947806	0.923	0.423	PIS54931.1	TRK1	Potassium transporter; mediates K+ and Cl- influx; role in sensitivity to cationic antimicrobial peptides, not by direct uptake, possibly related to membrane permeability; contains 5' UTR intron; Spider biofilm induced
PIS49741.1	27.46740803	27.52525876	26.60466805	27.3804338	27.70639509	27.77526766	0.268067671	0.862	0.422	PIS49741.1	ECM29	Putative scaffold protein; assists in association of the proteasome core particle with the regulatory particle; ortholog of S. cerevisiae Ecm29; transposon mutation affects filamentous growth; flow model biofilm repressed

PIS54732.1	27.68562423	26.84717098	28.07352696	28.3340666	27.76059047	27.77647849	0.303248084	0.877	0.422	PIS54732.1	orf19.5012	Ortholog of C. dubliniensis CD36 : Cd36_12740, C. parapsilosis CDC317 : CPAR2_201620, C. auris B8441 : B9J08_002511 and Candida tenuis NRRL Y-1498 : CANTEDRAFT_107586
PIS54657.1	29.95661232	29.98705097	30.09332768	30.23921066	30.66286013	30.39798466	0.198541671	0.816	0.421	PIS54657.1	NUO1	NADH-ubiquinone oxidoreductase subunit with roles in mitochondrial respiratory chain complex I assembly
PIS51368.1	30.03569841	29.98735415	30.33786218	30.2694541	30.77656603	30.57792676	0.211968449	0.827	0.421	PIS51368.1	orf19.185	Ortholog(s) have structural constituent of ribosome activity, role in mitochondrial translation, mitochondrial translational initiation and mitochondrial small ribosomal subunit localization
PIS48725.1	27.08035688	26.70030006	27.14380541	27.09225449	27.52219888	27.57242515	0.257761222	0.856	0.421	PIS48725.1	orf19.7153	Putative exportin, member of the Exportin-T family; flow model biofilm repressed
PIS50414.1	23.89160892	23.45796039	23.70210783	24.87164968	23.48388428	23.95535489	0.52622406	0.928	0.42	PIS50414.1	BUD6	Protein required for Spitzenkorper formation in hyphal cells (wild-type localization of Mlc1p to the Spitzenkorper); localizes to polarisome
PIS52474.1	27.79654864	29.4654636	28.4839327	29.17290731	28.9002483	28.93378341	0.405938933	0.907	0.42	PIS52474.1	orf19.2821	Protein of unknown function; Hap43-repressed gene; repressed by nitric oxide
PIS50596.1	30.02539964	29.78023443	29.86186436	30.36536643	30.26564787	30.29671229	0.290487781	0.872	0.42	PIS50596.1	orf19.4163	Component of a complex with Ypp1p and Stt4p that is required for phosphatidylinositol-4-phosphate, PI(4)P, in plasma membrane; required for invasive growth and cell wall organization
PIS51962.1	27.70395837	28.09796168	28.2075392	28.81438215	28.06835435	28.38734609	0.529849179	0.928	0.42	PIS51962.1	orf19.6739	Ortholog(s) have phosphopentomutase activity and role in guanosine catabolic process, inosine catabolic process, purine ribonucleoside salvage
PIS58191.1	31.3078151	31.40801707	31.34106226	31.45013343	32.05848611	31.80394103	0.181497896	0.8	0.419	PIS58191.1	PMT1	Protein mannosyltransferase; required for virulence in mice and for adhesion to epithelial cells; role in hyphal growth and drug sensitivity; Als1, Sec20, Kre9, Pir1 are substrates; 1 of 5 PMT family members
PIS52233.1	28.74644368	28.3297093	29.0153703	29.4010438	28.99991797	28.94515903	0.197265808	0.815	0.418	PIS52233.1	MNN24	Alpha-1,2-mannosyltransferase; required for normal cell wall mannan content

PIS55594.1	27.67730757	28.52776319	28.73111186	29.33215738	28.02855736	28.82947297	0.292389073	0.873	0.418	PIS55594.1	orf19.6637	Predicted glycosyl hydrolase; hypoxia induced; flow model biofilm induced
PIS50420.1	28.68617887	28.69556635	28.58961582	28.74413793	29.32966883	29.14935553	0.330787687	0.887	0.417	PIS50420.1	LAG1	Putative ceramide synthase component; Hap43p-repressed gene; amphotericin B repressed
PIS54645.1	24.26486278	24.1643631	23.81295733	24.9674047	24.27277813	24.24779538	0.53392372	0.929	0.415	PIS54645.1	CDC47	Phosphorylated protein described as having role in control of cell division; RNA abundance regulated by tyrosol and cell density; merged with orf19.201 in Assembly 20; unmerged from orf19.201 in a revision of Assembly 21
PIS50390.1	28.13657019	28.0210175	28.55768805	28.81810558	28.53385088	28.6070846	0.238284672	0.845	0.415	PIS50390.1	orf19.5296	Ortholog(s) have role in mitochondrial cytochrome c oxidase assembly and mitochondrial inner membrane, mitochondrial membrane localization
PIS49813.1	27.75701572	27.63441676	27.36775434	27.96450616	28.0671218	27.97233549	0.358940544	0.896	0.415	PIS49813.1	YCP4	Flavodoxin-like protein involved in oxidative stress protection and virulence; flow model, rat catheter and Spider biofilm repressed
PIS58475.1	25.64575827	25.75478525	25.47716834	25.56543682	26.01201123	26.54323936	0.636490415	0.94	0.414	PIS58475.1	orf19.2961	Putative transcription factor with zinc finger DNA-binding motif
PIS58115.1	29.0421052	28.61195213	29.32186591	29.57300746	29.42253612	29.22204325	0.373611874	0.9	0.414	PIS58115.1	orf19.6264.3	Ortholog(s) have role in endoplasmic reticulum to Golgi vesicle-mediated transport and COPII-coated ER to Golgi transport vesicle, Golgi apparatus, endoplasmic reticulum, peroxisome localization
PIS58737.1	28.5791432	29.54548835	28.08170354	28.84292981	29.12062352	29.47861561	0.601095715	0.937	0.412	PIS58737.1	RKI1	Ortholog(s) have ribose-5-phosphate isomerase activity and role in pentose-phosphate shunt, pyridoxine biosynthetic process
PIS52092.1	23.09382442	24.62253004	23.08585044	24.19877617	23.57743348	24.26302716	0.602543154	0.937	0.412	PIS52092.1	SSU1	Protein similar to S. cerevisiae Ssu1 sulfite transport protein; Tn mutation affects filamentous growth; regulated by Gcn2 and Gcn4; induced by nitric oxide; Hap43-repressed; Spider and flow model biofilm induced
PIS58902.1	25.86238851	24.14051452	26.24903015	26.0043645	25.75315439	25.72136297	0.39423632	0.905	0.409	PIS58902.1	orf19.2412	Ortholog(s) have role in late endosome to vacuole transport via multivesicular body sorting pathway and Vps55/Vps68 complex, late endosome localization

PIS48554.1	26.59630197	26.96654105	27.28867252	27.35242077	27.05801699	27.66809356	0.439788124	0.914	0.409	PIS48554.1	RER2	Putative cis-prenyltransferase involved in dolichol synthesis; participates in endoplasmic reticulum (ER) protein sorting; flow model biofilm induced
PIS55655.1	27.81560511	28.65482515	27.75564475	28.53960747	28.36464787	28.54895844	0.359635659	0.896	0.409	PIS55655.1	SCS7	Putative ceramide hydroxylase; regulated by Nrg1; induced in high iron; fluconazole-induced; Hap43-repressed; Spider biofilm induced
PIS54766.1	24.98492963	23.276602	25.30199967	24.58608687	24.97043574	25.23095813	0.430133121	0.912	0.408	PIS54766.1	orf19.4358	Putative protein of unknown function; Hap43p-repressed gene; S. cerevisiae ortholog YDL157C localizes to mitochondria
PIS49506.1	28.23445232	28.91480111	28.34257308	28.48482385	29.09342306	29.13664718	0.368628017	0.898	0.408	PIS49506.1	orf19.5682	Ortholog(s) have nuclear import signal receptor activity, nuclear localization sequence binding, protein-containing complex binding activity
PIS58735.1	26.65292662	26.65909631	27.36517345	27.67364985	27.1525452	27.07278464	0.49011313	0.923	0.407	PIS58735.1	BZZ1	Protein similar to S. cerevisiae Bzz1p, which is an SH3 domain protein involved in the regulation of actin polymerization
PIS54608.1	25.91931872	27.91790401	23.2122701	25.97525989	26.26604922	26.02959936	0.767503278	0.95	0.407	PIS54608.1	orf19.7442	Has domain(s) with predicted mismatch base pair DNA N-glycosylase activity and role in base-excision repair, AP site formation
PIS55782.1	26.84249009	26.69559641	26.66466466	27.19915726	27.14034434	27.08459858	0.210692015	0.826	0.407	PIS55782.1	SPC98	Putative component of the microtubule-nucleating Tub4p (gamma-tubulin) complex; periodic mRNA expression, peak at cell-cycle S/G2 phase
PIS52446.1	22.9581467	24.07765532	23.77426927	24.22092733	23.96762985	23.83574415	0.615651917	0.938	0.405	PIS52446.1	orf19.4220	Predicted pyridoxal 5'-phosphate synthase; regulated by Gcn4; repressed by amino acid starvation (3-AT treatment); rat catheter biofilm repressed
PIS48250.1	26.67376913	27.09282849	26.38663668	26.98151333	27.44456007	26.93697285	0.504780026	0.925	0.403	PIS48250.1	IQG1	Actomyosin ring component at bud neck; cell-cycle regulated ser phosphorylation at CDK sites regulate association with Bni1/Bnr1, Iqg1 degradation, and ring disassembly; mutation causes cytokinetic defects; rat catheter biofilm repressed
PIS56724.1	23.15203097	23.01022499	23.63682297	24.35614595	23.67217358	22.97967444	0.42463244	0.911	0.403	PIS56724.1	orf19.7665	Ortholog(s) have role in mitochondrial cytochrome c oxidase assembly and mitochondrial inner membrane localization

PIS58105.1	24.36629612	24.77517907	24.26559895	24.55799385	25.09938531	24.95353854	0.460926798	0.918	0.401	PIS58105.1	orf19.177	Has domain(s) with predicted phosphatidylinositol binding activity
PIS56634.1	30.9141715	31.07324621	30.82519369	31.2225898	31.32535763	31.46665893	0.317879397	0.883	0.401	PIS56634.1	orf19.2582	Ortholog(s) have alpha-aminoacyl-tRNA binding, ribosomal large subunit binding, tRNA binding activity
PIS58822.1	23.0558433	22.48638086	21.93654779	23.15025623	22.42262406	23.10999946	0.441182992	0.915	0.401	PIS58822.1	SSQ1	Protein involved in intracellular sequestering of iron ion and mitochondrial iron-sulfur cluster assembly; repression leads to defects in respiratory growth, activation of autophagy, attenuated virulence
PIS51029.1	26.47791834	25.66996682	27.54753692	26.6848875	27.45973868	26.75014456	0.389687591	0.904	0.4	PIS51029.1	orf19.2938	Putative mitochondrial inner membrane protein with a predicted role in the assembly of respiratory complex III; Hap43p-repressed gene; S. cerevisiae ortholog FMP25 localizes to mitochondrion
PIS54604.1	22.90979001	22.96046223	22.75416235	22.59399407	23.51620034	23.71106715	0.569995193	0.933	0.399	PIS54604.1	OPI3	Phosphatidylethanolamine N-methyltransferase; acts in phosphatidylcholine biosynthesis; downregulation correlates with clinical development of fluconazole resistance; amphotericin B, caspofungin repressed; Hap43-induced
PIS54987.1	28.28358168	27.68529042	28.41889089	28.47728732	28.72290423	28.38366976	0.477082259	0.921	0.399	PIS54987.1	orf19.5569	Ortholog(s) have role in establishment of mitotic sister chromatid cohesion, maintenance of rDNA, mitotic sister chromatid segregation and chromosome, telomeric region, nuclear envelope localization
PIS49585.1	24.79279466	23.88337615	24.22785337	24.20688832	25.60038435	24.29429423	0.502944701	0.925	0.399	PIS49585.1	orf19.746	Ortholog(s) have phosphatidic acid binding activity, role in nuclear pore complex assembly and nuclear inner membrane localization
PIS51703.1	30.07178011	30.34028928	30.18274957	30.60154008	30.52970575	30.65721371	0.189400975	0.807	0.398	PIS51703.1	ALI1	Putative NADH-ubiquinone oxidoreductase; in detergent-resistant membrane fraction (possible lipid raft component); predicted N-terminal acetylation; nitric oxide-repressed; plasma membrane-localized; protein decreases in stationary phase
PIS51580.1	27.65746599	28.51826538	27.84236934	28.17493685	28.19927378	28.83631879	0.305973107	0.878	0.397	PIS51580.1	orf19.3706	Ortholog of C. dubliniensis CD36 : Cd36_33440, C. parapsilosis CDC317 : CPAR2_702080, C. auris B8441 : B9J08_003174 and Candida tenuis NRRL Y-1498 : CANTEDRAFT_105591
PIS51095.1	31.05240179	30.78734603	30.68229095	31.30775789	31.34790072	31.05657642	0.455849249	0.917	0.397	PIS51095.1	orf19.7511	Ortholog(s) have structural constituent of nuclear pore activity, role in nuclear pore organization, regulation of nucleocytoplasmic transport and nuclear periphery, nuclear pore, nuclear pore inner ring localization

PIS48268.1	26.35698803	26.47868163	26.37408103	27.10238952	27.07668404	26.22277242	0.48292056	0.922	0.397	PIS48268.1	TSR2	Protein with a predicted role in pre-rRNA processing; repressed by prostaglandins
PIS54479.1	24.48257773	27.40781968	23.73282974	24.21586911	26.11942751	26.47211726	0.746284183	0.949	0.395	PIS54479.1	orf19.7097	Putative cytoplasmic RNA-binding protein; heterozygous null mutant exhibits hypersensitivity to parnafungin and cordycepin in C. albicans fitness test; Spider biofilm repressed
PIS51657.1	23.47356596	23.96382613	23.47057369	24.14110214	24.03309182	23.91620735	0.291471407	0.872	0.394	PIS51657.1	orf19.2986	Ortholog of C. dubliniensis CD36 : Cd36_02750, C. parapsilosis CDC317 : CPAR2_106015, C. auris B8441 : B9J08_003254 and Candida tenuis NRRL Y-1498 : CANTEDRAFT_107500
PIS55596.1	24.10480815	25.09964286	23.67658657	24.36087855	24.62551984	25.07447894	0.444463092	0.915	0.393	PIS55596.1	orf19.6635	Ortholog of C. dubliniensis CD36 : Cd36_31170, C. parapsilosis CDC317 : CPAR2_205340, C. auris B8441 : B9J08_001700 and Candida tenuis NRRL Y-1498 : CANTEDRAFT_133521
PIS51959.1	30.37188193	31.17392191	30.4266667	30.80094224	31.17921582	31.17038314	0.324599288	0.885	0.393	PIS51959.1	orf19.6756	Ortholog(s) have small GTPase binding activity and Golgi apparatus localization
PIS48537.1	25.47255713	25.68618552	24.33830371	25.06835615	25.79167439	25.81300427	0.392887746	0.904	0.392	PIS48537.1	CPA1	Putative carbamoyl-phosphate synthase subunit; alkaline repressed; rat catheter, Spider and flow model biofilm induced
PIS58138.1	26.78652713	27.31973002	27.75421367	27.96903745	27.0502177	28.01777833	0.527234736	0.928	0.392	PIS58138.1	orf19.4467	Mitochondrial protein required for expression of mitochondrial respiratory chain complex I (NADH:ubiquinone oxidoreductase)
PIS58386.1	27.77649601	27.65082427	27.7597399	28.21558287	28.14521843	27.99949996	0.455087486	0.917	0.391	PIS58386.1	orf19.6886	Ortholog(s) have rRNA binding activity and role in maturation of LSU-rRNA from tricistronic rRNA transcript (SSU-rRNA, 5.8S rRNA, LSU-rRNA), rRNA processing, ribosomal large subunit export from nucleus
PIS51277.1	28.15303159	28.02344209	28.57101971	28.75212931	28.4601995	28.70403478	0.483094371	0.922	0.39	PIS51277.1	ERG4	Protein similar to sterol C-24 reductase; shows Mob2p-dependent hyphal regulation; fluconazole-induced; caspofungin repressed; rat catheter biofilm repressed
PIS58647.1	30.6982131	30.2372479	30.51081265	31.0603558	30.87127098	30.68416021	0.279983222	0.867	0.39	PIS58647.1	orf19.1403	Ortholog(s) have ATP:ADP antiporter activity, calcium ion binding activity and role in ADP transport, ATP transport, mitochondrial transport

PIS54642.1	30.38614116	30.46451571	30.48559213	30.71385738	30.8123393	30.97976671	0.257990228	0.856	0.39	PIS54642.1	SEC7	Putative guanine nucleotide exchange factor (GEF); mutation confers hypersensitivity to Brefeldin A; caspofungin repressed
PIS49635.1	30.16333145	30.1913689	30.14364208	30.26158059	30.69246411	30.71402005	0.530492322	0.929	0.39	PIS49635.1	UGA2	Predicted succinate semialdehyde dehydrogenase; predicted role in glutamate catabolism; transcription regulated by Mig1, Tup1, Gcn4; mutants are viable
PIS55726.1	30.69638094	29.94952697	30.69358665	31.17139332	30.83136102	30.50352929	0.738892272	0.948	0.389	PIS55726.1	orf19.2452	Protein of unknown function; induced in high iron; repressed in core caspofungin response; ketoconazole-repressed; colony morphology-related gene regulation by Ssn6; possibly subject to Kex2 processing
PIS56900.1	29.82319876	28.48152861	30.02287895	29.91728572	29.7624849	29.8158711	0.395027167	0.905	0.389	PIS56900.1	SAM51	Component of the SAM complex involved in mitochondrial protein import, involved in beta-barrel protein assembly; member of the Omp85 protein family
PIS51659.1	25.54093171	25.12831634	24.45938248	26.37513806	24.88637726	25.03121248	0.640596583	0.941	0.388	PIS51659.1	MST1	Ortholog(s) have threonine-tRNA ligase activity, role in mitochondrial threonyl-tRNA aminoacylation and mitochondrion localization
PIS58415.1	26.61183661	25.43561395	21.52253424	25.91220034	24.58205838	24.24101045	0.717911875	0.947	0.388	PIS58415.1	orf19.2914	Ortholog of C. dubliniensis CD36 : Cd36_45760, C. parapsilosis CDC317 : CPAR2_401920, C. auris B8441 : B9J08_000913 and Candida tenuis NRRL Y-1498 : CANTEDRAFT_126814
PIS51663.1	28.0398358	28.20308406	28.32699436	28.64444163	28.47376839	28.61420891	0.259204523	0.857	0.388	PIS51663.1	orf19.2978	Protein of unknown function; Hap43-repressed gene
PIS54882.1	30.24125778	29.30686447	30.55719707	30.64492733	30.37842493	30.24494121	0.618832071	0.939	0.388	PIS54882.1	orf19.4947	Mitochondrial membrane protein of unknown function; Spider biofilm induced
PIS58098.1	28.84395791	29.06126366	28.89786015	29.24539662	29.30876771	29.41262371	0.37321847	0.899	0.388	PIS58098.1	orf19.5077	Subunit of mitochondrial respiratory chain complex I; Hap43-repressed gene; repressed by nitric oxide
PIS49700.1	24.78696683	24.51934361	22.40111655	24.75275134	24.29438823	23.82312427	0.563593189	0.933	0.388	PIS49700.1	orf19.6566	Mitochondrial protein required for expression of respiratory chain complex IV (cytochrome c oxidase)

PIS54576.1	24.93466522	25.3610088	24.98696972	25.26402835	25.68979113	25.49280272	0.445284952	0.915	0.388	PIS54576.1	orf19.6929	Ortholog(s) have acid phosphatase activity, protein tyrosine phosphatase activity, metal-dependent activity
PIS56721.1	27.70668238	28.53938637	27.43365147	28.11844313	28.02588726	28.69917115	0.349491291	0.893	0.388	PIS56721.1	orf19.7662	Ortholog(s) have RNA polymerase II C-terminal domain phosphoserine binding activity, role in mRNA 3'-end processing, retrotransposon silencing and chromatin, site of double-strand break localization
PIS55692.1	26.16944432	25.92476202	26.18223925	26.25722377	27.16123155	26.01888332	0.63006791	0.94	0.387	PIS55692.1	orf19.109	Probable mitochondrial tyrosyl-tRNA synthetase, based on conservation in other fungi
PIS56667.1	25.19374328	25.84771855	26.19924104	26.469855	25.84789421	26.08392838	0.416231788	0.91	0.387	PIS56667.1	THI6	Putative thiamin-phosphate pyrophosphorylase, hydroxyethylthiazole kinase; fungal-specific; Spider biofilm induced
PIS58352.1	28.30873631	28.1240923	28.5594565	28.88813435	28.78988464	28.47127348	0.204030489	0.82	0.386	PIS58352.1	PEX13	Protein required for peroxisomal protein import mediated by PTS1 and PTS2 targeting sequences; transcript induced in an RHE model of oral candidiasis; Hap43-repressed gene
PIS56879.1	24.24469495	25.54470345	24.45648076	25.02255721	25.27606388	25.10301901	0.449009642	0.916	0.385	PIS56879.1	orf19.2682	Ortholog(s) have TBP-class protein binding, transcription coregulator activity and role in RNA polymerase II preinitiation complex assembly, transcription initiation at RNA polymerase II promoter
PIS54494.1	27.91877576	27.55918229	27.20141224	27.92852471	27.63879128	28.26611483	0.581769916	0.935	0.385	PIS54494.1	PRE9	Alpha3 (C9) subunit of the 20S proteasome; transcript regulated by Mig1; flow model biofilm repressed
PIS55689.1	26.18348395	26.98334859	27.01458136	27.77945276	26.53474015	27.01524576	0.486337137	0.922	0.383	PIS55689.1	ALG6	Putative glucosyltransferase involved in cell wall mannan biosynthesis; transcription is elevated in chk1, nik1, and sln1 homozygous null mutants; repressed by nitric oxide; possibly essential gene, disruptants not obtained by UAU1 method
PIS56887.1	29.50492609	29.38404527	29.37730962	29.55446566	29.9093057	29.95108612	0.696418063	0.945	0.383	PIS56887.1	orf19.5547	Protein of unknown function; Hap43-repressed gene
PIS56755.1	28.80908393	27.02649063	28.09751998	28.32846493	28.60642605	28.14680961	0.389957767	0.904	0.383	PIS56755.1	orf19.967	Major mitochondrial nuclease; has RNAse and DNA endo- and exonucleolytic activities; roles in mitochondrial recombination, apoptosis and maintenance of polyploidy; Spider biofilm repressed

PIS51224.1	25.65214388	25.11035126	26.10439095	25.8645686	26.25253424	25.89700867	0.718633534	0.947	0.382	PIS51224.1	OLE2	Protein with similarity to fatty acid desaturase (stearoyl-CoA desaturase); homozygous null mutant shows decreased production of prostaglandin E2
PIS51063.1	30.87364184	31.13129836	30.71779201	31.05205949	31.46382244	31.3533675	0.701395221	0.946	0.382	PIS51063.1	orf19.1619	Putative kinase subunit of RNA polymerase II carboxy-terminal domain kinase I; possibly an essential gene, disruptants not obtained by UAU1 method
PIS55071.1	26.13962176	27.76576545	26.33525399	26.43508389	26.8629511	28.08975671	0.775041686	0.951	0.382	PIS55071.1	orf19.6147	Putative histone chaperone; role in chromatin remodeling; rat catheter and Spider biofilm repressed
PIS49603.1	23.9596307	24.14694017	22.98040091	25.36970728	23.71166031	23.14738488	0.626660349	0.939	0.381	PIS49603.1	MPT5	Putative RNA-binding protein; Hap43p-repressed gene; protein induced during the mating process
PIS55807.1	30.68921443	30.72675743	31.23165496	31.55666568	31.28653325	30.94865958	0.687536802	0.944	0.381	PIS55807.1	orf19.7386	Ortholog(s) have mitochondrial ribosome binding activity and role in inner mitochondrial membrane organization, positive regulation of mitochondrial translation, protein insertion into mitochondrial inner membrane from matrix
PIS58427.1	25.57427323	26.00713299	24.45505864	26.07835476	25.49171043	25.60443767	0.483271775	0.922	0.379	PIS58427.1	orf19.2848	Predicted regulatory subunit of the Atg1 signaling complex; required for vesicle formation during autophagy, biofilm formation, and the cytoplasm-to-vacuole targeting (Cvt) pathway; Spider biofilm induced
PIS58038.1	26.43695166	24.8125039	25.87591031	26.73869901	25.7326134	25.78968339	0.444214514	0.915	0.379	PIS58038.1	PKH3	Probable serine/threonine protein kinase; appears to act redundantly with Pkh2 within the Ypk1 signaling pathway
PIS56531.1	27.00493243	25.86026841	27.1530789	27.51045549	26.8099793	26.83054149	0.584650452	0.935	0.378	PIS56531.1	orf19.5517	Similar to alcohol dehydrogenases; induced by benomyl treatment, nitric oxide; induced in core stress response; oxidative stress-induced via Cap1; Spider biofilm repressed
PIS49789.1	21.7354855	24.58911734	23.29489266	24.18346202	22.47286515	24.0967155	0.627269015	0.939	0.378	PIS49789.1	orf19.578	Ortholog(s) have GTPase activator activity and role in endocytosis, exocytosis, formin-nucleated actin cable assembly, regulation of protein localization
PIS56805.1	29.68314098	28.63997363	29.56506246	29.8233604	29.56677586	29.63302676	0.288057478	0.871	0.378	PIS56805.1	orf19.7270	Protein of unknown function; Spider biofilm repressed

PIS51528.1	29.17474467	27.87519208	29.06768249	29.40338967	29.04950948	28.79487784	0.323552973	0.885	0.377	PIS51528.1 CDS1	Protein similar to S. cerevisiae Cds1p; transposon mutation affects filamentous growth
PIS56774.1	30.51704041	30.77680504	30.69464017	30.73053766	31.03387326	31.35504676	0.256333161	0.855	0.377	PIS56774.1 orf19.2065	Ortholog(s) have allantoicase activity and role in allantoin catabolic process
PIS54761.1	27.26307106	27.08197685	27.45816553	27.73395203	27.63363231	27.56565449	0.382886665	0.902	0.377	PIS54761.1 orf19.2459	Protein of unknown function; mRNA binds to She3; Hap43 repressed gene; Spider biofilm induced
PIS58353.1	29.66132912	30.72495389	29.63900693	30.37909683	30.21675154	30.55695285	0.508113964	0.926	0.376	PIS58353.1 ASR2	Adenylyl cyclase and stress responsive protein; induced in cyr1 or ras1 mutant; stationary phase enriched protein; Spider biofilm induced
PIS48820.1	28.62076203	28.83339034	28.09920326	28.82716938	28.81511946	29.04034829	0.362108905	0.896	0.376	PIS48820.1 orf19.4811	Putative tricarboxylate carrier family protein; localized to the mitochondrial membrane
PIS51026.1	24.28949475	24.09019593	24.92660975	25.23946531	24.00642327	25.18702775	0.669019279	0.943	0.376	PIS51026.1 SCW4	Putative cell wall protein; substrate for Kex2p processing in vitro; expression regulated by white-opaque switch; alkaline repressed; possibly essential (UAU1 method); flow model biofilm induced; Spider biofilm induced
PIS48258.1	28.65698993	27.8933821	28.77288901	28.87662	28.75624323	28.81457618	0.342378733	0.891	0.375	PIS48258.1 ERG2	C-8 sterol isomerase; enzyme of ergosterol biosynthesis; converts fecosterol to episterol; mutant is hypersensitive to multiple drugs; ketoconazole-induced; flow model and Spider biofilm repressed
PIS51560.1	29.20314339	29.90665036	28.67265581	29.36750679	29.70741482	29.83028807	0.462280994	0.918	0.374	PIS51560.1 BBC1	Putative SH3-domain-containing protein
PIS50377.1	36.24049306	36.07826033	36.27286101	36.61118007	36.54003668	36.56181267	0.168457242	0.785	0.374	PIS50377.1 GDH2	Mitochondrial NAD-dependent glutamate dehydrogenase; catalyzes deamination of glutamate to alpha-ketoglutarate; fungal-specific; regulated by Nrg1p, Mig1p, Tup1p, and Gcn4p; stationary phase enriched; Spider and flow model biofilm induced
PIS51690.1	29.79727695	29.97973049	30.1087887	30.16039754	30.40624926	30.44197886	0.546989455	0.931	0.374	PIS51690.1 RTF1	Putative RNA polymerase II-associated Paf1 complex subunit; induced during the mating process

PIS51271.1	28.35015705	27.7762136	28.12595507	28.5033917	28.57690716	28.29200929	0.360307038	0.896	0.373	PIS51271.1	SCT1	Putative glycerol-3-phosphate O-acyltransferase; fungal-specific (no human or murine homolog)
PIS51280.1	35.1513052	34.88367426	35.33083432	35.47322481	35.60084065	35.40654327	0.194986529	0.813	0.372	PIS51280.1	PMA1	Plasma membrane H(+)-ATPase; highly expressed, comprises 20-40% of total plasma membrane protein; levels increase at stationary phase transition; fluconazole induced; caspofungin repressed; upregulated in RHE model; Spider biofilm repressed
PIS51159.1	26.66224484	27.39770039	27.0845575	27.04736873	27.62395008	27.58631769	0.473325673	0.92	0.371	PIS51159.1	orf19.6328	Putative protein of the mitochondrial intermembrane space; predicted role in acetate utilization and gluconeogenesis; Spider biofilm repressed
PIS54615.1	29.03412803	29.56096278	28.82332481	29.1203582	29.57404193	29.83657285	0.389308676	0.903	0.371	PIS54615.1	PMI1	Phosphomannose isomerase; cell wall biosynthesis enzyme; drug target; functional homolog of S. cerevisiae, E. coli phosphomannose isomerase; Gcn4-regulated; induced on adherence to polystyrene, phagocytosis; 3-AT, Spider biofilm
PIS48497.1	24.73366039	24.39511511	23.70916996	24.14302363	25.11328789	24.69130804	0.510642495	0.926	0.37	PIS48497.1	MFG1	Regulator of filamentous growth; required for biofilm formation, virulence; interacts with Flo8 and Mss11
PIS51575.1	29.81383096	30.59140665	29.97720257	30.59283319	30.5351402	30.36371778	0.595228166	0.936	0.37	PIS51575.1	orf19.6062.3	Mitochondrial protein; component of the mitochondrial inner membrane organizing system ; role in maintenance of crista junctions and inner membrane architecture; Spider biofilm repressed
PIS55515.1	27.11776119	27.53209673	27.73519717	27.80966362	27.90081365	27.78480162	0.376388885	0.9	0.37	PIS55515.1	PEX3	Putative peroxisomal protein involved in targeting proteins into peroxisomes; possibly an essential gene, disruptants not obtained by UAU1 method
PIS50572.1	23.34354121	24.15828476	23.2797846	23.74949141	23.9211664	24.21568264	0.270985214	0.863	0.368	PIS50572.1	MSM1	Mitochondrial methionyl-tRNA synthetase (MetRS); functionally complements methionine auxotrophy of an E. coli MetRS mutant; transcript regulated by Nrg1; flow model biofilm induced
PIS54914.1	26.46260112	29.47007466	27.54263976	28.04179103	28.01825149	28.52007745	0.658471258	0.942	0.368	PIS54914.1	orf19.1940	Ortholog(s) have 3-demethoxyubiquinol 3-hydroxylase activity, role in ubiquinone biosynthetic process and mitochondrial inner membrane, mitochondrion localization
PIS48420.1	25.09911272	23.60443288	22.86669079	24.90980013	24.0054415	23.75586974	0.499184511	0.924	0.367	PIS48420.1	orf19.5846	Putative TFIIH and nucleotide excision repair factor 3 complex subunit; possibly an essential gene, disruptants not obtained by UAU1 method

PIS55832.1	31.84359524	31.52553693	31.95189312	32.34259088	32.1794456	31.89841873	0.797612673	0.952	0.366	PIS55832.1	orf19.2352	Ortholog(s) have triglyceride lipase activity, role in cellular lipid metabolic process and mitochondrion localization
PIS58727.1	26.6350443	27.05269863	27.19126787	27.67618051	27.52714331	26.77043899	0.463135761	0.918	0.365	PIS58727.1	orf19.5397	Putative conserved oligomeric Golgi complex subunit; decreased transcription is observed upon fluphenazine treatment
PIS48733.1	28.14486728	26.8567193	28.43516239	28.33150592	28.05389045	28.14660681	0.458785066	0.918	0.365	PIS48733.1	orf19.6198.1	Ortholog(s) have lipid binding activity, role in mitochondrial fission, mitophagy and mitochondrial intermembrane space localization
PIS54724.1	25.72391888	24.40496118	24.20840778	24.87046946	24.96308436	25.59911248	0.651762192	0.942	0.365	PIS54724.1	orf19.6357	Ortholog(s) have kinetochore adaptor activity, protein-containing complex binding activity
PIS55543.1	27.51590929	26.87781223	26.97260169	27.50682777	27.36395893	27.5857217	0.686102304	0.944	0.363	PIS55543.1	orf19.2092	Putative peroxisomal cystathionine beta-lyase; Gcn4p-regulated
PIS55578.1	26.18432709	25.83241135	27.22032265	27.0961266	25.54158955	27.68950967	0.643519849	0.941	0.363	PIS55578.1	orf19.6941	Putative diacylglycerol acyltransferase; catalyzes the terminal step of triacylglycerol formation; flow model biofilm induced; Spider biofilm induced
PIS56624.1	27.66374731	29.03536612	27.97575234	28.09631829	28.80749403	28.85701939	0.678816486	0.944	0.362	PIS56624.1	ALR1	Putative transporter of divalent cations; hyphal-induced expression; rat catheter biofilm induced
PIS51713.1	29.26462746	28.63035703	29.09744609	29.24107136	29.54986042	29.28220855	0.255556373	0.855	0.36	PIS51713.1	GEA2	Putative ARF GTP/GDP exchange factor; induced in low iron; flow model biofilm repressed
PIS55044.1	28.46838029	29.74626972	27.54113928	28.28622893	29.2216449	29.32788896	0.542421298	0.93	0.36	PIS55044.1	orf19.2089	Ortholog of Nyv1, v-SNARE component of the vacuolar SNARE complex involved in vesicle fusion in S. cerevisiae; Hap43-repressed gene
PIS55455.1	26.69557151	26.59752715	26.25291023	26.85787009	27.02554362	26.74273277	0.396062773	0.905	0.36	PIS55455.1	orf19.4247	Protein of unknown function; rat catheter and Spider biofilm repressed

PIS58508.1	25.95515609	26.27550315	26.46951778	26.78084859	26.65634215	26.33659095	0.302849934	0.877	0.358	PIS58508.1	orf19.3728	Ortholog(s) have protein phosphatase 1 binding, protein phosphatase regulator activity and role in chromosome segregation
PIS58264.1	26.76096961	28.64329703	24.3318011	27.09444528	26.52350705	27.18911334	0.703516817	0.946	0.357	PIS58264.1	MGE1	Putative mitochondrial matrix cochaperone; overexpression increases resistance to fluconazole; macrophage/pseudohyphal-repressed
PIS51739.1	27.02802308	27.27458686	28.06677484	27.62205205	28.04322925	27.77460772	0.485767412	0.922	0.357	PIS51739.1	orf19.2030	Plasma membrane-associated protein; induced in cyr1 or ras1 mutant; induced by hypoxia, ketoconazole and during growth in the mouse cecum; induced in oralpharyngeal candidasis; Spider biofilm induced
PIS48254.1	28.3482794	28.64971742	28.39750578	28.31877474	29.13434151	29.01416547	0.366501252	0.898	0.357	PIS48254.1	orf19.6020	Ortholog(s) have Atg8-family ligase activity
PIS58628.1	28.78309961	28.988728	28.65722678	28.95136685	29.11648212	29.43333963	0.304439695	0.878	0.357	PIS58628.1	RTS1	Putative serine/threonine-protein phosphatase B-type regulatory subunit; transcription is regulated upon yeast-hyphal switch
PIS51926.1	27.03935076	27.4676973	26.94997805	27.15571717	27.39637135	27.97374975	0.633031247	0.94	0.356	PIS51926.1	SEC72	ER protein-translocation complex component; regulated by Gcn4p; repressed in response to amino acid starvation (3-aminotriazole treatment); sumoylation target
PIS56601.1	28.92558683	29.42309978	28.71339527	29.11942184	29.37718518	29.63481929	0.320513563	0.883	0.356	PIS56601.1	TOR1	Protein similar to TOR family phosphatidylinositol kinases; mutation confers resistance to rapamycin; involved in regulation of ribosome protein synthesis, starvation response, and adhesion
PIS52321.1	29.87909237	30.10359066	29.86949198	30.34991041	30.38767467	30.18037037	0.581823263	0.935	0.355	PIS52321.1	MRPS9	Mitochondrial ribosomal protein S9; has N-terminal mitochondrial targeting signal and an S9 consensus motif; overexpression in S. cerevisiae causes respiratory defect that is reversible upon cessation of C. albicans gene expression
PIS48596.1	25.02251335	23.34283696	23.63040593	24.26839279	24.44776061	24.3412067	0.600702101	0.937	0.354	PIS48596.1	orf19.4043	Protein with a predicted pleckstrin homology domain; induced by alpha pheromone in SpiderM medium
PIS58592.1	28.26618219	28.38754922	28.52823674	28.7830057	28.78214841	28.67901358	0.535500956	0.929	0.354	PIS58592.1	orf19.7409.1	Ortholog(s) have structural constituent of ribosome activity and mitochondrial large ribosomal subunit localization

PIS48265.1	29.77474401	29.67699659	29.84220142	29.95952025	30.34484587	30.04891861	0.40741635	0.908	0.353	PIS48265.1	CMP1	Catalytic subunit of calcineurin (Ca[2+]-calmodulin-regulated S/T protein phosphatase); required for wild-type virulence, resistance to high pH, Na(+), Li(+), Mn(2+), and fluconazole tolerance; micafungin is fungicidal to null mutant
PIS51100.1	27.27756321	26.5697784	27.20555924	27.54790452	27.13759191	27.42714048	0.495347213	0.924	0.353	PIS51100.1	RRD1	Putative peptidyl-prolyl cis/trans-isomerase; caspofungin induced
PIS58202.1	30.33843583	30.33366869	29.84985968	30.19997062	30.82482698	30.55292761	0.75491754	0.949	0.352	PIS58202.1	YRB1	Functional homolog of S. cerevisiae Yrb1p; regulates Gsp1 GTPase activity and thereby affects nucleocytoplasmic transport and cytoskeletal dynamics; transcript is not regulated by white-opaque switch or by dimorphic transition
PIS52473.1	25.41043466	25.42815975	25.14284268	25.26821323	26.28903134	25.47664169	0.39909205	0.906	0.351	PIS52473.1	orf19.2822	Essential component of the conserved oligomeric Golgi complex; role in fusion of transport vesicles to Golgi compartments; rat catheter biofilm repressed
PIS56558.1	24.27326967	24.12464061	25.05686913	25.15739141	24.75153118	24.59635476	0.772741888	0.95	0.35	PIS56558.1	CKB2	Regulatory subunit of protein kinase CK2 (casein kinase II), beta' subunit; null mutants are hypersensitive to caspofungin
PIS58060.1	25.16942279	24.30948806	25.39205532	27.83971202	24.27766427	23.80434901	0.691881892	0.945	0.35	PIS58060.1	orf19.7254	Ortholog(s) have role in mRNA metabolic process, mitochondrial translational initiation
PIS56610.1	28.11707716	26.98175231	28.42614908	28.03974026	28.67461791	27.85530032	0.645745878	0.941	0.348	PIS56610.1	orf19.1873	Protein of unknown function; Hap43-repressed gene; repressed by nitric oxide
PIS56913.1	22.30157958	23.49740562	22.13524593	22.90526575	23.10183384	22.96975017	0.585129461	0.935	0.348	PIS56913.1	orf19.3204	Ortholog of C. dubliniensis CD36 : Cd36_51610, C. parapsilosis CDC317 : CPAR2_303630, C. auris B8441 : B9J08_001459 and Candida tenuis NRRL Y-1498 : CANTEDRAFT_135125
PIS58688.1	32.01125982	32.44840151	31.21104339	31.3874875	32.73709353	32.58947993	0.482177596	0.922	0.348	PIS58688.1	RPL38	60S ribosomal ribosomal protein subunit; genes encoding cytoplasmic ribosomal subunits, translation factors, tRNA synthetases are downregulated upon phagocytosis by murine macrophage
PIS51896.1	30.32584668	29.73073246	30.71916465	30.78004506	30.60189395	30.43477626	0.331677029	0.887	0.347	PIS51896.1	orf19.2533.1	Protein with a predicted role in protein translocation from the endoplasmic reticulum

PIS54588.1	28.52883033	28.06151156	28.82989195	29.13219142	28.67040556	28.65893424	0.2542935	0.854	0.347	PIS54588.1	PAM17	Predicted component of the presequence translocase-associated import motor (PAM complex) involved in protein import into mitochondrial matrix
PIS55746.1	32.47163755	33.19253963	32.17146811	32.74479271	33.04505634	33.08646887	0.319158599	0.883	0.347	PIS55746.1	RPL15A	Putative ribosomal protein; repressed upon phagocytosis by murine macrophage; positively regulated by Tbf1; Spider biofilm repressed
PIS50570.1	27.6562859	27.52866189	27.70402147	28.12001481	28.19643681	27.61134098	0.66695585	0.943	0.346	PIS50570.1	GDE1	Glycerophosphocholine phosphodiesterase; mutation confers hypersensitivity to 5-fluorouracil (5-FU); F-12/CO2 early biofilm induced
PIS58879.1	27.34846169	29.26197279	26.26104695	26.69518703	28.39836689	28.81559348	0.638048301	0.94	0.346	PIS58879.1	orf19.2296	Predicted mucin-like protein; ketoconazole-induced; fluconazole-repressed; induced in cyr1 mutant; colony morphology-related gene regulation by Ssn6; flow model biofilm induced; Spider biofilm induced
PIS59036.1	29.08127766	28.3568492	27.57287448	28.85828905	28.28598479	28.9013377	0.581735772	0.935	0.345	PIS59036.1	PHM7	Putative transporter, possibly involved in ion homeostasis, drug tolerance, filamentous growth, virulence; fungal-specific; Hog1-repressed; repressed by 17-beta-estradiol, ethynyl estradiol; Hap43-induced; Spider biofilm induced
PIS49680.1	28.70231666	29.16401644	27.87451158	28.44301103	29.0756166	29.25496454	0.417548771	0.91	0.344	PIS49680.1	RPN8	Putative regulatory subunit of the 26S proteasome; mutation confers hypersensitivity to amphotericin B; regulated by Mig1, Gcn2 and Gcn4; Spider biofilm repressed
PIS52414.1	25.24878021	26.70344354	24.33712064	25.65716316	25.97506611	25.69053385	0.702077506	0.946	0.344	PIS52414.1	TPK1	cAMP-dependent protein kinase (PKA) catalytic subunit; isoform of Tpk2; involved in regulation of filamentation, phenotypic switching and mating; WT nuclear localization requires Bcy1; produced during stationary, not exponential growth
PIS51841.1	31.39200117	30.76820702	32.55105739	32.45871017	31.53602131	31.74628636	0.509026573	0.926	0.343	PIS51841.1	orf19.86	Putative glutathione peroxidase; induced by peroxide, exposure to neutrophils and macrophage blood fractions; repressed during infection of macrophages; Spider biofilm induced; flow model biofilm repressed
PIS58201.1	26.6880742	24.04062829	25.24985575	25.06495754	25.6905595	26.25340956	0.745007668	0.949	0.343	PIS58201.1	PHO81	Protein involved in regulation of hyphal development; required for response to farnesoic acid; possibly adherence-induced
PIS52305.1	32.6190663	33.26451312	32.1465945	32.42994351	33.29373727	33.33351354	0.406814047	0.908	0.342	PIS52305.1	RPL27A	Ribosomal protein L27; Spider biofilm repressed

PIS58377.1	30.87268224	30.98244452	30.72444428	31.07422248	31.31688079	31.21115734	0.395615566	0.905	0.341	PIS58377.1	OSM1	Putative flavoprotein subunit of fumarate reductase; soluble protein in hyphae; caspofungin repressed; stationary phase enriched protein; flow model biofilm induced; Spider biofilm repressed
PIS54889.1	25.66521809	26.2127454	25.2823066	25.78236659	25.55457872	26.84272055	0.459185815	0.918	0.34	PIS54889.1	orf19.4951	Protein of unknown function; Spider biofilm induced
PIS54812.1	30.65767768	30.26219774	30.80644986	31.01628587	30.74645899	30.98373453	0.464717988	0.919	0.34	PIS54812.1	SPS20	Peroxisomal 2,4-dienoyl-CoA reductase; stationary phase enriched protein; Spider biofilm induced
PIS52453.1	26.08834259	27.19048424	26.42939468	26.49051139	27.02028617	27.21239126	0.374840484	0.9	0.338	PIS52453.1	orf19.4340.1	Ortholog(s) have poly(U) RNA binding, splicing factor binding activity and U1 snRNP, U2 snRNP, U4/U6 x U5 tri-snRNP complex, U5 snRNP, post-mRNA release spliceosomal complex, spliceosomal complex localization
PIS48807.1	26.06009025	25.58613892	26.33399151	26.12525851	26.77597324	26.09025474	0.556358825	0.932	0.337	PIS48807.1	ATF1	Putative alcohol acetyltransferase; caspofungin repressed; expression depends on Tac1p
PIS51448.1	23.88728997	24.71035688	23.88744111	24.3028669	24.51038671	24.68363862	0.722179331	0.947	0.337	PIS51448.1	MED18	RNA polymerase II mediator complex subunit; rat catheter biofilm repressed
PIS56711.1	27.70324929	25.94685663	25.74668306	26.72536712	27.57138626	26.10700963	0.583391065	0.935	0.336	PIS56711.1	orf19.7675	Mitochondrial ribosomal protein of the large subunit; Spider biofilm repressed
PIS58123.1	29.58817597	31.33420621	28.92483186	29.09247061	30.69701951	31.05971824	0.693018089	0.945	0.334	PIS58123.1	orf19.6260	Ubiquitin-specific protease; cleaves ubiquitin from ubiquitinated proteins; Spider biofilm induced
PIS54957.1	24.50557408	24.22890916	25.03300194	25.33569651	24.98728674	24.44692358	0.699206498	0.945	0.334	PIS54957.1	orf19.7243	Deoxycytidine monophosphate (dCMP) deaminase; role in dUMP and dTMP biosynthesis; Spider biofilm repressed
PIS51732.1	26.29777118	25.76084894	26.58683426	25.89331246	26.48015306	27.2705732	0.569141987	0.933	0.333	PIS51732.1	orf19.2040	Ortholog(s) have oxidoreductase activity, acting on NAD(P)H activity, role in iron-sulfur cluster assembly, nitric oxide biosynthetic process, positive regulation of nitric oxide biosynthetic process and mitochondrion localization

PIS58114.1	29.05241568	28.72935572	29.25026257	29.33231445	29.34175891	29.35460327	0.291994664	0.872	0.332	PIS58114.1	LKH11	leukotriene A-4 hydrolase/aminopeptidase
PIS48310.1	25.54898522	25.70590678	26.62969903	26.22043531	26.34480514	26.31585931	0.497693428	0.924	0.332	PIS48310.1	orf19.6403	Ortholog(s) have adenyl-nucleotide exchange factor activity and role in SRP-dependent cotranslational protein targeting to membrane, translocation
PIS51844.1	25.82946449	26.02963748	25.78834467	26.47907106	26.44718853	25.71604654	0.661372674	0.942	0.332	PIS51844.1	orf19.867	Ortholog(s) have superoxide-generating NAD(P)H oxidase activity, role in apoptotic process, regulation of actin cytoskeleton organization and perinuclear endoplasmic reticulum localization
PIS49575.1	31.49242566	31.26459474	31.75304166	31.94322206	31.84184762	31.71986362	0.318491018	0.883	0.332	PIS49575.1	orf19.909.1	Ortholog of C. dubliniensis CD36 : Cd36_17965, C. parapsilosis CDC317 : CPAR2_211700, C. auris B8441 : B9J08_004599 and Candida tenuis NRRL Y-1498 : CANTEDRAFT_112898
PIS51434.1	24.38006562	24.83785974	23.78027699	24.11302369	24.90986227	24.96852682	0.581791307	0.935	0.331	PIS51434.1	orf19.1433	Protein of unknown function; Hap43-repressed; colony morphology-related gene regulation by Ssn6; Spider biofilm induced
PIS51305.1	28.10089088	28.90885075	28.34985817	28.68957566	28.57760564	29.08660746	0.710397243	0.946	0.331	PIS51305.1	orf19.1975	Ortholog(s) have role in mRNA splicing, via spliceosome and U4/U6 x U5 tri-snRNP complex, U5 snRNP localization
PIS51602.1	22.06640904	25.19467687	24.41688256	23.97604134	23.5553619	25.13945925	0.680861443	0.944	0.331	PIS51602.1	orf19.4801	Ortholog(s) have tRNA binding, tRNA dimethylallyltransferase activity, role in tRNA modification and cytosol, mitochondrion, nucleolus, nucleus localization
PIS48816.1	26.2871895	25.71147117	27.19738791	27.12132415	26.76838761	26.29999946	0.450901151	0.916	0.331	PIS48816.1	orf19.4805	Putative membrane protein; induced by alpha pheromone in SpiderM medium; Hap4-induced gene; Spider biofilm induced
PIS58089.1	25.74937455	25.32693915	25.51206404	25.92888017	25.94595214	25.70641185	0.238769416	0.845	0.331	PIS58089.1	orf19.6396	Putative patatin-like phospholipase; similar to S. cerevisiae Nte1p, which is predicted to be a membrane protein; antigenic during human oral infection; Hap43p-repressed gene
PIS54756.1	26.20190672	23.77395724	26.45626309	26.82580504	26.09584527	24.50388169	0.765425462	0.95	0.331	PIS54756.1	SUR2	Putative ceramide hydroxylase; predicted enzyme of sphingolipid biosynthesis; regulated by Tsa1, Tsa1B under H2O2 stress conditions; Spider and flow model biofilm induced

PIS55029.1	25.72418655	24.82200042	25.35223414	26.40992987	24.97467914	25.50619518	0.516332241	0.927	0.331	PIS55029.1	TLG2	Putative syntaxin-like t-SNARE; macrophage/pseudohyphal-repressed
PIS51129.1	27.63521714	26.9373378	27.3980339	27.4934757	27.81178554	27.65784639	0.54547602	0.93	0.331	PIS51129.1	UFE1	Protein interacting with Sec20p, possibly involved in retrograde transport between the Golgi and the endoplasmic reticulum; functional homolog of S. cerevisiae Ufe1p, which is an ER t-SNARE that mediates the retrograde traffic
PIS49667.1	27.18255872	27.14300623	27.18478335	27.66453349	27.48772275	27.34662379	0.308571655	0.879	0.33	PIS49667.1	HGT3	Putative glucose transporter of the major facilitator superfamily; the C. albicans glucose transporter family comprises 20 members; 12 probable membrane-spanning segments, extended C terminus; expressed in rich medium with 2% glucose
PIS54885.1	25.87040157	26.17849554	25.88695612	26.35158632	26.15225368	26.42268167	0.270763617	0.863	0.33	PIS54885.1	PSA2	Mannose-1-phosphate guanyltransferase; Hap43, macrophage-repressed; stationary phase enriched protein; Spider biofilm induced; rat catheter biofilm repressed
PIS51171.1	27.29873071	27.68015289	27.26056181	27.788339	27.47692337	27.96307876	0.360303892	0.896	0.33	PIS51171.1	SHY1	Cytochrome c oxidase biosynthesis protein; transcript regulated by Nrg1 and Mig1
PIS52065.1	29.02895898	28.89245964	28.83944488	29.09606605	29.39785979	29.25510846	0.490522193	0.923	0.329	PIS52065.1	orf19.6252	Ortholog(s) have RNA polymerase II complex binding, RNA polymerase II-specific DNA-binding transcription factor binding, chromatin binding activity
PIS48805.1	27.25264068	27.73102599	27.30330933	26.69419984	28.25954414	28.31648806	0.546985833	0.931	0.328	PIS48805.1	orf19.5884	Putative nucleolar preribosomal-associated protein; decreased transcription is observed upon benomyl treatment or in an azole-resistant strain that overexpresses MDR1; Spider biofilm induced
PIS58329.1	31.06087658	31.30923436	30.71544992	31.00097749	31.7345746	31.33394998	0.481014819	0.921	0.328	PIS58329.1	RPL42	Putative 60S ribosomal subunit protein; colony morphology-related gene regulation by Ssn6; Spider biofilm repressed
PIS58447.1	29.31695337	29.94093513	28.58091912	28.92076599	29.91208627	29.9889987	0.503889201	0.925	0.328	PIS58447.1	SPE7	Septin, required for wild-type invasive growth in vitro but not required for virulence in a mouse model of systemic infection; localizes to hyphal septum or bud neck; Asn-rich; aberrant gel mobility; phosphorylated in vitro by Gin4p
PIS52189.1	27.2237528	27.0697078	26.84540923	26.80989338	27.77140675	27.537523	0.550716268	0.931	0.327	PIS52189.1	orf19.1772	Ortholog of S. cerevisiae : MRX1, C. glabrata CBS138 : CAGL0J03278g, C. dubliniensis CD36 : Cd36_24130, C. parapsilosis CDC317 : CPAR2_407430 and C. auris B8441 : B9J08_003802

PIS54734.1	23.98786435	24.61061281	23.97255909	23.46412395	25.00335123	25.08602073	0.712943863	0.946	0.327	PIS54734.1	orf19.5014	Ortholog of S. cerevisiae : LCL3, C. glabrata CBS138 : CAGL0H03201g, C. dubliniensis CD36 : Cd36_12760, C. parapsilosis CDC317 : CPAR2_201640 and C. auris B8441 : B9J08_002513
PIS54996.1	30.07717103	30.10675758	30.30536194	30.61870716	30.4268703	30.42513026	0.387625572	0.903	0.327	PIS54996.1	YCF1	Putative glutathione S-conjugate transporter; MRP/CFTR-subfamily, ABC type transporter; human neutrophil-induced; oxidative stress-induced via Cap1; possible association with multidrug resistance; possibly essential; Spider biofilm induced
PIS58472.1	23.96251203	23.38762574	23.60083054	24.04866818	24.05833666	23.82219905	0.72925523	0.948	0.326	PIS58472.1	NUP159	FG-nucleoporin component of central core of the nuclear pore complex (NPC); contributes to nucleocytoplasmic transport; rat catheter biofilm repressed
PIS48583.1	28.55338959	27.5617363	29.06172736	29.19957454	28.68607513	28.26765885	0.557559683	0.932	0.325	PIS48583.1	MRP2	Protein similar to S. cerevisiae Mrp2p, which is a component of the small subunit of the mitochondrial ribosome; transposon mutation affects filamentous growth
PIS58875.1	26.75960703	24.5451149	25.63537876	27.10902996	26.25730992	24.54798908	0.719101509	0.947	0.325	PIS58875.1	NUP84	Ortholog(s) have structural constituent of nuclear pore activity
PIS51106.1	30.9080673	30.4325842	31.23580982	31.53305971	30.94897957	31.06891324	0.533254125	0.929	0.325	PIS51106.1	orf19.7531	Protein of unknown function; stationary phase enriched protein; induced upon yeast-hypha transition; benomyl or caspofungin induced; Hap43-repressed; Spider biofilm induced
PIS58470.1	31.1227196	31.2577281	31.02844113	31.14431335	31.64953696	31.58452708	0.498561758	0.924	0.323	PIS58470.1	ENA2	Putative sodium transporter; induced by ciclopirox olamine; alkaline induced by Rim101; repressed by high-level peroxide stress; induced in oral candidiasis clinical isolates; possibly essential gene; rat catheter and Spider biofilm induced
PIS49606.1	23.25014008	25.10054314	24.46701397	23.9118691	24.81470751	25.06004404	0.511584139	0.926	0.323	PIS49606.1	NUP49	Nuclear pore protein
PIS52250.1	24.34878088	24.48856323	24.64097087	24.6761996	24.95320298	24.81790405	0.374688042	0.9	0.323	PIS52250.1	orf19.1458	Ortholog(s) have tRNA-specific adenosine deaminase activity and role in tRNA modification
PIS51611.1	31.36338192	31.52221181	31.37644843	31.53929607	31.92640795	31.76523244	0.695325341	0.945	0.323	PIS51611.1	orf19.1791	Putative protein with a predicted role in 60S ribosomal subunit biogenesis; Hap43p-induced gene; ortholog of S. cerevisiae MAK11

PIS58690.1	24.96565557	25.47370971	25.25819615	25.6617033	25.10857538	25.89759395	0.643102061	0.941	0.323	PIS58690.1	orf19.2110	Ortholog(s) have arginyl-tRNA--protein transferase activity and role in protein arginylation
PIS54760.1	30.03654088	29.3668609	30.07595758	30.10067623	29.94710274	30.40067233	0.547937981	0.931	0.323	PIS54760.1	orf19.2460	Protein of unknown function; substrate for Kex2 processing in vitro; repressed by alpha pheromone in SpiderM medium; Spider biofilm induced; Bcr1-repressed in a/a RPMI biofilms
PIS55581.1	27.01688681	27.38040501	28.05182862	28.06029731	27.56868685	27.78873528	0.37911757	0.901	0.323	PIS55581.1	orf19.3353	Protein similar to a mitochondrial complex I intermediate-associated protein; fluconazole-repressed; Spider biofilm induced; rat catheter biofilm repressed
PIS51401.1	25.11753483	26.58824373	24.03631127	25.56614611	25.53680585	25.60440336	0.67075888	0.943	0.322	PIS51401.1	KTI12	Protein similar to S. cerevisiae Kti12p, which associates with Elongator complex; has a role in resistance to killer toxin; predicted Kex2p substrate; Hap43p-induced gene
PIS52379.1	25.62074452	25.71716617	23.82884229	25.56881878	24.98405414	25.58013523	0.718179868	0.947	0.322	PIS52379.1	NPR2	Putative urea transporter; induced during infection of murine kidney, compared to growth in vitro; has murine homolog
PIS58686.1	27.9111992	27.61666616	27.9580023	27.93142228	28.36049354	28.16128856	0.346408004	0.892	0.322	PIS58686.1	orf19.2113	Putative integral peroxisomal membrane protein; Hap43p-repressed gene
PIS58751.1	24.60450004	25.36546943	23.41156108	24.15740776	24.6811221	25.50906564	0.533202783	0.929	0.322	PIS58751.1	orf19.5704	Ortholog(s) have rRNA binding activity, role in RNA splicing, mitochondrial RNA processing, mitochondrial genome maintenance, positive regulation of rRNA processing, rRNA metabolic process and mitochondrion localization
PIS48225.1	29.68100077	29.12800388	29.66926632	29.86175826	29.93843963	29.64148652	0.578920014	0.934	0.321	PIS48225.1	orf19.6438	Ortholog(s) have serine C-palmitoyltransferase activity, role in sphingolipid biosynthetic process and SPOTS complex, endoplasmic reticulum localization
PIS52463.1	26.88198289	26.38210156	27.05785084	27.13073073	27.13568676	27.01530047	0.529509492	0.928	0.32	PIS52463.1	RRP9	Ribosomal protein; mutation confers resistance to 5-fluorocytosine (5-FC), 5-fluorouracil (5-FU), and tubercidin (7-deazaadenosine); physically interacts with TAP-tagged Nop1; Hap43-induced; Spider biofilm induced
PIS59033.1	31.68933154	32.39143606	31.02089821	32.07508359	31.98150488	32.00038773	0.542267636	0.93	0.318	PIS59033.1	IPP1	Putative inorganic pyrophosphatase; antigenic; soluble protein in hyphae; macrophage-induced protein; protein present in exponential and stationary phase yeast; possibly essential (UAU1 method); sumoylation target; Spider biofilm repressed

PIS50375.1	27.32930125	27.69210041	27.32355302	27.23440675	27.84912504	28.21162062	0.685369184	0.944	0.317	PIS50375.1	VRP1	Verprolin-related protein involved in actin cytoskeleton organization and polarized morphogenesis; interacts with Wal1p and Myo5p; downregulated upon adherence to polystyrene
PIS58436.1	27.80661745	27.92524701	27.5899799	27.91947325	27.93259948	28.41687068	0.534037587	0.929	0.316	PIS58436.1	NOG2	Putative nucleolar GTPase; repressed by prostaglandins; Hap43-induced, rat catheter and Spider biofilm induced
PIS49637.1	25.00711176	26.53014307	25.29728477	25.14320638	26.16253349	26.47765328	0.536584037	0.929	0.316	PIS49637.1	SWI4	Putative component of the SBF transcription complex involved in G1/S cell-cycle progression; periodic mRNA expression, peak at cell-cycle G1/S phase; predicted, conserved MBF binding sites upstream of G1/S-regulated genes
PIS48393.1	31.14921482	31.97787336	30.81829727	31.40495323	31.6969701	31.78994256	0.412359183	0.909	0.315	PIS48393.1	orf19.2489	Putative karyopherin beta; repressed by nitric oxide
PIS49570.1	28.766205	27.26146669	29.26360927	29.12039622	28.63742138	28.47840624	0.529855956	0.928	0.315	PIS49570.1	orf19.4203	Ortholog(s) have structural constituent of nuclear pore activity, role in nuclear pore organization, spindle pole body duplication and nuclear envelope, nuclear pore, nuclear pore transmembrane ring, spindle pole body localization
PIS48467.1	29.4013436	29.18000682	29.65034076	29.75526602	29.74815602	29.6736922	0.570069493	0.933	0.315	PIS48467.1	orf19.6189	Chalcone related protein family; flow model biofilm induced
PIS48601.1	24.12925602	23.46226214	23.56608665	23.84684555	23.96609531	24.29073473	0.48708823	0.922	0.315	PIS48601.1	PET127	Protein involved in maturation of mitochondrial RNA with a predicted 5'-to-3' exoribonuclease activity; member of the PD-(D/E)XK superfamily; ortholog of S. cerevisiae Pet127; Hap43-induced; rat catheter and Spider biofilm induced
PIS48494.1	31.87067032	31.99447545	31.9943985	32.29175116	32.2489249	32.26017834	0.27811864	0.866	0.314	PIS48494.1	TIF4631	Putative translation initiation factor eIF4G; overexpression causes hyperfilamentation; hyphal- and macrophage-induced; genes encoding some translation factors are downregulated upon phagocytosis by murine macrophage
PIS51762.1	29.65286859	30.29296524	28.87585573	29.4204849	30.19057281	30.15084295	0.60899892	0.938	0.313	PIS51762.1	CDC3	Septin; essential for viability; functional homolog of S. cerevisiae Cdc3p; down-regulation associated with azole resistance; macrophage/pseudohyphal-repressed; virulence-group-correlated expression; gene has intron
PIS58287.1	25.65405404	24.35210439	25.14271928	25.68653574	25.23415064	25.16806018	0.629415784	0.94	0.313	PIS58287.1	orf19.3027	Component of the RSC chromatin remodeling complex; putative DNA translocase; Spider biofilm repressed

PIS49489.1	29.74371183	29.42184712	29.7249991	29.90532792	30.00338772	29.92101399	0.213310376	0.828	0.313	PIS49489.1	SCO1	Putative copper transporter; Hap43p-repressed gene
PIS51578.1	31.15073614	30.77714104	31.01245685	31.3092874	31.37255807	31.19344442	0.533742447	0.929	0.312	PIS51578.1	GCN20	YEF3-subfamily ABC family protein, predicted not to be a transporter
PIS55836.1	23.62419314	23.90367355	24.41170782	23.76485415	24.60833821	24.50222336	0.562496158	0.933	0.312	PIS55836.1	orf19.3163	Ortholog of C. dubliniensis CD36 : Cd36_51910, C. parapsilosis CDC317 : CPAR2_101650, C. auris B8441 : B9J08_001942 and Candida tenuis NRRL Y-1498 : CANTEDRAFT_94797
PIS50510.1	25.97644906	27.28432728	27.20455875	27.05619155	27.01983292	27.32424452	0.661295188	0.942	0.312	PIS50510.1	TIP20	Protein interacting with Sec20p, possibly involved in retrograde transport between the Golgi and the endoplasmic reticulum; similar to S. cerevisiae Tip20p
PIS51742.1	29.12206912	28.27816495	29.66650969	29.56506392	29.21092133	29.22397383	0.684972744	0.944	0.311	PIS51742.1	MXR1	Putative methionine sulfoxide reductase; Plc1-regulated; induced by human neutrophils, flucytosine; macrophage regulated (gene induced, protein decreased); possibly adherence-induced; Spider biofilm induced
PIS51334.1	27.26582435	26.33765024	27.78516551	27.27138456	27.77299592	27.27621967	0.670506255	0.943	0.311	PIS51334.1	SWI6	Putative component of the MBF and SBF transcription complexes involved in G1/S cell-cycle progression; periodic mRNA expression, peak at cell-cycle G1/S phase
PIS48353.1	29.56328886	29.62979595	29.66713311	29.75781649	29.98389349	30.0521535	0.435606737	0.913	0.311	PIS48353.1	YIM1	Protein similar to protease of mitochondrial inner membrane; increased transcription is observed upon benomyl treatment; macrophage-downregulated gene
PIS48597.1	27.8570276	28.40573659	28.13643853	28.78964436	28.27360101	28.2664455	0.296538497	0.874	0.31	PIS48597.1	CHO1	Phosphatidylserine synthase; binds diacylglycerol and serine; appears to form hexamers comprising three dimers; transposon mutation affects filamentous growth; regulated by Nrg1, Tup1
PIS50556.1	25.81918172	20.98851885	26.14893489	24.85925609	24.57646045	24.44863711	0.804390739	0.952	0.309	PIS50556.1	orf19.4258	Puttaive pre-mRNA polyadenylation factor; heterozygous null mutant exhibits hypersensitivity to parnafungin and cordycepin in the C. albicans fitness test
PIS48586.1	29.54683821	29.1288034	29.63628301	29.54364556	29.89472197	29.79495159	0.489478841	0.923	0.307	PIS48586.1	APL4	Predicted gamma-adaptin, large subunit of the clathrin-associated protein (AP-1) complex; that binds clathrin and is involved in vesicle mediated transport; induced in core caspofungin response; rat catheter biofilm repressed

PIS51724.1	27.99019176	26.82921344	27.42325915	27.81623197	27.62816852	27.7199655	0.507082304	0.925	0.307	PIS51724.1	PAM16	Putative maltase; regulated by Gcn4; repressed by amino acid starvation (3-AT); rat catheter biofilm induced
PIS50410.1	30.43854222	29.96773313	30.89131946	31.02809392	30.62437103	30.5626382	0.36645279	0.898	0.306	PIS50410.1	orf19.5079.1	Ortholog of C. dubliniensis CD36 : Cd36_07590, C. parapsilosis CDC317 : CPAR2_206680, C. auris B8441 : B9J08_004229 and Candida tenuis NRRL Y-1498 : CANTEDRAFT_104385
PIS58216.1	24.90030956	25.11992567	25.04401659	25.08201732	25.62067177	25.27744037	0.445668143	0.915	0.305	PIS58216.1	VID21	Subunit of the NuA4 histone acetyltransferase complex; soluble protein in hyphae; Spider biofilm repressed
PIS51750.1	26.16146144	27.30393601	26.72107355	26.8837051	27.14447685	27.06879667	0.425398864	0.911	0.304	PIS51750.1	CWH41	Processing alpha glucosidase I, involved in N-linked protein glycosylation and assembly of cell wall beta 1,6 glucan; rat catheter biofilm repressed
PIS56729.1	26.01482669	26.04270543	24.95914387	25.80078057	26.06768771	26.06081184	0.609585224	0.938	0.304	PIS56729.1	orf19.6103	Ortholog(s) have role in ascospore formation, ascospore wall assembly, ascospore-type prospore membrane formation and cytosol, prospore membrane localization
PIS58967.1	26.97425006	27.0684144	26.13165607	27.35645974	26.93189334	26.79692303	0.76689433	0.95	0.304	PIS58967.1	PUS4	Putative pseudouridine synthase; transcript regulated by Nrg1, Mig1, and Tup1
PIS52247.1	29.3154878	28.70789076	29.55526548	29.47057292	29.52806674	29.49195541	0.36113979	0.896	0.304	PIS52247.1	SPT5	Protein similar to S. cerevisiae Spt5p transcription elongation factor; transposon mutation affects filamentous growth
PIS51456.1	26.03568439	26.40039559	24.59853591	26.20884897	26.04962411	25.68191888	0.601630252	0.937	0.302	PIS51456.1	SGT1	Putative co-chaperone protein with a predicted role in kinetochore assembly; mutation confers hypersensitivity to radicicol; sumoylation target
PIS55013.1	29.35727605	29.48053142	29.26137448	29.75944301	29.61887618	29.62528308	0.423900541	0.911	0.301	PIS55013.1	orf19.1179	Subunit of mitochondrial respiratory chain complex I; induced in high iron; possibly subject to Kex2 processing; Hap43-repressed
PIS51660.1	28.11396961	28.31447024	27.97505864	28.37509914	28.36214442	28.56743782	0.561436779	0.932	0.3	PIS51660.1	CDC73	Putative transcription elongation factor; cdc73 kap114 double transposon mutation affects filamentous growth; Spider biofilm repressed

PIS49582.1	24.61962897	25.29094405	24.72059935	24.62801389	24.91580654	25.98735233	0.495299451	0.924	0.3	PIS49582.1	orf19.4128	Ortholog of C. dubliniensis CD36 : Cd36_19390, C. parapsilosis CDC317 : CPAR2_209600, C. auris B8441 : B9J08_004606 and Candida tenuis NRRL Y-1498 : CANTEDRAFT_134010
PIS56588.1	29.55955308	29.5572512	31.00172166	29.68919307	29.77385542	31.55626836	0.567570285	0.933	0.3	PIS56588.1	SNQ2	Protein similar to S. cerevisiae Snq2p transporter; member of PDR subfamily of ABC family; transposon mutation affects filamentation; benomyl-induced transcription; detected at yeast-form cell plasma membrane by mass spec
PIS55802.1	27.87362637	27.47600331	27.71655184	28.25836873	27.95041658	27.75411359	0.374789178	0.9	0.299	PIS55802.1	MNT2	Alpha-1,2-mannosyl transferase; adds 3rd mannose in cell-wall mannoprotein biosynthesis; partially redundant with Mnt1; role in adherence and virulence; expressed in yeast and hyphae; fungal-specific; Hap43-induced; Spider biofilm induced
PIS55712.1	29.71597514	29.99366223	29.56416737	29.9347503	30.14244558	30.0933512	0.299086242	0.875	0.299	PIS55712.1	RGD1	GTPase activator protein; transcript induced in low iron; alkaline upregulated; localized to the bud emergence area in G1 phase and in the bud tip during S and G2 phases
PIS48309.1	30.56363772	31.35563222	30.52480724	30.91710917	31.29321241	31.12946916	0.616160967	0.938	0.299	PIS48309.1	RPP2A	Acidic ribosomal protein; likely role in translation elongation regulation; interacts with Rpp1B; 1 of 4 similar ribosomal proteins (Rpp1A, Rpp1B, Rpp2A, Rpp2B); CUG start codon; upstream uORFs; Tbf1-induced; Spider biofilm repressed
PIS58510.1	29.88389315	28.98786906	30.13837587	30.20210765	29.98747691	29.71447693	0.393086888	0.904	0.298	PIS58510.1	orf19.1367.1	Ortholog of C. parapsilosis CDC317 : CPAR2_407330, C. auris B8441 : B9J08_001010, Candida tenuis NRRL Y-1498 : CANTEDRAFT_114646 and Debaryomyces hansenii CBS767 : DEHA2B05654g
PIS58166.1	30.9853567	31.46261386	31.17683779	31.39400039	31.37825622	31.73948965	0.450028882	0.916	0.296	PIS58166.1	GRP2	NAD(H)-linked methylglyoxal oxidoreductase involved in regulation of methylglyoxal and pyruvate levels; regulation associated with azole resistance; induced in core stress response or by oxidative stress via Cap1, fluphenazine, benomyl
PIS49790.1	26.29252234	27.30929225	26.17076181	26.34739375	27.24200748	27.07005989	0.443336715	0.915	0.296	PIS49790.1	orf19.3183	Protein with a role in insertion of tail-anchored proteins into the ER membrane; required for efficient mating, in shmoo formation and nuclear migration in the pre-zygote of S. cerevisiae; Hap43-repressed
PIS58789.1	23.81966251	24.98940774	23.15955433	24.21168816	24.38232858	24.26172746	0.688528814	0.945	0.296	PIS58789.1	UBA4	Putative ubiquitin activating protein; Hap43-repressed; induced by prostaglandins; clade-associated gene expression
PIS55676.1	28.57413297	29.02367399	28.98104171	29.19111969	29.41034316	28.86144822	0.51812725	0.927	0.295	PIS55676.1	SEC6	Predicted subunit of the exocyst complex, involved in exocytosis; localizes to a crescent on the surface of the hyphal tip; Hap43p-repressed

PIS49581.1	29.32768863	29.48467217	29.46534465	29.59815041	29.6801466	29.87993769	0.701906482	0.946	0.294	PIS49581.1	CDC11	Septin; cell and hyphal morphology, agar-invasive growth, full virulence and kidney tissue invasion in mouse, but not kidney colonization, immunogenicity; hyphal and cell-cycle-regulated phosphorylation; rat catheter biofilm repressed
PIS52238.1	26.64387428	27.04250767	26.51819674	26.99514842	27.12847269	26.96241464	0.568645239	0.933	0.294	PIS52238.1	DCW1	Protein with predicted GPI modification; dfg5 dcw1 double mutant is inviable; not required for wild-type hyphal growth; upregulated in cyr1 mutant (yeast or hyphal form); Hap43-induced
PIS54475.1	29.774028	29.6745729	30.00277169	29.83600015	30.15234592	30.34601672	0.646375675	0.941	0.294	PIS54475.1	orf19.4779	Putative transporter; slightly similar to the Sit1p siderophore transporter; Gcn4p-regulated; fungal-specific; induced by Mnl1p under weak acid stress
PIS52011.1	26.9585151	27.14143525	24.50615087	25.08310131	26.92971641	27.47529765	0.740267534	0.948	0.294	PIS52011.1	orf19.813	Protein of unknown function; mutants are viable; Hap43-induced gene; oxidative stress-induced via Cap1; rat catheter and Spider biofilm induced
PIS55475.1	25.13260384	26.39760131	24.49841527	25.56679714	25.44950231	25.89098722	0.529790549	0.928	0.293	PIS55475.1	orf19.1163	Ortholog(s) have guanyl nucleotide binding activity, role in mitochondrial translational elongation, mitochondrial translational initiation and matrix side of mitochondrial inner membrane localization
PIS58144.1	32.19347466	32.48952701	32.12466613	32.60886231	32.56805944	32.50966693	0.277045939	0.866	0.293	PIS58144.1	UBI3	Fusion of ubiquitin with the S34 protein of the small ribosomal subunit; mRNA decreases upon heat shock, appears to be degraded; functional homolog of S. cerevisiae RPS31; Hap43-induced; Spider biofilm repressed
PIS48614.1	29.66467222	29.95905098	29.22288395	29.48065526	30.17573977	30.06617414	0.472717205	0.92	0.292	PIS48614.1	MRP17	Predicted mitochondrial ribosomal protein
PIS58745.1	28.92378097	28.56246768	29.19492361	29.49823298	29.26169582	28.79799768	0.374653459	0.9	0.292	PIS58745.1	orf19.1917	Protein of unknown function; Spider biofilm repressed
PIS48566.1	23.17584029	24.05249366	23.99837577	25.19836454	23.31089769	23.5893661	0.568380386	0.933	0.291	PIS48566.1	DPP2	Protein similar to S. cerevisiae pyrophosphate phosphatase Lpp1p; possible role in farnesol biosynthesis
PIS51261.1	26.7887647	26.27343346	26.57734156	26.62821087	26.99776506	26.8855397	0.460936068	0.918	0.291	PIS51261.1	MNR2	Putative ion transporter; fungal-specific (no human or murine homolog)

PIS48456.1	23.97146021	24.41039813	23.42207657	24.43871672	24.0774908	24.16157655	0.357022097	0.895	0.291	PIS48456.1	MRP8	Mitochondrial ribosomal protein; ortholog of S. cerevisiae Mrp8; transcript induced in hyphal form; mutant is viable; flow model and rat catheter biofilm repressed
PIS51276.1	28.52547833	28.58684209	29.69232408	29.44459585	28.70403747	29.5275224	0.710285179	0.946	0.291	PIS51276.1	SCL1	Proteasome subunit YC7alpha; protein present in exponential and stationary growth phase yeast cultures; flow model and Spider biofilm repressed
PIS48781.1	27.51812848	27.17638289	27.48945275	28.20662596	27.18908818	27.66255696	0.664767687	0.943	0.291	PIS48781.1	SNP3	Putative U6 snRNA-associated protein; transcript regulated by Mig1
PIS51307.1	29.47683057	29.14007591	28.88044386	29.22029997	29.66988783	29.47905138	0.470175797	0.92	0.291	PIS51307.1	URA4	Dihydroorotase; protein present in exponential and stationary growth phase yeast cultures
PIS55672.1	27.84387873	26.28673162	28.47353784	28.39955764	27.46434672	27.60876724	0.776531839	0.951	0.29	PIS55672.1	orf19.5455	Ortholog(s) have GTPase regulator activity, mRNA binding activity
PIS48667.1	30.33810575	30.7592285	29.56559581	29.66091532	30.97857674	30.89460866	0.493272836	0.923	0.29	PIS48667.1	YDJ1	Putative type I HSP40 co-chaperone; heavy metal (cadmium) stress-induced
PIS52008.1	32.25421543	32.16460511	32.31034622	32.23653491	32.89191442	32.4678199	0.396993832	0.905	0.289	PIS52008.1	MED15	RNA polymerase II mediator complex subunit; possibly an essential gene, disruptants not obtained by UAU1 method
PIS52434.1	29.09077623	28.39269142	28.91381967	29.21616023	28.91396995	29.13336754	0.610419734	0.938	0.289	PIS52434.1	TVP18	Putative integral membrane protein; fluconazole-induced
PIS56855.1	27.67781877	27.60660401	27.79684152	27.78132762	28.00428073	28.15977152	0.712868533	0.946	0.288	PIS56855.1	HGH1	Putative HMG1/2-related protein; transcript regulated by Mig1
PIS58713.1	28.01468514	27.47026597	27.8061572	28.13700426	28.05244026	27.96598828	0.655994212	0.942	0.288	PIS58713.1	PRP39	Putative component of the U1 snRNP; involved in splicing; Hap43-induced gene; Spider biofilm induced

PIS51626.1	28.71508532	28.14150425	29.20845473	29.14366195	28.73332563	29.04848911	0.55215465	0.931	0.287	PIS51626.1	orf19.1239	Secreted protein; exogenously expressed protein is a substrate for Kex2 processing in vitro; fluconazole-regulated; Spider biofilm induced
PIS49649.1	25.48387277	26.95484902	24.72389583	25.77351305	26.06991899	26.17944259	0.645960899	0.941	0.287	PIS49649.1	orf19.1861	BAR domain-containing protein, forms heterodimer with Rvs162p that binds liposomes in vitro; flow model biofilm induced
PIS58432.1	22.64567249	24.47635394	23.47556401	23.76626419	24.06264665	23.62974296	0.612578047	0.938	0.287	PIS58432.1	orf19.2851	Putative ornithine transport protein; localized to the mitochondrial membrane
PIS51165.1	28.64427392	28.94911291	28.64267309	28.76571806	28.97716173	29.35374823	0.437919369	0.914	0.287	PIS51165.1	orf19.4850	Ortholog of S. cerevisiae : CUB1, C. dubliniensis CD36 : Cd36_09270, C. parapsilosis CDC317 : CPAR2_803450, C. auris B8441 : B9J08_002739 and Candida tenuis NRRL Y-1498 : CANTEDRAFT_131760
PIS52331.1	28.43255749	28.98240579	28.54636933	28.85616288	29.05015871	28.91577354	0.344332775	0.891	0.287	PIS52331.1	orf19.6346	Putative nuclear export protein; Hap43p-induced gene; decreased transcription is observed in an azole-resistant strain that overexpresses MDR1
PIS56782.1	28.63184765	27.74666103	28.8912115	29.1995833	28.71532776	28.2168848	0.601263234	0.937	0.287	PIS56782.1	VTI1	Ortholog(s) have SNAP receptor activity and role in Golgi to vacuole transport, intra-Golgi vesicle-mediated transport, macroautophagy, vacuole fusion, non-autophagic, vesicle fusion
PIS58832.1	26.66269766	26.06636925	26.74923966	27.03003135	26.82531625	26.48159975	0.519522059	0.927	0.286	PIS58832.1	MAK21	Putative 66S pre-ribosomal particle subunit; mutation confers hypersensitivity to tubercidin (7-deazaadenosine)
PIS51943.1	26.63147471	26.72567169	26.46289698	26.95832037	26.93849699	26.78076576	0.529206667	0.928	0.286	PIS51943.1	MET10	Sulfite reductase; role in sulfur amino acid metabolism; induced by human whole blood or PMNs; Hog1-induced; possibly adherence-induced; flow model, Spider model, F-12/CO2 biofilm induced
PIS50540.1	26.04191559	24.5610128	25.09108436	24.8779106	25.78670897	25.88638849	0.605141538	0.937	0.286	PIS50540.1	orf19.894	Ortholog(s) have guanyl-nucleotide exchange factor activity and role in retrograde transport, endosome to Golgi
PIS58982.1	28.36727083	27.80333393	28.61872404	28.60057657	28.28171308	28.76080793	0.666988009	0.943	0.285	PIS58982.1	BUD2	GTPase activating protein (GAP) for Rsr1; negative regulator of filament branching, acts in hyphal growth guidance; required for wild-type budding pattern; induced in low iron; regulated by tyrosol and cell density

PIS51708.1	27.77433618	26.05407493	27.58132287	28.41127849	27.2727788	26.57809501	0.634963036	0.94	0.284	PIS51708.1	CSC25	Guanyl-nucleotide exchange factor; activator of Ras/adenylyl cyclase pathway; functional homolog of S. cerevisiae Cdc25p; commonly called Cdc25; transposon mutation affects filamentous growth
PIS52271.1	24.85376215	25.1660551	25.30087596	25.34282449	25.71488429	25.11585701	0.472709129	0.92	0.284	PIS52271.1	MIT1	Mannosylinositol phosphorylceramide (MIPC) synthase catalytic subunit; sphingolipid biosynthesis; fluconazole, caspofungin induced; macrophage-repressed; Spider biofilm induced
PIS56807.1	26.27905149	26.23252954	26.56158175	27.47072016	25.76166186	26.69188886	0.625827731	0.939	0.284	PIS56807.1	orf19.2313	Putative ortholog of S. cerevisiae Pex32 a peroxisomal integral membrane protein with a role in negative regulation of peroxisome size; Hap43-repressed gene
PIS51077.1	25.83062916	25.54681707	26.627275	26.52940281	26.1665775	26.16031194	0.474630204	0.92	0.284	PIS51077.1	orf19.2378	Ortholog(s) have role in vacuolar proton-transporting V-type ATPase complex assembly and endoplasmic reticulum membrane localization
PIS50423.1	23.68033904	22.54105842	23.67857413	23.36494894	23.34350588	24.0431018	0.589148821	0.935	0.284	PIS50423.1	orf19.3237	Ortholog(s) have proteasome regulatory particle binding, ubiquitin protein ligase activity and role in free ubiquitin chain polymerization, protein K29-linked ubiquitination, ubiquitin-dependent protein catabolic process
PIS56848.1	26.01902436	25.55109073	25.95099815	25.90412811	26.35021822	26.11559164	0.517922143	0.927	0.283	PIS56848.1	CYT2	Cytochrome c1 heme lyase; transcript regulated by Nrg1; induced in high iron
PIS50475.1	32.48636862	32.63323547	31.9478259	32.67767913	32.69314665	32.5454916	0.647410654	0.941	0.283	PIS50475.1	SSD1	Protein with role in resistance to host antimicrobial peptides; virulence role in murine infection; functional homolog of S. cerevisiae Ssd1p, which suppresses various mutant phenotypes; constitutively expressed and not cell-cycle regulated
PIS52096.1	28.58945831	29.08432272	28.61017586	28.86613797	29.08402266	29.17752064	0.352836794	0.894	0.281	PIS52096.1	PRD1	Putative proteinase; transcript regulated by Nrg1, Mig1, and Tup1; Hogp-induced; stationary phase enriched protein; Hap43-repressed; rat catheter biofilm repressed
PIS51111.1	28.08885131	27.18644825	28.40376888	28.1166961	28.12783437	28.27472379	0.469918098	0.92	0.28	PIS51111.1	orf19.7522	Protein with a pyridoxal phosphate-dependent transferase domain; Hap43-repressed; mutation confers hypersensitivity to amphotericin B; Spider biofilm repressed
PIS52049.1	27.9906592	27.45757082	27.9638691	28.28609143	27.87580148	28.08687281	0.56947545	0.933	0.279	PIS52049.1	PBP2	Putative RNA binding protein; transcript regulated by Nrg1, Mig1, and Tup1

PIS50544.1	28.50093494	28.10872926	29.11125244	29.02606837	28.67990138	28.8452269	0.469659139	0.92	0.277	PIS50544.1	HEM2	Putative porphobilinogen synthase; induced in high iron; protein level decrease in stationary phase cultures; Spider biofilm repressed
PIS52352.1	25.17383284	26.00843182	26.02233287	25.3128554	26.09437213	26.62448692	0.510083742	0.926	0.276	PIS52352.1	orf19.4959	Transcriptional activator recruited by the scaffold protein Rep1p when galactose is present to activate galactose-inducible genes; null mutant shows severe growth defect on galactose; Spider biofilm repressed
PIS58850.1	26.02790675	27.44323412	25.81314356	26.85526279	26.73118694	26.52552262	0.758540937	0.95	0.276	PIS58850.1	RAM1	Protein that acts in prenylation; transcription is alpha-factor induced; regulated in response to lovastatin and fluconazole; Hap43p-repressed gene
PIS51993.1	27.69060086	27.03277865	27.33597057	27.47830014	27.508139	27.89892532	0.569281066	0.933	0.275	PIS51993.1	NPR1	Predicted serine/threonine protein kinase, involved in regulation of ammonium transport; induced in core stress response; Hap43p-repressed gene
PIS58835.1	26.22478145	24.54369985	26.37170791	25.76915117	26.20172164	25.99502044	0.666043019	0.943	0.275	PIS58835.1	orf19.5897	Ortholog(s) have role in positive regulation of TORC1 signaling and GATOR2 complex, Seh1-associated complex localization
PIS49545.1	26.04041665	25.62075315	24.07500916	25.77114502	25.55667935	25.23129231	0.667290438	0.943	0.274	PIS49545.1	GIM5	Putative heterohexameric cochaperone prefoldin complex subunit; macrophage/pseudohyphal-repressed gene and macrophage-induced protein
PIS51606.1	31.08914705	31.26035481	31.20980946	30.89204856	31.73393997	31.75614964	0.668836919	0.943	0.274	PIS51606.1	orf19.4123	Putative THO complex subunit; possibly an essential gene, disruptants not obtained by UAU1 method; protein newly produced during adaptation to the serum
PIS52118.1	30.29799406	30.0421328	30.66595207	30.81772205	30.50704138	30.50193091	0.744224182	0.949	0.274	PIS52118.1	orf19.4521	S. cerevisiae ortholog Env9 has similarity to oxidoreductases and is proposed to have vacuolar functions, found in lipid particles; hyphal-induced expression
PIS58437.1	29.61787287	29.80686549	29.19622537	29.57798066	29.73529711	30.12924625	0.505795452	0.925	0.274	PIS58437.1	orf19.5621	Putative protein of unknown function; mutation confers hypersensitivity to amphotericin B; overlaps orf19.5621
PIS54484.1	29.96549974	29.74035199	29.79513311	30.30363827	30.15345444	29.86398903	0.443182015	0.915	0.273	PIS54484.1	CCC1	Manganese transporter; required for normal filamentous growth; mRNA binds She3, localized to hyphal tips; repressed by NO, alkaline pH; colony morphology-related regulation by Ssn6; regulated by Sef1, Sfu1, Hap43; Spider biofilm induced

PIS51761.1	24.11142741	24.56709225	25.49148985	24.97096541	24.45943106	25.55944618	0.63631042	0.94	0.273	PIS51761.1	orf19.1057	Ortholog of C. dubliniensis CD36 : Cd36_03980, C. parapsilosis CDC317 : CPAR2_107050, C. auris B8441 : B9J08_003358 and Candida tenuis NRRL Y-1498 : CANTEDRAFT_113999
PIS55717.1	27.56175717	27.90227585	27.2597254	27.5136026	27.99921086	28.02930536	0.494760586	0.924	0.273	PIS55717.1	orf19.2438	Ortholog(s) have structural constituent of ribosome activity and mitochondrial small ribosomal subunit localization
PIS54994.1	24.83666078	25.39735136	24.79360598	25.43379073	25.99637456	24.41417811	0.620096994	0.939	0.272	PIS54994.1	orf19.6476	Putative protein with a predicted role in exocytic transport from the Golgi; filament induced
PIS48660.1	23.57644707	23.37763647	23.98016302	23.27985087	24.00087249	24.46632124	0.844058795	0.955	0.271	PIS48660.1	EAF7	Subunit of the NuA4 histone acetyltransferase complex
PIS51421.1	26.09568372	25.77020015	26.1765207	26.27269746	26.368744	26.21354994	0.617945599	0.938	0.271	PIS51421.1	FTR1	High-affinity iron permease; required for mouse virulence, low-iron growth; iron, amphotericin B, caspofungin, ciclopirox, Hog1p, Sef1p, Sfu1p, and Hap43p regulated; complements S. cerevisiae ftr1 iron transport; Hap43p-repressed
PIS50383.1	27.97046087	27.50906664	28.22147477	28.41384474	28.07794543	28.02177908	0.665468494	0.943	0.271	PIS50383.1	orf19.2196	Ortholog(s) have K63-linked polyubiquitin modification-dependent protein binding, ubiquitin binding activity, role in rescue of stalled ribosome and cytosolic ribosome localization
PIS55837.1	29.15527293	30.18244766	28.56648903	29.46252691	29.57345798	29.6806502	0.566780623	0.933	0.271	PIS55837.1	orf19.3166	Ortholog(s) have role in cell morphogenesis, retrograde transport, endosome to Golgi, retrograde transport, vesicle recycling within Golgi and endosome, trans-Golgi network localization
PIS56602.1	27.29360473	26.22044432	27.58455533	27.41680202	27.23809639	27.25771328	0.695666783	0.945	0.271	PIS56602.1	RCL1	Putative U3-containing 90S preribosome processome complex subunit; Hap43-induced; essential; S. cerevisiae ortholog is essential; represses in core stress response;
PIS58302.1	24.43263305	25.09492746	24.40638483	25.18752283	24.55996874	24.99760816	0.537406145	0.929	0.27	PIS58302.1	orf19.4550	Predicted MFS membrane transporter, member of the drug:proton antiporter (12 spanner) (DHA1) family; flow model biofilm induced
PIS49690.1	24.61867162	24.78109946	25.16051017	25.25433215	25.1009158	25.0107384	0.641261722	0.941	0.269	PIS49690.1	orf19.1304	Ortholog(s) have role in U4 snRNA 3'-end processing, exonucleolytic trimming to generate mature 3'-end of 5.8S rRNA from tricistronic rRNA transcript (SSU-rRNA, 5.8S rRNA and LSU-rRNA), more

PIS58154.1	30.44789405	30.25691026	30.25266055	30.4482831	30.73036685	30.58443824	0.509055126	0.926	0.269	PIS58154.1	SEC18	Functional homolog of S. cerevisiae Sec18p, which acts in protein transport; conserved ATP binding site; not glycosylated
PIS51040.1	31.65960218	31.56733125	31.93301986	31.98977942	32.05678214	31.9168325	0.327009895	0.886	0.268	PIS51040.1	ATP17	Mitochondrial ATPase complex subunit; downregulated by Efg1p; flucytosine induced; caspofungin repressed
PIS56859.1	26.88490299	26.60397476	27.10342873	27.13388259	27.18281048	27.07932248	0.596582648	0.936	0.268	PIS56859.1	orf19.4574	Ortholog(s) have lysophospholipase activity, role in lipid homeostasis and lipid droplet localization
PIS51733.1	32.4212287	31.9143908	32.37225353	32.6691156	32.45942842	32.3815488	0.729920077	0.948	0.267	PIS51733.1	MSF1	Putative phenylalanine-tRNA ligase; protein level decreases in stationary phase cultures; Hap43p-repressed gene
PIS58549.1	31.027487	30.96882965	30.88198137	31.33579756	31.215236	31.1268371	0.391218938	0.904	0.267	PIS58549.1	orf19.3341	Putative tRNA-Arg synthetase; essential; genes encoding ribosomal subunits, translation factors, and tRNA synthetases are downregulated upon phagocytosis by murine macrophage; downregulated by growth in the mouse cecum
PIS48390.1	29.8190129	30.02857108	30.07713486	30.33159155	30.22134244	30.16387675	0.34945999	0.893	0.264	PIS48390.1	AUT7	Putative autophagosome protein; acts synergistically with Ysy6p to regulate unfolded protein response and mitochondrial function under ER stress; macrophage/pseudohyphal-repressed; alternatively spliced intron in 5' UTR
PIS52325.1	27.16616372	26.80431564	26.95140911	27.0052904	27.33212661	27.37703996	0.523094213	0.928	0.264	PIS52325.1	orf19.4913	Ortholog(s) have ubiquitin binding activity
PIS58031.1	29.3397478	28.88011179	28.4067578	28.74916537	29.40985261	29.25744561	0.504073159	0.925	0.263	PIS58031.1	FET31	Putative multicopper oxidase; ketoconazole/caspofungin/amphotericin B repressed; Sef1/Sfu1/Hap43 regulated; reports differ if functional homolog of ScFet3; rat catheter and Spider biofilm induced
PIS51140.1	28.60234238	29.10212855	28.39825956	28.89841614	28.87379838	29.11939153	0.5865142	0.935	0.263	PIS51140.1	orf19.5129	Ortholog of C. dubliniensis CD36 : Cd36_72910, C. parapsilosis CDC317 : CPAR2_704100, C. auris B8441 : B9J08_002713 and Candida tenuis NRRL Y-1498 : CANTEDRAFT_113193
PIS49457.1	22.64217134	25.79329569	23.4499665	23.97604134	23.5553619	25.13945925	0.76574157	0.95	0.262	PIS49457.1	CEX1	Ortholog(s) have tRNA binding activity, role in retrograde vesicle-mediated transport, Golgi to endoplasmic reticulum, tRNA export from nucleus and cytoplasm, nuclear pore localization

PIS58765.1	30.73231545	30.89668935	30.70581221	30.9439798	31.17199506	31.00398556	0.644273948	0.941	0.262	PIS58765.1	orf19.2686	Ortholog(s) have carboxypeptidase activity, role in nitrogen compound metabolic process, proteolysis involved in protein catabolic process and fungal-type vacuole lumen localization
PIS52263.1	25.06429858	24.52064774	24.16672507	24.8386685	25.38495558	24.31509277	0.669627673	0.943	0.262	PIS52263.1	REI1	Putative cytoplasmic pre-60S factor; Hap43-induced; repressed by prostaglandins
PIS48345.1	28.48866437	28.65564703	28.29065453	28.64059662	28.72148954	28.8517017	0.441713757	0.915	0.26	PIS48345.1	GYP2	Ortholog(s) have GTPase activator activity, role in activation of GTPase activity and cytoplasm, mating projection tip localization
PIS51294.1	28.57737779	29.03621864	28.60785608	29.293627	28.94299164	28.76527517	0.582994134	0.935	0.26	PIS51294.1	MUQ1	Putative choline phosphate cytidylyltransferase/phosphoethanolamine cytidylyltransferase; repressed in hyphae compared vs yeast; Hap43-repressed; flow model biofilm induced; Spider biofilm repressed
PIS50568.1	24.67612577	26.34366288	26.80010627	26.49949387	26.04089381	26.0582531	0.730676209	0.948	0.26	PIS50568.1	orf19.3938	Putative mitochondrial ribosomal protein of the small subunit; mutation confers hypersensitivity to 5-fluorocytosine (5-FC), 5-fluorouracil (5-FU), and tubercidin (7-deazaadenosine)
PIS51204.1	31.71812773	31.93696081	31.81578344	31.88924918	32.24251583	32.11772803	0.371293366	0.899	0.26	PIS51204.1	THS1	Putative threonyl-tRNA synthetase; transcript regulated by Mig1 and Tup1; repressed upon phagocytosis by murine macrophages; stationary phase enriched protein; Spider biofilm repressed
PIS52066.1	32.5581065	32.49117116	32.60155044	32.67131113	33.01207972	32.74458548	0.362879713	0.897	0.259	PIS52066.1	RPS23A	Putative ribosomal protein; repressed upon phagocytosis by murine macrophage; Spider biofilm repressed
PIS51389.1	30.06823193	30.2581177	30.48512918	30.61557155	30.55410331	30.41553609	0.447574681	0.916	0.258	PIS51389.1	MBF1	Putative transcriptional coactivator; caspofungin repressed; involved in virulence
PIS56527.1	25.59910838	25.85803647	26.28463331	26.27822691	26.03342391	26.20519707	0.517548573	0.927	0.258	PIS56527.1	orf19.3482	Ortholog(s) have NAD+ diphosphatase activity, role in NAD-cap decapping, NADH metabolic process, RNA decapping and peroxisome localization
PIS48385.1	31.65909342	32.12623112	31.03507061	31.38162904	32.16284548	32.04591978	0.49705867	0.924	0.257	PIS48385.1	UCF1	Upregulated by cAMP in filamentous growth; induced in high iron, decreased upon yeast-hypha switch; downregulation correlates with clinical fluconazole resistance; Ras1-regulated; Hap43-repressed; flow model biofilm induced

PIS58985.1	29.29209178	29.31085636	28.88558336	29.30291768	29.2441729	29.70853738	0.471619648	0.92	0.256	PIS58985.1	CTN3	Peroxisomal carnitine acetyl transferase; no obvious metabolic, hyphal, virulence defects in Ura+ strain; induced by macrophage engulfment, hyphal growth, starvation, nonfermentable carbon sources; rat catheter, Spider biofilm induced
PIS48296.1	25.44303385	28.22985403	25.05891667	26.10057415	26.32519541	27.07429365	0.68948712	0.945	0.256	PIS48296.1	PDR16	Phosphatidylinositol transfer protein; induction correlates with CDR1, CDR2 overexpression/azole resistance; fluphenazine, 17-beta-estradiol, ethynyl estradiol, NO induced; farnesol-downregulated in biofilm; rat catheter biofilm induced
PIS48457.1	33.6875189	34.04718159	33.40632991	33.5128428	34.31003744	34.08709733	0.427668508	0.912	0.256	PIS48457.1	RPL2	Putative 60S ribosomal protein L2; Hap43-induced gene; repressed in infected rabbit kidney in SC5314, but not NGY152, strain background; Spider biofilm repressed
PIS58450.1	29.10529187	28.41486851	29.35658476	29.26337435	29.25329974	29.12500193	0.805816372	0.952	0.255	PIS58450.1	AGE3	Putative ADP-ribosylation factor GTPase activating protein, functional ortholog of S. cerevisiae GCS1; mutation affects endocytosis, hyphal growth, chemical and drug resistance, and sensitivity to cell wall inhibitors
PIS54669.1	23.6970664	24.41687315	23.36690092	24.16979331	23.98716636	24.08892914	0.621602483	0.939	0.255	PIS54669.1	ASG1	Gal4p family zinc-finger transcription factor with similarity to S. cerevisiae Asg1p
PIS56938.1	29.34857824	30.44770389	29.48912736	29.89016465	29.55128699	30.61031736	0.584510745	0.935	0.255	PIS56938.1	POT1	Putative peroxisomal 3-oxoacyl CoA thiolase; transcript regulated by Nrg1 and Mig1; farnesol regulated; Hap43-repressed
PIS51888.1	26.69441517	27.6254708	26.53850421	26.86729285	27.49512257	27.25727239	0.64560551	0.941	0.254	PIS51888.1	orf19.2404	Ortholog(s) have RNA binding, ribonuclease MRP activity, ribonuclease P activity, tRNA binding activity
PIS49587.1	27.16753716	27.40761705	27.3286997	27.36172215	27.52783138	27.77499451	0.661261275	0.942	0.254	PIS49587.1	orf19.748	Ortholog(s) have RNA binding, structural constituent of nuclear pore activity
PIS58435.1	26.47687098	26.60534306	27.48847534	27.49825381	26.8012732	27.03215359	0.631060831	0.94	0.254	PIS58435.1	POP2	Component of the Ccr4-Pop2 mRNA deadenylase; heterozygous null mutant exhibits resistance to parnafungin and cordycepin in the C. albicans fitness test
PIS52060.1	25.30202969	26.21807931	25.44492376	25.84645916	26.03690272	25.84080336	0.579893203	0.934	0.253	PIS52060.1	orf19.3057	Ortholog(s) have role in mitochondrial genome maintenance

PIS56564.1	25.08210831	24.6915477	25.3020738	25.39830505	25.00325576	25.43325012	0.444676776	0.915	0.253	PIS56564.1	orf19.314	Ortholog of S. cerevisiae : STB6, C. glabrata CBS138 : CAGL0L05016g, C. dubliniensis CD36 : Cd36_83110, C. parapsilosis CDC317 : CPAR2_103210 and C. auris B8441 : B9J08_001101
PIS54603.1	27.12442211	27.50819056	27.42275415	27.12047726	27.56687246	28.12790583	0.532656098	0.929	0.253	PIS54603.1	orf19.3447	Ortholog(s) have mannosyltransferase activity, role in GPI anchor biosynthetic process, protein processing, ubiquitin-dependent ERAD pathway and endoplasmic reticulum, glycosylphosphatidylinositol-mannosyltransferase I complex localization
PIS52109.1	28.69933944	27.98488298	28.62446979	29.04697178	28.49366877	28.52607068	0.39859605	0.906	0.253	PIS52109.1	orf19.4502	Ortholog(s) have ATPase, DNA binding, TBP-class protein binding, molybdate ion transmembrane transporter activity
PIS55650.1	29.28064294	29.33757528	29.03225901	29.50489436	29.33469404	29.56986683	0.532400952	0.929	0.253	PIS55650.1	TIP120	Protein similar to human CAND1 (Cullin-Associated Nedd8-Dissociated) protein involved in regulation of SCF complexes; binds unnedddylated cullin Cdc53; mutants are viable
PIS51339.1	22.88355946	24.06372485	23.25465709	23.69050553	23.35244968	23.91542026	0.526355144	0.928	0.252	PIS51339.1	HST1	Putative histone deacetylase, involved in regulation of white-opaque switching
PIS52252.1	30.53732163	30.42156955	30.68377568	30.56713812	30.91216508	30.91913451	0.677954709	0.944	0.252	PIS52252.1	orf19.1460	Putative glutamine-dependent NAD synthetase, involved in NAD salvage pathway
PIS48533.1	24.48102899	24.49670127	25.34178207	25.32720054	25.19605193	24.55252366	0.550954147	0.931	0.252	PIS48533.1	orf19.4370	Protein of unknown function; induced by nitric oxide; oxidative stress-induced via Cap1; fungal-specific (no human or murine homolog)
PIS52266.1	25.4407387	25.96846442	25.12896863	26.15567006	25.87938724	25.26047732	0.719547076	0.947	0.252	PIS52266.1	PSF2	Ortholog(s) have role in DNA-templated DNA replication, double-strand break repair via break-induced replication, mitotic DNA replication
PIS48612.1	27.71538421	28.13868662	28.24482836	28.88409646	28.04706107	27.92074048	0.721096958	0.947	0.251	PIS48612.1	MSH2	Putative DNA mismatch repair factor; transcript regulated by Nrg1; flucytosine repressed; transcript regulated by tyrosol and cell density
PIS58932.1	28.29634081	28.44375806	28.81964885	28.89700069	28.74997164	28.66435342	0.742588457	0.948	0.251	PIS58932.1	NAT2	Putative N-terminal acetyltransferase; Hap43p-repressed gene; mutation confers hypersensitivity to toxic ergosterol analog

PIS54654.1	28.35129948	29.22665632	28.56167521	29.02675359	29.05096101	28.81091286	0.447950398	0.916	0.25	PIS54654.1	DFG10	Predicted polyprenol reductase with role in dolichol biosynthesis and dolichol-linked oligosaccharide biosynthetic process
PIS52071.1	27.98616522	28.28736079	28.04086818	28.47993294	28.10361964	28.48087846	0.510290174	0.926	0.25	PIS52071.1	orf19.3508	Putative protein of unknown function; stationary phase enriched protein
PIS51019.1	24.06698655	25.19804442	24.16943831	24.72923494	24.70689876	24.74869106	0.520635572	0.927	0.25	PIS51019.1	orf19.6076	Ortholog(s) have cargo adaptor activity, role in intracellular protein transport, retrograde transport, endosome to Golgi and endosome, retromer complex, retromer, cargo-selective complex localization
PIS49632.1	29.99246444	29.70443094	29.88947921	30.1406393	29.9394192	30.25269823	0.500165186	0.924	0.249	PIS49632.1	orf19.7288	Protein with predicted oxidoreductase and dehydrogenase domains; Hap43-repressed; Spider biofilm induced
PIS58099.1	24.41692553	26.32856421	23.54451668	25.43561121	24.35623745	25.24466769	0.777359059	0.951	0.249	PIS58099.1	PFY1	Profilin; functional homolog of S. cerevisiae Pfy1; hyphae, macrophage/pseudohyphal-induced; regulated by Nrg1, Tup1; gene lacks intron (unlike S. cerevisiae PFY1); complements growth of S. cerevisiae srv2 mutant; nonessential
PIS51413.1	29.37113095	29.13333121	29.50053609	29.62117818	29.56939175	29.55904686	0.48264178	0.922	0.248	PIS51413.1	ADE1	Phosphoribosylaminoimadazole succinocarboxamide synthetase, enzyme of adenine biosynthesis; not induced in GCN response, unlike the S. cerevisiae ortholog; fungal-specific (no human or murine homolog); levels decrease in stationary phase
PIS52210.1	26.90119984	26.57756255	26.71278355	27.26847257	27.04209254	26.62399428	0.780045169	0.951	0.248	PIS52210.1	IST1	Protein with a positive role in the multivesicular body sorting pathway; rat catheter biofilm repressed
PIS50337.1	29.95277655	30.02019516	30.4037826	30.44678529	30.33794189	30.33423021	0.500387231	0.924	0.247	PIS50337.1	HMX1	Heme oxygenase; utilization of hemin iron; transcript induced by heat, low iron, or hemin; repressed by Efg1; induced by low iron; upregulated by Rim101 at pH 8; Hap43-induced; Spider and flow model biofilm induced
PIS56606.1	30.90755469	30.68067585	31.08786486	31.01128346	31.19238225	31.21137102	0.434170144	0.913	0.246	PIS56606.1	HEM15	Putative ferrochelatase involved in heme biosynthesis; transcript not regulated by iron levels and not affected by a yfh1 null mutation; Spider biofilm repressed
PIS58341.1	29.225306	28.69732511	29.00449568	29.06733991	29.33355355	29.26531358	0.460581934	0.918	0.246	PIS58341.1	PMR1	Secretory pathway P-type Ca2+/Mn2+-ATPase; calcium pump involved in control of calcium homeostasis; required for protein glycosylation and cell wall maintenance; required for hyphal tip oscillation in semisolid substrate

PIS56691.1	30.53204217	30.13330684	30.55315626	30.60621297	30.74774062	30.60174998	0.497768098	0.924	0.246	PIS56691.1	TPD3	Subunit of protein serine/threonine phosphatase PPA2, involved in regulation of cytokinesis and morphogenesis; forms complex with catalytic subunit Pph21p that dephosphorylates septin Sep7p
PIS48553.1	29.4971781	29.21593024	29.82424912	30.06990513	29.57507945	29.62614438	0.407332331	0.908	0.245	PIS48553.1	HIS1	ATP phosphoribosyl transferase; enzyme of histidine biosynthesis; acid upregulated/alkaline repressed by Rim101; regulated by Gcn2, Gcn4; strain CA9 is a his1 mutant; flow model biofilm induced; Spider biofilm repressed
PIS49612.1	31.49850506	31.42717987	31.349676	31.57174616	31.62652093	31.81304902	0.585233225	0.935	0.245	PIS49612.1	KEM1	5'->3' exoribonuclease of cytoplasmic stress granules; role in filamentous growth; complements slow growth/mating of S. cerevisiae kem1 mutant; required for SD or Spider medium biofilm formation
PIS49677.1	30.92752548	30.82283603	30.75370314	31.01188533	31.09357658	31.12976604	0.503930651	0.925	0.244	PIS49677.1	ACH1	Acetyl-coA hydrolase; acetate utilization; nonessential; soluble protein in hyphae; antigenic in human; induced on polystyrene adherence; farnesol-, ketoconazole-induced; no human or murine homolog; stationary phase-enriched protein
PIS51725.1	24.54896315	24.297127	24.87385364	24.76644908	24.89970307	24.78494503	0.690545462	0.945	0.244	PIS51725.1	SET3	NAD-dependent histone deacetylase; mutations affect filamentous growth; genetic evidence suggests Set3/Hos2 function as a complex to regulate white-opaque switching, morphogenesis, and virulence; flow model biofilm induced
PIS54850.1	24.26970547	24.28408115	23.09945907	24.14376095	24.35607958	23.88147556	0.763674557	0.95	0.243	PIS54850.1	KTR4	Mannosyltransferase; induced during cell wall regeneration; fungal-specific (no human or murine homolog); Bcr1-repressed in RPMI a/a biofilms
PIS51332.1	25.55279519	27.2428357	25.66269338	26.34663179	26.87376268	25.96725957	0.778880784	0.951	0.243	PIS51332.1	orf19.4727	Ortholog(s) have role in mitochondrial respiratory chain complex II assembly, protein flavinylation, tricarboxylic acid cycle and mitochondrial matrix localization
PIS51540.1	24.62189219	26.0164113	24.27927098	25.31490981	25.27316293	25.0573726	0.688338512	0.945	0.243	PIS51540.1	RPB4	Protein similar to S. cerevisiae Rpb4p, which is a component of RNA polymerase II; transposon mutation affects filamentous growth
PIS48372.1	31.80408256	32.71435206	32.18387253	32.29717682	32.38686957	32.74493078	0.596609902	0.936	0.242	PIS48372.1	orf19.5169	Ortholog of C. dubliniensis CD36 : Cd36_72580, C. parapsilosis CDC317 : CPAR2_703580, C. auris B8441 : B9J08_005062 and Candida tenuis NRRL Y-1498 : CANTEDRAFT_114495
PIS51649.1	30.09831437	29.62227013	30.12883289	30.08981823	30.30154163	30.18310167	0.617907356	0.938	0.242	PIS51649.1	orf19.6035	Protein of unknown function; repressed by nitric oxide

PIS58073.1	28.48864287	29.14967227	28.87549271	29.04221626	28.80069488	29.39796077	0.677732064	0.944	0.242	PIS58073.1	orf19.6596	Putative esterase; possibly transcriptionally regulated by Tac1; induced by Mnl1 under weak acid stress; protein present in exponential and stationary growth phase yeast cultures; Spider biofilm repressed
PIS58814.1	29.5588278	29.41348808	29.13389549	29.72117107	29.16498789	29.94275134	0.553380795	0.931	0.241	PIS58814.1	GCF1	HMG box mitochondrial protein; binds to mt DNA and the HWP1 promoter; mutant phenotype and functional complementation of an S. cerevisiae abf2 mutation suggest role in mt genome replication, maintenance; flow and Spider biofilm repressed
PIS58981.1	28.8845689	28.69734017	29.02765763	29.06669838	29.14914611	29.11329314	0.498937536	0.924	0.24	PIS58981.1	NAM7	Putative role in nonsense-mediated mRNA decay; similar to S. cerevisiae Nam7p; gene induced by ciclopirox olamine treatment
PIS56956.1	30.86333634	30.84517537	31.20977903	31.41731245	31.12315046	31.09931324	0.464729264	0.919	0.24	PIS56956.1	orf19.5369	Uroporphyrinogen decarboxylase; catalyzes the 5th step in the heme biosynthetic pathway; flow model and Spider biofilm repressed
PIS56719.1	27.01187486	27.54645981	25.73477176	26.81516748	26.56136043	27.63648573	0.724472935	0.947	0.24	PIS56719.1	VPS52	Subunit of GARP (Golgi-associated retrograde protein) complex, which has roles in Golgi to vacuole transport, ascospore wall assembly, cellular sphingolipid homeostasis; required for filamentous growth
PIS56623.1	29.30786017	30.54137786	29.09390703	29.53278206	29.95134379	30.176916	0.67886214	0.944	0.239	PIS56623.1	HEM13	Coproporphyrinogen III oxidase; antigenic; on yeast cell surface, not hyphae; iron-regulated expression; Hap43, macrophage-repressed; farnesol-induced; possibly essential; flow model biofilm induced; rat catheter, Spider biofilm repressed
PIS56704.1	26.10944053	25.02260399	26.17843638	26.48035712	25.76538752	25.7823327	0.509989091	0.926	0.239	PIS56704.1	orf19.7627	Ortholog of S. cerevisiae : YNL320W, C. glabrata CBS138 : CAGL0M04125g, C. dubliniensis CD36 : Cd36_35340, C. parapsilosis CDC317 : CPAR2_200350 and C. auris B8441 : B9J08_001244
PIS51038.1	27.81494109	30.15927974	28.16548665	27.54884994	29.22974027	30.07634319	0.737230815	0.948	0.238	PIS51038.1	TPM2	Putative tropomyosin isoform 2; regulated by Gcn4; repressed by amino acid starvation; macrophage-induced; protein levels decrease in stationary cells; Hap43-induced; rat catheter and Spider biofilm repressed
PIS51235.1	29.86887611	29.86119273	29.76070197	29.81538291	30.28677375	30.09890085	0.419195166	0.91	0.237	PIS51235.1	orf19.1967	Ortholog(s) have structural constituent of ribosome activity and mitochondrial large ribosomal subunit localization
PIS55668.1	28.85674896	28.67447256	29.09932141	28.77486444	29.36324451	29.20474925	0.560580876	0.932	0.237	PIS55668.1	PRS1	Phosphoribosylpyrophosphate synthetase; enzyme of purine, pyrimidine, histidine, and tryptophan biosynthesis; essential; flucytosine induced; macrophage/pseudohyphal-induced

PIS50333.1	32.84283963	32.2314122	32.15222946	32.92183179	32.25693988	32.75538493	0.563402192	0.933	0.236	PIS50333.1	OP4	Ala- Leu- and Ser-rich protein; secreted; N-terminal hydrophobic region; possible glycosylation; opaque-specific transcript; repressed by alpha pheromone in opaque MTLa homozygotes; fluconazole-induced; Spider biofilm induced
PIS54533.1	28.44056163	28.83544232	27.97312483	28.57153499	28.62654801	28.75915876	0.55324478	0.931	0.236	PIS54533.1	orf19.4796	Putative eIF-4E-binding repressor of CAP-dependent translation; stationary phase enriched protein
PIS51121.1	27.84654963	27.26835982	28.1208045	27.98710616	27.9170103	28.03923954	0.497624469	0.924	0.236	PIS51121.1	orf19.639.1	Ortholog(s) have structural constituent of ribosome activity and mitochondrial large ribosomal subunit localization
PIS48142.1	26.92748101	27.14161605	26.12344489	27.15721539	26.50392124	27.23997492	0.64328256	0.941	0.236	PIS48142.1	SHA3	Putative ser/thr kinase involved in glucose transport; Tn mutation affects filamentous growth; fluconazole-induced; ketoconazole-repressed; induced in by alpha pheromone in SpiderM; possibly essential; flow model biofilm induced
PIS58625.1	31.77808109	32.20821334	30.8610788	31.57441088	31.83820407	32.13971773	0.537462842	0.929	0.235	PIS58625.1	LSP1	Eisosome component with a predicted role in endocytosis; protein present in exponential and stationary growth phase yeast cultures; caspofungin repressed; biofilm induced; fungal-specific (no human/murine homolog); sumoylation target
PIS50413.1	30.22816992	30.16256265	30.49565547	30.6446738	30.48974949	30.45815626	0.565165316	0.933	0.235	PIS50413.1	orf19.5085	Ortholog(s) have nuclear import signal receptor activity, nuclear localization sequence binding activity
PIS51900.1	26.97335423	27.52089264	27.71691354	27.74625849	27.99895197	27.16721374	0.708340202	0.946	0.234	PIS51900.1	orf19.2541	Ortholog(s) have 3'-5'-DNA exonuclease activity, endonuclease activity and role in apoptotic DNA fragmentation, cellular response to oxidative stress
PIS52221.1	28.99284878	28.85201922	29.11378644	29.39347258	29.15154584	29.11216576	0.363301979	0.897	0.233	PIS52221.1	NAG6	Protein required for wild-type mouse virulence and wild-type cycloheximide resistance; putative GTP-binding motif; similar to S. cerevisiae Yor165Wp; in gene cluster that encodes enzymes of GlcNAc catabolism; no human
PIS56860.1	28.66399354	29.25913533	28.42721175	28.76880978	28.98985456	29.28995918	0.480040889	0.921	0.233	PIS56860.1	orf19.2755	Subunit of the 20S core particle of the proteasome
PIS54773.1	29.45119883	26.56794098	27.46549979	28.13478004	28.20938971	27.83807428	0.743136408	0.949	0.233	PIS54773.1	orf19.4228	Protein with a role in insertion of tail-anchored proteins into the ER membrane; Spider biofilm repressed

PIS59018.1	31.74474861	31.56065301	31.47346915	31.92638226	31.78770438	31.76117832	0.718130042	0.947	0.232	PIS59018.1	HSP70	Putative hsp70 chaperone; role in entry into host cells; heat-shock, amphotericin B, cadmium, ketoconazole-induced; surface localized in yeast and hyphae; antigenic in host; farnesol-downregulated in biofilm; Spider biofilm induced
PIS55784.1	27.43537427	27.47130186	27.47136335	27.49158581	27.74046989	27.84135139	0.59059792	0.936	0.232	PIS55784.1	PPH21	Catalytic subunit of protein phosphatase of the Type 2A-related family (serine/threonine-specific), involved in dephosphorylation of septin Sep7p; caspofungin repressed; possibly an essential gene, disruptants not obtained by UAU1 method
PIS52368.1	30.02183575	29.29796736	30.1549998	30.38132006	29.97722152	29.81361427	0.793855261	0.952	0.232	PIS52368.1	PPZ1	Protein phosphatase Z; fungal-specific type 1 family serine/threonine protein phosphatase involved in cation homeostasis and cell wall integrity
PIS51757.1	30.73185164	31.49310971	30.89699323	31.15393981	31.44850987	31.21629899	0.566928191	0.933	0.232	PIS51757.1	VPS13	Putative vacuolar protein sorting-associated protein; gene used for multilocus sequence typing
PIS48649.1	24.9883412	25.29739797	25.43502469	25.70778537	25.4959046	25.21048623	0.771360925	0.95	0.231	PIS48649.1	orf19.3223.1	Putative 12kDa subunit of mitochondrial NADH-ubiquinone oxidoreductase; gene has intron
PIS54984.1	25.80677319	27.09738888	26.23829802	26.38847645	26.03934468	27.40741455	0.721947907	0.947	0.231	PIS54984.1	orf19.4269	Ortholog of C. dubliniensis CD36 : Cd36_52330, C. parapsilosis CDC317 : CPAR2_101200, C. auris B8441 : B9J08_002133 and Candida tenuis NRRL Y-1498 : CANTEDRAFT_111562
PIS55675.1	28.59104643	29.98953144	28.27977639	29.24742249	29.26204575	29.04530761	0.594547215	0.936	0.231	PIS55675.1	orf19.5459	mRNA polyadenylation regulating protein; Hap43-repressed; transcript is upregulated in RHE model of oral candidiasis and in clinical isolates from HIV+ patients with oral candidiasis; Spider biofilm repressed
PIS50409.1	27.8232434	27.58251366	26.93287566	27.85510984	27.53545494	27.63701089	0.612121838	0.938	0.23	PIS50409.1	PHO15	HAD-family 2-phosphoglycolate phosphatase, likely involved in a metabolic repair system, not in protein dephosphorylation; involved in regulation of white-opaque switch; hyphal repressed; induced in core stress response
PIS54820.1	28.24863555	28.26880237	28.27261825	28.34877755	28.63155041	28.49532772	0.526593217	0.928	0.229	PIS54820.1	orf19.1804	Ortholog(s) have glycine transmembrane transporter activity, role in glycine import into mitochondrion, heme biosynthetic process and mitochondrion localization
PIS52423.1	27.37085176	27.9891873	27.60158768	27.39737378	28.20517344	28.04302798	0.554338389	0.932	0.228	PIS52423.1	CDL1	Putative RNAse III, ortholog of S. cerevisiae RNT1; merged with orf19.3772 in Assembly 21

PIS56869.1	25.87816758	25.31151212	25.97423172	26.49478224	26.58113255	24.77097223	0.707569147	0.946	0.228	PIS56869.1	orf19.2697	Ortholog(s) have ubiquitin protein ligase activity, ubiquitin-protein transferase activity
PIS54942.1	25.62111034	25.38275291	24.9758024	24.56200595	25.44889245	26.65299278	0.796498135	0.952	0.228	PIS54942.1	orf19.7365	Ortholog(s) have ubiquitin protein ligase activity, role in negative regulation of apoptotic process, negative regulation of gluconeogenesis, proteasome-mediated ubiquitin-dependent protein catabolic process and GID complex localization
PIS51442.1	34.25026865	34.72526403	33.9618134	34.15369362	34.8379029	34.62827	0.498051145	0.924	0.228	PIS51442.1	RPL3	Ribosomal protein, large subunit; induced by ciclopirox olamine treatment; genes encoding cytoplasmic ribosomal subunits are downregulated upon phagocytosis by murine macrophages; Hap43-induced gene; Spider biofilm repressed
PIS58721.1	24.28310612	24.48677534	25.1928112	24.48294894	25.11537021	25.04569294	0.853947206	0.955	0.227	PIS58721.1	FTH1	Putative iron permease involved in the production of prostaglandin E2; mutants show decreased metabolic activity in biofilms
PIS54855.1	26.91898741	27.83574819	26.72322186	27.26958414	27.24865746	27.64120223	0.554746397	0.932	0.227	PIS54855.1	HBR3	Essential protein; regulated by hemoglobin; S. cerevisiae ortholog is essential; Hap43p-induced gene
PIS58990.1	23.81915159	24.92785794	24.05697325	25.39334998	24.59622084	23.49536354	0.75824909	0.95	0.227	PIS58990.1	LEU3	Zn(II)2Cys6 transcription factor; predicted regulator branched-c ofhain amino acid biosynthesis genes; alkaline induced; induced by Mnl1 under weak acid stress; required for yeast cell adherence to silicone substrate; Spider biofilm induced
PIS58446.1	29.66724529	29.20614342	29.88652186	30.12777144	29.81421963	29.49571249	0.53879888	0.93	0.226	PIS58446.1	orf19.3679	Putative protein of unknown function; stationary phase enriched protein
PIS51125.1	26.80561207	25.62108144	25.8359327	26.40237284	25.97340449	26.56072572	0.59431697	0.936	0.225	PIS51125.1	orf19.642	Regulatory subunit of PP2A-like protein phosphatase Sit4p, involved in cell wall maintenance, regulation of hyphal growth, and virulence
PIS52432.1	29.74305901	29.5596612	29.55393336	29.84537935	29.91850043	29.76857798	0.392097192	0.904	0.225	PIS52432.1	SFH5	Putative phosphatidylinositol transporter; rat catheter and Spider biofilm repressed
PIS51468.1	30.5610293	30.55507417	30.57360127	30.7218419	30.76105953	30.879261	0.750836304	0.949	0.224	PIS51468.1	SMP2	Putative Mg2+-dependent phosphatidate phosphatase; transcript regulated by Nrg1

PIS51561.1	29.68149231	30.10992741	29.81069568	30.04314886	30.10028884	30.12485917	0.469705935	0.92	0.222 PIS51561.1	IST2	Ortholog(s) have lipid binding activity and role in endoplasmic reticulum membrane organization, protein localization to plasma membrane, regulation of phosphatidylinositol dephosphorylation
PIS58607.1	30.4456685	30.12715667	30.71445145	30.59198391	30.75038711	30.61209875	0.516923868	0.927	0.222 PIS58607.1	TOM20	Putative mitochondrial primary import receptor
PIS51881.1	27.58157165	27.25151377	27.82132191	27.60216365	28.20250845	27.51005396	0.543291879	0.93	0.22 PIS51881.1	TRM1	Putative N2,N2-dimethylguanine tRNA methyltransferase; induced upon adherence to polystyrene
PIS51743.1	25.2686804	22.74273161	25.29170822	24.14945377	25.53924108	24.27511186	0.788537564	0.951	0.22 PIS51743.1	UBP13	Ortholog of S. cerevisiae Ubp13; putative ubiquitin carboxyl-terminal hydrolase; flow model biofilm induced; rat catheter biofilm repressed
PIS48726.1	28.96786018	29.20022894	28.97753432	29.16853173	29.24887679	29.38604428	0.419052977	0.91	0.219 PIS48726.1	orf19.7152	Protein similar to Aspergillus CYSK O-acetylserine sulfhydrylase, suggesting that C. albicans uses an O-acetyl-serine (OAS) pathway of sulfur assimilation; upregulated in biofilm; protein level decreases in stationary phase cultures
PIS55526.1	28.12400399	27.89098114	27.80407161	28.4244812	28.27327124	27.77813417	0.562189706	0.932	0.219 PIS55526.1	TSM1	Putative transcription initiation factor TFIID subunit; transcript is upregulated in clinical isolates from HIV+ patients with oral candidiasis; Nrg1-regulated
PIS50381.1	28.84243836	29.2754328	28.15993903	28.90271785	28.82935227	29.19900508	0.634309219	0.94	0.218 PIS50381.1	APM4	Cargo-binding subunit of the clathrin associated protein complex (AP-2), involved in endocytosis; regulates polarized growth through endocytic recycling of chitin synthase Chs3p
PIS48233.1	34.10601391	34.38325912	33.90795239	34.09056856	34.41836102	34.54374637	0.428824216	0.912	0.218 PIS48233.1	HSP90	Essential chaperone, regulates several signal transduction pathways and temperature-induced morphogenesis; activated by heat shock, stress; localizes to surface of hyphae, not yeast cells; mediates echinocandin and biofilm azole resistance
PIS49774.1	25.65570861	26.16248667	25.865586	26.37727307	25.80132917	26.15851149	0.686391763	0.944	0.218 PIS49774.1	POP4	Ortholog of S. cerevisiae Pop4; a subunit of both RNase MRP and nuclear RNase P; filament induced; regulated by Nrg1, Tup1; likely essential, based on UAU1 strategy; rat catheter and Spider biofilm induced
PIS58215.1	31.04067732	31.17782833	30.62729942	30.88909865	31.30149342	31.31071042	0.55892097	0.932	0.218 PIS58215.1	RLI1	Member of RNase L inhibitor (RLI) subfamily of ABC family; predicted not to be a transporter; regulated by Sef1p, Sfu1p, and Hap43p

PIS58638.1	25.79705677	28.4140009	24.36057014	26.11937266	26.34386058	26.75853786	0.827862336	0.954	0.217	PIS58638.1	ATP14	Putative mitochondrial F1F0 ATP synthase subunit; macrophage/pseudohyphal-induced
PIS58766.1	30.48171981	30.88443657	29.93158115	30.21530068	30.83216628	30.90132168	0.556640108	0.932	0.217	PIS58766.1	MRP7	Mitochondrial ribosomal protein of the large subunit; rat catheter biofilm induced
PIS51367.1	25.55177417	21.26441071	23.66126681	23.38003083	23.44346252	24.30479673	0.775196251	0.951	0.217	PIS51367.1	orf19.190	Ortholog(s) have role in chromatin remodeling, endoplasmic reticulum organization and Swr1 complex localization
PIS49551.1	24.852912	24.7701943	24.83937665	25.07936627	25.28012639	24.75428488	0.877420376	0.956	0.217	PIS49551.1	orf19.2478	Ortholog of C. dubliniensis CD36 : Cd36_05410, C. parapsilosis CDC317 : CPAR2_107480, C. auris B8441 : B9J08_004575 and Candida tenuis NRRL Y-1498 : CANTEDRAFT_94695
PIS48311.1	24.74020905	25.38551175	22.88547112	25.25196057	23.49402589	24.91580849	0.746458857	0.949	0.217	PIS48311.1	orf19.3528	Protein of unknown function; Spider biofilm induced
PIS58379.1	24.32315768	24.19554756	24.4658532	25.11106346	24.24369187	24.27815931	0.614794335	0.938	0.216	PIS58379.1	GWT1	Inositol acyltransferase with role in early steps of GPI anchor biosynthetic process; antifungal drug target
PIS49569.1	27.11972839	26.96274654	27.06973051	27.12399587	27.40678309	27.26913075	0.636829061	0.94	0.216	PIS49569.1	NHX1	Protein similar to S. cerevisiae Nhx1p, which is an Na+/H+ exchanger required for intracellular sequestration of Na+
PIS58851.1	23.83092888	24.47374229	23.39412295	24.22540113	24.12100247	23.99604876	0.640466772	0.941	0.215	PIS58851.1	PTP2	Predicted protein tyrosine phosphatase; involved in regulation of MAP kinase Hog1 activity; induced by Mnl1 under weak acid stress; rat catheter and Spider biofilm induced
PIS58325.1	30.41023626	30.92050034	30.20202935	30.19674717	30.93291187	31.04093076	0.5353019	0.929	0.213	PIS58325.1	GCR3	Functional homolog of S. cerevisiae Gcr3, which acts in regulation of glycolytic genes; no intron predicted, in contrast to intron in S. cerevisiae GCR3 gene
PIS54666.1	26.28370671	26.6584225	25.47432762	26.66130079	25.9553625	26.438306	0.667703271	0.943	0.213	PIS54666.1	orf19.6980	Ortholog(s) have cell periphery, cellular bud neck localization

PIS48282.1	32.3210537	32.04631117	32.22354108	32.48132669	32.41028073	32.33212227	0.770253965	0.95	0.211	PIS48282.1	HYU1	Putative hydantoin utilization protein A; induced upon adherence to polystyrene; regulated by Gcn2p and Gcn4p
PIS48671.1	28.97317425	29.2449441	28.67188386	29.06738681	29.26665	29.18942473	0.509635019	0.926	0.211	PIS48671.1	orf19.3394	Predicted membrane protein; induced by alpha pheromone in SpiderM medium
PIS51918.1	25.1666877	26.58726079	25.49272257	26.52913459	25.51331232	25.83692758	0.755376557	0.949	0.211	PIS51918.1	orf19.4173	Ortholog(s) have role in protein histidyl modification to diphthamide
PIS55500.1	28.75366059	29.05958269	28.97334847	29.29272094	28.96962841	29.1561351	0.592134334	0.936	0.211	PIS55500.1	orf19.5052	Ortholog of S. cerevisiae : MRX20, C. glabrata CBS138 : CAGL0C02013g, C. dubliniensis CD36 : Cd36_07340, C. parapsilosis CDC317 : CPAR2_208340 and C. auris B8441 : B9J08_001600
PIS51385.1	29.71189077	30.49620779	29.69501797	29.88242564	30.37634729	30.27812424	0.618576619	0.938	0.211	PIS51385.1	orf19.7215	Nucleolar protein; component of the small subunit processome containing the U3 snoRNA; involved in pre-18S rRNA processing; flow model biofilm repressed
PIS50407.1	26.64096436	26.80320814	27.55584436	26.71175467	27.44021221	27.47724717	0.688537031	0.945	0.21	PIS50407.1	ALG9	Putative mannosyltransferase; similar to S. cerevisiae Alg9p; has HKEXRF motif
PIS50385.1	25.54279935	26.30104716	25.54143902	26.59128147	25.55474293	25.86890056	0.830303639	0.954	0.21	PIS50385.1	orf19.1265	Ortholog(s) have guanyl-nucleotide exchange factor activity and role in cytoplasm to vacuole transport by the Cvt pathway, early endosome to Golgi transport, intra-Golgi vesicle-mediated transport, macroautophagy
PIS51614.1	25.25430777	25.59467163	25.02130279	25.5344525	25.56445465	25.39979095	0.581853761	0.935	0.209	PIS51614.1	orf19.1794	Ortholog(s) have mRNA 5'-UTR binding, pre-mRNA intronic binding, translation regulator activity and role in Group I intron splicing, mitochondrial mRNA processing, positive regulation of mitochondrial translation
PIS48357.1	26.3685362	27.98556341	26.36076621	27.23647167	26.98901866	27.1143572	0.739875956	0.948	0.208	PIS48357.1	ERV46	Putative ER-derived vesicle protein; COPII-coated vesicle complex subunit; transcript induced by filamentous growth; Spider biofilm repressed
PIS49662.1	31.57569988	31.19245962	31.52727872	31.71469853	31.65414452	31.55134344	0.450589666	0.916	0.208	PIS49662.1	SEC26	Secretory vesicles coatomer complex protein

PIS49571.1	29.17141258	29.3450142	28.90486339	29.38934571	29.3730132	29.27984924	0.456792074	0.917	0.207	PIS49571.1	orf19.4204	Ortholog(s) have structural constituent of ribosome activity and mitochondrial small ribosomal subunit localization
PIS55477.1	27.73127368	27.92001727	27.18035607	27.78085484	28.10134254	27.57149478	0.65372563	0.942	0.207	PIS55477.1	PLD1	Phospholipase D1; required for phosphatidic acid and for most diacylglycerol production; required for wild-type mouse virulence, but not rat oral virulence; mutant defect in hyphal growth on solid substrates; similar to S. cerevisiae Spo14p
PIS48332.1	27.83121988	27.72609555	28.60147738	28.65343199	27.93133464	28.19099591	0.605413491	0.937	0.206	PIS48332.1	BNA4	Putative kynurenine 3-monooxygenase, involved in NAD biosynthesis; transposon mutation affects filamentous growth; Hap43p-repressed gene; oral infection upregulated; mutants have reduced capacity to damage oral epithelial cells
PIS55641.1	31.39410962	31.46406043	31.57786271	31.53208298	31.81003542	31.71139947	0.661377257	0.942	0.206	PIS55641.1	DBP5	Ortholog(s) have ATP-dependent activity, acting on RNA, RNA helicase activity, inositol hexakisphosphate binding activity
PIS52206.1	30.23942582	30.69951032	29.07249979	29.89732828	30.26116603	30.47187905	0.71317635	0.946	0.206	PIS52206.1	GLR1	Glutathione reductase; upregulated by human neutrophils; oxidative stress-induced regulation via Cap1p; overexpression correlates with multidrug resistance in a cap1 mutant, farnesol induced; stationary phase enriched protein
PIS58777.1	28.77767623	28.9900404	29.03820294	29.27793329	29.19599413	28.94986662	0.60752161	0.937	0.206	PIS58777.1	SPL1	Protein similar to S. cerevisiae Spl1p, which is involved in tRNA splicing; member of pyridoxal-phosphate-dependent aminotransferase protein family; predicted to be essential
PIS51574.1	28.9933215	29.47035227	29.54154204	29.39948856	29.63560804	29.58697019	0.623016057	0.939	0.206	PIS51574.1	UBP6	Putative ubiquitin-specific protease of the 26S proteasome; oxidative stress-induced via Cap1p
PIS51047.1	28.88911148	28.46713523	29.15327477	29.26900386	28.93979759	28.91672457	0.767263295	0.95	0.205	PIS51047.1	FLC1	Protein involved in heme uptake; putative FAD transporter, similar to S. cerevisiae Flc1; regulated by iron; macrophage-induced; mutant defective in filamentous growth; Spider biofilm induced
PIS58236.1	32.71867595	32.81897512	32.15239444	32.48576024	32.98046596	32.83462704	0.579069061	0.934	0.204	PIS58236.1	RPL39	Ribosomal protein L39; transcript induced upon germ tube formation; colony morphology-related gene regulation by Ssn6; Hap43-induced
PIS52144.1	33.13541009	33.18558012	33.19232697	33.24305483	33.49296696	33.38949291	0.528179046	0.928	0.204	PIS52144.1	RPS15	Putative ribosomal protein; macrophage/pseudohyphal-induced after 16 h; repressed upon phagocytosis by murine macrophage; Spider biofilm repressed

PIS58281.1	29.97797521	29.51683725	30.17944908	30.03910318	30.23654525	30.00703855	0.482206957	0.922	0.203	PIS58281.1	TPS2	Trehalose-6-phosphate (Tre6P) phosphatase; mutant heat sensitive, accumulates Tre6P, decreased mouse virulence; possible drug target; 2 conserved phospohydrolase motifs; no mammalian homolog; Hap43-repressed; flow model biofilm induced
PIS52222.1	24.92563374	25.06499602	25.2042848	24.97514078	25.38034983	25.44484078	0.599347138	0.937	0.202	PIS52222.1	HXK1	N-acetylglucosamine (GlcNAc) kinase; involved in GlcNAc utilization; required for wild-type hyphal growth and mouse virulence; GlcNAc-induced transcript; induced by alpha pheromone in SpiderM medium
PIS52081.1	29.71577243	29.11559464	29.87651735	29.77135215	29.91280491	29.62948341	0.714296857	0.947	0.202	PIS52081.1	PMT5	Protein mannosyltransferase (PMT), expressed at extremely low levels; not required for wild-type hyphal growth, drug resistance, or virulence in mouse systemic infection; one of five PMT family members
PIS49633.1	29.78274461	29.65428122	29.2878295	29.77840389	29.73384253	29.81909016	0.567380749	0.933	0.202	PIS49633.1	RPN7	Subunit of the proteasome regulatory particle; regulated by Gcn2p and Gcn4p; protein present in exponential and stationary growth phase yeast cultures
PIS50342.1	28.7509143	28.17085177	29.23300049	29.10916993	29.29898049	28.35162588	0.650512143	0.941	0.202	PIS50342.1	SSH1	Protein with a role in protein translocation across membranes
PIS56961.1	29.90661287	29.90766655	30.32668821	30.1415866	30.39178026	30.21376221	0.531299541	0.929	0.202	PIS56961.1	STE24	Prenyl-dependent protease
PIS50571.1	27.73845326	27.1504428	28.01850348	27.98543954	27.85162175	27.67765476	0.531940429	0.929	0.202	PIS50571.1	TIP1	Protein with a predicted role in biogenesis of ER-derived COPII transport vesicles; mutation confers hypersensitivity to toxic ergosterol analog
PIS55753.1	26.61162521	28.91605308	26.84336528	27.13828278	27.61230915	28.22294405	0.804777613	0.952	0.201	PIS55753.1	orf19.3648	Ortholog(s) have DNA endonuclease activity, crossover junction DNA endonuclease activity
PIS55527.1	27.552018	27.87967383	26.9347298	27.51903445	27.71935644	27.73106212	0.881197119	0.956	0.201	PIS55527.1	orf19.5533	Ortholog(s) have guanyl-nucleotide exchange factor activity, ubiquitin binding activity
PIS54631.1	28.38051269	28.03811718	29.24779411	28.97612979	28.60438097	28.68889274	0.787196165	0.951	0.201	PIS54631.1	OYE2	Putative NAPDH dehydrogenase; induced by nitric oxide; Spider biofilm induced

PIS49477.1	30.99004864	30.8339806	31.10144543	31.25595434	31.13752427	31.1313089	0.47146925	0.92	0.2	PIS49477.1	ERG13	3-hydroxy-3-methylglutaryl coenzyme A synthase; ergosterol biosynthesis; sumoylation target; Tn mutation affects filamentation; amphotericin B, caspofungin repressed; exponential, stationary growth phase expressed; Spider biofilm repressed
PIS56897.1	28.09434065	27.78852354	28.25365696	28.56288297	28.07844028	28.09444915	0.535263261	0.929	0.2	PIS56897.1	MHP1	Protein similar to S. cerevisiae Mhp1p, which is involved in microtubule stabilization; transposon mutation affects filamentous growth; possibly transcriptionally regulated upon hyphal formation; possibly an essential gene (by UAU1 method)
PIS56542.1	25.42155603	25.99956543	26.10493955	25.84820658	25.70164986	26.57280041	0.675359925	0.944	0.199	PIS56542.1	orf19.5539	Ortholog(s) have SNAP receptor activity, role in retrograde vesicle-mediated transport, Golgi to endoplasmic reticulum and SNARE complex, cytoplasmic side of endoplasmic reticulum membrane localization
PIS58978.1	22.37180742	23.32386932	23.46336899	23.4044467	22.84660292	23.50417711	0.904021136	0.957	0.199	PIS58978.1	orf19.934	Predicted ORF from Assembly 19; removed from Assembly 20; subsequently reinstated in Assembly 21 and merged with orf19.2224, based on comparative genome analysis; flow model biofilm repressed
PIS48413.1	26.07410996	26.53945256	25.17965206	24.90638744	26.78551394	26.69867459	0.791846968	0.952	0.199	PIS48413.1	STE13	Putative pheromone-processing dipeptidyl aminopeptidase; possible Kex2 substrate; transposon mutation affects filamentous growth; induced by low nitrogen, germ tube formation; flow model biofilm repressed
PIS52236.1	27.71237562	27.47141773	28.02597005	27.84550773	28.07080506	27.88613426	0.69323795	0.945	0.198	PIS52236.1	orf19.1991	Has domain(s) with predicted membrane localization
PIS52113.1	27.15993138	27.07299338	27.13704247	27.04706371	27.49780537	27.42010523	0.515193474	0.927	0.198	PIS52113.1	orf19.4516	Ortholog(s) have tetrahydrofolylpolyglutamate synthase activity, role in one-carbon metabolic process and cytoplasm, mitochondrion localization
PIS50453.1	26.82385944	26.31012906	27.13147386	26.71358903	26.87817503	27.26821597	0.710739993	0.946	0.198	PIS50453.1	orf19.875	Protein required for respiratory growth
PIS54929.1	32.98033929	32.7142124	32.78281793	33.07486486	33.09850259	32.89843312	0.69019148	0.945	0.198	PIS54929.1	URA2	Putative bifunctional carbamoylphosphate synthetase-aspartate transcarbamylase; flucytosine induced; macrophage/pseudohyphal-induced; 5'-UTR intron; flow model biofilm repressed
PIS54525.1	28.36415886	27.68971139	28.4904052	28.59023321	28.45199909	28.09200315	0.683329304	0.944	0.197	PIS54525.1	orf19.6230	Ortholog(s) have 5'-hydroxyl dinucleotide hydrolase activity, GDP binding, RNA NAD-cap (NAD-forming) hydrolase activity, enzyme regulator activity, mRNA 5'-diphosphatase activity, phosphodiesterase decapping endonuclease activity

PIS52407.1	24.01321189	25.61677895	24.26632165	24.02451633	24.90038174	25.55809797	0.741159219	0.948	0.196	PIS52407.1	orf19.4878	Protein of unknown function; Hap43-repressed; rat catheter biofilm repressed
PIS54679.1	28.37600269	27.80553211	28.65172975	28.10141858	28.82025994	28.49603829	0.692720039	0.945	0.195	PIS54679.1	HAT1	Hat1-Hat2 histone acetyltransferase complex subunit; involved in DNA damage repair and morphogenesis; mutations cause constitutive pseudohyphal growth, white to opaque switch, caspofungin sensitivity; rat catheter, Spider biofilm repressed
PIS56626.1	28.8708119	28.06091865	28.9313447	28.87541026	28.74785494	28.82088414	0.561635	0.932	0.194	PIS56626.1	PEX8	Putative peroxisomal biogenesis factor; expression regulated during planktonic growth
PIS55826.1	28.70788986	27.53354221	26.8421078	27.61020937	27.69291379	28.36036875	0.818224538	0.953	0.193	PIS55826.1	ASR1	Heat shock protein; transcript regulated by cAMP, osmotic stress, ciclopirox olamine, ketoconazole; repressed by Cyr1, Ras1; colony morphology-related regulated by Ssn6; stationary phase enriched; Hap43-induced; Spider biofilm induced
PIS56906.1	27.78186544	27.58224909	28.19577214	27.940671	27.96901953	28.22792554	0.753900028	0.949	0.193	PIS56906.1	MAS2	Putative processing peptidase, catalytic (alpha) subunit; protein level decreases in stationary phase cultures
PIS52429.1	29.98010432	29.57787982	30.32976556	30.15559571	30.13287895	30.17926203	0.707387062	0.946	0.193	PIS52429.1	TRP3	Putative bifunctional enzyme with predicted indole-3-glycerol-phosphate synthase and anthranilate synthase activities; regulated by Gcn2p and Gcn4p
PIS55811.1	34.40349986	34.11913137	34.74449757	34.73561814	34.59034018	34.51574045	0.490561685	0.923	0.192	PIS55811.1	CAM1	Putative translation elongation factor eEF1 gamma; protein level decreased in stationary phase cultures; Spider biofilm repressed
PIS55052.1	26.32098215	25.935008	26.95493599	26.91931628	26.90674834	25.96149922	0.791307336	0.952	0.192	PIS55052.1	PHO89	Putative phosphate permease; transcript regulated upon white-opaque switch; alkaline induced by Rim101; possibly adherence-induced; F-12/CO2 model, rat catheter and Spider biofilm induced
PIS58753.1	33.72145504	33.76469221	33.86255723	33.9275238	33.99196078	34.0042669	0.47902025	0.921	0.192	PIS58753.1	RPS9B	Predicted ribosomal protein; repressed upon phagocytosis by murine macrophage; transcript possibly regulated upon hyphal formation; Spider biofilm repressed
PIS56752.1	23.91406625	24.21473428	24.27000612	24.40186182	23.92617923	24.64703909	0.6170901	0.938	0.192	PIS56752.1	SOD4	Cu-containing superoxide dismutase; role in response to host innate immune ROS; regulated on white-opaque switch; induced under iron starvation; ciclopirox olamine induced; caspofungin repressed; SOD1,4,5,6 gene family

PIS58328.1	29.589135	29.92642002	29.77993417	30.0273854	29.9997559	29.8420511	0.722187492	0.947	0.191	PIS58328.1	CBK1	Ser/Thr kinase of cell wall integrity pathway; mutants show abnormal morphology and aggregation; Mob2p associated; required for wild-type hyphal growth and transcriptional regulation of cell-wall-associated genes
PIS52105.1	27.10084845	27.40581134	27.60072138	27.24621645	27.52227384	27.91078746	0.668849591	0.943	0.191	PIS52105.1	GDT1	Golgi Ca2+/H+ exchanger, plays a compensatory role for the calcium pump Pmr1p in regulation of calcium homeostasis
PIS56583.1	25.97935094	27.99749763	25.62710837	25.98260078	27.00964261	27.18214607	0.756511572	0.949	0.19	PIS56583.1	CTA8	Essential transcription factor, mediates heat shock transcriptional induction; in the absence of heat stress, Cta8p levels are modulated by growth temperature to regulate basal expression of genes involved in protein folding
PIS58422.1	29.34367404	29.32465001	28.79196508	29.48326542	29.34453309	29.20305305	0.585449967	0.935	0.19	PIS58422.1	ERG26	C-3 sterol dehydrogenase, catalyzes the 2nd of 3 steps required to remove 2 C-4 methyl groups from an intermediate in ergosterol biosynthesis; amphotericin B, caspofungin repressed; Spider biofilm repressed
PIS54477.1	30.79463149	30.80729131	30.99002551	31.18650183	30.85698816	31.11826532	0.509921628	0.926	0.19	PIS54477.1	HGT2	Putative MFS glucose transporter; 20 member C. albicans glucose transporter family; 12 probable membrane-spanning segments; expressed in rich medium with 2% glucose; rat catheter and Spider biofilm induced
PIS58746.1	26.79936216	26.3830004	26.75058803	26.83141639	26.83516	26.83500811	0.794558906	0.952	0.19	PIS58746.1	NUP82	Linker nucleoporin of the nuclear pore complex; role in mRNA andexport from nucleus, protein import into nucleus, ribosomal large subunit export from nucleus, ribosomal small subunit export from nucleus; rat catheter biofilm repressed
PIS58046.1	23.17085706	23.38664875	22.98540037	23.39132705	23.36672772	23.3533697	0.645652798	0.941	0.19	PIS58046.1	orf19.3898	Ortholog(s) have SNAP receptor activity, role in endocytosis, vesicle fusion and SNARE complex, endosome, trans-Golgi network localization
PIS51382.1	32.99075207	33.33860806	32.28862542	32.62302513	33.38181735	33.18265253	0.60032792	0.937	0.19	PIS51382.1	RPS8A	Small 40S ribosomal subunit protein; induced by ciclopirox olamine; repressed upon phagocytosis by murine macrophage; 5'-UTR intron; Hap43-induced; Spider biofilm repressed
PIS56928.1	23.17853361	23.47222692	24.03399658	23.98877357	23.06377417	24.20008124	0.73008327	0.948	0.189	PIS56928.1	orf19.4030	Ortholog(s) have DNA primase activity, single-stranded DNA binding activity and role in DNA replication, DNA replication, synthesis of RNA primer, mitotic DNA replication initiation
PIS58090.1	28.75605401	28.1500926	28.85775577	28.69816372	28.80764494	28.82405645	0.55070447	0.931	0.189	PIS58090.1	orf19.597	Protein with an aspartate aminotransferase domain; Gcn4-regulated

PIS58573.1	28.29219793	28.62241942	27.42950905	27.79070913	28.47480965	28.64529817	0.718123028	0.947	0.189	PIS58573.1	orf19.6853	Protein of unknown function; Spider biofilm repressed
PIS48436.1	25.77846273	28.34093943	25.45482089	27.10685928	26.29076099	26.74290868	0.771722233	0.95	0.189	PIS48436.1	PUP2	Alpha5 subunit of the 20S proteasome; macrophage/pseudohyphal-repressed; regulated by Gcn2p and Gcn4p; protein present in exponential and stationary growth phase yeast cultures
PIS48323.1	24.11031912	25.08186856	24.01823656	24.05125201	24.82235258	24.89977989	0.650828382	0.941	0.188	PIS48323.1	CDC7	Catalytic subunit of Dbf4p-regulated serine/threonine protein kinase; negative regulator of hyphal development; cell-cycle regulated periodic mRNA expression; S. cerevisiae ortholog is not cell-cycle regulated
PIS52345.1	29.3106564	29.18307877	29.53446233	29.30630828	29.57620804	29.70972731	0.556520043	0.932	0.188	PIS52345.1	SLC1	Putative fatty acyltransferase; protein repressed during the mating process
PIS55824.1	30.15762497	31.66506271	31.56520791	31.23865241	31.38957004	31.31974683	0.698676827	0.945	0.187	PIS55824.1	AOX2	Alternative oxidase; cyanide-resistant respiration; induced by antimycin A, oxidants; growth; Hap43, chlamydospore formation repressed; rat catheter, Spider biofilm induced; regulated in Spider biofilms by Bcr1, Tec1, Ndt80, Brg1
PIS51702.1	25.98485773	26.43606547	26.24236529	26.22080679	26.15702961	26.84510225	0.709420778	0.946	0.187	PIS51702.1	END3	Protein involved in endocytosis, cell wall integrity and morphology; regulated by Gcn4p; induced in response to amino acid starvation (3-aminotriazole treatment)
PIS52229.1	25.76913249	25.00922421	25.42031448	25.97293961	25.69576715	25.09231913	0.780062162	0.951	0.187	PIS52229.1	orf19.6379	Ortholog of C. dubliniensis CD36 : Cd36_33820, C. parapsilosis CDC317 : CPAR2_206210, C. auris B8441 : B9J08_003843 and Candida tenuis NRRL Y-1498 : CANTEDRAFT_92567
PIS48705.1	24.91666247	24.42980806	26.1566311	26.12569931	25.35175492	24.58505835	0.773832502	0.951	0.186	PIS48705.1	orf19.3449.2	Putative mitochondrial phosphatidylglycerophosphatase (PGP phosphatase); essential for cardiolipin biosynthesis; rat catheter biofilm induced
PIS49500.1	25.04632055	26.01245803	25.67817256	25.89699587	25.21223354	26.18257874	0.738777297	0.948	0.185	PIS49500.1	orf19.429	Putative non-canonical poly(A) polymerase; repressed by nitric oxide; Spider biofilm induced
PIS56959.1	29.60347716	29.38510186	29.83991843	29.80708928	29.84327146	29.73280156	0.760894069	0.95	0.185	PIS56959.1	orf19.5633	F-box domain-containing protein; flow model biofilm induced

PIS59029.1	27.39617639	27.9506222	27.55232958	27.48023724	27.80183217	28.17198842	0.768906801	0.95	0.185	PIS59029.1	PHO91	Putative low-affinity phosphate transporter; fungal-specific (no human or murine homolog)
PIS55738.1	27.30920113	27.80188786	24.72628587	26.69020691	27.7188302	25.97976983	0.797706585	0.952	0.184	PIS55738.1	MRPL40	Putative mitochondrial ribosomal protein; Spider biofilm repressed
PIS58718.1	24.93844696	24.51815637	24.10821323	23.71449076	25.4731308	24.92881218	0.77972435	0.951	0.184	PIS58718.1	orf19.332	Member of a complex that contains Prp19; stabilizes U6 snRNA in catalytic forms of the spliceosome containing U2, U5, and U6 snRNAs; Spider biofilm induced
PIS58381.1	32.5211198	32.26323459	32.59264748	32.67506453	32.80588331	32.44831512	0.810372835	0.953	0.184	PIS58381.1	orf19.6882.1	Ribosomal 60S subunit protein; Spider biofilm repressed
PIS49810.1	30.11977851	29.70048963	29.6220973	29.93721145	30.15732879	29.89886219	0.708040557	0.946	0.184	PIS49810.1	PDX3	Pyridoxamine-phosphate oxidase; transcript regulated by yeast-hypha switch and by Nrg1, Mig1, Tup1; Hap43, caspofungin repressed; present in exponential and stationary phase yeast cultures
PIS58645.1	33.61260524	33.85608957	32.94623172	33.1784994	33.97713274	33.80723787	0.636516023	0.94	0.183	PIS58645.1	RPS19A	Putative ribosomal protein S19; protein level decreases in stationary phase cultures; Spider biofilm repressed
PIS58585.1	28.94345571	29.78206218	28.1625755	28.71313286	29.24693406	29.47516856	0.709853046	0.946	0.182	PIS58585.1	NAP1	Nucleosome assembly protein; mutants show constitutive filamentous growth; present in exponential and stationary growth phase yeast cultures
PIS58319.1	28.12122312	28.46573365	27.87093276	28.32747613	28.31535191	28.36203932	0.672115823	0.943	0.182	PIS58319.1	orf19.4316	Trimethyllysine dioxygenase, the first enzyme in the carnitine biosynthesis pathway; hypha-induced expression, regulated by Cyr1, Ras1, Efg1; rat catheter biofilm repressed
PIS55493.1	28.9010634	28.49055087	29.196192	29.00863131	29.11571494	29.00657042	0.589925624	0.936	0.181	PIS55493.1	orf19.1229	Ortholog(s) have nuclear export signal receptor activity, role in protein export from nucleus, snRNA import into nucleus and nuclear envelope, nuclear periphery localization
PIS51987.1	28.84351287	28.21764963	28.99657501	29.05834355	28.76400129	28.77397666	0.644755683	0.941	0.18	PIS51987.1	CSH3	Functional homolog of S. cerevisiae Shr3p, which is a chaperone specific for amino acid permeases; localized to ER; required for wild-type amino-acid responsive hyphal growth and for mouse systemic virulence; regulated by Gcn2p and Gcn4p

PIS55021.1	24.00565079	26.11237399	24.83434722	23.9354959	25.24933732	26.3073899	0.767217863	0.95	0.18	PIS55021.1	HPA2	Ortholog(s) have D-amino-acid N-acetyltransferase activity, N-acetyltransferase activity and role in D-amino acid metabolic process, cellular detoxification of nitrogen compound, protein acetylation
PIS51133.1	26.77647196	27.30513175	26.41141058	27.54437273	27.02679471	26.46186863	0.789035304	0.951	0.18	PIS51133.1	MDL1	Putative mitochondrial, half-size MDR-subfamily ABC transporter
PIS56689.1	31.60102793	31.22091933	31.62821104	31.57053284	31.70112512	31.71891556	0.557635189	0.932	0.18	PIS56689.1	PMT2	Protein mannosyltransferase (PMT) with roles in hyphal growth and drug sensitivity; member of the PMT family which includes Pmt1p, Pmt2p, Pmt4p, Pmt5p, and Pmt6p; induced during cell wall regeneration; essential
PIS51726.1	28.46374323	28.67146936	28.71319152	28.75238956	28.84190024	28.79226784	0.687978714	0.945	0.179	PIS51726.1	YOR1	Protein similar to S. cerevisiae Yor1; ABC-type plasma membrane transporter involved in resistance to aureobasidin A; white cell type-specific transcript; Spider biofilm induced
PIS51537.1	28.4444776	28.23297828	28.2833857	28.59780258	28.43185087	28.46486253	0.583936684	0.935	0.178	PIS51537.1	orf19.4488	Predicted ortholog of S. cerevisiae Swi3, subunit of the SWI/SNF chromatin remodeling complex; possibly an essential gene, disruptants not obtained by UAU1 method
PIS58476.1	30.10114259	30.31570176	29.95516667	30.23103728	30.32619795	30.34276992	0.558622204	0.932	0.176	PIS58476.1	FRS2	Putative tRNA-Phe synthetase; downregulated upon phagocytosis by murine macrophage; protein present in exponential and stationary growth phase yeast cultures; Spider biofilm repressed
PIS48400.1	28.51123132	27.90579845	28.13098431	28.21087484	28.34057608	28.52424176	0.796235461	0.952	0.176	PIS48400.1	orf19.6553	Membrane-localized protein of unknown function; possibly secreted; fluconazole-induced
PIS56917.1	24.05835187	24.41244528	24.2329175	24.55999599	24.20646473	24.45620509	0.711044854	0.946	0.173	PIS56917.1	MYO1	Component of actomyosin ring at neck of newly-emerged bud
PIS51445.1	28.07082032	29.94502958	27.96441967	28.7541207	28.59990672	29.13839033	0.728373917	0.948	0.171	PIS51445.1	ERG24	C-14 sterol reductase, has a role in ergosterol biosynthesis; mutation confers increased sensitivity to dyclonine; rat catheter and Spider biofilm repressed
PIS55752.1	28.75360323	29.0362872	28.9461746	29.06641223	29.10594958	29.07585085	0.546889783	0.931	0.171	PIS55752.1	SEC8	Predicted subunit of the exocyst complex, involved in exocytosis; localizes to a crescent on the surface of the hyphal tip

PIS55806.1	27.40457592	26.74609249	27.22893713	27.3651698	27.29800889	27.2273531	0.835922067	0.954	0.17	PIS55806.1	ELP3	Predicted histone acetyltransferase; role in regulation of transcription, tRNA wobble uridine modification; Spider biofilm induced
PIS54752.1	27.81143013	27.99731018	27.5585198	27.81994291	28.17744698	27.87639815	0.842808527	0.954	0.169	PIS54752.1	orf19.5812	Ortholog of S. cerevisiae Ett1, a nuclear protein that inhibits replication of Brome mosaic virus; early-stage flow model biofilm induced
PIS58047.1	26.39854391	26.56549678	25.62281118	25.71429297	26.55083702	26.82775658	0.727673839	0.947	0.169	PIS58047.1	SPT3	Functional homolog of S. cerevisiae Spt3p; required for virulence in mouse systemic infection; homozygous null mutant is hyperfilamentous
PIS51352.1	26.88639008	25.9775217	26.51339485	27.31178035	26.86388233	25.70892854	0.712830835	0.946	0.169	PIS51352.1	ZCF13	Predicted Zn(II)2Cys6 transcription factor; similar to but not the true ortholog of S. cerevisiae Hap1; mutants display decreased colonization of mouse kidneys
PIS51577.1	28.36860306	27.8469938	27.97972147	27.90436219	28.5041818	28.29088456	0.831263865	0.954	0.168	PIS51577.1	orf19.6061	Ortholog(s) have role in N-acylethanolamine metabolic process, N-acylphosphatidylethanolamine metabolic process and mitochondrial inner membrane localization
PIS56565.1	30.84108068	30.72549946	30.38428591	30.59268611	31.09798684	30.7641022	0.625641011	0.939	0.168	PIS56565.1	RSR1	RAS-related protein; GTP/GDP cycling required for wild-type polar bud site selection, hyphal growth guidance; role in systemic virulence in mouse; geranylgeranylation predicted; suppresses S. cerevisiae cdc24-4 mutant heat sensitivity
PIS51437.1	28.32269195	29.40674833	27.71238633	27.84711411	28.91943297	29.17524627	0.900035566	0.957	0.167	PIS51437.1	orf19.4701	Ortholog(s) have ATPase, tRNA binding activity, role in protein urmylation, regulation of transcription by RNA polymerase II, tRNA wobble uridine modification and elongator holoenzyme complex localization
PIS51266.1	27.03073507	27.41792572	26.8814787	27.47579937	27.15028003	27.20636136	0.64008939	0.94	0.167	PIS51266.1	orf19.6156	Ortholog of S. cerevisiae : AIM11, C. glabrata CBS138 : CAGL0I04928g, C. dubliniensis CD36 : Cd36_80770, C. parapsilosis CDC317 : CPAR2_102260 and C. auris B8441 : B9J08_002841
PIS58780.1	26.84357251	26.95769085	26.97339325	26.84557102	27.56372975	26.86527378	0.655652295	0.942	0.167	PIS58780.1	RPL7	Ribosomal protein L7; repressed upon phagocytosis by murine macrophages; Hap43-induced; rat catheter and Spider biofilm induced
PIS50318.1	30.08300124	29.51390042	30.29666952	30.15525673	30.30371949	29.93360257	0.785827827	0.951	0.166	PIS50318.1	BLM3	Putative proteasome activator; binds core proteasome and stimulates proteasome-mediated protein degradation by inducing gate opening; ortholog of S. cerevisiae Blm10; transcript regulated by Nrg1 and Mig1

PIS51105.1	29.06153788	29.12716883	29.18280095	29.40192285	29.28844166	29.17959994	0.830227896	0.954	0.166	PIS51105.1	MIS12	Mitochondrial C1-tetrahydrofolate synthase precursor
PIS51760.1	24.71083016	23.94655448	24.3918182	24.74693138	24.60387591	24.19573228	0.838727352	0.954	0.166	PIS51760.1	orf19.1058	Subunit of the 19S regulatory base of the proteasome
PIS56840.1	31.60545403	31.61814627	31.18588036	31.34518813	31.85937529	31.70165918	0.556147294	0.932	0.166	PIS56840.1	ZUO1	Ortholog of S. cerevisiae Zuo1; a cytosolic ribosome-associated chaperone; likely to be essential for growth, based on an insertional mutagenesis strategy; Spider biofilm repressed
PIS58773.1	30.35916762	31.15835366	29.33501288	29.73490605	30.68856269	30.92330034	0.725619687	0.947	0.165	PIS58773.1	GBP2	Putative single-strand telomeric DNA-binding protein; protein level decreases in stationary phase cultures; Spider biofilm repressed
PIS56767.1	24.92344799	24.98723713	24.75145172	25.19599081	24.92994413	25.03041303	0.611294078	0.938	0.165	PIS56767.1	orf19.956	Ortholog(s) have role in fermentation, phospholipid homeostasis, protein maturation
PIS52367.1	25.66566755	25.83203484	26.50922585	26.15254051	26.01959096	26.3273847	0.761267344	0.95	0.164	PIS52367.1	orf19.6152	Ortholog of C. dubliniensis CD36 : Cd36_32830, C. parapsilosis CDC317 : CPAR2_205930, C. auris B8441 : B9J08_003982 and Candida tenuis NRRL Y-1498 : CANTEDRAFT_115846
PIS58208.1	32.66382825	31.96890827	32.73855057	32.72002294	32.50526665	32.63163843	0.761636107	0.95	0.162	PIS58208.1	MRPL6	Putative mitochondrial ribosomal protein
PIS52047.1	28.93430432	28.97066701	28.87738074	28.92049247	29.17315722	29.17339734	0.641564392	0.941	0.162	PIS52047.1	SNF4	Putative subunit of the AMP-activated Snf1p kinase; ortholog of S. cerevisiae Snf4; caspofungin repressed; transposon mutation affects filamentation
PIS56964.1	27.13935447	27.64453074	26.34897466	26.51825247	27.3099861	27.79145442	0.758716813	0.95	0.162	PIS56964.1	SYS1	Putative Golgi integral membrane protein; transcript regulated by Mig1
PIS50370.1	28.4381215	29.21903366	28.24254858	28.61803833	28.88765308	28.87783125	0.740887755	0.948	0.161	PIS50370.1	orf19.4751	Ortholog(s) have structural constituent of ribosome activity and mitochondrial small ribosomal subunit localization

PIS48401.1	28.75012772	28.73922166	28.51424487	28.76062451	28.94868365	28.7772963	0.591752736	0.936	0.161	PIS48401.1	orf19.6552	Flavin-linked sulfhydryl oxidase; predicted localization to endoplasmic reticulum lumen; involved in disulfide bond formation within the ER; Spider biofilm induced
PIS51884.1	24.95318137	26.01109708	24.58544299	25.0798007	24.94705499	26.00416611	0.799125016	0.952	0.16	PIS51884.1	CCE1	Putative Holliday junction resolving enzyme; similar to S. cerevisiae Cce1p
PIS50521.1	27.80922631	27.47573523	27.77399844	27.86126007	27.89519653	27.78193039	0.721386251	0.947	0.16	PIS50521.1	orf19.1323	Phosphorylated protein of unknown function; Hap43p-repressed gene
PIS51269.1	28.14259884	28.50343778	28.69723012	28.65532576	28.6309432	28.53254027	0.637774589	0.94	0.159	PIS51269.1	ARP9	Protein similar to S. cerevisiae Arp3p, a component of the Arp2/3 complex involved in actin-dependent processes; likely to be essential for growth, based on an insertional mutagenesis strategy
PIS56841.1	24.50208183	25.36582229	24.16031366	25.40756399	24.79314637	24.30486295	0.817559227	0.953	0.159	PIS56841.1	orf19.2710	Ortholog(s) have enzyme activator activity and role in chromosome segregation
PIS55775.1	28.30723503	28.49252984	27.87389169	28.30583871	28.4092476	28.43630083	0.742482779	0.948	0.159	PIS55775.1	orf19.6752	Ortholog(s) have structural constituent of ribosome activity and mitochondrial small ribosomal subunit localization
PIS54733.1	29.45723683	29.02936185	29.35145903	29.43869391	29.36423873	29.50574705	0.638924635	0.94	0.157	PIS54733.1	AGM1	Phosphoacetylglucosamine mutase (N-acetylglucosamine-phosphate mutase); enzyme of UDP-N-acetylglucosamine (UDP-GlcNAc) biosynthesis
PIS56708.1	25.39140654	26.20196488	25.34531868	24.97634451	26.38824568	26.04465447	0.776468529	0.951	0.157	PIS56708.1	MCD1	Alpha-kleisin cohesin complex subunit; for sister chromatid cohesion in mitosis and meiosis; repressed by alpha pheromone in SpiderM medium; periodic cell-cycle expression; Hap43-repressed; rat catheter and Spider biofilm repressed
PIS58731.1	29.11323488	29.08874797	29.24403561	29.50056442	29.32871207	29.08839044	0.707932852	0.946	0.157	PIS58731.1	MET18	Putative protein with a predicted role in nucleotide excision repair (NER) and RNA polymerase II (RNAP II) transcription; Plc1p-regulated
PIS51504.1	27.44207685	28.07448698	27.7770629	27.96734592	27.97741572	27.81880983	0.664118877	0.943	0.157	PIS51504.1	MNN21	Ortholog(s) have alpha-1,2-mannosyltransferase activity, role in protein glycosylation and Golgi apparatus localization

PIS51067.1	28.9728124	29.35196994	29.43494596	29.23535737	29.47083187	29.52602723	0.621891101	0.939	0.157	PIS51067.1	orf19.1626	Deoxyhypusine synthase; catalyzes formation of deoxyhypusine, the first step in hypusine biosynthesis; Spider biofilm repressed
PIS54814.1	30.18357477	30.03695785	30.42148794	30.663627	30.1567349	30.29209285	0.637473341	0.94	0.157	PIS54814.1	SAP9	Secreted aspartyl protease; roles in adhesion, cell surface integrity; induced by antifungal drugs, stationary phase, or in white-phase cells; farnesol-downregulated in biofilm; autocatalytic processing; GPI-anchor; Spider biofilm induced
PIS48574.1	29.62589477	29.24908118	29.8738477	29.92739706	29.70053187	29.58895103	0.585425138	0.935	0.156	PIS48574.1	STE23	Ortholog of S. cerevisiae Ste23 metalloprotease; role in N-terminal processing of pro-a-factor to the mature form; Tn mutation affects filamentous growth; Spider biofilm induced
PIS52180.1	30.46637329	30.78096608	29.42675692	30.21150515	30.30200106	30.6265574	0.722426913	0.947	0.155	PIS52180.1	CPY1	Carboxypeptidase Y; transcript regulated at yeast-hypha transition or macrophage response; induced human neutrophils; regulated by Gcn2 and Gcn4; putative N-glycosylation
PIS58152.1	27.31402021	27.76343483	27.0273857	27.52728286	27.62657057	27.41497957	0.598533958	0.936	0.155	PIS58152.1	orf19.1340	Putative aldose reductase; protein level decreases in stationary phase cultures; Spider biofilm repressed
PIS51281.1	24.15639396	23.63635283	23.17359534	23.66756868	24.07204392	23.68939818	0.833733197	0.954	0.154	PIS51281.1	CHS8	Chitin synthase required for synthesis of long-chitin fibrils; nonessential; 8 or 9 membrane spanning regions; mRNA present in yeast and hyphae; induced during cell wall regeneration; flow model biofilm repressed
PIS58736.1	33.12983612	33.38577528	31.96711118	32.38475744	33.39123095	33.16923584	0.710969318	0.946	0.154	PIS58736.1	RPS7A	Ribosomal protein S7; genes encoding cytoplasmic ribosomal subunits, translation factors, and tRNA synthetases are downregulated upon phagocytosis by murine macrophage; Spider biofilm repressed
PIS54489.1	27.94036008	29.5341089	28.78947247	28.92569937	28.53554347	29.26248738	0.807666865	0.953	0.153	PIS54489.1	KRE6	Essential beta-1,6-glucan synthase subunit; change in mRNA length, not abundance, at yeast-hypha transition; alkaline induced by Rim101, on cell wall regeneration; Spider biofilm induced; Bcr1-repressed in RPMI a/a biofilms
PIS52023.1	32.08896613	32.3401298	31.14880623	31.43566588	32.30587934	32.29116501	0.686249899	0.944	0.152	PIS52023.1	orf19.3572.3	Ribosomal 60S subunit protein L31B; Spider biofilm repressed
PIS51144.1	32.627296	32.74892783	32.46397027	32.59671445	32.96803978	32.73059529	0.625626005	0.939	0.152	PIS51144.1	RPL24A	Predicted ribosomal protein; downregulated upon phagocytosis by murine macrophage; intron in 5'-UTR; Hap43-induced; Spider biofilm repressed

PIS54624.1	29.35465424	29.68535201	29.40592336	29.84113989	29.50376195	29.55584469	0.638819034	0.94	0.152	PIS54624.1	SSO2	Plasma membrane t-SNARE; involved in fusion of secretory vesicles at the plasma membrane
PIS48532.1	32.43543133	32.50531412	32.3161877	32.38875864	32.51479269	32.80732603	0.581545703	0.935	0.151	PIS48532.1	GLK1	Putative glucokinase; transcript regulated upon yeast-hyphal switch; Efg1 regulated; fluconazole-induced; induced in core stress response; colony morphology-related gene regulation by Ssn6; GlcNAc-induced protein
PIS48272.1	31.03348745	31.00044349	30.67679623	31.00040038	31.02558688	31.13664986	0.730017236	0.948	0.151	PIS48272.1	orf19.3003	Tricalbin-family endoplasmic reticulum-plasma membrane tethering protein; required for transport of cell wall proteins; involved in stress responses, including sensitivity to caspofungin
PIS48441.1	27.4132177	28.6540707	26.83469762	27.70927027	27.71621095	27.93080919	0.772486859	0.95	0.151	PIS48441.1	SOL3	Putative 6-phosphogluconolactonase; present in exponential and stationary growth phase yeast cultures; macrophage-downregulated protein; Spider biofilm repressed
PIS50565.1	29.78480838	28.96372716	29.54101983	29.7964941	29.58095751	29.36532994	0.770497098	0.95	0.151	PIS50565.1	URA7	CTP synthase 1; flucytosine induced; protein present in exponential and stationary growth phase yeast cultures
PIS51842.1	29.57918742	28.84847486	29.63657137	29.6026983	29.46391458	29.44537705	0.634722991	0.94	0.149	PIS51842.1	orf19.1764	Protein of unknown function; rat catheter and Spider biofilm induced
PIS50311.1	29.05723365	29.18694733	28.66790339	28.73884726	29.18242674	29.43749304	0.701101307	0.946	0.149	PIS50311.1	PRO1	Putative gamma-glutamyl kinase; transcript regulated by Nrg1; regulated by Gcn2 and Gcn4; Hap43-repressed gene; early-stage flow model biofilm induced gene
PIS50557.1	30.71163826	31.01759818	30.4519234	30.91578961	30.88703101	30.82422118	0.793537055	0.952	0.149	PIS50557.1	TIF5	Putative translation initiation factor; repressed upon phagocytosis by murine macrophage; Spider biofilm repressed
PIS58175.1	28.12914262	27.40753773	28.55956597	28.0261579	28.33803781	28.17637383	0.708717923	0.946	0.148	PIS58175.1	orf19.1114	Cytochrome c oxidase subunit; membrane-localized protein; rat catheter biofilm induced
PIS54541.1	32.66068525	31.81700542	32.79845913	32.77007971	32.51297243	32.43590844	0.684557862	0.944	0.148	PIS54541.1	orf19.3799	Ortholog(s) have role in ER-dependent peroxisome organization, endoplasmic reticulum inheritance, endoplasmic reticulum tubular network maintenance and endoplasmic reticulum tubular network organization, more

PIS58926.1	31.64888272	31.24017914	31.84938796	31.73576986	31.6792308	31.76848292	0.572615778	0.934	0.148	PIS58926.1	orf19.6082	Ortholog(s) have unfolded protein binding activity, role in protein folding, protein localization to cell surface and endoplasmic reticulum membrane localization
PIS50329.1	27.52389951	27.73294877	27.49630658	27.95175982	27.44468953	27.79942908	0.625693311	0.939	0.148	PIS50329.1	SPC3	Essential protein; similar to S. cerevisiae Spc3p, a component of the signal peptidase complex required for signal peptidase activity; predicted integral ER membrane protein; complements spc3 and suppresses sec61 S. cerevisiae mutants
PIS50547.1	32.02779201	32.02878849	31.20864939	31.39498556	32.09914446	32.21310827	0.714668673	0.947	0.147	PIS50547.1	GPM1	Phosphoglycerate mutase; surface protein that binds host complement Factor H and FHL-1; antigenic; fluconazole, or amino acid starvation (3-AT) induced, farnesol-repressed; Hap43, flow model biofilm induced; Spider biofilm repressed
PIS55565.1	25.55865544	25.93787038	25.3596771	25.99166087	25.64549521	25.65972728	0.693414777	0.945	0.147	PIS55565.1	MNN14	Predicted alpha-1,3-mannosyltransferase activity with a role in protein glycosylation; Hap43-repressed; Spider biofilm induced
PIS48432.1	23.7207544	25.33502864	23.29418537	25.11809581	23.37501714	24.29820526	0.813381472	0.953	0.147	PIS48432.1	orf19.2363	Ortholog(s) have thiosulfate sulfurtransferase activity and role in sulfur compound metabolic process
PIS58661.1	32.68277963	32.46500076	32.1808285	32.15313461	32.92646702	32.68902514	0.768857314	0.95	0.147	PIS58661.1	RPS20	Putative ribosomal protein; repressed upon phagocytosis by murine macrophage; transcript positively regulated by Tbf1; Spider biofilm repressed
PIS52388.1	26.26990956	26.09766024	26.25327504	25.62385124	25.91011221	27.52608802	0.824574442	0.953	0.146	PIS52388.1	orf19.5418	Ortholog(s) have role in endoplasmic reticulum to Golgi vesicle-mediated transport
PIS48607.1	28.4832762	28.27561382	28.78881975	28.87100776	28.47516569	28.63631103	0.827168787	0.954	0.145	PIS48607.1	orf19.2299	Ortholog(s) have protein tag activity
PIS50462.1	29.1676593	29.13409301	28.90690191	28.86781737	29.35411682	29.42166364	0.739950986	0.948	0.145	PIS50462.1	PAN1	Essential protein involved in endocytosis and polarized growth; ortholog of S. cerevisiae Pan1, which is a part of a complex that regulates actin cytoskeleton; Spider biofilm repressed
PIS58630.1	24.13909296	24.67773185	25.07087404	25.32038609	24.54614408	24.45324076	0.839900233	0.954	0.144	PIS58630.1	SCP1	Putative cortical actin cytoskeleton protein; constitutive expression independent of MTL or white-opaque status

PIS55734.1	29.01978082	28.54985412	28.86125928	29.28838437	28.57465389	29.00040793	0.810557772	0.953	0.144	PIS55734.1	SEC22	Ortholog(s) have SNAP receptor activity and role in endoplasmic reticulum to Golgi vesicle-mediated transport, retrograde vesicle-mediated transport, Golgi to endoplasmic reticulum, vesicle fusion, vesicle fusion with Golgi apparatus
PIS58849.1	24.10867903	25.08048282	24.17073278	24.05954693	24.54685935	25.18393436	0.760599148	0.95	0.143	PIS58849.1	orf19.1595	Ortholog(s) have clathrin binding activity, role in SREBP signaling pathway, clathrin-dependent endocytosis and Dsc E3 ubiquitin ligase complex, clathrin-coated vesicle localization
PIS58416.1	29.47106033	31.50366061	29.14151733	30.6039477	29.30468867	30.63464817	0.84008178	0.954	0.142	PIS58416.1	EFB1	Translation elongation factor EF-1 beta; repressed by human whole blood or PMNs; macrophage/pseudohyphal-induced; antigenic in mouse; farnesol-downregulated; snoRNA snR18 is encoded within the EFB1 intron; Spider biofilm
PIS55796.1	32.15346799	31.7752115	32.19022816	32.13915593	32.18482377	32.22125823	0.622317199	0.939	0.142	PIS55796.1	orf19.1672	Alpha subunit of COPI vesicle coatomer complex; role in ER to Golgi vesicle-mediated transport, retrograde vesicle-mediated transport, Golgi to ER transport; flow model biofilm repressed
PIS55813.1	29.47216133	29.90815476	29.08138969	29.11532404	29.86633323	29.90585272	0.757933819	0.95	0.142	PIS55813.1	orf19.7380	Has domain(s) with predicted nucleic acid binding activity
PIS52186.1	26.65557864	27.36501169	27.83962015	28.03375424	27.009095	27.23980284	0.75945036	0.95	0.141	PIS52186.1	MP65	Cell surface mannoprotein; cell-wall glucan metabolism, adhesion; adhesin motif; O-glycosylation; induced by heat, germ tube formation, wall regeneration; mycelial antigen; diagnostic marker; fluconazole-repressed; Spider biofilm
PIS49671.1	31.35045621	31.15179164	31.15709802	31.42426195	31.32755939	31.3310543	0.767249383	0.95	0.141	PIS49671.1	THR4	Putative threonine synthase; protein present in exponential and stationary growth phase yeast cultures; Gcn4p-regulated; sumoylation target
PIS56868.1	28.88347113	28.5155756	29.14437239	29.11803013	29.13149263	28.71767269	0.767052288	0.95	0.141	PIS56868.1	UBR1	Protein similar to S. cerevisiae Ubr1p ubiquitin-protein ligase; regulates stability of Ume6p in response to oxygen availability; transposon mutation affects filamentous growth; Spider biofilm induced
PIS51168.1	29.76976397	30.07925137	30.1185761	30.12901528	29.92874024	30.33392947	0.606737156	0.937	0.141	PIS51168.1	URA1	Dihydroorotate dehydrogenase; de novo pyrimidine biosynthesis; regulated by yeast-hypha switch, Nrg1/Mig1/Tup1; flow model biofilm induced; rat catheter and Spider biofilm repressed
PIS48299.1	23.81818366	24.26171958	24.2602805	24.29143461	24.27559739	24.1904795	0.789150017	0.951	0.139	PIS48299.1	BNI4	Protein required for wild-type cell wall chitin distribution, morphology, hyphal growth; not essential; similar to S. cerevisiae Bni4p (targeting subunit for Glc7p phosphatase, involved in bud-neck localization of chitin synthase III)

PIS50519.1	28.33892338	27.56366998	28.48620854	28.48968788	28.00752489	28.30894415	0.728780624	0.948	0.139	PIS50519.1	orf19.2677	Putative GPI transamidase component; possibly an essential gene, disruptants not obtained by UAU1 method
PIS51430.1	27.87158758	27.9058205	28.02772517	28.29877139	27.98175191	27.94162432	0.690375125	0.945	0.139	PIS51430.1	orf19.2852	Subunit of the mitochondrial ribosome; identified in a mutant screen as necessary for filamentation inside phagocytes
PIS58882.1	31.51976351	31.12664368	31.8327403	31.76391008	31.57558235	31.55072154	0.647427886	0.941	0.137	PIS58882.1	COX5	Cytochrome oxidase subunit V; putative upstream CCAAT box regulatory element; macrophage/pseudohyphal-induced; repressed by nitric oxide; intron in 5'-UTR; Hap43p-dependent repression in low iron medium
PIS58873.1	27.90534029	28.51652339	27.60065454	28.29487533	28.02934595	28.11049583	0.709127303	0.946	0.137	PIS58873.1	orf19.1300	Putative mitochondrial membrane protein; homozygous transposon insertion causes decreased colony wrinkling under filamentous growth-inducing conditions, but does not block true hyphal formation in liquid media
PIS50343.1	29.46510009	30.0088896	29.84305098	29.70170324	29.88102321	30.14394521	0.84318325	0.954	0.137	PIS50343.1	RIB4	Lumazine synthase (6,7-dimethyl-8-ribityllumazine synthase, DMRL synthase); catalyzes the penultimate step in the synthesis of riboflavin; Hap43-induced; rat catheter and Spider biofilm repressed
PIS58811.1	30.4433742	30.5605952	30.03426542	30.61355685	30.41721579	30.41965479	0.611119569	0.938	0.137	PIS58811.1	TCP1	Chaperonin-containing T-complex subunit, induced by alpha pheromone in SpiderM medium; stationary phase enriched protein
PIS48415.1	23.29304331	25.42012599	24.60069085	24.22260411	23.89137172	25.60640061	0.837374745	0.954	0.136	PIS48415.1	CWT1	Zn2Cys6 transcription factor involved in negative regulation of nitrosative stress response; regulates formation of mating projections and same-sex mating; mutant has cell wall defects; transcription increased at stationary phase
PIS58530.1	27.28324453	26.62518286	27.28049913	27.45182142	26.68110805	27.46363724	0.859454579	0.955	0.136	PIS58530.1	orf19.6453	Ortholog of C. dubliniensis CD36 : Cd36_72300, C. parapsilosis CDC317 : CPAR2_703060, C. auris B8441 : B9J08_001030 and Candida tenuis NRRL Y-1498 : CANTEDRAFT_92002
PIS48223.1	23.16265147	23.98147675	24.61582993	24.76752268	22.50394362	24.89798313	0.856313171	0.955	0.136	PIS48223.1	PRP8	Protein similar to S. cerevisiae Prp8, a component of the U4/U6-U5 snRNP complex; repressed by alpha pheromone in SpiderM medium
PIS58498.1	29.9187225	30.18639228	29.80003416	30.07482793	30.24487115	29.9934669	0.787320078	0.951	0.136	PIS58498.1	RPS21B	Ribosomal protein S21; regulated by Nrg1, Tup1; colony morphology-related gene regulation by Ssn6; positively regulated by Tbf1, Hap43; Spider biofilm repressed

PIS55456.1	27.35667866	27.12649162	27.11048548	27.15887441	27.35415783	27.48635286	0.783445456	0.951	0.135	PIS55456.1	orf19.3737	Protein with a Vps9 vacuolar protein sorting protein domain; Hap43-repressed; repressed by ciclopirox olamine; Spider biofilm induced
PIS54737.1	25.70624309	24.67451214	24.95838401	24.89368345	24.80627771	26.04397563	0.843487608	0.954	0.135	PIS54737.1	SHE3	mRNA-binding protein that localizes specific mRNAs to daughter yeast cells and to hyphal tips; required for normal filamentation and host epithelial cell damage; ortholog of S. cerevisiae She3 but target mRNAs differs
PIS54993.1	29.24709999	29.52752466	29.47164663	30.36510286	28.95993244	29.3221061	0.85779518	0.955	0.134	PIS54993.1	AHP2	Putative thiol-specific peroxiredoxin; macrophage-downregulated gene
PIS58285.1	24.73886202	24.56830694	25.05233312	23.84677372	25.38002672	25.53501382	0.816382309	0.953	0.134	PIS58285.1	orf19.3030	Ortholog(s) have phosphatidylinositol-3,5-bisphosphate binding, phosphatidylinositol-3-phosphate binding, phosphatidylinositol-4-phosphate binding activity
PIS58969.1	24.78626444	23.93602689	24.23852756	24.63341788	24.11838309	24.60746699	0.774377246	0.951	0.133	PIS58969.1	orf19.1950	Ortholog(s) have ADP-ribosyl-[dinitrogen reductase] hydrolase activity, phosphatase activity
PIS58150.1	29.58369709	29.96505416	29.47988846	29.45159947	29.98977787	29.98541428	0.727857897	0.947	0.133	PIS58150.1	orf19.3022	Ortholog(s) have structural constituent of ribosome activity and mitochondrial small ribosomal subunit localization
PIS49842.1	26.97991398	26.50604361	27.25896479	27.01912175	27.47294338	26.64953169	0.766122989	0.95	0.132	PIS49842.1	EXO70	Predicted subunit of the exocyst complex, involved in exocytosis; localizes to a crescent on the surface of the hyphal tip
PIS56931.1	30.60248289	30.06702939	30.49768349	30.72232032	30.62064832	30.22164292	0.912004049	0.958	0.132	PIS56931.1	PRE1	Putative beta 4 subunit of the 20S proteasome; Rim101-induced at acid pH; flucytosine induced; amphotericin B repressed
PIS54490.1	27.49797491	27.88021769	26.04920031	25.99548239	27.88511557	27.9394434	0.843504642	0.954	0.131	PIS54490.1	CEM1	Protein similar to S. cerevisiae Cem1p, an acyl carrier protein involved in fatty acid biosynthesis; likely to be essential for growth, based on an insertional mutagenesis strategy
PIS48186.1	28.12322659	28.14370175	28.24353294	28.06333219	28.53988005	28.29905996	0.697681961	0.945	0.131	PIS48186.1	orf19.6503	Ortholog(s) have structural constituent of ribosome activity, role in mitochondrial translation and mitochondrial large ribosomal subunit localization

PIS55083.1	28.34873168	28.67735652	28.53612655	28.5198236	28.74769163	28.68643022	0.839110654	0.954	0.131	PIS55083.1	orf19.73	Putative metalloprotease; associates with ribosomes and is involved in ribosome biogenesis; Spider biofilm induced
PIS51766.1	31.5516508	31.26754921	31.60085284	31.63080264	31.62010321	31.55984521	0.691049332	0.945	0.13	PIS51766.1	ACS2	Acetyl-CoA synthetase; antigenic during human and murine infection; induced by Efg1; macrophage-induced protein; soluble protein in hyphae; gene contains intron; flow model and Spider biofilm repressed
PIS58192.1	29.1518793	28.93005308	30.06059241	29.59575657	29.39174252	29.54455518	0.82303797	0.953	0.13	PIS58192.1	GLO2	Ortholog(s) have hydroxyacylglutathione hydrolase activity, role in methylglyoxal catabolic process to D-lactate via S-lactoyl-glutathione and cytoplasm localization
PIS51953.1	27.91171731	28.35004716	28.92919518	29.49078204	28.36572622	27.72523248	0.854917873	0.955	0.13	PIS51953.1	NOP13	Ortholog of S. cerevisiae Nop13; a nucleolar protein found in preribosomal complexes; Hap43-induced gene; rat catheter biofilm induced
PIS58670.1	24.9805444	25.25796668	24.84877416	24.66229735	25.5340404	25.27961698	0.779937891	0.951	0.13	PIS58670.1	PGA31	Cell wall protein; putative GPI anchor; expression regulated upon white-opaque switch; induced by Congo Red and cell wall regeneration; Bcr1-repressed in RPMI a/a biofilms
PIS55831.1	27.94419394	28.54377337	28.35686884	29.3372915	27.7718923	28.12365845	0.776451437	0.951	0.129	PIS55831.1	NIT3	Putative nitrilase; regulated by Gcn2p and Gcn4p; protein present in exponential and stationary growth phase yeast cultures
PIS55005.1	28.58857468	28.94527542	27.94987989	28.51712556	28.63600047	28.71873274	0.694075246	0.945	0.129	PIS55005.1	orf19.3286	Ortholog(s) have alpha-1,4-glucosidase activity, role in N-glycan processing, polysaccharide biosynthetic process and endoplasmic reticulum lumen, glucosidase II complex localization
PIS58284.1	30.77854089	30.53412847	30.8249812	30.96148535	30.80905837	30.75269017	0.685443039	0.944	0.129	PIS58284.1	SEC62	Putative endoplasmic reticulum (ER) protein-translocation complex subunit
PIS55649.1	26.01538733	24.90587458	26.06305211	25.81827488	25.77283433	25.77835204	0.700803087	0.946	0.128	PIS55649.1	orf19.6730	Ortholog(s) have RNA binding activity, role in maturation of LSU-rRNA, ribosomal large subunit biogenesis and nucleolus localization
PIS49574.1	23.73198957	25.95619696	25.21744699	25.34329719	24.98218238	24.95972905	0.860918538	0.955	0.127	PIS49574.1	PRP3	Predicted splicing factor, component of the U4/U6-U5 snRNP complex; Hap43-induced gene; rat catheter biofilm induced

PIS55491.1	30.29042597	30.43194597	30.49358834	30.61246477	30.22201981	30.76054687	0.734579322	0.948	0.126	PIS55491.1	GAD1	Putative glutamate decarboxylase; alkaline, macrophage-downregulated gene; amphotericin B induced; induced by Mnl1 under weak acid stress; stationary phase enriched protein; rat catheter biofilm repressed
PIS51117.1	31.79371015	31.16386967	32.03118768	32.13560407	31.59553134	31.63437706	0.76201849	0.95	0.126	PIS51117.1	SDH2	Succinate dehydrogenase, Fe-S subunit; localizes to surface of yeast cells, but not hyphae; induced in high iron and during log phase aerobic growth; repressed by nitric oxide, Hap43
PIS58543.1	27.74837426	27.74326701	27.8902421	28.03146745	27.89637252	27.8291329	0.74154751	0.948	0.125	PIS58543.1	orf19.3351	Protein of unknown function; Hap43-induced; Spider biofilm induced
PIS58203.1	30.55149645	30.38679779	30.31471358	30.25056472	30.77520247	30.60068196	0.753050446	0.949	0.124	PIS58203.1	CRM1	Functional homolog of S. cerevisiae Crm1, which acts in protein nuclear export; predicted to be resistant to antifungal drug leptomycin B; partially suppresses signal transduction defects of S. cerevisiae ste20 mutant
PIS58755.1	28.0152937	28.61402342	26.87395421	27.91044747	28.13111293	27.83517751	0.819671286	0.953	0.124	PIS58755.1	orf19.1637	Ortholog of C. dubliniensis CD36 : Cd36_82070, C. parapsilosis CDC317 : CPAR2_202790, C. auris B8441 : B9J08_000205 and Candida tenuis NRRL Y-1498 : CANTEDRAFT_114065
PIS52002.1	27.78728098	28.14119892	27.6321795	27.49088028	28.20301534	28.24006617	0.694685738	0.945	0.124	PIS52002.1	orf19.5114	Sorting nexin; role in maintaining late-Golgi resident enzymes in their proper location by recycling molecules from the prevacuolar compartment; Spider biofilm induced
PIS58652.1	29.26198265	28.85129391	29.41575293	29.572705	29.18183929	29.14618898	0.818902526	0.953	0.124	PIS58652.1	VPS21	Late endosomal Rab small monomeric GTPase involved in transport of endocytosed proteins to the vacuole; involved in filamentous growth and virulence; Spider biofilm induced
PIS48624.1	33.47393784	33.23422422	33.36983465	33.324215	33.60477067	33.51597633	0.694336203	0.945	0.122	PIS48624.1	YST1	Ribosome-associated protein; antigenic in mice; complements S. cerevisiae yst1 yst2 mutant; similar to laminin receptor; predicted S/T phosphorylation, N-glycosylation, myristoylation, Hap43-, Gcn4-regulated; Spider biofilm repressed
PIS54872.1	30.91986369	30.44758154	31.27812861	31.22148347	31.00751304	30.78093912	0.704208615	0.946	0.121	PIS54872.1	MRS7	Member of the LETM1-like protein family, mitochondrial membrane protein
PIS58134.1	27.94585741	28.4703378	27.93796742	27.88098063	28.35355746	28.48299017	0.790292309	0.952	0.121	PIS58134.1	orf19.3010	Putative lipoyl ligase; role in modification of mitochondrial enzymes by attachment of lipoic acid groups; rat catheter biofilm induced

PIS58120.1	27.48568402	25.82608484	27.47386705	27.46231365	26.7868841	26.8999093	0.78305505	0.951	0.121	PIS58120.1	orf19.6264	Ortholog of C. dubliniensis CD36 : Cd36_06080, C. parapsilosis CDC317 : CPAR2_803360, C. auris B8441 : B9J08_000608 and Candida tenuis NRRL Y-1498 : CANTEDRAFT_112428
PIS51130.1	30.30187776	30.73329019	30.643424	30.46815433	30.50798019	31.06544691	0.771948562	0.95	0.121	PIS51130.1	orf19.7140	Putative catechol o-methyltransferase; stationary phase enriched protein; transcription upregulated in clinical isolates from HIV+ patients with oral candidiasis; Spider biofilm repressed
PIS52450.1	34.42822003	34.38713682	34.43255288	34.57421503	34.67930544	34.35360393	0.647302738	0.941	0.12	PIS52450.1	RPS5	Ribosomal protein S5; macrophage/pseudohyphal-induced after 16 h; downregulated upon phagocytosis by murine macrophage; Hap43-induced; Spider biofilm repressed
PIS48136.1	32.33231619	32.78214462	32.94351836	32.88938391	32.4847628	33.04195542	0.843533443	0.954	0.119	PIS48136.1	ALD6	Putative aldehyde dehydrogenase; stationary phase enriched protein; expression regulated upon white-opaque switch; rat catheter biofilm induced; rat catheter and Spider biofilm induced
PIS48814.1	30.99349803	30.56147043	31.1514666	30.74562288	31.16961575	31.1481156	0.888194805	0.957	0.119	PIS48814.1	orf19.2114	Predicted uricase; ortholog of S. pombe SPCC1223.09; Spider biofilm induced
PIS56888.1	28.49015497	27.91779064	28.76577507	28.66024602	28.64909443	28.2195728	0.687590928	0.944	0.118	PIS56888.1	MRT4	Putative mRNA turnover protein; Hap43-induced; mutation confers hypersensitivity to tubercidin (7-deazaadenosine); rat catheter biofilm induced
PIS48572.1	29.30659862	29.34447263	29.0884637	29.04907295	29.56202038	29.48258082	0.743509876	0.949	0.118	PIS48572.1	RBF1	Transcription factor; glutamine-rich activation domain; binds RPG-box DNA sequences; predominantly nuclear; mutation causes accelerated induction of filamentous growth; antigenic during human oral infection; Sko1p-repressed
PIS52136.1	32.23657902	31.92583654	32.42597123	32.54697171	32.27927544	32.10962055	0.70459523	0.946	0.116	PIS52136.1	ATP20	Putative mitochondrial ATP synthase; shows colony morphology-related gene regulation by Ssn6p; flucytosine induced; caspofungin repressed; macrophage/pseudohyphal-induced
PIS51693.1	30.44188718	30.03188047	30.76896854	30.77447399	30.62548309	30.19081753	0.719699803	0.947	0.116	PIS51693.1	GLN4	Putative tRNA-Gln synthetase; genes encoding ribosomal subunits, translation factors, tRNA synthetases are downregulated upon phagocytosis by murine macrophage; protein present in exponential and stationary growth phase yeast cultures
PIS48698.1	23.76318	24.79399796	25.93205773	24.5200297	25.30989389	25.00612026	0.863768737	0.956	0.116	PIS48698.1	orf19.131.2	Has domain(s) with predicted protein folding chaperone activity, role in protein insertion into ER membrane and endoplasmic reticulum membrane localization

PIS58457.1	34.0034178	33.6723705	34.19981955	34.30728408	34.08913205	33.82830573	0.67714321	0.944	0.116	PIS58457.1	orf19.3690.2	Ribosomal 60S subunit protein; Spider biofilm repressed
PIS55467.1	30.67646058	31.16691056	30.52913949	30.5079799	31.28346179	30.92991645	0.749714778	0.949	0.116	PIS55467.1	orf19.5281	Predicted essential RNA-binding G protein; ortholog an effector of mating response pathway in S. cerevisiae; mainly associated with nuclear envelope and ER; flow model and Spider biofilm repressed
PIS48487.1	28.99840274	28.46368018	29.20624589	29.17465694	29.01688389	28.8219912	0.694582884	0.945	0.115	PIS48487.1	MRS4	Mitochondrial carrier family member, involved in iron homeostasis; putative membrane transporter localized to the mitochondrial membrane; transcription under control of Atf2p; regulated by Sef1p, Sfu1p, and Hap43p
PIS58699.1	27.30141487	27.12614531	27.43520909	27.22492981	27.51454487	27.4696487	0.787714764	0.951	0.115	PIS58699.1	orf19.1477	Protein of unknown function; possible ER protein; Hap43p-repressed; Spider biofilm induced
PIS48411.1	31.48816654	31.23729401	31.80739285	31.82802984	31.43095627	31.61757793	0.748807937	0.949	0.115	PIS48411.1	orf19.3518	Ortholog(s) have dicarboxylic acid transmembrane transporter activity, role in mitochondrial transport and mitochondrial inner membrane, mitochondrion localization
PIS51986.1	26.34867888	27.80324156	27.23765349	27.54843086	26.90594164	27.27597853	0.785918183	0.951	0.114	PIS51986.1	orf19.3366.1	Protein of unknown function; Hap43-repressed gene
PIS51647.1	27.86239192	27.91658819	27.09278683	26.71324384	28.3397088	28.15850563	0.855880187	0.955	0.113	PIS51647.1	orf19.6039	Ortholog(s) have SNAP receptor activity
PIS55504.1	27.63277559	28.049238	27.07402044	27.60551882	28.08070834	27.40872886	0.831327584	0.954	0.113	PIS55504.1	SMI1	Cell wall biosynthesis protein; Hap43, caspofungin-repressed; Cyr1-induced in hyphal cells; reduced biofilm cell wall glucan in mutant; possibly essential (UAU1 method); flow model biofilm induced
PIS49597.1	26.1194396	24.95290105	25.63718779	23.5791945	26.83817776	26.62693603	0.881437516	0.956	0.112	PIS49597.1	orf19.7285	Ortholog(s) have role in co-transcriptional mRNA 3'-end processing, cleavage and polyadenylation pathway, mRNA processing, sno(s)RNA 3'-end processing, termination of RNA polymerase II transcription, exosome-dependent
PIS48666.1	31.07591826	31.1387108	31.07309381	31.12703821	31.1876844	31.30996104	0.76355411	0.95	0.112	PIS48666.1	SRV2	Adenylate cyclase-associated protein; regulates adenylate cyclase activity; required for wild-type germ tube formation and for virulence in mice; mutant defects in filamentous growth are rescued by cAMP or dbcAMP; Spider biofilm repressed

PIS55716.1	25.88944627	25.94284568	25.13187812	25.32517756	25.79956707	26.16798883	0.84341117	0.954	0.11	PIS55716.1	ACP1	Putative mitochondrial acyl carrier protein involved in fatty acid biosynthesis; shows colony morphology-related gene regulation by Ssn6p; protein newly produced during adaptation to the serum
PIS58824.1	25.45944459	26.31459679	25.29880227	25.41362779	26.0997347	25.8907012	0.78270512	0.951	0.11	PIS58824.1	orf19.7182	Ortholog(s) have RNA binding activity and role in tRNA modification
PIS48710.1	24.4426669	25.07374186	24.5011712	24.51384128	24.39505226	25.43611644	0.847614607	0.955	0.109	PIS48710.1	GCN2	Translation initiation factor 2-alpha (eIF2alpha) kinase; has nonessential role in amino acid starvation response, in contrast to S. cerevisiae homolog; similar to S. cerevisiae Gcn2p
PIS52363.1	25.06222931	26.69347052	26.14469306	25.88434878	25.73626858	26.60529771	0.869692876	0.956	0.109	PIS52363.1	SDH4	Succinate dehydrogenase, membrane subunit; induced in high iron
PIS51302.1	28.26354743	27.64993436	27.72745067	27.9869536	27.84883091	28.13038894	0.778609014	0.951	0.108	PIS51302.1	PMT6	Protein mannosyltransferase; required for virulence in mice, adhesion to endothelium; role in hyphal growth signaling, hygromycin B sensitivity; no major role in cellular PMT activity; Hap4-repressed; Bcr1-repressed in RPMI a/a biofilms
PIS49518.1	28.71395736	26.96716245	27.28383759	27.86077438	27.2064217	28.21972858	0.90047954	0.957	0.107	PIS49518.1	CHS6	Protein with tetratricopeptide repeats (TPRs); ortholog of S. cerevisiae Chs6, which has role in localizing chitin synthase; Cyr1-regulated in hyphae; possibly essential, disruptants not obtained by UAU1 method; flow model biofilm repressed
PIS48251.1	29.90673661	30.16587439	29.53888652	29.90618218	30.17219927	29.85337032	0.870755662	0.956	0.107	PIS48251.1	CRN1	Coronin; cortical actin cytoskeletal component; predicted role in regulation of actin patch assembly; rat catheter and Spider biofilm repressed
PIS48246.1	30.87594474	30.87240807	30.6618646	30.69585544	30.8653544	31.16865792	0.735718392	0.948	0.107	PIS48246.1	FAA2-3	Predicted acyl CoA synthetase
PIS51031.1	33.00534289	33.13005323	32.9146806	32.95565109	33.26166033	33.15376256	0.772362941	0.95	0.107	PIS51031.1	RPL10	Ribosomal protein L10; intron in 5'-UTR; downregulated upon phagocytosis by murine macrophage; transcription regulated by yeast-hypha switch; Spider biofilm repressed
PIS51195.1	29.87368445	29.86653236	29.92873306	29.96616792	29.86618063	30.15476966	0.739076455	0.948	0.106	PIS51195.1	FDH3	Glutathione-dependent formaldehyde dehydrogenase; glycine catabolism; repressed by Efg1 in yeast, not hyphal growth conditions; induced by Mnl1 under weak acid stress; Spider biofilm repressed

PIS51972.1	30.63630731	30.59921459	29.60465922	29.82260822	30.72711931	30.60952741	0.894776495	0.957	0.106	PIS51972.1	GYP5	Putative Rab GTPase activator; role in ER to Golgi vesicle-mediated transport; Spider biofilm induced
PIS58662.1	29.82599289	29.38786342	29.50632736	29.24958804	29.90620957	29.88271785	0.791083322	0.952	0.106	PIS58662.1	PTC5	Mitochondrial protein phosphatase of the Type 2C-related family (serine/threonine-specific), involved in drug response and cadmium tolerance
PIS48591.1	24.83555655	24.56114105	24.82976358	25.03437743	25.20470123	24.30218686	0.904830514	0.957	0.105	PIS48591.1	orf19.1400	Ortholog of C. dubliniensis CD36 : Cd36_23640, C. parapsilosis CDC317 : CPAR2_406130, C. auris B8441 : B9J08_005288 and Candida tenuis NRRL Y-1498 : CANTEDRAFT_116273
PIS58234.1	26.99881397	26.77247647	27.448343	27.11275605	27.28953482	27.13265689	0.76196932	0.95	0.105	PIS58234.1	orf19.4764	Ortholog(s) have poly(A)-specific ribonuclease activity, role in nuclear-transcribed mRNA poly(A) tail shortening, postreplication repair and PAN complex localization
PIS58537.1	25.99537179	27.06941661	24.94999591	25.41182638	26.22452763	26.69275343	0.83444415	0.954	0.105	PIS58537.1	TOM22	Putative mitochondrial import receptor subunit; colony morphology-related gene regulation by Ssn6
PIS48263.1	25.70348373	25.25345314	23.74877427	25.34609785	24.72664899	24.94749535	0.885439283	0.957	0.105	PIS48263.1	VPS27	Putative ESCRT-0 complex protein with a role in multivesicular body (MVB) trafficking
PIS49684.1	30.56677581	30.8121589	30.30432217	30.45582563	30.57189826	30.96876708	0.700968315	0.946	0.104	PIS49684.1	NCP1	NADPH-cytochrome P450 reductase, acts with Erg11p in sterol 14 alpha-demethylation in ergosterol biosynthesis; subject to hypoxic regulation; ketoconazole-induced; caspofungin repressed
PIS55466.1	28.05582117	29.09833584	27.70325836	28.21370689	28.31310594	28.63884554	0.845118308	0.955	0.103	PIS55466.1	orf19.92	Protein that stimulates actin assembly; interacts with polarisome components Bni1p and Bud6p; Hap43-repressed; induced by prostaglandins
PIS58586.1	29.2596562	28.80190844	29.09773786	29.28937885	28.9874642	29.19099772	0.773490281	0.95	0.103	PIS58586.1	PXA1	Putative peroxisomal, half-size adrenoleukodystrophy protein (ALD or ALDp) subfamily ABC family transporter
PIS55766.1	28.15016891	29.48067367	27.07356067	27.66599581	28.80627295	28.54239993	0.85777264	0.955	0.103	PIS55766.1	TIF11	Translation initiation factor eIF1a; possibly transcriptionally regulated upon hyphal formation; genes encoding ribosomal subunits, translation factors, and tRNA synthetases are downregulated upon phagocytosis by murine macrophage

PIS51763.1	32.20954293	31.92017574	32.26424378	32.29742846	32.18951338	32.21047623	0.795387454	0.952	0.101	PIS51763.1	orf19.1054	Ortholog(s) have role in endoplasmic reticulum tubular network membrane organization, mitotic spindle pole body insertion into the nuclear envelope and mitotic spindle pole body localization, more
PIS58092.1	28.37267108	28.3112094	28.34658494	28.23016032	28.37857201	28.72302785	0.84989277	0.955	0.1	PIS58092.1	orf19.1985	Has aminoglycoside phosphotransferase and protein kinase domains; rat catheter and flow model biofilm induced
PIS50315.1	26.77168787	26.64723836	26.65803457	27.10237951	26.66714972	26.60821629	0.855163701	0.955	0.1	PIS50315.1	UTP9	Small-subunit processome protein; Ssr1-induced; repressed by prostaglandins; physically interacts with TAP-tagged Nop1
PIS48371.1	24.47770171	24.26464981	24.00253603	25.11673389	23.22019392	24.70701071	0.871307225	0.956	0.0997	PIS48371.1	DAO2	Putative D-amino acid oxidase; rat catheter biofilm induced
PIS52387.1	28.90315569	28.93712709	28.19874154	28.25886378	29.04183994	29.03728043	0.812630916	0.953	0.0997	PIS52387.1	DOT5	Putative nuclear thiol peroxidase; alkaline downregulated; sumoylation target; Spider and flow model biofilm induced
PIS54660.1	27.23162	27.22485436	27.958704	27.63706651	27.10952007	27.96773668	0.833679428	0.954	0.0997	PIS54660.1	orf19.6602	Ortholog(s) have role in ubiquinone biosynthetic process, ubiquinone-6 biosynthetic process and mitochondrial inner membrane, mitochondrion localization
PIS52175.1	28.03836547	28.23337468	27.58384364	28.23735887	27.9373019	27.97904114	0.855122585	0.955	0.0994	PIS52175.1	orf19.6705	Putative guanyl nucleotide exchange factor with Sec7 domain; required for normal filamentous growth; regulated by yeast-hyphal switch; filament induced; regulated by Nrg1, Tup1, Mob2, Hap43; mRNA binds She3; Spider biofilm induced
PIS54997.1	28.65233736	28.25535004	28.82998173	28.69071756	28.61214731	28.73181997	0.718480852	0.947	0.099	PIS54997.1	SEC1	Ortholog(s) have SNARE binding activity, role in exocytosis, positive regulation of vesicle fusion, vesicle docking involved in exocytosis and cellular bud neck, cellular bud tip, plasma membrane, prospore membrane localization
PIS48470.1	32.65753055	32.28602445	32.96950252	32.73481407	32.74827717	32.72663012	0.790922107	0.952	0.0989	PIS48470.1	FBP1	Fructose-1,6-bisphosphatase; key gluconeogenesis enzyme; regulated by Efg1, Ssn6; induced by phagocytosis; effects switch from glycolysis to gluconeogenesis in macrophage; rat flow model biofilm induced; overlaps orf19.6179
PIS51790.1	32.02538924	31.24543412	32.00580924	32.01823724	31.84949897	31.70527242	0.783236337	0.951	0.0988	PIS51790.1	RHO1	Small GTPase of Rho family; regulates beta-1,3-glucan synthesis activity and binds Gsc1p; essential; expected to be geranylgeranylated by geranylgeranyltransferase type I; plasma membrane-localized

PIS54789.1	28.20015585	28.06348432	28.46173874	28.50401955	28.19306973	28.32276354	0.869297095	0.956	0.0982	PIS54789.1	orf19.547	Ortholog(s) have 5'-3' DNA exonuclease activity, 5'-3' exonuclease activity, 5'-flap endonuclease activity, double-stranded DNA 5'-3' exodeoxyribonuclease activity, single-stranded DNA 5'-3' DNA exonuclease activity
PIS52451.1	28.47739451	28.5593735	28.2695807	28.76973401	28.11577073	28.71387007	0.789691063	0.951	0.0977	PIS52451.1	VPS4	AAA-ATPase involved in transport from MVB to the vacuole and ESCRT-III complex disassembly; mutation decreases SAP secretion and virulence in murine intravenous infection; regulated by Gcn2p, Gcn4p; required for normal Rim8p processing
PIS48326.1	28.67449231	28.80710062	28.65344134	28.79164191	28.830836	28.80359759	0.81644495	0.953	0.097	PIS48326.1	orf19.3552	Ortholog(s) have structural constituent of nuclear pore activity
PIS52444.1	27.69986411	26.6208316	27.85108838	27.7712419	27.74257267	26.94845469	0.836945837	0.954	0.0968	PIS52444.1	orf19.7370	Possible G-protein coupled receptor; vacuolar membrane transporter for cationic amino acids; PQ-loop motif; rat catheter and Spider biofilm induced
PIS51394.1	27.65404722	27.29603781	27.01722259	26.95105995	27.81237841	27.48892548	0.919971782	0.958	0.095	PIS51394.1	orf19.4398	Protein of unknown function; rat catheter biofilm induced
PIS52270.1	30.95309098	30.37140957	30.52770923	30.4868882	30.74166961	30.90701897	0.754541058	0.949	0.0945	PIS52270.1	orf19.4066	Putative glycerol-3-phosphate acyltransferase; Hog1-repressed
PIS50528.1	27.69427019	27.77894807	27.84086354	27.90440289	27.77208459	27.9207268	0.802085313	0.952	0.0944	PIS50528.1	orf19.3914	Has domain(s) with predicted RNA binding, translation initiation factor activity, role in translational initiation and cytoplasm localization
PIS52204.1	26.87165916	26.08249274	26.97348129	26.54308402	26.82507341	26.84259937	0.875512762	0.956	0.0944	PIS52204.1	orf19.6931	Putative cleavage factor I subunit; heterozygous null mutant exhibits hypersensitivity to parnafungin and cordycepin in the C. albicans fitness test
PIS51141.1	29.90762946	30.26936213	30.10660646	29.99908743	30.31804326	30.24911224	0.762915952	0.95	0.0942	PIS51141.1	orf19.5126	Putative adhesin-like protein
PIS51815.1	26.74382845	27.37468032	27.41406389	27.26096415	27.56563346	26.98827403	0.828047923	0.954	0.0941	PIS51815.1	orf19.6818	Has domain(s) with predicted ATP binding, nucleic acid binding activity

PIS48512.1	30.31288519	29.9455275	30.55033958	30.31308807	30.45027121	30.32740069	0.816589414	0.953	0.094	PIS48512.1	orf19.5293	Ortholog of C. dubliniensis CD36 : Cd36_43710, C. parapsilosis CDC317 : CPAR2_402940, C. auris B8441 : B9J08_005206 and Candida tenuis NRRL Y-1498 : CANTEDRAFT_114940
PIS58363.1	26.56220941	27.96611413	26.43746599	26.9714369	27.11138647	27.16388572	0.889949143	0.957	0.0936	PIS58363.1	TAF14	YEATS domain-containing protein involved in transcription regulation; TFIIF and TFIID subunit; detects crotonylated lysine residues in histone H3; macrophage-induced protein; possibly required for yeast cell adherence to silicone substrate
PIS51035.1	26.27745328	26.70052645	26.1402941	25.8453973	26.94251808	26.60989583	0.816046093	0.953	0.0932	PIS51035.1	orf19.6406	Ortholog(s) have structural constituent of ribosome activity and mitochondrial small ribosomal subunit, peroxisome localization
PIS51936.1	24.04332734	25.61029444	25.0958695	24.5033074	24.84729966	25.67829318	0.90114875	0.957	0.0931	PIS51936.1	orf19.5869	Ortholog(s) have role in metal ion transport, protein targeting to vacuole, ubiquitin-dependent protein catabolic process and endoplasmic reticulum, fungal-type vacuole, fungal-type vacuole membrane localization
PIS51433.1	24.6235417	24.04967307	24.46207483	23.98141219	25.13710867	24.29395695	0.891786696	0.957	0.0924	PIS51433.1	SRP40	Putative chaperone of small nucleolar ribonucleoprotein particles; macrophage/pseudohyphal-induced; rat catheter biofilm induced
PIS51483.1	32.58992682	32.47936298	32.75198538	32.72352741	32.76343936	32.61057324	0.708942654	0.946	0.0921	PIS51483.1	CHC1	Clathrin heavy chain; subunit of the major coat protein; role in intracellular protein transport and endocytosis; flow model and rat catheter biofilm repressed
PIS54940.1	27.4834498	27.59910244	27.39272959	27.07000312	26.78316931	28.89793303	0.868383354	0.956	0.0919	PIS54940.1	IFG3	Putative D-amino acid oxidase; Spider biofilm induced
PIS48540.1	29.16090281	28.71713368	29.05583938	29.24709292	29.24051379	28.72065467	0.783151384	0.951	0.0915	PIS48540.1	orf19.4627	Ortholog(s) have structural constituent of nuclear pore activity
PIS52010.1	23.0025228	25.93831369	23.04133392	22.69372359	23.27473121	26.28770064	0.95498043	0.96	0.0913	PIS52010.1	PLB5	Putative GPI-linked phospholipase B, fungal-specific (no mammalian homolog); null mutation eliminates cell-associated phospholipase A2 activity and attenuates virulence; fluconazole-repressed; flow model biofilm repressed
PIS48650.1	28.47859456	28.62370278	28.25821741	28.38601856	28.50432758	28.74129285	0.758795367	0.95	0.0904	PIS48650.1	CWH43	Putative sensor/transporter protein with a predicted role in cell wall biogenesis; possibly an essential gene, disruptants not obtained by UAU1 method; flow model and rat catheter biofilm repressed

PIS58925.1	31.52297876	31.87194422	31.44010137	31.44864658	31.78170078	31.87542689	0.802160821	0.952	0.0902	PIS58925.1	MET6	Essential 5-methyltetrahydropteroyltriglutamate-homocysteine methyltransferase (cobalamin-independent methionine synthase); antigenic in murine/human systemic infection; heat shock, estrogen, GCN-induced; Spider biofilm repressed
PIS48639.1	30.05702185	30.16857276	30.05132747	30.03967526	29.98998973	30.51743287	0.816727791	0.953	0.0901	PIS48639.1	orf19.1632	Has domain(s) with predicted role in carbohydrate metabolic process
PIS54947.1	25.21424215	25.11089635	24.86345576	25.1463306	25.18392613	25.12750156	0.892158461	0.957	0.0897	PIS54947.1	CRZ1	Calcineurin-regulated C2H2 transcription factor; role in maintenance of membrane integrity, azole tolerance; not required for mouse virulence; repressed by low iron; regulates Ca++ influx during alkaline pH response; Spider biofilm induced
PIS58137.1	30.15026771	30.02211107	30.59470976	30.6045338	30.12983157	30.30062623	0.871805223	0.956	0.0893	PIS58137.1	orf19.4468	Putative succinate dehydrogenase; localized to the mitochondrial membrane; Hap43p-repressed gene
PIS54979.1	25.23253531	26.01598032	25.98655442	25.82392919	26.19557277	25.48312992	0.81336018	0.953	0.0892	PIS54979.1	orf19.4278	Ortholog(s) have chromatin binding, methylated histone binding activity and role in negative regulation of transcription by RNA polymerase II
PIS56748.1	28.50289551	28.7938975	27.82236364	28.46767389	28.31612247	28.60259237	0.875839658	0.956	0.0891	PIS56748.1	NPL6	Component of the RSC chromatin remodeling complex; Hap43-induced; Spider biofilm repressed
PIS56798.1	28.55464076	29.06407058	28.79759383	28.86031994	28.7371519	29.0856638	0.870150972	0.956	0.0889	PIS56798.1	RAD3	Ortholog of S. cerevisiae Rad3; 5' to 3' DNA helicase, nucleotide excision repair and transcription, subunit of RNA polII initiation factor TFIIH and Nucleotide Excision Repair Factor 3 (NEF3)
PIS51991.1	24.67801256	26.10022825	24.25781294	24.76601912	25.58035627	24.95498464	0.875328755	0.956	0.0884	PIS51991.1	orf19.6234	Putative U2 snRNP component; mutation confers hypersensitivity to 5-fluorocytosine (5-FC), 5-fluorouracil (5-FU), and tubercidin (7-deazaadenosine); Hap43-induced, Spider biofilm induced
PIS58531.1	28.68329541	29.53679434	28.98879656	29.76353203	28.77342039	28.9370279	0.894982833	0.957	0.0884	PIS58531.1	RBP1	Peptidyl-prolyl cis-trans isomerase; rapamycin-binding protein; homozygous null mutation confers rapamycin resistance; regulated by Gcn4p; macrophage-induced protein; repressed in response to 3-AT; functional homolog of S. cerevisiae Rbp1p
PIS56886.1	31.73092212	31.50321361	31.58251712	31.57343934	31.75774536	31.75003502	0.828203742	0.954	0.0882	PIS56886.1	SAC6	Fimbrin; actin filament bundling protein; involved in hyphal growth, oxidative stress response and virulence; transcript regulated by Nrg1 and Mig1; protein level decreases in stationary phase

PIS51055.1	24.38232556	25.00853135	25.12336209	24.5218955	25.06039712	25.19562611	0.902399432	0.957	0.0879	PIS51055.1	orf19.5876	Protein of unknown function; Cyr1-repressed; induced by alpha pheromone in SpiderM medium; rat catheter and Spider biofilm induced
PIS51192.1	25.96549122	24.8426022	25.41762253	25.63669865	25.30812824	25.54375986	0.855704612	0.955	0.0876	PIS51192.1	orf19.7604	Ortholog(s) have guanyl-nucleotide exchange factor activity, role in early endosome to Golgi transport, intra-Golgi vesicle-mediated transport and TRAPPII protein complex, early endosome, trans-Golgi network localization
PIS48681.1	28.99832448	29.71241975	28.63982143	28.97041373	29.37041725	29.27141582	0.898724568	0.957	0.0872	PIS48681.1	FMP27	Putative mitochondrial protein; mRNA binds She3
PIS48320.1	27.58496415	27.81238538	27.95054632	28.19391747	27.75153709	27.66369348	0.920701343	0.958	0.0871	PIS48320.1	RPC40	Putative RNA polymerase; protein level decreases in stationary phase cultures; Hap43p-induced gene
PIS50444.1	29.76228904	29.82004542	30.09730307	30.00080937	29.88190937	30.05789464	0.929032504	0.959	0.087	PIS50444.1	orf19.5660.1	Ortholog(s) have proton-transporting ATP synthase activity, rotational mechanism, structural molecule activity and role in cristae formation, protein-containing complex assembly, proton motive force-driven ATP synthesis
PIS54967.1	31.42074821	31.24506631	31.36380888	31.35466655	31.499533	31.43475447	0.734204539	0.948	0.0864	PIS54967.1	DED81	Putative tRNA-Asn synthetase; genes encoding ribosomal subunits, translation factors, tRNA synthetases are downregulated upon phagocytosis by murine macrophage; protein enriched in stationary phase yeast cultures
PIS58996.1	31.86413666	31.36054297	32.06402709	31.99969309	31.86336646	31.68407201	0.808826297	0.953	0.0861	PIS58996.1	EMP24	COPII-coated vesicle component
PIS51218.1	28.18124434	28.01641528	27.94813171	27.83523705	28.42457304	28.14430102	0.837554819	0.954	0.0861	PIS51218.1	RCK2	Predicted MAP kinase-activated protein kinase, similar to S. cerevisiae serine/threonine protein kinase Rck2p; induced by osmotic stress via Hog1p; macrophage/pseudohyphal-repressed; mutants are sensitive to rapamycin
PIS54852.1	22.82738064	24.45447677	25.26864943	24.54945319	23.87045489	24.38543574	0.939272954	0.959	0.0849	PIS54852.1	orf19.4478	Ortholog(s) have aspartate-tRNA ligase activity, role in mitochondrial aspartyl-tRNA aminoacylation and mitochondrion localization
PIS50530.1	20.52496055	22.51058345	20.27091961	20.12442601	21.63920075	21.79572245	0.896833727	0.957	0.0843	PIS50530.1	orf19.3916	Ortholog of C. dubliniensis CD36 : Cd36_53980, C. parapsilosis CDC317 : CPAR2_100980, C. auris B8441 : B9J08_004358 and Candida tenuis NRRL Y-1498 : CANTEDRAFT_116966

PIS58807.1	31.06229846	31.57998357	30.28423382	30.9368479	31.00206726	31.24022023	0.958473048	0.96	0.0842	PIS58807.1	GCD6	Ortholog of S. cerevisiae Gcd6; catalytic epsilon subunit of the translation initiation factor eIF2B; genes encoding translation factors are repressed by phagocytosis by murine macrophages
PIS50626.1	30.05183066	29.87736377	30.19978804	30.24906781	30.03589092	30.09501756	0.773585399	0.95	0.0837	PIS50626.1	orf19.4639	Protein present in exponential and stationary growth phase yeast cultures
PIS55073.1	30.99308097	30.62719335	30.97384112	31.08298102	30.9786269	30.78368952	0.751569168	0.949	0.0837	PIS55073.1	YPT31	Protein required for resistance to toxic ergosterol analog
PIS56844.1	28.20546967	27.72401744	28.30172706	28.15276533	28.18226469	28.14627935	0.828008264	0.954	0.0834	PIS56844.1	ERG8	Putative phosphomevalonate kinase; enzyme of the mevalonate pathway of isoprenoid biosynthesis; transcript regulated by Nrg1; possible drug target; not conserved in H. sapiens
PIS58399.1	27.33780731	26.54052636	27.49204159	27.41214509	27.14160844	27.06664436	0.870202119	0.956	0.0833	PIS58399.1	orf19.7109	Ortholog(s) have myosin I binding activity and cytoplasm localization
PIS51937.1	30.15709368	29.23972697	30.11591053	29.7915341	30.02444855	29.94607597	0.875318497	0.956	0.0831	PIS51937.1	CTP1	Putative citrate transport protein; flucytosine induced; amphotericin B repressed, caspofungin repressed; Hap43p-induced gene
PIS51329.1	25.83865732	26.52763056	26.3444597	26.27832785	26.17586613	26.50457663	0.873899332	0.956	0.0827	PIS51329.1	orf19.6828	Ortholog(s) have role in rRNA processing and preribosome, large subunit precursor localization
PIS58501.1	26.21918457	25.94472061	25.561075	26.97137476	26.1778064	24.82155829	0.867489157	0.956	0.0819	PIS58501.1	CRL1	Predicted GTPase of RHO family; CAAX motif geranylgeranylated; expression in S. cerevisiae causes dominant-negative inhibition of pheromone response
PIS52427.1	28.90318287	29.88151223	28.88124168	29.05264274	29.35931514	29.49699123	0.850910931	0.955	0.081	PIS52427.1	DOA1	WD repeat protein; required for ubiquitin-mediated protein degradation; role in control of cellular ubiquitin levels; may promote efficient NHEJ in postdiauxic/stationary phase; Spider biofilm repressed
PIS54901.1	27.94307653	27.3225479	27.99419085	27.91549117	27.8922907	27.69311541	0.929014569	0.959	0.0804	PIS54901.1	orf19.927	Ortholog(s) have tRNA binding activity, role in DNA recombination, positive regulation of transcription by RNA polymerase II, telomere maintenance, telomere maintenance via recombination and EKC/KEOPS complex localization

PIS55549.1	24.51701193	24.82128917	25.17201943	25.28664598	24.62390052	24.83887286	0.849872345	0.955	0.0797	PIS55549.1	CKB1	Regulatory subunit of protein kinase CK2 (casein kinase II), beta subunit; null mutants are hypersensitive to caspofungin and hydrogen peroxide medium
PIS48526.1	28.16679097	28.68348624	28.25196179	28.44531146	28.52167978	28.37392747	0.836922738	0.954	0.0796	PIS48526.1	orf19.2631	Subunit of Elongator complex; required for modification of wobble nucleosides in tRNA in S. cerevisiae; Hap43-induced gene
PIS58542.1	23.2696706	23.83353078	24.45980599	22.91185812	24.88632145	24.00248458	0.914514306	0.958	0.0792	PIS58542.1	DSL1	Protein similar to S. cerevisiae Dsl1p, which is a member of the t-SNARE complex of the endoplasmic reticulum
PIS51391.1	24.88970629	24.25686815	25.23632294	25.2253985	25.24137564	24.15036878	0.850812541	0.955	0.0781	PIS51391.1	orf19.3292	Ortholog(s) have peptide-methionine (R)-S-oxide reductase activity, role in cellular response to oxidative stress and mitochondrion localization
PIS52235.1	29.08437508	28.77074526	29.22990468	29.58467259	28.99361203	28.74031318	0.861008458	0.955	0.0779	PIS52235.1	orf19.1993	Subunit of the 19S regulatory particle lid of the proteasome
PIS58286.1	31.8467502	31.460183	32.03072989	31.91678241	31.7715321	31.8794681	0.866354276	0.956	0.0767	PIS58286.1	EHD3	Predicted 3-hydroxyisobutyryl-CoA hydrolase; mitochondrially localized; Spider biofilm induced
PIS55765.1	33.90747553	33.68280561	33.95828753	33.82720716	34.02324947	33.92656712	0.804726942	0.952	0.0762	PIS55765.1	RPS13	Putative ribosomal protein of the small subunit
PIS55830.1	25.48967973	26.10237235	25.46411658	25.34471171	26.31921573	25.61755971	0.888207611	0.957	0.0751	PIS55830.1	orf19.2350	Protein similar to S. cerevisiae Yor378w; MFS family transporter; transposon mutation affects filamentous growth; null mutants are viable; fungal-specific (no human or murine homolog)
PIS56878.1	27.61057452	27.86584259	28.11938922	28.29824227	27.78725545	27.73504696	0.833236314	0.954	0.0749	PIS56878.1	BEM3	Putative GTPase-activating protein (GAP) for Rho-type GTPase Cdc42p; involved in cell signaling pathways that control cell polarity; similar to S. cerevisiae Bem3p
PIS51734.1	28.72596038	28.11512843	29.13839502	29.11320164	28.72201693	28.36842772	0.863948387	0.956	0.0747	PIS51734.1	orf19.2019	Ortholog(s) have structural constituent of ribosome activity, role in mitochondrial translation and mitochondrial large ribosomal subunit localization

PIS48693.1	25.74218767	25.16611202	25.18200052	25.47184373	25.32339857	25.51927513	0.840670908	0.954	0.0747	PIS48693.1	orf19.5352	Protein with a predicted magnesium transporter domain; mutants are viable
PIS51652.1	34.12812613	34.19597818	33.98502222	33.74570121	34.46068493	34.32542331	0.915256697	0.958	0.0742	PIS51652.1	RPL13	Putative ribosomal subunit; antigenic during murine infection; downregulated upon phagocytosis by murine macrophage; Spider biofilm repressed
PIS59016.1	26.3575829	26.12551104	26.72678147	26.85951632	26.24406105	26.3260512	0.867318534	0.956	0.0733	PIS59016.1	MCU1	Mitochondrial protein involved in utilization of carbon sources, filamentous growth and virulence
PIS51765.1	33.95917133	34.56353871	33.39356159	33.61374878	34.2847632	34.23714994	0.856054413	0.955	0.0731	PIS51765.1	SSA2	HSP70 family chaperone; cell wall fractions; antigenic; beta-defensin peptides impport; ATPase domain binds histatin 5; at hyphal surface, not yeast; farnesol-repressed in biofilm; flow model, Spider biofilm repressed; caspofungin repressed
PIS54983.1	29.5882385	28.62993214	30.004928	29.67023837	29.46984151	29.30211968	0.840491901	0.954	0.073	PIS54983.1	orf19.4271	Predicted ORF from Assembly 19; removed from Assembly 20; subsequently reinstated in Assembly 21 based on comparative genome analysis
PIS58689.1	25.07108924	25.04535942	23.36686421	22.9508938	25.02778036	25.72327615	0.929402326	0.959	0.0729	PIS58689.1	orf19.2111	Ortholog(s) have RNA polymerase II complex binding, RNA polymerase II general transcription initiation factor activity
PIS58825.1	28.20140987	27.83970153	28.48404681	28.22979851	28.16994509	28.34187617	0.813966653	0.953	0.0722	PIS58825.1	orf19.7183	Ortholog(s) have EMC complex localization
PIS51923.1	27.91102487	28.00057561	27.70236104	27.60271451	28.06750104	28.15999261	0.842791194	0.954	0.0721	PIS51923.1	orf19.4176	Ortholog(s) have structural constituent of ribosome activity and mitochondrial small ribosomal subunit localization
PIS54891.1	27.41939869	28.01885121	26.82170714	27.13827097	27.63557348	27.70055012	0.901987222	0.957	0.0715	PIS54891.1	orf19.4952.1	Ortholog(s) have FK506 binding, peptidyl-prolyl cis-trans isomerase activity and membrane localization
PIS58936.1	30.58299458	31.216894	30.60083786	30.67280696	30.88077783	31.05899379	0.813574317	0.953	0.0706	PIS58936.1	orf19.7067	Ortholog(s) have RNA polymerase II C-terminal domain phosphoserine binding, RNA polymerase II complex binding and RNA polymerase II-specific DNA-binding transcription factor binding, more

PIS58488.1	30.69587514	30.51460253	30.98303891	31.01441367	30.59994874	30.79016686	0.881606885	0.956	0.0703	PIS58488.1	orf19.7296	Plasma membrane protein implicated in stress response; similar to stomatin mechanoreception proteins; overexpression induces apoptotic-like cell death; absent from hyphal cells; induced by Rgt1; rat catheter and Spider biofilm induced
PIS58679.1	25.06029047	25.00727803	25.43266427	24.07762302	25.83793476	25.79513814	0.912499137	0.958	0.0702	PIS58679.1	ARH2	Putative adrenodoxin-NADPH oxidoreductase; role in heme biosynthesis
PIS51851.1	31.1672165	31.6841754	31.19857268	30.95346162	31.64077368	31.66638269	0.927197045	0.958	0.0702	PIS51851.1	UGA11	Putative gamma-aminobutyrate (GABA) transaminase; macrophage-induced; overlaps orf19.854.1, which is a region annotated as a blocked reading frame; Spider biofilm induced
PIS52086.1	30.95211732	30.98332827	31.27677696	31.20435624	31.09762505	31.12047113	0.904088776	0.957	0.0701	PIS52086.1	orf19.415	Putative protein of unknown function; Hap43p-repressed gene
PIS51237.1	28.06494147	27.07834593	27.80483889	27.46803279	27.93441764	27.75510969	0.889610145	0.957	0.0698	PIS51237.1	orf19.1970	Putative vacuole biogenesis protein
PIS49819.1	27.71022066	27.33615577	27.95722934	27.93512492	27.95337362	27.32428123	0.893782338	0.957	0.0697	PIS49819.1	orf19.4382	Ortholog(s) have role in retrograde vesicle-mediated transport, Golgi to endoplasmic reticulum and COPI vesicle coat localization
PIS49624.1	26.84590205	28.43791123	26.99496816	27.69295442	27.29546199	27.49888781	0.928663879	0.959	0.0695	PIS49624.1	orf19.7196	Putative vacuolar protease; upregulated in the presence of human neutrophils; Spider biofilm induced
PIS48490.1	26.13630646	27.15316244	24.51001285	26.35287259	25.87237197	25.78239334	0.912384653	0.958	0.0694	PIS48490.1	APE3	Putative vacuolar aminopeptidase Y,; regulated by Gcn2 and Gcn4; rat catheter and Spider biofilm repressed
PIS58845.1	27.0796812	27.2669326	27.67417395	27.72527123	27.20173566	27.30148217	0.875742354	0.956	0.0692	PIS58845.1	FZO1	Mitochondrial biogenesis protein; rat catheter and Spider biofilm induced
PIS56864.1	27.67715806	28.33780255	27.98951726	28.1192444	28.00659222	28.08450965	0.826208044	0.954	0.0686	PIS56864.1	orf19.2760	Putative mRNA cleavage and polyadenylation factor; heterozygous null mutant exhibits hypersensitivity to parnafungin and cordycepin in the C. albicans fitness test

PIS48663.1	27.75119639	28.07198273	27.9083976	27.94045037	27.83544373	28.16073745	0.882157512	0.956	0.0684	PIS48663.1	orf19.500	Ortholog(s) have tRNA (adenine(58)-N1)-methyltransferase activity, role in tRNA methylation and nucleus, tRNA (m1A) methyltransferase complex localization
PIS51089.1	26.45625654	26.70971954	27.12950799	27.0052888	26.90361094	26.58849088	0.8436871	0.955	0.0673	PIS51089.1	RAT1	5'-->3' exoribonuclease; similar to S. cerevisiae nuclear exoribonuclease Rat1p; suppresses S. cerevisiae kem1 mutant slow growth, mating defect, and haploid invasive growth defect
PIS58101.1	29.69212065	30.38868901	29.74569268	29.97113649	30.04771949	30.00810592	0.924994091	0.958	0.0668	PIS58101.1	DPM1	Dolichol-phosphate mannose synthase catalytic subunit; filament induced; Tup1-regulated; flow model and rat catheter biofilm repressed
PIS55047.1	25.59543122	26.77401156	25.19724372	25.5519386	26.00463238	26.20985544	0.887864341	0.957	0.0666	PIS55047.1	orf19.2097	Predicted DNA-dependent ATPase/helicase; Spider biofilm induced
PIS58527.1	25.25240642	25.75196978	25.25836093	25.46697137	25.77460276	25.22074572	0.953243357	0.96	0.0665	PIS58527.1	orf19.6457	Ortholog of S. cerevisiae : YBL086C, C. glabrata CBS138 : CAGL0C01815g, C. dubliniensis CD36 : Cd36_72270, C. parapsilosis CDC317 : CPAR2_702850 and C. auris B8441 : B9J08_001027
PIS58481.1	33.30883656	33.41795823	33.65107916	33.36663609	33.70080843	33.50835548	0.94981171	0.959	0.066	PIS58481.1	IDP2	Isocitrate dehydrogenase; white-opaque switch regulated; morphology-regulation by Ssn6; protein in exponential and stationary phase yeast; Hap43-repressed; Spider biofilm repressed by Bcr1, Tec1, Ndt80, Rob1, Brg1; Spider biofilm induced
PIS54683.1	26.66866925	26.43724744	27.41477627	27.00964918	26.76922805	26.93880716	0.905543482	0.958	0.0657	PIS54683.1	orf19.3222	Predicted vacuolar protein; rat catheter biofilm repressed; flow model biofilm repressed
PIS52120.1	26.08780546	25.83681992	27.68416189	27.37539842	26.60298763	25.82606913	0.917079074	0.958	0.0652	PIS52120.1	orf19.1592	Protein of unknown function; Spider biofilm induced
PIS54589.1	31.51666815	31.45560238	31.56219189	31.1372928	31.8109305	31.78112145	0.851468322	0.955	0.065	PIS54589.1	orf19.239	Putative ATP-dependent helicase, component of the RSC chromatin remodeling complex; essential gene; induced by nitric oxide
PIS54980.1	24.71702961	23.8103145	24.5282749	25.18864617	24.30704449	23.75490633	0.924322959	0.958	0.065	PIS54980.1	RAD9	DNA damage-dependent checkpoint protein; involved in regulation of DNA-damage-induced filamentous growth; induced by alpha pheromone in SpiderM medium

PIS50516.1	28.02987023	28.05535596	28.57385518	28.42549884	28.24986903	28.17767167	0.841334812	0.954	0.0647	PIS50516.1	orf19.2680	Putative aminophospholipid translocase (flippase); type 4 P-type ATPase; likely involved in phospholipid translocation
PIS48477.1	24.33597204	25.15390578	24.64891155	24.41123487	25.05244919	24.86681375	0.865850548	0.956	0.0639	PIS48477.1	orf19.1267.1	Ortholog(s) have cysteine desulfurase activity, role in iron-sulfur cluster assembly and L-cysteine desulfurase complex, mitochondrial matrix localization
PIS49471.1	25.51994902	23.74088786	26.0648132	25.65539502	25.53897638	24.32229546	0.931773431	0.959	0.0637	PIS49471.1	orf19.52	Ortholog(s) have role in intracellular iron ion homeostasis and mitochondrion localization
PIS55038.1	27.79191598	28.60287559	28.39944621	28.32934053	27.917689	28.7347185	0.927697752	0.959	0.0625	PIS55038.1	DFG5	N-linked mannoprotein of cell wall and membrane; possible signal transducer; role in hyphal growth and HWP1 induction in alkaline pH; GPI modification predicted; dfg5 dcw1 double mutant is inviable; caspofungin-induced, fungal-specific
PIS48811.1	26.04278382	26.35341335	25.2680752	25.07168055	26.34013475	26.43764674	0.941184753	0.959	0.0617	PIS48811.1	SNF5	SWI/SNF chromatin remodeling complex subunit involved in transcriptional regulation; mutants have defects in silicone adherence, biofilm formation, hyphal morphogenesis, cell wall defects; increased cell aggregation during yeast form growth
PIS51427.1	30.79012983	31.17535732	31.05493203	31.1390926	31.08630387	30.97966684	0.811510418	0.953	0.0615	PIS51427.1	CDC39	Protein similar to S. cerevisiae Cdc39p, which is part of the CCR4-NOT transcription regulatory complex; transposon mutation affects filamentous growth
PIS55693.1	25.88629357	26.40689555	25.82586237	25.19796931	26.37194539	26.73327551	0.916754158	0.958	0.0614	PIS55693.1	HAL22	Putative phosphoadenosine-5'-phosphate or 3'-phosphoadenosine 5'-phosphosulfate phosphatase; possible role in sulfur recycling; Hap43-repressed; F-12/CO2 biofilm induced
PIS48634.1	23.2872162	22.83558741	22.59576021	22.68982726	23.16660753	23.04614368	0.951406683	0.96	0.0613	PIS48634.1	STE50	Protein with sterile alpha motif (SAM) and Ras-associated domain (RAD); similar to S. cerevisiae Rad50p, which is involved in signal transduction via interaction with and regulation of MAPKKK
PIS54696.1	23.86437833	23.87210584	24.42147018	24.19700865	24.23939265	23.90481239	0.923997869	0.958	0.0611	PIS54696.1	orf19.2521	Ortholog of C. dubliniensis CD36 : Cd36_26390, Debaryomyces hansenii CBS767 : DEHA2C05302g, Pichia stipitis Pignal : PICST_68531 and Candida tropicalis NEW ASSEMBLY : CTRG1_00947
PIS51501.1	32.48142834	32.28717982	32.54160584	32.78087307	32.40066295	32.31179442	0.932433396	0.959	0.061	PIS51501.1	RPS24	Predicted ribosomal protein; hyphal downregulated; repressed upon phagocytosis by murine macrophage; transcriptionally activated by Tbf1; Spider biofilm repressed

PIS58117.1	32.8538131	32.60653341	32.74404128	32.6911345	32.92079531	32.77414534	0.82820059	0.954	0.0606	PIS58117.1	RPS14B	Putative ribosomal protein; repressed upon phagocytosis by murine macrophage; transcript positively regulated by Tbf1; Spider biofilm repressed
PIS51623.1	29.69086547	30.40715778	28.63712401	29.14731271	30.09789834	29.67030109	0.904841483	0.957	0.0601	PIS51623.1	GVP36	BAR domain protein; ocalizes to early and late Golgi vesicles; predicted role in adaptation to varying nutrient concentrations, fluid-phase endocytosis, actin cytoskeleton polarization and vacuole biogenesis; rat catheter biofilm repressed
PIS58772.1	23.95259263	23.50927858	24.72381766	24.59265285	23.76818499	24.00080729	0.926652849	0.958	0.0587	PIS58772.1	orf19.7074	Ortholog(s) have methylated histone binding activity and role in SAGA complex localization to transcription regulatory region, positive regulation of transcription by RNA polymerase II, protein localization
PIS49721.1	27.68816145	28.06989066	26.49246094	26.77680729	27.58892873	28.0581761	0.90364405	0.957	0.0578	PIS49721.1	POS5	Protein similar to S. cerevisiae Pos5p, a mitochondrial NADH kinase involved in the oxidative stress response; planktonic growth-induced gene; likely to be essential for growth, based on an insertional mutagenesis strategy
PIS49681.1	24.83085545	25.68953287	25.95755087	25.92402256	25.51776419	25.20895089	0.883372621	0.956	0.0576	PIS49681.1	orf19.3167	Heme A:farnesyltransferase; catalyzes the 1st step in conversion of protoheme to the heme A prosthetic group required for cytochrome c oxidase activity; Spider biofilm repressed
PIS48284.1	27.86923628	29.43558354	27.281283	27.99650092	28.23604963	28.5236066	0.936347269	0.959	0.0567	PIS48284.1	SGT2	Putative small tetratricopeptide repeat (TPR)-containing protein; protein abundance is affected by URA3 expression in the CAI-4 strain background; Mig1-regulated
PIS50502.1	23.00157857	26.48056331	24.40283517	24.5483107	23.50235654	26.00416611	0.947619434	0.959	0.0566	PIS50502.1	RIM13	Protease of the pH response pathway; likely to mediate activation of Rim101 via C-terminal cleavage; required for alkaline pH-induced hyphal growth and for normal chlamydospore formation; Hap43-repressed; flow model biofilm induced
PIS51158.1	30.18110586	30.12610659	30.07488078	30.0781457	30.1728045	30.30021661	0.842033482	0.954	0.0564	PIS51158.1	HET1	Putative sphingolipid transfer protein; involved in localization of glucosylceramide which is important for virulence; Spider biofilm repressed
PIS58507.1	23.5455467	23.95470048	25.23794371	23.90534353	24.57643871	24.42529645	0.905476946	0.958	0.0563	PIS58507.1	orf19.2594	Ortholog(s) have RNA polymerase I activity, role in nucleolar large rRNA transcription by RNA polymerase I, transcription by RNA polymerase I, transcription initiation at RNA polymerase I promoter and RNA polymerase I complex localization
PIS56698.1	22.27463286	22.35323433	20.25119099	21.95360474	21.38771619	21.70594861	0.96064698	0.96	0.0561	PIS56698.1	orf19.1137	Thymidylate kinase of unknown role; forms a dimer; potential target for antifungal drugs

PIS51620.1	29.79535325	29.78632323	29.8238974	30.01893696	29.79618253	29.75679117	0.825002292	0.954	0.0554	PIS51620.1	CBR1	Putative cytochrome B5 reductase; plasma membrane-localized
PIS55465.1	25.08694815	25.89017761	24.36827499	25.03385924	24.81877314	25.65599065	0.915210024	0.958	0.0544	PIS55465.1	CDC34	Putative ubiquitin-protein ligase; transcript regulated by Nrg1 and Tup1, and by Gcn2 and Gcn4; rat catheter biofilm induced
PIS58448.1	28.51087823	28.61358243	28.7106523	28.79071128	28.53252459	28.67503223	0.887159347	0.957	0.0544	PIS58448.1	orf19.3681	Ortholog(s) have guanyl-nucleotide exchange factor activity, nuclear import signal receptor activity, protein-containing complex binding activity
PIS49497.1	27.67223717	27.30190221	27.05178891	27.39898028	27.0808433	27.7078677	0.914506494	0.958	0.0539	PIS49497.1	orf19.426	Ortholog of C. dubliniensis CD36 : Cd36_05120, C. parapsilosis CDC317 : CPAR2_107740, C. auris B8441 : B9J08_004520 and Candida tenuis NRRL Y-1498 : CANTEDRAFT_136277
PIS54590.1	29.48486051	29.5038479	29.01805065	29.41334823	29.02050558	29.73194316	0.935780528	0.959	0.053	PIS54590.1	CCP1	Cytochrome-c peroxidase N terminus; Rim101, alkaline pH repressed; induced in low iron or by macrophage interaction; oxygen-induced activity; regulated by Sef1, Sfu1, and Hap43; Spider biofilm induced; rat catheter biofilm repressed
PIS55431.1	24.88180191	25.46385261	25.7958763	25.52807542	25.16825815	25.60348122	0.878412642	0.956	0.0528	PIS55431.1	DIP5	Dicarboxylic amino acid permease; mutation confers hypersensitivity to toxic ergosterol analog; induced upon phagocytosis by macrophage; Gcn4-regulated; upregulated by Rim101 at pH 8; rat catheter and Spider biofilm induced
PIS49696.1	30.20886352	29.53773708	30.36163481	30.10341205	30.13348691	30.02943428	0.888171172	0.957	0.0527	PIS49696.1	orf19.5278	Protein of unknown function; Spider biofilm induced
PIS58365.1	24.8034173	24.50188434	25.95856801	26.05567579	24.64764183	24.71854305	0.939019054	0.959	0.0527	PIS58365.1	TBF1	Essential transcription factor; induces ribosomal protein genes and the rDNA locus; acts with Cbf1 at subset of promoters; recruits Fhl1 and Ifh1 to promoters; role is analogous to that of S. cerevisiae Rap1; Spider biofilm induced
PIS58340.1	28.75817164	29.0214603	29.15760061	28.95163243	29.04403887	29.09949075	0.910123033	0.958	0.0526	PIS58340.1	orf19.7092	Putative NAD dependent epimerase/dehydratase family protein; Spider biofilm repressed
PIS51825.1	30.54482376	29.98574324	30.69558742	30.32424695	30.57568847	30.48359178	0.882402892	0.956	0.0525	PIS51825.1	SIN3	Protein similar to S. cerevisiae Sin3p (transcriptional corepressor involved in histone deacetylase recruitment); has paired amphipathic helix PAH1 domain; interacts with ScOpi1p, not CaOpi1p; transposon mutation affects filamentous growth

PIS58546.1	29.17469273	29.16911694	29.86246268	29.54684164	29.49003201	29.32265804	0.932036326	0.959	0.0511	PIS58546.1	orf19.3348	Ortholog(s) have structural constituent of ribosome activity and fungal-type vacuole, mitochondrial large ribosomal subunit localization
PIS59010.1	31.00146265	30.86513864	31.07046094	31.03694089	30.99680209	31.05666654	0.843901812	0.955	0.0511	PIS59010.1	STT4	Phosphatidylinositol-4-kinase; forms a complex with Ypp1p and Efr3p that is required for phosphatidylinositol-4-phosphate, PI(4)P, in plasma membrane; required for invasive growth and cell wall organization
PIS50595.1	27.113215	27.32866891	27.03296946	27.21891697	27.13925486	27.26865834	0.963869183	0.96	0.0507	PIS50595.1	orf19.4164	Ortholog(s) have role in ribosome disassembly
PIS51447.1	23.51848648	24.4198247	25.58185761	25.24717385	24.11438027	24.3078397	0.920012476	0.958	0.0497	PIS51447.1	orf19.4090.1	Ortholog(s) have role in vacuolar proton-transporting V-type ATPase complex assembly and endoplasmic reticulum membrane localization
PIS56671.1	24.0992197	24.30904957	24.60552272	24.5775504	24.31991834	24.26554907	0.937900021	0.959	0.0497	PIS56671.1	orf19.6722	Protein similar to S. cerevisiae Rad4p; down-regulation associated with azole resistance
PIS49687.1	27.97081327	28.05704605	28.27908812	27.92621081	28.33616248	28.19334119	0.905360126	0.958	0.0496	PIS49687.1	OSH3	Protein required for wild-type filamentation; has oxysterol binding protein domain and pleckstrin homology motif; overproducton suppresses cph1 homozygous null filamnetation defect; similar to S. cerevisiae Osh3p
PIS51340.1	32.02106147	31.49776173	32.19030705	32.07033656	31.83905481	31.94739062	0.860477188	0.955	0.0492	PIS51340.1	MTS1	Sphingolipid C9-methyltransferase; catalyzes methylation of the 9th carbon in the long chain base component of glucosylceramides; glucosylceramide biosynthesis is important for virulence; Spider biofilm repressed
PIS52040.1	31.18223613	31.48582728	31.69789466	31.32173711	31.64106333	31.55073148	0.866104683	0.956	0.0492	PIS52040.1	orf19.1549	Plasma membrane-associated protein identified in detergent-resistant membrane fraction (possible lipid raft component); repressed by nitric oxide; predicted transmembrane helix
PIS51852.1	28.6116375	28.62202575	28.140037	28.40539661	28.75296045	28.3611176	0.875034711	0.956	0.0486	PIS51852.1	CMK2	Putative calmodulin-dependent protein kinase; involved in cell wall integrity and oxidative stress response
PIS48534.1	34.24657635	33.88776226	34.21640849	34.05451562	34.18087588	34.26089062	0.856881424	0.955	0.0485	PIS48534.1	TAL1	Transaldolase; protein present in exponential and stationary growth phase yeast cultures; oxidative stress-induced via Cap1; induced by nitric oxide independent of Yhb1p; sumoylation target; rat catheter biofilm repressed

PIS52182.1	28.5909525	28.22073674	28.78179202	28.57704721	28.44890907	28.71251859	0.904400968	0.957	0.0483	PIS52182.1	orf19.1335	Ortholog of S. cerevisiae Mtr4, an ATP-dependent 3'-5' RNA helicase of the DEAD-box family; Hap43-induced gene; Spider biofilm induced
PIS49811.1	30.01093869	29.3417421	30.58348281	30.3288183	29.87513205	29.87530662	0.935394014	0.959	0.0477	PIS49811.1	PST3	Flavodoxin-like protein involved in oxidative stress protection and virulence; YNB biofilm induced; stationary phase enriched protein; rat catheter and Spider biofilm repressed
PIS58952.1	26.81227056	25.86005261	26.9816887	26.62479531	26.67569541	26.49546767	0.916553269	0.958	0.0473	PIS58952.1	DCG1	Protein of unknown function; ortholog of S. cerevisiae Dcg1; transcript regulated by Nrg1 and Mig1
PIS54964.1	26.73961203	27.76230889	26.52310611	26.71988508	27.14176801	27.30516612	0.942463694	0.959	0.0473	PIS54964.1	orf19.6698	Ortholog(s) have asparagine-tRNA ligase activity, role in asparaginyl-tRNA aminoacylation, mitochondrial asparaginyl-tRNA aminoacylation and mitochondrion localization
PIS51092.1	27.48669606	27.76749671	26.12093811	26.39149837	27.30598927	27.81676803	0.942242698	0.959	0.0464	PIS51092.1	MLP1	Ortholog(s) have promoter-terminator loop anchoring activity, ribonucleoprotein complex binding activity
PIS51518.1	27.32185885	26.76497843	26.54299201	26.85270974	26.91124979	27.00481519	0.946592529	0.959	0.0463	PIS51518.1	orf19.2638	Protein of unknown function; Spider biofilm induced
PIS48474.1	26.98146446	28.51993615	26.21969432	27.12913041	27.2960575	27.43360496	0.938071135	0.959	0.0459	PIS48474.1	orf19.7556	Ortholog of C. dubliniensis CD36 : Cd36_34965, C. parapsilosis CDC317 : CPAR2_200910, C. auris B8441 : B9J08_005167 and Candida tenuis NRRL Y-1498 : CANTEDRAFT_95984
PIS58757.1	25.21262462	24.03875536	26.49667377	25.895326	24.9613664	25.02811952	0.938127345	0.959	0.0456	PIS58757.1	orf19.1642	Ortholog of S. cerevisiae Loc1, a nuclear protein involved in asymmetric localization of ASH1 mRNA in S. cerevisiae; Hap43-induced gene; Spider biofilm induced
PIS51297.1	25.39212636	24.5475354	24.91466989	24.84620516	25.53842144	24.60593683	0.941360604	0.959	0.0454	PIS51297.1	ALG1	Protein similar to S. cerevisiae Alg1p, a mannosyltransferase involved in N-linked protein glycosylation; likely to be essential for growth, based on an insertional mutagenesis strategy
PIS54928.1	21.28782709	22.24595692	21.18347968	21.72741883	21.76862624	21.35468948	0.965298123	0.96	0.0445	PIS54928.1	SPT10	Ortholog(s) have promoter-specific chromatin binding, sequence-specific DNA binding activity

PIS52460.1	28.15892015	28.91729583	27.6897706	27.55548864	28.52188468	28.81912447	0.927794031	0.959	0.0435	PIS52460.1	orf19.2639.1	Lsm (Like Sm) protein; predicted role in involved in mRNA decay; Spider biofilm repressed
PIS48237.1	24.31667833	23.67640367	25.12061925	24.57783285	24.15379659	24.5120543	0.926817371	0.958	0.0433	PIS48237.1	orf19.7163	Ortholog of C. dubliniensis CD36 : Cd36_73790, C. parapsilosis CDC317 : CPAR2_702880, C. auris B8441 : B9J08_004922 and Candida tenuis NRRL Y-1498 : CANTEDRAFT_92070
PIS52191.1	30.35310934	30.61997114	30.87839785	30.38457529	30.82569531	30.76919596	0.902979297	0.957	0.0427	PIS52191.1	CYC1	Cytochrome c; complements defects of S. cerevisiae cyc1 cyc7 double mutant; induced in high iron; alkaline repressed; repressed by nitric oxide; Hap43-dependent repression in low iron; regulated by Sef1, Sfu1
PIS51405.1	31.48321178	31.31852344	31.54183742	31.42853424	31.36436524	31.67786428	0.873405462	0.956	0.0424	PIS51405.1	GLC3	Putative 1,4-glucan branching enzyme; fluconazole-induced; colony morphology-related gene regulation by Ssn6; stationary phase enriched protein
PIS50601.1	27.73211731	28.48373765	26.67957082	26.95531069	28.01856708	28.04824123	0.951482486	0.96	0.0422	PIS50601.1	orf19.321	Ortholog(s) have L-methionine transmembrane transporter activity and role in methionine import across plasma membrane
PIS48228.1	29.33288308	28.46339146	29.81204153	29.67164297	29.12392354	28.93901796	0.964236282	0.96	0.0421	PIS48228.1	orf19.6435	Highly conserved subunit of mitochondrial pyruvate carrier; Hap43-repressed; Spider biofilm repressed
PIS52100.1	28.50374015	28.87259421	28.39050955	28.40497433	28.47490846	29.00894275	0.903810063	0.957	0.0407	PIS52100.1	orf19.439	Ortholog(s) have DNA binding, DNA strand exchange activity, single-stranded DNA binding, structural constituent of ribosome activity
PIS55523.1	25.57874625	25.53711149	26.15343956	25.71976457	25.96400625	25.70583426	0.954694115	0.96	0.0401	PIS55523.1	orf19.2143	Ortholog(s) have role in endocytic recycling, tRNA methylation, wobble position ribose methylation and cytoplasm, endosome localization
PIS54594.1	27.90632484	28.54756848	27.50953535	28.09693506	28.08650893	27.89905373	0.91397653	0.958	0.0397	PIS54594.1	MED7	Subunit of the RNA polymerase II mediator complex
PIS58965.1	29.30231043	29.4396979	29.33162288	29.64483037	29.19504866	29.35121061	0.9178577	0.958	0.0392	PIS58965.1	orf19.1956	Ortholog(s) have role in mitochondrial translational initiation, reciprocal meiotic recombination and mitochondrial matrix, mitochondrion localization

PIS52342.1	30.80704751	31.1419238	30.47988977	30.46809367	31.07642445	31.00080947	0.904242777	0.957	0.0388	PIS52342.1	orf19.5917.3	RNA binding protein required for export of poly(A)+ mRNA from the nucleus; Spider biofilm repressed
PIS50310.1	25.7207421	25.69516881	25.90164143	25.08738457	25.59233754	26.75342837	0.950695255	0.959	0.0385	PIS50310.1	orf19.7642	Ortholog of S. cerevisiae Vps3; CORVET tethering complex component involved in vacuolar protein sorting; Hap43-repressed gene
PIS50440.1	23.46740078	25.9519029	23.86907747	24.58723216	24.63013737	24.18597368	0.953217077	0.96	0.0383	PIS50440.1	CDC45	Putative DNA replication initiation factor; transcriptionally regulated by interaction with macrophage
PIS58274.1	22.7451973	22.7907037	22.5834492	22.47193733	22.6761653	23.08405255	0.98067576	0.961	0.0376	PIS58274.1	orf19.4657	Ortholog(s) have phosphoprotein phosphatase activity
PIS58465.1	32.29643541	32.06953207	32.17171963	31.98198448	32.34887293	32.318926	0.909598468	0.958	0.0374	PIS58465.1	GSC1	Essential beta-1,3-glucan synthase subunit; gsc1 allele determines resistance/sensitivity to echinocandins; 16 predicted membrane-spanning regions; mRNA abundance declines after yeast-to-hypha transition; Spider biofilm induced
PIS58961.1	27.41100016	27.38880435	26.99393101	28.17060485	26.48384542	27.25109425	0.968017144	0.96	0.0373	PIS58961.1	orf19.1961	Planktonic growth-induced gene
PIS54537.1	28.00857011	28.50972709	28.0754437	27.79129876	28.4337994	28.48023518	0.931570904	0.959	0.0372	PIS54537.1	orf19.5433	Ortholog(s) have protein folding chaperone activity, role in mitochondrial respiratory chain complex III assembly and mitochondrial matrix, mitochondrion localization
PIS55507.1	31.00869955	30.70022719	31.41321713	31.30531314	30.96141171	30.96388051	0.960693793	0.96	0.0362	PIS55507.1	MRPL3	Ribosomal protein of the large subunit, mitochondrial; repressed in core stress response; protein present in exponential and stationary growth phase yeast cultures
PIS54897.1	29.04999673	29.40941096	29.32437491	29.28893929	29.17111662	29.42987483	0.937744641	0.959	0.0354	PIS54897.1	orf19.4705	Ortholog(s) have ATP binding, ATP:3'-cytidine-cytidine-tRNA adenylyltransferase activity, CCA tRNA nucleotidyltransferase activity, CTP:tRNA cytidylyltransferase activity, tRNA binding activity
PIS51343.1	33.83386054	33.86372974	33.92774095	33.87718121	33.99654002	33.85729876	0.893457963	0.957	0.0352	PIS51343.1	orf19.4149.1	Protein component of the small (40S) ribosomal subunit; Spider biofilm repressed

PIS55558.1	32.87844801	33.02792147	33.30263915	33.13851888	32.98411648	33.19119696	0.911617147	0.958	0.0349	PIS55558.1	AMO2	Protein similar to A. niger predicted peroxisomal copper amino oxidase; mutation confers hypersensitivity to toxic ergosterol analog; F-12/CO2 early biofilm induced
PIS51183.1	24.96769799	24.83896084	24.41223143	25.02836939	25.15988494	24.13521841	0.95408088	0.96	0.0349	PIS51183.1	orf19.7580	Ortholog of C. dubliniensis CD36 : Cd36_35095, C. parapsilosis CDC317 : CPAR2_200710, C. auris B8441 : B9J08_002757 and Candida tenuis NRRL Y-1498 : CANTEDRAFT_112338
PIS52058.1	31.2359077	31.03107644	31.31780773	31.42101717	31.05455991	31.20951298	0.933303221	0.959	0.0334	PIS52058.1	RPN3	Putative non-ATPase regulatory subunit of the 26S proteasome lid; amphotericin B repressed; oxidative stress-induced via Cap1p
PIS56839.1	28.99541429	29.76724712	28.23876076	29.29495111	28.80393434	28.99812228	0.934489062	0.959	0.0319	PIS56839.1	orf19.2708	Ortholog(s) have 5.8S rRNA binding, guanyl-nucleotide exchange factor activity, large ribosomal subunit rRNA binding, rRNA primary transcript binding, small ribosomal subunit rRNA binding activity and role in cytosolic ribosome assembly
PIS48637.1	28.86951007	27.84669027	28.99345142	28.93160358	28.39630502	28.47660039	0.948133	0.959	0.0316	PIS48637.1	orf19.1634	Has domain(s) with predicted fatty-acyl-CoA binding activity
PIS58484.1	30.15480306	30.2075282	30.24265648	30.40304155	30.19790762	30.09764664	0.903626086	0.957	0.0312	PIS58484.1	orf19.3782.2	Ortholog(s) have role in cristae formation, membrane organization and MICOS complex, mitochondrial crista junction localization
PIS49739.1	28.37830109	29.16991928	28.47184305	28.55730734	28.71586482	28.84025218	0.925555817	0.958	0.0311	PIS49739.1	orf19.6778	Aminophospholipid translocase (flippase); maintains membrane lipid asymmetry in post-Golgi secretory vesicles; contributes to clathrin-coated vesicle formation and endocytosis; flow model biofilm repressed
PIS51534.1	30.60788867	30.37942067	30.76581571	30.51297871	30.68779312	30.64430539	0.92187708	0.958	0.0307	PIS51534.1	ERG20	Putative farnesyl pyrophosphate synthetase involved in isoprenoid and sterol biosynthesis, based on similarity to S. cerevisiae Erg20p; likely to be essential for growth, based on an insertional mutagenesis strategy
PIS48763.1	27.2715717	26.8477515	27.38996302	27.54708273	26.93332354	27.12049278	0.93457502	0.959	0.0305	PIS48763.1	orf19.6462	Ortholog(s) have EMC complex localization
PIS55618.1	24.69449974	25.22500737	25.48031068	25.51652738	24.6249633	25.349584	0.961981325	0.96	0.0304	PIS55618.1	orf19.5249	Ortholog(s) have role in autophagosome assembly, macroautophagy and Atg1/ULK1 kinase complex, phagophore assembly site localization

PIS48422.1	23.56161283	23.37346031	24.25642884	23.56341089	23.92382302	23.79480306	0.951419672	0.96	0.0302	PIS48422.1	MTG2	Putative Obg family GTPase member; peripheral protein of the mitochondrial inner membrane; associates with the large ribosomal subunit; required for mitochondrial translation; rat catheter biofilm repressed
PIS52454.1	26.29695808	26.02711746	26.57883617	26.42880635	26.35594832	26.20738738	0.968695274	0.96	0.0297	PIS52454.1	orf19.4341	Ortholog(s) have role in attachment of GPI anchor to protein and GPI-anchor transamidase complex localization
PIS54581.1	27.76626633	27.0939107	28.32518794	28.12546023	27.51826151	27.62974057	0.974663679	0.96	0.0294	PIS54581.1	orf19.3154	Ortholog(s) have role in inner mitochondrial membrane organization and mitochondrial inner membrane localization
PIS56611.1	28.6755416	28.98399231	28.61391641	28.65883384	28.74349081	28.95901645	0.947151882	0.959	0.0293	PIS56611.1	orf19.1872	Plasma membrane protein; repressed by nitric oxide
PIS48304.1	34.23084465	34.15584559	34.40260738	34.36043603	34.35367097	34.16245096	0.910587737	0.958	0.0291	PIS48304.1	RPS21	Protein component of the small (40S) subunit; repressed upon phagocytosis by murine macrophage; positively regulated by Tbf1; Spider biofilm repressed
PIS54684.1	25.05361455	25.1774109	25.1902067	25.15801589	25.11287907	25.23452522	0.951243994	0.96	0.0281	PIS54684.1	orf19.775	Ortholog of C. dubliniensis CD36 : Cd36_04450, C. parapsilosis CDC317 : CPAR2_105460, C. auris B8441 : B9J08_002463, Debaryomyces hansenii CBS767 : DEHA2D07128g and Pichia stipitis Pignal : PICST_80203
PIS52043.1	28.43239786	29.03002732	29.23591846	28.99152927	29.30393863	28.48497129	0.949417769	0.959	0.0274	PIS52043.1	orf19.1546	Membrane-localized protein of unknown function
PIS54729.1	30.207629	29.99658324	30.2344324	30.14554601	29.96737837	30.40775509	0.97443777	0.96	0.0273	PIS54729.1	DIM1	Putative 18S rRNA dimethylase; predicted role in rRNA modification and processing; Hap43-induced; likely to be essential for growth based on insertional mutagenesis strategy; F-12/CO2 early biofilm induced
PIS52185.1	27.09119782	27.33097153	26.46390589	26.97120268	27.08063107	26.914604	0.953747622	0.96	0.0268	PIS52185.1	RAP1	Transcription factor; binds telomeres and regulatory sequences in DNA; involved in telomere maintenance; represses hyphal growth under yeast-favoring conditions; similar to (but shorter than) S. cerevisiae Rap1
PIS58644.1	34.23896263	34.03930667	34.31381724	34.27155189	34.36661099	34.03297684	0.921697058	0.958	0.0264	PIS58644.1	RPL18	Predicted ribosomal protein; Plc1p-regulated, Tbf1-activated; repressed upon phagocytosis by murine macrophage; Hap43p-induced; Spider biofilm repressed

PIS52273.1	30.1696164	31.02958676	29.70252679	29.68248542	30.7105469	30.58722136	0.960760592	0.96	0.0262	PIS52273.1	orf19.3610	Protein of unknown function; upregulation correlates with clinical development of fluconazole resistance; regulated by Sef1, Sfu1, and Hap43
PIS51052.1	28.63924631	29.01944315	28.97250286	28.61634875	29.0364459	29.05607839	0.944275314	0.959	0.0259	PIS51052.1	MRPL33	Putative mitochondrial ribosomal protein of the large subunit; Ssr1-repressed; rat catheter biofilm induced
PIS55773.1	31.13310623	31.58233501	30.88608291	30.94552519	31.34376037	31.38930934	0.936487957	0.959	0.0257	PIS55773.1	KRS1	Putative tRNA-Lys synthetase; repressed upon phagocytosis by murine macrophages; stationary phase enriched protein; Spider biofilm repressed
PIS56836.1	25.35341948	25.27643791	22.72107498	25.1219991	24.81875597	23.48729236	0.971410648	0.96	0.0257	PIS56836.1	SOD1	Cytosolic copper- and zinc-containing superoxide dismutase; role in protection from oxidative stress; required for full virulence; alkaline induced by Rim101; induced by human blood; rat catheter, flow model and Spider biofilm repressed
PIS51356.1	25.67647033	26.56075936	25.65259621	25.78572417	25.7011546	26.47914276	0.969303163	0.96	0.0254	PIS51356.1	orf19.2650.1	Mitochondrial ribosomal protein of the small subunit; Spider biofilm repressed
PIS51342.1	26.87096236	26.1388618	27.0905555	26.5019833	27.10425574	26.56975281	0.974563772	0.96	0.0252	PIS51342.1	orf19.4150	Putative glutaredoxin; induced by nitric oxide; Spider biofilm induced
PIS56902.1	29.73567357	29.63619112	30.72457948	30.08382206	29.95794765	30.12946556	0.94980068	0.959	0.0249	PIS56902.1	ERG11	Lanosterol 14-alpha-demethylase; cytochrome P450 family; role in ergosterol biosynthesis; target of azole antifungals; may contribute to drug resistance; azole or flow model biofilm induced; drug treated biofilm induced; hypoxia regulated
PIS49473.1	29.24208366	29.72025768	29.43859337	29.94931368	29.19961651	29.32540119	0.955027278	0.96	0.0245	PIS49473.1	orf19.7306	Aldo-keto reductase; increased transcript associated with MDR1 overexpression, benomyl or long-term fluconazole treatment; overexpression does not affect drug or oxidative stress sensitivity; stationary phase enriched; flow biofim repressed
PIS54927.1	26.18655841	24.38127847	26.91548091	26.20175553	25.92164752	25.43172254	0.967111211	0.96	0.0239	PIS54927.1	orf19.1857	Putative L-azetidine-2-carboxylic acid acetyltransferase; mutants are viable
PIS49629.1	30.8074339	30.63993901	31.05688193	30.8308525	30.87590322	30.86789925	0.934787163	0.959	0.0235	PIS49629.1	ARP2	Component of the Arp2/3 complex; required for virulence, hyphal growth, cell wall/cytoskeleton organization, not for endocytosis; mutation confers hypersensitivity to cytochalasin D; regulated by Gcn2 and Gcn4; Spider biofilm repressed

PIS48552.1	29.7605149	28.90379298	29.74298802	29.3929458	29.55908815	29.52316567	0.941667771	0.959	0.0226	PIS48552.1	RPN5	Putative COP9 signalosome component; macrophage/pseudohyphal-repressed
PIS55486.1	27.00576699	26.82952875	27.23338859	27.79312708	26.93766716	26.4044316	0.973796741	0.96	0.0222	PIS55486.1	SEC34	Ortholog(s) have cargo adaptor activity
PIS58919.1	28.15820303	28.37610624	27.21175592	27.43575836	28.11498513	28.25817315	0.957222543	0.96	0.021	PIS58919.1	orf19.649	Ortholog of C. dubliniensis CD36 : Cd36_30530, C. parapsilosis CDC317 : CPAR2_203340, C. auris B8441 : B9J08_000376 and Candida tenuis NRRL Y-1498 : CANTEDRAFT_117008
PIS48481.1	27.36298994	26.91553218	27.19149586	27.21030006	27.17965668	27.14307651	0.962205132	0.96	0.021	PIS48481.1	PHO86	Putative endoplasmic reticulum protein; possibly adherence-induced
PIS55715.1	28.68302776	29.16162494	29.71180341	29.03677009	29.06584801	29.51560762	0.983576048	0.961	0.0206	PIS55715.1	orf19.2439.1	Protein whose ortholog(s) have ubiquinol-cytochrome-c reductase activity and roles in aerobic respiration and mitochondrial electron transport; null mutant is viable and shows normal vegetative growth on various carbon sources
PIS55631.1	24.75720143	25.5247569	24.78269715	23.93615397	25.48543537	25.70476356	0.969155163	0.96	0.0206	PIS55631.1	orf19.6843	Ortholog(s) have role in CENP-A containing chromatin assembly, chromatin remodeling and nucleoplasm localization
PIS58804.1	25.63360619	25.98830081	25.66496232	25.85608619	25.89227407	25.59949846	0.985805868	0.961	0.0203	PIS58804.1	orf19.2330	Putative U3 snoRNA-associated protein; Hap43-induced; transposon mutation affects filamentous growth; repressed by prostaglandins
PIS51345.1	26.78009034	26.53751493	26.06725103	26.03942345	26.72689543	26.67796028	0.969691671	0.96	0.0198	PIS51345.1	SMD3	Putative core snRNP protein; induced upon adherence to polystyrene
PIS48315.1	30.21970505	30.17740153	30.11431619	30.13079673	30.20127813	30.23731727	0.962037063	0.96	0.0193	PIS48315.1	MRPL10	Putative mitochondrial large subunit ribosomal protein; colony morphology-related gene regulation by Ssn6
PIS51828.1	23.93301082	23.71795873	24.29034306	23.88155438	24.12276686	23.99498555	0.981666067	0.961	0.0193	PIS51828.1	RRS1	Putative ribosome biogenesis and nuclear export protein; Hap43p-induced gene; mutation confers hypersensitivity to 5-fluorocytosine (5-FC), 5-fluorouracil (5-FU), and tubercidin (7-deazaadenosine)

PIS56683.1	30.28241232	30.33256474	30.42993092	30.15138404	30.47429413	30.47678329	0.984592927	0.961	0.0192	PIS56683.1	RPN10	Putative 19S regulatory particle of the 26S proteasome; macrophage/pseudohyphal-repressed; regulated by Gcn2 and Gcn4; Spider biofilm repressed
PIS50599.1	21.31647297	21.92085029	25.23070752	19.24452781	24.55251995	24.72804435	0.988667784	0.961	0.019	PIS50599.1	orf19.4159	Ortholog(s) have magnesium ion transmembrane transporter activity, role in magnesium ion export from mitochondrion, magnesium ion transport and mitochondrial inner membrane localization
PIS51086.1	26.86057667	27.7540126	28.21567454	27.63292731	27.68935775	27.55934404	0.959068449	0.96	0.0171	PIS51086.1	orf19.4287	Putative oxidoreductase; Hap43-repressed gene; clade-associated gene expression
PIS50316.1	27.76733176	27.81255144	27.39859823	27.70605771	27.35220428	27.97102127	0.96667111	0.96	0.0169	PIS50316.1	HPT11	Putative hypoxanthine-guanine phosphoribosyltransferase; protein abundance affected by URA3 expression in the CAI4 strain background; protein level decreases in stationary phase; Spider biofilm induced
PIS52272.1	28.99339661	28.73508322	28.936273	28.87280217	28.99830937	28.84396749	0.952156233	0.96	0.0168	PIS52272.1	SLP2	Protein similar to stomatin mechanoreception protein
PIS51864.1	30.55885282	30.23230463	30.96650532	30.45999825	30.52401299	30.82372236	0.969418075	0.96	0.0167	PIS51864.1	MDH1-3	Predicted malate dehydrogenase; farnesol regulated; protein present in exponential and stationary growth phase yeast; Hap43p-repressed gene
PIS52039.1	29.03994565	28.9548038	29.27122499	28.92086105	28.94179577	29.45317582	0.968737613	0.96	0.0166	PIS52039.1	CPR3	Putative peptidyl-prolyl cis-trans isomerase; macrophage-induced protein; protein levels decrease in stationary phase yeast cultures; predicted mitochondrial localization; overlaps orf19.1551
PIS49491.1	24.36392952	24.66069474	24.91214679	24.72365444	24.71563346	24.54577321	0.975718887	0.961	0.0161	PIS49491.1	orf19.7316	Putative phytanoyl-CoA dioxygenase family protein; mutation confers hypersensitivity to 5-fluorocytosine (5-FC), 5-fluorouracil (5-FU), and tubercidin (7-deazaadenosine); induced by nitric oxide
PIS58525.1	31.95242677	31.48754166	31.92600143	31.68464646	31.88820258	31.83958803	0.960554046	0.96	0.0155	PIS58525.1	GSY1	Glycogen synthase (UDP glucose/starch glucosyltransferase); transcript repressed by yeast-hyphal switch, Efg1-regulated; strong oxidative stress induced; colony morphology-related regulation by Ssn6; stationary phase enriched
PIS52034.1	27.40492395	28.40474151	27.2787711	27.40543532	27.68967339	28.03917955	0.97150629	0.96	0.0153	PIS52034.1	POB3	Protein involved in chromatin assembly and disassembly; ortholog of S. cerevisiae Pob3; transposon mutation affects filamentous growth; rat catheter biofilm repressed

PIS58526.1	27.86905362	27.96422474	26.96451369	27.03732158	27.85100455	27.95215267	0.976848103	0.961	0.0142	PIS58526.1	orf19.6458.1	Ortholog(s) have U2 snRNA binding, U6 snRNA binding activity and role in P-body assembly, mRNA splicing, via spliceosome
PIS51470.1	28.50803068	29.72319594	27.31823613	27.83320954	28.84217134	28.91544515	0.983137916	0.961	0.0138	PIS51470.1	NPL3	Putative RNA-binding protein; required for normal flow model biofilm growth; nuclear export is facilitated by Hmt1p; transcript upregulated in RHE model of oralcandidiasis; Spider biofilm repressed
PIS50425.1	27.85590156	27.34662922	27.86904861	27.26450222	28.14253821	27.70557227	0.969417384	0.96	0.0137	PIS50425.1	ERG27	3-Keto sterol reductase of ergosterol biosynthesis; acts in C-4 sterol demethylation with Erg25p and Erg26p; possible drug target, essential for viability; functional homolog of S. cerevisiae Erg27p
PIS50583.1	27.90776392	27.27725276	27.95705239	28.18159749	27.39901659	27.60176818	0.980709456	0.961	0.0134	PIS50583.1	orf19.4184	Protein with a predicted role in clathrin cage assembly; Hap43-repressed; Spider biofilm repressed
PIS54771.1	24.79817357	25.85122084	23.55106352	24.241313	23.99191299	26.00712821	0.985093456	0.961	0.0133	PIS54771.1	orf19.4225.1	Ortholog(s) have oxoglutarate dehydrogenase (succinyl-transferring) activity, structural constituent of ribosome activity and role in 2-oxoglutarate metabolic process, tricarboxylic acid cycle
PIS58641.1	26.22649167	23.16460614	26.01663338	25.84909106	25.34866349	24.24885997	0.986662681	0.961	0.013	PIS58641.1	CDC46	Putative hexameric MCM complex subunit; predicted role in control of cell division; periodic mRNA expression. peak at cell-cycle M/G1 phase; regulated by tyrosol, cell density, Plc1; repressed by alpha pheromone in SpiderM medium
PIS54866.1	27.64428577	28.85664038	26.75078901	27.71725938	28.50012476	27.07323471	0.983587652	0.961	0.013	PIS54866.1	orf19.3312	Protein of unknown function; flow model and Spider biofilm repressed
PIS49663.1	23.85109727	26.04239652	24.29024393	24.66998905	24.24799429	25.30226644	0.983488955	0.961	0.0122	PIS49663.1	orf19.527	Protein of unknown function; flow model biofilm induced
PIS58973.1	35.46696376	35.11121695	35.65408242	35.57191336	35.39184168	35.30431772	0.969967082	0.96	0.0119	PIS58973.1	PET9	Mitochondrial ADP/ATP carrier protein involved in ATP biosynthesis; possible lipid raft component; 3 predicted transmembrane helices; flucytosine induced; ketoconazole-induced; downregulated by Efg1p
PIS52301.1	28.83130435	28.76995457	29.01893397	28.90833026	28.8953751	28.85069841	0.964338207	0.96	0.0114	PIS52301.1	orf19.5229	Ortholog(s) have 3'-5'-RNA exonuclease activity, RNA endonuclease activity, RNA exonuclease activity, RNA nuclease activity, small GTPase binding, tRNA binding activity

PIS51338.1	30.17134697	29.73646695	30.15519851	29.97000194	30.11967895	30.00299939	0.988410975	0.961	0.00989	PIS51338.1	orf19.4721	Ortholog(s) have RNA binding activity, role in mRNA processing, mitochondrial translation and mitochondrion localization
PIS51041.1	28.16731183	28.29137372	28.12826358	28.26758813	28.00378196	28.34472017	0.981535633	0.961	0.00971	PIS51041.1	KIN2	Protein with similarity to S. cerevisiae Kin2p, transcription is positively regulated by Tbf1
PIS58078.1	29.29860439	29.41813054	29.60912147	29.58159273	29.49230307	29.27978556	0.983758108	0.961	0.00927	PIS58078.1	MRPL8	Mitochondrial 60S ribosomal protein subunit; Hap43p-repressed gene
PIS56648.1	28.25162809	27.84583477	28.37348534	28.33651264	28.28199618	27.8795472	0.978584437	0.961	0.00904	PIS56648.1	orf19.1533	Possible vacuolar protein; Hap43-induced gene
PIS58950.1	25.11457134	26.18162066	26.55833704	25.91846406	25.97382265	25.98715416	0.988986716	0.961	0.0083	PIS58950.1	MET13	Putative methionine biosynthesis protein; ketoconazole-induced; amphotericin B repressed; Spider biofilm repressed
PIS51839.1	30.17667852	30.81540839	29.76536069	30.30379667	30.20117655	30.27688974	0.987337301	0.961	0.00814	PIS51839.1	ILV5	Ketol-acid reductoisomerase; antigenic; regulated by Gcn4; GlcNAc, amino acid starvation (3-AT)-induced; macrophage-repressed protein; protein present in exponential and stationary phase; flow model and Spider biofilm repressed
PIS56743.1	30.21701386	29.89034948	30.67859508	30.45786052	30.21158492	30.14045719	0.992981856	0.961	0.00798	PIS56743.1	orf19.2017	Ortholog(s) have RNA polymerase I activity and role in nucleolar large rRNA transcription by RNA polymerase I, transcription by RNA polymerase I, transcription elongation by RNA polymerase I
PIS51196.1	32.60255535	32.9088863	32.35790215	32.3837351	32.76529279	32.74056462	0.982444591	0.961	0.00675	PIS51196.1	IDH2	Putative mitochondrial NAD-isocitrate dehydrogenase subunit; induced by ciclopirox; induced in high iron; present in exponential and stationary growth phases; Spider biofilm repressed
PIS56559.1	27.03026255	27.07426308	27.69601938	27.41620243	27.1138287	27.2895562	0.99376735	0.961	0.00635	PIS56559.1	MSW1	Protein similar to S. cerevisiae Msw1p, which is mitochondrial tryptophanyl-tRNA synthetase; Hap43p-repressed gene; likely to be essential for growth, based on an insertional mutagenesis strategy
PIS54823.1	26.16782394	27.09069758	25.75890854	25.94514396	26.01206142	27.07859439	0.99446834	0.961	0.00612	PIS54823.1	orf19.6305	Hydroxytrimethyllysine aldolase, the second enzyme in the carnitine biosynthesis pathway; rat catheter biofilm repressed

PIS51085.1	25.14973159	25.62582403	25.1089616	23.41389425	26.20743739	26.28141013	0.993774416	0.961	0.00607	PIS51085.1	orf19.4286	Protein of unknown function; flow model biofilm induced; Spider biofilm induced
PIS50422.1	29.35283358	29.83127437	29.16791039	29.17399038	29.72076613	29.47315435	0.989280104	0.961	0.0053	PIS50422.1	orf19.3235	Putative F-actin capping protein subunit alpha; possibly an essential gene, disruptants not obtained by UAU1 method
PIS54762.1	25.85613415	25.8274855	25.05241862	25.70537169	25.56777689	25.47794877	0.993786033	0.961	0.00502	PIS54762.1	SIP5	Protein of unknown function; flow model, rat catheter and Spider biofilm induced
PIS54680.1	33.82872454	34.17885355	32.87017768	33.33376033	33.78202791	33.77649278	0.989791806	0.961	0.00484	PIS54680.1	PIL1	Eisosome component; predicted role in endocytosis; echinocandin-binding protein; localizes to cell surface of hyphae, but not yeast-form cells; Hap43, YNB biofilm induced; rat catheter biofilm repressed
PIS55580.1	28.23344371	28.59584378	27.80571624	27.73578418	28.47579328	28.43583643	0.992761047	0.961	0.00414	PIS55580.1	MEU1	Putative methylthioadenosine phosphorylase; protein level decreases in stationary phase cultures
PIS51613.1	26.17708146	27.00885636	26.52018159	27.23894357	26.26854706	26.21059021	0.995249481	0.961	0.00399	PIS51613.1	orf19.1793	Ortholog(s) have phosphatidylinositol-3,5-bisphosphate binding, phosphatidylinositol-3-phosphate binding, phosphatidylinositol-4-phosphate binding, phosphatidylinositol-5-phosphate binding activity
PIS50579.1	25.26250193	25.90295709	25.82990865	25.77760765	25.6852448	25.54431594	0.996110753	0.961	0.00393	PIS50579.1	MMM1	Subunit of ER-mitochondrion tether ERMES complex, required for evasion of host immune response
PIS52371.1	28.12880563	27.04640873	28.16285776	28.38853091	28.05579445	26.90355971	0.994116746	0.961	0.00327	PIS52371.1	orf19.714	Ortholog(s) have GTPase activity
PIS54863.1	30.71229682	29.51530861	30.42091642	30.61163369	30.17864354	29.86660177	0.996054178	0.961	0.00279	PIS54863.1	SAR1	Functional homolog of S. cerevisiae Sar1; which is required for ER-to-Golgi protein transport; binds GTP; similar to small GTPase superfamily proteins; gene has intron; Hap43-induced; rat catheter biofilm repressed
PIS56881.1	30.21201072	30.4013994	30.04886539	30.25864353	30.22757129	30.18280291	0.993493213	0.961	0.00225	PIS56881.1	SLA2	Actin binding protein with roles in growth control and morphogenesis; required for alkaline pH-induced hyphal formation; localized to actin patches; rat catheter biofilm repressed

PIS48383.1	26.95889011	25.22659751	26.85807941	26.32989847	26.47902657	26.2373444	0.999086539	0.961	0.000901	PIS48383.1	orf19.2222	Putative casein kinase; plasma membrane-localized
PIS49765.1	24.66558057	25.03552847	23.9357965	25.36940372	24.60585861	23.66224842	0.999792519	0.961	0.000202	PIS49765.1	orf19.3607	Ortholog of C. dubliniensis CD36 : Cd36_22860, C. parapsilosis CDC317 : CPAR2_806540, C. auris B8441 : B9J08_004792 and Candida tenuis NRRL Y-1498 : CANTEDRAFT_108823
PIS48439.1	27.20793501	27.21722984	26.99713932	26.48102878	27.69339901	27.24389658	0.998586105	0.961	-0.00133	PIS48439.1	NMD3	Putative nonsense-mediated mRNA decay protein; repressed in core stress response; repressed by prostaglandins
PIS51243.1	29.30282694	30.08163257	29.54851852	29.40089561	29.73648954	29.79079858	0.997311052	0.961	-0.0016	PIS51243.1	BCY1	Protein kinase A regulatory subunit; involved in regulation of filamentation, phenotypic switching and mating; required for nuclear localization of Tpk1; physically interacts with Tpk1; apoptosis-regulated
PIS48527.1	28.65973043	29.0406868	28.78896832	28.53694123	28.91160866	29.03534832	0.99815272	0.961	-0.00183	PIS48527.1	VPH1	Vacuolar H(+)-ATPase; transcription regulated by Nrg1, Mig1, and Tup1
PIS56640.1	25.98890372	27.36434284	25.82505074	26.21008382	26.12942001	26.8291614	0.994656637	0.961	-0.00321	PIS56640.1	MCI4	Putative NADH-ubiquinone dehydrogenase; Hap43p-repressed gene
PIS56734.1	29.52355439	29.75722055	29.06698366	29.15258257	29.31996115	29.86352286	0.991134618	0.961	-0.0039	PIS56734.1	KEL1	Kelch repeat domain-containing protein; localizes to sites of polarized growth; mutant colonies exhibit slightly decreased filamentation ratio; not required for buccal epithelial cell adherence or virulence in mice
PIS58618.1	33.28251965	32.97040207	33.20951816	33.10984861	33.27612792	33.06368716	0.987257043	0.961	-0.00426	PIS58618.1	RPL11	Ribosomal protein; repressed by phagocytosis; colony morphology-related gene regulation by Ssn6; Hap43-induced; Spider biofilm repressed
PIS54992.1	31.97293238	31.68460732	32.13757412	32.04114022	31.71684537	32.02416527	0.994368862	0.961	-0.00432	PIS54992.1	CYP1	Peptidyl-prolyl cis-trans isomerase; cyclosporin A sensitive activity; soluble in hyphae; biofilm induced, macrophage-induced protein; downregulated upon treatment of biofilm with farnesol; present in exponential and stationary phase cells
PIS49642.1	28.40236049	27.96284082	28.4820986	28.59674315	27.97243413	28.26428714	0.990252428	0.961	-0.00461	PIS49642.1	orf19.3064.1	Ortholog of C. parapsilosis CDC317 : CPAR2_108915, C. auris B8441 : B9J08_004667, Candida tenuis NRRL Y-1498 : CANTEDRAFT_102387 and Debaryomyces hansenii CBS767 : DEHA2B06776g

PIS51373.1	25.96429696	26.29768411	25.66990781	25.53519692	26.32995628	26.05108726	0.995284909	0.961	-0.00522	PIS51373.1	LAS1	Putative bud formation and morphogenesis protein; mutation confers hypersensitivity to 5-fluorocytosine (5-FC), 5-fluorouracil (5-FU); macrophage-induced; Spider biofilm induced
PIS51619.1	28.57342197	28.26875634	28.40260245	28.43306092	28.61860424	28.17661531	0.988507005	0.961	-0.0055	PIS51619.1	orf19.1800	Protein of unknown function; Spider biofilm induced
PIS50372.1	25.60827105	24.47520745	25.39147584	25.38293617	25.69227094	24.38318858	0.992882249	0.961	-0.00552	PIS50372.1	orf19.1412	Ortholog of C. dubliniensis CD36 : Cd36_44020, C. parapsilosis CDC317 : CPAR2_302330, C. auris B8441 : B9J08_004187 and Candida tenuis NRRL Y-1498 : CANTEDRAFT_105448
PIS51362.1	27.34723732	26.60505873	26.59887318	27.18556477	27.62137386	25.72763288	0.994058046	0.961	-0.00553	PIS51362.1	MED14	RNA polymerase II mediator complex subunit
PIS50480.1	29.40041131	29.28431332	29.20756504	29.55742851	29.20046239	29.11683084	0.990053109	0.961	-0.00586	PIS50480.1	orf19.3984	Protein of unknown function; induced in core caspofungin response; induced in ssr1 mutant; induced by nitric oxide independent of Yhb1; Spider biofilm induced
PIS56618.1	32.84873522	32.83201051	32.95884905	32.90729207	32.88286349	32.83004981	0.979936038	0.961	-0.00646	PIS56618.1	GUS1	Putative glutamine-tRNA ligase; stationary phase enriched protein; Spider biofilm repressed
PIS49660.1	29.76856051	30.41774503	29.67106287	29.80690455	29.94254404	30.08504877	0.979382233	0.961	-0.00762	PIS49660.1	orf19.6507	Putative curved DNA-binding protein orthologous to S. pombe Cdb4; stationary phase enriched protein; rat catheter and Spider biofilm repressed
PIS52422.1	33.88066261	34.14048435	34.15492207	33.96853882	33.97959993	34.20427218	0.980580952	0.961	-0.00789	PIS52422.1	MIR1	Putative mitochondrial phosphate transporter; caspofungin repressed; expression is increased in a fluconazole-resistant isolate; induced upon adherence to polystyrene
PIS51408.1	30.84693322	31.30055374	31.25889094	31.37435508	31.03694735	30.96921435	0.976690132	0.961	-0.00862	PIS51408.1	orf19.4396	Mitochondrial inner membrane protein; mammalian mitofilin domain; Spider biofilm repressed
PIS52132.1	26.27816917	26.9228599	26.98643188	26.66481419	27.09853254	26.39748768	0.992803765	0.961	-0.00888	PIS52132.1	orf19.5322	Ortholog(s) have phosphatidylinositol-3-phosphate binding activity

PIS49648.1	29.28219816	29.13288838	29.09191725	29.13432997	29.15226195	29.19374439	0.985932272	0.961	-0.00889	PIS49648.1	orf19.1860.1	Has domain(s) with predicted peptidase activity and role in proteolysis
PIS52469.1	26.63229118	24.70904625	26.16125869	25.98439019	26.09774612	25.39373225	0.991325841	0.961	-0.00891	PIS52469.1	orf19.2827	Ortholog(s) have kinetochore adaptor activity, microtubule binding activity
PIS51331.1	26.6002029	26.03731797	26.85977048	26.31922513	26.34448662	26.80596758	0.982256902	0.961	-0.0092	PIS51331.1	orf19.5275	Ortholog(s) have ATPase activator activity, protein-macromolecule adaptor activity and role in late endosome to vacuole transport, late endosome to vacuole transport via multivesicular body sorting pathway
PIS58058.1	23.78795247	23.80535558	24.73861104	24.14818636	24.10475528	24.05065871	0.984569902	0.961	-0.00944	PIS58058.1	CTM1	Lysine methyltransferase that trimethylates cytochrome c; involved in repression of hyphal growth via methylation activity; regulated by Gcn2 and Gcn4; transcript induced by Mnl1 under weak acid stress
PIS54966.1	32.31766378	31.25598866	32.3096678	32.38212679	31.92784145	31.54436257	0.991518477	0.961	-0.00966	PIS54966.1	orf19.6701	Protein with similarity to amino acid-tRNA ligase; stationary phase enriched protein; GlcNAc-induced protein
PIS48715.1	24.66260186	25.21660467	25.14354187	25.50844614	24.56195561	24.92323306	0.99092192	0.961	-0.0097	PIS48715.1	orf19.7036	Ortholog(s) have protein phosphatase binding activity
PIS49547.1	28.13195421	28.55903531	28.75566325	28.30382587	28.66484788	28.4486859	0.991469456	0.961	-0.00976	PIS49547.1	orf19.2473	Component of the RSC chromatin remodeling complex; decreased transcription is observed upon fluphenazine treatment or in an azole-resistant strain that overexpresses CDR1 and CDR2
PIS51571.1	25.2419557	23.03301447	26.18789934	24.68318891	24.92640317	24.82397128	0.991871791	0.961	-0.00977	PIS51571.1	orf19.6064	Phosphatidylinositol-3-phosphate-binding protein; component of the core autophagy machinery; gets recruited to the phagophore assembly site during onset of autophagy
PIS51700.1	23.26850917	22.45592591	22.6076137	23.22523324	22.3054955	22.76926367	0.989580627	0.961	-0.0107	PIS51700.1	orf19.4642	Protein of unknown function; Hap43-induced gene
PIS54606.1	24.76662412	25.67926856	23.97705272	25.0498165	24.46789074	24.87068091	0.993079748	0.961	-0.0115	PIS54606.1	orf19.7444	Ortholog(s) have transcription factor TFIIH holo complex localization

PIS51248.1	31.34852746	30.7844006	31.74887787	31.41231983	31.24725066	31.18773281	0.97013102	0.96	-0.0115	PIS51248.1	SPF1	P-type calcium-transporting ATPase, involved in control of calcium homeostasis, response to ER stress, hyphal growth, biofilm formation and virulence
PIS49694.1	23.98075218	24.52915683	24.18204334	25.13883852	24.0358714	23.48191258	0.985677934	0.961	-0.0118	PIS49694.1	orf19.4744	Putative phosphatidylinositol 3-phosphate (PI3P) phosphatase; repressed by alpha pheromone in SpiderM medium
PIS51097.1	34.83045102	34.42769469	34.87490928	34.82805463	34.67153597	34.59631945	0.986517089	0.961	-0.0124	PIS51097.1	PCK1	Phosphoenolpyruvate carboxykinase; glucose, C-source, yeast-hypha, Hap43 regulated; fluconazole, phagocytosis, H2O2, oral candidasis, Spider/rat catheter/flow model biofilm induced; repressed in biofilm by Bcr1, Tec1, Ndt80, Rob1, Brg1
PIS48551.1	29.68719161	29.92501473	29.31524861	29.27409845	29.65224357	29.96261585	0.98109153	0.961	-0.0128	PIS48551.1	PGA4	GPI-anchored cell surface protein; beta-1,3-glucanosyltransferase with similarity to the A. fumigatus GEL family; transcript induced in RHE model of oral candidiasis; fluconazol-induced
PIS48288.1	24.3759105	24.90014925	25.2503931	25.11061456	25.09788255	24.27511186	0.965202367	0.96	-0.0143	PIS48288.1	orf19.7347	Ortholog(s) have ubiquitin-protein transferase activity, role in protein monoubiquitination, protein polyubiquitination, ubiquitin-dependent ERAD pathway and endoplasmic reticulum membrane localization
PIS48683.1	30.87709673	31.66721866	29.94338377	30.71037399	30.79184531	30.94161229	0.982961902	0.961	-0.0146	PIS48683.1	TIF3	Putative translation initiation factor; genes encoding ribosomal subunits, translation factors, and tRNA synthetases are downregulated upon phagocytosis by murine macrophage
PIS51777.1	29.40615256	28.96445633	29.6152139	29.39877483	29.20521099	29.33766127	0.978034078	0.961	-0.0147	PIS51777.1	PHO85	Functional homolog of S. cerevisiae Pho85p, a cyclin-dependent kinase that regulates transcription of PHO genes involved in phosphate metabolism; necessary for geldanamycin-induced filamentation; gene has intron
PIS56849.1	27.98203389	27.67093511	27.98754798	27.93506149	27.61563098	28.04457906	0.984343273	0.961	-0.0151	PIS56849.1	ERV29	Putative SURF4 family member; plasma membrane-localized; flow model biofilm repressed
PIS54694.1	28.60999201	28.92797705	28.49210109	28.39939841	28.76660859	28.81877307	0.968252435	0.96	-0.0151	PIS54694.1	orf19.2520	Ortholog(s) have structural constituent of ribosome activity and mitochondrial small ribosomal subunit localization
PIS58262.1	27.95678018	29.53425881	28.57800773	29.10354181	28.45726274	28.46180429	0.971574966	0.96	-0.0155	PIS58262.1	LYS12	Homoisocitrate dehydrogenase; catalyzes 4th step in the alpha-aminoadipate pathway of lysine biosynthesis; clade-associated gene expression; protein level decreases in stationary phase cultures; Spider biofilm repressed

PIS48742.1	26.81217601	26.68969238	26.93065226	26.73990811	27.02010136	26.62537542	0.970637081	0.96	-0.0157	PIS48742.1	SAM35	Predicted component of the sorting and assembly machinery (SAM complex) of the mitochondrial outer membrane, involved in protein import into mitochondria
PIS58954.1	25.55816576	25.31873166	25.85870558	25.60121927	25.42320774	25.66309625	0.980037966	0.961	-0.016	PIS58954.1	orf19.252	Protein of unknown function; S. cerevisiae ortholog Fmp37 which localizes to mitochondria; Hap43-repressed; Spider biofilm repressed
PIS52426.1	30.89869114	30.668932	30.92848054	30.77695136	30.76163764	30.90896092	0.959681835	0.96	-0.0162	PIS52426.1	SAC1	Putative phosphatidylinositol phosphate (PtdInsP) phosphatase, involved in cell wall integrity and morphogenesis
PIS54487.1	29.28697544	29.33851766	29.03861111	29.43371905	29.00989886	29.16641618	0.965338312	0.96	-0.018	PIS54487.1	NCE102	Non classical protein export protein; localized to plasma membrane; Hap43-induced gene; flow model biofilm induced; Spider biofilm induced
PIS54787.1	26.67772705	26.12285902	26.32782984	26.47257913	26.26510651	26.33649507	0.964134406	0.96	-0.0181	PIS54787.1	MIM1	Predicted mitochondrial protein involved in outer membrane protein import; rat catheter biofilm repressed
PIS54974.1	28.1100464	27.95899743	28.17028729	28.13809544	27.9106347	28.13635335	0.969721508	0.96	-0.0181	PIS54974.1	OCH1	Alpha-1,6-mannosyltransferase; initiates N-glycan outer chain branch addition; similar to S. cerevisiae Och1p; required for wild-type virulence in mouse intravenous infection; fungal-specific (no human or murine homolog)
PIS58998.1	32.10296076	31.72356369	32.18012464	31.89734035	32.1172236	31.93727207	0.973577977	0.96	-0.0183	PIS58998.1	RPS27	Putative ribosomal protein; repressed upon phagocytosis by murine macrophage; Spider biofilm repressed
PIS50613.1	24.24818157	24.39412937	24.63696921	25.4110321	24.24706559	23.56595937	0.967371471	0.96	-0.0184	PIS50613.1	orf19.6968	Protein of unknown function; Hog1-repressed; Spider biofilm induced
PIS58306.1	30.45497419	30.49658827	29.86639806	29.94512656	30.32193938	30.49387824	0.954208929	0.96	-0.019	PIS58306.1	ADE13	Adenylosuccinate lyase; enzyme of adenine biosynthesis; soluble protein in hyphae; not induced during GCN response, in contrast to the S. cerevisiae ortholog; repressed by nitric oxide
PIS51027.1	29.55324501	29.86599602	29.29559118	29.25665	29.80790763	29.5928601	0.962219475	0.96	-0.0191	PIS51027.1	orf19.2940	Putative v-SNARE of the endoplasmic reticulum membrane; possibly an essential gene, disruptants not obtained by UAU1 method

PIS58801.1	32.62736455	32.54993969	32.09916316	32.16946877	32.48347137	32.56488112	0.956009673	0.96	-0.0195	PIS58801.1	RPS17B	Ribosomal protein 17B; downregulated upon phagocytosis by murine macrophages; Hap43-induced; Spider biofilm repressed
PIS52455.1	30.3395739	29.77258871	30.44744115	30.2522282	30.27430334	29.97309131	0.971958002	0.96	-0.02	PIS52455.1	QCR2	Ubiquinol-cytochrome-c reductase; antigenic; induced by interaction with macrophage; repressed by nitric oxide; in detergent-resistant membrane fraction (possible lipid raft component); levels decrease in stationary phase; Hap43p-repressed
PIS58762.1	27.77662327	27.57508741	28.03557437	27.68741165	27.72652534	27.91303369	0.961893652	0.96	-0.0201	PIS58762.1	orf19.2690	Putative mitochondrial GTPase; required for mitochondrial morphology and genome maintenance; Spider biofilm induced
PIS56911.1	28.47444635	28.70610531	28.58402855	28.50708247	28.48618286	28.71031035	0.976611295	0.961	-0.0203	PIS56911.1	RCY1	Putative F-box protein involved in endocytic membrane traffic and/or recycling; fungal-specific (no human or murine homolog)
PIS48545.1	32.76189103	33.69119539	32.3628194	32.89008571	32.74464045	33.11903428	0.959562133	0.96	-0.0207	PIS48545.1	FBA1	Fructose-bisphosphate aldolase; glycolytic enzyme; antigenic in murine/human infection; regulated by yeast-hypha switch; induced by Efg1, Gcn4, Hog1, fluconazole; phagocytosis-repressed; flow model biofilm induced; Spider biofilm repressed
PIS51914.1	27.99135915	28.94695104	28.36778177	28.76423242	28.23111015	28.24877299	0.95649054	0.96	-0.0207	PIS51914.1	orf19.374	Ortholog(s) have role in protein transport to vacuole involved in ubiquitin-dependent protein catabolic process via the multivesicular body sorting pathway
PIS54613.1	31.10152927	30.97549312	31.0163588	31.74770385	30.63404346	30.64905816	0.956608443	0.96	-0.0209	PIS54613.1	UBA1	Ubiquitin-activating enzyme; protein level decreases in stationary phase cultures
PIS51175.1	28.49775882	28.48088924	29.12035937	28.77071623	28.64419418	28.62138892	0.951052501	0.96	-0.0209	PIS51175.1	UBC4	Ortholog(s) have proteasome binding, protein-macromolecule adaptor activity, ubiquitin binding, ubiquitin conjugating enzyme activity, ubiquitin-protein transferase activity
PIS54619.1	27.29306924	26.61190252	27.97414318	27.65637338	26.96394758	27.19590543	0.967631301	0.96	-0.021	PIS54619.1	orf19.1386	Ortholog(s) have SNAP receptor activity and role in endoplasmic reticulum to Golgi vesicle-mediated transport, retrograde vesicle-mediated transport, Golgi to endoplasmic reticulum, vesicle fusion
PIS48412.1	27.57233846	27.60896814	27.81781831	27.72270993	27.80406873	27.40874919	0.977353015	0.961	-0.0212	PIS48412.1	orf19.5852	Protein of unknown function; rat catheter biofilm repressed

PIS49744.1	29.57170812	27.84728597	28.94084911	29.3769393	28.96050549	27.95852518	0.962310374	0.96	-0.0213	PIS49744.1	orf19.6769	Ortholog of S. cerevisiae : OCA5, C. glabrata CBS138 : CAGL0E03784g, C. dubliniensis CD36 : Cd36_87200, C. parapsilosis CDC317 : CPAR2_809020 and C. auris B8441 : B9J08_004771
PIS51511.1	27.0942757	26.64864135	26.77699772	26.47481019	27.02329718	26.95739508	0.967260369	0.96	-0.0215	PIS51511.1	SMD2	Putative Core Sm protein; Hap43p-induced gene; flucytosine induced
PIS56867.1	31.06159233	31.16545889	31.16768633	31.0260606	31.1071036	31.19717939	0.938593668	0.959	-0.0215	PIS56867.1	TYS1	Putative tRNA-Tyr synthetase; downregulated upon phagocytosis by murine macrophages; stationary phase enriched protein; Spider biofilm repressed
PIS54516.1	29.66427357	29.09184116	29.78770776	29.70676511	29.27091311	29.50045349	0.953439753	0.96	-0.0219	PIS54516.1	ADE4	Putative phosphoribosylpyrophosphate amidotransferase; flucytosine induced
PIS52256.1	26.87366626	27.15183036	26.82513423	26.89331621	27.18816812	26.70166935	0.977765263	0.961	-0.0225	PIS52256.1	orf19.7198	CCR4-NOT complex component; involved in controlling mRNA initiation, elongation and degradation; rat catheter biofilm induced
PIS52401.1	25.33029316	23.72469774	25.42642006	25.64279152	23.90271084	24.86396134	0.966773103	0.96	-0.024	PIS52401.1	orf19.3627	Ortholog of C. dubliniensis CD36 : Cd36_22640, C. parapsilosis CDC317 : CPAR2_406910, C. auris B8441 : B9J08_004017 and Candida tenuis NRRL Y-1498 : CANTEDRAFT_104937
PIS51949.1	24.96019899	24.56681884	25.08939026	24.72818644	25.0072756	24.80868003	0.97367918	0.96	-0.0241	PIS51949.1	orf19.993	Protein of unknown function; rat catheter biofilm repressed
PIS48231.1	29.84967173	30.55367824	30.07486389	29.89750105	30.15607422	30.35233266	0.962914238	0.96	-0.0241	PIS48231.1	SPE3	Putative spermidine synthase; predicted role in pantothenate and spermidine biosynthesis; Spider biofilm repressed
PIS50561.1	24.77180796	24.96437005	25.88450046	25.30962131	24.56837463	25.66996875	0.977490926	0.961	-0.0242	PIS50561.1	GRR1	F-box protein component of the SCF ubiquitin-ligase complex required for cell cycle progression; involved in negative control of pseudohyphal growth; regulates stability of Ume6p in response to CO2
PIS52459.1	28.44156882	27.83697989	28.56499561	27.43052777	28.71043589	28.62689367	0.953890839	0.96	-0.0252	PIS52459.1	FUR1	Uracil phosphoribosyltransferase; predicted tetrameric enzyme of pyrimidine salvage; mutations associated with flucytosine resistance in clade I clinical isolates; flucytosine, macrophage-induced protein; levels decrease in stationary phase

PIS54939.1	27.64374916	27.87515688	29.66787962	28.66096276	28.34031908	28.10930645	0.96477404	0.96	-0.0254	PIS54939.1	RHB1	Putative small G protein from the Ras superfamily involved in cell wall integrity and control of filamentous growth under nitrogen starvation; involved in activation of TOR1C during starvation response
PIS48544.1	26.34056207	25.77244115	26.29621371	26.42257138	26.05304385	25.85617517	0.948089901	0.959	-0.0258	PIS48544.1	orf19.4621	Ortholog(s) have P-body localization
PIS48790.1	25.38711588	25.46924771	25.1893315	25.64005452	24.75689077	25.56956352	0.942608374	0.959	-0.0264	PIS48790.1	FPG1	Formamidopyrimidine DNA glycosylase, involved in repair of gamma-irradiated DNA; Hap43p-repressed gene
PIS49749.1	24.77041201	24.50151743	25.28022375	24.36913734	24.96340762	25.13905299	0.949354075	0.959	-0.0269	PIS49749.1	orf19.4736	Ortholog(s) have alkaline phosphatase activity, zinc ion sensor activity, role in nicotinamide nucleotide metabolic process and fungal-type vacuole, fungal-type vacuole membrane localization
PIS48303.1	32.37334346	32.02850098	32.31041997	31.81901924	32.27609643	32.53376427	0.938550818	0.959	-0.0278	PIS48303.1	orf19.3335	Plasma membrane protein of unknown function; colony morphology-related gene regulation by Ssn6; repressed by nitric oxide
PIS52433.1	30.33557867	30.58593595	30.15890933	30.18693374	30.51551529	30.29466408	0.975167522	0.96	-0.0278	PIS52433.1	orf19.4846	GlcNAc-induced protein
PIS54601.1	25.68272496	26.33246621	25.87193088	26.74616614	25.61676978	25.44027452	0.96855056	0.96	-0.028	PIS54601.1	LIP1	Secreted lipase, member of a lipase gene family whose members are expressed differentially in response to carbon source and during infection; may have a role in nutrition and/or in creating an acidic microenvironment
PIS49483.1	27.81939423	27.06865312	28.1861771	27.38883872	27.60125072	27.9988417	0.958487302	0.96	-0.0284	PIS49483.1	orf19.7322	Protein of unknown function; S. cerevisae ortholog Ypl225w interacts with ribosomes; rat catheter biofilm induced
PIS54746.1	25.29624615	27.05273436	25.3692679	26.5718678	25.33413669	25.72438529	0.962948986	0.96	-0.0293	PIS54746.1	CUE5	Predicted ubiquitin-binding protein; rat catheter biofilm repressed
PIS58913.1	29.97940013	29.73680267	29.94349782	29.6335041	29.91534501	30.02192896	0.961333043	0.96	-0.0296	PIS58913.1	ARD	D-arabitol dehydrogenase, NAD-dependent (ArDH); enzyme of D-arabitol and D-arabinose catabolism; D-arabitol is a marker for active infection in humans; rat catheter and Spider biofilm induced

PIS54960.1	26.21478152	27.63439411	26.72771359	26.46238034	27.00609455	27.01900242	0.975841747	0.961	-0.0298	PIS54960.1	ENG1	Endo-1,3-beta-glucanase; controls exposure of cell wall beta-glucan to host immune system; caspofungin, fluconazole repressed; repressed by alpha pheromone in SpiderM medium; flow model biofilm induced; rat catheter biofilm repressed
PIS51472.1	29.1730906	28.76130511	29.01485818	28.7940734	29.13740581	28.92783519	0.921167546	0.958	-0.03	PIS51472.1	TIF35	Putative translation initiation factor; repressed upon phagocytosis by murine macrophage; Spider biofilm repressed
PIS49640.1	23.87758015	23.92471106	24.5652171	23.87367952	24.20764936	24.19423386	0.933990224	0.959	-0.0306	PIS49640.1	orf19.71	Ortholog(s) have protein-containing complex binding activity, role in endoplasmic reticulum to Golgi vesicle-mediated transport, protein secretion and Golgi membrane, cis-Golgi network, cytoplasm localization
PIS55583.1	28.10792238	28.37552097	28.48306793	28.00350867	28.33549678	28.53487335	0.926115193	0.958	-0.0309	PIS55583.1	ISN1	Putative inosine 5'-monophosphate 5'-nucleotidase; fungal-specific (no human or murine homolog)
PIS51496.1	26.76457988	25.23705892	26.30430545	25.35059396	26.49570733	26.3663582	0.968080525	0.96	-0.0311	PIS51496.1	SER2	Ortholog(s) have L-phosphoserine phosphatase activity and role in L-serine biosynthetic process
PIS48305.1	29.02704126	29.07807747	28.19265908	28.74270406	28.71662215	28.74416053	0.964848239	0.96	-0.0314	PIS48305.1	orf19.3333	Ortholog(s) have 5S rRNA binding, 7S RNA binding, poly(A) binding, ribonuclease P RNA binding, tRNA binding activity
PIS48757.1	27.03444906	27.4454437	26.65860467	27.21300671	26.89638583	26.93376956	0.960408113	0.96	-0.0318	PIS48757.1	orf19.6908	Dihydrofolate synthetase involved in folic acid biosynthesis
PIS51018.1	27.13012717	28.12225157	26.15535768	26.26121133	27.45481	27.59600113	0.95337666	0.96	-0.0319	PIS51018.1	orf19.6077	Putative protein of unknown function; shows colony morphology-related gene regulation by Ssn6p
PIS48745.1	30.06450512	30.03721919	30.07569162	30.00413794	30.0324827	30.04365798	0.902451443	0.957	-0.0324	PIS48745.1	GDI1	Putative Rab GDP-dissociation inhibitor; GlcNAc-induced protein; Spider biofilm repressed
PIS58817.1	28.4267471	28.11844877	28.62427057	28.62061049	28.3799453	28.07024602	0.918537367	0.958	-0.0329	PIS58817.1	orf19.392	Ortholog(s) have role in mitochondrial respiratory chain complex III assembly and mitochondrial membrane, mitochondrion localization

PIS58934.1	27.94398413	28.79895394	28.20765827	28.07267397	28.44474223	28.33434426	0.926460866	0.958	-0.0329	PIS58934.1	orf19.7069	Putative AdoMet-dependent proline methyltransferase; Hap43-induced; required for normal flow model biofilm growth; Spider biofilm repressed
PIS49674.1	33.06191153	33.09108621	32.82074912	32.79248951	33.14129049	32.94073591	0.962926646	0.96	-0.0331	PIS49674.1	RPS25B	Ribosomal protein; macrophage/pseudohyphal-induced after 16 h; repressed upon phagocytosis by murine macrophage; transcript positively regulated by Tbf1; 5'-UTR intron; Hap43-induced; Spider biofilm repressed
PIS49567.1	30.37335619	29.63840025	30.86862248	30.44546456	30.19206179	30.14218063	0.935899715	0.959	-0.0336	PIS49567.1	YHM2	Predicted carrier protein; exports citrate from and imports oxoglutarate into the mitochondrion; alkaline induced; Spider biofilm repressed
PIS51971.1	34.71488779	34.86528914	34.76480042	34.48445514	34.93665476	34.82230278	0.890719027	0.957	-0.0339	PIS51971.1	RPS4A	Predicted ribosomal protein, component of the small ribosomal subunit; repressed upon phagocytosis by murine macrophage; positively regulated by Tbf1; mutant is defective in filamentous growth and sensitive to osmotic stress
PIS48645.1	28.47286621	28.17266065	28.72490398	28.3913216	28.53352546	28.34363089	0.961034832	0.96	-0.034	PIS48645.1	BOI2	Putative SH3-domain-containing protein; mutation confers hypersensitivity to toxic ergosterol analog; Spider biofilm induced
PIS48760.1	24.04987058	24.67827482	23.99597358	24.37876102	24.33702958	23.90390278	0.979535147	0.961	-0.0348	PIS48760.1	MDM12	Ortholog(s) have lipid transfer activity
PIS52337.1	31.88169189	32.20765129	31.95525471	31.89293243	32.04980144	31.9951276	0.962656229	0.96	-0.0356	PIS52337.1	ADE2	Phosphoribosylaminoimadazole carboxylase; role in adenine biosynthesis; required for normal growth and virulence in immunosuppressed mouse infection; not induced in GCN response, in contrast to S. cerevisiae ADE2; stationary phase-enriched
PIS54504.1	29.60806498	29.29830177	30.04460871	29.69712972	29.37170672	29.77260873	0.918319074	0.958	-0.0365	PIS54504.1	orf19.5965	Ortholog(s) have ubiquitin-ubiquitin ligase activity
PIS58492.1	24.52851615	23.92781606	25.11215125	24.21169321	24.57308639	24.67219797	0.936508092	0.959	-0.0372	PIS58492.1	SEN2	Putative tRNA splicing endonuclease subunit; mutation confers hypersensitivity to toxic ergosterol analog and to amphotericin B; 5'-UTR intron; Hap43-induced; Spider biofilm induced
PIS55032.1	29.78060341	30.19045595	29.58542071	29.64124238	29.97149186	29.83080055	0.934508313	0.959	-0.0376	PIS55032.1	orf19.1514	Ortholog(s) have enzyme binding, phosphatidylinositol-4-phosphate binding activity

PIS56557.1	25.78405673	28.29636422	26.70520349	26.25183821	26.0519539	28.36850261	0.955220545	0.96	-0.0378	PIS56557.1	orf19.4295	Component of the HIR complex, a nucleosome assembly factor involved in chromatin formation; involved in regulation of white-opaque switching frequency, hyphal initiation, and azole sensitivity
PIS56874.1	31.1103299	31.25459524	30.31093552	30.02989575	31.29401856	31.23730242	0.926684396	0.958	-0.0382	PIS56874.1	BFR1	Protein involved in the maintenance of normal ploidy; S. cerevisiae ortholog cofers Brefeldin A resistance; stationary phase enriched protein; Spider biofilm repressed
PIS58551.1	29.41518139	29.58480128	28.83109544	29.05796417	29.32827532	29.32778902	0.928894136	0.959	-0.039	PIS58551.1	orf19.3342	Ortholog(s) have role in positive regulation of protein autoubiquitination, protein deubiquitination
PIS54932.1	25.96043117	25.84082847	26.1436126	25.97923333	25.83248524	26.01142331	0.915516473	0.958	-0.0406	PIS54932.1	IMP4	Putative SSU processome component; Hap43-induced; repressed by prostaglandins; Spider biofilm induced
PIS48489.1	25.97135448	27.34138499	24.79203972	26.79216645	25.7603318	25.42981683	0.944217038	0.959	-0.0408	PIS48489.1	orf19.225	Predicted 2-hydroxyacid dehydrogenase; Hap43-repressed gene
PIS50578.1	23.75706052	24.60114618	25.17240685	25.05795356	24.67833597	23.6671123	0.939894311	0.959	-0.0424	PIS50578.1	orf19.4252	Ortholog(s) have ATPase, protein serine/threonine kinase activity
PIS58708.1	26.64028511	26.8720056	27.2459195	26.67576723	26.96165841	26.99298806	0.944827483	0.959	-0.0426	PIS58708.1	orf19.1485	Mitochondrial ribosomal protein of the large subunit; rat catheter biofilm induced
PIS54573.1	31.22221823	30.456037	31.34085887	31.01974907	31.05512132	30.81541797	0.894375208	0.957	-0.0429	PIS54573.1	ERG5	Putative C-22 sterol desaturase; fungal C-22 sterol desaturases are cytochrome P450 enzymes of ergosterol biosynthesis, catalyze formation of the C-22(23) double bond in the sterol side chain; transposon mutation affects filamentous growth
PIS58316.1	28.86141295	28.26576608	28.73718572	28.34446028	28.66991621	28.72109681	0.904286928	0.957	-0.043	PIS58316.1	ARC15	Putative ARP2/3 complex subunit; mutation confers hypersensitivity to cytochalasin D
PIS59026.1	28.41379474	28.95190714	28.32213781	28.87587966	28.16340333	28.51820337	0.922442352	0.958	-0.0435	PIS59026.1	CYB2	Putative cytochrome b2 precursor; induced in high iron; alkaline repressed; colony morphology-related gene regulation by Ssn6; Hap43-repressed; pider biofilm induced

PIS48802.1	29.83951012	29.68953729	29.13745976	29.32600944	29.56164481	29.64761112	0.880844016	0.956	-0.0437	PIS48802.1	NUP85	Ortholog of S. cerevisiae Nup85; a structural constituent of the nuclear pore; required for alkaline-induced hyphal morphogenesis and for SD or Spider media biofilm formation
PIS48356.1	29.35275522	28.80089389	29.50728189	29.25526202	29.21335855	29.06088801	0.884615226	0.957	-0.0438	PIS48356.1	orf19.585	Ortholog(s) have structural constituent of ribosome activity and mitochondrial large ribosomal subunit localization
PIS55623.1	29.18316605	29.47356899	28.65909806	29.03182031	29.18273722	28.96972018	0.90843783	0.958	-0.0439	PIS55623.1	orf19.5943	Ortholog(s) have role in peroxisome organization and peroxisomal membrane localization
PIS56766.1	24.85701805	22.42842794	25.05608687	24.61738226	23.62220996	23.96734503	0.952335437	0.96	-0.0449	PIS56766.1	orf19.962	Protein with a fungal RNA polymerase I subunit RPA14 domain; proposed to play a role in the recruitment of pol I to the promoter; Hap43-induced gene
PIS51856.1	30.40804687	31.43994776	29.80417289	30.05141004	30.68181611	30.7837622	0.936459882	0.959	-0.0451	PIS51856.1	orf19.5660	Ortholog(s) have ubiquitin protein ligase activity
PIS56795.1	29.64818327	29.82424399	29.78733177	29.61083036	29.89022208	29.6224333	0.886431475	0.957	-0.0454	PIS56795.1	RVS161	Protein required for endocytosis; contains a BAR domain, which is found in proteins involved in membrane curvature; null mutant exhibits defects in hyphal growth, virulence, cell wall integrity, and actin patch localization
PIS54706.1	28.62683159	27.3941207	28.19381245	28.39978571	27.83453605	27.84181238	0.939420819	0.959	-0.0462	PIS54706.1	LEM3	Putative membrane protein; mutation increases resistance to miltefosine; early-stage flow model biofilm induced
PIS56548.1	25.90251293	25.51355461	25.94240644	25.97280504	25.52636288	25.71914959	0.914162916	0.958	-0.0467	PIS56548.1	orf19.916	Protein that promotes apoptosis and negatively regulates filamentation; null mutant shows increased virulence and hyperfilamentation; Spider biofilm induced
PIS58280.1	31.41330736	31.91470207	30.891899	31.04049067	31.50357844	31.53253686	0.885127508	0.957	-0.0478	PIS58280.1	orf19.3037	Putative poly(A)-binding protein; regulated by Gcn4p; induced in response to amino acid starvation (3-AT treatment); protein present in exponential and stationary growth phase yeast cultures
PIS48767.1	22.57714634	23.54272383	23.4099368	23.95442648	22.55183431	22.87939402	0.9545036	0.96	-0.0481	PIS48767.1	orf19.4375	Ortholog(s) have N-terminal protein N-methyltransferase activity, S-adenosylmethionine-dependent methyltransferase activity, protein-lysine N-methyltransferase activity and role in rDNA heterochromatin formation

PIS52108.1	26.95941111	27.07916856	26.57180128	26.00300789	27.11285234	27.34949749	0.957545296	0.96	-0.0483	PIS52108.1	RIM2	Putative mitochondrial carrier protein; induced by alpha pheromone in SpiderM medium; Spider biofilm induced
PIS55827.1	27.31740476	28.26365383	27.89808248	27.35233658	27.58968356	28.39051187	0.944364265	0.959	-0.0489	PIS55827.1	orf19.2346	Putative protein of unknown function, transcription is positively regulated by Tbf1p
PIS56896.1	29.2217488	28.94593482	29.22404573	29.13242078	29.12870912	28.98098652	0.918297658	0.958	-0.0499	PIS56896.1	SRP54	Putative signal recognition particle (SRP) subunit; induced in ssr1 null; Spider biofilm repressed
PIS48522.1	26.64723489	27.72195727	27.23282907	26.68089997	27.60758046	27.16324605	0.92097534	0.958	-0.0501	PIS48522.1	SSK2	MAP kinase kinase kinase (MAPKKK); regulates Hog1 activation and signaling; repressed by ciclopirox olamine
PIS48793.1	32.99973426	33.17495577	33.04874078	33.1757397	33.02913065	32.86775324	0.914341604	0.958	-0.0503	PIS48793.1	ADK1	Putative adenylate kinase; repressed in hyphae; macrophage-induced protein; adenylate kinase release used as marker for cell lysis; possibly essential (UAU1 method); flow model biofilm induced; rat catheter and Spider biofilm repressed
PIS51816.1	30.45064069	30.46344146	30.7215021	30.73275856	30.42368569	30.32665247	0.894956999	0.957	-0.0508	PIS51816.1	NUC2	Putative NADH-ubiquinone oxidoreductase; identified in detergent-resistant membrane fraction (possible lipid raft component); alkaline repressed; Hap43-repressed; Spider biofilm repressed
PIS58910.1	25.82521778	25.25540088	25.67939245	25.5158615	25.43596914	25.65395324	0.952290597	0.96	-0.0514	PIS58910.1	ERG25	Putative C-4 methyl sterol oxidase; C4-demethylation of ergosterol biosynthesis intermediates, based on similarity to S. cerevisiae Erg25; fluconazole-induced; induced in azole-resistant strain; rat catheter and Spider biofilm induced
PIS58183.1	25.52202137	23.89862043	25.0917789	23.38298254	25.35185813	25.6227267	0.942190899	0.959	-0.0516	PIS58183.1	orf19.3220	Putative rRNA processing protein; Spider biofilm induced
PIS48306.1	31.57995412	32.32483277	30.7731982	31.26089222	31.63122743	31.62897447	0.926087638	0.958	-0.0523	PIS48306.1	CCT2	Chaperonin of the cytosolic TCP1 ring complex; protein present in exponential and stationary-phase yeast cells, but higher amounts in stationary phase; GlcNAc-induced protein
PIS51917.1	24.74718203	23.20736231	26.85739084	25.89395364	23.98473404	24.77102204	0.942474823	0.959	-0.0541	PIS51917.1	orf19.4182	Has domain(s) with predicted oxidoreductase activity

PIS55030.1	26.88815364	26.11818134	26.83041946	27.05720714	26.01616327	26.60059861	0.93428485	0.959	-0.0543	PIS55030.1	SRD1	Sur7 family protein with PalI-like domains; not required for stress responses; mutant is viable
PIS55460.1	25.29688538	26.2415757	25.19489006	25.02555518	25.97488864	25.56906321	0.944028753	0.959	-0.0546	PIS55460.1	orf19.6527	Pheromone-regulated protein (Prm10) of S. cerevisiae; colony morphology-related gene regulation by Ssn6; induced by Mnl1 under weak acid stress; possibly essential gene, disruptants not obtained by UAU1 method; Spider biofilm induced
PIS52340.1	31.06499211	30.81202853	31.04230791	30.64881708	31.09219013	31.01284632	0.922016797	0.958	-0.0552	PIS52340.1	RAX1	Protein with a predicted role in bud site selection; hypha-induced expression; Spider biofilm induced
PIS58269.1	25.86340051	26.20216831	25.75908126	26.04422615	25.48812473	26.12589535	0.927722186	0.959	-0.0555	PIS58269.1	orf19.4601	Putative RNA polymerase III transcription initiation factor complex (TFIIIC) subunit; possibly an essential gene, disruptants not obtained by UAU1 method
PIS51251.1	32.19762551	32.08900507	32.20666853	32.05552554	32.15890186	32.11183126	0.876672245	0.956	-0.0557	PIS51251.1	VAS1	Putative tRNA-Val synthetase; genes encoding ribosomal subunits, translation factors, and tRNA synthetases are downregulated upon phagocytosis by murine macrophage
PIS48724.1	27.50398371	23.89955222	26.12558516	27.48933537	24.94867065	24.92333939	0.950183017	0.959	-0.0559	PIS48724.1	UTP18	Putative U3 snoRNA-associated protein; Hap43-induced; repressed in core stress response; physically interacts with TAP-tagged Nop1
PIS51950.1	26.0047199	26.0036948	25.00434767	24.37782933	26.14954787	26.31578476	0.931854931	0.959	-0.0565	PIS51950.1	DJP1	Similar to bacterial DnaJ; reported to have a role in peroxisome biogenesis; induced in low iron and upon adherence to polystyrene
PIS54528.1	24.85491528	23.37054765	23.76079342	23.36640665	24.22128665	24.22914556	0.932109782	0.959	-0.0565	PIS54528.1	MSO1	Putative secretory protein involved in S. cerevisiae sporulation; repressed during pseudohyphal growth in the presence of lysed macrophages; Hap43-repressed; Spider biofilm induced
PIS56915.1	30.8892683	31.09739556	31.01366891	30.98162839	30.97027852	30.87585125	0.859028679	0.955	-0.0575	PIS56915.1	CCT7	Cytosolic chaperonin Cct ring complex; protein is present in exponential and stationary growth phase yeast cultures; sumoylation target
PIS56746.1	29.9493491	30.24325097	30.00274885	29.76914967	30.10609678	30.14746822	0.853475688	0.955	-0.0575	PIS56746.1	orf19.2057	Ortholog(s) have ATP binding, ATPase, metallopeptidase activity, role in protein-containing complex assembly, signal peptide processing and m-AAA complex, mitochondrial inner boundary membrane, mitochondrial inner membrane

PIS58694.1	31.50580553	31.65846952	30.97153554	31.33331063	31.4531582	31.17591826	0.923557968	0.958	-0.0578	PIS58694.1	RPS26A	Ribosomal protein; regulated by Nrg1, Tup1; repressed upon phagocytosis by murine macrophage; alternatively spliced intron in 5'-UTR; Spider biofilm repressed
PIS56793.1	29.87247761	29.7890705	29.94728059	29.69567638	29.87127945	29.86815833	0.869544304	0.956	-0.0579	PIS56793.1	RPT1	Putative 26S proteasome regulatory subunit 7; Hap43p-repressed gene; regulated by Gcn2p and Gcn4p; overlaps orf19.442
PIS58248.1	29.17904076	28.80520028	28.77908058	28.97270484	28.73068262	28.88623596	0.848588394	0.955	-0.0579	PIS58248.1	TIM54	Predicted component of the mitochondrial TIM22 complex; involved in protein import into mitochondrial inner membrane; Spider biofilm repressed
PIS52163.1	26.5036229	25.26177767	26.30092229	26.488976	26.51376934	24.88938413	0.9026611	0.957	-0.0581	PIS52163.1	orf19.3132	Predicted endoplasmic reticulum zinc transporter; induced by nitric oxide
PIS52278.1	25.89130049	26.77223771	25.61874806	24.95117745	26.4666026	26.68995011	0.915815705	0.958	-0.0582	PIS52278.1	ORC4	Phosphorylated protein similar to S. cerevisiae Orc4, subunit of the origin recognition complex (ORC); induced by alpha pheromone in SpiderM medium
PIS55818.1	32.24798714	31.90687239	32.34890126	32.14118095	32.09271274	32.09452633	0.882523401	0.956	-0.0584	PIS55818.1	SEC27	Ortholog(s) have ubiquitin binding activity
PIS52192.1	24.0342402	26.81164051	23.62527909	24.46943379	24.6780192	25.14586375	0.952222991	0.96	-0.0593	PIS52192.1	orf19.1394	Putative protein of unknown function; mutant is viable; protein level decreases in stationary phase cultures; Spider biofilm induced
PIS50403.1	29.33762555	29.02903337	29.16157625	29.24586425	28.90493019	29.19951088	0.890797624	0.957	-0.0593	PIS50403.1	RPA190	Putative RNA polymerase I subunit A190; Hap43p-induced gene; flucytosine induced
PIS52262.1	27.65854332	27.33933235	27.76970625	27.90567293	27.23191881	27.45188486	0.876734163	0.956	-0.0594	PIS52262.1	RRP6	Putative nuclear exosome exonuclease component; Hap43p-induced gene; mutation confers hypersensitivity to 5-fluorocytosine (5-FC), 5-fluorouracil (5-FU), and tubercidin (7-deazaadenosine)
PIS54911.1	29.38437057	29.55802079	29.44712409	29.47728056	29.40686835	29.32540396	0.870522223	0.956	-0.06	PIS54911.1	SNF1	Functional homolog of S. cerevisiae Snf1p, which regulates sugar metabolism; constitutively expressed; Thr208 phosphorylation may have regulatory role; up-regulation associated with azole resistance; essential gene in some experiments

PIS51838.1	24.86984023	25.47305171	24.87861466	25.0194862	24.93075851	25.09089424	0.869015228	0.956	-0.0601	PIS51838.1	orf19.90	Ortholog(s) have sterol transfer activity and endoplasmic reticulum-plasma membrane contact site, perinuclear endoplasmic reticulum membrane localization
PIS55821.1	27.29276654	28.38516261	26.22486204	26.99112389	26.88657719	27.8427588	0.914876038	0.958	-0.0608	PIS55821.1	orf19.1214	Ortholog(s) have metalloaminopeptidase activity and role in protein maturation
PIS58606.1	30.21990845	30.26724307	30.55359322	30.29343049	30.22657443	30.33785776	0.823447555	0.953	-0.061	PIS58606.1	orf19.2954	Protein of unknown function; Hap43-repressed gene; repressed by nitric oxide
PIS51666.1	30.62826105	30.47287164	31.05358593	30.76342608	30.50729541	30.70037164	0.864363172	0.956	-0.0612	PIS51666.1	YKT6	Putative protein of the vacuolar SNARE complex; predicted role in vacuolar fusion; rat catheter biofilm repressed
PIS58821.1	29.57061836	29.35188039	29.66276839	29.61041091	29.48453548	29.30578509	0.873016441	0.956	-0.0615	PIS58821.1	PRE5	Alpha6 subunit of the 20S proteasome; regulated by Gcn4; induced in response to amino acid starvation (3-AT); Spider biofilm repressed
PIS50388.1	27.01786156	26.79173796	26.9914287	26.8567763	26.69675436	27.06023851	0.904567834	0.957	-0.0624	PIS50388.1	orf19.5300	Calnexin; integral membrane ER chaperone involved in folding and quality control of glycoproteins; caspofungin induced
PIS49675.1	26.88124817	26.10402016	25.81253943	25.65630174	26.4520633	26.50212394	0.948413362	0.959	-0.0624	PIS49675.1	orf19.6665	Ortholog(s) have small GTPase binding, structural constituent of nuclear pore activity
PIS54569.1	30.61727278	31.54465591	29.95376047	29.84469566	30.96589385	31.1137805	0.90045583	0.957	-0.0638	PIS54569.1	EGD2	Nascent polypeptide associated complex protein alpha subunit; soluble protein in hyphae; macrophage/pseudohyphal-induced; protein level decrease in stationary phase cultures; GlcNAc-induced protein; Spider biofilm repressed
PIS56638.1	30.68214085	30.92284972	30.9366063	30.48521445	30.82553897	31.03810837	0.807537422	0.953	-0.0642	PIS56638.1	FRS1	Phenylalanyl-tRNA synthetase; possible role in early cell wall biosynthesis; downregulated by phagocytosis by macrophages; possibly essential gene, disruptants not obtained by UAU1 method; protein present in exponential and stationary phase
PIS54722.1	30.8143799	29.99185926	30.83349951	30.67432685	30.37908536	30.39024177	0.842586503	0.954	-0.0654	PIS54722.1	orf19.3859	Putative microsomal beta-keto-reductase; transcript upregulated by treatment with ciclopirox olamine; induced by alpha pheromone in SpiderM medium; regulated by Sef1, Sfu1, and Hap43

PIS51429.1	30.4997358	30.28915554	30.58325844	30.39175749	30.50965146	30.27027759	0.833220413	0.954	-0.0668	PIS51429.1	orf19.1086	Ortholog(s) have role in protein deubiquitination, protein retention in Golgi apparatus, regulation of ER to Golgi vesicle-mediated transport, regulation of retrograde vesicle-mediated transport, Golgi to ER, ribophagy
PIS48245.1	31.36364461	31.41868912	31.22480599	31.07912059	31.34604401	31.3807628	0.886387158	0.957	-0.0671	PIS48245.1	ARO10	Aromatic decarboxylase; Ehrlich fusel oil pathway of aromatic alcohol biosynthesis; alkaline repressed; protein abundance affected by URA3 expression in CAI-4 strain; Spider biofilm induced
PIS58763.1	27.72852386	27.79919371	27.50156631	27.70893331	28.01197956	27.10475361	0.93680434	0.959	-0.0679	PIS58763.1	NAN1	Putative U3 snoRNP protein; Hap43p-induced gene; physically interacts with TAP-tagged Nop1p
PIS50307.1	23.91291697	24.54107903	24.06478398	25.7590073	23.18221264	23.37224083	0.910804919	0.958	-0.0684	PIS50307.1	orf19.7645	Biotin protein ligase; catalyzes covalent attachment of biotin to biotin-dependent enzymes including acetyl CoA carboxylase and pyruvate carboxylase; transcription regulated by biotin availability and Vhr1p
PIS56543.1	25.48567024	25.54794668	26.12826616	25.37524186	25.84516389	25.73562621	0.885552452	0.957	-0.0686	PIS56543.1	WSC2	Putative cell wall integrity and stress response protein; mRNA binds She3; Spider biofilm induced
PIS49538.1	29.75792029	30.08981178	29.91925894	29.76521888	29.86325521	29.93234478	0.84281076	0.954	-0.0687	PIS49538.1	GLN1	Putative glutamate synthase; regulated by Tsa1, Tsa1B under H2O2 stress conditions; Spider biofilm induced
PIS51676.1	34.20048627	33.82922364	34.16555755	33.84701682	34.14094278	33.9995438	0.805965867	0.952	-0.0693	PIS51676.1	RPS18	Predicted ribosomal protein; repressed upon phagocytosis by murine macrophage; repressed by nitric oxide; Hap43-induced; Spider biofilm repressed
PIS49466.1	30.67769843	30.46439654	30.85781003	30.52681684	30.71797574	30.54675063	0.84462327	0.955	-0.0695	PIS49466.1	SEC21	Ortholog(s) have role in endoplasmic reticulum to Golgi vesicle-mediated transport, retrograde vesicle-mediated transport, Golgi to endoplasmic reticulum and COPI vesicle coat, endosome localization
PIS51446.1	27.72674817	27.93029735	28.12000809	27.62699955	27.89797733	28.04264032	0.863635829	0.956	-0.0698	PIS51446.1	ABG1	Vacuolar membrane protein; depletion causes abnormal vacuolar morphology, cell separation defect, sensitivity to cell wall stress, increased hyphal branching; essential, no mammalian homolog; Cyr1-regulated; rat catheter biofilm repressed
PIS52143.1	24.40531169	25.64751288	23.78086165	24.1772998	24.57568217	24.87057841	0.905168918	0.958	-0.07	PIS52143.1	ARG11	Putative ornithine transporter of the mitochondrial inner membrane; induced during the mating process

PIS56675.1	24.40531169	25.64751288	23.78086165	24.1772998	24.57568217	24.87057841	0.941634495	0.959	-0.07	PIS56675.1	orf19.4117	Ortholog of C. dubliniensis CD36 : Cd36_20450, C. parapsilosis CDC317 : CPAR2_104160, C. auris B8441 : B9J08_001214 and Candida tenuis NRRL Y-1498 : CANTEDRAFT_95516
PIS51865.1	24.75486831	24.17684209	24.47449519	23.99529485	24.80181839	24.3981146	0.945592851	0.959	-0.0703	PIS51865.1	CDC54	Putative pre-replication complex helicase subunit; transcript regulated by Nrg1 and Mig1; periodic mRNA expression, peak at cell-cycle M/G1 phase; Hap43-induced
PIS51320.1	29.62294514	29.05411678	29.53631987	29.35203716	29.41814728	29.23217197	0.934493241	0.959	-0.0703	PIS51320.1	TAF60	Putative TFIID and SAGA complex subunit; mutation confers hypersensitivity to amphotericin B
PIS49594.1	30.75921427	30.89734134	30.64695214	30.54414078	30.78705051	30.75921068	0.811332543	0.953	-0.071	PIS49594.1	CYS4	Cystathionine beta-synthase; sulfur amino acid biosynthesis; antigenic in mouse; flow model biofilm induced; alkaline induced; macrophage/pseudohyphal-induced; present in exponential and stationary growth phase yeast
PIS56669.1	31.55051849	31.82661067	31.63777205	31.92943208	31.54340868	31.32835237	0.822071679	0.953	-0.0712	PIS56669.1	FUM12	Putative fumarate hydratase; enzyme of citric acid cycle; fluconazole, Efg1 repressed; induced in high iron; protein present in exponential and stationary growth phase
PIS54831.1	29.50206422	29.94907351	29.20238736	29.48151833	29.54354999	29.41468837	0.881287151	0.956	-0.0713	PIS54831.1	ADE6	5-Phosphoribosylformyl glycinamidine synthetase; adenine biosynthesis; not induced in GCN response, in contrast to S. cerevisiae Ade6; protein in stationary phase yeast-form cultures; flow model and rat catheter biofilm repressed
PIS49542.1	29.44556578	30.04181014	29.19619127	28.72041197	29.90286298	29.842678	0.856096532	0.955	-0.0725	PIS49542.1	orf19.1849	Ortholog(s) have U3 snoRNA binding, rRNA binding activity and role in maturation of SSU-rRNA from tricistronic rRNA transcript (SSU-rRNA, 5.8S rRNA, LSU-rRNA), ribosomal small subunit biogenesis
PIS51488.1	29.69357335	29.88491149	29.24579563	29.50504793	29.6267914	29.47371601	0.877261798	0.956	-0.0729	PIS51488.1	orf19.5834	Ortholog(s) have nuclear import signal receptor activity, role in mRNA export from nucleus, protein import into nucleus and cytoplasm, nucleus localization
PIS51699.1	28.89162767	29.13664111	28.75480634	28.7012603	28.83907404	29.02339866	0.822655089	0.953	-0.0731	PIS51699.1	NMT1	Myristoyl-CoA:protein N-myristoyltransferase; attaches the fatty acid myristate to a small number of proteins at an N-terminal Gly; essential; antifungal drug target; functional homolog of S. cerevisiae Nmt1p
PIS49647.1	33.46450716	33.21136979	33.64132199	33.40215103	33.42994133	33.26546426	0.792687755	0.952	-0.0732	PIS49647.1	orf19.3061.1	Ortholog of S. cerevisiae Rps22Ap and Rps22Bp; gene contains 5' UTR intron

PIS58918.1	29.44465058	28.85308826	29.97326262	29.50098734	29.22186329	29.32834431	0.874232234	0.956	-0.0733	PIS58918.1	orf19.556	Protein of unknown function; regulated by Ssn6
PIS55724.1	24.22681902	24.32674534	23.00219411	23.82389495	24.46262967	23.04912738	0.908106622	0.958	-0.0734	PIS55724.1	orf19.2447	Putative Type II phosphatidylinositol 4-kinase; Ssr1-repressed; flow model biofilm repressed
PIS58227.1	25.01371044	23.57454623	24.83283304	23.83121441	24.84667878	24.52307524	0.969001203	0.96	-0.0734	PIS58227.1	orf19.7006	Ortholog(s) have role in reciprocal meiotic recombination
PIS56677.1	29.82295589	29.21037683	30.02361987	29.72462177	29.6364586	29.47503037	0.836144686	0.954	-0.0736	PIS56677.1	PMT4	Protein mannosyltransferase; required for WT cell wall composition and virulence in mice; roles in hyphal growth and drug sensitivity; one of 5 family members; Axl2 a substrate; Spider biofilm repressed; Bcr1-repressed in RPMI a/a biofilm
PIS54598.1	27.89041272	26.51659216	28.17056035	27.48075228	27.50519469	27.36933492	0.877712464	0.956	-0.0741	PIS54598.1	HOC1	Protein with similarity to mannosyltransferases; similar to S. cerevisiae Hoc1p and C. albicans Och1p
PIS54547.1	33.61372654	33.51315461	33.50118945	33.37816401	33.5270628	33.49934272	0.772905568	0.95	-0.0745	PIS54547.1	BMH1	Sole 14-3-3 protein in C. albicans; role in hyphal growth; possibly regulated by host interaction; localizes to yeast-form cell surface, not hyphae; alternatively spliced 5' UTR intron; Spider biofilm repressed
PIS48257.1	23.90245305	26.29249562	24.53960324	24.36307103	24.87120471	25.27557548	0.900680027	0.957	-0.0749	PIS48257.1	orf19.6024	Ortholog of C. dubliniensis CD36 : Cd36_00770, C. auris B8441 : B9J08_004942, Candida tenuis NRRL Y-1498 : CANTEDRAFT_112621 and Debaryomyces hansenii CBS767 : DEHA2D09812g
PIS58856.1	29.87852585	29.43814263	29.80953266	29.58270989	29.66386842	29.65308466	0.770506114	0.95	-0.0755	PIS58856.1	orf19.5038	Predicted tRNA (guanine) methyltransferase activity; Spider biofilm induced
PIS51774.1	23.65510358	24.62649672	23.45632781	24.04327992	23.80376159	23.6623804	0.863474133	0.956	-0.0762	PIS51774.1	orf19.1070	Ortholog(s) have role in phospholipid translocation, retrograde transport, endosome to Golgi and endosome membrane, trans-Golgi network membrane localization
PIS52053.1	30.91409158	30.51766719	31.34633267	31.04759549	30.76499283	30.73624458	0.880151483	0.956	-0.0764	PIS52053.1	POL5	Putative DNA Polymerase phi; F-12/CO2 early biofilm induced

PIS50606.1	34.50721156	34.44429609	34.39569686	34.27206043	34.49134486	34.35249108	0.783917677	0.951	-0.0771	PIS50606.1	RPL8B	Predicted ribosomal protein; regulated upon yeast-hypha switch; repressed upon phagocytosis by murine macrophage; Spider biofilm repressed
PIS52117.1	32.93481569	32.60573554	33.11003709	32.79245473	32.75629849	32.86970526	0.934047923	0.959	-0.0774	PIS52117.1	orf19.4520	Putative gluconokinase; rat catheter biofilm induced
PIS58999.1	30.69526859	30.97678531	30.35471786	30.19329424	30.83522596	30.76198351	0.79270413	0.952	-0.0788	PIS58999.1	GLC7	Putative catalytic subunit of type 1 serine/threonine protein phosphatase; regulated by Shp1; induced in high iron; alternatively spliced intron in 5' UTR
PIS48322.1	28.98324509	29.47932841	28.53890727	28.98594849	28.76101969	29.0177812	0.875117171	0.956	-0.0789	PIS48322.1	orf19.3559	S. cerevisiae ortholog Mrps35p is a structural constituent of ribosome and localizes to mitochondrial small ribosomal subunit; the snoRNA CD39 is encoded within the MRPS35 intron
PIS54851.1	25.75685184	26.10462177	25.33992662	24.55162246	26.75559735	25.65712804	0.921110889	0.958	-0.079	PIS54851.1	OPT2	Oligopeptide transporter; induced upon phagocytosis by macrophage; macrophage/pseudohyphal-repressed after 16h; fluconazole-induced; virulence-group-correlated expression; Hap43-repressed
PIS51399.1	23.41134394	23.27378403	23.80019129	23.59816097	23.56973154	23.08032653	0.919049721	0.958	-0.079	PIS51399.1	orf19.2387	Putative tRNA-Pro synthetase; genes encoding ribosomal subunits, translation factors, and tRNA synthetases are downregulated upon phagocytosis by murine macrophage
PIS58536.1	27.89168989	28.10636416	27.83376328	27.39641971	27.78934415	28.4087064	0.924385434	0.958	-0.0791	PIS58536.1	orf19.3695	Diacylglycerol cholinephosphotransferase and ethanolaminephosphotransferase, catalyzes the final step in Kennedy pathway of phosphatidylcholine and phosphatidylethanolamine biosynthesis
PIS51988.1	29.63752713	28.88493832	29.75293406	29.61166372	29.36219995	29.06384725	0.865921809	0.956	-0.0792	PIS51988.1	RAC1	G-protein of RAC subfamily; required for embedded filamentous growth, not for serum-induced hyphal growth; dynamic localization at plasma membrane and nucleus; similar to, but not interchangeable with, Cdc42p; lacks S. cerevisiae homolog
PIS52009.1	25.4058471	27.07080691	27.44184913	26.73680284	27.14736573	25.79588226	0.894780796	0.957	-0.0795	PIS52009.1	DIP2	Putative small ribonucleoprotein complex; Tn mutation affects filamentous growth; physically interacts with TAP-tagged Nop1; heterozygous null mutant exhibits resistance to parnafungin; Hap43-induced gene; Spider biofilm induced
PIS55433.1	30.37615114	30.17911583	30.2293806	30.46361869	29.93071994	30.15187018	0.766650182	0.95	-0.0795	PIS55433.1	VPS70	Ortholog of S. cerevisiae : VPS70, C. glabrata CBS138 : CAGL0M00616g, C. dubliniensis CD36 : Cd36_70380, C. parapsilosis CDC317 : CPAR2_300190 and Candida tenuis NRRL Y-1498 : CANTEDRAFT_108527

PIS54697.1	23.02049242	25.13049019	24.10312047	23.81936906	24.84602873	23.34955247	0.88221624	0.956	-0.0797	PIS54697.1	GZF3	GATA-type transcription factor; oxidative stress-induced via Cap1; mutant has abnormal colony morphology and altered sensitivity to fluconazole, LiCl, and copper; Spider biofilm induced
PIS51904.1	27.26618181	27.97777059	27.12655657	26.56589637	28.08173245	27.48258001	0.917292167	0.958	-0.0801	PIS51904.1	PRO3	Delta 1-pyrroline-5-carboxylate reductase; protein induced during the mating process; alkaline induced; stationary phase enriched protein; Spider biofilm repressed
PIS58970.1	32.0322162	32.11209909	31.927125	32.08010881	31.96384504	31.78682611	0.780685833	0.951	-0.0802	PIS58970.1	VPS1	Dynamin-family GTPase-related protein; induced upon adherence to polystyrene; regulated by Gcn2p and Gcn4p
PIS49791.1	29.61097859	29.97408736	29.64698472	29.71673721	29.59697168	29.67549181	0.825794994	0.954	-0.0809	PIS49791.1	orf19.3185	Ortholog(s) have acetyltransferase activator activity, peptide alpha-N-acetyltransferase activity, ribosome binding activity, role in N-terminal protein amino acid acetylation and NatA complex localization
PIS51244.1	31.63511848	32.23450583	30.74973727	30.97642936	31.57225714	31.82672908	0.838351224	0.954	-0.0813	PIS51244.1	KAR2	Similar to Hsp70 family chaperones; role in translocation of proteins into the ER; induced in high iron; protein present in exponential and stationary growth phase yeast cultures; flow model and Spider biofilm repressed
PIS56604.1	29.54696112	29.26071887	29.52117547	29.31087403	29.37693678	29.39680003	0.87150041	0.956	-0.0814	PIS56604.1	YCS4	Putative condensin complex subunit; cell-cycle regulated periodic mRNA expression
PIS48791.1	28.03700857	28.75615833	29.07434539	28.20541821	28.64551625	28.77165589	0.850498547	0.955	-0.0816	PIS48791.1	MAE1	Malic enzyme, mitochondrial; transcription regulated by Mig1, Tup1; colony morphology-related gene regulation by Ssn6; Hap43-repressed; Spider biofilm repressed
PIS48321.1	30.22252021	29.87452929	30.3987646	30.08554398	30.12230293	30.04313463	0.806960616	0.952	-0.0816	PIS48321.1	orf19.3558	Ortholog of S. cerevisiae : ERP3, C. glabrata CBS138 : CAGL0K01793g, C. dubliniensis CD36 : Cd36_19780, C. parapsilosis CDC317 : CPAR2_206420 and C. auris B8441 : B9J08_005011
PIS51287.1	26.37618731	26.25997892	26.0546918	26.42267205	25.89571208	26.12394715	0.854590595	0.955	-0.0828	PIS51287.1	orf19.5394.1	Ortholog(s) have role in mitochondrial cytochrome c oxidase assembly and mitochondrial inner membrane, mitochondrial intermembrane space localization
PIS58790.1	29.34544408	28.92041066	29.59020213	29.91980958	29.18767203	28.50000734	0.913344081	0.958	-0.0829	PIS58790.1	ERP5	Protein involved in ER to Golgi transport; rat catheter and Spider biofilm repressed

PIS55473.1	29.72094364	29.98980195	29.99208817	29.89898977	29.7104346	29.84430181	0.779299976	0.951	-0.083	PIS55473.1	CTA3	Protein similar to S. cerevisiae Ede1p, which is involved in endocytosis; activates transcription in 1-hybrid assay in S. cerevisiae
PIS48359.1	30.0614364	29.33935253	29.96055937	29.72926221	29.78584257	29.5969048	0.927927391	0.959	-0.0831	PIS48359.1	APL5	Ortholog of S. cerevisiae and S. pombe Apl5; subunit of the AP-3 adaptor complex involved in Golgi-to-vacuole transport; phosphorylated protein; mutant is viable;
PIS54539.1	23.96695713	25.0609886	24.27741548	25.02349839	23.91061507	24.11826432	0.884556711	0.957	-0.0843	PIS54539.1	VPS11	Protein involved in protein trafficking; putative role in vesicle-target membrane fusion; mutant lacks vacuole; role in hyphal growth, possibly via vacuole expansion into hypha; role in killing of and survival within macrophage
PIS55736.1	28.17599697	27.38579741	27.83235163	27.62582851	27.71420053	27.79760769	0.839553104	0.954	-0.0855	PIS55736.1	GCD1	Putative translation initiation factor; transcript regulated by Mig1; repressed upon phagocytosis by murine macrophage
PIS58696.1	30.73317246	30.77450723	30.97321266	30.85817494	30.72733752	30.63769217	0.820774632	0.953	-0.0859	PIS58696.1	orf19.1796	Putative glyoxylate reductase; acts on glyoxylate and hydroxypyruvate substrates; Spider biofilm repressed
PIS51415.1	26.77131012	26.24870472	26.58298764	26.27911882	26.7705848	26.295501	0.8664566	0.956	-0.0859	PIS51415.1	VPS2	Ortholog(s) have role in ATP export, intralumenal vesicle formation, late endosome to vacuole transport and protein retention in Golgi apparatus, more
PIS55542.1	27.94968581	27.61219799	28.30497257	28.1695268	27.8596301	27.5796346	0.916457038	0.958	-0.086	PIS55542.1	RFA1	Putative DNA replication factor A; RNA abundance regulated by cell cycle, tyrosol and cell density
PIS52162.1	24.76389704	23.26353963	24.87994642	24.28953549	24.3332518	24.02453004	0.827750254	0.954	-0.0867	PIS52162.1	orf19.3130	Ortholog of S. cerevisiae Gpn3 a GTPase with a role in biogenesis of RNA pol II and polIII; possibly an essential gene, disruptants not obtained by UAU1 method
PIS52374.1	25.11092359	23.72635095	24.90399365	25.46754676	24.03163962	23.98206679	0.860494544	0.955	-0.0867	PIS52374.1	orf19.719	Putative ubiquitin protein ligase with ubiquitin-protein transferase activity; null mutant is sensitive to proteotoxic stress and shows differential expression of other proteins likely involved in ER-associated protein catabolism
PIS48354.1	27.27433434	27.71238959	27.45756437	27.67545095	27.516934	26.99170336	0.839673664	0.954	-0.0867	PIS48354.1	YME1	Ortholog(s) have ATP-dependent peptidase activity

PIS55744.1	30.91461904	30.91368225	30.79470615	30.9042766	30.69349885	30.76463232	0.7393862	0.948	-0.0869	PIS55744.1	orf19.491	Endoplasmic reticulum (ER) protein-translocation complex subunit
PIS48459.1	26.51561716	24.17577063	25.65732575	25.60429219	25.41806327	25.06459471	0.883924324	0.957	-0.0873	PIS48459.1	RPL29	Ribosomal protein L29; induced upon germ tube formation; colony morphology-related gene regulation by Ssn6; intron in 5'-UTR; Spider biofilm repressed
PIS58024.1	29.63138223	29.22366183	29.81460924	29.58867706	29.47507194	29.34327983	0.89482774	0.957	-0.0875	PIS58024.1	ROM2	Putative GDP/GTP exchange factor; possibly an essential gene, disruptants not obtained by UAU1 method; Spider biofilm induced
PIS51186.1	26.76982929	26.89504321	26.58704181	27.03411088	26.55192774	26.40175573	0.901452995	0.957	-0.088	PIS51186.1	orf19.7589	Protein of unknown function; Hap43-repressed gene; transcript induced by elevated CO2
PIS54767.1	29.50647905	29.45490102	29.69799884	29.33381046	29.3384917	29.72215102	0.90105965	0.957	-0.0883	PIS54767.1	orf19.4357	Putative protein similar to S. cerevisiae Mgr3p, a subunit of the i-AAA protease supercomplex that degrades misfolded mitochondrial proteins
PIS49517.1	28.1186493	28.23668649	28.47923261	28.09261876	28.27494478	28.19987814	0.874893348	0.956	-0.089	PIS49517.1	CYR1	Class III adenylyl cyclase; mutant lacks cAMP; involved in regulation of filamentation, phenotypic switching and mating; mutant hyphal growth defect rescued by exogenous cAMP; downstream of Ras1p and CO2 signaling
PIS58668.1	27.97116321	27.35963624	28.20721816	27.77257778	27.83540126	27.66310884	0.834714651	0.954	-0.089	PIS58668.1	orf19.6423	Ortholog(s) have fructose-2,6-bisphosphate 2-phosphatase activity and role in glucose metabolic process
PIS56738.1	34.30167506	33.43512553	34.49259252	34.32006973	33.9114076	33.73090463	0.842832345	0.954	-0.089	PIS56738.1	RPL16A	Ribosomal protein; transposon mutation affects filamentous growth; repressed upon phagocytosis by murine macrophages; Hap43-induced; Spider biofilm repressed
PIS51517.1	33.52558297	33.05785615	33.52075472	33.16657729	33.48880095	33.18188923	0.773331737	0.95	-0.089	PIS51517.1	RPS10	Ribosomal protein S10; downregulated in the presence of human whole blood or PMNs; Spider biofilm repressed
PIS55732.1	28.42093391	28.5910055	28.54782733	27.86403422	28.54165264	28.88683781	0.883019107	0.956	-0.0891	PIS55732.1	orf19.477	Ortholog(s) have role in mitochondrial translation and mitochondrion localization

PIS55722.1	23.55845592	25.02731801	24.92867897	24.94783642	24.86980289	23.42821366	0.854310273	0.955	-0.0895	PIS55722.1	BUD16	Putative pyridoxal kinase; a key enzyme in pyridoxal 5'-phosphate synthesis, the active form of vitamin B6; involved in bud-site selection and genome integrity in S. cerevisiae; induced by alpha pheromone in SpiderM medium
PIS58702.1	29.93298456	29.21266674	30.05376718	29.78152039	29.53361644	29.61521825	0.796271214	0.952	-0.0897	PIS58702.1	orf19.1480	Putative succinate dehydrogenase; enzyme of citric acid cycle; repressed by nitric oxide; Efg1, Hap43 repressed
PIS56761.1	28.02179139	27.86333902	27.76924734	28.5162937	27.1955649	27.67255498	0.856118602	0.955	-0.09	PIS56761.1	ROT2	Alpha-glucosidase II, catalytic subunit, required for N-linked protein glycosylation and normal cell wall synthesis; alkaline downregulated
PIS49562.1	29.45302959	28.1511901	29.41990748	28.52217922	28.94928803	29.28238363	0.890537234	0.957	-0.0901	PIS49562.1	orf19.3615	Protein of unknown function; induced in core caspofungin response; expression upregulated in an ssr1 null mutant; induced by nitric oxide independent of Yhb1p
PIS55551.1	30.1557036	30.4413399	30.6352779	30.41133962	30.24455189	30.3060743	0.775348595	0.951	-0.0901	PIS55551.1	SEC23	Putative GTPase-activating protein; regulated upon yeast-hypha switch; Spider biofilm repressed
PIS50331.1	34.09165429	33.66899105	34.27716011	33.78039572	34.13162539	33.8547379	0.781884706	0.951	-0.0903	PIS50331.1	RPL14	Ribosomal protein L14; promoter bound directly by Tbf1p; Hap43p-induced gene
PIS56713.1	24.8760236	23.84553647	26.20387805	25.04591939	24.0576566	25.54515628	0.91174879	0.958	-0.0922	PIS56713.1	orf19.7672	Ortholog(s) have protein folding chaperone, protein-containing complex binding activity
PIS58754.1	27.53493957	27.39200593	27.32507548	27.32341783	27.11353512	27.53671869	0.924105354	0.958	-0.0928	PIS58754.1	orf19.841	Ortholog(s) have SNARE binding activity, role in Golgi vesicle docking, Golgi vesicle transport and Golgi membrane localization
PIS50589.1	26.96181219	26.41220867	26.22346218	25.78289162	26.48910231	27.04465852	0.9082811	0.958	-0.0936	PIS50589.1	orf19.4172	Ortholog of C. dubliniensis CD36 : Cd36_40750, C. parapsilosis CDC317 : CPAR2_402570, C. auris B8441 : B9J08_004417 and Candida tenuis NRRL Y-1498 : CANTEDRAFT_112981
PIS58434.1	28.25444156	28.03300577	28.54184395	28.34775348	28.13337592	28.06648936	0.786672679	0.951	-0.0939	PIS58434.1	CDC50	Predicted non-catalytic subunit of phospholipid flippase; involved in endocytosis, hyphal development, drug resistance; mutants show attenuated virulence in mouse model; induced by Mnl1p under weak acid stress

PIS54643.1	32.76631616	32.8404511	31.87156676	31.83278309	32.6587355	32.70507081	0.85516993	0.955	-0.0939	PIS54643.1	orf19.5943.1	Protein of unknown function; transcript upregulated in an RHE model of oral candidiasis; rat catheter and Spider biofilm repressed
PIS51781.1	26.26346401	27.19408293	26.19093303	26.18381186	26.74441513	26.4386886	0.854590708	0.955	-0.0939	PIS51781.1	VPS16	Protein similar to S. cerevisiae Vps16p, which is involved in protein-vacuolar targeting; likely to be essential for growth, based on insertional mutagenesis; downregulated in biofilm or in azole-resistant strain that overexpresses MDR1
PIS56585.1	30.53404489	30.89158151	30.1596165	30.09735081	30.51572137	30.68948667	0.758416445	0.95	-0.0942	PIS56585.1	ALA1	Alanyl-tRNA synthetase; translational regulation generates cytoplasmic and mitochondrial forms; Gcn4p-regulated; repressed by amino acid starvation (3-AT); translation-related genes downregulated upon phagocytosis by murine macrophages
PIS51030.1	32.23421292	32.07905879	31.87066313	31.75676987	32.09458871	32.04987604	0.776894286	0.951	-0.0942	PIS51030.1	PMM1	Phosphomannomutase; enzyme of O- and N-linked mannosylation; interconverts mannose-6-phosphate and mannose-l-phosphate; functional homolog of S. cerevisiae Sec53; antigenic in mice; Hap43-induced; flow model and Spider biofilm repressed
PIS50609.1	30.25719887	28.4391419	30.43051349	30.4850007	29.25869075	29.10021337	0.934000113	0.959	-0.0943	PIS50609.1	orf19.6007	Predicted fatty acid acyl transferase-related protein domain; repressed by prostaglandins
PIS48367.1	30.18329757	29.97299742	29.98332123	29.90632502	30.17472942	29.77502862	0.747512219	0.949	-0.0945	PIS48367.1	SES1	Seryl-tRNA synthetase; charges the tRNA that recognizes the CUG codon, which typically specifies Leu, but specifies Ser in C. albicans; complements S. cerevisiae ses1 mutant viability; soluble protein in hyphae; macrophage-regulated
PIS52356.1	27.8933633	27.11725421	26.63433607	26.82215997	27.71581338	26.82289058	0.835096521	0.954	-0.0947	PIS52356.1	orf19.4013	Putative protein of unknown function; Hap43p-repressed gene; S. cerevisiae ortholog YHR045W localizes to the endoplasmic reticulum
PIS48748.1	24.941767	27.15626806	25.8255145	26.37125979	25.7815396	25.48571468	0.9054104	0.958	-0.095	PIS48748.1	DOS2	Protein of unknown function; induced during planktonic growth
PIS51982.1	23.56331797	21.46078426	23.27479346	23.38250371	22.42829392	22.20078752	0.899255943	0.957	-0.0958	PIS51982.1	JAB1	Component of the COP9 signalosome (CSN) complex, provides catalytic activity for deneddylation that cleaves the ubiquitin-like protein tag Rub1p off the cullin (Cdc53p) molecule
PIS49486.1	31.1303088	30.93812718	31.68935225	31.08673019	31.02052642	31.36099679	0.747673379	0.949	-0.0965	PIS49486.1	orf19.7328	Protein with a Staphylococcal nuclease domain; transcript regulated by Mig1 and Tup1; flow model and Spider biofilm repressed

PIS52292.1	28.21158942	27.15559146	28.02020397	27.86034521	27.95172606	27.28413418	0.863130334	0.956	-0.0971	PIS52292.1	MPP10	Putative SSU processome and 90S preribosome component; repressed in core stress response; repressed by prostaglandins
PIS55489.1	30.85695923	30.28659303	30.79037234	30.84219452	30.49471837	30.30585926	0.771365194	0.95	-0.0971	PIS55489.1	orf19.4532	Protein of unknown function; present in exponential and stationary growth phase yeast cultures
PIS51478.1	26.90389787	26.31771921	27.65949103	26.63624196	27.28569433	26.66715698	0.924704704	0.958	-0.0973	PIS51478.1	orf19.4749	Protein of unknown function; hyphal-induced expression, regulated by Cyr1, Ras1, Efg1; Hap43-induced gene; Spider biofilm induced
PIS56655.1	27.76004436	27.19360639	27.40631203	27.08207493	27.68874526	27.29686172	0.77177241	0.95	-0.0974	PIS56655.1	orf19.1525	Ortholog(s) have role in nuclear-transcribed mRNA catabolic process, nonsense-mediated decay
PIS51527.1	27.76878392	27.5135501	27.38923504	26.55649696	27.83995501	27.9777291	0.875441353	0.956	-0.0991	PIS51527.1	SUI1	Putative translation initiation factor; flucytosine induced; genes encoding ribosomal subunits, translation factors, and tRNA synthetases are downregulated upon phagocytosis by murine macrophage
PIS58209.1	28.46590235	27.92691544	28.47014068	28.55311375	28.10640978	27.90385818	0.871881307	0.956	-0.0999	PIS58209.1	orf19.7488	Component of the SSU processome; predicted role in pre-18S rRNA processing; Spider biofilm induced
PIS51898.1	30.13917789	28.9802859	30.56290086	30.2211013	29.75131062	29.40997162	0.827683557	0.954	-0.1	PIS51898.1	PTC2	Protein phosphatase of the Type 2C-related family (serine/threonine-specific); with protein kinase Ssn3p controls hyphal elongation through regulation of phosphorylation of transcription factor Ume6p and its stability
PIS52121.1	28.71838833	28.88603108	29.0437235	28.61899012	28.84069659	28.88561995	0.745999572	0.949	-0.101	PIS52121.1	RIM11	Ortholog of S. cerevisiae Rim11; a protein kinase involved in meiosis and sporulation in S. cerevisiae; mutant is viable
PIS58700.1	30.34758895	29.9459321	30.39090317	30.08800707	30.07673421	30.21707015	0.731276827	0.948	-0.101	PIS58700.1	STT3	Putative oligosaccharyltransferase complex component; flow model and rat catheter biofilm repressed
PIS51627.1	30.65451713	30.0256434	30.86042893	30.56966383	30.41748892	30.24863896	0.861890362	0.955	-0.102	PIS51627.1	HMG1	HMG-CoA reductase; enzyme of sterol pathway; inhibited by lovastatin; gene not transcriptionally regulated in response to lovastatin and fluconazole

PIS52313.1	28.71831414	28.71927925	28.68565704	29.01055731	28.86952645	27.93333668	0.910927092	0.958	-0.103	PIS52313.1	CSI2	Putative 66S pre-ribosomal particle component; Hap43-induced; essential for growth; transposon mutation affects filamentous growth; Spider biofilm induced
PIS51786.1	25.88527589	25.45453384	25.43002498	25.4690639	25.55934247	25.43251889	0.818678949	0.953	-0.103	PIS51786.1	orf19.5201.1	Has domain(s) with predicted ATPase inhibitor activity, role in negative regulation of ATP-dependent activity and mitochondrion localization
PIS54782.1	27.95923993	27.18290695	28.0123892	28.02121558	27.34051258	27.48340987	0.869493007	0.956	-0.103	PIS54782.1	orf19.536	Ortholog(s) have chromatin binding, molecular adaptor activity, ubiquitin binding activity and role in chromatin organization, transcription by RNA polymerase II
PIS51831.1	30.57641336	30.36353864	30.46774935	30.44425262	30.27116821	30.38441718	0.93197146	0.959	-0.103	PIS51831.1	PRT1	Putative translation initiation factor eIF3; mutation confers hypersensitivity to roridin A, verrucarin A; genes encoding ribosomal subunits, translation factors, tRNA synthetases are downregulated upon phagocytosis by murine macrophages
PIS58449.1	23.45970832	24.52242351	24.3125517	23.7974265	24.10696546	24.07965377	0.799255405	0.952	-0.104	PIS58449.1	CWH8	Putative dolichyl pyrophosphate (Dol-P-P) phosphatase; ketoconazole-induced; expression is increased in a fluconazole-resistant isolate; clade-associated gene expression; Hap43p-induced gene
PIS55801.1	32.20915373	31.05191212	32.45136384	32.22186136	31.60344543	31.57536875	0.834008268	0.954	-0.104	PIS55801.1	MNT1	Alpha-1,2-mannosyl transferase; predicted type II Golgi membrane protein; adds 2nd mannose during cell-wall mannoprotein biosynthesis; required for wild-type virulence and adherence to epithelial cells; Hap43-induced
PIS54665.1	27.47348642	26.63516865	27.35360611	27.09133472	27.79772224	26.2608552	0.882064449	0.956	-0.104	PIS54665.1	orf19.6979	Ortholog(s) have role in intracellular magnesium ion homeostasis, intracellular manganese ion homeostasis, mitochondrion organization
PIS50389.1	24.41175901	23.58534572	24.63713446	24.43086313	24.14195827	23.74683341	0.86581435	0.956	-0.105	PIS50389.1	ECM1	Putative pre-ribosomal factor; decreased mRNA abundance observed in cyr1 homozygous mutant hyphae; induced by heavy metal (cadmium) stress; Hog1p regulated
PIS51288.1	28.62824907	28.64990145	28.64836775	28.37928264	28.71418461	28.51865367	0.753242871	0.949	-0.105	PIS51288.1	orf19.5393	Putative cysteine sulfinate decarboxylase; transcript positively regulated by Tbf1; Spider biofilm induced
PIS48331.1	28.13685746	28.57079372	28.18986272	28.11019841	28.2952482	28.17696771	0.820051464	0.953	-0.105	PIS48331.1	orf19.5442	Ortholog of C. dubliniensis CD36 : Cd36_80240, C. parapsilosis CDC317 : CPAR2_504040, C. auris B8441 : B9J08_005021 and Candida tenuis NRRL Y-1498 : CANTEDRAFT_130872

PIS51812.1	23.1972534	24.29594473	23.99404457	23.93300326	23.57528913	23.664045	0.814131207	0.953	-0.105	PIS51812.1	orf19.6821	Ortholog(s) have ubiquitin protein ligase activity
PIS55000.1	25.49643368	24.82086075	24.80827049	25.05930812	24.93893352	24.81233389	0.840829027	0.954	-0.105	PIS55000.1	SLN1	Histidine kinase involved in a two-component signaling pathway that regulates cell wall biosynthesis; mutants are sensitive to growth on H2O2 medium; rat catheter and Spider biofilm induced
PIS58458.1	28.03548393	27.28009884	28.35019905	28.33430306	27.39184026	27.62335668	0.912620905	0.958	-0.105	PIS58458.1	TIM21	Component of the Translocase of the Inner Mitochondrial membrane (TIM23 complex); for protein import into mitochondria; Hap43, ketoconazole-repressed; Spider biofilm repressed
PIS54854.1	29.95572723	29.84806687	30.16326723	29.84352216	29.59611441	30.20962995	0.698482406	0.945	-0.106	PIS54854.1	GTT12	Ortholog(s) have glutathione peroxidase activity, glutathione transferase activity, role in glutathione metabolic process, protein glutathionylation and endoplasmic reticulum localization
PIS58858.1	23.42609045	25.05748205	24.6348252	24.04615815	24.56981686	24.18274627	0.859735611	0.955	-0.107	PIS58858.1	orf19.1297	Ortholog(s) have phosphatidylinositol-3-phosphate binding activity and role in autophagy of mitochondrion, cytoplasm to vacuole transport by the Cvt pathway, early endosome to Golgi transport, macroautophagy
PIS51390.1	29.79884438	29.28776941	30.10946215	29.68959773	29.55179086	29.63284448	0.741845541	0.948	-0.107	PIS51390.1	orf19.3293	Putative enoyl reductase involved in very long chain fatty acid elongation; possibly an essential gene, disruptants not obtained by UAU1 method
PIS56957.1	29.08115984	28.88681203	28.67119768	28.76865717	28.75773806	28.79189857	0.679658551	0.944	-0.107	PIS56957.1	orf19.5370	Ortholog(s) have fungal-type vacuole membrane localization
PIS48381.1	30.16321271	29.48575933	30.11542429	30.13066195	29.55583254	29.75589463	0.781599093	0.951	-0.107	PIS48381.1	RSP5	Putative NEDD4 family E3 ubiquitin ligase; induced during infection of murine kidney, compared to growth in vitro; has murine homolog; possibly an essential gene, disruptants not obtained by UAU1 method
PIS49609.1	31.60410168	31.27304042	32.00295938	31.70963506	31.39862515	31.44921623	0.73894247	0.948	-0.108	PIS49609.1	AGC1	Putative mitochondrial carrier protein; transcript is alkaline upregulated rat catheter biofilm induced
PIS55552.1	27.62351039	27.89112127	28.00625957	27.67936876	27.96465654	27.55403239	0.883944358	0.957	-0.108	PIS55552.1	orf19.2835	Ortholog(s) have SUMO activating enzyme activity, role in mitotic chromosome condensation, protein sumoylation and SUMO activating enzyme complex, cytosol, nucleus localization

PIS58085.1	25.75515607	27.51689191	26.10544655	25.80475783	26.69191563	26.55562365	0.886833644	0.957	-0.108	PIS58085.1	orf19.6134	Ortholog(s) have role in ER-dependent peroxisome organization, retrograde vesicle-mediated transport, Golgi to endoplasmic reticulum, vesicle-mediated transport
PIS58082.1	26.23805752	26.51471375	26.43981971	25.98847157	25.94076924	26.93846372	0.854577894	0.955	-0.108	PIS58082.1	orf19.6136	Ortholog of S. cerevisiae Mrpl4, a mitochondrial ribosomal protein of the large subunit; repressed by nitric oxide
PIS58797.1	26.7337726	26.7119661	24.40462316	25.57039439	25.93412379	26.02069081	0.854194342	0.955	-0.108	PIS58797.1	RTG3	Transcription factor with bZIP motif involved in regulation of galactose catabolism genes; performs role analogous to Gal4p in S. cerevisiae; regulates carbon source-dependent stress response; regulates sphingolipid homeostasis
PIS52006.1	28.25420408	27.4613266	26.64857294	26.74287217	27.47380044	27.81938155	0.820628203	0.953	-0.109	PIS52006.1	NOT5	Protein with similarity to S. cerevisiae Not5p, a member of the transcription regulatory CCR4-NOT complex; required for hyphal growth; antigenic during human oral infection; greater expression in yeast-form cells than hyphae
PIS49627.1	30.6080559	29.64368185	30.86100131	30.51354326	30.18939939	30.08387368	0.949044198	0.959	-0.109	PIS49627.1	orf19.6693	Has domain(s) with predicted metal ion binding activity
PIS51788.1	26.21127104	26.59509619	22.75551284	23.74072976	25.72748559	25.76648144	0.906835666	0.958	-0.109	PIS51788.1	orf19.676	Microtubule-binding protein of the cortical microtubule; delays exit from mitosis when the spindle is abnormally oriented; Spider biofilm repressed
PIS54698.1	31.82532112	31.858109	31.58481862	31.2476769	31.79609677	31.89347541	0.731886184	0.948	-0.11	PIS54698.1	PGM2	Ortholog of S. cerevisiae Pgm2; induced in planktonic culture; Tye7p-regulated; flow model biofilm induced; rat catheter biofilm repressed
PIS54887.1	28.7660391	28.22637618	29.04698076	28.54212701	28.63879132	28.52581705	0.716327991	0.947	-0.111	PIS54887.1	AKR1	Ankyrin-repeat protein; induced by fluphenazine
PIS48344.1	23.59440872	23.58003176	25.3765554	25.2994356	23.29603149	23.62394816	0.888444678	0.957	-0.111	PIS48344.1	orf19.1240	Ortholog of S. cerevisiae : HOB2, C. glabrata CBS138 : CAGL0D04510g, C. dubliniensis CD36 : Cd36_45200, C. parapsilosis CDC317 : CPAR2_500480 and C. auris B8441 : B9J08_005034
PIS48653.1	24.13467326	24.16731338	23.80015667	23.94506622	23.81939483	24.00423741	0.887038786	0.957	-0.111	PIS48653.1	orf19.3289	Phosphorylated protein of unknown function

PIS51801.1	25.31757108	25.55859763	24.78112114	23.41339364	25.69522995	26.21484461	0.887480911	0.957	-0.111	PIS51801.1	orf19.3297	Ortholog(s) have structural constituent of ribosome activity and mitochondrial small ribosomal subunit localization
PIS50612.1	27.50501282	28.1348583	27.70887327	27.42460028	28.17061879	27.41981746	0.721000442	0.947	-0.111	PIS50612.1	orf19.6966	Ortholog(s) have choline kinase activity, ethanolamine kinase activity and role in phosphatidylcholine biosynthetic process, phosphatidylethanolamine biosynthetic process
PIS51632.1	26.14947078	25.88042784	25.98039072	26.09632792	25.86387233	25.7176126	0.808806969	0.953	-0.111	PIS51632.1	SKO1	bZIP transcription factor involved in cell wall damage response; represses the yeast-to-hypha transition; mutants are caspofungin sensitive; induced by osmotic stress via Hog1; activated by Rlm1p; induced by Mnl1 under weak acid stress
PIS54593.1	25.61070726	24.94712181	25.34197943	26.19593001	25.22048924	24.14723036	0.823575263	0.953	-0.112	PIS54593.1	HRT1	Ortholog of S. cerevisiae Hrt1; component of a nuclear ubiquitin-protein ligase complex involved in cell cycle control; induced by hydroxyurea; Spider biofilm induced
PIS49753.1	24.91351346	24.87800062	23.52127196	24.08089365	24.06661071	24.8298816	0.887639886	0.957	-0.112	PIS49753.1	orf19.4734	Protein of unknown function; Hap43-repressed
PIS58042.1	33.05169386	32.44277864	33.59097742	33.20576626	32.81495756	32.72809057	0.826286045	0.954	-0.112	PIS58042.1	SOD2	Mitochondrial Mn-containing superoxide dismutase; protection against oxidative stress; homotetramer active; N-terminal 34 amino acids removed on mitochondrial import; H2O2-induced via Cap1p; Hap43p-, alkaline-downregulated, farnesol-induced
PIS48542.1	25.06601985	25.27409268	23.38308675	24.08986736	24.01874851	25.27807282	0.885141138	0.957	-0.112	PIS48542.1	TOA2	Putative TFIIA small subunit; protein abundance decreased in CAI4 strain compared to the SC5314 strain, abundance not affected by reintegration of URA3 in CAI4; flucytosine induced; possibly an essential gene (UAU1 method)
PIS52016.1	29.89771112	30.66377434	29.13613359	29.55776785	29.59378301	30.20617933	0.799800896	0.952	-0.113	PIS52016.1	HSP21	Small heat shock protein; role in stress response and virulence; fluconazole-downregulated; induced in cyr1 or ras1 mutant; stationary phase enriched protein; detected in some, not all, biofilm extracts; Spider biofilm induced
PIS51466.1	30.67871625	30.30321033	30.71480539	30.58197262	30.45856902	30.31631769	0.776840756	0.951	-0.113	PIS51466.1	orf19.2002	Ortholog(s) have structural constituent of nuclear pore activity and role in nuclear pore organization, poly(A)+ mRNA export from nucleus, protein import into nucleus, ribosomal large subunit export from nucleus
PIS48824.1	26.46593914	25.63680625	25.85350612	26.49958084	25.20655447	25.91126609	0.894959127	0.957	-0.113	PIS48824.1	orf19.4816	Protein of unknown function; induced by nitric oxide

PIS56574.1	28.83372185	27.93065153	28.59811752	28.62615482	28.34726544	28.05096144	0.769342802	0.95	-0.113	PIS56574.1	orf19.6245	Protein of unknown function; regulated by osmotic stress via Hog1 and oxidative stress (Hog1- and Cap1-independent); induced by alpha pheromone in SpiderM medium; Spider biofilm induced
PIS48589.1	29.5162655	29.39680187	29.89585125	29.77811587	29.32503032	29.36694802	0.74049935	0.948	-0.113	PIS48589.1	VMA5	Putative vacuolar H(+)-ATPase; plasma membrane localized; rat catheter biofilm repressed
PIS52378.1	30.29600035	30.10941941	30.61319779	30.45865753	30.19333804	30.02566028	0.796688235	0.952	-0.114	PIS52378.1	HRT2	Protein described as having a role in Ty3 transposition; repressed in hyphae; stationary phase enriched protein; rat catheter and Spider biofilm repressed
PIS52409.1	23.52828019	24.08808661	22.66616615	23.1202001	23.33029429	23.48924615	0.83449055	0.954	-0.114	PIS52409.1	orf19.4882	TFIIE small subunit; involved in RNA polymerase II transcription initiation; Spider biofilm induced
PIS51411.1	30.95442619	31.75918982	29.0743218	29.76236096	30.43545226	31.24767238	0.918320883	0.958	-0.114	PIS51411.1	PRA1	Cell surface protein that sequesters zinc from host tissue; enriched at hyphal tips; released extracellularly; binds to host complement regulators; mediates leukocyte adhesion and migration; immunogenic in mouse; produced at ambient pH
PIS58715.1	23.32253581	24.65402232	24.51551988	23.7902605	24.30111813	24.05816734	0.798115129	0.952	-0.114	PIS58715.1	THG1	tRNA guanylyltransferase, with role in tRNA modification; catalyzes reverse (3'-5') nucleotide polymerization
PIS55545.1	23.23452475	23.96328276	23.67627374	24.12781197	24.07257538	22.32998281	0.879777255	0.956	-0.115	PIS55545.1	CDH1	Protein involved in regulation of mitosis; similar to S. cerevisiae Cdh1, which is an APC/C component; transcriptionally induced by Mnl1 under weak acid stress
PIS48784.1	29.88438914	30.05775837	29.08796702	29.52579779	29.46691688	29.69200122	0.874579714	0.956	-0.115	PIS48784.1	orf19.511	Ortholog(s) have ribosylnicotinamide kinase activity and role in NAD biosynthesis via nicotinamide riboside salvage pathway, NAD biosynthetic process, nicotinamide riboside metabolic process
PIS52051.1	31.69342187	31.64219281	31.86338124	31.79693339	31.55763742	31.49869928	0.782493692	0.951	-0.115	PIS52051.1	orf19.5773	Putative dipeptidyl-peptidase III; protein detected by mass spec in exponential and stationary phase cultures; Hog1p-induced; clade-associated gene expression
PIS58397.1	26.72362022	27.24245541	26.20280735	26.95153725	26.37677476	26.49412844	0.875263024	0.956	-0.115	PIS58397.1	orf19.7108	D-ribulose-5-phosphate 3-epimerase; stationary phase enriched protein

PIS54553.1	27.51671934	27.75951858	26.83663235	27.48220576	27.12821963	27.15361638	0.849765193	0.955	-0.116	PIS54553.1	orf19.2966	Predicted dienelactone hydrolase domain; clade-associated gene expression; farnesol-downregulated; rat catheter biofilm repressed
PIS58456.1	28.26936372	28.24821107	28.11880222	28.19539756	28.0426922	28.04959823	0.713575334	0.946	-0.116	PIS58456.1	orf19.3689	Putative protein similar to 6-phosphofructo-2-kinase/fructose-2,6-bisphosphatase; expression downregulated in an ssr1 null mutant
PIS54550.1	24.62376236	24.63508569	24.55327301	24.75062459	24.5001473	24.21350535	0.754334183	0.949	-0.116	PIS54550.1	PHM5	Endo/exopolyphosphatase, hydrolyzes inorganic polyphosphate (poly P) into Pi; involved in polyphosphate catabolic process; redundant with exopolyphosphatase Ppx1p
PIS51094.1	28.35631582	27.93541146	28.30314577	27.94609826	28.36128392	27.93249001	0.885837343	0.957	-0.118	PIS51094.1	AGP2	Amino acid permease; hyphal repressed; white-opaque switch regulated; induced in core caspofungin response, during cell wall regeneration, by flucytosine; regulated by Sef1, Sfu1, and Hap43; rat catheter and Spider biofilm induced
PIS58724.1	31.68733664	31.51655839	31.66863611	31.38071594	31.51892143	31.62025445	0.857384563	0.955	-0.118	PIS58724.1	CYT1	Cytochrome c1; induced in high iron; alkaline repressed; possibly an essential gene, disruptants not obtained by UAU1 method; Hap43-repressed; Spider biofilm repressed
PIS49563.1	30.33448186	30.2455704	30.69614399	30.38476649	30.30558968	30.23313046	0.717348827	0.947	-0.118	PIS49563.1	ERG9	Putative farnesyl-diphosphate farnesyl transferase (squalene synthase); sterol biosynthesis pathway; likely essential for growth; regulated by fluconazole, lovastatin; amphotericin B, caspofungin repressed; Spider biofilm repressed
PIS55469.1	27.71874526	28.53896611	27.96520599	27.22002872	28.37889356	28.2685057	0.772755365	0.95	-0.118	PIS55469.1	orf19.5279	Ortholog(s) have structural constituent of ribosome activity, role in cell redox homeostasis, mitochondrial genome maintenance and mitochondrial large ribosomal subunit, mitochondrion localization
PIS58238.1	27.47414077	26.98240557	27.5400616	27.3591327	27.59305305	26.68800724	0.711075779	0.946	-0.119	PIS58238.1	SCH9	Protein kinase; involved in growth control, ribosomal protein synthesis, cell size, resistance to rapamycin,, chlamydospore formation, filamentous growth, and virulence; prevents hyphal growth in hypoxia at high CO2
PIS56639.1	30.46352805	30.13817725	30.85337099	30.66248563	30.19906676	30.23586254	0.714793956	0.947	-0.119	PIS56639.1	SEC4	Small GTPase of Rab family; role in post-Golgi secretion; possible C-terminal palmitoylation; downregulated on adherence to polystyrene; localizes to the Spitzenkorper during hyphal growth; functional homolog of S. cerevisiae Sec4p
PIS51341.1	32.7998422	32.62760536	32.80972397	32.46199251	32.61033875	32.80435433	0.64423451	0.941	-0.12	PIS51341.1	MLS1	Malate synthase; glyoxylate cycle enzyme; no mammalian homolog; regulated upon white-opaque switch; phagocytosis, strong oxidative stress induced; stationary phase enriched; flow model biofilm repressed; rat catheter, Spider biofilm induced

PIS58848.1	25.58846018	27.49440915	25.94748231	25.17577339	26.59944329	26.89440253	0.841727372	0.954	-0.12	PIS58848.1	orf19.789.1	Ortholog(s) have 7S RNA binding activity, role in SRP-dependent cotranslational protein targeting to membrane, translocation and signal recognition particle, endoplasmic reticulum targeting localization
PIS55513.1	29.05540871	30.03571716	29.1342354	29.50228557	28.9573384	29.40216797	0.856674975	0.955	-0.121	PIS55513.1	PHO100	Putative inducible acid phosphatase; DTT-extractable and observed in culture supernatant in low-phosphate conditions; slight effect on murine virulence; virulence-group-correlated expression; N-glycosylated; F-12/CO2 early biofilm induced
PIS55569.1	24.82696814	25.47939095	24.80428258	25.04986087	24.78594095	24.90777583	0.78761092	0.951	-0.122	PIS55569.1	GAP2	General broad specificity amino acid permease; ketoconazole, flucytosine repressed; Ssy1-dependent histidine induction; regulated by Nrg1, Tup1; colony morphology-related gene regulation by Ssn6; Spider and flow model biofilm induced
PIS51260.1	30.18320496	29.69904077	30.22202734	29.91218389	29.89536851	29.93049252	0.689828215	0.945	-0.122	PIS51260.1	orf19.5669	Protein with B-cell receptor-associated protein 31-like domain; membrane-localized protein
PIS51264.1	23.6204124	24.72085537	24.52797327	23.65098733	24.68682376	24.16451106	0.747365592	0.949	-0.122	PIS51264.1	RCH1	Plasma membrane protein; involved in regulation of cytosolic calcium homeostasis; null mutation confers sensitivity to calcium and resistance to azoles and terbinafine; rat catheter biofilm induced
PIS48516.1	26.48246188	26.78649324	26.30936148	26.45401456	26.59672107	26.15500447	0.664171006	0.943	-0.124	PIS48516.1	ARG8	Putative acetylornithine aminotransferase; Gcn2, Gcn4 regulated; rat catheter biofilm induced; Spider biofilm induced
PIS51804.1	23.91712281	24.10555544	22.36297387	24.055972	22.67475266	23.28284161	0.852236054	0.955	-0.124	PIS51804.1	orf19.3301	Putative ubiquitin ligase complex component; induced by heavy metal (cadmium) stress; Hog1-induced; transcript induced by Mnl1p under weak acid stress; flow model biofilm induced; Spider biofilm induced
PIS50524.1	34.66301416	34.44391728	34.98985577	34.66110025	34.49727858	34.56568068	0.643939576	0.941	-0.124	PIS50524.1	SAH1	S-adenosyl-L-homocysteine hydrolase; sulfur amino acid metabolism; antigenic in human; alkaline-, fluconazole-induced expression; Gcn4-regulated; amino acid starvation (3-AT) repressed; flow model biofilm induced; Spider biofilm repressed
PIS55591.1	29.78686999	29.7857441	30.01500683	29.67123855	30.01830181	29.52540436	0.763710523	0.95	-0.124	PIS55591.1	TPS1	Trehalose-6-phosphate synthase; role in hyphal growth and virulence in mouse systemic infection; induced in presence of human neutrophils; macrophage/pseudohyphal-repressed after 16h; stationary phase enriched protein; Hap43-repressed
PIS58207.1	27.55162612	28.68405806	27.55888581	27.3033535	28.03092134	28.08220952	0.763505472	0.95	-0.126	PIS58207.1	orf19.7485	Putative mitochondrial ribosomal protein of the large subunit; Hap43p-repressed gene; ortholog of S. cerevisiae MRPL9

PIS51491.1	30.50126613	30.49075075	30.19457375	30.27356306	30.39556725	30.13672037	0.75254571	0.949	-0.127	PIS51491.1	LHS1	Protein similar to S. cerevisiae Hsp70p; predicted Kex2p substrate; possibly essential, disruptants not obtained by UAU1 method; flow model biofilm repressed
PIS52343.1	27.11668297	27.13747851	26.83929621	26.08349299	26.87380924	27.75618635	0.810262495	0.953	-0.127	PIS52343.1	MEA1	Protein similar to A. nidulans MesA, which is involved in localization of actin cables; Hap43p-induced gene
PIS49779.1	27.1014767	27.66172718	26.8664658	27.39965571	26.77443804	27.07329194	0.884884492	0.957	-0.127	PIS49779.1	TFC4	Putative RNA polymerase III transcription initiation factor complex (TFIIIC) subunit; induced by Mnl1 under weak acid stress; possibly essential gene, disruptants not obtained by UAU1 method
PIS52145.1	30.73645029	31.62974738	30.3886746	30.57270717	30.95913339	30.83818234	0.828747713	0.954	-0.128	PIS52145.1	RPP2B	Conserved acidic ribosomal protein; possibly involved in regulation of translation elongation; interacts with Rpp1A; 1 of 4 similar C. albicans proteins (Rpp1A, Rpp1B, Rpp2A, Rpp2B); macrophage/pseudohyphal-induced; Spider biofilm
PIS49578.1	24.40255619	25.11893589	24.66316372	24.43155304	24.69664986	24.67031921	0.860632877	0.955	-0.129	PIS49578.1	OPT1	Oligopeptide transporter; transports 3-to-5-residue peptides; alleles are distinct, one has intron; suppresses S. cerevisiae ptr2-2 mutant defects; induced by BSA or peptides; Stp3p, Hog1p regulated; flow model biofilm induced
PIS55781.1	27.40831621	28.64731424	27.8956553	27.47200387	27.96119762	28.12835419	0.744569441	0.949	-0.13	PIS55781.1	HEM1	Putative 5-aminolevulinate synthase; caspofungin repressed; induced by high iron, nitric oxide; regulated by Ssn6; Hap43-repressed; Spider biofilm induced
PIS54527.1	29.4905018	29.27474534	28.71470098	28.89993256	29.35423096	28.83683258	0.691868671	0.945	-0.13	PIS54527.1	MSS116	Putative DEAD-box protein; required for efficient splicing of mitochondrial Group I and II introns; Hap43-induced; rat catheter biofilm induced
PIS52240.1	26.59553467	27.2306734	26.82120361	26.41653561	27.03354006	26.80672094	0.792874215	0.952	-0.13	PIS52240.1	orf19.1444	Epsin, involved in endocytosis; mutants are defective in hyphal growth and virulence
PIS58574.1	36.14781939	35.92354874	36.55368792	36.00238086	36.15359715	36.07701301	0.639465442	0.94	-0.131	PIS58574.1	ATP1	ATP synthase alpha subunit; antigenic in human/mouse; at hyphal surface; ciclopirox, ketoconazole, flucytosine induced; Efg1, caspofungin repressed; may be essential; sumoylation target; stationary phase-enriched; Spider biofilm repressed
PIS51727.1	27.72907646	27.53233553	27.68663072	28.01993155	27.51522052	27.02026223	0.873629521	0.956	-0.131	PIS51727.1	orf19.2045	Ortholog(s) have CTP-dependent diacylglycerol kinase activity and role in mitotic nuclear membrane biogenesis, phosphatidic acid biosynthetic process, regulation of isopentenyl diphosphate biosynthetic process, mevalonate pathway

PIS55608.1	27.91844604	28.32774636	27.74335919	27.91539972	27.77554228	27.90522259	0.720784606	0.947	-0.131	PIS55608.1	orf19.5693	Subunit of the GPI (glycosylphosphatidylinositol):protein transamidase complex; removes the GPI-anchor signal and attaches GPI to proteins in the ER; Spider biofilm repressed
PIS54607.1	25.32059877	24.52478455	24.26192713	24.93722175	24.18314653	24.59291032	0.840668539	0.954	-0.131	PIS54607.1	orf19.7443	Ortholog of C. dubliniensis CD36 : Cd36_86510, C. parapsilosis CDC317 : CPAR2_206110, C. auris B8441 : B9J08_002384 and Candida tenuis NRRL Y-1498 : CANTEDRAFT_112184
PIS52037.1	23.58572684	23.75795926	23.43086964	23.48362079	23.459823	23.43772341	0.919493353	0.958	-0.131	PIS52037.1	SAC3	Putative nuclear pore-associated protein, required for small ribosomal subunit biogenesis; possibly an essential gene, disruptants not obtained by UAU1 method
PIS51775.1	30.71435773	30.49903019	30.64966266	30.24210141	30.56850364	30.65352969	0.730175903	0.948	-0.133	PIS51775.1	ICL1	Isocitrate lyase; glyoxylate cycle enzyme; required for virulence in mice; induced upon phagocytosis by macrophage; farnesol regulated; Pex5-dependent peroxisomal localization; stationary phase enriched; rat catheter, Spider biofilm induced
PIS51502.1	25.75449668	24.75981627	24.85678331	25.38854066	25.1019114	24.48235381	0.896990025	0.957	-0.133	PIS51502.1	KCS1	Predicted inositol polyphosphate kinase; Spider biofilm induced
PIS55665.1	29.60672282	29.39226399	29.27470322	29.12809977	29.47953581	29.26717879	0.701080273	0.946	-0.133	PIS55665.1	orf19.1578	Ortholog of S. cereviaie Rrp5, an RNA binding protein involved in synthesis of 18S and 5.8S rRNAs; Hap43-induced gene
PIS48503.1	23.93360172	23.29225334	24.74159392	24.34290194	24.09483888	23.13096055	0.783882356	0.951	-0.133	PIS48503.1	orf19.6325.1	Ortholog of S. cerevisiae : MRX7, C. glabrata CBS138 : CAGL0K04785g, C. dubliniensis CD36 : Cd36_19820, C. parapsilosis CDC317 : CPAR2_603375 and C. auris B8441 : B9J08_005197
PIS58853.1	26.25393694	26.97004917	25.79998118	26.34308151	26.26786208	26.01143758	0.831747727	0.954	-0.134	PIS58853.1	orf19.5041	Ortholog of C. dubliniensis CD36 : Cd36_43600, C. parapsilosis CDC317 : CPAR2_403780, Candida tenuis NRRL Y-1498 : CANTEDRAFT_93767 and Debaryomyces hansenii CBS767 : DEHA2G16984g
PIS54995.1	28.15195747	26.90991462	28.19349132	27.81023701	27.40169029	27.64223525	0.800586543	0.952	-0.134	PIS54995.1	orf19.6477	Ortholog(s) have enzyme activator activity, role in tRNA (guanine-N7)-methylation and cytosol, nucleus, tRNA (m7G46) methyltransferase complex localization
PIS48271.1	33.98127112	34.1205018	34.07918539	33.55109738	34.14627944	34.08168287	0.645650172	0.941	-0.134	PIS48271.1	RPS1	Putative ribosomal protein 10 of the 40S subunit; elicits host antibody response during infection; transcript induced during active growth; Spider biofilm repressed

PIS55626.1	26.52339971	24.44756222	25.72328023	25.74073699	24.2668191	26.28390903	0.865129259	0.956	-0.134	PIS55626.1	SEN1	Putative helicase; repressed by prostaglandins
PIS51495.1	23.79808718	25.70493758	25.01127878	24.87426657	25.71354865	23.52265991	0.859284298	0.955	-0.135	PIS51495.1	orf19.5837	Ortholog(s) have 4-amino-4-deoxychorismate lyase activity and role in folic acid biosynthetic process
PIS48619.1	26.82220435	28.0231219	26.09050658	27.79259959	26.10612062	26.6315846	0.892302418	0.957	-0.135	PIS48619.1	orf19.7499	Putative nicotinic acid mononucleotide adenylyltransferase, involved in NAD salvage pathway; Spider biofilm repressed
PIS58322.1	33.16156598	32.7798678	33.32420244	32.94692171	32.86644349	33.04369932	0.762727232	0.95	-0.136	PIS58322.1	YNK1	Nucleoside diphosphate kinase (NDP kinase); homo-hexameric; soluble protein in hyphae; flucytosine induced; biofilm induced; macrophage-induced protein; stationary phase enriched protein; Spider biofilm repressed
PIS52209.1	28.12517435	27.19238104	28.06089252	28.06182983	27.55618733	27.35012244	0.761763791	0.95	-0.137	PIS52209.1	HRK1	Putative serine/threonine kinase; predicted role in cellular ion homeostasis; Spider biofilm repressed
PIS48364.1	25.25772291	26.30788652	25.41110291	25.08423967	25.34768653	26.13277594	0.797765288	0.952	-0.137	PIS48364.1	SLD1	Sphingolipid delta-8 desaturase; catalyzes desaturation at C8 in the long-chain base moiety of ceramides in glucosylceramide synthesis, important for virulence; ketoconazole and hypoxia induced; Hap43-repressed; Spider biofilm induced
PIS48430.1	32.71336481	32.93935989	32.87412405	32.66583213	32.71268079	32.73100829	0.652203353	0.942	-0.139	PIS48430.1	MIS11	Predicted mitochondrial C1-tetrahydrofolate synthase precursor; putative protein of glycine catabolism; repressed by Efg1; fluconazole-induced; stationary phase enriched protein; rat catheter and Spider biofilm repressed
PIS48414.1	30.09138743	29.82829783	30.30353413	30.15680333	29.90965189	29.73878285	0.721813447	0.947	-0.139	PIS48414.1	NOC2	Putative nucleolar complex protein; Hap43-induced; transposon mutation affects filamentous growth; mutation confers hypersensitivity to 5-fluorouracil (5-FU), tubercidin (7-deazaadenosine); repressed in core stress response
PIS51573.1	31.85958422	31.41054528	32.01723602	31.49212212	31.54346115	31.83176075	0.676816037	0.944	-0.14	PIS51573.1	orf19.6066	Hexadecenal dehydrogenase; involved in the conversion of sphingosine 1-phosphate breakdown product hexadecenal to hexadecenoic acid; Spider biofilm induced
PIS49798.1	29.79073827	30.05972937	29.6519391	29.45556088	29.7963092	29.82754119	0.601360269	0.937	-0.141	PIS49798.1	orf19.1395	Ortholog(s) have copper ion transmembrane transporter activity, inorganic phosphate transmembrane transporter activity

PIS51870.1	29.51553404	29.28897476	29.63571741	29.33738507	29.30899081	29.37036608	0.783578667	0.951	-0.141	PIS51870.1	PEP1	Type I transmembrane sorting receptor for multiple vacuolar hydrolases; cycles between late-Golgi and prevacuolar endosome-like compartments; rat catheter biofilm repressed
PIS48445.1	33.65241393	33.38995273	34.16863291	33.80413522	33.42603097	33.5581321	0.778036656	0.951	-0.141	PIS48445.1	POR1	Mitochondrial outer membrane porin; in detergent-resistant membrane fraction (possible lipid raft component); antigenic in human, mouse; Hap43p-induced; flucytosine-, macrophage-, farnesol-induced; fluconazole, caspofungin repressed
PIS51189.1	32.95806671	33.03538746	32.99720209	32.74428558	32.78973758	33.03171041	0.783858907	0.951	-0.142	PIS51189.1	FAA4	Acyl CoA synthase involved in uptake of long-chain fatty acids and biofilm formation
PIS56541.1	27.44805567	27.22688798	26.51667807	26.43874969	27.27518246	27.05305783	0.75652539	0.949	-0.142	PIS56541.1	orf19.5541	Protein with similarity to S. pombe Nrd1p; transcription induced upon induction of hyphal growth; regulated by Cph1p, Efg1p, Cph2p; low-level expression; alkaline upregulated; fungal-specific (no human or murine homolog)
PIS54529.1	28.22545502	28.33109043	29.18508163	28.68476605	28.17072027	28.45838712	0.686302536	0.944	-0.143	PIS54529.1	ARG5%2C6	activity; Gcn4 regulated; alkaline repressed; Spider biofilm induced
PIS58310.1	25.16603758	25.89194951	26.47217672	26.40669634	25.65849224	25.03543236	0.837911182	0.954	-0.143	PIS58310.1	orf19.3874	Predicted cation transmembrane transporter; Spider biofilm induced
PIS55537.1	28.17302733	27.64294749	28.57424241	28.58675481	27.95782308	27.41732649	0.81563264	0.953	-0.143	PIS55537.1	orf19.5522	Ortholog of C. dubliniensis CD36 : Cd36_62760, C. parapsilosis CDC317 : CPAR2_601700, C. auris B8441 : B9J08_001639 and Candida tenuis NRRL Y-1498 : CANTEDRAFT_115220
PIS51610.1	29.37756908	29.06678022	29.40801262	29.37509569	28.99467784	29.04922682	0.765165659	0.95	-0.144	PIS51610.1	LYS1	Saccharopine dehydrogenase (biosynthetic); enzyme of alpha-aminoadipate lysine biosynthesis pathway; functionally complements S. cerevisiae lys1 mutation; fungal-specific (no human or murine homolog)
PIS58225.1	30.20529278	29.59551393	30.3534298	29.5370747	30.04636874	30.13788376	0.67684713	0.944	-0.144	PIS58225.1	orf19.6898.1	Ortholog of C. dubliniensis CD36 : Cd36_71020, C. parapsilosis CDC317 : CPAR2_300360, C. auris B8441 : B9J08_000715 and Candida tenuis NRRL Y-1498 : CANTEDRAFT_105022
PIS58922.1	29.47507706	30.02014988	30.06167603	29.55378237	29.99774042	29.57240274	0.665625807	0.943	-0.144	PIS58922.1	TRP2	Putative anthranilate synthase with a predicted role in tryptophan biosynthesis; regulated by Gcn2p and Gcn4p

PIS51490.1	26.42424394	27.49027037	27.48579338	27.03773645	27.07954602	26.84744294	0.719506125	0.947	-0.145	PIS51490.1	HPT1	Putative hypoxanthine-guanine phosphoribosyltransferase; protein abundance affected by URA3 expression in the CAI4 strain background; protein level decreases in stationary phase; Spider biofilm induced
PIS54572.1	25.93036698	25.25377179	25.41973058	25.6617129	25.35886033	25.14886458	0.868197978	0.956	-0.145	PIS54572.1	orf19.7197	Putative intranuclear transport and DNA replication mediator; heterozygous null mutant exhibits resistance to parnafungin in the C. albicans fitness test; Spider biofilm induced
PIS49756.1	32.41849414	32.33655064	32.52797607	32.54814269	32.25629224	32.04148204	0.59868732	0.936	-0.146	PIS49756.1	orf19.2892	Ortholog of C. dubliniensis CD36 : Cd36_45910, C. parapsilosis CDC317 : CPAR2_501110, C. auris B8441 : B9J08_004783 and Candida tenuis NRRL Y-1498 : CANTEDRAFT_119393
PIS48279.1	29.72506742	29.41040463	29.49883743	29.19646142	29.41179683	29.58929226	0.708885208	0.946	-0.146	PIS48279.1	orf19.5799	Ortholog of S. cerevisiae : TPH3, C. dubliniensis CD36 : Cd36_17790, C. parapsilosis CDC317 : CPAR2_212150, C. auris B8441 : B9J08_004964 and Candida tenuis NRRL Y-1498 : CANTEDRAFT_128815
PIS58869.1	25.99021343	23.98894264	25.20086808	25.31802963	24.77568825	24.64506048	0.863601998	0.956	-0.147	PIS58869.1	HEM14	Putative protoporphyrinogen oxidase; involved in heme biosynthesis; predicted Kex2p substrate; iron regulated transcript; Yfh1-induced; Hap43-repressed; rat catheter biofilm repressed
PIS58609.1	30.24008259	31.53822981	30.4717622	29.80073443	30.91933459	31.08876097	0.795948539	0.952	-0.147	PIS58609.1	HOM6	Putative homoserine dehydrogenase; Gcn4-regulated; induced by amino acid starvation (3-ATtreatment); macrophage-induced protein; protein level decreases in stationary phase cultures; flow model biofilm repressed
PIS48795.1	31.26144117	30.8946012	31.38376985	31.17471092	31.03199622	30.8914352	0.693179024	0.945	-0.147	PIS48795.1	YHM1	Putative mitochondrial carrier protein; fungal-specific (no human or murine homolog); Hap43p-repressed gene
PIS56919.1	29.34950928	28.4690246	29.37067415	29.09635899	29.04861867	28.59961562	0.76773408	0.95	-0.148	PIS56919.1	DPP1	Putative diacylglycerol pyrophosphate phosphatase of diacylglycerol production for phospholipid biosynthesis; downregulation correlates with clinical development of fluconazole resistance
PIS56909.1	27.40326907	26.77845352	27.2057175	27.55362598	26.2371699	27.15211079	0.87619663	0.956	-0.148	PIS56909.1	PIKA	Phosphatidylinositol 4-kinase; controls levels of phosphatidylinositol-4-phosphate (PI(4)P) in the Golgi; non-sex gene located in MTLa mating-type-like locus; nonidentical gene encoding PI(4)P kinase, PIKALPHA, located in MTLalpha locus
PIS49634.1	24.87117825	27.08573414	24.75905156	25.68703433	25.31175695	25.27330459	0.799060252	0.952	-0.148	PIS49634.1	UBC8	Predicted ubiquitin-conjugating enzyme that negatively regulates gluconeogenesis by mediating the glucose-induced ubiquitination of fructose-1,6-bisphosphatase; induced by alpha pheromone in SpiderM medium

PIS51978.1	31.03423397	31.26335615	31.00635924	30.99400032	30.9997157	30.86270791	0.632782871	0.94	-0.149	PIS51978.1	GCN1	Ortholog(s) have protein kinase activator activity, protein kinase regulator activity, stalled ribosome sensor activity
PIS54637.1	29.05717443	29.47473292	29.19734214	28.847262	29.03530935	29.39635066	0.686892482	0.944	-0.15	PIS54637.1	DNM1	Putative dynamin-related GTPase involved in mitochondrial fission; transcript upregulated inbRHE model of oral candidiasis; transcript regulated by Nrg1, Mig1, and Tup1
PIS51372.1	27.83086758	28.47686902	26.9011148	27.21145969	27.78638363	27.76247967	0.82077073	0.953	-0.15	PIS51372.1	FRP6	Putative ammonia transport protein; regulated by Nrg1 and Tup1; regulated by Ssn6; induced by human neutrophils
PIS54739.1	29.71913109	29.74616596	28.48949789	28.11836145	29.42159086	29.96492683	0.777092905	0.951	-0.15	PIS54739.1	HBR2	Putative alanine glyoxylate aminotransferase; regulated by Gcn4p and hemoglobin; stationary phase enriched protein
PIS58298.1	23.92739274	24.1677249	23.91894249	23.96350332	23.54564822	24.05403819	0.691723386	0.945	-0.15	PIS58298.1	orf19.6709	Predicted alpha/beta hydrolase; Spider biofilm induced
PIS54790.1	23.94144672	24.33122271	25.8615109	24.56819341	24.66424849	24.45293895	0.872311892	0.956	-0.15	PIS54790.1	orf19.7433	Ortholog(s) have structural constituent of nuclear pore activity
PIS55768.1	29.64753693	29.56743525	29.71654556	29.48644336	29.4494175	29.54683399	0.654447498	0.942	-0.15	PIS55768.1	TPS3	Predicted trehalose-phosphate synthase regulatory subunit; regulated by Efg1; regulated by Tsa1, Tsa1B under H2O2 stress conditions; flow model biofilm induced (reports differ)
PIS58695.1	27.49077213	28.47365766	27.31742703	27.97720676	27.29437455	27.55773219	0.78334083	0.951	-0.151	PIS58695.1	COX4	Putative cytochrome c oxidase subunit IV; Mig1-regulated; macrophage/pseudohyphal-induced gene; macrophage-induced protein; repressed by nitric oxide; 5'-UTR intron; Hap43-repressed
PIS56600.1	30.70849984	30.87135647	30.93263248	30.5534389	30.77248856	30.73129897	0.596233195	0.936	-0.152	PIS56600.1	ARP3	Protein with Myo5p-dependent localization to cortical actin patches at hyphal tip; mutation confers hypersensitivity to cytochalasin D; Spider biofilm repressed
PIS52126.1	29.07884412	28.45928948	29.0488543	29.2440688	28.40978269	28.47336137	0.629523791	0.94	-0.153	PIS52126.1	ERG3	C-5 sterol desaturase; introduces C-5(6) double bond into episterol; some clinical isolates show increased azole resistance and defects in hyphal growth and virulence; Efg1p-repressed; fluconazole-induced

PIS55679.1	30.71703606	30.74234229	30.67206567	30.50268685	30.56402256	30.60589927	0.588272352	0.935	-0.153	PIS55679.1	RPN1	Putative 19S regulatory particle of the 26S proteasome; regulated by Gcn2p and Gcn4p
PIS54848.1	27.6601709	27.98343331	27.93040322	27.63921404	27.19631365	28.27796302	0.728435325	0.948	-0.154	PIS54848.1	GTT13	Putative glutathione S-transferase; opaque-specific transcript; repressed by alpha pheromone in SpiderM medium; Spider biofilm induced
PIS55695.1	26.14734248	25.73823261	26.92921861	26.48224332	25.72000734	26.15026418	0.859328721	0.955	-0.154	PIS55695.1	orf19.3135	Ortholog(s) have protein-macromolecule adaptor activity
PIS52394.1	27.22023292	28.18428897	25.50460622	26.89999676	26.39558019	27.15283281	0.822125995	0.953	-0.154	PIS52394.1	PLB4.5	Phospholipase B; Hog1-induced; regulated by Ssn6; putative GPI-anchor; repressed during cell wall regeneration; clade-associated gene expression; Hap43-induced; rat catheter and Spider biofilm repressed
PIS52027.1	25.52434409	26.43813426	25.02557188	24.27519285	26.09113126	26.16039015	0.838356184	0.954	-0.154	PIS52027.1	RXT3	Putative transcriptional repressor
PIS52138.1	28.9982094	29.22095035	29.23861755	29.02042118	29.22678504	28.74617011	0.714360269	0.947	-0.155	PIS52138.1	orf19.3755	Ortholog(s) have structural constituent of ribosome activity and mitochondrial large ribosomal subunit localization
PIS54880.1	28.6789966	29.36884744	28.69370595	28.6573109	28.82977214	28.78615205	0.663941224	0.943	-0.156	PIS54880.1	ADR1	C2H2 transcription factor; activates genes involved in ergosterol biosynthesis, unlike the S. cerevisiae ortholog which acts in fatty acid metabolism; activation of the protein increases resistance to azoles
PIS58611.1	28.03375626	27.75846342	27.73752001	27.66482667	27.82110667	27.57478014	0.566728375	0.933	-0.156	PIS58611.1	SNO1	Protein with a predicted role in pyridoxine metabolism; stationary phase protein; regulated by Tup1, Efg1; Spider biofilm induced
PIS52167.1	29.14336954	30.01157048	29.02540794	29.75267467	29.1357013	28.81972261	0.742883741	0.949	-0.157	PIS52167.1	orf19.3103	Ortholog(s) have RNA polymerase III activity, role in tRNA transcription by RNA polymerase III and RNA polymerase III complex, chromatin localization
PIS48673.1	25.92223427	26.36696028	25.74915629	25.93115651	25.61121946	26.02588935	0.749492597	0.949	-0.157	PIS48673.1	orf19.3406	Predicted chloride transporter; member of conserved Mcm1 regulon; Spider biofilm repressed

PIS58237.1	29.13284891	29.04361963	28.8250051	28.50109407	28.97846888	29.04990484	0.784167542	0.951	-0.157	PIS58237.1	orf19.828	Putative ribosomal protein, large subunit, mitochondrial precursor; repressed by prostaglandins; Spider biofilm repressed
PIS58924.1	25.28383405	26.82218784	24.47642371	25.41529456	25.1902954	25.50607542	0.760122467	0.95	-0.157	PIS58924.1	SHP1	Regulator of the type 1 protein phosphatase Glc7p activity, involved in control of morphogenesis, progression through the cell cycle and response to DNA damage
PIS50628.1	26.36984635	28.17153602	26.48090043	26.9180957	26.72263917	26.90656917	0.877677093	0.956	-0.158	PIS50628.1	BEM1	Protein required for wild-type budding, hyphal growth, and virulence in a mouse systemic infection; suppresses pseudohyphal and filamentous growth defects of various S. cerevisiae mutants and heat sensitivity of S. cerevisiae cdc24-4 mutant
PIS55742.1	27.58609551	28.45422037	27.71738829	27.04784612	28.30964349	27.92578541	0.856970103	0.955	-0.158	PIS55742.1	DAP1	Similar to mammalian membrane-associated progesterone receptors involved in DNA damage response; induced in core stress response; Hog1 regulated; clade-associated expression; Hap43-repressed
PIS58800.1	31.20892484	31.19855723	30.51471785	30.54302477	30.85209605	31.05203618	0.841760247	0.954	-0.158	PIS58800.1	orf19.2333	Ortholog(s) have role in retrograde transport, endosome to Golgi and cytoplasm, late endosome localization
PIS51631.1	29.50039739	29.29933626	29.77853327	29.33128639	29.11285586	29.65765849	0.573027522	0.934	-0.159	PIS51631.1	MNS1	Alpha-1,2-mannosidase; processes Man9GlcNAc2 to Man8GlcNAc2 isomer B; member of ER localized glycosyl hydrolase family 47; ER form is converted by Kex2 to cytosolic form; flow model biofilm repressed
PIS56533.1	28.2529892	28.33569507	28.91947267	28.06451261	28.47971882	28.48607868	0.859969263	0.955	-0.159	PIS56533.1	orf19.5514	Ortholog of S. pombe SPCC550.08, an N-acetyltransferase; transcript induced during growth in the mouse cecum
PIS54793.1	24.66228469	23.69104342	24.57293827	24.18612783	24.021592	24.2425266	0.746788263	0.949	-0.159	PIS54793.1	orf19.7101	Ortholog(s) have telomeric DNA binding activity, role in protein localization to chromosome, telomere maintenance, telomere maintenance via telomerase and chromosome, telomeric region localization
PIS49492.1	24.62154581	25.42033106	24.16977248	24.20774387	24.42088844	25.10241213	0.775683891	0.951	-0.16	PIS49492.1	orf19.419	Protein of unknown function; flow model biofilm induced; Spider biofilm induced
PIS51719.1	24.54401109	25.58673168	24.58843907	24.88955985	24.92461326	24.42565183	0.634206992	0.94	-0.16	PIS51719.1	orf19.7228	Possible Golgi membrane protein; transcript positively regulated by Tbf1; mRNA binds She3

PIS51677.1	28.05562253	28.56432379	28.0866627	27.75664549	28.28370324	28.18524246	0.612353263	0.938	-0.16	PIS51677.1	YML6	Putative mitochondrial ribosomal protein; induced upon adherence to polystyrene
PIS48391.1	26.61198726	26.47619509	26.8163595	26.95244197	25.6785206	26.79033484	0.802074863	0.952	-0.161	PIS48391.1	orf19.2494	Ortholog(s) have glutaminyl-tRNA synthase (glutamine-hydrolyzing) activity, role in glutaminyl-tRNAGln biosynthesis via transamidation, mitochondrial translation and glutamyl-tRNA(Gln) amidotransferase complex, mitochondrion
PIS51622.1	27.45557292	26.11913639	27.21440346	26.84158498	27.08670019	26.37559434	0.717733905	0.947	-0.162	PIS51622.1	HOM3	Putative L-aspartate 4-P-transferase; fungal-specific (no human or murine homolog); regulated by Gcn2 and Gcn4; early-stage flow model biofilm induced
PIS54718.1	25.43752255	25.4145469	24.73675141	24.26260884	25.63870998	25.19869715	0.796578657	0.952	-0.163	PIS54718.1	CDC28	Cyclin-dependent protein kinase; interacts with regulatory subunit Cyb1; determination of cell morphology during the cell cycle; phosphorylated mostly by Swe1 and phosphorylation is regulated by Hsl1; 5'-UTR intron; Spider biofilm repressed
PIS58371.1	28.20321647	29.32600793	28.50827921	28.37655185	28.66722272	28.50085491	0.793098094	0.952	-0.164	PIS58371.1	CHS5	Putative chitin biosynthesis protein; fungal-specific; repressed upon yeast-to-hypha switch; rat catheter biofilm repressed
PIS48695.1	23.43332509	25.7417527	23.84367543	23.55910448	24.63299756	24.33376337	0.791308875	0.952	-0.164	PIS48695.1	FCA1	Cytosine deaminase; enzyme of pyrimidine salvage; functional homolog of S. cerevisiae Fcy1p; mutation is associated with resistance to flucytosine (5-FC) in a clinical isolate; hyphal downregulated; gene has intron
PIS49843.1	29.75379724	29.49271215	29.66145862	29.55966964	29.54184267	29.31176762	0.76615002	0.95	-0.165	PIS49843.1	CUP9	Transcription factor; represses SOK1 expression in response to farnesol inhibition; yeast-hypha switch repressed; ketoconazole-induced; Plc1-regulated; colony morphology-related Ssn6 regulation; Spider, flow model biofilm induced
PIS48397.1	24.63299004	24.89010152	24.75874484	24.63015773	24.60303351	24.5528792	0.7048187	0.946	-0.165	PIS48397.1	orf19.2484	Has domain(s) with predicted metalloexopeptidase activity and role in proteolysis
PIS51127.1	29.66561967	28.94693826	29.69938865	29.20514346	29.30727732	29.30589182	0.619215619	0.939	-0.165	PIS51127.1	VMA13	Predicted proton-transporting ATPase; predicted role in ATP hydrolysis coupled proton transport; rat catheter biofilm repressed
PIS48292.1	29.19262687	29.66402978	29.38241122	29.31866746	29.17111758	29.25519356	0.534084275	0.929	-0.165	PIS48292.1	YCK2	Plasma membrane protein similar to S. cerevisiae casein kinase I; mutation or inhibition impairs virulence and morphogenesis; transcription is activated in weak acid stress or on contact with with host cells

PIS58569.1	28.55860943	28.47944788	28.63843986	28.36152081	28.43145501	28.38603795	0.624031545	0.939	-0.166	PIS58569.1	LYS21	Homocitrate synthase, major isoform; lysine biosynthesis; expression increased in a fluconazole-resistant isolate; colony morphology-related gene regulation by Ssn6; ketoconazole-repressed; regulated by Gcn2, Gcn4, Hap43
PIS58261.1	25.1145088	26.34690076	25.63650707	25.30906873	25.37281881	25.91405108	0.724486684	0.947	-0.167	PIS58261.1	orf19.2604	S. pombe ortholog SPAC2C4.06c is a predicted tRNA (cytosine-5-)-methyltransferase; Spider biofilm induced
PIS58357.1	31.88298714	30.96277299	31.82175232	31.52417729	31.40183635	31.24006595	0.850849932	0.955	-0.167	PIS58357.1	orf19.5136	Putative pyridoxamine 5'-phosphate oxidase; planktonic growth and early-stage flow model biofilm induced
PIS51963.1	27.31214412	26.92316211	28.02860658	26.01224636	27.77434576	27.97504508	0.794301428	0.952	-0.167	PIS51963.1	orf19.6740	Ortholog(s) have ubiquitin-protein transferase activity and role in generation of catalytic spliceosome for first transesterification step
PIS58254.1	27.51957163	27.96664982	26.91934094	27.09188056	27.34046549	27.47133227	0.694295859	0.945	-0.167	PIS58254.1	orf19.7489.3	Ortholog(s) have 2 iron, 2 sulfur cluster binding, iron-sulfur cluster binding activity
PIS48518.1	25.75352399	25.05653274	25.85295182	26.18669905	24.91896792	25.05704868	0.75215933	0.949	-0.167	PIS48518.1	SRB8	Putative RNA polymerase II mediator complex subunit; early-stage flow model biofilm induced
PIS52097.1	31.82918615	31.98386141	31.78996563	31.61846268	31.68347356	31.79625609	0.553962195	0.932	-0.168	PIS52097.1	GRS1	Putative tRNA-Gly synthetase; genes encoding ribosomal subunits, translation factors, tRNA synthetases are downregulated upon phagocytosis by murine macrophage; stationary phase enriched protein
PIS58588.1	26.23240165	27.19268516	25.43230359	25.5896453	26.51754541	26.24649348	0.758904786	0.95	-0.168	PIS58588.1	OAC1	Putative mitochondrial inner membrane transporter; rat catheter biofilm induced
PIS58806.1	30.06036266	30.0968611	30.7631998	30.51180726	30.19284439	29.71230257	0.630810804	0.94	-0.168	PIS58806.1	orf19.409	Ortholog of S. cerevisiae Aim38/Rcf2, cytochrome c oxidase subunit; plasma membrane localized; Hap43-repressed; induced in oralpharyngeal candidasis; flow model biofilm induced; Spider biofilm repressed
PIS58485.1	27.95076079	28.1635316	27.83202013	27.57420834	27.91311386	27.95269982	0.687952075	0.945	-0.169	PIS58485.1	CST20	Protein kinase of Ste20p/p65PAK family, required for wild-type mating efficiency and virulence in a mouse model; Cst20p-Hst7p-Cek1p-Cph1p MAPK pathway regulates some hyphal growth; involved in Cdc42p growth regulation

PIS49754.1	26.35349007	26.95934523	25.92265235	26.55918849	25.73177704	26.43639786	0.74235363	0.948	-0.169	PIS49754.1	LEU1	3-isopropylmalate dehydratase; antigenic in humans; repressed in hyphae; alkaline repressed; upregulated by human whole blood or PMNs; regulated by Sef1, Sfu1, and Hap43; rat catheter biofilm induced, Spider biofilm repressed
PIS55819.1	28.44235035	28.11165878	28.54761778	28.24409197	27.72661629	28.6211639	0.675865058	0.944	-0.17	PIS55819.1	ALG11	Alpha-1,2-mannosyltransferase; catalyzes sequential addition of 2 terminal alpha 1,2-mannose residues to the Man5GlcNAc2-PP-dolichol intermediate during asparagine-linked glycosylation in the ER; Spider biofilm induced
PIS59015.1	31.8608175	32.41749766	31.81991015	31.76121324	31.85498758	31.97227494	0.627089639	0.939	-0.17	PIS59015.1	CDC48	Putative microsomal ATPase; plasma membrane-localized; regulated by Gcn2 and Gcn4; induced by amino acid starvation (3-AT); macrophage/pseudohyphal-repressed; protein levels decrease in stationary phase yeast; Spider biofilm
PIS58347.1	22.607787	23.11519987	22.6997821	22.56903638	22.53072204	22.81138826	0.880915559	0.956	-0.171	PIS58347.1	orf19.1338	Protein of unknown function; possible COPI-coated vesicle, Golgi apparatus, ribosome localization; rat catheter biofilm repressed
PIS51535.1	30.04666456	29.80105903	30.28206247	30.1263516	29.66808166	29.82164218	0.574052506	0.934	-0.171	PIS51535.1	QCR8	Putative ubiquinol cytochrome c reductase; macrophage and pseudohyphal-induced protein; colony morphology-related gene regulation by Ssn6; Hap43-repressed; Spider biofilm repressed
PIS54985.1	25.69221387	25.82738209	24.19056478	24.75395905	25.10277671	25.34130111	0.804782292	0.952	-0.171	PIS54985.1	UTP13	Putative U3 snoRNA-associated protein; Hap43-induced; repressed in core stress response; physically interacts with TAP-tagged Nop1
PIS56577.1	26.23566742	25.30199558	25.65590252	25.49421278	25.84782956	25.33417544	0.804701859	0.952	-0.172	PIS56577.1	orf19.102	Protein required for expression of mitochondrial ATP synthase and cytochrome c oxidase (respiratory chain complex IV)
PIS58920.1	29.20544902	28.60089418	28.93961165	28.5796493	28.95600296	28.6952154	0.759789636	0.95	-0.172	PIS58920.1	orf19.2544	Ortholog(s) have aminoacyl-tRNA hydrolase activity and role in negative regulation of proteasomal ubiquitin-dependent protein catabolic process
PIS49692.1	30.2984517	30.11253226	29.89050895	29.90436585	29.73772619	30.14344295	0.545295905	0.93	-0.172	PIS49692.1	orf19.5095	Putative oxysterol-binding protein; caspofungin induced; possibly an essential gene, disruptants not obtained by UAU1 method
PIS55611.1	24.26462636	25.31721603	23.20240072	24.89912606	23.92807821	23.44055587	0.756089634	0.949	-0.172	PIS55611.1	URK1	Protein similar to uridine kinase; repressed by ciclopirox olamine; Spider biofilm induced

PIS50473.1	25.67983214	25.79680575	24.80497249	25.05314357	25.30991105	25.39823964	0.830711059	0.954	-0.173	PIS50473.1	orf19.3956	Ortholog(s) have glutaminyl-tRNA synthase (glutamine-hydrolyzing) activity and role in endoplasmic reticulum organization, glutaminyl-tRNAGln biosynthesis via transamidation
PIS48641.1	24.63170742	24.92365846	25.34126365	24.63206049	24.81957776	24.92696521	0.674644119	0.943	-0.173	PIS48641.1	YCG1	Putative condensin G
PIS51064.1	27.86348071	26.5082501	27.81605495	27.7946971	26.62600959	27.2439775	0.692841048	0.945	-0.174	PIS51064.1	GPA2	G-protein alpha subunit; regulates filamentous growth, copper resistance; involved in cAMP-mediated glucose signaling; reports differ on role in cAMP-PKA pathway, MAP kinase cascade; Gpr1 C terminus binds Gpa2; regulates HWP1 and ECE1
PIS51662.1	25.11827918	25.72583197	25.05675217	25.45563511	24.73280215	25.1909205	0.767981252	0.95	-0.174	PIS51662.1	orf19.2980	Ortholog(s) have RNA helicase activity and role in generation of catalytic spliceosome for first transesterification step, spliceosomal complex disassembly
PIS51104.1	24.7074952	25.47822097	24.19191601	24.60253214	24.65658925	24.59453593	0.75452261	0.949	-0.175	PIS51104.1	BNR1	Formin; probable role in hyphal cytoskeletal polarity; synthetic lethality if Bnr1p and Bni1p are absent
PIS55795.1	26.46726114	27.04341082	26.31055185	26.3150957	26.27965018	26.70270341	0.661041082	0.942	-0.175	PIS55795.1	UTR2	Putative GPI anchored cell wall glycosidase; role in adhesion, hyphal growth on Spider (not serum) medium; chitin-binding, glycosyl hydrolase domains; induced during cell wall regeneration; mRNA in yeast-form cells; Spider biofilm induced
PIS48406.1	27.6234915	27.84114617	27.56459756	27.03855744	27.73221588	27.72996093	0.66521528	0.943	-0.176	PIS48406.1	LPI9	Protein phosphatase type 1 regulator; role in chromosome segregation, regulation of phosphoprotein phosphatase activity; rat catheter and Spider biofilm induced
PIS49809.1	26.66719763	25.7733978	26.01245739	26.48768087	26.31695328	25.12018858	0.80582671	0.952	-0.176	PIS49809.1	orf19.551	Ortholog of C. dubliniensis CD36 : Cd36_29980, C. parapsilosis CDC317 : CPAR2_204170, C. auris B8441 : B9J08_004836 and Candida tenuis NRRL Y-1498 : CANTEDRAFT_110235
PIS58636.1	32.29351697	31.99962693	32.25457284	31.88179069	32.07206251	32.06295405	0.664052158	0.943	-0.177	PIS58636.1	GSP1	Small RAN G-protein; essential; no prenylation predicted; can rescue S. cerevisiae gsp1 viability; macrophage/pseudohyphal-induced; transcript not regulated by white-opaque, yeast-hypha switching; GlcNAc-induced; Spider biofilm repressed
PIS51322.1	30.35004649	30.46145095	30.40251863	30.1554211	30.1704895	30.35644474	0.552520509	0.931	-0.177	PIS51322.1	orf19.7459	Putative mitochondrial protein with a predicted role in respiratory growth; fluconazole-induced; ketoconazole-repressed; mutants display a strong defect in flow model biofilm formation; Spider biofilm induced

PIS48656.1	24.61210625	24.67171503	23.89755865	23.97602651	24.27426871	24.39723641	0.70601611	0.946	-0.178	PIS48656.1	orf19.496	Ortholog(s) have ATP-dependent activity, acting on DNA, dinucleotide insertion or deletion binding, guanine/thymine mispair binding activity
PIS58889.1	31.00968076	32.3810676	30.67502417	30.52602593	31.30590285	31.69933789	0.708657255	0.946	-0.178	PIS58889.1	RPL28	Putative ribosomal protein; Plc1-regulated; downregulated upon phagocytosis by murine macrophage; Spider biofilm repressed
PIS58252.1	29.0648515	28.68967073	28.47234709	28.48967776	28.57134524	28.62859951	0.691569002	0.945	-0.179	PIS58252.1	NIF3	Protein of unknown function; ortholog of S. cerevisiae Nif3; rat catheter biofilm repressed
PIS51062.1	26.79866821	26.03661781	26.76736113	26.68838149	26.13444884	26.24217396	0.839851293	0.954	-0.179	PIS51062.1	orf19.1618.1	Ortholog(s) have role in mitochondrial fission and mitochondrial intermembrane space localization
PIS49825.1	22.99267487	22.8961028	23.02351392	22.55732279	22.90653673	22.90846892	0.609679841	0.938	-0.18	PIS49825.1	orf19.4365	Has domain(s) with predicted RNA methyltransferase activity and role in RNA processing
PIS55688.1	25.46805377	25.69761933	26.1447654	25.66589964	25.72901068	25.3742935	0.680308978	0.944	-0.18	PIS55688.1	orf19.4666	Protein of unknown function; hyphal-induced expression, regulated by Cyr1, Ras1, Efg1; Spider biofilm induced
PIS51475.1	29.65540972	29.71502625	29.28319145	29.39718692	29.40164331	29.31605485	0.570042408	0.933	-0.18	PIS51475.1	orf19.7234	Component of the RSC chromatin remodeling complex; possibly an essential gene, disruptants not obtained by UAU1 method
PIS48599.1	29.56021363	29.55966474	29.25651564	28.47914485	29.59581253	29.76120429	0.670306599	0.943	-0.18	PIS48599.1	TIM50	Predicted component of the Translocase of the Inner Mitochondrial membrane (TIM23 complex); involved in mitochondrial protein import; Spider biofilm repressed
PIS51547.1	33.08948875	32.84615075	33.35704068	33.09603677	32.88293252	32.77060691	0.534074798	0.929	-0.181	PIS51547.1	ATP4	Putative F0-ATP synthase subunit 4; macrophage/pseudohyphal-induced; present in exponential and stationary growth phases
PIS58176.1	30.31296732	30.04168581	30.32746115	29.97148954	30.17354378	29.99290248	0.640285804	0.941	-0.181	PIS58176.1	BUD7	Ortholog(s) have small GTPase binding activity and role in Golgi to plasma membrane protein transport, Golgi to plasma membrane transport, Golgi to vacuole transport, fungal-type cell wall chitin biosynthetic process

PIS54554.1	27.72946375	28.71408238	27.11182821	27.22409298	27.78793663	27.99940426	0.711689863	0.946	-0.181	PIS54554.1	orf19.2965	Putative guanyl-nucleotide exchange factor; Spider biofilm repressed
PIS55725.1	26.09370202	26.3861337	25.21916275	24.95172249	25.63134691	26.57429163	0.786484923	0.951	-0.181	PIS55725.1	PGA45	Putative GPI-anchored cell wall protein; repressed in core caspofungin response; Hog1-induced; regulated by Ssn6; Mob2-dependent hyphal regulation; flow model biofilm induced
PIS58165.1	30.00226593	30.36163374	30.01729248	29.96352906	29.87441358	29.99958734	0.683755858	0.944	-0.181	PIS58165.1	SVF1	Putative survival factor; stationary phase enriched protein; fluconazole-induced; regulated by Gcn4p; repressed in response to amino acid starvation (3-AT treatment); fungal-specific (no human or murine homolog)
PIS52300.1	25.87634966	25.58814653	25.58648365	25.35707232	25.25426027	25.89283904	0.669983491	0.943	-0.182	PIS52300.1	orf19.2726	Putative plasma membrane protein; Plc1-regulated; Spider biofilm induced
PIS51403.1	26.97826602	27.20558139	26.57943183	25.72782301	27.34098476	27.14806295	0.729233152	0.948	-0.182	PIS51403.1	YKU80	Yku70p-Yku80p Ku complex subunit involved in nonhomologous end joining during double-strand break repair repair; Hap43-repressed gene; flow model biofilm induced
PIS54564.1	26.59646062	25.86739711	26.21488918	25.87984472	25.72732425	26.52395983	0.619925082	0.939	-0.183	PIS54564.1	orf19.4153	Ortholog(s) have NEDD8 activating enzyme activity and role in protein neddylation
PIS56732.1	24.6498384	24.1044698	25.82577876	25.24402316	24.286877	24.50162714	0.75882384	0.95	-0.183	PIS56732.1	TRP1	Phosphoribosylanthranilate isomerase; tryptophan biosynthesis; expected unifunctional, unlike trifunctional enzyme of some other fungi; complements E. coli trpC, S. cerevisiae trp1 mutant; CCT1 and TRP1 overlap; Spider biofilm repressed
PIS58369.1	24.34629664	25.41535164	24.07200928	24.11272276	24.43835365	24.72759572	0.64870308	0.941	-0.185	PIS58369.1	orf19.805	Putative protein of unknown function; possibly mitochondrial; Hap43-repressed; Spider biofilm repressed
PIS54896.1	30.96351108	30.70834754	31.13663561	30.77004344	30.60332988	30.87574763	0.667419681	0.943	-0.186	PIS54896.1	ARO1	Putative pentafunctional arom enzyme; required for aromatic amino acid biosynthesis; involved in cell wall integrity and formation of biofilm; fungal-specific (no human or murine homolog); Gcn2p-, Gcn4p-regulated
PIS51515.1	28.37942505	28.87124978	28.49088252	28.16658736	28.33410673	28.68384726	0.634134479	0.94	-0.186	PIS51515.1	ILV1	Putative threonine dehydratase; regulated by Gcn4 and Gcn2; induced by amino acid starvation (3-AT); protein present in yeast exponential and stationary phase cultures; early-stage flow model biofilm induced

PIS58214.1	31.85015003	31.32875293	32.03342152	31.67716403	31.60667233	31.36930991	0.607846127	0.937	-0.186	PIS58214.1	orf19.3035	Ortholog(s) have ATP-dependent activity, acting on DNA, ATP-dependent chromatin remodeler activity, DNA binding, chromatin DNA binding, methylated histone binding, nucleosome binding and rDNA binding, more
PIS51531.1	30.7771423	30.85333631	30.94100323	30.63146161	30.7264929	30.65301499	0.497710929	0.924	-0.187	PIS51531.1	NDH51	Nicotinamide adenine dinucleotide dehydrogenase complex I subunit of the mitochondrial electron transport chain; required for wild-type filamentous growth; alkaline repressed; Hap43-repressed; Spider biofilm repressed
PIS48648.1	30.73429888	31.12977459	30.59124282	30.30366666	30.82115202	30.76681352	0.77936079	0.951	-0.188	PIS48648.1	ATP3	F1-ATP synthase complex subunit; caspofungin repressed; flucytosine and macrophage/pseudohyphal-induced; present in exponential and stationary growth phases; Hap43p-induced gene
PIS54622.1	31.76798853	31.83993713	31.80722046	31.34788605	31.76967444	31.73476126	0.678187585	0.944	-0.188	PIS54622.1	SUP35	Translation factor eRF3; shows prion-like aggregation in some, not all, studies; partially complements S. cerevisiae sup35 mutant translation defect; species barrier with S. cerevisiae Sup35p prion; gene not regulated by yeast-hyphal switch
PIS55053.1	28.58642428	28.87861252	28.53689688	28.63281718	28.21072902	28.5907198	0.88576648	0.957	-0.189	PIS55053.1	orf19.4597	Putative F-actin-capping protein subunit beta; possibly an essential gene, disruptants not obtained by UAU1 method
PIS50468.1	29.53583608	30.4899136	29.56349825	29.59443019	29.51370392	29.9115834	0.759642332	0.95	-0.19	PIS50468.1	CAR1	Arginase; arginine catabolism; transcript regulated by Nrg1, Mig1, Tup1; colony morphology-related regulation by Ssn6; alkaline induced; protein decreased in stationary phase; sumoylation target; flow model biofilm induced
PIS51459.1	26.27564359	27.11299338	25.3298152	23.84413539	27.31306093	26.98917691	0.843230635	0.954	-0.191	PIS51459.1	orf19.5022	Ortholog(s) have inorganic cation transmembrane transporter activity and role in cobalt ion transport, intracellular cobalt ion homeostasis, intracellular manganese ion homeostasis, manganese ion transport
PIS51717.1	25.19147119	26.16923354	25.08886927	25.57671147	25.11936769	25.1794097	0.665592417	0.943	-0.191	PIS51717.1	RBE1	Pry family cell wall protein; Rim101, Efg1, Ssn6, alkaline repressed; O-glycosylation; no GPI anchor predicted; ketoconazol induced; regulated by Sef1, Sfu1, Hap4; flow model biofilm induced; rat catheter and Spider biofilm repressed
PIS55640.1	23.83505202	25.33127391	22.61074923	22.87239923	23.81635027	24.51151905	0.867609841	0.956	-0.192	PIS55640.1	orf19.1662	Ortholog(s) have structural constituent of ribosome activity and mitochondrial small ribosomal subunit localization
PIS49487.1	31.97163474	31.50921248	32.12035727	31.81353233	31.63957506	31.57339566	0.664631194	0.943	-0.192	PIS49487.1	PHO88	Protein with a role in phosphate transport; biofilm-regulated expression; amphotericin B repressed

PIS58861.1	25.23646842	24.59846414	25.60325537	25.12290567	24.86264863	24.8736027	0.539862031	0.93	-0.193	PIS58861.1	KIP4	S. cerevisiae Smy1 ortholog; Tn mutation affects filamentous growth; filament induced; has Mob2-dependent hyphal regulation; regulated by Nrg1, Tup1; oralpharyngeal candidasis induced; Spider biofilm induced; flow model biofilm repressed
PIS48817.1	25.70743095	24.98135438	25.45166775	25.27824473	25.12004618	25.16446399	0.721853999	0.947	-0.193	PIS48817.1	orf19.4807	Ortholog(s) have inorganic diphosphate phosphatase activity, role in aerobic respiration and mitochondrion localization
PIS55799.1	30.42149047	30.59409586	30.58213819	30.83661995	30.09552402	30.08436432	0.654323469	0.942	-0.194	PIS55799.1	AFG3	Similar to S. cerevisiae Afg3p, a subunit of the mitochondrial inner membrane m-AAA protease; likely to be essential for growth, based on an insertional mutagenesis strategy
PIS55822.1	31.8182558	31.55613965	31.98483376	31.57458158	31.59736032	31.60636868	0.550487465	0.931	-0.194	PIS55822.1	orf19.1212	Ortholog(s) have FFAT motif binding, phosphatidylinositol binding activity
PIS49717.1	29.8379485	29.59077252	29.68593838	29.64517845	29.39797824	29.48958417	0.728500697	0.948	-0.194	PIS49717.1	orf19.6804	Ortholog(s) have role in SRP-dependent cotranslational protein targeting to membrane and signal recognition particle, endoplasmic reticulum targeting localization
PIS56843.1	27.93713454	27.7290325	27.6088681	27.75046768	27.27493304	27.66791345	0.579352085	0.934	-0.194	PIS56843.1	TYR1	Putative prepephenate dehydrogenase; enzyme of tyrosine biosynthesis; fungal-specific (no human or murine homolog)
PIS51061.1	32.06456733	31.15140048	31.98402549	31.60593192	31.5321032	31.47807661	0.512867395	0.926	-0.195	PIS51061.1	GFA1	Glucosamine-6-phosphate synthase, homotetrameric enzyme of chitin/hexosamine biosynthesis; inhibited by UDP-GlcNAc, FMDP, N-acyl peptide, kanosamine-6-P; functional homolog of S. cerevisiae Gfa1p; Cagrowth-phase regulated; catalytic Cys
PIS51058.1	26.98901948	27.96203092	27.29593341	27.11196352	27.31607412	27.23360105	0.648012078	0.941	-0.195	PIS51058.1	ILV2	Putative acetolactate synthase; regulated by Gcn4p; induced by amino acid starvation (3-AT treatment); stationary phase enriched protein
PIS55602.1	22.96034044	23.27250665	23.7186674	23.16748219	23.05530481	23.14505392	0.769039575	0.95	-0.195	PIS55602.1	orf19.4533	Ortholog(s) have RNA binding activity and role in rRNA processing
PIS58834.1	28.23031658	27.59530326	28.35429138	27.83013182	27.63761779	28.12846539	0.679849362	0.944	-0.195	PIS58834.1	PKC1	Protein kinase C; functional homolog of S. cerevisiae Pkc1p; mutant has abnormal yeast-form cell morphology and increased cell lysis; activated by phosphatidylserine; target of antifungal, cercosporamide; R400P mutant is activated

PIS51707.1	27.53552112	26.85306516	26.585497	27.08061138	26.72843156	26.5786373	0.884326504	0.957	-0.195	PIS51707.1	SYS3	Protein similar to S. cerevisiae Sys3p; putative role in endosome-Golgi vesicle docking; upregulated in biofilm; induced upon adherence to polystyrene
PIS52332.1	27.04385572	28.38204959	26.27048564	26.43970898	27.19312547	27.4759038	0.805721065	0.952	-0.196	PIS52332.1	orf19.6348	Predicted cysteine proteinase domain; mutants are viable
PIS55815.1	28.44917776	28.46060963	28.57555549	28.41372334	28.58636277	27.89720747	0.564101182	0.933	-0.196	PIS55815.1	orf19.7378	Putative phosphopantothenoylcysteine decarboxylase, binds to protein phosphatase Ppz1p and regulates its activity
PIS48273.1	33.75829202	33.70764161	34.02489579	33.51904165	33.75110206	33.63199929	0.532945789	0.929	-0.196	PIS48273.1	RPL6	Ortholog of S. cerevisiae ribosomal subunit, Rpl6B; transposon mutation affects filamentous growth; translation-related genes are downregulated upon phagocytosis by murine macrophage; Hap43-induced; Spider biofilm repressed
PIS56546.1	28.86979991	29.36656755	28.74506688	28.808259	28.62444771	28.96134626	0.580747455	0.935	-0.196	PIS56546.1	SLM2	Ortholog(s) have role in TOR signaling, actin cytoskeleton organization, actin filament bundle assembly, cell-cell fusion, eisosome assembly and endosomal transport, more
PIS55529.1	28.02195363	28.21274341	27.7343904	27.63099172	27.80935704	27.93889315	0.548025759	0.931	-0.197	PIS55529.1	CDC37	Chaperone for Crk1p; interacts with Crk1p kinase domain and with Sti1p; putative phosphorylation site at Ser14; functional homolog of S. cerevisiae Cdc37p; likely to be essential for growth; regulated by Gcn2p and Gcn4p
PIS54568.1	23.7141621	25.26893882	24.26721106	24.60942734	23.9176493	24.1283582	0.771387959	0.95	-0.198	PIS54568.1	MBP1	Putative component of the MBF transcription complex involved in G1/S cell-cycle progression; non-periodic mRNA expression; predicted, conserved MBF binding sites upstream of G1/S-regulated genes
PIS54614.1	28.67587282	29.62718375	28.87639702	28.98264233	28.7358228	28.86753273	0.598425409	0.936	-0.198	PIS54614.1	orf19.1392	Ortholog(s) have protein disulfide isomerase activity, protein-disulfide reductase (glutathione) activity, unfolded protein binding activity and role in protein retention in ER lumen, ubiquitin-dependent ERAD pathway
PIS55042.1	24.01831701	24.32307199	23.58604768	23.19308593	23.89634021	24.24412196	0.644221711	0.941	-0.198	PIS55042.1	POM152	Putative nuclear pore membrane glycoprotein
PIS49548.1	30.09495152	30.40673248	29.86673928	29.68906968	29.95365948	30.13166883	0.54221362	0.93	-0.198	PIS49548.1	PRC3	Putative carboxypeptidase Y precursor; transcript regulated by Nrg1 and Mig1; regulated by Gcn2 and Gcn4

PIS52376.1	32.03241982	32.74501123	32.38021411	32.73095698	32.00537167	31.82366406	0.792566048	0.952	-0.199	PIS52376.1	HSP60	Heat shock protein; soluble in hyphae; regulated by Nrg1 and by iron; induced in high iron; heavy metal (cadmium) stress-induced; sumoylation target; protein present in exponential and stationary phase cells; Hap43-repressed
PIS48519.1	31.18364224	30.60590608	31.6843609	31.22876033	30.81023985	30.83781257	0.681369944	0.944	-0.199	PIS48519.1	MYO5	Class I myosin; nonessential; role in cortical actin patch polarity and polar budding; required for hyphal growth, white-opaque switch; regulatory phosphorylation on S366; downregulated on adherence to polystyrene
PIS48450.1	26.30991616	27.19823929	26.89730129	26.64377037	26.98875982	26.17460077	0.804866317	0.952	-0.199	PIS48450.1	orf19.1043	Ortholog(s) have lysophosphatidic acid acyltransferase activity, role in intracellular triglyceride homeostasis, lipid droplet organization and endoplasmic reticulum, lipid droplet localization
PIS55540.1	30.66595304	30.82108685	30.35682473	30.3920342	30.33238355	30.52171508	0.57086764	0.933	-0.199	PIS55540.1	orf19.2095	Ortholog(s) have role in negative regulation of transcription by RNA polymerase II and cytosol localization
PIS58247.1	23.92701588	24.27458745	24.61336737	23.9564278	25.14875381	23.11169571	0.714242743	0.947	-0.199	PIS58247.1	orf19.5165	Ortholog(s) have GTPase activity, enzyme activator activity, zinc chaperone activity and role in cellular response to zinc ion starvation, protein maturation
PIS51845.1	29.80399691	29.70971009	29.84569381	29.7652895	29.5647594	29.43314084	0.669394147	0.943	-0.199	PIS51845.1	RAS1	RAS signal transduction GTPase; regulates cAMP and MAP kinase pathways; role in hyphal induction, virulence, apoptosis, heat-shock sensitivity; nonessential; plasma membrane-localized; complements viability of S. cerevisiae ras1 ras2
PIS58650.1	26.91383736	27.0921134	27.18129204	26.95241398	26.74889174	26.88647563	0.691274444	0.945	-0.2	PIS58650.1	ECM22	Zn(II)2Cys6 transcription factor; rat catheter and Spider biofilm induced
PIS50314.1	31.40839815	31.20047959	31.5266771	31.6417835	31.05068837	30.84438036	0.710950303	0.946	-0.2	PIS50314.1	KRE30	YEF3-subfamily ABC family protein; predicted not to be a transporter; repressed in core stress response; mutation confers hypersensitivity to amphotericin B
PIS51747.1	26.27901586	26.72475383	26.39018691	27.69154194	24.59514579	26.50596935	0.786035979	0.951	-0.2	PIS51747.1	orf19.2037	Has domain(s) with predicted role in cellular response to amino acid stimulus, cholesterol homeostasis, endosomal transport, lysosome organization and positive regulation of MAPK cascade, more
PIS48776.1	29.19287426	29.2579035	29.58964978	29.20517527	28.98193694	29.25297994	0.493594418	0.923	-0.2	PIS48776.1	orf19.518	Ortholog(s) have tRNA (cytidine-5-)-methyltransferase activity, tRNA binding activity, role in tRNA methylation, tRNA wobble base cytosine methylation and nucleus localization

PIS48434.1	24.90168519	24.82760702	24.93049613	24.13591572	25.39292685	24.53064774	0.790944371	0.952	-0.2	PIS48434.1	orf19.5363	Component of UDP-GlcNAc transferase; required for the 2nd step of dolichyl-linked oligosaccharide synthesis; Spider biofilm induced
PIS55706.1	28.91169513	28.74286427	29.25803053	29.35884906	28.70591597	28.24812383	0.61384041	0.938	-0.2	PIS55706.1	YVC1	Putative vacuolar cation channel shock; repressed by alpha pheromone in SpiderM medium; flow model biofilm repressed
PIS54675.1	25.63253845	25.24210984	25.5851757	25.39039049	25.17147227	25.29377629	0.708371338	0.946	-0.201	PIS54675.1	HYM1	Protein of RAM cell wall integrity signaling network; involved in regulation of Ace2 activity and cellular morphogenesis; role in cell separation, azole sensitivity; required for hyphal growth
PIS51807.1	25.82834939	24.1690561	25.73106837	25.25264265	24.74245255	25.13119871	0.716923615	0.947	-0.201	PIS51807.1	orf19.3304	Exosome non-catalytic core component; involved in 3'-5' RNA processing and degradation in the nucleus and cytoplasm; Spider biofilm induced
PIS58027.1	29.96607977	29.43097903	30.15909569	29.88706638	29.49672988	29.56790606	0.554309075	0.932	-0.201	PIS58027.1	TOM1	Putative E3 ubiquitin ligase; transcript regulated by Nrg1 and Mig1
PIS58539.1	31.83304657	31.7057899	31.4872063	31.22464171	31.47606992	31.71793088	0.46015824	0.918	-0.202	PIS58539.1	orf19.4246	Phosphatase involved in stress response; Tn mutation affects filamentation; Hog1-repressed; colony morphology-related gene regulation by Ssn6p; induced during cell wall regeneration
PIS51590.1	24.60650967	23.38121804	24.62717981	24.95993735	24.26143136	22.78640751	0.787983888	0.951	-0.202	PIS51590.1	VPS33	Ortholog(s) have ATP binding, phosphatidylinositol binding activity
PIS48753.1	28.23813369	27.9025605	27.74902635	27.51491546	27.85670664	27.90915476	0.698933997	0.945	-0.203	PIS48753.1	GCN3	Putative translation initiator; downregulated in the presence of human whole blood or polymorphonuclear (PMN) cells
PIS54558.1	28.41914215	28.23305779	27.51439483	27.84859454	27.75164832	27.95689247	0.698378313	0.945	-0.203	PIS54558.1	orf19.1964	Protein of unknown function; repressed by fluphenazine treatment; induced by benomyl treatment and in an RHE model; regulated by Nrg1, Tup1
PIS52056.1	29.60570955	29.09159649	29.73910561	29.41736956	29.13137156	29.27820194	0.496569446	0.924	-0.203	PIS52056.1	YPT1	Functional homolog of S. cerevisiae Ypt1p, which is an essential small Ras-type GTPase involved in protein secretion at ER-to-Golgi; dominant-negative mutation causes SAP secretion defect and accumulation of intracellular secretory vesicles

PIS52129.1	23.50772649	23.59276662	23.36777245	23.12694045	23.20148327	23.52803366	0.80958067	0.953	-0.204	PIS52129.1	RAD1	Putative single-stranded DNA endonuclease; transcript regulated by Nrg1; macrophage-induced gene
PIS54754.1	27.19348929	27.58839663	26.96836375	26.9458919	27.45686248	26.7350854	0.623526245	0.939	-0.204	PIS54754.1	SCT2	Putative glycerol-3-phosphate acyltransferase; fungal-specific (no human or murine homolog)
PIS49794.1	29.84083692	30.23611564	29.51284974	29.68953153	29.65311739	29.63610825	0.590021531	0.936	-0.204	PIS49794.1	STI1	Protein that interacts with Cdc37 and Crk1 in two-hybrid; may be involved in Cdc37 chaperone activity; soluble protein in hyphae; protein in exponential and stationary phase yeast cultures; YNB biofilm induced; Spider biofilm induced
PIS49478.1	31.16427805	30.1238466	31.30000691	31.1499599	30.49671747	30.32550214	0.632249685	0.94	-0.205	PIS49478.1	ALO1	D-Arabinono-1,4-lactone oxidase involved in biosynthesis of dehydro-D-arabinono-1,4-lactone, which has a protective role against oxidative damage; plasma membrane-localized;required for full virulence in a mouse model of systemic infection
PIS51697.1	32.02428125	31.66136987	32.01284381	31.600781	31.84647448	31.63246573	0.501889108	0.925	-0.206	PIS51697.1	NIP1	Putative translation initiation factor; mutation confers hypersensitivity to roridin A and verrucarin A; genes encoding ribosomal subunits, translation factors, and tRNA synthetases are downregulated upon phagocytosis by murine macrophage
PIS49550.1	34.72034094	34.34570066	34.75185765	34.38876132	34.50184063	34.30886386	0.554594922	0.932	-0.206	PIS49550.1	orf19.2478.1	60S ribosomal protein L7; snoRNA snR39b encoded within the 2nd intron
PIS54963.1	26.90731062	26.95790295	26.78329302	27.27235889	26.31671182	26.44144476	0.843531421	0.954	-0.206	PIS54963.1	TIM9	Predicted protein of the mitochondrial intermembrane space; rat catheter biofilm induced; Spider biofilm repressed
PIS48524.1	27.27573142	27.84174212	27.98185159	27.97786219	27.40131106	27.09607322	0.672608771	0.943	-0.208	PIS48524.1	ABZ1	Ortholog(s) have 4-amino-4-deoxychorismate synthase activity and role in para-aminobenzoic acid biosynthetic process
PIS48485.1	28.51254054	28.11560287	28.48553353	28.10197642	28.19590991	28.19076566	0.677981517	0.944	-0.208	PIS48485.1	orf19.1888	Putative nicotinamide riboside hydrolase; cleaves N-glycosidic bonds in nucleosides; pyrimidine salvage and nicotinamide riboside salvage pathways; Spider biofilm induced
PIS51050.1	22.74140852	24.54923217	23.07580867	22.76609095	23.40668959	23.56749238	0.72258169	0.947	-0.209	PIS51050.1	orf19.2513	Protein required for expression of NADH:ubiquinone oxidoreductase (mitochondrial complex I)

PIS55022.1	26.07186256	26.02253564	26.15398047	26.35348418	25.62545447	25.64173923	0.672731551	0.943	-0.209	PIS55022.1	orf19.6056	Ortholog(s) have phosphatase activity and role in dephosphorylation
PIS58313.1	22.84995873	24.86615055	22.72238234	22.72935943	24.09335148	22.98653266	0.699031456	0.945	-0.21	PIS58313.1	FGR50	Protein lacking an ortholog in S. cerevisiae; transposon mutation affects filamentous growth; Spider biofilm repressed
PIS58880.1	30.93872921	31.50523308	30.85581782	30.76849687	30.90485943	30.99562312	0.563079621	0.933	-0.21	PIS58880.1	orf19.3843	Ortholog(s) have protein transmembrane transporter activity and role in filamentous growth, post-translational protein targeting to membrane, translocation
PIS54709.1	25.16352646	24.48331795	27.91822112	25.58156066	25.95506583	25.39852125	0.78258108	0.951	-0.21	PIS54709.1	PEX2	Ortholog(s) have ubiquitin protein ligase activity
PIS54591.1	33.54326132	33.3203582	33.7388227	33.26902169	33.41000178	33.29309945	0.500599011	0.924	-0.21	PIS54591.1	RPL9B	Ribosomal protein L9; repressed upon phagocytosis by murine macrophages; repressed by nitric oxide; protein levels decrease in stationary phase; Hap43-induced; Spider biofilm repressed
PIS51773.1	23.91015768	24.96139421	23.058541	24.30563058	23.7017783	23.293225	0.688735514	0.945	-0.21	PIS51773.1	RPN4	C2H2 transcription factor; regulator of proteasome genes; induced by Hap43, Spider biofilm, and within core stress response; null mutants show increased susceptibility to fluconazole and kill macrophages more slowly than wild type
PIS50418.1	30.06210518	29.71701491	29.86750272	29.45687243	29.73177739	29.8271355	0.801950208	0.952	-0.21	PIS50418.1	SBA1	Similar to co-chaperones; induced in high iron; farnesol-, heavy metal (cadmium) stress-induced; protein level decreases in stationary phase cultures; Hap43-repressed
PIS51147.1	25.33141502	24.02055709	25.44209727	24.1877712	24.63975548	25.33316891	0.648034589	0.941	-0.211	PIS51147.1	orf19.3787	Ortholog(s) have chromatin DNA binding, nucleotidase activity
PIS56697.1	25.00906119	25.19035329	24.16584329	24.59401445	24.54627972	24.59037524	0.650344913	0.941	-0.212	PIS56697.1	orf19.1136	Ortholog(s) have AP-2 adaptor complex, clathrin-coated vesicle localization
PIS49701.1	28.91925115	26.88706539	29.25393059	29.24875443	27.43654388	27.73759536	0.759661975	0.95	-0.212	PIS49701.1	orf19.6565	Conserved mitochondrial inner membrane insertase; mediates insertion of mitochondrial- and nuclear-encoded proteins from the matrix into the inner membrane; Spider biofilm repressed

PIS56857.1	28.99373261	29.78989006	28.89231247	29.0186809	28.86901679	29.14756664	0.613323611	0.938	-0.214	PIS56857.1	orf19.4609	Putative dienelactone hydrolase; protein abundance is affected by URA3 expression in the CAI-4 strain background; protein present in exponential and stationary growth phase yeast cultures; rat catheter biofilm repressed
PIS51821.1	26.42364749	25.64041047	26.05809577	26.03792781	26.31035703	25.13178376	0.698958992	0.945	-0.214	PIS51821.1	TOP1	DNA topoisomerase I; required for wild-type growth and for wild-type mouse virulence; sensitive to camptothecin; induced upon adherence to polystyrene; rat catheter biofilm induced
PIS54578.1	29.53528717	29.23155861	29.34333282	29.35557006	28.98649195	29.12723288	0.53989519	0.93	-0.214	PIS54578.1	UTP20	Putative snoRNA-binding protein; S. cerevisiae Utp20 ortholog; likely essential for growth; repressed in core stress response; mutation confers resistance to 5-fluorocytosine (5-FC) and parnafungin
PIS49561.1	27.42717645	26.84729388	27.29083686	26.3339603	26.96303226	27.62417004	0.670878595	0.943	-0.215	PIS49561.1	orf19.3613	Ortholog(s) have RNA polymerase II C-terminal domain phosphoserine binding, RNA polymerase II complex binding, chromatin binding activity
PIS55812.1	27.34339852	27.63720339	26.99746761	26.85625952	27.0682031	27.40546431	0.627334181	0.939	-0.216	PIS55812.1	AHR1	Zn(II)2Cys6 transcription factor; involved in regulation of adhesion genes; involved in white-opaque switch; acts as repressor of START; forms complex with Mcm1; mutant is sensitive to 5-fluorocytosine and lithium chloride
PIS52030.1	22.94948682	24.63037373	23.73828763	23.54374637	22.91175432	24.21585651	0.839191758	0.954	-0.216	PIS52030.1	orf19.1565	Protein of unknown function
PIS58327.1	23.94140747	24.59842755	24.94664143	24.04578232	24.13215062	24.65925367	0.681887785	0.944	-0.216	PIS58327.1	orf19.4907	Putative protein of unknown function; Hap43p-repressed gene; increased transcription is observed upon fluphenazine treatment; possibly transcriptionally regulated by Tac1p; induced by nitric oxide; fungal-specific (no human/murine homolog
PIS54949.1	28.89656465	28.84270785	29.32001988	28.84781281	28.85933977	28.70553706	0.833552937	0.954	-0.216	PIS54949.1	orf19.7357	Ortholog(s) have phosphopantothenate--cysteine ligase activity, role in acetyl-CoA biosynthetic process from pantothenate, coenzyme A biosynthetic process and CoA-synthesizing protein complex, cytoplasm, nucleus localization
PIS48236.1	30.52610732	30.14175291	30.41181105	30.20072208	30.14005861	30.09238506	0.43097101	0.913	-0.216	PIS48236.1	SUI3	Putative translation initiation factor; genes encoding ribosomal subunits, translation factors, and tRNA synthetases are downregulated upon phagocytosis by murine macrophage
PIS48559.1	30.51944914	30.51509085	29.55102912	29.38945089	30.33960221	30.20485305	0.839387733	0.954	-0.217	PIS48559.1	RPL37B	Ribosomal protein L37; Hap43-induced; Spider biofilm repressed

PIS52165.1	28.63014801	29.24128103	27.90303805	27.73479281	28.72431283	28.66498972	0.593206419	0.936	-0.217	PIS52165.1	RPT5	26S proteasome regulatory subunit; transcript regulated by Nrg1 and Mig1; regulated by Gcn2 and Gcn4; protein level decreases in stationary phase
PIS58842.1	27.84265377	26.57530369	27.57895267	27.03444905	27.10199517	27.2108244	0.578618907	0.934	-0.217	PIS58842.1	SEC15	Exocyst subunit, involved in polarized growth; cellular bud tip-associated protein; interacts with Ras-related GTPase Rsr1p; ortholog of S. cerevisiae SEC15
PIS58868.1	21.81711271	23.8977694	22.71701058	21.27769877	23.53750234	22.96223505	0.883516243	0.956	-0.218	PIS58868.1	ECM21	Predicted regulator of endcytosis of plasma membrane proteins; fluconazole induced, alkaline induced by Rim101; repressed by caspofungin and in azole-resistant strain overexpressing MDR1; flow model, rat catheter and Spider biofilm induced
PIS58776.1	28.28840018	28.15414435	28.17446312	28.25415489	27.8199999	27.8888814	0.616364909	0.938	-0.218	PIS58776.1	LEU2	Isopropyl malate dehydrogenase; leucine biosynthesis; induced by human whole blood or PMNs; protein level decreases in stationary phase; GlcNAc-induced protein; flow model biofilm repressed
PIS58877.1	28.03908255	28.48328669	27.94827481	28.0980064	27.89888396	27.82000383	0.695768566	0.945	-0.218	PIS58877.1	orf19.2304	Protein similar to S. cerevisiae Gvp36p; transposon mutation affects filamentous growth
PIS55082.1	30.74084178	30.60581018	30.99623562	30.41368252	30.63544267	30.63039297	0.455003341	0.917	-0.221	PIS55082.1	ATM1	Member of MDR subfamily of ABC family; ortholog of S. cerevisiae ABC transporter, Atm1; induced in low iron; induced by nitric oxide independent of Yhb1
PIS50332.1	29.34997872	30.05316164	29.55978354	29.36024239	29.53540397	29.40000459	0.686415687	0.944	-0.222	PIS50332.1	orf19.4932	Ortholog(s) have role in mitochondrial translation and mitochondrion localization
PIS55072.1	25.4550844	26.51047964	25.84036755	25.38317715	25.67811603	26.07452898	0.63581891	0.94	-0.223	PIS55072.1	orf19.2621	Ortholog(s) have U1 snRNP, U2 snRNP, U2-type prespliceosome, U4/U6 x U5 tri-snRNP complex, U5 snRNP, post-mRNA release spliceosomal complex, spliceosomal complex localization
PIS55754.1	25.85690156	26.94698229	25.2092534	25.18213339	25.82345765	26.33705336	0.665892483	0.943	-0.223	PIS55754.1	orf19.3649	Ortholog(s) have adenyl-nucleotide exchange factor activity, role in cytoplasm protein quality control by the ubiquitin-proteasome system and cytosol localization
PIS54759.1	23.51503893	24.11047149	23.67857161	23.35978224	23.7259487	23.54971034	0.583656979	0.935	-0.223	PIS54759.1	POL32	Subunit of DNA polymerase delta, involved in chromosomal DNA replication; required for pathogenesis; null mutant is viable but defective in resistance to DNA damage, has abnormal cells and reduced hyphal growth, and appears avirulent

PIS48484.1	27.58072007	27.9309045	24.8687731	25.6588492	27.21665927	26.83390856	0.769280463	0.95	-0.224	PIS48484.1	orf19.2202	Protein of unknown function; induced by alpha pheromone in SpiderM medium
PIS58987.1	31.60732519	31.23038264	31.74216332	31.38001635	31.31003727	31.21641837	0.414371205	0.909	-0.224	PIS58987.1	orf19.6658	Stationary phase enriched protein; predicted ORF from Assembly 19; removed from Assembly 20; subsequently reinstated in Assembly 21 based on comparative genome analysis
PIS50472.1	29.78000535	29.43994501	29.73114441	29.57946769	29.40669817	29.29047827	0.516913363	0.927	-0.225	PIS50472.1	MES1	Cytoplasmic methionyl-tRNA synthetase; zinc-binding motif; ribosomal subunits, translation factors, tRNA synthetases are downregulated upon phagocytosis by murine macrophage; protein present in exponential and stationary phase yeast
PIS58205.1	31.8591327	31.38477284	32.02678729	31.89574255	31.36522034	31.33575385	0.460634406	0.918	-0.225	PIS58205.1	NTH1	Neutral trehalase; hyphal induction in mutant delayed but not reduced overall; not required for virulence in mice; possible regulatory cAMP-dependent phosphorylation at S10,S213; Hap43-repressed gene; Spider biofilm induced
PIS58593.1	30.23928529	29.62787546	30.13859373	29.60332331	29.65937964	30.0635016	0.574367386	0.934	-0.227	PIS58593.1	ERV25	Component of COPII-coated vesicles; transcript induced upon filamentous growth; rat catheter biofilm repressed
PIS56673.1	24.92330488	24.63245656	25.41298219	24.08222653	25.88800125	24.31839796	0.800949982	0.952	-0.227	PIS56673.1	orf19.6719	Component of the HIR complex, a nucleosome assembly factor involved in chromatin formation; involved in regulation of white-opaque switching, hyphal initiation, nitrogen utilization, and azole sensitivity
PIS51407.1	27.89040961	28.18651429	27.02857087	27.42538929	27.53607947	27.45576686	0.611487997	0.938	-0.229	PIS51407.1	ARP4	Subunit of the NuA4 histone acetyltransferase complex
PIS48740.1	25.23076295	25.96154052	24.00580119	24.48295112	24.7499025	25.27820599	0.647302089	0.941	-0.229	PIS48740.1	HAT2	Putative Hat1-Hat2 histone acetyltransferase complex subunit; role in DNA damage repair and morphogenesis; mutations cause constitutive pseudohyphal growth, caspofungin sensitivity; rat catheter and Spider biofilm repressed
PIS48713.1	25.29453228	25.10932689	23.81309691	24.20830701	24.62578596	24.69470315	0.737251507	0.948	-0.229	PIS48713.1	orf19.7038	Ortholog(s) have phosphatidylinositol-3-phosphate binding activity, role in plasma membrane tubulation, protein targeting to vacuole, retrograde transport, endosome to Golgi and cytoplasm, endosome, nucleus localization
PIS54768.1	29.13106696	29.76203433	28.88427749	28.69217378	28.95126722	29.44643918	0.56502284	0.933	-0.229	PIS54768.1	RET2	Delta subunit of the coatomer complex (COPI); coats Golgi-derived transport vesicles; involved in retrograde transport between Golgi and ER; interacts with Crk1 in the two-hybrid system; Spider biofilm repressed

PIS54471.1	30.01989752	29.83025829	30.24399904	29.70722624	29.76574022	29.92859543	0.621984684	0.939	-0.231	PIS54471.1	orf19.3139	Putative NADP-dependent oxidoreductase; Hap43-repressed; induced by benomyl treatment; oxidative stress-induced via Cap1; rat catheter biofilm repressed
PIS56676.1	25.26988167	25.03341215	24.75288878	25.53963591	24.21916895	24.60477431	0.74461219	0.949	-0.231	PIS56676.1	orf19.4110	Ortholog of C. dubliniensis CD36 : Cd36_20490, C. parapsilosis CDC317 : CPAR2_104180, C. auris B8441 : B9J08_001215 and Candida tenuis NRRL Y-1498 : CANTEDRAFT_116074
PIS56589.1	25.84245493	25.3034653	25.09743693	25.0858382	25.13625447	25.32872037	0.717875946	0.947	-0.231	PIS56589.1	orf19.5755	Ortholog(s) have cyclin-dependent protein serine/threonine kinase regulator activity
PIS58272.1	32.25006644	32.0155419	32.48811315	31.96899799	32.13283984	31.95729058	0.654569704	0.942	-0.232	PIS58272.1	AAT22	Aspartate aminotransferase; nitrogen metabolism; similar but not orthologous to S. cerevisiae Aat2; clade-associated gene expression; protein levels decrease in stationary phase yeast; mutant is viable; flow model biofilm repressed
PIS56650.1	27.81949286	27.93270855	27.95239309	27.78927857	27.62956232	27.58995385	0.783579471	0.951	-0.232	PIS56650.1	orf19.1531	Ortholog(s) have RNA binding activity and role in co-transcriptional mRNA 3'-end processing, cleavage and polyadenylation pathway, mRNA processing, response to DNA damage checkpoint signaling
PIS54563.1	24.49676952	24.97695242	23.80059803	24.26965134	24.17304856	24.13481591	0.548724576	0.931	-0.232	PIS54563.1	SPB4	Putative ATP-dependent RNA helicase; flucytosine repressed; Spider biofilm induced
PIS49476.1	30.33792157	30.37033201	29.94218149	29.98818079	29.9659846	29.99940563	0.590455001	0.936	-0.232	PIS49476.1	TUB1	Alpha-tubulin; gene has intron; complements cold-sensitivity of S. cerevisiae tub1 mutant; C. albicans has single alpha-tubulin gene, whereas S. cerevisiae has two (TUB1, TUB3); farnesol-upregulated in biofilm; sumoylation target
PIS58785.1	27.83861197	27.79248765	27.97077687	27.15697242	27.92328655	27.82128033	0.621368676	0.939	-0.233	PIS58785.1	HNT1	Histidine triad nucleotide-binding protein; protein level decreases in stationary phase cultures
PIS49505.1	27.55182795	28.45095739	27.98697989	28.06860288	28.03628384	27.18604579	0.698299193	0.945	-0.233	PIS49505.1	orf19.5681	Ortholog of C. dubliniensis CD36 : Cd36_50190, C. parapsilosis CDC317 : CPAR2_302680, C. auris B8441 : B9J08_004528 and Candida tenuis NRRL Y-1498 : CANTEDRAFT_109949
PIS51894.1	34.1323868	33.77217544	34.07010357	33.71841699	33.68795836	33.86547232	0.598906691	0.937	-0.234	PIS51894.1	CSP37	Hyphal cell wall protein; role in progression of mouse systemic infection; predicted P-loop, divalent cation binding, N-glycosylation sites; expressed in yeast and hyphae; hyphal downregulated; stationary-phase enriched; GlcNAc-induced

PIS52380.1	29.61852547	29.80336485	29.77470682	29.3187483	29.40291471	29.77256603	0.57612805	0.934	-0.234	PIS52380.1	HTA3	Putative histone H2A; amphotericin B repressed; flucytosine induced; RNA abundance regulated by tyrosol and cell density; Spider biofilm repressed
PIS58819.1	27.3876344	26.39702463	27.80467999	27.63865841	27.21639621	26.03291921	0.659470434	0.942	-0.234	PIS58819.1	orf19.394	Putative kynureninase; predicted role in NAD biosynthesis; Hap43-repressed gene; flow model biofilm induced
PIS49827.1	28.98372532	29.50094923	28.79696039	28.34610255	29.0814169	29.15161795	0.770853014	0.95	-0.234	PIS49827.1	orf19.4368	Ortholog of C. dubliniensis CD36 : Cd36_28890, C. parapsilosis CDC317 : CPAR2_801690, C. auris B8441 : B9J08_004854 and Candida tenuis NRRL Y-1498 : CANTEDRAFT_106471
PIS56921.1	31.60884163	31.72397747	31.64919594	31.42978003	31.38514953	31.46389581	0.589192746	0.935	-0.234	PIS56921.1	SAM2	S-adenosylmethionine synthetase; localizes to surface of hyphae, not yeast cells; alkaline, Hog1-induced; farnesol-downregulated; F-12/CO2 early biofilm induced; Spider biofilm repressed
PIS48704.1	30.92173731	30.800973	30.74612253	30.4896315	30.60587921	30.67146501	0.469170201	0.92	-0.234	PIS48704.1	TRA1	Subunit of the NuA4 histone acetyltransferase complex
PIS50406.1	30.95901923	30.81341723	30.85306661	30.73275024	30.56277995	30.62593002	0.417181786	0.91	-0.235	PIS50406.1	ADE12	Adenylosuccinate synthase; upregulated in biofilm; decreased expression in hyphae vs yeast-form cells; not induced during GCN response, in contrast to S. cerevisiae ADE12, which is induced by Gcn4p; stationary phase-enriched protein
PIS55085.1	28.5002768	28.01804892	27.92309495	28.10096574	27.84058963	27.79423338	0.472303997	0.92	-0.235	PIS55085.1	HRR25	Predicted protein serine/threonine kinase, involved in regulation of response to cell wall and membrane stress; Spider biofilm induced
PIS54894.1	27.53751888	28.09187288	28.25172329	27.94954637	27.49345919	27.73222631	0.71326439	0.946	-0.235	PIS54894.1	MDN1	Putative midasin, a very large dynein-related AAA-type ATPase; induced during the mating process
PIS58933.1	28.38527651	27.28642689	28.44646876	28.09223458	27.55795653	27.7637578	0.596072313	0.936	-0.235	PIS58933.1	orf19.2115	Putative molybdopterin-converting factor; fungal-specific (no human or murine homolog)
PIS48396.1	30.57448928	30.39631068	30.59894307	30.19429661	30.30775056	30.36390473	0.377834605	0.901	-0.235	PIS48396.1	orf19.2485	Ortholog(s) have chromatin binding, promoter-specific chromatin binding, protein-containing complex binding, structural constituent of nuclear pore activity

PIS58392.1	26.70653595	26.04575856	26.80874074	27.03656879	25.4428691	26.37794871	0.648517246	0.941	-0.235	PIS58392.1	orf19.6400	Ortholog(s) have nuclear import signal receptor activity, role in inositol metabolic process, protein import into nucleus and nuclear envelope localization
PIS55684.1	30.13545363	29.65483752	30.4652072	30.10935766	29.75886972	29.6776524	0.446792443	0.916	-0.237	PIS55684.1	EMP70	Protein with a role in endosome-to-vacuole sorting; rat catheter biofilm repressed
PIS49726.1	30.53642047	30.90631642	30.56010009	30.35870867	30.60031256	30.332559	0.751562466	0.949	-0.237	PIS49726.1	FESUR1	Putative ubiquinone reductase; transcriptionally induced by interaction with macrophage; alkaline downregulated; repressed by nitric oxide; clade-associated gene expression; Hap43p-repressed gene
PIS54909.1	28.64881852	29.32539595	28.66859406	28.80541709	28.63820991	28.48733596	0.764887204	0.95	-0.237	PIS54909.1	orf19.1933	Ortholog(s) have role in ER-dependent peroxisome organization, peroxisome organization and endoplasmic reticulum, peroxisomal membrane, peroxisome localization
PIS51139.1	30.04353531	31.58412315	29.62072532	30.51531622	29.80237852	30.22106967	0.614645876	0.938	-0.237	PIS51139.1	PDI1	Putative protein disulfide-isomerase; antigenic in human infection; soluble protein in hyphae; induced by filamentous growth; protein present in exponential and stationary growth phase yeast cultures; flow model biofilm repressed
PIS52330.1	32.25540614	31.85161536	32.23765254	31.83104901	31.9621604	31.8412878	0.386211056	0.903	-0.237	PIS52330.1	RPG1A	Putative translation initiation factor; mutation confers hypersensitivity to roridin A and verrucarin A; repressed upon phagocytosis by murine macrophage; Spider biofilm repressed
PIS49584.1	29.85928911	29.64693131	29.72399274	29.56468503	29.34765865	29.60795982	0.426662123	0.912	-0.237	PIS49584.1	VAC8	Protein involved in vacuolar inheritance; required for hyphal growth; contains armadillo repeats
PIS49564.1	27.23868382	26.82080656	26.5049835	26.4743151	26.75138021	26.62553595	0.433565261	0.913	-0.238	PIS49564.1	GTR1	Putative GTP-binding protein; involved in activation of TOR1C during starvation response; transcript is upregulated in clinical isolates from HIV+ patients with oral candidiasis; (see Locus History Note for Assembly 19 correction)
PIS54668.1	27.69513826	27.75694071	27.04125535	27.01809448	27.11599071	27.64544534	0.731267605	0.948	-0.238	PIS54668.1	orf19.6982	Ortholog(s) have mitochondrial ribosome binding activity and role in mitochondrial translational initiation
PIS55035.1	29.17891174	30.01548527	29.27355176	29.32668744	29.04302266	29.38175683	0.474988102	0.92	-0.239	PIS55035.1	ARO3	3-deoxy-D-arabinoheptulosonate-7-phosphate synthase; aromatic amino acid synthesis; GCN-regulated; feedback-inhibited by phe if expressed in S. cerevisiae; decreased in stationary phase; flow model biofilm repressed

PIS55791.1	30.00126507	29.4484957	29.8916105	29.57350356	29.54109392	29.51012449	0.469759158	0.92	-0.239	PIS55791.1	CDC53	Cullin, a scaffold subunit of the SCF ubiquitin-ligase complexes; depletion leads to increased filamentous growth and premature cell death
PIS52184.1	24.50845588	25.20752097	24.65442059	24.45387793	24.72919845	24.46701824	0.670921025	0.943	-0.24	PIS52184.1	orf19.6707	Predicted vacuolar protein with a calcineurin-like phosphoesterase domain; repressed by alpha pheromone in SpiderM medium
PIS58571.1	26.21829754	25.80230165	26.51610468	26.53619247	26.27739615	25.00432346	0.790119228	0.952	-0.24	PIS58571.1	orf19.782	Ortholog(s) have hydrolase activity, acting on ester bonds, triglyceride lipase activity, role in lipid homeostasis and lipid droplet localization
PIS50323.1	29.96088097	29.00442147	30.40748831	29.40301946	29.55394956	29.69381834	0.743751612	0.949	-0.241	PIS50323.1	BNI1	Formin; role in cytoskeletal organization, cell polarity; role in systemic virulence in mouse; cell-cycle regulated localization to site of polarized growth, bud neck; localizes to Spitzenkorper of hyphae, minor localization at septum
PIS55048.1	29.00648279	27.73253242	29.01704318	28.96446586	28.15764334	27.91196758	0.739057137	0.948	-0.241	PIS55048.1	orf19.2070	Component of the RSC chromatin remodeling complex
PIS58080.1	28.60814638	27.71723684	28.77371776	28.35009713	27.87717082	28.14770601	0.610335424	0.938	-0.241	PIS58080.1	orf19.6132	Has domain(s) with predicted role in cristae formation and MICOS complex, mitochondrial crista junction localization
PIS58480.1	25.81930001	26.06006854	25.2462343	25.51166059	25.39148161	25.49795051	0.543868216	0.93	-0.242	PIS58480.1	CET1	mRNA 5'-triphosphatase; large subunit of mRNA capping enzyme; positively regulates Ceg1 activity; functional homolog of S. cerevisiae Cet1; Cet1 and Cgt1 form a 2:1 complex; flow model biofilm induced
PIS55694.1	30.62897741	30.66768086	30.76348124	30.3882817	30.48626624	30.46020479	0.508658699	0.926	-0.242	PIS55694.1	GUT2	Glycerol-3-phosphate dehydrogenase; Plc1p-regulated; rat catheter biofilm induced; Spider biofilm induced
PIS55636.1	33.18618876	32.90900628	33.59209066	33.14061992	32.72932205	33.09128869	0.56051693	0.932	-0.242	PIS55636.1	POX1-3	Predicted acyl-CoA oxidase; farnesol regulated; stationary phase enriched protein; Spider biofilm induced
PIS58383.1	26.68368605	26.43769682	27.15972788	26.80216052	26.46995418	26.28203372	0.521318741	0.927	-0.242	PIS58383.1	RFC1	Putative DNA replication factor C subunit; ortholog of S. cerviaie Rfc1; likely essential, based on an insertional mutagenesis strategy; rat catheter biofilm repressed

PIS58657.1	23.64330184	24.67362472	24.55618943	25.08871486	23.78275313	23.2748826	0.629217093	0.939	-0.242	PIS58657.1	RIO2	Putative serine kinase with a predicted role in the processing of the 20S pre-rRNA into mature 18S rRNA; null mutants are hypersensitive to caspofungin
PIS48530.1	27.08507428	25.75443653	27.65100092	26.37513447	26.80030277	26.58514593	0.657056352	0.942	-0.243	PIS48530.1	orf19.6860	Phosphatidylinositol synthase, essential protein of lipid metabolism; involved in maintenance of endoplasmic reticulum, cell wall integrity and virulence
PIS54956.1	27.38663921	27.68857955	27.02403517	26.78241074	27.38589578	27.1986641	0.59032129	0.936	-0.244	PIS54956.1	NCR1	Putative vacuolar membrane protein; predicted role in sphingolipid metabolism; transcript regulated by Nrg1 and Mig1; induced by prostaglandins
PIS51695.1	30.09601572	29.96914664	30.39911026	30.09489036	29.76678595	29.86856037	0.584232251	0.935	-0.245	PIS51695.1	orf19.4633	Ortholog(s) have carbonyl reductase (NADPH) activity, diacetyl reductase ((S)-acetoin forming) activity, oxidoreductase activity, serine 3-dehydrogenase activity and role in acetoin metabolic process
PIS51256.1	30.47945316	30.03445485	30.07497739	29.96051918	29.97621175	29.91486757	0.39906273	0.906	-0.246	PIS51256.1	FRP3	Putative ammonium transporter; upregulated in the presence of human neutrophils; fluconazole-downregulated; repressed by nitric oxide; Spider biofilm induced; rat catheter biofilm repressed
PIS49838.1	26.20878122	25.50975161	26.53339901	26.15447927	25.40239293	25.9578807	0.706705535	0.946	-0.246	PIS49838.1	orf19.5575	Putative peripheral peroxisomal membrane peroxin; required for regulating peroxisome size and maintenance; Spider biofilm induced
PIS49531.1	31.01029571	30.71425612	31.23873835	30.68585863	30.69394368	30.84678511	0.419278833	0.91	-0.246	PIS49531.1	SEC24	Protein with a possible role in ER to Golgi transport; induced upon yeast-hyphal switch; sumoylation target; Spider biofilm repressed
PIS56847.1	26.02494503	26.05571165	25.96046773	25.47516556	25.824244	26.00357812	0.72729569	0.947	-0.246	PIS56847.1	TIM10	Predicted protein of the mitochondrial intermembrane space with role in protein import into mitochondrial inner membrane
PIS58577.1	32.92296946	32.72606051	32.97894127	32.75647437	32.5166692	32.61506829	0.432079435	0.913	-0.247	PIS58577.1	APE2	Neutral arginine, alanine, leucine specific metallo-aminopeptidase; purified from cell wall/intracellular fractions; protein repressed during mating; Hog1, farnesol-induced; may be essential (UAU1 method); rat catheter biofilm repressed
PIS51152.1	29.03824622	29.89975279	29.03805469	29.22829883	28.91319644	29.09448909	0.511980639	0.926	-0.247	PIS51152.1	orf19.3213	Protein of unknown function; Spider biofilm induced

PIS56678.1	27.16131481	27.2974833	27.09066567	27.01390268	26.82027137	26.97426072	0.504558324	0.925	-0.247	PIS56678.1	orf19.4107	Exopolyphosphatase, hydrolyzes inorganic polyphosphate (poly P) into Pi residues; redundant with polyphosphatase Phm5p; Spider biofilm repressed
PIS58866.1	31.36942043	31.05874845	31.34511394	31.23247128	30.86271253	30.93703548	0.411595721	0.909	-0.247	PIS58866.1	RPN2	Putative 26S proteasome subunit; transcript regulated by Mig1; caspofungin repressed; regulated by Gcn2 and Gcn4; gene used for strain identification by multilocus sequence typing
PIS59022.1	30.55514456	30.06683574	30.5639873	30.08377949	30.10932813	30.24794998	0.400267991	0.906	-0.248	PIS59022.1	OSM2	Putative mitochondrial fumarate reductase; regulated by Ssn6p, Gcn2p, and Gcn4p; Hog1p-downregulated; stationary phase enriched protein; Hap43p-repressed gene
PIS58894.1	29.77886522	30.10821195	29.48038579	29.23816781	29.59951563	29.77993218	0.432737609	0.913	-0.25	PIS58894.1	ARC1	Putative G4 nucleic acid binding protein; macrophage/pseudohyphal-repressed; protein enriched in stationary phase yeast-form cultures; Spider biofilm repressed
PIS55050.1	27.29038799	26.65782842	26.36658706	26.42652813	26.03924118	27.0991171	0.772372379	0.95	-0.25	PIS55050.1	orf19.2068	Ortholog of C. dubliniensis CD36 : Cd36_15550, C. parapsilosis CDC317 : CPAR2_213190, C. auris B8441 : B9J08_002200 and Candida tenuis NRRL Y-1498 : CANTEDRAFT_119026
PIS48792.1	26.5398147	26.78891677	25.72395215	24.88281704	26.4110362	27.0066293	0.737752834	0.948	-0.251	PIS48792.1	orf19.6681	Protein of unknown function; Spider biofilm induced
PIS51691.1	31.42466539	31.23862207	31.66608147	31.50910125	31.27135169	30.79558097	0.738890667	0.948	-0.251	PIS51691.1	RPS28B	Putative ribosomal protein S28B
PIS54641.1	33.5343395	33.52334789	33.51756309	33.23634024	33.27511987	33.30713142	0.330070026	0.887	-0.252	PIS54641.1	FAS2	Alpha subunit of fatty-acid synthase; required for virulence in mouse systemic infection and rat oropharyngeal infection models; regulated by Efg1; fluconazole-induced; amphotericin B repressed; flow model and Spider biofilm repressed
PIS49470.1	29.99751617	30.48666606	30.21456883	30.09205257	30.01233398	29.83805072	0.584557628	0.935	-0.252	PIS49470.1	orf19.51	Ortholog(s) have RNA binding activity
PIS56535.1	29.64984312	28.92834581	29.02493296	29.16200609	29.16026473	28.52417949	0.504811805	0.925	-0.252	PIS56535.1	orf19.5516	Ortholog(s) have role in SRP-dependent cotranslational protein targeting to membrane and signal recognition particle, endoplasmic reticulum targeting localization

PIS51882.1	27.18151484	25.51515358	26.69018183	26.3042014	26.13930968	26.18632051	0.75331166	0.949	-0.252	PIS51882.1	PLB3	GPI-anchored cell surface phospholipase B; possibly secreted; fungal-specific (no mammalian homolog); induced by Tbf1; fluconazole-induced; possible essential gene (UAU1 method); Spider and flow model biofilm induced
PIS52408.1	23.56719134	26.32482747	23.93942153	23.94190082	24.36415094	24.76768256	0.673166985	0.943	-0.253	PIS52408.1	NTF2	Putative nuclear envelope protein; regulated by Ssn6; rat catheter biofilm repressed
PIS49704.1	27.46226069	27.07574799	27.65507645	27.35442394	27.33823315	26.74073011	0.753177295	0.949	-0.253	PIS49704.1	orf19.6558	Ortholog(s) have GTPase activator activity and cytosol localization
PIS55483.1	30.68124521	30.3927249	30.79224319	30.37011394	30.23274478	30.49999348	0.445752104	0.915	-0.254	PIS55483.1	orf19.4864	Ortholog(s) have acylglycerol lipase activity, role in triglyceride metabolic process and lipid droplet, membrane localization
PIS51857.1	28.51104487	27.96930205	28.89702491	28.67445035	28.289699	27.6467735	0.517967393	0.927	-0.255	PIS51857.1	MNN10	Alpha-1,6-mannosyltransferase involved in biosynthesis and organization of cell wall polysaccharides
PIS51025.1	30.07668932	30.5018708	29.99900933	29.50477745	30.17731157	30.12921071	0.54321777	0.93	-0.255	PIS51025.1	orf19.2964	Component of the RSC chromatin remodeling complex
PIS55459.1	27.80703964	27.62998682	27.3658396	27.37130068	27.1803468	27.48557566	0.634103797	0.94	-0.255	PIS55459.1	orf19.7131	Butyrobetaine dioxygenase, the fourth enzyme of the carnitine biosynthesis pathway
PIS56633.1	30.85025262	31.73507003	30.76419223	31.11741617	30.48031562	30.98399972	0.724311467	0.947	-0.256	PIS56633.1	ADH5	Putative alcohol dehydrogenase; regulated by white-opaque switch; fluconazole-induced; antigenic in murine infection; regulated by Nrg1, Tup1; Hap43, macrophage repressed, flow model biofilm induced; Spider biofilm induced
PIS51738.1	24.1456216	25.11431241	24.87600413	25.04543623	24.04501267	24.27659334	0.733734821	0.948	-0.256	PIS51738.1	VPS24	Protein similar to S. cerevisiae Vps24p, which is a member of the ESCRT III protein sorting complex; downregulated upon adherence to polystyrene
PIS48555.1	28.09224953	27.73543935	28.08271479	27.58963364	27.92815505	27.62025899	0.70178639	0.946	-0.257	PIS48555.1	GIN4	Autophosphorylated kinase; role in pseudohyphal-hyphal switch and cytokinesis; phosphorylates Cdc11p on S395; necessary for septin ring within germ tube but not for septin band at mother cell junction; physically associates with septins

PIS58451.1	26.81674681	26.69368593	26.78859887	26.38472917	26.48749233	26.65495258	0.726515407	0.947	-0.257	PIS58451.1	orf19.3684	Putative oxidoreductase; Spider biofilm induced
PIS51374.1	26.77334448	26.2585922	27.55942231	26.64059956	26.49874566	26.6804572	0.557059563	0.932	-0.257	PIS51374.1	SPO72	Protein described as similar to S. cerevisiae sporulation protein; ortholog of S. cerevisiae Atg2, an autophagic vesicle formation protein; up-regulation associated with azole resistance; Spider biofilm induced
PIS48654.1	29.055537	28.24209109	29.61720569	28.72523901	28.73193462	28.68428409	0.600861296	0.937	-0.258	PIS48654.1	orf19.3290	Plasma membrane-localized protein; repressed by nitric oxide; Hap43p-repressed gene
PIS52064.1	26.99193767	27.54942604	26.70138135	26.11808351	27.09817172	27.25292743	0.54239964	0.93	-0.258	PIS52064.1	orf19.6250	Ortholog(s) have eukaryotic initiation factor 4G binding, mRNA binding activity, role in P-body assembly, negative regulation of translational initiation, stress granule assembly and P-body, cytoplasmic stress granule localization
PIS48680.1	32.10285819	32.72534109	31.38286913	30.83859281	32.32978882	32.26834184	0.67775613	0.944	-0.258	PIS48680.1	RPL32	Component of the large (60S) ribosomal subunit; Spider biofilm repressed
PIS51644.1	26.33544083	26.11967946	26.28862256	26.67488443	25.48903055	25.80333798	0.648774457	0.941	-0.259	PIS51644.1	MOB2	Mob1/phocein domain protein of RAM signaling network; cell wall integrity; role in cell separation, cortical actin polarization; required for hyphal growth; phosphorylated by Cdc28 on hyphal induction; activates Cbk1; mRNA binds She3
PIS52044.1	29.21932484	28.75663724	29.88646914	29.18486877	29.06604958	28.8345661	0.434643053	0.913	-0.259	PIS52044.1	orf19.1545	Ortholog(s) have structural constituent of ribosome activity and mitochondrial small ribosomal subunit localization
PIS50469.1	29.37234748	30.10788642	29.39502509	29.39765392	29.1815657	29.51997254	0.670869245	0.943	-0.259	PIS50469.1	SRP101	Signal recognition particle (SRP) receptor alpha subunit; involved in SRP-dependent protein targeting; rat catheter biofilm repressed
PIS48611.1	24.57044461	24.58200863	25.19413741	24.87763423	24.23876921	24.45220108	0.598176008	0.936	-0.259	PIS48611.1	YDC1	Alkaline dihydroceramidase; involved in sphingolipid metabolism; Mob2-dependent hyphal regulation; transcript is regulated by Nrg1 and Mig1; Hap43-repressed
PIS58157.1	25.89329337	25.38536088	24.10432515	24.74695287	24.93792374	24.91745062	0.772029922	0.95	-0.26	PIS58157.1	HNT2	Putative dinucleoside triphosphate hydrolase; induced upon low-level peroxide stress

PIS52178.1	30.70778577	31.34627732	30.70732866	30.54139159	30.64762895	30.79283703	0.528207889	0.928	-0.26	PIS52178.1	SHM1	Mitochondrial serine hydroxymethyltransferase; complements the glycine auxotrophy of an S. cerevisiae shm1 null shm2 null gly1-1 triple mutant; protein present in exponential and stationary growth phase yeast cultures
PIS49461.1	28.11710887	27.38817006	26.89725889	27.30650486	27.16320726	27.14863916	0.599899363	0.937	-0.261	PIS49461.1	orf19.827	Ortholog of C. dubliniensis CD36 : Cd36_18620, C. parapsilosis CDC317 : CPAR2_212480, C. auris B8441 : B9J08_004484 and Candida tenuis NRRL Y-1498 : CANTEDRAFT_115371
PIS50451.1	27.60044926	27.75581697	26.95898403	27.34905697	26.9626549	27.21348847	0.657120941	0.942	-0.263	PIS50451.1	COX6	Putative cytochrome c oxidase; flucytosine induced
PIS58315.1	24.61910668	25.45742625	24.51079159	24.51090632	24.47395793	24.81239737	0.585109352	0.935	-0.263	PIS58315.1	orf19.3887	Ortholog of S. cerevisiae : YML108W, C. glabrata CBS138 : CAGL0J06666g, C. dubliniensis CD36 : Cd36_31830, C. parapsilosis CDC317 : CPAR2_204870 and C. auris B8441 : B9J08_000809
PIS54659.1	23.88222186	24.03604602	24.51802053	23.25645443	23.97635996	24.41569586	0.652220925	0.942	-0.263	PIS54659.1	orf19.6604	Ortholog of S. cerevisiae Pba1 that is involved in 20S proteasome assembly; upregulated in a cyr1 null mutant; contains a 5' UTR intron
PIS56914.1	27.29038722	28.36834552	27.45532574	27.15822336	27.61355733	27.55044363	0.543305193	0.93	-0.264	PIS56914.1	orf19.3205	Mitochondrial ribosomal protein of the large subunit; rat catheter biofilm induced
PIS58566.1	26.52853524	27.13854804	26.87543249	26.18488875	27.37226383	26.19304285	0.679083872	0.944	-0.264	PIS58566.1	orf19.4611	Ortholog(s) have ribose phosphate diphosphokinase activity, role in 5-phosphoribose 1-diphosphate biosynthetic process, fungal-type cell wall organization and ribose phosphate diphosphokinase complex localization
PIS58774.1	28.16186734	28.49422985	28.19181203	27.69152717	28.12559945	28.23826375	0.428059398	0.912	-0.264	PIS58774.1	orf19.7078	Ortholog of S. cerevisiae : YCL012C, C. dubliniensis CD36 : Cd36_65270, C. parapsilosis CDC317 : CPAR2_805430, C. auris B8441 : B9J08_000227 and Candida tenuis NRRL Y-1498 : CANTEDRAFT_96597
PIS52448.1	27.09298011	26.08810445	27.20716786	26.78035709	26.53423611	26.27773597	0.714987244	0.947	-0.265	PIS52448.1	orf19.3215	Putative plasma membrane protein; in S. cerevisiae it is localized to the cell bud and mating projection membrane; repressed by alpha pheromone in SpiderM medium
PIS49822.1	28.45832561	28.18986542	28.72599276	28.0799016	28.31459989	28.18331221	0.371922389	0.899	-0.265	PIS49822.1	orf19.4380.1	Ortholog(s) have role in mitochondrial genome maintenance and mitochondrial inner membrane localization

PIS49481.1	30.24684887	29.8824393	30.04796105	29.71568481	29.42814934	30.23518844	0.521690249	0.927	-0.266	PIS49481.1	ELF1	Putative mRNA export protein; Walker A and B (ATP/GTP binding) motifs; required for wild-type morphology, growth; expressed in hyphal, pseudohyphal, and yeast form; Hap43-induced; Spider and flow model biofilm induced
PIS58354.1	29.83751175	29.51606326	29.84440006	30.09635655	28.94547602	29.35684899	0.501716543	0.925	-0.266	PIS58354.1	orf19.2175	Mitochondrial apoptosis-inducing factor; induced by nitric oxide; Spider biofilm induced; rat catheter biofilm repressed
PIS49806.1	26.3850422	26.22166526	25.80964909	25.91949085	25.67745853	26.01918818	0.450296463	0.916	-0.267	PIS49806.1	GLG2	Putative self-glucosylating initiator of glycogen synthesis; expression regulated upon white-opaque switch; hypha-induced; Spider biofilm induced
PIS48435.1	32.81361421	33.17009692	32.8732938	32.78205013	32.59797193	32.67509378	0.377835575	0.901	-0.267	PIS48435.1	LSC2	Putative succinate-CoA ligase beta subunit; regulated by Mig1, Tup1; induced in high iron; protein present in exponential and stationary growth phase yeast cells; Spider biofilm repressed
PIS51551.1	27.72084341	26.99832926	27.43397442	27.01002242	26.84060225	27.50129047	0.444427455	0.915	-0.267	PIS51551.1	orf19.3583	Ortholog(s) have role in protein export from nucleus, ribosomal small subunit export from nucleus and cytosol, nucleus localization
PIS51643.1	28.8590626	26.90056887	28.9808287	28.03157624	28.08305935	27.82499768	0.668706867	0.943	-0.267	PIS51643.1	PSD1	Phosphatidylserine decarboxylase; involved in phosphatidylethanolamine synthesis; Spider biofilm repressed
PIS58716.1	32.19007424	31.8427604	32.1408904	31.73876169	31.82000076	31.81065045	0.344381049	0.891	-0.268	PIS58716.1	CDR1	Multidrug transporter of ABC superfamily; transports phospholipids in an in-to-out direction; induced by beta-estradiol, progesterone, corticosteroid, or cholesterol; Spider biofilm induced
PIS51642.1	25.89071851	25.01490014	25.79061159	26.42499253	24.21145058	25.25250677	0.823842072	0.953	-0.269	PIS51642.1	APC1	Putative Anaphase-Promoting Complex/Cyclosome subunit; essential for growth; periodic mRNA expression, peak at cell-cycle S/G2 phase
PIS51834.1	29.41357608	29.51727997	29.00433516	28.33424092	29.35969558	29.43432443	0.631153921	0.94	-0.269	PIS51834.1	PRE10	Alpha7 (C8) subunit of the 20S proteasome; multiple phosphorylated residues; transcript induced upon filamentous growth; Spider biofilm repressed
PIS56884.1	23.50478269	25.35577461	24.38311552	24.68538269	24.06303202	23.68690077	0.56333417	0.933	-0.269	PIS56884.1	SPB1	Putative AdoMet-dependent methyltransferase; Hap43-induced; repressed by prostaglandins; possibly essential gene, disruptants not obtained by UAU1 method; Spider biofilm induced

PIS48472.1	30.32649589	29.81235738	30.43317049	30.0443301	29.87787626	29.84042899	0.510961958	0.926	-0.27	PIS48472.1	SEC61	ER protein-translocation complex subunit; essential; 10 predicted transmembrane regions; chimeric mutant partially functionally complements S. cerevisiae sec61 defects; Spider biofilm repressed
PIS48361.1	28.79918104	28.6288379	28.74145858	28.48370934	28.46553896	28.39973772	0.702864172	0.946	-0.273	PIS48361.1	orf19.246	Predicted metalloendopeptidase; Spider biofilm induced
PIS48507.1	31.71361295	31.5834541	32.00280326	31.54770932	31.46223401	31.4702929	0.33213959	0.887	-0.273	PIS48507.1	PYC2	Putative pyruvate carboxylase; binds biotin cofactor; repressed by Ssk1 response regulator, by benomyl treatment, or in an azole-resistant strain overexpressing MDR1; stationary phase enriched protein; flow model biofilm repressed
PIS56837.1	23.90166213	26.06452191	23.87004054	24.07244858	24.56091674	24.3796259	0.644438583	0.941	-0.274	PIS56837.1	orf19.2769	Putative protease B inhibitor; hyphal-induced expression; Cyr1p- and Ras1p-repressed
PIS55566.1	24.62088413	24.72970137	24.80325781	24.55046933	24.33945823	24.44090523	0.593784075	0.936	-0.274	PIS55566.1	orf19.4306	Ortholog(s) have methylthioribulose 1-phosphate dehydratase activity and role in L-methionine salvage from methylthioadenosine
PIS49527.1	31.85769233	31.94137646	31.78619859	31.78427666	31.45093538	31.52685318	0.338156198	0.889	-0.274	PIS49527.1	ZWF1	Glucose-6-phosphate dehydrogenase; antigenic in mice; activity induced by O2 or oxidizing agents H2O2, menadione, macrophage; caspofungin repressed; induced in core stress response; regulated by Gcn2, Gcn4; rat catheter biofilm
PIS51985.1	27.76363311	27.730311	27.76629516	27.02066583	27.85406266	27.56164931	0.639237629	0.94	-0.275	PIS51985.1	orf19.3367	Ortholog(s) have structural constituent of ribosome activity and mitochondrial large ribosomal subunit localization
PIS56664.1	29.94822446	29.58062435	30.1588576	29.78684073	29.45500654	29.61779957	0.444414717	0.915	-0.276	PIS56664.1	NUO2	NADH-ubiquinone oxidoreductase subunit; Hap43p-repressed gene; repressed by nitric oxide; identified in detergent-resistant membrane fraction (possible lipid raft component)
PIS54740.1	28.86230045	28.16762547	29.08227164	28.48685657	28.48509641	28.31144729	0.828549669	0.954	-0.276	PIS54740.1	orf19.2875	Protein involved in cell cycle regulation; ortholog of S. pombe SPAC1071.09c DNAJ domain protein; Hap43-induced gene
PIS50513.1	29.20727442	30.73193162	29.84233199	29.28383255	29.62051036	30.04966891	0.709511947	0.946	-0.276	PIS50513.1	orf19.6239	Putative serine/threonine protein kinase, involved in control of filamentous growth; possibly an essential gene, disruptants not obtained by UAU1 method

PIS51803.1	28.6006313	29.77948531	28.05524669	27.63021528	28.88754469	29.08922772	0.814632473	0.953	-0.276	PIS51803.1	ZPR1	Protein with putative zinc finger; regulated by Gcn4p; repressed in response to amino acid starvation (3-aminotriazole treatment); upregulation correlates with clinical development of fluconazole resistance
PIS54517.1	30.14458624	30.87515418	30.03179779	29.72844211	30.09463613	30.3980638	0.589245594	0.936	-0.277	PIS54517.1	EGD1	Putative GAL4 DNA-binding enhancer protein; soluble protein in hyphae; biofilm induced; macrophage/pseudohyphal-induced; equal level of protein in exponential and stationary growth phase yeast cultures; Spider biofilm repressed
PIS51967.1	23.26659978	26.10099975	23.08788198	23.78014072	25.23727725	22.60313612	0.754192788	0.949	-0.278	PIS51967.1	orf19.5345	Putative ubiquitin-like polyubiquitin-binding protein; induced by nitric oxide independent of Yhb1; Spider biofilm repressed
PIS54629.1	26.41681581	26.88845159	24.78219791	26.98016135	25.86668403	24.40646496	0.695382478	0.945	-0.278	PIS54629.1	PRE3	Putative beta-1 proteasome subunit; macrophage-induced protein; regulated by Gcn2p and Gcn4p; GlcNAc-induced protein
PIS51393.1	22.37809697	21.48456948	22.30725505	22.32482204	21.62000623	21.38922248	0.656261566	0.942	-0.279	PIS51393.1	orf19.2397	Predicted membrane transporter, involved in biotin import; member of the anion:cation symporter (ACS) family, major facilitator superfamily (MFS); biotin-dependent transcription regulated by Vhr1p; amphotericin B, caspofungin repressed
PIS48550.1	27.81171664	27.1508711	27.96075555	27.43699656	27.03585969	27.61088062	0.46867091	0.919	-0.28	PIS48550.1	APM1	Ortholog of S. cerevisiae/S. pombe Apm1; a clathrin-associated protein complex (AP-1) subunit; phosphorylated protein; Tn mutation affects filamentous growth; Spider biofilm repressed
PIS58867.1	24.00820802	24.7698584	24.31291787	24.37539121	24.39789115	23.47881719	0.788153729	0.951	-0.28	PIS58867.1	LCB4	Putative sphingosine kinase; Tac1p-regulated expression; rat catheter biofilm induced
PIS56591.1	27.2411955	26.22246888	27.79480989	26.92647171	26.3906124	27.09788312	0.618098956	0.938	-0.281	PIS56591.1	orf19.2214	Ortholog(s) have structural constituent of ribosome activity and mitochondrial large ribosomal subunit localization
PIS55661.1	32.18432041	31.90542256	32.30348277	31.89099813	31.67621485	31.97993202	0.399480542	0.906	-0.282	PIS55661.1	CDC60	Cytosolic leucyl tRNA synthetase; conserved amino acid and ATP binding class I signature, tRNA binding, proofreading motifs; likely essential for growth; interacts with benzoxaborole antifungals; present in exponential and stationary phase
PIS49557.1	28.47534946	28.69027917	28.35740177	28.16899277	28.17496468	28.32892799	0.446378636	0.916	-0.283	PIS49557.1	YPT72	Vacuolar Rab small monomeric GTPase involved in vacuolar biogenesis; involved in filamentous growth and virulence

PIS52224.1	24.23147428	23.82283269	24.56777807	24.57145711	23.5261505	23.67178953	0.519789832	0.927	-0.284	PIS52224.1	DAC1	N-acetylglucosamine-6-phosphate (GlcNAcP) deacetylase; N-acetylglucosamine utilization; required for wild-type hyphal growth and virulence in mouse systemic infection; gene and protein are GlcNAc-induced; Spider biofilm induced
PIS58279.1	29.44053831	29.46009338	29.47743964	29.25428891	29.1884497	29.08256752	0.510730554	0.926	-0.284	PIS58279.1	orf19.4346	Ortholog(s) have protein-membrane adaptor activity and role in COPII vesicle coating, macroautophagy, protein localization to endoplasmic reticulum exit site
PIS58855.1	25.51366493	23.95977487	25.3724744	25.23802704	23.46767061	25.28824751	0.770741864	0.95	-0.284	PIS58855.1	RRP42	Putative exosome non-catalytic core component; involved in 3'-5' RNA processing; rat catheter biofilm induced
PIS52280.1	31.49637927	31.38941286	31.64404924	31.08705089	31.2574335	31.33151112	0.293135261	0.873	-0.285	PIS52280.1	GCD11	Gamma subunit of translation initiation factor eIF2; involved in identification of the start codon; likely essential for growth, based on an insertional mutagenesis strategy; Spider biofilm repressed
PIS54748.1	24.43910853	26.59940911	24.11091609	24.8969945	24.79384928	24.5972765	0.719901673	0.947	-0.287	PIS54748.1	orf19.446.1	Protein with a NADH-ubiquinone oxidoreductase B18 subunit domain; gene has intron
PIS52024.1	27.63364416	28.46041168	28.17562392	27.53030253	27.78866793	28.08652442	0.483055152	0.922	-0.288	PIS52024.1	orf19.3572	Ortholog of C. dubliniensis CD36 : Cd36_19880, C. auris B8441 : B9J08_003635, Candida tenuis NRRL Y-1498 : CANTEDRAFT_117672 and Debaryomyces hansenii CBS767 : DEHA2D11154g
PIS48625.1	27.90887615	27.04336427	27.74665905	26.93365501	27.70603225	27.19401181	0.598117483	0.936	-0.288	PIS48625.1	orf19.835	Ortholog(s) have role in chromatin remodeling and Ino80 complex, cytosol, nucleus localization
PIS51651.1	33.57424242	33.52257884	33.56753073	32.86432636	33.48725772	33.44934232	0.634404875	0.94	-0.288	PIS51651.1	RPS16A	Putative 40S ribosomal subunit; macrophage/pseudohyphal-induced after 16 h; Spider biofilm repressed
PIS51181.1	29.04305245	28.19003264	29.24555858	29.07609158	28.25359369	28.28265625	0.551345551	0.931	-0.289	PIS51181.1	MSS51	Putative mRNA maturation factor; fungal-specific (no human or murine homolog)
PIS56769.1	25.73496633	25.2048554	23.46437761	24.64474658	25.66177917	23.23002572	0.741323164	0.948	-0.289	PIS56769.1	orf19.954	Putative DnaJ-like chaperone; Hap43-repressed gene

PIS58044.1	29.53845087	29.81610776	29.68632304	29.0738103	29.18545801	29.91311269	0.630519733	0.94	-0.29	PIS58044.1	orf19.3900	Ortholog(s) have role in mitochondrial fusion and mitochondrial outer membrane localization
PIS56870.1	29.42853385	30.83106373	28.50189278	28.74738062	29.38453007	29.75778939	0.619822889	0.939	-0.291	PIS56870.1	ABP1	Ortholog of S. cerevisiae Abp1; actin-binding protein of the cortical actin cytoskeleton; caspofungin induced; protein only detected in stationary phase yeast-form cultures; Spider biofilm repressed
PIS55461.1	26.06872419	26.86407453	25.64627095	25.87263298	25.73126926	26.10056549	0.853567498	0.955	-0.292	PIS55461.1	orf19.5168	Ortholog(s) have unfolded protein binding activity, role in maturation of SSU-rRNA, ribosomal small subunit biogenesis and cytoplasm localization
PIS56716.1	30.08667285	30.02915485	29.89610135	29.74919693	29.8174729	29.5699126	0.331167692	0.887	-0.292	PIS56716.1	RPO21	RNA polymerase II; ortholog of S. cerevisiae Rpo21, transposon mutation affects filamentous growth; flow model biofilm repressed
PIS51646.1	27.19806309	26.54565859	26.64811043	26.00517578	26.61328041	26.8981403	0.672238537	0.943	-0.292	PIS51646.1	SNF7	ESCRT III complex protein; role in proteolytic activation of Rim101 and Rim8 processing/activation; separable roles in RIM101 pathway and in transport from MVB to vacuole; involved in echinocandin and azole sensitivity
PIS55061.1	22.61394058	22.83137094	22.58073635	22.18603163	22.53877109	22.42132891	0.615277693	0.938	-0.293	PIS55061.1	SAS10	Putative U3-containing small subunit processome complex subunit; Hap43p-induced gene; mutation confers resistance to 5-fluorocytosine (5-FC); repressed upon high-level peroxide stress
PIS56763.1	29.46539022	29.69684579	29.3400996	29.11307791	29.54860105	28.95864534	0.417458514	0.91	-0.294	PIS56763.1	BDF1	Essential chromatin-binding bromodomain protein; repressed upon adherence to polystyrene; reduced mRNA abundance detected in null mutant; macrophage/pseudohyphal-repressed
PIS55573.1	28.80137976	29.55488021	29.02210617	29.25395968	28.72540531	28.51756944	0.438470087	0.914	-0.294	PIS55573.1	STV1	Predicted subunit a of vacuolar proton-translocating ATPase V0 domain, Golgi isoform
PIS51247.1	27.76224231	27.493445	28.31259656	27.86324089	27.5564894	27.26502845	0.411237392	0.908	-0.295	PIS51247.1	orf19.2008	Ortholog(s) have S-methyl-5-thioribose-1-phosphate isomerase activity and role in L-methionine salvage from methylthioadenosine
PIS55043.1	22.36552179	24.01781091	24.99101063	23.84155704	23.32544824	23.32150299	0.725845796	0.947	-0.295	PIS55043.1	orf19.2090	Ortholog of S. cerevisiae Ecm16, an essential DEAH-box ATP-dependent RNA helicase specific to the U3 snoRNP required for 18S rRNA synthesis; Hap43-induced; Spider biofilm induced

PIS48523.1	29.28470877	28.91383468	29.84900385	29.55329581	28.75531589	28.85399287	0.392620778	0.904	-0.295	PIS48523.1	orf19.3792	Ortholog(s) have RNA binding, chromatin binding, mRNA binding activity
PIS54824.1	32.26074452	32.21991015	32.96008643	32.42290381	32.06285742	32.07041485	0.520702608	0.927	-0.295	PIS54824.1	orf19.6306	Trimethylaminobutyraldehyde dehydrogenase, the third enzyme of the carnitine biosynthesis pathway
PIS48762.1	27.98026602	28.93311482	27.67317889	27.5607092	27.88391211	28.25633102	0.640393248	0.941	-0.295	PIS48762.1	orf19.6461	Ortholog(s) have unfolded protein binding activity, role in mitochondrial cytochrome c oxidase assembly, negative regulation of programmed cell death and mitochondrial inner membrane localization
PIS51813.1	28.26587232	28.65872568	28.50218284	28.2849055	28.16514958	28.09303728	0.423196934	0.911	-0.295	PIS51813.1	TAF12L	Non-essential component of the SAGA complex, involved in transcription regulation
PIS54587.1	33.65185187	33.7411387	33.63107756	33.39967341	33.33756774	33.39808506	0.348369319	0.893	-0.296	PIS54587.1	KGD1	Putative 2-oxoglutarate dehydrogenase; regulated by Efg1 under yeast but not hyphal growth conditions; transcript induced in an RHE model of oral candidiasis; stationary phase enriched protein; Hap43-repressed; rat catheter biofilm induced
PIS49734.1	31.28883041	32.98025992	30.22767853	31.17673703	31.07929795	31.35139234	0.792708446	0.952	-0.296	PIS49734.1	RPS12	Acidic ribosomal protein S12; regulated by Gcn4, activated by Tbf1; repressed by amino acid starvation (3-AT); protein abundance is affected by URA3 expression in CAI-4 strain background; sumoylation target; Spider biofilm repressed
PIS58758.1	24.93691446	24.97477019	24.13194926	24.3681542	24.33487512	24.4508102	0.476009417	0.921	-0.297	PIS58758.1	orf19.1643	Ortholog(s) have serine-type endopeptidase activity, role in regulation of mitochondrion organization, signal peptide processing and mitochondrial inner membrane localization
PIS58343.1	24.74699947	26.36404195	24.92150031	24.27213274	25.25572537	25.61281076	0.631937467	0.94	-0.297	PIS58343.1	orf19.7086	Ortholog(s) have nuclear import signal receptor activity, role in NLS-bearing protein import into nucleus, protein import into nucleus and cytoplasm, nucleus localization
PIS51194.1	27.03508324	26.72748024	27.51194844	27.15801882	26.57268405	26.64883831	0.715167784	0.947	-0.298	PIS51194.1	AHA1	Putative Hsp90p co-chaperone; Hap43-repressed; heavy metal (cadmium) stress-induced; oxidative stress-induced via Cap1; rat catheter biofilm induced; flow model biofilm repressed
PIS56614.1	28.05118069	27.67568449	28.32647832	27.67022199	27.71875522	27.7667039	0.468230205	0.919	-0.299	PIS56614.1	MSC7	S. cerevisiae ortholog Mcs7 has a role in reciprocal meiotic recombination; Spider biofilm repressed

PIS58515.1	24.57151101	25.0512553	23.21458762	23.4428521	24.28855764	24.2059067	0.590524608	0.936	-0.3	PIS58515.1	HGT13	Predicted sugar transporter, involved in glycerol utilization; member of the major facilitator superfamily; 12 transmembrane; gene has intron; oxidative stress-induced via Cap1p; expressed in rich medium, 2% glucose
PIS54548.1	29.69735309	29.54839919	29.91621031	29.69797526	29.32741298	29.23127601	0.5158407	0.927	-0.302	PIS54548.1	CDC12	Septin; essential for viability; forms ring at sites of cell division and also forms filaments in mature chlamydospore; filamentous growth induced; regulated by Nrg1, Tup1, tyrosol and cell density; rat catheter biofilm repressed
PIS52316.1	26.2984129	27.72289375	25.43739328	26.06293056	26.19851214	26.290242	0.657177418	0.942	-0.302	PIS52316.1	orf19.5235	Putative mitochondrial ribosomal protein of the large subunit; Hap43-induced; mutants are viable; protein level decreases in stationary phase
PIS55494.1	23.02676031	24.47287144	23.62489393	23.22932088	23.4775642	23.50747553	0.596856582	0.936	-0.303	PIS55494.1	HAP2	CCAAT-binding transcription factor; regulates low-iron induction of FRP1; in these conditions CBF comprises Hap43 and probably Hap2 and Hap3; possibly essential, disruptants not obtained by UAU1 method; Cap1-dependent induction in low iron
PIS52267.1	24.69105324	24.7268661	23.52110443	22.76782393	24.90106177	24.35847275	0.591656981	0.936	-0.304	PIS52267.1	ARG2	Putative enzyme of arginine biosynthesis; transcription of genes of arginine biosynthesis pathway, except for ARG2, is induced upon phagocytosis by macrophage
PIS51310.1	27.72505074	27.62154055	28.16609611	27.47096331	27.14226667	27.98384348	0.417759301	0.91	-0.305	PIS51310.1	CCR4	Component of the Ccr4-Pop2 mRNA deadenylase; transposon mutation affects filamentous growth
PIS48347.1	27.21995903	28.49666971	27.49386924	27.87174048	26.96800446	27.45340534	0.733134972	0.948	-0.306	PIS48347.1	orf19.1247	Has domain(s) with predicted mitochondrion autophagosome adaptor activity and role in mitophagy
PIS49729.1	27.73405456	26.96054706	26.44147581	27.096233	26.6963426	26.42421467	0.432813675	0.913	-0.306	PIS49729.1	orf19.6790	Ortholog(s) have mRNA binding activity, role in 3'-UTR-mediated mRNA destabilization, mitochondrion organization and P-body, cytoplasmic stress granule, perinuclear region of cytoplasm localization
PIS48340.1	28.41177024	27.04709021	28.69751126	27.94438329	27.78782838	27.50689428	0.435649552	0.913	-0.306	PIS48340.1	RHO3	Putative Rho family GTPase; possible substrate of protein farnesyltransferase and geranylgeranyltransferase type I; greater transcription in hyphal form than yeast form; plasma membrane-localized
PIS49784.1	27.39228522	29.22096082	26.99127503	26.77503584	27.97164026	27.93666829	0.70438032	0.946	-0.307	PIS49784.1	ABD1	SAM-dependent RNA methyltransferase; methylates mRNA 5' cap; binds phosphorylated RNA Pol II C-term domain peptide; does not bind mRNA TPase and mRNA GTase (Cet1,Cgt1); functional homolog of S. cerevisiae Abd1; rat catheter biofilm induced

PIS49600.1	25.59258104	25.06695649	25.16492895	24.51137262	25.23299248	25.15873955	0.611905013	0.938	-0.307	PIS49600.1	orf19.7397	Ortholog(s) have role in negative regulation of transcription by RNA polymerase II, positive regulation of transcription by RNA polymerase II, regulation of fungal-type cell wall organization and chromatin, nucleolus localization
PIS56907.1	29.90473023	29.25266248	28.93586797	29.05814575	29.09222052	29.02317756	0.321952607	0.884	-0.307	PIS56907.1	PAP1	Poly(A) polymerase, likely involved in mRNA polyadenylation; PAP is inhibited by parnafungin antifungals; non-sex gene located within the MTLa mating-type-like locus
PIS52029.1	27.78749475	27.60254467	27.69919574	27.41114707	27.54608846	27.21018506	0.52964795	0.928	-0.307	PIS52029.1	UTP21	Putative U3 snoRNP protein; Hap43-induce; physically interacts with TAP-tagged Nop1; Spider biofilm induced
PIS55644.1	26.61076727	27.09131757	26.76282961	26.63461272	26.28253295	26.62499116	0.500058303	0.924	-0.308	PIS55644.1	TCC1	Putative transcription factor/corepressor; regulation of filamentation and virulence; interacts with Tup1; regulates hypha-specific gene expression; contains 4 tetratricopeptide repeat (TPR) motifs; flucytosine repressed; Tbp1-induced
PIS54839.1	28.69211044	28.9636622	28.72205479	27.77696399	28.79711148	28.8759236	0.67805729	0.944	-0.309	PIS54839.1	orf19.1664	Protein of unknown function; expression downregulated in an ssr1 null mutant
PIS54973.1	24.58243905	25.87461185	24.38760581	24.78915383	24.46358441	24.66071937	0.671584673	0.943	-0.31	PIS54973.1	PNC1	Putative nicotinamidase, involved in NAD salvage pathway; decreased transcription is observed in an azole-resistant strain that overexpresses MDR1
PIS58564.1	31.30628126	31.19420757	31.84405733	31.06109189	31.26619581	31.08414128	0.427332599	0.912	-0.311	PIS58564.1	CAT2	Major carnitine acetyl transferase; intracellular acetyl-CoA transport; localized in peroxisomes and mitochondria; induced in macrophages; Hog1-repressed; stationary phase enriched; farnesol-upregulated in biofilm; Spider biofilm induced
PIS51819.1	28.16166952	28.66654563	28.72875038	28.04244902	28.15858808	28.42071685	0.578186911	0.934	-0.312	PIS51819.1	orf19.94	Protein of unknown function; Spider biofilm induced
PIS58617.1	28.52738226	26.98953653	28.56328037	27.50785756	27.73689631	27.9002295	0.755723999	0.949	-0.312	PIS58617.1	PRE2	Putative proteasome beta-5 subunit; macrophage-induced protein
PIS58533.1	31.85622953	31.72449205	32.03823172	31.5804102	31.59595486	31.50778997	0.232608563	0.841	-0.312	PIS58533.1	TOM70	Ortholog(s) have mitochondrion targeting sequence binding, protein transmembrane transporter activity

PIS48765.1	28.66924784	28.56662339	28.98574428	28.55550032	28.4474611	28.28108963	0.412449929	0.909	-0.313	PIS48765.1	orf19.6464	Protein of unknown function; induced upon adherence to polystyrene; oxidative stress-induced via Cap1
PIS55597.1	30.18115745	30.85965891	29.64596076	29.91217637	29.86485422	29.96960433	0.556103087	0.932	-0.313	PIS55597.1	VMA2	Vacuolar H(+)-ATPase; protein present in exponential and stationary growth phase yeast cultures; plasma membrane localized; amphotericin B repressed, caspofungin repressed
PIS54699.1	32.99503282	32.79290266	33.22168611	32.53559758	32.7159552	32.81563178	0.484735986	0.922	-0.314	PIS54699.1	AAT1	Aspartate aminotransferase; soluble protein in hyphae; macrophage-induced protein; alkaline upregulated; amphotericin B repressed; gene used for strain identification by multilocus sequence typing; farnesol-, Hap43p-induced; GlcNAc-induced
PIS49503.1	25.30758997	26.4541681	24.9092606	24.69136997	25.27356902	25.76118149	0.677754408	0.944	-0.315	PIS49503.1	ECM14	Has domain(s) with predicted metallocarboxypeptidase activity, zinc ion binding activity and role in proteolysis
PIS58701.1	24.19840372	23.08125839	24.25607648	23.88906562	23.32588759	23.37410276	0.392000149	0.904	-0.316	PIS58701.1	orf19.1479	Ortholog of the mitochondria localized S. cerevisiae Pib2 protein of unknown function; has a FYVE zinc finger domain; Spider biofilm induced
PIS48578.1	25.73635797	26.42817344	25.96034323	25.99421061	25.91049958	25.27113189	0.732565858	0.948	-0.316	PIS48578.1	orf19.5710	Nucleoporin component of central core of the nuclear pore complex; mRNA binds She3
PIS51572.1	30.25835719	30.87358377	30.46317656	29.80137494	30.45444736	30.38890787	0.639750324	0.94	-0.317	PIS51572.1	orf19.6065	RNA polymerase II holoenzyme/mediator subunit; regulated by Mig1, Tup1; amphotericin B, caspofungin repressed; protein present in exponential and stationary growth phase yeast; Hap43-repressed; Spider biofilm repressed
PIS49616.1	29.84449076	28.9296641	30.30932336	29.23221406	29.41200268	29.48532358	0.529155834	0.928	-0.318	PIS49616.1	SIM1	Adhesin-like protein; involved in cell wall maintenance, redundant with Sun41; possibly secreted; macrophage-repressed; repressed by Rim101, Cyr1, Ras1; Spider biofilm induced
PIS48247.1	30.17374705	30.34571474	29.98293031	29.65095345	29.6141203	30.27899264	0.336706377	0.889	-0.319	PIS48247.1	RVB2	Putative trancription modulator; RuvB-like protein family member; heterozygous null mutant displays sensitivity to virgineone
PIS51481.1	24.44210783	26.26320935	25.32851938	24.05052048	24.82427677	26.20227186	0.593323163	0.936	-0.319	PIS51481.1	WSC4	Putative cell wall integrity and stress response subunit 4 precursor; transcription is specific to white cell type

PIS48337.1	30.54780894	29.44273061	30.58330769	30.05494822	29.80750311	29.75229735	0.344544244	0.891	-0.32	PIS48337.1	ETR1	Putative 2-enoyl thioester reductase; protein present in exponential and stationary growth phase yeast cultures; rat catheter biofilm repressed
PIS59032.1	25.80035584	26.52433229	26.1489607	26.42860558	25.39042047	25.69475344	0.589242896	0.936	-0.32	PIS59032.1	MAF1	Putative negative regulator of RNA polymerase III; decreased expression in hyphae vs yeast cells; caspofungin repressed; Spider biofilm repressed
PIS54584.1	23.59108497	24.15617629	25.13729691	24.17130692	23.89770603	23.85704148	0.480670059	0.921	-0.32	PIS54584.1	orf19.2516	Ortholog of C. dubliniensis CD36 : Cd36_81040, C. parapsilosis CDC317 : CPAR2_101980, C. auris B8441 : B9J08_002360 and Candida tenuis NRRL Y-1498 : CANTEDRAFT_113332
PIS58243.1	25.16591237	27.49572065	25.09921131	24.73085207	26.20042565	25.87008594	0.823042914	0.953	-0.32	PIS58243.1	orf19.5161	Ortholog(s) have structural constituent of ribosome activity and mitochondrial large ribosomal subunit localization
PIS51599.1	27.220864	26.34523368	22.84020664	24.43005803	24.32428736	26.68584759	0.775223312	0.951	-0.322	PIS51599.1	orf19.2007	Subunit of GARP (Golgi-associated retrograde protein) complex, which has roles in Golgi to vacuole transport, ascospore wall assembly, cellular sphingolipid homeostasis, and filamentous growth
PIS55530.1	25.0740942	24.0724345	25.0723102	25.68138043	23.98814946	23.58063283	0.793865439	0.952	-0.323	PIS55530.1	NAB3	Putative nuclear polyadenylated RNA-binding protein; flucytosine repressed
PIS54538.1	27.1805898	27.5580415	28.20551058	27.52162835	27.51746437	26.93628121	0.421500164	0.911	-0.323	PIS54538.1	UTP8	Essential nucleolar protein; involved in tRNA export from the nucleus and ribosomal small subunit biogenesis; physically interacts with TAP-tagged Nop1; Spider biofilm induced
PIS49849.1	26.88869862	26.77887187	26.62343784	26.46645944	26.47706336	26.37673656	0.474475869	0.92	-0.324	PIS49849.1	LEA1	Predicted component of U2 snRNP; induced by alpha pheromone in SpiderM medium
PIS51226.1	24.97256239	25.73532299	24.06373509	24.15151846	25.22397939	24.42435382	0.583998158	0.935	-0.324	PIS51226.1	orf19.2261	Ortholog(s) have RNA binding activity, role in mRNA splicing, via spliceosome and U2 snRNP, U2-type prespliceosome localization
PIS51886.1	29.40633876	29.17961476	29.21987338	28.94195737	28.98792385	28.90459829	0.528465293	0.928	-0.324	PIS51886.1	orf19.3259	Ortholog(s) have peptidase activity, role in protein targeting to ER, signal peptide processing and endoplasmic reticulum, signal peptidase complex localization

PIS54871.1	28.70187257	28.10172032	28.07667	28.02581678	27.98955898	27.89304985	0.510363836	0.926	-0.324	PIS54871.1	orf19.3319	Putative thioredoxin; Spider biofilm repressed
PIS51267.1	30.23015432	31.18365666	29.18419169	29.15773259	30.18145314	30.28698032	0.627358085	0.939	-0.324	PIS51267.1	orf19.6160	Ortholog(s) have role in eisosome assembly and eisosome localization
PIS49593.1	25.35080777	25.96063322	25.14243188	25.79361382	25.04969306	24.63394788	0.445454247	0.915	-0.326	PIS49593.1	PTR3	Protein similar to S. cerevisiae Ptr3p, which is a sensor of external amino acids; expression upregulated in an ssr1 null mutant
PIS48318.1	28.47361763	27.13634174	28.75439146	27.76186849	27.75594725	27.86473357	0.474122112	0.92	-0.327	PIS48318.1	UTP22	Putative U3 snoRNP protein; Ssr1-induced; repressed by prostaglandins; heterozygous null mutant is resistant to parnafungin
PIS49689.1	27.55739117	27.86286107	27.68496144	27.70355135	27.10485238	27.31146003	0.398722657	0.906	-0.328	PIS49689.1	orf19.1305	Ortholog(s) have tRNA (guanine(37)-N1)-methyltransferase activity, role in mitochondrial tRNA methylation, tRNA N1-guanine methylation and cytoplasm, mitochondrial matrix localization
PIS48678.1	31.62906277	30.83161562	31.39198447	31.34158537	30.82746602	30.69932969	0.389617649	0.904	-0.328	PIS48678.1	SUR7	Protein required for normal cell wall, plasma membrane, cytoskeletal organization, endocytosis; localizes to eisosome subdomains of plasma membrane; 4 transmembrane motifs; cytoplasmic C terminus regulates morphogenesis and stress
PIS56731.1	31.58331071	31.44902869	31.80582801	31.38075355	31.28977566	31.17754826	0.519898698	0.927	-0.33	PIS56731.1	CCT8	Chaperonin-containing T-complex subunit; role in hyphal morphogenesis, particularly starvation-induced; essential; expression in S. cerevisiae inhibits Ras2-mediated pathways; CCT8 and TRP1 overlap; Spider biofilm repressed
PIS54959.1	29.08150996	28.42900298	28.44299082	28.56496766	28.14191941	28.25784946	0.345571073	0.892	-0.33	PIS54959.1	FTR2	High-affinity iron permease; probably interacts with ferrous oxidase; regulated by iron level, ciclopirox olamine, amphotericin B, caspofungin; complements S. cerevisiae ftr1 iron transport defect; Hap43-repressed; Spider biofilm induced
PIS48220.1	25.63278574	27.14979958	25.51293856	25.05294799	26.05996421	26.19153149	0.750041589	0.949	-0.33	PIS48220.1	orf19.6448	Protein of unknown function; Spider biofilm repressed
PIS54741.1	24.77676997	26.42722408	26.86202199	26.70213419	26.8259761	23.54894537	0.75638981	0.949	-0.33	PIS54741.1	TOP2	DNA topoisomerase II; catalyzes ATP-dependent DNA relaxation and decatenation in vitro; Y842 predicted to be catalytic; functional homolog of S. cerevisiae Top2p; sensitive to amsacrine or doxorubicin; farnesol-upregulated in biofilm

PIS48679.1	28.23667191	28.36934308	28.20997628	27.24988244	28.20270806	28.36694268	0.457270876	0.917	-0.332	PIS48679.1	PTK2	Putative protein kinase of polyamine import; mutation confers hypersensitivity to high concentrations of tunicamycin; YPD flow model biofilm induced; rat catheter and Spider biofilm induced
PIS56756.1	33.88487368	33.90850084	33.84751444	33.46849261	33.53609089	33.63862256	0.35779211	0.895	-0.333	PIS56756.1	FAS1	Beta subunit of fatty-acid synthase; multifunctional enzyme; Hap43, fluconazole-induced; amphotericin B, caspofungin repressed; macrophage/pseudohyphal-induced; flow model and Spider biofilm repressed
PIS51797.1	30.31452991	30.33283353	30.06577326	29.55438086	30.06570988	30.0944333	0.368171675	0.898	-0.333	PIS51797.1	PGA63	Component COPII vesicle coat; required for vesicle formation in ER to Golgi transport; predicted GPI-anchor; flow model and Spider biofilm repressed
PIS55789.1	28.31008982	28.14634176	27.34413238	27.72840881	27.44747302	27.62327419	0.570109032	0.933	-0.334	PIS55789.1	orf19.1676	Predicted potassium ion transporter; Spider biofilm induced
PIS48783.1	28.81065629	28.57604289	28.86597403	28.55673299	28.40343151	28.28665859	0.427716578	0.912	-0.335	PIS48783.1	orf19.512	Ortholog of S. cerevisiae Kre33; essential; S. cerevisiae ortholog is essential and is required for biogenesis of the small ribosomal subunit
PIS58309.1	29.45607357	29.52450136	29.49287115	28.92351155	29.34779737	29.19290286	0.270016834	0.863	-0.336	PIS58309.1	ARC40	Protein similar to S. cerevisiae Arc40; involved in actin filament organization in S. cerevisiae; transposon mutation affects filamentous growth; rat catheter and Spider biofilm repressed
PIS51209.1	24.16518307	25.33136592	25.55444361	24.20237394	24.15153747	25.68845831	0.583823706	0.935	-0.336	PIS51209.1	DQD1	Putative 3-dehydroquinate dehydratase; ketoconazole-repressed; protein abundance downregulated by macrophages; flow model biofilm induced
PIS49799.1	28.36496768	28.55612536	28.70571582	28.37297784	28.46384607	27.78265277	0.321817516	0.884	-0.336	PIS49799.1	NOP14	Putative nucleolar protein; Hap43-induced; mutation confers resistance to 5-fluorocytosine (5-FC), 5-fluorouracil (5-FU), and tubercidin (7-deazaadenosine); heterozygous mutant is resistant to parnafungin; Spider biofilm induced
PIS48728.1	31.36586564	31.31238449	31.29992951	30.97958524	31.01353691	30.97732316	0.344287503	0.891	-0.336	PIS48728.1	ROA1	Putative PDR-subfamily ABC transporter involved in sensitivity to azoles; Spider biofilm induced
PIS52244.1	24.5221517	24.98201989	24.22447604	24.15976807	24.46024547	24.10033085	0.449944615	0.916	-0.336	PIS52244.1	SRB9	Subunit of the RNA polymerase II mediator complex; transposon mutation affects filamentous growth; suppresses S. cerevisiae diploid filamentous (flo8, ste7, ste12, tec1) or haploid invasive (flo8) mutant growth defects

PIS50611.1	25.09833941	25.64748026	24.05488791	24.38880755	24.46303591	24.93873765	0.733566299	0.948	-0.337	PIS50611.1	PEX7	Ortholog(s) have peroxisome matrix targeting signal-2 binding activity, role in protein import into peroxisome matrix, docking and cytosol, peroxisome localization
PIS49853.1	28.82878815	28.53172183	28.56162714	27.99621689	28.55762271	28.35452656	0.243440295	0.848	-0.338	PIS49853.1	EMP46	Protein similar to S. cerevisiae Emp46, an integral membrane component of ER-derived COPII-coated vesicles; functions in ER to Golgi transport; induced by alpha pheromone in SpiderM medium
PIS58580.1	29.44775617	28.78733082	29.67087247	29.12189724	28.80703416	28.96353311	0.262932061	0.859	-0.338	PIS58580.1	URA6	Ortholog(s) have UMP/dUMP kinase activity, adenylate kinase activity, role in 'de novo' pyrimidine nucleobase biosynthetic process, nucleobase-containing compound metabolic process and cytoplasm, nucleus localization
PIS50367.1	30.5917341	30.55548526	30.8676107	30.31642232	30.39297196	30.28770725	0.370470288	0.899	-0.339	PIS50367.1	CHO2	Phosphatidyl-ethanolamine N-methyltransferase; fungal-specific (no human or murine homolog); amphotericin B repressed; Hap43p-induced gene
PIS58725.1	29.99171315	29.65455782	29.84447347	29.49856278	29.42716981	29.54839923	0.341473904	0.89	-0.339	PIS58725.1	CYS3	Cystathionine gamma-lyase; induced by alkaline, amphotericin B, cadmium stress, oxidative stress via Cap1; possibly adherence-induced; Hog1 regulated; reduced levels in stationary phase yeast cells; Spider and flow model biofilm induced
PIS56787.1	27.69307671	27.86747803	28.30040259	27.16464206	27.78489175	27.88768392	0.404539643	0.907	-0.341	PIS56787.1	FOL1	Putative dihydroneopterin aldolase (dihydro-6-hydroxymethylpterin pyrophosphokinase); fungal-specific (no human or murine homolog)
PIS50539.1	30.39421754	30.76222143	29.46828159	30.07847606	29.43455862	30.0879914	0.53056456	0.929	-0.341	PIS50539.1	orf19.3932	Predicted RNA binding protein; stationary phase enriched; induced in core caspofungin response; induced by nitric oxide independent of Yhb1; repressed in ssr1 null; ketoconazole, hypoxia induced; Spider biofilm induced
PIS54546.1	29.18546211	28.86791133	28.61948803	28.98192727	28.47201127	28.19112835	0.606060749	0.937	-0.343	PIS54546.1	ARX1	Putative ribosomal large subunit biogenesis protein; repressed in core stress response; repressed by prostaglandins
PIS49718.1	22.97622075	22.82326361	27.81245643	23.92799304	24.15933582	24.49515641	0.725837168	0.947	-0.343	PIS49718.1	HUT1	Ortholog(s) have UDP-galactose transmembrane transporter activity and role in UDP-galactose transmembrane transport, UDP-glucose transmembrane transport, UDP-glucose transmembrane transport into endoplasmic reticulum
PIS48541.1	26.83621374	25.88023098	27.08483559	25.82714754	26.10784101	26.8367649	0.657614912	0.942	-0.343	PIS48541.1	orf19.4626	Ortholog(s) have role in positive regulation of transcription by RNA polymerase I, regulation of TORC1 signaling and cytosol localization

PIS48761.1	28.05686783	27.8520817	28.09723299	28.15793432	27.50367744	27.31653488	0.523995636	0.928	-0.343	PIS48761.1	PEX1	Peroxisomal ATPase involved in import of peroxisomal matrix proteins; involved in oxidative stress tolerance
PIS58863.1	30.06904074	30.13069744	30.45637452	29.97967986	29.82383716	29.82363934	0.355298945	0.895	-0.343	PIS58863.1	SER33	Predicted enzyme of amino acid biosynthesis; Gcn4p-regulated; upregulated in biofilm; protein present in exponential and stationary growth phase yeast cultures; S. cerevisiae ortholog is Gcn4p regulated
PIS52020.1	24.31651655	25.75414935	24.71537927	24.28408593	24.7743895	24.69550834	0.47189738	0.92	-0.344	PIS52020.1	COQ5	Putative methyltransferase of ubiquinone biosynthesis; regulated by Gcn4; repressed by amino acid starvation (3-AT), Hap43; induced upon adherence to polystyrene; Spider biofilm repressed
PIS56670.1	24.88600413	25.06872351	24.72468434	24.49827446	24.69056853	24.45976079	0.663403272	0.943	-0.344	PIS56670.1	orf19.6723	Protein of unknown function; Spider biofilm induced
PIS50313.1	24.75959757	26.04706066	25.09703511	25.53913614	24.80761982	24.52047469	0.647587495	0.941	-0.345	PIS50313.1	IAH1	Protein similar to S. cerevisiae Iah1p, which is involved in acetate metabolism; mutation confers hypersensitivity to tunicamycin; transposon mutation affects filamentous growth
PIS58260.1	31.3721596	31.02655492	31.84926113	31.06850781	31.09429199	31.05024519	0.638146743	0.94	-0.345	PIS58260.1	PRK1	Putative protein serine/threonine kinase; mutants sensitive to growth on hydrogen peroxide medium
PIS56822.1	24.24002475	22.22255787	24.42023796	24.98615277	22.80581598	22.0536329	0.731159434	0.948	-0.346	PIS56822.1	ARG81	Zn(II)2Cys6 transcription factor; required for utilization of ornithine as a nitrogen source and for wild-type resistance to caffeine; required for yeast cell adherence to silicone substrate
PIS48514.1	24.28392255	25.20535473	24.92257626	23.71624855	24.71799116	24.94047704	0.807448543	0.953	-0.346	PIS48514.1	orf19.3782	Predicted membrane transporter; member of the peptide-acetyl-coA transporter (PAT) family, major facilitator superfamily (MFS); induced by alpha pheromone in Spider medium
PIS54502.1	32.04388533	31.51195943	32.27091945	31.61786142	31.53177752	31.6356202	0.371846833	0.899	-0.347	PIS54502.1	ARF2	Essential protein, putative ADP-ribosylation factor; involved in invasive growth; mutation confers hypersensitivity to Brefeldin A; Spider biofilm repressed
PIS49493.1	25.66513902	26.15979944	25.38065145	24.57798545	26.2257812	25.36147177	0.507567013	0.925	-0.347	PIS49493.1	SPT20	Putative transcription factor; downregulated upon adherence to polystyrene; flucytosine repressed

PIS48468.1	31.67747263	31.9616977	31.6384421	31.3713543	31.29442316	31.57154011	0.500059706	0.924	-0.347	PIS48468.1	SRB1	Essential GDP-mannose pyrophosphorylase; makes GDP-mannose for protein glycosylation; functional in S. cerevisiae psa1; on yeast-form, not hyphal cell surface; alkaline induced; induced on adherence to polystyrene; Spider biofilm repressed
PIS54716.1	27.70128056	28.42853697	28.38450196	28.13774546	27.670191	27.66348183	0.56466195	0.933	-0.348	PIS54716.1	GUK1	Putative guanylate kinase; identified in extracts from biofilm and planktonic cells; protein level decrease in stationary phase cultures; Hap43p-induced gene
PIS55748.1	30.53906293	29.9918468	31.29019624	30.22663965	30.16959786	30.37711482	0.672342209	0.943	-0.349	PIS55748.1	orf19.4898	Putative protein of unknown function; induced by prostaglandins
PIS55787.1	31.35783877	31.94597193	31.03948701	30.56496055	31.07598645	31.65574539	0.383587955	0.902	-0.349	PIS55787.1	TFP1	Subunit of vacuolar H+-ATPase; stationary phase enriched protein; sumoylation target; Spider biofilm repressed
PIS54566.1	31.67214882	31.43792381	31.64621337	31.12987763	31.25338698	31.32345745	0.182534473	0.801	-0.35	PIS54566.1	NDE1	Putative NADH dehydrogenase; may act alternatively to complex I in respiration; caspofungin repressed; rat catheter biofilm induced; Spider biofilm repressed
PIS54647.1	29.09785642	29.90784181	28.69401994	28.21324436	29.24552852	29.1898499	0.439412076	0.914	-0.35	PIS54647.1	orf19.200	Putative nuclear RNA-binding protein; Spider biofilm repressed
PIS58385.1	30.27777655	29.35240846	30.53521245	29.96991911	29.62833337	29.51089182	0.457078541	0.917	-0.352	PIS58385.1	orf19.6887	Predicted ORF from Assembly 19; repressed by nitric oxide; removed from Assembly 20; subsequently reinstated in Assembly 21 based on comparative genome analysis
PIS50484.1	30.2220231	30.2982842	30.56514363	29.94421468	30.09617741	29.98503977	0.284770526	0.869	-0.353	PIS50484.1	PFK1	Phosphofructokinase alpha subinit; activated by fructose 2,6-bisphosphate, AMP, ATP inhibited; activity reduced on hyphal induction; phagocytosis-repressed; fluconazole, flow model biofilm induced; rat catheter and Spider biofilm repressed
PIS58659.1	28.67501437	28.62557796	28.18265004	28.44374949	28.08932888	27.88632268	0.410081354	0.908	-0.355	PIS58659.1	DCP2	Ortholog(s) have chromatin binding, hydrolase activity, mRNA binding activity
PIS48455.1	26.58820422	26.53446907	25.20102991	24.7018393	25.7208127	26.8350845	0.580097385	0.935	-0.355	PIS48455.1	FMP45	Predicted membrane protein induced during mating; mutation confers hypersensitivity to toxic ergosterol analog, to amphotericin B; alkaline repressed; repressed by alpha pheromone in SpiderM medium; rat catheter, Spider biofilm induced

PIS56908.1	34.87751267	32.79079835	35.98697317	35.21535812	33.76990155	33.60408304	0.888906531	0.957	-0.355	PIS56908.1	OBPA	Putative oxysterol binding protein; non-sex gene located within the MTLa mating-type-like locus; Plc1p-regulated
PIS58224.1	29.48332655	28.1048324	28.79848727	28.3751222	28.40253787	28.53701602	0.299644319	0.876	-0.357	PIS58224.1	orf19.7011	Ortholog(s) have role in maturation of SSU-rRNA from tricistronic rRNA transcript (SSU-rRNA, 5.8S rRNA, LSU-rRNA) and nucleus, preribosome, small subunit precursor localization
PIS51752.1	30.3179706	30.30419211	30.92776841	30.07570304	30.15171693	30.24705211	0.310724016	0.88	-0.358	PIS51752.1	TRP5	Predicted tryptophan synthase; identified in detergent-resistant membrane fraction (possible lipid raft component); predicted N-terminal acetylation; Gcn4p-regulated; S. cerevisiae ortholog is Gcn4p regulated; upregulated in biofilm;
PIS49751.1	27.74978895	27.35003134	27.64426567	27.06242395	27.43266967	27.17193536	0.496159247	0.924	-0.359	PIS49751.1	orf19.4735	Ornithine cyclodeaminase family protein; Sef1, Sfu1, and Hap43-regulated; ortholog of S. cerevisiae YGL159W and S. pombe SPAP11E10.01; rat catheter biofilm induced
PIS54486.1	26.94530805	27.47598553	25.79394969	26.36495223	26.46586107	26.30670789	0.576801502	0.934	-0.359	PIS54486.1	orf19.5961	Ortholog(s) have proteasome regulatory particle binding, protein folding chaperone activity, role in proteasome regulatory particle assembly and cytosol, nucleus localization
PIS58874.1	30.12875675	29.97840738	29.55886151	29.45508954	29.59618015	29.5371434	0.280297351	0.867	-0.359	PIS58874.1	RPN6	Putative 26S proteasome subunit; Hap43p-induced gene; regulated by Gcn2p and Gcn4p
PIS56568.1	28.22430276	28.09869855	28.40356778	27.91931383	27.71714081	28.00921833	0.310601791	0.88	-0.36	PIS56568.1	MCM6	Putative MCM DNA replication initiation complex component; mRNA expression peak at cell-cycle M/G1 phase; regulated by tyrosol and cell density; repressed by alpha pheromone in SpiderM medium; Hap43-induced gene
PIS51457.1	28.7893188	28.93870249	27.56076757	27.52098325	28.71856464	27.96807525	0.397957495	0.906	-0.36	PIS51457.1	orf19.4530.1	Protein of unknown function; regulated by Nrg1, Tup1; Spider and flow model biofilm induced
PIS58074.1	26.54564215	26.27975308	26.29153484	25.70855363	26.23992496	26.08810209	0.545017296	0.93	-0.36	PIS58074.1	orf19.6597	Has domain(s) with predicted role in regulation of cell cycle
PIS51124.1	29.62492818	30.03267203	28.94249556	28.9546797	29.27474041	29.28828128	0.464606442	0.919	-0.361	PIS51124.1	orf19.641	Ortholog(s) have role in protein folding and endoplasmic reticulum localization

PIS49735.1	29.08919532	29.13219625	29.22418325	28.97361645	28.76481129	28.62386718	0.322174031	0.884	-0.361	PIS49735.1	orf19.6783	Putative geranylgeranyltransferase regulatory component
PIS48766.1	24.84937786	24.80707237	24.86051241	24.39423834	24.4193985	24.61913125	0.648429259	0.941	-0.361	PIS48766.1	orf19.6918	Mitochondrial protein required for expression of respiratory chain complex III (coenzyme Q:cytochrome c oxidoreductase); Hap43-induced gene; Spider biofilm induced
PIS48564.1	28.11218783	28.22087192	28.53014596	28.3616226	27.68738339	27.73195133	0.544738623	0.93	-0.361	PIS48564.1	SMT3	SUMO, small ubiquitin-like protein; Smt3p-conjugated proteins localize to septation site and mother side of bud neck; C. albicans septins appear not to be Smt3p-modified, in contrast to S. cerevisiae septins
PIS54720.1	28.96776798	29.24409708	28.65405773	28.71630135	28.79706929	28.26576795	0.504848888	0.925	-0.362	PIS54720.1	orf19.2671	Protein with a predicted FAD-dependent pyridine nucleotide reductase domains; putative oxidoreductase; Plc1-regulated; possibly an essential gene, disruptants not obtained by UAU1 method
PIS55544.1	29.41120131	29.55506199	29.60790315	29.06784211	29.20578193	29.20717424	0.37160369	0.899	-0.364	PIS55544.1	orf19.2091	Putative NADH dehydrogenase; repressed by nitric oxide, Hap43p-repressed
PIS50566.1	26.35951507	28.31975482	27.5738059	28.99120017	25.70203822	26.46707524	0.726997147	0.947	-0.364	PIS50566.1	orf19.3939	Ortholog(s) have EMC complex localization
PIS50503.1	25.00465065	25.01098356	24.22348477	23.84378863	24.86901797	24.43398429	0.670927442	0.943	-0.364	PIS50503.1	orf19.3996	Putative mannosyltransferase; similar to S. cerevisiae Gpi10p; has HKEXRF motif
PIS48449.1	25.37527147	21.36693165	25.7112163	25.0776657	24.3234664	21.95635058	0.835752181	0.954	-0.365	PIS48449.1	orf19.1045	Putative U2 snRNP protein; Hap43p-induced gene; mutation confers hypersensitivity to 5-fluorocytosine (5-FC), 5-fluorouracil (5-FU), and tubercidin (7-deazaadenosine)
PIS48395.1	24.2278961	25.12403559	25.27125386	24.30514464	24.67334869	24.54940218	0.570710796	0.933	-0.365	PIS48395.1	orf19.2487	Protein with a predicted phosphoribulokinase/uridine kinase domain; Spider biofilm induced
PIS52382.1	33.36136307	33.00612427	33.34691422	32.982898	32.74767186	32.88450933	0.214163946	0.828	-0.366	PIS52382.1	ACS1	Acetyl-CoA synthetase; induced by human neutrophils; fluconazole-repressed; regulated by Nrg1/Mig1; colony morphology-related gene regulation by Ssn6; only in stationary phase cultures; rat catheter biofilm repressed, Spider biofilm induced

PIS58318.1	31.59854337	32.21239892	31.52411661	31.3701819	31.33097722	31.5310937	0.567196165	0.933	-0.368	PIS58318.1	GRE3	Putative D-xylose reductase; antigenic in murine systemic infection; soluble protein in hyphae; induced by farnesol, macrophage interaction and by Mnl1 under weak acid stress; stationary-phase enriched protein; Spider biofilm induced
PIS54626.1	34.84277933	32.67643417	35.9724773	35.19641364	33.67364573	33.51878055	0.907490337	0.958	-0.368	PIS54626.1	orf19.6610	Ortholog(s) have microtubule binding, microtubule plus end polymerase activity
PIS58921.1	24.06109293	25.41101149	23.73828793	23.3277104	24.19914377	24.57540283	0.558150995	0.932	-0.369	PIS58921.1	DOT6	Protein with a predicted role in telomeric gene silencing and filamentation; repressed by high-level peroxide stress; Spider biofilm induced
PIS49583.1	30.34284984	30.11751056	30.50239487	29.91532219	29.93992558	29.99944652	0.168185729	0.785	-0.369	PIS49583.1	orf19.4127	Ortholog(s) have actin filament binding activity, role in actin cortical patch localization, actin cytoskeleton organization, actin filament bundle assembly, endocytosis and actin cortical patch localization
PIS55532.1	24.89909752	25.41298795	24.81855922	24.92021008	24.32160158	24.78178155	0.477467624	0.921	-0.369	PIS55532.1	SEC20	Essential protein; similar to S. cerevisiae Sec20p; depletion causes membrane accumulation and drug sensitivity; expression regulated by growth phase; O-mannosylation regulates proteolysis; does not complement S. cerevisiae sec20-1 mutant
PIS54586.1	26.06285604	25.59926237	26.50959473	26.13047696	26.81451423	24.11789583	0.668559112	0.943	-0.37	PIS54586.1	orf19.6166	Ortholog of C. dubliniensis CD36 : Cd36_80810, C. parapsilosis CDC317 : CPAR2_101800, C. auris B8441 : B9J08_002362 and Candida tenuis NRRL Y-1498 : CANTEDRAFT_103482
PIS54830.1	34.81544358	34.41499728	34.95288388	34.35550305	34.42151613	34.29744647	0.387238851	0.903	-0.37	PIS54830.1	RPS3	Ribosomal protein S3; Hog1, Hap43-induced; grepressed upon phagocytosis by murine macrophage; present in exponential and stationary phase cells; Spider biofilm repressed
PIS49807.1	31.09338108	30.50245669	31.13793589	30.54548227	30.56358152	30.5093762	0.422573771	0.911	-0.372	PIS49807.1	CDC10	Septin, required for wild-type cell, hyphal, or chlamydospore morphology; role in virulence and kidney tissue invasion in mouse infection; forms ring at sites of cell division and filaments in mature chlamydospore; Spider biofilm repressed
PIS50640.1	28.46309691	29.54822817	28.36607562	28.20282974	28.48419907	28.57522587	0.599507681	0.937	-0.372	PIS50640.1	FRP1	Ferric reductase-related protein involved in heme acquisition; alkaline-induced by Rim101; iron-chelation-induced by CCAAT-binding factor; fluconazole-repressed; ciclopirox-, hypoxia-, Hap43-induced
PIS56861.1	26.62475917	27.03136861	26.87262799	26.27335387	26.47762777	26.66282468	0.501943427	0.925	-0.372	PIS56861.1	orf19.2756	Ortholog(s) have HDEL sequence binding activity, role in endoplasmic reticulum to Golgi vesicle-mediated transport and endoplasmic reticulum membrane localization

PIS50581.1	26.16352143	25.83439162	25.36575166	25.36881927	25.61100274	25.26872806	0.283418881	0.869	-0.372	PIS50581.1	orf19.4185	Ortholog(s) have phosphatase activity, phosphoprotein phosphatase activity, thiosulfate sulfurtransferase activity and role in dephosphorylation
PIS49703.1	26.27553308	26.71243051	24.86312601	25.67198568	25.37227041	25.68843534	0.424201036	0.911	-0.373	PIS49703.1	MSK1	Putative mitochondrial lysine-tRNA synthetase; flucytosine repressed
PIS58431.1	25.45890356	27.01242494	25.17711251	24.60523497	25.31047574	26.61443081	0.560564851	0.932	-0.373	PIS58431.1	orf19.2850	Protein of unknown function; induced by nitric oxide independent of Yhb1p
PIS52068.1	23.98141894	25.41013606	23.82987619	23.69271128	24.33109842	24.07797791	0.446386496	0.916	-0.373	PIS52068.1	orf19.6255	Putative protein of unknown function; Hap43p-repressed gene
PIS48764.1	24.41718319	24.97425095	24.68867985	25.02866397	23.65177065	24.28046444	0.517068292	0.927	-0.373	PIS48764.1	orf19.6463	Putative ortholog of S. cerevisiae Npa3p; possibly essential for growth (however, depletion mutant is viable)
PIS55825.1	23.73265782	23.92415532	23.49323665	22.9540921	23.67253512	23.40467393	0.739143523	0.948	-0.373	PIS55825.1	SSU81	Predicted adaptor protein involved in activation of MAP kinase-dependent signaling pathways; links response to oxidative stress to morphogenesis and cell wall biosynthesis; mediates formation of microcolonies; caspofungin repressed
PIS52344.1	25.90856455	25.63400919	26.80464053	25.9896294	25.57557985	25.66072478	0.30121829	0.876	-0.374	PIS52344.1	orf19.5920	Ortholog of C. dubliniensis CD36 : Cd36_84570, C. parapsilosis CDC317 : CPAR2_404420, C. auris B8441 : B9J08_003958 and Candida tenuis NRRL Y-1498 : CANTEDRAFT_115339
PIS54819.1	26.83503442	28.28255893	27.79689992	27.26953078	27.4230966	27.10044286	0.59162245	0.936	-0.374	PIS54819.1	PEX14	Ortholog(s) have protein-macromolecule adaptor activity, role in protein import into peroxisome matrix, docking and peroxisomal importomer complex, peroxisomal membrane, peroxisome localization
PIS51961.1	28.05752705	28.04619451	28.3463356	27.8131905	27.80739405	27.70782958	0.528101816	0.928	-0.374	PIS51961.1	VAN1	Member of Mnn9 family of mannosyltransferases; ortholog of S. cerevisiae Van1p; fungal-specific (no human or murine homolog)
PIS48822.1	30.33987971	30.5565022	30.39243605	29.96466953	30.12272048	30.07667252	0.226110382	0.837	-0.375	PIS48822.1	GUA1	Putative GMP synthase, involved in the final step of guanine biosynthesis; soluble protein in hyphae; flucytosine induced; macrophage-downregulated protein abundance; protein level decreases in stationary phase cultures

PIS55797.1	30.03272805	30.38556739	30.05700908	29.82171339	29.80592901	29.72155592	0.194320012	0.812	-0.375	PIS55797.1	PPT1	Putative serine/threonine phosphatase; induced in high iron
PIS55015.1	26.22410654	26.93474184	26.00746611	25.97343227	25.45858659	26.60607487	0.417262597	0.91	-0.376	PIS55015.1	orf19.1182	Ortholog(s) have SNAP receptor activity, phosphatidylinositol-3-phosphate binding activity
PIS49793.1	27.85380313	28.35950483	27.5900269	27.54350007	27.3955467	27.73256888	0.650847424	0.941	-0.377	PIS49793.1	HAL9	Putative Zn(II)2Cys6 transcription factor; gene in zinc cluster region of Chr. 5; induced by Mnl1 in weak acid; similar to S. cerevisiae Hal9, a putative transcription factor involved in salt tolerance
PIS49536.1	29.12551082	29.15354547	29.68177815	29.09132093	28.9068658	28.831869	0.417500872	0.91	-0.377	PIS49536.1	orf19.5158	Protein with similarity to a human gene associated with colon cancer and to orf19.5158; regulated by Gcn4, Cyr1; induced by amino acid starvation; macrophage-induced protein, macrophage-repressed; Spider biofilm induced
PIS54962.1	23.43865625	22.54232583	23.78622241	23.05260902	21.99506775	23.58865296	0.6595974	0.942	-0.377	PIS54962.1	orf19.6694	Ortholog of C. dubliniensis CD36 : Cd36_73320, C. parapsilosis CDC317 : CPAR2_703860, C. auris B8441 : B9J08_002111 and Candida tenuis NRRL Y-1498 : CANTEDRAFT_114480
PIS58895.1	26.98817376	26.28111912	26.93128474	26.43586536	26.36510497	26.26510428	0.526803132	0.928	-0.378	PIS58895.1	orf19.1900	Ortholog(s) have RNA methyltransferase activity and role in 7-methylguanosine cap hypermethylation, RNA methylation, meiotic cell cycle, nucleologenesis, regulation of telomere maintenance via telomerase, tRNA processing
PIS51386.1	27.63498701	28.8474643	28.63314006	28.32301918	27.79521963	27.86395283	0.653677075	0.942	-0.378	PIS51386.1	orf19.7215.3	Ortholog(s) have protein-folding chaperone binding, unfolded protein binding activity and role in chaperone-mediated protein complex assembly, protein folding, protein import into mitochondrial intermembrane space, protein refolding
PIS51176.1	25.68775227	26.01227486	25.42380994	25.32299424	25.23329507	25.43344744	0.593401286	0.936	-0.378	PIS51176.1	SPT7	Putative SAGA transcriptional regulatory complex subunit; mutation confers hypersensitivity to toxic ergosterol analog, and to amphotericin B
PIS52004.1	32.9586812	32.94254715	32.98884463	32.38757976	32.66070127	32.70860692	0.215614207	0.83	-0.378	PIS52004.1	TKL1	Putative transketolase; localizes to surface of yeast cells, not hyphae; soluble protein in hyphae; transcript regulated by Nrg1, Mig1, and Tup1; antigenic in human or murine infection; possibly essential (by UAU1 method)
PIS58159.1	28.57730947	29.08351413	28.77847368	28.14128183	28.39132079	28.77006971	0.251670334	0.853	-0.379	PIS58159.1	CYP5	localization

PIS55609.1	29.05043376	29.1436953	29.30235754	28.83653892	28.63191284	28.88849389	0.166811189	0.783	-0.38	PIS55609.1	ALT1	Putative alanine transaminase; mutation confers hypersensitivity to 5-fluorocytosine (5-FC); rat catheter and flow model biofilm induced
PIS54574.1	34.68275254	34.77110254	34.63048136	34.24184753	34.24880597	34.45327149	0.282989663	0.869	-0.38	PIS54574.1	GND1	6-phosphogluconate dehydrogenase; soluble in hyphae; farnesol, macrophage-induced protein; antigenic in mice; dual localization to cytosol and peroxisomes depends on alternative splicing; rat catheter and Spider biofilm repressed
PIS54828.1	27.65914255	27.89396312	27.51439888	27.21502332	27.0525683	27.66037891	0.171186711	0.788	-0.38	PIS54828.1	orf19.1734	Putative ATPase and nucleosome spacing factor; required for hyphal growth; removes histone variant H2A.Z (Hta3p) from hypha-specific promoters ; heterozygous null mutant displays sensitivity to virgineone
PIS58160.1	28.63237302	28.41173811	28.21236451	27.52453306	28.19391883	28.39708209	0.326177138	0.885	-0.38	PIS58160.1	orf19.3357	Ortholog(s) have structural constituent of ribosome activity and mitochondrial small ribosomal subunit, mitochondrion localization
PIS56726.1	28.77762282	29.02459066	28.88989463	28.62684331	28.38246849	28.53926826	0.239405513	0.846	-0.381	PIS56726.1	MVD	Mevalonate diphosphate decarboxylase; functional homolog of S. cerevisiae Erg19; possible drug target; regulated by carbon source, yeast-hypha switch, growth phase, antifungals; gene has intron; rat catheter, Spider biofilm repressed
PIS55785.1	32.07650982	31.89691748	32.21905606	31.81500712	31.61251614	31.61946457	0.489578603	0.923	-0.382	PIS55785.1	orf19.1682	Membrane protein; Hap43p-repressed gene; repressed by nitric oxide
PIS55758.1	28.49795068	27.43805488	29.20395869	28.4993792	28.15062198	27.34166952	0.408566753	0.908	-0.383	PIS55758.1	COX15	Cytochrome oxidase assembly protein; transcript regulated by Nrg1 and Tup1; alkaline repressed; Hap43-repressed; early-stage flow model biofilm induced; Spider biofilm repressed
PIS48700.1	28.53280513	29.26028931	27.88878709	28.56912293	28.16372397	27.79946736	0.345184176	0.892	-0.383	PIS48700.1	EXO84	Predicted subunit of the exocyst complex, involved in exocytosis; localizes to a crescent on the surface of the hyphal tip
PIS50400.1	28.4826702	28.27383734	28.46996067	28.32826979	27.85344933	27.89677415	0.517325728	0.927	-0.383	PIS50400.1	NSA1	Putative 66S pre-ribosomal particles conmponent; Hap43-induced; repressed by prostaglandins
PIS55548.1	29.70056616	29.546565	29.73893645	29.56688763	29.2340054	29.03475465	0.307190088	0.879	-0.383	PIS55548.1	orf19.2101	Ortholog(s) have protein transmembrane transporter activity, protein-membrane adaptor activity

PIS54791.1	25.13266033	23.75416573	24.70507054	23.95597728	24.33360808	24.14592248	0.517109289	0.927	-0.385	PIS54791.1	APN1	Ortholog of S. cerevisiae Apn1; an AP endonuclease; transcript induced by interaction with macrophages; fungal-specific (no human or murine homolog)
PIS48529.1	26.23326653	25.97409695	26.21904241	25.69536069	24.86768583	26.70788732	0.495494379	0.924	-0.385	PIS48529.1	orf19.6861	Ortholog(s) have ubiquitin protein ligase activity, role in anaphase-promoting complex-dependent catabolic process, chromatin organization, protein ubiquitination and anaphase-promoting complex localization
PIS56645.1	28.32375051	28.78824868	27.88567155	27.80655099	28.05395381	27.97774366	0.255411995	0.855	-0.386	PIS56645.1	orf19.1536	Putative zinc transporter essential for tolerance to zinc; plays a role in zincosome formation; Hap43-induced; required for normal filamentous growth; mRNA binds She3 and is localized to hyphal tips
PIS52021.1	32.83015503	33.13361538	32.67956661	32.17463779	32.56469877	32.74294613	0.282475711	0.868	-0.387	PIS52021.1	CDC19	Pyruvate kinase at yeast cell surface; Gcn4/Hog1/GlcNAc regulated; Hap43/polystyrene adherence induced; repressed by phagocytosis/farnesol; hyphal growth role; stationary phase enriched; flow model biofilm induced; Spider
PIS48307.1	31.31134195	31.41638154	31.50143511	31.09985903	30.93858481	31.03100992	0.166129008	0.783	-0.387	PIS48307.1	DPS1-1	Putative tRNA-Asp synthetase; genes encoding ribosomal subunits, translation factors, tRNA synthetases are downregulated upon phagocytosis by murine macrophage; protein enriched in stationary phase yeast cultures
PIS58429.1	26.17990517	25.65760157	26.46588186	25.84573485	25.9017534	25.39484237	0.235264071	0.843	-0.387	PIS58429.1	orf19.2847	Ortholog(s) have RNA polymerase III activity, role in tRNA transcription by RNA polymerase III and RNA polymerase III complex localization
PIS50465.1	32.14043185	31.95900435	32.00425263	31.48647695	31.78720131	31.66911827	0.279721742	0.867	-0.387	PIS50465.1	orf19.891	Putative AMP deaminase; possibly an essential gene, disruptants not obtained by UAU1 method
PIS48232.1	23.61536736	24.08000951	22.87998635	23.27879781	23.66964697	22.46671005	0.551564612	0.931	-0.387	PIS48232.1	RAD14	Putative DNA repair protein; transcription is regulated upon yeast-hyphal switch; flucytosine repressed
PIS54476.1	30.29705883	29.92238188	30.34349173	29.75766127	29.8564771	29.7849483	0.324476859	0.885	-0.388	PIS54476.1	HGT12	Glucose, fructose, mannose transporter; major facilitator superfamily; role in macrophage-induced hyphal growth; detected at germ tube plasma membrane by mass spectrometry; Snf3p-induced; 12 probable transmembrane segments
PIS48473.1	24.71366604	26.44162959	25.65302928	25.10472668	25.16307526	25.37693881	0.652463968	0.942	-0.388	PIS48473.1	YTA6	Protein similar to S. cerevisiae Yta6p ATPase but ortholog of S. cervisiae Sap1; transposon mutation affects filamentous growth; induced by Mnl1 under weak acid stress

PIS51861.1	27.85875464	28.92594795	28.50581694	27.66447169	28.22073017	28.23853191	0.502477297	0.925	-0.389	PIS51861.1	HIS4	Multifunctional enzyme that catalyzes three steps of histidine biosynthesis, with phosphoribosyl-AMP cyclohydrolase, phosphoribosyl-ATP diphosphatase, and histidinol dehydrogenase activities; required for wild-type adhesion to human calls
PIS58901.1	26.8844567	26.94443957	26.31271746	25.64662079	27.07309649	26.25393331	0.372995836	0.899	-0.389	PIS58901.1	orf19.2414	Ortholog of S. cerevisiae Mpm1; a mitochondrial intermembrane space protein of unknown function; Hap43-repressed; Spider biofilm induced
PIS54609.1	27.07058264	27.25379185	25.84663713	26.53398556	26.70509152	25.75958587	0.545767054	0.931	-0.391	PIS54609.1	orf19.7441	Ortholog(s) have chromatin binding activity, role in lipid homeostasis, nuclear envelope organization, regulation of transcription by RNA polymerase II and nuclear envelope localization
PIS52213.1	27.67684485	26.82171489	28.0109159	26.85916502	27.26563614	27.2089338	0.460403573	0.918	-0.392	PIS52213.1	PUS7	Pseudouridine synthase; catalyzes pseudouridylation in U2 snRNA, 5S rRNA, cytoplasmic tRNAs and in pre-tRNA(Tyr); F-12/CO2 early biofilm induced
PIS52351.1	29.15709841	29.05950774	28.94463661	28.7582783	28.98739064	28.23634233	0.57163728	0.934	-0.393	PIS52351.1	orf19.4960	Ortholog(s) have spermine synthase activity and role in pantothenate biosynthetic process, spermine biosynthetic process
PIS49730.1	24.05313759	23.35818515	24.00683904	23.22139304	23.50728995	23.50929488	0.374923886	0.9	-0.393	PIS49730.1	orf19.6789	S. cerevisiae ortholog Nud1 is a spindle pole body outer plaque component; it acts through the mitotic exit network to specify asymmetric spindle pole body inheritance; Hap43-induced gene
PIS51948.1	28.55330901	28.60947515	29.01911831	28.22748883	28.23202489	28.54228542	0.42531912	0.911	-0.393	PIS51948.1	SNL1	Ribosome-associated protein predicted to function in protein synthesis; 1 predicted transmembrane domain; rat catheter biofilm repressed
PIS49727.1	27.15593567	27.05577409	26.795043	26.64449026	26.88304406	26.29729429	0.335481926	0.889	-0.394	PIS49727.1	HHT1	Histone H3 variant, specific to CTG-clade species; acts in regulation of planktonic and biofilm growth; reduced mRNA abundance in fkh2 mutant; farnesol regulated; Spider biofilm induced; rat catheter biofilm repressed
PIS58622.1	29.09011372	29.68208442	28.76284969	29.24179994	28.56030692	28.54988683	0.713161078	0.946	-0.394	PIS58622.1	orf19.2227	Protein of unknown function; flow model biofilm induced; nitric oxide-repressed
PIS51238.1	34.95546264	34.70323099	35.12050627	34.4361096	34.66118701	34.49719556	0.178747402	0.797	-0.395	PIS51238.1	CEF3	Translation elongation factor 3; antigenic in humans; predicted C-term nucleotide-binding active site; protein on surface of yeast, not hyphae; polystyrene adherence induced; higher protein amount in stationary phase; possibly essential

PIS51507.1	27.8017552	28.6566966	26.90051965	27.60307727	27.69122965	26.87980828	0.723603034	0.947	-0.395	PIS51507.1	orf19.5989	Putative cleavage factor I subunit; required for the cleavage and polyadenylation of pre-mRNA 3' ends; Spider biofilm repressed
PIS52232.1	23.71064489	22.5579383	24.22037241	23.77047337	22.80830792	22.72063215	0.78707395	0.951	-0.397	PIS52232.1	orf19.6377	Ortholog(s) have protein C-terminal leucine carboxyl O-methyltransferase activity and role in C-terminal protein methylation, protein-containing complex assembly, regulation of autophagy
PIS50490.1	32.18251272	31.98904339	32.71003524	32.04022724	31.68049609	31.97056948	0.599119829	0.937	-0.397	PIS50490.1	PUT2	Putative delta-1-pyrroline-5-carboxylate dehydrogenase; regulated by Put3p; null mutant cannot grow on proline as a nitrogen source and shows reduced invasive growth both in vitro and in vivo; Spider biofilm induced
PIS58414.1	24.16511533	24.09551464	25.300229	23.71769446	24.90881863	23.74020527	0.517476136	0.927	-0.398	PIS58414.1	orf19.2915	Ortholog(s) have ADP phosphatase activity, ATPase, CDP phosphatase activity, GDP phosphatase activity, GTPase activity, UDP phosphatase activity, nucleoside diphosphate phosphatase activity, ribonucleoside triphosphate phosphatase activity
PIS51791.1	24.42081104	24.56179046	24.4953616	24.42241474	24.15293483	23.70894358	0.283761799	0.869	-0.398	PIS51791.1	PCL7	Putative cyclin-like protein; possible Pho85 cyclin; hyphal repressed; induced by Mnl1 under weak acid stress
PIS56599.1	29.94204116	30.46508678	29.39814148	29.53281616	29.45665037	29.61850647	0.43415427	0.913	-0.399	PIS56599.1	CCT5	T-complex protein 1, epsilon subunit; protein present in exponential and stationary growth phase yeast cultures; Spider biofilm repressed
PIS51992.1	24.66557518	25.30599811	25.58873294	24.63129237	24.9076283	24.82416086	0.622391834	0.939	-0.399	PIS51992.1	orf19.6233	Ortholog of S. cerevisiae : PBR1, C. glabrata CBS138 : CAGL0J11264g, C. dubliniensis CD36 : Cd36_06340, C. parapsilosis CDC317 : CPAR2_206960 and C. auris B8441 : B9J08_003603
PIS48744.1	29.64227861	30.13682989	29.68075731	29.51120388	29.37251767	29.37980867	0.734939129	0.948	-0.399	PIS48744.1	orf19.7263	Putative X-Pro aminopeptidase; Spider biofilm repressed
PIS51629.1	23.89469038	24.59514525	26.32459389	24.58538709	23.88472057	25.14404829	0.596912217	0.936	-0.4	PIS51629.1	orf19.1034	Protein with a predicted cytochrome b5-like Heme/Steroid binding domain; Hap43, caspofungin repressed; flow model biofilm induced
PIS58886.1	28.8451912	29.13501058	28.73189277	28.2524168	28.44102862	28.81724466	0.450380836	0.916	-0.4	PIS58886.1	orf19.2863	Ortholog of C. dubliniensis CD36 : Cd36_28090, C. parapsilosis CDC317 : CPAR2_802560, C. auris B8441 : B9J08_000343 and Candida tenuis NRRL Y-1498 : CANTEDRAFT_104949

PIS48669.1	26.38184631	23.97346556	27.00114957	26.32743219	26.49886158	23.32966941	0.672537613	0.943	-0.4	PIS48669.1	orf19.3399	Ortholog(s) have histone binding activity, role in chromatin remodeling and Swr1 complex localization
PIS51930.1	28.85087701	28.84438454	29.45217621	28.46822929	28.96207181	28.51793315	0.581257739	0.935	-0.4	PIS51930.1	orf19.364	Putative vacuolar H+-ATPase subunit; required for proper vacuolar ATPase assembly and vacuolar functions; Spider biofilm repressed
PIS52076.1	26.5223968	25.85279194	26.35792218	25.70783394	26.1012137	25.72264812	0.331004632	0.887	-0.4	PIS52076.1	SSF1	Protein involved in ribosome biogenesis; ortholog of S. cerevisiae Ssf1; Hap43-induced; rat catheter and Spider biofilm induced
PIS54944.1	27.55917834	26.00454796	27.5419708	25.48173208	26.88383082	27.53998511	0.682437489	0.944	-0.4	PIS54944.1	UBP1	Ortholog(s) have cysteine-type deubiquitinase activity, role in protein deubiquitination and cytoplasm, endoplasmic reticulum, endoplasmic reticulum membrane localization
PIS51955.1	31.47432859	32.02040015	30.74021705	30.48407749	31.17248669	31.37582212	0.346376832	0.892	-0.401	PIS51955.1	SLK19	Alkaline-induced protein of plasma membrane; affects cell aggregation, cell wall; similar to S. cerevisiae Slk19p (a kinetochore protein with roles in mitosis, meiosis); required for wild-type virulence in mouse; macrophage-downregulated
PIS58514.1	28.78876838	28.47800773	28.77765852	28.09160769	28.47657804	28.2733695	0.278576993	0.867	-0.401	PIS58514.1	TIM23	Protein involved in mitochondrial matrix protein import
PIS49579.1	25.96723436	25.70463159	24.50528181	24.97179932	24.57908746	25.41942873	0.389458549	0.904	-0.402	PIS49579.1	orf19.5780	Putative protein of unknown function; Hap43p-repressed gene
PIS51867.1	26.04111245	26.39447405	25.66131982	25.29555587	25.85083107	25.74026147	0.298256924	0.875	-0.403	PIS51867.1	orf19.3763	Has domain(s) with predicted role in retrograde vesicle-mediated transport, Golgi to endoplasmic reticulum and membrane localization
PIS58505.1	29.71582571	30.615806	28.33832232	28.41450073	29.4684671	29.5769657	0.573758218	0.934	-0.403	PIS58505.1	VMA4	H+ transporting ATPase E chain; transcript regulated by Mig1; caspofungin repressed; protein level decreases in stationary phase cultures; rat catheter biofilm repressed
PIS51263.1	27.90725109	27.33771461	27.89702472	26.6754999	27.68037865	27.57390867	0.62024663	0.939	-0.404	PIS51263.1	HOF1	Protein involved in cytokinesis and DNA damage response; interacts genetically with Rad53p-dependent checkpoint; mutant is viable

PIS56635.1	24.31074674	24.42431147	24.13105706	23.87076266	23.93296957	23.84814456	0.319524233	0.883	-0.405	PIS56635.1	HST2	Putative histone deacetylase; role in regulation of white-opaque switch; Spider biofilm induced
PIS54815.1	26.68105944	26.5949786	26.77560038	25.76169201	26.4425589	26.62813136	0.637959415	0.94	-0.406	PIS54815.1	NIK1	Histidine kinase involved in a two-component signaling pathway that regulates cell wall biosynthesis; required for wild-type virulence in mouse systemic infection but not for wild-type growth or drug sensitivity/resistance; 9 HAMP domains
PIS58057.1	27.85860646	29.21677083	28.81356622	28.93474893	27.98397315	27.75000387	0.502103462	0.925	-0.407	PIS58057.1	TRX1	Thioredoxin; involved in response to reactive oxygen species; biofilm, benomyl, flucytosine, peroxide, Hap43 induced; amphotericin B, caspofungin repressed; induced by human neutrophils; macrophage-repressed gene
PIS48536.1	34.29853605	33.70764322	34.48029349	34.01876008	33.69846394	33.54614968	0.230454792	0.84	-0.408	PIS48536.1	ERG251	C-4 sterol methyl oxidase; role in ergosterol biosynthesis; Hap43-induced; ketoconazole-induced; amphotericin B, caspofungin repressed; possibly essential gene, disruptants not obtained by UAU1 method; Spider biofilm repressed
PIS49678.1	21.82431223	21.72372087	21.91758156	21.50985129	21.32216631	21.41021701	0.50380886	0.925	-0.408	PIS49678.1	orf19.3170	Ortholog(s) have mRNA binding activity
PIS52304.1	28.85307515	28.6369725	28.92263236	28.54072949	28.48203133	28.16428649	0.304620176	0.878	-0.409	PIS52304.1	WRS1	Putative tRNA-Trp synthetase; genes encoding ribosomal subunits, translation factors, tRNA synthetases are downregulated upon phagocytosis by murine macrophages
PIS58572.1	28.85226741	28.85083816	29.46484107	28.92274355	28.53346537	28.48310615	0.58769725	0.935	-0.41	PIS58572.1	orf19.783	Ortholog(s) have role in endocytosis, phospholipid translocation, retrograde vesicle-mediated transport, Golgi to endoplasmic reticulum, vacuole organization
PIS49555.1	37.26067946	37.21738118	37.24152193	36.85806827	36.90854837	36.71663354	0.136536886	0.74	-0.412	PIS49555.1	TEF2	Translation elongation factor 1-alpha; genes encoding ribosomal subunits, translation factors, and tRNA synthetases are downregulated upon phagocytosis by murine macrophage
PIS52005.1	27.67554103	27.84972888	27.79433465	27.75641881	27.11947051	27.2029567	0.606509835	0.937	-0.414	PIS52005.1	OPY2	Predicted transmembrane protein; role in cell wall biogenesis; required for Cek1 phosphorylation; Spider biofilm induced
PIS49462.1	31.23626514	31.43975499	31.35534237	30.67271778	31.01197768	31.10479944	0.28365717	0.869	-0.414	PIS49462.1	YBN5	P-loop ATPase with similarity to human OLA1 and bacterial YchF; Spider biofilm repressed

PIS58518.1	32.77426605	32.50893465	32.81960026	32.28394288	32.27901568	32.29523446	0.121475607	0.712	-0.415	PIS58518.1	ACC1	Putative acetyl-coenzyme-A carboxylases; regulated by Efg1; amphotericin B repressed; caspofungin repressed; 5'-UTR intron; gene used for strain identification by multilocus sequence typing; Hap43-induced; flow model biofilm repressed
PIS51555.1	31.55890841	31.70215713	31.15756211	30.48029948	31.27297035	31.41644076	0.525868643	0.928	-0.416	PIS51555.1	ATP7	Putative subunit of the F1F0-ATPase complex; colony morphology-related gene regulation by Ssn6; farnesol, macrophage-downregulated protein abundance; protein present in exponential and stationary yeast growth phases; Hap43-induced
PIS58051.1	26.99407045	24.32277151	27.52926541	26.6341891	25.80630355	25.15270054	0.602664549	0.937	-0.418	PIS58051.1	orf19.7618	Putative nucleolar protein with a predicted role in pre-18S rRNA processing; Plc1p-regulated; Spider biofilm induced
PIS52032.1	22.24957757	23.83704965	23.17028472	23.52523741	21.92307791	22.55307547	0.56879908	0.933	-0.419	PIS52032.1	ECM3	Has domain(s) with predicted role in transmembrane transport and membrane localization
PIS56820.1	29.21067518	29.20832502	28.98486461	28.84485293	28.59829988	28.70232358	0.160686211	0.776	-0.419	PIS56820.1	ERO1	Ortholog of S. cerevisiae Ero1; role in formation of disulfide bonds in the endoplasmic reticulum; fluconazole-induced; induced by Mnl1 under weak acid stress; Spider biofilm induced
PIS55804.1	32.71745494	33.35479409	32.56000545	32.45035382	32.42127394	32.50097288	0.207732641	0.823	-0.42	PIS55804.1	MSI3	Essential HSP70 family protein; required for fluconazole resistance and calcineurin-dependent transcription; interacts with Cgr1; transcript regulated by iron; rat catheter biofilm induced; farnesol repressed in biofilm; sumoylation target
PIS58277.1	33.35086011	33.22129628	33.07204801	32.36632454	32.98346771	33.02840603	0.382975206	0.902	-0.422	PIS58277.1	RPS6A	Ribosomal protein 6A; localizes to cell surface of yeast cells but not hyphae; repressed upon phagocytosis by murine macrophage; possibly essential; Hap43-induced; Spider biofilm repressed
PIS50405.1	32.98837252	32.80244528	33.25308338	32.66765968	32.55288339	32.55546487	0.224046544	0.836	-0.423	PIS50405.1	IDH1	Putative mitochondrial NAD-isocitrate dehydrogenase subunit 1; soluble protein in hyphae; protein level decrease in stationary phase cultures
PIS51794.1	31.76588518	31.80553606	31.22026677	30.11806312	31.76955732	31.63421879	0.408673475	0.908	-0.423	PIS51794.1	orf19.6220.4	Ribosomal 60S subunit protein; Spider biofilm repressed
PIS55770.1	33.27414009	33.65060243	32.98772296	32.89366107	32.83922633	32.91048023	0.561932077	0.932	-0.423	PIS55770.1	TPI1	Triose-phosphate isomerase; antigenic in mouse/human; mutation affects filamentation; macrophage-repressed; protein in exponential and stationary growth phase yeast; possibly essential; flow model biofilm induced; Spider biofilm repressed

PIS58204.1	31.4230923	32.49613929	31.22539804	31.42015022	31.0752778	31.37777901	0.468668224	0.919	-0.424	PIS58204.1	MDH1	Mitochondrial malate dehydrogenase; regulated by Mig1, Tup1, white-opaque switch, phagocytosis; induced in high iron; antigenic during murine and human infection; repressed in Spider biofilms by Bcr1, Tec1, Ndt80, Rob1, Brg1
PIS58693.1	28.87358037	29.15585203	29.4483007	28.87698167	28.67827554	28.64750979	0.553190818	0.931	-0.425	PIS58693.1	CDC55	Ortholog(s) have protein phosphatase regulator activity
PIS56570.1	26.96317102	26.06972491	26.22932385	25.17206916	26.56809639	26.24713491	0.425523307	0.911	-0.425	PIS56570.1	SEF1	Zn2-Cys6 transcription factor; regulates iron uptake; negatively regulated by Sfu1p, positively regulated by Tbf1; promotes virulence in mice; mutants display decreased colonization of mouse kidneys; Spider biofilm induced
PIS50537.1	29.27863604	28.12753445	29.11977841	28.23264226	28.52239279	28.49697832	0.570352502	0.933	-0.425	PIS50537.1	SFC1	Putative succinate-fumarate transporter; involved in repression of growth on sorbose; alkaline induced; rat catheter biofilm induced; Spider biofilm induced
PIS48642.1	25.85453237	27.10678342	25.43975076	25.39283527	26.05279951	25.67882991	0.624798876	0.939	-0.426	PIS48642.1	orf19.1624	Non-catalytic subunit of N-terminal acetyltransferase of the NatC type; flow model biofilm repressed
PIS51994.1	27.51257846	26.86524063	28.5053859	26.55275016	27.72986635	27.3199123	0.346892744	0.892	-0.427	PIS51994.1	MRPL19	Putative ribosomal protein; induced upon adherence to polystyrene; Spider biofilm repressed
PIS51586.1	29.00138866	28.69244719	29.0064819	28.07821488	28.6660101	28.66331228	0.257976003	0.856	-0.431	PIS51586.1	IDP1	Putative isocitrate dehydrogenase; transcriptionally induced by interaction with macrophage; alkaline induced; Spider biofilm repressed
PIS48771.1	26.94141103	26.48081415	27.35299664	26.33757442	26.69915016	26.4443509	0.511084771	0.926	-0.431	PIS48771.1	MCM2	Phosphorylated protein of unknown function; transcription is periodic with a peak at M/G1 phase of the cell cycle
PIS58545.1	32.05113532	31.92027565	31.99432338	31.60315891	31.5504853	31.51914733	0.314257631	0.881	-0.431	PIS58545.1	orf19.3349	Putative RNA polymerase II subunit B150; heterozygous null mutant exhibits resistance to parnafungin in the C. albicans fitness test
PIS51293.1	24.07860233	26.94078312	23.94867476	24.71909166	24.43927017	24.51532808	0.61842029	0.938	-0.431	PIS51293.1	STF2	Protein involved in ATP biosynthesis; repressed in hyphae; repressed by Efg1, Hap43; transcript upregulated in clinical isolates from HIV+ patients with oral candidiasis; rat catheter, flow model and Spider biofilm induced

PIS48664.1	29.06947649	29.62389788	28.59493391	28.85424626	28.57095636	28.56742359	0.44808937	0.916	-0.432	PIS48664.1	orf19.501	Ortholog(s) have rRNA (cytosine-C5-)-methyltransferase activity
PIS58374.1	33.01480887	32.80931714	32.87876186	32.3436914	32.49393278	32.56260513	0.28446222	0.869	-0.434	PIS58374.1	LAP3	Putative aminopeptidase; positively regulated by Sfu1; clade-associated gene expression; virulence-group-correlated expression; induced by alpha pheromone in SpiderM medium; Hap43-induced; Spider and flow model biofilm induced
PIS54552.1	27.60774048	28.05002531	28.21868251	27.89523806	27.1936973	27.48563156	0.298050082	0.875	-0.434	PIS54552.1	TIF34	Putative translation initiation factor eIF3, p39 subunit; mutation confers hypersensitivity to roridin A, verrucarin A; downregulated upon phagocytosis by murine macrophages; Spider biofilm repressed
PIS52103.1	28.9089644	29.24810385	29.40249541	28.34806883	28.87211246	29.03558173	0.423139755	0.911	-0.435	PIS52103.1	ALG2	Putative mannosyltransferase involved in cell wall mannan biosynthesis; transcription is elevated in chk1, nik1, and sln1 homozygous null mutants
PIS51304.1	27.49132546	28.70734338	26.17433374	27.49954534	27.15701944	26.41039165	0.445765105	0.915	-0.435	PIS51304.1	TFS1	Putative carboxypeptidase y inhibitor; transcript regulated upon yeast-hypha switch; colony morphology-related gene regulation by Ssn6
PIS51929.1	26.73774935	27.83130059	26.77948511	26.44682315	26.64561814	26.94259995	0.500338103	0.924	-0.438	PIS51929.1	orf19.36	Component of the conserved oligomeric Golgi complex; predicted to mediate fusion of transport vesicles to Golgi compartments; clade-associated gene expression
PIS58567.1	28.89976302	28.6857238	28.89994127	28.18005524	28.43517687	28.5523704	0.234198225	0.842	-0.439	PIS58567.1	ILV3	Dihydroxyacid dehydratase; repressed by nitric oxide; macrophage-induced protein; protein in exponential and stationary growth phase; Sef1, Sfu1p, Hap43-regulated; farnesol-repressed; flow model biofilm induced; Spider biofilm repressed
PIS58750.1	25.61740515	24.42430894	24.54925665	23.99407255	24.04996929	25.23025082	0.583652142	0.935	-0.439	PIS58750.1	NAM2	Mitochondrial leucyl-tRNA synthetase
PIS58612.1	30.90088154	32.06163466	30.71565124	31.08979929	30.52165972	30.7460803	0.365649696	0.897	-0.44	PIS58612.1	SNZ1	Stationary phase protein; vitamin B synthesis; induced byyeast-hypha switch, 3-AT or in azole-resistant strain overexpressing MDR1; soluble in hyphae; regulated by Gcn4, macrophage; Spider biofilm induced; rat catheter biofilm repressed
PIS59012.1	32.20681283	32.35793393	32.01253109	31.7806127	31.70296136	31.76640346	0.122122013	0.714	-0.442	PIS59012.1	HSP104	Heat-shock protein; roles in biofilm and virulence; complements chaperone, prion activity in S. cerevisiae; guanidine-insensitive; heat shock/stress induced; repressed in farnesol-treated biofilm; sumoylation target; Spider biofilm induced

PIS54627.1	29.04700405	30.66785696	28.82184767	28.65433441	29.06785546	29.48962925	0.391228338	0.904	-0.442	PIS54627.1	orf19.6612	Putative mitochondrial protein; Hap43p-induced gene
PIS56692.1	26.83546422	27.38261647	27.18069319	27.35648656	26.52758036	26.186917	0.409665889	0.908	-0.443	PIS56692.1	orf19.6809	Putative phosphomutase-like protein; protein present in exponential and stationary growth phase yeast; Hap43-repressed; Spider biofilm repressed
PIS48661.1	29.21093575	29.39675692	29.77583734	28.6037299	29.15725889	29.29129853	0.356080498	0.895	-0.444	PIS48661.1	orf19.498	Putative mitochondrial ribosomal component of the small subunit; possibly an essential gene, disruptants not obtained by UAU1 method; Spider biofilm repressed
PIS50585.1	26.17090942	23.48407112	25.75129252	24.77593018	23.38893504	25.9075685	0.625880545	0.939	-0.445	PIS50585.1	RLP24	Putative ribosomal protein; Hap43-induced; essential gene; heterozygous mutation confers hypersensitivity to 5-fluorocytosine (5-FC), 5-fluorouracil (5-FU), and tubercidin (7-deazaadenosine); Spider biofilm induced
PIS49740.1	28.78074039	28.86876453	28.78480854	28.46067979	28.11386665	28.52061646	0.524708036	0.928	-0.446	PIS49740.1	GCD2	Putative translation initiation factor; genes encoding ribosomal subunits, translation factors, and tRNA synthetases are downregulated upon phagocytosis by murine macrophage
PIS55733.1	26.40176483	24.7095688	26.81941221	25.91067082	25.62771319	25.0513407	0.739355441	0.948	-0.447	PIS55733.1	orf19.478	Protein required for fusion of cvt-vesicles and autophagosomes with the vacuole; plays a role in autophagy, protein targeting to vacuole and vesicle docking; flow model biofilm induced
PIS58975.1	23.34613815	24.42185743	24.33321414	22.7698482	22.8490685	25.1425477	0.426117314	0.912	-0.447	PIS58975.1	orf19.7095	Ortholog(s) have fluoride transmembrane transporter activity, role in cellular detoxification of fluoride, fluoride export across plasma membrane, fluoride transmembrane transport and plasma membrane localization
PIS50445.1	24.36779599	25.44409748	24.00925105	24.3971139	23.55870477	24.52458562	0.355873422	0.895	-0.447	PIS50445.1	PTC7	Protein phosphatase, type 2C; has S/T phosphatase activity, Mn2+/Mg2+ dependent; predicted membrane-spanning segment and mitochondrion-targeting signal
PIS52254.1	24.52048306	25.25327158	25.30510993	24.82705808	24.43702173	24.47249127	0.339059577	0.89	-0.447	PIS52254.1	SOG2	Leucine-rich-repeat domain protein of RAM cell wall integrity signaling network; role in cell separation, azole sensitivity; required for hyphal growth; lacks orthologs in higher eukaryotes
PIS54970.1	28.2465531	28.61804928	28.0080275	27.76516408	27.91413296	27.84806585	0.783914684	0.951	-0.448	PIS54970.1	orf19.6687	Protein of unknown function; rat catheter biofilm repressed

PIS54515.1	31.13445625	30.32964861	31.10523949	30.88040526	30.11024768	30.23134726	0.271328705	0.863	-0.449	PIS54515.1	DHH1	Putative RNA helicase
PIS52160.1	28.32327332	28.54053278	28.27827801	27.7494524	28.2193034	27.82646185	0.132001	0.732	-0.449	PIS52160.1	orf19.3128	Ortholog(s) have SNARE binding, syntaxin binding activity
PIS48743.1	28.46586137	29.021453	28.43851444	28.20649726	28.11708121	28.25207534	0.215521843	0.83	-0.45	PIS48743.1	orf19.7264	Metalloprotease subunit of the 19S regulatory particle of the 26S proteasome lid; couples the deubiquitination and degradation of proteasome substrates; role in fission of mitochondria and peroxisome; Spider biofilm repressed
PIS52366.1	25.05030584	25.07187555	24.3092966	24.03312363	24.45873777	24.58697051	0.259156634	0.857	-0.451	PIS52366.1	MDR1	Plasma membrane MDR/MFS multidrug efflux pump; methotrexate is preferred substrate; overexpression in drug-resistant clinical isolates confers fluconazole resistance; repressed in young biofilms; rat catheter biofilm induced
PIS51668.1	26.34898198	25.94742897	27.03809185	26.09285281	25.30347054	26.5845494	0.562283637	0.932	-0.451	PIS51668.1	PDE2	High affinity cyclic nucleotide phosphodiesterase; moderates signaling by cAMP; required for virulence, switching, cell wall, hyphal, not pseudohyphal growth; expressed shortly after hyphal induction; rat catheter and Spider biofilm induced
PIS48616.1	28.21699763	29.32258986	27.85068016	27.92463093	27.8731961	28.23561927	0.375181052	0.9	-0.452	PIS48616.1	orf19.1946	Similar to an aldose 1-epimerase-related protein; antigenic during murine systemic infection; protein present in exponential and stationary phase yeast cultures; Hap43-induced; Spider biofilm repressed
PIS51214.1	30.77255127	30.48008958	30.9347413	30.33196659	30.22393259	30.27462459	0.295434034	0.874	-0.452	PIS51214.1	TPK2	cAMP-dependent protein kinase (PKA) catalytic subunit; isoform of Tpk1; involved in regulation of filamentation, phenotypic switching and mating; needed for epithelial cell damage, engulfment and oral virulence in mice
PIS49676.1	29.02168505	29.15452453	29.29278761	28.16995549	29.03854814	28.90297748	0.270120211	0.863	-0.453	PIS49676.1	MDJ1	Putative member of the HSP40 (DnaJ) family of chaperones; rat catheter and Spider biofilm induced
PIS51965.1	27.49243901	27.55072636	27.10179629	26.85126993	26.3947005	27.53981483	0.469855934	0.92	-0.453	PIS51965.1	orf19.6742	Ortholog(s) have RNA polymerase II CTD heptapeptide repeat phosphatase activity, protein serine/threonine phosphatase activity, role in regulation of transcription by RNA polymerase II and cytosol, nucleus localization
PIS50485.1	25.56380084	24.97138494	24.04939844	24.6608356	24.48141128	24.08085803	0.329218038	0.886	-0.454	PIS50485.1	SFL2	Transcription factor involved in regulation of morphogenesis; regulates transcription in response to carbon dioxide levels; required for filamentous growth, for virulence in RHE model but not in mice; Spider biofilm induced

PIS56725.1	29.95570397	30.0897664	30.36686591	29.71743133	29.572679	29.76015435	0.159348124	0.774	-0.454	PIS56725.1	TUP1	Transcriptional corepressor; represses filamentous growth; regulates switching; role in germ tube induction, farnesol response; in repression pathways with Nrg1, Rfg1; farnesol upregulated in biofilm; rat catheter, Spider biofilm repressed
PIS55780.1	31.30122831	31.21702087	31.50610262	30.72606368	30.93082259	31.00317683	0.471695329	0.92	-0.455	PIS55780.1	orf19.1409.1	Ribosomal 60S subunit protein L22B; Spider biofilm repressed
PIS56823.1	28.82600198	28.9145269	27.83449345	28.1681476	28.02504703	28.01264865	0.418185591	0.91	-0.456	PIS56823.1	DAK2	Putative dihydroxyacetone kinase; repressed by yeast-hypha switch; fluconazole-induced; caspofungin repressed; protein enriched in stationary phase yeast cultures; flow model biofilm induced; rat catheter and Spider biofilm repressed
PIS48482.1	28.88849969	28.69940703	28.9981861	28.55740494	28.39516377	28.26584142	0.190655424	0.809	-0.456	PIS48482.1	orf19.2200	Protein of unknown function; rat catheter biofilm repressed
PIS49823.1	26.87647402	28.13125972	27.06789792	27.0056689	26.42243157	27.28070122	0.443764695	0.915	-0.456	PIS49823.1	orf19.4362	Ortholog(s) have membrane protein dislocase activity, role in protein targeting to mitochondrion and mitochondrial outer membrane, peroxisomal membrane localization
PIS49577.1	29.98642575	29.87964778	29.64637348	29.29954795	29.44557102	29.39705271	0.230732054	0.84	-0.457	PIS49577.1	RNR1	Ribonucleotide reductase large subunit; induced in low iron; transposon mutation affects filamentous growth; farnesol upregulated in biofilm; regulated by cell cycle, tyrosol, cell density; regulated by Sef1, Sfu1, and Hap43
PIS52475.1	27.55556742	28.50965988	28.3574733	27.00977997	27.97761646	28.06018617	0.54945015	0.931	-0.458	PIS52475.1	orf19.2820	Ortholog(s) have structural constituent of nuclear pore activity and role in NLS-bearing protein import into nucleus, nuclear pore organization, protein import into nucleus, protein localization to nuclear inner membrane
PIS48377.1	24.87409407	25.13244631	25.13602891	24.71674789	24.34371753	24.7074618	0.26162405	0.858	-0.458	PIS48377.1	orf19.3633	Has domain(s) with predicted hydroxyisourate hydrolase activity and role in purine nucleobase metabolic process
PIS51450.1	28.228562	28.01187036	27.47904311	27.43497669	27.60100273	27.31080125	0.263820627	0.859	-0.458	PIS51450.1	PES1	Pescadillo homolog required for dispersal of biofilm cells into planktonic yeast cells; essential in yeast cells, not in hyphal cells; mutation confers hypersensitivity to 5-fluorocytosine, 5-fluorouracil, tubercidin
PIS58997.1	33.68787527	33.12480618	34.05240938	33.25671877	33.01495997	33.21275066	0.443592223	0.915	-0.46	PIS58997.1	AAT21	Putative aspartate aminotransferase; stationary phase enriched protein; Gcn4-regulated; Spider biofilm induced

PIS58887.1	26.05771453	25.66671882	27.26203693	26.28554142	25.87091065	25.45029258	0.423276541	0.911	-0.46	PIS58887.1	ERV1	Predicted component of the mitochondrial intermembrane space (IMS), involved in protein import into mitochondrial intermembrane space
PIS56688.1	35.62541685	35.30215277	35.477468	35.04313915	34.97791724	35.00263772	0.327895458	0.886	-0.46	PIS56688.1	TDH3	NAD-linked glyceraldehyde-3-phosphate dehydrogenase; binds fibronectin, laminin; at cell surface; antigenic in infection; farnesol-repressed; stationary phase-enriched; GlcNAc-induced; flow model biofilm induced; Spider biofilm repressed
PIS49834.1	28.12900122	27.6355632	27.77208667	27.40438254	27.4757885	27.27213998	0.212861266	0.827	-0.461	PIS49834.1	CTF1	Putative zinc-finger transcription factor, similar to A. nidulans FarA and FarB; activates genes required for fatty acid degradation; induced by oleate; null mutant displays carbon source utilization defects and slightly reduced virulence
PIS49732.1	30.68194286	30.21876108	30.65991354	30.1388028	30.04084468	29.99929382	0.608557774	0.937	-0.461	PIS49732.1	orf19.6787	Ortholog(s) have cargo receptor activity and role in ascospore formation, axial cellular bud site selection, endoplasmic reticulum to Golgi vesicle-mediated transport
PIS58380.1	28.41178517	27.9099734	28.0545923	27.84588931	27.48279621	27.66323087	0.409777875	0.908	-0.461	PIS58380.1	orf19.6883	Putative oxysterol binding protein; probable peripheral membrane protein of the Golgi complex; involved in invasive growth and plasma membrane organization; flow model and Spider biofilm repressed
PIS58608.1	24.18718981	24.28811191	24.4775134	24.09590961	24.59435859	22.87578205	0.506781614	0.925	-0.462	PIS58608.1	EXG2	GPI-anchored cell wall protein, similar to S. cerevisiae exo-1,3-beta-glucosidase Exg2p; predicted Kex2p substrate; induced during cell wall regeneration; possibly an essential gene, disruptants not obtained by UAU1 method; Hap43p-repressed
PIS55593.1	27.9433692	27.25751132	28.25300646	27.77063094	27.37013131	26.92719028	0.296283956	0.874	-0.462	PIS55593.1	PTC4	Type PP2C serine/threonine phosphatase; localized to mitochondria; mutation causes sensitivity to sodium, potassium and azole drugs; decreased expression in hyphae compared to yeast-form cells
PIS58373.1	27.28018561	27.3302259	27.97324532	27.44709403	26.92820783	26.81928362	0.275285715	0.865	-0.463	PIS58373.1	orf19.809	Ortholog(s) have rRNA binding activity, role in maturation of LSU-rRNA from tricistronic rRNA transcript (SSU-rRNA, 5.8S rRNA, LSU-rRNA) and nucleolus, preribosome localization
PIS54524.1	32.43953309	32.89681395	32.77019267	32.51461267	31.92620585	32.27484931	0.445836418	0.915	-0.464	PIS54524.1	CAT1	Catalase; resistance to oxidative stress, neutrophils, peroxide; role in virulence; regulated by iron, ciclopirox, fluconazole, carbon source, pH, Rim101, Ssn6, Hog1, Hap43, Sfu1, Sef1, farnesol, core stress response; Spider biofilm induced
PIS58066.1	28.26940281	28.38543039	28.61735254	28.07619488	27.71594494	28.08622266	0.235205322	0.843	-0.465	PIS58066.1	orf19.3459	Putative serine/threonine/tyrosine (dual-specificity) kinase; disruptants not obtained by UAU1 method

PIS49707.1	31.65100785	31.30472398	31.13800968	31.0283371	30.89570975	30.76953591	0.297852384	0.875	-0.467	PIS49707.1	orf19.7052	Putative polyphosphatidylinositol phosphatase; possibly an essential gene, disruptants not obtained by UAU1 method
PIS50355.1	29.76366188	30.17684486	29.7004264	29.23309514	29.59015259	29.41295778	0.125534232	0.72	-0.468	PIS50355.1	DBP2	Putative DEAD-box family ATP-dependent RNA helicase; flucytosine induced; repressed in core stress response
PIS52264.1	26.99332377	26.66079907	26.65035409	26.38102392	26.34412471	26.17623829	0.278254591	0.866	-0.468	PIS52264.1	orf19.5184	Putative Ran guanyl-nucleotide exchange factor; probable signal transducer; Spider biofilm repressed
PIS54770.1	28.40120671	28.35488893	28.37419834	27.59250259	27.77796719	28.35352518	0.296497351	0.874	-0.469	PIS54770.1	orf19.4230	20S proteasome subunit (beta7); protein present in exponential and stationary growth phase yeast cultures
PIS48622.1	26.63208304	27.06937903	24.80710327	26.10864067	25.84146294	25.15186734	0.488231797	0.923	-0.469	PIS48622.1	orf19.6976	Predicted MFS membrane transporter; member of the proton coupled folate transporter/heme carrier protein family; virulence-group-correlated expression; Spider biofilm induced
PIS51640.1	26.59347974	25.60144619	26.00566764	26.07611378	25.42780617	25.28510986	0.406201225	0.907	-0.471	PIS51640.1	orf19.6048	Protein of unknown function; Spider biofilm induced
PIS52239.1	28.38792041	28.13197812	28.24374121	28.0414075	27.62457243	27.68310413	0.271378121	0.863	-0.472	PIS52239.1	orf19.1441	Component of the RSC chromatin remodeling complex; only present in CTG clade
PIS52171.1	26.83088852	26.25381702	26.52437006	26.63974494	26.17345822	25.3794993	0.42051849	0.91	-0.472	PIS52171.1	orf19.3098	Predicted RNA-dependent ATPase RNA helicase; Hap43-induced gene
PIS52261.1	25.61437609	23.32050303	24.79566493	24.85692926	24.20976337	23.24654825	0.501904993	0.925	-0.472	PIS52261.1	orf19.5504	Ortholog of C. dubliniensis CD36 : Cd36_73400, C. parapsilosis CDC317 : CPAR2_703180, C. auris B8441 : B9J08_003875 and Candida tenuis NRRL Y-1498 : CANTEDRAFT_104803
PIS56632.1	28.34497468	27.30103255	29.13448536	28.32070442	27.57430429	27.46581614	0.463465562	0.919	-0.473	PIS56632.1	URE2	Functional homolog of S. cerevisiae Ure2p, which is a regulator of nitrogen utilization, and which also has an infectious prion form called [URE3]; forms [URE3] prion when expressed in S. cerevisiae

PIS55809.1	30.1952728	30.07438299	30.31219844	29.57323771	29.94191598	29.64518319	0.284157208	0.869	-0.474	PIS55809.1	NOG1	Putative GTPase; mutation confers hypersensitivity to 5-fluorocytosine (5-FC), 5-fluorouracil (5-FU), and tubercidin (7-deazaadenosine); repressed by prostaglandins; Hap43-induced
PIS48688.1	30.45630667	29.82105167	30.22246011	29.46173728	29.877951	29.73589074	0.195414777	0.813	-0.475	PIS48688.1	orf19.3430	Plasma membrane-associated protein; physically interacts with TAP-tagged Nop1p
PIS52306.1	26.58218565	24.87934916	25.29107429	26.21526571	23.74118128	25.36755435	0.558982125	0.932	-0.476	PIS52306.1	PKH2	Probable serine/threonine protein kinase; predicted role in sphingolipid-mediated signaling pathway that controls endocytosis; mRNA binds She3 and is localized to hyphal tips; appears redundant with Pkh3 within the Ypk1 signaling pathway
PIS49722.1	29.22559348	29.27916536	29.29380722	28.38557581	28.96807599	29.01092128	0.253665488	0.854	-0.478	PIS49722.1	SSN6	Functional homolog of S. cerevisiae Cyc8/Ssn6; hyphal growth regulator; repressed during hyphal growth; Ssn6 and Tup1 regulate distinct sets of genes; overexpression or mutation causes avirulence in mouse IV infection; TPR motifs
PIS52253.1	28.64859833	29.05315193	28.8956375	28.10583449	28.4872424	28.56598906	0.774032874	0.951	-0.479	PIS52253.1	orf19.1461	S. pombe ortholog SPCC576.01c is a predicted sulfonate dioxygenase; possibly transcriptionally regulated upon hyphal formation; Spider biofilm induced
PIS48336.1	23.70256429	24.00658349	23.64378261	22.36709255	23.85281347	23.69491645	0.683022185	0.944	-0.479	PIS48336.1	orf19.5449	Predicted integral membrane protein; Spider biofilm induced
PIS58994.1	24.37071769	25.8626035	22.64348355	24.69696894	23.80690305	22.93309301	0.611653547	0.938	-0.48	PIS58994.1	orf19.2887	Ortholog in S. cerevisiae is localizedto the bud, mating projection tip, and associates with ribosomes; Spider biofilm induced
PIS50545.1	28.04286522	27.10103409	27.19448085	25.95141283	27.43839193	27.50849088	0.700760055	0.946	-0.48	PIS50545.1	orf19.899	Ortholog of C. dubliniensis CD36 : Cd36_18040, C. parapsilosis CDC317 : CPAR2_211790, C. auris B8441 : B9J08_004373 and Candida tenuis NRRL Y-1498 : CANTEDRAFT_109928
PIS50633.1	30.22150628	29.41923078	30.49483085	29.73436976	29.48724693	29.4723538	0.475422011	0.921	-0.481	PIS50633.1	PHB2	Prohibitin 2; plasma membrane localized
PIS55637.1	32.22864066	32.65950728	32.54560965	31.80964582	31.73756592	32.4439064	0.127616199	0.724	-0.481	PIS55637.1	PXP2	Putative acyl-CoA oxidase; enzyme of fatty acid beta-oxidation; induced during macrophage infection; opaque specific transcript; putative peroxisome targeting signal; Spider biofilm induced

PIS48242.1	29.58134863	30.42696943	29.91393835	29.25050629	29.31937876	29.90835501	0.198354243	0.816	-0.481	PIS48242.1	TOM40	Protein involved in mitochondrial protein import; Spider biofilm repressed
PIS49657.1	26.60543914	28.66880985	26.02072012	25.81363247	26.21438138	27.82141995	0.583337013	0.935	-0.482	PIS49657.1	ECM42	Ornithine acetyltransferase; Gcn2, Gcn4-regulated; clade-specific gene expression; possibly essential gene, disruptants not obtained by UAU1 method; Spider biofilm induced
PIS56875.1	28.70250967	28.63915698	28.81895299	27.91704342	28.26285453	28.53586979	0.153006788	0.766	-0.482	PIS56875.1	IDI1	Ortholog(s) have isopentenyl-diphosphate delta-isomerase activity and role in ergosterol biosynthetic process, farnesyl diphosphate biosynthetic process
PIS54579.1	24.4252685	23.95850991	25.13121076	24.90560353	22.78106363	24.38235115	0.491259726	0.923	-0.482	PIS54579.1	orf19.3158	Ortholog of S. cerevisiae : RMD1, C. glabrata CBS138 : CAGL0M10483g, C. dubliniensis CD36 : Cd36_81090, C. parapsilosis CDC317 : CPAR2_101950 and C. auris B8441 : B9J08_002355
PIS51995.1	22.94965911	26.48654207	23.00989132	23.01554343	24.12695867	23.85740057	0.565037528	0.933	-0.482	PIS51995.1	orf19.4906	Putative adhesin-like protein; positively regulated by Tbf1; Spider biofilm induced
PIS55671.1	30.16444319	29.20886049	29.97600561	29.56952427	29.28527248	29.04659233	0.62572841	0.939	-0.483	PIS55671.1	ATO7	Putative fungal-specific transmembrane protein
PIS55708.1	28.91446073	28.01113938	28.88947325	28.00733223	28.27575081	28.07975833	0.142513187	0.75	-0.484	PIS55708.1	orf19.4437	Ortholog(s) have ATPase, DNA binding, mRNA 3'-UTR binding, nucleosome binding, rDNA binding, transcription cis-regulatory region binding activity
PIS48568.1	28.00697997	28.10602055	27.70540008	27.94792782	26.69540448	27.72210609	0.371143518	0.899	-0.484	PIS48568.1	orf19.5274	Protein involved in regulation of ergosterol biosynthesis genes; null mutation causes increased sensitivity to azoles and abnormal hyphal growth
PIS58548.1	21.90524759	21.2876072	21.91652145	22.29725599	21.72684433	19.62967784	0.680400134	0.944	-0.485	PIS58548.1	SIZ1	Possible SUMO/Smt3 ligase; Rim101-repressed
PIS51588.1	24.28368435	24.4543742	23.90733408	23.14993655	24.22240533	23.81355875	0.226315876	0.837	-0.486	PIS51588.1	orf19.5213	Putative protein of unknown function; transcript is upregulated in clinical isolates from HIV+ patients with oral candidiasis

PIS54977.1	29.49888616	29.33050468	29.80874156	29.10289401	28.92030585	29.15461509	0.275963059	0.865	-0.487	PIS54977.1	GDA1	Golgi membrane GDPase/UDPase, required for wild-type O-mannosylation, not N-glycosylation; required for wild-type hyphal induction, cell wall, and cell surface charge; functional homolog of S. cerevisiae Gda1
PIS51667.1	25.16767737	25.5843286	24.99972304	24.33076376	24.43311791	25.52715968	0.451259873	0.916	-0.487	PIS51667.1	orf19.2973	Ortholog(s) have RNA binding, flap-structured DNA binding activity and role in nuclear-transcribed mRNA catabolic process, nuclear-transcribed mRNA poly(A) tail shortening, positive regulation of DNA metabolic process
PIS49532.1	25.65704561	25.15030978	25.24653723	25.69807831	24.49444232	24.3907884	0.429029984	0.912	-0.49	PIS49532.1	orf19.4731	Ortholog(s) have role in cytoplasm to vacuole transport by the Cvt pathway, intra-Golgi vesicle-mediated transport and Golgi transport complex localization
PIS51120.1	27.91844331	27.70752575	28.41459099	27.49734453	27.20880925	27.86147505	0.342104392	0.891	-0.491	PIS51120.1	orf19.7204	Has domain(s) with predicted nitronate monooxygenase activity
PIS51198.1	35.06490279	35.02866226	35.18471265	34.61662926	34.6150277	34.57147691	0.091542273	0.635	-0.492	PIS51198.1	EFT2	Elongation Factor 2 (eEF2); GTPase; essential; highly expressed; target of sordarin antifungals; antigenic in human/mouse; lacks site for regulatory phosphorylation by eEF2 kinase; GCN-regulated; higher protein amount in stationary phase
PIS56757.1	29.75396133	28.83059875	29.83530651	29.40746525	28.89062	28.64345578	0.375382908	0.9	-0.493	PIS56757.1	AFL1	Predicted inositol polyphosphate kinase, involved in autophagy, energy metabolism, virulence
PIS48675.1	26.9441384	26.78167811	26.50864714	26.09026937	26.12639569	26.53906144	0.191490917	0.809	-0.493	PIS48675.1	SEC12	Putative guanyl-nucleotide exchange factor; induced in high iron; Hap43-repressed
PIS49764.1	20.52699749	20.21007359	21.24891975	21.3743957	19.58151737	19.54477847	0.678969948	0.944	-0.495	PIS49764.1	MSH3	Mismatch repair protein; predicted role in repair of insertion or deletion mutations and removal of nonhomologous DNA ends; rat catheter biofilm repressed
PIS49580.1	28.69619142	27.81045297	28.42536866	27.67974473	27.95661066	27.80962603	0.110317925	0.687	-0.495	PIS49580.1	orf19.5689	Epsilon-COP subunit of the coatomer; regulates retrograde Golgi-to-ER protein traffic; Spider biofilm repressed
PIS51772.1	26.63504583	27.35504304	27.20764167	27.01101266	26.32184716	26.37708393	0.211475918	0.826	-0.496	PIS51772.1	GPM2	Putative phosphoglycerate mutase; repressed in hyphae; macrophage/pseudohyphal-repressed; induced by high levels of peroxide stress, farnesol; flow model biofilm induced; rat catheter and Spider biofilm repressed

PIS58581.1	29.04763519	28.7528875	29.22896141	28.59850783	28.22950119	28.71306654	0.298077772	0.875	-0.496	PIS58581.1	orf19.5194.1	Putative protein of unknown function; clade-associated gene expression
PIS52077.1	25.90153475	27.29861233	25.631608	24.04246848	26.71955145	26.57453626	0.561605104	0.932	-0.498	PIS52077.1	orf19.7546	Protein involved in rRNA processing; required for maturation of the 35S primary transcript of pre-rRNA and for cleavage leading to mature 18S rRNA; Spider biofilm induced
PIS50415.1	25.79422117	26.4654998	25.89875102	26.22815217	25.08795299	25.34493219	0.539407179	0.93	-0.499	PIS50415.1	orf19.5093	Component of the RSC chromatin remodeling complex
PIS48386.1	26.71353606	26.47024577	27.46332462	26.51693549	26.29472335	26.3337731	0.525284554	0.928	-0.501	PIS48386.1	TIM22	Mitochondrial inner membrane protein; predicted role in protein import; Hap43-repressed gene; flow model biofilm induced; Spider biofilm repressed
PIS58885.1	28.31866064	24.12305948	28.92453544	26.63049161	26.64898577	26.58498215	0.662989446	0.942	-0.501	PIS58885.1	YMC2	Putative mitochondrial carrier protein; Gcn4-regulated; F-12/ CO2 early biofilm induced; Spider biofilm induced
PIS54829.1	27.68371001	27.17764866	27.80975154	27.19895807	26.88272876	27.08335877	0.521540475	0.927	-0.502	PIS54829.1	orf19.6318	Has domain(s) with predicted membrane localization
PIS50303.1	28.15884113	28.28687338	28.40311378	28.04087882	27.91728025	27.37822201	0.230253129	0.84	-0.504	PIS50303.1	CKA1	Putative alpha subunit (catalytic subunit) of protein kinase CK2; Cka1p and Cka2p have a common target with respect to fluconazole resistance; synthetically lethal with CKA2; flucytosine induced
PIS56876.1	25.81850544	25.98662442	25.85927041	25.54379038	25.21582758	25.39333622	0.444445418	0.915	-0.504	PIS56876.1	LAB5	Ortholog(s) have role in protein lipoylation
PIS51314.1	24.6671699	25.07888209	25.07422364	25.38917335	24.75857242	23.16191656	0.540238173	0.93	-0.504	PIS51314.1	orf19.4901	Predicted methyltransferase; Spider biofilm induced
PIS58656.1	33.67053553	34.50372955	33.28176036	33.19650221	33.36010322	33.38754745	0.219576126	0.832	-0.504	PIS58656.1	SSB1	HSP70 family heat shock protein; mRNA in yeast and germ tubes; at yeast cell surface, not hyphae; antigenic in human/mouse infection; macrophage, GCN-induced; possibly essential; sumoylation target; Hap43-induced; Spider biofilm repressed

PIS51185.1	31.25270364	31.94995444	31.00607531	30.93350886	30.79827091	30.96046956	0.363529588	0.897	-0.505	PIS51185.1	orf19.7590	Putative NADH-ubiquinone oxidoreductase; identified in detergent-resistant membrane fraction (possible lipid raft component); predicted N-terminal acetylation; repressed by nitric oxide
PIS55080.1	26.72330249	27.82114473	24.96689594	25.31996508	25.86533101	26.79402568	0.510362112	0.926	-0.511	PIS55080.1	CBF1	Transcription factor; binds ribosomal protein gene promoters and rDNA locus with Tbf1; regulates sulfur starvation-response, respiratory, glycolytic genes; does not bind to centromeres as does S. cerevisiae Cbf1; Spider biofilm repressed
PIS51068.1	28.72708766	28.81882135	29.21655884	28.59175008	28.33303551	28.30490361	0.503981216	0.925	-0.511	PIS51068.1	orf19.1625	Putative ubiquinone oxidoreductase; repressed by nitric oxide; Hap43p-repressed
PIS48343.1	28.50613988	29.09405942	27.95345576	27.95269853	28.08461911	27.983839	0.391402016	0.904	-0.511	PIS48343.1	PR26	Protein involved in resistance to caspofungin and anidulafungin; has similarity to proteasomal 26S regulatory subunit of S. cerevisiae, H. sapiens, Methanobacterium thermoautotrophicum (Archaebacterium)
PIS51045.1	27.1681841	28.89363794	26.31526508	26.94996451	26.54813328	27.34211382	0.608688769	0.938	-0.512	PIS51045.1	BMS1	Putative GTPase; Hap43-induced gene; mutation confers resistance to 5-fluorocytosine (5-FC); flucytosine induced; repressed by prostaglandins; Spider biofilm induced
PIS58884.1	19.96798998	21.10608617	20.21518547	19.38431509	19.9532626	20.4145913	0.359072171	0.896	-0.512	PIS58884.1	SFU1	GATA-type transcription factor; regulator of iron-responsive genes; represses iron utilization genes if iron is present; Hap43-repressed; promotes gastrointestinal commensalism in mice; Spider biofilm induced
PIS51508.1	25.11536636	25.33682057	25.40075031	24.29032745	24.75775559	25.26210874	0.539863379	0.93	-0.514	PIS51508.1	orf19.5987	Ortholog(s) have tRNA (guanine(10)-N2)-methyltransferase activity, role in mRNA methylation, tRNA methylation and cytoplasm, tRNA (m2G10) methyltransferase complex localization
PIS59027.1	31.9557093	31.42314724	31.84651128	31.37422619	31.20032957	31.10729795	0.212266213	0.827	-0.515	PIS59027.1	MYO2	Class V myosin; nonessential; sole class V myosin in C. albicans; required for WT actin cytoskeletal polarity, nuclear organization, migration, hyphal growth; conserved myosin ATPase/tail domains; Hap43-induced; flow model biofilm repressed
PIS49526.1	22.48329407	22.69351185	22.2529594	22.79641376	21.43371229	21.65309782	0.569692241	0.933	-0.516	PIS49526.1	KEX2	Subtilisin-like protease (proprotein convertase); processes aspartyl proteinase Sap2; required for hyphal growth and wild-type virulence in mice; required for maturation of candidalysin Ece1p
PIS51220.1	25.80412191	25.71383992	24.24608818	25.46288474	24.63351905	24.11954466	0.632521166	0.94	-0.516	PIS51220.1	RFA2	Putative DNA replication factor A; RNA abundance regulated by cell cycle, tyrosol and cell density

PIS58496.1	33.43217422	33.35091937	33.54844859	32.89987513	32.9051115	32.9759951	0.077721778	0.586	-0.517	PIS58496.1	TIF	Translation initiation factor; upregulated in highly virulent strain compared to less virulent strain; antigenic in human; flucytosine induced; downregulated upon phagocytosis by macrophages; Spider biofilm repressed
PIS55718.1	30.6407191	29.39642994	30.59427769	30.10094167	29.61462114	29.358846	0.317096604	0.882	-0.519	PIS55718.1	ARC35	Putative ARP2/3 complex subunit; shows colony morphology-related gene regulation by Ssn6p; mutation confers hypersensitivity to cytochalasin D
PIS55704.1	29.72358756	29.25388212	29.67525668	28.88157499	29.04002985	29.17311987	0.139309281	0.745	-0.519	PIS55704.1	SOU1	Enzyme involved in utilization of L-sorbose; has sorbitol dehydrogenase, fructose reductase, and sorbose reductase activities; NAD-binding site motif; transcriptional regulation affected by chromosome 5 copy number; Hap43p-induced gene
PIS52212.1	28.78980723	29.25984937	29.26302152	28.56760757	28.58671064	28.59798341	0.11794723	0.705	-0.52	PIS52212.1	orf19.1356	Ortholog(s) have thiosulfate sulfurtransferase activity and role in tRNA wobble position uridine thiolation, tRNA wobble uridine modification
PIS52364.1	28.60468325	28.36553555	28.67821846	28.34796226	27.82136671	27.91869839	0.233812451	0.842	-0.52	PIS52364.1	orf19.5809	Putative arylformamidase, enzyme of the NAD biosynthesis pathway; Gcn4p-regulated
PIS54858.1	30.13355291	29.83599446	29.77668497	29.04685928	29.55333278	29.58523977	0.448155618	0.916	-0.52	PIS54858.1	orf19.6951	Ortholog(s) have sphinganine-1-phosphate aldolase activity and role in calcium-mediated signaling, cellular response to starvation, sphingolipid metabolic process
PIS51452.1	23.27084526	23.83085436	24.65412717	22.69303906	23.81814611	23.68288989	0.316321026	0.882	-0.521	PIS51452.1	orf19.4097	Ortholog of C. dubliniensis CD36 : Cd36_23480, C. parapsilosis CDC317 : CPAR2_407050, C. auris B8441 : B9J08_003033 and Candida tenuis NRRL Y-1498 : CANTEDRAFT_116822
PIS58786.1	24.15014505	24.36890797	25.21230586	24.1154342	23.96726655	24.08704692	0.209412748	0.825	-0.521	PIS58786.1	SFT2	Putative membrane protein; transcript regulated by Mig1; Spider biofilm induced
PIS58375.1	30.38188146	28.93440102	30.21623964	29.53201877	29.1838412	29.24950648	0.591872241	0.936	-0.522	PIS58375.1	COG4	Ortholog(s) have role in cytoplasm to vacuole transport by the Cvt pathway, intra-Golgi vesicle-mediated transport, macroautophagy, pexophagy, retrograde transport, vesicle recycling within Golgi and Golgi transport complex localization
PIS54499.1	30.90718618	30.49899558	31.50103079	30.77043381	30.30737782	30.2631033	0.285170152	0.87	-0.522	PIS54499.1	PHB1	Putative prohibitin; identified in detergent-resistant membrane fraction (possible lipid raft component); predicted N-terminal acetylation; Hap43p-repressed gene

PIS51582.1	28.88750149	29.27000164	28.48158283	28.48952426	28.07585439	28.49904566	0.18034627	0.798	-0.525	PIS51582.1	orf19.5206	Putative chaperone protein; role in the assembly of box H/ACA snoRNPs and thus for pre-rRNA processing; Spider biofilm induced
PIS58161.1	31.64477401	31.9087499	31.16688814	30.69076026	31.13681997	31.30370295	0.466950916	0.919	-0.53	PIS58161.1	LSC1	Putative succinate-CoA ligase subunit; induced by high iron; fluconazole-induced; protein present in exponential and stationary growth phase yeast cultures; Spider biofilm repressed
PIS52230.1	27.48291718	27.27995921	26.37726865	24.49521208	27.63624659	27.41845361	0.615672904	0.938	-0.53	PIS52230.1	TRM9	Putative tRNA methyltransferase; repressed during the mating process
PIS52188.1	25.77127887	26.26456362	26.20801109	26.29666982	24.0793648	26.27549513	0.36152267	0.896	-0.531	PIS52188.1	orf19.1776	Putative pantetheine-phosphate adenylyltransferase (PPAT); which catalyzes 4th step in coenzyme A biosynthesis from pantothenate; rat catheter biofilm repressed
PIS54844.1	29.13116815	28.86480398	29.65036381	28.97014854	28.62175338	28.45834367	0.337732156	0.889	-0.532	PIS54844.1	NPT1	Putative nicotinate phosphoribosyltransferase, involved in NAD salvage pathway; fungal-specific (no human or murine homolog)
PIS51906.1	30.97278082	31.05159109	30.79768347	30.22343629	30.43464677	30.56731117	0.280210999	0.867	-0.532	PIS51906.1	SUB2	Putative TREX complex component with a predicted role in nuclear mRNA export; transcription is regulated by Mig1; Hap43-induced gene; Spider biofilm repressed
PIS49841.1	26.72583687	28.31094543	26.70867567	27.26816085	26.52267605	26.35329428	0.297672771	0.875	-0.534	PIS49841.1	LIG1	tRNA ligase; functional homolog of S. cerevisiae Trl1
PIS58677.1	28.21874341	29.03961002	28.30966582	28.0732614	28.05083352	27.84165136	0.33015342	0.887	-0.534	PIS58677.1	orf19.6411	Protein similar to S. cerevisiae Vac14p; a protein involved in regulated synthesis of PtdIns(3,5)P(2); transposon mutation affects filamentous growth
PIS58798.1	29.19653212	29.48912858	28.04162744	28.11198771	28.19263614	28.81635412	0.311273089	0.88	-0.535	PIS58798.1	orf19.2335	Putative aspartyl aminopeptidase; stationary phase enriched protein; mutation confers hypersensitivity to 5-fluorouracil (5-FU); Hog1-induced; planktonic growth-induced; rat catheter and Spider biofilm repressed
PIS58294.1	29.00262396	29.77711784	29.30833331	28.88558041	28.83632048	28.7575043	0.237707537	0.845	-0.536	PIS58294.1	HTS1	Putative tRNA-His synthetase; downregulated upon phagocytosis by murine macrophage; stationary phase enriched protein; Spider biofilm repressed

PIS51066.1	27.29928536	27.52921831	27.61688141	27.63623685	26.32933395	26.87015666	0.26064568	0.858	-0.537	PIS51066.1	LAP41	Putative aminopeptidase yscI precursor; mutant is viable; protein present in exponential and stationary growth phase yeast cultures; Spider biofilm repressed
PIS48221.1	30.43113988	29.82343485	30.95201715	30.04313946	29.6205201	29.9282393	0.34990935	0.893	-0.538	PIS48221.1	ECI1	Protein similar to S. cerevisiae Eci1p, which is involved in fatty acid oxidation; transposon mutation affects filamentous growth; expression is regulated upon white-opaque switching
PIS48579.1	26.37058859	26.63725797	26.07457819	25.96989561	25.8470303	25.65015715	0.258411552	0.857	-0.538	PIS48579.1	orf19.5702	Ortholog(s) have role in diacylglycerol metabolic process, response to osmotic stress, vacuole fusion and vacuole-mitochondrion membrane contact site localization
PIS58717.1	25.84084736	27.71040668	25.48297927	24.96207841	25.96832643	26.48681056	0.368173919	0.898	-0.539	PIS58717.1	RUB1	modulates the ubiquitin ligase activity of the SCF complexes
PIS58477.1	32.52725385	33.05834168	32.13929364	31.84083946	32.07705101	32.18788985	0.339003969	0.89	-0.54	PIS58477.1	LPD1	Putative dihydrolipoamide dehydrogenase; soluble in hyphae; antigenic in human oral infection and murine systemic infection; macrophage-induced protein; protein present in exponential and stationary phase yeast cultures; Hap43p-repressed
PIS51210.1	26.61205198	26.3075391	25.60953817	25.96525014	25.39542624	25.54995088	0.355402142	0.895	-0.54	PIS51210.1	orf19.2278	Putative 20S proteasome assembly protein; filament induced; induced by alpha pheromone in SpiderM medium
PIS58547.1	26.36639527	25.49049682	25.33520496	24.06760249	25.73221484	25.77317746	0.315026372	0.882	-0.54	PIS58547.1	RPB7	Functional homolog of S. cerevisiae Rpb7; essential subunit of RNA Polymerase II; enhances hyperfilamentation of an S. cerevisiae rpb4 mutant; suppresses defects of S. cerevisiae rpb4 and ess1 mutants; Spider biofilm repressed
PIS54879.1	26.90230536	27.36983599	26.28921241	26.91191718	25.71086122	26.30923135	0.42579621	0.912	-0.543	PIS54879.1	GRX3	Putative glutaredoxin; flucytosine induced; regulated by Gcn4p; repressed in response to amino acid starvation (3-aminotriazole treatment)
PIS51380.1	25.87643906	24.51402224	25.5533877	25.06166187	24.78194489	24.47184113	0.412549135	0.909	-0.543	PIS51380.1	orf19.6874	Putative helix-loop-helix (HLH) transcription factor with a role in filamentous growth
PIS51172.1	29.22873238	28.86481255	28.5868757	28.14275954	28.45577661	28.45198274	0.119412864	0.708	-0.543	PIS51172.1	SIK1	Putative U3 snoRNP protein; Hap43p-induced gene; physically interacts with TAP-tagged Nop1p

PIS52268.1	30.64923199	30.09405061	30.7754203	30.05053353	29.86283603	29.97438982	0.086466143	0.618	-0.544	PIS52268.1	ARO4	3-deoxy-D-arabinoheptulosonate-7-phosphate synthase; aromatic amino acid biosynthesis; GCN-regulated; feedback-inhibited by tyrosine if produced in S. cerevisiae Aro3p and Aro4p catalyze same reaction; protein decreases in stationary phase
PIS51270.1	23.97182534	24.27247049	23.94450271	23.22812605	23.19822957	24.12970855	0.260346136	0.858	-0.544	PIS51270.1	orf19.6155	Ortholog(s) have DNA ligase (ATP) activity, DNA ligase activity
PIS48640.1	31.73261553	31.12604196	31.82553243	31.06252702	31.02829747	30.95862596	0.413767265	0.909	-0.545	PIS48640.1	ERG6	Delta(24)-sterol C-methyltransferase, converts zymosterol to fecosterol, ergosterol biosynthesis; mutation confers nystatin resistance; Hap43, GlcNAc-, fluconazole-induced; upregulated in azole-resistant strain; Spider biofilm repressed
PIS55810.1	27.66762005	29.82626819	27.75206922	28.04591977	27.78657353	27.774291	0.688470544	0.945	-0.546	PIS55810.1	MNN9	Protein of N-linked outer-chain mannan biosynthesis; mutant has defective cell wall; required for wild-type hyphal growth; mutant is hygromycin B sensitive and vanadate resistant; has N-terminal membrane-spanning segment (positions 18-34)
PIS51351.1	23.14607476	22.8140537	24.95030443	22.8083522	23.52160775	22.94222962	0.445937594	0.915	-0.546	PIS51351.1	YBL053	Putative subunit of a replication fork-pausing checkpoint complex
PIS49552.1	24.89344207	24.83397674	24.90108837	24.1600261	23.93943948	24.88709734	0.506019574	0.925	-0.547	PIS49552.1	orf19.2476	Ortholog(s) have chromatin binding activity and role in cellular response to oxidative stress, heterochromatin boundary formation, regulatory ncRNA-mediated heterochromatin formation
PIS52339.1	32.4136831	31.89705422	32.97408544	32.33954626	31.87366682	31.43191459	0.558241654	0.932	-0.547	PIS52339.1	RPL19A	Ribosomal protein L19; repressed upon phagocytosis by murine macrophages; Hap43-induced gene; Spider biofilm repressed
PIS54650.1	30.64514714	30.54927105	30.78664685	30.14588731	30.11138071	30.08000775	0.094464051	0.644	-0.548	PIS54650.1	ASN1	Putative asparagine synthetase; soluble protein in hyphae; regulated by Rim101; decreased expression at pH 4 vs pH 8; protein detected during exponential and stationary phases of yeast-form growth
PIS52389.1	30.19407214	30.49262366	30.02743445	29.75663233	29.59437792	29.71979773	0.210661673	0.826	-0.548	PIS52389.1	ATP5	Putative F0-ATP synthase FO subunit B; caspofungin repressed; protein level decreased in stationary phase yeast cultures; Spider biofilm repressed
PIS58228.1	23.32407353	23.66182234	24.01421301	23.09058505	23.18844839	23.07643708	0.10860534	0.683	-0.548	PIS58228.1	orf19.7010	Ortholog(s) have ubiquitin ligase complex localization

PIS51259.1	29.22357962	27.59919651	29.1738584	28.33927562	27.78640239	28.2226591	0.299275097	0.875	-0.549	PIS51259.1	EFG1	bHLH transcription factor; required for white-phase cell type, RPMI and Spider biofilm formation, hyphal growth, cell-wall gene regulation; roles in adhesion, virulence; Cph1 and Efg1 have role in host cytokine response; binds E-box
PIS58169.1	29.64087585	29.18601444	29.86605161	29.46936781	28.85866493	28.71453593	0.519745953	0.927	-0.55	PIS58169.1	orf19.458	Ortholog(s) have ATPase-coupled transmembrane transporter activity, protein transmembrane transporter activity
PIS58784.1	31.52444506	31.18285557	31.96014703	31.21044661	30.95641323	30.84649995	0.38693691	0.903	-0.551	PIS58784.1	GUP1	Putative O-acyltransferase with a role in glycerol uptake; functionally complements growth of S. cerevisiae gup1 mutant under salt stress; required for normal ergosterol distribution, hyphal growth, biofilm formation
PIS59028.1	26.48074378	25.54370794	26.71647646	27.03216007	26.18566929	23.86886738	0.566373376	0.933	-0.551	PIS59028.1	orf19.3663.1	Predicted plasma membrane protein; gene has intron
PIS55458.1	29.1744163	29.3007423	29.1503779	28.73978399	28.80288428	28.43084254	0.220075027	0.833	-0.551	PIS55458.1	SPT6	Putative transcription elongation factor; transposon mutation affects filamentous growth; transcript induced in an RHE model of oral candidiasis and in clinical isolates from oral candidiasis
PIS58946.1	26.49515719	21.41279077	27.08628115	24.5719735	24.79095025	23.9739362	0.708510702	0.946	-0.552	PIS58946.1	orf19.1096	Has domain(s) with predicted voltage-gated chloride channel activity, role in chloride transport, transmembrane transport and membrane localization
PIS52198.1	26.34967101	26.52225933	26.92552867	25.64118337	26.05308434	26.4459249	0.464517602	0.919	-0.552	PIS52198.1	orf19.5411	Ortholog(s) have NEDD8 transferase activity and role in protein neddylation
PIS48643.1	25.10067769	26.07802391	25.97838409	24.51807535	25.60478397	25.37485538	0.391946597	0.904	-0.553	PIS48643.1	CAP1	AP-1 bZIP transcription factor; apoptotic, oxidative stress response/resistance, multidrug resistance; nuclear in oxidative stress; complements S. cerevisiae yap1 mutant; oralpharyngeal candidasis-, human neutrophil, Spider biofilm induced
PIS58226.1	23.68516857	25.35826878	23.66388028	24.07223253	24.00910468	22.96348993	0.474299366	0.92	-0.554	PIS58226.1	orf19.6898	Protein similar to S. pombe SPBC1709.16c a predicted aromatic ring-opening dioxygenase; induced by benomyl treatment or in azole-resistant strain that overexpresses MDR1; Spider biofilm induced
PIS51440.1	27.48997665	26.84293829	27.43392595	26.62074902	26.78845742	26.69596141	0.218338082	0.832	-0.554	PIS51440.1	UTP15	Small subunit (SSU) processome component; mutation confers resistance to 5-fluorocytosine (5-FC); physically interacts with TAP-tagged Nop1p

PIS51897.1	28.89286652	29.31876275	28.91862073	28.94513946	28.23416657	28.28324463	0.114814929	0.698	-0.556	PIS51897.1	PIN4	Protein with similarity to S. cerevisiae Pin4p; transposon mutation affects filamentous growth
PIS49646.1	27.64342259	27.91971686	28.17411772	27.57525746	27.31498326	27.17263649	0.194380595	0.812	-0.558	PIS49646.1	orf19.4294	Ortholog(s) have oxidoreductase activity, role in cytochrome c-heme linkage, mitochondrial membrane organization and mitochondrial inner membrane localization
PIS48737.1	23.13118469	23.71514576	23.05956035	21.68955992	23.39785103	23.14458906	0.42087987	0.911	-0.558	PIS48737.1	orf19.6205	Ortholog of C. dubliniensis CD36 : Cd36_06550, C. parapsilosis CDC317 : CPAR2_209030, C. auris B8441 : B9J08_005439 and Candida tenuis NRRL Y-1498 : CANTEDRAFT_111035
PIS49737.1	30.3821672	30.41250772	30.08039096	29.96532352	29.55519197	29.68050613	0.155455236	0.769	-0.558	PIS49737.1	PRO2	Putative gamma-glutamyl phosphate reductase with a predicted role in proline biosynthesis; regulated by Gcn2p and Gcn4p
PIS51414.1	24.76378521	25.98647084	25.63617848	25.87139167	23.56705298	25.27117212	0.334020793	0.888	-0.559	PIS51414.1	MET14	Putative adenylylsulfate kinase; predicted role in sulfur metabolism; possibly adherence-induced; protein present in exponential and stationary growth phase yeast; F-12/CO2 biofilm induced
PIS54811.1	26.22217499	26.72333021	26.94830847	26.22556927	26.15730536	25.82426631	0.400591811	0.906	-0.562	PIS54811.1	MCA1	Putative metacaspase, cysteine protease involved in apoptosis in response to stresses; has similarity to S. cerevisiae Mca1p; fungal-specific (no human or murine homolog); farnesol-induced
PIS54975.1	30.70639136	31.24182395	30.85758933	29.9338973	30.85342146	30.32923009	0.303657532	0.877	-0.563	PIS54975.1	DED1	Predicted ATP-dependent RNA helicase; RNA strand annealing activity; Spider biofilm induced
PIS50488.1	25.50453732	24.40241595	24.52185288	23.56684459	24.68394979	24.48155999	0.300693496	0.876	-0.565	PIS50488.1	orf19.3972	Ortholog(s) have role in endoplasmic reticulum to Golgi vesicle-mediated transport, retrograde transport, endosome to Golgi
PIS50553.1	29.58774649	29.60022785	29.46992493	28.85968928	28.9219216	29.1792121	0.257228868	0.856	-0.566	PIS50553.1	UAP1	UDP-N-acetylglucosamine pyrophosphorylase, catalyzes biosynthesis of UDP-N-acetylglucosamine from UTP and N-acetylglucosamine 1-phosphate; functional homolog of S. cerevisiae Qri1p; alkaline upregulated
PIS56838.1	31.36274078	30.94708181	31.33001669	30.56229733	30.57488942	30.79894507	0.061484658	0.51	-0.568	PIS56838.1	AMS1	Putative alpha-mannosidase; transcript regulated by Nrg1; induced during cell wall regeneration; flow model biofilm induced; Spider biofilm induced

PIS54634.1	28.95593219	29.23341457	29.0322093	28.39736926	28.47847374	28.64274931	0.184411618	0.803	-0.568	PIS54634.1	OST1	Alpha subunit of the oligosaccharyltransferase complex of the ER lumen; catalyzes asparagine-linked glycosylation of newly synthesized proteins; Spider biofilm repressed
PIS48539.1	24.00177709	24.125467	23.61539748	22.88669321	23.7665415	23.37870552	0.156590483	0.77	-0.57	PIS48539.1	orf19.4628	Putative cleavage and polyadenylation factor; heterozygous null mutant exhibits hypersensitivity to parnafungin and cordycepin in the C. albicans fitness test; possibly an essential gene, disruptants not obtained by UAU1 method
PIS58878.1	27.51680059	26.38086003	26.95245994	25.9496029	26.71161212	26.47511406	0.167355727	0.784	-0.571	PIS58878.1	ARL3	Putative Ras superfamily GTPase; induced by nitric oxide independent of Yhb1p
PIS51093.1	32.7041943	32.66811933	32.86601082	32.05285833	32.36642536	32.10680463	0.626254387	0.939	-0.571	PIS51093.1	orf19.4686	Ortholog of S. cerevisiae : ESL1, C. glabrata CBS138 : CAGL0H06611g, C. dubliniensis CD36 : Cd36_41030, C. parapsilosis CDC317 : CPAR2_401340 and C. auris B8441 : B9J08_002665
PIS51098.1	26.03787498	24.27926575	24.24016807	24.87920821	23.95000939	24.00929363	0.261075188	0.858	-0.573	PIS51098.1	NGS1	N-acetyltransferase related to Gcn5p, acts as N-acetylglucosamine (GlcNAc) sensor required for GlcNAc-induced histone acetylation at promoters of GlcNAc-inducible genes and activation of their transcription by Rep1p; Hap43-induced gene
PIS56549.1	30.37209767	29.65242999	30.70311687	29.92675091	29.81039637	29.27218842	0.199611348	0.817	-0.573	PIS56549.1	RPT2	Putative ATPase of the 19S regulatory particle of the 26S proteasome; oxidative stress-induced via Cap1; Spider biofilm repressed
PIS48706.1	22.56581362	22.08437378	23.44024364	23.35825988	21.51471836	21.49559361	0.453305206	0.917	-0.574	PIS48706.1	orf19.3449	Ortholog(s) have stalled ribosome sensor activity, ubiquitin protein ligase activity
PIS52415.1	24.59384141	24.01490099	25.84824056	23.19877479	24.4062836	25.12850085	0.50191708	0.925	-0.574	PIS52415.1	orf19.4893	Ortholog of C. dubliniensis CD36 : Cd36_09630, C. parapsilosis CDC317 : CPAR2_805080, C. auris B8441 : B9J08_004031 and Candida tenuis NRRL Y-1498 : CANTEDRAFT_136811
PIS55755.1	34.52945607	34.71149662	34.44768492	33.83493545	33.98771829	34.14295604	0.419802846	0.91	-0.574	PIS55755.1	PGK1	Phosphoglycerate kinase; localizes to cell wall and cytoplasm; antigenic in murine/human infection; flow model biofilm, Hog1-, Hap43-, GCN-induced; repressed upon phagocytosis; repressed in Spider biofilms by Bcr1, Ndt80, Rob1, Brg1
PIS58125.1	29.91712091	29.77990336	29.7729283	29.58067878	29.10590352	29.0585402	0.306094499	0.878	-0.575	PIS58125.1	GLT1	Putative glutamate synthase; regulated by Sef1, Sfu1, and Hap43; rat catheter biofilm repressed

PIS49480.1	28.83181243	28.0338297	29.70410007	28.56497678	28.1922264	28.08894258	0.584709361	0.935	-0.575	PIS49480.1	PRE8	Putative alpha-2_sc subunit of proteasome; macrophage-induced protein; regulated by Gcn2p and Gcn4p; transcription is positively regulated by Tbf1p; stationary phase enriched protein
PIS55786.1	27.67554103	27.8949073	27.98592769	27.60052106	27.04230949	27.18331763	0.286681099	0.87	-0.577	PIS55786.1	orf19.1681	Ortholog of C. dubliniensis CD36 : Cd36_81530, C. parapsilosis CDC317 : CPAR2_503590, C. auris B8441 : B9J08_001892 and Candida tenuis NRRL Y-1498 : CANTEDRAFT_115755
PIS58917.1	27.60141862	26.93061245	27.20213857	26.88450303	26.75119337	26.36861127	0.261867012	0.858	-0.577	PIS58917.1	SMI1B	Putative cell wall assembly regulatory protein; Rim101-repressed; possibly an essential gene, disruptants not obtained by UAU1 method
PIS51844.1	28.00232766	27.59259246	28.20569685	27.45301013	27.35580069	27.2575228	0.385952303	0.903	-0.578	PIS51844.1	orf19.1761	Predicted olichyl-diphosphooligosaccharide-protein glycotransferase; role in protein N-linked glycosylation; Spider biofilm repressed
PIS51634.1	28.62409585	30.4805189	29.17944898	28.976888	28.73411863	28.83598912	0.186650937	0.805	-0.579	PIS51634.1	CTN1	Carnitine acetyl transferase; required for growth on nonfermentable carbon sources, not for hyphal growth or virulence in mice; induced in macrophage; macrophage/pseudohyphal-repressed after 16 hr; rat catheter, Spider biofilm induced
PIS51471.1	24.33184197	24.65547196	24.86410545	24.47782993	24.30089784	23.33444275	0.253233756	0.854	-0.579	PIS51471.1	orf19.7237	Ortholog(s) have arylformamidase activity and role in NAD biosynthetic process
PIS58103.1	30.09378341	29.73740908	30.14263473	29.82640062	29.23436269	29.17304506	0.516259086	0.927	-0.58	PIS58103.1	orf19.1823	Predicted membrane protein; transcript repressed by ciclopirox olamine
PIS50474.1	26.16662519	26.68865083	26.65263869	25.93456924	25.80730818	26.02532853	0.331624092	0.887	-0.58	PIS50474.1	orf19.3957	Ortholog(s) have GTP cyclohydrolase I activity, role in folic acid-containing compound biosynthetic process and nucleus localization
PIS51533.1	25.63630847	25.29506709	24.50316305	25.0137113	25.14249798	23.53852142	0.448641002	0.916	-0.58	PIS51533.1	orf19.4492	Ortholog(s) have role in nuclear division, rRNA processing, ribosomal large subunit biogenesis and nuclear periphery, nucleolus, preribosome, large subunit precursor localization
PIS55745.1	31.42235855	31.53032007	31.53517708	30.82245344	30.76787241	31.15112737	0.14073438	0.747	-0.582	PIS55745.1	ADE17	5-Aminoimidazole-4-carboxamide ribotide transformylase, enzyme of adenine biosynthesis; antigenic in human; soluble protein in hyphae; not induced during GCN response, in contrast to the S. cerevisiae ortholog

PIS52137.1	24.85780578	25.32782531	24.49694524	23.66575471	24.90203327	24.36901647	0.306462581	0.878	-0.582	PIS52137.1	CHR1	Predicted DEAD-box ATP-dependent RNA helicase; functional homolog of S. cerevisiae Rok1; Hap43-induced; Spider biofilm induced
PIS58133.1	25.02460277	24.44328388	24.11174673	23.88685202	24.08787085	23.85472306	0.099367014	0.659	-0.583	PIS58133.1	SPC19	Essential subunit of the Dam1 (DASH) complex, which acts in chromosome segregation by coupling kinetochores to spindle microtubules
PIS48341.1	27.92320248	28.38912815	26.96956253	27.0862836	28.10114462	26.3434815	0.676096627	0.944	-0.584	PIS48341.1	UTP5	Putative U3 snoRNA-associated protein; Hap43p-induced gene; mutation confers resistance to 5-fluorocytosine (5-FC), 5-fluorouracil (5-FU), and tubercidin (7-deazaadenosine); physically interacts with TAP-tagged Nop1p
PIS51206.1	27.36200294	27.62759712	27.16814475	27.39195976	26.13053454	26.88140856	0.414894489	0.909	-0.585	PIS51206.1	AIP2	Putative actin interacting protein; regulated by Gcn4; induced in response to amino acid starvation (3-AT); repressed by elevated CO2; flow model biofilm repressed
PIS58992.1	28.2932095	28.35668353	28.09290714	27.71314202	27.44484922	27.82396704	0.178457668	0.796	-0.587	PIS58992.1	FGR32	Protein similar to S. cerevisiae Swa2p; induced upon adherence to polystyrene; transposon mutation affects filamentous growth; Hap43p-repressed gene
PIS51679.1	33.03597392	32.92676415	32.67914941	32.21039559	32.39518557	32.27463829	0.032068198	0.328	-0.587	PIS51679.1	GPH1	Putative glycogen phosphorylase; role in glycogen metabolism; regulated by Ssk1, Mig1, Tup1, Hap43; fluconazole-induced; localizes to cell surface of hyphae, not yeast; stationary phase enriched protein; Spider biofilm induced
PIS58113.1	25.45522664	23.72484794	25.96116134	24.18484833	23.92405794	25.27161776	0.315513547	0.882	-0.587	PIS58113.1	orf19.6268	Putative cohesin complex subunit; expression downregulated in an ssr1 null mutant
PIS49631.1	25.34771273	26.0756722	25.03810218	24.14196387	25.04721626	25.51104456	0.44757507	0.916	-0.587	PIS49631.1	orf19.7290	Ortholog(s) have chromatin binding, mRNA binding activity and role in deadenylation-dependent decapping of nuclear-transcribed mRNA, nuclear-transcribed mRNA catabolic process, deadenylation-dependent decay
PIS54595.1	29.87327495	30.45513689	29.49585536	29.35753038	29.27157701	29.42740002	0.131534911	0.732	-0.589	PIS54595.1	APL2	Phosphorylated protein of unknown function; mutation confers hypersensitivity to toxic ergosterol analog
PIS52226.1	24.84545061	25.13140011	25.29056186	23.52415064	25.31072605	24.66314898	0.502910768	0.925	-0.59	PIS52226.1	ARO7	Putative chorismate mutase; fungal-specific (no human or murine homolog); alkaline upregulated

PIS56741.1	28.20766667	28.67970157	28.00578458	28.0942003	27.20078812	27.82208689	0.49173309	0.923	-0.592	PIS56741.1	COQ6	Ortholog(s) have oxidoreductase activity, acting on paired donors, with incorporation or reduction of molecular oxygen, reduced flavin or flavoprotein as one donor, and incorporation of one atom of oxygen activity
PIS54717.1	27.23674882	28.88312257	26.99551657	26.88825333	27.81765789	26.63278194	0.593378221	0.936	-0.592	PIS54717.1	orf19.3854	Ortholog of S. cerevisiae Sat4; amphotericin B induced; clade-associated gene expression; Spider biofilm induced
PIS52329.1	25.13448178	25.21175284	24.92290359	24.88258306	24.83228663	23.77558781	0.278882474	0.867	-0.593	PIS52329.1	RBK1	Has domain(s) with predicted kinase activity, ribokinase activity and role in D-ribose metabolic process
PIS56616.1	28.12200029	29.55349805	28.00737084	28.21036173	27.30552665	28.3847573	0.341730543	0.89	-0.594	PIS56616.1	orf19.1862	Possible stress protein; increased transcription associated with CDR1 and CDR2 overexpression or fluphenazine treatment; regulated by Sfu1, Nrg1, Tup1; stationary phase enriched protein; Spider biofilm induced
PIS51506.1	24.38617104	25.17212659	24.23171963	23.60799859	24.05589595	24.33410119	0.164480522	0.781	-0.597	PIS51506.1	orf19.5991	Ortholog(s) have role in assembly of large subunit precursor of preribosome, maturation of 5.8S rRNA from tricistronic rRNA transcript (SSU-rRNA, 5.8S rRNA and LSU-rRNA), more
PIS51951.1	29.52786937	29.48889357	30.0709859	29.15054675	29.23898557	28.90579646	0.079034899	0.591	-0.597	PIS51951.1	orf19.989	Mitochondrial ribosomal protein of the small subunit; Spider biofilm repressed
PIS48460.1	29.04776634	28.86973169	29.03452231	28.58596747	28.3601524	28.21373619	0.079021494	0.591	-0.597	PIS48460.1	RPL82	Predicted ribosomal protein; genes encoding cytoplasmic ribosomal subunits, translation factors, and tRNA synthetases are downregulated upon phagocytosis by murine macrophage
PIS51241.1	28.76353634	28.82358689	28.72449555	28.7327848	27.54434382	28.24304958	0.09655123	0.651	-0.597	PIS51241.1	YMC1	Putative inner mitochondrial membrane transporter; flucytosine induced; Spider biofilm repressed
PIS52070.1	29.87059466	30.21969592	30.47949185	29.61731598	29.34671303	29.81057861	0.222898775	0.835	-0.598	PIS52070.1	MCR1	NADH-cytochrome-b5 reductase; soluble in hyphae; alkaline downregulated; farnesol, ketoconazole or flucytosine induced; protein present in exponential and stationary growth phase yeast; YNB biofilm induced; rat catheter biofilm repressed
PIS51233.1	27.39057349	26.79906614	27.93414691	25.7083178	26.75781932	27.8599197	0.482470884	0.922	-0.599	PIS51233.1	ARE2	Acyl CoA:sterol acyltransferase; uses cholesterol and oleoyl-CoA substrates; protoberberine derivative drug inhibits enzyme activity; ketoconazole-induced; Hap43-repressed; flow model biofilm induced; Spider biofilm induced

PIS55803.1	29.030563	28.9540006	29.020839	28.45962574	28.33281474	28.41457465	0.321035401	0.884	-0.599	PIS55803.1	NPL4	Putative ubiquitin-binding protein; regulated by Gcn2p and Gcn4p
PIS50424.1	30.11420066	29.65805855	30.01456777	29.32482284	29.59129599	29.06935995	0.509662457	0.926	-0.6	PIS50424.1	orf19.3232	Putative transporter; mutation confers hypersensitivity to toxic ergosterol analog; fungal-specific (no human or murine homolog)
PIS56872.1	25.80333969	26.8827348	24.45600813	25.45405094	24.78741101	25.09356811	0.459098347	0.918	-0.602	PIS56872.1	BGL2	Cell wall 1,3-beta-glucosyltransferase; mutant has cell-wall and growth defects, but wild-type 1,3- or 1,6-beta-glucan content; antigenic; virulence role in mouse systemic infection; rat catheter biofilm induced
PIS56790.1	27.29011867	26.48849532	27.15736239	26.85929295	26.37466062	25.89645307	0.19384363	0.812	-0.602	PIS56790.1	orf19.445	Protein of unknown function; repressed by prostaglandins
PIS51630.1	27.16951079	26.94896099	26.68967589	26.27041918	26.22444796	26.50360265	0.038687801	0.371	-0.603	PIS51630.1	WAR1	Zn(II)2Cys6 transcription factor; plays a role in resistance to weak organic acids; required for yeast cell adherence to silicone substrate; Spider biofilm induced
PIS50563.1	31.21118062	30.49231062	30.50945792	29.66378139	30.43919659	30.29492916	0.116019062	0.701	-0.605	PIS50563.1	RPL43A	Putative ribosomal protein, large subunit; repressed by human whole blood or PMNs; colony morphology-related gene regulation by Ssn6; Spider biofilm repressed
PIS49720.1	27.92122984	28.31497838	28.14653385	26.379563	28.40741164	27.77267498	0.373616046	0.9	-0.608	PIS49720.1	RPD31	Putative histone deacetylase; involved in regulation of white-opaque switch; Spider biofilm repressed
PIS51759.1	25.54777531	24.94256927	26.05431521	27.28763726	22.80272986	24.61538739	0.553347322	0.931	-0.613	PIS51759.1	orf19.3840	Serine/threonine protein kinase, acts as an upstream activating factor for the SNF1 complex that regulates responses to nutrient stress; Spider biofilm induced
PIS49668.1	24.09120033	24.35276294	25.53923564	24.98118706	24.03755262	23.12302951	0.445702505	0.915	-0.614	PIS49668.1	CAC2	Component of the chromatin assembly factor I (CAF-1); involved in regulation of white-opaque switching frequency and biofilm initiation; null mutant shows hyperfilamentous wrinkled colonies; macrophage-induced
PIS54922.1	28.0029735	27.87834493	27.52709411	27.11945078	26.56959225	27.8769116	0.322069698	0.884	-0.614	PIS54922.1	orf19.1852	Ortholog(s) have mitochondrion localization

PIS58839.1	25.68483799	25.90235498	25.9775542	25.82346084	24.61281566	25.27327931	0.189579335	0.808	-0.618	PIS58839.1	orf19.397	Ortholog(s) have structural constituent of ribosome activity and mitochondrial large ribosomal subunit localization
PIS52119.1	31.80306421	31.79428767	32.05011106	31.55979246	31.11144065	31.11852768	0.304923276	0.878	-0.619	PIS52119.1	ERG10	Acetyl-CoA acetyltransferase; role in ergosterol biosynthesis; soluble in hyphae; changes in protein abundance associated with azole resistance; fluconazole or ketoconazole induced; macrophage-downregulated protein; GlcNAc-induced protein
PIS52134.1	27.99134016	26.99200866	28.20699759	27.94867977	27.30444383	26.07710343	0.560549762	0.932	-0.62	PIS52134.1	orf19.3759	Putative elongator complex subunit; for modification of wobble nucleosides in tRNA; Spider biofilm induced
PIS58748.1	27.52205377	26.6774795	27.82840691	26.98849131	26.49568568	26.68479658	0.142722654	0.75	-0.62	PIS58748.1	SKI8	Ortholog(s) have role in nuclear-transcribed mRNA catabolic process, 3'-5' exonucleolytic nonsense-mediated decay, nuclear-transcribed mRNA catabolic process, exonucleolytic and 3'-5', more
PIS51134.1	23.93017139	26.35073956	24.03600275	23.78338048	24.00574641	24.6678927	0.331618914	0.887	-0.62	PIS51134.1	UGT51C1	UDP-glucose:sterol glucosyltransferase; enzyme of sterol glucoside (membrane-bound lipid) biosynthesis; has UDP-sugar binding domain; activity is UDP-glucose-specific in vitro; enzyme does not use UDP-mannose; Mig1-regulated
PIS48736.1	25.24057208	27.41503275	24.96059381	24.18152109	25.4837535	26.08902628	0.55292676	0.931	-0.621	PIS48736.1	PUT3	Zn(II)2Cys6 transcription factor; has similarity to S. cerevisiae Put3, a transcription factor involved in the regulation of proline utilization genes
PIS51843.1	25.89560189	25.43072849	26.83455637	25.82707112	25.78157021	24.68696083	0.165751907	0.782	-0.622	PIS51843.1	OCA1	Putative protein phosphatase of the PTP family (tyrosine-specific); ortholog of S. cerevisiae Oca1; mutant is viable; mutant shows virulence defect
PIS54704.1	24.42198156	23.92145819	24.99056966	25.46719759	24.9658943	21.03213496	0.561025908	0.932	-0.623	PIS54704.1	MAK5	Putative nucleolar DEAD-box RNA helicase; oxidative stress-repressed via Cap1; repressed by prostaglandins
PIS48355.1	27.23567664	27.72086933	26.79277915	26.63740798	26.32875577	26.91540168	0.233461616	0.842	-0.623	PIS48355.1	orf19.581	Putative RNA-binding protein; transcript is upregulated in an RHE model of oral candidiasis
PIS54965.1	28.87967798	29.72369497	27.912744	28.399956	29.31355861	26.93001151	0.277575963	0.866	-0.624	PIS54965.1	orf19.6699	Ortholog(s) have histidinol-phosphatase activity and role in histidine biosynthetic process

PIS52059.1	23.87365737	25.73266664	25.69974022	24.40489256	24.84746248	24.17875868	0.34550768	0.892	-0.625	PIS52059.1	MED17	Putative RNA polymerase II mediator complex subunit; possibly an essential gene, disruptants not obtained by UAU1 method
PIS55698.1	29.68779121	29.96358662	28.86315682	28.70828306	29.06633612	28.86637286	0.110824046	0.689	-0.625	PIS55698.1	NOP1	Nucleolar protein; flucytosine induced; Hap43-induced; Spider biofilm repressed
PIS58036.1	28.00881993	28.16036657	28.04021212	27.29329595	27.58241099	27.45640886	0.578910882	0.934	-0.626	PIS58036.1	orf19.1201	Ortholog(s) have serine-tRNA ligase activity, role in mitochondrial seryl-tRNA aminoacylation and mitochondrion localization
PIS54570.1	30.57228192	30.70394857	30.38815812	30.15995015	29.87825554	29.74708088	0.338788947	0.89	-0.626	PIS54570.1	orf19.323	Aminophospholipid translocase (flippase), involved in phospholipid and sphingolipid translocation; type 4 P-type ATPase, localized to plasma membrane
PIS55816.1	34.08405711	33.79142834	34.13312106	33.25973155	33.48533913	33.38652306	0.082664922	0.605	-0.626	PIS55816.1	RPL10A	Predicted ribosomal protein; downregulated upon phagocytosis by murine macrophages; Hap43-induced; Spider biofilm repressed
PIS51716.1	33.73517815	34.13131494	33.12060589	32.29157866	33.36586186	33.4520795	0.30538828	0.878	-0.626	PIS51716.1	RPL4B	Ribosomal protein 4B; repressed upon phagocytosis by murine macrophage; Spider biofilm repressed
PIS51769.1	32.64085991	32.67461707	32.28757861	31.79003061	32.15449667	31.77282336	0.158154546	0.773	-0.629	PIS51769.1	HHT2	Putative histone H3; farnesol regulated; Hap43-induced; rat catheter and Spider biofilm repressed
PIS50501.1	27.26668496	25.53183745	26.94133092	25.58649335	26.19657584	26.06887555	0.344780807	0.891	-0.629	PIS50501.1	orf19.3994	Dolichyl-diphosphooligosaccharide-protein glycotransferase; predicted role in protein N-linked glycosylation, protein O-linked mannosylation; Spider biofilm repressed
PIS49494.1	27.68309106	27.48679631	27.74593122	27.27121785	26.87690342	26.88031911	0.072851221	0.565	-0.629	PIS49494.1	orf19.423	Ortholog(s) have enzyme activator activity, mRNA binding activity, role in deadenylation-dependent decapping of nuclear-transcribed mRNA and P-body, RNA decapping complex, cytoplasm, cytoplasmic side of membrane, nucleus localization
PIS51285.1	29.82840528	30.20647576	29.68865493	29.02751646	29.33316923	29.47264847	0.26674234	0.861	-0.63	PIS51285.1	FKH2	Forkhead transcription factor; morphogenesis regulator; required for wild-type hyphal transcription, cell separation, and for virulence in cell culture; mutant lacks true hyphae, is constitutively pseudohyphal; upregulated in RHE model

PIS58897.1	25.65391936	28.13380552	25.18526824	27.09401025	24.64039743	25.33147331	0.522997448	0.928	-0.636	PIS58897.1	MLC1	Microtubule-dependent localized protein; at Spitzenkorper and cytokinetic ring in hyphae; cell-cycle dependent localization to tip polarisome, bud neck in yeast and pseudohyphae; sumoylation target; rat catheter, Spider biofilm repressed
PIS58730.1	25.42125604	25.95825701	25.150514	24.35158988	24.15994882	26.11123464	0.25457989	0.855	-0.636	PIS58730.1	orf19.1708	Protein of unknown function; Spider biofilm induced
PIS48398.1	27.85085965	26.58584117	27.15741946	27.6811385	25.77021834	26.23619861	0.274585292	0.865	-0.636	PIS48398.1	RIM1	Putative single-stranded DNA-binding protein; protein level decreases in stationary phase cultures; rat catheter biofilm repressed
PIS55010.1	26.8235938	27.31140651	27.55816135	26.23817217	26.90899239	26.63288229	0.487596707	0.922	-0.638	PIS55010.1	orf19.6319	Ortholog(s) have cysteine-type deubiquitinase activity, mRNA binding activity
PIS51088.1	31.26497501	30.57876071	31.639044	30.9490907	30.46883432	30.14926617	0.428773731	0.912	-0.639	PIS51088.1	HGT17	Putative MFS family glucose transporter; 20 members in C. albicans; 12 probable membrane-spanning segments; induced at low (0.2%, compared to 2%) glucose in rich media; Spider biofilm induced
PIS51628.1	28.1974196	28.16004343	28.17949664	27.7026438	28.05704204	26.86063402	0.340543788	0.89	-0.639	PIS51628.1	STR2	Ortholog(s) have cystathionine gamma-synthase activity and role in sulfur compound metabolic process, transsulfuration
PIS48333.1	29.55832773	29.36031459	29.72426528	28.77368123	28.88821933	29.05768505	0.123247072	0.716	-0.641	PIS48333.1	TIM44	Protein involved in transport across membranes; Spider biofilm repressed
PIS55838.1	29.2486144	28.32645512	30.00268175	29.16072802	28.33425118	28.15353143	0.392648908	0.904	-0.643	PIS55838.1	orf19.4248	Ortholog of S. cerevisiae YLR118C (alias Apt1); acyl-protein thioesterase responsible for depalmitoylation of Gpa1 in S. cerevisiae; regulated by Gcn4; repressed in response to amino acid starvation (3AT); rat catheter biofilm repressed
PIS52320.1	24.58528055	25.71656884	24.53738645	24.20553586	24.33077137	24.37385292	0.206865457	0.823	-0.643	PIS52320.1	orf19.5239	Predicted alanine-tRNA ligase; oxidative stress-induced via Cap1
PIS52057.1	29.07029531	29.43020418	29.20437098	28.10446396	28.70499364	28.96146688	0.078442128	0.589	-0.645	PIS52057.1	orf19.3053	Protein of unknown function; present in exponential and stationary phase yeast; identified in extracts from biofilm and planktonic cells; flow model biofilm induced gene; GlcNAc-induced protein

PIS50511.1	32.00051288	32.42941919	32.00713766	31.52126028	31.44726574	31.52956709	0.193276889	0.811	-0.646	PIS50511.1	CCT3	Putative cytosolic chaperonin Cct ring complex subunit; mutation confers hypersensitivity to cytochalasin D
PIS48334.1	30.86731304	31.21048717	30.6391468	30.12577404	30.27878456	30.37395567	0.261828106	0.858	-0.646	PIS48334.1	GLO3	Putative ARF GTPase activator; role in COPI coating of Golgi vesicle, ER to Golgi vesicle-mediated transport, retrograde Golgi to ER vesicle-mediated transport; Spider biofilm repressed
PIS48224.1	25.49644443	26.91939985	25.77617202	25.69334684	25.33558728	25.22469733	0.144312283	0.753	-0.646	PIS48224.1	orf19.6440	Ortholog(s) have ubiquitin-protein transferase activity and role in cellular response to amino acid stimulus, proteasome-mediated ubiquitin-dependent protein catabolic process, ubiquitin-dependent protein catabolic process
PIS55039.1	26.94757013	24.7642237	27.01511645	26.89780704	25.42344325	24.46384692	0.424175542	0.911	-0.647	PIS55039.1	orf19.2076	Protein of unknown function; S. pombe ortholog SPAC7D4.05 encodes a predicted hydrolase; Hap43-repressed; Spider biofilm induced
PIS48569.1	23.64161438	25.97709574	25.56338082	25.27879867	23.26248531	24.69961062	0.49480638	0.924	-0.647	PIS48569.1	orf19.7029	Putative guanine deaminase; mutation confers hypersensitivity to toxic ergosterol analog; Spider biofilm induced
PIS59039.1	29.03342158	28.87764229	28.79566989	28.34597012	28.41854835	27.998996	0.194934771	0.813	-0.648	PIS59039.1	INP51	Putative phosphatidylinositol-4,5-bisphosphate phosphatase; involved in maintenance of phosphoinositide levels; affects hyphal growth, virulence, cell integrity; interacts with Irs4p
PIS54948.1	28.63480204	27.63296703	29.23195876	29.04072023	27.21594014	27.29871886	0.265760213	0.86	-0.648	PIS54948.1	SAM50	Predicted component of the SAM complex involved in mitochondrial protein import
PIS48799.1	28.64404285	29.73931788	28.67072746	28.88947493	28.10259964	28.09918447	0.192239457	0.81	-0.654	PIS48799.1	RIP1	Putative ubiquinol cytochrome c-reductase; induced by high iron; Hap43, nitric oxide, alkaline repressed; Spider biofilm repressed
PIS48636.1	33.48023838	33.52976786	33.47867258	32.81183264	32.93671781	32.77910585	0.076031192	0.579	-0.654	PIS48636.1	RPL12	Ribosomal protein L12, 60S ribosomal subunit; downregulated by human whole blood or polymorphonuclear cells; genes encoding cytoplasmic ribosomal subunits are downregulated upon phagocytosis by macrophage; Tbf1p-activated;
PIS52170.1	26.45444833	26.42401869	26.67388549	26.42387863	25.09963503	26.06689026	0.129491859	0.728	-0.654	PIS52170.1	TRP4	Predicted enzyme of amino acid biosynthesis; upregulated in biofilm; regulated by Gcn2p and Gcn4p; S. cerevisiae ortholog is Gcn4p regulated

PIS55478.1	25.3020242	24.51913769	26.05101522	24.6856482	24.30091842	24.92102586	0.196734911	0.814	-0.655	PIS55478.1	orf19.1160	Ortholog(s) have COPII receptor activity and role in COPII-coated vesicle cargo loading, endoplasmic reticulum to Golgi vesicle-mediated transport, fungal-type cell wall organization, protein retention in Golgi apparatus
PIS51357.1	27.36400221	27.88273354	27.38491486	25.97398106	27.09027701	27.60212323	0.192339228	0.81	-0.655	PIS51357.1	RMS1	Putative lysine methyltransferase; Hap43-induced; protein induced during mating; possibly essential, disruptants not obtained by UAU1 method; rat catheter and Spider biofilm induced
PIS51128.1	29.76599354	29.94458293	29.77733025	29.03955287	29.20190629	29.27446836	0.294073886	0.873	-0.657	PIS51128.1	orf19.7144	Ortholog(s) have GTP binding, GTPase activity, ribosome binding activity
PIS51685.1	24.74901574	24.84007885	25.73657572	22.25989309	25.21582277	25.87836178	0.551706918	0.931	-0.657	PIS51685.1	REX2	Putative 3'-5' RNA exonuclease with a predicted role in 3'-end processing of U4 and U5 snRNAs, 5S and 5.8S rRNAs; rat catheter biofilm induced
PIS58034.1	28.61778902	28.68608406	29.13914113	28.25659128	28.24930032	27.96333832	0.047108127	0.426	-0.658	PIS58034.1	SRO77	Protein with a predicted role in docking and fusion of post-Golgi vesicles with the plasma membrane; filament induced; fungal-specific (no human or murine homolog)
PIS55074.1	30.97648172	30.1376238	30.98452726	30.08161064	30.03195672	30.006624	0.045766787	0.418	-0.659	PIS55074.1	CDC68	Functional homolog of S. cerevisiae Cdc68, a transcription elongation factor; essential; possible drug target
PIS54623.1	26.68449051	26.10931987	26.51244616	25.4064463	25.7542894	26.16631332	0.679918809	0.944	-0.66	PIS54623.1	IPK2	Putative inositol polyphosphate multikinase; involved in regulation of calcium homeostasis, secretion and hyphal growth
PIS52287.1	25.61478715	23.60690527	24.6673754	24.88901693	23.30702901	23.71422171	0.313206563	0.881	-0.66	PIS52287.1	orf19.1910	Predicted NADH-dependent flavin oxidoreductase; Hap43-repressed gene
PIS49839.1	27.03061113	26.6722701	26.52881385	26.31136948	26.41273666	25.52645869	0.380490418	0.901	-0.66	PIS49839.1	orf19.5576	Putative pantothenate kinase; ortholog of S. cerevisiae Cab1; transposon mutation affects filamentous growth; repressed in core stress response
PIS58601.1	25.73247462	26.61822416	26.90820041	24.19659157	26.79050735	26.28889235	0.257374599	0.856	-0.661	PIS58601.1	orf19.7426	Ortholog(s) have protein-disulfide reductase activity, role in protein N-linked glycosylation, protein-containing complex assembly and oligosaccharyltransferase complex localization

PIS51145.1	31.02471631	30.12148827	31.26751736	30.03455402	30.14748904	30.2455729	0.353481533	0.894	-0.662	PIS51145.1	RPL30	Ribosomal 60S subunit protein; pre-rRNA processing; pre-mRNA alternatively spliced to productive/unproductive transcripts; temp-regulated splicing; colony morphology-related regulation by Ssn6, Tup1, Nrg1 regulated; Spider biofilm repressed
PIS58815.1	30.6190286	29.91819989	30.99745028	30.22741466	29.83595196	29.48274112	0.278138417	0.866	-0.663	PIS58815.1	CDC42	Rho-type GTPase; required for budding and maintenance of hyphal growth; GGTase I geranylgeranylated; misexpression blocks hyphal growth, causes avirulence in mouse IV infection; shows actin-dependent localization to hyphal tip
PIS56742.1	26.44890939	24.78189021	26.34462851	25.52450651	25.16135508	24.9018207	0.350330105	0.893	-0.663	PIS56742.1	orf19.6625	NAP1 family histone chaperone
PIS54543.1	28.89704125	28.40614601	28.84215566	28.17734637	28.22696335	27.74603495	0.099903624	0.66	-0.665	PIS54543.1	orf19.3797	Ortholog(s) have structural constituent of ribosome activity and mitochondrial large ribosomal subunit localization
PIS56784.1	24.92336103	24.47784524	25.57659868	24.52748891	24.12768588	24.32817208	0.301587672	0.876	-0.665	PIS56784.1	orf19.732	Possible dehydrogenase; flow model biofilm induced; rat catheter biofilm induced; Spider biofilm induced
PIS58196.1	29.78268833	29.60176976	30.26265238	29.78533829	28.73535619	29.12441307	0.252023	0.853	-0.667	PIS58196.1	ARG1	Argininosuccinate synthase; arginine synthesis; Gcn4, Rim101 regulated; induced by amino acid starvation (3-AT), benomyl treatment; stationary phase enriched protein; repressed in alkalinizing medium; rat catheter, Spider biofilm induced
PIS51874.1	30.02349869	29.81431054	30.51001095	29.80191145	29.12880097	29.41030156	0.042979935	0.399	-0.669	PIS51874.1	ARC19	Putative ARP2/3 complex subunit; Hap43-induced gene; mutation confers hypersensitivity to cytochalasin D; rat catheter biofilm repressed
PIS55502.1	29.39858194	29.39081809	29.5379436	28.92950509	28.56166928	28.82999269	0.060733586	0.506	-0.669	PIS55502.1	orf19.5054	Putative quinolinate phosphoribosyl transferase, involved in NAD biosynthesis; Hap43p-repressed gene
PIS55049.1	26.54403412	26.02617243	27.59270183	26.16693347	26.30287457	25.68564024	0.255418133	0.855	-0.669	PIS55049.1	SMF3	Putative vacuolar iron transporter; alkaline upregulated; caspofungin repressed; induced by Mnl1 under weak acid stress; Hap43-repressed
PIS51641.1	29.43761005	30.98469142	29.92934222	29.57732545	29.33041632	29.43720759	0.397162532	0.905	-0.669	PIS51641.1	TUF1	Translation elongation factor TU; macrophage/pseudohyphal-induced; repressed upon phagocytosis by murine macrophage; levels decrease in stationary phase cells; Spider biofilm repressed

PIS56551.1	29.25037267	29.46217615	29.10091344	28.51515304	28.58786125	28.70166209	0.069032203	0.548	-0.67	PIS56551.1	orf19.3547	Ortholog(s) have large ribosomal subunit rRNA binding, mRNA 3'-UTR binding, mRNA 5'-UTR binding, mRNA regulatory element binding translation repressor activity
PIS55496.1	27.38350541	27.134415	28.50113761	27.47906576	26.99747937	26.53191651	0.12684774	0.723	-0.67	PIS55496.1	orf19.4713	Ortholog of C. dubliniensis CD36 : Cd36_07300, C. parapsilosis CDC317 : CPAR2_208300, C. auris B8441 : B9J08_001596 and Candida tenuis NRRL Y-1498 : CANTEDRAFT_95879
PIS51460.1	28.78599189	29.90123329	28.62421614	29.01411506	28.00453785	28.28349211	0.2762976	0.866	-0.67	PIS51460.1	PDX1	Pyruvate dehydrogenase complex protein X; essential component of the mitochondrial pyruvate dehydrogenase complex; role in the respiratory pathway; protein present in exponential and stationary growth phase yeast; Spider biofilm
PIS48451.1	32.78530775	33.35301752	32.7785058	31.7437574	32.36868244	32.79254477	0.150004968	0.761	-0.671	PIS48451.1	HTA2	Putative histone H2A; farnesol regulated; rat catheter biofilm repressed; Spider biofilm repressed; Hap43-induced
PIS51958.1	26.94066834	28.38311705	27.29115455	26.71921376	26.60817631	27.27553907	0.156758513	0.771	-0.671	PIS51958.1	orf19.7437	Putative protein of unknown function; Hap43p-repressed gene; ortholog of S. cerevisiae YJL218W
PIS56526.1	26.55739756	24.59406877	24.98047188	25.82042873	24.34599521	23.94999711	0.445149985	0.915	-0.672	PIS56526.1	orf19.3481	Putative mitochondrial ATP-dependent RNA helicase of the DEAD-box family, transcription is activated in the presence of elevated CO2
PIS52361.1	30.15797758	30.59573053	29.70819226	28.22888746	29.94178011	30.26187782	0.328621289	0.886	-0.676	PIS52361.1	orf19.4018	Ortholog(s) have structural constituent of ribosome activity and mitochondrial small ribosomal subunit localization
PIS51193.1	28.7146195	26.55579739	28.70894707	27.62418334	28.09470636	26.23299321	0.438337088	0.914	-0.676	PIS51193.1	PUP1	Putative beta 2 subunit of the 20S proteasome; macrophage/pseudohyphal-repressed; Spider biofilm repressed
PIS51081.1	33.72810215	33.71573878	34.44412493	33.61125788	33.08754686	33.15818147	0.208731124	0.824	-0.677	PIS51081.1	CIT1	Citrate synthase; induced by phagocytosis; induced in high iron; Hog1-repressed; Efg1-regulated under yeast, not hyphal growth conditions; present in exponential and stationary phase; Spider biofilm repressed; rat catheter biofilm induced
PIS58576.1	24.45704324	24.7963483	25.420683	23.25201703	24.79085127	24.59597118	0.170662055	0.788	-0.678	PIS58576.1	orf19.6852	Ortholog of Rmd6 involved in S. cerevisiae sporulation; flow model biofilm induced

PIS54846.1	28.49426761	28.28907036	28.72585141	27.91366497	27.80510677	27.75473672	0.156814933	0.771	-0.679	PIS54846.1	COX11	Cytochrome oxidase assembly protein; transcript regulated by Nrg1; protein repressed during the mating process; Hap43-repressed gene; rat catheter biofilm induced
PIS51208.1	25.75087806	25.64535724	25.93843882	25.45948951	24.96812504	24.86888937	0.04996618	0.444	-0.679	PIS51208.1	orf19.2284	Protein with an FMN-binding domain; Hap43-repressed; flow model biofilm induced
PIS51326.1	29.29253362	29.54774723	29.67576487	28.75904419	28.80230107	28.91895043	0.073071338	0.566	-0.679	PIS51326.1	orf19.6830	Putative enoyl-CoA hydratase; Spider biofilm induced
PIS51792.1	26.42387641	26.36261976	26.61996584	26.48766857	26.97088397	23.90736558	0.519738394	0.927	-0.68	PIS51792.1	orf19.6224	RTA domain protein; predicted role in response to stress; Spider biofilm induced
PIS48652.1	27.58665603	27.65367763	28.81398625	27.4832473	27.15654272	27.36789469	0.25182035	0.853	-0.682	PIS48652.1	NMA111	Putative serine protease that promotes apoptosis and negatively regulates filamentation; null mutant shows increased virulence and hyperfilamentation; expression is induced by macrophages
PIS51369.1	28.70071334	29.07006731	28.99267318	28.27185497	28.25812106	28.18305518	0.166952144	0.784	-0.683	PIS51369.1	MDM10	Predicted component of the mitochondrial sorting and assembly machinery (SAM complex) and ER-mitochondria encounter structure (ERMES) complex with a role in protein import into mitochondria
PIS51848.1	30.06490318	30.19952071	30.12986636	29.4161515	29.34413251	29.5797025	0.026338554	0.285	-0.685	PIS51848.1	GPD1	Glycerol-3-phosphate dehydrogenase; glycerol biosynthesis; regulated by Efg1; regulated by Tsa1, Tsa1B under H2O2 stress conditions; Sflow model and Spider biofilm induced
PIS56631.1	25.6802853	25.03638267	26.22401826	25.59975068	24.30338197	24.97779174	0.276583934	0.866	-0.687	PIS56631.1	orf19.6872	Putative protein of unknown function; Hap43p-repressed gene
PIS55508.1	24.44742412	25.14161626	25.19835995	25.00691065	25.00238879	22.71262835	0.460659003	0.918	-0.688	PIS55508.1	ERD1	Putative membrane protein required for the retention of lumenal endoplasmic reticulum proteins; rat catheter biofilm induced
PIS54674.1	31.07838067	30.92071104	31.27512175	30.48341782	30.32147237	30.39707753	0.062596018	0.516	-0.691	PIS54674.1	BAT21	Putative branched chain amino acid aminotransferase; regulated by Gcn4, Gcn2; induced in response to amino acid starvation (3-aminotriazole treatment); early-stage flow model biofilm formation

PIS56703.1	29.96277047	29.84533708	29.94800144	29.79099826	28.71748303	29.17317547	0.229109796	0.839	-0.691	PIS56703.1	EIF4E	Translation initiation factor eIF4E; genes encoding ribosomal subunits, translation factors, tRNA synthetases downregulated by phagocytosis by macrophage; alternatively spliced intron in 5' UTR; protein levels decrease in stationary phase
PIS58820.1	33.68262125	34.55185663	32.83635409	33.14802264	32.75920517	33.08824615	0.396925271	0.905	-0.692	PIS58820.1	ENO1	Enolase, involved in glycolysis and gluconeogenesis; also has transglutaminase activity involved in assembly of cell wall polysaccharides; major cell-surface antigen; binds host plasmin/plasminogen; immunoprotective; may be essential
PIS49852.1	30.50238366	30.75917036	30.20055316	29.60749734	29.76973608	30.00703386	0.156800419	0.771	-0.693	PIS49852.1	orf19.5671	Ortholog(s) have enzyme inhibitor activity and role in actin cortical patch assembly, septin cytoskeleton organization
PIS48686.1	28.82101007	28.43912097	28.81068418	28.01930477	27.92142984	28.04801255	0.174840306	0.793	-0.694	PIS48686.1	orf19.3428	Protein of unknown function; flow model biofilm induced
PIS52245.1	26.57188508	26.87807383	27.60596406	26.73383441	26.54033533	25.69414812	0.322263879	0.884	-0.696	PIS52245.1	orf19.1448.1	Ortholog(s) have plus-end-directed microtubule motor activity, protein-containing complex binding activity and role in establishment of mitotic spindle localization, nuclear migration along microtubule, nuclear pore complex assembly
PIS58479.1	28.82406263	28.87413197	28.72670455	27.70923753	28.31434344	28.31153958	0.175679575	0.794	-0.697	PIS58479.1	ALG5	Putative glucosyltransferase involved in cell wall mannan biosynthesis; possibly an essential gene, disruptants not obtained by UAU1 method
PIS54501.1	26.63583468	27.79223418	27.65029646	26.6755422	26.76839412	26.54110676	0.247831855	0.851	-0.698	PIS54501.1	orf19.5963	Putative prenyltransferase; essential gene in S. cerevisiae; Spider biofilm induced
PIS55833.1	26.73866048	25.0360567	24.9936339	26.84045965	23.32025197	24.51451806	0.534243047	0.929	-0.698	PIS55833.1	RIX7	Putative ATPase of the AAA family; role in ribosomal subunit export from the nucleus; mutation impairs hyphal growth and biofilm formation
PIS49651.1	24.8620373	24.90857455	24.55289941	24.4633267	24.21771945	23.54926211	0.151723003	0.764	-0.698	PIS49651.1	WHI3	Putative RNA binding protein; induced during infection of murine kidney, compared to growth in vitro; has murine homolog
PIS52246.1	30.61826702	30.86413173	30.31688774	29.98286511	29.63197904	30.08768244	0.214751565	0.829	-0.699	PIS52246.1	APT1	Adenine phosphoribosyltransferase; flucytosine induced; repressed by nitric oxide; protein level decreased in stationary phase yeast cultures

PIS56925.1	27.65586226	26.29185731	27.36813545	26.77087922	26.20081122	26.24806943	0.468683711	0.919	-0.699	PIS56925.1	KRR1	Putative nucleolar protein; repressed benomyl treatment or in an azole-resistant strain that overexpresses MDR1; F-12/CO2 early biofilm induced
PIS48702.1	30.11428933	30.27556748	29.71418617	29.22242612	29.64551956	29.13972599	0.42328325	0.911	-0.699	PIS48702.1	orf19.137	Putative transferase involved in phospholipid biosynthesis; induced by alpha pheromone in SpiderM medium
PIS56723.1	25.65532306	25.38011816	23.73168558	22.71030321	25.31133919	24.64580474	0.337877319	0.889	-0.7	PIS56723.1	orf19.7664	Has domain(s) with predicted DNA binding activity
PIS49835.1	24.44487077	24.07622783	22.8911537	24.06229553	22.67970478	22.56731997	0.17849417	0.797	-0.701	PIS49835.1	NBN1	Subunit of the NuA4 histone acetyltransferase complex; Plc1p-regulated
PIS55683.1	24.65153304	24.48461049	25.42831642	24.2382702	24.73342904	23.48657966	0.203715693	0.82	-0.702	PIS55683.1	RGT1	Zn(II)2Cys6 transcription factor; transcriptional repressor involved in the regulation of glucose transporter genes; ortholog of S. cerevisiae Rgt1; mutants display decreased colonization of mouse kidneys
PIS49816.1	28.43033318	28.12344296	28.53515726	27.48718186	27.58813214	27.90321324	0.152466116	0.765	-0.703	PIS49816.1	orf19.4283	Ortholog(s) have role in cytoplasmic translational initiation
PIS59006.1	30.91263943	31.600383	30.86117498	30.34155309	30.3226656	30.59745491	0.146042348	0.755	-0.704	PIS59006.1	KGD2	Putative dihydrolipoamide S-succinyltransferase; induced in high iron; Hap43-repressed; Spider biofilm repressed
PIS55590.1	25.30195882	25.09003869	24.36900597	24.19740301	24.54440543	23.90276317	0.05473925	0.471	-0.705	PIS55590.1	orf19.6642	Ortholog(s) have role in endoplasmic reticulum to Golgi vesicle-mediated transport and COPII-coated ER to Golgi transport vesicle, Golgi membrane, endoplasmic reticulum membrane localization
PIS50497.1	27.37255478	26.66320002	27.20790494	26.42263051	26.51719168	26.18665957	0.526674928	0.928	-0.706	PIS50497.1	orf19.2445	Putative dicarboxylic amino acid permease; fungal-specific (no human or murine homolog); induced by alpha pheromone in SpiderM medium
PIS51516.1	28.1594267	27.59532284	28.55098973	27.41793721	27.58713556	27.17928429	0.080954621	0.598	-0.707	PIS51516.1	orf19.2180	Ortholog(s) have calcium ion binding, zinc ion binding activity and role in axial cellular bud site selection, bipolar cellular bud site selection, regulation of COPII vesicle coating

PIS58466.1	30.89612267	30.66649447	30.87024141	29.52887471	30.07966509	30.70402866	0.054276641	0.468	-0.707	PIS58466.1	orf19.2930	Predicted translation initiation factor; role in translational initiation; Spider biofilm repressed
PIS56556.1	23.35351947	23.69029003	23.55671673	22.08211474	23.34292477	23.05267228	0.517111397	0.927	-0.708	PIS56556.1	orf19.4293	Ortholog(s) have role in protein maturation by iron-sulfur cluster transfer, tRNA wobble uridine modification and CIA complex, cytosol, nucleus localization
PIS51222.1	27.06907983	26.03165223	26.76230813	26.094118	26.17399104	25.46711926	0.118390916	0.706	-0.709	PIS51222.1	orf19.2266	Ortholog(s) have ATPase, DNA binding, nucleosome binding activity, role in sister chromatid cohesion and Isw1a complex localization
PIS58412.1	28.46741291	28.54360979	28.73153587	26.74850049	28.14219028	28.72385107	0.287563524	0.871	-0.709	PIS58412.1	orf19.2917	Putative GTPase; heterozygous null mutant exhibits resistance to parnafungin in the C. albicans fitness test; Hap43p-induced gene
PIS48510.1	25.08824502	23.56958757	25.24899791	25.40710963	24.26476206	22.10704655	0.374978433	0.9	-0.709	PIS48510.1	orf19.5291	Ortholog(s) have role in phospholipid biosynthetic process, phospholipid homeostasis, phospholipid metabolic process and endoplasmic reticulum localization
PIS51624.1	26.68737502	25.99182061	27.01959486	25.64028086	25.68906114	26.23662291	0.151451535	0.763	-0.711	PIS51624.1	ARO9	Aromatic transaminase; Ehrlich fusel oil pathway of aromatic alcohol biosynthesis; Rim101-dependent pH-regulation (alkaline induced); Hap43-induced gene
PIS52161.1	29.62423389	29.9481962	29.28679932	28.547714	29.12578757	29.05182379	0.20724478	0.823	-0.711	PIS52161.1	orf19.3129	Putative chromatin remodelling complex protein; heterozygous null mutant displays sensitivity to virgineone; Spider biofilm repressed; sumoylation regulated by Hsp90p
PIS49639.1	25.25053897	25.62505959	24.68036853	24.15009824	24.48961769	24.78083753	0.065802133	0.532	-0.712	PIS49639.1	MAK32	Putative protein involved in the structural stability of L-A double-stranded RNA-containing particles; downregulated upon adherence to polystyrene
PIS51149.1	26.55791623	27.04553853	25.7390153	27.07341382	25.73776899	24.39413702	0.260312399	0.858	-0.712	PIS51149.1	orf19.4245	Protein with a predicted pleckstrin domain; Hap43-repressed gene; mutation causes decreased interaction with macrophages
PIS52353.1	24.16260026	26.12000805	24.73866138	23.71449076	24.55459483	24.61188763	0.213608141	0.828	-0.713	PIS52353.1	ECM25	Non-essential protein involved in cell morphogenesis

PIS51075.1	23.69378291	23.72278614	23.86698545	23.15971325	22.30095492	23.67960808	0.078478395	0.589	-0.714	PIS51075.1	HXT5	Putative sugar transporter; induced by ciclopirox olamine; Snf3-induced; alkaline repressed; colony morphology-related gene regulation by Ssn6; possibly essential gene
PIS51563.1	28.07764193	28.41979091	27.91838742	26.86174255	27.53953208	27.87301016	0.305184816	0.878	-0.714	PIS51563.1	LHP1	Ortholog(s) have RNA binding, RNA folding chaperone, tRNA binding activity, role in tRNA 3'-end processing, tRNA 5'-leader removal, tRNA folding, tRNA processing and cytoplasm, nucleolus, nucleoplasm, nucleus localization
PIS52260.1	25.44232588	23.92364528	24.94092682	24.19556176	24.20147967	23.76626539	0.611650613	0.938	-0.715	PIS52260.1	HIS7	Putative imidazole glycerol phosphate synthase; histidine biosynthesis; no human/murine homolog; transcription induced by histidine starvation; regulated by Gcn2p and Gcn4p; higher protein level in stationary phase
PIS51682.1	26.90030708	26.52472549	26.79945865	24.77232812	26.62776796	26.67842521	0.396082832	0.905	-0.715	PIS51682.1	orf19.7027	Protein of unknown function; Spider biofilm induced
PIS58983.1	30.87487867	30.37215974	31.12618632	30.2395993	29.82868577	30.16063443	0.057585991	0.488	-0.715	PIS58983.1	SEC14	Essential protein; functional homolog of S. cerevisiae Sec14p, a Golgi phosphatidylinositol/phosphatidylcholine transfer protein that regulates choline-phosphate cytidyltransferase and thereby affects secretion;
PIS51718.1	30.00873529	30.09758418	30.28359799	29.88170306	29.13103001	29.22922888	0.383238208	0.902	-0.716	PIS51718.1	IML2	Protein of unknown function; early-stage flow model biofilm induced; Hap43-repressed; Spider biofilm repressed
PIS50328.1	25.42072042	24.97532611	25.33355968	25.05555009	23.80222133	24.722937	0.427374706	0.912	-0.716	PIS50328.1	orf19.4929	Ortholog(s) have mRNA binding, translation regulator activity, role in mitochondrial cytochrome c oxidase assembly, positive regulation of mitochondrial translational initiation and mitochondrial inner membrane localization
PIS50541.1	27.50019038	27.05192126	27.66648073	26.28886369	26.53847847	27.2415023	0.26529222	0.86	-0.717	PIS50541.1	HOG1	MAP kinase of osmotic-, heavy metal-, and core stress response; role in regulation of response to stress; phosphorylated in response to H2O2 or NaCl; acts as repressor of START; mutant induces protective mouse immune response
PIS58521.1	24.41458656	25.61894224	25.17587657	24.69461809	24.91896683	23.44239711	0.263865909	0.859	-0.718	PIS58521.1	orf19.3272	Ortholog(s) have protein-folding chaperone binding activity, role in box C/D snoRNP assembly, protein folding and R2TP complex localization
PIS48227.1	25.80600743	23.68416803	24.41921693	25.25810471	23.35754356	23.14013783	0.380922093	0.901	-0.718	PIS48227.1	orf19.6436	Ortholog of C. dubliniensis CD36 : Cd36_34210, C. parapsilosis CDC317 : CPAR2_205590, C. auris B8441 : B9J08_004911 and Candida tenuis NRRL Y-1498 : cten_CGOB_00133

PIS51998.1	30.18003242	31.30015266	30.29004032	29.65939553	29.80253838	30.14983177	0.137272102	0.742	-0.719	PIS51998.1	GCV2	Glycine decarboxylase P subunit; protein of glycine catabolism; repressed by Efg1; Hog1-induced; induced by Rim101 at acid pH; transcript induced in elevated CO2; stationary phase enriched protein
PIS49540.1	28.55934148	27.39209848	29.42752434	28.26589295	27.35749918	27.59241759	0.293127059	0.873	-0.721	PIS49540.1	orf19.697	Ortholog of S. cerevisiae : YSC83, C. glabrata CBS138 : CAGL0A02134g, C. dubliniensis CD36 : Cd36_32160, C. parapsilosis CDC317 : CPAR2_203070 and C. auris B8441 : B9J08_004564
PIS48407.1	32.52432473	32.59910905	32.16137097	31.59887127	31.64877354	31.87045067	0.164097622	0.78	-0.722	PIS48407.1	RPL5	Ribosomal protein; repressed upon phagocytosis by murine macrophages; Hap43-induced; Spider biofilm repressed
PIS54867.1	24.95644818	24.76472408	25.49901449	24.91764685	23.34292477	24.79043376	0.432507042	0.913	-0.723	PIS54867.1	TRS20	Ortholog(s) have role in endoplasmic reticulum to Golgi vesicle-mediated transport, protein-containing complex assembly and TRAPPI protein complex, TRAPPII protein complex, TRAPPIII protein complex localization
PIS48668.1	29.16371942	28.24882793	29.06294211	28.14305895	28.10677882	28.05378281	0.118175445	0.705	-0.724	PIS48668.1	COQ3	Protein with a predicted role in coenzyme Q biosynthesis; transcriptionally induced by interaction with macrophages; possibly an essential gene, disruptants not obtained by UAU1 method
PIS48606.1	30.69641726	30.99063401	30.5638442	29.6737323	30.23488833	30.16964974	0.101260752	0.664	-0.724	PIS48606.1	WBP1	Putative oligosaccharyltransferase subunit; Spider biofilm repressed
PIS58048.1	24.478035	25.44814202	23.85209125	24.05829502	23.7452726	23.79683607	0.227313025	0.838	-0.726	PIS58048.1	orf19.7621	Putative subunit of an alternative replication factor C complex; role in DNA replication, genome integrity, homologous recombination-mediated repair and telomere homeostasis;
PIS54788.1	27.8723848	27.4409867	27.95465381	27.76650005	27.1753147	26.14730485	0.181380573	0.8	-0.726	PIS54788.1	PRE6	Putative alpha-4 subunit of the proteasome; reported as macrophage-induced protein and macrophage/pseudohyphal-repressed gene; regulated by Gcn2p and Gcn4p; removed from/reinstated in Assembly 20 (see Locus History)
PIS51494.1	26.56642034	26.16958436	26.59920826	25.60223029	25.68552342	25.86524699	0.039677172	0.377	-0.727	PIS51494.1	orf19.5835	Ortholog(s) have rRNA binding activity, role in rRNA processing, ribosomal small subunit assembly and CURI complex, UTP-C complex localization
PIS52187.1	29.39725304	28.9937675	29.39785711	29.08512892	28.19470959	28.3216853	0.137380224	0.742	-0.729	PIS52187.1	orf19.1777	Ortholog(s) have cysteine-type deubiquitinase activity, deubiquitinase activity, endopeptidase activity, metal-dependent deubiquitinase activity

PIS51784.1	26.72007507	26.29669731	26.96956687	25.57585287	26.60909979	25.61458905	0.336268946	0.889	-0.729	PIS51784.1	SIT4	Serine/threonine protein phosphatase catalytic subunit; has a role in cell wall maintenance, hyphal growth, and virulence in a mouse systemic infection model
PIS51912.1	24.59872302	23.67053432	24.25784208	24.23994745	23.23867031	22.85928518	0.118359559	0.706	-0.73	PIS51912.1	CNH1	Na+/H+ antiporter; required for wild-type growth, cell morphology, and virulence in a mouse model of systemic infection; not transcriptionally regulated by NaCl; fungal-specific (no human or murine homolog)
PIS52176.1	30.74194418	30.32305772	30.91484727	29.88829685	30.00333936	29.89437595	0.473565976	0.92	-0.731	PIS52176.1	HSM3	Ortholog(s) have protein folding chaperone activity, role in mismatch repair, proteasome regulatory particle assembly and cytosol, nucleus localization
PIS51065.1	26.66338275	25.88979808	26.85350996	25.95562952	26.59986229	24.65848916	0.261627784	0.858	-0.731	PIS51065.1	orf19.1630	Putative RNA-binding protein involved in translational regulation; null mutant shows reduced autophagy in response to DNA damage and nitrogen starvation, possibly due to effects on protein levels of Atg1p and Atg13p
PIS51119.1	32.12359791	32.51699019	32.68790318	31.78738011	31.67415113	31.67474429	0.120645149	0.71	-0.731	PIS51119.1	YHB1	Nitric oxide dioxygenase; acts in nitric oxide scavenging/detoxification; role in virulence in mouse; transcript activated by NO, macrophage interaction; Hap43, hypha repressed; mRNA binds She3
PIS54503.1	31.19560808	32.13739104	30.74774638	29.42539124	31.05752353	31.39141033	0.355150876	0.894	-0.735	PIS54503.1	RPL35	Ribosomal protein; downregulation correlates with clinical development of fluconazole resistance; colony morphology-related gene regulation by Ssn6; Hap43-induced; Spider biofilm repressed
PIS52399.1	25.22749361	25.93475671	25.06453878	22.95781468	25.63875881	25.42204753	0.267709638	0.861	-0.736	PIS52399.1	GCA1	Extracellular/plasma membrane-associated glucoamylase; expressed in rat oral infection; regulated by carbohydrates, pH, galactose; promotes biofilm matrix formation; flow model biofilm induced; Bcr1 repressed in RPMI a/a biofilms
PIS50399.1	28.51865309	29.83672222	28.12484486	27.80797582	27.86088027	28.59570954	0.232978021	0.842	-0.739	PIS50399.1	SSZ1	Putative HSP70 chaperone; protein level decreases in stationary phase cultures; Spider biofilm repressed
PIS51868.1	24.79264916	23.77400937	24.191105	22.53955821	23.97619453	24.01867814	0.143069884	0.751	-0.741	PIS51868.1	GSG1	Putative subunit of the TRAPP complex; involved in targeting of ER-to-Golgi transport vesicles; flow model biofilm induced
PIS48755.1	31.25216408	31.06305381	31.39061238	30.90535439	30.30160316	30.27086018	0.309827866	0.88	-0.743	PIS48755.1	ASC1	40S ribosomal subunit similar to G-beta subunits; glucose or N starvation induced filamentation; required for virulence in mice; snoRNA snR24 encoded in ASC1 intron; repressed in stationary phase; GlcNAc-induced; Spider biofilm repressed

PIS58349.1	26.35158507	26.40839271	25.67689055	26.0308573	24.35643236	25.82173163	0.22924804	0.839	-0.743	PIS58349.1	NIT2	Putative carbon-nitrogen hydrolase; rat catheter biofilm repressed
PIS58068.1	32.31283231	32.31135778	31.73900068	30.93734556	31.56712229	31.62707872	0.47229658	0.92	-0.744	PIS58068.1	RPL25	Putative rRNA-binding ribosomal protein component of the 60S ribosomal subunit; Hap43-induced; colony morphology-related gene regulation by Ssn6
PIS58444.1	26.12048402	26.6508026	26.63087501	26.64023449	24.42110658	26.09077866	0.511494605	0.926	-0.75	PIS58444.1	ABP140	Ortholog of S. cerevisiae actin-binding protein Abp140; Hap43-induced; F-12/CO2 early biofilm induced
PIS58463.1	30.36403541	30.49459544	30.05585509	29.577907	29.38993202	29.69425706	0.426863936	0.912	-0.751	PIS58463.1	orf19.2928	Has domain(s) with predicted role in retrograde vesicle-mediated transport, Golgi to endoplasmic reticulum and membrane localization
PIS50529.1	29.67503997	30.90907309	29.35489426	30.01591526	28.86991062	28.7999936	0.528441504	0.928	-0.751	PIS50529.1	orf19.3915	Putative metallodipeptidase; protein present in exponential and stationary growth phase yeast; Hog1-induced; Hap43-repressed; sumoylation target; Spider biofilm repressed
PIS56718.1	26.85015466	29.34496829	27.05364731	26.18048775	26.97125814	27.84460727	0.355584346	0.895	-0.751	PIS56718.1	RFC4	Putative heteropentameric replication factor C subunit; flucytosine induced; periodic mRNA expression, peak at cell-cycle G1/S phase
PIS56736.1	29.19905411	28.80324347	28.40960796	28.07760994	28.03006601	28.04152361	0.202040577	0.819	-0.754	PIS56736.1	orf19.6090	Putative nucleolar protein with a predicted role in pre-rRNA processing and ribosome biogenesis; repressed by nitric oxide; required for flow model biofilm formation; Spider biofilm repressed
PIS52385.1	24.41082376	26.08337116	24.00807797	24.64685879	24.84048316	22.75427689	0.483718937	0.922	-0.754	PIS52385.1	SLR1	Protein similar to mammalian SR-like RNA splicing factor; involved in filamentous growth and virulence; gene has intron; Spider biofilm repressed
PIS59021.1	34.56733069	34.49004849	34.61312763	33.71625954	33.78226194	33.90259651	0.040030508	0.379	-0.756	PIS59021.1	ACT1	Actin; gene has intron; transcript regulated by growth phase, starvation; at polarized growth site in budding and hyphal cells; required for wild-type Cdc42 localization; unprocessed N terminus; Hap43-induced; Spider biofilm repressed
PIS51966.1	25.3736961	26.7435812	26.50399724	26.68831924	24.20475495	25.45029978	0.269133952	0.862	-0.759	PIS51966.1	orf19.5346	Ortholog(s) have role in transcription by RNA polymerase II and transcription factor TFIID complex localization

PIS58211.1	27.05479407	26.32814773	26.75492981	26.26671931	26.07360869	25.51648307	0.131477549	0.731	-0.76	PIS58211.1	FRE10	Major cell-surface ferric reductase under low-iron conditions; 7 transmembrane regions and a secretion signal predicted; Tup1, Rim101, Ssn6, Hog1, caspofungin repressed; ciclopirox olamine induced; rat catheter biofilm induced
PIS58370.1	23.86043142	26.23684084	23.75727873	24.14169574	24.47240951	22.9588453	0.234426433	0.843	-0.761	PIS58370.1	VMA7	Putative subunit of the V-ATPase complex, which is involved in control of vacuolar pH; highly similar to S. cerevisiae Vma7p; interacts with phosphatidylinositol 3-kinase Vps34p
PIS52468.1	27.00613838	27.9194191	27.59859558	25.84564731	27.27250687	27.11197075	0.291048138	0.872	-0.765	PIS52468.1	orf19.2639	Ortholog(s) have structural constituent of ribosome activity and mitochondrial large ribosomal subunit localization
PIS55604.1	28.15259882	28.07454309	28.86287874	27.78820856	27.56445658	27.44322247	0.141473641	0.748	-0.765	PIS55604.1	orf19.5698	Putative mitochondrial ribosomal protein of the large subunit; transcript is upregulated in clinical isolates from HIV+ patients with oral candidiasis; Spider biofilm repressed
PIS52289.1	25.59322098	24.60570239	25.77011758	24.24006497	24.90144141	24.53216379	0.243939731	0.848	-0.765	PIS52289.1	PGA52	GPI-anchored cell surface protein of unknown function; Hap43p-repressed gene; fluconazole-induced; possibly an essential gene, disruptants not obtained by UAU1 method
PIS52177.1	27.39257713	28.54208601	27.08964876	27.40202583	26.8283246	26.49539481	0.334747689	0.888	-0.766	PIS52177.1	MED16	Putative RNA polymerase II mediator complex subunit; induced by nitric oxide
PIS58896.1	26.20732319	26.54343297	26.03161221	26.11418296	25.05776378	25.30805191	0.278575631	0.867	-0.767	PIS58896.1	DOM34	Endonuclease involved in regulation of translation; stimulates translation of protein O-mannosyltransferase family which includes PMT1, PMT2, PMT4, PMT5, and PMT6; peloto ortholog; Hap43-repressed gene
PIS58332.1	24.52023614	24.77445649	24.77272618	25.1118857	23.97550261	22.67369501	0.377633355	0.901	-0.769	PIS58332.1	orf19.7450	Ortholog(s) have myosin II tail binding, protein-macromolecule adaptor activity, role in protein localization to cell division site, septin ring assembly and cellular bud neck, cellular bud neck septin ring localization
PIS58829.1	27.25303027	28.75801208	27.73634442	26.86948058	26.82860563	27.73559147	0.36604735	0.898	-0.771	PIS58829.1	orf19.3016	Ortholog of C. dubliniensis CD36 : Cd36_03040, C. parapsilosis CDC317 : CPAR2_108610, C. auris B8441 : B9J08_000283, Debaryomyces hansenii CBS767 : DEHA2G16500g and Pichia stipitis Pignal : PICST_32835
PIS48329.1	27.55580108	27.8809232	27.36794825	26.45217179	26.96137253	27.07256508	0.286459657	0.87	-0.773	PIS48329.1	orf19.3548	Ortholog(s) have mitochondrial inner membrane localization

PIS56530.1	23.72450364	23.65163627	27.02295579	22.10513637	24.20252761	25.77152476	0.602241842	0.937	-0.773	PIS56530.1	orf19.5510	Ortholog(s) have role in negative regulation of transcription by RNA polymerase II, subtelomeric heterochromatin formation and CHRAC localization
PIS51692.1	27.78536109	26.69886608	27.75311815	26.96493398	26.41477166	26.52561889	0.152583383	0.765	-0.777	PIS51692.1	orf19.7166	Predicted mitochondrial cardiolipin-specific phospholipase; upregulated in an azole-resistant strain that overexpresses MDR1; induced by Mnl1 under weak acid stress; rat catheter and Spider biofilm induced
PIS52251.1	25.36568172	25.09611849	25.19729557	24.33591086	24.56802604	24.42391107	0.205105235	0.821	-0.777	PIS52251.1	PPE1	Protein similar to S. cerevisiae Ppe1p; shows genetic interaction with some genes involved in diploid filamentous growth and haploid invasive growth in S. cerevisiae
PIS55741.1	27.97874969	28.55321359	28.11652091	26.61130323	27.38422606	28.31745715	0.129533412	0.728	-0.778	PIS55741.1	MEX67	Nuclear export protein; has NTF2-like domain; interacts with Mtr2p via the NTF2-like domain
PIS50417.1	23.87064933	23.757142	24.86634218	23.38079949	23.14186141	23.6351503	0.185852942	0.804	-0.779	PIS50417.1	orf19.5587	Protein of unknown function; transcript is upregulated in clinical isolates from HIV+ patients with oral candidiasis
PIS58425.1	28.49777068	28.27984659	28.34783328	27.36626222	28.1521768	27.26916328	0.070126956	0.553	-0.779	PIS58425.1	SWC4	Subunit of the NuA4 histone acetyltransferase complex
PIS49757.1	25.58066225	24.07720149	26.58161225	23.86893975	25.31701886	24.71210759	0.272500426	0.864	-0.78	PIS49757.1	orf19.2893	Protein of unknown function; regulation correlates with clinical development of fluconazole resistance; transcript is upregulated in an RHE model of oral candidiasis
PIS49783.1	26.57143435	26.51639887	26.53459961	26.11393535	25.91207655	25.24556686	0.026787486	0.288	-0.784	PIS49783.1	orf19.6717	Putative serine hydrolase; Spider biofilm repressed
PIS50630.1	24.28990901	25.11292902	24.27005407	23.38003083	23.38249608	24.55555527	0.178813169	0.797	-0.785	PIS50630.1	ZCF27	Putative Zn(II)2Cys6 transcription factor
PIS58410.1	25.43958054	26.53235605	24.6035792	25.05808169	24.5125165	24.64786251	0.11465808	0.697	-0.786	PIS58410.1	orf19.2920	Ortholog(s) have role in ribosome biogenesis and cytosol localization

PIS58041.1	29.05980352	28.22802591	28.39821786	28.96100935	27.00631845	27.36194403	0.314715406	0.881	-0.786	PIS58041.1	orf19.4307	Ortholog(s) have role in ESCRT III complex assembly, late endosome to vacuole transport, late endosome to vacuole transport via multivesicular body sorting pathway, protein targeting to vacuole and cytoplasm, late endosome localization
PIS49829.1	23.49110076	27.19369918	23.73481115	23.76153023	23.85368835	24.4467479	0.412647155	0.909	-0.786	PIS49829.1	RAD23	S. cerevisiae Rad23 ortholog; binds damaged DNA; Spider biofilm repressed
PIS48226.1	22.012622	22.73003606	22.34894925	21.49114836	21.42458151	21.81075536	0.570730209	0.933	-0.788	PIS48226.1	CDC23	Similar to anaphase-promoting complex component; possibly transcriptionally regulated by Tac1p
PIS50446.1	25.45510394	24.37733037	26.10896387	24.76130073	24.76500516	24.05131417	0.15938404	0.774	-0.788	PIS50446.1	orf19.864	Ortholog(s) have role in DNA recombination, nuclear-transcribed mRNA catabolic process, 3'-5' exonucleolytic nonsense-mediated decay, nuclear-transcribed mRNA catabolic process, nonsense-mediated decay and cytoplasm localization
PIS55028.1	26.65580277	26.93655251	26.2950108	26.41898066	25.33514788	25.76848277	0.223861801	0.836	-0.788	PIS55028.1	ROD1	Protein similar to S. cerevisiae Rod1;a membrane protein with a role in drug tolerance; repressed by Rgt1; mutant is viable
PIS49508.1	20.70447986	22.19842395	21.13353719	20.17310328	20.63050133	20.86218682	0.197035172	0.814	-0.79	PIS49508.1	orf19.3418	Guanidinobutyrase (Gbase), enzyme involved in metabolism of guanidinobutyrate
PIS58021.1	31.92893819	32.23614222	31.82247082	31.37603923	30.98428696	31.2568401	0.080343407	0.596	-0.79	PIS58021.1	SDH12	Succinate dehydrogenase; soluble protein in hyphae; macrophage-downregulated protein level; downregulated by Efg1p; repressed by nitric oxide; protein present in exponential and stationary growth phase yeast cultures; Hap43p-repressed gene
PIS51795.1	28.03878398	29.33627796	27.90423305	28.01349359	27.28028793	27.61179924	0.336646918	0.889	-0.791	PIS51795.1	MMD1	Mitochondrial protein; possibly required for transamination of isoleucine; macrophage-downregulated protein abundance; rat catheter and Spider biofilm repressed
PIS55669.1	24.65055411	25.92094061	24.83257038	24.50854203	24.23308867	24.28605041	0.121426573	0.712	-0.792	PIS55669.1	orf19.1574	Putative TFIID subunit involved in RNA polymerase II transcription initiation; possibly an essential gene, disruptants not obtained by UAU1 method
PIS58522.1	25.48402472	26.37024067	24.55157061	24.22926692	24.40918105	25.39078377	0.414351584	0.909	-0.792	PIS58522.1	orf19.3273	Ortholog(s) have actin monomer binding, polysome binding, protein kinase inhibitor activity, ribosome binding activity and role in negative regulation of protein phosphorylation

PIS48325.1	27.67761506	27.9945129	28.22892632	27.00421771	27.06943893	27.45102654	0.156733558	0.771	-0.792	PIS48325.1	RPF2	Putative pre-rRNA processing protein; Hap43p-induced gene; mutation confers hypersensitivity to 5-fluorocytosine (5-FC), 5-fluorouracil (5-FU), and tubercidin (7-deazaadenosine)
PIS56733.1	26.10911501	25.63051102	26.22724361	25.04675852	25.74070535	24.80467327	0.261724254	0.858	-0.792	PIS56733.1	VPS53	Subunit of GARP (Golgi-associated retrograde protein) complex, which has roles in Golgi to vacuole transport, cellular sphingolipid homeostasis, retrograde transport, and filamentous growth
PIS55081.1	33.02184231	33.01538616	33.17996431	32.34650054	32.28960472	32.20076068	0.058772737	0.495	-0.793	PIS55081.1	PDC11	Pyruvate decarboxylase; antigenic; on hyphal not yeast cell surface; Hap43, Gcn4, Efg1, Efh1, Hsf1 regulated; fluconazole, farnesol induced; amino acid starvation repressed; flow model biofilm induced; Spider biofilm repressed
PIS56883.1	29.22478522	29.27189227	29.21009834	28.62750515	28.52257971	28.17217216	0.282758551	0.868	-0.795	PIS56883.1	APA2	Putative ATP adenylyltransferase II; regulated by Gcn4; repressed by amino acid starvation (3-AT); induced by prostaglandins; Hap43-repressed; Spider biofilm repressed
PIS52018.1	28.31828538	27.76230108	27.96646442	27.83502488	26.9924373	26.83461917	0.094075799	0.643	-0.795	PIS52018.1	GCD7	Putative translation initiator; downregulated in the presence of human whole blood or polymorphonuclear (PMN) cells
PIS48819.1	28.24502655	28.33974626	28.82329516	27.71157149	27.76491481	27.54398572	0.394787346	0.905	-0.796	PIS48819.1	ERG12	Ortholog(s) have mevalonate kinase activity and role in ergosterol biosynthetic process, farnesyl diphosphate biosynthetic process, mevalonate pathway, isopentenyl diphosphate biosynthetic process, mevalonate pathway
PIS48230.1	32.82074369	32.81040046	32.881756	32.39830999	31.76110258	31.96100693	0.100111134	0.661	-0.797	PIS48230.1	ACO1	Aconitase; induced in high iron; 2 upstream CCAAT motifs; amino acid starvation (3-AT), amphotericin B, phagocytosis, farnesol induced; Hap43, fluconazole-repressed; Gcn4-regulated; antigenic in infection; flow and Spider biofilm repressed
PIS51542.1	26.52444898	25.50661509	27.00956193	26.42325626	25.0383164	25.18742088	0.153218469	0.766	-0.797	PIS51542.1	TIM17	Predicted component of the Translocase of the Inner Mitochondrial membrane (TIM23 complex), involved in protein import into mitochondria
PIS50569.1	28.10296664	27.35957557	27.9347282	27.65392314	26.72279436	26.62830243	0.254386541	0.854	-0.797	PIS50569.1	TIP41	Protein involved in TOR signaling pathway; regulates protein phosphatase 2A (PP2A) activity; regulates Rad53p during recovery from DNA damage
PIS50478.1	24.55350854	24.63521116	25.19650409	24.16697756	23.6692498	24.14972018	0.361751689	0.896	-0.8	PIS50478.1	PPR1	Transcription factor with zinc cluster DNA-binding motif involved in regulation of purine catabolism; has similarity to S. cerevisiae Ppr1p, which is a transcription factor involved in the regulation of uracil biosynthesis genes

PIS56561.1	30.83004711	30.54586839	30.9094757	29.59531613	30.09662278	30.18790962	0.069308942	0.549	-0.802	PIS56561.1	PNP1	Purine nucleoside phosphorylase; metabolizes inosine and guanosine nucleosides; in the nicotinamide riboside salvage pathway; Spider biofilm repressed
PIS54915.1	24.25978313	24.02165305	24.58863767	24.35188594	23.83738007	22.27319201	0.53597994	0.929	-0.803	PIS54915.1	NUF2	Kinetochore component; amount of Nuf2p and Mtw1p protein detected at each centromere is consistent with a single kinetochore microtubule attachment site
PIS51505.1	26.149077	24.79668453	24.96608539	23.77589223	23.91454722	25.80692895	0.31903511	0.883	-0.805	PIS51505.1	orf19.1012	Ortholog(s) have role in Golgi to vacuole transport, endosome to plasma membrane protein transport, retrograde transport, endosome to Golgi and AP-1 adaptor complex, Golgi apparatus, endosome localization
PIS51796.1	26.50771358	26.0402082	26.97630509	25.31541586	25.94724529	25.84541936	0.294436704	0.874	-0.805	PIS51796.1	orf19.6220	Ortholog(s) have role in positive regulation of transcription elongation by RNA polymerase II and CCR4-NOT core complex localization
PIS58221.1	34.07171402	33.4508522	33.83781711	32.87321988	33.09454589	32.97666528	0.077426397	0.585	-0.805	PIS58221.1	RPP0	Putative ribosomal protein; antigenic in mouse; repressed upon phagocytosis by murine macrophage; induced by Tbf1; overlaps orf19.7014; Spider biofilm repressed
PIS58629.1	29.6238026	30.34193521	29.71585789	29.06157454	29.08770629	29.11142751	0.09088795	0.633	-0.807	PIS58629.1	IMH3	Inosine monophosphate (IMP) dehydrogenase; enzyme of GMP biosynthesis; target of mycophenolic acid and mizoribine monophosphate; antigenic during infection; repressed in core stress response; snoRNA snR54 encoded within IMH3 intron
PIS54817.1	24.99588244	26.24443044	24.90221961	25.71657304	24.62207595	23.38399285	0.266052821	0.861	-0.807	PIS54817.1	MED5	RNA polymerase II mediator complex subunit; transcription positively regulated by Tbf1p
PIS59001.1	27.50574336	26.63443002	27.4362072	26.52609682	26.53101175	26.09407389	0.282034261	0.868	-0.808	PIS59001.1	orf19.6284	Ortholog(s) have signal recognition particle binding activity, role in protein targeting to ER and endoplasmic reticulum membrane, signal recognition particle receptor complex localization
PIS49459.1	20.23832947	19.90023624	19.94609524	19.45240114	19.40054527	18.80156597	0.466441561	0.919	-0.81	PIS49459.1	orf19.811	Protein of unknown function; mutant is viable; Hap43-repressed
PIS51116.1	26.19186736	27.62326128	26.8565198	26.80971334	26.03387717	25.39008286	0.153197653	0.766	-0.813	PIS51116.1	FDH1	Formate dehydrogenase; oxidizes formate to CO2; Mig1 regulated; induced by macrophages; fluconazole-repressed; repressed by Efg1 in yeast, not hyphal conditions; stationary phase enriched; rat catheter and Spider biofilm induced

PIS58888.1	23.64811079	25.11469331	23.6582337	23.07217176	23.18885382	23.7121653	0.149048529	0.76	-0.816	PIS58888.1	orf19.2426	Predicted HD domain metal dependent phosphohydrolase; Spider biofilm repressed
PIS51180.1	25.13262301	25.87244275	25.22420526	24.34292383	23.7422141	25.68136576	0.196801651	0.814	-0.821	PIS51180.1	FGR34	Protein lacking an ortholog in S. cerevisiae; transposon mutation affects filamentous growth
PIS58131.1	28.95329258	28.65788522	29.57387971	28.91062316	28.03372649	27.77329158	0.211888559	0.827	-0.822	PIS58131.1	COQ4	Protein with a putative role in coenzyme Q biosynthesis; transcriptionally induced by interaction with macrophage; Hap43p-repressed gene
PIS50411.1	30.73584949	30.38428866	30.19618708	29.67661961	29.74348745	29.42068968	0.028501198	0.3	-0.825	PIS50411.1	FUN12	Functional homolog of S. cerevisiae Fun12 translation initiation factor eIF5B; genes encoding ribosomal subunits, translation factors, and tRNA synthetases are downregulated upon phagocytosis by murine macrophage
PIS52157.1	27.71735594	27.47404226	27.76987734	26.45827482	26.78874378	27.23243109	0.237220568	0.844	-0.827	PIS52157.1	orf19.3124	Ortholog(s) have mRNA binding, metalloaminopeptidase activity, role in negative regulation of gene expression and cytosolic ribosome localization
PIS54945.1	24.49008445	23.41221735	24.12610555	23.54534849	23.02162197	22.98074794	0.27307275	0.864	-0.827	PIS54945.1	orf19.7368	Ortholog(s) have mRNA binding, poly(U) RNA binding, ribosome binding activity and role in nuclear-transcribed mRNA catabolic process, nonsense-mediated decay, regulation of mRNA stability, stress granule assembly, translational termination
PIS51309.1	26.54840979	26.86205447	25.14875911	25.70911458	25.084197	25.28296225	0.299584908	0.876	-0.828	PIS51309.1	orf19.5103	Protein with a predicted phosphoglycerate mutase family domain; Hap43-repressed; clade-associated gene expression; induced by hypoxia
PIS48506.1	25.5810241	26.27823435	25.9916391	24.32722188	25.20153074	25.82958799	0.302238363	0.877	-0.831	PIS48506.1	orf19.787.1	Protein of unknown function; ORF added to Assembly 21 based on comparative genome analysis; protein detected by mass spec in stationary phase cultures
PIS54853.1	27.1665191	26.1944795	27.29750923	26.42881543	26.54870628	25.17262042	0.301493224	0.876	-0.836	PIS54853.1	orf19.4479	Putative U3-containing 90S preribosome subunit; Hap43-induced; repressed in core stress response; Spider biofilm induced
PIS51042.1	26.31018207	24.95027418	26.34452786	24.28556723	25.88819005	24.92242202	0.162676829	0.778	-0.836	PIS51042.1	orf19.7506	Ortholog(s) have ATPase, DNA binding, nucleosome binding activity, role in chromatin remodeling and Isw1b complex localization

PIS55064.1	28.96969856	28.62181569	28.63610595	27.83170181	28.09051389	27.79317933	0.287234606	0.87	-0.837	PIS55064.1	CGR1	Negative regulator of yeast-form growth; HSP70 family member; induced by growth cessation at yeast-hyphal transition or in planktonic growth; physically interacts with Msi3p; similar to rat anti-aging gene, SMP30, stationary phase enriched
PIS48243.1	30.99272152	30.48420774	31.44729936	30.61756526	29.90489232	29.89206459	0.181973245	0.8	-0.837	PIS48243.1	PFK2	Phosphofructokinase beta subunit; fructose 2,6-bisphosphate, AMP activated; ATP inhibited; phagocytosis, hyphal repressed; fluconazole-induced; stationary-phase enriched; flow model biofilm induced; rat catheter/Spider biofilm repressed
PIS58367.1	24.7071502	25.74844978	24.10468057	24.70568531	24.57250928	22.76945304	0.283218111	0.869	-0.838	PIS58367.1	orf19.804	Putative mitochondrial carrier family transporter; rat catheter biofilm induced
PIS55055.1	29.81296513	29.40010115	30.57918168	29.65789692	29.05803855	28.56080511	0.527207641	0.928	-0.839	PIS55055.1	orf19.4595	Ortholog of C. dubliniensis CD36 : Cd36_41860, C. parapsilosis CDC317 : CPAR2_400440, C. auris B8441 : B9J08_002205 and Candida tenuis NRRL Y-1498 : CANTEDRAFT_103033
PIS52001.1	28.73408157	26.07402205	27.43310217	26.64177724	26.70353233	26.37635438	0.150133203	0.762	-0.84	PIS52001.1	OLE1	Fatty acid desaturase, essential protein involved in oleic acid synthesis; required for aerobic hyphal growth and chlamydospore formation; subject to hypoxic regulation; fluconazole-induced; caspofungin repressed; Hap43p-induced
PIS58809.1	26.49600017	26.50876949	26.98462322	26.70934768	24.83712263	25.92172949	0.412595613	0.909	-0.84	PIS58809.1	VCX1	Putative H+/Ca2+ antiporter; Spider biofilm repressed
PIS56727.1	25.33853943	24.73975042	25.64693243	24.76418274	23.70316835	24.72740327	0.19141431	0.809	-0.843	PIS56727.1	orf19.6100	Cardiolipin synthase; ortholog of S. cerevisiae Crd1; transcript is upregulated in clinical isolates from HIV+ patients with oral candidiasis; mutants are viable; Spider biofilm repressed
PIS55681.1	24.43456104	24.12559178	25.56610039	23.72448944	24.58543711	23.27865556	0.322444628	0.884	-0.846	PIS55681.1	orf19.2749	BTB/POZ domain protein; induced by Mnl1 under weak acid stress; flow model biofilm induced; Spider biofilm induced
PIS52381.1	27.87641268	28.46983245	27.7397006	26.33786873	27.43260937	27.76791638	0.219163961	0.832	-0.849	PIS52381.1	orf19.326	Protein of unknown function; Spider biofilm repressed
PIS58326.1	27.15593513	25.6284669	27.75409867	26.83198279	26.09730492	25.05057532	0.339250978	0.89	-0.853	PIS58326.1	SAM4	Putative S-adenosylmethionine-homocysteine methyltransferase; Hap43-repressed; alkaline induced; Spider biofilm repressed

PIS51956.1	25.8486761	22.40267427	25.2987649	23.72512167	25.19113077	22.06088787	0.410384592	0.908	-0.858	PIS51956.1	MDS3	TOR signaling pathway component; required for growth and hyphal formation at alkaline pH, for full virulence in a mouse model of systemic infection and for SD and Spider medium biofilm formation; role in chlamydospore formation
PIS48647.1	28.23133385	24.58370918	26.54719113	24.59950313	26.26076534	25.92313928	0.384590061	0.902	-0.86	PIS48647.1	orf19.3228	Putative endosomal transmembrane protein; Hap43p-induced; mutation confers hypersensitivity to amphotericin B
PIS50461.1	28.7925299	28.46999731	28.46822734	27.7575261	27.55543287	27.82953083	0.039257661	0.374	-0.863	PIS50461.1	HSP78	Heat-shock protein; regulated by macrophage response, Nrg1, Mig1, Gcn2, Gcn4, Mnl1p; heavy metal (cadmium) stress-induced; stationary phase enriched protein; rat catheter and Spider biofilm induced
PIS55682.1	24.22696311	23.99604419	24.49526261	23.60627047	24.8938331	21.62594196	0.237855403	0.845	-0.864	PIS55682.1	ARG83	GAL4-like Zn(II)2Cys6 transcription factor; clade-associated expression; null shows abnormal regulation of invasive colony growth, is unable to utilize proline as a nitrogen source; flow model biofilm induced
PIS56715.1	32.17550103	32.20807299	32.20613381	31.24504758	31.43753809	31.31625072	0.063064959	0.518	-0.864	PIS56715.1	orf19.3982	Maltase; induced during growth on sucrose; induced by alpha pheromone in SpiderM medium; early-stage flow model biofilm indced
PIS58742.1	29.41726603	29.24369118	29.99330392	29.29878603	28.20945606	28.54444512	0.057897584	0.49	-0.867	PIS58742.1	RPA135	Putative RNA polymerase I subunit A135; repressed by prostaglandins
PIS58802.1	24.17081514	26.4108912	23.4660122	24.4163274	23.04217076	23.98647815	0.229889737	0.84	-0.868	PIS58802.1	ADA2	Zinc finger and homeodomain transcriptional coactivator; role in cell wall integrity and in sensitivity to caspofungin; required for the normal transcriptional response to caspofungin; required for yeast cell adherence to silicone substrate
PIS50470.1	20.58586838	20.73021615	23.51440242	20.47164533	20.94940799	20.80406796	0.250953715	0.853	-0.868	PIS50470.1	PSD2	Ortholog(s) have phosphatidylserine decarboxylase activity, role in phosphatidylcholine biosynthetic process, phosphatidylethanolamine biosynthetic process and endosome localization
PIS50382.1	26.03982064	28.33203804	26.29679687	25.24334322	25.31745136	27.49273146	0.24673011	0.85	-0.872	PIS50382.1	PRS5	Putative 5-phospho-ribosyl-1(alpha)-pyrophosphate synthetase; Hap43-induced; repressed during core stress response
PIS48547.1	27.8417834	28.84067232	28.14629967	27.61921565	27.40294488	27.18192107	0.053871659	0.466	-0.875	PIS48547.1	POL30	Proliferating cell nuclear antigen (PCNA), forms homotrimeric sliding clamp for DNA polymerases; RNA abundance regulated by tyrosol, cell density; induced by flucytosine, interaction with macrophages; stationary phase enriched protein

PIS54565.1	28.21165869	27.80124591	28.07500068	27.54392391	27.20131939	26.71872187	0.20899515	0.824	-0.875	PIS54565.1	TRY3	RING-finger transcription factor; regulator of yeast form adherence; required for yeast cell adherence to silicone substrate; Spider biofilm induced
PIS48349.1	26.22170047	25.84580115	24.65681176	25.14198045	24.19539928	24.75916036	0.205322055	0.822	-0.876	PIS48349.1	orf19.1249	Ortholog(s) have 1-(5-phosphoribosyl)-5-[(5-phosphoribosylamino)methylideneamino]imidazole-4-carboxamide isomerase activity and role in histidine biosynthetic process
PIS55059.1	25.21308119	23.29244416	24.88331788	25.03240696	23.10356927	22.61632338	0.244783735	0.849	-0.879	PIS55059.1	HCA4	Putative role in regulation of cell wall biogenesis; Hap43p-induced gene; possibly an essential gene, disruptants not obtained by UAU1 method; flow model and rat catheter biofilm induced
PIS54857.1	30.83758814	31.63501646	30.82157715	30.89321523	29.76325682	30.00173938	0.15803395	0.772	-0.879	PIS54857.1	orf19.6952	Ortholog(s) have DNA binding, chromatin binding, histone deacetylase activity, role in negative regulation of transcription by RNA polymerase II, regulatory ncRNA-mediated gene silencing and HDA1 complex, cytosol, nucleus localization
PIS50387.1	24.71341078	25.75291559	26.14261206	25.23183686	24.38685993	24.34755123	0.180667166	0.799	-0.881	PIS50387.1	orf19.5295	Protein with a predicted endonuclease/exonuclease/phosphatase family domain and a carbon catabolite repressor protein 4 domain; induced by alpha pheromone in SpiderM medium
PIS51404.1	25.16147472	26.89793115	25.70324732	25.38351577	25.82374769	23.90940107	0.194941326	0.813	-0.882	PIS51404.1	orf19.4394	Protein of unknown function; induced by alpha pheromone in SpiderM medium
PIS56842.1	28.14841856	26.16901628	27.87717484	26.87036623	27.10351845	25.56641625	0.202155856	0.819	-0.885	PIS56842.1	ARL1	Putative GTPase in the late Golgi involved in regulation of polarized growth and secretion; mutation confers dose-dependent sensitivity to Brefeldin A
PIS49800.1	26.15688076	25.68393319	26.65221346	24.79267966	25.49467977	25.55210721	0.266767453	0.861	-0.885	PIS49800.1	ATG9	Protein similar to S. cerevisiae Atg9; required for early step in autophagy; required for cytoplasm to vacuole trafficking of Lap41; Spider biofilm induced
PIS51500.1	35.47883722	34.77833806	35.61004497	34.57278852	34.25444386	34.37843232	0.212020187	0.827	-0.887	PIS51500.1	ALD5	NAD-aldehyde dehydrogenase; decreased expression in fluconazole-resistant isolate, or in hyphae; biofilm induced; fluconazole-downregulated; protein abundance is affected by URA3 expression in the CAI-4 strain; stationary phase enriched
PIS56945.1	26.35490739	27.1173762	26.20838314	26.52475828	25.05653704	25.43688446	0.126973292	0.723	-0.887	PIS56945.1	orf19.5356	Protein with a predicted role in cell wall integrity; repressed in core stress response

PIS58494.1	30.30692478	30.50493337	30.50421935	29.69772711	29.27610724	29.67501478	0.017334983	0.204	-0.889	PIS58494.1	orf19.2737	Carbohydrate kinase domain-containing protein; Spider biofilm induced
PIS56932.1	27.67057749	26.67090344	28.50920029	27.80437051	26.20267939	26.17553612	0.219966789	0.833	-0.889	PIS56932.1	RIB5	Putative riboflavin synthase; fungal-specific (no human or murine homolog); farnesol-downregulated; protein present in exponential and stationary growth phase yeast cultures
PIS51592.1	29.11162323	26.68336266	29.8071896	27.7834059	27.34031876	27.80767767	0.332198293	0.887	-0.89	PIS51592.1	TES1	Putative acyl-CoA thioesterase
PIS50421.1	25.29008577	26.56872268	26.83976634	26.24686503	24.6317769	25.14691038	0.465746726	0.919	-0.891	PIS50421.1	HSE1	ESCRT-0 complex subunit; SH3-domain-containing protein
PIS54705.1	28.73283572	29.35517239	28.10868709	26.97760522	28.01493498	28.51324617	0.110413009	0.688	-0.897	PIS54705.1	ERF1	Putative translation release factor 1, which interacts with stop codons and promotes release of nascent peptides from ribosomes; Hap43p-induced gene
PIS52098.1	28.28155861	27.39504976	26.01113052	24.89549865	25.79183625	28.30757407	0.326016062	0.885	-0.898	PIS52098.1	IFK2	Putative thiol-specific monooxygenase; mutant is viable; flow model biofilm induced
PIS58111.1	24.07762252	23.73405883	24.8157884	24.52186106	22.89821512	22.51336866	0.162792167	0.778	-0.898	PIS58111.1	orf19.6271	Ortholog(s) have role in mRNA splicing, via spliceosome and euchromatin localization
PIS49697.1	25.09561243	24.60484778	23.30029293	23.04883417	23.6056943	23.64739265	0.299414591	0.876	-0.9	PIS49697.1	orf19.7199	Ortholog(s) have role in post-translational protein targeting to endoplasmic reticulum membrane and TRC complex, cytoplasm localization
PIS49535.1	23.95529348	24.73537837	24.3132229	22.5017138	23.28289986	24.51711673	0.114068896	0.696	-0.901	PIS49535.1	CPP1	VH1 family MAPK phosphatase; regulates Cst20-Hst7-Cek1-Cph1 filamentation pathway; negatively regulates mating, represses yeast-hyphal switch; required for virulence in mice; yeast-enriched; induced by alpha pheromone in SpiderM medium;
PIS52000.1	30.60220119	29.95532126	30.5699674	29.49395885	29.60948838	29.30282931	0.023937313	0.267	-0.907	PIS52000.1	SDS24	Protein similar to S. cerevisiae Sds24 involved in cell separation during budding; transcript regulated by Mig1 and Tup1; fluconazole-induced; flow model biofilm induced

PIS51806.1	21.92471058	21.46608253	23.83426672	22.12460293	21.1996756	21.17531636	0.332642434	0.888	-0.908	PIS51806.1	orf19.3303	Ortholog(s) have tRNA methyltransferase activity and role in tRNA methylation, wybutosine biosynthetic process
PIS56808.1	21.0650136	24.04762077	23.16220053	20.02248263	21.19674062	24.32778557	0.425841641	0.912	-0.909	PIS56808.1	orf19.2314	Protein with a role in nucleolar integrity and processing of pre-rRNA; mutation confers hypersensitivity to 5-fluorocytosine (5-FC), 5-fluorouracil (5-FU), and tubercidin (7-deazaadenosine); Hap43-induced; Spider biofilm
PIS51056.1	30.01856041	30.8071283	30.29871292	30.08904542	29.1817006	29.12268419	0.20224222	0.819	-0.91	PIS51056.1	XYL2	D-xylulose reductase; immunogenic in mice; soluble protein in hyphae; induced by caspofungin, fluconazole, Hog1 and during cell wall regeneration; Mnl1-induced in weak acid stress; stationary phase enriched; flow model biofilm induced
PIS54976.1	30.71640174	30.69748603	30.63580284	29.98096939	29.55561029	29.78029981	0.236622739	0.844	-0.911	PIS54976.1	orf19.7393	Predicted NAD+/NADH kinase; possible role in cellular iron ion homeostasis; Spider biofilm induced
PIS55750.1	23.88264725	24.76382953	23.12970579	23.33670788	22.63200192	23.0600982	0.037408449	0.363	-0.916	PIS55750.1	orf19.5247	Has domain(s) with predicted phosphatase activity
PIS48513.1	30.80519987	32.21982007	30.05634561	29.88854636	30.23769218	30.20137958	0.203066056	0.82	-0.918	PIS48513.1	PDB1	Putative pyruvate dehydrogenase; fluconazole-induced; protein level decreases in stationary phase cultures; Spider biofilm repressed
PIS55062.1	31.41220844	31.48238323	31.22365503	30.42053094	30.3864143	30.55367695	0.016598546	0.195	-0.919	PIS55062.1	orf19.2720	Cytosolic chaperonin Cct ring complex subunit; role in the assembly of actin and tubulins; Spider biofilm repressed
PIS50340.1	26.56986399	25.75151919	26.96560976	24.97726146	25.76145697	25.77638551	0.253542797	0.854	-0.924	PIS50340.1	DTD2	Ortholog(s) have D-leucyl-tRNA(Leu) deacylase activity, D-tyrosyl-tRNA(Tyr) deacylase activity and role in D-leucine catabolic process, D-tyrosine catabolic process, tRNA metabolic process
PIS55719.1	26.72695962	25.63530975	27.55366632	25.06503606	25.99823612	26.08164721	0.265411864	0.86	-0.924	PIS55719.1	PBS2	MAPK kinase (MAPKK); role in osmotic and oxidative stress responses, oxidative stress adaptation; required for stress regulation of Hog1p localization and activity; functional homolog of S. cerevisiae Pbs2p
PIS58697.1	27.58123556	28.47022226	26.21285856	26.92775914	26.66914439	25.89473279	0.137638358	0.742	-0.924	PIS58697.1	SLA1	Protein required for assembly of the cortical actin cytoskeleton; contains three SH3 domains; transcription is regulated by Nrg1 and Mig1; flow model biofilm repressed

PIS52172.1	32.38932455	32.51722085	32.55481316	31.38336939	31.64071297	31.64954714	0.00906996	0.112	-0.929	PIS52172.1	PDA1	Putative pyruvate dehydrogenase alpha chain; fluconazole-induced; protein present in exponential and stationary growth phase yeast cultures; Hap43-induced; Spider biofilm repressed
PIS51779.1	27.04482963	26.4169999	26.06629153	25.2444217	25.85040116	25.6438158	0.181921247	0.8	-0.93	PIS51779.1	orf19.6850	Putative transcription factor with C3HC4 zinc finger DNA-binding motif; mutants are viable
PIS58903.1	25.77130972	27.5220954	25.56529915	24.96786807	25.11638111	25.97765793	0.152299222	0.765	-0.932	PIS58903.1	SYN8	Putative endosomal SNARE; role in protein transport; rat catheter and Spider biofilm induced
PIS55657.1	28.83448667	28.79387845	29.03429743	27.69067618	28.01267813	28.15398026	0.174258652	0.792	-0.935	PIS55657.1	URA5	Putative orotate phosphoribosyltransferase; protein abundance is affected by URA3 expression in the CAI-4 strain background; flucytosine induced; protein level decreased in stationary phase cultures
PIS51083.1	25.28551359	26.05238209	25.24561018	24.75484439	25.35234994	23.66693264	0.032651038	0.332	-0.936	PIS51083.1	orf19.4390	Protein of unknown function; repressed by alpha pheromone in SpiderM medium; transcript induced by Mnl1 under weak acid stress
PIS51392.1	30.15241848	29.32679009	30.88180393	29.29341717	29.12384939	29.12533542	0.603009207	0.937	-0.939	PIS51392.1	HMT1	Major type I protein arginine methyltransferase (PRMT); involved in asymmetric dimethylation of arginine residues; involved in nuclear export of Npl3p; Spider biofilm repressed
PIS48438.1	26.60536483	26.04432546	25.87592047	25.45419931	24.50944589	25.74149928	0.183996786	0.802	-0.94	PIS48438.1	APG7	Ortholog(s) have Atg12 activating enzyme activity, Atg8 activating enzyme activity
PIS51615.1	30.19568798	29.59737104	30.29437895	29.47128836	28.7661808	29.02542856	0.14710029	0.757	-0.942	PIS51615.1	PUF3	RNA-binding protein involved in regulation of mitochondrial biogenesis
PIS52054.1	30.3144647	30.54606882	30.26262131	29.76614021	29.2486639	29.25790783	0.190883746	0.809	-0.95	PIS52054.1	ADO1	Adenosine kinase; heterozygous null mutant is resistant to cordycepin in C. albicans fitness test; ketoconazole-induced; protein level decrease in stationary phase cultures; sumoylation target; Spider biofilm repressed
PIS48543.1	30.81368791	31.19864356	30.68862201	29.56102698	29.85935368	30.42501313	0.126456642	0.722	-0.952	PIS48543.1	orf19.4622	Ortholog(s) have transcription factor TFIIH holo complex binding activity

PIS54833.1	27.85591638	28.1774189	26.96513819	26.42350395	26.86033187	26.84372784	0.237268018	0.844	-0.957	PIS54833.1	orf19.6316	Predicted membrane transporter, member of the L-amino acid transporter-3 (LAT3) family, major facilitator superfamily (MFS)
PIS48280.1	28.88160503	29.40869325	29.11151453	27.78248379	27.94724932	28.79813661	0.014486545	0.174	-0.958	PIS48280.1	RNR21	Ribonucleoside-diphosphate reductase; regulated by tyrosol and cell density; ciclopirox olamine, fluconazole or flucytosine induced; regulated by Sef1, Sfu1, and Hap43
PIS48418.1	27.76495702	27.47423854	27.68643708	26.43185713	27.26799874	26.34879758	0.17479425	0.793	-0.959	PIS48418.1	orf19.5847	Ortholog(s) have RNA polymerase III activity, role in tRNA transcription by RNA polymerase III, termination of RNA polymerase III transcription and RNA polymerase III complex, chromatin localization
PIS56762.1	25.71609248	25.65199657	25.446474	23.05579903	25.42445165	25.45506835	0.224737901	0.836	-0.96	PIS56762.1	BRE1	Putative transcription factor with C3HC4 zinc finger DNA-binding motif; similar to S. cerevisiae Bre1p; transposon mutation affects filamentous growth
PIS51217.1	27.14826305	28.84724034	27.10138745	26.05248674	26.94618119	27.21430215	0.103143056	0.669	-0.961	PIS51217.1	orf19.2269	Putative 3-phosphoserine phosphatase; induced by benomyl or in azole-resistant strain that overexpresses MDR1; early-stage flow model biofilm induced; Spider biofilm repressed
PIS58102.1	29.54004506	29.71131389	29.94577419	28.80042459	28.74214355	28.76533307	0.20592791	0.822	-0.963	PIS58102.1	NRP1	Ortholog(s) have role in cellular response to temperature stimulus, regulation of heterochromatin formation and cytoplasmic stress granule, nucleus, protein aggregate center localization
PIS58314.1	32.88128089	33.09579941	32.85385297	32.19135188	31.78604452	31.96555286	0.161179233	0.776	-0.963	PIS58314.1	PGI1	Glucose-6-phosphate isomerase; enzyme of glycolysis; antigenic; Efg1-regulated; induced upon adherence to polystyrene; repressed by phagocytosis, human neutrophils; flow model biofilm induced; rat catheter and Spider biofilm repressed
PIS52317.1	25.86833681	27.51561361	25.89433042	25.54906011	25.50316646	25.32283915	0.166007688	0.782	-0.968	PIS52317.1	orf19.5238	Ortholog of C. dubliniensis CD36 : Cd36_11490, C. parapsilosis CDC317 : CPAR2_701040, C. auris B8441 : B9J08_003931 and Candida tenuis NRRL Y-1498 : CANTEDRAFT_104365
PIS52418.1	27.28474485	27.01147542	27.38974068	26.64645906	26.48472748	25.64670034	0.145879435	0.755	-0.969	PIS52418.1	orf19.4895	Ortholog of C. dubliniensis CD36 : Cd36_09650, C. parapsilosis CDC317 : CPAR2_805060, C. auris B8441 : B9J08_004034 and Candida tenuis NRRL Y-1498 : CANTEDRAFT_95780
PIS54910.1	26.84691432	28.01458237	25.64292472	24.99361841	26.38000757	26.21936545	0.19074846	0.809	-0.97	PIS54910.1	HST3	Histone H3K56 deacetylase; reduced copy number increases opaque cell formation; repressed by MMS, hydroxyurea and high-levels of hydrogen peroxide; Hap43p-induced; ectopic expression blocks genotoxin-induced switching; nicotinamide target

PIS58402.1	27.88173159	28.29162805	27.47348751	26.26952475	26.89406565	27.5659018	0.087712695	0.623	-0.972	PIS58402.1	ALG8	Putative glucosyltransferase involved in cell wall mannan biosynthesis; transcription is elevated in nik1 and sln1 homozygous null mutants, but not in the chk1 null mutant; possibly an essential gene, disruptants not obtained by UAU1 method
PIS51080.1	29.04005819	28.99013613	28.86314925	28.44902999	27.7089462	27.8180338	0.258568538	0.857	-0.972	PIS51080.1	orf19.2382	Protein similar to isoleucyl-tRNA synthetase; isoleucyl-tRNA synthetase is the target of drugs including the cyclic beta-amino acid icofungipen/PLD-118/BAY-10-8888 and mupirocin
PIS51849.1	28.10043134	29.28097175	27.6662244	26.95437903	27.66171898	27.51360788	0.227399129	0.838	-0.973	PIS51849.1	SET2	Ortholog(s) have RNA binding, histone H3K36 methyltransferase activity
PIS48603.1	25.80455502	25.55311904	24.93995876	25.08715209	25.21066062	23.07791818	0.512909299	0.926	-0.974	PIS48603.1	orf19.2307	Ortholog(s) have role in co-transcriptional mRNA 3'-end processing, cleavage and polyadenylation pathway, mRNA processing and chromatin, mRNA cleavage and polyadenylation specificity factor complex, nucleolus, nucleus localization
PIS50398.1	24.98065718	23.3478897	23.30349913	21.25613581	24.52173401	22.92019913	0.416836387	0.91	-0.978	PIS50398.1	GYP1	Putative Cis-golgi GTPase-activating protein; required for hyphen growth and virulence; transcript regulated by Nrg1, Mig1, and Tup1
PIS50558.1	26.37055523	25.01236674	25.39710614	24.4817664	24.76746865	24.57515149	0.127999328	0.725	-0.985	PIS50558.1	orf19.3945	Predicted COP9 signalosome complex subunit 12; flow model biofilm induced
PIS59020.1	29.4888752	28.19754878	29.77711373	28.66139233	28.08462432	27.76277647	0.226790709	0.838	-0.985	PIS59020.1	orf19.5006.1	Ortholog(s) have SNAP receptor activity and role in Golgi to plasma membrane transport, ascospore-type prospore membrane formation, endocytosis, exocytosis, vesicle fusion
PIS51712.1	26.09045847	26.47719662	26.49141475	26.07608465	24.51765294	25.47838883	0.333106458	0.888	-0.996	PIS51712.1	URA3	Orotidine-5'-phosphate decarboxylase; pyrimidine biosynthesis; gene used as genetic marker; decreased expression when integrated at ectopic chromosomal locations can cause defects in hyphal growth and virulence; Spider biofilm repressed
PIS58782.1	25.54188355	25.39497333	25.62936265	25.10706371	23.96522231	24.48557256	0.09688104	0.652	-1	PIS58782.1	orf19.3862	Putative intracellular transport protein; heterozygous null mutant displays sensitivity to rapamycin; expression upregulated during growth in the mouse cecum
PIS48328.1	27.27253555	27.4698811	27.19462081	26.35231767	26.24546043	26.30088462	0.036607083	0.358	-1.01	PIS48328.1	CDC21	Putative thymidylate synthase; flucytosine induced; rat catheter biofilm repressed; Spider biofilm repressed

PIS51336.1	27.87264228	26.54817625	27.60433429	26.05112395	26.99073745	25.94870015	0.059119243	0.497	-1.01	PIS51336.1	FAD1	Ortholog(s) have FMN adenylyltransferase activity, role in FAD biosynthetic process and cytoplasm localization
PIS52035.1	30.31243022	30.79381571	29.91814922	29.46635876	28.94897679	29.58846566	0.027718183	0.295	-1.01	PIS52035.1	HOM2	Aspartate-semialdehyde dehydrogenase; forms a homodimer; conserved in bacteria, archaea, and fungi but not in mammals; ketoconazole-repressed; protein present in exponential and stationary growth phase yeast cultures; GlcNAc-induced protein
PIS55518.1	24.90875794	25.31596112	25.08904113	24.35119182	23.37302468	24.55414272	0.377038185	0.9	-1.01	PIS55518.1	orf19.4430	Ortholog(s) have ubiquitin binding activity and role in proteasome-mediated ubiquitin-dependent protein catabolic process
PIS54753.1	24.63958548	25.76034084	25.48070825	24.17701185	24.66457316	24.01604658	0.025857651	0.282	-1.01	PIS54753.1	orf19.5813	Putative adhesin-like protein; upregulated during growth in the mouse cecum; flow model, rat catheter and Spider biofilm induced
PIS51321.1	25.4287632	23.660547	25.8559398	25.28867732	24.21687434	22.3994747	0.198444605	0.816	-1.01	PIS51321.1	orf19.7458	Ortholog of C. dubliniensis CD36 : Cd36_86660, Candida tenuis NRRL Y-1498 : cten_CGOB_00028, Candida tropicalis NEW ASSEMBLY : CTRG1_05695 and Candida tropicalis MYA-3404 : CTRG_05695
PIS50584.1	28.00915878	26.8520831	28.53244973	26.92551369	26.55498483	26.8941283	0.087524122	0.622	-1.01	PIS50584.1	PAM18	Predicted component of the presequence translocase-associated import motor (PAM complex) involved in protein import into mitochondrial matrix; rat catheter biofilm induced
PIS54816.1	25.73752253	25.59104088	25.79199877	25.22030567	24.22690092	24.6295605	0.175771737	0.794	-1.01	PIS54816.1	POL3	Large subunit of DNA polymerase III; partially complements defects of an S. cerevisiae cdc2 mutant; differing reports about periodic (G1/S) or non-periodic mRNA expression through cell cycle; Hap43p-repressed
PIS48655.1	25.0701459	23.84657007	25.16023717	22.39148707	24.06494258	24.59074662	0.087316243	0.621	-1.01	PIS48655.1	SET1	Lysine histone methyltransferase; methylates histone H3 K4; regulates of white-opaque switch, epithelial cell adhesion, agar-embedded filamentation, virulence in mice; unique N-terminus immunogenic in human; rat catheter biofilm repressed
PIS51278.1	27.53812461	25.89303674	27.25896049	28.06989325	23.7334725	25.82746543	0.335407697	0.888	-1.02	PIS51278.1	LYS144	Zn(II)2Cys6 transcription factor; has similarity to S. cerevisiae Lys14, involved in the regulation of lysine biosynthesis genes
PIS58270.1	32.82178081	33.80987845	32.56767759	31.90917404	32.08175827	32.14833188	0.193608383	0.811	-1.02	PIS58270.1	MDH1-1	Predicted malate dehydrogenase precursor; macrophage-induced transcript; protein present in exponential and stationary growth phase yeast cultures; Spider biofilm repressed

PIS56813.1	29.49929059	28.74269062	28.54256582	27.56585557	28.06607638	28.10705351	0.064162102	0.524	-1.02	PIS56813.1	orf19.1833	Ortholog(s) have pseudouridine synthase activity, role in box H/ACA RNA 3'-end processing, mRNA pseudouridine synthesis, rRNA processing, rRNA pseudouridine synthesis, snRNA pseudouridine synthesis and box H/ACA snoRNP complex
PIS58200.1	27.03618453	24.20244098	27.7849974	25.09185822	25.29805401	25.56602387	0.380062539	0.901	-1.02	PIS58200.1	orf19.7473	Ortholog(s) have role in endocytosis and actin cortical patch localization
PIS49755.1	24.05432336	25.83757426	22.9403149	20.92268657	24.71217797	24.13012753	0.422393903	0.911	-1.02	PIS49755.1	orf19.7497	Ortholog(s) have ubiquitin-protein transferase activity, role in ubiquitin-dependent protein catabolic process and Cul3-RING ubiquitin ligase complex localization
PIS58355.1	24.96301164	25.48326258	24.45700514	24.73164963	24.06745971	23.02941264	0.137410464	0.742	-1.02	PIS58355.1	RTT109	Histone acetyltransferase, mutants are sensitive to DNA damage, show decreased virulence in mice, decreased white-to-opaque switching and increased susceptibility to killing by macrophages
PIS56901.1	25.20802914	26.37345845	24.77777005	24.57847694	24.57666064	24.13613105	0.014061136	0.17	-1.02	PIS56901.1	THR1	Putative homoserine kinase; regulated by Tup1; amphotericin B repressed; regulated by Gcn2 and Gcn4; Spider biofilm repressed
PIS48157.1	27.23516923	27.75906535	26.73583124	26.9702827	25.83914982	25.84473087	0.082639069	0.605	-1.03	PIS48157.1	RBT5	GPI-linked cell wall protein; hemoglobin utilization; Rfg1, Rim101, Tbf1, Fe regulated; Sfu1, Hog1, Tup1, serum, alkaline pH, antifungal drugs, geldamycin repressed; Hap43 induced; required for RPMI biofilms; Spider biofilm induced
PIS49747.1	25.34707585	25.8985811	25.45567711	23.67442238	24.86035246	25.03560721	0.044722975	0.411	-1.04	PIS49747.1	GLY1	L-threonine aldolase; complements glycine auxotrophy of S. cerevisiae shm1 shm2 gly1-1 triple mutant; macrophage/pseudohyphal-induced; the GLY1 locus has an RFLP and is triploid in strain SGY269; flow model biofilm induced
PIS51230.1	29.98672772	29.4510696	30.01820639	28.29882353	28.89536484	29.12921231	0.010777327	0.131	-1.04	PIS51230.1	orf19.2257	Predicted ER protein involved in ER-nucleus signaling; Spider biofilm repressed
PIS58142.1	27.97500459	28.72318732	28.06071394	28.21356392	26.04522356	27.37346139	0.229667183	0.839	-1.04	PIS58142.1	orf19.3083	Putative lipid phosphatase of the endoplasmic reticulum; role in DNA repair, actin cytoskeleton organization, cellular manganese ion homeostasis; Spider biofilm repressed
PIS50302.1	27.44397924	28.50521025	26.97328022	25.48288452	26.99392221	27.28341725	0.204632067	0.821	-1.05	PIS50302.1	CPR6	Putative peptidyl-prolyl cis-trans isomerase; macrophage/pseudohyphal-repressed; heavy metal (cadmium) stress-induced; heterozygous null mutant displays sensitivity to virgineone; rat catheter biofilm induced

PIS51902.1	33.53374609	34.34915154	33.34854909	32.76438263	32.63563311	32.64627033	0.110628422	0.688	-1.06	PIS51902.1	ATP2	F1 beta subunit of F1F0 ATPase complex; antigenic in human, mice; induced by ciclopirox olamine; caspofungin repressed; macrophage/pseudohyphal-induced; detected during exponential and stationary growth phases; Spider biofilm repressed
PIS58945.1	27.50429622	27.56793404	27.51351835	26.51875703	25.9934724	26.87895667	0.167156262	0.784	-1.06	PIS58945.1	GLE2	Putative nuclear pore complex; possibly an essential gene, disruptants not obtained by UAU1 method; rat catheter biofilm repressed
PIS48274.1	28.8251598	29.07433867	28.07552286	28.00724354	27.58989698	27.19030883	0.139180544	0.745	-1.06	PIS48274.1	orf19.1030	Putative peptidyl-prolyl cis-trans isomerase
PIS52094.1	23.54401186	24.16828671	24.73652105	24.61149654	22.60792459	22.05118784	0.229336831	0.839	-1.06	PIS52094.1	orf19.433	Ortholog(s) have guanyl-nucleotide exchange factor activity, role in intra-Golgi vesicle-mediated transport, protein-containing complex assembly and TRAPPII protein complex, trans-Golgi network localization
PIS52228.1	26.68400998	27.05315064	26.87197587	25.70581344	26.26720211	25.44893931	0.146897149	0.757	-1.06	PIS52228.1	orf19.6923	Ortholog(s) have chromatin binding activity, role in RNA polymerase II preinitiation complex assembly, transcription by RNA polymerase II and transcription factor TFIID complex localization
PIS48808.1	24.16333393	24.78099215	25.31835153	23.0820501	24.33553341	23.67771743	0.169783313	0.787	-1.06	PIS48808.1	VAM3	Predicted syntaxin-like vacuolar t-SNARE, involved in vacuolar inheritance
PIS51135.1	24.93200041	25.31490788	24.46348447	23.52280909	24.26113709	23.72336861	0.107986411	0.682	-1.07	PIS51135.1	MET2	Homoserine acetyltransferase; Hap43p-, Gcn4p-regulated; macrophage/pseudohyphal-repressed; not highly biofilm induced, in contrast to many sulfur amino acid metabolic genes; no human or murine homolog; virulence-group-correlated expression
PIS48806.1	25.65934211	26.60614385	26.2110981	24.19716396	25.5142569	25.55410477	0.098091973	0.655	-1.07	PIS48806.1	orf19.5880	Putative voltage-gated chloride channel; predicted role in copper ion and iron ion homeostasis; flow model biofilm induced
PIS51190.1	25.6559932	25.43654417	25.9680305	24.82907736	24.36234821	24.66568322	0.004287399	0.0441	-1.07	PIS51190.1	orf19.7593	Putative asparaginase; predicted role in asparagine catabolism; Spider biofilm induced
PIS48630.1	28.51248904	27.35821384	28.160661	28.22059943	26.34601893	26.26859392	0.227432781	0.838	-1.07	PIS48630.1	orf19.846	Predicted protein kinase similar to S. cerevisiae Nnk1; implicated in proteasome function in S. cerevisiae; induced by Mnl1 under weak acid stress

PIS51770.1	34.50541875	34.22086231	34.66597118	33.37478186	33.51277246	33.25644569	0.005346329	0.0595	-1.08	PIS51770.1	HHF22	Putative histone H4; regulated by Efg1; flucytosine, fluconazole-induced; amphotericin B, caspofungin repressed; colony morphology-related gene regulation by Ssn6; Hap43-induced; rat catheter and Spider biofilm repressed
PIS48486.1	28.08131946	28.28308496	27.84422785	26.26555292	27.33389689	27.37372969	0.130014355	0.729	-1.08	PIS48486.1	orf19.1887	Ortholog(s) have sterol esterase activity, role in sterol metabolic process and lipid droplet, membrane localization
PIS51232.1	27.71352315	27.13786171	27.23481281	27.21061451	25.81318685	25.83279971	0.033502966	0.338	-1.08	PIS51232.1	orf19.2249	Putative metalloprotease of the mitochondrial inner membrane; expression downregulated in an ssr1 null mutant
PIS54688.1	26.94376567	26.66188449	26.22587298	25.88226028	25.42585164	25.27356363	0.089519262	0.629	-1.08	PIS54688.1	orf19.3556	Transportin or cytosolic karyopherin beta; Spider biofilm induced
PIS49519.1	27.34682488	26.12865965	27.7014686	26.41471104	25.76804775	25.76821123	0.047504209	0.429	-1.08	PIS49519.1	orf19.5156	Protein similar to S. cerevisiae Phs1p, which is required for growth; has six putative membrane-spanning regions
PIS55635.1	27.48307568	28.44146666	27.13780947	26.22134908	26.0856381	27.51782347	0.118679178	0.706	-1.08	PIS55635.1	RNA1	Putative GTPase-activating protein; protein level decreases in stationary phase cultures; Spider biofilm repressed
PIS58257.1	21.65458013	22.21001674	20.82243689	20.17310328	20.23823402	20.99945254	0.034634506	0.346	-1.09	PIS58257.1	orf19.2528	Putative RNA polymerase III transcription factor (TFIIIB) subunit; flucytosine repressed
PIS52114.1	30.07549369	29.53156496	29.80680823	28.80349994	28.69656451	28.65348769	0.23832566	0.845	-1.09	PIS52114.1	orf19.4517	Putative translation initiation factor eIF3m; essential gene whose repression impedes translation; heterozygous mutant is sensitive to the translation inhibitor nourseothricin
PIS51376.1	28.67464528	29.3878558	28.69942818	28.52873128	27.66669646	27.30960159	0.01272978	0.155	-1.09	PIS51376.1	VPS35	Putative role in vacuolar sorting; downregulated in biofilm; induced upon adherence to polystyrene
PIS50504.1	34.46594077	34.03660694	34.76260227	33.48795667	33.20903586	33.28108428	0.094444831	0.644	-1.1	PIS50504.1	ADH1	Alcohol dehydrogenase; oxidizes ethanol to acetaldehyde; at yeast cell surface; immunogenic in humans/mice; complements S. cerevisiae adh1 adh2 adh3 mutant; fluconazole, farnesol-induced; flow model biofilm induced; Spider biofilm repressed

PIS54690.1	26.49058626	27.12452884	25.85726174	25.20976609	25.29433927	25.67302299	0.057727014	0.489	-1.1	PIS54690.1	CPA2	Putative arginine-specific carbamoylphosphate synthetase; protein enriched in stationary phase yeast cultures; rat catheter biofilm induced; Spider biofilm induced
PIS59011.1	27.72424692	28.40778637	27.96150969	26.92290839	26.82178706	27.04563896	0.027867112	0.296	-1.1	PIS59011.1	orf19.1815	Ortholog of S. cerevisae/S. pombe Tif6; constituent of 66S pre-ribosomal particles; Spider biofilm induced
PIS56890.1	26.53091207	26.11617236	27.18064035	26.27752332	25.23776076	24.99834335	0.037264869	0.362	-1.1	PIS56890.1	orf19.5552	Putative transcriptional regulator of ribonucleotide reductase genes; Spider biofilm induced
PIS56545.1	32.21320867	31.69578565	32.51433094	30.86864688	31.17921051	31.08249787	0.076660427	0.581	-1.1	PIS56545.1	RPL23A	Ribosomal protein; downregulated upon phagocytosis by murine macrophage; Hap43-induced; sumoylation target; Spider biofilm repressed
PIS51875.1	26.05114822	26.30696362	26.46804464	24.82718994	24.73283609	25.92953439	0.335633831	0.889	-1.11	PIS51875.1	DAL81	Zn(II)2Cys6 transcription factor; ortholog of S. cerevisiae Dal81, involved in the regulation of nitrogen-degradation genes; required for yeast cell adherence to silicone substrate; Spider biofilm induced
PIS48738.1	27.48971964	28.67521142	26.14272657	27.52881718	25.20765261	26.238413	0.134060714	0.736	-1.11	PIS48738.1	orf19.2150	Putative ortholog of mammalian electron transfer flavoprotein complex subunit ETF-alpha; Spider biofilm repressed
PIS58621.1	26.97987396	26.77747552	25.10508299	25.38506416	24.47734739	25.67127731	0.07194386	0.561	-1.11	PIS58621.1	orf19.2228	Ortholog(s) have actin filament binding, actin monomer binding activity
PIS54571.1	24.94086609	25.75796109	25.49649256	24.29828268	24.74457304	23.83525424	0.011604294	0.141	-1.11	PIS54571.1	orf19.325	Putative mRNA cleavage and polyadenlylation factor; heterozygous null mutant exhibits hypersensitivity to parnafungin and cordycepin in the C. albicans fitness test
PIS56770.1	29.11066999	30.86550662	28.59218994	28.37529491	27.9227698	28.90441709	0.09041004	0.632	-1.12	PIS56770.1	COF1	Putative cofilin; macrophage-induced protein; protein present in exponential and stationary-phase yeast cells, but higher amounts in stationary phase
PIS54982.1	24.24082967	27.23923267	24.66713094	23.37363525	24.12905132	25.28399779	0.139752785	0.746	-1.12	PIS54982.1	orf19.4273	Putative mitochondrial membrane protein; ortholog of S. cerevisiae Sls1; coordinates expression of mitochondrially-encoded genes; Hap43-induced

PIS48403.1	27.4930495	27.03905251	26.47593548	26.46195322	25.98835648	25.2095369	0.399547997	0.906	-1.12	PIS48403.1	orf19.6551	Ortholog(s) have SNAP receptor activity, role in Golgi vesicle transport, vesicle fusion and Golgi medial cisterna, SNARE complex localization
PIS52359.1	29.20138678	30.00189246	29.32422723	28.60468954	28.23562012	28.29672042	0.233835516	0.842	-1.13	PIS52359.1	orf19.4016	Putative ubiquinol-cytochrome-c reductase; amphotericin B induced; repressed by nitric oxide, Hap43p, and Spider biofilm; null mutant is viable but shows decreased vegetative growth on several carbon sources
PIS51636.1	25.60524055	23.51391095	25.47816364	24.79240178	23.80868309	22.60434165	0.238565812	0.845	-1.13	PIS51636.1	orf19.6054	Protein with a Bul1 domain; binds the ubiquitin ligase Rsp5 and is involved in intracellular trafficking of a general amino acid permease Gap1; repressed in an azole-resistant strain overexpressing MDR1; Spider biofilm induced
PIS56832.1	27.07648059	25.80002096	26.53596883	24.70751971	25.4450892	25.85497801	0.015450042	0.184	-1.13	PIS56832.1	RPF1	Putative nucleolar protein with a predicted role in the assembly and export of the large ribosomal subunit; essential for growth; rat catheter and Spider biofilm induced
PIS55662.1	25.09102336	24.0773997	27.67228774	23.12513476	27.62136928	22.67436888	0.399582883	0.906	-1.14	PIS55662.1	orf19.2563	Ortholog(s) have U6 snRNA binding, snRNA binding activity, role in spliceosomal complex assembly, spliceosomal tri-snRNP complex assembly and U6 snRNP localization
PIS51348.1	29.01177185	28.59404679	29.75409988	27.18755744	28.45823588	28.2917269	0.078375486	0.588	-1.14	PIS51348.1	orf19.4144	Predicted protein kinase; clade-associated gene expression
PIS58070.1	22.20140845	25.01251314	24.09123275	23.67442238	23.82103862	20.37811141	0.416069163	0.91	-1.14	PIS58070.1	orf19.5534	Protein with a predicted role in mitotic spindle elongation, vesicle-mediated transport; flow model biofilm induced
PIS51916.1	27.49277278	25.45163392	27.54950816	26.39137163	25.45336347	25.21881101	0.152796115	0.765	-1.14	PIS51916.1	SPC2	Signal peptidase complex component; role in ER protein translocation; transcript is induced upon filamentous growth
PIS59002.1	26.11391513	27.49085459	25.79422358	25.65432295	24.73902159	25.553868	0.083545168	0.608	-1.15	PIS59002.1	ARO2	Putative chorismate synthase; fungal-specific (no human or murine homolog); protein level decreased in stationary phase yeast cultures; GlcNAc-induced protein
PIS48452.1	34.19569171	33.85664793	34.08240802	32.67184422	32.95576693	33.06619075	0.110849244	0.689	-1.15	PIS48452.1	orf19.1052	Predicted histone H2B; Hap43-induced gene; Spider biofilm repressed

PIS58733.1	25.97684381	26.58717262	26.31817992	25.87363707	24.03524404	25.4964607	0.2482409	0.851	-1.16	PIS58733.1	orf19.1697	Ortholog(s) have role in cytoplasmic translation, poly(A)+ mRNA export from nucleus and cytoplasm localization
PIS51223.1	30.32921238	30.49479846	30.23930169	29.26604421	29.60255451	28.72292997	0.166481943	0.783	-1.16	PIS51223.1	orf19.2265	Component of the RSC chromatin remodeling complex
PIS52416.1	25.43968459	24.50218273	26.37602327	24.47242272	24.14380463	24.21428369	0.127368254	0.724	-1.16	PIS52416.1	orf19.4894	Protein with similarity to S. cerevisiae Yer010cp, a protein of unknown function belonging to the prokaryotic RraA family; repressed by benomyl; Hap43-induced; Spider biofilm induced
PIS58156.1	33.8360649	34.56679872	33.89605477	33.28031405	32.6622527	32.88790546	0.080748187	0.598	-1.16	PIS58156.1	TSA1	TSA/alkyl hydroperoxide peroxidase C (AhPC) family protein; similar to thiol-dependent peroxidases of oxidative stress signaling; antigenic; hyphal surface, nucleus; yeast-form nucleus, cytoplasm; biofilm, phagocytosis, peroxide induced
PIS51556.1	25.8394866	25.39481983	25.923293	26.25612418	23.22139654	24.15609598	0.105295326	0.675	-1.17	PIS51556.1	orf19.2786	Ortholog(s) have AP-2 adaptor complex, cellular bud neck localization
PIS51945.1	25.75946086	26.54801368	26.38092098	24.07953543	25.15500088	25.95387002	0.043244566	0.401	-1.17	PIS51945.1	orf19.2889	Ortholog(s) have role in CENP-A containing chromatin assembly, chromatin remodeling and Ino80 complex, chromatin localization
PIS48531.1	31.40450888	31.1285443	31.84257974	30.41298923	30.01743563	30.43322473	0.022893079	0.258	-1.17	PIS48531.1	SHM2	Cytoplasmic serine hydroxymethyltransferase; complements glycine auxotrophy of S. cerevisiae shm1 shm2 gly1-1 mutant; antigenic; farnesol-upregulated in biofilm; stationary-phase enriched protein; rat catheter and Spider biofilm repressed
PIS58032.1	26.582587	24.85875958	25.14091103	23.40580354	24.78010608	24.86974645	0.209353686	0.825	-1.18	PIS58032.1	orf19.1204	Phosphorylated protein of unknown function; transcript is upregulated clinical isolates from HIV positive patients with oral candidiasis
PIS48144.1	28.70391687	28.632368	28.86091387	27.58308173	27.25227058	27.83018168	0.016579626	0.195	-1.18	PIS48144.1	orf19.1890	Ortholog(s) have acylglycerol lipase activity, triglyceride lipase activity and role in medium-chain fatty acid biosynthetic process, triglyceride metabolic process
PIS51246.1	28.2433896	28.1835837	28.86382248	27.59028965	27.30780629	26.85357179	0.006044998	0.0695	-1.18	PIS51246.1	PEX12	Ortholog(s) have ubiquitin ligase activator activity, ubiquitin protein ligase activity and role in proteasome-mediated ubiquitin-dependent protein catabolic process, protein import into peroxisome matrix, protein polyubiquitination

PIS51603.1	28.47556954	27.02933601	29.22229258	27.17748574	27.03897618	26.98319062	0.029883441	0.311	-1.18	PIS51603.1	RIM20	Protein involved in the pH response pathway; binds to the transcription factor Rim101 and may serve as a scaffold to facilitate the C-terminal proteolytic cleavage that activates Rim101; required for alkaline pH-induced hyphal growth
PIS58805.1	25.93730246	26.97417719	27.03491347	25.44139197	25.72469099	25.20291963	0.086432638	0.618	-1.19	PIS58805.1	CTR2	Putative low-affinity copper transporter of the vacuolar membrane; induced by nitric oxide; clade-associated gene expression; rat catheter and flow model biofilm induced
PIS56598.1	28.40302894	30.07632435	26.84161915	27.45930147	27.02319983	27.2765453	0.165364271	0.782	-1.19	PIS56598.1	orf19.2286	Putative deoxyhypusine hydroxylase; ketoconazole-induced; protein level decreases in stationary phase cultures; required for biofilm formation; Spider biofilm repressed
PIS55617.1	26.74462188	26.20458729	27.21417742	25.5402839	25.66982808	25.37708736	0.298280487	0.875	-1.19	PIS55617.1	orf19.5250	Ortholog of C. dubliniensis CD36 : Cd36_11370, C. parapsilosis CDC317 : CPAR2_207480, C. auris B8441 : B9J08_001721 and Candida tenuis NRRL Y-1498 : CANTEDRAFT_120384
PIS58396.1	26.37793888	26.37290837	25.90939093	25.79158542	26.70728355	22.57815315	0.219178437	0.832	-1.19	PIS58396.1	orf19.7107	Ortholog(s) have role in ribosomal large subunit biogenesis and cytoplasm, nucleus localization
PIS51234.1	26.33013601	24.83144906	27.37381965	25.77360022	24.10504039	25.04900358	0.20101638	0.818	-1.2	PIS51234.1	BUD23	Putative methyltransferase; Hap43-induced; repressed by prostaglandins
PIS55840.1	28.58420133	28.90024853	26.75901818	26.15757479	26.11285068	28.36049499	0.220061921	0.833	-1.2	PIS55840.1	orf19.5026	C2H2 transcription factor; Spider biofilm induced; dominant-negative mutants showing hyper-invasive growth identified in clinical isolates
PIS50507.1	23.93808523	24.95685685	21.74550051	23.79881326	21.58715255	21.63864896	0.485668365	0.922	-1.21	PIS50507.1	GRF10	Putative homeodomain transcription factor, involved in copper homeostasis and control of filamentous growth; null mutant is an adenine auxotroph and shows increased copper resistance; promoter bound by Bcr1, Tec1, Efg1, Ndt80 and Brg1
PIS54513.1	26.31959146	24.54882621	26.84415771	25.73876888	23.93228525	24.37571124	0.150233893	0.762	-1.22	PIS54513.1	RIA1	Putative translation elongation factor; genes encoding ribosomal subunits, translation factors, and tRNA synthetases are downregulated upon phagocytosis by murine macrophage
PIS58022.1	28.51190921	28.2664563	26.34254401	27.17463532	26.12498845	26.14139878	0.134456178	0.737	-1.23	PIS58022.1	orf19.764	Ortholog(s) have role in negative regulation of TORC1 signaling and cytoplasm localization

PIS56737.1	28.71933118	29.2448555	29.46583197	28.41723118	27.57983044	27.71319961	0.01429174	0.172	-1.24	PIS56737.1	LEU4	Putative 2-isopropylmalate synthase; involved in resistance to caspofungin and anidulafungin; regulated by NRG1, MIG1, TUP1, GCN4; induced by human whole blood or PMNs; macrophage/pseudohyphal-repressed after 16h;
PIS51212.1	26.65656696	27.28803624	26.11003391	26.55253192	23.70007885	26.00924158	0.293583341	0.873	-1.26	PIS51212.1	orf19.2275	Putative mitochondrial ribosomal protein; predicted role in aerobic respiration; Spider biofilm repressed
PIS50408.1	25.79336512	27.89011018	27.1128001	24.75137065	25.96281896	26.3088019	0.075164557	0.575	-1.26	PIS50408.1	YPD1	Phosphohistidine intermediate protein in a phosphorelay signal transduction pathway; residue His69 is the phosphoacceptor histidine; predicted to be soluble and cytosolic; functional homolog of S. cerevisiae Ypd1p
PIS58059.1	26.47510399	27.68452137	25.57703071	24.54422988	25.13224022	26.26180456	0.128323509	0.726	-1.27	PIS58059.1	HCR1	Putative translation initiation factor; repressed upon phagocytosis by murine macrophage; Spider biofilm repressed
PIS50631.1	26.84502234	26.79026922	26.19974952	24.10301183	25.74332321	26.19056026	0.114812518	0.698	-1.27	PIS50631.1	ILV6	Putative regulatory subunit of acetolacetate synthase; alkaline induced; regulated by Gcn2 and Gcn4; protein present in exponential and stationary growth phase yeast; Spider biofilm repressed
PIS59030.1	26.72438862	27.77655784	27.89799782	25.29830285	25.35848033	27.94307991	0.09083209	0.633	-1.27	PIS59030.1	orf19.3661	Putative deubiquitinating enzyme; induced by Mnl1 under weak acid stress
PIS56858.1	27.49373951	25.97806053	28.96871984	26.03119563	26.11856199	26.48954695	0.150189027	0.762	-1.27	PIS56858.1	orf19.4575	Ortholog(s) have role in mitochondrion organization, phospholipid homeostasis and mitochondrial inner membrane, mitochondrial inner-outer membrane contact site localization
PIS58584.1	29.45533593	28.99547342	29.27947156	27.83049435	28.20680793	27.87402302	0.322203631	0.884	-1.27	PIS58584.1	orf19.7502	Protein of unknown function; Hap43-induced gene; upregulated in a cyr1 null mutant; Spider biofilm induced
PIS49711.1	26.60956522	26.4071632	26.02196897	25.78036944	25.50344028	23.90727966	0.028685949	0.301	-1.28	PIS49711.1	orf19.6559	RNA polymerase III transcription initiation factor complex (TFIIIC) subunit; growth phase regulated protein; downregulaated in stationary phase yeast cultures; Hap43-repressed; flow model biofilm induced; Spider biofilm repressed
PIS51562.1	26.1246675	26.18554836	26.41212949	25.56439883	25.18664077	24.09053653	0.151939385	0.764	-1.29	PIS51562.1	orf19.2794	Putative non-specific single-domain racemase; regulated by Gcn4p; repressed in response to amino acid starvation (3-AT treatment); alkaline upregulated; macrophage-induced protein

PIS58246.1	25.61557016	24.38657274	26.09783027	24.59767915	24.28402078	23.31035729	0.060342761	0.504	-1.3	PIS58246.1	ECM39	Putative mannosyltransferase similar to S. cerevisiae Ecm39p, which has a role in Calcofluor white resistance; predicted Kex2p substrate; has HKEXRF motif
PIS48428.1	29.72624319	29.82868172	29.38534544	28.25178659	28.40478184	28.38442934	0.009104519	0.112	-1.3	PIS48428.1	HMO1	HMG-box transcription factor; binds upstream of hexose and ergosterol metabolism and cell cycle genes; acts as repressor of START; activates pseudohyphal growth when expressed in S. cerevisiae; repressed in hyphae
PIS58663.1	31.44589803	26.60931203	29.17691494	28.64512443	27.56927472	27.10530651	0.282911953	0.869	-1.3	PIS58663.1	SKI2	Ortholog(s) have role in endoplasmic reticulum unfolded protein response, nuclear-transcribed mRNA catabolic process and 3'-5' exonucleolytic nonsense-mediated decay, more
PIS52003.1	34.23689496	34.00282903	34.42874422	33.0088423	32.67556499	33.04221552	0.024067017	0.268	-1.31	PIS52003.1	ADH2	Alcohol dehydrogenase; soluble in hyphae; expression regulated by white-opaque switching; regulated by Ssn6; indued by Mnl1 in weak acid stress; protein enriched in stationary phase yeast cultures; Spider biofilm induced
PIS54673.1	22.92779377	23.32745274	23.09300173	22.40416589	22.23166375	20.78767046	0.228875305	0.839	-1.31	PIS54673.1	orf19.834	Ortholog(s) have carbohydrate binding, mannosyl-oligosaccharide 1,2-alpha-mannosidase activity
PIS58599.1	26.21875947	29.31759818	26.13763683	26.10128303	24.95369745	26.66863626	0.306069115	0.878	-1.32	PIS58599.1	ISW2	Ortholog of S. cerevisiae Isw2; an ATPase involved in chromatin remodeling; required for chlamydospore formation; Hap43-induced gene; repressed by high-level peroxide stress
PIS48588.1	26.85051874	26.88042459	26.75720048	26.98114821	25.06638593	24.48844336	0.081102634	0.599	-1.32	PIS48588.1	orf19.2167	Ortholog(s) have role in ribosomal large subunit biogenesis, ribosomal small subunit biogenesis and nucleolus localization
PIS49588.1	27.05335759	27.92496411	27.77216215	25.99101979	26.14765436	26.61709638	0.077791597	0.586	-1.33	PIS49588.1	EDC3	Protein with mRNA binding activity, involved in regulation of activity of putative metacaspase Mca1p
PIS48732.1	26.70430002	26.64453459	26.91112412	25.54303288	25.78978919	24.93901753	0.03812362	0.368	-1.33	PIS48732.1	orf19.2778	Protein of unknown function; transcript is upregulated in clinical isolates from HIV+ patients with oral candidiasis
PIS58666.1	30.08594304	30.25941524	29.80416028	27.89712351	28.97384503	29.25828735	0.29080934	0.872	-1.34	PIS58666.1	PEX19	Ortholog(s) have peroxisome membrane targeting sequence binding activity

PIS48735.1	23.92047115	24.25992606	24.17653831	22.89688793	22.85179957	22.59373007	0.167740504	0.784	-1.34	PIS48735.1	RBT4	Pry family protein; required for virulence in mouse systemic/rabbit corneal infections; not filamentation; mRNA binds She3, is localized to hyphal tips; Hap43-induced; in both yeast and hyphal culture supernatants; Spider biofilm induced
PIS49787.1	23.69529599	28.10324853	28.88834794	25.85240302	25.67519232	25.10839235	0.325845918	0.885	-1.35	PIS49787.1	orf19.3177	Ortholog(s) have 2,5-diamino-6-ribitylamino-4(3H)-pyrimidinone 5'-phosphate deaminase activity, pseudouridine synthase activity and role in riboflavin biosynthetic process, tRNA pseudouridine synthesis
PIS51932.1	32.20129291	32.37453612	31.69096547	31.02094863	30.6473064	30.55568894	0.25891102	0.857	-1.35	PIS51932.1	orf19.371	Ortholog of C. dubliniensis CD36 : Cd36_40110, C. parapsilosis CDC317 : CPAR2_402300, C. auris B8441 : B9J08_003539 and Candida tenuis NRRL Y-1498 : CANTEDRAFT_94507
PIS50434.1	27.42507414	27.76466801	27.4799592	25.25369269	26.38233002	26.96896951	0.10423894	0.672	-1.35	PIS50434.1	orf19.6627	Protein of unknown function; possibly transcriptionally regulated upon hyphal formation
PIS52014.1	26.75296211	28.43273922	25.89312406	23.71449076	26.42155918	26.86580939	0.090597447	0.632	-1.36	PIS52014.1	ACP12	Putative mitochondrial acyl carrier protein
PIS58674.1	26.48904396	27.13349845	26.95229337	23.20427455	26.73381909	26.57118901	0.120753404	0.711	-1.36	PIS58674.1	TSR1	Component of 20S pre-rRNA processing unit; repressed by prostaglandins
PIS52220.1	26.5773482	26.46108936	26.59389527	24.98537543	23.08917858	27.44982881	0.149405122	0.76	-1.37	PIS52220.1	MEF2	Putative mitochondrial translation elongation factor; caspofungin induced
PIS58184.1	29.73559332	29.37845154	29.60789519	28.30512104	28.06526882	28.22214247	0.00464074	0.0493	-1.38	PIS58184.1	orf19.3219	Ortholog of S. cerevisiae Sia1; involved in activation of the Pma1 plasma membrane H+-ATPase by glucose in S. cerevisiae; Spider biofilm induced
PIS51862.1	27.41575405	29.21283327	28.21091541	26.89738839	26.2256563	27.58161926	0.142315493	0.75	-1.38	PIS51862.1	orf19.376	Protein of unknown function; Hap43-repressed; Spider biofilm induced
PIS58528.1	25.51050512	24.4024562	25.35066453	22.76605883	24.51556928	23.85276412	0.039262884	0.374	-1.38	PIS58528.1	orf19.6456	Ortholog of C. dubliniensis CD36 : Cd36_72280, C. parapsilosis CDC317 : CPAR2_703040, C. auris B8441 : B9J08_001028 and Candida tenuis NRRL Y-1498 : CANTEDRAFT_125842

PIS51284.1	26.45035972	22.4319258	25.72403496	22.45057767	23.83313298	24.19050519	0.191321897	0.809	-1.38	PIS51284.1	SWD1	Ortholog(s) have histone H3K4 methyltransferase activity, role in regulation of DNA-templated transcription, subtelomeric heterochromatin formation, telomere maintenance and Set1C/COMPASS complex localization
PIS49613.1	30.74350663	30.54417204	30.94887754	29.50547732	28.98279821	29.58129012	0.002707249	0.0243	-1.39	PIS49613.1	orf19.4970	Protein of unknown function; Spider biofilm induced
PIS56809.1	27.11471078	25.45155968	26.72437871	25.05847183	25.51953426	24.54892529	0.039169961	0.374	-1.39	PIS56809.1	TBP1	Transcription initiation factor; binds TATA box sequence, binding does not require TFIIA; caspofungin repressed; functional homolog of S. cerevisiae and human TATA-binding proteins; Spider biofilm induced
PIS54907.1	30.58985805	31.01311072	30.22765414	29.26141603	29.17389175	29.18057617	0.000676289	0.00231	-1.4	PIS54907.1	orf19.4131	Ortholog(s) have EMC complex localization
PIS51564.1	24.63869208	23.19515633	25.01761018	23.45943184	22.53855113	22.61605278	0.051037443	0.45	-1.41	PIS51564.1	orf19.2796	Ortholog(s) have DNA-directed DNA polymerase activity, role in DNA replication initiation, mitotic DNA replication initiation, telomere capping and alpha DNA polymerase:primase complex, nuclear envelope, nucleus localization
PIS50577.1	25.97927358	26.09950342	25.55401579	25.15405081	24.98969844	23.25406557	0.086173355	0.617	-1.41	PIS50577.1	orf19.4253	Ortholog(s) have role in negative regulation of gluconeogenesis, proteasome-mediated ubiquitin-dependent protein catabolic process, regulation of nitrogen utilization and GID complex localization
PIS54810.1	25.95557022	26.75252578	24.59553069	24.71673275	23.90059807	24.44453995	0.010665248	0.13	-1.41	PIS54810.1	orf19.4711	Ortholog of C. dubliniensis CD36 : Cd36_40890, C. parapsilosis CDC317 : CPAR2_401100, C. auris B8441 : B9J08_001954 and Candida tenuis NRRL Y-1498 : CANTEDRAFT_119698
PIS56581.1	28.07278286	27.97626437	27.15534543	27.32088653	26.41878099	25.17978328	0.106006849	0.677	-1.43	PIS56581.1	CIC1	Putative proteasome-interacting protein; rat catheter biofilm induced
PIS58523.1	20.71371082	21.68740809	21.82877507	22.33950625	19.70498992	17.90434497	0.280879377	0.868	-1.43	PIS58523.1	orf19.3275	Ortholog of C. dubliniensis CD36 : Cd36_25870, C. parapsilosis CDC317 : CPAR2_804000, C. auris B8441 : B9J08_001023 and Candida tenuis NRRL Y-1498 : CANTEDRAFT_115661
PIS51020.1	27.1362244	25.39861789	24.16420933	24.09938496	23.64812686	24.62620698	0.039670137	0.377	-1.44	PIS51020.1	orf19.6075	Putative CCR4-Not complex transcription factor; ortholog of S. cerevisiae Cdc36; Hap43-repressed gene

PIS52255.1	26.36776213	25.05946167	26.04359647	25.18270094	24.25550459	23.68545149	0.018264003	0.214	-1.45	PIS52255.1	CCS1	Copper chaperone involved in activation and protection of superoxide dismutase Sod1p
PIS55572.1	26.76138548	23.31153357	25.5209079	23.8862726	24.3777696	22.98550913	0.09192977	0.637	-1.45	PIS55572.1	orf19.1191	Ortholog(s) have ubiquitin-protein transferase activity and role in negative regulation of protein autoubiquitination, retrograde protein transport, ER to cytosol, ubiquitin-dependent ERAD pathway
PIS56572.1	28.20068339	28.02733439	28.49064559	26.76423926	26.84770462	26.74770488	0.032963604	0.334	-1.45	PIS56572.1	orf19.6247	Ortholog(s) have chromatin binding activity
PIS56877.1	29.16904687	28.37284277	30.34726902	28.82251279	27.235701	27.44092132	0.205255857	0.821	-1.46	PIS56877.1	HOS3	Histone deacetylase; similar to S. cerevisiae Hos3p; greater expression and longer mRNA in white cells, compared to opaque cells; has conserved deacetylation motif
PIS58126.1	31.17485307	32.04756043	30.57346161	28.35546987	30.08237753	30.90271462	0.057459003	0.487	-1.49	PIS58126.1	ECM33	GPI-anchored cell wall protein; mutants show cell-wall defects and reduced adhesion, host cell damage, and endocytosis; mutant infection is immunoprotective in murine model; fluconazole-induced; caspofungin repressed
PIS51079.1	26.49843484	26.04202322	26.88996872	26.47158321	24.50533332	23.97705273	0.030997184	0.32	-1.49	PIS51079.1	orf19.2381	Protein of unknown function; possibly an essential gene, disruptants not obtained by UAU1 method
PIS56554.1	31.13660974	31.63509247	31.05711945	29.22488777	30.03571781	30.10475521	0.028820243	0.302	-1.49	PIS56554.1	TRR1	Thioredoxin reductase; regulated by Tsa1/Tsa1B, Hap43; induced by nitric oxide, peroxide; oxidative stress-induce via Cap1; induced by human neutrophils; stationary phase enriched protein
PIS52258.1	26.80053098	27.6708817	26.25192157	25.26251834	25.02025855	25.92832392	0.019616356	0.228	-1.5	PIS52258.1	ENP1	Protein required for pre-rRNA processing and 40S ribosomal subunit synthesis; associated with U3 and U14 snoRNAs; transposon mutation affects filamentous growth; repressed by prostaglandins; Spider biofilm induced
PIS58905.1	25.62412731	27.15550239	26.39421901	27.34164051	21.96728832	25.29230693	0.330812716	0.887	-1.52	PIS58905.1	orf19.1723	Ortholog(s) have role in response to purine-containing compound
PIS55547.1	27.33696459	25.68419687	26.76570341	27.01809858	23.26824664	24.91607032	0.074164258	0.571	-1.53	PIS55547.1	HIR1	Component of the HIR complex, a nucleosome assembly factor involved in chromatin formation; involved in regulation of white-opaque switching frequency and regulation of nitrogen utilization

PIS49837.1	29.19654475	29.22793362	28.80867888	27.92274738	27.26543114	27.44723082	0.054921757	0.472	-1.53	PIS49837.1	orf19.5574	Has domain(s) with predicted role in cysteine biosynthetic process from serine
PIS58240.1	26.00720643	27.76567905	26.1499607	24.5559993	24.4899222	26.24056739	0.039372616	0.375	-1.55	PIS58240.1	GPI13	Major facilitator superfamily protein; has phosphodiesterase/nucleotide pyrophosphatase domain; similar to S. cerevisiae Gpi13p, which acts in GPI anchor biosynthesis; Hap43p-induced gene
PIS51745.1	26.47815389	26.09783922	26.78346214	25.43914187	24.25280758	25.01633531	0.085489105	0.615	-1.55	PIS51745.1	orf19.2047	Putative protein of unknown function; Hap43p-repressed gene; mutation confers hypersensitivity to toxic ergosterol analog, and to amphotericin B
PIS55474.1	24.4483752	26.23719556	22.84489909	22.49512685	23.46120519	22.87098862	0.252807054	0.854	-1.57	PIS55474.1	GAR1	Putative H/ACA snoRNP pseudouridylase complex protein; mutation confers hypersensitivity to tubercidin (7-deazaadenosine); macrophage/pseudohyphal-induced; Spider biofilm repressed
PIS58100.1	28.97683655	28.81342266	25.90188223	23.31411811	27.56416484	28.11632337	0.229115696	0.839	-1.57	PIS58100.1	UBA2	Ortholog(s) have SUMO activating enzyme activity, role in protein sumoylation and SUMO activating enzyme complex, nucleus localization
PIS51078.1	26.60471058	26.2172312	26.24187064	23.13506543	25.44926647	25.74919317	0.051854412	0.454	-1.58	PIS51078.1	NOT4	Putative E3 ubiquitin-protein ligase; required for maintenance, but not induction, of hyphal development; homozygous null mutant is avirulent in mouse systemic infection despite persistence in host; repressed in rat oral candidiasis
PIS58565.1	28.03225228	27.40545708	28.25411617	26.39478444	26.47191934	26.02607958	0.001576733	0.0114	-1.6	PIS58565.1	orf19.4614	Adapter protein for pexophagy and the cytoplasm-to-vacuole targeting (Cvt) pathway; Spider biofilm induced
PIS58646.1	24.09223212	24.4478676	25.5579333	24.30323796	21.07858319	23.88802299	0.262212287	0.859	-1.61	PIS58646.1	DYN1	Dynein heavy chain; motor protein that moves to microtubule minus end; required for yeast cell separation, spindle positioning, nuclear migration, hyphal growth; regulated by Mig1, Hap43; flow model and rat catheter biofilm repressed
PIS51122.1	28.10268539	27.68779908	28.18235307	26.78141471	25.88684453	26.46419775	0.001142201	0.00639	-1.61	PIS51122.1	orf19.7330	Protein with a predicted heme oxygenase domain; Spider biofilm induced
PIS51361.1	26.32157119	26.83864752	25.83531814	23.5613266	25.32974098	25.24718534	0.043944612	0.406	-1.62	PIS51361.1	orf19.4347	Putative serine/threonine protein kinase; Hog1p-induced

PIS52393.1	26.74005234	27.1842091	26.24578535	23.93038413	25.15615657	26.21238859	0.021558632	0.246	-1.62	PIS52393.1	orf19.5425	Ortholog(s) have 3'-tRNA processing endoribonuclease activity, role in mitochondrial tRNA 3'-end processing, tRNA 3'-end processing and mitochondrion, nucleus localization
PIS58672.1	27.96534134	28.03353219	27.77754855	26.10284637	26.0086261	26.81017293	0.000713446	0.00251	-1.62	PIS58672.1	orf19.6418	Ortholog(s) have unfolded protein binding activity and role in protein import into nucleus, ribosomal large subunit biogenesis
PIS50363.1	27.92999038	27.38318343	26.11369164	25.70397459	24.87234964	25.96917847	0.008712578	0.107	-1.63	PIS50363.1	orf19.3047	Protein kinase-related protein, required for normal sensitivity to caspofungin
PIS50586.1	26.86506431	27.50640094	25.73632124	24.87362003	24.81499534	25.52821629	0.008914747	0.11	-1.63	PIS50586.1	orf19.4168	Ortholog(s) have RNA binding, ribonuclease MRP activity, ribonuclease P activity and role in intronic box C/D RNA processing, nuclear-transcribed mRNA catabolic process, RNase MRP-dependent, rRNA processing, tRNA processing
PIS51940.1	26.89107907	26.23743613	27.24425749	23.88376409	25.77748485	25.80331776	0.16880288	0.786	-1.64	PIS51940.1	BUD31	Bud31 ortholog; not subject to mating-type regulation, in contrast to S. cerevisiae Bud31 which has a role in specifying the bud site; Spider biofilm induced
PIS55012.1	25.83335974	25.10194266	25.98958652	23.98621221	24.64702741	23.38050562	0.035120907	0.349	-1.64	PIS55012.1	orf19.1177	Ortholog of S. cerevisiae Rtt106; histone chaperone that regulates chromatin structure in transcribed and silenced chromosomal regions; affects transcriptional elongation; Hap43-repressed; Spider biofilm repressed
PIS52110.1	26.52961244	26.70680999	26.25097687	24.87424473	24.9576693	24.72850063	0.007192171	0.0868	-1.64	PIS52110.1	orf19.4503	Similar to HMG-box variant of S. pombe; Spider biofilm repressed
PIS48741.1	29.82765327	30.11097389	29.32733284	27.81161484	27.88935811	28.63311401	0.014668953	0.176	-1.64	PIS48741.1	orf19.7269	Putative polyamine acetyltransferase; acetylates polyamines (e.g. putrescine, spermidine, spermine) and aralkylamines (e.g. tryptamine, phenylethylamine); Spider biofilm repressed
PIS54575.1	27.88065856	27.57121803	27.1001941	25.92470571	26.05998143	25.63115754	0.000870562	0.00375	-1.65	PIS54575.1	MET3	ATP sulfurlyase; sulfate assimilation; repressed by Met, Cys, Sfu1, or in fluconazole-resistant isolate; Hog1, caspofungin, white phase-induced; induced on biofilm formation, even in presence of Met and Cys; Spider, F-12/CO2 biofilm induced
PIS58740.1	25.49482294	23.12573212	26.09261299	25.22942784	22.06326798	22.39760743	0.087757477	0.623	-1.67	PIS58740.1	ABC1	Putative ubiquinol-cytochrome-c reductase; induced upon adherence to polystyrene; flow model biofilm induced; Spider biofilm induced

PIS51553.1	27.78140505	26.12387265	22.80542277	23.68425974	23.12803224	24.87397288	0.101976531	0.666	-1.67	PIS51553.1	orf19.7149	Putative GTPase inhibitor; predicted role in endocytosis, protein targeting to vacuole; rat catheter biofilm repressed
PIS52158.1	30.90648022	30.70863984	30.80436498	29.2830203	29.00987707	29.07339083	0.002440956	0.0211	-1.68	PIS52158.1	CCT6	Putative cytosolic chaperonin Cct ring complex subunit; mutation confers hypersensitivity to cytochalasin D; GlcNAc-induced protein; Spider biofilm repressed
PIS52174.1	27.74922031	29.23373033	28.10731713	26.42883937	26.01433252	27.60391594	0.016160525	0.191	-1.68	PIS52174.1	PUP3	Putative beta 3 subunit of the 20S proteasome; macrophage/pseudohyphal-repressed
PIS55531.1	27.45487727	28.52113188	27.10335394	27.24429891	24.78236515	25.97879804	0.073418848	0.568	-1.69	PIS55531.1	orf19.5525	Putative oxidoreductase; protein levels affected by URA3 expression in CAI-4 strain background; Efg1, Efh1 regulated; Rgt1-repressed; protein present in exponential and stationary growth phase yeast; rat catheter biofilm repressed
PIS58079.1	26.3039249	25.8831606	26.55875401	25.10002681	25.02600134	23.48886983	0.020114449	0.233	-1.71	PIS58079.1	KSR1	3-ketosphinganine reductase, catalyzes the second step in phytosphingosine synthesis
PIS50632.1	24.74088271	25.85019024	24.78906689	23.28596218	23.45126643	23.51991994	0.002233113	0.019	-1.71	PIS50632.1	PIN3	Putative SH3-domain-containing protein; predicted role in actin cytoskeleton organization; Spider biofilm repressed
PIS58075.1	26.79726851	26.5994006	27.35453162	23.19922818	26.37615865	25.97740537	0.048444573	0.434	-1.73	PIS58075.1	WAL1	Protein required for hyphal growth and for wild-type cell morphology, polarized budding, endocytosis, vacuole morphology; similar to Wiskott-Aldrich syndrome protein; localizes to cortical actin patches and hyphal tips
PIS58062.1	24.52485768	24.23501353	25.23271642	21.07799264	23.59724214	24.0559677	0.097210652	0.653	-1.75	PIS58062.1	orf19.3455	Putative mitochondrial inner membrane magnesium transporter; possibly an essential gene, disruptants not obtained by UAU1 method
PIS51242.1	26.06998684	23.58308966	26.65178633	24.03918416	23.02449501	23.96446721	0.113279385	0.694	-1.76	PIS51242.1	ERG28	Ortholog(s) have protein-macromolecule adaptor activity, role in ergosterol biosynthetic process and endoplasmic reticulum membrane localization
PIS50531.1	25.49640568	25.92830164	26.5080015	25.75697609	23.68859427	23.15658597	0.025968412	0.283	-1.78	PIS50531.1	orf19.3922	Possible pyrimidine 5' nucleotidase; protein present in exponential and stationary growth phase yeast cultures; Hap43p-repressed gene

PIS48483.1	26.90849631	27.16914867	26.86941804	25.38965091	25.82008002	24.37211642	0.020797125	0.239	-1.79	PIS48483.1	orf19.2201	Ortholog(s) have ribosome binding activity, role in mitochondrial respiratory chain complex III assembly, positive regulation of mitochondrial translation and Cbp3p-Cbp6 complex, mitochondrial ribosome localization
PIS56562.1	25.28918695	25.85964602	24.81160192	25.66318665	22.48138745	22.4486083	0.18965393	0.808	-1.79	PIS56562.1	SEC13	Putative protein transport factor; antigenic during murine systemic infection; macrophage-downregulated protein; protein level decreases in stationary phase cultures; Spider biofilm repressed
PIS50344.1	23.08040015	25.26898819	25.23762447	22.83804912	22.61422026	22.72335624	0.260345281	0.858	-1.8	PIS50344.1	orf19.6554	Regulator of calcineurin; regulated by calcineurin-Crz1 pathway; feedback regulator of calcineurin-dependent signaling; Hap43-repressed; induced by ketoconazole, hypoxia, during growth in the mouse cecum; Spider biofilm induced
PIS51036.1	22.32863933	22.91218041	21.66918073	22.6734824	20.78010233	17.97706625	0.253479184	0.854	-1.83	PIS51036.1	orf19.6416	Protein involved in N-glycosylation; Spider biofilm induced; rat catheter biofilm repressed
PIS56833.1	27.84793067	26.51716999	28.04437687	25.97345141	25.46515819	25.4429634	0.058989088	0.496	-1.84	PIS56833.1	orf19.5297	Ortholog(s) have RNA polymerase II general transcription initiation factor activity, phosphatidylinositol-3-phosphate binding, phosphatidylinositol-5-phosphate binding activity
PIS54875.1	23.73935131	23.75854525	23.90426335	23.4836987	21.23545761	21.07347805	0.174305285	0.792	-1.87	PIS54875.1	orf19.2739	Putative component of the chromatin assembly factor I (CAF-1), which functions as a histone chaperone; involved in negative regulation of biofilm initiation; null mutant shows hyperfilamentous colony wrinkling
PIS51479.1	27.09624823	26.88814609	27.81736593	26.18920557	25.27252638	24.73491516	0.089777781	0.63	-1.87	PIS51479.1	orf19.276	Plasma membrane-associated protein; upregulated in an azole-resistant strain that overexpresses MDR1; Hap43-repressed; Spider biofilm induced
PIS58927.1	30.02295351	30.5942585	29.30416504	27.55992745	28.14589981	28.55762768	0.020282759	0.234	-1.89	PIS58927.1	PHR2	Glycosidase; role in vaginal not systemic infection (low pH not neutral); low pH, high iron, fluconazole, Hap43-induced; Rim101-repressed at pH8; rat catheter biofilm induced; Bcr1-repressed in RPMI a/a biofilms
PIS58180.1	26.36236345	26.38746713	26.42628748	23.61638316	24.48563673	25.36134442	0.010900053	0.133	-1.9	PIS58180.1	HAM1	Putative deoxyribonucleoside triphosphate pyrophosphohydrolase; caspofungin repressed; regulated by Gcn2p and Gcn4p
PIS58124.1	28.75791579	26.5263594	28.83213851	26.28763814	26.19008344	25.94623915	0.005771504	0.0653	-1.9	PIS58124.1	orf19.6259	Exosome non-catalytic core component; involved in 3'-5' RNA processing and degradation in the nucleus and cytoplasm; Spider biofilm induced

PIS52326.1	27.64285569	27.16859294	28.30080075	26.21564592	25.64629757	25.4506328	0.003322039	0.0312	-1.93	PIS52326.1	orf19.6340	Ortholog(s) have DNA-directed 5'-3' RNA polymerase activity, RNA polymerase I activity, RNA polymerase II activity, RNA polymerase III activity, RNA-dependent RNA polymerase activity
PIS58818.1	28.24177873	28.43716302	27.95901195	26.54765973	26.54449344	25.61332908	0.026502642	0.286	-1.98	PIS58818.1	APS3	Component of the adaptor complex AP-3, which is involved in vacuolar protein sorting
PIS48672.1	27.25710126	26.44438806	25.09619228	24.7540304	23.34504192	24.75924353	0.018077042	0.212	-1.98	PIS48672.1	orf19.3393	Putative DEAD-box helicase; Hap43-induced; Spider biofilm induced
PIS56818.1	27.71307479	27.8479515	25.86559795	26.52387675	24.94047922	23.9295129	0.013672927	0.165	-2.01	PIS56818.1	DBP3	Putative ATP-dependent DEAD-box RNA helicase; Hap43-induced; repressed by prostaglandins; Spider biofilm induced
PIS48691.1	26.34655645	24.64994366	26.81458386	24.20676563	24.13432879	23.44940419	0.032892946	0.334	-2.01	PIS48691.1	orf19.4820	Ortholog of S. cerevisiae : YKL162C, C. dubliniensis CD36 : Cd36_09010, C. parapsilosis CDC317 : CPAR2_214170, C. auris B8441 : B9J08_005393 and Candida tenuis NRRL Y-1498 : CANTEDRAFT_95066
PIS54496.1	29.05458954	28.10539152	29.22759609	27.31083371	26.31116386	26.67513363	0.075262949	0.576	-2.03	PIS54496.1	orf19.352	Ortholog of C. dubliniensis CD36 : Cd36_83550, C. parapsilosis CDC317 : CPAR2_404510, C. auris B8441 : B9J08_002270, Debaryomyces hansenii CBS767 : DEHA2B13794g and Pichia stipitis Pignal : PICST_30078
PIS52369.1	27.16854606	24.23238599	28.24436186	25.71947235	24.05767837	23.72701505	0.074240782	0.571	-2.05	PIS52369.1	orf19.725	Ortholog of C. dubliniensis CD36 : Cd36_31880, C. parapsilosis CDC317 : CPAR2_702220, C. auris B8441 : B9J08_003984 and Candida tenuis NRRL Y-1498 : CANTEDRAFT_115848
PIS55462.1	19.28356375	24.43131077	24.41982591	24.32441626	18.3381366	19.04599569	0.212890366	0.828	-2.14	PIS55462.1	IFM1	Putative mitochondrial translation initiation factor; transcript regulated by Nrg1, Mig1, and Tup1
PIS56628.1	21.92493752	24.20854556	24.50590002	22.87946035	16.92486972	24.3862084	0.151194216	0.763	-2.15	PIS56628.1	orf19.6866	Putative U1-70K component of the U1 snRNP, involved in splicing; ortholog of S. cerevisiae SNP1; downregulated upon adherence to polystyrene
PIS55511.1	27.16064593	26.43062366	27.50553837	23.76558079	24.25690014	26.51080136	0.018551607	0.217	-2.19	PIS55511.1	IRE1	Protein kinase involved in regulation of unfolded protein response; role in cell wall regulation; mutant is hypersensitive to caspofungin; Spider biofilm induced

PIS58132.1	26.79639113	27.19287426	25.84469777	24.32228572	24.57319543	24.33339241	0.009921886	0.122	-2.2	PIS58132.1	orf19.4471	Ortholog(s) have role in TOR signaling, re-entry into mitotic cell cycle after pheromone arrest and endoplasmic reticulum, endoplasmic reticulum membrane, endoplasmic reticulum-Golgi intermediate compartment localization
PIS56946.1	28.5910091	25.7066688	26.20095729	26.96754215	24.91887357	21.92709727	0.122490372	0.714	-2.23	PIS56946.1	AKL1	Putative serine/threonine protein kinase; induced during the mating process
PIS51299.1	25.26903842	28.40543114	24.22945411	23.66698571	22.79306021	24.6749708	0.025353948	0.278	-2.26	PIS51299.1	CMD1	Calmodulin; calmodulin inhibitors cause a defect in hyphal growth; transcript not regulated by yeast-hyphal transition; mutation confers hypersensitivity to cytochalasin D and high concentrations of tunicamycin; gene has intron
PIS52091.1	26.92424771	29.19974897	27.86598093	25.75046304	25.17807656	26.19340737	0.081468791	0.6	-2.29	PIS52091.1	CDG1	Putative cysteine dioxygenases; role in conversion of cysteine to sulfite; transcript regulated upon white-opaque switch; rat catheter, Spider and flow model biofilm induced
PIS51731.1	29.4938198	29.11723783	29.50478761	28.04404711	25.80954209	27.28359917	0.000712861	0.0025	-2.33	PIS51731.1	orf19.2041	Component of the RSC chromatin remodeling complex
PIS54835.1	26.9175356	27.38339178	26.24943423	23.71849117	24.68730696	25.04938632	6.49E-05	9.16E-06	-2.37	PIS54835.1	RPB8	Putative subunit of RNA polymerases I, II, and III; regulated by Gcn4p; repressed in response to amino acid starvation (3-aminotriazole treatment); heterozygous null mutant exhibits resistance to parnafungin
PIS56955.1	25.22688632	29.44211722	25.17885224	22.1255211	25.14938899	25.12317152	0.093827029	0.642	-2.48	PIS56955.1	orf19.5368	Ortholog of Vms1; component of a Cdc48-complex involved in protein quality control in S. cerevisiae; Spider biofilm repressed
PIS52151.1	26.7535334	25.73803762	26.42376726	26.2089393	26.72662811	18.51035219	0.161293148	0.777	-2.49	PIS52151.1	orf19.4007	Ortholog(s) have protein-lysine N-methyltransferase activity, role in peptidyl-lysine monomethylation, peptidyl-lysine trimethylation and nucleolus localization
PIS55772.1	27.9956921	28.26120783	27.16895861	27.19269921	23.48942073	25.11852272	0.003431603	0.0323	-2.54	PIS55772.1	orf19.6748	Ortholog(s) have eukaryotic 43S preinitiation complex, eukaryotic translation initiation factor 3 complex localization
PIS49748.1	27.63423847	27.43448814	27.33275804	24.6760874	24.90697682	25.0661691	0.012496332	0.152	-2.58	PIS49748.1	TPO3	Putative polyamine transporter; MFS-MDR family; induced by Sfu1, regulated upon white-opaque; decreased expression in hyphae vs yeast-form cells; regulated by Nrg1; Spider biofilm repressed

PIS56567.1	23.43676055	24.72924114	25.54990605	20.78388146	23.89191792	21.0833943	0.054493325	0.47	-2.65	PIS56567.1	orf19.2612	C2H2 zinc finger transcription factor; expression reduced in ssr1 null mutant; flow model biofilm induced
PIS56714.1	25.7988451	25.08251874	24.8324639	22.86144759	20.76114274	23.91508094	0.0155048	0.185	-2.73	PIS56714.1	orf19.7670	Putative Ca2+/H+ antiporter; oral infection upregulated gene; mutants have reduced capacity to damage oral epithelial cells
PIS50554.1	27.46463688	27.84769875	27.41194423	25.2718137	23.9617065	24.7301859	0.000561702	0.00166	-2.92	PIS50554.1	ECM331	GPI-anchored protein; mainly at plasma membrane, also at cell wall; Hap43, caspofungin-induced; Plc1-regulated; Hog1, Rim101-repressed; colony morphology-related regulated by Ssn6; induced by ketoconazole and hypoxia
PIS51485.1	23.29891656	21.58967254	24.8801351	22.94609837	18.34746066	19.69703365	0.025661314	0.28	-2.93	PIS51485.1	orf19.3535	Ortholog(s) have cellular bud neck, fungal-type vacuole localization
PIS58722.1	24.31585504	25.42314218	25.75402749	23.07644129	20.55007061	20.58143908	0.000272709	0.000325	-3.76	PIS58722.1	CRK1	Protein kinase of the Cdc2 subfamily involved in hyphal development, virulence; promotes hyphal development independently of Cph1 and Efg1; functionally complements pheromone hypersensitivity of S. cerevisiae sgv1 mutant; Hap43p-repressed
PIS54968.1	30.6460416	30.29489607	30.84305763	26.9125344	24.95188532	27.17428191	9.36E-07	6.99E-13	-4.25	PIS54968.1	ARG4	Argininosuccinate lyase, catalyzes the final step in the arginine biosynthesis pathway; alkaline downregulated; flow model biofilm induced; Spider biofilm induced

Protein expression profiles of the *mcu1* Δ mutant grown on YPD medium

Accession	YPD_WT_1	YPD_WT_2	YPD_WT_3	YPD_*mcu1* Δ _1	YPD_*mcu1* Δ _2	YPD_*mcu1* Δ _3	YPD_*mcu1* Δ _vs_YPD_WT_ p.val	YPD_*mcu1* Δ _vs_YPD_WT _p.adj	YPD_*mcu1* Δ _vs_YPD_WT _ratio	Protein_IDs	Gene_names	Description
PIS51256.1	31.0868163	31.2850342	31.7063797	25.59238161	25.5752434	26.06258123	3.79E-11	7.12E-13	-5.62	PIS51256.1	FRP3	Putative ammonium transporter; upregulated in the presence of human neutrophils; fluconazole-downregulated; repressed by nitric oxide; Spider biofilm induced; rat catheter biofilm repressed
PIS56887.1	29.6321963	29.980848	29.6942867	24.42559402	21.13193811	26.89538965	5.58E-05	0.15	-5.62	PIS56887.1	orf19.5547	Protein of unknown function; Hap43-repressed gene
PIS55671.1	31.360077	30.952665	31.4483766	22.79136414	27.6008243	27.86738236	0.000131087	0.237	-5.17	PIS55671.1	ATO7	Putative fungal-specific transmembrane protein
PIS52091.1	25.681518	28.9918905	26.790579	22.19590957	18.91735188	24.91851036	0.000954412	0.572	-5.14	PIS52091.1	CDG1	Putative cysteine dioxygenases; role in conversion of cysteine to sulfite; transcript regulated upon white-opaque switch; rat catheter, Spider and flow model biofilm induced
PIS58510.1	30.5419881	30.3926838	30.4367862	25.81883528	25.36789649	25.00984571	1.33E-09	1.21E-11	-5.06	PIS58510.1	orf19.1367.1	Ortholog of C. parapsilosis CDC317 : CPAR2_407330, C. auris B8441 : B9J08_001010, Candida tenuis NRRL Y-1498 : CANTEDRAFT_114646 and Debaryomyces hansenii CBS767 : DEHA2B05654g
PIS58666.1	27.9479026	28.631619	30.5715843	22.95512446	26.17243803	24.54742786	0.002705538	0.728	-4.49	PIS58666.1	PEX19	Ortholog(s) have peroxisome membrane targeting sequence binding activity

PIS51500.1	35.1665731	36.2316179	36.2538915	31.11742437	31.58920056	31.92754197	2.24E-05	0.0822	-4.34	PIS51500.1	ALD5	NAD-aldehyde dehydrogenase; decreased expression in fluconazole-resistant isolate, or in hyphae; biofilm induced; fluconazole-downregulated; protein abundance is affected by URA3 expression in the CAI-4 strain; stationary phase enriched
PIS48622.1	26.1295939	25.0913421	25.3798057	22.23586911	22.07677073	19.86030662	2.70E-05	0.0892	-4.14	PIS48622.1	orf19.6976	Predicted MFS membrane transporter; member of the proton coupled folate transporter/heme carrier protein family; virulence-group-correlated expression; Spider biofilm induced
PIS55780.1	29.7960845	30.8097781	30.751407	25.3148548	27.53776148	26.821345	2.51E-05	0.0865	-3.89	PIS55780.1	orf19.1409.1	Ribosomal 60S subunit protein L22B; Spider biofilm repressed
PIS51411.1	27.5642354	28.7776733	30.1388224	27.45597389	23.64378943	23.77407139	0.003584074	0.761	-3.87	PIS51411.1	PRA1	Cell surface protein that sequesters zinc from host tissue; enriched at hyphal tips; released extracellularly; binds to host complement regulators; mediates leukocyte adhesion and migration; immunogenic in mouse; produced at ambient pH
PIS55055.1	29.3023425	30.4797974	32.5557659	23.29652772	29.41680045	28.17165034	0.011074713	0.857	-3.82	PIS55055.1	orf19.4595	Ortholog of C. dubliniensis CD36 : Cd36_41860, C. parapsilosis CDC317 : CPAR2_400440, C. auris B8441 : B9J08_002205 and Candida tenuis NRRL Y-1498 : CANTEDRAFT_103033
PIS54875.1	24.5480238	23.4888887	22.7841973	18.85637699	18.37500299	22.37913788	0.013068217	0.868	-3.74	PIS54875.1	orf19.2739	Putative component of the chromatin assembly factor I (CAF-1), which functions as a histone chaperone; involved in negative regulation of biofilm initiation; null mutant shows hyperfilamentous colony wrinkling
PIS58098.1	30.0518574	29.2739266	29.1584278	26.69407356	25.58797933	25.0854627	7.50E-07	0.00265	-3.71	PIS58098.1	orf19.5077	Subunit of mitochondrial respiratory chain complex I; Hap43-repressed gene; repressed by nitric oxide

PIS48452.1	32.2045105	33.0944212	34.3239833	28.01288472	30.05022489	30.54126486	0.000105036	0.212	-3.67	PIS48452.1	orf19.1052	Predicted histone H2B; Hap43-induced gene; Spider biofilm repressed
PIS51272.1	33.2240034	33.7935964	34.1092688	29.83346817	30.16970116	30.19484197	3.30E-07	0.000588	-3.64	PIS51272.1	FOX2	3-hydroxyacyl-CoA epimerase; fatty acid beta-oxidation; induced by phagocytosis; regulated by Mig1, by white-opaque switch, by DNA methylation; transcriptional activation by oleate requires Ctf1; rat catheter and Spider biofilm induced
PIS50418.1	31.0341718	30.8796752	30.3982672	27.60718731	26.86875869	26.9522035	0.000691032	0.514	-3.63	PIS50418.1	SBA1	Similar to co-chaperones; induced in high iron; farnesol-, heavy metal (cadmium) stress-induced; protein level decreases in stationary phase cultures; Hap43-repressed
PIS58326.1	27.4738867	27.7735128	28.0255786	22.29804521	24.31628944	26.27652016	0.001433712	0.639	-3.46	PIS58326.1	SAM4	Putative S-adenosylmethionine-homocysteine methyltransferase; Hap43-repressed; alkaline induced; Spider biofilm repressed
PIS51682.1	29.744167	28.9476499	27.1559904	24.03378047	26.12682329	25.39102547	0.00101334	0.583	-3.43	PIS51682.1	orf19.7027	Protein of unknown function; Spider biofilm induced
PIS52382.1	34.0780445	34.0401696	34.0092578	30.73912073	30.477815	30.70723609	1.79E-08	2.65E-07	-3.4	PIS52382.1	ACS1	Acetyl-CoA synthetase; induced by human neutrophils; fluconazole-repressed; regulated by Nrg1/Mig1; colony morphology-related gene regulation by Ssn6; only in stationary phase cultures; rat catheter biofilm repressed, Spider biofilm induced
PIS58557.1	28.2795527	29.1107265	28.7533203	25.7543567	26.36776553	23.97475737	0.000108769	0.216	-3.35	PIS58557.1	ZRT1	Putative zinc transporter; acts with Pra1 in sequestration of zinc from host tissues during infection; hyphal, macrophage-induced; alkaline induced upon adherence to polystyrene; induced in oralpharyngeal candidasis; Spider biofilm induced

PIS52253.1	26.4264934	26.8654525	27.5333612	25.13819887	18.02239265	27.66277406	0.062319953	0.933	-3.33	PIS52253.1	orf19.1461	S. pombe ortholog SPCC576.01c is a predicted sulfonate dioxygenase; possibly transcriptionally regulated upon hyphal formation; Spider biofilm induced
PIS49777.1	29.5018053	30.438132	30.7758087	27.21861837	26.90485764	26.62845182	2.43E-06	0.0111	-3.32	PIS49777.1	HPD1	3-hydroxypropionate dehydrogenase; involved in degradation of toxic propionyl-CoA; rat catheter and Spider biofilm induced
PIS58068.1	28.9103149	31.0656635	32.2678842	26.64176686	28.11019789	27.5192503	0.005641751	0.806	-3.32	PIS58068.1	RPL25	Putative rRNA-binding ribosomal protein component of the 60S ribosomal subunit; Hap43-induced; colony morphology-related gene regulation by Ssn6
PIS54824.1	31.4937919	32.1807974	33.2212769	28.50539851	28.80252105	29.75947207	5.62E-06	0.0271	-3.28	PIS54824.1	orf19.6306	Trimethylaminobutyraldehyde dehydrogenase, the third enzyme of the carnitine biosynthesis pathway
PIS58836.1	25.8611234	26.3873534	26.8954458	26.09957657	24.94886273	18.3789131	0.038359421	0.918	-3.24	PIS58836.1	HSL1	Probable protein kinase involved in determination of morphology during the cell cycle of both yeast-form and hyphal cells via regulation of Swe1p and Cdc28p; required for full virulence and kidney colonization in mouse systemic infection
PIS55726.1	31.3721873	30.8941977	30.5429432	25.6668179	29.33820735	28.38836792	0.016564928	0.881	-3.14	PIS55726.1	orf19.2452	Protein of unknown function; induced in high iron; repressed in core caspofungin response; ketoconazole-repressed; colony morphology-related gene regulation by Ssn6; possibly subject to Kex2 processing
PIS51465.1	25.1865814	27.0876405	28.4948425	23.08472628	22.49984423	25.80390138	0.006340447	0.816	-3.13	PIS51465.1	orf19.2001	Has domain(s) with predicted L-ascorbic acid binding, iron ion binding, oxidoreductase activity, acting on paired donors, with incorporation or reduction of molecular oxygen activity

PIS50386.1	28.1995007	27.5346406	28.9126965	24.07492005	25.55381137	25.65762264	2.56E-05	0.0872	-3.12	PIS50386.1	JEN2	Dicarboxylic acid transporter; regulated by glucose repression; induced by Rgt1; disruptants not obtained by UAU1 method; rat catheter and Spider biofilm induced
PIS49635.1	29.2918423	30.218016	29.664586	27.89416786	24.79948504	27.11731874	0.000182221	0.275	-3.12	PIS49635.1	UGA2	Predicted succinate semialdehyde dehydrogenase; predicted role in glutamate catabolism; transcription regulated by Mig1, Tup1, Gcn4; mutants are viable
PIS55832.1	33.2006055	32.3397523	31.6427999	25.13033225	31.49128731	31.27555404	0.045534815	0.924	-3.1	PIS55832.1	orf19.2352	Ortholog(s) have triglyceride lipase activity, role in cellular lipid metabolic process and mitochondrion localization
PIS56761.1	26.8795703	27.5238034	27.8986658	24.34225282	23.71467741	25.16191	3.06E-05	0.0978	-3.03	PIS56761.1	ROT2	Alpha-glucosidase II, catalytic subunit, required for N-linked protein glycosylation and normal cell wall synthesis; alkaline downregulated
PIS50638.1	28.0503199	28.5687771	24.8535494	24.11822945	23.53598276	24.78313178	0.002561412	0.721	-3.01	PIS50638.1	ECM38	Putative gamma-glutamyltransferase; alkaline upregulated; Spider biofilm induced; possibly an essential gene, disruptants not obtained by UAU1 method
PIS52326.1	28.0762632	26.3552338	26.880307	24.60790367	24.38656094	23.34581785	9.39E-05	0.201	-2.99	PIS52326.1	orf19.6340	Ortholog(s) have DNA-directed 5'-3' RNA polymerase activity, RNA polymerase I activity, RNA polymerase II activity, RNA polymerase III activity, RNA-dependent RNA polymerase activity
PIS55636.1	33.209786	33.4814069	34.1485374	30.66287398	30.62728213	30.58594571	5.30E-06	0.0255	-2.99	PIS55636.1	POX1-3	Predicted acyl-CoA oxidase; farnesol regulated; stationary phase enriched protein; Spider biofilm induced

PIS51775.1	33.2080075	33.4758722	33.4945268	29.96171581	30.76022878	30.52307543	2.55E-06	0.0115	-2.98	PIS51775.1	ICL1	Isocitrate lyase; glyoxylate cycle enzyme; required for virulence in mice; induced upon phagocytosis by macrophage; farnesol regulated; Pex5-dependent peroxisomal localization; stationary phase enriched; rat catheter, Spider biofilm induced
PIS48669.1	27.0502237	26.6135159	25.693964	24.80361097	23.23796374	22.44136669	0.006993534	0.824	-2.96	PIS48669.1	orf19.3399	Ortholog(s) have histone binding activity, role in chromatin remodeling and Swr1 complex localization
PIS52118.1	32.0068846	31.3178032	32.206593	26.99954996	28.20905797	31.50199044	0.003328136	0.753	-2.94	PIS52118.1	orf19.4521	S. cerevisiae ortholog Env9 has similarity to oxidoreductases and is proposed to have vacuolar functions, found in lipid particles; hyphal-induced expression
PIS54626.1	34.6219959	36.4183741	39.2105291	31.35818038	34.31502516	35.81312242	0.363510053	0.968	-2.92	PIS54626.1	orf19.6610	Ortholog(s) have microtubule binding, microtubule plus end polymerase activity
PIS58997.1	32.596418	33.5207545	34.258354	29.56912111	31.22161768	30.93100369	0.000261995	0.335	-2.88	PIS58997.1	AAT21	Putative aspartate aminotransferase; stationary phase enriched protein; Gcn4-regulated; Spider biofilm induced
PIS51707.1	27.4064094	25.3171251	25.0102062	27.37978183	21.22632654	20.52448037	0.04847361	0.926	-2.87	PIS51707.1	SYS3	Protein similar to S. cerevisiae Sys3p; putative role in endosome-Golgi vesicle docking; upregulated in biofilm; induced upon adherence to polystyrene
PIS48744.1	27.5958472	28.3884201	28.6019723	21.31343335	27.40699937	27.27304708	0.02733341	0.905	-2.86	PIS48744.1	orf19.7263	Putative X-Pro aminopeptidase; Spider biofilm repressed

PIS58357.1	31.2457759	31.9768694	33.0321929	29.60189938	28.6568643	29.44914506	0.006075662	0.812	-2.85	PIS58357.1	orf19.5136	Putative pyridoxamine 5'-phosphate oxidase; planktonic growth and early-stage flow model biofilm induced
PIS58375.1	30.1518872	30.6060463	28.8242534	26.71926603	27.28105039	27.06900076	0.010497651	0.854	-2.84	PIS58375.1	COG4	Ortholog(s) have role in cytoplasm to vacuole transport by the Cvt pathway, intra-Golgi vesicle-mediated transport, macroautophagy, pexophagy, retrograde transport, vesicle recycling within Golgi and Golgi transport complex localization
PIS56908.1	34.6397823	36.4235699	39.2121663	31.57985272	34.33253035	35.83403803	0.274889337	0.964	-2.84	PIS56908.1	OBPA	Putative oxysterol binding protein; non-sex gene located within the MTLa mating-type-like locus; Plc1p-regulated
PIS48814.1	31.3327273	31.3703462	31.8827662	26.1835137	29.92692813	30.03343061	0.00480738	0.791	-2.81	PIS48814.1	orf19.2114	Predicted uricase; ortholog of S. pombe SPCC1223.09; Spider biofilm induced
PIS59006.1	29.4305301	29.9619791	30.7144483	27.55742334	26.97705171	27.15986835	3.41E-05	0.106	-2.8	PIS59006.1	KGD2	Putative dihydrolipoamide S-succinyltransferase; induced in high iron; Hap43-repressed; Spider biofilm repressed
PIS52176.1	26.8878236	29.2969548	30.3250617	25.87488787	25.76944734	26.50987444	0.014682085	0.875	-2.79	PIS52176.1	HSM3	Ortholog(s) have protein folding chaperone activity, role in mismatch repair, proteasome regulatory particle assembly and cytosol, nucleus localization
PIS54865.1	18.5937656	26.5010312	28.4358693	19.09898306	19.40285775	26.65620153	0.255813574	0.963	-2.79	PIS54865.1	orf19.3310	Protein of unknown function; Hap43-repressed; rat catheter and Spider biofilm induced

PIS54857.1	29.087553	30.1864896	31.3077744	27.28307858	27.37219384	27.55488989	0.000371976	0.396	-2.79	PIS54857.1	orf19.6952	Ortholog(s) have DNA binding, chromatin binding, histone deacetylase activity, role in negative regulation of transcription by RNA polymerase II, regulatory ncRNA-mediated gene silencing and HDA1 complex, cytosol, nucleus localization
PIS48221.1	29.4709171	30.5904762	31.563481	27.53524315	27.36417623	28.40117218	0.000241447	0.321	-2.77	PIS48221.1	ECI1	Protein similar to S. cerevisiae Eci1p, which is involved in fatty acid oxidation; transposon mutation affects filamentous growth; expression is regulated upon white-opaque switching
PIS52134.1	26.4420446	28.027499	29.2644069	25.01173469	25.27070357	25.18308052	0.019781648	0.89	-2.76	PIS52134.1	orf19.3759	Putative elongator complex subunit; for modification of wobble nucleosides in tRNA; Spider biofilm induced
PIS56883.1	27.4443514	28.4380449	29.8402202	26.77701784	24.88459265	25.83197866	0.001935466	0.684	-2.74	PIS56883.1	APA2	Putative ATP adenylyltransferase II; regulated by Gcn4; repressed by amino acid starvation (3-AT); induced by prostaglandins; Hap43-repressed; Spider biofilm repressed
PIS50323.1	30.45721	30.0907087	30.6487181	25.43510866	28.54546588	29.101892	0.002403166	0.713	-2.7	PIS50323.1	BNI1	Formin; role in cytoskeletal organization, cell polarity; role in systemic virulence in mouse; cell-cycle regulated localization to site of polarized growth, bud neck; localizes to Spitzenkorper of hyphae, minor localization at septum
PIS50468.1	29.2701595	28.7146083	29.4774706	25.18956765	27.80471473	26.45924574	0.000721116	0.522	-2.67	PIS50468.1	CAR1	Arginase; arginine catabolism; transcript regulated by Nrg1, Mig1, Tup1; colony morphology-related regulation by Ssn6; alkaline induced; protein decreased in stationary phase; sumoylation target; flow model biofilm induced
PIS48754.1	27.3592526	27.8600521	27.6266745	23.35259948	25.62138045	25.93693804	0.002412874	0.714	-2.65	PIS48754.1	orf19.6905	Has domain(s) with predicted LPPG:FO 2-phospho-L-lactate transferase activity

PIS51128.1	26.8631777	28.8644691	29.1507815	24.84558004	26.14976634	25.92872765	0.000699883	0.517	-2.65	PIS51128.1	orf19.7144	Ortholog(s) have GTP binding, GTPase activity, ribosome binding activity
PIS52339.1	31.29977	32.7326576	33.5052738	29.47923364	30.18164144	29.94747406	0.0122422	0.864	-2.64	PIS52339.1	RPL19A	Ribosomal protein L19; repressed upon phagocytosis by murine macrophages; Hap43-induced gene; Spider biofilm repressed
PIS52469.1	25.1571146	25.2000463	26.3886295	24.17142576	23.65323083	21.03195257	0.006055275	0.812	-2.63	PIS52469.1	orf19.2827	Ortholog(s) have kinetochore adaptor activity, microtubule binding activity
PIS51223.1	28.1413092	29.2912456	30.0723293	25.46918852	27.15106481	27.02855308	0.005551217	0.804	-2.62	PIS51223.1	orf19.2265	Component of the RSC chromatin remodeling complex
PIS49787.1	25.3380261	26.2154749	28.9609351	25.35505277	23.54517163	23.81308341	0.070943119	0.937	-2.6	PIS49787.1	orf19.3177	Ortholog(s) have 2,5-diamino-6-ribitylamino-4(3H)-pyrimidinone 5'-phosphate deaminase activity, pseudouridine synthase activity and role in riboflavin biosynthetic process, tRNA pseudouridine synthesis
PIS50447.1	28.6401061	28.8326972	27.9739531	25.72742062	25.05275125	26.86436584	0.00149824	0.646	-2.6	PIS50447.1	orf19.863	Ortholog(s) have structural constituent of ribosome activity, role in mitochondrial cytochrome c oxidase assembly and mitochondrial large ribosomal subunit localization
PIS58546.1	29.3574902	29.4614255	30.3565795	25.86440326	27.74345045	27.78637123	0.000694024	0.515	-2.59	PIS58546.1	orf19.3348	Ortholog(s) have structural constituent of ribosome activity and fungal-type vacuole, mitochondrial large ribosomal subunit localization

PIS58156.1	31.8763601	32.8901799	34.0309473	30.60878211	29.99126389	30.46150159	0.000990786	0.579	-2.58	PIS58156.1	TSA1	TSA/alkyl hydroperoxide peroxidase C (AhPC) family protein; similar to thiol-dependent peroxidases of oxidative stress signaling; antigenic; hyphal surface, nucleus; yeast-form nucleus, cytoplasm; biofilm, phagocytosis, peroxide induced
PIS56604.1	30.6559384	30.8394976	30.8654325	27.72980297	28.58278065	28.36792425	0.00017318	0.269	-2.56	PIS56604.1	YCS4	Putative condensin complex subunit; cell-cycle regulated periodic mRNA expression
PIS51970.1	27.8136796	26.7367754	25.1389049	21.58029657	25.26225232	25.19998854	0.147365812	0.953	-2.55	PIS51970.1	orf19.5342	Ortholog(s) have role in NAD catabolic process and cytosol, extracellular region localization
PIS54716.1	26.7109689	28.035373	28.406721	23.94874864	26.55123513	25.03174147	0.000828583	0.547	-2.54	PIS54716.1	GUK1	Putative guanylate kinase; identified in extracts from biofilm and planktonic cells; protein level decrease in stationary phase cultures; Hap43p-induced gene
PIS54503.1	28.6861519	29.7597174	30.9402126	27.3120959	26.71969006	27.73521731	0.005611942	0.805	-2.54	PIS54503.1	RPL35	Ribosomal protein; downregulation correlates with clinical development of fluconazole resistance; colony morphology-related gene regulation by Ssn6; Hap43-induced; Spider biofilm repressed
PIS49668.1	24.9065155	24.1068664	23.886836	23.23080235	22.49169798	19.58348018	0.006390443	0.817	-2.53	PIS49668.1	CAC2	Component of the chromatin assembly factor I (CAF-1); involved in regulation of white-opaque switching frequency and biofilm initiation; null mutant shows hyperfilamentous wrinkled colonies; macrophage-induced
PIS55507.1	30.4324437	31.4270565	31.9340052	27.23248629	29.4012027	29.56380591	0.0037623	0.766	-2.53	PIS55507.1	MRPL3	Ribosomal protein of the large subunit, mitochondrial; repressed in core stress response; protein present in exponential and stationary growth phase yeast cultures

PIS55635.1	24.7452186	26.6794197	26.5786654	22.39977297	23.55781146	24.44547436	0.001745374	0.669	-2.53	PIS55635.1	RNA1	Putative GTPase-activating protein; protein level decreases in stationary phase cultures; Spider biofilm repressed
PIS58385.1	30.1564799	30.5781831	30.7981473	27.78238052	28.10534893	28.0801364	0.00010293	0.21	-2.52	PIS58385.1	orf19.6887	Predicted ORF from Assembly 19; repressed by nitric oxide; removed from Assembly 20; subsequently reinstated in Assembly 21 based on comparative genome analysis
PIS48654.1	28.2908885	29.1106461	29.3086156	26.93322222	26.72646068	25.52135011	0.000163642	0.263	-2.51	PIS48654.1	orf19.3290	Plasma membrane-localized protein; repressed by nitric oxide; Hap43p-repressed gene
PIS55461.1	24.1417418	25.3107776	25.0285983	25.12733369	23.44716307	18.36869379	0.12859363	0.951	-2.51	PIS55461.1	orf19.5168	Ortholog(s) have unfolded protein binding activity, role in maturation of SSU-rRNA, ribosomal small subunit biogenesis and cytoplasm localization
PIS48638.1	29.053959	28.8169921	29.297778	25.98415668	26.84774593	26.84670586	0.000155914	0.257	-2.5	PIS48638.1	UTP4	Putative U3 snoRNA-associated protein; Hap43-induced; physically interacts with TAP-tagged Nop1; Spider biofilm induced
PIS51794.1	30.6191336	30.5392614	31.8860888	28.35183951	28.80412736	28.50216603	0.000255799	0.331	-2.46	PIS51794.1	orf19.6220.4	Ribosomal 60S subunit protein; Spider biofilm repressed
PIS51081.1	34.5230658	34.9323625	35.7517577	31.98019872	33.00004963	32.87822982	0.000353223	0.387	-2.45	PIS51081.1	CIT1	Citrate synthase; induced by phagocytosis; induced in high iron; Hog1-repressed; Efg1-regulated under yeast, not hyphal growth conditions; present in exponential and stationary phase; Spider biofilm repressed; rat catheter biofilm induced

PIS58197.1	26.4177655	27.7423723	27.6859266	25.81275742	23.34903168	25.33437762	0.004594046	0.787	-2.45	PIS58197.1	orf19.2063	Ortholog of C. dubliniensis CD36 : Cd36_15600, C. parapsilosis CDC317 : CPAR2_213140, C. auris B8441 : B9J08_000687 and Debaryomyces hansenii CBS767 : DEHA2B02442g
PIS55637.1	32.9279273	32.8335228	32.7446622	30.70314853	30.23331421	30.24902786	1.55E-06	0.00733	-2.44	PIS55637.1	PXP2	Putative acyl-CoA oxidase; enzyme of fatty acid beta-oxidation; induced during macrophage infection; opaque specific transcript; putative peroxisome targeting signal; Spider biofilm induced
PIS50537.1	29.0982737	30.581925	31.1192293	27.69766013	27.65961513	28.12049865	0.005239749	0.799	-2.44	PIS50537.1	SFC1	Putative succinate-fumarate transporter; involved in repression of growth on sorbose; alkaline induced; rat catheter biofilm induced; Spider biofilm induced
PIS56842.1	26.8394213	27.2918378	27.0541006	25.77428527	23.21860994	24.92856772	0.002784403	0.732	-2.42	PIS56842.1	ARL1	Putative GTPase in the late Golgi involved in regulation of polarized growth and secretion; mutation confers dose-dependent sensitivity to Brefeldin A
PIS48372.1	30.6437651	31.2803011	31.2617503	29.43605081	27.68792399	28.80327674	0.000116004	0.223	-2.42	PIS48372.1	orf19.5169	Ortholog of C. dubliniensis CD36 : Cd36_72580, C. parapsilosis CDC317 : CPAR2_703580, C. auris B8441 : B9J08_005062 and Candida tenuis NRRL Y-1498 : CANTEDRAFT_114495
PIS49480.1	27.9635887	29.4001042	30.4488211	24.74866458	27.7024775	28.09606379	0.03438154	0.914	-2.42	PIS49480.1	PRE8	Putative alpha-2_sc subunit of proteasome; macrophage-induced protein; regulated by Gcn2p and Gcn4p; transcription is positively regulated by Tbf1p; stationary phase enriched protein
PIS48361.1	26.3421241	28.4703753	29.3255387	24.58929749	25.68502767	26.65479801	0.004491669	0.784	-2.4	PIS48361.1	orf19.246	Predicted metalloendopeptidase; Spider biofilm induced

PIS51708.1	28.9807427	28.9657866	28.9043683	26.29452886	26.7638761	26.61566307	0.001262889	0.619	-2.39	PIS51708.1	CSC25	Guanyl-nucleotide exchange factor; activator of Ras/adenylyl cyclase pathway; functional homolog of S. cerevisiae Cdc25p; commonly called Cdc25; transposon mutation affects filamentous growth
PIS58738.1	26.404119	28.0674739	28.084038	23.52140024	25.68304204	26.19818436	0.005471984	0.803	-2.38	PIS58738.1	ARF3	Similar to but not orthologous to S. cerevisae Arf3; transcript filament induced; Tup1 regulated; rat catheter biofilm repressed (see Locus History Note for Assembly 19 correction)
PIS52312.1	26.4170296	27.573134	27.5864386	24.85628872	24.09047754	25.48195931	0.000175701	0.271	-2.38	PIS52312.1	ATP19	Subunit k of the mitochondrial F1F0 ATP synthase; a large enzyme complex required for ATP synthesis; Spider biofilm repressed
PIS58724.1	29.5768036	30.773549	31.0407324	26.83756367	27.76373073	29.68347522	0.002689158	0.727	-2.37	PIS58724.1	CYT1	Cytochrome c1; induced in high iron; alkaline repressed; possibly an essential gene, disruptants not obtained by UAU1 method; Hap43-repressed; Spider biofilm repressed
PIS51105.1	27.9432848	29.1007922	29.6013738	26.15151544	26.41045546	26.97610954	0.008114996	0.836	-2.37	PIS51105.1	MIS12	Mitochondrial C1-tetrahydrofolate synthase precursor
PIS58693.1	27.5883831	28.9091154	29.6434303	24.87707324	26.90112707	27.2860288	0.004923349	0.793	-2.36	PIS58693.1	CDC55	Ortholog(s) have protein phosphatase regulator activity
PIS58853.1	24.4754354	25.9185981	25.836166	23.3335816	23.86603926	22.02553661	0.002276623	0.706	-2.34	PIS58853.1	orf19.5041	Ortholog of C. dubliniensis CD36 : Cd36_43600, C. parapsilosis CDC317 : CPAR2_403780, Candida tenuis NRRL Y-1498 : CANTEDRAFT_93767 and Debaryomyces hansenii CBS767 : DEHA2G16984g

PIS58366.1	32.4633452	32.9801447	33.4579282	29.88705607	31.01199916	30.98412277	0.000169955	0.267	-2.34	PIS58366.1	UGA1	Putative GABA transaminase; transcription regulated by Mig1 and Tup1; stationary phase enriched protein; rat catheter and Spider biofilm induced
PIS56609.1	22.474897	26.0762104	28.050464	19.09898306	24.69084706	25.82245298	0.162361168	0.955	-2.33	PIS56609.1	orf19.1876	Ortholog(s) have mRNA binding activity and role in mRNA splice site recognition, mRNA splicing, via spliceosome, positive regulation of mRNA splicing, via spliceosome
PIS58270.1	30.6567852	31.8752998	32.8115644	27.86798993	30.24515416	30.26885962	0.008128727	0.836	-2.32	PIS58270.1	MDH1-1	Predicted malate dehydrogenase precursor; macrophage-induced transcript; protein present in exponential and stationary growth phase yeast cultures; Spider biofilm repressed
PIS52389.1	29.1930109	29.7532615	30.8572933	27.00173834	28.08706542	27.77061243	9.07E-05	0.197	-2.31	PIS52389.1	ATP5	Putative F0-ATP synthase FO subunit B; caspofungin repressed; protein level decreased in stationary phase yeast cultures; Spider biofilm repressed
PIS56749.1	24.2032868	22.409675	26.230441	23.32354569	23.3288518	19.26657571	0.098544674	0.945	-2.31	PIS56749.1	FGR15	Putative transcription factor with zinc finger DNA-binding motif; lacks an ortholog in S. cerevisiae; transposon mutation affects filamentous growth; caspofungin induced; mutation causes marginal increase in caspofungin sensitivity
PIS56611.1	28.1929772	27.511865	27.0412555	26.01546235	24.55490975	25.25933617	0.000138194	0.243	-2.31	PIS56611.1	orf19.1872	Plasma membrane protein; repressed by nitric oxide
PIS51217.1	25.9956266	25.9143557	26.9214529	24.79300114	23.59041373	23.56166385	0.001062658	0.591	-2.3	PIS51217.1	orf19.2269	Putative 3-phosphoserine phosphatase; induced by benomyl or in azole-resistant strain that overexpresses MDR1; early-stage flow model biofilm induced; Spider biofilm repressed

PIS51572.1	29.6761327	29.969951	31.0454007	26.85257985	28.40726791	28.5586804	0.004166565	0.777	-2.29	PIS51572.1	orf19.6065	RNA polymerase II holoenzyme/mediator subunit; regulated by Mig1, Tup1; amphotericin B, caspofungin repressed; protein present in exponential and stationary growth phase yeast; Hap43-repressed; Spider biofilm repressed
PIS55785.1	31.1163374	31.968549	32.5860854	29.72886908	29.47271223	29.68261136	0.001008617	0.582	-2.26	PIS55785.1	orf19.1682	Membrane protein; Hap43p-repressed gene; repressed by nitric oxide
PIS51479.1	23.9853626	26.6744045	28.4948963	22.7582885	24.99297711	24.66194814	0.046079826	0.924	-2.25	PIS51479.1	orf19.276	Plasma membrane-associated protein; upregulated in an azole-resistant strain that overexpresses MDR1; Hap43-repressed; Spider biofilm induced
PIS50609.1	30.8675456	30.4699865	31.2393754	26.67657748	29.55473551	29.60282735	0.065364868	0.935	-2.25	PIS50609.1	orf19.6007	Predicted fatty acid acyl transferase-related protein domain; repressed by prostaglandins
PIS54768.1	29.5696155	29.2599676	29.5928216	27.52235364	26.62326886	27.54727315	6.32E-05	0.161	-2.24	PIS54768.1	RET2	Delta subunit of the coatomer complex (COPI); coats Golgi-derived transport vesicles; involved in retrograde transport between Golgi and ER; interacts with Crk1 in the two-hybrid system; Spider biofilm repressed
PIS48386.1	26.2504531	26.9203982	27.9876961	23.44110071	26.57393791	24.43610415	0.012014166	0.863	-2.24	PIS48386.1	TIM22	Mitochondrial inner membrane protein; predicted role in protein import; Hap43-repressed gene; flow model biofilm induced; Spider biofilm repressed
PIS56664.1	29.6023743	29.3945422	30.0401268	26.84379034	27.60275377	27.92632283	2.51E-05	0.0866	-2.22	PIS56664.1	NUO2	NADH-ubiquinone oxidoreductase subunit; Hap43p-repressed gene; repressed by nitric oxide; identified in detergent-resistant membrane fraction (possible lipid raft component)

PIS49845.1	31.9620847	30.7328092	30.6650738	29.06185994	29.13595955	28.5016319	2.71E-05	0.0893	-2.22	PIS49845.1	orf19.1272	Protein of unknown function; may play a role in regulation of cell size; rat catheter biofilm repressed
PIS51106.1	29.9958505	30.6148018	31.3014278	28.40887835	28.31872949	28.54484728	0.00076641	0.533	-2.21	PIS51106.1	orf19.7531	Protein of unknown function; stationary phase enriched protein; induced upon yeast-hypha transition; benomyl or caspofungin induced; Hap43-repressed; Spider biofilm induced
PIS56799.1	29.1219879	30.3080298	30.3818464	27.42927931	28.12701909	27.6438008	0.001433363	0.639	-2.2	PIS56799.1	orf19.7118	Ortholog(s) have nucleoside triphosphate adenylate kinase activity, role in nucleotide metabolic process and mitochondrial inner membrane, mitochondrial matrix localization
PIS55459.1	27.4463084	27.9340604	26.158282	24.54539015	25.92475707	24.4679441	0.001030321	0.586	-2.2	PIS55459.1	orf19.7131	Butyrobetaine dioxygenase, the fourth enzyme of the carnitine biosynthesis pathway
PIS55617.1	24.7838884	27.7860303	28.6682741	25.24987834	25.62052179	23.79881381	0.067985497	0.936	-2.19	PIS55617.1	orf19.5250	Ortholog of C. dubliniensis CD36 : Cd36_11370, C. parapsilosis CDC317 : CPAR2_207480, C. auris B8441 : B9J08_001721 and Candida tenuis NRRL Y-1498 : CANTEDRAFT_120384
PIS48246.1	31.110159	31.324391	31.25141	29.31226466	29.44114073	28.39803751	8.54E-06	0.042	-2.18	PIS48246.1	FAA2-3	Predicted acyl CoA synthetase
PIS52114.1	25.3845419	27.5433234	29.1448442	26.76938537	24.83232968	23.94403388	0.027872565	0.906	-2.18	PIS52114.1	orf19.4517	Putative translation initiation factor eIF3m; essential gene whose repression impedes translation; heterozygous mutant is sensitive to the translation inhibitor nourseothricin

PIS56696.1	30.3487111	30.2076788	30.8574146	28.34149677	28.47798991	28.0816717	0.032504499	0.912	-2.17	PIS56696.1	CAS1	Putative transcription factor with Ku70/Ku80 beta-barrel DNA-binding motif; involved in telomerase regulation and telomere protection; mutation causes marginal increase in caspofungin sensitivity
PIS58564.1	30.8598113	31.36316	32.3656578	29.24369303	29.19567168	29.65348739	7.17E-05	0.172	-2.17	PIS58564.1	CAT2	Major carnitine acetyl transferase; intracellular acetyl-CoA transport; localized in peroxisomes and mitochondria; induced in macrophages; Hog1-repressed; stationary phase enriched; farnesol-upregulated in biofilm; Spider biofilm
PIS58332.1	24.9604802	24.7027938	26.3832298	21.27885993	23.3642446	24.91438952	0.023258334	0.898	-2.16	PIS58332.1	orf19.7450	Ortholog(s) have myosin II tail binding, protein-macromolecule adaptor activity, role in protein localization to cell division site, septin ring assembly and cellular bud neck, cellular bud neck septin ring localization
PIS54590.1	26.7041283	28.5235066	27.8801347	27.36622731	24.72610962	24.5595416	0.005358949	0.801	-2.15	PIS54590.1	CCP1	Cytochrome-c peroxidase N terminus; Rim101, alkaline pH repressed; induced in low iron or by macrophage interaction; oxygen-induced activity; regulated by Sef1, Sfu1, and Hap43; Spider biofilm induced; rat catheter biofilm repressed
PIS58661.1	32.5250528	32.7341274	32.2229287	28.975081	31.19335789	30.85164477	0.000703879	0.518	-2.15	PIS58661.1	RPS20	Putative ribosomal protein; repressed upon phagocytosis by murine macrophage; transcript positively regulated by Tbf1; Spider biofilm repressed
PIS48136.1	32.3687657	32.8946978	33.5633768	30.74112342	30.86325962	30.80295974	0.003160931	0.747	-2.14	PIS48136.1	ALD6	Putative aldehyde dehydrogenase; stationary phase enriched protein; expression regulated upon white-opaque switch; rat catheter biofilm induced; rat catheter and Spider biofilm induced
PIS48403.1	26.9913031	25.2454315	26.3390992	27.06229821	19.93241685	25.16371058	0.118881072	0.949	-2.14	PIS48403.1	orf19.6551	Ortholog(s) have SNAP receptor activity, role in Golgi vesicle transport, vesicle fusion and Golgi medial cisterna, SNARE complex localization

PIS49769.1	27.0925008	26.8674143	27.4182921	23.59632266	26.43080012	24.93882352	0.027155941	0.905	-2.14	PIS49769.1	orf19.7375	Putative U1A component of the U1 snRNP, involved in splicing; contains two RNA recognition motifs (RRMs); ortholog of S. cerevisiae MUD1
PIS49674.1	32.5557142	33.1567331	33.2846329	29.9222308	31.43609966	31.20873831	0.008916564	0.843	-2.14	PIS49674.1	RPS25B	Ribosomal protein; macrophage/pseudohyphal-induced after 16 h; repressed upon phagocytosis by murine macrophage; transcript positively regulated by Tbf1; 5'-UTR intron; Hap43-induced; Spider biofilm repressed
PIS51902.1	32.26002	32.7054905	33.6317627	29.81913739	31.0656405	31.41910307	0.004890773	0.793	-2.1	PIS51902.1	ATP2	F1 beta subunit of F1F0 ATPase complex; antigenic in human, mice; induced by ciclopirox olamine; caspofungin repressed; macrophage/pseudohyphal-induced; detected during exponential and stationary growth phases; Spider
PIS58051.1	26.5074869	26.8906713	27.4326312	23.99582998	24.52775059	26.01048215	0.018817719	0.888	-2.1	PIS58051.1	orf19.7618	Putative nucleolar protein with a predicted role in pre-18S rRNA processing; Plc1p-regulated; Spider biofilm induced
PIS55013.1	29.076382	29.4534917	28.8950444	26.81558856	26.51534138	27.83252536	7.02E-05	0.17	-2.09	PIS55013.1	orf19.1179	Subunit of mitochondrial respiratory chain complex I; induced in high iron; possibly subject to Kex2 processing; Hap43-repressed
PIS56610.1	28.4888617	27.8791971	28.6000305	27.64586853	27.01310726	24.07280676	0.014739797	0.875	-2.08	PIS56610.1	orf19.1873	Protein of unknown function; Hap43-repressed gene; repressed by nitric oxide
PIS51305.1	28.1195792	29.3984686	28.5556235	24.18710594	27.32138592	28.36351504	0.034040052	0.913	-2.07	PIS51305.1	orf19.1975	Ortholog(s) have role in mRNA splicing, via spliceosome and U4/U6 x U5 tri-snRNP complex, U5 snRNP localization

PIS55732.1	29.5561586	29.4035315	27.8597196	27.01727877	27.14034858	26.45085314	0.00399347	0.772	-2.07	PIS55732.1	orf19.477	Ortholog(s) have role in mitochondrial translation and mitochondrion localization
PIS55755.1	33.6439163	34.6475799	35.7324269	31.71830521	32.86185556	33.23631676	0.010184883	0.852	-2.07	PIS55755.1	PGK1	Phosphoglycerate kinase; localizes to cell wall and cytoplasm; antigenic in murine/human infection; flow model biofilm, Hog1-, Hap43-, GCN-induced; repressed upon phagocytosis; repressed in Spider biofilms by Bcr1, Ndt80, Rob1, Brg1
PIS58688.1	31.1902075	31.2842917	30.9180432	28.96721871	29.43058739	28.78977111	0.000853596	0.553	-2.07	PIS58688.1	RPL38	60S ribosomal ribosomal protein subunit; genes encoding cytoplasmic ribosomal subunits, translation factors, tRNA synthetases are downregulated upon phagocytosis by murine macrophage
PIS58729.1	30.5707796	31.3490292	31.3490493	29.25935012	28.97776078	28.86496223	6.08E-05	0.157	-2.06	PIS58729.1	orf19.1709	Sterol carrier domain protein; alkaline downregulated; colony morphology-related gene regulation by Ssn6; Spider biofilm induced
PIS55629.1	29.8120668	30.2997367	30.3963101	28.15676148	27.83952737	28.33291939	0.000225654	0.31	-2.06	PIS55629.1	orf19.6838	Putative protein of unknown function, transcript upregulated in clinical isolates from HIV+ patients with oral candidiasis; Spider biofilm induced
PIS58225.1	30.5297496	30.4492019	30.6488255	28.07719073	28.98964662	28.39548539	3.91E-05	0.118	-2.06	PIS58225.1	orf19.6898.1	Ortholog of C. dubliniensis CD36 : Cd36_71020, C. parapsilosis CDC317 : CPAR2_300360, C. auris B8441 : B9J08_000715 and Candida tenuis NRRL Y-1498 : CANTEDRAFT_105022
PIS52054.1	28.4896842	29.3368716	30.793599	27.336536	27.57054163	27.58160331	0.010862509	0.856	-2.04	PIS52054.1	ADO1	Adenosine kinase; heterozygous null mutant is resistant to cordycepin in C. albicans fitness test; ketoconazole-induced; protein level decrease in stationary phase cultures; sumoylation target; Spider biofilm repressed

PIS48648.1	28.8426187	30.9426717	30.352115	26.33807474	28.85918193	28.8097049	0.008250209	0.837	-2.04	PIS48648.1	ATP3	F1-ATP synthase complex subunit; caspofungin repressed; flucytosine and macrophage/pseudohyphal-induced; present in exponential and stationary growth phases; Hap43p-induced gene
PIS58790.1	28.1766391	28.5324231	29.6756251	26.73643534	26.62639151	26.90976257	0.017201274	0.883	-2.04	PIS58790.1	ERP5	Protein involved in ER to Golgi transport; rat catheter and Spider biofilm repressed
PIS51770.1	34.1123883	34.1090493	34.4709163	32.03403102	31.87759944	32.66312737	2.78E-05	0.0903	-2.04	PIS51770.1	HHF22	Putative histone H4; regulated by Efg1; flucytosine, fluconazole-induced; amphotericin B, caspofungin repressed; colony morphology-related gene regulation by Ssn6; Hap43-induced; rat catheter and Spider biofilm repressed
PIS55686.1	23.6523635	27.6121753	29.1691402	24.34681416	23.40876944	26.55654433	0.164825497	0.955	-2.04	PIS55686.1	orf19.2733	Putative subunit of phosphatidylinositol 3-kinase complexes I and II; transcription is activated in the presence of elevated CO2
PIS51695.1	29.63331	30.6446325	31.0217909	29.02648552	28.10347043	28.05475553	0.000431367	0.425	-2.04	PIS51695.1	orf19.4633	Ortholog(s) have carbonyl reductase (NADPH) activity, diacetyl reductase ((S)-acetoin forming) activity, oxidoreductase activity, serine 3-dehydrogenase activity and role in acetoin metabolic process
PIS51718.1	27.9653756	29.1498604	30.2630379	25.97159788	27.45538513	27.85104038	0.023573245	0.898	-2.03	PIS51718.1	IML2	Protein of unknown function; early-stage flow model biofilm induced; Hap43-repressed; Spider biofilm repressed
PIS52359.1	26.418937	28.131923	28.7345045	24.09157483	26.26048574	26.84858384	0.043104441	0.922	-2.03	PIS52359.1	orf19.4016	Putative ubiquinol-cytochrome-c reductase; amphotericin B induced; repressed by nitric oxide, Hap43p, and Spider biofilm; null mutant is viable but shows decreased vegetative growth on several carbon sources

PIS50377.1	34.6116135	34.7421339	34.3631186	32.57683187	32.58956315	32.48379397	2.56E-06	0.0115	-2.02	PIS50377.1	GDH2	Mitochondrial NAD-dependent glutamate dehydrogenase; catalyzes deamination of glutamate to alpha-ketoglutarate; fungal-specific; regulated by Nrg1p, Mig1p, Tup1p, and Gcn4p; stationary phase enriched; Spider and flow model biofilm induced
PIS51916.1	24.1237284	26.7501883	27.4128162	24.21380732	23.96047699	24.05073234	0.01875182	0.888	-2.02	PIS51916.1	SPC2	Signal peptidase complex component; role in ER protein translocation; transcript is induced upon filamentous growth
PIS58374.1	32.9971806	33.4276863	33.6720613	31.02999828	31.47688549	31.55058134	0.000176905	0.272	-2.01	PIS58374.1	LAP3	Putative aminopeptidase; positively regulated by Sfu1; clade-associated gene expression; virulence-group-correlated expression; induced by alpha pheromone in SpiderM medium; Hap43-induced; Spider and flow model biofilm induced
PIS48486.1	25.6103418	27.1563739	27.4640194	25.09908737	24.93830222	24.16834692	0.010027601	0.851	-2.01	PIS48486.1	orf19.1887	Ortholog(s) have sterol esterase activity, role in sterol metabolic process and lipid droplet, membrane localization
PIS58381.1	31.9853183	32.824604	32.2100951	30.02785703	30.99871777	29.97483867	0.019276189	0.889	-2.01	PIS58381.1	orf19.6882.1	Ribosomal 60S subunit protein; Spider biofilm repressed
PIS58171.1	27.5303177	25.776419	25.0760516	24.3428994	23.79353094	24.24256019	0.030805129	0.91	-2	PIS58171.1	SFL1	Transcription factor involved in negative regulation of morphogenesis, flocculation and virulence; induced in core caspofungin response; Spider biofilm induced
PIS58322.1	32.8031594	33.2789331	32.9502681	30.1117612	31.34977763	31.57487642	0.000567042	0.477	-2	PIS58322.1	YNK1	Nucleoside diphosphate kinase (NDP kinase); homo-hexameric; soluble protein in hyphae; flucytosine induced; biofilm induced; macrophage-induced protein; stationary phase enriched protein; Spider biofilm repressed

PIS48743.1	27.6805814	28.2269734	28.4873199	26.28681711	26.28326312	25.8407273	6.42E-05	0.162	-1.99	PIS48743.1	orf19.7264	Metalloprotease subunit of the 19S regulatory particle of the 26S proteasome lid; couples the deubiquitination and degradation of proteasome substrates; role in fission of mitochondria and peroxisome; Spider biofilm repressed
PIS51094.1	25.4776769	28.7500814	28.6886949	24.21601415	26.2024775	26.57064416	0.029463961	0.908	-1.98	PIS51094.1	AGP2	Amino acid permease; hyphal repressed; white-opaque switch regulated; induced in core caspofungin response, during cell wall regeneration, by flucytosine; regulated by Sef1, Sfu1, and Hap43; rat catheter and Spider biofilm induced
PIS51259.1	27.480636	28.9908727	28.6260528	27.00960915	26.48038934	25.67785129	0.00185476	0.678	-1.98	PIS51259.1	EFG1	bHLH transcription factor; required for white-phase cell type, RPMI and Spider biofilm formation, hyphal growth, cell-wall gene regulation; roles in adhesion, virulence; Cph1 and Efg1 have role in host cytokine response; binds E-box
PIS51036.1	23.4920683	22.3648856	23.6814109	18.69973365	19.51581586	25.39182194	0.218388531	0.96	-1.98	PIS51036.1	orf19.6416	Protein involved in N-glycosylation; Spider biofilm induced; rat catheter biofilm repressed
PIS54885.1	26.8883129	27.2226014	27.1825136	25.53042911	25.14254996	24.67399714	1.06E-05	0.0509	-1.98	PIS54885.1	PSA2	Mannose-1-phosphate guanyltransferase; Hap43, macrophage-repressed; stationary phase enriched protein; Spider biofilm induced; rat catheter biofilm repressed
PIS51685.1	23.565842	25.1288828	22.8351105	23.99281578	20.07595344	21.50614529	0.087801221	0.942	-1.98	PIS51685.1	REX2	Putative 3'-5' RNA exonuclease with a predicted role in 3'-end processing of U4 and U5 snRNAs, 5S and 5.8S rRNAs; rat catheter biofilm induced
PIS51263.1	27.4777643	27.9507815	28.0019085	27.16827159	26.42029614	23.93371921	0.027888794	0.906	-1.97	PIS51263.1	HOF1	Protein involved in cytokinesis and DNA damage response; interacts genetically with Rad53p-dependent checkpoint; mutant is viable

PIS49550.1	34.3709386	34.6908044	34.9037012	32.72231685	32.75134549	32.58967205	6.22E-05	0.159	-1.97	PIS49550.1	orf19.2478.1	60S ribosomal protein L7; snoRNA snR39b encoded within the 2nd intron
PIS48624.1	33.4953009	33.6533036	33.2394481	30.8442399	31.84354303	31.81061137	2.14E-05	0.0804	-1.96	PIS48624.1	YST1	Ribosome-associated protein; antigenic in mice; complements S. cerevisiae yst1 yst2 mutant; similar to laminin receptor; predicted S/T phosphorylation, N-glycosylation, myristoylation, Hap43-, Gcn4-regulated; Spider biofilm repressed
PIS55818.1	32.6831891	32.7013227	32.8760282	30.30315534	31.0031755	31.10410864	0.000229216	0.312	-1.95	PIS55818.1	SEC27	Ortholog(s) have ubiquitin binding activity
PIS48793.1	34.0301146	33.6385332	33.7858618	31.29004952	32.15371703	32.18676607	0.000967202	0.575	-1.94	PIS48793.1	ADK1	Putative adenylate kinase; repressed in hyphae; macrophage-induced protein; adenylate kinase release used as marker for cell lysis; possibly essential (UAU1 method); flow model biofilm induced; rat catheter and Spider biofilm repressed
PIS48607.1	28.0133507	28.4545714	28.7099946	24.93311638	27.14694654	27.27385433	0.010527722	0.854	-1.94	PIS48607.1	orf19.2299	Ortholog(s) have protein tag activity
PIS54782.1	26.858421	27.4533437	26.9039853	25.17037845	24.95016916	25.265448	0.007538654	0.83	-1.94	PIS54782.1	orf19.536	Ortholog(s) have chromatin binding, molecular adaptor activity, ubiquitin binding activity and role in chromatin organization, transcription by RNA polymerase II
PIS51866.1	27.5899592	26.9048606	26.7169163	24.3599705	25.87447115	25.19599004	0.000295148	0.356	-1.93	PIS51866.1	orf19.3762	Ortholog of C. dubliniensis CD36 : Cd36_11720, C. parapsilosis CDC317 : CPAR2_201890, C. auris B8441 : B9J08_003468 and Candida tenuis NRRL Y-1498 : CANTEDRAFT_91948

PIS51511.1	26.9302389	27.3572056	27.1407971	26.67835654	24.99620171	23.96992248	0.002383962	0.712	-1.93	PIS51511.1	SMD2	Putative Core Sm protein; Hap43p-induced gene; flucytosine induced
PIS54968.1	30.7878389	30.8745458	30.8959347	29.36057456	29.13069102	28.31341027	0.001819401	0.675	-1.92	PIS54968.1	ARG4	Argininosuccinate lyase, catalyzes the final step in the arginine biosynthesis pathway; alkaline downregulated; flow model biofilm induced; Spider biofilm induced
PIS52198.1	24.0468463	26.1244017	27.5800067	24.2282459	24.03250646	23.73810523	0.021323418	0.894	-1.92	PIS52198.1	orf19.5411	Ortholog(s) have NEDD8 transferase activity and role in protein neddylation
PIS49488.1	27.7832076	28.5125278	27.7641022	25.00550989	26.46506288	26.83158298	0.002342759	0.71	-1.92	PIS49488.1	orf19.7326	Ortholog(s) have protein-lysine N-methyltransferase activity and role in peptidyl-lysine dimethylation, peptidyl-lysine monomethylation
PIS54863.1	30.0769593	30.8570159	30.9429411	27.7198067	29.0400932	29.36690096	0.00414114	0.776	-1.92	PIS54863.1	SAR1	Functional homolog of S. cerevisiae Sar1; which is required for ER-to-Golgi protein transport; binds GTP; similar to small GTPase superfamily proteins; gene has intron; Hap43-induced; rat catheter biofilm repressed
PIS54513.1	26.5483068	27.1569965	28.1818628	26.8417195	24.34612546	24.97942431	0.032930293	0.912	-1.91	PIS54513.1	RIA1	Putative translation elongation factor; genes encoding ribosomal subunits, translation factors, and tRNA synthetases are downregulated upon phagocytosis by murine macrophage
PIS54915.1	23.5032234	23.013546	24.1653087	25.11602413	19.55692378	20.32215338	0.157040536	0.954	-1.9	PIS54915.1	NUF2	Kinetochore component; amount of Nuf2p and Mtw1p protein detected at each centromere is consistent with a single kinetochore microtubule attachment site

PIS56533.1	25.4824995	27.7458402	28.2115326	24.6868543	24.60475498	26.44266375	0.051037695	0.927	-1.9	PIS56533.1	orf19.5514	Ortholog of S. pombe SPCC550.08, an N-acetyltransferase; transcript induced during growth in the mouse cecum
PIS50490.1	30.7656581	31.5421501	33.3080958	28.7295834	30.49831076	30.69765331	0.022999899	0.897	-1.9	PIS50490.1	PUT2	Putative delta-1-pyrroline-5-carboxylate dehydrogenase; regulated by Put3p; null mutant cannot grow on proline as a nitrogen source and shows reduced invasive growth both in vitro and in vivo; Spider biofilm induced
PIS54830.1	33.8704222	34.5086312	34.5378149	31.52476921	32.78768567	32.89108992	0.000485731	0.448	-1.9	PIS54830.1	RPS3	Ribosomal protein S3; Hog1, Hap43-induced; grepressed upon phagocytosis by murine macrophage; present in exponential and stationary phase cells; Spider biofilm repressed
PIS49627.1	30.1996082	30.7490617	30.8915447	27.7714432	28.90506979	29.48334451	0.276631764	0.964	-1.89	PIS49627.1	orf19.6693	Has domain(s) with predicted metal ion binding activity
PIS56545.1	31.0769997	31.6271032	32.5143625	28.89608718	30.24662646	30.40488059	0.005609013	0.805	-1.89	PIS56545.1	RPL23A	Ribosomal protein; downregulated upon phagocytosis by murine macrophage; Hap43-induced; sumoylation target; Spider biofilm repressed
PIS54812.1	29.5454615	30.4697347	31.0649461	28.57357871	28.52878309	28.29568084	0.001042366	0.587	-1.89	PIS54812.1	SPS20	Peroxisomal 2,4-dienoyl-CoA reductase; stationary phase enriched protein; Spider biofilm induced
PIS48799.1	27.4597547	27.6404302	28.1657021	24.96335306	26.630568	26.04416595	0.001674561	0.663	-1.88	PIS48799.1	RIP1	Putative ubiquinol cytochrome c-reductase; induced by high iron; Hap43, nitric oxide, alkaline repressed; Spider biofilm repressed

PIS56688.1	34.7677077	35.2011542	35.7609402	33.3696339	33.38134099	33.33480484	0.001127426	0.601	-1.88	PIS56688.1	TDH3	NAD-linked glyceraldehyde-3-phosphate dehydrogenase; binds fibronectin, laminin; at cell surface; antigenic in infection; farnesol-repressed; stationary phase-enriched; GlcNAc-induced; flow model biofilm induced; Spider biofilm repressed
PIS51623.1	29.582911	30.5391266	29.8269117	28.05320946	28.20168783	28.07621556	0.002210223	0.702	-1.87	PIS51623.1	GVP36	BAR domain protein; ocalizes to early and late Golgi vesicles; predicted role in adaptation to varying nutrient concentrations, fluid-phase endocytosis, actin cytoskeleton polarization and vacuole biogenesis; rat catheter biofilm repressed
PIS52281.1	30.7490603	30.1494187	29.7218619	28.59584105	28.29870325	28.15977789	0.043849468	0.922	-1.86	PIS52281.1	orf19.3813	Ortholog of C. dubliniensis CD36 : Cd36_44340, C. parapsilosis CDC317 : CPAR2_302240, Candida tenuis NRRL Y-1498 : CANTEDRAFT_105331 and Debaryomyces hansenii CBS767 : DEHA2E03454g
PIS58542.1	24.9246064	27.8666733	27.9007557	25.33232381	24.94009161	24.89792021	0.024474363	0.9	-1.84	PIS58542.1	DSL1	Protein similar to S. cerevisiae Dsl1p, which is a member of the t-SNARE complex of the endoplasmic reticulum
PIS49806.1	27.3318397	26.5415292	26.9893244	25.31286286	24.8617041	25.16495519	0.000125837	0.232	-1.84	PIS49806.1	GLG2	Putative self-glucosylating initiator of glycogen synthesis; expression regulated upon white-opaque switch; hypha-induced; Spider biofilm induced
PIS52461.1	27.2908388	28.6935305	27.8794676	25.04811015	27.08075771	26.22672743	0.009746509	0.849	-1.84	PIS52461.1	orf19.2828	Ortholog(s) have alpha-tubulin binding, microtubule binding activity, role in cytoplasmic microtubule organization, post-chaperonin tubulin folding pathway, protein folding and cytoplasm localization
PIS52369.1	24.1348255	26.6518932	27.7041874	24.35008025	24.44379702	24.18215435	0.104904134	0.946	-1.84	PIS52369.1	orf19.725	Ortholog of C. dubliniensis CD36 : Cd36_31880, C. parapsilosis CDC317 : CPAR2_702220, C. auris B8441 : B9J08_003984 and Candida tenuis NRRL Y-1498 : CANTEDRAFT_115848

PIS48245.1	27.9316682	28.2730892	29.0259324	25.95758382	26.66889326	27.11906006	0.001585565	0.655	-1.83	PIS48245.1	ARO10	Aromatic decarboxylase; Ehrlich fusel oil pathway of aromatic alcohol biosynthesis; alkaline repressed; protein abundance affected by URA3 expression in CAI-4 strain; Spider biofilm induced
PIS58166.1	30.0485043	30.7093969	30.2840969	28.12149302	28.66020548	28.75714613	0.00032482	0.372	-1.83	PIS58166.1	GRP2	NAD(H)-linked methylglyoxal oxidoreductase involved in regulation of methylglyoxal and pyruvate levels; regulation associated with azole resistance; induced in core stress response or by oxidative stress via Cap1, fluphenazine, benomyl
PIS48543.1	29.0421379	29.8388441	31.1137661	27.23252125	28.25290248	29.02457652	0.007859392	0.833	-1.83	PIS48543.1	orf19.4622	Ortholog(s) have transcription factor TFIIH holo complex binding activity
PIS58100.1	26.468475	27.5957868	29.3717782	25.82838797	25.68844343	26.43359652	0.164339172	0.955	-1.83	PIS58100.1	UBA2	Ortholog(s) have SUMO activating enzyme activity, role in protein sumoylation and SUMO activating enzyme complex, nucleus localization
PIS58574.1	35.7349224	36.1854488	36.1799796	34.20623934	34.3566659	34.08452156	1.52E-05	0.0661	-1.82	PIS58574.1	ATP1	ATP synthase alpha subunit; antigenic in human/mouse; at hyphal surface; ciclopirox, ketoconazole, flucytosine induced; Efg1, caspofungin repressed; may be essential; sumoylation target; stationary phase-enriched; Spider biofilm repressed
PIS51372.1	27.0681341	27.6496425	28.2155343	25.40091783	26.12390348	25.95314928	0.0146621	0.874	-1.82	PIS51372.1	FRP6	Putative ammonia transport protein; regulated by Nrg1 and Tup1; regulated by Ssn6; induced by human neutrophils
PIS49734.1	27.826607	29.676103	30.0610674	25.8065222	27.88657787	28.39821062	0.122685417	0.95	-1.82	PIS49734.1	RPS12	Acidic ribosomal protein S12; regulated by Gcn4, activated by Tbf1; repressed by amino acid starvation (3-AT); protein abundance is affected by URA3 expression in CAI-4 strain background; sumoylation target; Spider biofilm repressed

PIS51637.1	24.6099841	28.2603675	28.1274871	24.73986952	24.51381828	26.32738544	0.054189027	0.929	-1.81	PIS51637.1	CNS1	Putative co-chaperone; Hap43p-induced gene; mutation confers hypersensitivity to radicicol
PIS49631.1	23.9837604	25.0857696	22.5893112	23.42431013	20.52570511	22.28465624	0.031342748	0.91	-1.81	PIS49631.1	orf19.7290	Ortholog(s) have chromatin binding, mRNA binding activity and role in deadenylation-dependent decapping of nuclear-transcribed mRNA, nuclear-transcribed mRNA catabolic process, deadenylation-dependent decay
PIS56838.1	32.7683601	32.2856666	32.2135192	30.60731472	30.66049013	30.5991244	2.02E-05	0.0781	-1.8	PIS56838.1	AMS1	Putative alpha-mannosidase; transcript regulated by Nrg1; induced during cell wall regeneration; flow model biofilm induced; Spider biofilm induced
PIS58985.1	29.4970804	29.3316058	29.2181789	28.10556669	27.3164307	27.25862622	0.00017356	0.269	-1.79	PIS58985.1	CTN3	Peroxisomal carnitine acetyl transferase; no obvious metabolic, hyphal, virulence defects in Ura+ strain; induced by macrophage engulfment, hyphal growth, starvation, nonfermentable carbon sources; rat catheter, Spider biofilm induced
PIS58230.1	21.6220613	23.0393006	25.348393	22.38499426	21.90711473	20.35193428	0.066829856	0.935	-1.79	PIS58230.1	DBP7	Putative ATP-dependent DEAD-box RNA helicase; Hap43-induced; rat catheter biofilm induced
PIS51068.1	29.9065923	29.5245706	30.3543982	29.38476672	28.28064259	26.73610273	0.031206494	0.91	-1.79	PIS51068.1	orf19.1625	Putative ubiquinone oxidoreductase; repressed by nitric oxide; Hap43p-repressed
PIS51193.1	28.6431726	29.0286905	29.6814563	27.13065055	27.58455643	27.25573603	0.053526658	0.929	-1.79	PIS51193.1	PUP1	Putative beta 2 subunit of the 20S proteasome; macrophage/pseudohyphal-repressed; Spider biofilm repressed

PIS56741.1	26.2477242	27.6532096	28.5784858	24.33404761	25.32747273	27.46485385	0.052570837	0.928	-1.78	PIS56741.1	COQ6	Ortholog(s) have oxidoreductase activity, acting on paired donors, with incorporation or reduction of molecular oxygen, reduced flavin or flavoprotein as one donor, and incorporation of one atom of oxygen activity
PIS51276.1	28.3958473	29.355838	29.6739986	25.23630405	28.25671872	28.60016435	0.036972286	0.917	-1.78	PIS51276.1	SCL1	Proteasome subunit YC7alpha; protein present in exponential and stationary growth phase yeast cultures; flow model and Spider biofilm repressed
PIS51080.1	26.756002	28.5792847	29.9247702	26.36647189	26.48548945	27.08451194	0.050366735	0.927	-1.77	PIS51080.1	orf19.2382	Protein similar to isoleucyl-tRNA synthetase; isoleucyl-tRNA synthetase is the target of drugs including the cyclic beta-amino acid icofungipen/PLD-118/BAY-10-8888 and mupirocin
PIS50310.1	27.2684881	24.8636043	26.0400187	24.78422897	24.40070055	23.67129317	0.012406408	0.865	-1.77	PIS50310.1	orf19.7642	Ortholog of S. cerevisiae Vps3; CORVET tethering complex component involved in vacuolar protein sorting; Hap43-repressed gene
PIS51831.1	26.989477	31.1381336	31.1809457	27.73908723	28.22614579	28.01871544	0.15611132	0.954	-1.77	PIS51831.1	PRT1	Putative translation initiation factor eIF3; mutation confers hypersensitivity to roridin A, verrucarin A; genes encoding ribosomal subunits, translation factors, tRNA synthetases are downregulated upon phagocytosis by murine macrophages
PIS54622.1	31.373467	31.4727636	31.816564	29.63277723	30.00130441	29.74190413	0.001541953	0.651	-1.76	PIS54622.1	SUP35	Translation factor eRF3; shows prion-like aggregation in some, not all, studies; partially complements S. cerevisiae sup35 mutant translation defect; species barrier with S. cerevisiae Sup35p prion; gene not regulated by yeast-hyphal switch
PIS58102.1	27.0116256	28.2163543	29.5520146	26.29831091	26.27394409	26.96172324	0.031023242	0.91	-1.75	PIS58102.1	NRP1	Ortholog(s) have role in cellular response to temperature stimulus, regulation of heterochromatin formation and cytoplasmic stress granule, nucleus, protein aggregate center localization

PIS48273.1	33.4636988	33.6807366	34.2673548	32.14241145	32.06114624	31.95306977	7.00E-05	0.17	-1.75	PIS48273.1	RPL6	Ortholog of S. cerevisiae ribosomal subunit, Rpl6B; transposon mutation affects filamentous growth; translation-related genes are downregulated upon phagocytosis by murine macrophage; Hap43-induced; Spider biofilm repressed
PIS51691.1	30.3411006	31.8618748	32.4096751	29.48772867	29.7543169	30.12922786	0.03393581	0.913	-1.75	PIS51691.1	RPS28B	Putative ribosomal protein S28B
PIS58786.1	25.5200869	25.265101	24.5782749	23.78793978	23.52378037	22.79626047	0.000657544	0.505	-1.75	PIS58786.1	SFT2	Putative membrane protein; transcript regulated by Mig1; Spider biofilm induced
PIS52399.1	23.923651	25.3684603	25.2386422	22.76518381	22.79225582	23.76160399	0.017056061	0.883	-1.74	PIS52399.1	GCA1	Extracellular/plasma membrane-associated glucoamylase; expressed in rat oral infection; regulated by carbohydrates, pH, galactose; promotes biofilm matrix formation; flow model biofilm induced; Bcr1 repressed in RPMI a/a biofilms
PIS48653.1	22.7876675	23.5283973	24.1959093	19.92184486	21.83385223	23.54661609	0.04132796	0.92	-1.74	PIS48653.1	orf19.3289	Phosphorylated protein of unknown function
PIS48559.1	28.3237378	29.8686341	31.1216783	26.03402348	27.9441741	30.12252452	0.121961552	0.949	-1.74	PIS48559.1	RPL37B	Ribosomal protein L37; Hap43-induced; Spider biofilm repressed
PIS48407.1	30.6670715	31.4096689	32.3940249	29.86053437	29.79687046	29.57914289	0.003461499	0.757	-1.74	PIS48407.1	RPL5	Ribosomal protein; repressed upon phagocytosis by murine macrophages; Hap43-induced; Spider biofilm repressed

PIS52443.1	28.5574313	28.2526443	27.959412	27.13676347	25.8715134	26.58328557	0.010465453	0.854	-1.73	PIS52443.1	FAA2	Putative acyl CoA synthetase; expression regulated upon white-opaque switch; rat catheter biofilm induced; Spider biofilm induced
PIS51501.1	31.6805991	31.7246146	31.6058432	29.22828242	30.19920488	30.3865256	0.028991905	0.907	-1.73	PIS51501.1	RPS24	Predicted ribosomal protein; hyphal downregulated; repressed upon phagocytosis by murine macrophage; transcriptionally activated by Tbf1; Spider biofilm repressed
PIS51932.1	28.5811655	30.8998753	32.8574945	28.60232768	29.01459742	29.57191755	0.15650899	0.954	-1.72	PIS51932.1	orf19.371	Ortholog of C. dubliniensis CD36 : Cd36_40110, C. parapsilosis CDC317 : CPAR2_402300, C. auris B8441 : B9J08_003539 and Candida tenuis NRRL Y-1498 : CANTEDRAFT_94507
PIS54564.1	26.9893525	27.3086234	26.8625464	25.45551328	25.89210259	24.65887176	0.000355684	0.388	-1.72	PIS54564.1	orf19.4153	Ortholog(s) have NEDD8 activating enzyme activity and role in protein neddylation
PIS55082.1	30.312226	30.3774269	30.5957304	29.17329025	28.58917516	28.38890625	4.69E-05	0.134	-1.71	PIS55082.1	ATM1	Member of MDR subfamily of ABC family; ortholog of S. cerevisiae ABC transporter, Atm1; induced in low iron; induced by nitric oxide independent of Yhb1
PIS58998.1	32.0866705	31.9368977	32.8005299	29.0801321	31.26089011	31.36377889	0.00759886	0.831	-1.71	PIS58998.1	RPS27	Putative ribosomal protein; repressed upon phagocytosis by murine macrophage; Spider biofilm repressed
PIS52212.1	29.4651038	29.5495346	30.242247	28.19842665	27.58912053	28.37943051	0.000108111	0.215	-1.7	PIS52212.1	orf19.1356	Ortholog(s) have thiosulfate sulfurtransferase activity and role in tRNA wobble position uridine thiolation, tRNA wobble uridine modification

PIS49583.1	30.3060238	30.1896864	30.0866653	28.67212466	28.26020098	28.55350125	1.42E-05	0.0634	-1.7	PIS49583.1	orf19.4127	Ortholog(s) have actin filament binding activity, role in actin cortical patch localization, actin cytoskeleton organization, actin filament bundle assembly, endocytosis and actin cortical patch localization
PIS58562.1	30.1504803	30.1559321	30.2322638	29.07469698	28.44968838	27.92479974	0.000184875	0.276	-1.7	PIS58562.1	orf19.7077	Putative ferric reductase; induced by Mac1 under copper starvation; Plc1-regulated; Rim101-repressed
PIS51898.1	30.0387648	30.6384642	30.8213372	28.88010345	28.6318366	28.91651596	0.002398749	0.713	-1.69	PIS51898.1	PTC2	Protein phosphatase of the Type 2C-related family (serine/threonine-specific); with protein kinase Ssn3p controls hyphal elongation through regulation of phosphorylation of transcription factor Ume6p and its stability
PIS56877.1	26.9249494	28.7467849	30.1811602	27.32731372	26.79930107	26.67487875	0.148885507	0.953	-1.68	PIS56877.1	HOS3	Histone deacetylase; similar to S. cerevisiae Hos3p; greater expression and longer mRNA in white cells, compared to opaque cells; has conserved deacetylation motif
PIS51851.1	31.0821588	31.3582767	31.4539429	28.76590618	30.31119274	29.76395793	0.043566804	0.922	-1.68	PIS51851.1	UGA11	Putative gamma-aminobutyrate (GABA) transaminase; macrophage-induced; overlaps orf19.854.1, which is a region annotated as a blocked reading frame; Spider biofilm induced
PIS50331.1	34.2431015	34.6441567	34.6036101	32.2514571	33.11488894	33.1236535	0.000165083	0.264	-1.67	PIS50331.1	RPL14	Ribosomal protein L14; promoter bound directly by Tbf1p; Hap43p-induced gene
PIS48230.1	31.1610031	31.9522118	32.9263297	29.8588855	30.50352958	30.70418339	0.002766978	0.731	-1.66	PIS48230.1	ACO1	Aconitase; induced in high iron; 2 upstream CCAAT motifs; amino acid starvation (3-AT), amphotericin B, phagocytosis, farnesol induced; Hap43, fluconazole-repressed; Gcn4-regulated; antigenic in infection; flow and Spider biofilm repressed

PIS50504.1	34.015317	34.5649704	35.3498554	31.71827523	33.5178466	33.72587111	0.017276956	0.883	-1.66	PIS50504.1	ADH1	Alcohol dehydrogenase; oxidizes ethanol to acetaldehyde; at yeast cell surface; immunogenic in humans/mice; complements S. cerevisiae adh1 adh2 adh3 mutant; fluconazole, farnesol-induced; flow model biofilm induced; Spider biofilm
PIS51634.1	30.3867145	30.2280788	30.697478	29.22282901	28.4054294	28.69175338	0.001486596	0.645	-1.66	PIS51634.1	CTN1	Carnitine acetyl transferase; required for growth on nonfermentable carbon sources, not for hyphal growth or virulence in mice; induced in macrophage; macrophage/pseudohyphal-repressed after 16 hr; rat catheter, Spider biofilm
PIS51189.1	32.0917557	32.6952502	33.3681821	30.55275478	31.30632423	31.30700939	0.005864031	0.809	-1.66	PIS51189.1	FAA4	Acyl CoA synthase involved in uptake of long-chain fatty acids and biofilm formation
PIS51392.1	22.580924	29.6508201	30.7570081	25.5781852	26.014777	26.41755703	0.363601346	0.968	-1.66	PIS51392.1	HMT1	Major type I protein arginine methyltransferase (PRMT); involved in asymmetric dimethylation of arginine residues; involved in nuclear export of Npl3p; Spider biofilm repressed
PIS55748.1	29.1870772	30.3113217	32.6213931	28.42867678	29.4570662	29.26674634	0.06090813	0.933	-1.66	PIS55748.1	orf19.4898	Putative protein of unknown function; induced by prostaglandins
PIS50540.1	25.6842805	25.7465724	26.1942744	23.6281966	23.81824561	25.18999382	0.008681534	0.841	-1.66	PIS50540.1	orf19.894	Ortholog(s) have guanyl-nucleotide exchange factor activity and role in retrograde transport, endosome to Golgi
PIS56909.1	28.5026019	28.403441	29.3979414	27.84266221	26.92172225	26.54475479	0.09749381	0.945	-1.66	PIS56909.1	PIKA	Phosphatidylinositol 4-kinase; controls levels of phosphatidylinositol-4-phosphate (PI(4)P) in the Golgi; non-sex gene located in MTLa mating-type-like locus; nonidentical gene encoding PI(4)P kinase, PIKALPHA, located in MTLalpha locus

PIS56874.1	30.2670943	30.7603231	30.7861583	29.17187282	29.01905003	28.6642318	0.001349299	0.63	-1.65	PIS56874.1	BFR1	Protein involved in the maintenance of normal ploidy; S. cerevisiae ortholog cofers Brefeldin A resistance; stationary phase enriched protein; Spider biofilm repressed
PIS51341.1	34.1460468	33.9656348	34.535652	32.57278236	32.50135738	32.620343	1.99E-05	0.0775	-1.65	PIS51341.1	MLS1	Malate synthase; glyoxylate cycle enzyme; no mammalian homolog; regulated upon white-opaque switch; phagocytosis, strong oxidative stress induced; stationary phase enriched; flow model biofilm repressed; rat catheter, Spider biofilm induced
PIS56572.1	25.0867812	27.2193006	28.0080672	24.78772902	25.61782576	24.96531952	0.018003982	0.885	-1.65	PIS56572.1	orf19.6247	Ortholog(s) have chromatin binding activity
PIS56731.1	31.2602323	31.5900058	32.1343283	29.17669991	30.36289912	30.5370348	0.005970312	0.811	-1.64	PIS56731.1	CCT8	Chaperonin-containing T-complex subunit; role in hyphal morphogenesis, particularly starvation-induced; essential; expression in S. cerevisiae inhibits Ras2-mediated pathways; CCT8 and TRP1 overlap; Spider biofilm repressed
PIS58815.1	29.3963918	30.4843783	30.5816207	27.24293039	29.17085325	29.13876176	0.015159854	0.876	-1.64	PIS58815.1	CDC42	Rho-type GTPase; required for budding and maintenance of hyphal growth; GGTase I geranylgeranylated; misexpression blocks hyphal growth, causes avirulence in mouse IV infection; shows actin-dependent localization to hyphal tip
PIS49542.1	29.0146394	29.4700027	29.1754028	27.01079923	27.99864072	27.72278907	0.001055095	0.59	-1.64	PIS49542.1	orf19.1849	Ortholog(s) have U3 snoRNA binding, rRNA binding activity and role in maturation of SSU-rRNA from tricistronic rRNA transcript (SSU-rRNA, 5.8S rRNA, LSU-rRNA), ribosomal small subunit biogenesis
PIS50541.1	25.3321653	27.5114737	27.8206283	25.76307948	25.12086101	24.92833737	0.020860147	0.893	-1.62	PIS50541.1	HOG1	MAP kinase of osmotic-, heavy metal-, and core stress response; role in regulation of response to stress; phosphorylated in response to H2O2 or NaCl; acts as repressor of START; mutant induces protective mouse immune response

PIS58184.1	27.6819269	28.4130064	28.78411	27.5828005	25.9688974	26.46911141	0.001472798	0.644	-1.62	PIS58184.1	orf19.3219	Ortholog of S. cerevisiae Sia1; involved in activation of the Pma1 plasma membrane H+-ATPase by glucose in S. cerevisiae; Spider biofilm induced
PIS51527.1	26.2458715	26.5885089	28.6278339	24.97438215	25.97635064	25.65476915	0.021520017	0.894	-1.62	PIS51527.1	SUI1	Putative translation initiation factor; flucytosine induced; genes encoding ribosomal subunits, translation factors, and tRNA synthetases are downregulated upon phagocytosis by murine macrophage
PIS55002.1	29.8084607	30.663285	30.0055924	28.34758158	28.54331782	28.7316282	0.001217429	0.613	-1.62	PIS55002.1	TMA19	Cell wall protein, ortholog of S. cerevisiae Tma19p (Ykl065cp)
PIS55741.1	27.8798631	28.1878796	27.7920265	26.25593359	27.1289614	25.6475858	0.005253537	0.799	-1.61	PIS55741.1	MEX67	Nuclear export protein; has NTF2-like domain; interacts with Mtr2p via the NTF2-like domain
PIS55803.1	28.2717032	28.1713049	29.9214227	26.74520416	27.07114332	27.70793074	0.015689522	0.878	-1.61	PIS55803.1	NPL4	Putative ubiquitin-binding protein; regulated by Gcn2p and Gcn4p
PIS51571.1	24.9322575	26.5893049	27.4877947	22.78401521	25.92812623	25.46143519	0.110274778	0.947	-1.61	PIS51571.1	orf19.6064	Phosphatidylinositol-3-phosphate-binding protein; component of the core autophagy machinery; gets recruited to the phagophore assembly site during onset of autophagy
PIS58801.1	32.3354935	32.8123166	32.2963562	31.14977232	31.13100616	30.34357347	0.000475024	0.443	-1.61	PIS58801.1	RPS17B	Ribosomal protein 17B; downregulated upon phagocytosis by murine macrophages; Hap43-induced; Spider biofilm repressed

PIS49484.1	26.0406566	25.9277066	24.2300956	25.07790611	24.19244305	22.1372865	0.067930787	0.936	-1.6	PIS49484.1	CBP1	Corticosteroid binding protein; transcription induced at late log-phase or upon adherence to polystyrene; not induced by corticosterone; contains a possible NAD/FAD binding region; regulated by Nrg1, Tup1; Spider biofilm induced
PIS56919.1	28.7754864	29.1134676	29.9156456	27.27114959	27.41258382	28.33389142	0.0063439	0.816	-1.6	PIS56919.1	DPP1	Putative diacylglycerol pyrophosphate phosphatase of diacylglycerol production for phospholipid biosynthesis; downregulation correlates with clinical development of fluconazole resistance
PIS51864.1	30.6438412	31.0733456	31.2402269	29.3230091	29.32705898	29.5183313	0.002472074	0.717	-1.6	PIS51864.1	MDH1-3	Predicted malate dehydrogenase; farnesol regulated; protein present in exponential and stationary growth phase yeast; Hap43p-repressed gene
PIS55719.1	25.7131021	25.5436309	26.6208268	25.89986485	22.26051567	24.92538976	0.065321151	0.934	-1.6	PIS55719.1	PBS2	MAPK kinase (MAPKK); role in osmotic and oxidative stress responses, oxidative stress adaptation; required for stress regulation of Hog1p localization and activity; functional homolog of S. cerevisiae Pbs2p
PIS48589.1	29.3257102	29.5559069	29.986023	27.61425753	28.4447171	27.99653239	0.000340794	0.381	-1.6	PIS48589.1	VMA5	Putative vacuolar H(+)-ATPase; plasma membrane localized; rat catheter biofilm repressed
PIS52387.1	29.8541534	30.3314544	29.8924135	28.80542667	28.43419736	28.07993818	0.001996916	0.688	-1.59	PIS52387.1	DOT5	Putative nuclear thiol peroxidase; alkaline downregulated; sumoylation target; Spider and flow model biofilm induced
PIS48755.1	29.273131	30.2684826	31.9344297	28.11074474	28.99687507	29.63064181	0.042580833	0.921	-1.58	PIS48755.1	ASC1	40S ribosomal subunit similar to G-beta subunits; glucose or N starvation induced filamentation; required for virulence in mice; snoRNA snR24 encoded in ASC1 intron; repressed in stationary phase; GlcNAc-induced; Spider biofilm repressed

PIS54992.1	31.4278958	31.725997	33.1674448	29.60124768	30.91848559	31.07023835	0.020911466	0.893	-1.58	PIS54992.1	CYP1	Peptidyl-prolyl cis-trans isomerase; cyclosporin A sensitive activity; soluble in hyphae; biofilm induced, macrophage-induced protein; downregulated upon treatment of biofilm with farnesol; present in exponential and stationary phase cells
PIS58161.1	31.352754	31.3091432	31.4006415	27.63166741	30.38684369	31.31450218	0.043927082	0.922	-1.58	PIS58161.1	LSC1	Putative succinate-CoA ligase subunit; induced by high iron; fluconazole-induced; protein present in exponential and stationary growth phase yeast cultures; Spider biofilm repressed
PIS48310.1	25.647524	27.7829083	26.0323347	24.86101401	25.06156331	24.8031726	0.005552944	0.804	-1.58	PIS48310.1	orf19.6403	Ortholog(s) have adenyl-nucleotide exchange factor activity and role in SRP-dependent cotranslational protein targeting to membrane, translocation
PIS51844.1	25.6265613	27.3753333	28.1609931	25.49476922	24.36883832	26.58306141	0.029747188	0.908	-1.57	PIS51844.1	orf19.1761	Predicted olichyl-diphosphooligosaccharide-protein glycotransferase; role in protein N-linked glycosylation; Spider biofilm repressed
PIS54993.1	28.901427	29.8495557	31.1764129	26.9574504	28.80985465	29.48660485	0.052719367	0.928	-1.56	PIS54993.1	AHP2	Putative thiol-specific peroxiredoxin; macrophage-downregulated gene
PIS58477.1	30.9876473	31.6346264	32.1878544	29.68811004	30.21724707	30.23477492	0.013293484	0.869	-1.56	PIS58477.1	LPD1	Putative dihydrolipoamide dehydrogenase; soluble in hyphae; antigenic in human oral infection and murine systemic infection; macrophage-induced protein; protein present in exponential and stationary phase yeast cultures; Hap43p-
PIS49766.1	23.2797071	24.5659255	22.4587237	24.21536268	22.34078473	19.06500146	0.164583741	0.955	-1.56	PIS49766.1	orf19.3606	Ortholog of S. cereviae Sna4 vacuolar outer membrane protein that plays a role in sensitivity to NA+; induced by Mnl1 under weak acid stress

PIS56911.1	27.7943671	27.9020157	29.7462799	25.78474005	27.66219898	27.30217483	0.038654478	0.918	-1.56	PIS56911.1	RCY1	Putative F-box protein involved in endocytic membrane traffic and/or recycling; fungal-specific (no human or murine homolog)
PIS51129.1	27.7095663	28.8033699	28.8661874	26.40173222	27.72215993	26.57012674	0.011679485	0.861	-1.56	PIS51129.1	UFE1	Protein interacting with Sec20p, possibly involved in retrograde transport between the Golgi and the endoplasmic reticulum; functional homolog of S. cerevisiae Ufe1p, which is an ER t-SNARE that mediates the retrograde traffic
PIS49784.1	28.7498413	28.9107195	29.4271637	27.00990283	27.73971618	27.68268426	0.071704413	0.937	-1.55	PIS49784.1	ABD1	SAM-dependent RNA methyltransferase; methylates mRNA 5' cap; binds phosphorylated RNA Pol II C-term domain peptide; does not bind mRNA TPase and mRNA GTase (Cet1,Cgt1); functional homolog of S. cerevisiae Abd1; rat catheter biofilm
PIS58525.1	31.6450519	31.6918851	31.2500933	30.08455917	30.15587027	29.70740453	0.000226888	0.311	-1.55	PIS58525.1	GSY1	Glycogen synthase (UDP glucose/starch glucosyltransferase); transcript repressed by yeast-hyphal switch, Efg1-regulated; strong oxidative stress induced; colony morphology-related regulation by Ssn6; stationary phase enriched
PIS58893.1	28.3084589	28.6050069	28.7145043	27.75341439	26.5502957	26.65985022	0.000346745	0.383	-1.55	PIS58893.1	orf19.2036	Predicted dihydrodiol dehydrogenase; ortholog of S. pombe SPAC513.06c; flow model and rat catheter biofilm repressed
PIS58260.1	30.1823617	30.8073828	30.9441449	28.67382228	29.12356117	29.4828678	0.049512205	0.926	-1.55	PIS58260.1	PRK1	Putative protein serine/threonine kinase; mutants sensitive to growth on hydrogen peroxide medium
PIS48564.1	27.6828783	28.3523697	29.9852567	27.26037593	27.43060569	26.66932328	0.018937496	0.888	-1.55	PIS48564.1	SMT3	SUMO, small ubiquitin-like protein; Smt3p-conjugated proteins localize to septation site and mother side of bud neck; C. albicans septins appear not to be Smt3p-modified, in contrast to S. cerevisiae septins

PIS54565.1	26.929007	28.5200127	29.3887714	26.48029132	27.06721194	26.65365379	0.036138187	0.916	-1.55	PIS54565.1	TRY3	RING-finger transcription factor; regulator of yeast form adherence; required for yeast cell adherence to silicone substrate; Spider biofilm induced
PIS54524.1	30.9284075	31.217081	32.7096264	30.42688989	29.57698195	30.24593112	0.021837801	0.895	-1.54	PIS54524.1	CAT1	Catalase; resistance to oxidative stress, neutrophils, peroxide; role in virulence; regulated by iron, ciclopirox, fluconazole, carbon source, pH, Rim101, Ssn6, Hog1, Hap43, Sfu1, Sef1, farnesol, core stress response; Spider biofilm induced
PIS56683.1	29.5011112	30.9067269	31.832145	28.70490249	29.57746654	29.32367271	0.136927939	0.952	-1.54	PIS56683.1	RPN10	Putative 19S regulatory particle of the 26S proteasome; macrophage/pseudohyphal-repressed; regulated by Gcn2 and Gcn4; Spider biofilm repressed
PIS54496.1	25.4140121	27.8762171	30.5142866	26.82554479	26.66051984	25.72502006	0.168558942	0.956	-1.53	PIS54496.1	orf19.352	Ortholog of C. dubliniensis CD36 : Cd36_83550, C. parapsilosis CDC317 : CPAR2_404510, C. auris B8441 : B9J08_002270, Debaryomyces hansenii CBS767 : DEHA2B13794g and Pichia stipitis Pignal : PICST_30078
PIS56792.1	24.1240308	24.530148	25.2103997	23.00642978	23.68464332	22.59760577	0.089350363	0.943	-1.53	PIS56792.1	orf19.443	Ortholog(s) have RNA polymerase III activity, role in tRNA transcription by RNA polymerase III, transcription initiation at RNA polymerase III promoter and RNA polymerase III complex, chromatin localization
PIS48568.1	26.1670516	27.7387093	27.1292119	24.3758475	25.80033148	26.26898352	0.011772852	0.861	-1.53	PIS48568.1	orf19.5274	Protein involved in regulation of ergosterol biosynthesis genes; null mutation causes increased sensitivity to azoles and abnormal hyphal growth
PIS58392.1	26.2279735	26.869402	26.6166099	23.9691109	25.25954446	25.88361159	0.009251164	0.845	-1.53	PIS58392.1	orf19.6400	Ortholog(s) have nuclear import signal receptor activity, role in inositol metabolic process, protein import into nucleus and nuclear envelope localization

PIS49540.1	28.6767663	29.7971519	30.1284433	28.01968219	27.96996724	28.01042764	0.036459138	0.916	-1.53	PIS49540.1	orf19.697	Ortholog of S. cerevisiae : YSC83, C. glabrata CBS138 : CAGL0A02134g, C. dubliniensis CD36 : Cd36_32160, C. parapsilosis CDC317 : CPAR2_203070 and C. auris B8441 : B9J08_004564
PIS51804.1	27.0695743	26.9494259	24.6098791	24.47426913	24.47244133	25.12057218	0.036556584	0.916	-1.52	PIS51804.1	orf19.3301	Putative ubiquitin ligase complex component; induced by heavy metal (cadmium) stress; Hog1-induced; transcript induced by Mnl1p under weak acid stress; flow model biofilm induced; Spider biofilm induced
PIS55840.1	27.2927345	26.1992442	27.2199948	27.13980788	25.96108992	23.0432635	0.127252022	0.95	-1.52	PIS55840.1	orf19.5026	C2H2 transcription factor; Spider biofilm induced; dominant-negative mutants showing hyper-invasive growth identified in clinical isolates
PIS51374.1	26.5182304	26.3561876	27.3194727	24.80284843	25.35241812	25.48872171	0.003513811	0.759	-1.52	PIS51374.1	SPO72	Protein described as similar to S. cerevisiae sporulation protein; ortholog of S. cerevisiae Atg2, an autophagic vesicle formation protein; up-regulation associated with azole resistance; Spider biofilm induced
PIS59011.1	26.4217135	27.173439	27.0507574	24.77493018	25.00720848	26.34254383	0.004848437	0.792	-1.51	PIS59011.1	orf19.1815	Ortholog of S. cerevisae/S. pombe Tif6; constituent of 66S pre-ribosomal particles; Spider biofilm induced
PIS58092.1	27.5756538	27.8894829	27.9059171	25.43376493	26.62526536	26.76766859	0.012098708	0.863	-1.51	PIS58092.1	orf19.1985	Has aminoglycoside phosphotransferase and protein kinase domains; rat catheter and flow model biofilm induced
PIS58829.1	25.6515932	27.1079334	27.413387	23.98558578	25.53024552	26.13843041	0.09036481	0.943	-1.51	PIS58829.1	orf19.3016	Ortholog of C. dubliniensis CD36 : Cd36_03040, C. parapsilosis CDC317 : CPAR2_108610, C. auris B8441 : B9J08_000283, Debaryomyces hansenii CBS767 : DEHA2G16500g and Pichia stipitis Pignal : PICST_32835

PIS55602.1	21.9416043	23.282395	22.980694	20.08722069	22.24562949	21.35158646	0.037058877	0.917	-1.51	PIS55602.1	orf19.4533	Ortholog(s) have RNA binding activity and role in rRNA processing
PIS51145.1	28.5907457	30.0558229	29.9477232	27.78739143	27.30436569	28.96099955	0.046430877	0.924	-1.51	PIS51145.1	RPL30	Ribosomal 60S subunit protein; pre-rRNA processing; pre-mRNA alternatively spliced to productive/unproductive transcripts; temp-regulated splicing; colony morphology-related regulation by Ssn6, Tup1, Nrg1 regulated; Spider
PIS51197.1	26.7859787	26.5350427	26.0253366	24.52168518	25.69144354	24.62098702	0.054023423	0.929	-1.5	PIS51197.1	ADE8	Putative phosphoribosylglycinamide formyl-transferase, enzyme of amino acid biosynthesis pathway; upregulated in biofilm; S. cerevisiae ortholog is Gcn4p regulated; protein enriched in stationary phase yeast-form cultures
PIS49545.1	27.2409963	26.6706712	25.0064559	25.33110495	24.3828548	24.7166672	0.03214262	0.911	-1.5	PIS49545.1	GIM5	Putative heterohexameric cochaperone prefoldin complex subunit; macrophage/pseudohyphal-repressed gene and macrophage-induced protein
PIS58776.1	28.6598107	28.9907249	28.834797	27.46086736	27.61851906	26.89232939	0.003593403	0.761	-1.5	PIS58776.1	LEU2	Isopropyl malate dehydrogenase; leucine biosynthesis; induced by human whole blood or PMNs; protein level decreases in stationary phase; GlcNAc-induced protein; flow model biofilm repressed
PIS48587.1	30.3135876	30.9003631	30.5457225	29.02873422	29.32384306	28.92035106	0.003509067	0.758	-1.5	PIS48587.1	orf19.2168.3	Ortholog(s) have role in ER-dependent peroxisome organization, endoplasmic reticulum inheritance, endoplasmic reticulum organization and endoplasmic reticulum tubular network membrane organization, more
PIS51606.1	30.7517708	30.9020886	31.0231547	30.05402752	29.23482761	28.88710208	0.032409056	0.912	-1.5	PIS51606.1	orf19.4123	Putative THO complex subunit; possibly an essential gene, disruptants not obtained by UAU1 method; protein newly produced during adaptation to the serum

PIS52433.1	29.8069117	30.3090678	29.6105807	28.50560171	28.21707101	28.48914625	0.109150356	0.947	-1.5	PIS52433.1	orf19.4846	GlcNAc-induced protein
PIS51194.1	27.11324	27.0667425	29.6456449	28.19885715	25.88827851	25.25602302	0.08439629	0.941	-1.49	PIS51194.1	AHA1	Putative Hsp90p co-chaperone; Hap43-repressed; heavy metal (cadmium) stress-induced; oxidative stress-induced via Cap1; rat catheter biofilm induced; flow model biofilm repressed
PIS51531.1	30.6624168	30.9500608	30.8798127	29.1321059	29.462763	29.43946498	9.32E-05	0.2	-1.49	PIS51531.1	NDH51	Nicotinamide adenine dinucleotide dehydrogenase complex I subunit of the mitochondrial electron transport chain; required for wild-type filamentous growth; alkaline repressed; Hap43-repressed; Spider biofilm repressed
PIS52448.1	24.3673685	26.1643441	27.571781	24.52930881	24.56724448	24.5405014	0.05632634	0.93	-1.49	PIS52448.1	orf19.3215	Putative plasma membrane protein; in S. cerevisiae it is localized to the cell bud and mating projection membrane; repressed by alpha pheromone in SpiderM medium
PIS48303.1	31.7252043	32.100689	32.2619006	30.95561036	30.32704423	30.33610556	0.001009817	0.582	-1.49	PIS48303.1	orf19.3335	Plasma membrane protein of unknown function; colony morphology-related gene regulation by Ssn6; repressed by nitric oxide
PIS56906.1	27.5750187	28.5197513	28.0345713	27.19440292	26.39450744	26.10079794	0.028592246	0.907	-1.48	PIS56906.1	MAS2	Putative processing peptidase, catalytic (alpha) subunit; protein level decreases in stationary phase cultures
PIS58708.1	27.5169275	27.5785872	28.4850086	24.80984566	27.10409996	27.22868612	0.029169399	0.907	-1.48	PIS58708.1	orf19.1485	Mitochondrial ribosomal protein of the large subunit; rat catheter biofilm induced

PIS58168.1	27.2777046	27.3983107	25.953462	25.65909955	25.8712826	24.64725676	0.033732396	0.913	-1.48	PIS58168.1	orf19.457	Ortholog(s) have K63-linked deubiquitinase activity, cysteine-type deubiquitinase activity
PIS51403.1	27.5911177	26.7964129	26.8400615	26.45871615	25.46557822	24.85124438	0.012817701	0.867	-1.48	PIS51403.1	YKU80	Yku70p-Yku80p Ku complex subunit involved in nonhomologous end joining during double-strand break repair repair; Hap43-repressed gene; flow model biofilm induced
PIS52224.1	24.2473738	24.3745122	25.4197889	23.77776986	23.15836739	22.68999568	0.004496174	0.784	-1.47	PIS52224.1	DAC1	N-acetylglucosamine-6-phosphate (GlcNAcP) deacetylase; N-acetylglucosamine utilization; required for wild-type hyphal growth and virulence in mouse systemic infection; gene and protein are GlcNAc-induced; Spider biofilm induced
PIS58784.1	28.8884974	31.4532177	31.8963585	29.06693043	28.83152083	29.9167024	0.032422785	0.912	-1.47	PIS58784.1	GUP1	Putative O-acyltransferase with a role in glycerol uptake; functionally complements growth of S. cerevisiae gup1 mutant under salt stress; required for normal ergosterol distribution, hyphal growth, biofilm formation
PIS51651.1	32.8858113	33.3231277	33.7901863	31.03508967	32.30473844	32.32286481	0.029414857	0.908	-1.45	PIS51651.1	RPS16A	Putative 40S ribosomal subunit; macrophage/pseudohyphal-induced after 16 h; Spider biofilm repressed
PIS51415.1	27.5366142	27.6535683	27.7606208	25.51337227	26.06209731	27.03906129	0.012740586	0.866	-1.45	PIS51415.1	VPS2	Ortholog(s) have role in ATP export, intralumenal vesicle formation, late endosome to vacuole transport and protein retention in Golgi apparatus, more
PIS52103.1	27.5757639	27.9777693	29.1119448	27.03010367	26.46595879	26.85097497	0.01684979	0.882	-1.44	PIS52103.1	ALG2	Putative mannosyltransferase involved in cell wall mannan biosynthesis; transcription is elevated in chk1, nik1, and sln1 homozygous null mutants

PIS55064.1	26.878065	28.10336	28.1470904	24.16070812	26.34678489	28.29557044	0.078322569	0.939	-1.44	PIS55064.1	CGR1	Negative regulator of yeast-form growth; HSP70 family member; induced by growth cessation at yeast-hyphal transition or in planktonic growth; physically interacts with Msi3p; similar to rat anti-aging gene, SMP30, stationary phase enriched [illegible]
PIS58877.1	27.0521164	27.9000695	27.9949817	27.69109818	26.10242122	24.84506689	0.020549394	0.892	-1.44	PIS58877.1	orf19.2304	Protein similar to S. cerevisiae Gvp36p; transposon mutation affects filamentous growth
PIS58765.1	30.6874606	31.0390389	30.5979203	27.96590892	29.89610731	30.12988905	0.02162453	0.894	-1.44	PIS58765.1	orf19.2686	Ortholog(s) have carboxypeptidase activity, role in nitrogen compound metabolic process, proteolysis involved in protein catabolic process and fungal-type vacuole lumen localization
PIS58344.1	26.7546098	26.7093063	25.6272739	25.03349768	25.44044837	24.30543059	0.009107842	0.844	-1.44	PIS58344.1	orf19.3076	Ortholog(s) have role in vesicle-mediated transport and Golgi membrane localization
PIS52151.1	24.4111727	26.0225158	26.6929423	24.76201761	22.43023267	25.62854471	0.407403973	0.969	-1.44	PIS52151.1	orf19.4007	Ortholog(s) have protein-lysine N-methyltransferase activity, role in peptidyl-lysine monomethylation, peptidyl-lysine trimethylation and nucleolus localization
PIS52145.1	29.7734815	30.3167523	29.1672861	29.68303118	27.92030019	27.34755274	0.028102853	0.906	-1.44	PIS52145.1	RPP2B	Conserved acidic ribosomal protein; possibly involved in regulation of translation elongation; interacts with Rpp1A; 1 of 4 similar C. albicans proteins (Rpp1A, Rpp1B, Rpp2A, Rpp2B); macrophage/pseudohyphal-induced; Spider biofilm repressed [illegible]
PIS51795.1	25.58874	27.3273707	27.7659762	24.27628775	25.25315274	26.86962363	0.095037617	0.944	-1.43	PIS51795.1	MMD1	Mitochondrial protein; possibly required for transamination of isoleucine; macrophage-downregulated protein abundance; rat catheter and Spider biofilm repressed

PIS50584.1	27.7572298	27.9142305	28.6069251	25.69163848	27.045947	27.24882116	0.020900743	0.893	-1.43	PIS50584.1	PAM18	Predicted component of the presequence translocase-associated import motor (PAM complex) involved in protein import into mitochondrial matrix; rat catheter biofilm induced
PIS51214.1	31.2000723	31.0156916	31.7442504	30.28358908	29.80356885	29.59406808	0.004396218	0.782	-1.43	PIS51214.1	TPK2	cAMP-dependent protein kinase (PKA) catalytic subunit; isoform of Tpk1; involved in regulation of filamentation, phenotypic switching and mating; needed for epithelial cell damage, engulfment and oral virulence in mice
PIS51911.1	31.5522866	31.8721225	31.582036	29.93996086	30.53569649	30.27429789	0.00896603	0.843	-1.42	PIS51911.1	CAR2	Ornithine aminotransferase; arginine metabolism; alkaline induced; mutant sensitivite to toxic ergosterol analog, to amphotericin B; exponential and stationary phase yeast; flow model biofilm induced; rat catheter, Spider biofilm repressed
PIS48819.1	24.1981321	27.5507473	28.2468859	23.79160981	25.12874227	26.80385581	0.13934179	0.952	-1.42	PIS48819.1	ERG12	Ortholog(s) have mevalonate kinase activity and role in ergosterol biosynthetic process, farnesyl diphosphate biosynthetic process, mevalonate pathway, isopentenyl diphosphate biosynthetic process, mevalonate pathway
PIS48765.1	28.6823231	28.8650017	29.5908438	27.23666335	27.80721522	27.81958965	0.001958485	0.686	-1.42	PIS48765.1	orf19.6464	Protein of unknown function; induced upon adherence to polystyrene; oxidative stress-induced via Cap1
PIS51535.1	30.6578893	30.3420584	31.1641119	29.2617229	29.11114591	29.52729525	0.000352969	0.387	-1.42	PIS51535.1	QCR8	Putative ubiquinol cytochrome c reductase; macrophage and pseudohyphal-induced protein; colony morphology-related gene regulation by Ssn6; Hap43-repressed; Spider biofilm repressed
PIS56738.1	34.1755325	34.2878236	34.9436837	33.32600222	32.89004019	32.93201678	0.006601833	0.819	-1.42	PIS56738.1	RPL16A	Ribosomal protein; transposon mutation affects filamentous growth; repressed upon phagocytosis by murine macrophages; Hap43-induced; Spider biofilm repressed

PIS58221.1	32.3232258	34.0253042	33.2814699	31.93770539	31.70379307	31.72960105	0.004917133	0.793	-1.42	PIS58221.1	RPP0	Putative ribosomal protein; antigenic in mouse; repressed upon phagocytosis by murine macrophage; induced by Tbf1; overlaps orf19.7014; Spider biofilm repressed
PIS55766.1	27.1478254	27.8858842	27.7231962	27.06101436	25.72390203	25.70409533	0.025828168	0.902	-1.42	PIS55766.1	TIF11	Translation initiation factor eIF1a; possibly transcriptionally regulated upon hyphal formation; genes encoding ribosomal subunits, translation factors, and tRNA synthetases are downregulated upon phagocytosis by murine macrophage
PIS58286.1	32.4625515	32.299795	32.800688	31.64087128	30.91913997	30.78736907	0.007730074	0.832	-1.41	PIS58286.1	EHD3	Predicted 3-hydroxyisobutyryl-CoA hydrolase; mitochondrially localized; Spider biofilm induced
PIS55039.1	25.9436399	27.4061941	27.4035042	26.11794889	26.09247699	24.32155848	0.096086634	0.944	-1.41	PIS55039.1	orf19.2076	Protein of unknown function; S. pombe ortholog SPAC7D4.05 encodes a predicted hydrolase; Hap43-repressed; Spider biofilm induced
PIS48228.1	29.5431703	29.7126676	29.9091552	25.28366935	29.68936298	29.94808851	0.148389715	0.953	-1.41	PIS48228.1	orf19.6435	Highly conserved subunit of mitochondrial pyruvate carrier; Hap43-repressed; Spider biofilm repressed
PIS58246.1	25.5907092	24.7199339	25.0624766	25.02312611	22.60704632	23.53595686	0.045285631	0.923	-1.4	PIS58246.1	ECM39	Putative mannosyltransferase similar to S. cerevisiae Ecm39p, which has a role in Calcofluor white resistance; predicted Kex2p substrate; has HKEXRF motif
PIS48702.1	29.7727849	30.0809345	29.7772966	28.78357781	28.55222058	28.09061102	0.121154988	0.949	-1.4	PIS48702.1	orf19.137	Putative transferase involved in phospholipid biosynthesis; induced by alpha pheromone in SpiderM medium

PIS52470.1	28.6617641	25.1082817	24.4199889	24.54833118	23.69407273	25.75489015	0.259110296	0.963	-1.4	PIS52470.1	orf19.2826	Ortholog of C. dubliniensis CD36 : Cd36_27680, C. parapsilosis CDC317 : CPAR2_801140, C. auris B8441 : B9J08_004087 and Candida tenuis NRRL Y-1498 : CANTEDRAFT_129982
PIS48671.1	28.1423613	28.8723565	28.5392913	27.09496134	27.55295272	26.71017317	0.000602962	0.489	-1.4	PIS48671.1	orf19.3394	Predicted membrane protein; induced by alpha pheromone in SpiderM medium
PIS48664.1	27.5569506	27.9673291	29.6088058	25.92068117	27.12541682	27.89199282	0.024965249	0.901	-1.4	PIS48664.1	orf19.501	Ortholog(s) have rRNA (cytosine-C5-)-methyltransferase activity
PIS56754.1	28.1824765	27.4482679	26.8587368	25.94988671	26.01115225	26.34210397	0.00274461	0.73	-1.4	PIS56754.1	orf19.5536	Ortholog of S. cerevisiae : AMD2, C. dubliniensis CD36 : Cd36_62950, C. parapsilosis CDC317 : CPAR2_301880, Candida tenuis NRRL Y-1498 : CANTEDRAFT_123468 and Debaryomyces hansenii CBS767 : [illegible]
PIS54901.1	25.2546133	29.0145346	29.0596874	25.0710255	26.61343703	27.45050379	0.138064544	0.952	-1.4	PIS54901.1	orf19.927	Ortholog(s) have tRNA binding activity, role in DNA recombination, positive regulation of transcription by RNA polymerase II, telomere maintenance, telomere maintenance via recombination and EKC/KEOPS complex localization [illegible]
PIS55816.1	33.4290418	33.5545924	33.6814627	32.25397335	32.40054066	31.810976	0.001021818	0.584	-1.4	PIS55816.1	RPL10A	Predicted ribosomal protein; downregulated upon phagocytosis by murine macrophages; Hap43-induced; Spider biofilm repressed
PIS49616.1	30.3547148	30.0147815	30.6284104	29.30863342	28.47729984	29.01155391	0.013706317	0.871	-1.4	PIS49616.1	SIM1	Adhesin-like protein; involved in cell wall maintenance, redundant with Sun41; possibly secreted; macrophage-repressed; repressed by Rim101, Cyr1, Ras1; Spider biofilm induced

PIS54831.1	30.8125704	30.0271721	30.2463469	29.13508983	29.38003601	28.39580052	0.010723776	0.855	-1.39	PIS54831.1	ADE6	5-Phosphoribosylformyl glycinamidine synthetase; adenine biosynthesis; not induced in GCN response, in contrast to S. cerevisiae Ade6; protein in stationary phase yeast-form cultures; flow model and rat catheter biofilm repressed
PIS51761.1	24.8576212	24.8353617	26.2782816	24.62380522	23.48627005	23.68262998	0.02821154	0.906	-1.39	PIS51761.1	orf19.1057	Ortholog of C. dubliniensis CD36 : Cd36_03980, C. parapsilosis CDC317 : CPAR2_107050, C. auris B8441 : B9J08_003358 and Candida tenuis NRRL Y-1498 : CANTEDRAFT_113999
PIS55715.1	27.2067877	28.8255739	29.3322774	26.43623582	27.3220423	27.4398596	0.180382213	0.957	-1.39	PIS55715.1	orf19.2439.1	Protein whose ortholog(s) have ubiquinol-cytochrome-c reductase activity and roles in aerobic respiration and mitochondrial electron transport; null mutant is viable and shows normal vegetative growth on various carbon sources
PIS50421.1	25.786037	24.964065	27.5721586	21.18536014	26.38403217	26.60166951	0.264127668	0.963	-1.38	PIS50421.1	HSE1	ESCRT-0 complex subunit; SH3-domain-containing protein
PIS58706.1	24.7775654	26.0826693	26.5725393	23.83216846	24.07302019	25.38633787	0.036629259	0.916	-1.38	PIS58706.1	orf19.1483	Ortholog(s) have copper ion binding activity, role in mitochondrial cytochrome c oxidase assembly and mitochondrial intermembrane space localization
PIS51734.1	29.2920155	30.5233172	30.7436371	28.4162519	29.05142785	28.96534422	0.006723546	0.821	-1.38	PIS51734.1	orf19.2019	Ortholog(s) have structural constituent of ribosome activity, role in mitochondrial translation and mitochondrial large ribosomal subunit localization
PIS58870.1	24.8537088	25.4499727	25.7348562	22.9426695	24.35093034	24.59748289	0.034973943	0.914	-1.38	PIS58870.1	orf19.4748	Putative U2B'' component of the U2 snRNP, involved in splicing; contains an RNA recognition motif (RRM); ortholog of S. cerevisiae MSL1; Hap43p-induced gene

PIS48242.1	30.2670833	29.3272457	29.925706	28.35116442	28.68669782	28.35501947	0.00191024	0.682	-1.38	PIS48242.1	TOM40	Protein involved in mitochondrial protein import; Spider biofilm repressed
PIS54476.1	29.2200408	28.9338908	29.7082837	27.57969865	28.15804279	28.00915977	0.003092472	0.744	-1.37	PIS54476.1	HGT12	Glucose, fructose, mannose transporter; major facilitator superfamily; role in macrophage-induced hyphal growth; detected at germ tube plasma membrane by mass spectrometry; Snf3p-induced; 12 probable transmembrane segments
PIS56559.1	24.255886	27.5937843	26.1919843	24.69430807	24.5563777	24.66977034	0.108449006	0.947	-1.37	PIS56559.1	MSW1	Protein similar to S. cerevisiae Msw1p, which is mitochondrial tryptophanyl-tRNA synthetase; Hap43p-repressed gene; likely to be essential for growth, based on an insertional mutagenesis strategy
PIS58066.1	28.1495389	28.3759251	28.9108326	27.88445247	26.70125931	26.72890936	0.002759524	0.73	-1.37	PIS58066.1	orf19.3459	Putative serine/threonine/tyrosine (dual-specificity) kinase; disruptants not obtained by UAU1 method
PIS56833.1	24.9595673	27.9559477	27.8110689	25.5405505	25.63042461	25.43781984	0.147202864	0.953	-1.37	PIS56833.1	orf19.5297	Ortholog(s) have RNA polymerase II general transcription initiation factor activity, phosphatidylinositol-3-phosphate binding, phosphatidylinositol-5-phosphate binding activity
PIS58080.1	27.6480832	28.0418308	28.8051135	26.32937031	27.12596994	26.93861583	0.011088945	0.858	-1.37	PIS58080.1	orf19.6132	Has domain(s) with predicted role in cristae formation and MICOS complex, mitochondrial crista junction localization
PIS48187.1	24.9175355	27.1078139	24.4434415	23.28551566	24.09851418	24.9807268	0.067870086	0.936	-1.37	PIS48187.1	orf19.6506	Ortholog(s) have role in negative regulation of antisense RNA transcription, positive regulation of transcription by RNA polymerase II, regulation of DNA-templated DNA replication initiation, transcription elongation by RNA polymerase II

PIS55496.1	27.2274436	27.0714131	26.5934742	25.45592701	25.49933003	25.84427464	0.005514721	0.804	-1.36	PIS55496.1	orf19.4713	Ortholog of C. dubliniensis CD36 : Cd36_07300, C. parapsilosis CDC317 : CPAR2_208300, C. auris B8441 : B9J08_001596 and Candida tenuis NRRL Y-1498 : CANTEDRAFT_95879
PIS58973.1	36.486806	36.102015	35.6022947	34.32712383	35.10034501	34.68899381	0.000757457	0.531	-1.36	PIS58973.1	PET9	Mitochondrial ADP/ATP carrier protein involved in ATP biosynthesis; possible lipid raft component; 3 predicted transmembrane helices; flucytosine induced; ketoconazole-induced; downregulated by Efg1p
PIS48728.1	30.9560603	30.869785	31.8330208	30.2171967	29.30669456	30.06549488	0.001612676	0.657	-1.36	PIS48728.1	ROA1	Putative PDR-subfamily ABC transporter involved in sensitivity to azoles; Spider biofilm induced
PIS58636.1	31.6581272	32.2537157	32.6758835	30.43132954	31.07426574	31.03819033	0.004863392	0.792	-1.35	PIS58636.1	GSP1	Small RAN G-protein; essential; no prenylation predicted; can rescue S. cerevisiae gsp1 viability; macrophage/pseudohyphal-induced; transcript not regulated by white-opaque, yeast-hypha switching; GlcNAc-induced; Spider biofilm
PIS58897.1	27.509806	26.2712381	27.2983305	27.08131354	26.30774629	23.65052456	0.187688739	0.958	-1.35	PIS58897.1	MLC1	Microtubule-dependent localized protein; at Spitzenkorper and cytokinetic ring in hyphae; cell-cycle dependent localization to tip polarisome, bud neck in yeast and pseudohyphae; sumoylation target; rat catheter, Spider biofilm repressed
PIS55801.1	32.1730327	32.4918072	33.2810602	30.7361161	31.50942665	31.65874022	0.015826987	0.879	-1.35	PIS55801.1	MNT1	Alpha-1,2-mannosyl transferase; predicted type II Golgi membrane protein; adds 2nd mannose during cell-wall mannoprotein biosynthesis; required for wild-type virulence and adherence to epithelial cells; Hap43-induced
PIS48144.1	27.6739239	27.2080815	28.058603	25.22167903	26.97487842	26.70561955	0.00776979	0.832	-1.35	PIS48144.1	orf19.1890	Ortholog(s) have acylglycerol lipase activity, triglyceride lipase activity and role in medium-chain fatty acid biosynthetic process, triglyceride metabolic process

PIS51225.1	27.9296965	28.3105679	27.2480077	23.65743284	27.94567739	27.82568268	0.139699082	0.952	-1.35	PIS51225.1	orf19.2263	Ortholog of C. dubliniensis CD36 : Cd36_21360, C. parapsilosis CDC317 : CPAR2_406560, C. auris B8441 : B9J08_002799 and Candida tenuis NRRL Y-1498 : CANTEDRAFT_103408
PIS49487.1	31.0229456	31.5946874	32.2599107	29.94174508	30.11273748	30.77765513	0.008066353	0.835	-1.35	PIS49487.1	PHO88	Protein with a role in phosphate transport; biofilm-regulated expression; amphotericin B repressed
PIS52003.1	32.5064304	32.7647796	33.7020991	30.69793662	32.16225231	32.10465244	0.022176923	0.896	-1.34	PIS52003.1	ADH2	Alcohol dehydrogenase; soluble in hyphae; expression regulated by white-opaque switching; regulated by Ssn6; indued by Mnl1 in weak acid stress; protein enriched in stationary phase yeast cultures; Spider biofilm induced
PIS59004.1	22.5042825	22.5314712	24.03287	20.77373197	22.52630229	21.76039563	0.179039598	0.957	-1.34	PIS59004.1	MNL1	Transcription factor; induces transcripts of stress response genes via SLE (STRE-like) elements; required for adaptation to weak acid stress; activates a subset of the genes that are repressed by Nrg1
PIS58746.1	26.3532893	27.0326682	25.9374956	23.34635231	26.22853313	25.7171041	0.082002458	0.94	-1.34	PIS58746.1	NUP82	Linker nucleoporin of the nuclear pore complex; role in mRNA andexport from nucleus, protein import into nucleus, ribosomal large subunit export from nucleus, ribosomal small subunit export from nucleus; rat catheter biofilm repressed
PIS51890.1	24.5192907	24.2342845	22.302521	23.90540445	23.08223836	20.0490798	0.217648758	0.96	-1.34	PIS51890.1	orf19.2401	Ortholog(s) have protein-phosphatidylethanolamide deconjugating activity
PIS54501.1	25.0433268	26.409138	27.2820244	24.54827151	25.29081933	24.86064787	0.036385132	0.916	-1.34	PIS54501.1	orf19.5963	Putative prenyltransferase; essential gene in S. cerevisiae; Spider biofilm induced

PIS52000.1	30.0341027	30.9408368	31.0516962	29.17877306	29.30333692	29.50972586	0.002247881	0.704	-1.34	PIS52000.1	SDS24	Protein similar to S. cerevisiae Sds24 involved in cell separation during budding; transcript regulated by Mig1 and Tup1; fluconazole-induced; flow model biofilm induced
PIS49555.1	36.7459965	36.9783993	37.5097029	35.68568793	35.75479952	35.78033543	0.000184748	0.276	-1.34	PIS49555.1	TEF2	Translation elongation factor 1-alpha; genes encoding ribosomal subunits, translation factors, and tRNA synthetases are downregulated upon phagocytosis by murine macrophage
PIS50557.1	29.9694853	30.6580749	31.6868951	29.04347862	29.41084307	29.8332535	0.031282959	0.91	-1.34	PIS50557.1	TIF5	Putative translation initiation factor; repressed upon phagocytosis by murine macrophage; Spider biofilm repressed
PIS55751.1	32.2681325	31.8871318	31.2685829	30.22264918	30.74596002	30.4701594	0.001676117	0.663	-1.33	PIS55751.1	CTR1	Copper transporter; transcribed in low copper; induced Mac1, Tye7, macrophage interaction, alkaline pH via Rim101; 17-beta-estradiol repressed; complements S. cerevisiae ctr1 ctr3 copper transport mutant; flow model/Spider biofilm induced
PIS58412.1	27.6033784	28.7584808	28.5179842	26.72107824	26.88054637	27.27412228	0.057157462	0.931	-1.33	PIS58412.1	orf19.2917	Putative GTPase; heterozygous null mutant exhibits resistance to parnafungin in the C. albicans fitness test; Hap43p-induced gene
PIS51518.1	24.0060012	26.8980173	26.3254846	24.47087772	23.98784238	24.79676488	0.07260572	0.937	-1.32	PIS51518.1	orf19.2638	Protein of unknown function; Spider biofilm induced
PIS50494.1	27.8823895	27.7619246	27.2176593	26.74336984	27.82439328	24.32860321	0.09504963	0.944	-1.32	PIS50494.1	orf19.3980	Ortholog(s) have ATPase activity, role in cytoplasmic translation, regulation of translation, rescue of stalled ribosome, ribosome disassembly and cytosolic ribosome localization

PIS49483.1	27.4003606	26.6117687	27.1613746	24.89876472	27.12651408	25.17558623	0.028011965	0.906	-1.32	PIS49483.1	orf19.7322	Protein of unknown function; S. cerevisae ortholog Ypl225w interacts with ribosomes; rat catheter biofilm induced
PIS56639.1	30.6285043	30.9587292	31.0467266	29.01090179	29.86964453	29.77846873	0.001132628	0.601	-1.32	PIS56639.1	SEC4	Small GTPase of Rab family; role in post-Golgi secretion; possible C-terminal palmitoylation; downregulated on adherence to polystyrene; localizes to the Spitzenkorper during hyphal growth; functional homolog of S. cerevisiae Sec4p
PIS51733.1	32.4448931	32.9133478	32.6220621	32.06208443	31.31491278	30.65939311	0.106451998	0.947	-1.31	PIS51733.1	MSF1	Putative phenylalanine-tRNA ligase; protein level decreases in stationary phase cultures; Hap43p-repressed gene
PIS56748.1	26.6529285	28.0093033	28.4887693	27.11352246	26.84046077	25.26543684	0.035548234	0.915	-1.31	PIS56748.1	NPL6	Component of the RSC chromatin remodeling complex; Hap43-induced; Spider biofilm repressed
PIS51052.1	29.8578674	29.7589586	29.285914	28.09404479	28.57311151	28.34964523	0.003443594	0.756	-1.3	PIS51052.1	MRPL33	Putative mitochondrial ribosomal protein of the large subunit; Ssr1-repressed; rat catheter biofilm induced
PIS58739.1	31.1156483	31.2041401	29.3529809	30.21105835	29.74106381	27.8301758	0.062671396	0.933	-1.3	PIS58739.1	orf19.1687	Ortholog of S. cerevisiae Prp43, an RNA helicase in the DEAH-box family that functions in both RNA polymerase I and polymerase II transcript metabolism; Hap43-induced gene
PIS58886.1	29.3905676	28.9213723	29.2488272	26.49556867	28.89222104	28.26011147	0.024875054	0.901	-1.3	PIS58886.1	orf19.2863	Ortholog of C. dubliniensis CD36 : Cd36_28090, C. parapsilosis CDC317 : CPAR2_802560, C. auris B8441 : B9J08_000343 and Candida tenuis NRRL Y-1498 : CANTEDRAFT_104949

PIS50626.1	30.6256037	31.0277422	30.6353305	29.0837833	29.62416234	29.67397699	0.000519762	0.461	-1.3	PIS50626.1	orf19.4639	Protein present in exponential and stationary growth phase yeast cultures
PIS51130.1	30.6382033	30.6548416	31.0381982	30.11322219	29.39162003	28.9384194	0.007362635	0.828	-1.3	PIS51130.1	orf19.7140	Putative catechol o-methyltransferase; stationary phase enriched protein; transcription upregulated in clinical isolates from HIV+ patients with oral candidiasis; Spider biofilm repressed
PIS48470.1	32.868048	32.9974198	33.0984109	30.91313601	32.02182686	32.15927366	0.003680322	0.764	-1.29	PIS48470.1	FBP1	Fructose-1,6-bisphosphatase; key gluconeogenesis enzyme; regulated by Efg1, Ssn6; induced by phagocytosis; effects switch from glycolysis to gluconeogenesis in macrophage; rat flow model biofilm induced; overlaps orf19.6179
PIS51093.1	33.0562092	32.3653382	32.1441124	31.8585048	31.1538265	30.6729322	0.278699491	0.964	-1.29	PIS51093.1	orf19.4686	Ortholog of S. cerevisiae : ESL1, C. glabrata CBS138 : CAGL0H06611g, C. dubliniensis CD36 : Cd36_41030, C. parapsilosis CDC317 : CPAR2_401340 and C. auris B8441 : B9J08_002665
PIS49666.1	25.0151728	26.4726807	26.83767	21.50950088	26.41000222	26.54009657	0.364058486	0.968	-1.29	PIS49666.1	SEC9	t-SNARE protein required for secretory vesicle-membrane fusion
PIS58316.1	28.0191781	28.977779	29.1603121	26.96406278	27.60419899	27.74789863	0.002902238	0.737	-1.28	PIS58316.1	ARC15	Putative ARP2/3 complex subunit; mutation confers hypersensitivity to cytochalasin D
PIS58913.1	30.4493549	30.2868665	29.5464504	28.43501499	28.77904262	29.22568513	0.052233132	0.928	-1.28	PIS58913.1	ARD	D-arabitol dehydrogenase, NAD-dependent (ArDH); enzyme of D-arabitol and D-arabinose catabolism; D-arabitol is a marker for active infection in humans; rat catheter and Spider biofilm induced

PIS50595.1	25.5777603	26.5608105	26.2240098	27.47638942	21.70696848	25.34543939	0.264945767	0.963	-1.28	PIS50595.1	orf19.4164	Ortholog(s) have role in ribosome disassembly
PIS48354.1	27.1191824	27.6103316	27.2139592	25.25722959	26.68364527	26.17307685	0.009496758	0.847	-1.28	PIS48354.1	YME1	Ortholog(s) have ATP-dependent peptidase activity
PIS56669.1	31.6950235	31.7176181	32.3877306	30.26243832	30.90674646	30.82376485	0.001273867	0.621	-1.27	PIS56669.1	FUM12	Putative fumarate hydratase; enzyme of citric acid cycle; fluconazole, Efg1 repressed; induced in high iron; protein present in exponential and stationary growth phase
PIS51088.1	29.4984436	30.7307638	32.6079691	30.43046119	29.77250174	28.81498207	0.127372925	0.95	-1.27	PIS51088.1	HGT17	Putative MFS family glucose transporter; 20 members in C. albicans; 12 probable membrane-spanning segments; induced at low (0.2%, compared to 2%) glucose in rich media; Spider biofilm induced
PIS59039.1	29.432781	28.716513	29.5172504	28.71075982	27.5286391	27.61916376	0.018950245	0.888	-1.27	PIS59039.1	INP51	Putative phosphatidylinositol-4,5-bisphosphate phosphatase; involved in maintenance of phosphoinositide levels; affects hyphal growth, virulence, cell integrity; interacts with Irs4p
PIS55822.1	31.0906573	31.2491597	31.5159325	30.39670466	30.09829085	29.55416423	0.001458492	0.642	-1.27	PIS55822.1	orf19.1212	Ortholog(s) have FFAT motif binding, phosphatidylinositol binding activity
PIS54966.1	31.102481	32.5819912	31.97032	28.7925507	31.14261199	31.90914476	0.177849737	0.957	-1.27	PIS54966.1	orf19.6701	Protein with similarity to amino acid-tRNA ligase; stationary phase enriched protein; GlcNAc-induced protein

PIS58067.1	25.8161291	26.0534246	27.3520026	25.07636096	24.12983542	26.21053754	0.093936787	0.944	-1.27	PIS58067.1	orf19.688	Mitochondrial ribosomal protein of the small subunit; S. cerevisiae ortholog is essential for viability; Spider biofilm repressed
PIS58571.1	26.5879847	26.0755047	26.0023357	24.26991099	25.34411877	25.2505497	0.174342959	0.956	-1.27	PIS58571.1	orf19.782	Ortholog(s) have hydrolase activity, acting on ester bonds, triglyceride lipase activity, role in lipid homeostasis and lipid droplet localization
PIS56554.1	28.8031368	30.2302308	30.3024859	27.0979939	29.09567253	29.3441604	0.056761506	0.93	-1.27	PIS56554.1	TRR1	Thioredoxin reductase; regulated by Tsa1/Tsa1B, Hap43; induced by nitric oxide, peroxide; oxidative stress-induce via Cap1; induced by human neutrophils; stationary phase enriched protein
PIS58505.1	26.1243053	27.3623544	27.9116902	24.68493625	25.71029578	27.1982197	0.092679696	0.943	-1.27	PIS58505.1	VMA4	H+ transporting ATPase E chain; transcript regulated by Mig1; caspofungin repressed; protein level decreases in stationary phase cultures; rat catheter biofilm repressed
PIS48527.1	28.247446	28.1802069	28.2982263	26.01777516	27.64929302	27.25708385	0.125937695	0.95	-1.27	PIS48527.1	VPH1	Vacuolar H(+)-ATPase; transcription regulated by Nrg1, Mig1, and Tup1
PIS55590.1	24.8854364	25.2906172	24.6383355	23.49264822	23.54788139	23.99177082	0.002322052	0.709	-1.26	PIS55590.1	orf19.6642	Ortholog(s) have role in endoplasmic reticulum to Golgi vesicle-mediated transport and COPII-coated ER to Golgi transport vesicle, Golgi membrane, endoplasmic reticulum membrane localization
PIS50311.1	28.7045195	29.1547079	28.2839088	26.63641132	27.81623912	27.90686349	0.005469898	0.803	-1.26	PIS50311.1	PRO1	Putative gamma-glutamyl kinase; transcript regulated by Nrg1; regulated by Gcn2 and Gcn4; Hap43-repressed gene; early-stage flow model biofilm induced gene

PIS52455.1	29.4709558	29.8384257	30.942269	29.00834019	28.60251247	28.8593607	0.041645265	0.921	-1.26	PIS52455.1	QCR2	Ubiquinol-cytochrome-c reductase; antigenic; induced by interaction with macrophage; repressed by nitric oxide; in detergent-resistant membrane fraction (possible lipid raft component); levels decrease in stationary phase; Hap43p-repressed
PIS48636.1	32.2976959	33.0679693	33.3896215	31.76215437	31.43634047	31.78914526	0.00264416	0.725	-1.26	PIS48636.1	RPL12	Ribosomal protein L12, 60S ribosomal subunit; downregulated by human whole blood or polymorphonuclear cells; genes encoding cytoplasmic ribosomal subunits are downregulated upon phagocytosis by macrophage; Tbf1p-activated; Hap43p-induced
PIS58021.1	29.7808469	30.4157163	31.5428788	29.13276709	29.46702599	29.36177116	0.009790628	0.849	-1.26	PIS58021.1	SDH12	Succinate dehydrogenase; soluble protein in hyphae; macrophage-downregulated protein level; downregulated by Efg1p; repressed by nitric oxide; protein present in exponential and stationary growth phase yeast cultures; Hap43p-
PIS54515.1	30.3015733	30.8805247	31.0749523	28.83080672	29.90700317	29.78317272	0.007145651	0.826	-1.25	PIS54515.1	DHH1	Putative RNA helicase
PIS52119.1	29.6644517	31.0115858	32.5409831	29.83945652	29.34225359	30.27244454	0.049685646	0.926	-1.25	PIS52119.1	ERG10	Acetyl-CoA acetyltransferase; role in ergosterol biosynthesis; soluble in hyphae; changes in protein abundance associated with azole resistance; fluconazole or ketoconazole induced; macrophage-downregulated protein; GlcNAc-induced protein
PIS58625.1	31.4288358	32.0947562	31.433309	30.41566062	30.3863963	30.3966305	0.004943288	0.794	-1.25	PIS58625.1	LSP1	Eisosome component with a predicted role in endocytosis; protein present in exponential and stationary growth phase yeast cultures; caspofungin repressed; biofilm induced; fungal-specific (no human/murine homolog); sumoylation target
PIS58073.1	26.8739637	28.1792179	28.7039634	25.87746861	26.62440005	27.51726217	0.047711375	0.925	-1.25	PIS58073.1	orf19.6596	Putative esterase; possibly transcriptionally regulated by Tac1; induced by Mnl1 under weak acid stress; protein present in exponential and stationary growth phase yeast cultures; Spider biofilm repressed

PIS49731.1	27.8651644	27.9799352	28.3152346	25.73968273	26.40415169	28.26879987	0.065216727	0.934	-1.25	PIS49731.1	orf19.6788	Protein with a predicted role in cotranslational protein targeting to membrane; induced during chlamydospore formation in both C. albicans and C. dubliniensis
PIS52463.1	26.237966	27.2307529	26.8166208	25.84211857	24.33323805	26.36913271	0.025668589	0.902	-1.25	PIS52463.1	RRP9	Ribosomal protein; mutation confers resistance to 5-fluorocytosine (5-FC), 5-fluorouracil (5-FU), and tubercidin (7-deazaadenosine); physically interacts with TAP-tagged Nop1; Hap43-induced; Spider biofilm induced
PIS58159.1	28.9784967	28.4381209	29.4924546	27.71317197	27.76945386	27.69908251	0.001704388	0.666	-1.24	PIS58159.1	CYP5	localization
PIS51163.1	30.1154066	30.6751686	30.1809976	28.57283373	29.18976269	29.4985903	0.00314775	0.746	-1.24	PIS51163.1	orf19.345	Succinate semialdehyde dehydrogenase; for utilization of gamma-aminobutyrate (GABA) as a nitrogen source; part of 4-aminobutyrate and glutamate degradation pathways; rat catheter biofilm induced
PIS56858.1	26.9123927	26.4759592	25.507273	25.42490908	26.2788994	23.46543748	0.15775075	0.955	-1.24	PIS56858.1	orf19.4575	Ortholog(s) have role in mitochondrion organization, phospholipid homeostasis and mitochondrial inner membrane, mitochondrial inner-outer membrane contact site localization
PIS49694.1	24.4953989	25.2822686	24.9083923	24.81219507	21.89446972	24.2529222	0.075644569	0.938	-1.24	PIS49694.1	orf19.4744	Putative phosphatidylinositol 3-phosphate (PI3P) phosphatase; repressed by alpha pheromone in SpiderM medium
PIS58597.1	28.0029091	28.0155331	28.7936479	26.72027108	26.72685659	27.64394355	0.026442441	0.903	-1.24	PIS58597.1	orf19.7403	Ortholog of S. cerevisiae : YML020W, C. glabrata CBS138 : CAGL0G07062g, C. dubliniensis CD36 : Cd36_86190, C. parapsilosis CDC317 : CPAR2_404740 and C. auris B8441 : B9J08_000043

PIS55661.1	31.3534499	31.9274776	32.0143238	30.28156019	30.56609743	30.75417216	0.002186061	0.701	-1.23	PIS55661.1	CDC60	Cytosolic leucyl tRNA synthetase; conserved amino acid and ATP binding class I signature, tRNA binding, proofreading motifs; likely essential for growth; interacts with benzoxaborole antifungals; present in exponential and stationary phase
PIS51244.1	31.2256172	31.7852367	31.2271653	29.96502969	30.48328782	30.08779221	0.007526266	0.83	-1.23	PIS51244.1	KAR2	Similar to Hsp70 family chaperones; role in translocation of proteins into the ER; induced in high iron; protein present in exponential and stationary growth phase yeast cultures; flow model and Spider biofilm repressed
PIS51278.1	25.7450541	26.3874218	26.7816874	25.27757097	25.44103675	24.51283642	0.249976521	0.962	-1.23	PIS51278.1	LYS144	Zn(II)2Cys6 transcription factor; has similarity to S. cerevisiae Lys14, involved in the regulation of lysine biosynthesis genes
PIS51745.1	24.1009743	25.3087256	27.4804931	23.50266818	24.75185753	24.94816375	0.163905143	0.955	-1.23	PIS51745.1	orf19.2047	Putative protein of unknown function; Hap43p-repressed gene; mutation confers hypersensitivity to toxic ergosterol analog, and to amphotericin B
PIS56715.1	30.8277748	30.9847328	30.8930744	29.21987654	29.7496599	30.05419245	0.012678485	0.866	-1.23	PIS56715.1	orf19.3982	Maltase; induced during growth on sucrose; induced by alpha pheromone in SpiderM medium; early-stage flow model biofilm indced
PIS52351.1	26.6976029	29.4916011	29.3311522	27.10509019	26.90055566	27.83605057	0.093374723	0.944	-1.23	PIS52351.1	orf19.4960	Ortholog(s) have spermine synthase activity and role in pantothenate biosynthetic process, spermine biosynthetic process
PIS56959.1	25.0282194	28.1448801	27.0671464	25.83238744	25.32850806	25.39782862	0.059655013	0.932	-1.23	PIS56959.1	orf19.5633	F-box domain-containing protein; flow model biofilm induced

PIS58022.1	27.4881122	27.9155809	27.134709	25.20154871	26.76708776	26.88447928	0.133933198	0.951	-1.23	PIS58022.1	orf19.764	Ortholog(s) have role in negative regulation of TORC1 signaling and cytoplasm localization
PIS48445.1	33.1788521	33.3971442	34.9790576	32.09608271	32.90414233	32.85152589	0.025495871	0.902	-1.23	PIS48445.1	POR1	Mitochondrial outer membrane porin; in detergent-resistant membrane fraction (possible lipid raft component); antigenic in human, mouse; Hap43p-induced; flucytosine-, macrophage-, farnesol- induced; fluconazole, caspofungin repressed
PIS52058.1	32.0190432	31.9048763	31.7902015	30.46616066	30.70259788	30.85114648	0.007757645	0.832	-1.23	PIS52058.1	RPN3	Putative non-ATPase regulatory subunit of the 26S proteasome lid; amphotericin B repressed; oxidative stress-induced via Cap1p
PIS55765.1	33.8750242	33.9130872	33.6428721	31.99557089	33.08191082	32.65068091	0.00127009	0.62	-1.23	PIS55765.1	RPS13	Putative ribosomal protein of the small subunit
PIS55074.1	30.5456295	30.1851889	30.3353437	28.86736766	29.17630343	29.36335789	0.001284668	0.622	-1.22	PIS55074.1	CDC68	Functional homolog of S. cerevisiae Cdc68, a transcription elongation factor; essential; possible drug target
PIS52115.1	26.7007813	26.7429601	27.5182475	25.89523709	26.06961899	25.34432027	0.030615343	0.909	-1.22	PIS52115.1	orf19.4518	Protein kinase that appears to be phosphorylated by Sky1p and Sky2p and itself phosphorylates Hrk1p; has a putative role in stress response; mutants are viable
PIS48806.1	24.0638404	25.798559	26.8205556	24.15598676	24.6564454	24.21028652	0.063168883	0.934	-1.22	PIS48806.1	orf19.5880	Putative voltage-gated chloride channel; predicted role in copper ion and iron ion homeostasis; flow model biofilm induced

PIS58954.1	25.9590315	25.5762985	26.7243535	24.56527566	24.43525507	25.62632562	0.076032561	0.939	-1.21	PIS58954.1	orf19.252	Protein of unknown function; S. cerevisiae ortholog Fmp37 which localizes to mitochondria; Hap43-repressed; Spider biofilm repressed
PIS54471.1	31.300153	30.4165604	30.8478923	29.17473994	30.20738207	29.54119683	0.01977953	0.89	-1.21	PIS54471.1	orf19.3139	Putative NADP-dependent oxidoreductase; Hap43-repressed; induced by benomyl treatment; oxidative stress-induced via Cap1; rat catheter biofilm repressed
PIS58446.1	30.1226286	30.4822985	30.3811029	29.05674819	29.24527892	29.04534416	0.004827749	0.791	-1.21	PIS58446.1	orf19.3679	Putative protein of unknown function; stationary phase enriched protein
PIS55786.1	26.3099068	26.0681509	28.0677822	25.60611131	24.92307799	26.32330496	0.038097551	0.918	-1.2	PIS55786.1	orf19.1681	Ortholog of C. dubliniensis CD36 : Cd36_81530, C. parapsilosis CDC317 : CPAR2_503590, C. auris B8441 : B9J08_001892 and Candida tenuis NRRL Y-1498 : CANTEDRAFT_115755
PIS50539.1	30.1452307	30.8865896	31.8520539	29.97999439	29.80480027	29.50624424	0.041480964	0.921	-1.2	PIS50539.1	orf19.3932	Predicted RNA binding protein; stationary phase enriched; induced in core caspofungin response; induced by nitric oxide independent of Yhb1; repressed in ssr1 null; ketoconazole, hypoxia induced; Spider biofilm induced
PIS58314.1	31.7786549	32.5152234	33.6734339	31.24866286	31.61834239	31.49522919	0.086436034	0.942	-1.2	PIS58314.1	PGI1	Glucose-6-phosphate isomerase; enzyme of glycolysis; antigenic; Efg1-regulated; induced upon adherence to polystyrene; repressed by phagocytosis, human neutrophils; flow model biofilm induced; rat catheter and Spider biofilm repressed
PIS49567.1	30.3489717	30.420193	30.6307062	29.13580039	29.17148522	29.49589154	0.011698434	0.861	-1.2	PIS49567.1	YHM2	Predicted carrier protein; exports citrate from and imports oxoglutarate into the mitochondrion; alkaline induced; Spider biofilm repressed

PIS58577.1	32.5806567	32.6906672	32.967775	31.82644058	31.46949044	31.37829034	0.001793106	0.673	-1.19	PIS58577.1	APE2	Neutral arginine, alanine, leucine specific metallo-aminopeptidase; purified from cell wall/intracellular fractions; protein repressed during mating; Hog1, farnesol-induced; may be essential (UAU1 method); rat catheter biofilm repressed
PIS58606.1	30.2791637	29.9251228	30.3600074	29.33145488	28.6814786	28.96794957	0.000637349	0.499	-1.19	PIS58606.1	orf19.2954	Protein of unknown function; Hap43-repressed gene; repressed by nitric oxide
PIS55010.1	23.9402918	25.3966104	27.4273081	25.85286687	24.84548518	22.50405948	0.206320642	0.959	-1.19	PIS55010.1	orf19.6319	Ortholog(s) have cysteine-type deubiquitinase activity, mRNA binding activity
PIS51687.1	27.255938	26.670855	26.9496121	25.70984058	25.06170218	26.54508625	0.114452161	0.948	-1.19	PIS51687.1	orf19.7043	Ortholog(s) have role in protein targeting to mitochondrion
PIS58927.1	26.1976016	27.0689949	28.5263656	26.18764443	25.05311407	26.97639996	0.118808171	0.949	-1.19	PIS58927.1	PHR2	Glycosidase; role in vaginal not systemic infection (low pH not neutral); low pH, high iron, fluconazole, Hap43-induced; Rim101-repressed at pH8; rat catheter biofilm induced; Bcr1-repressed in RPMI a/a biofilms
PIS49737.1	30.856731	29.7911829	30.4580205	29.34129424	29.05351663	29.15532623	0.006909189	0.823	-1.19	PIS49737.1	PRO2	Putative gamma-glutamyl phosphate reductase with a predicted role in proline biosynthesis; regulated by Gcn2p and Gcn4p
PIS54699.1	33.0142995	33.7302223	33.701572	31.48183948	32.66678754	32.74525075	0.01778964	0.885	-1.18	PIS54699.1	AAT1	Aspartate aminotransferase; soluble protein in hyphae; macrophage-induced protein; alkaline upregulated; amphotericin B repressed; gene used for strain identification by multilocus sequence typing; farnesol-, Hap43p-induced; GlcNAc-induced

PIS54548.1	27.9881837	28.9283007	28.9531259	26.32078121	28.08964102	27.91525578	0.021360095	0.894	-1.18	PIS54548.1	CDC12	Septin; essential for viability; forms ring at sites of cell division and also forms filaments in mature chlamydospore; filamentous growth induced; regulated by Nrg1, Tup1, tyrosol and cell density; rat catheter biofilm repressed
PIS55018.1	26.7678568	25.8885236	25.4298111	26.0019729	25.07451327	23.47884045	0.073410896	0.938	-1.18	PIS55018.1	MAL31	Putative high-affinity maltose transporter; transcript is upregulated in clinical isolates from HIV+ patients with oral candidiasis; alkaline induced; Spider biofilm induced
PIS48430.1	31.9374851	32.2620728	31.8582519	30.73016446	31.07505183	30.69768164	0.0017185	0.667	-1.18	PIS48430.1	MIS11	Predicted mitochondrial C1-tetrahydrofolate synthase precursor; putative protein of glycine catabolism; repressed by Efg1; fluconazole-induced; stationary phase enriched protein; rat catheter and Spider biofilm repressed
PIS52030.1	22.8626328	24.8110653	25.1048301	19.97317982	25.05135364	24.22046486	0.278433438	0.964	-1.18	PIS52030.1	orf19.1565	Protein of unknown function
PIS54589.1	30.8899195	31.5110132	31.5061469	30.1649589	30.29257525	29.89526791	0.004023533	0.773	-1.18	PIS54589.1	orf19.239	Putative ATP-dependent helicase, component of the RSC chromatin remodeling complex; essential gene; induced by nitric oxide
PIS50326.1	26.9219788	26.7252633	25.6368929	24.80630543	25.89142961	25.057055	0.086522613	0.942	-1.18	PIS50326.1	orf19.4922	Ortholog(s) have small GTPase binding activity and Golgi apparatus localization
PIS50444.1	28.9363032	29.4880913	30.2344397	28.12821341	28.21790791	28.7872943	0.241674809	0.962	-1.18	PIS50444.1	orf19.5660.1	Ortholog(s) have proton-transporting ATP synthase activity, rotational mechanism, structural molecule activity and role in cristae formation, protein-containing complex assembly, proton motive force-driven ATP synthesis

PIS50405.1	32.2737063	32.8935153	33.052876	31.18320233	31.89757472	31.63966212	0.003726372	0.765	-1.17	PIS50405.1	IDH1	Putative mitochondrial NAD-isocitrate dehydrogenase subunit 1; soluble protein in hyphae; protein level decrease in stationary phase cultures
PIS54927.1	25.7812028	26.8859997	25.969356	25.25966324	24.58314414	25.29457929	0.061238779	0.933	-1.17	PIS54927.1	orf19.1857	Putative L-azetidine-2-carboxylic acid acetyltransferase; mutants are viable
PIS58497.1	25.4722539	26.4416703	26.8874714	26.26200007	24.8117415	24.20408855	0.040275118	0.92	-1.17	PIS58497.1	orf19.3325	Putative glycogen synthesis initiator; regulated by Efg1 and Efh1; Hog1-repressed; colony morphology-related gene regulation by Ssn6; induced by prostaglandins; flow model biofilm induced
PIS58451.1	25.0962843	26.2487322	26.5906971	26.68997935	23.11034168	24.62731063	0.128216981	0.951	-1.17	PIS58451.1	orf19.3684	Putative oxidoreductase; Spider biofilm induced
PIS52450.1	34.3145864	34.4586296	34.4565809	33.40284091	33.32678382	32.98916511	0.000514838	0.459	-1.17	PIS52450.1	RPS5	Ribosomal protein S5; macrophage/pseudohyphal-induced after 16 h; downregulated upon phagocytosis by murine macrophage; Hap43-induced; Spider biofilm repressed
PIS48614.1	29.3650161	29.512968	29.129616	28.49325986	27.98156032	28.05425273	0.011535264	0.86	-1.16	PIS48614.1	MRP17	Predicted mitochondrial ribosomal protein
PIS58036.1	23.5251338	27.5842186	27.2021508	25.14772526	24.54773231	25.14836033	0.31218417	0.966	-1.16	PIS58036.1	orf19.1201	Ortholog(s) have serine-tRNA ligase activity, role in mitochondrial seryl-tRNA aminoacylation and mitochondrion localization

PIS55581.1	27.5579236	28.3493349	28.7932198	27.03082813	27.08273215	27.10135665	0.005995058	0.811	-1.16	PIS55581.1	orf19.3353	Protein similar to a mitochondrial complex I intermediate-associated protein; fluconazole-repressed; Spider biofilm induced; rat catheter biofilm repressed
PIS52002.1	28.3145243	28.2897075	27.9535728	27.23350972	26.82949797	27.01617625	0.002463533	0.716	-1.16	PIS52002.1	orf19.5114	Sorting nexin; role in maintaining late-Golgi resident enzymes in their proper location by recycling molecules from the prevacuolar compartment; Spider biofilm induced
PIS55772.1	27.2625384	26.6994809	27.3839783	25.16232079	26.1036135	26.60877836	0.128389809	0.951	-1.16	PIS55772.1	orf19.6748	Ortholog(s) have eukaryotic 43S preinitiation complex, eukaryotic translation initiation factor 3 complex localization
PIS49632.1	31.0133823	30.9111428	31.2123655	29.91436256	29.99765044	29.73162965	0.006358107	0.816	-1.16	PIS49632.1	orf19.7288	Protein with predicted oxidoreductase and dehydrogenase domains; Hap43-repressed; Spider biofilm induced
PIS58584.1	26.904593	29.3940642	30.4907829	30.516577	24.24544205	28.54451473	0.365084145	0.968	-1.16	PIS58584.1	orf19.7502	Protein of unknown function; Hap43-induced gene; upregulated in a cyr1 null mutant; Spider biofilm induced
PIS51716.1	31.5770251	32.8151283	33.0420753	30.7848934	31.31734793	31.84273848	0.068882467	0.936	-1.16	PIS51716.1	RPL4B	Ribosomal protein 4B; repressed upon phagocytosis by murine macrophage; Spider biofilm repressed
PIS49531.1	30.3605551	30.6781571	30.2715386	29.28076623	28.8879537	29.66290249	0.001689573	0.664	-1.16	PIS49531.1	SEC24	Protein with a possible role in ER to Golgi transport; induced upon yeast-hyphal switch; sumoylation target; Spider biofilm repressed

PIS48468.1	29.8980199	30.3956095	31.4606281	29.26300829	29.34444483	29.65375621	0.036564855	0.916	-1.16	PIS48468.1	SRB1	Essential GDP-mannose pyrophosphorylase; makes GDP-mannose for protein glycosylation; functional in S. cerevisiae psa1; on yeast-form, not hyphal cell surface; alkaline induced; induced on adherence to polystyrene; Spider
PIS52429.1	29.5560767	29.7965281	29.6722994	28.28994269	28.59307845	28.67529882	0.03908276	0.918	-1.16	PIS52429.1	TRP3	Putative bifunctional enzyme with predicted indole-3-glycerol-phosphate synthase and anthranilate synthase activities; regulated by Gcn2p and Gcn4p
PIS55491.1	32.3963916	32.7802689	32.6277121	31.5842279	31.39783924	31.36117547	0.007462034	0.829	-1.15	PIS55491.1	GAD1	Putative glutamate decarboxylase; alkaline, macrophage-downregulated gene; amphotericin B induced; induced by Mnl1 under weak acid stress; stationary phase enriched protein; rat catheter biofilm repressed
PIS58240.1	24.6547943	24.3513028	26.4262233	24.30486504	24.33749981	23.34570135	0.112705266	0.948	-1.15	PIS58240.1	GPI13	Major facilitator superfamily protein; has phosphodiesterase/nucleotide pyrophosphatase domain; similar to S. cerevisiae Gpi13p, which acts in GPI anchor biosynthesis; Hap43p-induced gene
PIS58551.1	28.6298208	28.5030725	28.7394729	27.82111047	28.00427563	26.58599191	0.018502479	0.887	-1.15	PIS58551.1	orf19.3342	Ortholog(s) have role in positive regulation of protein autoubiquitination, protein deubiquitination
PIS56857.1	27.6311757	28.3883264	28.6638035	26.88185372	27.08457793	27.25307415	0.015020203	0.876	-1.15	PIS56857.1	orf19.4609	Putative dienelactone hydrolase; protein abundance is affected by URA3 expression in the CAI-4 strain background; protein present in exponential and stationary growth phase yeast cultures; rat catheter biofilm repressed
PIS54586.1	24.6471451	26.7137922	25.8804538	23.10195794	24.71647757	25.96777075	0.195398411	0.958	-1.15	PIS54586.1	orf19.6166	Ortholog of C. dubliniensis CD36 : Cd36_80810, C. parapsilosis CDC317 : CPAR2_101800, C. auris B8441 : B9J08_002362 and Candida tenuis NRRL Y-1498 : CANTEDRAFT_103482

PIS58573.1	27.8823697	28.4215951	28.4242129	25.83786385	27.85388589	27.59060392	0.042890529	0.922	-1.15	PIS58573.1	orf19.6853	Protein of unknown function; Spider biofilm repressed
PIS55513.1	28.3485421	28.7057343	30.1069939	28.17940062	28.2156355	27.3158878	0.103902928	0.946	-1.15	PIS55513.1	PHO100	Putative inducible acid phosphatase; DTT-extractable and observed in culture supernatant in low-phosphate conditions; slight effect on murine virulence; virulence-group-correlated expression; N-glycosylated; F-12/CO2 early biofilm induced
PIS50302.1	25.4090839	22.9663348	26.3804549	24.63516412	23.80551789	22.90781921	0.173784067	0.956	-1.14	PIS50302.1	CPR6	Putative peptidyl-prolyl cis-trans isomerase; macrophage/pseudohyphal-repressed; heavy metal (cadmium) stress-induced; heterozygous null mutant displays sensitivity to virgineone; rat catheter biofilm induced
PIS52062.1	31.1750431	30.2853952	29.9981334	29.42308619	28.9126668	29.71773257	0.034363739	0.914	-1.14	PIS52062.1	orf19.3060	OPutative dolichyl-diphosphooligosaccharide-protein glycotransferase; role in protein N-linked glycosylation; Spider biofilm repressed
PIS49541.1	27.9557826	27.7802463	28.1548016	24.25628693	28.1815489	28.03843514	0.191517453	0.958	-1.14	PIS49541.1	orf19.698	Integral ER membrane protein; predicted role in maintenance of ER zinc homeostasis; Spider biofilm induced
PIS58351.1	28.5908499	28.3400194	28.3046908	27.56902722	27.27876524	26.97903261	0.002737036	0.729	-1.14	PIS58351.1	PDK2	Putative pyruvate dehydrogenase kinase; mutation confers hypersensitivity to amphotericin B
PIS56823.1	27.5672247	28.6660918	27.697097	26.77070109	27.94088671	25.82229221	0.058442548	0.931	-1.13	PIS56823.1	DAK2	Putative dihydroxyacetone kinase; repressed by yeast-hypha switch; fluconazole-induced; caspofungin repressed; protein enriched in stationary phase yeast cultures; flow model biofilm induced; rat catheter and Spider biofilm repressed

PIS51998.1	28.7119842	29.7148095	29.6700358	28.89337888	28.37218797	27.43131384	0.026836126	0.904	-1.13	PIS51998.1	GCV2	Glycine decarboxylase P subunit; protein of glycine catabolism; repressed by Efg1; Hog1-induced; induced by Rim101 at acid pH; transcript induced in elevated CO2; stationary phase enriched protein
PIS54619.1	26.8274461	27.5183605	28.1780091	26.88423304	25.87102996	26.38221272	0.044155041	0.923	-1.13	PIS54619.1	orf19.1386	Ortholog(s) have SNAP receptor activity and role in endoplasmic reticulum to Golgi vesicle-mediated transport, retrograde vesicle-mediated transport, Golgi to endoplasmic reticulum, vesicle fusion
PIS58488.1	30.0257874	30.1240101	31.000403	30.23561464	28.92230224	28.59604714	0.029467211	0.908	-1.13	PIS58488.1	orf19.7296	Plasma membrane protein implicated in stress response; similar to stomatin mechanoreception proteins; overexpression induces apoptotic-like cell death; absent from hyphal cells; induced by Rgt1; rat catheter and Spider biofilm induced
PIS56734.1	29.9946226	29.8618854	30.5857613	28.89974705	28.98211016	29.20579344	0.006310019	0.816	-1.12	PIS56734.1	KEL1	Kelch repeat domain-containing protein; localizes to sites of polarized growth; mutant colonies exhibit slightly decreased filamentation ratio; not required for buccal epithelial cell adherence or virulence in mice
PIS52248.1	27.9132831	24.6604254	26.3634973	25.62905997	23.82570469	26.12343274	0.376236962	0.968	-1.12	PIS52248.1	orf19.1457	Putative RNA polymerase transcription factor TFIIH core component; possibly an essential gene, disruptants not obtained by UAU1 method
PIS54828.1	27.428352	27.4162623	27.5820079	26.29977249	26.44934897	26.32602434	0.000925084	0.567	-1.12	PIS54828.1	orf19.1734	Putative ATPase and nucleosome spacing factor; required for hyphal growth; removes histone variant H2A.Z (Hta3p) from hypha-specific promoters ; heterozygous null mutant displays sensitivity to virgineone
PIS48321.1	29.8594357	29.8929611	29.6368176	29.27649101	28.72430218	28.0271363	0.00451443	0.785	-1.12	PIS48321.1	orf19.3558	Ortholog of S. cerevisiae : ERP3, C. glabrata CBS138 : CAGL0K01793g, C. dubliniensis CD36 : Cd36_19780, C. parapsilosis CDC317 : CPAR2_206420 and C. auris B8441 : B9J08_005011

PIS55502.1	28.677992	29.2681934	29.5486852	28.07076968	28.06199014	28.00789596	0.004441484	0.783	-1.12	PIS55502.1	orf19.5054	Putative quinolinate phosphoribosyl transferase, involved in NAD biosynthesis; Hap43p-repressed gene
PIS48783.1	28.3853638	28.6550946	29.1760474	27.97719608	27.31101486	27.55962161	0.016767282	0.882	-1.12	PIS48783.1	orf19.512	Ortholog of S. cerevisiae Kre33; essential; S. cerevisiae ortholog is essential and is required for biogenesis of the small ribosomal subunit
PIS50633.1	30.4083942	30.8911168	32.0987969	28.98521796	30.64237383	30.40063426	0.109442063	0.947	-1.12	PIS50633.1	PHB2	Prohibitin 2; plasma membrane localized
PIS54517.1	30.0187356	30.043711	30.7308392	29.26691294	29.2478234	28.94379239	0.044511492	0.923	-1.11	PIS54517.1	EGD1	Putative GAL4 DNA-binding enhancer protein; soluble protein in hyphae; biofilm induced; macrophage/pseudohyphal-induced; equal level of protein in exponential and stationary growth phase yeast cultures; Spider biofilm repressed
PIS48258.1	28.5956519	29.1610946	28.0031115	27.02330707	27.93720599	27.47032247	0.011955669	0.862	-1.11	PIS48258.1	ERG2	C-8 sterol isomerase; enzyme of ergosterol biosynthesis; converts fecosterol to episterol; mutant is hypersensitive to multiple drugs; ketoconazole-induced; flow model and Spider biofilm repressed
PIS52209.1	28.5223252	28.2232104	28.2393515	27.33677056	27.07461505	27.23553782	0.0256247	0.902	-1.11	PIS52209.1	HRK1	Putative serine/threonine kinase; predicted role in cellular ion homeostasis; Spider biofilm repressed
PIS54815.1	23.9201429	26.4846524	25.9771281	22.93977429	25.11822749	24.99178473	0.210638005	0.96	-1.11	PIS54815.1	NIK1	Histidine kinase involved in a two-component signaling pathway that regulates cell wall biosynthesis; required for wild-type virulence in mouse systemic infection but not for wild-type growth or drug sensitivity/resistance; 9 HAMP [illegible]

PIS48484.1	25.4731918	26.8056646	25.4640252	24.06966052	24.95420465	25.37472995	0.159298474	0.955	-1.11	PIS48484.1	orf19.2202	Protein of unknown function; induced by alpha pheromone in SpiderM medium
PIS55718.1	30.0404224	29.9584304	31.0088272	29.66896907	29.46483257	28.57046818	0.04582974	0.924	-1.1	PIS55718.1	ARC35	Putative ARP2/3 complex subunit; shows colony morphology-related gene regulation by Ssn6p; mutation confers hypersensitivity to cytochalasin D
PIS51884.1	26.4397979	24.8636043	26.1989605	24.78422897	24.40070055	25.01632214	0.098131972	0.945	-1.1	PIS51884.1	CCE1	Putative Holliday junction resolving enzyme; similar to S. cerevisiae Cce1p
PIS50367.1	30.901327	30.6156317	30.9265124	28.82825176	30.05548231	30.25498527	0.009974927	0.851	-1.1	PIS50367.1	CHO2	Phosphatidyl-ethanolamine N-methyltransferase; fungal-specific (no human or murine homolog); amphotericin B repressed; Hap43p-induced gene
PIS48451.1	32.3029284	32.2203996	32.0899914	30.49467264	30.62367079	32.20857086	0.026608406	0.904	-1.1	PIS48451.1	HTA2	Putative histone H2A; farnesol regulated; rat catheter biofilm repressed; Spider biofilm repressed; Hap43-induced
PIS54594.1	27.9221668	28.1480958	28.2079482	26.53864016	26.98138692	27.4620559	0.009291585	0.846	-1.1	PIS54594.1	MED7	Subunit of the RNA polymerase II mediator complex
PIS48519.1	31.7026031	31.6354941	32.5880287	30.26871441	30.42702766	31.941771	0.037691479	0.917	-1.1	PIS48519.1	MYO5	Class I myosin; nonessential; role in cortical actin patch polarity and polar budding; required for hyphal growth, white-opaque switch; regulatory phosphorylation on S366; downregulated on adherence to polystyrene

PIS49519.1	26.4796482	25.9007298	26.8690679	26.00719659	24.82397291	25.11822371	0.043309104	0.922	-1.1	PIS49519.1	orf19.5156	Protein similar to S. cerevisiae Phs1p, which is required for growth; has six putative membrane-spanning regions
PIS58617.1	26.5716867	28.8704777	29.1813403	24.61023361	28.71122129	27.99585855	0.281596069	0.964	-1.1	PIS58617.1	PRE2	Putative proteasome beta-5 subunit; macrophage-induced protein
PIS49557.1	28.8031788	28.7005215	29.016856	27.44509235	27.95538814	27.81314762	0.009196724	0.845	-1.1	PIS49557.1	YPT72	Vacuolar Rab small monomeric GTPase involved in vacuolar biogenesis; involved in filamentous growth and virulence
PIS58593.1	29.3249512	29.5617125	29.6924569	28.52143302	28.80600222	27.9713228	0.01555204	0.878	-1.09	PIS58593.1	ERV25	Component of COPII-coated vesicles; transcript induced upon filamentous growth; rat catheter biofilm repressed
PIS56743.1	30.716661	30.307189	31.3649093	27.87057908	30.6294144	30.60387004	0.240404482	0.962	-1.09	PIS56743.1	orf19.2017	Ortholog(s) have RNA polymerase I activity and role in nucleolar large rRNA transcription by RNA polymerase I, transcription by RNA polymerase I, transcription elongation by RNA polymerase I
PIS55483.1	31.1897476	31.3613497	31.0244926	30.2072919	29.93792699	30.15527023	0.004971154	0.794	-1.09	PIS55483.1	orf19.4864	Ortholog(s) have acylglycerol lipase activity, role in triglyceride metabolic process and lipid droplet, membrane localization
PIS54891.1	26.5860395	27.2293167	27.1663821	26.03736616	25.49794378	26.1841623	0.078331996	0.939	-1.09	PIS54891.1	orf19.4952.1	Ortholog(s) have FK506 binding, peptidyl-prolyl cis-trans isomerase activity and membrane localization

PIS58038.1	26.0084451	26.5355831	26.7086412	25.76478362	25.23824325	24.99142934	0.041287496	0.92	-1.09	PIS58038.1	PKH3	Probable serine/threonine protein kinase; appears to act redundantly with Pkh2 within the Ypk1 signaling pathway
PIS51712.1	24.3402984	24.1645745	26.8597616	24.95788466	23.37377842	23.74810073	0.288955323	0.965	-1.09	PIS51712.1	URA3	Orotidine-5'-phosphate decarboxylase; pyrimidine biosynthesis; gene used as genetic marker; decreased expression when integrated at ectopic chromosomal locations can cause defects in hyphal growth and virulence; Spider biofilm repressed
PIS51816.1	30.6763176	30.2768049	30.1412679	28.55686505	29.84828907	29.45799197	0.013546104	0.87	-1.08	PIS51816.1	NUC2	Putative NADH-ubiquinone oxidoreductase; identified in detergent-resistant membrane fraction (possible lipid raft component); alkaline repressed; Hap43-repressed; Spider biofilm repressed
PIS51856.1	27.9267212	29.3229985	29.3517533	27.8777679	27.94018018	27.55270674	0.07400027	0.938	-1.08	PIS51856.1	orf19.5660	Ortholog(s) have ubiquitin protein ligase activity
PIS48418.1	29.4047474	29.1473322	28.8354262	27.75109935	27.95851332	28.43449071	0.129599766	0.951	-1.08	PIS48418.1	orf19.5847	Ortholog(s) have RNA polymerase III activity, role in tRNA transcription by RNA polymerase III, termination of RNA polymerase III transcription and RNA polymerase III complex, chromatin localization
PIS55028.1	24.9796747	25.6701481	26.9920459	23.71682961	25.29024922	25.3862565	0.102833715	0.946	-1.08	PIS55028.1	ROD1	Protein similar to S. cerevisiae Rod1;a membrane protein with a role in drug tolerance; repressed by Rgt1; mutant is viable
PIS55787.1	30.4791746	30.9486667	31.2618504	29.40003252	30.07879961	29.97878887	0.015446239	0.877	-1.08	PIS55787.1	TFP1	Subunit of vacuolar H+-ATPase; stationary phase enriched protein; sumoylation target; Spider biofilm repressed

PIS56633.1	29.5644154	30.182634	31.3236636	28.76948652	29.41789164	29.67905088	0.156375488	0.954	-1.07	PIS56633.1	ADH5	Putative alcohol dehydrogenase; regulated by white-opaque switch; fluconazole-induced; antigenic in murine infection; regulated by Nrg1, Tup1; Hap43, macrophage repressed, flow model biofilm induced; Spider biofilm induced
PIS51206.1	26.386242	27.0543586	27.027737	26.94478143	23.97829378	26.32213781	0.145633191	0.953	-1.07	PIS51206.1	AIP2	Putative actin interacting protein; regulated by Gcn4; induced in response to amino acid starvation (3-AT); repressed by elevated CO2; flow model biofilm repressed
PIS51537.1	28.6751061	28.2823125	28.1771137	26.65822065	27.81579891	27.45234644	0.004948774	0.794	-1.07	PIS51537.1	orf19.4488	Predicted ortholog of S. cerevisiae Swi3, subunit of the SWI/SNF chromatin remodeling complex; possibly an essential gene, disruptants not obtained by UAU1 method
PIS52053.1	31.0491887	30.8958267	32.0060566	30.10878505	30.39202325	30.24229416	0.050750186	0.927	-1.07	PIS52053.1	POL5	Putative DNA Polymerase phi; F-12/CO2 early biofilm induced
PIS55797.1	29.8601557	29.6923603	29.4816194	28.67160418	28.95071933	28.21106155	0.001853658	0.678	-1.07	PIS55797.1	PPT1	Putative serine/threonine phosphatase; induced in high iron
PIS58898.1	28.0562401	28.280134	27.2349207	24.90191597	27.45871848	28.00686703	0.249811402	0.962	-1.07	PIS58898.1	SMC5	Protein similar to S. cerevisiae Smc5p, which is involved in DNA repair; transposon mutation affects filamentous growth
PIS51821.1	27.1156031	26.5757098	26.7476462	24.29637545	26.42362995	26.50093977	0.069017033	0.936	-1.07	PIS51821.1	TOP1	DNA topoisomerase I; required for wild-type growth and for wild-type mouse virulence; sensitive to camptothecin; induced upon adherence to polystyrene; rat catheter biofilm induced

PIS48233.1	33.3841559	33.9166051	33.5011687	32.63928039	32.47245864	32.51941888	0.001651283	0.661	-1.06	PIS48233.1	HSP90	Essential chaperone, regulates several signal transduction pathways and temperature-induced morphogenesis; activated by heat shock, stress; localizes to surface of hyphae, not yeast cells; mediates echinocandin and biofilm azole resistance
PIS54932.1	26.2169626	25.7329693	25.6829892	24.25313082	25.14191102	25.05619261	0.014238232	0.873	-1.06	PIS54932.1	IMP4	Putative SSU processome component; Hap43-induced; repressed by prostaglandins; Spider biofilm induced
PIS51562.1	24.229474	24.8631708	26.3049413	24.68272016	25.30463595	22.23822386	0.235451719	0.961	-1.06	PIS51562.1	orf19.2794	Putative non-specific single-domain racemase; regulated by Gcn4p; repressed in response to amino acid starvation (3-AT treatment); alkaline upregulated; macrophage-induced protein
PIS54983.1	29.2097768	29.4003833	29.1143218	28.2690871	27.88590364	28.40408565	0.010893213	0.856	-1.06	PIS54983.1	orf19.4271	Predicted ORF from Assembly 19; removed from Assembly 20; subsequently reinstated in Assembly 21 based on comparative genome analysis
PIS55531.1	25.4172054	27.0606472	26.745426	26.28276938	23.80276356	25.94884543	0.242760582	0.962	-1.06	PIS55531.1	orf19.5525	Putative oxidoreductase; protein levels affected by URA3 expression in CAI-4 strain background; Efg1, Efh1 regulated; Rgt1-repressed; protein present in exponential and stationary growth phase yeast; rat catheter biofilm repressed
PIS50511.1	31.4633591	31.3274727	32.1227511	30.69627902	30.46407037	30.60168615	0.043972881	0.923	-1.05	PIS50511.1	CCT3	Putative cytosolic chaperonin Cct ring complex subunit; mutation confers hypersensitivity to cytochalasin D
PIS52412.1	23.6030662	21.955948	25.6121598	21.074271	23.59832987	23.35008118	0.293856808	0.965	-1.05	PIS52412.1	CLA4	Ste20p family Ser/Thr kinase required for wild-type filamentous growth, organ colonization and virulence in mouse systemic infection; role in chlamydospore formation; functional homolog of S. cerevisiae Cla4p; mutant caspofungin sensitive

PIS48640.1	29.4478914	31.256575	30.9137388	29.1758989	29.74435348	29.5623524	0.129304279	0.951	-1.05	PIS48640.1	ERG6	Delta(24)-sterol C-methyltransferase, converts zymosterol to fecosterol, ergosterol biosynthesis; mutation confers nystatin resistance; Hap43, GlcNAc-, fluconazole-induced; upregulated in azole-resistant strain; Spider biofilm repressed
PIS52040.1	31.6979425	31.4022905	31.7769939	30.48446256	30.49373206	30.75704312	0.00286116	0.735	-1.05	PIS52040.1	orf19.1549	Plasma membrane-associated protein identified in detergent-resistant membrane fraction (possible lipid raft component); repressed by nitric oxide; predicted transmembrane helix
PIS58103.1	30.1601291	30.1263062	31.6392103	29.21703162	29.21715402	30.35630366	0.250659035	0.962	-1.05	PIS58103.1	orf19.1823	Predicted membrane protein; transcript repressed by ciclopirox olamine
PIS52117.1	33.7645694	34.0122189	34.0399279	32.8717411	32.93988933	32.8418534	0.270942768	0.964	-1.05	PIS52117.1	orf19.4520	Putative gluconokinase; rat catheter biofilm induced
PIS51812.1	24.3246393	24.2560866	24.4116659	23.9205802	23.46290856	22.45516063	0.03187417	0.911	-1.05	PIS51812.1	orf19.6821	Ortholog(s) have ubiquitin protein ligase activity
PIS58227.1	18.695116	26.0915702	24.8669366	17.78406808	23.23557484	25.48894595	0.581010833	0.974	-1.05	PIS58227.1	orf19.7006	Ortholog(s) have role in reciprocal meiotic recombination
PIS51857.1	28.3108137	28.8102678	29.3532094	27.86351704	27.65826786	27.8354384	0.018073277	0.886	-1.04	PIS51857.1	MNN10	Alpha-1,6-mannosyltransferase involved in biosynthesis and organization of cell wall polysaccharides

PIS52245.1	23.7874224	26.633672	25.8388345	23.32478424	24.32427972	25.49883608	0.149001616	0.953	-1.04	PIS52245.1	orf19.1448.1	Ortholog(s) have plus-end-directed microtubule motor activity, protein-containing complex binding activity and role in establishment of mitotic spindle localization, nuclear migration along microtubule, nuclear pore complex assembly
PIS55827.1	28.6753238	27.5073742	28.0993461	26.38759336	27.73583525	27.05079179	0.155378086	0.954	-1.04	PIS55827.1	orf19.2346	Putative protein of unknown function, transcription is positively regulated by Tbf1p
PIS58113.1	25.5417907	25.7229638	25.7948353	24.99351809	24.83371824	24.11580272	0.087451578	0.942	-1.04	PIS58113.1	orf19.6268	Putative cohesin complex subunit; expression downregulated in an ssr1 null mutant
PIS48234.1	26.9857023	24.0971078	25.7541515	24.11865409	25.09793811	24.50301692	0.134078907	0.951	-1.04	PIS48234.1	orf19.7159	Putative protein of unknown function; Hap43p-repressed gene; ortholog of S. cerevisiae YMR185W
PIS58201.1	27.8447939	26.9882146	27.2907161	24.85499313	26.77949645	27.36037453	0.331418186	0.967	-1.04	PIS58201.1	PHO81	Protein involved in regulation of hyphal development; required for response to farnesoic acid; possibly adherence-induced
PIS49811.1	30.2872345	30.9686325	31.2541657	29.73306894	29.74945498	29.91070314	0.095028444	0.944	-1.04	PIS49811.1	PST3	Flavodoxin-like protein involved in oxidative stress protection and virulence; YNB biofilm induced; stationary phase enriched protein; rat catheter and Spider biofilm repressed
PIS58277.1	32.1624055	32.2835275	33.5422274	32.25601831	31.76262184	30.84585325	0.044132409	0.923	-1.04	PIS58277.1	RPS6A	Ribosomal protein 6A; localizes to cell surface of yeast cells but not hyphae; repressed upon phagocytosis by murine macrophage; possibly essential; Hap43-induced; Spider biofilm repressed

PIS54948.1	29.533858	29.3763131	30.6574737	28.59788659	28.75224167	29.10645123	0.085512559	0.941	-1.04	PIS54948.1	SAM50	Predicted component of the SAM complex involved in mitochondrial protein import
PIS51940.1	25.8719367	26.4444952	29.8009063	26.25690368	26.67305748	26.10924171	0.377471676	0.968	-1.03	PIS51940.1	BUD31	Bud31 ortholog; not subject to mating-type regulation, in contrast to S. cerevisiae Bud31 which has a role in specifying the bud site; Spider biofilm induced
PIS56751.1	26.6256379	26.7213356	26.3993174	25.52355506	26.15765735	24.97382167	0.02176204	0.895	-1.03	PIS56751.1	orf19.2050	Ortholog(s) have sterol esterase activity, role in cellular lipid metabolic process, sterol metabolic process and lipid droplet, membrane localization
PIS54671.1	29.8978771	29.7563041	29.9684919	28.67162134	29.04008254	28.81601179	0.002998725	0.74	-1.03	PIS54671.1	orf19.4758	Putative reductase or dehydrogenase; Hap43-repressed gene; alkaline repressed
PIS58987.1	31.7892843	31.7044815	31.6825191	30.84566261	30.94033018	30.30719614	0.001959286	0.686	-1.03	PIS58987.1	orf19.6658	Stationary phase enriched protein; predicted ORF from Assembly 19; removed from Assembly 20; subsequently reinstated in Assembly 21 based on comparative genome analysis
PIS51048.1	30.1773459	29.7851176	29.1021031	28.36583484	29.52500436	28.09419057	0.175701102	0.957	-1.03	PIS51048.1	orf19.6973	ATP-dependent LON protease family member; Hap43-repressed gene; regulated by Gcn2 and Gcn4; Spider biofilm induced
PIS51386.1	28.0308698	28.8344401	29.6675115	26.92453544	28.37050026	28.16163765	0.234611722	0.961	-1.03	PIS51386.1	orf19.7215.3	Ortholog(s) have protein-folding chaperone binding, unfolded protein binding activity and role in chaperone-mediated protein complex assembly, protein folding, protein import into mitochondrial intermembrane space, protein refolding

PIS52081.1	29.6713022	29.9755323	29.6065379	27.36056778	29.51553261	29.28612548	0.078468519	0.939	-1.03	PIS52081.1	PMT5	Protein mannosyltransferase (PMT), expressed at extremely low levels; not required for wild-type hyphal growth, drug resistance, or virulence in mouse systemic infection; one of five PMT family members
PIS56561.1	28.9114032	30.3684673	29.8206446	28.87537931	28.37462015	28.77389988	0.025016191	0.901	-1.03	PIS56561.1	PNP1	Purine nucleoside phosphorylase; metabolizes inosine and guanosine nucleosides; in the nicotinamide riboside salvage pathway; Spider biofilm repressed
PIS48320.1	25.4485451	28.332278	28.5421663	24.36322753	27.48766653	27.39101604	0.252621297	0.963	-1.03	PIS48320.1	RPC40	Putative RNA polymerase; protein level decreases in stationary phase cultures; Hap43p-induced gene
PIS52230.1	25.340434	27.4149346	27.4717106	25.94181738	26.01623569	25.18368807	0.336551347	0.967	-1.03	PIS52230.1	TRM9	Putative tRNA methyltransferase; repressed during the mating process
PIS51056.1	29.0235077	29.8918303	30.9200678	27.92245774	29.39054261	29.42959718	0.152202221	0.954	-1.03	PIS51056.1	XYL2	D-xylulose reductase; immunogenic in mice; soluble protein in hyphae; induced by caspofungin, fluconazole, Hog1 and during cell wall regeneration; Mnl1-induced in weak acid stress; stationary phase enriched; flow model biofilm induced
PIS48332.1	29.6394601	29.0999896	28.8306936	28.78902543	27.71804266	27.98983572	0.020465721	0.892	-1.02	PIS48332.1	BNA4	Putative kynurenine 3-monooxygenase, involved in NAD biosynthesis; transposon mutation affects filamentous growth; Hap43p-repressed gene; oral infection upregulated; mutants have reduced capacity to damage oral epithelial cells
PIS52158.1	29.7826991	30.0241516	30.3914201	28.49298725	29.42027019	29.23048416	0.041309	0.92	-1.02	PIS52158.1	CCT6	Putative cytosolic chaperonin Cct ring complex subunit; mutation confers hypersensitivity to cytochalasin D; GlcNAc-induced protein; Spider biofilm repressed

PIS58992.1	26.4051628	27.1133083	27.877509	26.11595694	25.77553051	26.45228346	0.028343786	0.906	-1.02	PIS58992.1	FGR32	Protein similar to S. cerevisiae Swa2p; induced upon adherence to polystyrene; transposon mutation affects filamentous growth; Hap43p-repressed gene
PIS51222.1	25.1135124	25.6252634	25.9544995	24.09023522	24.83274764	24.69552446	0.031104432	0.91	-1.02	PIS51222.1	orf19.2266	Ortholog(s) have ATPase, DNA binding, nucleosome binding activity, role in sister chromatid cohesion and Isw1a complex localization
PIS51246.1	29.4269555	28.87562	29.8931938	28.19360241	28.34633328	28.58887702	0.014009208	0.872	-1.02	PIS51246.1	PEX12	Ortholog(s) have ubiquitin ligase activator activity, ubiquitin protein ligase activity and role in proteasome-mediated ubiquitin-dependent protein catabolic process, protein import into peroxisome matrix, protein polyubiquitination
PIS51220.1	23.5675178	23.7262325	27.1019842	21.36319707	24.48170833	25.4785922	0.348878547	0.967	-1.02	PIS51220.1	RFA2	Putative DNA replication factor A; RNA abundance regulated by cell cycle, tyrosol and cell density
PIS51293.1	26.1126783	25.633889	24.2723947	25.44562246	22.96200738	24.55242177	0.249324591	0.962	-1.02	PIS51293.1	STF2	Protein involved in ATP biosynthesis; repressed in hyphae; repressed by Efg1, Hap43; transcript upregulated in clinical isolates from HIV+ patients with oral candidiasis; rat catheter, flow model and Spider biofilm induced
PIS56733.1	26.3593223	26.7048637	26.8083075	23.96865644	25.97658654	26.85340369	0.152753738	0.954	-1.02	PIS56733.1	VPS53	Subunit of GARP (Golgi-associated retrograde protein) complex, which has roles in Golgi to vacuole transport, cellular sphingolipid homeostasis, retrograde transport, and filamentous growth
PIS58470.1	32.1024146	30.6228477	30.4133962	30.50532957	29.8066115	29.78654198	0.047911168	0.925	-1.01	PIS58470.1	ENA2	Putative sodium transporter; induced by ciclopirox olamine; alkaline induced by Rim101; repressed by high-level peroxide stress; induced in oral candidiasis clinical isolates; possibly essential gene; rat catheter and Spider biofilm induced

PIS51050.1	25.4507056	26.0828333	24.9416007	25.42650062	24.68965638	23.33375801	0.103119616	0.946	-1.01	PIS51050.1	orf19.2513	Protein required for expression of NADH:ubiquinone oxidoreductase (mitochondrial complex I)
PIS58457.1	34.388905	34.2023901	34.4453182	33.45761326	33.28478937	33.27535962	0.002746938	0.73	-1.01	PIS58457.1	orf19.3690.2	Ribosomal 60S subunit protein; Spider biofilm repressed
PIS48773.1	29.6326819	29.9006951	30.0457305	29.23200314	29.08710775	28.21890498	0.049924636	0.927	-1.01	PIS48773.1	PIM1	ATP-dependent Lon protease; role in degradation of misfolded proteins in mitochondria, biogenesis and maintenance of mitochondria; rat catheter biofilm induced
PIS48679.1	28.9620764	28.4814618	28.4379362	28.16923957	27.41001329	27.27989633	0.036947134	0.916	-1.01	PIS48679.1	PTK2	Putative protein kinase of polyamine import; mutation confers hypersensitivity to high concentrations of tunicamycin; YPD flow model biofilm induced; rat catheter and Spider biofilm induced
PIS51690.1	30.7486645	30.0895165	30.4486677	29.47691702	29.78169273	28.99984485	0.119182441	0.949	-1.01	PIS51690.1	RTF1	Putative RNA polymerase II-associated Paf1 complex subunit; induced during the mating process
PIS49722.1	27.4993887	28.1267677	28.9553738	27.91785828	26.8231157	26.82363521	0.025923563	0.903	-1.01	PIS49722.1	SSN6	Functional homolog of S. cerevisiae Cyc8/Ssn6; hyphal growth regulator; repressed during hyphal growth; Ssn6 and Tup1 regulate distinct sets of genes; overexpression or mutation causes avirulence in mouse IV infection; TPR motifs
PIS54843.1	32.5267892	32.7028643	32.4671178	31.43525029	31.61354155	31.61717713	0.000953971	0.572	-1.01	PIS54843.1	UGP1	UTP-glucose-1-phosphaturidyl transferase; localizes to yeast, not hyphal cell surface; Hog1-repressed; stationary phase enriched; induced in oralpharyngeal candidasis; rat catheter biofilm repressed; Bcr1-repressed in RPMI a/a biofilms

PIS58043.1	30.0636402	29.7402884	30.0655463	28.30367254	29.03551974	29.51500933	0.010823764	0.856	-1.01	PIS58043.1	VPS17	Ortholog(s) have phosphatidylinositol-3-phosphate binding, protein carrier activity
PIS51541.1	27.349331	28.3361139	27.6250253	26.14583344	27.02868773	27.09831262	0.227644553	0.961	-1.01	PIS51541.1	YAK1	Putative dual-specificity tyrosine-phosphorylation regulated kinase; involved in hyphal growth regulation and biofilm formation; appears to act either downstream or in parallel with the RAS/cAMP/PKA pathway
PIS58264.1	25.443429	23.7947644	26.7117337	23.37390592	24.89745131	24.67944021	0.295673406	0.965	-1	PIS58264.1	MGE1	Putative mitochondrial matrix cochaperone; overexpression increases resistance to fluconazole; macrophage/pseudohyphal-repressed
PIS48824.1	26.6465536	25.8836688	26.667169	23.53360897	26.24830754	26.41679298	0.254718991	0.963	-1	PIS48824.1	orf19.4816	Protein of unknown function; induced by nitric oxide
PIS54547.1	33.3406434	33.5052464	33.3110823	32.06838234	32.54699395	32.54610726	0.001655579	0.661	-0.998	PIS54547.1	BMH1	Sole 14-3-3 protein in C. albicans; role in hyphal growth; possibly regulated by host interaction; localizes to yeast-form cell surface, not hyphae; alternatively spliced 5' UTR intron; Spider biofilm repressed
PIS48583.1	28.9106631	29.6738707	30.8425445	27.99700639	29.21014867	29.24738457	0.089832786	0.943	-0.991	PIS48583.1	MRP2	Protein similar to S. cerevisiae Mrp2p, which is a component of the small subunit of the mitochondrial ribosome; transposon mutation affects filamentous growth
PIS55796.1	32.4752171	32.5690831	32.3281398	31.39742978	31.535488	31.46594632	0.003758752	0.766	-0.991	PIS55796.1	orf19.1672	Alpha subunit of COPI vesicle coatomer complex; role in ER to Golgi vesicle-mediated transport, retrograde vesicle-mediated transport, Golgi to ER transport; flow model biofilm repressed

PIS51679.1	32.8052719	33.0274075	33.150296	32.04133391	32.02391814	31.94685718	0.001377705	0.633	-0.99	PIS51679.1	GPH1	Putative glycogen phosphorylase; role in glycogen metabolism; regulated by Ssk1, Mig1, Tup1, Hap43; fluconazole-induced; localizes to cell surface of hyphae, not yeast; stationary phase enriched protein; Spider biofilm induced
PIS56640.1	25.0007394	26.2364999	26.3146538	25.23901015	24.79295219	24.54939324	0.055216763	0.93	-0.99	PIS56640.1	MCI4	Putative NADH-ubiquinone dehydrogenase; Hap43p-repressed gene
PIS50328.1	24.3446964	25.8113519	26.1674247	25.78686038	22.59695817	24.97195842	0.278272661	0.964	-0.989	PIS50328.1	orf19.4929	Ortholog(s) have mRNA binding, translation regulator activity, role in mitochondrial cytochrome c oxidase assembly, positive regulation of mitochondrial translational initiation and mitochondrial inner membrane localization
PIS51615.1	27.87197	28.9638334	30.4826633	28.03040323	28.09268176	28.23152823	0.129681478	0.951	-0.988	PIS51615.1	PUF3	RNA-binding protein involved in regulation of mitochondrial biogenesis
PIS58736.1	32.1822427	33.0585097	32.2222028	31.41409536	31.5541854	31.53812369	0.030787133	0.91	-0.986	PIS58736.1	RPS7A	Ribosomal protein S7; genes encoding cytoplasmic ribosomal subunits, translation factors, and tRNA synthetases are downregulated upon phagocytosis by murine macrophage; Spider biofilm repressed
PIS52187.1	28.4765975	29.2059314	29.7895992	27.77318449	27.9225232	28.82210665	0.052026586	0.928	-0.985	PIS52187.1	orf19.1777	Ortholog(s) have cysteine-type deubiquitinase activity, deubiquitinase activity, endopeptidase activity, metal-dependent deubiquitinase activity
PIS51825.1	29.9366657	30.3161971	30.0456844	29.3262733	29.1169241	28.89914049	0.014055971	0.872	-0.985	PIS51825.1	SIN3	Protein similar to S. cerevisiae Sin3p (transcriptional corepressor involved in histone deacetylase recruitment); has paired amphipathic helix PAH1 domain; interacts with ScOpi1p, not CaOpi1p; transposon mutation affects filamentous growth

PIS54585.1	26.8370545	28.0186679	28.4263147	26.59690242	26.63515173	27.0980059	0.343169333	0.967	-0.984	PIS54585.1	AYR1	Putative oxidoreductase; transcriptionally induced by interaction with macrophage; rat catheter biofilm repressed
PIS55783.1	31.3712224	31.7423076	31.8977407	30.47325456	31.58680913	30.00352966	0.098577354	0.945	-0.983	PIS55783.1	CRC1	Putative sodium transporter; induced by ciclopirox olamine; alkaline induced by Rim101; repressed by high-level peroxide stress; induced in oral candidiasis clinical isolates; possibly essential gene; rat catheter and Spider biofilm induced
PIS52139.1	25.8517265	26.29205	23.8450166	24.13171066	24.40544651	24.50817537	0.267661672	0.963	-0.981	PIS52139.1	orf19.5921	Ortholog of C. dubliniensis CD36 : Cd36_84580, C. parapsilosis CDC317 : CPAR2_404290, C. auris B8441 : B9J08_003750 and Candida tenuis NRRL Y-1498 : CANTEDRAFT_115338
PIS49837.1	25.349095	28.0603477	28.8930414	26.61611063	25.89848298	26.84700845	0.200453787	0.959	-0.98	PIS49837.1	orf19.5574	Has domain(s) with predicted role in cysteine biosynthetic process from serine
PIS48271.1	33.1193374	33.5499985	33.766933	32.57721138	32.42385168	32.49702747	0.004365785	0.782	-0.979	PIS48271.1	RPS1	Putative ribosomal protein 10 of the 40S subunit; elicits host antibody response during infection; transcript induced during active growth; Spider biofilm repressed
PIS48705.1	24.5446104	25.3526262	26.8657887	24.73509935	24.46754561	24.62861834	0.148037282	0.953	-0.977	PIS48705.1	orf19.3449.2	Putative mitochondrial phosphatidylglycerophosphatase (PGP phosphatase); essential for cardiolipin biosynthesis; rat catheter biofilm induced
PIS49492.1	24.2244357	24.7828207	24.5552766	24.45278067	23.61936999	22.55926573	0.099350445	0.945	-0.977	PIS49492.1	orf19.419	Protein of unknown function; flow model biofilm induced; Spider biofilm induced

PIS51226.1	23.9910037	23.9429314	25.2783342	23.64155923	22.80870038	23.83262997	0.114284313	0.948	-0.976	PIS51226.1	orf19.2261	Ortholog(s) have RNA binding activity, role in mRNA splicing, via spliceosome and U2 snRNP, U2-type prespliceosome localization
PIS51090.1	25.8980541	27.3331371	26.4006301	25.53074174	25.94386729	25.24801005	0.230294184	0.961	-0.97	PIS51090.1	orf19.4680	Possile protease; mutation confers hypersensitivity to toxic ergosterol analog
PIS52067.1	27.3140508	27.8302683	28.0161415	25.47997582	27.02408349	27.75242269	0.279786697	0.964	-0.968	PIS52067.1	ANT1	Peroxisomal adenine nucleotide transporter; role in beta-oxidation of medium-chain fatty acid and peroxisome proliferation; rat catheter biofilm induced
PIS56848.1	26.2172811	26.0498921	26.5415172	25.39269256	26.07137981	24.4448199	0.040792971	0.92	-0.967	PIS56848.1	CYT2	Cytochrome c1 heme lyase; transcript regulated by Nrg1; induced in high iron
PIS58272.1	31.2618439	31.5211834	31.4983892	30.12466648	30.66424422	30.60347816	0.079142211	0.94	-0.963	PIS58272.1	AAT22	Aspartate aminotransferase; nitrogen metabolism; similar but not orthologous to S. cerevisiae Aat2; clade-associated gene expression; protein levels decrease in stationary phase yeast; mutant is viable; flow model biofilm repressed
PIS56618.1	32.7406168	32.9772056	33.3465347	32.16217889	32.13870633	31.87327226	0.002109005	0.696	-0.963	PIS56618.1	GUS1	Putative glutamine-tRNA ligase; stationary phase enriched protein; Spider biofilm repressed
PIS52474.1	28.3036089	28.3663856	28.5390941	28.45869424	27.0263914	26.839374	0.071126856	0.937	-0.962	PIS52474.1	orf19.2821	Protein of unknown function; Hap43-repressed gene; repressed by nitric oxide

PIS51019.1	24.0667036	24.431641	24.9855528	23.44719182	24.03752179	23.1154395	0.024741416	0.901	-0.961	PIS51019.1	orf19.6076	Ortholog(s) have cargo adaptor activity, role in intracellular protein transport, retrograde transport, endosome to Golgi and endosome, retromer complex, retromer, cargo-selective complex localization
PIS48306.1	30.5089459	31.4087119	32.0183897	29.60706415	30.79935767	30.64913696	0.106036001	0.946	-0.96	PIS48306.1	CCT2	Chaperonin of the cytosolic TCP1 ring complex; protein present in exponential and stationary-phase yeast cells, but higher amounts in stationary phase; GlcNAc-induced protein
PIS54641.1	33.9689571	34.0687877	34.2252789	33.23261049	33.09159303	33.05998864	0.001999623	0.688	-0.96	PIS54641.1	FAS2	Alpha subunit of fatty-acid synthase; required for virulence in mouse systemic infection and rat oropharyngeal infection models; regulated by Efg1; fluconazole-induced; amphotericin B repressed; flow model and Spider biofilm
PIS58581.1	27.6119068	29.008648	29.4006281	26.96750776	27.828247	28.34832568	0.056254992	0.93	-0.959	PIS58581.1	orf19.5194.1	Putative protein of unknown function; clade-associated gene expression
PIS55548.1	29.2442727	29.3052262	30.4626897	28.86698887	28.68232829	28.5903431	0.01983439	0.89	-0.958	PIS55548.1	orf19.2101	Ortholog(s) have protein transmembrane transporter activity, protein-membrane adaptor activity
PIS51906.1	29.6177059	30.2756475	30.2301201	28.17252962	29.71801132	29.35999096	0.063595026	0.934	-0.958	PIS51906.1	SUB2	Putative TREX complex component with a predicted role in nuclear mRNA export; transcription is regulated by Mig1; Hap43-induced gene; Spider biofilm repressed
PIS58996.1	32.1027533	31.828841	32.4481283	30.84983009	31.52162015	31.13672185	0.016704695	0.882	-0.957	PIS58996.1	EMP24	COPII-coated vesicle component

PIS58748.1	26.9973626	26.8679322	26.6748832	26.62042308	25.16769226	25.8911471	0.031996178	0.911	-0.954	PIS58748.1	SKI8	Ortholog(s) have role in nuclear-transcribed mRNA catabolic process, 3'-5' exonucleolytic nonsense-mediated decay, nuclear-transcribed mRNA catabolic process, exonucleolytic and 3'-5', more
PIS51119.1	31.4518997	31.93647	32.6445698	30.43745684	31.44473259	31.2887434	0.049254878	0.926	-0.954	PIS51119.1	YHB1	Nitric oxide dioxygenase; acts in nitric oxide scavenging/detoxification; role in virulence in mouse; transcript activated by NO, macrophage interaction; Hap43, hypha repressed; mRNA binds She3
PIS48343.1	28.4606282	28.8504719	28.3629971	26.0526802	27.89825443	28.87760613	0.123625893	0.95	-0.949	PIS48343.1	PR26	Protein involved in resistance to caspofungin and anidulafungin; has similarity to proteasomal 26S regulatory subunit of S. cerevisiae, H. sapiens, Methanobacterium thermoautotrophicum (Archaebacterium)
PIS59010.1	31.3061207	31.0156089	31.0604995	30.29057358	30.03391027	30.22326576	0.002592975	0.723	-0.945	PIS59010.1	STT4	Phosphatidylinositol-4-kinase; forms a complex with Ypp1p and Efr3p that is required for phosphatidylinositol-4-phosphate, PI(4)P, in plasma membrane; required for invasive growth and cell wall organization
PIS48542.1	26.7988468	24.1681135	25.2515354	24.25688124	24.75616564	24.37251286	0.236779089	0.962	-0.944	PIS48542.1	TOA2	Putative TFIIA small subunit; protein abundance decreased in CAI4 strain compared to the SC5314 strain, abundance not affected by reintegration of URA3 in CAI4; flucytosine induced; possibly an essential gene (UAU1 method)
PIS58094.1	32.1361915	32.0605793	31.3754774	30.92940971	31.12704108	30.6922419	0.016010598	0.879	-0.941	PIS58094.1	orf19.6143	Predicted long-chain-alcohol oxidase; Spider biofilm induced
PIS51138.1	29.0181663	26.8655258	26.7982631	27.31459412	26.52272953	26.0272795	0.264986879	0.963	-0.939	PIS51138.1	orf19.5131	Ortholog of S. cerevisiae Gid7, a GID complex protein; involved in proteasome-dependent catabolite inactivation of fructose-1,6-bisphosphatase; Hap43-repressed gene

PIS51647.1	27.7059521	27.8830114	27.1093663	27.37016574	26.73401515	25.77858856	0.14856466	0.953	-0.939	PIS51647.1	orf19.6039	Ortholog(s) have SNAP receptor activity
PIS48460.1	28.9185492	28.8197543	28.8517424	28.32813607	27.54959015	27.89548705	0.010313145	0.853	-0.939	PIS48460.1	RPL82	Predicted ribosomal protein; genes encoding cytoplasmic ribosomal subunits, translation factors, and tRNA synthetases are downregulated upon phagocytosis by murine macrophage
PIS56616.1	26.2644764	27.6026537	28.8404246	27.08837936	26.00588929	26.80383531	0.143766204	0.953	-0.936	PIS56616.1	orf19.1862	Possible stress protein; increased transcription associated with CDR1 and CDR2 overexpression or fluphenazine treatment; regulated by Sfu1, Nrg1, Tup1; stationary phase enriched protein; Spider biofilm induced
PIS58463.1	28.7557986	29.9871693	31.6207426	29.27803757	29.15120476	29.1326948	0.326187006	0.966	-0.934	PIS58463.1	orf19.2928	Has domain(s) with predicted role in retrograde vesicle-mediated transport, Golgi to endoplasmic reticulum and membrane localization
PIS50606.1	34.5997915	34.6423906	35.044547	33.82349096	33.76087206	33.90664646	0.004876443	0.792	-0.932	PIS50606.1	RPL8B	Predicted ribosomal protein; regulated upon yeast-hypha switch; repressed upon phagocytosis by murine macrophage; Spider biofilm repressed
PIS58399.1	25.3673789	26.8431935	26.7835331	25.6440192	25.19137757	25.38130685	0.086910709	0.942	-0.926	PIS58399.1	orf19.7109	Ortholog(s) have myosin I binding activity and cytoplasm localization
PIS56756.1	34.090546	34.3939964	34.6513549	32.97934093	33.65370299	33.73050724	0.020010721	0.891	-0.924	PIS56756.1	FAS1	Beta subunit of fatty-acid synthase; multifunctional enzyme; Hap43, fluconazole-induced; amphotericin B, caspofungin repressed; macrophage/pseudohyphal-induced; flow model and Spider biofilm repressed

PIS52039.1	28.7260189	29.0303532	29.9072548	28.37625885	28.02168976	28.49911866	0.04502425	0.923	-0.922	PIS52039.1	CPR3	Putative peptidyl-prolyl cis-trans isomerase; macrophage-induced protein; protein levels decrease in stationary phase yeast cultures; predicted mitochondrial localization; overlaps orf19.1551
PIS52460.1	27.5446856	27.7088381	28.9970804	27.68214041	27.00028631	26.80249742	0.072002386	0.937	-0.922	PIS52460.1	orf19.2639.1	Lsm (Like Sm) protein; predicted role in involved in mRNA decay; Spider biofilm repressed
PIS58192.1	28.5746296	29.5279652	30.1409531	27.53515168	28.39274772	29.55149183	0.129311509	0.951	-0.921	PIS58192.1	GLO2	Ortholog(s) have hydroxyacylglutathione hydrolase activity, role in methylglyoxal catabolic process to D-lactate via S-lactoyl-glutathione and cytoplasm localization
PIS51314.1	23.4692964	25.5822793	26.0684592	22.78830164	25.54131301	24.03093175	0.27121988	0.964	-0.92	PIS51314.1	orf19.4901	Predicted methyltransferase; Spider biofilm induced
PIS52202.1	30.2547754	29.4960064	29.4584961	28.36496423	29.1755341	28.9128373	0.099823489	0.945	-0.919	PIS52202.1	orf19.6934	Ortholog(s) have role in protein insertion into mitochondrial inner membrane from matrix and mitochondrial inner membrane localization
PIS58204.1	29.5554645	30.4811032	31.8898153	29.09933269	30.28231783	29.79125832	0.129920442	0.951	-0.918	PIS58204.1	MDH1	Mitochondrial malate dehydrogenase; regulated by Mig1, Tup1, white-opaque switch, phagocytosis; induced in high iron; antigenic during murine and human infection; repressed in Spider biofilms by Bcr1, Tec1, Ndt80, Rob1, Brg1
PIS49797.1	30.1940056	28.7668504	28.681972	28.51906563	28.52238002	27.85003574	0.053182617	0.929	-0.917	PIS49797.1	AGE2	Ortholog(s) have GTPase activator activity and role in endoplasmic reticulum to Golgi vesicle-mediated transport, intra-Golgi vesicle-mediated transport

PIS51547.1	32.8038856	32.8214229	33.2308271	31.69694774	32.33569766	32.07114429	0.006468264	0.818	-0.917	PIS51547.1	ATP4	Putative F0-ATP synthase subunit 4; macrophage/pseudohyphal-induced; present in exponential and stationary growth phases
PIS51769.1	32.4161322	32.1106773	32.7366754	32.20881275	31.19555131	31.10684352	0.047716239	0.925	-0.917	PIS51769.1	HHT2	Putative histone H3; farnesol regulated; Hap43-induced; rat catheter and Spider biofilm repressed
PIS55515.1	27.9738798	27.6957683	27.6710276	25.77258758	27.61879151	27.1987865	0.040896533	0.92	-0.917	PIS55515.1	PEX3	Putative peroxisomal protein involved in targeting proteins into peroxisomes; possibly an essential gene, disruptants not obtained by UAU1 method
PIS58298.1	23.9426376	24.25871	24.5597782	23.9205802	23.66565017	22.43198869	0.028268153	0.906	-0.914	PIS58298.1	orf19.6709	Predicted alpha/beta hydrolase; Spider biofilm induced
PIS56531.1	27.5745136	26.3970247	28.8143493	25.34055742	27.47397354	27.23193185	0.198262563	0.959	-0.913	PIS56531.1	orf19.5517	Similar to alcohol dehydrogenases; induced by benomyl treatment, nitric oxide; induced in core stress response; oxidative stress-induced via Cap1; Spider biofilm repressed
PIS51478.1	27.1250236	27.9997198	26.3817343	23.09760988	27.60685256	28.06588007	0.382995993	0.968	-0.912	PIS51478.1	orf19.4749	Protein of unknown function; hyphal-induced expression, regulated by Cyr1, Ras1, Efg1; Hap43-induced gene; Spider biofilm induced
PIS49710.1	30.5226424	30.4892462	30.4348019	28.04151767	30.51451722	30.16183159	0.279503953	0.964	-0.91	PIS49710.1	LAT1	Putative dihydrolipoamide acetyltransferase component (E2) of pyruvate dehydrogenase complex; sumoylation target; Spider biofilm repressed

PIS54747.1	26.2689671	25.7809991	25.3163148	25.93601347	25.09122924	23.61314393	0.162520505	0.955	-0.909	PIS54747.1	orf19.446.2	Protein with a NADH-ubiquinone oxidoreductase B18 subunit domain; gene has intron
PIS51632.1	27.0108189	26.9271517	26.9648474	24.8301337	26.61588097	26.7303066	0.063847288	0.934	-0.909	PIS51632.1	SKO1	bZIP transcription factor involved in cell wall damage response; represses the yeast-to-hypha transition; mutants are caspofungin sensitive; induced by osmotic stress via Hog1; activated by Rlm1p; induced by Mnl1 under weak acid stress
PIS49476.1	28.9383387	30.0915565	30.4490554	28.9181212	28.46335787	29.37468826	0.050361411	0.927	-0.908	PIS49476.1	TUB1	Alpha-tubulin; gene has intron; complements cold-sensitivity of S. cerevisiae tub1 mutant; C. albicans has single alpha-tubulin gene, whereas S. cerevisiae has two (TUB1, TUB3); farnesol-upregulated in biofilm; sumoylation target
PIS48341.1	25.4664869	27.2500251	26.2783773	26.4173877	26.15326652	23.70085545	0.517840472	0.972	-0.908	PIS48341.1	UTP5	Putative U3 snoRNA-associated protein; Hap43p-induced gene; mutation confers resistance to 5-fluorocytosine (5-FC), 5-fluorouracil (5-FU), and tubercidin (7-deazaadenosine); physically interacts with TAP-tagged Nop1p [illegible]
PIS48524.1	28.0296156	28.1593498	29.5264733	27.83850525	27.18678677	27.96943178	0.08198172	0.94	-0.907	PIS48524.1	ABZ1	Ortholog(s) have 4-amino-4-deoxychorismate synthase activity and role in para-aminobenzoic acid biosynthetic process
PIS51874.1	29.057033	29.4992042	29.403943	28.38912185	28.65754607	28.19373796	0.009479139	0.847	-0.907	PIS51874.1	ARC19	Putative ARP2/3 complex subunit; Hap43-induced gene; mutation confers hypersensitivity to cytochalasin D; rat catheter biofilm repressed
PIS58059.1	23.3777873	25.0463254	25.961414	23.60639496	22.74573206	25.31773199	0.266463956	0.963	-0.905	PIS58059.1	HCR1	Putative translation initiation factor; repressed upon phagocytosis by murine macrophage; Spider biofilm repressed

PIS55704.1	28.8989102	28.8636186	29.9093736	28.33611689	27.96765885	28.65388122	0.016721417	0.882	-0.905	PIS55704.1	SOU1	Enzyme involved in utilization of L-sorbose; has sorbitol dehydrogenase, fructose reductase, and sorbose reductase activities; NAD-binding site motif; transcriptional regulation affected by chromosome 5 copy number; Hap43p-induced gene
PIS58255.1	26.5777657	28.0199561	26.9140045	26.66121983	26.47701001	25.66282087	0.085678712	0.942	-0.904	PIS58255.1	LRG1	GTPase activator (GAP) that negatively controls small GTPases Cdc42p and Ras1p, involved in signaling pathway that controls morphogenesis in response to environmental signals
PIS58234.1	27.8400664	27.1977639	26.6062237	26.0615382	26.41749659	26.45591512	0.019684557	0.89	-0.903	PIS58234.1	orf19.4764	Ortholog(s) have poly(A)-specific ribonuclease activity, role in nuclear-transcribed mRNA poly(A) tail shortening, postreplication repair and PAN complex localization
PIS51188.1	33.8458598	33.393062	33.2520607	31.9579266	32.85478078	32.976366	0.01686648	0.882	-0.901	PIS51188.1	INO1	Inositol-1-phosphate synthase; antigenic in human; repressed by farnesol in biofilm or by caspofungin; upstream inositol/choline regulatory element; glycosylation predicted; rat catheter, flow model induced; Spider biofilm repressed
PIS58208.1	34.0016959	32.9444403	32.7030851	31.37659796	33.21592317	32.35307696	0.108228193	0.947	-0.901	PIS58208.1	MRPL6	Putative mitochondrial ribosomal protein
PIS50387.1	27.4591509	26.6545242	26.1170045	26.21127994	26.11973748	25.19613428	0.171508816	0.956	-0.901	PIS50387.1	orf19.5295	Protein with a predicted endonuclease/exonuclease/phosphatase family domain and a carbon catabolite repressor protein 4 domain; induced by alpha pheromone in SpiderM medium
PIS52317.1	25.8725615	26.4082384	27.3766291	26.35491842	26.36618037	24.23712566	0.195602662	0.958	-0.9	PIS52317.1	orf19.5238	Ortholog of C. dubliniensis CD36 : Cd36_11490, C. parapsilosis CDC317 : CPAR2_701040, C. auris B8441 : B9J08_003931 and Candida tenuis NRRL Y-1498 : CANTEDRAFT_104365

PIS50513.1	28.794207	29.149314	29.4554295	27.83394053	28.24130665	28.62307465	0.235894057	0.961	-0.9	PIS50513.1	orf19.6239	Putative serine/threonine protein kinase, involved in control of filamentous growth; possibly an essential gene, disruptants not obtained by UAU1 method
PIS58294.1	28.3034084	28.5138149	29.1371578	27.16615746	27.98176217	28.11354467	0.058690621	0.931	-0.898	PIS58294.1	HTS1	Putative tRNA-His synthetase; downregulated upon phagocytosis by murine macrophage; stationary phase enriched protein; Spider biofilm repressed
PIS50529.1	26.6851205	28.4288675	29.5486213	25.47212374	28.1837319	28.31704787	0.453228809	0.971	-0.897	PIS50529.1	orf19.3915	Putative metallodipeptidase; protein present in exponential and stationary growth phase yeast; Hog1-induced; Hap43-repressed; sumoylation target; Spider biofilm repressed
PIS55770.1	32.4216008	32.9699316	34.1848052	32.27852278	32.24405842	32.36327405	0.229157043	0.961	-0.897	PIS55770.1	TPI1	Triose-phosphate isomerase; antigenic in mouse/human; mutation affects filamentation; macrophage-repressed; protein in exponential and stationary growth phase yeast; possibly essential; flow model biofilm induced; Spider biofilm repressed
PIS58318.1	30.6864766	31.572965	31.8301464	29.19581623	31.10625283	31.10162106	0.176304716	0.957	-0.895	PIS58318.1	GRE3	Putative D-xylose reductase; antigenic in murine systemic infection; soluble protein in hyphae; induced by farnesol, macrophage interaction and by Mnl1 under weak acid stress; stationary-phase enriched protein; Spider biofilm induced
PIS51784.1	25.0850882	26.1810458	25.52027	24.3396199	24.54257843	25.21802406	0.241750133	0.962	-0.895	PIS51784.1	SIT4	Serine/threonine protein phosphatase catalytic subunit; has a role in cell wall maintenance, hyphal growth, and virulence in a mouse systemic infection model
PIS58515.1	23.8740613	23.5007282	24.3223955	23.67712259	23.21458648	22.12355876	0.124106572	0.95	-0.894	PIS58515.1	HGT13	Predicted sugar transporter, involved in glycerol utilization; member of the major facilitator superfamily; 12 transmembrane; gene has intron; oxidative stress-induced via Cap1p; expressed in rich medium, 2% glucose

PIS58984.1	25.7536274	25.1075944	25.2499549	24.74228254	24.8435022	23.84200704	0.177067718	0.957	-0.894	PIS58984.1	orf19.318	Ortholog(s) have role in protein maturation by [2Fe-2S] cluster transfer, protein maturation by [4Fe-4S] cluster transfer and mitochondrial matrix localization
PIS56929.1	23.8656624	25.5517082	21.0762691	23.66907578	21.37988046	22.76881759	0.37290309	0.968	-0.892	PIS56929.1	orf19.4031	Ortholog(s) have steryl-beta-glucosidase activity, role in ergosteryl 3-beta-D-glucoside catabolic process and cytosol localization
PIS48334.1	29.5456768	29.6091673	31.2904121	29.8180889	28.78828	29.16602531	0.129749014	0.951	-0.891	PIS48334.1	GLO3	Putative ARF GTPase activator; role in COPI coating of Golgi vesicle, ER to Golgi vesicle-mediated transport, retrograde Golgi to ER vesicle-mediated transport; Spider biofilm repressed
PIS56703.1	28.6716609	29.8665652	30.2323816	29.86446887	28.68466057	27.5507377	0.12817272	0.951	-0.89	PIS56703.1	EIF4E	Translation initiation factor eIF4E; genes encoding ribosomal subunits, translation factors, tRNA synthetases downregulated by phagocytosis by macrophage; alternatively spliced intron in 5' UTR; protein levels decrease in stationary phase
PIS58800.1	32.2593667	31.4434461	30.7532132	29.77897866	31.36490846	30.64228787	0.272935927	0.964	-0.89	PIS58800.1	orf19.2333	Ortholog(s) have role in retrograde transport, endosome to Golgi and cytoplasm, late endosome localization
PIS58491.1	28.8700923	29.2727429	29.1653862	28.581559	28.08448233	27.98369652	0.056309447	0.93	-0.886	PIS58491.1	BIO32	Putative class III aminotransferase with a predicted role in biotin biosynthesis; Spider biofilm induced
PIS54601.1	26.6823743	27.5812264	25.8011565	27.3671023	25.51088002	24.52735918	0.224991848	0.961	-0.886	PIS54601.1	LIP1	Secreted lipase, member of a lipase gene family whose members are expressed differentially in response to carbon source and during infection; may have a role in nutrition and/or in creating an acidic microenvironment

PIS49647.1	33.5775318	33.5159579	33.076003	32.59791767	32.75134349	32.16210253	0.006347012	0.816	-0.886	PIS49647.1	orf19.3061.1	Ortholog of S. cerevisiae Rps22Ap and Rps22Bp; gene contains 5' UTR intron
PIS59001.1	26.6607449	27.7202912	27.9713329	28.73808838	25.61261063	25.34450652	0.240612283	0.962	-0.886	PIS59001.1	orf19.6284	Ortholog(s) have signal recognition particle binding activity, role in protein targeting to ER and endoplasmic reticulum membrane, signal recognition particle receptor complex localization
PIS58319.1	28.2470476	28.0982698	27.2738263	27.26590234	27.12534748	26.57347972	0.055636369	0.93	-0.885	PIS58319.1	orf19.4316	Trimethyllysine dioxygenase, the first enzyme in the carnitine biosynthesis pathway; hypha-induced expression, regulated by Cyr1, Ras1, Efg1; rat catheter biofilm repressed
PIS58820.1	31.7093599	33.609617	34.2254051	31.36603152	32.54126944	32.9843909	0.283091852	0.964	-0.884	PIS58820.1	ENO1	Enolase, involved in glycolysis and gluconeogenesis; also has transglutaminase activity involved in assembly of cell wall polysaccharides; major cell-surface antigen; binds host plasmin/plasminogen; immunoprotective; may be essential [illegible]
PIS48279.1	29.5033234	29.2777658	30.3895058	28.71527444	28.81107791	28.99329245	0.037450353	0.917	-0.884	PIS48279.1	orf19.5799	Ortholog of S. cerevisiae : TPH3, C. dubliniensis CD36 : Cd36_17790, C. parapsilosis CDC317 : CPAR2_212150, C. auris B8441 : B9J08_004964 and Candida tenuis NRRL Y-1498 : CANTEDRAFT_128815 [illegible]
PIS51326.1	29.212203	29.1608808	29.268895	28.13257402	28.40669122	28.44936174	0.024563173	0.9	-0.884	PIS51326.1	orf19.6830	Putative enoyl-CoA hydratase; Spider biofilm induced
PIS50631.1	24.4723034	24.4721203	27.2688492	24.33033407	24.39553165	24.84161408	0.260181575	0.963	-0.882	PIS50631.1	ILV6	Putative regulatory subunit of acetolacetate synthase; alkaline induced; regulated by Gcn2 and Gcn4; protein present in exponential and stationary growth phase yeast; Spider biofilm repressed

PIS55681.1	26.2401748	24.845286	24.463463	25.05576722	25.13881352	22.71252697	0.303747589	0.965	-0.881	PIS55681.1	orf19.2749	BTB/POZ domain protein; induced by Mnl1 under weak acid stress; flow model biofilm induced; Spider biofilm induced
PIS56849.1	27.2762463	27.7933154	28.0140481	26.81531724	26.55359073	27.0747172	0.264040173	0.963	-0.88	PIS56849.1	ERV29	Putative SURF4 family member; plasma membrane-localized; flow model biofilm repressed
PIS56762.1	24.3977615	24.1705938	25.2304125	25.31115992	23.36623878	22.48620076	0.264326978	0.963	-0.878	PIS56762.1	BRE1	Putative transcription factor with C3HC4 zinc finger DNA-binding motif; similar to S. cerevisiae Bre1p; transposon mutation affects filamentous growth
PIS48282.1	32.4694745	32.1725396	32.1953044	31.39980015	31.26732806	31.53626875	0.236421804	0.962	-0.878	PIS48282.1	HYU1	Putative hydantoin utilization protein A; induced upon adherence to polystyrene; regulated by Gcn2p and Gcn4p
PIS48536.1	34.0788068	34.4058469	34.6643129	33.7337517	33.56611409	33.22341668	0.01813331	0.886	-0.875	PIS48536.1	ERG251	C-4 sterol methyl oxidase; role in ergosterol biosynthesis; Hap43-induced; ketoconazole-induced; amphotericin B, caspofungin repressed; possibly essential gene, disruptants not obtained by UAU1 method; Spider biofilm repressed
PIS56603.1	28.4380469	27.9679129	27.3745877	26.55821128	27.63522254	26.96491727	0.04091069	0.92	-0.874	PIS56603.1	TAF4	Putative TFIID subunit; mutation confers hypersensitivity to toxic ergosterol analog
PIS51894.1	33.1079159	33.8315078	34.527305	32.91033447	33.14187802	32.79554533	0.065721086	0.935	-0.873	PIS51894.1	CSP37	Hyphal cell wall protein; role in progression of mouse systemic infection; predicted P-loop, divalent cation binding, N-glycosylation sites; expressed in yeast and hyphae; hyphal downregulated; stationary-phase enriched; GlcNAc-induced

PIS54697.1	23.922857	24.1018202	24.0065681	23.51649753	22.33577285	23.55983092	0.121839857	0.949	-0.873	PIS54697.1	GZF3	GATA-type transcription factor; oxidative stress-induced via Cap1; mutant has abnormal colony morphology and altered sensitivity to fluconazole, LiCl, and copper; Spider biofilm induced
PIS49675.1	23.1701033	25.3549606	24.7491429	24.02413435	23.15031547	23.48172672	0.373362416	0.968	-0.873	PIS49675.1	orf19.6665	Ortholog(s) have small GTPase binding, structural constituent of nuclear pore activity
PIS59021.1	33.8358685	34.2228285	34.7828948	33.31255846	33.53129934	33.38804719	0.021047325	0.893	-0.87	PIS59021.1	ACT1	Actin; gene has intron; transcript regulated by growth phase, starvation; at polarized growth site in budding and hyphal cells; required for wild-type Cdc42 localization; unprocessed N terminus; Hap43-induced; Spider biofilm repressed
PIS51610.1	29.1744383	30.7788482	30.757287	28.73434271	29.40887835	29.96248421	0.089660575	0.943	-0.868	PIS51610.1	LYS1	Saccharopine dehydrogenase (biosynthetic); enzyme of alpha-aminoadipate lysine biosynthesis pathway; functionally complements S. cerevisiae lys1 mutation; fungal-specific (no human or murine homolog)
PIS58618.1	33.029509	33.0968498	32.9966239	32.51556792	32.16885245	31.83417981	0.005523786	0.804	-0.868	PIS58618.1	RPL11	Ribosomal protein; repressed by phagocytosis; colony morphology-related gene regulation by Ssn6; Hap43-induced; Spider biofilm repressed
PIS52161.1	27.1729296	28.4945742	28.5544811	26.76042973	27.34066638	27.52124287	0.129856512	0.951	-0.867	PIS52161.1	orf19.3129	Putative chromatin remodelling complex protein; heterozygous null mutant displays sensitivity to virgineone; Spider biofilm repressed; sumoylation regulated by Hsp90p
PIS58814.1	28.7965292	28.6735162	29.9271651	28.17735529	28.13561306	28.48539413	0.047492363	0.925	-0.866	PIS58814.1	GCF1	HMG box mitochondrial protein; binds to mt DNA and the HWP1 promoter; mutant phenotype and functional complementation of an S. cerevisiae abf2 mutation suggest role in mt genome replication, maintenance; flow and Spider biofilm repressed

PIS48645.1	28.9195013	27.4032477	28.1217452	25.6867791	29.19704551	26.9646386	0.226932104	0.961	-0.865	PIS48645.1	BOI2	Putative SH3-domain-containing protein; mutation confers hypersensitivity to toxic ergosterol analog; Spider biofilm induced
PIS55716.1	26.824114	25.2203864	25.9388742	25.22183999	24.64044216	25.5299441	0.13597	0.952	-0.864	PIS55716.1	ACP1	Putative mitochondrial acyl carrier protein involved in fatty acid biosynthesis; shows colony morphology-related gene regulation by Ssn6p; protein newly produced during adaptation to the serum
PIS52292.1	27.6821307	29.2122051	27.9385002	27.42073512	27.52320399	27.2957782	0.141322146	0.952	-0.864	PIS52292.1	MPP10	Putative SSU processome and 90S preribosome component; repressed in core stress response; repressed by prostaglandins
PIS49852.1	30.2353146	29.873037	29.6817132	28.90748403	29.32341232	28.97319677	0.084113642	0.941	-0.862	PIS49852.1	orf19.5671	Ortholog(s) have enzyme inhibitor activity and role in actin cortical patch assembly, septin cytoskeleton organization
PIS51752.1	29.2453055	30.1256046	30.2607336	28.75912191	29.17229056	29.11801756	0.024968833	0.901	-0.861	PIS51752.1	TRP5	[illegible] identified in detergent-resistant membrane fraction (possible lipid raft component); predicted N-terminal acetylation; Gcn4p-regulated; S. cerevisiae ortholog is Gcn4p regulated; [illegible]
PIS52340.1	31.0045114	31.3811909	31.1166413	29.75815587	30.727315	30.43569552	0.143442295	0.953	-0.86	PIS52340.1	RAX1	Protein with a predicted role in bud site selection; hypha-induced expression; Spider biofilm induced
PIS58254.1	26.912651	27.2286564	28.4568847	26.19892389	27.01776395	26.80536985	0.059646299	0.932	-0.859	PIS58254.1	orf19.7489.3	Ortholog(s) have 2 iron, 2 sulfur cluster binding, iron-sulfur cluster binding activity

PIS48531.1	30.2406033	30.7905509	32.1312449	29.6678182	30.29943838	30.61863729	0.080992121	0.94	-0.859	PIS48531.1	SHM2	Cytoplasmic serine hydroxymethyltransferase; complements glycine auxotrophy of S. cerevisiae shm1 shm2 gly1-1 mutant; antigenic; farnesol-upregulated in biofilm; stationary-phase enriched protein; rat catheter and Spider biofilm
PIS56530.1	24.4380967	23.6539106	28.1910772	23.60620898	25.40833679	24.69515113	0.563658492	0.973	-0.858	PIS56530.1	orf19.5510	Ortholog(s) have role in negative regulation of transcription by RNA polymerase II, subtelomeric heterochromatin formation and CHRAC localization
PIS54720.1	27.2355773	28.284962	28.8616765	26.02621274	27.7886729	27.9948585	0.12843599	0.951	-0.857	PIS54720.1	orf19.2671	Protein with a predicted FAD-dependent pyridine nucleotide reductase domains; putative oxidoreductase; Plc1-regulated; possibly an essential gene, disruptants not obtained by UAU1 method
PIS58976.1	25.8360747	25.9444589	24.0944106	24.47003422	24.24215376	24.5926287	0.295344745	0.965	-0.857	PIS58976.1	orf19.933	Predicted ubiquitin conjugating enzyme involved in DNA damage response; forms a complex with Mms2p
PIS49478.1	31.5574596	31.4609064	31.7516732	30.29937707	30.85589157	31.04624	0.061756614	0.933	-0.856	PIS49478.1	ALO1	D-Arabinono-1,4-lactone oxidase involved in biosynthesis of dehydro-D-arabinono-1,4-lactone, which has a protective role against oxidative damage; plasma membrane-localized;required for full virulence in a mouse model of systemic infection
PIS58169.1	29.0245374	29.7301692	31.0421368	28.74596963	29.15144577	29.33512706	0.322651566	0.966	-0.855	PIS58169.1	orf19.458	Ortholog(s) have ATPase-coupled transmembrane transporter activity, protein transmembrane transporter activity
PIS48294.1	27.660325	28.022946	28.59275	26.75118299	27.39545707	27.56446169	0.064998643	0.934	-0.855	PIS48294.1	RPP1	Putative ortholog of S. cerevisiae Rpp1; subunit of both RNase MRP and nuclear RNase P; rat catheter and Spider biofilm induced

PIS58699.1	26.8157462	26.873322	25.9706409	24.80539906	26.4407761	25.85131979	0.062877271	0.933	-0.854	PIS58699.1	orf19.1477	Protein of unknown function; possible ER protein; Hap43p-repressed; Spider biofilm induced
PIS52113.1	27.6024084	27.8219307	27.7747528	27.37816068	26.4185673	26.84177076	0.012888975	0.867	-0.854	PIS52113.1	orf19.4516	Ortholog(s) have tetrahydrofolylpolyglutamate synthase activity, role in one-carbon metabolic process and cytoplasm, mitochondrion localization
PIS55771.1	31.0176059	30.3767462	29.6250799	27.08201209	30.16704824	31.20962549	0.429399858	0.97	-0.854	PIS55771.1	orf19.6747	Ortholog(s) have acid phosphatase activity and role in intracellular sterol transport
PIS51724.1	27.6703208	27.8743011	28.7220275	27.00019648	27.27364419	27.43228306	0.080474788	0.94	-0.854	PIS51724.1	PAM16	Putative maltase; regulated by Gcn4; repressed by amino acid starvation (3-AT); rat catheter biofilm induced
PIS55657.1	27.1074952	28.0568476	28.9014347	27.88589392	27.70036122	25.91813636	0.212114057	0.96	-0.854	PIS55657.1	URA5	Putative orotate phosphoribosyltransferase; protein abundance is affected by URA3 expression in the CAI-4 strain background; flucytosine induced; protein level decreased in stationary phase cultures
PIS48459.1	25.0547061	25.8282871	26.5967663	25.93237429	24.3427423	24.64723027	0.16943415	0.956	-0.852	PIS48459.1	RPL29	Ribosomal protein L29; induced upon germ tube formation; colony morphology-related gene regulation by Ssn6; intron in 5'-UTR; Spider biofilm repressed
PIS48639.1	29.7058837	30.0551099	30.8113722	28.82484529	29.55659164	29.6382088	0.043585082	0.922	-0.851	PIS48639.1	orf19.1632	Has domain(s) with predicted role in carbohydrate metabolic process

PIS50343.1	29.8345181	29.956632	29.5975527	27.44836678	29.40875133	29.9800288	0.230794221	0.961	-0.851	PIS50343.1	RIB4	Lumazine synthase (6,7-dimethyl-8-ribityllumazine synthase, DMRL synthase); catalyzes the penultimate step in the synthesis of riboflavin; Hap43-induced; rat catheter and Spider biofilm repressed
PIS50568.1	25.0295586	25.1641423	26.9164711	25.00021834	24.33423692	25.2265303	0.27028427	0.964	-0.85	PIS50568.1	orf19.3938	Putative mitochondrial ribosomal protein of the small subunit; mutation confers hypersensitivity to 5-fluorocytosine (5-FC), 5-fluorouracil (5-FU), and tubercidin (7-deazaadenosine)
PIS55558.1	31.8949502	32.2978594	32.7656727	31.88821583	31.04507741	31.48038029	0.016578607	0.881	-0.848	PIS55558.1	AMO2	Protein similar to A. niger predicted peroxisomal copper amino oxidase; mutation confers hypersensitivity to toxic ergosterol analog; F-12/CO2 early biofilm induced
PIS58397.1	22.9779813	26.5317893	26.1121938	24.60100668	24.75374136	23.72582618	0.2612478	0.963	-0.847	PIS58397.1	orf19.7108	D-ribulose-5-phosphate 3-epimerase; stationary phase enriched protein
PIS54502.1	30.9931799	31.5404487	31.1955157	30.1003982	30.51838902	30.57634746	0.042336848	0.921	-0.845	PIS54502.1	ARF2	Essential protein, putative ADP-ribosylation factor; involved in invasive growth; mutation confers hypersensitivity to Brefeldin A; Spider biofilm repressed
PIS56860.1	29.1468753	28.8956147	28.1924662	27.8512274	27.94585793	27.90154423	0.020321066	0.891	-0.845	PIS56860.1	orf19.2755	Subunit of the 20S core particle of the proteasome
PIS49548.1	29.2712262	30.0679277	29.506438	29.24996724	28.51945437	28.5441788	0.019309891	0.889	-0.844	PIS49548.1	PRC3	Putative carboxypeptidase Y precursor; transcript regulated by Nrg1 and Mig1; regulated by Gcn2 and Gcn4

PIS49782.1	25.2148836	24.5138976	26.2157969	24.55685063	26.00499469	22.85122328	0.642772336	0.975	-0.844	PIS49782.1	RIT1	Putative initiator tRNA methionine ribosyltransferase; fungal-specific (no human or murine homolog)
PIS54698.1	31.884574	32.0042155	31.604446	31.58003122	30.86833461	30.5190084	0.019176365	0.889	-0.842	PIS54698.1	PGM2	Ortholog of S. cerevisiae Pgm2; induced in planktonic culture; Tye7p-regulated; flow model biofilm induced; rat catheter biofilm repressed
PIS56667.1	26.2276987	25.9703627	27.2334641	26.05363619	25.46509241	25.38642323	0.090645962	0.943	-0.842	PIS56667.1	THI6	Putative thiamin-phosphate pyrophosphorylase, hydroxyethylthiazole kinase; fungal-specific; Spider biofilm induced
PIS49609.1	31.441806	31.4385603	31.7986933	31.09070229	30.70650602	30.35921199	0.019519033	0.89	-0.841	PIS49609.1	AGC1	Putative mitochondrial carrier protein; transcript is alkaline upregulated rat catheter biofilm induced
PIS48661.1	30.2575962	30.0427458	30.4633745	28.87796732	29.58371094	29.77759946	0.092689518	0.943	-0.841	PIS48661.1	orf19.498	Putative mitochondrial ribosomal component of the small subunit; possibly an essential gene, disruptants not obtained by UAU1 method; Spider biofilm repressed
PIS55756.1	28.5356184	28.5004207	27.8627938	26.0958409	28.29312499	27.99521478	0.114570312	0.948	-0.838	PIS55756.1	FAT1	Predicted enzyme of sphingolipid biosynthesis; upregulated in biofilm
PIS54770.1	26.4193453	27.5378849	27.6290808	26.47140743	26.32615305	26.27531195	0.073831188	0.938	-0.838	PIS54770.1	orf19.4230	20S proteasome subunit (beta7); protein present in exponential and stationary growth phase yeast cultures

PIS54880.1	28.8384333	28.7057137	28.8832724	27.47855935	27.95427057	28.48631789	0.033197501	0.913	-0.836	PIS54880.1	ADR1	C2H2 transcription factor; activates genes involved in ergosterol biosynthesis, unlike the S. cerevisiae ortholog which acts in fatty acid metabolism; activation of the protein increases resistance to azoles
PIS58725.1	29.2570186	29.4965559	29.2958595	28.83721927	28.54990128	28.15571916	0.030039098	0.909	-0.836	PIS58725.1	CYS3	Cystathionine gamma-lyase; induced by alkaline, amphotericin B, cadmium stress, oxidative stress via Cap1; possibly adherence-induced; Hog1 regulated; reduced levels in stationary phase yeast cells; Spider and flow model biofilm induced
PIS52056.1	29.7058173	29.5829521	29.6893415	28.59796127	28.94062148	28.94122709	0.013183053	0.868	-0.833	PIS52056.1	YPT1	Functional homolog of S. cerevisiae Ypt1p, which is an essential small Ras-type GTPase involved in protein secretion at ER-to-Golgi; dominant-negative mutation causes SAP secretion defect and accumulation of intracellular secretory vesicles
PIS54748.1	25.9530466	24.8913724	25.305706	25.98221591	25.03131021	22.6417741	0.307748076	0.965	-0.832	PIS54748.1	orf19.446.1	Protein with a NADH-ubiquinone oxidoreductase B18 subunit domain; gene has intron
PIS52008.1	32.5958217	32.6840419	31.911173	31.29665348	31.85145309	31.54895437	0.025608059	0.902	-0.831	PIS52008.1	MED15	RNA polymerase II mediator complex subunit; possibly an essential gene, disruptants not obtained by UAU1 method
PIS55753.1	25.6732196	26.3156308	27.2319302	27.02534455	23.78430127	25.92846798	0.317486215	0.966	-0.828	PIS55753.1	orf19.3648	Ortholog(s) have DNA endonuclease activity, crossover junction DNA endonuclease activity
PIS58531.1	29.6149803	29.6205322	30.6191927	28.34902127	29.63128572	29.39635286	0.230375346	0.961	-0.826	PIS58531.1	RBP1	Peptidyl-prolyl cis-trans isomerase; rapamycin-binding protein; homozygous null mutation confers rapamycin resistance; regulated by Gcn4p; macrophage-induced protein; repressed in response to 3-AT; functional homolog of S. cerevisiae

PIS58042.1	33.0780279	33.6264338	34.3305473	32.3805702	32.7662691	33.41347534	0.123614014	0.95	-0.825	PIS58042.1	SOD2	Mitochondrial Mn-containing superoxide dismutase; protection against oxidative stress; homotetramer active; N-terminal 34 amino acids removed on mitochondrial import; H2O2-induced via Cap1p; Hap43p-, alkaline-downregulated, farnesol-
PIS54722.1	30.886518	30.8195158	30.8115001	30.25917634	29.96839503	29.81769484	0.023954712	0.899	-0.824	PIS54722.1	orf19.3859	Putative microsomal beta-keto-reductase; transcript upregulated by treatment with ciclopirox olamine; induced by alpha pheromone in SpiderM medium; regulated by Sef1, Sfu1, and Hap43
PIS48272.1	30.4503727	30.830656	30.5474843	29.97729114	29.54474064	29.83845251	0.076228415	0.939	-0.823	PIS48272.1	orf19.3003	Tricalbin-family endoplasmic reticulum-plasma membrane tethering protein; required for transport of cell wall proteins; involved in stress responses, including sensitivity to caspofungin
PIS55086.1	29.2555833	29.2458701	28.7285385	27.4411549	28.71559562	28.60517255	0.061028523	0.933	-0.823	PIS55086.1	orf19.3477	Putative pseudouridine synthase; predicted role in snRNA pseudouridine synthesis, tRNA pseudouridine synthesis; Spider biofilm induced
PIS58536.1	30.2954819	29.6199131	28.5435262	27.94306683	29.2382903	28.80827057	0.332393972	0.967	-0.823	PIS58536.1	orf19.3695	Diacylglycerol cholinephosphotransferase and ethanolaminephosphotransferase, catalyzes the final step in Kennedy pathway of phosphatidylcholine and phosphatidylethanolamine biosynthesis
PIS51961.1	26.8460713	27.8391371	28.3986399	25.24520414	27.48619664	27.88270527	0.177005719	0.957	-0.823	PIS51961.1	VAN1	Member of Mnn9 family of mannosyltransferases; ortholog of S. cerevisiae Van1p; fungal-specific (no human or murine homolog)
PIS58137.1	30.4293349	30.5021093	31.2675864	29.37679928	29.89323658	30.46268674	0.153622521	0.954	-0.822	PIS58137.1	orf19.4468	Putative succinate dehydrogenase; localized to the mitochondrial membrane; Hap43p-repressed gene

PIS49854.1	24.1847734	24.3267432	24.5824715	23.15382075	24.23047013	23.24688245	0.082042151	0.94	-0.821	PIS49854.1	orf19.721	Ortholog(s) have polynucleotide 5'-hydroxyl-kinase activity
PIS51717.1	24.650426	24.1519925	25.5436394	24.27758778	23.27736463	24.32767368	0.080127083	0.94	-0.821	PIS51717.1	RBE1	Pry family cell wall protein; Rim101, Efg1, Ssn6, alkaline repressed; O-glycosylation; no GPI anchor predicted; ketoconazol induced; regulated by Sef1, Sfu1, Hap4; flow model biofilm induced; rat catheter and Spider biofilm repressed
PIS55042.1	23.6258425	23.8545477	24.2231809	22.91587874	23.33263784	22.99889654	0.072353901	0.937	-0.819	PIS55042.1	POM152	Putative nuclear pore membrane glycoprotein
PIS58401.1	24.0643174	25.8293286	25.6863834	23.75700171	24.76021346	24.61144552	0.197808594	0.959	-0.817	PIS58401.1	orf19.7111	Putative mitochondrial outer membrane protein membrane fission effector; possibly an essential gene, disruptants not obtained by UAU1 method
PIS49799.1	28.2660214	28.2427306	28.8337953	28.06248483	27.6813024	27.15108731	0.026424983	0.903	-0.816	PIS49799.1	NOP14	Putative nucleolar protein; Hap43-induced; mutation confers resistance to 5-fluorocytosine (5-FC), 5-fluorouracil (5-FU), and tubercidin (7-deazaadenosine); heterozygous mutant is resistant to parnafungin; Spider biofilm induced
PIS49594.1	29.8855327	30.8562395	30.4367003	29.33284065	29.78090104	29.62712266	0.015389432	0.877	-0.813	PIS49594.1	CYS4	Cystathionine beta-synthase; sulfur amino acid biosynthesis; antigenic in mouse; flow model biofilm induced; alkaline induced; macrophage/pseudohyphal-induced; present in exponential and stationary growth phase yeast cultures
PIS58437.1	29.9720299	29.8430472	29.3408363	29.11120188	28.82489352	28.78397352	0.063415895	0.934	-0.812	PIS58437.1	orf19.5621	Putative protein of unknown function; mutation confers hypersensitivity to amphotericin B; overlaps orf19.5621

PIS52274.1	26.9753572	25.9075683	27.1986547	27.47951412	26.05709721	24.11288725	0.255794881	0.963	-0.811	PIS52274.1	orf19.3611	Protein of unknown function; Hap43-repressed gene; repressed by nitric oxide
PIS55684.1	29.811336	30.0094018	29.7839271	28.62803865	29.32897465	29.22207156	0.018810894	0.888	-0.809	PIS55684.1	EMP70	Protein with a role in endosome-to-vacuole sorting; rat catheter biofilm repressed
PIS48333.1	28.9847982	29.2050018	29.3743244	28.52051818	28.72324453	27.89322875	0.057856076	0.931	-0.809	PIS48333.1	TIM44	Protein involved in transport across membranes; Spider biofilm repressed
PIS58499.1	26.7176948	25.6788658	25.4928154	24.00216821	25.26772506	26.20102687	0.226182792	0.961	-0.806	PIS58499.1	TRM2	Putative tRNA methyltransferase; repressed by prostaglandins; Spider biofilm induced
PIS50431.1	26.0650364	27.0718363	27.0986808	26.20658962	26.50075458	25.11432358	0.193819594	0.958	-0.805	PIS50431.1	orf19.2675	Ortholog(s) have mRNA binding activity, role in mRNA splicing, via spliceosome, spliceosomal complex assembly and U2 snRNP, U2-type prespliceosome localization
PIS51196.1	32.3814431	32.6566274	32.3261423	31.43801743	31.96159465	31.55399001	0.019212653	0.889	-0.804	PIS51196.1	IDH2	Putative mitochondrial NAD-isocitrate dehydrogenase subunit; induced by ciclopirox; induced in high iron; present in exponential and stationary growth phases; Spider biofilm repressed
PIS59012.1	32.2910791	32.4975631	32.5126431	31.41434398	31.91062071	31.57115641	0.010288912	0.853	-0.802	PIS59012.1	HSP104	Heat-shock protein; roles in biofilm and virulence; complements chaperone, prion activity in S. cerevisiae; guanidine-insensitive; heat shock/stress induced; repressed in farnesol-treated biofilm; sumoylation target; Spider biofilm induced

PIS48287.1	25.7178147	25.9753724	26.7276694	23.83576678	25.04198214	27.13876187	0.25298446	0.963	-0.801	PIS48287.1	CHS4	Activator of Chs3p chitin synthase; required for wild-type wall chitin content, but not for hyphal growth; mutant resistant to Calcofluor white; prenylation and 2 transmembrane segments predicted; functional homolog of S. cerevisiae Chs4p
PIS51238.1	34.001493	34.3277207	34.4546866	33.44479429	33.43673744	33.50919536	0.013089598	0.868	-0.798	PIS51238.1	CEF3	Translation elongation factor 3; antigenic in humans; predicted C-term nucleotide-binding active site; protein on surface of yeast, not hyphae; polystyrene adherence induced; higher protein amount in stationary phase; possibly essential
PIS48434.1	25.4992099	27.3457599	26.2400729	24.22156421	26.39391664	26.07445162	0.299882802	0.965	-0.798	PIS48434.1	orf19.5363	Component of UDP-GlcNAc transferase; required for the 2nd step of dolichyl-linked oligosaccharide synthesis; Spider biofilm induced
PIS51666.1	30.4376156	31.054213	31.4592654	30.53043909	30.25840193	29.77079901	0.040883827	0.92	-0.797	PIS51666.1	YKT6	Putative protein of the vacuolar SNARE complex; predicted role in vacuolar fusion; rat catheter biofilm repressed
PIS55518.1	22.5103347	25.4419014	26.8003177	23.51400497	24.60936236	24.24203208	0.484694045	0.971	-0.796	PIS55518.1	orf19.4430	Ortholog(s) have ubiquitin binding activity and role in proteasome-mediated ubiquitin-dependent protein catabolic process
PIS52394.1	24.517997	26.1441724	25.3897742	24.40846634	23.91263914	25.35371129	0.257718422	0.963	-0.792	PIS52394.1	PLB4.5	Phospholipase B; Hog1-induced; regulated by Ssn6; putative GPI-anchor; repressed during cell wall regeneration; clade-associated gene expression; Hap43-induced; rat catheter and Spider biofilm repressed
PIS48578.1	27.5956322	26.3502007	27.0962252	28.2311268	23.26384194	27.17630496	0.398895472	0.969	-0.79	PIS48578.1	orf19.5710	Nucleoporin component of central core of the nuclear pore complex; mRNA binds She3

PIS58804.1	26.3583878	25.4640602	24.7215699	21.55750831	26.76904476	25.84947721	0.493750295	0.972	-0.789	PIS58804.1	orf19.2330	Putative U3 snoRNA-associated protein; Hap43-induced; transposon mutation affects filamentous growth; repressed by prostaglandins
PIS51749.1	25.6866601	25.2073717	24.0409001	24.41894449	24.14512565	24.0032173	0.142348179	0.953	-0.789	PIS51749.1	orf19.3806	Ortholog(s) have role in negative regulation of gluconeogenesis, proteasome-mediated ubiquitin-dependent protein catabolic process, traversing start control point of mitotic cell cycle and GID complex localization
PIS49696.1	30.1281091	30.6253189	31.1547706	30.4571252	29.67045495	29.41779524	0.051543667	0.928	-0.788	PIS49696.1	orf19.5278	Protein of unknown function; Spider biofilm induced
PIS55059.1	23.816916	24.2483115	24.3259996	23.45397436	23.895194	22.68133686	0.295179203	0.965	-0.787	PIS55059.1	HCA4	Putative role in regulation of cell wall biogenesis; Hap43p-induced gene; possibly an essential gene, disruptants not obtained by UAU1 method; flow model and rat catheter biofilm induced
PIS49732.1	28.7925225	30.654988	31.2832625	28.33773088	29.87287379	30.16065426	0.386423513	0.969	-0.787	PIS49732.1	orf19.6787	Ortholog(s) have cargo receptor activity and role in ascospore formation, axial cellular bud site selection, endoplasmic reticulum to Golgi vesicle-mediated transport
PIS55578.1	27.3575202	26.8533431	26.2663622	26.60406793	26.14636688	25.37287016	0.324952694	0.966	-0.785	PIS55578.1	orf19.6941	Putative diacylglycerol acyltransferase; catalyzes the terminal step of triacylglycerol formation; flow model biofilm induced; Spider biofilm induced
PIS51198.1	34.6627289	34.5016202	35.0791	34.21160411	33.95653508	33.72415767	0.012299243	0.864	-0.784	PIS51198.1	EFT2	Elongation Factor 2 (eEF2); GTPase; essential; highly expressed; target of sordarin antifungals; antigenic in human/mouse; lacks site for regulatory phosphorylation by eEF2 kinase; GCN-regulated; higher protein amount in stationary phase

PIS58280.1	30.997032	31.4800713	30.7333307	30.02784492	30.44392165	30.39199173	0.031286783	0.91	-0.782	PIS58280.1	orf19.3037	Putative poly(A)-binding protein; regulated by Gcn4p; induced in response to amino acid starvation (3-AT treatment); protein present in exponential and stationary growth phase yeast cultures
PIS51187.1	24.7772522	25.408984	26.2201366	23.18560948	24.84512925	26.03162317	0.371646155	0.968	-0.781	PIS51187.1	CHT3	Major chitinase; secreted; functional homolog of S. cerevisiae Cts1p; 4 N-glycosylation motifs; possible O-mannosylation; putative signal peptide; hyphal-repressed; farnesol upregulated in biofilm; regulated by Efg1p, Cyr1p, Ras1p
PIS54554.1	26.9113611	26.5607153	26.8935286	25.35835282	26.37971314	26.28802707	0.128132218	0.951	-0.78	PIS54554.1	orf19.2965	Putative guanyl-nucleotide exchange factor; Spider biofilm repressed
PIS58458.1	29.257704	28.8358536	28.736624	25.26958767	29.45948257	29.76218331	0.422907815	0.97	-0.78	PIS58458.1	TIM21	Component of the Translocase of the Inner Mitochondrial membrane (TIM23 complex); for protein import into mitochondria; Hap43, ketoconazole-repressed; Spider biofilm repressed
PIS51456.1	25.5134595	25.0744543	26.8027836	25.63301386	24.95043982	24.47051044	0.190701448	0.958	-0.779	PIS51456.1	SGT1	Putative co-chaperone protein with a predicted role in kinetochore assembly; mutation confers hypersensitivity to radicicol; sumoylation target
PIS51627.1	31.1973438	31.1054235	32.8724058	30.33498532	30.79926273	31.7066943	0.197226818	0.959	-0.778	PIS51627.1	HMG1	HMG-CoA reductase; enzyme of sterol pathway; inhibited by lovastatin; gene not transcriptionally regulated in response to lovastatin and fluconazole
PIS51888.1	25.7010913	27.028541	26.0727386	25.91861732	25.15300966	25.40593037	0.174134195	0.956	-0.775	PIS51888.1	orf19.2404	Ortholog(s) have RNA binding, ribonuclease MRP activity, ribonuclease P activity, tRNA binding activity

PIS59015.1	32.1487823	32.364478	32.600851	31.27867268	31.82465711	31.68825424	0.041045414	0.92	-0.774	PIS59015.1	CDC48	Putative microsomal ATPase; plasma membrane-localized; regulated by Gcn2 and Gcn4; induced by amino acid starvation (3-AT); macrophage/pseudohyphal-repressed; protein levels decrease in stationary phase yeast; Spider biofilm repressed
PIS54574.1	34.2680847	34.8843337	34.8989367	33.88867785	33.767941	34.07258074	0.040044461	0.919	-0.774	PIS54574.1	GND1	6-phosphogluconate dehydrogenase; soluble in hyphae; farnesol, macrophage-induced protein; antigenic in mice; dual localization to cytosol and peroxisomes depends on alternative splicing; rat catheter and Spider biofilm repressed
PIS52305.1	33.2032719	32.6134599	32.4173067	31.75904588	32.13159648	32.02134061	0.074571063	0.938	-0.774	PIS52305.1	RPL27A	Ribosomal protein L27; Spider biofilm repressed
PIS50400.1	27.11144	28.1010956	28.5328456	26.46506484	27.52630917	27.43411792	0.201586253	0.959	-0.773	PIS50400.1	NSA1	Putative 66S pre-ribosomal particles conmponent; Hap43-induced; repressed by prostaglandins
PIS48357.1	25.3990801	27.0773549	27.0439185	24.60621891	26.20237206	26.39588336	0.230863771	0.961	-0.772	PIS48357.1	ERV46	Putative ER-derived vesicle protein; COPII-coated vesicle complex subunit; transcript induced by filamentous growth; Spider biofilm repressed
PIS58545.1	31.1479822	31.4921977	32.5924943	30.97873085	30.94425111	30.99221122	0.083203441	0.941	-0.772	PIS58545.1	orf19.3349	Putative RNA polymerase II subunit B150; heterozygous null mutant exhibits resistance to parnafungin in the C. albicans fitness test
PIS51375.1	26.3434675	24.9892293	24.6828039	23.80718083	23.15920981	26.75742124	0.431792144	0.97	-0.764	PIS51375.1	CNT	CNT family H(+)/nucleoside symporter; transports adenosine, uridine, inosine, guanosine, tubercidin; variant alleles for high/low-affinity isoforms; S or G at residue 328 affects specificity; Spider, flow model biofilm induced

PIS58261.1	25.3473639	24.5909025	25.6318426	24.12446659	25.05304989	24.11026664	0.125404701	0.95	-0.761	PIS58261.1	orf19.2604	S. pombe ortholog SPAC2C4.06c is a predicted tRNA (cytosine-5-)-methyltransferase; Spider biofilm induced
PIS52371.1	28.0623003	28.2080717	28.2078602	27.53054787	27.21821971	27.44704479	0.103914326	0.946	-0.761	PIS52371.1	orf19.714	Ortholog(s) have GTPase activity
PIS58191.1	31.0437358	31.2314482	31.2442299	30.68497394	30.62986742	29.92248143	0.023452671	0.898	-0.761	PIS58191.1	PMT1	Protein mannosyltransferase; required for virulence in mice and for adhesion to epithelial cells; role in hyphal growth and drug sensitivity; Als1, Sec20, Kre9, Pir1 are substrates; 1 of 5 PMT family members
PIS58343.1	26.1435563	24.8753041	24.6196687	25.26068253	23.28717144	24.81370581	0.232481059	0.961	-0.759	PIS58343.1	orf19.7086	Ortholog(s) have nuclear import signal receptor activity, role in NLS-bearing protein import into nucleus, protein import into nucleus and cytoplasm, nucleus localization
PIS50547.1	32.3869355	33.2156531	32.3295383	31.56944232	32.19201443	31.90298559	0.077414605	0.939	-0.756	PIS50547.1	GPM1	Phosphoglycerate mutase; surface protein that binds host complement Factor H and FHL-1; antigenic; fluconazole, or amino acid starvation (3-AT) induced, farnesol-repressed; Hap43, flow model biofilm induced; Spider biofilm repressed
PIS48541.1	26.088283	25.8554667	26.7730391	25.98420392	25.6238809	24.84053675	0.335878146	0.967	-0.756	PIS48541.1	orf19.4626	Ortholog(s) have role in positive regulation of transcription by RNA polymerase I, regulation of TORC1 signaling and cytosol localization
PIS58165.1	29.9183127	30.2830663	30.5667962	28.74378004	29.83778694	29.91886662	0.105810254	0.946	-0.756	PIS58165.1	SVF1	Putative survival factor; stationary phase enriched protein; fluconazole-induced; regulated by Gcn4p; repressed in response to amino acid starvation (3-AT treatment); fungal-specific (no human or murine homolog)

PIS58903.1	25.9376488	26.2397401	24.6427762	23.90682845	25.85793898	24.78816892	0.239537283	0.962	-0.756	PIS58903.1	SYN8	Putative endosomal SNARE; role in protein transport; rat catheter and Spider biofilm induced
PIS49751.1	26.5581494	27.7883781	26.5628983	25.54234729	26.68345574	26.417722	0.164505976	0.955	-0.755	PIS49751.1	orf19.4735	Ornithine cyclodeaminase family protein; Sef1, Sfu1, and Hap43-regulated; ortholog of S. cerevisiae YGL159W and S. pombe SPAP11E10.01; rat catheter biofilm induced
PIS52217.1	31.1462296	30.9964989	30.6976385	29.8450942	30.34091272	30.38835802	0.025194828	0.901	-0.755	PIS52217.1	PTR22	Oligopeptide transporter involved in uptake of di-/tripeptides; regulated by Stp2 and Stp3; transcript induced upon phagocytosis by macrophage; repressed by Rim101 at pH 8; flow model biofilm induced
PIS58306.1	30.4524101	30.2948136	29.8971938	29.44030665	29.83317857	29.10954437	0.037078639	0.917	-0.754	PIS58306.1	ADE13	Adenylosuccinate lyase; enzyme of adenine biosynthesis; soluble protein in hyphae; not induced during GCN response, in contrast to the S. cerevisiae ortholog; repressed by nitric oxide
PIS58588.1	28.7247864	26.9363653	27.2384016	26.75658406	26.35266512	27.53006465	0.182968348	0.957	-0.753	PIS58588.1	OAC1	Putative mitochondrial inner membrane transporter; rat catheter biofilm induced
PIS51930.1	27.3623237	29.1565414	29.3055989	26.65805983	28.06744883	28.83910185	0.30581599	0.965	-0.753	PIS51930.1	orf19.364	Putative vacuolar H+-ATPase subunit; required for proper vacuolar ATPase assembly and vacuolar functions; Spider biofilm repressed
PIS50570.1	29.1872747	28.1622943	27.6633935	26.79008563	28.30034181	27.66582389	0.35625853	0.968	-0.752	PIS50570.1	GDE1	Glycerophosphocholine phosphodiesterase; mutation confers hypersensitivity to 5-fluorouracil (5-FU); F-12/CO2 early biofilm induced

PIS51117.1	31.1478854	32.0215548	32.2749339	31.0877888	30.91546387	31.18521207	0.086834319	0.942	-0.752	PIS51117.1	SDH2	Succinate dehydrogenase, Fe-S subunit; localizes to surface of yeast cells, but not hyphae; induced in high iron and during log phase aerobic growth; repressed by nitric oxide, Hap43
PIS55546.1	30.8015205	30.5921894	29.9829512	29.58308639	30.34572236	29.20784015	0.058618261	0.931	-0.747	PIS55546.1	ARO8	Aromatic transaminase of the Ehrlich fusel oil pathway of aromatic alcohol biosynthesis; Rim101 independent alkaline induction; protein abundance affected by URA3 expression in CAI-4 strain; Gcn4-regulated; stationary phase enriched
PIS59027.1	31.5767183	31.5209473	32.2317534	30.64249948	30.98008659	31.46762345	0.079376034	0.94	-0.746	PIS59027.1	MYO2	Class V myosin; nonessential; sole class V myosin in C. albicans; required for WT actin cytoskeletal polarity, nuclear organization, migration, hyphal growth; conserved myosin ATPase/tail domains; Hap43-induced; flow model biofilm repressed
PIS54602.1	23.1131143	23.3466043	23.6828662	23.45674844	21.86272551	22.58499849	0.441670999	0.97	-0.746	PIS54602.1	orf19.3438	Ortholog(s) have protein-folding chaperone binding activity and role in protein folding in endoplasmic reticulum, response to unfolded protein, ubiquitin-dependent ERAD pathway
PIS54700.1	29.2179171	29.7271864	28.9046277	28.44438339	28.64496084	28.52681232	0.101660528	0.946	-0.745	PIS54700.1	orf19.2262	Probable quinone oxidoreductase; involved in protection from oxidative stress; induced by quinones, benomyl treatment, nitric oxide, and Spider biofilm; null mutant shows increased susceptibility to killing by neutrophils
PIS48313.1	31.3145435	32.2163798	31.7099192	30.06923284	32.07145477	30.86756459	0.402795413	0.969	-0.744	PIS48313.1	CKA2	Catalytic alpha-subunit of protein kinase CK2; interaction with calcineurin pathway affects fluconazole sensitivity; synthetically lethal with CKA1; attenuated virulence in a mouse oropharyngeal candidiasis but not in a systemic mouse model
PIS51321.1	24.7416138	25.0847421	26.2963521	24.5317363	24.92266081	24.43482541	0.337653241	0.967	-0.744	PIS51321.1	orf19.7458	Ortholog of C. dubliniensis CD36 : Cd36_86660, Candida tenuis NRRL Y-1498 : cten_CGOB_00028, Candida tropicalis NEW ASSEMBLY : CTRG1_05695 and Candida tropicalis MYA-3404 : CTRG_05695

PIS59016.1	26.1435562	26.3763105	25.6836746	24.3831574	25.89447999	25.69736574	0.107590078	0.947	-0.743	PIS59016.1	MCU1	Mitochondrial protein involved in utilization of carbon sources, filamentous growth and virulence
PIS48483.1	25.2620636	25.8237142	27.3335878	23.70819789	26.21949159	26.2653688	0.295118507	0.965	-0.742	PIS48483.1	orf19.2201	Ortholog(s) have ribosome binding activity, role in mitochondrial respiratory chain complex III assembly, positive regulation of mitochondrial translation and Cbp3p-Cbp6 complex, mitochondrial ribosome localization
PIS54629.1	26.0572924	26.5675345	27.1352363	25.12335916	26.64419867	25.77029496	0.305531313	0.965	-0.741	PIS54629.1	PRE3	Putative beta-1 proteasome subunit; macrophage-induced protein; regulated by Gcn2p and Gcn4p; GlcNAc-induced protein
PIS48218.1	26.7146821	26.9887258	27.1998042	26.86632272	26.14018727	25.68170633	0.070153481	0.936	-0.738	PIS48218.1	orf19.730	Ortholog(s) have GTPase activator activity and role in establishment or maintenance of actin cytoskeleton polarity, small GTPase mediated signal transduction
PIS55467.1	29.9222603	30.3556992	29.9936843	29.172277	29.46882226	29.4186329	0.059289956	0.932	-0.737	PIS55467.1	orf19.5281	Predicted essential RNA-binding G protein; ortholog an effector of mating response pathway in S. cerevisiae; mainly associated with nuclear envelope and ER; flow model and Spider biofilm repressed
PIS48353.1	29.1485002	30.0580462	29.6535362	29.79288328	28.68158641	28.17789696	0.079489754	0.94	-0.736	PIS48353.1	YIM1	Protein similar to protease of mitochondrial inner membrane; increased transcription is observed upon benomyl treatment; macrophage-downregulated gene
PIS48337.1	30.8157084	31.127815	31.1258652	30.47857725	30.15193675	30.23398787	0.041979535	0.921	-0.735	PIS48337.1	ETR1	Putative 2-enoyl thioester reductase; protein present in exponential and stationary growth phase yeast cultures; rat catheter biofilm repressed

PIS58753.1	34.2343775	34.2645035	34.2380997	33.17684017	33.9648145	33.39885728	0.015469577	0.877		-0.732	PIS58753.1	RPS9B	Predicted ribosomal protein; repressed upon phagocytosis by murine macrophage; transcript possibly regulated upon hyphal formation; Spider biofilm repressed
PIS58608.1	26.6033055	24.0862672	23.9716614	23.60297052	24.32535108	24.5437746	0.300740315	0.965		-0.73	PIS58608.1	EXG2	GPI-anchored cell wall protein, similar to S. cerevisiae exo-1,3-beta-glucosidase Exg2p; predicted Kex2p substrate; induced during cell wall regeneration; possibly an essential gene, disruptants not obtained by UAU1 method; Hap43p-repressed
PIS55488.1	29.2298981	29.8241123	28.8493558	27.64331186	28.76434352	29.30445762	0.21350451	0.96		-0.73	PIS55488.1	orf19.5728	Putative cytochrome P450; Spider biofilm induced
PIS51120.1	26.4778627	27.6054669	28.3869915	25.89956584	27.30251522	27.07806887	0.166333874	0.956		-0.73	PIS51120.1	orf19.7204	Has domain(s) with predicted nitronate monooxygenase activity
PIS55745.1	31.0213186	31.1153597	31.6858043	30.23210522	30.85525898	30.54966147	0.071382072	0.937		-0.728	PIS55745.1	ADE17	5-Aminoimidazole-4-carboxamide ribotide transformylase, enzyme of adenine biosynthesis; antigenic in human; soluble protein in hyphae; not induced during GCN response, in contrast to the S. cerevisiae ortholog
PIS59020.1	28.9477095	28.3915461	29.4482498	28.44435632	28.17003567	27.99515777	0.366764622	0.968		-0.726	PIS59020.1	orf19.5006.1	Ortholog(s) have SNAP receptor activity and role in Golgi to plasma membrane transport, ascospore-type prospore membrane formation, endocytosis, exocytosis, vesicle fusion
PIS51904.1	24.3154579	27.4904083	26.1891917	26.3974798	25.29808846	24.1312349	0.356787007	0.968		-0.723	PIS51904.1	PRO3	Delta 1-pyrroline-5-carboxylate reductase; protein induced during the mating process; alkaline induced; stationary phase enriched protein; Spider biofilm repressed

PIS58983.1	30.1574001	30.6290124	31.3109401	29.94837595	30.02378074	29.95522027	0.055038525	0.93	-0.723	PIS58983.1	SEC14	Essential protein; functional homolog of S. cerevisiae Sec14p, a Golgi phosphatidylinositol/phosphatidylcholine transfer protein that regulates choline-phosphate cytidyltransferase and thereby affects secretion; biofilm-regulated
PIS50465.1	31.7401399	32.1280284	32.9561902	31.60997562	31.44682261	31.6009867	0.055261007	0.93	-0.722	PIS50465.1	orf19.891	Putative AMP deaminase; possibly an essential gene, disruptants not obtained by UAU1 method
PIS48630.1	27.6840872	26.9639386	27.713319	27.18918339	24.4665757	28.54226928	0.40673619	0.969	-0.721	PIS48630.1	orf19.846	Predicted protein kinase similar to S. cerevisiae Nnk1; implicated in proteasome function in S. cerevisiae; induced by Mnl1 under weak acid stress
PIS56853.1	24.9338907	26.0902507	23.6962259	24.24664245	24.21250664	24.10467569	0.244826526	0.962	-0.719	PIS56853.1	orf19.4583	Protein with a mitochondrial carrier protein domain; possibly an essential gene, disruptants not obtained by UAU1 method; Spider biofilm repressed
PIS51017.1	25.7835131	25.2451264	24.3964586	24.76846558	24.46032417	24.04622698	0.106393302	0.947	-0.717	PIS51017.1	AFP99	Agmatinase, involved in metabolism of agmatine; downregulated upon adherence to polystyrene; regulated by Gcn2p and Gcn4p
PIS48719.1	24.3069273	23.9387927	24.9298537	23.14669107	24.11184385	23.76504498	0.133250408	0.951	-0.717	PIS48719.1	SAP3	Secreted aspartyl proteinase, acts in utilization of protein as nitrogen source; assessment of virulence role complicated by URA3 effects; regulated by growth phase; produced by opaque phase cells; alpha-pheromone repressed
PIS54929.1	32.7022866	33.1220822	33.6862957	31.59609069	32.5420071	33.2232755	0.164288629	0.955	-0.716	PIS54929.1	URA2	Putative bifunctional carbamoylphosphate synthetase-aspartate transcarbamylase; flucytosine induced; macrophage/pseudohyphal-induced; 5'-UTR intron; flow model biofilm repressed

PIS58125.1	29.0914647	29.3641292	29.6355386	27.26294864	29.22179549	29.46034702	0.207799573	0.959	-0.715	PIS58125.1	GLT1	Putative glutamate synthase; regulated by Sef1, Sfu1, and Hap43; rat catheter biofilm repressed
PIS58132.1	24.5251005	24.7782256	27.5137091	24.8376007	24.91264411	24.92278712	0.345579847	0.967	-0.715	PIS58132.1	orf19.4471	Ortholog(s) have role in TOR signaling, re-entry into mitotic cell cycle after pheromone arrest and endoplasmic reticulum, endoplasmic reticulum membrane, endoplasmic reticulum-Golgi intermediate compartment localization
PIS51055.1	24.7655158	24.3865711	25.0098417	24.47775219	25.00286603	22.54411538	0.329304122	0.966	-0.712	PIS51055.1	orf19.5876	Protein of unknown function; Cyr1-repressed; induced by alpha pheromone in SpiderM medium; rat catheter and Spider biofilm induced
PIS58754.1	26.3550969	26.7221489	27.1633572	27.16707042	22.83665546	28.09957373	0.469042927	0.971	-0.712	PIS58754.1	orf19.841	Ortholog(s) have SNARE binding activity, role in Golgi vesicle docking, Golgi vesicle transport and Golgi membrane localization
PIS51765.1	34.2592306	34.2161738	33.6768548	33.9574246	33.36000082	32.70465738	0.095602546	0.944	-0.71	PIS51765.1	SSA2	HSP70 family chaperone; cell wall fractions; antigenic; beta-defensin peptides impport; ATPase domain binds histatin 5; at hyphal surface, not yeast; farnesol-repressed in biofilm; flow model, Spider biofilm repressed; caspofungin repressed
PIS59022.1	30.2185381	30.3187123	30.427216	30.08449627	29.40411227	29.35325735	0.027677665	0.905	-0.708	PIS59022.1	OSM2	Putative mitochondrial fumarate reductase; regulated by Ssn6p, Gcn2p, and Gcn4p; Hog1p-downregulated; stationary phase enriched protein; Hap43p-repressed gene
PIS54788.1	27.0934253	27.4163929	28.1858913	27.27749254	26.12234906	27.17746372	0.192979825	0.958	-0.706	PIS54788.1	PRE6	Putative alpha-4 subunit of the proteasome; reported as macrophage-induced protein and macrophage/pseudohyphal-repressed gene; regulated by Gcn2p and Gcn4p; removed from/reinstated in Assembly 20 (see Locus History)

PIS58579.1	25.1822334	25.7608224	26.154815	25.4217034	23.89262552	25.67116605	0.351405028	0.967	-0.704	PIS58579.1	ESS1	Prolyl isomerase (parvulin class); essential; involved in yeast-hyphal switching, Cph1p pathway; has inflexible linker between WW and isomerase domains, unlike human homolog; functional homolog of S. cerevisiae Ess1p
PIS55541.1	26.6608905	26.5591314	26.0247109	25.19621056	25.69659976	26.23861829	0.063130394	0.934	-0.704	PIS55541.1	PDR6	Putative pleiotropic drug resistance regulatory protein 6; transcript regulated by Nrg1; induced by alpha pheromone in SpiderM medium
PIS58150.1	30.2458082	29.6809285	29.8442937	29.46865227	29.1503765	29.0481092	0.082806485	0.941	-0.701	PIS58150.1	orf19.3022	Ortholog(s) have structural constituent of ribosome activity and mitochondrial small ribosomal subunit localization
PIS50406.1	30.7764367	30.6118602	30.8408944	30.05838212	30.09435878	29.98152054	0.026882413	0.904	-0.698	PIS50406.1	ADE12	Adenylosuccinate synthase; upregulated in biofilm; decreased expression in hyphae vs yeast-form cells; not induced during GCN response, in contrast to S. cerevisiae ADE12, which is induced by Gcn4p; stationary phase-enriched protein
PIS56581.1	26.5040856	27.0289344	27.2817522	26.04031593	26.47620878	26.20541547	0.411635765	0.969	-0.698	PIS56581.1	CIC1	Putative proteasome-interacting protein; rat catheter biofilm induced
PIS52446.1	23.8027561	24.021296	23.9524954	24.14123308	22.55989917	22.9832089	0.391579257	0.969	-0.697	PIS52446.1	orf19.4220	Predicted pyridoxal 5'-phosphate synthase; regulated by Gcn4; repressed by amino acid starvation (3-AT treatment); rat catheter biofilm repressed
PIS52434.1	27.8948659	29.0880584	28.1923909	27.45736393	27.18464819	28.44788803	0.230896024	0.961	-0.695	PIS52434.1	TVP18	Putative integral membrane protein; fluconazole-induced

PIS55530.1	19.572189	22.8832767	22.4821631	19.57231669	22.31718604	20.97026474	0.576983362	0.974	-0.693	PIS55530.1	NAB3	Putative nuclear polyadenylated RNA-binding protein; flucytosine repressed
PIS55024.1	29.0593973	28.6013251	27.880277	28.37725315	27.31829271	27.7684487	0.093155063	0.944	-0.692	PIS55024.1	orf19.6071	Ortholog(s) have peptide alpha-N-acetyltransferase activity and role in N-terminal peptidyl-methionine acetylation, cytoskeleton organization, mitochondrion inheritance, regulation of actin cytoskeleton organization
PIS54569.1	28.8429223	29.9944581	29.1287137	29.29250567	28.39200536	28.21000822	0.19048123	0.958	-0.691	PIS54569.1	EGD2	Nascent polypeptide associated complex protein alpha subunit; soluble protein in hyphae; macrophage/pseudohyphal-induced; protein level decrease in stationary phase cultures; GlcNAc-induced protein; Spider biofilm repressed
PIS58025.1	29.6912021	29.3308635	28.6950453	27.05860931	28.84279322	29.74159532	0.244155726	0.962	-0.691	PIS58025.1	FEN12	Putative protein with a predicted role in the elongation of fatty acids; amphotericin B, caspofungin repressed
PIS58494.1	29.7635083	29.8148044	30.0092107	29.33587128	29.2077603	28.97387856	0.054328466	0.929	-0.69	PIS58494.1	orf19.2737	Carbohydrate kinase domain-containing protein; Spider biofilm induced
PIS58572.1	27.7472627	28.751731	29.5655121	29.11664352	27.56270501	27.3185779	0.36677232	0.968	-0.689	PIS58572.1	orf19.783	Ortholog(s) have role in endocytosis, phospholipid translocation, retrograde vesicle-mediated transport, Golgi to endoplasmic reticulum, vacuole organization
PIS58262.1	27.4909754	28.0536575	28.641232	26.9015624	27.5784652	27.64367742	0.130675395	0.951	-0.687	PIS58262.1	LYS12	Homoisocitrate dehydrogenase; catalyzes 4th step in the alpha-aminoadipate pathway of lysine biosynthesis; clade-associated gene expression; protein level decreases in stationary phase cultures; Spider biofilm repressed

PIS54764.1	24.6881369	25.1521086	25.7943949	24.32920739	24.72234371	24.52414134	0.291772616	0.965	-0.686	PIS54764.1	HST7	MAP kinase kinase involved in mating and hyphal growth signal transduction pathways; phosphorylates Cek1p; wild-type virulence in mouse systemic infection; functional homolog of S. cerevisiae Ste7p; mutants are hypersensitive to caspofungin
PIS56589.1	25.011843	25.4678626	26.064973	25.21857156	24.28819512	24.98107775	0.292643669	0.965	-0.686	PIS56589.1	orf19.5755	Ortholog(s) have cyclin-dependent protein serine/threonine kinase regulator activity
PIS52287.1	24.2292317	24.3679326	25.7854262	24.16224221	23.8235875	24.34804821	0.297095808	0.965	-0.683	PIS52287.1	orf19.1910	Predicted NADH-dependent flavin oxidoreductase; Hap43-repressed gene
PIS51965.1	27.4141201	27.1025265	26.9540269	26.38255917	25.94594137	27.0927503	0.281938715	0.964	-0.683	PIS51965.1	orf19.6742	Ortholog(s) have RNA polymerase II CTD heptapeptide repeat phosphatase activity, protein serine/threonine phosphatase activity, role in regulation of transcription by RNA polymerase II and cytosol, nucleus localization
PIS51849.1	24.1088731	26.9503687	27.8842591	26.22479879	25.39508655	25.2822401	0.391546176	0.969	-0.68	PIS51849.1	SET2	Ortholog(s) have RNA binding, histone H3K36 methyltransferase activity
PIS54621.1	26.3981632	25.9691623	25.6802259	26.51501399	25.58915679	23.90729128	0.233086147	0.961	-0.679	PIS54621.1	orf19.1381	Ortholog of S. cerevisiae/S. pombe Lsb5; predicted role in actin cortical patch localization, actin filament organization, endocytosis; flow model biofilm induced; Spider biofilm repressed
PIS58074.1	25.1736559	26.2499218	25.1712298	23.42473245	25.05107779	26.08251514	0.262382828	0.963	-0.679	PIS58074.1	orf19.6597	Has domain(s) with predicted role in regulation of cell cycle

PIS58881.1	30.1992462	30.3470604	29.6928576	29.7809118	29.37631615	29.04422515	0.205284534	0.959	-0.679	PIS58881.1	SEC3	Predicted subunit of the exocyst complex, involved in exocytosis; required for hyphal growth after the first septin ring formation; interacts with septins Cdc3p, Cdc10p, and Cdc11p; localizes to a crescent on the surface of the hyphal tip
PIS51285.1	28.5186959	28.5983051	29.9963119	28.48717006	28.28346329	28.30937053	0.233936785	0.961	-0.678	PIS51285.1	FKH2	Forkhead transcription factor; morphogenesis regulator; required for wild-type hyphal transcription, cell separation, and for virulence in cell culture; mutant lacks true hyphae, is constitutively pseudohyphal; upregulated in RHE model
PIS56855.1	27.7070125	26.7588943	26.5701176	26.59206661	26.96914299	25.44750595	0.393514723	0.969	-0.676	PIS56855.1	HGH1	Putative HMG1/2-related protein; transcript regulated by Mig1
PIS58837.1	28.5722867	28.6890318	27.4769424	27.34729227	27.69576921	27.66853547	0.264474286	0.963	-0.676	PIS58837.1	orf19.399	Probable serine/threonine protein kinase; null mutant grows slowly, lacks hyphal development, has abnormal actin polarization, and is hypersensitive to a sphingolipid inhibitor; appears to act in a pathway with FPK1, PKH2, and PKH3
PIS54848.1	29.2676787	28.3924692	28.609814	28.18774043	28.45108731	27.604783	0.142448923	0.953	-0.675	PIS54848.1	GTT13	Putative glutathione S-transferase; opaque-specific transcript; repressed by alpha pheromone in SpiderM medium; Spider biofilm induced
PIS48411.1	31.7453615	32.0169082	32.0367908	30.43372054	31.6839996	31.65967817	0.076490081	0.939	-0.674	PIS48411.1	orf19.3518	Ortholog(s) have dicarboxylic acid transmembrane transporter activity, role in mitochondrial transport and mitochondrial inner membrane, mitochondrion localization
PIS49462.1	30.6326905	31.0853439	31.3641228	29.92260304	30.35048234	30.78994835	0.092092384	0.943	-0.673	PIS49462.1	YBN5	P-loop ATPase with similarity to human OLA1 and bacterial YchF; Spider biofilm repressed

PIS58565.1	26.7056643	26.6443247	27.9441847	26.76367593	26.38621849	26.13106077	0.119250376	0.949	-0.671	PIS58565.1	orf19.4614	Adapter protein for pexophagy and the cytoplasm-to-vacuole targeting (Cvt) pathway; Spider biofilm induced
PIS51454.1	27.77886	27.9050349	27.952796	28.05152747	27.29730836	26.27491159	0.367651022	0.968	-0.671	PIS51454.1	RRP15	Putative nucleolar protein; constituent of pre-60S ribosomal particles; Hap43-induced; repressed by prostaglandins
PIS50519.1	27.7156231	28.1165416	27.8984635	27.799534	26.38473621	27.53549895	0.111500862	0.948	-0.67	PIS50519.1	orf19.2677	Putative GPI transamidase component; possibly an essential gene, disruptants not obtained by UAU1 method
PIS48347.1	26.3045622	27.0587972	27.9290619	25.97223064	25.95756711	27.3568786	0.459817665	0.971	-0.669	PIS48347.1	orf19.1247	Has domain(s) with predicted mitochondrion autophagosome adaptor activity and role in mitophagy
PIS55076.1	27.4126494	27.0659909	24.7426435	25.51615563	26.52432232	25.18052223	0.356842026	0.968	-0.667	PIS55076.1	CSO99	Protein of unknown function; Hap43-repressed gene; protein not conserved in S. cerevisiae
PIS51885.1	26.0367908	27.5380387	24.7101813	25.4943569	24.65973745	26.13371765	0.601989152	0.974	-0.666	PIS51885.1	orf19.3260	Putative phosphopantothenoylcysteine decarboxylase, binds to protein phosphatase Ppz1p and regulates its activity
PIS55744.1	30.1746595	30.83487	30.5042908	29.68259006	29.94846269	29.88759284	0.021884243	0.895	-0.665	PIS55744.1	orf19.491	Endoplasmic reticulum (ER) protein-translocation complex subunit

PIS58889.1	30.649699	30.7120506	31.3818185	30.12572543	29.97449908	30.64794994	0.177412341	0.957	-0.665	PIS58889.1	RPL28	Putative ribosomal protein; Plc1-regulated; downregulated upon phagocytosis by murine macrophage; Spider biofilm repressed
PIS48396.1	30.9178891	30.8345372	30.6672561	30.08939829	29.89717873	30.44780541	0.023014693	0.897	-0.662	PIS48396.1	orf19.2485	Ortholog(s) have chromatin binding, promoter-specific chromatin binding, protein-containing complex binding, structural constituent of nuclear pore activity
PIS50422.1	29.5130872	29.376434	28.9143891	28.6983518	28.97003695	28.15008904	0.110752918	0.947	-0.662	PIS50422.1	orf19.3235	Putative F-actin capping protein subunit alpha; possibly an essential gene, disruptants not obtained by UAU1 method
PIS48265.1	29.6955045	29.5289359	30.3175935	28.37454364	29.47078949	29.71264315	0.132796004	0.951	-0.661	PIS48265.1	CMP1	Catalytic subunit of calcineurin (Ca[2+]-calmodulin-regulated S/T protein phosphatase); required for wild-type virulence, resistance to high pH, Na(+), Li(+), Mn(2+), and fluconazole tolerance; micafungin is fungicidal to null mutant
PIS58990.1	27.1208322	24.9723105	24.131605	24.16870324	25.492065	24.58122983	0.376690158	0.968	-0.661	PIS58990.1	LEU3	Zn(II)2Cys6 transcription factor; predicted regulator branched-c ofhain amino acid biosynthesis genes; alkaline induced; induced by Mnl1 under weak acid stress; required for yeast cell adherence to silicone substrate; Spider biofilm induced
PIS48489.1	25.6938661	26.3254977	25.8468722	25.31664191	25.41459078	25.15361117	0.269179806	0.964	-0.66	PIS48489.1	orf19.225	Predicted 2-hydroxyacid dehydrogenase; Hap43-repressed gene
PIS51098.1	24.9815382	25.6426313	24.9914217	24.56802218	24.08647165	24.9852728	0.199828732	0.959	-0.659	PIS51098.1	NGS1	N-acetyltransferase related to Gcn5p, acts as N-acetylglucosamine (GlcNAc) sensor required for GlcNAc-induced histone acetylation at promoters of GlcNAc-inducible genes and activation of their transcription by Rep1p; Hap43-induced gene

PIS54760.1	30.0841074	30.1550105	29.275934	27.99791174	29.17945671	30.36782386	0.23188897	0.961	-0.657	PIS54760.1	orf19.2460	Protein of unknown function; substrate for Kex2 processing in vitro; repressed by alpha pheromone in SpiderM medium; Spider biofilm induced; Bcr1-repressed in a/a RPMI biofilms
PIS55542.1	26.8106234	27.0461501	28.0521419	26.52015529	27.29425991	26.12319198	0.428622102	0.97	-0.657	PIS55542.1	RFA1	Putative DNA replication factor A; RNA abundance regulated by cell cycle, tyrosol and cell density
PIS52368.1	30.2680831	30.0968458	30.254984	29.68344667	29.479117	29.48958444	0.465046813	0.971	-0.656	PIS52368.1	PPZ1	Protein phosphatase Z; fungal-specific type 1 family serine/threonine protein phosphatase involved in cation homeostasis and cell wall integrity
PIS51967.1	24.0954013	22.9985566	24.3232892	21.84458823	23.50740107	24.10148314	0.465317419	0.971	-0.655	PIS51967.1	orf19.5345	Putative ubiquitin-like polyubiquitin-binding protein; induced by nitric oxide independent of Yhb1; Spider biofilm repressed
PIS58101.1	31.6150774	29.8432301	29.9001868	28.63903818	31.61536038	29.14172453	0.364500605	0.968	-0.654	PIS58101.1	DPM1	Dolichol-phosphate mannose synthase catalytic subunit; filament induced; Tup1-regulated; flow model and rat catheter biofilm repressed
PIS58236.1	32.7360996	32.5351647	32.3518234	32.4063326	32.13229387	31.12382003	0.090792224	0.943	-0.654	PIS58236.1	RPL39	Ribosomal protein L39; transcript induced upon germ tube formation; colony morphology-related gene regulation by Ssn6; Hap43-induced
PIS51429.1	29.3183781	30.2577922	30.2984461	29.35319536	29.06293043	29.49944411	0.055802349	0.93	-0.653	PIS51429.1	orf19.1086	Ortholog(s) have role in protein deubiquitination, protein retention in Golgi apparatus, regulation of ER to Golgi vesicle-mediated transport, regulation of retrograde vesicle-mediated transport, Golgi to ER, ribophagy

PIS55591.1	29.8251122	30.0744627	30.9560353	29.2764626	29.26126154	30.35987962	0.130714345	0.951	-0.653	PIS55591.1	TPS1	Trehalose-6-phosphate synthase; role in hyphal growth and virulence in mouse systemic infection; induced in presence of human neutrophils; macrophage/pseudohyphal-repressed after 16h; stationary phase enriched protein; Hap43-repressed
PIS50597.1	24.9617154	24.7720928	23.1383053	23.057065	23.66962483	24.19044046	0.185265596	0.957	-0.652	PIS50597.1	MLH1	Putative mismatch repair protein; cell-cycle regulated periodic mRNA expression
PIS58205.1	30.7811945	30.7485782	31.0924894	30.22655124	30.39072084	30.04938468	0.04593547	0.924	-0.652	PIS58205.1	NTH1	Neutral trehalase; hyphal induction in mutant delayed but not reduced overall; not required for virulence in mice; possible regulatory cAMP-dependent phosphorylation at S10,S213; Hap43-repressed gene; Spider biofilm induced
PIS56802.1	27.8774407	28.8601013	27.7162594	27.87440841	27.38300498	27.24406375	0.177168711	0.957	-0.651	PIS56802.1	SAC7	Putative GTPase activating protein (GAP) for Rho1; repressed upon adherence to polystyrene; macrophage/pseudohyphal-repressed; transcript is upregulated in RHE model of oral candidiasis and in clinical oral candidiasis
PIS55810.1	31.3527762	27.661856	28.5724237	26.79294542	31.49099176	27.35387747	0.633968472	0.975	-0.65	PIS55810.1	MNN9	Protein of N-linked outer-chain mannan biosynthesis; mutant has defective cell wall; required for wild-type hyphal growth; mutant is hygromycin B sensitive and vanadate resistant; has N-terminal membrane-spanning segment (positions 18-34)
PIS48379.1	24.4690652	23.5877733	25.6120574	23.15303389	23.7652078	24.80143547	0.390204656	0.969	-0.65	PIS48379.1	RRP8	Ribosomal protein; Hap43-induced; F-12/CO2 early biofilm and rat catheter biofilm induced
PIS54650.1	29.6749623	29.8754915	30.1736335	29.05648301	29.2820716	29.443935	0.052771761	0.928	-0.647	PIS54650.1	ASN1	Putative asparagine synthetase; soluble protein in hyphae; regulated by Rim101; decreased expression at pH 4 vs pH 8; protein detected during exponential and stationary phases of yeast-form growth

PIS49466.1	29.9365718	30.5155063	30.4365507	28.91956385	29.81982072	30.2107178	0.085582773	0.941	-0.646	PIS49466.1	SEC21	Ortholog(s) have role in endoplasmic reticulum to Golgi vesicle-mediated transport, retrograde vesicle-mediated transport, Golgi to endoplasmic reticulum and COPI vesicle coat, endosome localization
PIS48231.1	28.8082796	28.1896284	29.3583876	28.23298148	27.85362348	28.33021916	0.225809753	0.961	-0.646	PIS48231.1	SPE3	Putative spermidine synthase; predicted role in pantothenate and spermidine biosynthesis; Spider biofilm repressed
PIS51971.1	34.8860188	35.0124522	34.9460091	34.31719821	34.33658803	34.25942585	0.01944084	0.889	-0.644	PIS51971.1	RPS4A	Predicted ribosomal protein, component of the small ribosomal subunit; repressed upon phagocytosis by murine macrophage; positively regulated by Tbf1; mutant is defective in filamentous growth and sensitive to osmotic stress
PIS48512.1	32.1573078	32.902306	33.2079676	31.5545885	32.44243588	32.34348633	0.129745119	0.951	-0.642	PIS48512.1	orf19.5293	Ortholog of C. dubliniensis CD36 : Cd36_43710, C. parapsilosis CDC317 : CPAR2_402940, C. auris B8441 : B9J08_005206 and Candida tenuis NRRL Y-1498 : CANTEDRAFT_114940
PIS51416.1	27.7815009	27.9491707	27.7560369	26.98791388	27.34175958	27.23369044	0.072718593	0.937	-0.641	PIS51416.1	FET33	Putative multicopper ferro-O2-oxidoreductase; repressed in core caspofungin response; fails to complement S. cerevisiae fet3 iron-related phenotypes; rat catheter and Spider biofilm induced
PIS55641.1	32.2512914	31.9372476	31.7526124	31.65628706	31.41830398	30.94699444	0.186837456	0.958	-0.64	PIS55641.1	DBP5	Ortholog(s) have ATP-dependent activity, acting on RNA, RNA helicase activity, inositol hexakisphosphate binding activity
PIS52380.1	29.1975349	28.6930337	28.6004975	27.99353039	27.35401871	29.22713798	0.141636328	0.953	-0.639	PIS52380.1	HTA3	Putative histone H2A; amphotericin B repressed; flucytosine induced; RNA abundance regulated by tyrosol and cell density; Spider biofilm repressed

PIS58694.1	31.3610763	31.3148733	31.9707517	29.96138692	31.23543086	31.53924586	0.300671733	0.965	-0.637	PIS58694.1	RPS26A	Ribosomal protein; regulated by Nrg1, Tup1; repressed upon phagocytosis by murine macrophage; alternatively spliced intron in 5'-UTR; Spider biofilm repressed
PIS52097.1	30.4616927	30.9832876	31.1695384	30.13975533	30.13296078	30.43780513	0.039238782	0.919	-0.635	PIS52097.1	GRS1	Putative tRNA-Gly synthetase; genes encoding ribosomal subunits, translation factors, tRNA synthetases are downregulated upon phagocytosis by murine macrophage; stationary phase enriched protein
PIS49486.1	30.8649945	30.838592	31.2110378	30.20026184	30.49872832	30.3179513	0.05054825	0.927	-0.633	PIS49486.1	orf19.7328	Protein with a Staphylococcal nuclease domain; transcript regulated by Mig1 and Tup1; flow model and Spider biofilm repressed
PIS51603.1	27.2930984	28.3329604	27.2176155	27.32700813	26.9728316	26.6458838	0.212407757	0.96	-0.633	PIS51603.1	RIM20	Protein involved in the pH response pathway; binds to the transcription factor Rim101 and may serve as a scaffold to facilitate the C-terminal proteolytic cleavage that activates Rim101; required for alkaline pH-induced hyphal growth
PIS50335.1	30.8748904	30.6929478	30.2681081	30.0054922	30.01251473	29.92130671	0.238937888	0.962	-0.632	PIS50335.1	CHS3	Major chitin synthase of yeast and hyphae; synthesizes short-chitin fibrils; Chs4-activated; transcript induced at yeast-hyphal transition; Chs1 and Chs2, but not Chs3, are inhibited by the protoberberine HWY-289; Spider biofilm induced
PIS58471.1	24.5833669	24.864789	24.5129728	24.49107211	23.53484663	24.03786551	0.188258543	0.958	-0.632	PIS58471.1	orf19.6170	Ortholog(s) have kinesin binding, microtubule binding, microtubule plus-end binding activity
PIS48406.1	27.68143	26.9131484	26.3891099	26.68084978	26.77938658	25.62997601	0.136640285	0.952	-0.631	PIS48406.1	LPI9	Protein phosphatase type 1 regulator; role in chromosome segregation, regulation of phosphoprotein phosphatase activity; rat catheter and Spider biofilm induced

PIS56626.1	28.8084704	29.0778522	28.8187365	28.47785299	28.39734629	27.93759702	0.074374398	0.938	-0.631	PIS56626.1	PEX8	Putative peroxisomal biogenesis factor; expression regulated during planktonic growth
PIS52057.1	29.4427868	29.4900326	29.7570351	28.62671258	29.12414293	29.04962631	0.084763887	0.941	-0.63	PIS52057.1	orf19.3053	Protein of unknown function; present in exponential and stationary phase yeast; identified in extracts from biofilm and planktonic cells; flow model biofilm induced gene; GlcNAc-induced protein
PIS51553.1	25.3153257	26.1950738	25.4720145	24.9581989	24.89329012	25.24017846	0.51950454	0.972	-0.63	PIS51553.1	orf19.7149	Putative GTPase inhibitor; predicted role in endocytosis, protein targeting to vacuole; rat catheter biofilm repressed
PIS58938.1	31.1138998	30.4521389	30.2768494	28.69530241	30.66726095	30.59120639	0.217711628	0.96	-0.63	PIS58938.1	PEX11	Putative peroxisomal membrane protein; role in fatty acid oxidation; expression is Tac1-regulated; Hms1p-dependent induction by geldamycin; Spider biofilm induced
PIS52184.1	23.8791819	23.1166395	23.4322302	21.88654963	22.34641159	24.30691747	0.275043105	0.964	-0.629	PIS52184.1	orf19.6707	Predicted vacuolar protein with a calcineurin-like phosphoesterase domain; repressed by alpha pheromone in SpiderM medium
PIS58131.1	28.5733742	29.0786604	30.2837261	28.62962738	28.75432708	28.6665192	0.334078305	0.967	-0.628	PIS58131.1	COQ4	Protein with a putative role in coenzyme Q biosynthesis; transcriptionally induced by interaction with macrophage; Hap43p-repressed gene
PIS52144.1	34.0317019	33.7741976	33.5180163	33.52233499	33.14695216	32.7698105	0.067339281	0.935	-0.628	PIS52144.1	RPS15	Putative ribosomal protein; macrophage/pseudohyphal-induced after 16 h; repressed upon phagocytosis by murine macrophage; Spider biofilm repressed

PIS52004.1	32.8258187	33.3422845	33.8012461	32.540159	32.56187435	32.98356134	0.049649571	0.926	-0.628	PIS52004.1	TKL1	Putative transketolase; localizes to surface of yeast cells, not hyphae; soluble protein in hyphae; transcript regulated by Nrg1, Mig1, and Tup1; antigenic in human or murine infection; possibly essential (by UAU1 method)
PIS51958.1	25.8264604	26.2879451	26.3735704	26.37324554	25.1461725	25.09037353	0.184067041	0.957	-0.626	PIS51958.1	orf19.7437	Putative protein of unknown function; Hap43p-repressed gene; ortholog of S. cerevisiae YJL218W
PIS54620.1	28.4296487	27.8867755	27.9322197	27.59108209	27.49846322	27.28398999	0.053173422	0.929	-0.625	PIS54620.1	orf19.1383	Protein of unknown function; induced by alpha pheromone in SpiderM medium
PIS58702.1	28.5564081	29.4691908	29.4119508	28.67034608	28.32037326	28.57235358	0.089284584	0.943	-0.625	PIS58702.1	orf19.1480	Putative succinate dehydrogenase; enzyme of citric acid cycle; repressed by nitric oxide; Efg1, Hap43 repressed
PIS50415.1	25.6208562	26.5664574	25.8642979	24.60730579	24.49499463	27.07456128	0.444175998	0.97	-0.625	PIS50415.1	orf19.5093	Component of the RSC chromatin remodeling complex
PIS49711.1	25.9086301	26.9722806	25.8228001	24.70244679	26.17092954	25.95406058	0.251859228	0.963	-0.625	PIS49711.1	orf19.6559	RNA polymerase III transcription initiation factor complex (TFIIIC) subunit; growth phase regulated protein; downregulaated in stationary phase yeast cultures; Hap43-repressed; flow model biofilm induced; Spider biofilm repressed
PIS51986.1	27.562283	26.6336684	27.1086117	26.25234178	26.11674389	27.06282152	0.151468197	0.954	-0.624	PIS51986.1	orf19.3366.1	Protein of unknown function; Hap43-repressed gene

PIS51741.1	27.6519738	27.3331937	26.8920712	27.02780683	26.82992947	26.14757758	0.182850084	0.957	-0.624	PIS51741.1	RFC5	Putative heteropentameric replication factor C subunit; periodic mRNA expression, peak at cell-cycle G1/S phase
PIS51483.1	32.3596297	32.5009168	32.4503339	31.79349197	31.92059405	31.72920965	0.022842266	0.897	-0.623	PIS51483.1	CHC1	Clathrin heavy chain; subunit of the major coat protein; role in intracellular protein transport and endocytosis; flow model and rat catheter biofilm repressed
PIS51261.1	25.7643292	26.2597748	25.8795621	25.14054475	25.90767549	24.98763524	0.127572001	0.95	-0.623	PIS51261.1	MNR2	Putative ion transporter; fungal-specific (no human or murine homolog)
PIS54814.1	30.247097	30.6772895	30.2156304	29.27500574	30.10743802	29.88742111	0.077277795	0.939	-0.623	PIS54814.1	SAP9	[illegible] adhesion, cell surface integrity; induced by antifungal drugs, stationary phase, or in white-phase cells; farnesol-downregulated in biofilm; autocatalytic processing; GPI-anchor; [illegible]
PIS55015.1	24.7935735	25.2016488	26.5408615	25.04878487	24.98956066	24.63756909	0.190413706	0.958	-0.62	PIS55015.1	orf19.1182	Ortholog(s) have SNAP receptor activity, phosphatidylinositol-3-phosphate binding activity
PIS51237.1	28.065191	28.3973977	27.8115447	28.1181059	27.90051262	26.39579156	0.230872868	0.961	-0.62	PIS51237.1	orf19.1970	Putative vacuole biogenesis protein
PIS55762.1	26.5182325	26.2808471	25.3483656	24.40686387	26.17335387	25.71025665	0.179525566	0.957	-0.619	PIS55762.1	orf19.4192.1	Ortholog of C. dubliniensis CD36 : Cd36_60590, C. parapsilosis CDC317 : CPAR2_602880, C. auris B8441 : B9J08_001867 and Candida tenuis NRRL Y-1498 : CANTEDRAFT_115034

PIS52416.1	28.0586576	26.0758965	27.961615	27.87473314	26.77106508	25.5941232	0.401767892	0.969	-0.619	PIS52416.1	orf19.4894	Protein with similarity to S. cerevisiae Yer010cp, a protein of unknown function belonging to the prokaryotic RraA family; repressed by benomyl; Hap43-induced; Spider biofilm induced
PIS50640.1	26.1494684	28.3794614	27.8830444	26.37558167	26.73336425	27.44756192	0.386755477	0.969	-0.618	PIS50640.1	FRP1	Ferric reductase-related protein involved in heme acquisition; alkaline-induced by Rim101; iron-chelation-induced by CCAAT-binding factor; fluconazole-repressed; ciclopirox-, hypoxia-, Hap43-induced
PIS58811.1	29.605682	30.0419795	29.791305	29.44751967	29.1284296	29.01163954	0.035836925	0.915	-0.617	PIS58811.1	TCP1	Chaperonin-containing T-complex subunit, induced by alpha pheromone in SpiderM medium; stationary phase enriched protein
PIS50533.1	28.651295	28.549214	28.1701954	28.31472261	27.97939545	27.23030148	0.220338757	0.96	-0.615	PIS50533.1	orf19.3920	Ortholog(s) have protein disulfide isomerase activity, protein-disulfide reductase (glutathione) activity, protein-disulfide reductase activity and role in protein folding
PIS54587.1	32.6502101	32.7939276	33.2419044	32.17658636	32.20270186	32.46811454	0.065297374	0.934	-0.613	PIS54587.1	KGD1	Putative 2-oxoglutarate dehydrogenase; regulated by Efg1 under yeast but not hyphal growth conditions; transcript induced in an RHE model of oral candidiasis; stationary phase enriched protein; Hap43-repressed; rat catheter biofilm
PIS49838.1	26.2015535	26.3228591	24.5570865	25.07636096	25.44558001	24.72534933	0.355957642	0.968	-0.611	PIS49838.1	orf19.5575	Putative peripheral peroxisomal membrane peroxin; required for regulating peroxisome size and maintenance; Spider biofilm induced
PIS51551.1	28.1177834	28.0587972	27.930929	27.42569611	27.36975685	27.48084248	0.094629907	0.944	-0.61	PIS51551.1	orf19.3583	Ortholog(s) have role in protein export from nucleus, ribosomal small subunit export from nucleus and cytosol, nucleus localization

PIS50410.1	31.0575853	30.8003828	30.5715839	29.76101732	30.45131168	30.38632534	0.08452506	0.941	-0.61	PIS50410.1	orf19.5079.1	Ortholog of C. dubliniensis CD36 : Cd36_07590, C. parapsilosis CDC317 : CPAR2_206680, C. auris B8441 : B9J08_004229 and Candida tenuis NRRL Y-1498 : CANTEDRAFT_104385
PIS58020.1	26.5656338	28.3421557	27.3629789	27.3437505	25.78918108	27.30779884	0.264747043	0.963	-0.61	PIS58020.1	VMA8	Putative vacuolar H+-ATPase subunit; regulated by Nrg1 and Mig1; transcript increases in populations of cells exposed to fluconazole over multiple generations; rat catheter and Spider biofilm repressed
PIS49807.1	29.8747159	30.2989378	30.8519236	29.06836977	30.01306896	30.11733533	0.19806852	0.959	-0.609	PIS49807.1	CDC10	Septin, required for wild-type cell, hyphal, or chlamydospore morphology; role in virulence and kidney tissue invasion in mouse infection; forms ring at sites of cell division and filaments in mature chlamydospore; Spider biofilm repressed
PIS58634.1	24.1418749	25.6426313	25.0471607	24.56802218	24.08647165	24.35238218	0.226983668	0.961	-0.608	PIS58634.1	AVT1	Putative vacuolar transporter; promoter bound by a1p and alpha2p by ChIP-chip analysis
PIS58952.1	24.8584295	26.5064865	26.2763683	25.08440636	25.33408436	25.40003501	0.193278024	0.958	-0.608	PIS58952.1	DCG1	Protein of unknown function; ortholog of S. cerevisiae Dcg1; transcript regulated by Nrg1 and Mig1
PIS52035.1	28.1566354	28.6729261	29.3797678	27.76573376	28.22981803	28.38855182	0.158267546	0.955	-0.608	PIS52035.1	HOM2	Aspartate-semialdehyde dehydrogenase; forms a homodimer; conserved in bacteria, archaea, and fungi but not in mammals; ketoconazole-repressed; protein present in exponential and stationary growth phase yeast cultures; GlcNAc-
PIS48572.1	29.6446683	29.4786511	29.4924714	28.16316745	29.38140281	29.24720776	0.108774168	0.947	-0.608	PIS48572.1	RBF1	Transcription factor; glutamine-rich activation domain; binds RPG-box DNA sequences; predominantly nuclear; mutation causes accelerated induction of filamentous growth; antigenic during human oral infection; Sko1p-repressed

PIS54607.1	25.8595246	25.5090384	25.1148229	23.40676757	25.57813179	25.6841446	0.362161512	0.968	-0.605	PIS54607.1	orf19.7443	Ortholog of C. dubliniensis CD36 : Cd36_86510, C. parapsilosis CDC317 : CPAR2_206110, C. auris B8441 : B9J08_002384 and Candida tenuis NRRL Y-1498 : CANTEDRAFT_112184
PIS55486.1	27.8341663	27.1514683	26.9370092	26.32689622	27.12587572	26.66287531	0.379715233	0.968	-0.602	PIS55486.1	SEC34	Ortholog(s) have cargo adaptor activity
PIS54949.1	28.9677029	28.8753522	29.0766946	27.99594965	28.66438688	28.45752282	0.560430216	0.973	-0.601	PIS54949.1	orf19.7357	Ortholog(s) have phosphopantothenate--cysteine ligase activity, role in acetyl-CoA biosynthetic process from pantothenate, coenzyme A biosynthetic process and CoA-synthesizing protein complex, cytoplasm, nucleus localization
PIS49677.1	31.4452835	31.5716151	31.5478145	30.71545353	31.22139326	30.8320704	0.115153175	0.948	-0.599	PIS49677.1	ACH1	Acetyl-coA hydrolase; acetate utilization; nonessential; soluble protein in hyphae; antigenic in human; induced on polystyrene adherence; farnesol-, ketoconazole-induced; no human or murine homolog; stationary phase-enriched protein
PIS58858.1	25.3582899	24.7657531	24.0680048	24.02285645	24.18482648	24.1893844	0.329929482	0.966	-0.598	PIS58858.1	orf19.1297	Ortholog(s) have phosphatidylinositol-3-phosphate binding activity and role in autophagy of mitochondrion, cytoplasm to vacuole transport by the Cvt pathway, early endosome to Golgi transport, macroautophagy
PIS58057.1	28.4756242	28.4910922	30.2702722	28.14514499	28.45282613	28.84380186	0.328295452	0.966	-0.598	PIS58057.1	TRX1	Thioredoxin; involved in response to reactive oxygen species; biofilm, benomyl, flucytosine, peroxide, Hap43 induced; amphotericin B, caspofungin repressed; induced by human neutrophils; macrophage-repressed gene
PIS58922.1	29.3079863	29.6947202	29.8302598	29.18810358	28.71530826	29.140096	0.090612761	0.943	-0.596	PIS58922.1	TRP2	Putative anthranilate synthase with a predicted role in tryptophan biosynthesis; regulated by Gcn2p and Gcn4p

PIS56770.1	27.3844152	26.9519546	28.5185575	27.36429967	26.95195945	26.75715344	0.350715453	0.967	-0.594	PIS56770.1	COF1	Putative cofilin; macrophage-induced protein; protein present in exponential and stationary-phase yeast cells, but higher amounts in stationary phase
PIS58880.1	30.537947	30.9393595	30.866696	29.53160716	30.23580933	30.79489821	0.117538555	0.949	-0.594	PIS58880.1	orf19.3843	Ortholog(s) have protein transmembrane transporter activity and role in filamentous growth, post-translational protein targeting to membrane, translocation
PIS51758.1	25.0219379	24.9140959	26.0285917	24.85221802	25.03416986	24.29933115	0.201801781	0.959	-0.593	PIS51758.1	ATG1	Putative protein serine/threonine kinase; predicted role in vesicle formation in autophagy and the cytoplasm-to-vacuole targeting (Cvt) pathway; Spider biofilm induced
PIS52071.1	28.0739391	28.504586	28.770403	28.00952256	27.91598015	27.64647096	0.132712736	0.951	-0.592	PIS52071.1	orf19.3508	Putative protein of unknown function; stationary phase enriched protein
PIS56787.1	27.0385154	27.9624769	27.7192355	26.16099956	27.25211991	27.53312198	0.159283858	0.955	-0.591	PIS56787.1	FOL1	Putative dihydroneopterin aldolase (dihydro-6-hydroxymethylpterin pyrophosphokinase); fungal-specific (no human or murine homolog)
PIS56558.1	23.4029217	25.1968275	24.4054392	19.89581078	24.77221366	26.56662231	0.627484656	0.975	-0.59	PIS56558.1	CKB2	Regulatory subunit of protein kinase CK2 (casein kinase II), beta' subunit; null mutants are hypersensitive to caspofungin
PIS58687.1	23.6706052	24.2291399	24.3282627	24.58971498	23.67702869	22.19387817	0.301464006	0.965	-0.589	PIS58687.1	orf19.2112	snRNP U5 splicing factor component; involved in positioning the 3' splice site during the 2nd catalytic step of splicing; Spider biofilm induced

PIS51846.1	25.5665927	26.5042671	27.0989575	25.35990343	24.89064878	27.15183401	0.317547974	0.966	-0.589	PIS51846.1	PHO23	Ortholog(s) have methylated histone binding activity
PIS50565.1	29.2772097	29.1862756	28.8570847	28.9382663	27.98978063	28.62454422	0.266085207	0.963	-0.589	PIS50565.1	URA7	CTP synthase 1; flucytosine induced; protein present in exponential and stationary growth phase yeast cultures
PIS56774.1	29.4648187	30.2048454	29.5544863	29.31191418	29.29251777	28.85480538	0.08669984	0.942	-0.588	PIS56774.1	orf19.2065	Ortholog(s) have allantoicase activity and role in allantoin catabolic process
PIS52328.1	26.5376247	26.9452115	26.9087637	25.85720885	26.55081733	26.22374552	0.117318726	0.949	-0.587	PIS52328.1	FEN1	Putative fatty acid elongase; predicted role in sphingolipid biosynthesis; possibly an essential gene, disruptants not obtained by UAU1 method; Spider and flow model biofilm induced
PIS56843.1	28.1525203	27.6132493	27.7515202	27.57647357	27.17442539	27.00464875	0.108742175	0.947	-0.587	PIS56843.1	TYR1	Putative prepephenate dehydrogenase; enzyme of tyrosine biosynthesis; fungal-specific (no human or murine homolog)
PIS56938.1	29.1990535	29.4864748	29.9833656	28.87760457	29.03620265	29.00128879	0.221779782	0.96	-0.585	PIS56938.1	POT1	Putative peroxisomal 3-oxoacyl CoA thiolase; transcript regulated by Nrg1 and Mig1; farnesol regulated; Hap43-repressed
PIS58856.1	29.5525128	29.0996253	29.4622813	28.81448809	28.81313628	28.73417739	0.038291501	0.918	-0.584	PIS58856.1	orf19.5038	Predicted tRNA (guanine) methyltransferase activity; Spider biofilm induced

PIS51676.1	33.9772767	34.166155	34.2217356	33.12526891	33.53935332	33.94853674	0.054355818	0.929	-0.584	PIS51676.1	RPS18	Predicted ribosomal protein; repressed upon phagocytosis by murine macrophage; repressed by nitric oxide; Hap43-induced; Spider biofilm repressed
PIS56712.1	25.0399737	25.4799169	25.5197605	25.24953585	24.89805195	24.14476268	0.369852777	0.968	-0.582	PIS56712.1	orf19.7673	Ortholog(s) have mRNA binding activity, role in mRNA splicing, via spliceosome and U1 snRNP, U2-type prespliceosome, U4/U6 x U5 tri-snRNP complex, U5 snRNP, commitment complex, cytosol, nucleus localization
PIS54752.1	27.3417613	27.7533382	25.9575847	25.17929818	27.33134839	26.81221193	0.501774161	0.972	-0.577	PIS54752.1	orf19.5812	Ortholog of S. cerevisiae Ett1, a nuclear protein that inhibits replication of Brome mosaic virus; early-stage flow model biofilm induced
PIS50636.1	28.8352588	29.438821	29.2489495	28.86651512	28.61907946	28.30578067	0.137823162	0.952	-0.577	PIS50636.1	RDI1	Putative rho GDP dissociation inhibitor; transposon mutation affects filamentous growth; farnesol, filament-induced; regulated by Nrg1, Tup1; protein levels low in stationary phase yeast
PIS48591.1	25.8993773	24.4036609	24.854321	25.89398769	24.03171971	23.50258876	0.514807503	0.972	-0.576	PIS48591.1	orf19.1400	Ortholog of C. dubliniensis CD36 : Cd36_23640, C. parapsilosis CDC317 : CPAR2_406130, C. auris B8441 : B9J08_005288 and Candida tenuis NRRL Y-1498 : CANTEDRAFT_116273
PIS54573.1	30.5146875	30.3177576	29.9058052	29.37957089	29.77557268	29.85734912	0.092806734	0.943	-0.575	PIS54573.1	ERG5	Putative C-22 sterol desaturase; fungal C-22 sterol desaturases are cytochrome P450 enzymes of ergosterol biosynthesis, catalyze formation of the C-22(23) double bond in the sterol side chain; transposon mutation affects filamentous growth
PIS51230.1	28.6177066	29.0914345	29.696118	28.51491074	28.74812035	28.41700562	0.125586995	0.95	-0.575	PIS51230.1	orf19.2257	Predicted ER protein involved in ER-nucleus signaling; Spider biofilm repressed

PIS48443.1	25.7923918	26.4969518	26.0645105	26.30310111	25.67637939	24.65197975	0.160836434	0.955	-0.574	PIS48443.1	MAD2	Protein required for the spindle assembly checkpoint of the cell cycle; necessary for survival in host cell macrophages and for virulence in a mouse model of systemic candidiasis
PIS54668.1	26.3193383	26.5569335	26.4522706	26.03093144	24.47496077	27.10790764	0.414450004	0.97	-0.572	PIS54668.1	orf19.6982	Ortholog(s) have mitochondrial ribosome binding activity and role in mitochondrial translational initiation
PIS51681.1	27.6079716	28.3521548	27.4890281	27.85964233	27.32186534	26.56327531	0.187522102	0.958	-0.568	PIS51681.1	MCM1	Transcription factor; regulator of hyphal growth; may act with Wor1p; canonical and non-canonical binding sites; MADS domain DNA-binding motif; similar to S. cerevisiae Mcm1p; greater expression in white than opaque cells; intron in 5'-UTR
PIS58644.1	33.9364016	34.2343429	34.3004035	33.64873012	33.61343892	33.50403154	0.049847617	0.927	-0.568	PIS58644.1	RPL18	Predicted ribosomal protein; Plc1p-regulated, Tbf1-activated; repressed upon phagocytosis by murine macrophage; Hap43p-induced; Spider biofilm repressed
PIS58783.1	29.3093922	30.3772941	30.4666172	29.53632021	29.22166555	29.6908744	0.389316251	0.969	-0.568	PIS58783.1	SIS1	Putative Type II HSP40 co-chaperone; macrophage/pseudohyphal-repressed; heavy metal (cadmium) stress-induced; heterozygous null mutant displays sensitivity to virgineone; rat catheter biofilm induced
PIS58450.1	29.3172633	29.0191188	28.2624991	29.27233659	28.98495087	26.63977694	0.586192565	0.974	-0.567	PIS58450.1	AGE3	Putative ADP-ribosylation factor GTPase activating protein, functional ortholog of S. cerevisiae GCS1; mutation affects endocytosis, hyphal growth, chemical and drug resistance, and sensitivity to cell wall inhibitors
PIS55628.1	24.6296489	24.7041386	25.0795142	25.34303545	22.56959738	24.80060952	0.333058274	0.967	-0.567	PIS55628.1	CNB1	Regulatory subunit of calcineurin B (Ca[2+]-calmodulin-regulated S/T protein phosphatase); required for wild-type resistance to fluconazole or to SDS; micafungin is fungicidal to null mutant

PIS58982.1	28.1399126	28.3079244	26.6482354	26.8808766	27.62534216	26.89593829	0.398237943	0.969	-0.565	PIS58982.1	BUD2	GTPase activating protein (GAP) for Rsr1; negative regulator of filament branching, acts in hyphal growth guidance; required for wild-type budding pattern; induced in low iron; regulated by tyrosol and cell density
PIS51886.1	28.5798997	28.9615702	29.7627529	27.23132324	29.1946647	29.18320912	0.278784412	0.964	-0.565	PIS51886.1	orf19.3259	Ortholog(s) have peptidase activity, role in protein targeting to ER, signal peptide processing and endoplasmic reticulum, signal peptidase complex localization
PIS52388.1	26.7298481	25.0844012	24.9701779	25.75194585	25.59561962	23.74209422	0.398596817	0.969	-0.565	PIS52388.1	orf19.5418	Ortholog(s) have role in endoplasmic reticulum to Golgi vesicle-mediated transport
PIS51834.1	28.7942792	29.6443644	29.1877678	28.97537064	28.62614315	28.33043953	0.320671416	0.966	-0.565	PIS51834.1	PRE10	Alpha7 (C8) subunit of the 20S proteasome; multiple phosphorylated residues; transcript induced upon filamentous growth; Spider biofilm repressed
PIS54844.1	28.3725389	28.7970424	29.9740709	28.42979607	28.56262998	28.45938821	0.310608445	0.966	-0.564	PIS54844.1	NPT1	Putative nicotinate phosphoribosyltransferase, involved in NAD salvage pathway; fungal-specific (no human or murine homolog)
PIS48349.1	25.6737226	25.2426656	27.16839	24.06163929	26.17832679	26.15209796	0.405996129	0.969	-0.564	PIS48349.1	orf19.1249	Ortholog(s) have 1-(5-phosphoribosyl)-5-[(5-phosphoribosylamino)methylideneamino]imidazole-4-carboxamide isomerase activity and role in histidine biosynthetic process
PIS55809.1	29.1941641	29.4509659	31.0091928	29.39880826	29.1730555	29.40248852	0.209514058	0.96	-0.56	PIS55809.1	NOG1	Putative GTPase; mutation confers hypersensitivity to 5-fluorocytosine (5-FC), 5-fluorouracil (5-FU), and tubercidin (7-deazaadenosine); repressed by prostaglandins; Hap43-induced

PIS49756.1	32.9976651	32.5145626	33.0966619	32.38175328	32.28008337	32.2683485	0.058690278	0.931	-0.56	PIS49756.1	orf19.2892	Ortholog of C. dubliniensis CD36 : Cd36_45910, C. parapsilosis CDC317 : CPAR2_501110, C. auris B8441 : B9J08_004783 and Candida tenuis NRRL Y-1498 : CANTEDRAFT_119393
PIS58138.1	28.400492	28.3737846	28.6248545	28.43833576	27.14912763	28.13155267	0.370464344	0.968	-0.56	PIS58138.1	orf19.4467	Mitochondrial protein required for expression of mitochondrial respiratory chain complex I (NADH:ubiquinone oxidoreductase)
PIS52304.1	28.6393004	28.1763007	29.1130894	27.64672397	28.00987035	28.59696344	0.16781181	0.956	-0.558	PIS52304.1	WRS1	Putative tRNA-Trp synthetase; genes encoding ribosomal subunits, translation factors, tRNA synthetases are downregulated upon phagocytosis by murine macrophages
PIS50554.1	23.9087989	26.3113915	26.2785196	24.53781879	24.94256715	25.34715243	0.403173019	0.969	-0.557	PIS50554.1	ECM331	GPI-anchored protein; mainly at plasma membrane, also at cell wall; Hap43, caspofungin-induced; Plc1-regulated; Hog1, Rim101-repressed; colony morphology-related regulated by Ssn6; induced by ketoconazole and hypoxia
PIS56757.1	27.9139073	28.7942546	30.0573488	28.33564209	28.31983942	28.44135331	0.319039192	0.966	-0.556	PIS56757.1	AFL1	Predicted inositol polyphosphate kinase, involved in autophagy, energy metabolism, virulence
PIS51402.1	25.2856276	27.1298724	26.6967527	24.99988825	25.71657105	26.73962157	0.516105568	0.972	-0.552	PIS51402.1	MTG1	Putative mitochondrial GTPase; likely essential for respiratory competence and in large ribosomal subunit assembly; mitochondrial translation; Spider biofilm induced
PIS51031.1	33.2175276	33.3416702	32.6794107	32.7786853	33.02707449	31.77784444	0.151669446	0.954	-0.552	PIS51031.1	RPL10	Ribosomal protein L10; intron in 5'-UTR; downregulated upon phagocytosis by murine macrophage; transcription regulated by yeast-hypha switch; Spider biofilm repressed

PIS51382.1	33.1781992	33.5854978	32.5627653	32.51843547	32.53904413	32.61390348	0.142568786	0.953	-0.552	PIS51382.1	RPS8A	Small 40S ribosomal subunit protein; induced by ciclopirox olamine; repressed upon phagocytosis by murine macrophage; 5'-UTR intron; Hap43-induced; Spider biofilm repressed
PIS54740.1	27.5326806	29.3162692	29.4404503	27.07464658	28.51216719	29.05119296	0.666960853	0.975	-0.55	PIS54740.1	orf19.2875	Protein involved in cell cycle regulation; ortholog of S. pombe SPAC1071.09c DNAJ domain protein; Hap43-induced gene
PIS51573.1	31.6380589	32.0080621	32.1729535	31.30833286	31.52731884	31.34327044	0.119780816	0.949	-0.547	PIS51573.1	orf19.6066	Hexadecenal dehydrogenase; involved in the conversion of sphingosine 1-phosphate breakdown product hexadecenal to hexadecenoic acid; Spider biofilm induced
PIS48555.1	26.4191737	26.845059	28.3365729	24.9824073	26.82693681	28.16054346	0.423336384	0.97	-0.544	PIS48555.1	GIN4	Autophosphorylated kinase; role in pseudohyphal-hyphal switch and cytokinesis; phosphorylates Cdc11p on S395; necessary for septin ring within germ tube but not for septin band at mother cell junction; physically associates with septins
PIS51785.1	31.5809401	29.6041202	29.0168467	29.58504771	29.95955131	29.02563242	0.539294706	0.973	-0.544	PIS51785.1	orf19.5201	Ortholog(s) have structural constituent of ribosome activity and mitochondrial small ribosomal subunit localization
PIS51649.1	30.5298201	30.6462681	30.3925257	30.340498	29.89179923	29.70761236	0.271659154	0.964	-0.543	PIS51649.1	orf19.6035	Protein of unknown function; repressed by nitric oxide
PIS49642.1	28.2991107	29.2091143	29.5919599	28.59959306	28.57074459	28.30293942	0.166778644	0.956	-0.542	PIS49642.1	orf19.3064.1	Ortholog of C. parapsilosis CDC317 : CPAR2_108915, C. auris B8441 : B9J08_004667, Candida tenuis NRRL Y-1498 : CANTEDRAFT_102387 and Debaryomyces hansenii CBS767 : DEHA2B06776g

PIS48647.1	25.6629211	28.2543061	26.9253084	27.24655326	25.63987135	26.33147894	0.580472007	0.974	-0.542	PIS48647.1	orf19.3228	Putative endosomal transmembrane protein; Hap43p-induced; mutation confers hypersensitivity to amphotericin B
PIS50492.1	29.8719818	29.5587137	29.5746021	29.34111838	29.32668697	28.7118579	0.301464893	0.965	-0.542	PIS50492.1	orf19.3977	Protein with a role in translation; flow model biofilm repressed
PIS51988.1	30.201825	29.8798635	30.198462	28.46467825	29.80782039	30.38149678	0.259797615	0.963	-0.542	PIS51988.1	RAC1	G-protein of RAC subfamily; required for embedded filamentous growth, not for serum-induced hyphal growth; dynamic localization at plasma membrane and nucleus; similar to, but not interchangeable with, Cdc42p; lacks S. cerevisiae homolog
PIS50479.1	29.6947214	29.07243	29.4517877	29.22177291	28.8109276	28.56572726	0.108163498	0.947	-0.54	PIS50479.1	orf19.3983	Protein with a predicted transcription factor BTF3 domain; flow model biofilm induced
PIS54739.1	28.1570368	29.209833	27.8800732	27.84333172	28.19435368	27.59403499	0.318280073	0.966	-0.538	PIS54739.1	HBR2	Putative alanine glyoxylate aminotransferase; regulated by Gcn4p and hemoglobin; stationary phase enriched protein
PIS48802.1	30.4430749	30.3892194	30.1957385	29.47304929	29.96037489	29.98038298	0.082605738	0.941	-0.538	PIS48802.1	NUP85	Ortholog of S. cerevisiae Nup85; a structural constituent of the nuclear pore; required for alkaline-induced hyphal morphogenesis and for SD or Spider media biofilm formation
PIS54595.1	28.3022262	28.1587665	29.426776	28.30094005	28.18205575	27.79347019	0.166170069	0.956	-0.537	PIS54595.1	APL2	Phosphorylated protein of unknown function; mutation confers hypersensitivity to toxic ergosterol analog

PIS48390.1	31.2233914	31.2102423	30.9273046	30.61259051	30.82146219	30.31492448	0.069826405	0.936	-0.537	PIS48390.1	AUT7	Putative autophagosome protein; acts synergistically with Ysy6p to regulate unfolded protein response and mitochondrial function under ER stress; macrophage/pseudohyphal-repressed; alternatively spliced intron in 5' UTR
PIS56537.1	30.4325403	31.1419687	30.5022192	29.18770095	30.35999383	30.91893332	0.400597933	0.969	-0.537	PIS56537.1	GCV1	Putative T subunit of glycine decarboxylase; transcript negatively regulated by Sfu1; Spider biofilm repressed
PIS49657.1	25.9370067	25.7215114	24.5657386	25.01430596	23.61640284	25.9862214	0.542484297	0.973	-0.536	PIS49657.1	ECM42	Ornithine acetyltransferase; Gcn2, Gcn4-regulated; clade-specific gene expression; possibly essential gene, disruptants not obtained by UAU1 method; Spider biofilm induced
PIS58252.1	27.9912506	28.7371456	28.6300446	26.81174017	28.62584544	28.31966023	0.248121032	0.962	-0.534	PIS58252.1	NIF3	Protein of unknown function; ortholog of S. cerevisiae Nif3; rat catheter biofilm repressed
PIS56546.1	28.1734868	28.809653	28.3461977	28.13352937	28.04292161	27.5510195	0.146355108	0.953	-0.534	PIS56546.1	SLM2	Ortholog(s) have role in TOR signaling, actin cytoskeleton organization, actin filament bundle assembly, cell-cell fusion, eisosome assembly and endosomal transport, more
PIS52086.1	31.7239923	31.1857738	32.0183355	30.88986289	31.13532661	31.30347214	0.366905104	0.968	-0.533	PIS52086.1	orf19.415	Putative protein of unknown function; Hap43p-repressed gene
PIS51948.1	28.4030863	27.5558901	28.8123067	27.46077109	28.07285851	27.63848125	0.2848886	0.964	-0.533	PIS51948.1	SNL1	Ribosome-associated protein predicted to function in protein synthesis; 1 predicted transmembrane domain; rat catheter biofilm repressed

PIS48694.1	23.2402745	25.4310153	26.0869263	24.17806355	24.4464284	24.53343957	0.455068497	0.971	-0.533	PIS48694.1	VPS15	Protein involved in retrograde endosome-to-Golgi protein transport; required for normal virulence
PIS48328.1	26.240602	26.9644199	27.6794405	25.6844721	26.76994598	26.83389355	0.242878894	0.962	-0.532	PIS48328.1	CDC21	Putative thymidylate synthase; flucytosine induced; rat catheter biofilm repressed; Spider biofilm repressed
PIS51643.1	27.0371055	28.2904131	28.6164951	27.52189404	27.28610791	27.54379402	0.399817002	0.969	-0.531	PIS51643.1	PSD1	Phosphatidylserine decarboxylase; involved in phosphatidylethanolamine synthesis; Spider biofilm repressed
PIS48680.1	30.9275574	31.2162371	31.4885864	30.10313838	30.3968242	31.54095025	0.398188178	0.969	-0.53	PIS48680.1	RPL32	Component of the large (60S) ribosomal subunit; Spider biofilm repressed
PIS58906.1	27.6183178	26.8389056	26.5519104	27.15435839	26.6295553	25.63887697	0.218710803	0.96	-0.529	PIS58906.1	NCE103	Carbonic anhydrase; converts of CO2 to bicarbonate; essential for virulence in host niches with limited CO2, normal white-opaque switch; Mnl1-induced in weak acid stress; Hap43-induced gene; F-12/CO2, rat catheter, Spider biofilm induced
PIS58696.1	30.434094	30.3169897	30.3082512	29.90854959	29.91692195	29.64854154	0.178461064	0.957	-0.528	PIS58696.1	orf19.1796	Putative glyoxylate reductase; acts on glyoxylate and hydroxypyruvate substrates; Spider biofilm repressed
PIS49853.1	28.7416179	29.1184059	29.023415	28.527593	28.36554964	28.40966153	0.079090779	0.94	-0.527	PIS49853.1	EMP46	Protein similar to S. cerevisiae Emp46, an integral membrane component of ER-derived COPII-coated vesicles; functions in ER to Golgi transport; induced by alpha pheromone in SpiderM medium

PIS48344.1	28.77552	26.6176838	27.8177502	26.41665615	28.32349837	26.89060631	0.507368361	0.972	-0.527	PIS48344.1	orf19.1240	Ortholog of S. cerevisiae : HOB2, C. glabrata CBS138 : CAGL0D04510g, C. dubliniensis CD36 : Cd36_45200, C. parapsilosis CDC317 : CPAR2_500480 and C. auris B8441 : B9J08_005034
PIS51628.1	27.1977122	28.0449075	28.610572	27.37305522	27.32454636	27.57577542	0.429485905	0.97	-0.527	PIS51628.1	STR2	Ortholog(s) have cystathionine gamma-synthase activity and role in sulfur compound metabolic process, transsulfuration
PIS50425.1	28.871816	28.085429	28.9861652	27.82012444	28.08167688	28.464885	0.156991744	0.954	-0.526	PIS50425.1	ERG27	3-Keto sterol reductase of ergosterol biosynthesis; acts in C-4 sterol demethylation with Erg25p and Erg26p; possible drug target, essential for viability; functional homolog of S. cerevisiae Erg27p
PIS51061.1	31.602946	31.9366273	31.7538531	31.18054713	31.42830821	31.10781936	0.092315708	0.943	-0.526	PIS51061.1	GFA1	Glucosamine-6-phosphate synthase, homotetrameric enzyme of chitin/hexosamine biosynthesis; inhibited by UDP-GlcNAc, FMDP, N-acyl peptide, kanosamine-6-P; functional homolog of S. cerevisiae [illegible]
PIS58410.1	24.9733655	24.667144	25.6614192	24.28139188	24.23726844	25.20710679	0.278624354	0.964	-0.525	PIS58410.1	orf19.2920	Ortholog(s) have role in ribosome biogenesis and cytosol localization
PIS52132.1	24.8939389	27.4980825	27.3860075	26.22734845	24.80704889	27.17219177	0.596563367	0.974	-0.524	PIS52132.1	orf19.5322	Ortholog(s) have phosphatidylinositol-3-phosphate binding activity
PIS52165.1	28.0159559	28.6951536	28.1754871	27.92633234	27.89686709	27.49417071	0.209119907	0.96	-0.523	PIS52165.1	RPT5	26S proteasome regulatory subunit; transcript regulated by Nrg1 and Mig1; regulated by Gcn2 and Gcn4; protein level decreases in stationary phase

PIS49707.1	30.6862073	31.4129449	31.4555992	29.60739984	31.07470065	31.30656267	0.246701733	0.962	-0.522	PIS49707.1	orf19.7052	Putative polyphosphatidylinositol phosphatase; possibly an essential gene, disruptants not obtained by UAU1 method
PIS51234.1	22.9156883	25.09896	26.6888595	24.20840281	24.12442934	24.8080532	0.569645299	0.973	-0.521	PIS51234.1	BUD23	Putative methyltransferase; Hap43-induced; repressed by prostaglandins
PIS48435.1	31.8041165	32.0970581	32.7602675	31.59043304	31.81784818	31.68934454	0.098305792	0.945	-0.521	PIS48435.1	LSC2	Putative succinate-CoA ligase beta subunit; regulated by Mig1, Tup1; induced in high iron; protein present in exponential and stationary growth phase yeast cells; Spider biofilm repressed
PIS51667.1	25.0241354	24.6076196	26.0464531	26.17889	23.28668529	24.65119768	0.421392544	0.97	-0.52	PIS51667.1	orf19.2973	Ortholog(s) have RNA binding, flap-structured DNA binding activity and role in nuclear-transcribed mRNA catabolic process, nuclear-transcribed mRNA poly(A) tail shortening, positive regulation of DNA metabolic process
PIS52131.1	29.0929431	29.2158593	29.2359899	28.18538268	28.76831213	29.03836747	0.3054946	0.965	-0.518	PIS52131.1	orf19.5321	Ortholog(s) have methylenetetrahydrofolate reductase (NAD(P)H) activity and role in methionine biosynthetic process
PIS51592.1	28.0283685	27.8430047	29.8676592	28.23108546	27.91015038	28.04373645	0.567865567	0.973	-0.518	PIS51592.1	TES1	Putative acyl-CoA thioesterase
PIS51427.1	30.9257159	30.6202612	31.0402002	30.54583347	30.14281823	30.3531966	0.062654901	0.933	-0.515	PIS51427.1	CDC39	Protein similar to S. cerevisiae Cdc39p, which is part of the CCR4-NOT transcription regulatory complex; transposon mutation affects filamentous growth

PIS51279.1	27.0562913	27.0558149	27.6135625	27.42426733	27.04281857	25.71424771	0.229318262	0.961	-0.515	PIS51279.1	orf19.5381	Ortholog(s) have mRNA binding activity and role in negative regulation of translation, nuclear-transcribed mRNA catabolic process, deadenylation-dependent decay, protein localization
PIS51085.1	24.4640314	24.2096146	25.6987994	23.60527893	24.67572678	24.54849222	0.512467011	0.972	-0.514	PIS51085.1	orf19.4286	Protein of unknown function; flow model biofilm induced; Spider biofilm induced
PIS51991.1	25.1370298	24.6374864	25.9789062	23.88169384	25.69433502	24.63427214	0.368943813	0.968	-0.514	PIS51991.1	orf19.6234	Putative U2 snRNP component; mutation confers hypersensitivity to 5-fluorocytosine (5-FC), 5-fluorouracil (5-FU), and tubercidin (7-deazaadenosine); Hap43-induced, Spider biofilm induced
PIS56832.1	25.6189847	26.4969109	26.4685137	25.75362579	25.39335224	25.89514652	0.229333568	0.961	-0.514	PIS56832.1	RPF1	Putative nucleolar protein with a predicted role in the assembly and export of the large ribosomal subunit; essential for growth; rat catheter and Spider biofilm induced
PIS55620.1	31.0236512	30.7092718	30.0014403	30.14423215	29.93568062	30.11704226	0.157297149	0.955	-0.512	PIS55620.1	PXA2	Putative peroxisomal, half-size adrenoleukodystrophy protein (ALD or ALDp) subfamily ABC transporter; Gcn4p-regulated
PIS55526.1	28.2212386	28.6861817	28.3732545	27.46251094	27.81504561	28.4670529	0.187540663	0.958	-0.512	PIS55526.1	TSM1	Putative transcription initiation factor TFIID subunit; transcript is upregulated in clinical isolates from HIV+ patients with oral candidiasis; Nrg1-regulated
PIS54947.1	23.5426501	21.5606376	24.3420082	23.71486661	22.75512466	21.44341923	0.445468174	0.97	-0.511	PIS54947.1	CRZ1	Calcineurin-regulated C2H2 transcription factor; role in maintenance of membrane integrity, azole tolerance; not required for mouse virulence; repressed by low iron; regulates Ca++ influx during alkaline pH response; Spider biofilm [illegible]

PIS54613.1	30.3719316	30.79464	31.6485017	30.44341097	30.48763744	30.35247555	0.197644839	0.959	-0.511	PIS54613.1	UBA1	Ubiquitin-activating enzyme; protein level decreases in stationary phase cultures
PIS52206.1	28.5352556	29.8683957	29.8461406	27.91099174	29.43096745	29.38072821	0.370777136	0.968	-0.509	PIS52206.1	GLR1	Glutathione reductase; upregulated by human neutrophils; oxidative stress-induced regulation via Cap1p; overexpression correlates with multidrug resistance in a cap1 mutant, farnesol induced; stationary phase enriched protein
PIS51750.1	26.8414855	26.0810977	26.1977992	25.31878301	26.45370393	25.82465435	0.19182779	0.958	-0.508	PIS51750.1	CWH41	Processing alpha glucosidase I, involved in N-linked protein glycosylation and assembly of cell wall beta 1,6 glucan; rat catheter biofilm repressed
PIS58466.1	29.9519663	30.2502836	30.523065	29.67969693	29.37084923	30.15097127	0.152431645	0.954	-0.508	PIS58466.1	orf19.2930	Predicted translation initiation factor; role in translational initiation; Spider biofilm repressed
PIS48757.1	25.4947528	26.7499507	25.9778852	26.88036335	25.3668288	24.4538556	0.433788634	0.97	-0.507	PIS48757.1	orf19.6908	Dihydrofolate synthetase involved in folic acid biosynthesis
PIS50566.1	29.3035073	26.3821592	27.5411494	25.40742628	29.11634233	27.18751452	0.629008809	0.975	-0.505	PIS50566.1	orf19.3939	Ortholog(s) have EMC complex localization
PIS54542.1	25.4023732	26.3141297	26.5210713	26.07599684	25.22676851	25.42390831	0.448181996	0.971	-0.504	PIS54542.1	orf19.3798	Ortholog(s) have tRNA (guanine(46)-N7)-methyltransferase activity, role in tRNA (guanine-N7)-methylation and tRNA (m7G46) methyltransferase complex localization

PIS50355.1	29.2231348	28.6154647	29.1924511	28.21507379	28.82469718	28.48287665	0.102221513	0.946	-0.503	PIS50355.1	DBP2	Putative DEAD-box family ATP-dependent RNA helicase; flucytosine induced; repressed in core stress response
PIS49682.1	31.952455	31.5663817	31.0223631	31.20323651	30.73860215	31.09016597	0.17362397	0.956	-0.503	PIS49682.1	orf19.2664	Ortholog(s) have mRNA 3'-UTR binding activity, role in DNA damage response and P-body, nuclear periphery localization
PIS49575.1	32.7394082	32.3493503	32.5234998	31.56502014	32.42565997	32.1138899	0.139926536	0.952	-0.503	PIS49575.1	orf19.909.1	Ortholog of C. dubliniensis CD36 : Cd36_17965, C. parapsilosis CDC317 : CPAR2_211700, C. auris B8441 : B9J08_004599 and Candida tenuis NRRL Y-1498 : CANTEDRAFT_112898
PIS48683.1	30.3115489	30.9121114	31.708451	29.99377668	30.7644325	30.66427053	0.467072082	0.971	-0.503	PIS48683.1	TIF3	Putative translation initiation factor; genes encoding ribosomal subunits, translation factors, and tRNA synthetases are downregulated upon phagocytosis by murine macrophage
PIS55804.1	31.6343155	32.5050332	32.4529924	31.70053167	31.67733823	31.70746115	0.136707726	0.952	-0.502	PIS55804.1	MSI3	Essential HSP70 family protein; required for fluconazole resistance and calcineurin-dependent transcription; interacts with Cgr1; transcript regulated by iron; rat catheter biofilm induced; farnesol repressed in biofilm; sumoylation target
PIS54591.1	32.8947752	33.2004994	32.7533605	31.8394448	32.78771535	32.72339066	0.123528295	0.95	-0.499	PIS54591.1	RPL9B	Ribosomal protein L9; repressed upon phagocytosis by murine macrophages; repressed by nitric oxide; protein levels decrease in stationary phase; Hap43-induced; Spider biofilm repressed
PIS51468.1	31.3533768	31.0947911	31.4481053	30.88509622	30.75626639	30.75739156	0.482893972	0.971	-0.499	PIS51468.1	SMP2	Putative Mg2+-dependent phosphatidate phosphatase; transcript regulated by Nrg1

PIS52237.1	26.3374101	27.1518597	26.5211144	26.6323387	24.88181305	27.00050714	0.42191798	0.97	-0.499	PIS52237.1	SNX4	Putative sorting nexin; induced during the mating process
PIS56918.1	28.9930278	27.8915475	26.3481516	27.58398947	27.41341105	26.74059713	0.56126947	0.973	-0.498	PIS56918.1	PHO84	High-affinity phosphate transporter; transcript regulated by white-opaque switch; Hog1, ciclopirox olamine or alkaline induced; caspofungin, stress repressed; required for normal TORC1 function
PIS55791.1	29.3525091	29.2974094	30.305419	29.27437014	29.18845103	29.00143651	0.145279992	0.953	-0.497	PIS55791.1	CDC53	Cullin, a scaffold subunit of the SCF ubiquitin-ligase complexes; depletion leads to increased filamentous growth and premature cell death
PIS56921.1	31.399697	31.8094008	32.0723683	30.40767074	31.53684383	31.84523399	0.261210234	0.963	-0.497	PIS56921.1	SAM2	S-adenosylmethionine synthetase; localizes to surface of hyphae, not yeast cells; alkaline, Hog1-induced; farnesol-downregulated; F-12/CO2 early biofilm induced; Spider biofilm repressed
PIS49457.1	26.6154581	24.9353513	25.167245	26.46209243	25.34337991	23.42846127	0.575149276	0.974	-0.495	PIS49457.1	CEX1	Ortholog(s) have tRNA binding activity, role in retrograde vesicle-mediated transport, Golgi to endoplasmic reticulum, tRNA export from nucleus and cytoplasm, nuclear pore localization
PIS56599.1	30.6685054	29.3894477	29.7061511	29.82148809	28.61053021	29.857442	0.338355348	0.967	-0.492	PIS56599.1	CCT5	T-complex protein 1, epsilon subunit; protein present in exponential and stationary growth phase yeast cultures; Spider biofilm repressed
PIS51842.1	29.5390195	29.8351844	29.6322301	28.71069144	29.42649152	29.39350384	0.132811961	0.951	-0.492	PIS51842.1	orf19.1764	Protein of unknown function; rat catheter and Spider biofilm induced

PIS55829.1	27.6466056	27.0332207	26.6457298	26.27971381	26.63952392	26.93447143	0.321339566	0.966	-0.491	PIS55829.1	MNN2	Alpha-1,2-mannosyltransferase; similar to S. cerevisiae Mnn2; role in cell wall integrity, temperature sensitivity; iron utilization in low iron; Tn mutation affects filamentous growth; filament induced; increased chitin exposes beta-glucan
PIS50545.1	26.0842821	28.1170261	28.343583	26.84267097	26.44004719	27.78783609	0.694038816	0.976	-0.491	PIS50545.1	orf19.899	Ortholog of C. dubliniensis CD36 : Cd36_18040, C. parapsilosis CDC317 : CPAR2_211790, C. auris B8441 : B9J08_004373 and Candida tenuis NRRL Y-1498 : CANTEDRAFT_109928
PIS51185.1	31.1159736	30.9847413	32.0549459	30.74628023	30.97832455	30.96225504	0.378338621	0.968	-0.49	PIS51185.1	orf19.7590	Putative NADH-ubiquinone oxidoreductase; identified in detergent-resistant membrane fraction (possible lipid raft component); predicted N-terminal acetylation; repressed by nitric oxide
PIS51491.1	28.6661497	29.5752706	29.8782548	29.36278458	28.77775441	28.51936149	0.23875898	0.962	-0.487	PIS51491.1	LHS1	Protein similar to S. cerevisiae Hsp70p; predicted Kex2p substrate; possibly essential, disruptants not obtained by UAU1 method; flow model biofilm repressed
PIS58540.1	25.5909923	24.430598	24.2514244	24.639713	24.42937891	23.74382466	0.350514712	0.967	-0.487	PIS58540.1	orf19.1841	Protein of unknown function; Hap43-induced gene
PIS51841.1	32.3284325	32.2337714	32.346765	31.79145442	31.89587228	31.76132399	0.353129416	0.967	-0.487	PIS51841.1	orf19.86	Putative glutathione peroxidase; induced by peroxide, exposure to neutrophils and macrophage blood fractions; repressed during infection of macrophages; Spider biofilm induced; flow model biofilm repressed
PIS52343.1	27.7167394	26.5859926	25.9454215	26.00179851	26.23847525	26.54904214	0.363969764	0.968	-0.486	PIS52343.1	MEA1	Protein similar to A. nidulans MesA, which is involved in localization of actin cables; Hap43p-induced gene

PIS50363.1	29.7802395	28.4570832	28.2493415	28.97674151	27.83584965	28.2168994	0.374218114	0.968	-0.486	PIS50363.1	orf19.3047	Protein kinase-related protein, required for normal sensitivity to caspofungin
PIS48398.1	27.9236084	27.1149032	26.7451731	27.63217222	26.39406426	26.29877963	0.39867241	0.969	-0.486	PIS48398.1	RIM1	Putative single-stranded DNA-binding protein; protein level decreases in stationary phase cultures; rat catheter biofilm repressed
PIS56681.1	30.9633664	30.0836698	29.5561799	27.33100777	31.07547178	30.74531236	0.584874891	0.974	-0.484	PIS56681.1	CSM3	Putative subunit of a replication fork-pausing checkpoint complex
PIS56556.1	23.8064123	22.1477879	20.0396285	23.50134204	20.65655761	20.38518545	0.656558533	0.975	-0.484	PIS56556.1	orf19.4293	Ortholog(s) have role in protein maturation by iron-sulfur cluster transfer, tRNA wobble uridine modification and CIA complex, cytosol, nucleus localization
PIS48502.1	26.2904315	27.1146225	25.8056251	26.65282909	25.47312731	25.63579896	0.517384923	0.972	-0.483	PIS48502.1	VID27	Protein similar to S. cerevisiae Vid27p; transposon mutation affects filamentous growth; mutation confers hypersensitivity to toxic ergosterol analog; fungal-specific (no human or murine homolog)
PIS48485.1	27.4032688	27.8052665	27.6059575	26.43238297	27.57088895	27.36589594	0.343931749	0.967	-0.482	PIS48485.1	orf19.1888	Putative nicotinamide riboside hydrolase; cleaves N-glycosidic bonds in nucleosides; pyrimidine salvage and nicotinamide riboside salvage pathways; Spider biofilm induced
PIS51896.1	30.521925	30.5045392	30.6322887	29.83310963	29.89885599	30.48707435	0.186482391	0.958	-0.48	PIS51896.1	orf19.2533.1	Protein with a predicted role in protein translocation from the endoplasmic reticulum

PIS51693.1	30.9309697	30.9646979	30.9792265	30.49153175	30.69715932	30.24889592	0.153753775	0.954	-0.479	PIS51693.1	GLN4	Putative tRNA-Gln synthetase; genes encoding ribosomal subunits, translation factors, tRNA synthetases are downregulated upon phagocytosis by murine macrophage; protein present in exponential and stationary growth phase yeast cultures
PIS56691.1	29.8299839	30.0788862	29.4954476	29.34326652	29.57683543	29.05618637	0.199661788	0.959	-0.476	PIS56691.1	TPD3	Subunit of protein serine/threonine phosphatase PPA2, involved in regulation of cytokinesis and morphogenesis; forms complex with catalytic subunit Pph21p that dephosphorylates septin Sep7p
PIS51067.1	28.0076656	28.4861114	28.6442325	27.64738612	27.98747857	28.08240281	0.152651905	0.954	-0.474	PIS51067.1	orf19.1626	Deoxyhypusine synthase; catalyzes formation of deoxyhypusine, the first step in hypusine biosynthesis; Spider biofilm repressed
PIS52021.1	33.0815651	33.6256696	33.6210236	32.64873808	33.19332972	33.0673452	0.193816411	0.958	-0.473	PIS52021.1	CDC19	Pyruvate kinase at yeast cell surface; Gcn4/Hog1/GlcNAc regulated; Hap43/polystyrene adherence induced; repressed by phagocytosis/farnesol; hyphal growth role; stationary phase enriched; flow model biofilm induced; Spider biofilm repressed
PIS52252.1	30.9873864	31.1103453	30.4729667	30.69816945	30.43847193	30.01474167	0.439364539	0.97	-0.473	PIS52252.1	orf19.1460	Putative glutamine-dependent NAD synthetase, involved in NAD salvage pathway
PIS48383.1	26.9131949	26.8632063	27.2013048	27.5475449	26.20195528	25.80960056	0.550674275	0.973	-0.473	PIS48383.1	orf19.2222	Putative casein kinase; plasma membrane-localized
PIS51473.1	27.6615395	26.9821577	27.3464153	26.57264998	27.02025691	26.98121958	0.375666835	0.968	-0.472	PIS51473.1	orf19.7235	Putative protein of unknown function; mutation confers hypersensitivity to amphotericin B

PIS56586.1	27.9190863	28.0032407	27.2870296	26.86695986	27.42720219	27.50411443	0.217009046	0.96	-0.47	PIS56586.1	orf19.5757	Ortholog(s) have FAD diphosphatase activity and role in flavin-containing compound metabolic process
PIS49481.1	29.7545747	29.7494265	30.9176952	29.53554919	29.90646975	29.57392433	0.266990792	0.963	-0.469	PIS49481.1	ELF1	Putative mRNA export protein; Walker A and B (ATP/GTP binding) motifs; required for wild-type morphology, growth; expressed in hyphal, pseudohyphal, and yeast form; Hap43-induced; Spider and flow model biofilm induced
PIS51962.1	26.5528125	28.458727	28.812134	28.02742947	27.24521891	27.14828467	0.485315362	0.972	-0.468	PIS51962.1	orf19.6739	Ortholog(s) have phosphopentomutase activity and role in guanosine catabolic process, inosine catabolic process, purine ribonucleoside salvage
PIS49629.1	31.0431581	31.0205864	30.4151386	30.23965934	30.66645944	30.17168972	0.120732695	0.949	-0.467	PIS49629.1	ARP2	Component of the Arp2/3 complex; required for virulence, hyphal growth, cell wall/cytoskeleton organization, not for endocytosis; mutation confers hypersensitivity to cytochalasin D; regulated by Gcn2 and Gcn4; Spider biofilm repressed
PIS58089.1	26.523244	26.3115526	26.0947391	25.74572977	25.58286897	26.19915697	0.104839455	0.946	-0.467	PIS58089.1	orf19.6396	Putative patatin-like phospholipase; similar to S. cerevisiae Nte1p, which is predicted to be a membrane protein; antigenic during human oral infection; Hap43p-repressed gene
PIS52289.1	25.1173232	24.6410928	24.799386	23.07457656	24.35979663	25.72191666	0.469412084	0.971	-0.467	PIS52289.1	PGA52	GPI-anchored cell surface protein of unknown function; Hap43p-repressed gene; fluconazole-induced; possibly an essential gene, disruptants not obtained by UAU1 method
PIS58821.1	30.1830093	30.5520475	30.0650841	29.65117671	30.2165111	29.53346157	0.238356735	0.962	-0.466	PIS58821.1	PRE5	Alpha6 subunit of the 20S proteasome; regulated by Gcn4; induced in response to amino acid starvation (3-AT); Spider biofilm repressed

PIS50502.1	24.7459499	24.3211482	25.8460081	24.69538818	24.66610489	24.15693914	0.591678442	0.974	-0.465	PIS50502.1	RIM13	Protease of the pH response pathway; likely to mediate activation of Rim101 via C-terminal cleavage; required for alkaline pH-induced hyphal growth and for normal chlamydospore formation; Hap43-repressed; flow model biofilm induced
PIS58894.1	28.7141363	28.7559913	29.4569148	28.78841948	28.51713697	28.22998932	0.156860253	0.954	-0.464	PIS58894.1	ARC1	Putative G4 nucleic acid binding protein; macrophage/pseudohyphal-repressed; protein enriched in stationary phase yeast-form cultures; Spider biofilm repressed
PIS52234.1	29.761008	29.772276	30.1797868	29.17876966	29.31417512	29.8281462	0.281194919	0.964	-0.464	PIS52234.1	orf19.1994	Ortholog(s) have phosphatidylinositol binding, phosphatidylinositol-3-phosphate binding activity, role in retrograde transport, endosome to Golgi and endosome localization
PIS52188.1	27.0601277	26.0359009	27.4434229	25.88491682	26.50188776	26.76321862	0.424123777	0.97	-0.463	PIS52188.1	orf19.1776	Putative pantetheine-phosphate adenylyltransferase (PPAT); which catalyzes 4th step in coenzyme A biosynthesis from pantothenate; rat catheter biofilm repressed
PIS54558.1	27.9297375	27.6110687	28.1569882	28.88923621	26.75686634	26.66194812	0.382696262	0.968	-0.463	PIS54558.1	orf19.1964	Protein of unknown function; repressed by fluphenazine treatment; induced by benomyl treatment and in an RHE model; regulated by Nrg1, Tup1
PIS58415.1	26.909628	26.8303943	25.174921	26.45445802	25.48711268	25.59023121	0.668404454	0.975	-0.461	PIS58415.1	orf19.2914	Ortholog of C. dubliniensis CD36 : Cd36_45760, C. parapsilosis CDC317 : CPAR2_401920, C. auris B8441 : B9J08_000913 and Candida tenuis NRRL Y-1498 : CANTEDRAFT_126814
PIS48586.1	29.7701191	29.8586447	29.3088544	28.92508578	29.45035607	29.18533966	0.307174756	0.965	-0.459	PIS48586.1	APL4	Predicted gamma-adaptin, large subunit of the clathrin-associated protein (AP-1) complex; that binds clathrin and is involved in vesicle mediated transport; induced in core caspofungin response; rat catheter biofilm repressed

PIS54839.1	26.4611812	28.7114973	28.829039	28.19331632	26.71761162	27.71485841	0.53980006	0.973	-0.459	PIS54839.1	orf19.1664	Protein of unknown function; expression downregulated in an ssr1 null mutant
PIS55703.1	27.9202581	27.8476108	26.9885364	26.59686413	27.24135563	27.54831571	0.211698341	0.96	-0.457	PIS55703.1	orf19.2867	Ortholog(s) have cargo adaptor activity, phosphatidylinositol-3-phosphate binding activity
PIS51662.1	25.02432	25.4056094	25.4318705	23.14669107	25.58070044	25.76466869	0.442975467	0.97	-0.457	PIS51662.1	orf19.2980	Ortholog(s) have RNA helicase activity and role in generation of catalytic spliceosome for first transesterification step, spliceosomal complex disassembly
PIS51805.1	25.2047365	25.6352834	25.4171356	25.49684697	24.3151506	25.07401143	0.373802498	0.968	-0.457	PIS51805.1	orf19.3302	Putative type-1 protein phosphatase targeting subunit; transcript repressed by yeast-hyphal switch; transcript induced by Mnl1p under weak acid stress; flow model biofilm induced
PIS56565.1	30.7751357	31.0457279	30.7602964	30.78853682	30.2186476	30.2032304	0.197133791	0.959	-0.457	PIS56565.1	RSR1	RAS-related protein; GTP/GDP cycling required for wild-type polar bud site selection, hyphal growth guidance; role in systemic virulence in mouse; geranylgeranylation predicted; suppresses S. cerevisiae cdc24-4 mutant heat sensitivity
PIS56809.1	27.1601942	26.3284478	26.4728233	25.17918013	26.56681035	26.84620751	0.46437021	0.971	-0.456	PIS56809.1	TBP1	Transcription initiation factor; binds TATA box sequence, binding does not require TFIIA; caspofungin repressed; functional homolog of S. cerevisiae and human TATA-binding proteins; Spider biofilm induced
PIS50495.1	29.7377736	27.9039864	29.4355867	29.41597538	28.37918158	27.91622596	0.395659884	0.969	-0.455	PIS50495.1	HAS1	Functional homolog of S. cerevisiae Has1p, which is a nucleolar protein of the DEAD-box ATP-dependent RNA helicase family that is involved in biogenesis of the ribosome, particularly the small (40S) subunit; caspofungin-downregulated

PIS51242.1	22.316398	26.449982	25.6410289	23.40360492	23.89317501	25.74840666	0.668354067	0.975	-0.454	PIS51242.1	ERG28	Ortholog(s) have protein-macromolecule adaptor activity, role in ergosterol biosynthetic process and endoplasmic reticulum membrane localization
PIS54810.1	25.4613308	25.3320431	25.7792912	25.20272832	24.77559971	25.2320151	0.356353682	0.968	-0.454	PIS54810.1	orf19.4711	Ortholog of C. dubliniensis CD36 : Cd36_40890, C. parapsilosis CDC317 : CPAR2_401100, C. auris B8441 : B9J08_001954 and Candida tenuis NRRL Y-1498 : CANTEDRAFT_119698
PIS48268.1	24.6032365	26.7826039	25.9691244	24.56828245	25.63619111	25.79529433	0.426393273	0.97	-0.452	PIS48268.1	TSR2	Protein with a predicted role in pre-rRNA processing; repressed by prostaglandins
PIS58737.1	25.6807646	26.9502318	28.1831902	26.37478588	26.06049234	27.0301348	0.568663595	0.973	-0.45	PIS58737.1	RKI1	Ortholog(s) have ribose-5-phosphate isomerase activity and role in pentose-phosphate shunt, pyridoxine biosynthetic process
PIS56777.1	26.2103287	25.6205774	25.0359526	23.72173508	26.0513758	25.74584966	0.475620069	0.971	-0.449	PIS56777.1	orf19.302	Ortholog(s) have guanyl-nucleotide exchange factor activity, role in endoplasmic reticulum to Golgi vesicle-mediated transport and TRAPPI protein complex, TRAPPII protein complex, TRAPPIII protein complex localization
PIS58742.1	27.8682912	28.488121	28.6946824	27.4837951	28.14759111	28.07328812	0.301411292	0.965	-0.449	PIS58742.1	RPA135	Putative RNA polymerase I subunit A135; repressed by prostaglandins
PIS50424.1	30.1767019	29.9331957	30.177709	29.91727917	29.65701065	29.37639467	0.623249343	0.974	-0.446	PIS50424.1	orf19.3232	Putative transporter; mutation confers hypersensitivity to toxic ergosterol analog; fungal-specific (no human or murine homolog)

PIS48243.1	31.1396493	31.6828278	33.1078616	31.50898099	31.8226739	31.26337184	0.466484391	0.971	-0.445	PIS48243.1	PFK2	Phosphofructokinase beta subunit; fructose 2,6-bisphosphate, AMP activated; ATP inhibited; phagocytosis, hyphal repressed; fluconazole-induced; stationary-phase enriched; flow model biofilm induced; rat catheter/Spider biofilm repressed
PIS52014.1	25.0968262	24.9457654	24.2267435	24.37087559	24.149445	24.41821201	0.56104204	0.973	-0.444	PIS52014.1	ACP12	Putative mitochondrial acyl carrier protein
PIS54975.1	30.2675802	30.518848	30.7530491	28.59341058	30.95003366	30.66541506	0.414152986	0.97	-0.444	PIS54975.1	DED1	Predicted ATP-dependent RNA helicase; RNA strand annealing activity; Spider biofilm induced
PIS50561.1	26.7947984	26.1436718	25.9740644	27.1637502	24.7390838	25.6767899	0.606900789	0.974	-0.444	PIS50561.1	GRR1	F-box protein component of the SCF ubiquitin-ligase complex required for cell cycle progression; involved in negative control of pseudohyphal growth; regulates stability of Ume6p in response to CO2
PIS48544.1	25.8095711	27.2703393	26.7156958	26.09686601	26.64939379	25.71874779	0.274556278	0.964	-0.444	PIS48544.1	orf19.4621	Ortholog(s) have P-body localization
PIS49704.1	26.4372124	27.0412904	27.9260412	24.59457655	26.65567453	28.82667751	0.584145159	0.974	-0.443	PIS49704.1	orf19.6558	Ortholog(s) have GTPase activator activity and cytosol localization
PIS58425.1	27.4381865	27.8271708	27.6967232	27.43287907	27.06541609	27.13479544	0.282359497	0.964	-0.443	PIS58425.1	SWC4	Subunit of the NuA4 histone acetyltransferase complex

PIS55431.1	23.2186793	23.4746081	23.9333541	22.8656914	22.83041173	23.60761404	0.214804455	0.96	-0.441	PIS55431.1	DIP5	Dicarboxylic amino acid permease; mutation confers hypersensitivity to toxic ergosterol analog; induced upon phagocytosis by macrophage; Gcn4-regulated; upregulated by Rim101 at pH 8; rat catheter and Spider biofilm induced
PIS58704.1	23.9384881	24.2301436	24.6298317	24.05938006	23.16300634	24.25216234	0.648236881	0.975	-0.441	PIS58704.1	HAP42	Predicted transcription factor; possibly an essential gene, disruptants not obtained by UAU1 method
PIS54634.1	26.703746	27.854816	28.4158187	27.69858077	27.28125186	26.67238662	0.296245913	0.965	-0.441	PIS54634.1	OST1	Alpha subunit of the oligosaccharyltransferase complex of the ER lumen; catalyzes asparagine-linked glycosylation of newly synthesized proteins; Spider biofilm repressed
PIS58835.1	25.1937197	26.7148782	27.8773808	26.38557699	26.10410031	25.98038287	0.493990197	0.972	-0.439	PIS58835.1	orf19.5897	Ortholog(s) have role in positive regulation of TORC1 signaling and GATOR2 complex, Seh1-associated complex localization
PIS48781.1	29.0977086	28.0550209	29.078651	27.28085771	28.52176745	29.11367865	0.516473837	0.972	-0.438	PIS48781.1	SNP3	Putative U6 snRNA-associated protein; transcript regulated by Mig1
PIS54940.1	27.1811241	26.7976734	28.3403582	26.58509733	26.87416599	27.54761545	0.435761424	0.97	-0.437	PIS54940.1	IFG3	Putative D-amino acid oxidase; Spider biofilm induced
PIS54790.1	24.359067	23.7850346	27.1956694	25.07126404	24.40723118	24.55353756	0.640825013	0.975	-0.436	PIS54790.1	orf19.7433	Ortholog(s) have structural constituent of nuclear pore activity

PIS49618.1	29.4317499	29.9197784	29.2378233	29.23727116	29.16586239	28.88405393	0.220715645	0.96	-0.434	PIS49618.1	YBP1	Protein involved in response to oxidative stress, binds and stabilizes Cap1p transcription factor in response to H2O2; essential for macrophage killing
PIS52070.1	29.8245943	29.6229881	31.0480999	29.31998137	29.94238704	29.94224222	0.374048619	0.968	-0.43	PIS52070.1	MCR1	NADH-cytochrome-b5 reductase; soluble in hyphae; alkaline downregulated; farnesol, ketoconazole or flucytosine induced; protein present in exponential and stationary growth phase yeast; YNB biofilm induced; rat catheter biofilm repressed
PIS58157.1	23.6693655	24.4385209	26.3893743	24.65826076	24.55248731	24.00343548	0.634894416	0.975	-0.428	PIS58157.1	HNT2	Putative dinucleoside triphosphate hydrolase; induced upon low-level peroxide stress
PIS49819.1	26.7020887	27.5820469	28.0915902	26.33732951	27.4121504	27.3427733	0.418535432	0.97	-0.428	PIS49819.1	orf19.4382	Ortholog(s) have role in retrograde vesicle-mediated transport, Golgi to endoplasmic reticulum and COPI vesicle coat localization
PIS51819.1	26.6245681	27.7844077	28.7085772	26.39128975	27.14366773	28.29913628	0.447826381	0.971	-0.428	PIS51819.1	orf19.94	Protein of unknown function; Spider biofilm induced
PIS51926.1	24.8588277	26.6829888	26.8722115	25.60994587	25.89268953	25.62781473	0.567136656	0.973	-0.428	PIS51926.1	SEC72	ER protein-translocation complex component; regulated by Gcn4p; repressed in response to amino acid starvation (3-aminotriazole treatment); sumoylation target
PIS48649.1	26.9350599	26.1455607	26.1615777	26.7796282	25.80628675	25.37526351	0.592851397	0.974	-0.427	PIS48649.1	orf19.3223.1	Putative 12kDa subunit of mitochondrial NADH-ubiquinone oxidoreductase; gene has intron

PIS58325.1	30.3130418	29.8408594	30.3152141	30.02097988	29.73626106	29.43556589	0.224842723	0.961	-0.425	PIS58325.1	GCR3	Functional homolog of S. cerevisiae Gcr3, which acts in regulation of glycolytic genes; no intron predicted, in contrast to intron in S. cerevisiae GCR3 gene
PIS51168.1	29.7950103	30.1088545	30.176594	29.28421496	29.73542802	29.78451171	0.136373408	0.952	-0.425	PIS51168.1	URA1	Dihydroorotate dehydrogenase; de novo pyrimidine biosynthesis; regulated by yeast-hypha switch, Nrg1/Mig1/Tup1; flow model biofilm induced; rat catheter and Spider biofilm repressed
PIS56888.1	28.4419339	28.2718677	28.2597902	27.75265153	27.91925713	28.03178948	0.165222353	0.955	-0.423	PIS56888.1	MRT4	Putative mRNA turnover protein; Hap43-induced; mutation confers hypersensitivity to tubercidin (7-deazaadenosine); rat catheter biofilm induced
PIS56931.1	31.6457056	31.1360105	31.9680014	31.59700901	31.23199946	30.65196791	0.7248199	0.976	-0.423	PIS56931.1	PRE1	Putative beta 4 subunit of the 20S proteasome; Rim101-induced at acid pH; flucytosine induced; amphotericin B repressed
PIS51536.1	33.8616008	33.6923209	33.2019116	32.90726334	33.65048984	32.93045854	0.215709927	0.96	-0.423	PIS51536.1	RPL17B	Ribosomal protein L17; mutation confers hypersensitivity to 5-FU, tubercidin; repressed upon phagocytosis by macrophage; Hap43-induced; Spider biofilm repressed
PIS51631.1	29.6204286	29.8070397	29.4923557	29.50265414	28.94594021	29.20604138	0.148652258	0.953	-0.422	PIS51631.1	MNS1	Alpha-1,2-mannosidase; processes Man9GlcNAc2 to Man8GlcNAc2 isomer B; member of ER localized glycosyl hydrolase family 47; ER form is converted by Kex2 to cytosolic form; flow model biofilm repressed
PIS54907.1	28.7658568	29.2867235	29.4063036	28.86541603	28.65127082	28.67797177	0.206855052	0.959	-0.421	PIS54907.1	orf19.4131	Ortholog(s) have EMC complex localization

PIS54767.1	30.0263929	29.5720914	30.0740926	29.83528439	29.35232582	29.22056725	0.555555702	0.973	-0.421	PIS54767.1	orf19.4357	Putative protein similar to S. cerevisiae Mgr3p, a subunit of the i-AAA protease supercomplex that degrades misfolded mitochondrial proteins
PIS58656.1	31.9067114	32.5831013	33.0201342	31.91981957	32.00884106	32.31899592	0.301107622	0.965	-0.421	PIS58656.1	SSB1	HSP70 family heat shock protein; mRNA in yeast and germ tubes; at yeast cell surface, not hyphae; antigenic in human/mouse infection; macrophage, GCN-induced; possibly essential; sumoylation target; Hap43-induced; Spider biofilm repressed
PIS52055.1	24.7705086	26.7999761	24.8885983	23.84216312	24.35054771	27.00568219	0.71955456	0.976	-0.42	PIS52055.1	orf19.3051	Protein of unknown function; S. pombe ortholog SPAC17A2.02c plays a role in resistance to cadmium; colony morphology-related gene regulation by Ssn6; Spider biofilm repressed
PIS51152.1	28.1073811	29.030368	29.3813538	28.28999783	28.52075795	28.45135758	0.272769117	0.964	-0.419	PIS51152.1	orf19.3213	Protein of unknown function; Spider biofilm induced
PIS52271.1	24.8630586	26.0303567	25.6534021	24.96983477	25.16710427	25.15549356	0.296452235	0.965	-0.418	PIS52271.1	MIT1	Mannosylinositol phosphorylceramide (MIPC) synthase catalytic subunit; sphingolipid biosynthesis; fluconazole, caspofungin induced; macrophage-repressed; Spider biofilm induced
PIS51798.1	28.6782417	25.5651079	25.4420846	27.35229198	25.46417079	25.61885915	0.586379316	0.974	-0.417	PIS51798.1	ATC1	Cell wall acid trehalase; catalyzes hydrolysis of the disaccharide trehalose; similar to S. cerevisiae vacuolar acid trehalase (Ath1p); Hap43p-repressed gene
PIS51900.1	28.198777	27.5214044	27.1050131	28.03630261	26.72305111	26.81526048	0.507231053	0.972	-0.417	PIS51900.1	orf19.2541	Ortholog(s) have 3'-5'-DNA exonuclease activity, endonuclease activity and role in apoptotic DNA fragmentation, cellular response to oxidative stress

PIS48738.1	26.0621728	24.9744179	27.1295516	25.49609295	25.49150528	25.93040235	0.559870277	0.973	-0.416	PIS48738.1	orf19.2150	Putative ortholog of mammalian electron transfer flavoprotein complex subunit ETF-alpha; Spider biofilm repressed
PIS58115.1	28.9922805	29.0650361	29.036608	27.44063688	29.04810163	29.35773271	0.371456359	0.968	-0.416	PIS58115.1	orf19.6264.3	Ortholog(s) have role in endoplasmic reticulum to Golgi vesicle-mediated transport and COPII-coated ER to Golgi transport vesicle, Golgi apparatus, endoplasmic reticulum, peroxisome localization
PIS51697.1	31.3093945	31.5680498	31.3051992	30.51353662	31.21308412	31.21024536	0.187652742	0.958	-0.415	PIS51697.1	NIP1	Putative translation initiation factor; mutation confers hypersensitivity to roridin A and verrucarin A; genes encoding ribosomal subunits, translation factors, and tRNA synthetases are downregulated upon phagocytosis by murine macrophage
PIS58834.1	29.0760933	28.0870047	28.4380829	28.880192	28.07021849	27.40438435	0.383794154	0.969	-0.415	PIS58834.1	PKC1	Protein kinase C; functional homolog of S. cerevisiae Pkc1p; mutant has abnormal yeast-form cell morphology and increased cell lysis; activated by phosphatidylserine; target of antifungal, cercosporamide; R400P mutant is activated
PIS54914.1	26.3662892	27.6331071	27.568117	25.45845297	26.87420337	27.99276536	0.619577654	0.974	-0.414	PIS54914.1	orf19.1940	Ortholog(s) have 3-demethoxyubiquinol 3-hydroxylase activity, role in ubiquinone biosynthetic process and mitochondrial inner membrane, mitochondrion localization
PIS51555.1	31.1029059	31.4735243	31.2462806	31.14709327	30.79377405	30.64310993	0.529196234	0.973	-0.413	PIS51555.1	ATP7	Putative subunit of the F1F0-ATPase complex; colony morphology-related gene regulation by Ssn6; farnesol, macrophage-downregulated protein abundance; protein present in exponential and stationary yeast growth phases; Hap43-induced
PIS52088.1	26.5770682	26.8240187	26.5874254	24.86326156	26.74229543	27.14952828	0.490614757	0.972	-0.411	PIS52088.1	MTM1	Ortholog(s) have pyridoxal phosphate binding activity, role in intracellular iron ion homeostasis, pyridoxal phosphate transport and mitochondrion localization

PIS51271.1	29.0319794	28.8874457	27.472903	27.83443445	28.08033324	28.2435284	0.315154229	0.966	-0.411	PIS51271.1	SCT1	Putative glycerol-3-phosphate O-acyltransferase; fungal-specific (no human or murine homolog)
PIS49493.1	24.9279317	26.2547178	26.7031316	25.64266435	25.35681147	25.65531852	0.434580081	0.97	-0.41	PIS49493.1	SPT20	Putative transcription factor; downregulated upon adherence to polystyrene; flucytosine repressed
PIS52337.1	31.7266951	31.7879609	31.7751843	31.47734137	31.40116819	31.18772658	0.593520311	0.974	-0.408	PIS52337.1	ADE2	Phosphoribosylaminoimadazole carboxylase; role in adenine biosynthesis; required for normal growth and virulence in immunosuppressed mouse infection; not induced in GCN response, in contrast to S. cerevisiae ADE2;
PIS56614.1	27.4435255	28.5132735	28.083846	27.68307776	27.50437161	27.6292399	0.326857246	0.966	-0.408	PIS56614.1	MSC7	S. cerevisiae ortholog Mcs7 has a role in reciprocal meiotic recombination; Spider biofilm repressed
PIS50403.1	29.7825853	28.4129214	28.7679052	28.47819475	28.49391627	28.77452341	0.355776532	0.968	-0.406	PIS50403.1	RPA190	Putative RNA polymerase I subunit A190; Hap43p-induced gene; flucytosine induced
PIS58612.1	28.8856206	29.4718029	30.5639837	28.99616697	29.57643782	29.13398614	0.404163432	0.969	-0.405	PIS58612.1	SNZ1	Stationary phase protein; vitamin B synthesis; induced byyeast-hypha switch, 3-AT or in azole-resistant strain overexpressing MDR1; soluble in hyphae; regulated by Gcn4, macrophage; Spider biofilm induced; rat catheter biofilm repressed
PIS58476.1	29.9122888	30.2386161	30.1955513	29.51925379	30.1869996	29.42936519	0.191826003	0.958	-0.404	PIS58476.1	FRS2	Putative tRNA-Phe synthetase; downregulated upon phagocytosis by murine macrophage; protein present in exponential and stationary growth phase yeast cultures; Spider biofilm repressed

PIS51577.1	29.2488443	29.1382841	28.4005262	29.20669787	28.42130475	27.95420318	0.611881276	0.974	-0.402	PIS51577.1	orf19.6061	Ortholog(s) have role in N-acylethanolamine metabolic process, N-acylphosphatidylethanolamine metabolic process and mitochondrial inner membrane localization
PIS55698.1	29.1445805	28.6377518	29.2888563	28.93621097	28.34752094	28.58505448	0.292136518	0.965	-0.401	PIS55698.1	NOP1	Nucleolar protein; flucytosine induced; Hap43-induced; Spider biofilm repressed
PIS49649.1	25.9195157	25.4567005	24.7738163	25.78422422	24.82825022	24.33581631	0.522679451	0.972	-0.401	PIS49649.1	orf19.1861	BAR domain-containing protein, forms heterodimer with Rvs162p that binds liposomes in vitro; flow model biofilm induced
PIS51584.1	24.8028283	24.7951214	24.8513441	24.85253747	24.63508939	23.75931345	0.359440393	0.968	-0.401	PIS51584.1	orf19.5209	Ortholog(s) have role in cytoplasm to vacuole transport by the Cvt pathway, intra-Golgi vesicle-mediated transport and Golgi transport complex localization
PIS54606.1	25.4170425	27.1534708	26.0572805	23.71674866	26.68819989	27.01844809	0.762848927	0.977	-0.401	PIS54606.1	orf19.7444	Ortholog(s) have transcription factor TFIIH holo complex localization
PIS54552.1	27.4903742	27.6605733	28.3629011	27.00704627	26.96247559	28.34013124	0.334316778	0.967	-0.401	PIS54552.1	TIF34	Putative translation initiation factor eIF3, p39 subunit; mutation confers hypersensitivity to roridin A, verrucarin A; downregulated upon phagocytosis by murine macrophages; Spider biofilm repressed
PIS50461.1	27.2689874	28.5590658	28.6547423	27.76949422	27.67368222	27.84713556	0.310361219	0.966	-0.397	PIS50461.1	HSP78	Heat-shock protein; regulated by macrophage response, Nrg1, Mig1, Gcn2, Gcn4, Mnl1p; heavy metal (cadmium) stress-induced; stationary phase enriched protein; rat catheter and Spider biofilm induced

PIS55658.1	23.9812158	27.6291735	26.4118398	25.04781516	26.14158159	25.64085844	0.632492235	0.975	-0.397	PIS55658.1	SEC65	Component of the protein-targeting Signal Recognition Particle (SRP); similar to mammalian Srp19; functional homolog of S. cerevisiae Sec65; Tbf1-induced
PIS49566.1	26.5965999	30.0092955	30.4613454	28.80337993	28.58418885	28.49374442	0.803098043	0.977	-0.395	PIS49566.1	FAD2	Delta-12 fatty acid desaturase, involved in production of linoleic acid, which is a major component of membranes
PIS55540.1	30.9377288	30.7676731	31.1382304	30.27880787	30.63178984	30.74935375	0.270246481	0.964	-0.395	PIS55540.1	orf19.2095	Ortholog(s) have role in negative regulation of transcription by RNA polymerase II and cytosol localization
PIS58951.1	26.3255068	26.857121	27.4653656	27.70789292	24.93021866	26.8263363	0.583122027	0.974	-0.395	PIS58951.1	OXR1	Ortholog(s) have role in vacuolar acidification and fungal-type vacuole membrane, mitochondrion localization
PIS56755.1	28.1157789	28.6639547	28.1545502	27.2276514	28.32270027	28.20341898	0.377310422	0.968	-0.394	PIS56755.1	orf19.967	Major mitochondrial nuclease; has RNAse and DNA endo- and exonucleolytic activities; roles in mitochondrial recombination, apoptosis and maintenance of polyploidy; Spider biofilm repressed
PIS58926.1	31.6725201	31.7377544	31.5751437	31.12685396	31.47027168	31.21558458	0.151121864	0.954	-0.391	PIS58926.1	orf19.6082	Ortholog(s) have unfolded protein binding activity, role in protein folding, protein localization to cell surface and endoplasmic reticulum membrane localization
PIS51404.1	23.6520187	23.6903973	24.9982757	23.76109399	22.9362031	24.47647434	0.55713319	0.973	-0.389	PIS51404.1	orf19.4394	Protein of unknown function; induced by alpha pheromone in SpiderM medium

PIS58209.1	29.0921831	28.4808214	28.7746648	28.54039752	28.3956036	28.25138377	0.535139322	0.973	-0.387	PIS58209.1	orf19.7488	Component of the SSU processome; predicted role in pre-18S rRNA processing; Spider biofilm induced
PIS54958.1	24.490538	27.4151193	27.4036212	25.83623576	25.8611456	26.45375169	0.588088331	0.974	-0.386	PIS54958.1	orf19.7244	Putative fumarylacetoacetate hydrolase; induced by nitric oxide independent of Yhb1; regulated by Sef1, Sfu1, Hap43; flow model biofilm induced
PIS49791.1	29.3271527	29.0751135	29.6462913	29.10217356	28.81711736	28.97507357	0.30519416	0.965	-0.385	PIS49791.1	orf19.3185	Ortholog(s) have acetyltransferase activator activity, peptide alpha-N-acetyltransferase activity, ribosome binding activity, role in N-terminal protein amino acid acetylation and NatA complex localization
PIS56830.1	25.3740946	26.2601985	24.4628104	25.65679319	24.9820033	24.30787998	0.601110484	0.974	-0.383	PIS56830.1	orf19.4760	Putative protein-histidine N-methyltransferase; Spider biofilm induced
PIS54537.1	28.0185649	27.9017662	28.5140856	27.63680353	27.73220339	27.91565487	0.383437432	0.969	-0.383	PIS54537.1	orf19.5433	Ortholog(s) have protein folding chaperone activity, role in mitochondrial respiratory chain complex III assembly and mitochondrial matrix, mitochondrion localization
PIS49612.1	31.6606559	31.5080847	31.2050083	30.92959281	31.09566533	31.20355531	0.399724174	0.969	-0.382	PIS49612.1	KEM1	5'->3' exoribonuclease of cytoplasmic stress granules; role in filamentous growth; complements slow growth/mating of S. cerevisiae kem1 mutant; required for SD or Spider medium biofilm formation
PIS58920.1	28.906298	29.3752012	28.4418831	28.9340656	28.62832419	28.01367224	0.498944885	0.972	-0.382	PIS58920.1	orf19.2544	Ortholog(s) have aminoacyl-tRNA hydrolase activity and role in negative regulation of proteasomal ubiquitin-dependent protein catabolic process

PIS51307.1	29.2209557	29.0524146	28.7021891	28.81371589	28.72358845	28.29655742	0.34777959	0.967	-0.381	PIS51307.1	URA4	Dihydroorotase; protein present in exponential and stationary growth phase yeast cultures
PIS52051.1	32.1674619	31.7285981	31.9455972	30.463785	31.88345858	32.35472639	0.369733555	0.968	-0.38	PIS52051.1	orf19.5773	Putative dipeptidyl-peptidase III; protein detected by mass spec in exponential and stationary phase cultures; Hog1p-induced; clade-associated gene expression
PIS52228.1	23.8379702	25.416706	26.6692091	24.20554843	25.17709657	25.40300471	0.591066558	0.974	-0.379	PIS52228.1	orf19.6923	Ortholog(s) have chromatin binding activity, role in RNA polymerase II preinitiation complex assembly, transcription by RNA polymerase II and transcription factor TFIID complex localization
PIS52378.1	30.3998688	30.5537966	30.7129571	30.00827235	30.27772525	30.24514522	0.397024144	0.969	-0.378	PIS52378.1	HRT2	Protein described as having a role in Ty3 transposition; repressed in hyphae; stationary phase enriched protein; rat catheter and Spider biofilm repressed
PIS51937.1	28.9946614	30.1256694	30.6051855	28.6961108	29.51070852	30.39142931	0.482161216	0.971	-0.376	PIS51937.1	CTP1	Putative citrate transport protein; flucytosine induced; amphotericin B repressed, caspofungin repressed; Hap43p-induced gene
PIS54894.1	27.9495691	27.5225312	27.0066215	26.95145354	26.97887539	27.42008404	0.558564241	0.973	-0.376	PIS54894.1	MDN1	Putative midasin, a very large dynein-related AAA-type ATPase; induced during the mating process
PIS49473.1	29.1965015	29.9520959	30.1372632	28.42431765	29.82552849	29.90889494	0.393311587	0.969	-0.376	PIS49473.1	orf19.7306	Aldo-keto reductase; increased transcript associated with MDR1 overexpression, benomyl or long-term fluconazole treatment; overexpression does not affect drug or oxidative stress sensitivity; stationary phase enriched; flow biofim repressed

PIS56758.1	27.7695038	27.8038596	26.9616962	26.67347586	27.60788008	27.12766848	0.331667884	0.967	-0.375	PIS56758.1	orf19.969	Ortholog(s) have ribose phosphate diphosphokinase activity, role in 5-phosphoribose 1-diphosphate biosynthetic process, fungal-type cell wall organization and cytoplasm, ribose phosphate diphosphokinase complex localization
PIS54499.1	30.919924	31.5776951	32.1658424	30.41816585	31.64404775	31.4748745	0.43720548	0.97	-0.375	PIS54499.1	PHB1	Putative prohibitin; identified in detergent-resistant membrane fraction (possible lipid raft component); predicted N-terminal acetylation; Hap43p-repressed gene
PIS58945.1	26.6479284	26.5404316	24.2344504	25.94805501	26.73012197	23.62507021	0.616725131	0.974	-0.373	PIS58945.1	GLE2	Putative nuclear pore complex; possibly an essential gene, disruptants not obtained by UAU1 method; rat catheter biofilm repressed
PIS54477.1	31.8872593	31.463527	31.0980076	30.94766626	31.23663851	31.14911437	0.207531639	0.959	-0.372	PIS54477.1	HGT2	Putative MFS glucose transporter; 20 member C. albicans glucose transporter family; 12 probable membrane-spanning segments; expressed in rich medium with 2% glucose; rat catheter and Spider biofilm induced
PIS58237.1	28.6230073	28.8750638	29.2567169	29.29897595	28.0301667	28.30919602	0.519900412	0.972	-0.372	PIS58237.1	orf19.828	Putative ribosomal protein, large subunit, mitochondrial precursor; repressed by prostaglandins; Spider biofilm repressed
PIS52263.1	24.783368	23.7774479	24.1161702	23.75354074	23.71608104	24.09369106	0.547458886	0.973	-0.371	PIS52263.1	REI1	Putative cytoplasmic pre-60S factor; Hap43-induced; repressed by prostaglandins
PIS58044.1	28.5707345	29.1522985	30.8046636	28.77242977	29.22825047	29.41644143	0.539681183	0.973	-0.37	PIS58044.1	orf19.3900	Ortholog(s) have role in mitochondrial fusion and mitochondrial outer membrane localization

PIS51692.1	27.6637759	27.7161294	27.9838094	26.66659107	27.22970444	28.35819298	0.482682566	0.971	-0.37	PIS51692.1	orf19.7166	Predicted mitochondrial cardiolipin-specific phospholipase; upregulated in an azole-resistant strain that overexpresses MDR1; induced by Mnl1 under weak acid stress; rat catheter and Spider biofilm induced
PIS51210.1	26.3198283	26.3735312	25.1849951	24.43797174	26.22337888	26.1090499	0.52327703	0.972	-0.369	PIS51210.1	orf19.2278	Putative 20S proteasome assembly protein; filament induced; induced by alpha pheromone in SpiderM medium
PIS55504.1	26.762393	25.8680404	27.0800956	27.47908765	25.79214689	25.33231379	0.490336534	0.972	-0.369	PIS55504.1	SMI1	Cell wall biosynthesis protein; Hap43, caspofungin-repressed; Cyr1-induced in hyphal cells; reduced biofilm cell wall glucan in mutant; possibly essential (UAU1 method); flow model biofilm induced
PIS58498.1	29.7792553	29.5939224	30.1595271	28.78524003	29.52649118	30.11765636	0.469648326	0.971	-0.368	PIS58498.1	RPS21B	Ribosomal protein S21; regulated by Nrg1, Tup1; colony morphology-related gene regulation by Ssn6; positively regulated by Tbf1, Hap43; Spider biofilm repressed
PIS48556.1	26.2305449	25.4005489	25.7595323	25.33163748	24.92575196	26.03126189	0.510330437	0.972	-0.367	PIS48556.1	orf19.4542	Ortholog(s) have cyclin-dependent protein serine/threonine kinase activator activity, cyclin-dependent protein serine/threonine kinase regulator activity
PIS49504.1	23.3492578	23.6454429	23.4752581	23.1379096	22.51717721	23.71326447	0.576610306	0.974	-0.367	PIS49504.1	orf19.5680	Protein with predicted hydrolase domains; similar to a universal stress protein family protein; possibly essential, disruptants not obtained by UAU1 method; Spider biofilm repressed
PIS58874.1	30.1264058	29.907138	29.9949422	29.01571232	30.02657037	29.88531122	0.270505111	0.964	-0.367	PIS58874.1	RPN6	Putative 26S proteasome subunit; Hap43p-induced gene; regulated by Gcn2p and Gcn4p

PIS52007.1	23.7571499	25.3937565	26.5852168	25.72772003	24.2796106	24.63169732	0.643288535	0.975	-0.366	PIS52007.1	LTP1	Putative protein phosphatase of the PTP family (tyrosine-specific), similar to S. cerevisiae Ltp1p
PIS58970.1	32.1656318	32.3144062	32.3678329	31.86324206	31.82973536	32.05814138	0.217652941	0.96	-0.366	PIS58970.1	VPS1	Dynamin-family GTPase-related protein; induced upon adherence to polystyrene; regulated by Gcn2p and Gcn4p
PIS49726.1	29.4135531	30.1233756	31.0860653	30.49846412	29.73913266	29.28975655	0.62671751	0.975	-0.365	PIS49726.1	FESUR1	Putative ubiquinone reductase; transcriptionally induced by interaction with macrophage; alkaline downregulated; repressed by nitric oxide; clade-associated gene expression; Hap43p-repressed gene
PIS58580.1	29.2809344	29.211917	29.4415916	28.65878482	29.06491407	29.11457389	0.227848442	0.961	-0.365	PIS58580.1	URA6	Ortholog(s) have UMP/dUMP kinase activity, adenylate kinase activity, role in 'de novo' pyrimidine nucleobase biosynthetic process, nucleobase-containing compound metabolic process and cytoplasm, nucleus localization
PIS48547.1	28.2412178	27.4009156	28.0317732	26.85564146	27.53130496	28.19486172	0.393939001	0.969	-0.364	PIS48547.1	POL30	Proliferating cell nuclear antigen (PCNA), forms homotrimeric sliding clamp for DNA polymerases; RNA abundance regulated by tyrosol, cell density; induced by flucytosine, interaction with macrophages; stationary phase enriched protein
PIS55736.1	27.8415741	27.5768596	27.5311962	27.10872958	27.10985597	27.64236457	0.396549001	0.969	-0.363	PIS55736.1	GCD1	Putative translation initiation factor; transcript regulated by Mig1; repressed upon phagocytosis by murine macrophage
PIS54475.1	30.5854549	30.0654824	30.1519608	29.47668143	30.40792208	29.82891787	0.572194008	0.973	-0.363	PIS54475.1	orf19.4779	Putative transporter; slightly similar to the Sit1p siderophore transporter; Gcn4p-regulated; fungal-specific; induced by Mnl1p under weak acid stress

PIS49700.1	24.1499598	25.521742	23.3794201	24.57862964	23.12057529	24.26350046	0.588513523	0.974	-0.363	PIS49700.1	orf19.6566	Mitochondrial protein required for expression of respiratory chain complex IV (cytochrome c oxidase)
PIS51882.1	24.5844474	26.429749	26.2888432	25.0855479	25.26753531	25.85982705	0.651540367	0.975	-0.363	PIS51882.1	PLB3	GPI-anchored cell surface phospholipase B; possibly secreted; fungal-specific (no mammalian homolog); induced by Tbf1; fluconazole-induced; possible essential gene (UAU1 method); Spider and flow model biofilm induced
PIS58875.1	25.7318806	26.3215557	27.1094515	24.24788199	26.85395202	26.97584427	0.688872632	0.976	-0.362	PIS58875.1	NUP84	Ortholog(s) have structural constituent of nuclear pore activity
PIS52089.1	30.3934404	27.4816475	27.7640571	28.21255362	28.52407551	27.82058311	0.616243264	0.974	-0.361	PIS52089.1	KOG1	Putative TORC1 subunit; heterozygous null mutant displays sensitivity to rapamycin; likely to be essential for growth, based on an insertional mutagenesis strategy
PIS51507.1	27.8059678	27.0788925	28.0564944	27.82611804	26.48893869	27.54778447	0.747419838	0.976	-0.36	PIS51507.1	orf19.5989	Putative cleavage factor I subunit; required for the cleavage and polyadenylation of pre-mRNA 3' ends; Spider biofilm repressed
PIS55794.1	29.1911287	28.8611208	28.4726846	28.03843785	28.38226468	29.02810721	0.438652472	0.97	-0.359	PIS55794.1	BRO1	Class E vacuolar protein sorting factor; role in transport from multivesicular body to vacuole; not involved in Rim101 pathway; macrophage and pseudohyphal-repressed; flow model biofilm induced
PIS58647.1	31.2117419	31.5075329	30.4282274	30.56752541	31.05946514	30.44331974	0.318047702	0.966	-0.359	PIS58647.1	orf19.1403	Ortholog(s) have ATP:ADP antiporter activity, calcium ion binding activity and role in ADP transport, ATP transport, mitochondrial transport

PIS51815.1	25.7729903	26.3047184	26.7627533	26.20279854	26.30954671	25.25378634	0.414219158	0.97	-0.358	PIS51815.1	orf19.6818	Has domain(s) with predicted ATP binding, nucleic acid binding activity
PIS52262.1	28.6933379	27.7297711	27.974581	27.03770685	28.02420959	28.26226621	0.357707133	0.968	-0.358	PIS52262.1	RRP6	Putative nuclear exosome exonuclease component; Hap43p-induced gene; mutation confers hypersensitivity to 5-fluorocytosine (5-FC), 5-fluorouracil (5-FU), and tubercidin (7-deazaadenosine)
PIS50453.1	27.5750268	26.4773248	25.9990423	27.53383215	25.86646663	25.57913759	0.506266367	0.972	-0.357	PIS50453.1	orf19.875	Protein required for respiratory growth
PIS48552.1	29.5794141	29.5243198	29.0839898	29.11961296	29.1100141	28.89333137	0.263052311	0.963	-0.355	PIS48552.1	RPN5	Putative COP9 signalosome component; macrophage/pseudohyphal-repressed
PIS52268.1	29.5514319	29.8860729	30.2197538	29.5890125	29.42231358	29.58429436	0.248892437	0.962	-0.354	PIS52268.1	ARO4	3-deoxy-D-arabinoheptulosonate-7-phosphate synthase; aromatic amino acid biosynthesis; GCN-regulated; feedback-inhibited by tyrosine if produced in S. cerevisiae Aro3p and Aro4p catalyze same reaction; protein decreases in stationary phase
PIS50303.1	27.7874286	28.1062597	28.0087156	27.06863184	27.89490071	27.87639895	0.392637334	0.969	-0.354	PIS50303.1	CKA1	Putative alpha subunit (catalytic subunit) of protein kinase CK2; Cka1p and Cka2p have a common target with respect to fluconazole resistance; synthetically lethal with CKA2; flucytosine induced
PIS52098.1	28.2549069	26.4813415	26.3772764	26.2747545	26.25018728	27.52611048	0.693710241	0.976	-0.354	PIS52098.1	IFK2	Putative thiol-specific monooxygenase; mutant is viable; flow model biofilm induced

PIS58354.1	30.8159737	31.0583012	31.0862505	30.51685082	30.48420762	30.89818522	0.375580855	0.968	-0.354	PIS58354.1	orf19.2175	Mitochondrial apoptosis-inducing factor; induced by nitric oxide; Spider biofilm induced; rat catheter biofilm repressed
PIS58659.1	28.1950075	28.3498222	28.2678091	27.22426337	28.51579659	28.01317054	0.411983602	0.969	-0.353	PIS58659.1	DCP2	Ortholog(s) have chromatin binding, hydrolase activity, mRNA binding activity
PIS56564.1	24.5106423	24.6908713	24.7418399	24.06178728	24.21449906	24.60932218	0.29193507	0.965	-0.353	PIS56564.1	orf19.314	Ortholog of S. cerevisiae : STB6, C. glabrata CBS138 : CAGL0L05016g, C. dubliniensis CD36 : Cd36_83110, C. parapsilosis CDC317 : CPAR2_103210 and C. auris B8441 : B9J08_001101
PIS56932.1	29.3695337	28.3564861	28.5994198	27.05781269	29.43410904	28.77589898	0.618060797	0.974	-0.353	PIS56932.1	RIB5	Putative riboflavin synthase; fungal-specific (no human or murine homolog); farnesol-downregulated; protein present in exponential and stationary growth phase yeast cultures
PIS51352.1	26.732318	26.1226745	26.8105989	26.38111742	26.45848698	25.77167333	0.44827444	0.971	-0.351	PIS51352.1	ZCF13	Predicted Zn(II)2Cys6 transcription factor; similar to but not the true ortholog of S. cerevisiae Hap1; mutants display decreased colonization of mouse kidneys
PIS48693.1	25.2047365	25.4177827	25.4171356	25.74551557	24.99616185	24.25809658	0.35882243	0.968	-0.347	PIS48693.1	orf19.5352	Protein with a predicted magnesium transporter domain; mutants are viable
PIS49702.1	26.5302058	26.1752513	26.69797	26.84904306	25.68004709	25.8363255	0.428863107	0.97	-0.346	PIS49702.1	BEM2	Putative Rho1p GTPase activating protein (GAP); serum-induced transcript; Spider biofilm induced; flow model biofilm repressed

PIS50596.1	29.071609	30.0624541	30.833049	29.34978728	30.00272509	29.57820981	0.381337906	0.968	-0.345	PIS50596.1	orf19.4163	Component of a complex with Ypp1p and Stt4p that is required for phosphatidylinositol-4-phosphate, PI(4)P, in plasma membrane; required for invasive growth and cell wall organization
PIS50585.1	25.7068232	24.3310044	26.5091495	23.59113485	26.60856306	25.32274367	0.707511864	0.976	-0.342	PIS50585.1	RLP24	Putative ribosomal protein; Hap43-induced; essential gene; heterozygous mutation confers hypersensitivity to 5-fluorocytosine (5-FC), 5-fluorouracil (5-FU), and tubercidin (7-deazaadenosine); Spider biofilm induced
PIS55060.1	25.1898945	25.1600514	26.1607209	24.73811833	24.62808165	26.12415246	0.460353305	0.971	-0.34	PIS55060.1	RPC53	Ortholog(s) have RNA polymerase III activity, role in tRNA transcription by RNA polymerase III and RNA polymerase III complex localization
PIS55458.1	28.2901946	28.5708212	28.4865471	29.32842797	27.33708095	27.66084299	0.440118171	0.97	-0.34	PIS55458.1	SPT6	Putative transcription elongation factor; transposon mutation affects filamentous growth; transcript induced in an RHE model of oral candidiasis and in clinical isolates from oral candidiasis
PIS58807.1	30.2886867	31.3520375	31.287338	28.02109423	31.21187344	32.68080351	0.834529475	0.978	-0.338	PIS58807.1	GCD6	Ortholog of S. cerevisiae Gcd6; catalytic epsilon subunit of the translation initiation factor eIF2B; genes encoding translation factors are repressed by phagocytosis by murine macrophages
PIS54964.1	26.7359759	26.3195094	24.5268946	24.5263226	25.8571769	26.19053697	0.609586693	0.974	-0.336	PIS54964.1	orf19.6698	Ortholog(s) have asparagine-tRNA ligase activity, role in asparaginyl-tRNA aminoacylation, mitochondrial asparaginyl-tRNA aminoacylation and mitochondrion localization
PIS49720.1	26.1382482	26.9662418	28.0700892	27.51050345	25.61288632	27.04180957	0.618505446	0.974	-0.336	PIS49720.1	RPD31	Putative histone deacetylase; involved in regulation of white-opaque switch; Spider biofilm repressed

PIS58060.1	24.4878627	24.0128922	23.6698186	22.53846115	24.83756656	23.79381147	0.705769471	0.976	-0.334	PIS58060.1	orf19.7254	Ortholog(s) have role in mRNA metabolic process, mitochondrial translational initiation
PIS48304.1	34.6429028	34.2696334	34.5551837	34.02109275	34.17161122	34.27380852	0.211585445	0.96	-0.334	PIS48304.1	RPS21	Protein component of the small (40S) subunit; repressed upon phagocytosis by murine macrophage; positively regulated by Tbf1; Spider biofilm repressed
PIS54729.1	30.1137128	30.3551651	29.8703545	28.72735875	29.56430392	31.05235015	0.698321061	0.976	-0.332	PIS54729.1	DIM1	Putative 18S rRNA dimethylase; predicted role in rRNA modification and processing; Hap43-induced; likely to be essential for growth based on insertional mutagenesis strategy; F-12/CO2 early biofilm induced
PIS52046.1	29.1521017	28.5949026	29.1793408	28.40105243	28.6530912	28.8831089	0.604279077	0.974	-0.33	PIS52046.1	ZRT2	Zinc transporter, essential for zinc uptake and acidic conditions tolerance; transcript induced by amphotericin B, interaction with macrophages; induced in oralpharyngeal candidiasis; Spider biofilm induced
PIS51389.1	32.6074632	31.3753732	31.7706045	31.87898027	31.68095987	31.20972196	0.337536827	0.967	-0.328	PIS51389.1	MBF1	Putative transcriptional coactivator; caspofungin repressed; involved in virulence
PIS52023.1	31.9900788	32.0450443	31.3631603	31.66722706	31.40639647	31.33989002	0.387293699	0.969	-0.328	PIS52023.1	orf19.3572.3	Ribosomal 60S subunit protein L31B; Spider biofilm repressed
PIS48393.1	31.1645613	30.3682999	30.6153314	29.95168061	30.98596478	30.22891045	0.395681315	0.969	-0.327	PIS48393.1	orf19.2489	Putative karyopherin beta; repressed by nitric oxide

PIS58535.1	26.8231152	26.2709293	25.8892638	25.99520062	27.09161478	24.91404354	0.58124706	0.974	-0.327	PIS58535.1	orf19.3698	Protein of unknown function that may function in RNA processing; filament induced
PIS54680.1	33.8448373	34.1271229	33.2863531	33.40783127	33.80000851	33.06812728	0.393732114	0.969	-0.327	PIS54680.1	PIL1	Eisosome component; predicted role in endocytosis; echinocandin-binding protein; localizes to cell surface of hyphae, but not yeast-form cells; Hap43, YNB biofilm induced; rat catheter biofilm repressed
PIS59030.1	25.660132	27.3088015	26.6567616	26.49860387	26.46259417	25.68740594	0.646378725	0.975	-0.326	PIS59030.1	orf19.3661	Putative deubiquitinating enzyme; induced by Mnl1 under weak acid stress
PIS51748.1	27.9615095	27.6438241	26.9549849	27.15168979	27.86557412	26.56749613	0.445362556	0.97	-0.325	PIS51748.1	orf19.3804	Protein of unknown function; filament induced
PIS48704.1	31.297738	31.3665007	31.0553431	30.94956308	30.86257767	30.93604606	0.32089842	0.966	-0.324	PIS48704.1	TRA1	Subunit of the NuA4 histone acetyltransferase complex
PIS51466.1	31.0092152	31.0444037	30.9230651	30.07629746	31.03885237	30.89136001	0.423638013	0.97	-0.323	PIS51466.1	orf19.2002	Ortholog(s) have structural constituent of nuclear pore activity and role in nuclear pore organization, poly(A)+ mRNA export from nucleus, protein import into nucleus, ribosomal large subunit export from nucleus
PIS49667.1	27.7419974	27.027472	26.8988182	26.81405533	26.71818735	27.16896506	0.318772913	0.966	-0.322	PIS49667.1	HGT3	Putative glucose transporter of the major facilitator superfamily; the C. albicans glucose transporter family comprises 20 members; 12 probable membrane-spanning segments, extended C terminus; expressed in rich medium with 2% glucose

PIS55489.1	29.9947155	30.4958131	30.6493481	29.77717936	30.04868564	30.34729538	0.342477305	0.967	-0.322	PIS55489.1	orf19.4532	Protein of unknown function; present in exponential and stationary growth phase yeast cultures
PIS51209.1	26.3548338	24.2779616	25.5648588	24.68361223	25.81136703	24.74031899	0.601005099	0.974	-0.321	PIS51209.1	DQD1	Putative 3-dehydroquinate dehydratase; ketoconazole-repressed; protein abundance downregulated by macrophages; flow model biofilm induced
PIS58569.1	28.5603967	29.0238629	27.8862675	27.8262412	28.29378684	28.39089631	0.350498179	0.967	-0.32	PIS58569.1	LYS21	Homocitrate synthase, major isoform; lysine biosynthesis; expression increased in a fluconazole-resistant isolate; colony morphology-related gene regulation by Ssn6; ketoconazole-repressed; regulated by Gcn2, Gcn4, Hap43
PIS55717.1	28.2208217	27.9036401	28.1191032	26.94892706	28.67665912	27.65761	0.42457977	0.97	-0.32	PIS55717.1	orf19.2438	Ortholog(s) have structural constituent of ribosome activity and mitochondrial small ribosomal subunit localization
PIS51452.1	23.443575	23.9618725	23.9328575	23.86703166	23.02120087	23.49113369	0.533358721	0.973	-0.32	PIS51452.1	orf19.4097	Ortholog of C. dubliniensis CD36 : Cd36_23480, C. parapsilosis CDC317 : CPAR2_407050, C. auris B8441 : B9J08_003033 and Candida tenuis NRRL Y-1498 : CANTEDRAFT_116822
PIS55566.1	23.8295395	25.1118381	24.6526893	23.53988269	23.98447528	25.11283503	0.535983248	0.973	-0.319	PIS55566.1	orf19.4306	Ortholog(s) have methylthioribulose 1-phosphate dehydratase activity and role in L-methionine salvage from methylthioadenosine
PIS51772.1	29.4519903	28.3662348	28.2177347	28.01250607	28.76590195	28.3043447	0.415018652	0.97	-0.318	PIS51772.1	GPM2	Putative phosphoglycerate mutase; repressed in hyphae; macrophage/pseudohyphal-repressed; induced by high levels of peroxide stress, farnesol; flow model biofilm induced; rat catheter and Spider biofilm repressed

PIS56795.1	29.1652411	29.7217228	30.255734	29.31017736	29.21717994	29.66411368	0.327826332	0.966	-0.317	PIS56795.1	RVS161	Protein required for endocytosis; contains a BAR domain, which is found in proteins involved in membrane curvature; null mutant exhibits defects in hyphal growth, virulence, cell wall integrity, and actin patch localization
PIS55624.1	27.235889	27.7027384	27.8003044	26.89651041	27.36696826	27.52660142	0.645183718	0.975	-0.316	PIS55624.1	orf19.5941	Ortholog(s) have role in endoplasmic reticulum to Golgi vesicle-mediated transport and COPII-coated ER to Golgi transport vesicle, Golgi membrane, endoplasmic reticulum membrane localization
PIS51626.1	28.5258727	28.2788261	28.4481912	27.4207558	27.55469476	29.3320994	0.514227747	0.972	-0.315	PIS51626.1	orf19.1239	Secreted protein; exogenously expressed protein is a substrate for Kex2 processing in vitro; fluconazole-regulated; Spider biofilm induced
PIS54543.1	28.0299256	29.2447086	29.3183437	28.12585664	28.65278949	28.86924404	0.41657884	0.97	-0.315	PIS54543.1	orf19.3797	Ortholog(s) have structural constituent of ribosome activity and mitochondrial large ribosomal subunit localization
PIS51790.1	32.0624532	32.1427896	32.3847351	31.44724483	31.88468416	32.31175843	0.386134849	0.969	-0.315	PIS51790.1	RHO1	Small GTPase of Rho family; regulates beta-1,3-glucan synthesis activity and binds Gsc1p; essential; expected to be geranylgeranylated by geranylgeranyltransferase type I; plasma membrane-localized
PIS49740.1	26.5508212	27.5079001	29.224848	26.31157133	28.03901451	27.99027216	0.652999732	0.975	-0.314	PIS49740.1	GCD2	Putative translation initiation factor; genes encoding ribosomal subunits, translation factors, and tRNA synthetases are downregulated upon phagocytosis by murine macrophage
PIS49562.1	27.5265649	29.2470631	28.5319563	27.93180158	28.34844824	28.08544306	0.633632284	0.975	-0.313	PIS49562.1	orf19.3615	Protein of unknown function; induced in core caspofungin response; expression upregulated in an ssr1 null mutant; induced by nitric oxide independent of Yhb1p

PIS51580.1	28.8131602	28.1388972	28.8497815	28.35991665	28.50782576	27.99626945	0.417118552	0.97	-0.313	PIS51580.1	orf19.3706	Ortholog of C. dubliniensis CD36 : Cd36_33440, C. parapsilosis CDC317 : CPAR2_702080, C. auris B8441 : B9J08_003174 and Candida tenuis NRRL Y-1498 : CANTEDRAFT_105591
PIS58133.1	24.1486811	24.2306682	23.6451629	24.15107679	23.4745243	23.46050491	0.35876137	0.968	-0.313	PIS58133.1	SPC19	Essential subunit of the Dam1 (DASH) complex, which acts in chromosome segregation by coupling kinetochores to spindle microtubules
PIS55544.1	28.9371081	29.5576018	29.5308632	29.65836307	28.42002126	29.01202426	0.442846672	0.97	-0.312	PIS55544.1	orf19.2091	Putative NADH dehydrogenase; repressed by nitric oxide, Hap43p-repressed
PIS55062.1	30.0463455	30.2535531	31.1946402	30.05951931	30.2864006	30.21335906	0.368464014	0.968	-0.312	PIS55062.1	orf19.2720	Cytosolic chaperonin Cct ring complex subunit; role in the assembly of actin and tubulins; Spider biofilm repressed
PIS54928.1	19.7204751	21.469577	19.848978	19.13230944	20.8575822	20.12363126	0.763301841	0.977	-0.309	PIS54928.1	SPT10	Ortholog(s) have promoter-specific chromatin binding, sequence-specific DNA binding activity
PIS54896.1	30.4742534	30.6905182	31.9211184	30.93910045	30.76490436	30.46041024	0.481747503	0.971	-0.307	PIS54896.1	ARO1	Putative pentafunctional arom enzyme; required for aromatic amino acid biosynthesis; involved in cell wall integrity and formation of biofilm; fungal-specific (no human or murine homolog); Gcn2p-, Gcn4p-regulated
PIS54872.1	31.8034173	31.5354186	31.8598189	30.89316516	31.67587465	31.70837764	0.344330851	0.967	-0.307	PIS54872.1	MRS7	Member of the LETM1-like protein family, mitochondrial membrane protein

PIS48414.1	29.8561769	30.6355122	30.8917929	30.06398294	30.16754142	30.22975407	0.436514885	0.97	-0.307	PIS48414.1	NOC2	Putative nucleolar complex protein; Hap43-induced; transposon mutation affects filamentous growth; mutation confers hypersensitivity to 5-fluorouracil (5-FU), tubercidin (7-deazaadenosine); repressed in core stress response
PIS52393.1	25.1309663	26.2814725	26.7046999	25.38667999	24.91750027	26.89339609	0.630439492	0.975	-0.307	PIS52393.1	orf19.5425	Ortholog(s) have 3'-tRNA processing endoribonuclease activity, role in mitochondrial tRNA 3'-end processing, tRNA 3'-end processing and mitochondrion, nucleus localization
PIS54867.1	24.1315897	24.5011227	24.3208684	25.50651507	23.28228561	23.24768764	0.737484647	0.976	-0.306	PIS54867.1	TRS20	Ortholog(s) have role in endoplasmic reticulum to Golgi vesicle-mediated transport, protein-containing complex assembly and TRAPPI protein complex, TRAPPII protein complex, TRAPPIII protein complex localization
PIS49588.1	26.1252819	27.5709226	26.9164798	27.12813225	25.7162244	26.85421789	0.668529927	0.975	-0.305	PIS49588.1	EDC3	Protein with mRNA binding activity, involved in regulation of activity of putative metacaspase Mca1p
PIS56606.1	30.9686992	31.4901766	31.6244385	30.8051231	31.00747175	31.35803247	0.33705601	0.967	-0.304	PIS56606.1	HEM15	Putative ferrochelatase involved in heme biosynthesis; transcript not regulated by iron levels and not affected by a yfh1 null mutation; Spider biofilm repressed
PIS56632.1	27.7166494	28.1255038	28.6216058	27.06293903	28.0214868	28.46711348	0.635476484	0.975	-0.304	PIS56632.1	URE2	Functional homolog of S. cerevisiae Ure2p, which is a regulator of nitrogen utilization, and which also has an infectious prion form called [URE3]; forms [URE3] prion when expressed in S. cerevisiae
PIS59007.1	27.7865481	26.2320887	25.0365662	26.55586594	26.27317336	25.3172401	0.7109505	0.976	-0.303	PIS59007.1	orf19.9	Ortholog of C. dubliniensis CD36 : Cd36_32700, C. parapsilosis CDC317 : CPAR2_204280, C. auris B8441 : B9J08_000471 and Candida tenuis NRRL Y-1498 : CANTEDRAFT_113831

PIS54627.1	28.8240146	29.2214705	28.424516	28.08762028	28.93469046	28.54181238	0.554681231	0.973	-0.302	PIS54627.1	orf19.6612	Putative mitochondrial protein; Hap43p-induced gene
PIS58713.1	28.7801908	29.1028778	28.8221772	28.29824102	28.61400778	28.8876119	0.640904198	0.975	-0.302	PIS58713.1	PRP39	Putative component of the U1 snRNP; involved in splicing; Hap43-induced gene; Spider biofilm induced
PIS52364.1	27.8841464	28.2090174	27.8060221	28.46502946	27.24365317	27.28871634	0.48333415	0.971	-0.301	PIS52364.1	orf19.5809	Putative arylformamidase, enzyme of the NAD biosynthesis pathway; Gcn4p-regulated
PIS54936.1	23.6488976	28.5628842	23.2470433	25.25065295	24.64046383	24.66869545	0.797847018	0.977	-0.3	PIS54936.1	ASF1	Protein similar to S. cerevisiae Asf1p, a chromatin assembly complex component; likely to be essential for growth, based on an insertional mutagenesis strategy
PIS48726.1	29.8534103	29.9435183	29.3963057	29.35945863	29.52997616	29.40260696	0.273529418	0.964	-0.3	PIS48726.1	orf19.7152	Protein similar to Aspergillus CYSK O-acetylserine sulfhydrylase, suggesting that C. albicans uses an O-acetyl-serine (OAS) pathway of sulfur assimilation; upregulated in biofilm; protein level decreases in stationary phase cultures
PIS51089.1	26.2996683	26.2548006	25.4979102	25.90049646	26.00218653	25.2496307	0.38614501	0.969	-0.3	PIS51089.1	RAT1	5'-->3' exoribonuclease; similar to S. cerevisiae nuclear exoribonuclease Rat1p; suppresses S. cerevisiae kem1 mutant slow growth, mating defect, and haploid invasive growth defect
PIS55584.1	31.2054038	30.3092376	29.5941842	30.04891381	30.1397405	30.02324403	0.416999318	0.97	-0.299	PIS55584.1	orf19.3352	Ortholog of C. dubliniensis CD36 : Cd36_01490, C. parapsilosis CDC317 : CPAR2_109390, C. auris B8441 : B9J08_001688 and Candida tenuis NRRL Y-1498 : CANTEDRAFT_97929

PIS51394.1	28.0432151	27.3139063	26.7566437	26.45883991	27.27510686	27.48227992	0.75216931	0.977	-0.299	PIS51394.1	orf19.4398	Protein of unknown function; rat catheter biofilm induced
PIS48442.1	28.0888428	27.7096759	28.3977686	27.57572446	27.86367481	27.86101657	0.652693916	0.975	-0.299	PIS48442.1	orf19.703	Ortholog(s) have 2',3'-cyclic-nucleotide 3'-phosphodiesterase activity and role in cyclic nucleotide metabolic process
PIS49717.1	29.0102162	29.330245	29.3091216	29.48512481	28.52362291	28.74740473	0.595256988	0.974	-0.298	PIS49717.1	orf19.6804	Ortholog(s) have role in SRP-dependent cotranslational protein targeting to membrane and signal recognition particle, endoplasmic reticulum targeting localization
PIS58279.1	29.0036111	29.341629	29.5316516	29.09481604	28.87426215	29.01564195	0.491740398	0.972	-0.297	PIS58279.1	orf19.4346	Ortholog(s) have protein-membrane adaptor activity and role in COPII vesicle coating, macroautophagy, protein localization to endoplasmic reticulum exit site
PIS55611.1	24.4201363	24.8108285	25.4201343	24.45575872	24.64630366	24.6620588	0.595088963	0.974	-0.296	PIS55611.1	URK1	Protein similar to uridine kinase; repressed by ciclopirox olamine; Spider biofilm induced
PIS52321.1	31.3663164	30.5753651	31.027566	29.03994163	31.09903887	31.9441589	0.646430976	0.975	-0.295	PIS52321.1	MRPS9	Mitochondrial ribosomal protein S9; has N-terminal mitochondrial targeting signal and an S9 consensus motif; overexpression in S. cerevisiae causes respiratory defect that is reversible upon cessation of C. albicans gene expression
PIS58448.1	28.734358	28.6094982	28.7275913	28.76007607	27.99159225	28.43340873	0.445850508	0.97	-0.295	PIS58448.1	orf19.3681	Ortholog(s) have guanyl-nucleotide exchange factor activity, nuclear import signal receptor activity, protein-containing complex binding activity

PIS54566.1	31.2903197	31.3630659	31.5706217	31.02501372	31.15753862	31.17414696	0.265487249	0.963	-0.289	PIS54566.1	NDE1	Putative NADH dehydrogenase; may act alternatively to complex I in respiration; caspofungin repressed; rat catheter biofilm induced; Spider biofilm repressed
PIS52306.1	24.6303949	25.5591753	27.5106065	24.61822921	25.49072952	26.72646868	0.722417636	0.976	-0.288	PIS52306.1	PKH2	Probable serine/threonine protein kinase; predicted role in sphingolipid-mediated signaling pathway that controls endocytosis; mRNA binds She3 and is localized to hyphal tips; appears redundant with Pkh3 within the Ypk1 signaling pathway
PIS51485.1	23.4208613	23.4488169	23.485033	22.41562494	23.04991174	24.02982296	0.809141269	0.977	-0.286	PIS51485.1	orf19.3535	Ortholog(s) have cellular bud neck, fungal-type vacuole localization
PIS59002.1	25.3543186	25.2933115	26.835943	25.77526422	25.69463583	25.15853483	0.650156975	0.975	-0.285	PIS59002.1	ARO2	Putative chorismate synthase; fungal-specific (no human or murine homolog); protein level decreased in stationary phase yeast cultures; GlcNAc-induced protein
PIS50472.1	28.8321571	28.7634874	29.6859933	29.32849457	28.70799935	28.39102456	0.414081321	0.97	-0.285	PIS50472.1	MES1	Cytoplasmic methionyl-tRNA synthetase; zinc-binding motif; ribosomal subunits, translation factors, tRNA synthetases are downregulated upon phagocytosis by murine macrophage; protein present in exponential and stationary phase yeast
PIS49648.1	29.1536841	28.0275298	28.0755303	28.0701215	28.7932063	27.54447103	0.576938586	0.974	-0.283	PIS49648.1	orf19.1860.1	Has domain(s) with predicted peptidase activity and role in proteolysis
PIS56729.1	24.1840422	25.5165171	26.2036726	24.04254176	25.69263007	25.31971283	0.634362642	0.975	-0.283	PIS56729.1	orf19.6103	Ortholog(s) have role in ascospore formation, ascospore wall assembly, ascospore-type prospore membrane formation and cytosol, prospore membrane localization

PIS58396.1	26.2585585	25.3699974	26.3364958	27.07675125	24.00422058	26.03425142	0.76432638	0.977	-0.283	PIS58396.1	orf19.7107	Ortholog(s) have role in ribosomal large subunit biogenesis and cytoplasm, nucleus localization
PIS50475.1	32.3996788	33.0615339	32.5616571	30.57912289	32.85692585	33.73882553	0.647753207	0.975	-0.283	PIS50475.1	SSD1	Protein with role in resistance to host antimicrobial peptides; virulence role in murine infection; functional homolog of S. cerevisiae Ssd1p, which suppresses various mutant phenotypes; constitutively expressed and not cell-cycle regulated
PIS50407.1	26.958849	26.8969599	25.8625225	27.01170664	26.34788057	25.51258646	0.590792843	0.974	-0.282	PIS50407.1	ALG9	Putative mannosyltransferase; similar to S. cerevisiae Alg9p; has HKEXRF motif
PIS52136.1	32.3079744	32.5767837	32.8475166	32.07332573	32.48177192	32.33298895	0.363538512	0.968	-0.281	PIS52136.1	ATP20	Putative mitochondrial ATP synthase; shows colony morphology-related gene regulation by Ssn6p; flucytosine induced; caspofungin repressed; macrophage/pseudohyphal-induced
PIS49747.1	24.889065	24.0949305	24.8181777	23.56697842	24.97827219	24.41488372	0.560621368	0.973	-0.281	PIS49747.1	GLY1	L-threonine aldolase; complements glycine auxotrophy of S. cerevisiae shm1 shm2 gly1-1 triple mutant; macrophage/pseudohyphal-induced; the GLY1 locus has an RFLP and is triploid in strain SGY269; flow model biofilm induced
PIS56808.1	24.7282841	24.0401199	23.8256011	24.27714468	22.3553334	25.12061848	0.803907003	0.977	-0.28	PIS56808.1	orf19.2314	Protein with a role in nucleolar integrity and processing of pre-rRNA; mutation confers hypersensitivity to 5-fluorocytosine (5-FC), 5-fluorouracil (5-FU), and tubercidin (7-deazaadenosine); Hap43-induced; Spider biofilm induced
PIS51338.1	28.5757933	29.682271	30.4559756	27.97666846	29.4770915	30.42045061	0.681944378	0.975	-0.28	PIS51338.1	orf19.4721	Ortholog(s) have RNA binding activity, role in mRNA processing, mitochondrial translation and mitochondrion localization

PIS48691.1	25.0510852	24.264281	26.5479713	24.90935133	24.74327844	25.37601286	0.746068671	0.976	-0.278	PIS48691.1	orf19.4820	Ortholog of S. cerevisiae : YKL162C, C. dubliniensis CD36 : Cd36_09010, C. parapsilosis CDC317 : CPAR2_214170, C. auris B8441 : B9J08_005393 and Candida tenuis NRRL Y-1498 : CANTEDRAFT_95066
PIS52360.1	28.4587368	26.7879444	26.1116212	26.97428044	26.84472401	26.70874172	0.669670757	0.975	-0.277	PIS52360.1	orf19.4017	Ortholog of C. dubliniensis CD36 : Cd36_54840, C. parapsilosis CDC317 : CPAR2_100270, C. auris B8441 : B9J08_003974 and Candida tenuis NRRL Y-1498 : cten_CGOB_00242
PIS49684.1	30.2307666	30.2276388	30.0577041	30.09121061	29.81023937	29.79078438	0.320570484	0.966	-0.275	PIS49684.1	NCP1	NADPH-cytochrome P450 reductase, acts with Erg11p in sterol 14 alpha-demethylation in ergosterol biosynthesis; subject to hypoxic regulation; ketoconazole-induced; caspofungin repressed
PIS49613.1	28.6608392	29.0854672	28.3779197	27.86659656	28.63731	28.79591805	0.478251907	0.971	-0.275	PIS49613.1	orf19.4970	Protein of unknown function; Spider biofilm induced
PIS51357.1	27.23579	27.4843421	27.8861094	28.08286646	26.6341846	27.06382872	0.57350008	0.974	-0.275	PIS51357.1	RMS1	Putative lysine methyltransferase; Hap43-induced; protein induced during mating; possibly essential, disruptants not obtained by UAU1 method; rat catheter and Spider biofilm induced
PIS55734.1	29.4679782	28.9653402	29.2535836	29.16868679	28.82327924	28.87087778	0.648884552	0.975	-0.275	PIS55734.1	SEC22	Ortholog(s) have SNAP receptor activity and role in endoplasmic reticulum to Golgi vesicle-mediated transport, retrograde vesicle-mediated transport, Golgi to endoplasmic reticulum, vesicle fusion, vesicle fusion with Golgi apparatus
PIS51340.1	32.380413	32.2826651	32.1148771	31.86252908	31.99029258	32.10401445	0.336914606	0.967	-0.274	PIS51340.1	MTS1	Sphingolipid C9-methyltransferase; catalyzes methylation of the 9th carbon in the long chain base component of glucosylceramides; glucosylceramide biosynthesis is important for virulence; Spider biofilm repressed

PIS51111.1	27.2984424	27.2897377	27.4675632	27.37683582	26.52205634	27.34059508	0.482408841	0.971	-0.272	PIS51111.1	orf19.7522	Protein with a pyridoxal phosphate-dependent transferase domain; Hap43-repressed; mutation confers hypersensitivity to amphotericin B; Spider biofilm repressed
PIS54624.1	29.3428117	29.7495009	29.7539004	28.81053487	29.54734712	29.67315641	0.404663484	0.969	-0.272	PIS54624.1	SSO2	Plasma membrane t-SNARE; involved in fusion of secretory vesicles at the plasma membrane
PIS51025.1	29.2312915	29.8849718	28.9184401	29.39531267	29.02854558	28.79790594	0.519318425	0.972	-0.271	PIS51025.1	orf19.2964	Component of the RSC chromatin remodeling complex
PIS52352.1	25.5096547	25.6595226	25.1680401	24.79495233	24.97850388	25.75514967	0.519438023	0.972	-0.27	PIS52352.1	orf19.4959	Transcriptional activator recruited by the scaffold protein Rep1p when galactose is present to activate galactose-inducible genes; null mutant shows severe growth defect on galactose; Spider biofilm repressed
PIS54976.1	29.9620213	30.0527256	31.435984	30.17128448	30.01381196	30.45693327	0.719423629	0.976	-0.27	PIS54976.1	orf19.7393	Predicted NAD+/NADH kinase; possible role in cellular iron ion homeostasis; Spider biofilm induced
PIS55819.1	28.8490627	27.7222987	28.1098634	27.2451506	28.49522518	28.13325909	0.509897305	0.972	-0.269	PIS55819.1	ALG11	Alpha-1,2-mannosyltransferase; catalyzes sequential addition of 2 terminal alpha 1,2-mannose residues to the Man5GlcNAc2-PP-dolichol intermediate during asparagine-linked glycosylation in the ER; Spider biofilm induced
PIS55649.1	26.37803	26.8655635	26.7827998	26.46934253	26.47718198	26.27287914	0.425274198	0.97	-0.269	PIS55649.1	orf19.6730	Ortholog(s) have RNA binding activity, role in maturation of LSU-rRNA, ribosomal large subunit biogenesis and nucleolus localization

PIS54609.1	25.9530952	25.6536405	24.90438	25.51522763	26.40646925	23.78092618	0.675804949	0.975	-0.269	PIS54609.1	orf19.7441	Ortholog(s) have chromatin binding activity, role in lipid homeostasis, nuclear envelope organization, regulation of transcription by RNA polymerase II and nuclear envelope localization
PIS55712.1	30.2681447	30.418782	30.2034097	30.19120233	29.82230714	30.06861866	0.347448744	0.967	-0.269	PIS55712.1	RGD1	GTPase activator protein; transcript induced in low iron; alkaline upregulated; localized to the bud emergence area in G1 phase and in the bud tip during S and G2 phases
PIS58496.1	32.517677	32.8316478	32.7121663	32.30852229	32.65820864	32.28706292	0.336718729	0.967	-0.269	PIS58496.1	TIF	Translation initiation factor; upregulated in highly virulent strain compared to less virulent strain; antigenic in human; flucytosine induced; downregulated upon phagocytosis by macrophages; Spider biofilm repressed
PIS50322.1	26.1820763	26.1660007	25.2228287	24.75794654	25.76976278	26.24224991	0.530153659	0.973	-0.267	PIS50322.1	SEC2	Guanyl-nucleotide exchange factor for the small G-protein Sec4; delivery of post-Golgi secretory vesicles to sites of polarized growth; phosphorylation by Cdc28 needed for normal hyphal growth; Hap43-repressed; flow model biofilm induced
PIS58274.1	22.8560645	22.9198751	22.2697731	25.47512068	23.80621118	17.96501748	0.86379687	0.978	-0.266	PIS58274.1	orf19.4657	Ortholog(s) have phosphoprotein phosphatase activity
PIS58601.1	27.5697272	27.3535835	27.3924332	27.40258628	27.10240434	27.01283096	0.641517829	0.975	-0.266	PIS58601.1	orf19.7426	Ortholog(s) have protein-disulfide reductase activity, role in protein N-linked glycosylation, protein-containing complex assembly and oligosaccharyltransferase complex localization
PIS56645.1	27.3716095	27.1059708	27.5822105	26.87556205	27.42433936	26.96588991	0.429889562	0.97	-0.265	PIS56645.1	orf19.1536	Putative zinc transporter essential for tolerance to zinc; plays a role in zincosome formation; Hap43-induced; required for normal filamentous growth; mRNA binds She3 and is localized to hyphal tips

PIS55623.1	28.7094521	29.2397893	29.5232514	28.7473687	28.85827808	29.07088139	0.490509921	0.972	-0.265	PIS55623.1	orf19.5943	Ortholog(s) have role in peroxisome organization and peroxisomal membrane localization
PIS54615.1	28.3413324	27.6825448	27.4166096	28.27854978	27.4586576	26.91121052	0.537037393	0.973	-0.264	PIS54615.1	PMI1	Phosphomannose isomerase; cell wall biosynthesis enzyme; drug target; functional homolog of S. cerevisiae, E. coli phosphomannose isomerase; Gcn4-regulated; induced on adherence to polystyrene, phagocytosis; 3-AT, Spider biofilm repressed
PIS55746.1	33.1691691	32.6434716	32.7489024	32.89620375	32.47583432	32.39871678	0.445336062	0.97	-0.264	PIS55746.1	RPL15A	Putative ribosomal protein; repressed upon phagocytosis by murine macrophage; positively regulated by Tbf1; Spider biofilm repressed
PIS54489.1	27.3340472	27.1833491	26.4105521	28.09199415	26.59931881	25.44692098	0.676555869	0.975	-0.263	PIS54489.1	KRE6	Essential beta-1,6-glucan synthase subunit; change in mRNA length, not abundance, at yeast-hypha transition; alkaline induced by Rim101, on cell wall regeneration; Spider biofilm induced; Bcr1-repressed in RPMI a/a biofilms
PIS56591.1	26.5818261	27.3907262	27.2762537	26.90342908	27.15220417	26.40821724	0.642472686	0.975	-0.262	PIS56591.1	orf19.2214	Ortholog(s) have structural constituent of ribosome activity and mitochondrial large ribosomal subunit localization
PIS58921.1	25.0131082	24.4573892	24.8738995	23.69336722	24.43816456	25.43098765	0.678445134	0.975	-0.261	PIS58921.1	DOT6	Protein with a predicted role in telomeric gene silencing and filamentation; repressed by high-level peroxide stress; Spider biofilm induced
PIS51356.1	27.6137858	26.6773083	26.5794373	24.93616604	27.30685661	27.84433511	0.693334858	0.976	-0.261	PIS51356.1	orf19.2650.1	Mitochondrial ribosomal protein of the small subunit; Spider biofilm repressed

PIS48359.1	28.4455269	29.5824837	30.1862348	27.55208954	29.57317437	30.3085992	0.777408274	0.977	-0.26	PIS48359.1	APL5	Ortholog of S. cerevisiae and S. pombe Apl5; subunit of the AP-3 adaptor complex involved in Golgi-to-vacuole transport; phosphorylated protein; mutant is viable;
PIS54674.1	30.6321481	30.5389105	31.4842163	30.08525966	30.7314486	31.05937994	0.457580069	0.971	-0.26	PIS54674.1	BAT21	Putative branched chain amino acid aminotransferase; regulated by Gcn4, Gcn2; induced in response to amino acid starvation (3-aminotriazole treatment); early-stage flow model biofilm formation
PIS51897.1	30.4913498	31.2522406	31.5802876	31.02866083	30.65962661	30.85689751	0.444210503	0.97	-0.26	PIS51897.1	PIN4	Protein with similarity to S. cerevisiae Pin4p; transposon mutation affects filamentous growth
PIS49848.1	23.2354162	23.1876069	23.5985549	22.13479041	23.56859451	23.54018471	0.721724129	0.976	-0.259	PIS49848.1	orf19.1259	Ortholog(s) have ubiquitin protein ligase activity, role in histone catabolic process, regulation of transcription by RNA polymerase II and Lid2 complex localization
PIS48251.1	29.7814652	29.6023278	29.4756623	29.63134326	29.12133225	29.33306712	0.694999595	0.976	-0.258	PIS48251.1	CRN1	Coronin; cortical actin cytoskeletal component; predicted role in regulation of actin patch assembly; rat catheter and Spider biofilm repressed
PIS50427.1	24.8975491	24.9346969	24.4832607	24.53812098	24.59121327	24.41217912	0.586651847	0.974	-0.258	PIS50427.1	orf19.3242	Putative TFIID and SAGA complex subunit; possibly an essential gene, disruptants not obtained by UAU1 method
PIS58949.1	27.3587322	27.3878207	27.3050205	26.25029714	28.16508637	26.86565175	0.62248507	0.974	-0.257	PIS58949.1	KRE5	UDP-glucose:glycoprotein glucosyltransferase; 1,6-beta-D-glucan biosynthesis, hyphal growth, virulence in mouse IV model; partially complements S. cerevisiae kre5 mutant defects; flow biofilm repressed, Bcr1-repressed in RPMI a/a biofilms

PIS58284.1	31.4924169	30.9254511	31.2791651	31.21495535	31.17528775	30.53722735	0.4232638	0.97	-0.257	PIS58284.1	SEC62	Putative endoplasmic reticulum (ER) protein-translocation complex subunit
PIS48377.1	24.2851806	25.7359045	24.8161338	24.85473439	24.82897353	24.38552096	0.523506052	0.972	-0.256	PIS48377.1	orf19.3633	Has domain(s) with predicted hydroxyisourate hydrolase activity and role in purine nucleobase metabolic process
PIS52178.1	31.5499399	31.2911347	31.3512471	30.74360508	31.10924678	31.57093614	0.533955652	0.973	-0.256	PIS52178.1	SHM1	Mitochondrial serine hydroxymethyltransferase; complements the glycine auxotrophy of an S. cerevisiae shm1 null shm2 null gly1-1 triple mutant; protein present in exponential and stationary growth phase yeast cultures
PIS56820.1	29.1669789	29.1239734	29.7136968	29.21828121	29.2759044	28.74597171	0.382585587	0.968	-0.255	PIS56820.1	ERO1	Ortholog of S. cerevisiae Ero1; role in formation of disulfide bonds in the endoplasmic reticulum; fluconazole-induced; induced by Mnl1 under weak acid stress; Spider biofilm induced
PIS55812.1	25.8733954	26.8612964	27.0802198	25.56847716	26.57052756	26.91245471	0.568114599	0.973	-0.254	PIS55812.1	AHR1	Zn(II)2Cys6 transcription factor; involved in regulation of adhesion genes; involved in white-opaque switch; acts as repressor of START; forms complex with Mcm1; mutant is sensitive to 5-fluorocytosine and lithium chloride
PIS54705.1	27.4330405	27.7512324	27.9650592	28.01880211	26.91278118	27.45697307	0.636247329	0.975	-0.254	PIS54705.1	ERF1	Putative translation release factor 1, which interacts with stop codons and promotes release of nascent peptides from ribosomes; Hap43p-induced gene
PIS51950.1	26.2370403	25.9145436	23.798114	25.11586915	25.35392514	24.7197172	0.702292162	0.976	-0.253	PIS51950.1	DJP1	Similar to bacterial DnaJ; reported to have a role in peroxisome biogenesis; induced in low iron and upon adherence to polystyrene

PIS52330.1	31.8585762	32.1763203	32.1206359	32.1408166	31.59632784	31.66231276	0.357193639	0.968	-0.252	PIS52330.1	RPG1A	Putative translation initiation factor; mutation confers hypersensitivity to roridin A and verrucarin A; repressed upon phagocytosis by murine macrophage; Spider biofilm repressed
PIS56870.1	28.8042963	28.3757343	29.7520609	28.98624144	28.83340025	28.35949796	0.667920863	0.975	-0.251	PIS56870.1	ABP1	Ortholog of S. cerevisiae Abp1; actin-binding protein of the cortical actin cytoskeleton; caspofungin induced; protein only detected in stationary phase yeast-form cultures; Spider biofilm repressed
PIS51028.1	24.4035779	24.0827351	24.0711661	24.10178631	23.8492919	23.85268203	0.429791209	0.97	-0.251	PIS51028.1	orf19.2939	Ortholog(s) have role in mitochondrial cytochrome c oxidase assembly, regulation of proline metabolic process and mitochondrial inner membrane, mitochondrion localization
PIS58538.1	28.0314878	27.6627359	27.3428364	27.72107775	27.48478289	27.07809604	0.68917358	0.976	-0.251	PIS58538.1	orf19.3697	Ortholog(s) have role in mitotic intra-S DNA damage checkpoint signaling, positive regulation of macroautophagy, re-entry into mitotic cell cycle after pheromone arrest and Golgi trans cisterna, endoplasmic reticulum localization
PIS51253.1	26.5102282	27.2827965	26.7980648	26.12152304	26.81007271	26.90779086	0.698231289	0.976	-0.251	PIS51253.1	orf19.81	Ortholog(s) have role in exonucleolytic trimming to generate mature 3'-end of 5.8S rRNA from tricistronic rRNA transcript (SSU-rRNA, 5.8S rRNA and LSU-rRNA), more
PIS58654.1	25.1522601	25.9242682	27.3650985	25.27205158	25.90144249	26.52183022	0.719910525	0.976	-0.249	PIS58654.1	PTP1	Phosphotyrosine-specific protein phosphatase; rat catheter biofilm induced
PIS51228.1	27.7561624	28.4120052	27.5136536	27.14208615	27.75383975	28.04198553	0.534600402	0.973	-0.248	PIS51228.1	orf19.2259	Ortholog(s) have structural constituent of ribosome activity and mitochondrial large ribosomal subunit localization

PIS50531.1	24.4433747	25.375304	25.8798581	24.59048295	25.13604823	25.23019928	0.732286046	0.976	-0.247	PIS50531.1	orf19.3922	Possible pyrimidine 5' nucleotidase; protein present in exponential and stationary growth phase yeast cultures; Hap43p-repressed gene
PIS48441.1	26.5621151	26.1405573	27.4033448	26.89835741	26.37093456	26.09696369	0.638701361	0.975	-0.247	PIS48441.1	SOL3	Putative 6-phosphogluconolactonase; present in exponential and stationary growth phase yeast cultures; macrophage-downregulated protein; Spider biofilm repressed
PIS55048.1	28.0489958	28.8652527	31.0526761	28.91469398	28.51350531	29.80002634	0.733269077	0.976	-0.246	PIS55048.1	orf19.2070	Component of the RSC chromatin remodeling complex
PIS50576.1	28.1503536	26.8654884	27.168484	28.74892192	26.58996752	26.10628833	0.745471964	0.976	-0.246	PIS50576.1	orf19.4264	Protein of unknown function; induced during chlamydospore formation in both C. albicans and C. dubliniensis; flow model biofilm induced
PIS48315.1	29.6426529	30.0110296	30.9222988	29.81476472	29.93604638	30.09539511	0.551818866	0.973	-0.243	PIS48315.1	MRPL10	Putative mitochondrial large subunit ribosomal protein; colony morphology-related gene regulation by Ssn6
PIS55714.1	28.3379745	28.1773387	27.1188449	26.26410066	28.16199249	28.47805152	0.649237765	0.975	-0.243	PIS55714.1	RTT101	Putative cullin subunit of E3 ubiquitin ligase complex, involved in response to DNA damage; induced by alpha pheromone in SpiderM medium
PIS54922.1	27.321904	25.1648266	25.9518853	26.32859391	25.20590312	26.17777304	0.69149571	0.976	-0.242	PIS54922.1	orf19.1852	Ortholog(s) have mitochondrion localization

PIS55737.1	28.6760479	28.699687	28.5571737	28.78735646	28.71212266	27.71029612	0.503986171	0.972	-0.241	PIS55737.1	RPT4	26S proteasome regulatory subunit; regulated by Gcn2p and Gcn4p; protein level decreases in stationary phase cultures
PIS52109.1	29.2331459	29.0316848	28.8301451	28.79627903	28.87289834	28.71886936	0.430309651	0.97	-0.236	PIS52109.1	orf19.4502	Ortholog(s) have ATPase, DNA binding, TBP-class protein binding, molybdate ion transmembrane transporter activity
PIS58282.1	28.5285215	28.125149	27.8370482	26.9985601	28.21471454	28.57181026	0.602864384	0.974	-0.235	PIS58282.1	EHT1	Putative acyl-coenzymeA:ethanol O-acyltransferase; regulated by Sef1, Sfu1, and Hap43; induced by alpha pheromone in SpiderM medium; Spider biofilm induced; promoter bound by Ndt80
PIS48277.1	24.3961002	28.620807	28.8444639	27.17422028	26.7859411	27.19777864	0.835844444	0.978	-0.234	PIS48277.1	FGR22	Putative phosphatidylinositol-specific phospholipase C (PI-PLC); predicted type 2 membrane protein; no S. cerevisiae ortholog; role in, and regulated by, filamentation, Hap43p; almost identical to orf19.5797
PIS50412.1	28.8424796	28.5593356	28.0321811	27.98231982	28.43318106	28.3200496	0.456874323	0.971	-0.233	PIS50412.1	DRG1	Member of the DRG family of GTP-binding proteins; involved in regulation of invasive filamentous growth
PIS51779.1	23.3104847	24.2795333	24.9802232	24.75907378	24.10930751	23.00158511	0.72900579	0.976	-0.233	PIS51779.1	orf19.6850	Putative transcription factor with C3HC4 zinc finger DNA-binding motif; mutants are viable
PIS58144.1	32.2419377	32.1820852	32.3845142	31.84550146	32.34325354	31.92020453	0.382936754	0.968	-0.233	PIS58144.1	UBI3	Fusion of ubiquitin with the S34 protein of the small ribosomal subunit; mRNA decreases upon heat shock, appears to be degraded; functional homolog of S. cerevisiae RPS31; Hap43-induced; Spider biofilm repressed

PIS48455.1	23.7093302	25.4752836	25.8849763	24.64309987	24.86372535	24.86532888	0.716435953	0.976	-0.232	PIS48455.1	FMP45	Predicted membrane protein induced during mating; mutation confers hypersensitivity to toxic ergosterol analog, to amphotericin B; alkaline repressed; repressed by alpha pheromone in SpiderM medium; rat catheter, Spider biofilm induced
PIS48808.1	24.5251908	24.5970677	26.2424008	25.07601193	24.44226938	25.15056915	0.754587116	0.977	-0.232	PIS48808.1	VAM3	Predicted syntaxin-like vacuolar t-SNARE, involved in vacuolar inheritance
PIS51703.1	29.9253274	29.8838566	29.6345494	29.72459719	29.70913444	29.31971368	0.437629368	0.97	-0.23	PIS51703.1	ALI1	Putative NADH-ubiquinone oxidoreductase; in detergent-resistant membrane fraction (possible lipid raft component); predicted N-terminal acetylation; nitric oxide-repressed; plasma membrane-localized; protein decreases in stationary phase
PIS51448.1	24.7063411	24.4677183	22.5297723	24.18536857	23.06075616	23.7691263	0.808554033	0.977	-0.23	PIS51448.1	MED18	RNA polymerase II mediator complex subunit; rat catheter biofilm repressed
PIS58046.1	23.0615957	24.0916103	23.9578936	23.57202192	23.30644713	23.54691749	0.579886765	0.974	-0.229	PIS58046.1	orf19.3898	Ortholog(s) have SNAP receptor activity, role in endocytosis, vesicle fusion and SNARE complex, endosome, trans-Golgi network localization
PIS51475.1	29.0443962	28.8777304	28.6353994	28.74719418	28.5806343	28.54448613	0.471765861	0.971	-0.228	PIS51475.1	orf19.7234	Component of the RSC chromatin remodeling complex; possibly an essential gene, disruptants not obtained by UAU1 method
PIS51122.1	27.6477622	28.2039047	28.5534589	27.78760704	27.74565807	28.19323243	0.570945057	0.973	-0.226	PIS51122.1	orf19.7330	Protein with a predicted heme oxygenase domain; Spider biofilm induced

PIS55000.1	25.2404586	23.9699913	25.3748108	24.05544955	24.77618392	25.07438776	0.665870891	0.975	-0.226	PIS55000.1	SLN1	Histidine kinase involved in a two-component signaling pathway that regulates cell wall biosynthesis; mutants are sensitive to growth on H2O2 medium; rat catheter and Spider biofilm induced
PIS52172.1	32.122449	32.6359804	32.7302289	32.13180563	32.53386063	32.14990061	0.472895955	0.971	-0.224	PIS52172.1	PDA1	Putative pyruvate dehydrogenase alpha chain; fluconazole-induced; protein present in exponential and stationary growth phase yeast cultures; Hap43-induced; Spider biofilm repressed
PIS48487.1	29.5754482	29.9826825	30.0117237	29.70045887	29.45165946	29.74960892	0.451018872	0.971	-0.223	PIS48487.1	MRS4	Mitochondrial carrier family member, involved in iron homeostasis; putative membrane transporter localized to the mitochondrial membrane; transcription under control of Atf2p; regulated by Sef1p, Sfu1p, and Hap43p
PIS48776.1	29.0237667	28.948743	29.0829946	28.81008414	28.5222328	29.05475947	0.446903277	0.97	-0.223	PIS48776.1	orf19.518	Ortholog(s) have tRNA (cytidine-5-)-methyltransferase activity, tRNA binding activity, role in tRNA methylation, tRNA wobble base cytosine methylation and nucleus localization
PIS51288.1	29.5331701	28.728498	28.5338416	28.79565896	29.0215965	28.30934879	0.506494551	0.972	-0.223	PIS51288.1	orf19.5393	Putative cysteine sulfinate decarboxylase; transcript positively regulated by Tbf1; Spider biofilm induced
PIS51811.1	25.5369475	23.9754424	25.7937028	25.14329781	24.07985563	25.41542396	0.726757473	0.976	-0.223	PIS51811.1	orf19.6822	Ortholog(s) have cullin family protein binding, protein-macromolecule adaptor activity, ubiquitin conjugating enzyme binding, ubiquitin-like protein binding activity and role in protein neddylation
PIS58428.1	30.8263791	30.8684848	31.0952754	30.44487171	30.93522921	30.7418696	0.394531136	0.969	-0.223	PIS58428.1	SKI3	Ortholog(s) have role in nuclear-transcribed mRNA catabolic process, 3'-5' exonucleolytic nonsense-mediated decay, nuclear-transcribed mRNA catabolic process, exonucleolytic and 3'-5', more

PIS49477.1	31.1146246	31.1871026	31.317639	31.16697089	30.93191376	30.85316792	0.423792207	0.97	-0.222	PIS49477.1	ERG13	3-hydroxy-3-methylglutaryl coenzyme A synthase; ergosterol biosynthesis; sumoylation target; Tn mutation affects filamentation; amphotericin B, caspofungin repressed; exponential, stationary growth phase expressed; Spider biofilm repressed
PIS51079.1	24.5873878	26.5212834	25.540132	24.9810715	25.21561731	25.78552083	0.724612451	0.976	-0.222	PIS51079.1	orf19.2381	Protein of unknown function; possibly an essential gene, disruptants not obtained by UAU1 method
PIS56635.1	25.0968262	24.231042	23.9246813	24.56034557	23.96417818	24.06423376	0.581164404	0.974	-0.221	PIS56635.1	HST2	Putative histone deacetylase; role in regulation of white-opaque switch; Spider biofilm induced
PIS51247.1	27.7159274	28.1137359	28.6284477	28.16409006	27.41607692	28.21521462	0.535290257	0.973	-0.221	PIS51247.1	orf19.2008	Ortholog(s) have S-methyl-5-thioribose-1-phosphate isomerase activity and role in L-methionine salvage from methylthioadenosine
PIS52454.1	25.3674166	26.3098639	25.4363867	25.8926134	25.68615978	24.87150361	0.770831606	0.977	-0.221	PIS52454.1	orf19.4341	Ortholog(s) have role in attachment of GPI anchor to protein and GPI-anchor transamidase complex localization
PIS51642.1	27.2536472	26.7847289	27.3172255	26.97735408	27.1040585	26.61459868	0.855629726	0.978	-0.22	PIS51642.1	APC1	Putative Anaphase-Promoting Complex/Cyclosome subunit; essential for growth; periodic mRNA expression, peak at cell-cycle S/G2 phase
PIS54553.1	25.8672905	26.8300629	24.9389931	26.98597521	25.58090081	24.41461668	0.722620205	0.976	-0.218	PIS54553.1	orf19.2966	Predicted dienelactone hydrolase domain; clade-associated gene expression; farnesol-downregulated; rat catheter biofilm repressed

PIS49494.1	26.267943	26.808384	27.4048193	26.89884695	26.63157436	26.2962958	0.510504544	0.972	-0.218	PIS49494.1	orf19.423	Ortholog(s) have enzyme activator activity, mRNA binding activity, role in deadenylation-dependent decapping of nuclear-transcribed mRNA and P-body, RNA decapping complex, cytoplasm, cytoplasmic side of membrane, nucleus localization
PIS56798.1	26.6205427	27.9368057	28.6095365	27.24632605	27.36807289	27.89913276	0.689739094	0.976	-0.218	PIS56798.1	RAD3	Ortholog of S. cerevisiae Rad3; 5' to 3' DNA helicase, nucleotide excision repair and transcription, subunit of RNA polII initiation factor TFIIH and Nucleotide Excision Repair Factor 3 (NEF3)
PIS54583.1	26.4308488	27.112412	28.7015783	26.08856343	27.39580864	28.10989766	0.78402	0.977	-0.217	PIS54583.1	orf19.158	Ortholog of S. cerevisiae Apd1; required for normal localization of actin patches and normal tolerance of sodium ions and hydrogen peroxide; Hap43-induced; Spider biofilm induced
PIS58919.1	27.0642322	27.6022671	27.7587535	27.72341	27.16034256	26.89287049	0.582128914	0.974	-0.216	PIS58919.1	orf19.649	Ortholog of C. dubliniensis CD36 : Cd36_30530, C. parapsilosis CDC317 : CPAR2_203340, C. auris B8441 : B9J08_000376 and Candida tenuis NRRL Y-1498 : CANTEDRAFT_117008
PIS54608.1	22.0730246	26.2908372	28.1117819	25.50047369	24.83646498	25.49198889	0.875456192	0.978	-0.216	PIS54608.1	orf19.7442	Has domain(s) with predicted mismatch base pair DNA N-glycosylase activity and role in base-excision repair, AP site formation
PIS52409.1	23.1137502	23.4320669	23.5861478	23.08210388	22.58925813	23.81888698	0.696307133	0.976	-0.214	PIS52409.1	orf19.4882	TFIIE small subunit; involved in RNA polymerase II transcription initiation; Spider biofilm induced
PIS49577.1	29.3657926	29.0220163	29.2298968	29.89400018	28.63040748	28.45371773	0.5673564	0.973	-0.213	PIS49577.1	RNR1	Ribonucleotide reductase large subunit; induced in low iron; transposon mutation affects filamentous growth; farnesol upregulated in biofilm; regulated by cell cycle, tyrosol, cell density; regulated by Sef1, Sfu1, and Hap43

PIS55073.1	31.1691881	31.0576939	31.0278949	31.08979352	30.76227344	30.7645723	0.426158855	0.97	-0.213	PIS55073.1	YPT31	Protein required for resistance to toxic ergosterol analog
PIS56897.1	28.3363884	27.8371553	28.1066223	28.01166142	27.63940358	27.99366122	0.511297888	0.972	-0.212	PIS56897.1	MHP1	Protein similar to S. cerevisiae Mhp1p, which is involved in microtubule stabilization; transposon mutation affects filamentous growth; possibly transcriptionally regulated upon hyphal formation; possibly an essential gene (by UAU1 method)
PIS55553.1	25.3960898	25.0715194	24.5508488	24.85995655	24.90299079	24.62901198	0.587364918	0.974	-0.209	PIS55553.1	orf19.7344	Ortholog(s) have DNA binding, chromatin binding, histone deacetylase activity and role in chromosome segregation, negative regulation of transcription by RNA polymerase II, regulatory ncRNA-mediated gene silencing
PIS48534.1	34.4629184	34.3462292	33.9455661	33.78395269	34.27009233	34.08160829	0.447915029	0.971	-0.206	PIS48534.1	TAL1	Transaldolase; protein present in exponential and stationary growth phase yeast cultures; oxidative stress-induced via Cap1; induced by nitric oxide independent of Yhb1p; sumoylation target; rat catheter biofilm repressed
PIS51630.1	27.8882291	27.7517476	27.6105304	27.73673728	27.46591199	27.43095012	0.447244412	0.97	-0.206	PIS51630.1	WAR1	Zn(II)2Cys6 transcription factor; plays a role in resistance to weak organic acids; required for yeast cell adherence to silicone substrate; Spider biofilm induced
PIS54787.1	27.0274996	26.9254856	27.5872519	27.36208398	26.84978836	26.71317423	0.611822867	0.974	-0.205	PIS54787.1	MIM1	Predicted mitochondrial protein involved in outer membrane protein import; rat catheter biofilm repressed
PIS58770.1	26.1177104	27.2946857	27.171052	27.07560707	26.37143963	26.51997432	0.683780093	0.976	-0.205	PIS58770.1	orf19.2128	Has domain(s) with predicted ATP binding, ATPase, GTP binding activity

PIS50473.1	26.2010892	25.5661576	25.7885996	27.31760973	25.98572383	23.63842392	0.800863317	0.977	-0.205	PIS50473.1	orf19.3956	Ortholog(s) have glutaminyl-tRNA synthase (glutamine-hydrolyzing) activity and role in endoplasmic reticulum organization, glutaminyl-tRNAGln biosynthesis via transamidation
PIS52239.1	27.1575199	27.4982686	28.7005475	27.82598494	27.26793473	27.65022118	0.62741525	0.975	-0.204	PIS52239.1	orf19.1441	Component of the RSC chromatin remodeling complex; only present in CTG clade
PIS51267.1	28.8548384	29.5531909	29.3722933	29.20048251	28.66504016	29.30721227	0.760851885	0.977	-0.203	PIS51267.1	orf19.6160	Ortholog(s) have role in eisosome assembly and eisosome localization
PIS55573.1	28.6079977	28.7815627	28.7196232	28.16319732	28.2686408	29.06745396	0.589606901	0.974	-0.203	PIS55573.1	STV1	Predicted subunit a of vacuolar proton-translocating ATPase V0 domain, Golgi isoform
PIS49757.1	25.3932038	25.1207193	24.8723102	26.33287055	24.80026993	23.65159259	0.773136328	0.977	-0.201	PIS49757.1	orf19.2893	Protein of unknown function; regulation correlates with clinical development of fluconazole resistance; transcript is upregulated in an RHE model of oral candidiasis
PIS55626.1	27.5343212	27.1056206	25.279239	26.75572012	25.75138891	26.80911205	0.799430226	0.977	-0.201	PIS55626.1	SEN1	Putative helicase; repressed by prostaglandins
PIS48637.1	27.9816409	28.4859102	28.9452653	28.03094055	28.27899458	28.50211685	0.681325927	0.975	-0.2	PIS48637.1	orf19.1634	Has domain(s) with predicted fatty-acyl-CoA binding activity

PIS56677.1	30.0331199	29.9222748	29.248852	28.98009285	29.89254434	29.7311487	0.575921975	0.974	-0.2	PIS56677.1	PMT4	Protein mannosyltransferase; required for WT cell wall composition and virulence in mice; roles in hyphal growth and drug sensitivity; one of 5 family members; Axl2 a substrate; Spider biofilm repressed; Bcr1-repressed in RPMI a/a biofilm
PIS54560.1	29.9036646	29.252792	29.5755887	29.30177568	29.39715474	29.43729677	0.575978576	0.974	-0.199	PIS54560.1	orf19.577	Predicted protein tyrosine phosphatase; rat catheter biofilm induced
PIS52361.1	29.3127343	29.4645239	29.1186436	30.53598158	29.22523802	27.54170945	0.771482273	0.977	-0.198	PIS52361.1	orf19.4018	Ortholog(s) have structural constituent of ribosome activity and mitochondrial small ribosomal subunit localization
PIS51097.1	35.1607268	34.8193058	35.359586	35.15584442	34.7600066	34.8304626	0.787515522	0.977	-0.198	PIS51097.1	PCK1	Phosphoenolpyruvate carboxykinase; glucose, C-source, yeast-hypha, Hap43 regulated; fluconazole, phagocytosis, H2O2, oral candidasis, Spider/rat catheter/flow model biofilm induced; repressed in biofilm by Bcr1, Tec1, Ndt80, Rob1, Brg1
PIS58773.1	29.4044033	29.8889943	29.5641776	29.75138027	28.83709485	29.67801565	0.675001531	0.975	-0.197	PIS58773.1	GBP2	Putative single-strand telomeric DNA-binding protein; protein level decreases in stationary phase cultures; Spider biofilm repressed
PIS54541.1	32.8896453	32.9949824	32.5935005	31.99201014	32.87482605	33.02050225	0.588751844	0.974	-0.197	PIS54541.1	orf19.3799	Ortholog(s) have role in ER-dependent peroxisome organization, endoplasmic reticulum inheritance, endoplasmic reticulum tubular network maintenance and endoplasmic reticulum tubular network organization, more
PIS58722.1	26.045086	25.3587537	25.3532312	23.89556885	25.76910922	26.50294299	0.800814509	0.977	-0.196	PIS58722.1	CRK1	Protein kinase of the Cdc2 subfamily involved in hyphal development, virulence; promotes hyphal development independently of Cph1 and Efg1; functionally complements pheromone hypersensitivity of S. cerevisiae sgv1 mutant; Hap43p-

PIS49676.1	28.3077442	29.1218964	29.4355803	28.41819092	28.76548249	29.09302511	0.625886223	0.975	-0.196	PIS49676.1	MDJ1	Putative member of the HSP40 (DnaJ) family of chaperones; rat catheter and Spider biofilm induced
PIS54994.1	24.7711601	24.5276948	24.8288651	24.34453304	25.53664411	23.66677243	0.724283022	0.976	-0.193	PIS54994.1	orf19.6476	Putative protein with a predicted role in exocytic transport from the Golgi; filament induced
PIS51243.1	29.6772546	29.7368431	29.6718491	30.15118202	29.40860302	28.95435591	0.688663937	0.976	-0.191	PIS51243.1	BCY1	Protein kinase A regulatory subunit; involved in regulation of filamentation, phenotypic switching and mating; required for nuclear localization of Tpk1; physically interacts with Tpk1; apoptosis-regulated
PIS48326.1	29.0340025	28.3436707	28.2810345	28.49233835	28.12336622	28.47045761	0.648931327	0.975	-0.191	PIS48326.1	orf19.3552	Ortholog(s) have structural constituent of nuclear pore activity
PIS54762.1	25.4369481	25.4758176	25.496865	24.84071043	25.27346432	25.7272954	0.769264738	0.977	-0.189	PIS54762.1	SIP5	Protein of unknown function; flow model, rat catheter and Spider biofilm induced
PIS58684.1	27.1679531	26.2519769	23.8219271	23.20827156	26.16368104	27.30538862	0.858278432	0.978	-0.188	PIS58684.1	ANP1	Putative mannosyltransferase of Golgi; member of Mnn9p family; similar to S. cerevisiae Anp1p; fungal-specific (no human or murine homolog)
PIS48240.1	26.1777316	24.9488926	25.1945559	26.53601737	25.0781778	24.14699346	0.781813818	0.977	-0.187	PIS48240.1	orf19.6526	Ortholog(s) have nuclear periphery localization

PIS55529.1	27.8518674	27.2082779	27.6063456	26.85643957	27.65545775	27.59833051	0.570739035	0.973	-0.185	PIS55529.1	CDC37	Chaperone for Crk1p; interacts with Crk1p kinase domain and with Sti1p; putative phosphorylation site at Ser14; functional homolog of S. cerevisiae Cdc37p; likely to be essential for growth; regulated by Gcn2p and [illegible]
PIS52153.1	28.5981118	28.1408483	27.9583923	28.31091852	28.14166649	27.68846066	0.548348559	0.973	-0.185	PIS52153.1	orf19.4005	Putative dephospho-CoA kinase; protein likely to be essential for growth, based on an insertional mutagenesis strategy
PIS52167.1	29.7300726	28.6341174	29.8484182	29.4809787	29.83467213	28.34400069	0.701129481	0.976	-0.184	PIS52167.1	orf19.3103	Ortholog(s) have RNA polymerase III activity, role in tRNA transcription by RNA polymerase III and RNA polymerase III complex, chromatin localization
PIS51725.1	25.0202603	24.8762236	26.1945244	25.08566806	25.10474093	25.34820407	0.763273438	0.977	-0.184	PIS51725.1	SET3	NAD-dependent histone deacetylase; mutations affect filamentous growth; genetic evidence suggests Set3/Hos2 function as a complex to regulate white-opaque switching, morphogenesis, and virulence; flow [illegible]
PIS54598.1	26.9191724	27.820136	27.2073294	27.84548357	26.62843172	26.92425796	0.704865995	0.976	-0.183	PIS54598.1	HOC1	Protein with similarity to mannosyltransferases; similar to S. cerevisiae Hoc1p and C. albicans Och1p
PIS58994.1	25.7729668	23.16607	24.8547944	24.68908727	24.78068755	23.77624453	0.846165142	0.978	-0.183	PIS58994.1	orf19.2887	Ortholog in S. cerevisiae is localizedto the bud, mating projection tip, and associates with ribosomes; Spider biofilm induced
PIS55811.1	34.9890371	34.9243039	34.6590757	34.79876323	34.7751351	34.45565369	0.51438582	0.972	-0.181	PIS55811.1	CAM1	Putative translation elongation factor eEF1 gamma; protein level decreased in stationary phase cultures; Spider biofilm repressed

PIS56896.1	28.9599118	28.9116002	29.0948383	28.7730144	27.54781697	30.10571819	0.71199366	0.976	-0.18	PIS56896.1	SRP54	Putative signal recognition particle (SRP) subunit; induced in ssr1 null; Spider biofilm repressed
PIS51442.1	34.719413	34.3195493	34.2449077	34.76264818	34.05119797	33.93217965	0.592121369	0.974	-0.179	PIS51442.1	RPL3	Ribosomal protein, large subunit; induced by ciclopirox olamine treatment; genes encoding cytoplasmic ribosomal subunits are downregulated upon phagocytosis by murine macrophages; Hap43-induced gene; Spider biofilm repressed
PIS58422.1	28.7238496	29.3537533	29.7725831	28.7196992	29.12429093	29.47379608	0.610511122	0.974	-0.177	PIS58422.1	ERG26	C-3 sterol dehydrogenase, catalyzes the 2nd of 3 steps required to remove 2 C-4 methyl groups from an intermediate in ergosterol biosynthesis; amphotericin B, caspofungin repressed; Spider biofilm repressed
PIS58097.1	26.5123277	26.6798137	26.3090026	25.7680782	26.11067609	27.09140458	0.747446152	0.976	-0.177	PIS58097.1	orf19.6393	Putative Arf3p GTPase activating protein; Hap43p-repressed gene; possibly an essential gene, disruptants not obtained by UAU1 method
PIS49735.1	28.3562211	28.7650472	28.5180525	28.23584857	27.65933667	29.2128955	0.622232713	0.974	-0.177	PIS49735.1	orf19.6783	Putative geranylgeranyltransferase regulatory component
PIS54673.1	23.0172074	23.3969769	22.4797461	24.68067494	24.19979359	19.48389518	0.86734838	0.978	-0.177	PIS54673.1	orf19.834	Ortholog(s) have carbohydrate binding, mannosyl-oligosaccharide 1,2-alpha-mannosidase activity
PIS50329.1	28.4534946	28.097229	27.8645543	27.80822537	28.21683574	27.86018499	0.560017312	0.973	-0.177	PIS50329.1	SPC3	Essential protein; similar to S. cerevisiae Spc3p, a component of the signal peptidase complex required for signal peptidase activity; predicted integral ER membrane protein; complements spc3 and suppresses sec61 S. cerevisiae mutants

PIS51985.1	28.1078967	27.8977322	27.5800502	27.98666181	28.13110492	26.93942098	0.763053804	0.977	-0.176	PIS51985.1	orf19.3367	Ortholog(s) have structural constituent of ribosome activity and mitochondrial large ribosomal subunit localization
PIS56872.1	25.6430385	24.2404206	25.1122016	25.91628845	24.94922312	23.60632405	0.828400696	0.978	-0.175	PIS56872.1	BGL2	Cell wall 1,3-beta-glucosyltransferase; mutant has cell-wall and growth defects, but wild-type 1,3- or 1,6-beta-glucan content; antigenic; virulence role in mouse systemic infection; rat catheter biofilm induced
PIS54603.1	27.8065301	27.4165185	26.5042078	27.03930221	26.83640396	27.32556541	0.664561036	0.975	-0.175	PIS54603.1	orf19.3447	Ortholog(s) have mannosyltransferase activity, role in GPI anchor biosynthetic process, protein processing, ubiquitin-dependent ERAD pathway and endoplasmic reticulum, glycosylphosphatidylinositol-mannosyltransferase I complex
PIS49753.1	25.9325492	23.4259076	24.0711661	24.03740461	24.41840802	24.44923328	0.825202846	0.978	-0.175	PIS49753.1	orf19.4734	Protein of unknown function; Hap43-repressed
PIS51862.1	26.8541698	29.1948006	25.4474533	27.14190961	27.04629105	26.78521135	0.846461707	0.978	-0.174	PIS51862.1	orf19.376	Protein of unknown function; Hap43-repressed; Spider biofilm induced
PIS51083.1	24.5356044	24.3211741	24.7215363	24.49219212	24.19095398	24.37338908	0.664425237	0.975	-0.174	PIS51083.1	orf19.4390	Protein of unknown function; repressed by alpha pheromone in SpiderM medium; transcript induced by Mnl1 under weak acid stress
PIS48247.1	29.5243517	29.6449813	29.6871614	29.62290248	29.41781395	29.29484261	0.596830932	0.974	-0.174	PIS48247.1	RVB2	Putative trancription modulator; RuvB-like protein family member; heterozygous null mutant displays sensitivity to virgineone

PIS56753.1	22.7219412	25.6643276	26.2585456	24.94864288	24.26603492	24.91110364	0.839027415	0.978	-0.173	PIS56753.1	OPT6	Putative oligopeptide transporter; fungal-specific (no human or murine homolog); expression of OPT6, OPT7, or OPT8 does not suppress defect of mutant lacking Opt1p, Opt2p, and Opt3p; alleles are nonidentical
PIS56783.1	32.4965523	32.1867134	32.0110181	32.15513195	31.7279762	32.29936364	0.52836254	0.973	-0.171	PIS56783.1	orf19.338	Putative glycoside hydrolase; stationary phase enriched protein; Hog1p-downregulated; shows colony morphology-related gene regulation by Ssn6p
PIS56945.1	26.6299324	25.7048387	25.0224844	26.36582309	25.37246227	25.10565872	0.758275776	0.977	-0.171	PIS56945.1	orf19.5356	Protein with a predicted role in cell wall integrity; repressed in core stress response
PIS56956.1	31.4875931	31.047123	31.3586427	31.66221255	31.23504972	30.48336041	0.601483911	0.974	-0.171	PIS56956.1	orf19.5369	Uroporphyrinogen decarboxylase; catalyzes the 5th step in the heme biosynthetic pathway; flow model and Spider biofilm repressed
PIS58202.1	26.3355185	30.3133495	30.8555369	29.10009849	28.63977107	29.25556907	0.880203428	0.978	-0.17	PIS58202.1	YRB1	Functional homolog of S. cerevisiae Yrb1p; regulates Gsp1 GTPase activity and thereby affects nucleocytoplasmic transport and cytoskeletal dynamics; transcript is not regulated by white-opaque switch or by dimorphic transition
PIS49670.1	24.3301345	24.2381387	25.8520238	23.23693794	25.90430123	24.77206732	0.878105859	0.978	-0.169	PIS49670.1	PTH1	Putative gluconate transport protein; antigenic during human oral infection; possibly an essential gene, disruptants not obtained by UAU1 method
PIS56631.1	24.9467585	26.2131206	26.934179	25.32262766	25.99901772	26.27361704	0.78763401	0.977	-0.166	PIS56631.1	orf19.6872	Putative protein of unknown function; Hap43p-repressed gene

PIS58078.1	29.7081725	29.5280411	30.1762788	29.50412701	29.6456766	29.77182681	0.720177818	0.976	-0.164	PIS58078.1	MRPL8	Mitochondrial 60S ribosomal protein subunit; Hap43p-repressed gene
PIS49810.1	30.7768823	31.1923597	29.949509	30.42349758	30.42265701	30.58165267	0.738500565	0.976	-0.164	PIS49810.1	PDX3	Pyridoxamine-phosphate oxidase; transcript regulated by yeast-hypha switch and by Nrg1, Mig1, Tup1; Hap43, caspofungin repressed; present in exponential and stationary phase yeast cultures
PIS49633.1	29.9618394	29.8852469	29.3208142	29.86164254	29.39935346	29.41402559	0.641356227	0.975	-0.164	PIS49633.1	RPN7	Subunit of the proteasome regulatory particle; regulated by Gcn2p and Gcn4p; protein present in exponential and stationary growth phase yeast cultures
PIS59033.1	30.4084983	31.3502497	31.0295744	29.89129803	31.56889402	30.84014315	0.754297105	0.977	-0.163	PIS59033.1	IPP1	Putative inorganic pyrophosphatase; antigenic; soluble protein in hyphae; macrophage-induced protein; protein present in exponential and stationary phase yeast; possibly essential (UAU1 method); sumoylation target; Spider biofilm repressed
PIS50336.1	28.6495843	28.2586993	28.2103403	28.31409813	28.14159868	28.17803622	0.548663709	0.973	-0.162	PIS50336.1	MON2	Peripheral membrane protein; role in endocytosis and vacuole integrity; flow model and rat catheter biofilm repressed
PIS58683.1	25.1167966	23.1509149	24.4136649	24.61442233	24.37140654	23.21007564	0.814123756	0.977	-0.162	PIS58683.1	RGA2	Putative GTPase-activating protein (GAP) for Rho-type GTPase Cdc42; involved in cell signaling pathways controlling cell polarity; induced by low-level peroxide stress; flow model biofilm induced
PIS58537.1	24.4362357	24.3480055	24.6183801	24.60546837	24.60307609	23.70785233	0.746754997	0.976	-0.162	PIS58537.1	TOM22	Putative mitochondrial import receptor subunit; colony morphology-related gene regulation by Ssn6

PIS55523.1	23.3977161	23.6409618	25.7619377	25.17814209	23.47977777	23.67202292	0.824264381	0.978	-0.157	PIS55523.1	orf19.2143	Ortholog(s) have role in endocytic recycling, tRNA methylation, wobble position ribose methylation and cytoplasm, endosome localization
PIS58443.1	24.3359763	25.7052898	24.0777645	23.41721496	25.00137844	25.2358561	0.852772261	0.978	-0.155	PIS58443.1	orf19.3673	Ortholog(s) have guanyl-nucleotide exchange factor activity and role in chromosome organization, endoplasmic reticulum to Golgi vesicle-mediated transport
PIS58866.1	30.8997443	31.2972888	31.076363	30.432055	31.27048163	31.11170078	0.608005351	0.974	-0.153	PIS58866.1	RPN2	Putative 26S proteasome subunit; transcript regulated by Mig1; caspofungin repressed; regulated by Gcn2 and Gcn4; gene used for strain identification by multilocus sequence typing
PIS54599.1	33.2526856	33.1027645	33.2116132	33.07772131	33.29276844	32.74196519	0.771183102	0.977	-0.152	PIS54599.1	LYS9	Saccharopine dehydrogenase; lysine biosynthesis; soluble protein in hyphae; amphotericin B repressed; Gcn4-regulated; colony morphology-related gene regulation by Ssn6; protein present in exponential and stationary growth phase yeast
PIS52138.1	29.904994	29.1881139	30.0014294	30.13310432	29.26630278	29.23985429	0.71970608	0.976	-0.152	PIS52138.1	orf19.3755	Ortholog(s) have structural constituent of ribosome activity and mitochondrial large ribosomal subunit localization
PIS48574.1	29.3130171	29.3239785	29.7203175	29.56766847	29.2025797	29.13838994	0.600878033	0.974	-0.15	PIS48574.1	STE23	Ortholog of S. cerevisiae Ste23 metalloprotease; role in N-terminal processing of pro-a-factor to the mature form; Tn mutation affects filamentous growth; Spider biofilm induced
PIS58933.1	27.7186923	27.4921439	27.0230523	27.35932347	26.49674431	27.930024	0.735238787	0.976	-0.149	PIS58933.1	orf19.2115	Putative molybdopterin-converting factor; fungal-specific (no human or murine homolog)

PIS52451.1	28.5139122	29.2129436	28.067835	28.41532966	28.34280793	28.58925841	0.684471419	0.976	-0.149	PIS52451.1	VPS4	AAA-ATPase involved in transport from MVB to the vacuole and ESCRT-III complex disassembly; mutation decreases SAP secretion and virulence in murine intravenous infection; regulated by Gcn2p, Gcn4p; required for normal Rim8p processing
PIS59026.1	29.4335458	28.4037787	28.8099369	27.72502419	29.22459845	29.25324755	0.740458257	0.976	-0.148	PIS59026.1	CYB2	Putative cytochrome b2 precursor; induced in high iron; alkaline repressed; colony morphology-related gene regulation by Ssn6; Hap43-repressed; pider biofilm induced
PIS54962.1	23.3411761	22.1951508	21.9674904	23.82800155	22.57519151	20.65727424	0.862459341	0.978	-0.148	PIS54962.1	orf19.6694	Ortholog of C. dubliniensis CD36 : Cd36_73320, C. parapsilosis CDC317 : CPAR2_703860, C. auris B8441 : B9J08_002111 and Candida tenuis NRRL Y-1498 : CANTEDRAFT_114480
PIS48816.1	26.9189461	26.5849572	26.6941854	27.2904157	26.12934281	26.33754308	0.735762849	0.976	-0.147	PIS48816.1	orf19.4805	Putative membrane protein; induced by alpha pheromone in SpiderM medium; Hap4-induced gene; Spider biofilm induced
PIS51141.1	30.4050738	30.4535312	29.578289	29.79791583	29.99574242	30.20672125	0.642132903	0.975	-0.146	PIS51141.1	orf19.5126	Putative adhesin-like protein
PIS55035.1	29.3914223	29.0510757	28.6427745	28.54806685	28.98460927	29.11646429	0.661660554	0.975	-0.145	PIS55035.1	ARO3	3-deoxy-D-arabinoheptulosonate-7-phosphate synthase; aromatic amino acid synthesis; GCN-regulated; feedback-inhibited by phe if expressed in S. cerevisiae; decreased in stationary phase; flow model biofilm repressed
PIS54786.1	32.906678	33.0840293	32.8191678	32.56857239	33.04135886	32.77071161	0.638674445	0.975	-0.143	PIS54786.1	HXK2	Hexokinase II; antigenic in humans; repressed by human neutrophils; Efg1-regulated; fluconazole-induced; gene regulation by Ssn6; present in exponential and stationary growth phase; flow model biofilm induced; Spider biofilm repressed

PIS52094.1	26.0881283	24.9415134	23.5795308	25.76430333	23.55178756	24.86448722	0.867560023	0.978	-0.143	PIS52094.1	orf19.433	Ortholog(s) have guanyl-nucleotide exchange factor activity, role in intra-Golgi vesicle-mediated transport, protein-containing complex assembly and TRAPPII protein complex, trans-Golgi network localization
PIS49619.1	27.2073759	26.6534191	25.6700675	25.33743591	27.07354022	26.69615537	0.764402259	0.977	-0.141	PIS49619.1	HPC2	Ortholog(s) have DNA binding, nucleosome binding activity, role in chromatin organization, regulation of transcription by RNA polymerase II, transcription elongation by RNA polymerase II and HIR complex localization
PIS48367.1	30.0064435	30.5498996	30.5669264	30.41839307	30.23546383	30.04627222	0.631774272	0.975	-0.141	PIS48367.1	SES1	Seryl-tRNA synthetase; charges the tRNA that recognizes the CUG codon, which typically specifies Leu, but specifies Ser in C. albicans; complements S. cerevisiae ses1 mutant viability; soluble protein in hyphae; macrophage-regulated
PIS48666.1	30.1149164	30.5553534	31.2011577	30.99963635	30.26151416	30.18859836	0.706866542	0.976	-0.141	PIS48666.1	SRV2	Adenylate cyclase-associated protein; regulates adenylate cyclase activity; required for wild-type germ tube formation and for virulence in mice; mutant defects in filamentous growth are rescued by cAMP or dbcAMP; Spider biofilm repressed
PIS58373.1	27.2939319	27.46378	28.4214468	27.27110276	27.39607059	28.09177979	0.735970542	0.976	-0.14	PIS58373.1	orf19.809	Ortholog(s) have rRNA binding activity, role in maturation of LSU-rRNA from tricistronic rRNA transcript (SSU-rRNA, 5.8S rRNA, LSU-rRNA) and nucleolus, preribosome localization
PIS58663.1	27.8058506	27.7593061	27.2062351	27.10150941	27.26787871	27.98266659	0.906277599	0.979	-0.14	PIS58663.1	SKI2	Ortholog(s) have role in endoplasmic reticulum unfolded protein response, nuclear-transcribed mRNA catabolic process and 3'-5' exonucleolytic nonsense-mediated decay, more
PIS58369.1	25.093748	25.3082178	25.3678294	24.71127046	25.09917154	25.54410424	0.73274529	0.976	-0.138	PIS58369.1	orf19.805	Putative protein of unknown function; possibly mitochondrial; Hap43-repressed; Spider biofilm repressed

PIS51251.1	32.2729761	32.0143252	32.1421678	32.19618556	32.05880884	31.76132124	0.701786577	0.976	-0.138	PIS51251.1	VAS1	Putative tRNA-Val synthetase; genes encoding ribosomal subunits, translation factors, and tRNA synthetases are downregulated upon phagocytosis by murine macrophage
PIS56905.1	26.1384574	25.675613	24.8710945	24.55422361	25.85733057	25.86625429	0.824792948	0.978	-0.136	PIS56905.1	RMT2	Minor protein arginine methyltransferases (PRMT) involved in methylation of arginine residues
PIS55773.1	30.3758219	30.3138743	30.911604	30.74797066	30.54147877	29.90926763	0.678234888	0.975	-0.134	PIS55773.1	KRS1	Putative tRNA-Lys synthetase; repressed upon phagocytosis by murine macrophages; stationary phase enriched protein; Spider biofilm repressed
PIS54642.1	30.5979619	30.2226282	29.7265755	29.99908833	30.02049627	30.12800066	0.692713528	0.976	-0.133	PIS54642.1	SEC7	Putative guanine nucleotide exchange factor (GEF); mutation confers hypersensitivity to Brefeldin A; caspofungin repressed
PIS52468.1	27.3409441	27.3465096	29.4191997	27.43329858	28.73372629	27.54487822	0.852727934	0.978	-0.132	PIS52468.1	orf19.2639	Ortholog(s) have structural constituent of ribosome activity and mitochondrial large ribosomal subunit localization
PIS58628.1	29.3094417	29.0609415	28.6143759	29.22853644	28.55365264	28.80684877	0.699529454	0.976	-0.132	PIS58628.1	RTS1	Putative serine/threonine-protein phosphatase B-type regulatory subunit; transcription is regulated upon yeast-hyphal switch
PIS51582.1	27.6726941	28.3223658	28.8853216	28.5276737	28.19099867	27.76881367	0.72958634	0.976	-0.131	PIS51582.1	orf19.5206	Putative chaperone protein; role in the assembly of box H/ACA snoRNPs and thus for pre-rRNA processing; Spider biofilm induced

PIS48686.1	26.6059772	26.9794282	28.5532922	27.79955824	27.32944075	26.62202135	0.793500776	0.977	-0.129	PIS48686.1	orf19.3428	Protein of unknown function; flow model biofilm induced
PIS52180.1	32.021798	32.0066493	30.8868323	31.42039032	31.37903677	31.73521903	0.771505679	0.977	-0.127	PIS52180.1	CPY1	Carboxypeptidase Y; transcript regulated at yeast-hypha transition or macrophage response; induced human neutrophils; regulated by Gcn2 and Gcn4; putative N-glycosylation
PIS55444.1	27.1344636	27.7058474	27.8554471	27.1047619	27.82220697	27.38853663	0.738057557	0.976	-0.127	PIS55444.1	orf19.1124.2	Ortholog(s) have diphthine synthase activity and role in protein histidyl modification to diphthamide
PIS54939.1	28.6076858	28.0429303	29.5929362	29.2333705	28.66180012	27.96721628	0.825312976	0.978	-0.127	PIS54939.1	RHB1	Putative small G protein from the Ras superfamily involved in cell wall integrity and control of filamentous growth under nitrogen starvation; involved in activation of TOR1C during starvation response
PIS54733.1	30.1521242	29.4211442	29.0144462	29.44605126	29.74053372	29.03246901	0.712749321	0.976	-0.123	PIS54733.1	AGM1	Phosphoacetylglucosamine mutase (N-acetylglucosamine-phosphate mutase); enzyme of UDP-N-acetylglucosamine (UDP-GlcNAc) biosynthesis
PIS56568.1	28.4650186	27.4697149	27.7627314	27.78188643	27.91384791	27.63624869	0.727014799	0.976	-0.122	PIS56568.1	MCM6	Putative MCM DNA replication initiation complex component; mRNA expression peak at cell-cycle M/G1 phase; regulated by tyrosol and cell density; repressed by alpha pheromone in SpiderM medium; Hap43-induced gene
PIS56839.1	27.8495135	28.4025117	27.889114	28.25860178	27.83002325	27.68517458	0.752524457	0.977	-0.122	PIS56839.1	orf19.2708	Ortholog(s) have 5.8S rRNA binding, guanyl-nucleotide exchange factor activity, large ribosomal subunit rRNA binding, rRNA primary transcript binding, small ribosomal subunit rRNA binding activity and role in cytosolic ribosome assembly

PIS52320.1	23.7017064	23.3356534	23.9186493	23.38734676	22.69747888	24.50450488	0.804606382	0.977	-0.122	PIS52320.1	orf19.5239	Predicted alanine-tRNA ligase; oxidative stress-induced via Cap1
PIS58717.1	26.1723815	25.8656446	26.4530669	26.52659078	25.88614879	25.71413778	0.836938132	0.978	-0.121	PIS58717.1	RUB1	modulates the ubiquitin ligase activity of the SCF complexes
PIS52272.1	28.8762438	29.5780471	29.3565173	29.06508736	28.98496233	29.39882958	0.667233942	0.975	-0.121	PIS52272.1	SLP2	Protein similar to stomatin mechanoreception protein
PIS56548.1	24.3577363	25.5915763	25.2012715	25.3039703	25.04340739	24.44268306	0.781850542	0.977	-0.12	PIS56548.1	orf19.916	Protein that promotes apoptosis and negatively regulates filamentation; null mutant shows increased virulence and hyperfilamentation; Spider biofilm induced
PIS58024.1	29.886352	29.8527359	29.2826026	29.55598823	29.24372762	29.86209177	0.856288928	0.978	-0.12	PIS58024.1	ROM2	Putative GDP/GTP exchange factor; possibly an essential gene, disruptants not obtained by UAU1 method; Spider biofilm induced
PIS51602.1	26.0814441	23.6752438	25.6559996	25.41200236	25.25909069	24.38531672	0.882332818	0.978	-0.119	PIS51602.1	orf19.4801	Ortholog(s) have tRNA binding, tRNA dimethylallyltransferase activity, role in tRNA modification and cytosol, mitochondrion, nucleolus, nucleus localization
PIS49800.1	29.1392666	27.0408781	26.0404489	27.21536477	27.14235171	27.50845157	0.879222624	0.978	-0.118	PIS49800.1	ATG9	Protein similar to S. cerevisiae Atg9; required for early step in autophagy; required for cytoplasm to vacuole trafficking of Lap41; Spider biofilm induced

PIS51763.1	32.9085549	32.6665061	32.4078939	32.24615054	32.46257286	32.92025144	0.76238333	0.977	-0.118	PIS51763.1	orf19.1054	Ortholog(s) have role in endoplasmic reticulum tubular network membrane organization, mitotic spindle pole body insertion into the nuclear envelope and mitotic spindle pole body localization, more
PIS50404.1	29.3349097	29.5753195	29.573495	29.47501819	29.76241058	28.89133561	0.734433547	0.976	-0.118	PIS50404.1	orf19.4825	Mitochondrial matrix protein; required for assembly/stability of the F1 sector of mitochondrial F1F0 ATP synthase at high temperature; rat catheter biofilm repressed
PIS49727.1	27.677778	27.0618267	28.1237889	27.39332929	26.79769767	28.32435569	0.773041533	0.977	-0.116	PIS49727.1	HHT1	Histone H3 variant, specific to CTG-clade species; acts in regulation of planktonic and biofilm growth; reduced mRNA abundance in fkh2 mutant; farnesol regulated; Spider biofilm induced; rat catheter biofilm repressed
PIS49637.1	24.9514752	25.0397858	25.2360649	25.47428978	24.70006629	24.70610981	0.820116982	0.978	-0.116	PIS49637.1	SWI4	Putative component of the SBF transcription complex involved in G1/S cell-cycle progression; periodic mRNA expression, peak at cell-cycle G1/S phase; predicted, conserved MBF binding sites upstream of G1/S-regulated genes
PIS49634.1	25.4355531	24.8087715	26.0777836	25.20330085	25.14323135	25.62618958	0.841109186	0.978	-0.116	PIS49634.1	UBC8	Predicted ubiquitin-conjugating enzyme that negatively regulates gluconeogenesis by mediating the glucose-induced ubiquitination of fructose-1,6-bisphosphatase; induced by alpha pheromone in SpiderM medium
PIS58427.1	23.9166178	25.4190873	25.7290512	24.83097572	24.71182729	25.17566167	0.829599352	0.978	-0.115	PIS58427.1	orf19.2848	Predicted regulatory subunit of the Atg1 signaling complex; required for vesicle formation during autophagy, biofilm formation, and the cytoplasm-to-vacuole targeting (Cvt) pathway; Spider biofilm induced
PIS54789.1	29.6412454	29.3354443	29.0947925	27.43587937	30.1912534	30.09822934	0.846667929	0.978	-0.115	PIS54789.1	orf19.547	Ortholog(s) have 5'-3' DNA exonuclease activity, 5'-3' exonuclease activity, 5'-flap endonuclease activity, double-stranded DNA 5'-3' exodeoxyribonuclease activity, single-stranded DNA 5'-3' DNA exonuclease activity

PIS51405.1	32.2736612	31.9368745	31.6406401	31.84288126	31.93056728	31.7353175	0.668938124	0.975	-0.114	PIS51405.1	GLC3	Putative 1,4-glucan branching enzyme; fluconazole-induced; colony morphology-related gene regulation by Ssn6; stationary phase enriched protein
PIS58518.1	32.5511147	32.555824	32.8267472	32.71910916	32.39563467	32.48104094	0.660294608	0.975	-0.113	PIS58518.1	ACC1	Putative acetyl-coenzyme-A carboxylases; regulated by Efg1; amphotericin B repressed; caspofungin repressed; 5'-UTR intron; gene used for strain identification by multilocus sequence typing; Hap43-induced; flow model biofilm repressed
PIS55044.1	26.995881	28.9332964	27.6096764	27.32022133	28.09435492	27.78650744	0.84790986	0.978	-0.113	PIS55044.1	orf19.2089	Ortholog of Nyv1, v-SNARE component of the vacuolar SNARE complex involved in vesicle fusion in S. cerevisiae; Hap43-repressed gene
PIS51767.1	24.5759712	24.1251685	24.3758339	24.64766828	23.89115256	24.20115942	0.806522031	0.977	-0.112	PIS51767.1	orf19.1063	Ortholog(s) have alpha-1,6-mannosyltransferase activity, mannosyltransferase activity, role in GPI anchor biosynthetic process and endoplasmic reticulum, endoplasmic reticulum membrane, mannosyltransferase complex
PIS51801.1	23.744599	24.5698005	24.8508493	26.04382064	23.29621878	23.48962165	0.886890081	0.978	-0.112	PIS51801.1	orf19.3297	Ortholog(s) have structural constituent of ribosome activity and mitochondrial small ribosomal subunit localization
PIS51260.1	30.0347359	30.3929905	30.6587176	29.86519769	30.4106039	30.47860566	0.717328556	0.976	-0.111	PIS51260.1	orf19.5669	Protein with B-cell receptor-associated protein 31-like domain; membrane-localized protein
PIS50333.1	32.8872834	32.5894663	32.0598863	31.91723603	32.34581643	32.94463696	0.787183984	0.977	-0.11	PIS50333.1	OP4	Ala- Leu- and Ser-rich protein; secreted; N-terminal hydrophobic region; possible glycosylation; opaque-specific transcript; repressed by alpha pheromone in opaque MTLa homozygotes; fluconazole-induced; Spider biofilm induced

PIS52223.1	26.7169669	26.5242776	26.3683986	26.54750566	26.45658113	26.2801598	0.875112554	0.978	-0.108	PIS52223.1	NAG1	Glucosamine-6-phosphate deaminase; required for normal hyphal growth and mouse virulence; converts glucosamine 6-P to fructose 6-P; reversible reaction in vitro; gene and protein is GlcNAc-induced; Spider biofilm induced
PIS58695.1	26.5853976	27.8230714	28.157705	27.67747169	27.24560723	27.32138977	0.8449118	0.978	-0.107	PIS58695.1	COX4	Putative cytochrome c oxidase subunit IV; Mig1-regulated; macrophage/pseudohyphal-induced gene; macrophage-induced protein; repressed by nitric oxide; 5'-UTR intron; Hap43-repressed
PIS50528.1	27.6052715	27.6003829	26.9198948	27.37024814	27.94903652	26.48378264	0.775353886	0.977	-0.107	PIS50528.1	orf19.3914	Has domain(s) with predicted RNA binding, translation initiation factor activity, role in translational initiation and cytoplasm localization
PIS51075.1	23.4866434	23.8430123	23.6018607	23.98983715	23.4333874	23.19279907	0.783085222	0.977	-0.105	PIS51075.1	HXT5	Putative sugar transporter; induced by ciclopirox olamine; Snf3-induced; alkaline repressed; colony morphology-related gene regulation by Ssn6; possibly essential gene
PIS51517.1	33.621271	33.6770508	33.6197254	33.10203941	33.59359872	33.91068181	0.736749813	0.976	-0.104	PIS51517.1	RPS10	Ribosomal protein S10; downregulated in the presence of human whole blood or PMNs; Spider biofilm repressed
PIS56868.1	28.506906	28.5600964	28.3892308	28.5664932	28.21355714	28.3649946	0.827659522	0.978	-0.104	PIS56868.1	UBR1	Protein similar to S. cerevisiae Ubr1p ubiquitin-protein ligase; regulates stability of Ume6p in response to oxygen availability; transposon mutation affects filamentous growth; Spider biofilm induced
PIS58785.1	28.5133466	28.9759076	28.857826	28.6485265	28.82712807	28.56189542	0.82653696	0.978	-0.103	PIS58785.1	HNT1	Histidine triad nucleotide-binding protein; protein level decreases in stationary phase cultures

PIS55597.1	29.2155077	29.1837205	29.7662672	29.66950604	28.93914287	29.24659611	0.845044638	0.978	-0.103	PIS55597.1	VMA2	Vacuolar H(+)-ATPase; protein present in exponential and stationary growth phase yeast cultures; plasma membrane localized; amphotericin B repressed, caspofungin repressed
PIS48566.1	24.8077373	25.0351316	24.7707851	25.2120834	24.89403262	24.20140528	0.840372479	0.978	-0.102	PIS48566.1	DPP2	Protein similar to S. cerevisiae pyrophosphate phosphatase Lpp1p; possible role in farnesol biosynthesis
PIS51674.1	30.7621155	30.7634049	30.6176775	30.62193664	30.50840887	30.70634541	0.705797335	0.976	-0.102	PIS51674.1	LYS2	Heterodimeric alpha-aminoadipate reductase large subunit; lysine biosynthesis; predicted binding sites for AMP and alpha-aminoadipate; inhibited by lys or thialysine; regulated by Gcn2 and Gcn4; Spider biofilm induced, flow model repressed
PIS51742.1	30.027662	29.1690029	29.0110117	27.08774851	30.29593711	30.5188692	0.894170468	0.978	-0.102	PIS51742.1	MXR1	Putative methionine sulfoxide reductase; Plc1-regulated; induced by human neutrophils, flucytosine; macrophage regulated (gene induced, protein decreased); possibly adherence-induced; Spider biofilm induced
PIS58969.1	24.1317824	24.0740664	24.8163964	24.49080899	23.53815836	24.68699556	0.825486852	0.978	-0.102	PIS58969.1	orf19.1950	Ortholog(s) have ADP-ribosyl-[dinitrogen reductase] hydrolase activity, phosphatase activity
PIS51774.1	24.3887026	23.2869303	24.1457473	23.71338167	23.87346461	23.93075156	0.819259802	0.977	-0.101	PIS51774.1	orf19.1070	Ortholog(s) have role in phospholipid translocation, retrograde transport, endosome to Golgi and endosome membrane, trans-Golgi network membrane localization
PIS52247.1	29.227343	29.1472578	29.9808321	29.50939092	29.42953832	29.11224248	0.757178739	0.977	-0.101	PIS52247.1	SPT5	Protein similar to S. cerevisiae Spt5p transcription elongation factor; transposon mutation affects filamentous growth

PIS58824.1	23.9168485	24.58353	24.2162526	24.47547256	24.03350953	23.91234061	0.805706318	0.977	-0.0984	PIS58824.1	orf19.7182	Ortholog(s) have RNA binding activity and role in tRNA modification
PIS52229.1	26.4417645	25.7762023	24.9236449	27.17094444	24.53831527	25.14266434	0.885494224	0.978	-0.0966	PIS52229.1	orf19.6379	Ortholog of C. dubliniensis CD36 : Cd36_33820, C. parapsilosis CDC317 : CPAR2_206210, C. auris B8441 : B9J08_003843 and Candida tenuis NRRL Y-1498 : CANTEDRAFT_92567
PIS58214.1	32.3175279	31.4309166	31.8354851	31.41340884	32.01651493	31.8645347	0.789732207	0.977	-0.0965	PIS58214.1	orf19.3035	Ortholog(s) have ATP-dependent activity, acting on DNA, ATP-dependent chromatin remodeler activity, DNA binding, chromatin DNA binding, methylated histone binding, nucleosome binding and rDNA binding, more
PIS50630.1	25.3767851	25.0273931	24.4603916	25.07280642	24.01334585	25.4916996	0.865301778	0.978	-0.0956	PIS50630.1	ZCF27	Putative Zn(II)2Cys6 transcription factor
PIS51515.1	27.8255393	27.7623795	28.7783987	27.58848009	28.30178243	28.19442597	0.809223247	0.977	-0.0939	PIS51515.1	ILV1	Putative threonine dehydratase; regulated by Gcn4 and Gcn2; induced by amino acid starvation (3-AT); protein present in yeast exponential and stationary phase cultures; early-stage flow model biofilm induced
PIS48507.1	31.9883658	32.109844	32.3713647	31.87847748	32.24035232	32.07138959	0.736843689	0.976	-0.0931	PIS48507.1	PYC2	Putative pyruvate carboxylase; binds biotin cofactor; repressed by Ssk1 response regulator, by benomyl treatment, or in an azole-resistant strain overexpressing MDR1; stationary phase enriched protein; flow model biofilm repressed
PIS58485.1	26.9964599	27.7673269	27.9000228	27.90663589	27.22945817	27.24900467	0.824632225	0.978	-0.0929	PIS58485.1	CST20	Protein kinase of Ste20p/p65PAK family, required for wild-type mating efficiency and virulence in a mouse model; Cst20p-Hst7p-Cek1p-Cph1p MAPK pathway regulates some hyphal growth; involved in Cdc42p growth regulation

PIS58841.1	24.3800743	22.7790164	18.4534214	24.84863235	24.26767286	16.21852024	0.962620481	0.979	-0.0926	PIS58841.1	orf19.1417	Ortholog of C. dubliniensis CD36 : Cd36_43970, C. parapsilosis CDC317 : CPAR2_401720, C. auris B8441 : B9J08_000295 and Candida tenuis NRRL Y-1498 : CANTEDRAFT_102588
PIS48254.1	28.7076276	28.9426353	28.2523193	29.0487982	28.10999355	28.47901117	0.820802385	0.978	-0.0883	PIS48254.1	orf19.6020	Ortholog(s) have Atg8-family ligase activity
PIS55609.1	28.6695413	28.778842	29.0043594	28.87290071	28.57206784	28.74392884	0.739984378	0.976	-0.0879	PIS55609.1	ALT1	Putative alanine transaminase; mutation confers hypersensitivity to 5-fluorocytosine (5-FC); rat catheter and flow model biofilm induced
PIS58649.1	26.7995423	26.1702143	26.3251386	25.80628323	26.93883743	26.28697127	0.820354035	0.978	-0.0876	PIS58649.1	orf19.1604	Zinc cluster DNA-binding transcription factor; positive regulator of filamentous growth; Spider biofilm induced
PIS58757.1	25.0485586	25.6503198	26.2906123	26.00187633	25.66407521	25.06284244	0.882402226	0.978	-0.0869	PIS58757.1	orf19.1642	Ortholog of S. cerevisiae Loc1, a nuclear protein involved in asymmetric localization of ASH1 mRNA in S. cerevisiae; Hap43-induced gene; Spider biofilm induced
PIS56861.1	26.90102	26.5809309	27.2093209	26.97020369	26.35351353	27.11029539	0.875842861	0.978	-0.0858	PIS56861.1	orf19.2756	Ortholog(s) have HDEL sequence binding activity, role in endoplasmic reticulum to Golgi vesicle-mediated transport and endoplasmic reticulum membrane localization
PIS55807.1	32.0235361	30.7108072	31.798833	30.49333343	31.98480032	31.79830754	0.927875656	0.979	-0.0856	PIS55807.1	orf19.7386	Ortholog(s) have mitochondrial ribosome binding activity and role in inner mitochondrial membrane organization, positive regulation of mitochondrial translation, protein insertion into mitochondrial inner membrane from matrix

PIS58079.1	25.9766566	25.9314269	23.8201989	24.73790501	25.88499182	24.84898956	0.896818904	0.978	-0.0855	PIS58079.1	KSR1	3-ketosphinganine reductase, catalyzes the second step in phytosphingosine synthesis
PIS56876.1	25.5404383	25.7229141	27.2747409	26.27958132	26.1919103	25.81402145	0.897166214	0.978	-0.0842	PIS56876.1	LAB5	Ortholog(s) have role in protein lipoylation
PIS58281.1	30.427077	30.6923135	31.0461026	30.39028194	30.71714209	30.80550677	0.768688724	0.977	-0.0842	PIS58281.1	TPS2	Trehalose-6-phosphate (Tre6P) phosphatase; mutant heat sensitive, accumulates Tre6P, decreased mouse virulence; possible drug target; 2 conserved phospohydrolase motifs; no mammalian homolog; Hap43-repressed; flow model biofilm induced
PIS50524.1	34.0277701	34.1785696	34.3555298	33.92942058	34.18950642	34.19092144	0.754103479	0.977	-0.084	PIS50524.1	SAH1	S-adenosyl-L-homocysteine hydrolase; sulfur amino acid metabolism; antigenic in human; alkaline-, fluconazole-induced expression; Gcn4-regulated; amino acid starvation (3-AT) repressed; flow model biofilm induced; Spider biofilm repressed
PIS54726.1	25.8376159	23.7980623	26.938899	25.15073373	26.17377687	24.99908163	0.906963225	0.979	-0.0837	PIS54726.1	orf19.6355	Ortholog(s) have unfolded protein binding activity, role in ribosome biogenesis and nucleolus localization
PIS51510.1	24.9825031	24.2176214	23.4447417	24.2417636	23.44422592	24.70885071	0.92704957	0.979	-0.0833	PIS51510.1	orf19.5486	Putative endoribonuclease; heterozygous null mutant exhibits hypersensitivity to parnafungin and cordycepin in the C. albicans fitness test
PIS54507.1	24.9204326	24.9523233	23.9412331	25.12777594	24.26419396	24.17661725	0.915566092	0.979	-0.0818	PIS54507.1	TAF145	Protein similar to S. cerevisiae Taf145p, a component of RNA polymerase II transcription factor TFIID; flucytosine repressed; likely to be essential for growth, based on an insertional mutagenesis strategy

PIS54942.1	27.0958526	27.5312654	25.0335085	27.04091502	26.28232371	26.09420717	0.926889349	0.979	-0.0811	PIS54942.1	orf19.7365	Ortholog(s) have ubiquitin protein ligase activity, role in negative regulation of apoptotic process, negative regulation of gluconeogenesis, proteasome-mediated ubiquitin-dependent protein catabolic process and GID complex
PIS50591.1	24.4014006	24.2085136	26.1973274	25.143456	24.52635181	24.89600845	0.889650744	0.978	-0.0805	PIS50591.1	orf19.4191.1	Ortholog(s) have ubiquitin-ubiquitin ligase activity and role in free ubiquitin chain polymerization, protein polyubiquitination, ubiquitin-dependent ERAD pathway, ubiquitin-dependent protein catabolic process
PIS51451.1	27.1173489	27.537978	27.0381713	27.56992165	26.97489271	26.90980641	0.852003772	0.978	-0.0796	PIS51451.1	TAZ1	Putative lyso-phosphatidylcholine acyltransferase, required for normal phospholipid content of mitochondrial membranes; rat catheter biofilm induced
PIS49584.1	30.2275958	29.567089	30.0900121	30.15717474	29.67704977	29.81160592	0.786809014	0.977	-0.0796	PIS49584.1	VAC8	Protein involved in vacuolar inheritance; required for hyphal growth; contains armadillo repeats
PIS54715.1	29.3552838	29.4406767	28.743018	28.94121603	29.14698139	29.21258657	0.824763812	0.978	-0.0794	PIS54715.1	MTR10	Putative importin; member of a family of fungal-specific nuclear importins; Mig1-regulated
PIS48532.1	33.5770255	33.6332716	33.1215491	33.25099417	33.62310779	33.22220524	0.773941573	0.977	-0.0785	PIS48532.1	GLK1	Putative glucokinase; transcript regulated upon yeast-hyphal switch; Efg1 regulated; fluconazole-induced; induced in core stress response; colony morphology-related gene regulation by Ssn6; GlcNAc-induced protein
PIS55775.1	28.414448	28.4768245	28.9066633	27.97424939	28.81506187	28.77340783	0.871277274	0.978	-0.0784	PIS55775.1	orf19.6752	Ortholog(s) have structural constituent of ribosome activity and mitochondrial small ribosomal subunit localization

PIS56689.1	31.8198136	31.5392821	31.132732	31.55711541	31.42627646	31.2754171	0.799315684	0.977	-0.0777	PIS56689.1	PMT2	Protein mannosyltransferase (PMT) with roles in hyphal growth and drug sensitivity; member of the PMT family which includes Pmt1p, Pmt2p, Pmt4p, Pmt5p, and Pmt6p; induced during cell wall regeneration; essential
PIS52432.1	30.5378599	30.6658681	30.1403177	30.4128715	30.45847708	30.23973904	0.765084526	0.977	-0.0777	PIS52432.1	SFH5	Putative phosphatidylinositol transporter; rat catheter and Spider biofilm repressed
PIS54494.1	27.1559081	26.5312599	27.861476	27.02974588	26.8235788	27.46334158	0.911333029	0.979	-0.0773	PIS54494.1	PRE9	Alpha3 (C9) subunit of the 20S proteasome; transcript regulated by Mig1; flow model biofilm repressed
PIS52221.1	29.0544232	29.2379534	28.9343732	29.04908573	29.01666181	28.93787682	0.768230604	0.977	-0.0744	PIS52221.1	NAG6	Protein required for wild-type mouse virulence and wild-type cycloheximide resistance; putative GTP-binding motif; similar to S. cerevisiae Yor165Wp; in gene cluster that encodes enzymes of GlcNAc catabolism; no human
PIS52233.1	28.7999817	28.6435813	28.6478379	28.70934332	28.75868527	28.40138979	0.813749893	0.977	-0.074	PIS52233.1	MNN24	Alpha-1,2-mannosyltransferase; required for normal cell wall mannan content
PIS56955.1	26.7963527	29.7410268	24.4107783	27.32178576	27.9073588	25.50122475	0.958663463	0.979	-0.0726	PIS56955.1	orf19.5368	Ortholog of Vms1; component of a Cdc48-complex involved in protein quality control in S. cerevisiae; Spider biofilm repressed
PIS58686.1	29.3410722	28.4339974	28.2541709	28.69918274	28.85456954	28.25833193	0.829814003	0.978	-0.0724	PIS58686.1	orf19.2113	Putative integral peroxisomal membrane protein; Hap43p-repressed gene

PIS55433.1	31.492819	31.3155986	31.0531976	31.14017761	31.28812384	31.21694056	0.787637696	0.977	-0.0721	PIS55433.1	VPS70	Ortholog of S. cerevisiae : VPS70, C. glabrata CBS138 : CAGL0M00616g, C. dubliniensis CD36 : Cd36_70380, C. parapsilosis CDC317 : CPAR2_300190 and Candida tenuis NRRL Y-1498 : CANTEDRAFT_108527
PIS48667.1	29.1880606	29.0096969	28.8424172	29.1440044	29.04546358	28.63885543	0.866527247	0.978	-0.0706	PIS48667.1	YDJ1	Putative type I HSP40 co-chaperone; heavy metal (cadmium) stress-induced
PIS51797.1	29.831613	29.8503585	30.2045508	29.39795252	30.09661114	30.18249474	0.847988913	0.978	-0.0698	PIS51797.1	PGA63	Component COPII vesicle coat; required for vesicle formation in ER to Golgi transport; predicted GPI-anchor; flow model and Spider biofilm repressed
PIS56878.1	27.6439226	27.9900875	28.2266626	27.59611914	27.77191403	28.28416232	0.84512532	0.978	-0.0695	PIS56878.1	BEM3	Putative GTPase-activating protein (GAP) for Rho-type GTPase Cdc42p; involved in cell signaling pathways that control cell polarity; similar to S. cerevisiae Bem3p
PIS51719.1	25.8509158	25.7361682	25.3204272	25.57975186	25.57379638	25.54851242	0.837822707	0.978	-0.0685	PIS51719.1	orf19.7228	Possible Golgi membrane protein; transcript positively regulated by Tbf1; mRNA binds She3
PIS48413.1	27.5308234	24.7975897	26.2309922	25.85558905	26.06829966	26.43383585	0.928914211	0.979	-0.0672	PIS48413.1	STE13	Putative pheromone-processing dipeptidyl aminopeptidase; possible Kex2 substrate; transposon mutation affects filamentous growth; induced by low nitrogen, germ tube formation; flow model biofilm repressed
PIS58340.1	28.2760226	29.0251442	28.9892848	27.5962659	29.3364131	29.16385181	0.889773274	0.978	-0.0646	PIS58340.1	orf19.7092	Putative NAD dependent epimerase/dehydratase family protein; Spider biofilm repressed

PIS55050.1	27.8049617	25.8838228	28.0272759	27.17434208	26.98161599	27.37101594	0.941765175	0.979	-0.063	PIS55050.1	orf19.2068	Ortholog of C. dubliniensis CD36 : Cd36_15550, C. parapsilosis CDC317 : CPAR2_213190, C. auris B8441 : B9J08_002200 and Candida tenuis NRRL Y-1498 : CANTEDRAFT_119026
PIS49721.1	28.263654	27.195601	27.1061938	27.29169063	27.52887028	27.56135007	0.898043491	0.978	-0.0612	PIS49721.1	POS5	Protein similar to S. cerevisiae Pos5p, a mitochondrial NADH kinase involved in the oxidative stress response; planktonic growth-induced gene; likely to be essential for growth, based on an insertional mutagenesis strategy
PIS48309.1	30.8659514	30.7200893	29.2798527	31.14109492	30.29261006	29.24845759	0.91770964	0.979	-0.0612	PIS48309.1	RPP2A	Acidic ribosomal protein; likely role in translation elongation regulation; interacts with Rpp1B; 1 of 4 similar ribosomal proteins (Rpp1A, Rpp1B, Rpp2A, Rpp2B); CUG start codon; upstream uORFs; Tbf1-induced; Spider biofilm repressed
PIS52235.1	29.6965806	28.837101	27.8053522	28.26207944	29.10837568	28.78599556	0.891109822	0.978	-0.0609	PIS52235.1	orf19.1993	Subunit of the 19S regulatory particle lid of the proteasome
PIS52066.1	32.4341975	32.7448847	32.30476	32.60559161	32.30775136	32.39671606	0.836266823	0.978	-0.0579	PIS52066.1	RPS23A	Putative ribosomal protein; repressed upon phagocytosis by murine macrophage; Spider biofilm repressed
PIS50317.1	28.5550283	27.5790514	26.7543905	27.1949757	27.85314961	27.6688593	0.905215983	0.979	-0.0572	PIS50317.1	NMD5	Karyopherin; carrier protein involved in nuclear import of proteins; repressed in core stress response; Hap43-induced; Spider biofilm induced
PIS48186.1	28.9912515	28.4278894	28.6130167	28.04704939	29.06882951	28.74665692	0.866130224	0.978	-0.0565	PIS48186.1	orf19.6503	Ortholog(s) have structural constituent of ribosome activity, role in mitochondrial translation and mitochondrial large ribosomal subunit localization

PIS51923.1	27.862346	28.4619334	27.7922447	27.42597293	28.03272048	28.48869472	0.876691151	0.978	-0.0564	PIS51923.1	orf19.4176	Ortholog(s) have structural constituent of ribosome activity and mitochondrial small ribosomal subunit localization
PIS58377.1	31.6624451	31.4395132	31.4691166	31.40616445	31.48980007	31.50687591	0.887314079	0.978	-0.0561	PIS58377.1	OSM1	Putative flavoprotein subunit of fumarate reductase; soluble protein in hyphae; caspofungin repressed; stationary phase enriched protein; flow model biofilm induced; Spider biofilm repressed
PIS48525.1	27.8491757	27.2439673	26.8410706	27.82889565	27.0724177	26.87255158	0.910771906	0.979	-0.0534	PIS48525.1	USO1	Ortholog(s) have role in Golgi vesicle docking, SNARE complex assembly, endoplasmic reticulum to Golgi vesicle-mediated transport and ER to Golgi transport vesicle membrane, Golgi membrane localization
PIS56782.1	29.2327571	28.9912614	29.435485	28.80949803	29.1932809	29.49988138	0.923857163	0.979	-0.0523	PIS56782.1	VTI1	Ortholog(s) have SNAP receptor activity and role in Golgi to vacuole transport, intra-Golgi vesicle-mediated transport, macroautophagy, vacuole fusion, non-autophagic, vesicle fusion
PIS50313.1	26.3346585	25.5580481	25.9058257	26.94720445	25.91685692	24.78226787	0.946263811	0.979	-0.0507	PIS50313.1	IAH1	Protein similar to S. cerevisiae Iah1p, which is involved in acetate metabolism; mutation confers hypersensitivity to tunicamycin; transposon mutation affects filamentous growth
PIS51949.1	24.792258	24.1238637	24.738825	25.45368291	23.18903871	24.86052896	0.944787213	0.979	-0.0506	PIS51949.1	orf19.993	Protein of unknown function; rat catheter biofilm repressed
PIS51376.1	29.4234714	28.7182371	29.7824526	29.16870196	29.22033653	29.38426146	0.895721315	0.978	-0.0503	PIS51376.1	VPS35	Putative role in vacuolar sorting; downregulated in biofilm; induced upon adherence to polystyrene

PIS58479.1	27.2450025	27.2628395	27.0933418	26.23585092	27.74998394	27.46470912	0.919356037	0.979	-0.0502	PIS58479.1	ALG5	Putative glucosyltransferase involved in cell wall mannan biosynthesis; possibly an essential gene, disruptants not obtained by UAU1 method
PIS51661.1	24.6204035	24.6760186	24.1984926	24.21796874	24.56655009	24.5597441	0.945745276	0.979	-0.0502	PIS51661.1	orf19.2982	Putative autophagy-related gene; upregulated during biofilm production
PIS51058.1	27.8249576	27.6032546	28.0853248	27.51173549	27.72019758	28.13147158	0.906428574	0.979	-0.05	PIS51058.1	ILV2	Putative acetolactate synthase; regulated by Gcn4p; induced by amino acid starvation (3-AT treatment); stationary phase enriched protein
PIS50573.1	29.6736369	28.08152	29.2984972	29.32092712	28.49992325	29.0840209	0.909240538	0.979	-0.0496	PIS50573.1	orf19.3949	Ortholog(s) have ATP-dependent H3-H4 histone complex chaperone activity, ATP-dependent chromatin remodeler activity, ATPase, chromatin binding, histone binding activity
PIS51280.1	34.8024191	35.0229933	34.6334127	35.08127049	34.752664	34.47623163	0.858243718	0.978	-0.0496	PIS51280.1	PMA1	Plasma membrane H(+)-ATPase; highly expressed, comprises 20-40% of total plasma membrane protein; levels increase at stationary phase transition; fluconazole induced; caspofungin repressed; upregulated in RHE model; Spider biofilm repressed
PIS56890.1	26.3121882	26.2639041	27.2014832	26.75286518	26.91110389	25.96552843	0.919051499	0.979	-0.0494	PIS56890.1	orf19.5552	Putative transcriptional regulator of ribonucleotide reductase genes; Spider biofilm induced
PIS58090.1	29.3694518	29.2361844	29.0080968	28.7691009	29.40782932	29.28964706	0.875896564	0.978	-0.0491	PIS58090.1	orf19.597	Protein with an aspartate aminotransferase domain; Gcn4-regulated

PIS52185.1	27.6863497	27.0808132	28.4661027	27.4964779	27.45772584	28.13304006	0.916078671	0.979	-0.0487	PIS52185.1	RAP1	Transcription factor; binds telomeres and regulatory sequences in DNA; involved in telomere maintenance; represses hyphal growth under yeast-favoring conditions; similar to (but shorter than) S. cerevisiae Rap1
PIS58701.1	23.1909534	23.9167275	23.8504667	23.62478959	23.37289128	23.81531606	0.894094234	0.978	-0.0484	PIS58701.1	orf19.1479	Ortholog of the mitochondria localized S. cerevisiae Pib2 protein of unknown function; has a FYVE zinc finger domain; Spider biofilm induced
PIS51504.1	29.1527783	28.5424805	28.1384058	29.11428925	28.52578808	28.04964835	0.893836104	0.978	-0.048	PIS51504.1	MNN21	Ortholog(s) have alpha-1,2-mannosyltransferase activity, role in protein glycosylation and Golgi apparatus localization
PIS48522.1	27.1875905	27.0376488	27.171271	26.74333708	28.02014784	26.49597733	0.927918169	0.979	-0.0457	PIS48522.1	SSK2	MAP kinase kinase kinase (MAPKKK); regulates Hog1 activation and signaling; repressed by ciclopirox olamine
PIS52264.1	25.3904324	25.4427412	26.4970076	25.63801496	24.99685507	26.56148322	0.915704683	0.979	-0.0446	PIS52264.1	orf19.5184	Putative Ran guanyl-nucleotide exchange factor; probable signal transducer; Spider biofilm repressed
PIS49470.1	29.8317202	29.9496136	30.1826403	30.2240463	29.62716778	29.98025758	0.923252283	0.979	-0.0442	PIS49470.1	orf19.51	Ortholog(s) have RNA binding activity
PIS50612.1	28.3199193	28.1940601	28.6858672	28.56772546	28.32967987	28.17335143	0.889889367	0.978	-0.043	PIS50612.1	orf19.6966	Ortholog(s) have choline kinase activity, ethanolamine kinase activity and role in phosphatidylcholine biosynthetic process, phosphatidylethanolamine biosynthetic process

PIS58662.1	30.0501349	30.259863	29.51289	30.17501974	30.03718605	29.48882659	0.919134474	0.979	-0.0406	PIS58662.1	PTC5	Mitochondrial protein phosphatase of the Type 2C-related family (serine/threonine-specific), involved in drug response and cadmium tolerance
PIS49482.1	25.9511017	24.4416575	27.9007557	27.08422777	25.28246882	25.8101618	0.97209734	0.979	-0.0389	PIS49482.1	orf19.7321	Ortholog(s) have role in re-entry into mitotic cell cycle after pheromone arrest and endoplasmic reticulum localization
PIS51192.1	25.6583137	25.9574692	25.6601813	25.56919375	25.79020562	25.80702763	0.939566285	0.979	-0.0365	PIS51192.1	orf19.7604	Ortholog(s) have guanyl-nucleotide exchange factor activity, role in early endosome to Golgi transport, intra-Golgi vesicle-mediated transport and TRAPPII protein complex, early endosome, trans-Golgi network localization
PIS51955.1	31.1923723	31.1504686	30.7873574	31.52280354	31.16122438	30.33817804	0.931423409	0.979	-0.036	PIS51955.1	SLK19	Alkaline-induced protein of plasma membrane; affects cell aggregation, cell wall; similar to S. cerevisiae Slk19p (a kinetochore protein with roles in mitosis, meiosis); required for wild-type virulence in mouse; macrophage-downregulated
PIS52092.1	26.0845203	24.5850107	23.1679575	25.46058252	24.4181219	23.85275387	0.964207928	0.979	-0.0353	PIS52092.1	SSU1	Protein similar to S. cerevisiae Ssu1 sulfite transport protein; Tn mutation affects filamentous growth; regulated by Gcn2 and Gcn4; induced by nitric oxide; Hap43-repressed; Spider and flow model biofilm induced
PIS58285.1	27.0273125	26.7008511	25.00797	25.96905585	26.78834696	25.87852455	0.953833479	0.979	-0.0334	PIS58285.1	orf19.3030	Ortholog(s) have phosphatidylinositol-3,5-bisphosphate binding, phosphatidylinositol-3-phosphate binding, phosphatidylinositol-4-phosphate binding activity
PIS48318.1	27.4848334	27.7355276	28.3384145	27.48391975	28.38475548	27.59045342	0.94150128	0.979	-0.0332	PIS48318.1	UTP22	Putative U3 snoRNP protein; Ssr1-induced; repressed by prostaglandins; heterozygous null mutant is resistant to parnafungin

PIS58854.1	24.6735179	24.9140959	24.8679653	25.06889735	24.82737176	24.46000436	0.952077976	0.979	-0.0331	PIS58854.1	orf19.5040	Ortholog(s) have phospholipid binding, single-stranded DNA binding, structural constituent of nuclear pore activity and role in mRNA export from nucleus in response to heat stress, nuclear pore organization
PIS58154.1	30.1008182	30.7364657	30.7290396	30.79510832	30.39721258	30.27640642	0.935698745	0.979	-0.0325	PIS58154.1	SEC18	Functional homolog of S. cerevisiae Sec18p, which acts in protein transport; conserved ATP binding site; not glycosylated
PIS50337.1	30.5354876	30.1724016	30.4210831	31.05497883	30.14156848	29.83848714	0.931426603	0.979	-0.0313	PIS50337.1	HMX1	Heme oxygenase; utilization of hemin iron; transcript induced by heat, low iron, or hemin; repressed by Efg1; induced by low iron; upregulated by Rim101 at pH 8; Hap43-induced; Spider and flow model biofilm induced
PIS49564.1	26.7965515	27.2512054	27.30205	27.11871954	26.95584466	27.18338861	0.918703862	0.979	-0.0306	PIS49564.1	GTR1	Putative GTP-binding protein; involved in activation of TOR1C during starvation response; transcript is upregulated in clinical isolates from HIV+ patients with oral candidiasis; (see Locus History Note for Assembly 19 correction)
PIS58032.1	24.3633751	23.0752258	23.3995099	23.73268774	21.64336873	25.37027596	0.973104754	0.979	-0.0306	PIS58032.1	orf19.1204	Phosphorylated protein of unknown function; transcript is upregulated clinical isolates from HIV positive patients with oral candidiasis
PIS56902.1	29.4216908	29.2721815	29.8225218	29.87489111	29.48229894	29.06781059	0.938679643	0.979	-0.0305	PIS56902.1	ERG11	Lanosterol 14-alpha-demethylase; cytochrome P450 family; role in ergosterol biosynthesis; target of azole antifungals; may contribute to drug resistance; azole or flow model biofilm induced; drug treated biofilm induced; hypoxia regulated
PIS52313.1	28.1193378	28.3954625	30.105376	28.85193921	28.48058659	29.19781218	0.974117208	0.979	-0.0299	PIS52313.1	CSI2	Putative 66S pre-ribosomal particle component; Hap43-induced; essential for growth; transposon mutation affects filamentous growth; Spider biofilm induced

PIS55725.1	23.414938	24.2182837	24.3283241	23.82578452	23.05965528	24.99011009	0.965647102	0.979	-0.0287	PIS55725.1	PGA45	Putative GPI-anchored cell wall protein; repressed in core caspofungin response; Hog1-induced; regulated by Ssn6; Mob2-dependent hyphal regulation; flow model biofilm induced
PIS51936.1	24.3189515	23.7728273	26.3362165	25.01956015	24.48858872	24.83481033	0.969836774	0.979	-0.0283	PIS51936.1	orf19.5869	Ortholog(s) have role in metal ion transport, protein targeting to vacuole, ubiquitin-dependent protein catabolic process and endoplasmic reticulum, fungal-type vacuole, fungal-type vacuole membrane localization
PIS52186.1	25.5514095	25.7911874	27.0023252	26.54070681	25.86252612	25.85914001	0.952201963	0.979	-0.0275	PIS52186.1	MP65	Cell surface mannoprotein; cell-wall glucan metabolism, adhesion; adhesin motif; O-glycosylation; induced by heat, germ tube formation, wall regeneration; mycelial antigen; diagnostic marker; fluconazole-repressed; Spider biofilm induced [illegible]
PIS55768.1	31.0526843	30.6773641	30.2049518	30.43205281	30.69967544	30.72064668	0.93407794	0.979	-0.0275	PIS55768.1	TPS3	Predicted trehalose-phosphate synthase regulatory subunit; regulated by Efg1; regulated by Tsa1, Tsa1B under H2O2 stress conditions; flow model biofilm induced (reports differ)
PIS48652.1	26.5098298	27.4407985	27.7037031	28.49112679	27.15523534	25.9287174	0.963668463	0.979	-0.0264	PIS48652.1	NMA111	Putative serine protease that promotes apoptosis and negatively regulates filamentation; null mutant shows increased virulence and hyperfilamentation; expression is induced by macrophages
PIS49729.1	27.6637199	27.5575833	27.1951328	27.83720186	27.46260169	27.0441384	0.950075634	0.979	-0.0242	PIS49729.1	orf19.6790	Ortholog(s) have mRNA binding activity, role in 3'-UTR-mediated mRNA destabilization, mitochondrion organization and P-body, cytoplasmic stress granule, perinuclear region of cytoplasm localization
PIS52422.1	33.6804068	33.8320102	33.5663173	33.04723885	33.72527433	34.23746087	0.943606508	0.979	-0.0229	PIS52422.1	MIR1	Putative mitochondrial phosphate transporter; caspofungin repressed; expression is increased in a fluconazole-resistant isolate; induced upon adherence to polystyrene

PIS49841.1	26.6044315	27.3863411	27.3034444	27.92234675	26.84770397	26.45680032	0.964292582	0.979	-0.0225	PIS49841.1	LIG1	tRNA ligase; functional homolog of S. cerevisiae Trl1
PIS55081.1	34.5547483	35.0056897	35.2640867	33.98998997	35.39936351	35.36946731	0.955282638	0.979	-0.0219	PIS55081.1	PDC11	Pyruvate decarboxylase; antigenic; on hyphal not yeast cell surface; Hap43, Gcn4, Efg1, Efh1, Hsf1 regulated; fluconazole, farnesol induced; amino acid starvation repressed; flow model biofilm induced; Spider biofilm repressed
PIS54593.1	25.1437226	25.2321359	25.8518595	25.47731555	25.74139684	24.94408068	0.96562131	0.979	-0.0216	PIS54593.1	HRT1	Ortholog of S. cerevisiae Hrt1; component of a nuclear ubiquitin-protein ligase complex involved in cell cycle control; induced by hydroxyurea; Spider biofilm induced
PIS58203.1	31.1307499	31.146781	30.1515946	30.23851432	31.1031998	31.02417508	0.957431268	0.979	-0.0211	PIS58203.1	CRM1	Functional homolog of S. cerevisiae Crm1, which acts in protein nuclear export; predicted to be resistant to antifungal drug leptomycin B; partially suppresses signal transduction defects of S. cerevisiae ste20 mutant
PIS50601.1	25.0122337	26.9163543	27.1931542	26.31140255	26.11273114	26.63805869	0.977182983	0.979	-0.0198	PIS50601.1	orf19.321	Ortholog(s) have L-methionine transmembrane transporter activity and role in methionine import across plasma membrane
PIS56655.1	28.0097976	28.0381267	28.3261579	27.84497314	28.67888221	27.79129894	0.953292187	0.979	-0.0196	PIS56655.1	orf19.1525	Ortholog(s) have role in nuclear-transcribed mRNA catabolic process, nonsense-mediated decay
PIS55524.1	33.2240679	32.4928136	31.8311555	31.95544684	32.89497103	32.63912945	0.96202288	0.979	-0.0195	PIS55524.1	ILS1	Putative isoleucyl-tRNA synthetase, the target of drugs including the cyclic beta-amino acid icofungipen/PLD-118/BAY-10-8888 and mupirocin; protein present in exponential and stationary growth phase yeast cultures

PIS55722.1	25.4893077	25.0274007	25.398679	25.10348335	25.46603057	25.28782568	0.968325802	0.979	-0.0193	PIS55722.1	BUD16	Putative pyridoxal kinase; a key enzyme in pyridoxal 5'-phosphate synthesis, the active form of vitamin B6; involved in bud-site selection and genome integrity in S. cerevisiae; induced by alpha pheromone in SpiderM medium
PIS52064.1	26.7163763	27.3036851	27.4710692	27.70591663	26.765489	26.96865618	0.967684111	0.979	-0.017	PIS52064.1	orf19.6250	Ortholog(s) have eukaryotic initiation factor 4G binding, mRNA binding activity, role in P-body assembly, negative regulation of translational initiation, stress granule assembly and P-body, cytoplasmic stress granule localization
PIS51139.1	29.926659	29.8291974	30.4101204	29.83389648	30.04294231	30.24321099	0.973876181	0.979	-0.0153	PIS51139.1	PDI1	Putative protein disulfide-isomerase; antigenic in human infection; soluble protein in hyphae; induced by filamentous growth; protein present in exponential and stationary growth phase yeast cultures; flow model biofilm repressed
PIS58864.1	29.4389825	29.631434	29.0372224	29.25600206	29.21977132	29.5862151	0.956794009	0.979	-0.0152	PIS58864.1	HGT19	Putative MFS glucose/myo-inositol transporter; 20 member family; 12 transmembrane segments, extended N terminus; expressed in rich medium; Hap43, phagocytosis, rat catheter, Spider and flow model biofilm induced
PIS48606.1	29.1889429	29.8073628	30.3377345	29.56223806	29.7440049	29.9827247	0.971374004	0.979	-0.015	PIS48606.1	WBP1	Putative oligosaccharyltransferase subunit; Spider biofilm repressed
PIS51699.1	28.9022865	28.929374	28.3055616	28.76489698	28.70692907	28.62199416	0.964591992	0.979	-0.0145	PIS51699.1	NMT1	Myristoyl-CoA:protein N-myristoyltransferase; attaches the fatty acid myristate to a small number of proteins at an N-terminal Gly; essential; antifungal drug target; functional homolog of S. cerevisiae Nmt1p
PIS48523.1	29.4296574	28.9952862	29.3427072	29.64002694	29.10652819	28.98094863	0.968611882	0.979	-0.0134	PIS48523.1	orf19.3792	Ortholog(s) have RNA binding, chromatin binding, mRNA binding activity

PIS51481.1	25.2367221	24.4961104	25.9564089	25.18731973	25.32399751	25.13821507	0.982217152	0.979	-0.0132	PIS51481.1	WSC4	Putative cell wall integrity and stress response subunit 4 precursor; transcription is specific to white cell type
PIS58379.1	24.0240093	24.6265297	25.2503163	24.8236864	24.02091745	25.01727819	0.975746442	0.979	-0.013	PIS58379.1	GWT1	Inositol acyltransferase with role in early steps of GPI anchor biosynthetic process; antifungal drug target
PIS49689.1	27.7645356	27.8706726	27.9684476	27.63924191	28.14892669	27.77821539	0.97417223	0.979	-0.0124	PIS49689.1	orf19.1305	Ortholog(s) have tRNA (guanine(37)-N1)-methyltransferase activity, role in mitochondrial tRNA methylation, tRNA N1-guanine methylation and cytoplasm, mitochondrial matrix localization
PIS50474.1	25.8421037	24.9717154	24.518934	23.87114068	25.42441276	26.00275235	0.984381493	0.979	-0.0115	PIS50474.1	orf19.3957	Ortholog(s) have GTP cyclohydrolase I activity, role in folic acid-containing compound biosynthetic process and nucleus localization
PIS48748.1	24.8046284	25.732692	26.7828322	26.83045704	25.02059413	25.43520563	0.988722391	0.979	-0.0113	PIS48748.1	DOS2	Protein of unknown function; induced during planktonic growth
PIS56914.1	27.1249971	27.3155472	27.7154599	28.02992133	27.71678517	26.37933249	0.981514122	0.979	-0.00999	PIS56914.1	orf19.3205	Mitochondrial ribosomal protein of the large subunit; rat catheter biofilm induced
PIS48305.1	27.0994132	28.1126165	29.6052402	27.3580705	28.65476134	28.77532342	0.989142471	0.98	-0.0097	PIS48305.1	orf19.3333	Ortholog(s) have 5S rRNA binding, 7S RNA binding, poly(A) binding, ribonuclease P RNA binding, tRNA binding activity

PIS58328.1	30.0107043	29.9264975	30.1274252	30.00136156	30.46531334	29.57556143	0.988901218	0.979	-0.00746	PIS58328.1	CBK1	Ser/Thr kinase of cell wall integrity pathway; mutants show abnormal morphology and aggregation; Mob2p associated; required for wild-type hyphal growth and transcriptional regulation of cell-wall-associated genes
PIS51658.1	29.0843211	28.5539947	27.6746064	27.89501178	28.17934049	29.21649484	0.992776957	0.98	-0.00736	PIS51658.1	orf19.2985	Protein of unknown function; Hap43-repressed gene
PIS58269.1	25.5110614	26.2653383	27.1976046	24.89060503	26.89434465	27.16888576	0.991226393	0.98	-0.00672	PIS58269.1	orf19.4601	Putative RNA polymerase III transcription initiation factor complex (TFIIIC) subunit; possibly an essential gene, disruptants not obtained by UAU1 method
PIS51621.1	28.0029609	28.7078323	27.5692019	28.09218798	28.20117033	27.97685915	0.997169511	0.98	-0.00326	PIS51621.1	OFD1	Putative prolyl hydroxylase family member; regulates Ume6p stability in response to oxygen; inhibited by hypoxia; repressed by prostaglandins; Spider biofilm induced
PIS56543.1	25.8927181	24.7514376	26.0355696	25.94102843	25.11941855	25.61024433	0.994957983	0.98	-0.00301	PIS56543.1	WSC2	Putative cell wall integrity and stress response protein; mRNA binds She3; Spider biofilm induced
PIS54977.1	30.3968261	28.5956986	29.3610625	29.59487673	29.86273025	28.89831565	0.998576528	0.98	0.000778	PIS54977.1	GDA1	Golgi membrane GDPase/UDPase, required for wild-type O-mannosylation, not N-glycosylation; required for wild-type hyphal induction, cell wall, and cell surface charge; functional homolog of S. cerevisiae Gda1
PIS52031.1	30.1118259	30.2267197	30.030726	30.10963136	30.24735499	30.01474643	0.997683677	0.98	0.00082	PIS52031.1	orf19.1564	Plasma membrane-localized protein of unknown function

PIS55569.1	25.0930081	26.1106465	26.2011185	26.41195091	25.91546828	25.0829886	0.996695445	0.98	0.00188	PIS55569.1	GAP2	General broad specificity amino acid permease; ketoconazole, flucytosine repressed; Ssy1-dependent histidine induction; regulated by Nrg1, Tup1; colony morphology-related gene regulation by Ssn6; Spider and flow model biofilm induced
PIS51078.1	24.7593738	25.7818835	24.7691425	25.70736913	23.99058662	25.61862604	0.997811085	0.98	0.00206	PIS51078.1	NOT4	Putative E3 ubiquitin-protein ligase; required for maintenance, but not induction, of hyphal development; homozygous null mutant is avirulent in mouse systemic infection despite persistence in host; repressed in rat oral candidiasis
PIS48723.1	22.3553987	23.2515827	24.5527662	23.2240647	23.38763781	23.55459028	0.997994617	0.98	0.00218	PIS48723.1	orf19.6563.1	Ortholog(s) have role in mitochondrial cytochrome c oxidase assembly, negative regulation of mitochondrial translation and mitochondrial inner membrane localization
PIS51095.1	31.4558656	31.3205662	30.2394026	30.22515718	31.19700024	31.60150475	0.99604345	0.98	0.00261	PIS51095.1	orf19.7511	Ortholog(s) have structural constituent of nuclear pore activity, role in nuclear pore organization, regulation of nucleocytoplasmic transport and nuclear periphery, nuclear pore, nuclear pore inner ring localization
PIS58126.1	28.0231699	27.361673	28.5084521	29.07845458	28.12708061	26.70158475	0.994939765	0.98	0.00461	PIS58126.1	ECM33	GPI-anchored cell wall protein; mutants show cell-wall defects and reduced adhesion, host cell damage, and endocytosis; mutant infection is immunoprotective in murine model; fluconazole-induced; caspofungin repressed
PIS52273.1	30.2833758	29.5332744	30.4373946	28.96037082	30.25886038	31.04913296	0.992841269	0.98	0.00477	PIS52273.1	orf19.3610	Protein of unknown function; upregulation correlates with clinical development of fluconazole resistance; regulated by Sef1, Sfu1, and Hap43
PIS56562.1	26.2023649	23.2910394	25.0028898	25.00132347	24.94527628	24.57549292	0.994793667	0.98	0.0086	PIS56562.1	SEC13	Putative protein transport factor; antigenic during murine systemic infection; macrophage-downregulated protein; protein level decreases in stationary phase cultures; Spider biofilm repressed

PIS50307.1	25.2090197	25.0895554	24.0647678	25.41017136	24.58522142	24.4012529	0.985499559	0.979	0.0111	PIS50307.1	orf19.7645	Biotin protein ligase; catalyzes covalent attachment of biotin to biotin-dependent enzymes including acetyl CoA carboxylase and pyruvate carboxylase; transcription regulated by biotin availability and Vhr1p
PIS52345.1	29.2804765	29.8810531	28.7945469	29.57044101	29.28486107	29.13431605	0.971914703	0.979	0.0112	PIS52345.1	SLC1	Putative fatty acyltransferase; protein repressed during the mating process
PIS58901.1	28.1068283	28.9921406	27.9956794	28.71282721	28.38603985	28.03136698	0.978001216	0.979	0.0119	PIS58901.1	orf19.2414	Ortholog of S. cerevisiae Mpm1; a mitochondrial intermembrane space protein of unknown function; Hap43-repressed; Spider biofilm induced
PIS58950.1	24.737066	26.6495039	25.5554113	25.44034873	26.16498687	25.37629467	0.982472502	0.979	0.0132	PIS58950.1	MET13	Putative methionine biosynthesis protein; ketoconazole-induced; amphotericin B repressed; Spider biofilm repressed
PIS51351.1	23.8449841	23.2005912	23.0686637	23.74352956	22.7812883	23.63456362	0.983049021	0.979	0.015	PIS51351.1	YBL053	Putative subunit of a replication fork-pausing checkpoint complex
PIS48688.1	29.2974323	29.9582323	29.1099526	29.28383468	29.6894275	29.43997514	0.964295238	0.979	0.0159	PIS48688.1	orf19.3430	Plasma membrane-associated protein; physically interacts with TAP-tagged Nop1p
PIS51813.1	29.1886895	29.1873116	28.7933151	28.88868632	29.12926364	29.19952164	0.964743876	0.979	0.0161	PIS51813.1	TAF12L	Non-essential component of the SAGA complex, involved in transcription regulation

PIS58819.1	26.1397073	26.4513415	26.2169692	26.27793587	26.97961706	25.60227813	0.97393202	0.979	0.0173	PIS58819.1	orf19.394	Putative kynureninase; predicted role in NAD biosynthesis; Hap43-repressed gene; flow model biofilm induced
PIS58758.1	25.8784729	26.0400299	25.7260517	26.10670397	25.71746861	25.87793904	0.962856522	0.979	0.0192	PIS58758.1	orf19.1643	Ortholog(s) have serine-type endopeptidase activity, role in regulation of mitochondrion organization, signal peptide processing and mitochondrial inner membrane localization
PIS51343.1	33.7818205	33.7368708	33.7568072	34.05135929	33.33806832	33.94584904	0.939602165	0.979	0.0199	PIS51343.1	orf19.4149.1	Protein component of the small (40S) ribosomal subunit; Spider biofilm repressed
PIS55549.1	24.7259552	24.0428722	25.4693736	24.90315353	24.47134046	24.92547611	0.960973879	0.979	0.0206	PIS55549.1	CKB1	Regulatory subunit of protein kinase CK2 (casein kinase II), beta subunit; null mutants are hypersensitive to caspofungin and hydrogen peroxide medium
PIS58665.1	25.7755075	25.8187102	25.8113525	25.56278837	25.70889703	26.20167239	0.982892301	0.979	0.0226	PIS58665.1	orf19.6432	Ortholog(s) have ATPase activity, role in response to xenobiotic stimulus, ribosomal large subunit biogenesis and preribosome, large subunit precursor localization
PIS48749.1	28.0355888	27.5491087	27.9614834	27.54499826	27.95445488	28.1175194	0.964187917	0.979	0.0236	PIS48749.1	MODF	Has domain(s) with predicted ATP binding, ATPase activity
PIS54688.1	24.6173859	24.9145325	24.9183814	25.43097436	24.79256697	24.29895472	0.968112539	0.979	0.0241	PIS54688.1	orf19.3556	Transportin or cytosolic karyopherin beta; Spider biofilm induced

PIS48668.1	27.7501469	27.2315063	29.0636135	27.57307741	28.22899334	28.31742269	0.955276415	0.979	0.0247	PIS48668.1	COQ3	Protein with a predicted role in coenzyme Q biosynthesis; transcriptionally induced by interaction with macrophages; possibly an essential gene, disruptants not obtained by UAU1 method
PIS49662.1	31.5779496	31.6100945	31.0819066	31.51898709	31.50940816	31.31590589	0.927659515	0.979	0.0248	PIS49662.1	SEC26	Secretory vesicles coatomer complex protein
PIS48685.1	31.7127796	31.4060945	30.3005307	30.61870196	31.57737468	31.30769068	0.9574798	0.979	0.0281	PIS48685.1	ANB1	Translation initiation factor eIF-5A; repressed in hyphae vs yeast cells; downregulated upon phagocytosis by murine macrophage; Hap43-induced; GlcNAc-induced protein; Spider biofilm repressed
PIS58645.1	33.7289709	33.8645857	33.1296715	34.22491772	33.09210221	33.49370668	0.939586586	0.979	0.0292	PIS58645.1	RPS19A	Putative ribosomal protein S19; protein level decreases in stationary phase cultures; Spider biofilm repressed
PIS51440.1	27.5090997	26.3017365	26.9915736	27.26292517	26.76190923	26.8653241	0.946584684	0.979	0.0292	PIS51440.1	UTP15	Small subunit (SSU) processome component; mutation confers resistance to 5-fluorocytosine (5-FC); physically interacts with TAP-tagged Nop1p
PIS54724.1	23.6569199	25.223052	23.9444543	24.10642777	23.28712877	25.52108385	0.970230544	0.979	0.0301	PIS54724.1	orf19.6357	Ortholog(s) have kinetochore adaptor activity, protein-containing complex binding activity
PIS52329.1	24.2787502	24.5925011	24.994602	25.04002366	24.60291854	24.31624631	0.953620262	0.979	0.0311	PIS52329.1	RBK1	Has domain(s) with predicted kinase activity, ribokinase activity and role in D-ribose metabolic process

PIS48395.1	24.8386265	24.8441115	24.3485363	23.89335107	25.31277285	24.91943451	0.960824937	0.979	0.0314	PIS48395.1	orf19.2487	Protein with a predicted phosphoribulokinase/uridine kinase domain; Spider biofilm induced
PIS58302.1	24.3325653	23.9059865	24.2357471	24.40428434	23.80731229	24.35814083	0.941721732	0.979	0.0318	PIS58302.1	orf19.4550	Predicted MFS membrane transporter, member of the drug:proton antiporter (12 spanner) (DHA1) family; flow model biofilm induced
PIS48417.1	29.5652694	29.5891522	29.5740317	29.45070502	29.73097462	29.6458323	0.906311444	0.979	0.033	PIS48417.1	orf19.1840	Tricalbin-family endoplasmic reticulum-plasma membrane tethering protein; required for transport of cell wall proteins; involved in stress responses, including sensitivity to caspofungin; Spider biofilm induced
PIS51951.1	29.7927369	29.8581109	30.211998	29.94796715	29.76484232	30.24996574	0.917023942	0.979	0.0333	PIS51951.1	orf19.989	Mitochondrial ribosomal protein of the small subunit; Spider biofilm repressed
PIS56793.1	30.3299969	30.2600487	30.0209439	29.64131156	30.83055301	30.24063708	0.923514425	0.979	0.0338	PIS56793.1	RPT1	Putative 26S proteasome regulatory subunit 7; Hap43p-repressed gene; regulated by Gcn2p and Gcn4p; overlaps orf19.442
PIS52473.1	25.0117762	25.5843747	25.4369917	25.65197512	25.42212953	25.06262031	0.932927631	0.979	0.0345	PIS52473.1	orf19.2822	Essential component of the conserved oligomeric Golgi complex; role in fusion of transport vesicles to Golgi compartments; rat catheter biofilm repressed
PIS51848.1	29.6915263	30.1967585	30.5593101	30.27667562	30.23057002	30.04441987	0.900990422	0.979	0.0347	PIS51848.1	GPD1	Glycerol-3-phosphate dehydrogenase; glycerol biosynthesis; regulated by Efg1; regulated by Tsa1, Tsa1B under H2O2 stress conditions; Sflow model and Spider biofilm induced

PIS55774.1	25.5935271	24.0116596	25.0727778	24.65150155	25.18760388	24.94603088	0.939533538	0.979	0.0357	PIS55774.1	orf19.6751	Ortholog(s) have tRNA (cytidine(32)-2'-O)-methyltransferase activity, tRNA (guanine(34)-2'-O)-methyltransferase activity, tRNA 2'-O-methyltransferase activity
PIS56721.1	28.4629742	28.8336451	27.8383291	28.71311986	28.45198754	28.07714761	0.930061555	0.979	0.0358	PIS56721.1	orf19.7662	Ortholog(s) have RNA polymerase II C-terminal domain phosphoserine binding activity, role in mRNA 3'-end processing, retrotransposon silencing and chromatin, site of double-strand break localization
PIS52174.1	25.4748629	26.2657934	27.3969906	26.85869748	25.98410184	26.40379925	0.953342863	0.979	0.0363	PIS52174.1	PUP3	Putative beta 3 subunit of the 20S proteasome; macrophage/pseudohyphal-repressed
PIS55799.1	30.9961262	31.0089592	31.726441	30.52647858	31.54353549	31.77321971	0.931157753	0.979	0.0372	PIS55799.1	AFG3	Similar to S. cerevisiae Afg3p, a subunit of the mitochondrial inner membrane m-AAA protease; likely to be essential for growth, based on an insertional mutagenesis strategy
PIS58936.1	30.4871467	30.87531	31.22931	30.84094988	30.89395587	30.96878293	0.900775498	0.979	0.0373	PIS58936.1	orf19.7067	Ortholog(s) have RNA polymerase II C-terminal domain phosphoserine binding, RNA polymerase II complex binding and RNA polymerase II-specific DNA-binding transcription factor binding, more
PIS55029.1	25.5609876	26.4844996	25.8651483	25.84182624	26.03601783	26.14545547	0.940778377	0.979	0.0376	PIS55029.1	TLG2	Putative syntaxin-like t-SNARE; macrophage/pseudohyphal-repressed
PIS58961.1	28.7290855	25.9628677	28.1145601	27.50896056	27.39587025	28.01608634	0.967275331	0.979	0.0381	PIS58961.1	orf19.1961	Planktonic growth-induced gene

PIS58355.1	23.9034491	24.0696184	24.3385229	25.02070966	23.63889902	23.77193755	0.951698384	0.979	0.04	PIS58355.1	RTT109	Histone acetyltransferase, mutants are sensitive to DNA damage, show decreased virulence in mice, decreased white-to-opaque switching and increased susceptibility to killing by macrophages
PIS48450.1	27.4937737	26.6633246	26.2494813	26.19250188	27.36065709	26.97364855	0.960360821	0.979	0.0401	PIS48450.1	orf19.1043	Ortholog(s) have lysophosphatidic acid acyltransferase activity, role in intracellular triglyceride homeostasis, lipid droplet organization and endoplasmic reticulum, lipid droplet localization
PIS54694.1	28.3241684	29.1383598	28.9379529	28.7641789	28.67191781	29.08526688	0.915416434	0.979	0.0403	PIS54694.1	orf19.2520	Ortholog(s) have structural constituent of ribosome activity and mitochondrial small ribosomal subunit localization
PIS56737.1	28.101469	27.843893	29.4716361	28.33243383	28.55776577	28.65300498	0.925098203	0.979	0.0421	PIS56737.1	LEU4	Putative 2-isopropylmalate synthase; involved in resistance to caspofungin and anidulafungin; regulated by NRG1, MIG1, TUP1, GCN4; induced by human whole blood or PMNs; macrophage/pseudohyphal-repressed after 16h; Spider biofilm repressed
PIS49526.1	20.7456685	22.5836744	23.0946474	24.65811386	20.59540262	21.29857491	0.962202173	0.979	0.0427	PIS49526.1	KEX2	Subtilisin-like protease (proprotein convertase); processes aspartyl proteinase Sap2; required for hyphal growth and wild-type virulence in mice; required for maturation of candidalysin Ece1p
PIS48761.1	27.9373167	28.4634118	28.5025056	28.35600057	28.55037181	28.12551193	0.935939149	0.979	0.0429	PIS48761.1	PEX1	Peroxisomal ATPase involved in import of peroxisomal matrix proteins; involved in oxidative stress tolerance
PIS56805.1	29.2715592	30.0905755	29.3700174	29.98043458	29.39147771	29.49006669	0.901117166	0.979	0.0433	PIS56805.1	orf19.7270	Protein of unknown function; Spider biofilm repressed

PIS50604.1	30.7390573	30.6924195	30.8845868	31.36144649	30.77624165	30.30877839	0.914400127	0.979	0.0435	PIS50604.1	SBP1	Similar to RNA binding proteins; downregulated upon adherence to polystyrene; stationary-phase enriched protein
PIS58151.1	28.8348456	27.7782396	26.173572	28.23083006	27.96435358	26.72499943	0.956280172	0.979	0.0445	PIS58151.1	orf19.3021	Putative protein of unknown function; Hap43-repressed gene; Spider biofilm induced
PIS51390.1	30.4283246	30.0416112	30.2172329	30.48762731	30.41070327	29.9234225	0.890245827	0.978	0.0449	PIS51390.1	orf19.3293	Putative enoyl reductase involved in very long chain fatty acid elongation; possibly an essential gene, disruptants not obtained by UAU1 method
PIS49703.1	25.0848898	26.4519452	25.5747006	25.74599672	25.65218023	25.84828816	0.922228387	0.979	0.045	PIS49703.1	MSK1	Putative mitochondrial lysine-tRNA synthetase; flucytosine repressed
PIS56585.1	30.0257463	30.1999945	30.2542898	30.61126591	29.94309137	30.0621966	0.881750183	0.978	0.0455	PIS56585.1	ALA1	Alanyl-tRNA synthetase; translational regulation generates cytoplasmic and mitochondrial forms; Gcn4p-regulated; repressed by amino acid starvation (3-AT); translation-related genes downregulated upon phagocytosis by murine macrophages
PIS55675.1	28.6684442	28.1255566	28.6728431	28.8090003	28.91605421	27.88151437	0.914253931	0.979	0.0466	PIS55675.1	orf19.5459	mRNA polyadenylation regulating protein; Hap43-repressed; transcript is upregulated in RHE model of oral candidiasis and in clinical isolates from HIV+ patients with oral candidiasis; Spider biofilm repressed
PIS56648.1	28.2989841	28.365136	27.6238156	28.45622267	28.01377033	27.95873611	0.889157938	0.978	0.0469	PIS56648.1	orf19.1533	Possible vacuolar protein; Hap43-induced gene

PIS58774.1	27.4997234	28.227103	28.212886	28.43236768	27.64808057	28.00064854	0.886209952	0.978	0.0471	PIS58774.1	orf19.7078	Ortholog of S. cerevisiae : YCL012C, C. dubliniensis CD36 : Cd36_65270, C. parapsilosis CDC317 : CPAR2_805430, C. auris B8441 : B9J08_000227 and Candida tenuis NRRL Y-1498 : CANTEDRAFT_96597
PIS56875.1	27.4587179	27.7548701	27.3263381	27.95784542	27.50644063	27.2195877	0.882143488	0.978	0.048	PIS56875.1	IDI1	Ortholog(s) have isopentenyl-diphosphate delta-isomerase activity and role in ergosterol biosynthetic process, farnesyl diphosphate biosynthetic process
PIS51107.1	23.873083	24.3143826	25.084575	24.66951012	23.42757043	25.32091417	0.973954389	0.979	0.0487	PIS51107.1	EPL1	Subunit of the NuA4 histone acetyltransferase complex
PIS52016.1	31.5462478	32.0560118	32.1415185	31.21279264	32.39624718	32.28202516	0.912401204	0.979	0.0491	PIS52016.1	HSP21	Small heat shock protein; role in stress response and virulence; fluconazole-downregulated; induced in cyr1 or ras1 mutant; stationary phase enriched protein; detected in some, not all, biofilm extracts; Spider biofilm induced
PIS56864.1	28.485133	27.7870634	27.9368228	28.51555902	27.79153787	28.04937498	0.874978772	0.978	0.0492	PIS56864.1	orf19.2760	Putative mRNA cleavage and polyadenylation factor; heterozygous null mutant exhibits hypersensitivity to parnafungin and cordycepin in the C. albicans fitness test
PIS58528.1	23.1428545	23.3862403	22.6642212	23.84644761	22.32016268	23.17619119	0.935163896	0.979	0.0498	PIS58528.1	orf19.6456	Ortholog of C. dubliniensis CD36 : Cd36_72280, C. parapsilosis CDC317 : CPAR2_703040, C. auris B8441 : B9J08_001028 and Candida tenuis NRRL Y-1498 : CANTEDRAFT_125842
PIS55552.1	27.5911033	28.2369653	27.6421223	27.86056627	27.72579744	28.04099302	0.943329119	0.979	0.0524	PIS55552.1	orf19.2835	Ortholog(s) have SUMO activating enzyme activity, role in mitotic chromosome condensation, protein sumoylation and SUMO activating enzyme complex, cytosol, nucleus localization

PIS51539.1	25.2630462	24.1365015	23.3366018	24.42139895	24.54360369	23.92879686	0.943736529	0.979	0.0526	PIS51539.1	SNU114	Protein similar to S. cerevisiae Snu114p, which is an RNA helicase involved in pre-mRNA splicing; likely to be essential for growth, based on an insertional mutagenesis strategy
PIS51144.1	32.197986	32.3363499	32.7843955	32.59084159	32.05417679	32.83267685	0.86414209	0.978	0.053	PIS51144.1	RPL24A	Predicted ribosomal protein; downregulated upon phagocytosis by murine macrophage; intron in 5'-UTR; Hap43-induced; Spider biofilm repressed
PIS58429.1	26.4199334	25.7253879	26.3188406	26.00656776	26.19618673	26.42263049	0.865467587	0.978	0.0537	PIS58429.1	orf19.2847	Ortholog(s) have RNA polymerase III activity, role in tRNA transcription by RNA polymerase III and RNA polymerase III complex localization
PIS48236.1	30.472932	30.5387679	30.689108	30.70931393	30.41932356	30.73525012	0.840733402	0.978	0.0544	PIS48236.1	SUI3	Putative translation initiation factor; genes encoding ribosomal subunits, translation factors, and tRNA synthetases are downregulated upon phagocytosis by murine macrophage
PIS58065.1	27.6231546	28.0670338	27.6263762	27.08606929	28.33251185	28.0633252	0.93912863	0.979	0.0551	PIS58065.1	orf19.3458	Ortholog(s) have role in late endosome to vacuole transport via multivesicular body sorting pathway and Vps55/Vps68 complex, fungal-type vacuole membrane localization
PIS58128.1	30.4602233	30.2761774	30.7259128	30.92762341	30.5295719	30.1706063	0.840933488	0.978	0.0552	PIS58128.1	GGA2	Protein involved in Golgi trafficking; rat catheter and Spider biofilm repressed
PIS51284.1	23.8390058	23.232334	23.932089	24.32482258	24.0255208	22.8188509	0.956745803	0.979	0.0553	PIS51284.1	SWD1	Ortholog(s) have histone H3K4 methyltransferase activity, role in regulation of DNA-templated transcription, subtelomeric heterochromatin formation, telomere maintenance and Set1C/COMPASS complex localization

PIS58609.1	30.5034028	29.9011633	30.3726919	31.64741322	29.70061843	29.59814977	0.921050578	0.979	0.0563	PIS58609.1	HOM6	Putative homoserine dehydrogenase; Gcn4-regulated; induced by amino acid starvation (3-ATtreatment); macrophage-induced protein; protein level decreases in stationary phase cultures; flow model biofilm repressed
PIS50507.1	23.0808576	23.9194935	20.7086756	23.24358407	21.12141912	23.5176835	0.973029003	0.979	0.0579	PIS50507.1	GRF10	Putative homeodomain transcription factor, involved in copper homeostasis and control of filamentous growth; null mutant is an adenine auxotroph and shows increased copper resistance; promoter bound by Bcr1, Tec1, Efg1, Ndt80 and Brg1
PIS48741.1	27.713577	28.5862488	28.1666572	28.53731507	27.82959402	28.27420294	0.922217888	0.979	0.0582	PIS48741.1	orf19.7269	Putative polyamine acetyltransferase; acetylates polyamines (e.g. putrescine, spermidine, spermine) and aralkylamines (e.g. tryptamine, phenylethylamine); Spider biofilm repressed
PIS49561.1	27.0983166	25.9926068	27.6614359	26.42159356	27.34278783	27.16569098	0.906364534	0.979	0.0592	PIS49561.1	orf19.3613	Ortholog(s) have RNA polymerase II C-terminal domain phosphoserine binding, RNA polymerase II complex binding, chromatin binding activity
PIS48820.1	28.0340211	28.0950767	27.5604338	27.23919366	28.34658643	28.28120385	0.884290223	0.978	0.0592	PIS48820.1	orf19.4811	Putative tricarboxylate carrier family protein; localized to the mitochondrial membrane
PIS52255.1	24.6828985	25.9180676	26.8684707	25.67532529	25.9944834	25.97859222	0.913288509	0.979	0.0597	PIS52255.1	CCS1	Copper chaperone involved in activation and protection of superoxide dismutase Sod1p
PIS58629.1	28.7951232	28.2152041	28.7658069	28.21316734	28.85376855	28.88856493	0.894469762	0.978	0.0598	PIS58629.1	IMH3	Inosine monophosphate (IMP) dehydrogenase; enzyme of GMP biosynthesis; target of mycophenolic acid and mizoribine monophosphate; antigenic during infection; repressed in core stress response; snoRNA snR54 encoded within IMH3 intron

PIS49556.1	26.5202109	26.1762887	26.8269748	26.44470344	26.07741205	27.18543634	0.90808387	0.979	0.0614	PIS49556.1	orf19.2246	Ortholog(s) have role in nuclear-transcribed mRNA catabolic process, non-stop decay
PIS55831.1	28.3015181	28.0823846	28.4618204	27.56158266	28.78182149	28.68783795	0.891875291	0.978	0.0618	PIS55831.1	NIT3	Putative nitrilase; regulated by Gcn2p and Gcn4p; protein present in exponential and stationary growth phase yeast cultures
PIS58652.1	29.905009	30.2814744	30.5452974	29.31107465	30.60000079	31.00691001	0.908615656	0.979	0.0621	PIS58652.1	VPS21	Late endosomal Rab small monomeric GTPase involved in transport of endocytosed proteins to the vacuole; involved in filamentous growth and virulence; Spider biofilm induced
PIS51978.1	31.3150815	30.7210735	30.9067499	31.31287666	30.96240654	30.85495903	0.840883867	0.978	0.0624	PIS51978.1	GCN1	Ortholog(s) have protein kinase activator activity, protein kinase regulator activity, stalled ribosome sensor activity
PIS51100.1	27.6122572	27.5686801	27.6007858	27.40053044	27.65421499	27.91580193	0.902440972	0.979	0.0629	PIS51100.1	RRD1	Putative peptidyl-prolyl cis/trans-isomerase; caspofungin induced
PIS50593.1	24.1256803	24.8958434	23.9944977	24.43846501	24.5765503	24.18959808	0.872139898	0.978	0.0629	PIS50593.1	ZCF21	Predicted Zn(II)2Cys6 transcription factor; mutants display increased colonization of mouse kidneys; Spider biofilm induced
PIS54490.1	28.552893	27.8643325	27.7213516	27.57177452	28.66590347	28.08997793	0.924219669	0.979	0.063	PIS54490.1	CEM1	Protein similar to S. cerevisiae Cem1p, an acyl carrier protein involved in fatty acid biosynthesis; likely to be essential for growth, based on an insertional mutagenesis strategy

PIS54911.1	29.5302827	29.6531989	29.3364866	29.87190124	29.47925632	29.35843892	0.863642225	0.978	0.0632	PIS54911.1	SNF1	Functional homolog of S. cerevisiae Snf1p, which regulates sugar metabolism; constitutively expressed; Thr208 phosphorylation may have regulatory role; up-regulation associated with azole resistance; essential gene in some experiments
PIS51629.1	24.3076918	24.5072479	25.8028754	24.96913543	23.85031175	25.99301428	0.931286593	0.979	0.0649	PIS51629.1	orf19.1034	Protein with a predicted cytochrome b5-like Heme/Steroid binding domain; Hap43, caspofungin repressed; flow model biofilm induced
PIS58772.1	24.5241539	25.1264619	25.2447706	26.4702214	25.3079293	23.31267724	0.918560422	0.979	0.0651	PIS58772.1	orf19.7074	Ortholog(s) have methylated histone binding activity and role in SAGA complex localization to transcription regulatory region, positive regulation of transcription by RNA polymerase II, protein localization
PIS51868.1	23.8064123	24.7734731	24.8494089	25.13294052	24.57308405	23.91878795	0.893088785	0.978	0.0652	PIS51868.1	GSG1	Putative subunit of the TRAPP complex; involved in targeting of ER-to-Golgi transport vesicles; flow model biofilm induced
PIS52065.1	29.6284496	29.431761	29.8513934	30.27888594	29.21547917	29.61414308	0.889737617	0.978	0.0656	PIS52065.1	orf19.6252	Ortholog(s) have RNA polymerase II complex binding, RNA polymerase II-specific DNA-binding transcription factor binding, chromatin binding activity
PIS55464.1	25.664373	24.8675366	25.538515	25.6714024	25.37460604	25.22413387	0.883196484	0.978	0.0666	PIS55464.1	orf19.6528	Has domain(s) with predicted role in signal transduction and Ragulator complex localization
PIS52013.1	29.3140341	29.3824064	29.8698667	29.57060555	29.66653809	29.53237752	0.816127249	0.977	0.0677	PIS52013.1	DCK1	Putative guanine nucleotide exchange factor; required for embedded filamentous growth; activates Rac1; has a DOCKER domain; similar to adjacent DCK2 and to S. cerevisiae Ylr422wp; regulated by Nrg1; Spider biofilm induced

PIS58839.1	25.3907209	25.2983746	26.0670655	25.62034011	25.35632458	25.98300487	0.881649301	0.978	0.0678	PIS58839.1	orf19.397	Ortholog(s) have structural constituent of ribosome activity and mitochondrial large ribosomal subunit localization
PIS48420.1	23.3178171	24.0778628	24.1743776	24.06841786	23.73403381	23.97165577	0.899449479	0.978	0.068	PIS48420.1	orf19.5846	Putative TFIIH and nucleotide excision repair factor 3 complex subunit; possibly an essential gene, disruptants not obtained by UAU1 method
PIS55724.1	24.9172884	23.3978083	23.5325788	24.39186347	24.08480058	23.57564683	0.914537002	0.979	0.0682	PIS55724.1	orf19.2447	Putative Type II phosphatidylinositol 4-kinase; Ssr1-repressed; flow model biofilm repressed
PIS51494.1	25.9635727	25.3214514	26.166927	25.53638787	25.97200505	26.1538516	0.82911049	0.978	0.0701	PIS51494.1	orf19.5835	Ortholog(s) have rRNA binding activity, role in rRNA processing, ribosomal small subunit assembly and CURI complex, UTP-C complex localization
PIS54974.1	28.6377811	28.2485965	27.4297171	27.26838846	28.63728558	28.62133629	0.882721381	0.978	0.0703	PIS54974.1	OCH1	Alpha-1,6-mannosyltransferase; initiates N-glycan outer chain branch addition; similar to S. cerevisiae Och1p; required for wild-type virulence in mouse intravenous infection; fungal-specific (no human or murine homolog)
PIS51482.1	25.3603922	25.0079114	24.0543903	25.99558261	24.91878905	23.72031248	0.924586851	0.979	0.0707	PIS51482.1	CTF5	Predicted component of the kinetochore sub-complex COMA; induced during the mating process; repressed by alpha pheromone in SpiderM medium
PIS58567.1	27.2090318	28.2232851	28.7084861	28.12774697	28.08779679	28.13797346	0.84353734	0.978	0.0709	PIS58567.1	ILV3	Dihydroxyacid dehydratase; repressed by nitric oxide; macrophage-induced protein; protein in exponential and stationary growth phase; Sef1, Sfu1p, Hap43-regulated; farnesol-repressed; flow model biofilm induced; Spider biofilm repressed

PIS58514.1	27.0177362	28.2974099	27.9871544	27.65413376	28.17357433	27.68980302	0.842815768	0.978	0.0717	PIS58514.1	TIM23	Protein involved in mitochondrial matrix protein import
PIS56732.1	23.5735673	25.3959443	24.7039608	24.22723035	24.59509726	25.07094651	0.901759097	0.979	0.0733	PIS56732.1	TRP1	Phosphoribosylanthranilate isomerase; tryptophan biosynthesis; expected unifunctional, unlike trifunctional enzyme of some other fungi; complements E. coli trpC, S. cerevisiae trp1 mutant; CCT1 and TRP1 overlap; Spider biofilm
PIS58119.1	21.9056541	22.0220175	22.9920336	24.49510915	18.47964161	24.17081075	0.954564018	0.979	0.0753	PIS58119.1	orf19.6263	Predicted MFS membrane transporter; member of the monocarboxylate porter (MCP) family; Spider biofilm induced
PIS55072.1	25.8808143	26.0326376	26.3192616	26.50103657	25.59111767	26.36905221	0.871222215	0.978	0.0762	PIS55072.1	orf19.2621	Ortholog(s) have U1 snRNP, U2 snRNP, U2-type prespliceosome, U4/U6 x U5 tri-snRNP complex, U5 snRNP, post-mRNA release spliceosomal complex, spliceosomal complex localization
PIS51700.1	24.8510059	23.813704	22.8565511	25.6585797	23.96984082	22.12408034	0.924961226	0.979	0.0771	PIS51700.1	orf19.4642	Protein of unknown function; Hap43-induced gene
PIS58887.1	27.3504788	27.3352177	28.7792652	27.49688498	28.18549512	28.01606888	0.890876479	0.978	0.0778	PIS58887.1	ERV1	Predicted component of the mitochondrial intermembrane space (IMS), involved in protein import into mitochondrial intermembrane space
PIS55016.1	31.5419507	30.9668266	30.5661071	31.4640961	30.97412308	30.87548587	0.863646669	0.978	0.0796	PIS55016.1	orf19.1183	Ortholog of S. cerevisiae : YNL115C, C. glabrata CBS138 : CAGL0L03938g, C. dubliniensis CD36 : Cd36_60200, C. parapsilosis CDC317 : CPAR2_603320 and C. auris B8441 : B9J08_002166

PIS48825.1	26.3527268	26.1366972	27.0184486	26.63315815	26.35963153	26.75517448	0.867664673	0.978	0.08	PIS48825.1	RAM2	Alpha subunit of heterodimeric protein geranylgeranyltransferase type I and farnesyltransferase; a-specific transcript; lovastatin, fluconazole regulated; GGTase I binds zinc, is Mg-dependent; Cdc42 substrate; rat catheter biofilm repressed
PIS51399.1	22.8054948	23.4479838	23.1324894	23.20125889	23.55302427	22.87228431	0.917859734	0.979	0.0802	PIS51399.1	orf19.2387	Putative tRNA-Pro synthetase; genes encoding ribosomal subunits, translation factors, and tRNA synthetases are downregulated upon phagocytosis by murine macrophage
PIS54852.1	25.8194533	25.1711981	25.3160474	24.38363779	26.13501966	26.03214053	0.941826553	0.979	0.0814	PIS54852.1	orf19.4478	Ortholog(s) have aspartate-tRNA ligase activity, role in mitochondrial aspartyl-tRNA aminoacylation and mitochondrion localization
PIS48232.1	21.2102193	22.5863591	22.8248563	21.82413285	22.06760521	22.97512444	0.89909739	0.978	0.0818	PIS48232.1	RAD14	Putative DNA repair protein; transcription is regulated upon yeast-hyphal switch; flucytosine repressed
PIS52207.1	27.2431911	28.3537183	28.043343	27.17003345	28.29143241	28.4258352	0.855093582	0.978	0.0823	PIS52207.1	orf19.5406	Predicted plasma membrane associated protein phosphatase; required for normal filamentous growth; mRNA binds She3 and is localized to hyphal tips
PIS58896.1	23.2677821	23.3948828	26.398814	24.50275338	24.04973983	24.75871934	0.904263741	0.979	0.0832	PIS58896.1	DOM34	Endonuclease involved in regulation of translation; stimulates translation of protein O-mannosyltransferase family which includes PMT1, PMT2, PMT4, PMT5, and PMT6; peloto ortholog; Hap43-repressed gene
PIS54527.1	28.7383326	29.6134931	29.3889776	29.39352233	29.11802863	29.48051363	0.797576454	0.977	0.0838	PIS54527.1	MSS116	Putative DEAD-box protein; required for efficient splicing of mitochondrial Group I and II introns; Hap43-induced; rat catheter biofilm induced

PIS54965.1	27.0324906	26.8560219	26.9461042	27.09762957	27.09320414	26.89763696	0.880271514	0.978	0.0846	PIS54965.1	orf19.6699	Ortholog(s) have histidinol-phosphatase activity and role in histidine biosynthetic process
PIS51766.1	30.9125412	31.5346394	32.0679543	31.23806021	31.71263368	31.8202659	0.794325215	0.977	0.0853	PIS51766.1	ACS2	Acetyl-CoA synthetase; antigenic during human and murine infection; induced by Efg1; macrophage-induced protein; soluble protein in hyphae; gene contains intron; flow model and Spider biofilm repressed
PIS51624.1	26.9327542	26.5706877	26.7805615	27.12947376	26.63320749	26.78025768	0.856082873	0.978	0.0863	PIS51624.1	ARO9	Aromatic transaminase; Ehrlich fusel oil pathway of aromatic alcohol biosynthesis; Rim101-dependent pH-regulation (alkaline induced); Hap43-induced gene
PIS58188.1	27.2404236	27.6836014	28.5201564	26.94240407	27.97774489	28.7866899	0.939316053	0.979	0.0876	PIS58188.1	orf19.6025	Ortholog(s) have N-acetylglucosaminyldiphosphodolichol N-acetylglucosaminyltransferase activity, role in dolichol-linked oligosaccharide biosynthetic process and UDP-N-acetylglucosamine transferase complex, cytoplasm
PIS58607.1	30.5558103	30.6080513	30.4162573	30.48506824	30.88823195	30.47653133	0.791918003	0.977	0.0899	PIS58607.1	TOM20	Putative mitochondrial primary import receptor
PIS48257.1	24.2032868	23.7355636	23.8189395	23.93966577	23.69833047	24.38985963	0.88078165	0.978	0.09	PIS48257.1	orf19.6024	Ortholog of C. dubliniensis CD36 : Cd36_00770, C. auris B8441 : B9J08_004942, Candida tenuis NRRL Y-1498 : CANTEDRAFT_112621 and Debaryomyces hansenii CBS767 : DEHA2D09812g
PIS51505.1	26.0129933	25.3318355	26.3015399	26.4813856	25.13643755	26.30748801	0.906554885	0.979	0.093	PIS51505.1	orf19.1012	Ortholog(s) have role in Golgi to vacuole transport, endosome to plasma membrane protein transport, retrograde transport, endosome to Golgi and AP-1 adaptor complex, Golgi apparatus, endosome localization

PIS58879.1	27.4391431	26.3427524	28.0177215	27.08298195	27.77964297	27.22095078	0.897154547	0.978	0.0947	PIS58879.1	orf19.2296	Predicted mucin-like protein; ketoconazole-induced; fluconazole-repressed; induced in cyr1 mutant; colony morphology-related gene regulation by Ssn6; flow model biofilm induced; Spider biofilm induced
PIS58861.1	24.8042176	24.5984733	25.1427954	24.877252	25.00271802	24.95018624	0.761825658	0.977	0.0949	PIS58861.1	KIP4	S. cerevisiae Smy1 ortholog; Tn mutation affects filamentous growth; filament induced; has Mob2-dependent hyphal regulation; regulated by Nrg1, Tup1; oralpharyngeal candidasis induced; Spider biofilm induced; flow model biofilm repressed
PIS51614.1	25.5760344	25.6592569	26.5924791	26.39743469	25.79230012	25.92489884	0.800597264	0.977	0.0956	PIS51614.1	orf19.1794	Ortholog(s) have mRNA 5'-UTR binding, pre-mRNA intronic binding, translation regulator activity and role in Group I intron splicing, mitochondrial mRNA processing, positive regulation of mitochondrial translation
PIS54862.1	24.1454719	23.7712937	24.9259334	24.36388156	23.42951967	25.33595061	0.897783293	0.978	0.0956	PIS54862.1	orf19.3463	Putative GTPase; role in 60S ribosomal subunit biogenesis; Spider biofilm induced
PIS48535.1	25.8212834	25.6189845	25.0304497	25.96045768	25.08557485	25.71278868	0.914473907	0.979	0.096	PIS48535.1	orf19.4373	Protein similar to S. cerevisiae Fmn1p, which is riboflavin kinase; predicted Kex2p substrate; Hap43p-repressed gene
PIS49660.1	29.985486	29.664931	30.3106059	29.95898659	30.10580311	30.18441074	0.745216015	0.976	0.0961	PIS49660.1	orf19.6507	Putative curved DNA-binding protein orthologous to S. pombe Cdb4; stationary phase enriched protein; rat catheter and Spider biofilm repressed
PIS58918.1	29.0221214	28.9854872	28.9996524	29.57149635	28.22390899	29.50056763	0.835361811	0.978	0.0962	PIS58918.1	orf19.556	Protein of unknown function; regulated by Ssn6

PIS54753.1	25.0722471	24.1177944	24.1155322	24.90279356	24.42661833	24.26765643	0.812244257	0.977	0.0972	PIS54753.1	orf19.5813	Putative adhesin-like protein; upregulated during growth in the mouse cecum; flow model, rat catheter and Spider biofilm induced
PIS58292.1	28.8970711	27.6186298	27.996152	28.21931525	28.60587313	27.97870439	0.896030928	0.978	0.0973	PIS58292.1	EST1	Telomerase subunit; allosteric activator of catalytic activity, but not required for catalytic activity; has TPR domain
PIS58386.1	25.8088469	27.5394969	27.4206031	26.03386307	27.63891447	27.39107434	0.849534584	0.978	0.0983	PIS58386.1	orf19.6886	Ortholog(s) have rRNA binding activity and role in maturation of LSU-rRNA from tricistronic rRNA transcript (SSU-rRNA, 5.8S rRNA, LSU-rRNA), rRNA processing, ribosomal large subunit export from nucleus
PIS56602.1	26.4907176	27.847324	27.3026547	27.98507409	27.33665409	26.61399118	0.886962503	0.978	0.0983	PIS56602.1	RCL1	Putative U3-containing 90S preribosome processome complex subunit; Hap43-induced; essential; S. cerevisiae ortholog is essential; represses in core stress response;
PIS54981.1	28.3950701	28.5090059	28.6949832	28.74038169	28.65052194	28.50720795	0.830794521	0.978	0.0997	PIS54981.1	PUT1	Putative proline oxidase; regulated by Rim101p and Put3p; null mutant cannot grow on proline as a nitrogen source and shows reduced filamentation and reduced invasive growth both in vitro and in vivo; Spider biofilm induced
PIS49518.1	28.6535466	28.539672	28.0922959	27.88284676	28.16353684	29.53845854	0.907440947	0.979	0.0998	PIS49518.1	CHS6	Protein with tetratricopeptide repeats (TPRs); ortholog of S. cerevisiae Chs6, which has role in localizing chitin synthase; Cyr1-regulated in hyphae; possibly essential, disruptants not obtained by UAU1 method; flow model biofilm repressed
PIS55802.1	28.0306279	28.2815634	28.2549967	28.28326723	28.38725119	28.2027615	0.75871531	0.977	0.102	PIS55802.1	MNT2	Alpha-1,2-mannosyl transferase; adds 3rd mannose in cell-wall mannoprotein biosynthesis; partially redundant with Mnt1; role in adherence and virulence; expressed in yeast and hyphae; fungal-specific; Hap43-induced; Spider biofilm induced

PIS48409.1	26.6856882	26.6911832	25.4023179	26.04428459	26.9954572	26.04469656	0.901357985	0.979	0.102	PIS48409.1	orf19.3515	Putative 3-hydroxyanthranilic acid dioxygenase, involved in NAD biosynthesis; Hap43p-repressed gene
PIS54957.1	24.6274873	26.0490366	24.2903355	26.38554454	24.26231443	24.62515886	0.905807727	0.979	0.102	PIS54957.1	orf19.7243	Deoxycytidine monophosphate (dCMP) deaminase; role in dUMP and dTMP biosynthesis; Spider biofilm repressed
PIS49736.1	27.2080287	27.3656262	24.639884	26.30100093	26.05109199	27.17035901	0.926937144	0.979	0.103	PIS49736.1	MET8	Putative bifunctional dehydrogenase and ferrochelatase with a predicted role in siroheme biosynthesis; regulated by Gcn2p and Gcn4p
PIS49552.1	24.896932	24.0953645	22.705571	25.50997301	24.5261672	21.969574	0.899930376	0.978	0.103	PIS49552.1	orf19.2476	Ortholog(s) have chromatin binding activity and role in cellular response to oxidative stress, heterochromatin boundary formation, regulatory ncRNA-mediated heterochromatin formation
PIS51564.1	24.2759407	26.3704767	24.8425445	25.6830561	24.69863214	25.42019742	0.876367795	0.978	0.104	PIS51564.1	orf19.2796	Ortholog(s) have DNA-directed DNA polymerase activity, role in DNA replication initiation, mitotic DNA replication initiation, telomere capping and alpha DNA polymerase:primase complex, nuclear envelope, nucleus localization
PIS48711.1	25.5532578	25.7583037	26.1292009	26.32692031	25.24464802	26.18494238	0.836113291	0.978	0.105	PIS48711.1	orf19.6912	Putative ethanolamine kinase
PIS55547.1	26.8280177	24.675572	25.9648237	25.9871208	25.69010584	26.11063687	0.894469274	0.978	0.106	PIS55547.1	HIR1	Component of the HIR complex, a nucleosome assembly factor involved in chromatin formation; involved in regulation of white-opaque switching frequency and regulation of nitrogen utilization

PIS48494.1	32.2082259	31.8675815	32.1927973	32.38582361	32.29344513	31.90721129	0.708476534	0.976	0.106	PIS48494.1	TIF4631	Putative translation initiation factor eIF4G; overexpression causes hyperfilamentation; hyphal- and macrophage-induced; genes encoding some translation factors are downregulated upon phagocytosis by murine macrophage
PIS59009.1	27.8993949	27.3092436	26.9825577	26.92351097	27.84484693	27.74437032	0.861561085	0.978	0.107	PIS59009.1	FLC2	Protein involved in heme uptake; putative FAD transporter, similar to S. cerevisiae Flc2p
PIS51952.1	28.4801597	28.009679	27.9050502	28.12128402	28.38735147	28.20740572	0.91395868	0.979	0.107	PIS51952.1	orf19.988	Glycerophosphocholine acyltransferase; synthesizes phosphatidylcholine (PC); oral infection upregulated gene; null mutant shows reduced ability to damage oral epithelial cells and decreased resistance to drugs targeting lipid
PIS52375.1	24.6394063	26.3005411	26.3788566	26.03606788	25.97392063	25.63125694	0.840224664	0.978	0.107	PIS52375.1	RRN11	Putative RNA polymerase I subunit; rat catheter biofilm induced; Spider biofilm induced
PIS58689.1	25.436367	25.7232112	24.0532812	24.93516507	24.99176897	25.6087503	0.895939832	0.978	0.108	PIS58689.1	orf19.2111	Ortholog(s) have RNA polymerase II complex binding, RNA polymerase II general transcription initiation factor activity
PIS51270.1	23.2064727	23.0216716	23.7542192	23.78732149	22.90917035	23.60920011	0.81936191	0.977	0.108	PIS51270.1	orf19.6155	Ortholog(s) have DNA ligase (ATP) activity, DNA ligase activity
PIS51959.1	30.0637553	29.9567932	29.8469254	29.60551988	29.62396129	30.96163559	0.782946486	0.977	0.108	PIS51959.1	orf19.6756	Ortholog(s) have small GTPase binding activity and Golgi apparatus localization

PIS48745.1	30.5934733	30.5562894	30.145599	30.6241347	30.70611217	30.29194521	0.680977879	0.975	0.109	PIS48745.1	GDI1	Putative Rab GDP-dissociation inhibitor; GlcNAc-induced protein; Spider biofilm repressed
PIS58084.1	27.4556974	26.6267037	26.4922523	26.66746467	27.21846008	27.01511166	0.789024699	0.977	0.109	PIS58084.1	orf19.6135	Ortholog of C. dubliniensis CD36 : Cd36_32970, C. parapsilosis CDC317 : CPAR2_701710, C. auris B8441 : B9J08_000571 and Candida tenuis NRRL Y-1498 : CANTEDRAFT_106599
PIS55708.1	28.7610499	28.7690807	28.9304425	29.13559298	29.05930732	28.59619844	0.728098954	0.976	0.11	PIS55708.1	orf19.4437	Ortholog(s) have ATPase, DNA binding, mRNA 3'-UTR binding, nucleosome binding, rDNA binding, transcription cis-regulatory region binding activity
PIS48465.1	25.815719	25.6028103	25.7378885	26.07937688	25.68299423	25.7239054	0.837774525	0.978	0.11	PIS48465.1	orf19.6187	possible pseudouridine monophosphate glycosidase; overlaps orf19.6185 and orf19.618; has intron; Spider biofilm repressed
PIS48422.1	23.2590411	23.7958985	23.2025257	23.71021277	23.95018504	22.93388811	0.820855558	0.978	0.112	PIS48422.1	MTG2	Putative Obg family GTPase member; peripheral protein of the mitochondrial inner membrane; associates with the large ribosomal subunit; required for mitochondrial translation; rat catheter biofilm repressed
PIS58763.1	26.8297766	28.4865146	26.624757	28.78471308	26.33612824	27.15480973	0.896357411	0.978	0.112	PIS58763.1	NAN1	Putative U3 snoRNP protein; Hap43p-induced gene; physically interacts with TAP-tagged Nop1p
PIS52042.1	25.9054758	26.0080093	26.596002	27.42936106	26.00633936	25.41098007	0.853173106	0.978	0.112	PIS52042.1	orf19.1547	Ortholog(s) have double-stranded DNA binding, polynucleotide 3'-phosphatase activity and role in double-strand break repair

PIS50550.1	25.1120242	24.8375861	25.7279095	25.51970289	25.53797474	24.95807882	0.829648649	0.978	0.113	PIS50550.1	RHR2	[illegible] osmotic tolerance, glycerol accumulation in response to salt; Spider/flow model biofilm induced; regulated by macrophage, stress, yeast-hyphal switch, pheromone, [illegible]
PIS54866.1	27.8727219	27.3153095	28.3441105	27.59616875	28.59536372	27.6847244	0.855706888	0.978	0.115	PIS54866.1	orf19.3312	Protein of unknown function; flow model and Spider biofilm repressed
PIS51861.1	27.7795352	27.36294	29.3689694	28.78354784	27.3822311	28.69315996	0.840427063	0.978	0.116	PIS51861.1	HIS4	[illegible] three steps of histidine biosynthesis, with phosphoribosyl-AMP cyclohydrolase, phosphoribosyl-ATP diphosphatase, and histidinol dehydrogenase activities; required for [illegible]
PIS55005.1	28.7351836	28.3343895	28.1347129	28.39596088	28.23399022	28.92140421	0.724886169	0.976	0.116	PIS55005.1	orf19.3286	Ortholog(s) have alpha-1,4-glucosidase activity, role in N-glycan processing, polysaccharide biosynthetic process and endoplasmic reticulum lumen, glucosidase II complex localization
PIS48467.1	30.3595523	30.1409419	29.6969301	30.31496396	30.18006397	30.05007941	0.833650192	0.978	0.116	PIS48467.1	orf19.6189	Chalcone related protein family; flow model biofilm induced
PIS58650.1	26.8223718	27.2993597	27.2729314	27.02552592	27.35479866	27.3654708	0.815689704	0.977	0.117	PIS58650.1	ECM22	Zn(II)2Cys6 transcription factor; rat catheter and Spider biofilm induced
PIS54571.1	23.9532255	24.1339068	24.9275304	24.7390242	24.41104618	24.21470408	0.761750731	0.977	0.117	PIS54571.1	orf19.325	Putative mRNA cleavage and polyadenlylation factor; heterozygous null mutant exhibits hypersensitivity to parnafungin and cordycepin in the C. albicans fitness test

PIS49593.1	26.2094212	25.3805846	25.4852743	25.24322258	25.89302203	26.28971281	0.781975784	0.977	0.117	PIS49593.1	PTR3	Protein similar to S. cerevisiae Ptr3p, which is a sensor of external amino acids; expression upregulated in an ssr1 null mutant
PIS58544.1	30.9135086	30.0658067	30.4209768	30.78678311	30.88951364	30.07986533	0.709996071	0.976	0.119	PIS58544.1	MRP20	Component of mitochondrial ribosome; decreased expression in hyphae compared to yeast-form cells
PIS52240.1	26.8604934	26.8347176	26.5214687	27.46211378	26.53042111	26.58312396	0.809272317	0.977	0.12	PIS52240.1	orf19.1444	Epsin, involved in endocytosis; mutants are defective in hyphal growth and virulence
PIS51914.1	27.9802722	27.9957328	27.4375349	27.22685499	28.23567571	28.31215087	0.751015974	0.977	0.12	PIS51914.1	orf19.374	Ortholog(s) have role in protein transport to vacuole involved in ubiquitin-dependent protein catabolic process via the multivesicular body sorting pathway
PIS52270.1	30.775501	30.8020952	30.6949714	30.82840428	31.02652433	30.77903236	0.690432616	0.976	0.12	PIS52270.1	orf19.4066	Putative glycerol-3-phosphate acyltransferase; Hog1-repressed
PIS51502.1	24.3428125	23.9281997	22.6860117	25.9027008	22.78702962	22.63600416	0.904597688	0.979	0.123	PIS51502.1	KCS1	Predicted inositol polyphosphate kinase; Spider biofilm induced
PIS49547.1	28.5173235	28.5640715	27.8700474	28.37147366	28.61421395	28.3332615	0.893332696	0.978	0.123	PIS49547.1	orf19.2473	Component of the RSC chromatin remodeling complex; decreased transcription is observed upon fluphenazine treatment or in an azole-resistant strain that overexpresses CDR1 and CDR2

PIS49822.1	29.0999925	28.8720617	28.9939829	29.5599633	28.82717664	28.9481307	0.675117574	0.975	0.123	PIS49822.1	orf19.4380.1	Ortholog(s) have role in mitochondrial genome maintenance and mitochondrial inner membrane localization
PIS49794.1	28.4556334	29.6012329	29.6043558	29.10949423	29.4411816	29.47823804	0.744870622	0.976	0.123	PIS49794.1	STI1	Protein that interacts with Cdc37 and Crk1 in two-hybrid; may be involved in Cdc37 chaperone activity; soluble protein in hyphae; protein in exponential and stationary phase yeast cultures; YNB biofilm induced; Spider biofilm induced
PIS54657.1	30.8816483	30.3034439	29.9062847	30.69755202	30.30276031	30.4670444	0.693307388	0.976	0.125	PIS54657.1	NUO1	NADH-ubiquinone oxidoreductase subunit with roles in mitochondrial respiratory chain complex I assembly
PIS56907.1	28.9634286	29.3415925	29.2719875	29.34064945	29.40218254	29.21323204	0.678255096	0.975	0.126	PIS56907.1	PAP1	Poly(A) polymerase, likely involved in mRNA polyadenylation; PAP is inhibited by parnafungin antifungals; non-sex gene located within the MTLa mating-type-like locus
PIS51743.1	24.4130174	26.0160353	23.2988404	24.65910289	24.77428511	24.67280492	0.877830244	0.978	0.126	PIS51743.1	UBP13	Ortholog of S. cerevisiae Ubp13; putative ubiquitin carboxyl-terminal hydrolase; flow model biofilm induced; rat catheter biofilm repressed
PIS49842.1	27.691997	26.4752342	26.0818343	26.40393849	27.36865058	26.85680805	0.775463814	0.977	0.127	PIS49842.1	EXO70	Predicted subunit of the exocyst complex, involved in exocytosis; localizes to a crescent on the surface of the hyphal tip
PIS56613.1	25.9678594	26.9770915	25.8782791	25.49295006	26.18636933	27.52401564	0.836776336	0.978	0.127	PIS56613.1	VMA10	Subunit G of the V1 peripheral membrane domain of the vacuolar H+-ATPase (V-ATPase); involved in vacuolar acidification; rat catheter biofilm repressed

PIS58123.1	31.0442942	29.9804657	28.0697904	30.59224292	29.69818394	29.18802346	0.879465893	0.978	0.128	PIS58123.1	orf19.6260	Ubiquitin-specific protease; cleaves ubiquitin from ubiquitinated proteins; Spider biofilm induced
PIS55580.1	28.0221686	27.8926113	27.4072469	28.15403687	27.80616372	27.74834263	0.777844289	0.977	0.129	PIS55580.1	MEU1	Putative methylthioadenosine phosphorylase; protein level decreases in stationary phase cultures
PIS58762.1	28.1642076	28.8180388	27.8155089	29.08349747	28.1317488	27.96808644	0.760455065	0.977	0.129	PIS58762.1	orf19.2690	Putative mitochondrial GTPase; required for mitochondrial morphology and genome maintenance; Spider biofilm induced
PIS58660.1	27.954406	26.8728556	27.0788123	27.59263738	27.62042779	27.08114954	0.857186862	0.978	0.129	PIS58660.1	orf19.6374	Ortholog(s) have unfolded protein binding activity, role in mitochondrial proton-transporting ATP synthase complex assembly and mitochondrial inner membrane, mitochondrial membrane localization
PIS56638.1	30.830805	30.8765875	30.6910433	31.18340067	30.85116407	30.75719344	0.620386258	0.974	0.131	PIS56638.1	FRS1	Phenylalanyl-tRNA synthetase; possible role in early cell wall biosynthesis; downregulated by phagocytosis by macrophages; possibly essential gene, disruptants not obtained by UAU1 method; protein present in exponential and stationary
PIS52011.1	27.8606662	26.3198596	25.2940748	27.68007126	26.70758897	25.47865356	0.882753167	0.978	0.131	PIS52011.1	orf19.813	Protein of unknown function; mutants are viable; Hap43-induced gene; oxidative stress-induced via Cap1; rat catheter and Spider biofilm induced
PIS54854.1	30.6032545	30.7113436	30.9006308	30.99563757	30.70983837	30.90651327	0.629218631	0.975	0.132	PIS54854.1	GTT12	Ortholog(s) have glutathione peroxidase activity, glutathione transferase activity, role in glutathione metabolic process, protein glutathionylation and endoplasmic reticulum localization

PIS54669.1	25.7729668	23.8370208	23.9482074	24.60589483	25.13456934	24.21656313	0.796240191	0.977	0.133	PIS54669.1	ASG1	Gal4p family zinc-finger transcription factor with similarity to S. cerevisiae Asg1p
PIS50628.1	25.877874	26.0566823	24.0438033	26.16192781	25.43295558	24.78153947	0.897350934	0.978	0.133	PIS50628.1	BEM1	Protein required for wild-type budding, hyphal growth, and virulence in a mouse systemic infection; suppresses pseudohyphal and filamentous growth defects of various S. cerevisiae mutants and heat sensitivity of S. cerevisiae cdc24-4 mutant
PIS58484.1	30.8804061	30.6399724	30.6332399	30.72472926	30.94333332	30.88442737	0.60767125	0.974	0.133	PIS58484.1	orf19.3782.2	Ortholog(s) have role in cristae formation, membrane organization and MICOS complex, mitochondrial crista junction localization
PIS58223.1	28.0413063	28.4092106	26.9056841	27.84420495	28.11221631	27.80000122	0.779102091	0.977	0.133	PIS58223.1	orf19.7012	Ortholog(s) have structural constituent of ribosome activity and mitochondrial small ribosomal subunit localization
PIS51183.1	24.4056232	23.6699524	23.4943512	24.64540238	23.90511668	23.41799041	0.826444084	0.978	0.133	PIS51183.1	orf19.7580	Ortholog of C. dubliniensis CD36 : Cd36_35095, C. parapsilosis CDC317 : CPAR2_200710, C. auris B8441 : B9J08_002757 and Candida tenuis NRRL Y-1498 : CANTEDRAFT_112338
PIS52213.1	26.9456709	25.4562585	26.5058301	27.22695877	26.56609951	25.51291732	0.800716867	0.977	0.133	PIS52213.1	PUS7	Pseudouridine synthase; catalyzes pseudouridylation in U2 snRNA, 5S rRNA, cytoplasmic tRNAs and in pre-tRNA(Tyr); F-12/CO2 early biofilm induced
PIS58453.1	23.7222775	24.431641	24.488881	24.27567971	24.36384195	24.40513341	0.858020761	0.978	0.134	PIS58453.1	orf19.3686	Ortholog(s) have protein domain specific binding activity, role in mitochondrial proton-transporting ATP synthase complex assembly and mitochondrion localization

PIS49829.1	21.9340688	23.5203673	24.1469647	23.72064908	23.50393541	22.78279157	0.886363961	0.978	0.135	PIS49829.1	RAD23	S. cerevisiae Rad23 ortholog; binds damaged DNA; Spider biofilm repressed
PIS55555.1	27.0946727	25.5991269	25.3467782	28.08952015	26.14726221	24.21824652	0.870884796	0.978	0.138	PIS55555.1	orf19.6606	Ortholog of C. parapsilosis CDC317 : CPAR2_201040, C. auris B8441 : B9J08_001657, Candida tenuis NRRL Y-1498 : CANTEDRAFT_112677 and Debaryomyces hansenii CBS767 : DEHA2E02420g
PIS51845.1	29.1282199	30.0822306	30.2780036	29.19013878	30.36690517	30.34861749	0.764684578	0.977	0.139	PIS51845.1	RAS1	RAS signal transduction GTPase; regulates cAMP and MAP kinase pathways; role in hyphal induction, virulence, apoptosis, heat-shock sensitivity; nonessential; plasma membrane-localized; complements viability of S. cerevisiae ras1 ras2
PIS49527.1	32.4142094	32.6705151	32.6357565	32.62659316	32.66482765	32.84574772	0.62314121	0.974	0.139	PIS49527.1	ZWF1	Glucose-6-phosphate dehydrogenase; antigenic in mice; activity induced by O2 or oxidizing agents H2O2, menadione, macrophage; caspofungin repressed; induced in core stress response; regulated by Gcn2, Gcn4; rat catheter biofilm repressed
PIS51108.1	26.6331811	26.7874901	26.9953393	26.75602571	26.61578065	27.46915559	0.735086654	0.976	0.142	PIS51108.1	orf19.7527	Ortholog of C. dubliniensis CD36 : Cd36_25130, C. parapsilosis CDC317 : CPAR2_800100, C. auris B8441 : B9J08_002680 and Candida tenuis NRRL Y-1498 : CANTEDRAFT_107256
PIS58730.1	25.2359905	24.5448164	25.2476	25.34041334	25.65402476	24.46395098	0.792375228	0.977	0.143	PIS58730.1	orf19.1708	Protein of unknown function; Spider biofilm induced
PIS51865.1	20.3953162	24.5860251	24.2796425	24.53007474	21.23016736	23.93224554	0.889029831	0.978	0.144	PIS51865.1	CDC54	Putative pre-replication complex helicase subunit; transcript regulated by Nrg1 and Mig1; periodic mRNA expression, peak at cell-cycle M/G1 phase; Hap43-induced

PIS50510.1	27.1621798	27.1953509	25.8842292	27.17008362	26.716719	26.78605971	0.839426687	0.978	0.144	PIS50510.1	TIP20	Protein interacting with Sec20p, possibly involved in retrograde transport between the Golgi and the endoplasmic reticulum; similar to S. cerevisiae Tip20p
PIS52376.1	29.6372955	32.2620585	32.4264234	31.5780177	31.75630468	31.42762676	0.847704836	0.978	0.145	PIS52376.1	HSP60	Heat shock protein; soluble in hyphae; regulated by Nrg1 and by iron; induced in high iron; heavy metal (cadmium) stress-induced; sumoylation target; protein present in exponential and stationary phase cells; Hap43-repressed
PIS58878.1	26.4887256	27.1675032	26.3738887	27.38357033	26.72077118	26.36513246	0.713784932	0.976	0.146	PIS58878.1	ARL3	Putative Ras superfamily GTPase; induced by nitric oxide independent of Yhb1p
PIS52459.1	29.2978159	29.2720186	28.2900167	28.89004575	29.60240635	28.80430649	0.739083593	0.976	0.146	PIS52459.1	FUR1	Uracil phosphoribosyltransferase; predicted tetrameric enzyme of pyrimidine salvage; mutations associated with flucytosine resistance in clade I clinical isolates; flucytosine, macrophage-induced protein; levels decrease in stationary phase
PIS56716.1	29.6068526	29.37293	29.6342409	30.26121332	29.51690899	29.27638903	0.619989547	0.974	0.147	PIS56716.1	RPO21	RNA polymerase II; ortholog of S. cerevisiae Rpo21, transposon mutation affects filamentous growth; flow model biofilm repressed
PIS51781.1	26.160386	26.7079917	26.5590419	26.08869735	26.68439855	27.09462654	0.774641278	0.977	0.147	PIS51781.1	VPS16	Protein similar to S. cerevisiae Vps16p, which is involved in protein-vacuolar targeting; likely to be essential for growth, based on insertional mutagenesis; downregulated in biofilm or in azole-resistant strain that overexpresses
PIS51269.1	29.0668939	28.8714651	28.6140996	29.11006784	29.06156549	28.82459291	0.66022571	0.975	0.148	PIS51269.1	ARP9	Protein similar to S. cerevisiae Arp3p, a component of the Arp2/3 complex involved in actin-dependent processes; likely to be essential for growth, based on an insertional mutagenesis strategy

PIS48659.1	26.4503109	25.9603563	26.6982671	27.1281579	26.28484875	26.13895123	0.836471964	0.978	0.148	PIS48659.1	CAN2	Basic amino acid permease; arginine metabolism; regulated by Nrg1/Tup1; caspofungin, flucytosine induced; colony morphology-related regulation by Ssn6; Hap43-repressed; rat catheter and Spider biofilm induced; promoter bound by Efg1
PIS51613.1	26.6015481	27.1280557	24.3107126	25.50973404	26.24519183	26.72801315	0.825786137	0.978	0.148	PIS51613.1	orf19.1793	Ortholog(s) have phosphatidylinositol-3,5-bisphosphate binding, phosphatidylinositol-3-phosphate binding, phosphatidylinositol-4-phosphate binding, phosphatidylinositol-5-phosphate binding activity
PIS52426.1	31.4170762	30.8336671	30.2884244	31.32416803	30.98481169	30.67449359	0.64502763	0.975	0.148	PIS52426.1	SAC1	Putative phosphatidylinositol phosphate (PtdInsP) phosphatase, involved in cell wall integrity and morphogenesis
PIS51407.1	26.0143525	27.4095377	25.9622859	27.05459517	26.54944115	26.23396333	0.738059575	0.976	0.151	PIS51407.1	ARP4	Subunit of the NuA4 histone acetyltransferase complex
PIS58745.1	30.4106023	29.6184923	29.982057	30.30877646	30.15673658	29.99879689	0.642556751	0.975	0.151	PIS58745.1	orf19.1917	Protein of unknown function; Spider biofilm repressed
PIS51297.1	25.8907748	24.6036897	25.335363	24.16071984	26.87076159	25.25905936	0.803755344	0.977	0.154	PIS51297.1	ALG1	Protein similar to S. cerevisiae Alg1p, a mannosyltransferase involved in N-linked protein glycosylation; likely to be essential for growth, based on an insertional mutagenesis strategy
PIS48322.1	29.7423502	29.5561464	29.6293267	29.91992305	29.34780651	30.12166277	0.759597178	0.977	0.154	PIS48322.1	orf19.3559	S. cerevisiae ortholog Mrps35p is a structural constituent of ribosome and localizes to mitochondrial small ribosomal subunit; the snoRNA CD39 is encoded within the MRPS35 intron

PIS51304.1	28.4328537	28.6780211	27.9138222	28.09334276	28.71278248	28.6811343	0.785016742	0.977	0.154	PIS51304.1	TFS1	Putative carboxypeptidase y inhibitor; transcript regulated upon yeast-hypha switch; colony morphology-related gene regulation by Ssn6
PIS56623.1	29.4201813	30.1331772	30.3400987	29.80100596	30.27541444	30.28276762	0.787838947	0.977	0.155	PIS56623.1	HEM13	Coproporphyrinogen III oxidase; antigenic; on yeast cell surface, not hyphae; iron-regulated expression; Hap43, macrophage-repressed; farnesol-induced; possibly essential; flow model biofilm induced; rat catheter, Spider biofilm repressed
PIS56541.1	27.8715238	27.2207636	25.9283906	27.35911383	27.2023717	26.9235132	0.734684946	0.976	0.155	PIS56541.1	orf19.5541	Protein with similarity to S. pombe Nrd1p; transcription induced upon induction of hyphal growth; regulated by Cph1p, Efg1p, Cph2p; low-level expression; alkaline upregulated; fungal-specific (no human or murine homolog)
PIS48340.1	28.4568271	28.5654566	28.8512794	28.7983877	28.77153384	28.76779606	0.690620216	0.976	0.155	PIS48340.1	RHO3	Putative Rho family GTPase; possible substrate of protein farnesyltransferase and geranylgeranyltransferase type I; greater transcription in hyphal form than yeast form; plasma membrane-localized
PIS55631.1	23.8465167	23.7923781	24.5634346	25.06447791	23.89126509	23.71500775	0.769456761	0.977	0.156	PIS55631.1	orf19.6843	Ortholog(s) have role in CENP-A containing chromatin assembly, chromatin remodeling and nucleoplasm localization
PIS50462.1	29.3667831	29.4290024	29.4616564	29.69437578	29.54086361	29.4914499	0.720366516	0.976	0.156	PIS50462.1	PAN1	Essential protein involved in endocytosis and polarized growth; ortholog of S. cerevisiae Pan1, which is a part of a complex that regulates actin cytoskeleton; Spider biofilm repressed
PIS54584.1	24.4303262	25.1761667	25.3537674	25.24194215	25.29329504	24.89598839	0.727006424	0.976	0.157	PIS54584.1	orf19.2516	Ortholog of C. dubliniensis CD36 : Cd36_81040, C. parapsilosis CDC317 : CPAR2_101980, C. auris B8441 : B9J08_002360 and Candida tenuis NRRL Y-1498 : CANTEDRAFT_113332

PIS58430.1	28.9832148	28.5655364	28.3045303	28.24668168	28.92538876	29.15462692	0.670371485	0.975	0.158	PIS58430.1	AQY1	Aquaporin water channel; osmotic shock resistance, WT freeze tolerance; virulent in mice; flucytosine repressed; flow model/RPMI/Spider/rat catheter biofilm induced; required for RPMI biofilm formation; Bcr1-induced in a/a RPMI biofilms
PIS50385.1	27.6383389	25.2815603	25.9055901	28.73314464	26.19113968	24.37628979	0.871487562	0.978	0.158	PIS50385.1	orf19.1265	Ortholog(s) have guanyl-nucleotide exchange factor activity and role in cytoplasm to vacuole transport by the Cvt pathway, early endosome to Golgi transport, intra-Golgi vesicle-mediated transport, macroautophagy
PIS58685.1	23.4677039	23.9660898	26.1589494	25.03104262	24.4216276	24.6137366	0.816632064	0.977	0.158	PIS58685.1	SMC2	Protein similar to S. cerevisiae Smc2p, which is a component of the condensin complex involved in mitotic chromosome condensation; induced under hydroxyurea treatment
PIS58576.1	25.4071446	24.2089896	25.4081136	24.93369216	25.26658221	25.30178367	0.738952166	0.976	0.159	PIS58576.1	orf19.6852	Ortholog of Rmd6 involved in S. cerevisiae sporulation; flow model biofilm induced
PIS51373.1	26.3563988	25.4277654	25.7485336	27.07086406	25.24144045	25.70064536	0.85617427	0.978	0.16	PIS51373.1	LAS1	Putative bud formation and morphogenesis protein; mutation confers hypersensitivity to 5-fluorocytosine (5-FC), 5-fluorouracil (5-FU); macrophage-induced; Spider biofilm induced
PIS50413.1	31.062052	30.5739193	30.3304718	30.90697587	30.92053581	30.61791687	0.695422841	0.976	0.16	PIS50413.1	orf19.5085	Ortholog(s) have nuclear import signal receptor activity, nuclear localization sequence binding activity
PIS49585.1	23.6034736	24.1215597	24.5389575	23.04408781	25.66148712	24.03744256	0.787192626	0.977	0.16	PIS49585.1	orf19.746	Ortholog(s) have phosphatidic acid binding activity, role in nuclear pore complex assembly and nuclear inner membrane localization

PIS48631.1	24.6041463	25.7761785	25.5875177	25.32677836	25.81271268	25.30768171	0.697059789	0.976	0.16	PIS48631.1	STE11	Protein similar to S. cerevisiae Ste11p; mutants are sensitive to growth on H2O2 medium
PIS58646.1	26.489313	21.3171608	24.5287734	25.96419998	25.73436004	21.11909698	0.908573915	0.979	0.161	PIS58646.1	DYN1	Dynein heavy chain; motor protein that moves to microtubule minus end; required for yeast cell separation, spindle positioning, nuclear migration, hyphal growth; regulated by Mig1, Hap43; flow model and rat catheter biofilm repressed
PIS58303.1	26.9878921	25.5011116	24.5326937	26.56333156	25.58707503	25.35390295	0.839463437	0.978	0.161	PIS58303.1	orf19.3432	Predicted membrane transporter, member of the drug:proton antiporter (12 spanner) (DHA1) family, major facilitator superfamily (MFS); induced by nitric oxide
PIS51232.1	26.7276164	26.7638336	27.2602396	26.87694952	27.11582712	27.24352616	0.727144168	0.976	0.162	PIS51232.1	orf19.2249	Putative metalloprotease of the mitochondrial inner membrane; expression downregulated in an ssr1 null mutant
PIS48220.1	26.5782378	24.9200107	25.5801565	25.94127451	26.55303737	25.07053786	0.875554526	0.978	0.162	PIS48220.1	orf19.6448	Protein of unknown function; Spider biofilm repressed
PIS55478.1	25.0979102	24.7288311	25.3526584	24.99688077	25.1766717	25.49411013	0.740653518	0.976	0.163	PIS55478.1	orf19.1160	Ortholog(s) have COPII receptor activity and role in COPII-coated vesicle cargo loading, endoplasmic reticulum to Golgi vesicle-mediated transport, fungal-type cell wall organization, protein retention in Golgi apparatus
PIS54577.1	28.308674	26.861543	26.0906518	27.87901165	27.68844344	26.18841142	0.805057101	0.977	0.165	PIS54577.1	orf19.1730	HMG-box protein; Spider biofilm repressed

PIS54892.1	28.0485209	27.924199	27.3785969	28.29815059	27.70734399	27.84054923	0.742153783	0.976	0.165	PIS54892.1	orf19.4953	Putative ATPase; predicted role in ER-associated protein catabolism; induced during chlamydospore formation in both C. albicans and C. dubliniensis; rat catheter biofilm repressed
PIS48805.1	26.0481788	27.294169	27.710971	26.90069723	27.22178016	27.42518978	0.760796297	0.977	0.165	PIS48805.1	orf19.5884	Putative nucleolar preribosomal-associated protein; decreased transcription is observed upon benomyl treatment or in an azole-resistant strain that overexpresses MDR1; Spider biofilm induced
PIS51867.1	24.8099504	25.6094429	25.9544707	25.73905223	25.5711081	25.56253223	0.662531047	0.975	0.166	PIS51867.1	orf19.3763	Has domain(s) with predicted role in retrograde vesicle-mediated transport, Golgi to endoplasmic reticulum and membrane localization
PIS51191.1	26.3924784	27.3109468	27.4429027	25.88910968	28.35385994	27.40114457	0.871107918	0.978	0.166	PIS51191.1	orf19.7603	Protein with a predicted role in cytochrome c oxidase assembly; rat catheter biofilm induced
PIS48284.1	26.4620198	26.6666285	27.1608179	27.48006118	26.47034157	26.83785869	0.814967766	0.977	0.166	PIS48284.1	SGT2	Putative small tetratricopeptide repeat (TPR)-containing protein; protein abundance is affected by URA3 expression in the CAI-4 strain background; Mig1-regulated
PIS49503.1	23.3490593	22.2088115	25.4982647	23.18135348	23.69771488	24.67780049	0.825291405	0.978	0.167	PIS49503.1	ECM14	Has domain(s) with predicted metallocarboxypeptidase activity, zinc ion binding activity and role in proteolysis
PIS51248.1	31.6341427	31.3292271	31.3612368	31.83637283	31.55099669	31.43966885	0.587742039	0.974	0.167	PIS51248.1	SPF1	P-type calcium-transporting ATPase, involved in control of calcium homeostasis, response to ER stress, hyphal growth, biofilm formation and virulence

PIS51983.1	26.9967226	27.3961021	26.9771765	26.89025192	27.57417906	27.40988956	0.912092804	0.979	0.168	PIS51983.1	DOT4	Protein similar to ubiquitin C-terminal hydrolase; localizes to cell surface of hyphal cells, but not yeast-form cells; repressed upon high-level peroxide; Hap43p-induced; rat catheter biofilm induced
PIS56725.1	30.7964025	30.0921909	30.5976849	30.67142774	30.81233802	30.50576533	0.590640272	0.974	0.168	PIS56725.1	TUP1	Transcriptional corepressor; represses filamentous growth; regulates switching; role in germ tube induction, farnesol response; in repression pathways with Nrg1, Rfg1; farnesol upregulated in biofilm; rat catheter, Spider biofilm repressed
PIS52073.1	33.0115995	31.496996	30.9687234	31.45331911	32.48439825	32.04707159	0.789690621	0.977	0.169	PIS52073.1	GPD2	Surface protein similar to glycerol 3-P dehydrogenase; binds host Factor H, FHL-1, plasminogen; regulated by Ssn6, Nrg1, Efg1; induced by cell wall regeneration, macrophage/pseudohyphal growth, core stress response; Spider biofilm
PIS58309.1	29.216082	29.1272757	29.3475595	29.75968998	28.93124988	29.50983337	0.57051539	0.973	0.17	PIS58309.1	ARC40	Protein similar to S. cerevisiae Arc40; involved in actin filament organization in S. cerevisiae; transposon mutation affects filamentous growth; rat catheter and Spider biofilm repressed
PIS50613.1	24.189775	24.4952461	25.3773354	25.06726298	24.92480829	24.58082435	0.706085566	0.976	0.17	PIS50613.1	orf19.6968	Protein of unknown function; Hog1-repressed; Spider biofilm induced
PIS51557.1	26.1678408	23.8610286	24.3836469	24.81235556	23.39258709	26.71852314	0.822225184	0.978	0.17	PIS51557.1	PRY1	Pry family pathogenesis-related protein; extracellular; opaque specific transcript; repressed by alpha pheromone in SpiderM medium; possibly essential, disruptants not obtained by UAU1 method; Spider biofilm induced
PIS51241.1	28.9994482	28.7638069	28.2700658	28.5123272	29.12169053	28.90925475	0.61872538	0.974	0.17	PIS51241.1	YMC1	Putative inner mitochondrial membrane transporter; flucytosine induced; Spider biofilm repressed

PIS49535.1	24.0876442	23.2525305	24.9994355	24.13505502	24.44864488	24.2680129	0.753316418	0.977	0.171	PIS49535.1	CPP1	VH1 family MAPK phosphatase; regulates Cst20-Hst7-Cek1-Cph1 filamentation pathway; negatively regulates mating, represses yeast-hyphal switch; required for virulence in mice; yeast-enriched; induced by alpha pheromone in SpiderM medium;
PIS58447.1	29.2278494	29.2842411	27.7691385	29.31450057	28.52440075	28.95428437	0.726089008	0.976	0.171	PIS58447.1	SPE7	Septin, required for wild-type invasive growth in vitro but not required for virulence in a mouse model of systemic infection; localizes to hyphal septum or bud neck; Asn-rich; aberrant gel mobility; phosphorylated in vitro by Gin4p
PIS55639.1	26.9040501	26.3781543	27.0696073	27.16743741	26.42708215	27.27448088	0.596764079	0.974	0.172	PIS55639.1	orf19.1658	Protein involved in filamentous growth and maintenance of cell membrane integrity; null mutant has normal growth but shows sensitivity to sodium dodecyl sulfate and reduced filamentation under hyphae-inducing agents
PIS50440.1	24.4636235	26.586967	24.3864702	25.52087479	25.17280974	25.26386556	0.790806136	0.977	0.173	PIS50440.1	CDC45	Putative DNA replication initiation factor; transcriptionally regulated by interaction with macrophage
PIS54811.1	26.1325484	26.3067237	27.1892419	27.01140549	26.52304872	26.61378352	0.793079475	0.977	0.173	PIS54811.1	MCA1	Putative metacaspase, cysteine protease involved in apoptosis in response to stresses; has similarity to S. cerevisiae Mca1p; fungal-specific (no human or murine homolog); farnesol-induced
PIS58863.1	29.834594	29.698763	29.7924895	30.65418528	29.56870098	29.62623289	0.634075264	0.975	0.174	PIS58863.1	SER33	Predicted enzyme of amino acid biosynthesis; Gcn4p-regulated; upregulated in biofilm; protein present in exponential and stationary growth phase yeast cultures; S. cerevisiae ortholog is Gcn4p regulated
PIS56967.1	28.7936616	27.9314087	26.2311559	28.12093122	28.75849007	26.60183331	0.802873784	0.977	0.175	PIS56967.1	MNN12	Predicted alpha-1,3-mannosyltransferase activity with a role in protein glycosylation

PIS58349.1	26.6757002	26.325781	27.311088	28.11846391	26.58671513	26.13161753	0.771273836	0.977	0.175	PIS58349.1	NIT2	Putative carbon-nitrogen hydrolase; rat catheter biofilm repressed
PIS51850.1	24.5474358	24.480381	23.6690147	25.2728319	23.86654108	24.08459457	0.755646755	0.977	0.176	PIS51850.1	orf19.871	Ortholog(s) have GTPase activator activity, role in positive regulation of TORC1 signaling and FNIP-folliculin RagC/D GAP, cytoplasm, vacuolar membrane localization
PIS48457.1	34.0965027	33.7453827	33.5488159	33.91446219	33.75301748	34.25222557	0.582834242	0.974	0.176	PIS48457.1	RPL2	Putative 60S ribosomal protein L2; Hap43-induced gene; repressed in infected rabbit kidney in SC5314, but not NGY152, strain background; Spider biofilm repressed
PIS51204.1	32.4507252	32.153356	31.7518832	31.94100315	32.45440703	32.4892728	0.54036432	0.973	0.176	PIS51204.1	THS1	Putative threonyl-tRNA synthetase; transcript regulated by Mig1 and Tup1; repressed upon phagocytosis by murine macrophages; stationary phase enriched protein; Spider biofilm repressed
PIS55679.1	30.9961264	30.9104312	30.6293386	31.36186133	30.90166551	30.80432164	0.531073249	0.973	0.177	PIS55679.1	RPN1	Putative 19S regulatory particle of the 26S proteasome; regulated by Gcn2p and Gcn4p
PIS51320.1	29.7159055	29.6646099	29.0249676	29.05860548	29.97655225	29.8998385	0.836715824	0.978	0.177	PIS51320.1	TAF60	Putative TFIID and SAGA complex subunit; mutation confers hypersensitivity to amphotericin B
PIS51738.1	26.6103257	25.1256076	22.527023	25.04134212	24.94533016	24.80839272	0.813675501	0.977	0.177	PIS51738.1	VPS24	Protein similar to S. cerevisiae Vps24p, which is a member of the ESCRT III protein sorting complex; downregulated upon adherence to polystyrene

PIS48767.1	22.9652246	23.3580213	23.074466	23.46340351	22.63883607	23.83253323	0.831810015	0.978	0.179	PIS48767.1	orf19.4375	Ortholog(s) have N-terminal protein N-methyltransferase activity, S-adenosylmethionine-dependent methyltransferase activity, protein-lysine N-methyltransferase activity and role in rDNA heterochromatin formation
PIS50411.1	29.649056	29.7383899	30.6865386	30.30895114	30.16545314	30.13911901	0.600556409	0.974	0.18	PIS50411.1	FUN12	Functional homolog of S. cerevisiae Fun12 translation initiation factor eIF5B; genes encoding ribosomal subunits, translation factors, and tRNA synthetases are downregulated upon phagocytosis by murine macrophage
PIS58341.1	29.5834131	29.8440763	28.9492124	29.86273146	29.66528739	29.38771353	0.588599816	0.974	0.18	PIS58341.1	PMR1	Secretory pathway P-type Ca2+/Mn2+-ATPase; calcium pump involved in control of calcium homeostasis; required for protein glycosylation and cell wall maintenance; required for hyphal tip oscillation in semisolid substrate
PIS50445.1	24.8736261	24.0218284	23.5300331	24.70746284	23.98209902	24.27988708	0.704018906	0.976	0.181	PIS50445.1	PTC7	Protein phosphatase, type 2C; has S/T phosphatase activity, Mn2+/Mg2+ dependent; predicted membrane-spanning segment and mitochondrion-targeting signal
PIS56925.1	26.5247317	25.6083934	26.5622366	26.18090637	26.87189099	26.1927536	0.847699767	0.978	0.183	PIS56925.1	KRR1	Putative nucleolar protein; repressed benomyl treatment or in an azole-resistant strain that overexpresses MDR1; F-12/CO2 early biofilm induced
PIS58503.1	27.4297392	26.7659889	27.2283771	27.05478481	27.15755596	27.76372793	0.766125008	0.977	0.184	PIS58503.1	orf19.1686	Has domain(s) with predicted kinase activity and role in carbohydrate metabolic process
PIS51513.1	27.3643445	26.6104063	27.1772515	27.02406437	26.978982	27.70301959	0.677143046	0.975	0.185	PIS51513.1	SER1	Putative 3-phosphoserine aminotransferase; predicted role in serine and glycine biosynthesis; protein present in exponential and stationary yeast growth phases; Spider biofilm repressed

PIS58034.1	28.6170782	28.8895936	28.7334624	28.90842811	28.92256369	28.96751072	0.545839637	0.973	0.186	PIS58034.1	SRO77	Protein with a predicted role in docking and fusion of post-Golgi vesicles with the plasma membrane; filament induced; fungal-specific (no human or murine homolog)
PIS58700.1	30.6877391	30.1427007	30.1326305	30.81972092	30.23063743	30.46962459	0.529638588	0.973	0.186	PIS58700.1	STT3	Putative oligosaccharyltransferase complex component; flow model and rat catheter biofilm repressed
PIS51534.1	30.7485407	30.3230614	30.0802385	30.94837046	30.69755926	30.06781888	0.55170488	0.973	0.187	PIS51534.1	ERG20	Putative farnesyl pyrophosphate synthetase involved in isoprenoid and sterol biosynthesis, based on similarity to S. cerevisiae Erg20p; likely to be essential for growth, based on an insertional mutagenesis strategy
PIS54683.1	27.0072482	26.6057422	28.1744563	27.86090069	27.70502227	26.78272088	0.735829905	0.976	0.187	PIS54683.1	orf19.3222	Predicted vacuolar protein; rat catheter biofilm repressed; flow model biofilm repressed
PIS50397.1	27.7659023	27.9201842	26.7163163	27.16860786	27.65351711	28.14296257	0.644249437	0.975	0.188	PIS50397.1	orf19.3810	Ortholog(s) have methylenetetrahydrofolate dehydrogenase (NAD+) activity, role in folic acid-containing compound biosynthetic process, one-carbon metabolic process, purine nucleobase biosynthetic process and cytosol [illegible]
PIS58869.1	26.3959575	25.3602554	24.39427	23.51340443	26.12764555	27.0755663	0.825613839	0.978	0.189	PIS58869.1	HEM14	Putative protoporphyrinogen oxidase; involved in heme biosynthesis; predicted Kex2p substrate; iron regulated transcript; Yfh1-induced; Hap43-repressed; rat catheter biofilm repressed
PIS48641.1	24.5356044	24.2827663	24.4337329	24.76471193	24.39017083	24.66817357	0.643846336	0.975	0.19	PIS48641.1	YCG1	Putative condensin G

PIS55838.1	27.5470017	29.0316285	30.7362057	29.82511269	28.33681064	29.72478109	0.797363272	0.977	0.191	PIS55838.1	orf19.4248	Ortholog of S. cerevisiae YLR118C (alias Apt1); acyl-protein thioesterase responsible for depalmitoylation of Gpa1 in S. cerevisiae; regulated by Gcn4; repressed in response to amino acid starvation (3AT); rat catheter biofilm repressed
PIS49653.1	28.2651221	27.1364199	28.1249615	27.77803111	28.49997196	27.82215546	0.756517696	0.977	0.191	PIS49653.1	TRS33	Putative TRAPP complex subunit; constitutive expression independent of MTL or white-opaque status
PIS58431.1	25.067244	24.2728227	25.4122356	24.46696347	24.73444901	26.12750854	0.76297832	0.977	0.192	PIS58431.1	orf19.2850	Protein of unknown function; induced by nitric oxide independent of Yhb1p
PIS51650.1	25.6787419	23.0737895	25.2248628	23.77107485	25.67953549	25.10442775	0.863537452	0.978	0.193	PIS51650.1	RIC1	Ortholog of S. cerevisiae Ric1 guanyl-nucleotide exchange factor; mutant is viable; rat catheter biofilm repressed
PIS49600.1	24.9955398	24.6950427	25.085254	25.77336298	24.80162316	24.78410156	0.747337115	0.976	0.194	PIS49600.1	orf19.7397	Ortholog(s) have role in negative regulation of transcription by RNA polymerase II, positive regulation of transcription by RNA polymerase II, regulation of fungal-type cell wall organization and chromatin, nucleolus localization
PIS58114.1	28.9931545	29.0273591	29.4128143	29.40331035	29.6302636	28.98353845	0.531209043	0.973	0.195	PIS58114.1	LKH11	leukotriene A-4 hydrolase/aminopeptidase
PIS51235.1	31.0604	30.7345113	30.1636694	30.94524159	30.7732674	30.82485354	0.504246065	0.972	0.195	PIS51235.1	orf19.1967	Ortholog(s) have structural constituent of ribosome activity and mitochondrial large ribosomal subunit localization

PIS51175.1	28.7796941	28.1032152	28.691354	29.24156574	28.49181261	28.42666905	0.568821474	0.973	0.195	PIS51175.1	UBC4	Ortholog(s) have proteasome binding, protein-macromolecule adaptor activity, ubiquitin binding, ubiquitin conjugating enzyme activity, ubiquitin-protein transferase activity
PIS49517.1	28.9385698	28.2785632	28.7692936	28.44411428	28.81489403	29.31927825	0.72770508	0.976	0.197	PIS49517.1	CYR1	Class III adenylyl cyclase; mutant lacks cAMP; involved in regulation of filamentation, phenotypic switching and mating; mutant hyphal growth defect rescued by exogenous cAMP; downstream of Ras1p and CO2 signaling
PIS51127.1	28.7469964	29.1930569	28.5046909	28.82686882	28.84600145	29.36143859	0.553638711	0.973	0.197	PIS51127.1	VMA13	Predicted proton-transporting ATPase; predicted role in ATP hydrolysis coupled proton transport; rat catheter biofilm repressed
PIS58941.1	27.5980376	26.4328726	26.4938469	27.56957719	27.95140183	25.59743836	0.752677731	0.977	0.198	PIS58941.1	orf19.1092	Dolichol-P-Man dependent alpha(1-3) mannosyltransferase; role in the synthesis of dolichol-linked oligosaccharide donor for N-linked glycosylation of proteins; rat catheter biofilm repressed
PIS48474.1	27.6069821	26.212433	26.7479881	27.15551716	26.70229189	27.30259415	0.738458661	0.976	0.198	PIS48474.1	orf19.7556	Ortholog of C. dubliniensis CD36 : Cd36_34965, C. parapsilosis CDC317 : CPAR2_200910, C. auris B8441 : B9J08_005167 and Candida tenuis NRRL Y-1498 : CANTEDRAFT_95984
PIS56549.1	29.7609565	31.1279159	31.1006791	30.46599498	30.77365702	31.34381776	0.648186443	0.975	0.198	PIS56549.1	RPT2	Putative ATPase of the 19S regulatory particle of the 26S proteasome; oxidative stress-induced via Cap1; Spider biofilm repressed
PIS58363.1	24.8459774	24.2226865	27.113632	24.79754386	25.91967452	26.05795573	0.770558301	0.977	0.198	PIS58363.1	TAF14	YEATS domain-containing protein involved in transcription regulation; TFIIF and TFIID subunit; detects crotonylated lysine residues in histone H3; macrophage-induced protein; possibly required for yeast cell adherence to silicone substrate

PIS58825.1	28.7420766	28.5031935	28.2665695	28.61594453	29.03821648	28.45335962	0.520243836	0.972	0.199	PIS58825.1	orf19.7183	Ortholog(s) have EMC complex localization
PIS51773.1	24.9620735	25.4167735	25.8507698	25.0256918	25.59548496	26.20729658	0.703046378	0.976	0.2	PIS51773.1	RPN4	C2H2 transcription factor; regulator of proteasome genes; induced by Hap43, Spider biofilm, and within core stress response; null mutants show increased susceptibility to fluconazole and kill macrophages more slowly than wild type
PIS54516.1	30.860478	30.2771348	29.7629312	30.5002209	30.61153679	30.39109044	0.594462362	0.974	0.201	PIS54516.1	ADE4	Putative phosphoribosylpyrophosphate amidotransferase; flucytosine induced
PIS55749.1	25.0603315	24.6555761	24.1849257	24.51483393	24.35870936	25.63175751	0.62121835	0.974	0.201	PIS55749.1	orf19.3644	Protein of unknown function; Cyr1-repressed; rat catheter and Spider biofilm induced
PIS55043.1	25.0748074	23.0231638	24.5591951	24.06423483	24.43020433	24.76991258	0.809815824	0.977	0.202	PIS55043.1	orf19.2090	Ortholog of S. cerevisiae Ecm16, an essential DEAH-box ATP-dependent RNA helicase specific to the U3 snoRNP required for 18S rRNA synthesis; Hap43-induced; Spider biofilm induced
PIS52029.1	26.4418959	27.9781304	28.4021659	28.14587764	27.73646374	27.54701579	0.677535175	0.975	0.202	PIS52029.1	UTP21	Putative U3 snoRNP protein; Hap43-induce; physically interacts with TAP-tagged Nop1; Spider biofilm induced
PIS48545.1	32.0470099	32.3014108	33.0630027	32.62127088	32.65144654	32.74901827	0.620213412	0.974	0.203	PIS48545.1	FBA1	Fructose-bisphosphate aldolase; glycolytic enzyme; antigenic in murine/human infection; regulated by yeast-hypha switch; induced by Efg1, Gcn4, Hog1, fluconazole; phagocytosis-repressed; flow model biofilm induced; Spider biofilm

PIS55446.1	27.1127993	26.8645251	26.2627472	27.61451332	27.64433076	25.59468134	0.695613899	0.976	0.204	PIS55446.1	orf19.6679	Ortholog(s) have cytoplasm, nucleus localization
PIS54997.1	28.8647587	28.4745682	28.5189881	28.9620962	28.74885707	28.75849447	0.461915162	0.971	0.204	PIS54997.1	SEC1	Ortholog(s) have SNARE binding activity, role in exocytosis, positive regulation of vesicle fusion, vesicle docking involved in exocytosis and cellular bud neck, cellular bud tip, plasma membrane, prospore membrane localization
PIS56601.1	29.1611559	28.4048939	28.533319	29.10095975	29.14588607	28.46359475	0.565185311	0.973	0.204	PIS56601.1	TOR1	Protein similar to TOR family phosphatidylinositol kinases; mutation confers resistance to rapamycin; involved in regulation of ribosome protein synthesis, starvation response, and adhesion
PIS58480.1	27.0997597	26.0469883	26.1570491	26.72298971	26.54627498	26.65094503	0.60484423	0.974	0.205	PIS58480.1	CET1	mRNA 5'-triphosphatase; large subunit of mRNA capping enzyme; positively regulates Ceg1 activity; functional homolog of S. cerevisiae Cet1; Cet1 and Cgt1 form a 2:1 complex; flow model biofilm induced
PIS58779.1	26.5840885	25.3995135	24.6958015	26.84269924	25.5688219	24.89248749	0.720895695	0.976	0.208	PIS58779.1	DCC1	Protein with a predicted role in sister chromatid cohesion and telomere length maintenance; cell-cycle regulated periodic mRNA expression
PIS54704.1	26.5474116	25.8467303	25.3713152	24.48156303	26.61461362	27.30227469	0.842986839	0.978	0.211	PIS54704.1	MAK5	Putative nucleolar DEAD-box RNA helicase; oxidative stress-repressed via Cap1; repressed by prostaglandins
PIS58287.1	26.360296	25.3971049	26.742781	25.07126364	26.36513988	27.69695415	0.744497121	0.976	0.211	PIS58287.1	orf19.3027	Component of the RSC chromatin remodeling complex; putative DNA translocase; Spider biofilm repressed

PIS55053.1	29.5514049	28.8588854	29.8470786	29.74406237	29.67415314	29.47218834	0.872731325	0.978	0.211	PIS55053.1	orf19.4597	Putative F-actin-capping protein subunit beta; possibly an essential gene, disruptants not obtained by UAU1 method
PIS58238.1	28.5294195	28.0758312	27.9747332	28.63136015	28.41866113	28.16202946	0.513676199	0.972	0.211	PIS58238.1	SCH9	Protein kinase; involved in growth control, ribosomal protein synthesis, cell size, resistance to rapamycin,, chlamydospore formation, filamentous growth, and virulence; prevents hyphal growth in hypoxia at high CO2
PIS52102.1	22.9195527	23.0092089	22.5012732	27.8678912	20.46325524	20.73537824	0.903963824	0.979	0.212	PIS52102.1	orf19.3470	Putative flavodoxin; similar to S. cerevisiae Tyw1, an iron-sulfur protein required for synthesis of wybutosine modified tRNA; predicted Kex2p substrate; Spider biofilm induced
PIS51792.1	27.499415	24.0673486	27.6675293	27.38908913	27.69629876	24.78388958	0.840060918	0.978	0.212	PIS51792.1	orf19.6224	RTA domain protein; predicted role in response to stress; Spider biofilm induced
PIS58798.1	28.3405644	28.3353798	28.5013859	29.31788275	28.18426977	28.31363527	0.682245542	0.975	0.213	PIS58798.1	orf19.2335	Putative aspartyl aminopeptidase; stationary phase enriched protein; mutation confers hypersensitivity to 5-fluorouracil (5-FU); Hog1-induced; planktonic growth-induced; rat catheter and Spider biofilm repressed
PIS52342.1	31.0414957	30.7860062	30.6644549	30.90613695	31.13629264	31.08913043	0.512302302	0.972	0.213	PIS52342.1	orf19.5917.3	RNA binding protein required for export of poly(A)+ mRNA from the nucleus; Spider biofilm repressed
PIS54816.1	23.0777272	24.4848791	25.7311877	24.49219212	26.19065385	23.25392325	0.767097255	0.977	0.214	PIS54816.1	POL3	Large subunit of DNA polymerase III; partially complements defects of an S. cerevisiae cdc2 mutant; differing reports about periodic (G1/S) or non-periodic mRNA expression through cell cycle; Hap43p-repressed

PIS54871.1	28.6205865	28.9976809	28.0110416	29.96848303	28.03659026	28.27051554	0.660047901	0.975	0.215	PIS54871.1	orf19.3319	Putative thioredoxin; Spider biofilm repressed
PIS58845.1	27.2122428	26.6020301	27.5344985	26.75312914	27.46629806	27.78197826	0.624665162	0.974	0.218	PIS58845.1	FZO1	Mitochondrial biogenesis protein; rat catheter and Spider biofilm induced
PIS55672.1	25.0994696	28.0244136	28.3190985	26.21987428	27.18415399	28.6974374	0.82949876	0.978	0.219	PIS55672.1	orf19.5455	Ortholog(s) have GTPase regulator activity, mRNA binding activity
PIS48554.1	27.5215134	26.4650796	25.2475628	27.02166604	26.62758956	26.24456303	0.675339616	0.975	0.22	PIS48554.1	RER2	Putative cis-prenyltransferase involved in dolichol synthesis; participates in endoplasmic reticulum (ER) protein sorting; flow model biofilm induced
PIS51561.1	30.2683608	29.8949191	29.4533214	30.54268422	29.94890276	29.78855304	0.471434054	0.971	0.221	PIS51561.1	IST2	Ortholog(s) have lipid binding activity and role in endoplasmic reticulum membrane organization, protein localization to plasma membrane, regulation of phosphatidylinositol dephosphorylation
PIS58048.1	24.3644247	23.9068108	23.3693126	24.43989119	22.90942248	24.95932765	0.703788114	0.976	0.223	PIS58048.1	orf19.7621	Putative subunit of an alternative replication factor C complex; role in DNA replication, genome integrity, homologous recombination-mediated repair and telomere homeostasis;
PIS51030.1	31.595335	31.703618	31.1459763	31.93699413	31.77487883	31.40299229	0.504975465	0.972	0.223	PIS51030.1	PMM1	Phosphomannomutase; enzyme of O- and N-linked mannosylation; interconverts mannose-6-phosphate and mannose-l-phosphate; functional homolog of S. cerevisiae Sec53; antigenic in mice; Hap43-induced; flow model and Spider biofilm repressed

PIS51310.1	27.9878483	28.4631906	28.4494414	29.16743435	28.13421476	28.27500302	0.547222356	0.973	0.225	PIS51310.1	CCR4	Component of the Ccr4-Pop2 mRNA deadenylase; transposon mutation affects filamentous growth
PIS52280.1	30.9760965	31.1119908	30.9466028	31.23364709	30.907442	31.56795007	0.402580927	0.969	0.225	PIS52280.1	GCD11	Gamma subunit of translation initiation factor eIF2; involved in identification of the start codon; likely essential for growth, based on an insertional mutagenesis strategy; Spider biofilm repressed
PIS51620.1	29.8278798	29.8248719	29.9357842	29.93902283	30.19991228	30.14375706	0.363557615	0.968	0.231	PIS51620.1	CBR1	Putative cytochrome B5 reductase; plasma membrane-localized
PIS49723.1	25.6826424	26.4316778	24.2202746	25.83730144	25.56852792	25.62423062	0.890227684	0.978	0.232	PIS49723.1	orf19.6797	Ortholog of C. dubliniensis CD36 : Cd36_86960, C. parapsilosis CDC317 : CPAR2_808800, C. auris B8441 : B9J08_004750 and Candida tenuis NRRL Y-1498 : CANTEDRAFT_113621
PIS58380.1	27.7203314	27.8779686	29.1718865	28.61878724	28.12126516	28.72759129	0.674891994	0.975	0.232	PIS58380.1	orf19.6883	Putative oxysterol binding protein; probable peripheral membrane protein of the Golgi complex; involved in invasive growth and plasma membrane organization; flow model and Spider biofilm repressed
PIS49805.1	25.0340247	26.547415	27.3694102	26.89481814	26.59135504	26.16401088	0.66724452	0.975	0.233	PIS49805.1	ECM15	Protein of unknown function; predicted role in cell wall organization; Hap43-repressed; caspofungin repressed; Spider biofilm induced; rat catheter biofilm repressed
PIS54534.1	26.4833261	26.4765046	26.0707457	27.90665185	26.92357876	24.90067142	0.717478037	0.976	0.233	PIS54534.1	orf19.4798	Component of a complex with Ypp1p and Stt4p that is required for phosphatidylinositol-4-phosphate, PI(4)P, in plasma membrane; required for invasive growth and cell wall organization

PIS58838.1	22.1805537	23.7212801	24.6286894	23.96640571	22.7975061	24.47841416	0.72237832	0.976	0.237	PIS58838.1	orf19.398	Ortholog of C. dubliniensis CD36 : Cd36_08040, C. parapsilosis CDC317 : CPAR2_207180, C. auris B8441 : B9J08_000292 and Candida tenuis NRRL Y-1498 : CANTEDRAFT_114140
PIS56570.1	26.2918203	25.6671169	27.4144978	26.78893141	26.41224567	26.88293379	0.654113271	0.975	0.237	PIS56570.1	SEF1	Zn2-Cys6 transcription factor; regulates iron uptake; negatively regulated by Sfu1p, positively regulated by Tbf1; promotes virulence in mice; mutants display decreased colonization of mouse kidneys; Spider biofilm induced
PIS51839.1	29.0796899	30.0738513	30.0689781	29.99053653	30.00748363	29.94484141	0.641041969	0.975	0.24	PIS51839.1	ILV5	Ketol-acid reductoisomerase; antigenic; regulated by Gcn4; GlcNAc, amino acid starvation (3-AT)-induced; macrophage-repressed protein; protein present in exponential and stationary phase; flow model and Spider biofilm repressed
PIS58755.1	27.0994648	27.3924652	28.0725067	26.88658334	27.50603274	28.89202513	0.661096376	0.975	0.24	PIS58755.1	orf19.1637	Ortholog of C. dubliniensis CD36 : Cd36_82070, C. parapsilosis CDC317 : CPAR2_202790, C. auris B8441 : B9J08_000205 and Candida tenuis NRRL Y-1498 : CANTEDRAFT_114065
PIS56840.1	31.4435915	31.4072567	31.1644554	31.39708932	31.46589922	31.87837687	0.39324028	0.969	0.242	PIS56840.1	ZUO1	Ortholog of S. cerevisiae Zuo1; a cytosolic ribosome-associated chaperone; likely to be essential for growth, based on an insertional mutagenesis strategy; Spider biofilm repressed
PIS54967.1	31.3806769	31.2498012	31.0568249	31.6465544	31.44875268	31.3196121	0.348114692	0.967	0.243	PIS54967.1	DED81	Putative tRNA-Asn synthetase; genes encoding ribosomal subunits, translation factors, tRNA synthetases are downregulated upon phagocytosis by murine macrophage; protein enriched in stationary phase yeast cultures
PIS50583.1	28.5706853	27.4980572	27.4351962	28.80298396	27.62096159	27.80749389	0.663764043	0.975	0.243	PIS50583.1	orf19.4184	Protein with a predicted role in clathrin cage assembly; Hap43-repressed; Spider biofilm repressed

PIS51852.1	28.5780803	28.6321209	28.7657802	28.7461617	28.85254042	29.10906665	0.435175217	0.97	0.244	PIS51852.1	CMK2	Putative calmodulin-dependent protein kinase; involved in cell wall integrity and oxidative stress response
PIS48311.1	23.4105557	25.2297231	25.2850078	24.99316226	24.69630484	24.97050672	0.715193421	0.976	0.245	PIS48311.1	orf19.3528	Protein of unknown function; Spider biofilm induced
PIS49744.1	29.061648	28.5227474	28.8663427	29.17141734	29.14342381	28.87943732	0.584477656	0.974	0.248	PIS49744.1	orf19.6769	Ortholog of S. cerevisiae : OCA5, C. glabrata CBS138 : CAGL0E03784g, C. dubliniensis CD36 : Cd36_87200, C. parapsilosis CDC317 : CPAR2_809020 and C. auris B8441 : B9J08_004771
PIS52143.1	24.9643773	24.254774	22.607227	23.75270878	24.52556525	24.29658249	0.67231025	0.975	0.249	PIS52143.1	ARG11	Putative ornithine transporter of the mitochondrial inner membrane; induced during the mating process
PIS56675.1	24.9643773	24.254774	22.607227	23.75270878	24.52556525	24.29658249	0.794497201	0.977	0.249	PIS56675.1	orf19.4117	Ortholog of C. dubliniensis CD36 : Cd36_20450, C. parapsilosis CDC317 : CPAR2_104160, C. auris B8441 : B9J08_001214 and Candida tenuis NRRL Y-1498 : CANTEDRAFT_95516
PIS51601.1	23.1877886	26.6376956	25.9935692	26.83774816	25.68907352	24.04038018	0.785159215	0.977	0.249	PIS51601.1	REG1	Putative protein phosphatase regulatory subunit; Hap43-repressed gene; macrophage/pseudohyphal-induced; possibly regulated upon hyphal formation; flow model biofilm induced
PIS58692.1	28.9136872	27.9265256	28.2369674	28.77370825	28.72258856	28.33013447	0.570248743	0.973	0.25	PIS58692.1	COX13	Cytochrome c oxidase; flucytosine induced; repressed by nitric oxide

PIS51588.1	23.5300428	23.127405	23.3537732	24.28195285	23.46477405	23.01947354	0.522668193	0.972	0.252	PIS51588.1	orf19.5213	Putative protein of unknown function; transcript is upregulated in clinical isolates from HIV+ patients with oral candidiasis
PIS55782.1	27.3669996	27.692067	28.0176664	28.47966559	27.96087326	27.39170313	0.430071194	0.97	0.252	PIS55782.1	SPC98	Putative component of the microtubule-nucleating Tub4p (gamma-tubulin) complex; periodic mRNA expression, peak at cell-cycle S/G2 phase
PIS54847.1	26.8363483	26.5843768	26.4080254	26.93613748	26.75596322	26.89873934	0.584784097	0.974	0.254	PIS54847.1	BET4	Ortholog(s) have Rab geranylgeranyltransferase activity and role in endoplasmic reticulum to Golgi vesicle-mediated transport, protein geranylgeranylation, protein targeting to membrane
PIS58782.1	24.9645616	26.2773472	24.1332228	25.30207247	26.1242923	24.71327121	0.656874835	0.975	0.255	PIS58782.1	orf19.3862	Putative intracellular transport protein; heterozygous null mutant displays sensitivity to rapamycin; expression upregulated during growth in the mouse cecum
PIS51203.1	30.3056548	29.6377419	28.7742555	29.515471	30.2561878	29.7138444	0.54635501	0.973	0.256	PIS51203.1	orf19.5686	Protein of unknown function; Spider biofilm induced
PIS54660.1	29.2868035	27.2561406	28.1960572	27.96987633	28.80048714	28.73775304	0.591082037	0.974	0.256	PIS54660.1	orf19.6602	Ortholog(s) have role in ubiquinone biosynthetic process, ubiquinone-6 biosynthetic process and mitochondrial inner membrane, mitochondrion localization
PIS51202.1	27.8649823	27.113156	26.4730444	27.2738441	27.46204004	27.4854645	0.590063964	0.974	0.257	PIS51202.1	orf19.5782	Ortholog(s) have phospholipase activity, role in cardiolipin metabolic process, phosphatidylethanolamine metabolic process, phospholipid metabolic process and mitochondrial matrix localization

PIS56881.1	30.3753463	30.3716139	30.2681391	30.86309391	30.49159811	30.43879267	0.354657809	0.967	0.259	PIS56881.1	SLA2	Actin binding protein with roles in growth control and morphogenesis; required for alkaline pH-induced hyphal formation; localized to actin patches; rat catheter biofilm repressed
PIS48713.1	25.7230602	25.7496499	23.6779336	24.90683363	25.25951234	25.76386883	0.704077771	0.976	0.26	PIS48713.1	orf19.7038	Ortholog(s) have phosphatidylinositol-3-phosphate binding activity, role in plasma membrane tubulation, protein targeting to vacuole, retrograde transport, endosome to Golgi and cytoplasm, endosome, nucleus localization
PIS56901.1	24.678223	24.6322178	24.4703533	24.85713202	24.83280175	24.87358944	0.482507034	0.971	0.261	PIS56901.1	THR1	Putative homoserine kinase; regulated by Tup1; amphotericin B repressed; regulated by Gcn2 and Gcn4; Spider biofilm repressed
PIS51586.1	29.9392733	29.9345277	29.5720698	30.04632233	30.29172649	29.89247354	0.48545447	0.972	0.262	PIS51586.1	IDP1	Putative isocitrate dehydrogenase; transcriptionally induced by interaction with macrophage; alkaline induced; Spider biofilm repressed
PIS58143.1	28.0441016	26.8325093	27.1443009	27.44442317	27.37050834	27.99143619	0.738418458	0.976	0.262	PIS58143.1	SEC10	Ortholog(s) have role in Golgi to plasma membrane transport, exocytosis and cell division site, cell tip, exocyst localization
PIS50614.1	25.9252607	25.0016816	25.0005868	26.66423955	26.4875159	23.56488751	0.804815575	0.977	0.263	PIS50614.1	orf19.6970	Ortholog of C. dubliniensis CD36 : Cd36_85310, C. parapsilosis CDC317 : CPAR2_807370, C. auris B8441 : B9J08_004442 and Candida tenuis NRRL Y-1498 : CANTEDRAFT_115544
PIS58456.1	27.8522576	27.5856337	28.0389612	28.1556753	27.8362155	28.27744002	0.409251585	0.969	0.264	PIS58456.1	orf19.3689	Putative protein similar to 6-phosphofructo-2-kinase/fructose-2,6-bisphosphatase; expression downregulated in an ssr1 null mutant

PIS56685.1	25.3089387	27.5485953	26.3496455	26.11440289	27.00588037	26.88243068	0.754573487	0.977	0.265	PIS56685.1	ECM17	Putative sulfite reductase beta subunit; role in cell wall biogenesis; regulated by Tsa1/Tsa1B in H2O2 stress; Gcn4-regulated; Tbf1-activated; Hap43-repressed; Spider, flow, F-12/CO2 model biofilm induced
PIS49578.1	23.2677821	24.3824761	25.7226882	24.96830801	24.45181685	24.74911416	0.717840557	0.976	0.265	PIS49578.1	OPT1	Oligopeptide transporter; transports 3-to-5-residue peptides; alleles are distinct, one has intron; suppresses S. cerevisiae ptr2-2 mutant defects; induced by BSA or peptides; Stp3p, Hog1p regulated; flow model biofilm induced
PIS49823.1	26.9372227	25.5626451	27.8573531	27.4923447	27.27233837	26.38690059	0.653781033	0.975	0.265	PIS49823.1	orf19.4362	Ortholog(s) have membrane protein dislocase activity, role in protein targeting to mitochondrion and mitochondrial outer membrane, peroxisomal membrane localization
PIS54819.1	24.4735943	26.8025314	27.3732727	25.34618127	26.99738219	27.10132732	0.702730587	0.976	0.265	PIS54819.1	PEX14	Ortholog(s) have protein-macromolecule adaptor activity, role in protein import into peroxisome matrix, docking and peroxisomal importomer complex, peroxisomal membrane, peroxisome localization
PIS58957.1	28.2377331	27.9386421	28.2269586	28.41692022	28.28253735	28.50237536	0.313969897	0.966	0.266	PIS58957.1	CDC24	GDP-GTP exchange factor for Cdc42p; phosphorylated; required for maintenance of hyphal growth; misexpression blocks hyphal growth and causes avirulence in a mouse model of systemic infection; antigenic during human oral infection
PIS48753.1	28.3108301	28.3682376	26.6312436	28.53951555	27.84507254	27.72356376	0.613089337	0.974	0.266	PIS48753.1	GCN3	Putative translation initiator; downregulated in the presence of human whole blood or polymorphonuclear (PMN) cells
PIS55493.1	29.5865425	29.0160334	28.4615257	29.23128188	29.26080108	29.36961139	0.431676732	0.97	0.266	PIS55493.1	orf19.1229	Ortholog(s) have nuclear export signal receptor activity, role in protein export from nucleus, snRNA import into nucleus and nuclear envelope, nuclear periphery localization

PIS49497.1	27.7300714	26.7928385	26.6549663	27.51987083	27.69837603	26.76863541	0.593340043	0.974	0.27	PIS49497.1	orf19.426	Ortholog of C. dubliniensis CD36 : Cd36_05120, C. parapsilosis CDC317 : CPAR2_107740, C. auris B8441 : B9J08_004520 and Candida tenuis NRRL Y-1498 : CANTEDRAFT_136277
PIS55032.1	30.0167281	29.8200194	28.4399342	29.70807883	29.7716253	29.60868876	0.557454317	0.973	0.271	PIS55032.1	orf19.1514	Ortholog(s) have enzyme binding, phosphatidylinositol-4-phosphate binding activity
PIS58026.1	30.1671236	29.7774654	29.7533051	30.02351092	30.35731015	30.12915416	0.340249273	0.967	0.271	PIS58026.1	orf19.5777	Protein of unknown function; F-12/CO2 early biofilm induced
PIS48253.1	28.597818	29.075734	29.2852573	29.57035607	29.29322454	28.91063496	0.43266021	0.97	0.272	PIS48253.1	LRO1	Acyltransferase that catalyzes diacylglycerol esterification of phospholipids; role in lipid storage, triglyceride biosynthesis; flow model biofilm repressed
PIS58085.1	24.8652495	25.3658916	24.0095401	23.82696086	25.11269826	26.12194688	0.719983318	0.976	0.274	PIS58085.1	orf19.6134	Ortholog(s) have role in ER-dependent peroxisome organization, retrograde vesicle-mediated transport, Golgi to endoplasmic reticulum, vesicle-mediated transport
PIS52020.1	24.650426	23.8693633	24.0931398	24.79698644	24.39274028	24.24726953	0.564254663	0.973	0.275	PIS52020.1	COQ5	Putative methyltransferase of ubiquinone biosynthesis; regulated by Gcn4; repressed by amino acid starvation (3-AT), Hap43; induced upon adherence to polystyrene; Spider biofilm repressed
PIS54575.1	27.2965826	27.2016651	28.3249039	27.37779836	27.77972383	28.49150395	0.485382563	0.972	0.275	PIS54575.1	MET3	ATP sulfurlyase; sulfate assimilation; repressed by Met, Cys, Sfu1, or in fluconazole-resistant isolate; Hog1, caspofungin, white phase-induced; induced on biofilm formation, even in presence of Met and Cys; Spider, F-12/CO2 biofilm induced

PIS51334.1	28.2859206	26.2167935	26.6246102	28.27992842	27.24105298	26.43244177	0.705865474	0.976	0.275	PIS51334.1	SWI6	Putative component of the MBF and SBF transcription complexes involved in G1/S cell-cycle progression; periodic mRNA expression, peak at cell-cycle G1/S phase
PIS48276.1	24.7799712	25.1637147	25.7885046	26.25912119	24.95070747	25.34908955	0.638310048	0.975	0.276	PIS48276.1	SHE9	Protein with similarity to S. cerevisiae She9p, which inhibits growth when overproduced; gene has possible growth-regulation element; fungal-specific (no human or murine homolog)
PIS54504.1	29.9338016	29.6903988	29.0911896	30.30983745	29.81377312	29.422598	0.441918468	0.97	0.277	PIS54504.1	orf19.5965	Ortholog(s) have ubiquitin-ubiquitin ligase activity
PIS51777.1	28.9126638	29.0438045	29.8860438	28.09489139	30.08875019	30.49045142	0.606141873	0.974	0.277	PIS51777.1	PHO85	Functional homolog of S. cerevisiae Pho85p, a cyclin-dependent kinase that regulates transcription of PHO genes involved in phosphate metabolism; necessary for geldanamycin-induced filamentation; gene has intron
PIS56854.1	26.1674894	25.1636236	25.25351	27.04529969	25.69130981	24.67788525	0.706598467	0.976	0.277	PIS56854.1	TFG1	Protein similar to S. cerevisiae Tfg1p, which is part of transcription factor TFIIF; transposon mutation affects filamentous growth; possibly an essential gene, disruptants not obtained by UAU1 method
PIS48342.1	19.4221045	23.1557613	24.2822608	20.90926307	22.99221213	23.79349171	0.799354822	0.977	0.278	PIS48342.1	SMP3	Mannosyltransferase of glycosylphosphatidylinositol (GPI) biosynthesis; catalyzes mannosylation of Man3-GPI precursor; essential for viability; 8-9 transmembrane regions predicted; has HQEXRF motif; functional homolog of S. cerevisiae
PIS56698.1	19.8366752	22.9851259	18.004753	19.89367545	20.83734806	20.93385008	0.805973362	0.977	0.279	PIS56698.1	orf19.1137	Thymidylate kinase of unknown role; forms a dimer; potential target for antifungal drugs

PIS49771.1	27.8651644	27.8892549	28.1350586	27.88186982	28.09566059	28.7532714	0.439961181	0.97	0.28	PIS49771.1	ARG3	Putative ornithine carbamoyltransferase; Gcn4-regulated; Hap43-induced; repressed in alkalinizing medium; rat catheter and Spider biofilm induced
PIS58885.1	26.1629838	27.2159071	28.1720869	27.34570047	27.37657266	27.66877477	0.806920698	0.977	0.28	PIS58885.1	YMC2	Putative mitochondrial carrier protein; Gcn4-regulated; F-12/ CO2 early biofilm induced; Spider biofilm induced
PIS48331.1	28.3244025	28.12647	27.5141977	27.86542541	28.61744273	28.32557688	0.545133373	0.973	0.281	PIS48331.1	orf19.5442	Ortholog of C. dubliniensis CD36 : Cd36_80240, C. parapsilosis CDC317 : CPAR2_504040, C. auris B8441 : B9J08_005021 and Candida tenuis NRRL Y-1498 : CANTEDRAFT_130872
PIS54746.1	25.1256418	25.9778337	26.153486	26.43531539	24.68572304	26.98213754	0.655829643	0.975	0.282	PIS54746.1	CUE5	Predicted ubiquitin-binding protein; rat catheter biofilm repressed
PIS50558.1	25.9985277	24.7836393	26.074942	24.61496524	26.84600829	26.24286401	0.649160719	0.975	0.282	PIS50558.1	orf19.3945	Predicted COP9 signalosome complex subunit 12; flow model biofilm induced
PIS58398.1	25.7119749	24.1064169	25.3683437	26.3964133	25.47991562	24.16679244	0.595944952	0.974	0.285	PIS58398.1	orf19.2517	Predicted membrane transporter, member of the drug:proton antiporter (12 spanner) (DHA1) family, major facilitator superfamily (MFS)
PIS52374.1	25.9409554	25.919178	25.8797324	25.7049733	26.23957395	26.65082701	0.565416673	0.973	0.285	PIS52374.1	orf19.719	Putative ubiquitin protein ligase with ubiquitin-protein transferase activity; null mutant is sensitive to proteotoxic stress and shows differential expression of other proteins likely involved in ER-associated protein catabolism

PIS54679.1	29.0445805	28.4334874	27.8248351	29.20945329	28.78214114	28.17007058	0.562872033	0.973	0.286	PIS54679.1	HAT1	Hat1-Hat2 histone acetyltransferase complex subunit; involved in DNA damage repair and morphogenesis; mutations cause constitutive pseudohyphal growth, white to opaque switch, caspofungin sensitivity; rat catheter, Spider biofilm repressed
PIS51331.1	27.0762669	27.265597	27.4787357	27.58670877	27.5867088	27.50891979	0.491709744	0.972	0.287	PIS51331.1	orf19.5275	Ortholog(s) have ATPase activator activity, protein-macromolecule adaptor activity and role in late endosome to vacuole transport, late endosome to vacuole transport via multivesicular body sorting pathway
PIS58224.1	28.6873264	28.9777716	28.5389051	29.13312948	29.07816308	28.85428043	0.401006012	0.969	0.287	PIS58224.1	orf19.7011	Ortholog(s) have role in maturation of SSU-rRNA from tricistronic rRNA transcript (SSU-rRNA, 5.8S rRNA, LSU-rRNA) and nucleus, preribosome, small subunit precursor localization
PIS51047.1	29.5800633	29.4604674	29.7856964	29.60953839	29.69817046	30.38762601	0.676790984	0.975	0.29	PIS51047.1	FLC1	Protein involved in heme uptake; putative FAD transporter, similar to S. cerevisiae Flc1; regulated by iron; macrophage-induced; mutant defective in filamentous growth; Spider biofilm induced
PIS59029.1	29.959035	27.465713	27.1200009	27.65795113	29.16300045	28.59323439	0.646101601	0.975	0.29	PIS59029.1	PHO91	Putative low-affinity phosphate transporter; fungal-specific (no human or murine homolog)
PIS58296.1	33.3902091	30.6783682	30.0488536	29.83343486	32.80567053	32.35039988	0.732173515	0.976	0.291	PIS58296.1	RVS167	SH3-domain- and BAR domain-containing protein involved in endocytosis; null mutant exhibits defects in hyphal growth, virulence, cell wall integrity, and actin patch localization; cosediments with phosphorylated Myo5p
PIS58492.1	23.1290546	24.1749038	24.743551	24.15383303	24.61320875	24.15538373	0.535019828	0.973	0.292	PIS58492.1	SEN2	Putative tRNA splicing endonuclease subunit; mutation confers hypersensitivity to toxic ergosterol analog and to amphotericin B; 5'-UTR intron; Hap43-induced; Spider biofilm induced

PIS51648.1	29.5011323	29.3673741	30.0809713	29.97594228	29.86264452	29.98660602	0.380006481	0.968	0.292	PIS51648.1	TUB2	Beta-tubulin; functional homolog of ScTub2; overproduction makes S. cerevisiae inviable; has two introns; GlcNAc, hypha fluconazole-induced; slow growth, ectopic expression increases white-to opaque switch; rat catheter biofilm repressed
PIS49580.1	28.113794	28.1014234	28.2073753	28.28200822	28.24618803	28.77874476	0.326467414	0.966	0.295	PIS49580.1	orf19.5689	Epsilon-COP subunit of the coatomer; regulates retrograde Golgi-to-ER protein traffic; Spider biofilm repressed
PIS52162.1	24.7319237	24.513616	24.7610077	25.26379646	24.81175937	24.82182939	0.460511045	0.971	0.297	PIS52162.1	orf19.3130	Ortholog of S. cerevisiae Gpn3 a GTPase with a role in biogenesis of RNA pol II and polIII; possibly an essential gene, disruptants not obtained by UAU1 method
PIS54723.1	26.6324792	25.6235383	27.1023704	26.82238183	26.50938053	26.92111569	0.537072886	0.973	0.298	PIS54723.1	orf19.6358	Predicted ubiquitin conjugating enzyme involved in DNA damage response; forms a complex with Ubc13p
PIS48530.1	27.3094411	27.391826	26.7762924	26.51731481	28.21777373	27.63703572	0.58708442	0.974	0.298	PIS48530.1	orf19.6860	Phosphatidylinositol synthase, essential protein of lipid metabolism; involved in maintenance of endoplasmic reticulum, cell wall integrity and virulence
PIS49641.1	27.6954538	27.0406299	27.091769	27.35741358	27.84394716	27.52783865	0.557324899	0.973	0.3	PIS49641.1	DAO1	Putative D-amino acid oxidase; transcription is regulated upon yeast-hyphal switch
PIS50334.1	28.3959199	27.5652644	28.0671984	28.64400561	28.31393825	27.97028057	0.587641673	0.974	0.3	PIS50334.1	FAD3	Omega-3 fatty acid desaturase; production of alpha-linolenic acid, a major component of membranes; caspofungin induced; Plc1-regulated; colony morphology-related gene regulation by Ssn6; Spider biofilm induced, flow model biofilm repressed

PIS54578.1	29.5639782	29.3640608	28.9705219	29.57053345	29.82832994	29.39896811	0.393069907	0.969	0.3	PIS54578.1	UTP20	Putative snoRNA-binding protein; S. cerevisiae Utp20 ortholog; likely essential for growth; repressed in core stress response; mutation confers resistance to 5-fluorocytosine (5-FC) and parnafungin
PIS56856.1	26.2528367	27.0468424	26.8168338	26.21042117	27.61030995	27.19918225	0.529731025	0.973	0.301	PIS56856.1	orf19.4610	Predicted metallocarboxypeptidase; role in proteolysis; rat catheter biofilm repressed
PIS48655.1	23.1924695	24.1964081	23.97928	24.34117697	23.85611245	24.07438796	0.590768423	0.974	0.301	PIS48655.1	SET1	Lysine histone methyltransferase; methylates histone H3 K4; regulates of white-opaque switch, epithelial cell adhesion, agar-embedded filamentation, virulence in mice; unique N-terminus immunogenic in human; rat catheter biofilm repressed
PIS51038.1	27.8469673	27.4158371	27.1207967	28.6391136	27.59347249	27.05651637	0.671407924	0.975	0.302	PIS51038.1	TPM2	Putative tropomyosin isoform 2; regulated by Gcn4; repressed by amino acid starvation; macrophage-induced; protein levels decrease in stationary cells; Hap43-induced; rat catheter and Spider biofilm repressed
PIS56867.1	30.8709738	30.9159373	30.9012079	31.33202534	31.22239576	31.0391354	0.289426659	0.965	0.302	PIS56867.1	TYS1	Putative tRNA-Tyr synthetase; downregulated upon phagocytosis by murine macrophages; stationary phase enriched protein; Spider biofilm repressed
PIS48716.1	28.3659196	27.1885635	26.9778989	27.57744401	28.19058674	27.67316172	0.51894517	0.972	0.303	PIS48716.1	RFC2	Putative heteropentameric replication factor C subunit; periodic mRNA expression, peak at cell-cycle G1/S phase
PIS56654.1	26.8028671	27.4005091	27.8670817	26.63711632	28.15497391	28.19159723	0.647682867	0.975	0.304	PIS56654.1	SNF2	Protein involved in transcriptional regulation; ortholog of S. cerevisiae Snf2p, which is the catalytic subunit of the SWI/SNF chromatin remodeling complex; interacts with Swi1p; SWI/SNF complex is essential for hyphal growth and virulence

PIS51843.1	25.1083968	25.7083192	26.1150173	26.0726807	25.96966245	25.8034318	0.484549525	0.971	0.305	PIS51843.1	OCA1	Putative protein phosphatase of the PTP family (tyrosine-specific); ortholog of S. cerevisiae Oca1; mutant is viable; mutant shows virulence defect
PIS58416.1	28.1691362	29.2152889	29.7419236	28.87318436	29.58945352	29.58067866	0.665714967	0.975	0.306	PIS58416.1	EFB1	Translation elongation factor EF-1 beta; repressed by human whole blood or PMNs; macrophage/pseudohyphal-induced; antigenic in mouse; farnesol-downregulated; snoRNA snR18 is encoded within the EFB1 intron; Spider biofilm repressed
PIS56917.1	24.3083671	24.0428577	23.4898167	24.42449553	23.77848286	24.56698903	0.509761503	0.972	0.31	PIS56917.1	MYO1	Component of actomyosin ring at neck of newly-emerged bud
PIS51212.1	22.275	24.572044	27.3830746	24.60604858	24.78079653	25.77272464	0.792747715	0.977	0.31	PIS51212.1	orf19.2275	Putative mitochondrial ribosomal protein; predicted role in aerobic respiration; Spider biofilm repressed
PIS56727.1	26.0027984	25.1322046	25.4657489	24.9673405	26.22241517	26.34138194	0.620874843	0.974	0.31	PIS56727.1	orf19.6100	Cardiolipin synthase; ortholog of S. cerevisiae Crd1; transcript is upregulated in clinical isolates from HIV+ patients with oral candidiasis; mutants are viable; Spider biofilm repressed
PIS51066.1	26.085348	27.6047669	27.8736946	27.33490215	27.55806354	27.60454934	0.50710137	0.972	0.311	PIS51066.1	LAP41	Putative aminopeptidase yscl precursor; mutant is viable; protein present in exponential and stationary growth phase yeast cultures; Spider biofilm repressed
PIS51993.1	28.1792901	27.2669576	26.1874296	27.2471727	27.95745131	27.36249492	0.520874185	0.972	0.311	PIS51993.1	NPR1	Predicted serine/threonine protein kinase, involved in regulation of ammonium transport; induced in core stress response; Hap43p-repressed gene

PIS54882.1	30.2501403	31.4296671	31.0927551	29.89144924	31.63722048	32.17608716	0.689541164	0.976	0.311	PIS54882.1	orf19.4947	Mitochondrial membrane protein of unknown function; Spider biofilm induced
PIS49651.1	23.202592	23.5235406	24.3152201	24.39484188	24.40410658	23.17575932	0.509043064	0.972	0.311	PIS49651.1	WHI3	Putative RNA binding protein; induced during infection of murine kidney, compared to growth in vitro; has murine homolog
PIS49495.1	23.8984135	23.9192521	24.0664665	24.33932516	23.98305279	24.50003756	0.438225677	0.97	0.313	PIS49495.1	TRP99	Putative thioredoxin peroxidase/alkyl hydroperoxide reductase; induced in low iron; regulated by Gcn4; induced in response to amino acid starvation (3-AT treatment)
PIS51575.1	30.5022838	30.2365987	31.0355711	31.09137629	31.10995741	30.51549386	0.651234386	0.975	0.314	PIS51575.1	orf19.6062.3	Mitochondrial protein; component of the mitochondrial inner membrane organizing system ; role in maintenance of crista junctions and inner membrane architecture; Spider biofilm repressed
PIS48513.1	27.8905742	29.47257	30.3884708	29.52514362	29.42697771	29.74499345	0.652981657	0.975	0.315	PIS48513.1	PDB1	Putative pyruvate dehydrogenase; fluconazole-induced; protein level decreases in stationary phase cultures; Spider biofilm repressed
PIS58449.1	23.6176198	23.359321	23.2348483	23.31691164	24.23079953	23.61294157	0.442055165	0.97	0.316	PIS58449.1	CWH8	Putative dolichyl pyrophosphate (Dol-P-P) phosphatase; ketoconazole-induced; expression is increased in a fluconazole-resistant isolate; clade-associated gene expression; Hap43p-induced gene
PIS51086.1	28.2930331	27.999665	28.4235537	28.61507231	28.42888927	28.62095393	0.351496194	0.967	0.316	PIS51086.1	orf19.4287	Putative oxidoreductase; Hap43-repressed gene; clade-associated gene expression

PIS55695.1	26.673967	25.4679126	26.7604804	26.17833103	27.14255114	26.53522151	0.715178188	0.976	0.318	PIS55695.1	orf19.3135	Ortholog(s) have protein-macromolecule adaptor activity
PIS52160.1	28.0465547	28.0419675	27.8537564	28.75097092	27.96675228	28.18553281	0.272176035	0.964	0.32	PIS52160.1	orf19.3128	Ortholog(s) have SNARE binding, syntaxin binding activity
PIS48533.1	25.9637464	25.7461954	25.0502947	25.53350246	26.24184708	25.94696029	0.450023007	0.971	0.321	PIS48533.1	orf19.4370	Protein of unknown function; induced by nitric oxide; oxidative stress-induced via Cap1; fungal-specific (no human or murine homolog)
PIS51413.1	28.7691857	29.2609607	28.3444964	29.46941186	29.01169126	28.86531691	0.362758084	0.968	0.324	PIS51413.1	ADE1	Phosphoribosylaminoimadazole succinocarboxamide synthetase, enzyme of adenine biosynthesis; not induced in GCN response, unlike the S. cerevisiae ortholog; fungal-specific (no human or murine homolog); levels decrease in stationary phase
PIS58467.1	30.3644818	31.2050173	31.0868399	27.72172874	32.39864102	33.50672936	0.778226903	0.977	0.324	PIS58467.1	orf19.1144	Protein with SEL-1 like protein domain; early-stage flow model biofilm induced
PIS54955.1	31.9570708	31.8033669	31.1893364	32.1511961	31.97217085	31.80495177	0.375824596	0.968	0.326	PIS54955.1	MDG1	Ortholog(s) have role in pheromone-dependent signal transduction involved in conjugation with cellular fusion and eisosome, plasma membrane localization
PIS48760.1	23.6537362	23.9315357	24.0111906	24.26659903	24.76405127	23.5439474	0.81032716	0.977	0.326	PIS48760.1	MDM12	Ortholog(s) have lipid transfer activity

PIS56678.1	26.7859897	26.4274281	27.1477725	27.55793848	26.45700938	27.32309449	0.382047068	0.968	0.326	PIS56678.1	orf19.4107	Exopolyphosphatase, hydrolyzes inorganic polyphosphate (poly P) into Pi residues; redundant with polyphosphatase Phm5p; Spider biofilm repressed
PIS58324.1	28.6165596	27.8510488	27.703192	28.11532931	28.30896454	28.73404815	0.52794044	0.973	0.329	PIS58324.1	CAF16	ABC family protein, predicted not to be a transporter; Hap43, caspofungin repressed; rat catheter and Spider biofilm repressed
PIS48371.1	23.1354741	23.63113	25.2236105	24.61966367	23.43986543	24.91671651	0.595117833	0.974	0.329	PIS48371.1	DAO2	Putative D-amino acid oxidase; rat catheter biofilm induced
PIS49739.1	28.8366259	28.4169292	27.8673762	29.02260716	28.42662067	28.66350014	0.329956902	0.966	0.331	PIS49739.1	orf19.6778	Aminophospholipid translocase (flippase); maintains membrane lipid asymmetry in post-Golgi secretory vesicles; contributes to clathrin-coated vesicle formation and endocytosis; flow model biofilm repressed
PIS54873.1	24.0755891	25.0281509	24.1616328	24.55559313	24.94881671	24.75586913	0.452830669	0.971	0.332	PIS54873.1	DUT1	dUTP pyrophosphatase; cell-cycle regulated if expressed in S. cerevisiae; upstream MluI and SCB elements; 17-beta-estradiol, ethynyl estradiol, macrophage induced; decreased in stationary phase yeast; rat catheter, Spider biofilm repressed
PIS59018.1	32.6240764	32.6383219	32.6928156	32.54961539	32.65626987	33.74455891	0.606916512	0.974	0.332	PIS59018.1	HSP70	Putative hsp70 chaperone; role in entry into host cells; heat-shock, amphotericin B, cadmium, ketoconazole-induced; surface localized in yeast and hyphae; antigenic in host; farnesol-downregulated in biofilm; Spider
PIS55644.1	26.7761593	25.8656446	26.0782442	27.01666247	26.98518936	25.71413778	0.467345376	0.971	0.332	PIS55644.1	TCC1	Putative transcription factor/corepressor; regulation of filamentation and virulence; interacts with Tup1; regulates hypha-specific gene expression; contains 4 tetratricopeptide repeat (TPR) motifs; flucytosine repressed; Tbp1-induced

PIS51917.1	26.0306112	25.5270693	25.8835232	25.78922929	25.79388847	26.85732654	0.657913659	0.975	0.333	PIS51917.1	orf19.4182	Has domain(s) with predicted oxidoreductase activity
PIS48656.1	24.8969574	23.8107445	25.4483729	25.535895	25.40424513	24.21443733	0.483514331	0.971	0.333	PIS48656.1	orf19.496	Ortholog(s) have ATP-dependent activity, acting on DNA, dinucleotide insertion or deletion binding, guanine/thymine mispair binding activity
PIS58677.1	28.7045771	30.5141091	28.9526017	28.66744059	30.06298063	30.43969382	0.539206814	0.973	0.333	PIS58677.1	orf19.6411	Protein similar to S. cerevisiae Vac14p; a protein involved in regulated synthesis of PtdIns(3,5)P(2); transposon mutation affects filamentous growth
PIS52047.1	29.1866276	28.9668805	28.0347857	28.91067192	29.14132785	29.1357237	0.343662142	0.967	0.333	PIS52047.1	SNF4	Putative subunit of the AMP-activated Snf1p kinase; ortholog of S. cerevisiae Snf4; caspofungin repressed; transposon mutation affects filamentation
PIS51110.1	29.7988077	28.8577603	29.0106872	29.51880768	29.14001869	30.01060745	0.714203465	0.976	0.334	PIS51110.1	MKC1	MAP kinase; role in biofilm formation, contact-induced invasive filamentation, systemic virulence in mouse, cell wall structure/maintenance, caspofungin response; phosphorylated on surface contact, membrane perturbation, or cell wall stress
PIS51096.1	25.0603315	25.4715563	24.7039681	26.2591862	24.35870936	25.63175751	0.629226513	0.975	0.338	PIS51096.1	ALK2	N-Alkane inducible cytochrome P450
PIS55469.1	28.7022521	28.9702036	29.6051957	29.40287698	29.42911773	29.46022032	0.415244812	0.97	0.338	PIS55469.1	orf19.5279	Ortholog(s) have structural constituent of ribosome activity, role in cell redox homeostasis, mitochondrial genome maintenance and mitochondrial large ribosomal subunit, mitochondrion localization

PIS51713.1	29.4636153	29.6625126	29.10174	29.93387374	29.69147154	29.61999361	0.283068776	0.964	0.339	PIS51713.1	GEA2	Putative ARF GTP/GDP exchange factor; induced in low iron; flow model biofilm repressed
PIS58105.1	25.1712873	24.7856039	23.6199716	24.89579118	24.91896798	24.77946195	0.531967499	0.973	0.339	PIS58105.1	orf19.177	Has domain(s) with predicted phosphatidylinositol binding activity
PIS58672.1	27.4883554	27.4898994	27.4127015	27.9563491	27.44446056	28.00808631	0.373044952	0.968	0.339	PIS58672.1	orf19.6418	Ortholog(s) have unfolded protein binding activity and role in protein import into nucleus, ribosomal large subunit biogenesis
PIS48553.1	29.7791706	29.8345643	29.7412537	30.04058417	30.25607333	30.07815608	0.255255682	0.963	0.34	PIS48553.1	HIS1	ATP phosphoribosyl transferase; enzyme of histidine biosynthesis; acid upregulated/alkaline repressed by Rim101; regulated by Gcn2, Gcn4; strain CA9 is a his1 mutant; flow model biofilm induced; Spider biofilm repressed
PIS56567.1	19.964444	24.7504177	24.4289689	24.24858558	22.07374285	23.84161853	0.790750007	0.977	0.34	PIS56567.1	orf19.2612	C2H2 zinc finger transcription factor; expression reduced in ssr1 null mutant; flow model biofilm induced
PIS48646.1	27.3372228	25.8920542	25.4252827	26.89210704	26.56826338	26.21712211	0.471175374	0.971	0.341	PIS48646.1	CDC27	Putative ubiquitin-protein ligase; periodic mRNA expression, peak at cell-cycle S/G2 phase
PIS51406.1	31.9526891	30.6226819	31.3891393	32.63086239	31.65222995	30.70588997	0.680901867	0.975	0.341	PIS51406.1	orf19.4395	Putative actin cytoskeleton component; protein present in exponential and stationary growth phase yeast cultures

PIS48742.1	27.6630328	27.7049993	27.6107961	27.93214331	28.17019671	27.89881012	0.430363351	0.97	0.341	PIS48742.1	SAM35	Predicted component of the sorting and assembly machinery (SAM complex) of the mitochondrial outer membrane, involved in protein import into mitochondria
PIS58917.1	25.6979175	26.5203451	26.6649177	27.26412388	26.20080542	26.44283581	0.499481012	0.972	0.342	PIS58917.1	SMI1B	Putative cell wall assembly regulatory protein; Rim101-repressed; possibly an essential gene, disruptants not obtained by UAU1 method
PIS51757.1	31.8101	31.2949141	30.8548309	32.29708635	31.41940966	31.27059305	0.402111528	0.969	0.342	PIS51757.1	VPS13	Putative vacuolar protein sorting-associated protein; gene used for multilocus sequence typing
PIS48415.1	22.943802	24.4777598	25.2053928	24.1229504	24.40640762	25.12779492	0.604578472	0.974	0.343	PIS48415.1	CWT1	Zn2Cys6 transcription factor involved in negative regulation of nitrosative stress response; regulates formation of mating projections and same-sex mating; mutant has cell wall defects; transcription increased at stationary phase
PIS58524.1	29.3452115	27.2386811	27.4214327	29.27137762	27.7473977	28.02159671	0.610530051	0.974	0.345	PIS58524.1	PWP2	Putative 90S pre-ribosomal component; repressed in core stress response; repressed by prostaglandins; physically interacts with TAP-tagged Nop1; Hap43-induced
PIS48795.1	31.9607444	31.7072026	32.233495	31.98853635	32.32284833	32.63063465	0.359167843	0.968	0.347	PIS48795.1	YHM1	Putative mitochondrial carrier protein; fungal-specific (no human or murine homolog); Hap43p-repressed gene
PIS48404.1	27.7957858	26.4847806	26.9621705	27.36797679	27.48425351	27.43589293	0.621985305	0.974	0.348	PIS48404.1	orf19.6550	Mitochondrial outer membrane protein, component of vacuole and mitochondria patches (vCLAMPs); involved in mitophagy; mutants are defective in mitochondrial function and virulence; regulated by Sef1p, Sfu1p, and Hap43p

PIS58802.1	24.9144781	24.2878686	24.4557722	25.53511232	24.27755649	24.89234573	0.620858425	0.974	0.349	PIS58802.1	ADA2	Zinc finger and homeodomain transcriptional coactivator; role in cell wall integrity and in sensitivity to caspofungin; required for the normal transcriptional response to caspofungin; required for yeast cell adherence to silicone substrate
PIS48472.1	29.3065883	29.7758124	30.1903714	29.43431666	30.3727307	30.511808	0.398214195	0.969	0.349	PIS48472.1	SEC61	ER protein-translocation complex subunit; essential; 10 predicted transmembrane regions; chimeric mutant partially functionally complements S. cerevisiae sec61 defects; Spider biofilm repressed
PIS51446.1	28.3584564	28.2716738	28.7697917	28.85506692	28.51471832	29.07901632	0.396212383	0.969	0.35	PIS51446.1	ABG1	Vacuolar membrane protein; depletion causes abnormal vacuolar morphology, cell separation defect, sensitivity to cell wall stress, increased hyphal branching; essential, no mammalian homolog; Cyr1-regulated; rat catheter biofilm repressed
PIS51508.1	23.3018374	24.6832451	24.2306591	25.76686239	23.48696189	24.01161576	0.675385833	0.975	0.35	PIS51508.1	orf19.5987	Ortholog(s) have tRNA (guanine(10)-N2)-methyltransferase activity, role in mRNA methylation, tRNA methylation and cytoplasm, tRNA (m2G10) methyltransferase complex localization
PIS58176.1	31.5629503	30.4994413	30.117763	31.44283633	31.25611965	30.53488397	0.371106057	0.968	0.351	PIS58176.1	BUD7	Ortholog(s) have small GTPase binding activity and role in Golgi to plasma membrane protein transport, Golgi to plasma membrane transport, Golgi to vacuole transport, fungal-type cell wall chitin biosynthetic process
PIS54846.1	29.8216591	28.9223686	29.3174716	29.0077838	29.93555054	30.1706406	0.451155153	0.971	0.351	PIS54846.1	COX11	Cytochrome oxidase assembly protein; transcript regulated by Nrg1; protein repressed during the mating process; Hap43-repressed gene; rat catheter biofilm induced
PIS52182.1	28.6541878	28.4264055	28.8805866	29.5337322	29.11856107	28.36052983	0.390515253	0.969	0.351	PIS52182.1	orf19.1335	Ortholog of S. cerevisiae Mtr4, an ATP-dependent 3'-5' RNA helicase of the DEAD-box family; Hap43-induced gene; Spider biofilm induced

PIS54820.1	28.7925195	28.7241871	27.8969128	28.13826007	29.10368266	29.22378212	0.336243943	0.967	0.351	PIS54820.1	orf19.1804	Ortholog(s) have glycine transmembrane transporter activity, role in glycine import into mitochondrion, heme biosynthetic process and mitochondrion localization
PIS48616.1	26.6326952	27.2866281	27.2083148	26.47671185	27.79837018	27.91186749	0.486094623	0.972	0.353	PIS48616.1	orf19.1946	Similar to an aldose 1-epimerase-related protein; antigenic during murine systemic infection; protein present in exponential and stationary phase yeast cultures; Hap43-induced; Spider biofilm repressed
PIS51205.1	28.7357705	29.1166864	28.1678891	28.00096369	29.36522153	29.7131376	0.394990878	0.969	0.353	PIS51205.1	orf19.5684	Ortholog(s) have structural constituent of ribosome activity and mitochondrial large ribosomal subunit localization
PIS58508.1	27.0577824	26.7598248	26.8501513	26.88140684	27.13238081	27.71465881	0.308467621	0.966	0.354	PIS58508.1	orf19.3728	Ortholog(s) have protein phosphatase 1 binding, protein phosphatase regulator activity and role in chromosome segregation
PIS50599.1	23.7070384	25.5796123	25.079948	24.83443117	24.76468348	25.82999615	0.791695859	0.977	0.354	PIS50599.1	orf19.4159	Ortholog(s) have magnesium ion transmembrane transporter activity, role in magnesium ion export from mitochondrion, magnesium ion transport and mitochondrial inner membrane localization
PIS51924.1	30.9111169	30.7242866	30.8080733	31.65527291	31.03762668	30.81622699	0.35400301	0.967	0.355	PIS51924.1	HIS5	Putative histidinol-phosphate aminotransferase; Gcn4p-regulated; protein present in exponential and stationary growth phase yeast cultures
PIS49708.1	21.1778332	21.2282901	23.4146216	21.70364774	22.79715249	22.38458155	0.700347301	0.976	0.355	PIS49708.1	orf19.6532	Ortholog(s) have FAD transmembrane transporter activity, role in FAD transport and mitochondrion localization

PIS55473.1	29.7828539	29.5338828	29.9574497	29.74366328	30.03903611	30.55853555	0.242067723	0.962	0.356	PIS55473.1	CTA3	Protein similar to S. cerevisiae Ede1p, which is involved in endocytosis; activates transcription in 1-hybrid assay in S. cerevisiae
PIS56692.1	26.8915626	25.3241969	26.5872545	27.13530743	25.92218957	26.81764064	0.503610738	0.972	0.357	PIS56692.1	orf19.6809	Putative phosphomutase-like protein; protein present in exponential and stationary growth phase yeast; Hap43-repressed; Spider biofilm repressed
PIS52096.1	27.4194306	27.100483	27.1785852	27.55613697	27.60238769	27.61713763	0.240409835	0.962	0.359	PIS52096.1	PRD1	Putative proteinase; transcript regulated by Nrg1, Mig1, and Tup1; Hogp-induced; stationary phase enriched protein; Hap43-repressed; rat catheter biofilm repressed
PIS48428.1	28.5125758	28.7738594	30.186505	29.9912896	28.85676323	29.70455118	0.41220086	0.969	0.36	PIS48428.1	HMO1	HMG-box transcription factor; binds upstream of hexose and ergosterol metabolism and cell cycle genes; acts as repressor of START; activates pseudohyphal growth when expressed in S. cerevisiae; repressed in hyphae
PIS55723.1	27.7128095	26.7484421	26.5526103	27.50582939	27.23177698	27.35900381	0.544891795	0.973	0.361	PIS55723.1	orf19.2446	Has domain(s) with predicted 2-dehydropantoate 2-reductase activity, oxidoreductase activity and role in pantothenate biosynthetic process
PIS51388.1	29.7518248	29.3994493	29.5095493	29.7927128	29.98649826	29.96513619	0.174048017	0.956	0.361	PIS51388.1	YPT52	Rab-family GTPase involved in vacuolar trafficking, colocolizes with Vps1p and Ypt53p in late endosome
PIS50516.1	28.9522454	29.3815668	28.8414762	29.6119304	29.0045524	29.64497095	0.273339125	0.964	0.362	PIS50516.1	orf19.2680	Putative aminophospholipid translocase (flippase); type 4 P-type ATPase; likely involved in phospholipid translocation

PIS54858.1	29.2896402	29.6989081	29.2443828	29.93957821	29.81716485	29.56331326	0.595146734	0.974	0.362	PIS54858.1	orf19.6951	Ortholog(s) have sphinganine-1-phosphate aldolase activity and role in calcium-mediated signaling, cellular response to starvation, sphingolipid metabolic process
PIS54821.1	23.8497132	24.0513497	23.3031709	24.95176304	21.67771991	25.66268078	0.703565879	0.976	0.363	PIS54821.1	CDC43	Beta subunit of heterodimeric protein geranylgeranyltransferase type I; GGTase I enzyme binds zinc, is Mg-dependent; Cdc42p is GGTase I substrate
PIS58585.1	29.5522052	28.8211218	28.8841606	30.06096313	29.52773564	28.75824916	0.46237889	0.971	0.363	PIS58585.1	NAP1	Nucleosome assembly protein; mutants show constitutive filamentous growth; present in exponential and stationary growth phase yeast cultures
PIS54829.1	26.1841239	27.213998	28.1460924	26.29813106	27.70455757	28.63332336	0.640892898	0.975	0.364	PIS54829.1	orf19.6318	Has domain(s) with predicted membrane localization
PIS55665.1	29.6013244	29.3838208	28.490458	29.53676957	29.31858756	29.7200587	0.298840171	0.965	0.367	PIS55665.1	orf19.1578	Ortholog of S. cereviaie Rrp5, an RNA binding protein involved in synthesis of 18S and 5.8S rRNAs; Hap43-induced gene
PIS58639.1	25.769367	26.251877	24.6700533	26.51612961	26.16113502	25.11632488	0.581175596	0.974	0.367	PIS58639.1	orf19.5491	Ortholog of C. dubliniensis CD36 : Cd36_20670, C. parapsilosis CDC317 : CPAR2_104720, C. auris B8441 : B9J08_000085 and Candida tenuis NRRL Y-1498 : CANTEDRAFT_103989
PIS54533.1	28.1919011	28.5901509	28.7301871	29.12782806	28.30071912	29.19051599	0.358763899	0.968	0.369	PIS54533.1	orf19.4796	Putative eIF-4E-binding repressor of CAP-dependent translation; stationary phase enriched protein

PIS48385.1	31.6509459	31.7772334	31.2630774	32.29183091	32.05884923	31.44804108	0.333272033	0.967	0.369	PIS48385.1	UCF1	Upregulated by cAMP in filamentous growth; induced in high iron, decreased upon yeast-hypha switch; downregulation correlates with clinical fluconazole resistance; Ras1-regulated; Hap43-repressed; flow model biofilm induced
PIS51143.1	24.0743539	23.947109	23.6956792	24.75753425	24.44385356	23.62714545	0.456265475	0.971	0.37	PIS51143.1	FGR10	Putative asparaginase; lacks ortholog in S. cerevisiae; transposon mutation affects filamentous growth; Spider biofilm induced
PIS58882.1	31.9984575	31.9047555	31.8846158	31.96728544	32.31840564	32.6194196	0.225470278	0.961	0.372	PIS58882.1	COX5	Cytochrome oxidase subunit V; putative upstream CCAAT box regulatory element; macrophage/pseudohyphal-induced; repressed by nitric oxide; intron in 5'-UTR; Hap43p-dependent repression in low iron medium
PIS49603.1	23.034827	23.9883091	25.30192	24.13376269	23.61204157	25.69525181	0.634396246	0.975	0.372	PIS49603.1	MPT5	Putative RNA-binding protein; Hap43p-repressed gene; protein induced during the mating process
PIS52438.1	26.2876743	25.6911978	25.4398124	25.53107556	26.53964672	26.46531494	0.624532732	0.974	0.372	PIS52438.1	orf19.5276	Putative nuclear pore-associated protein; Hap43p-induced gene; induced upon low-level peroxide stress; possibly an essential gene, disruptants not obtained by UAU1 method
PIS55583.1	28.9975122	28.8992324	28.1559547	28.86017789	29.04744954	29.26392665	0.274019348	0.964	0.373	PIS55583.1	ISN1	Putative inosine 5'-monophosphate 5'-nucleotidase; fungal-specific (no human or murine homolog)
PIS51181.1	28.8969972	29.6843702	29.9658839	29.92879422	29.66944744	30.06942709	0.443124975	0.97	0.373	PIS51181.1	MSS51	Putative mRNA maturation factor; fungal-specific (no human or murine homolog)

PIS55497.1	23.7530886	26.5089924	23.5222832	24.82689131	25.03889862	25.03630362	0.763239682	0.977	0.373	PIS55497.1	orf19.5049	Putative U3-containing 90S preribosome processome complex subunit; Hap43-induced gene; rat catheter and Spider biofilm induced; F-12/CO2 early biofilm induced
PIS54732.1	28.4243584	28.4586596	27.3468438	28.12246721	28.68104814	28.55083448	0.357978636	0.968	0.375	PIS54732.1	orf19.5012	Ortholog of C. dubliniensis CD36 : Cd36_12740, C. parapsilosis CDC317 : CPAR2_201620, C. auris B8441 : B9J08_002511 and Candida tenuis NRRL Y-1498 : CANTEDRAFT_107586
PIS48516.1	27.1452183	26.943607	27.1844301	27.13530608	27.74171402	27.52349747	0.201865182	0.959	0.376	PIS48516.1	ARG8	Putative acetylornithine aminotransferase; Gcn2, Gcn4 regulated; rat catheter biofilm induced; Spider biofilm induced
PIS48822.1	30.0193109	29.8412036	30.4599903	30.34577315	30.59990851	30.51135981	0.221556183	0.96	0.379	PIS48822.1	GUA1	Putative GMP synthase, involved in the final step of guanine biosynthesis; soluble protein in hyphae; flucytosine induced; macrophage-downregulated protein abundance; protein level decreases in stationary phase cultures
PIS50480.1	26.4663051	26.6877795	27.345203	27.87844283	26.33448142	27.4221502	0.426078355	0.97	0.379	PIS50480.1	orf19.3984	Protein of unknown function; induced in core caspofungin response; induced in ssr1 mutant; induced by nitric oxide independent of Yhb1; Spider biofilm induced
PIS51803.1	27.6729667	28.6050284	27.0906417	28.24319614	28.28911498	27.97341991	0.747814953	0.977	0.379	PIS51803.1	ZPR1	Protein with putative zinc finger; regulated by Gcn4p; repressed in response to amino acid starvation (3-aminotriazole treatment); upregulation correlates with clinical development of fluconazole resistance
PIS58522.1	20.1325978	22.0415271	24.3627645	21.5867696	23.13951671	22.95266624	0.691925669	0.976	0.381	PIS58522.1	orf19.3273	Ortholog(s) have actin monomer binding, polysome binding, protein kinase inhibitor activity, ribosome binding activity and role in negative regulation of protein phosphorylation

PIS48329.1	27.1900923	26.7611718	27.8937965	27.73502385	27.34562745	27.90824515	0.59274461	0.974	0.381	PIS48329.1	orf19.3548	Ortholog(s) have mitochondrial inner membrane localization
PIS58329.1	31.6651391	30.9250947	31.1283374	32.23905707	31.55321714	31.06904996	0.414695058	0.97	0.381	PIS58329.1	RPL42	Putative 60S ribosomal subunit protein; colony morphology-related gene regulation by Ssn6; Spider biofilm repressed
PIS49835.1	23.8279448	24.3809582	24.2182284	24.71847945	24.47310235	24.38134472	0.452277961	0.971	0.382	PIS49835.1	NBN1	Subunit of the NuA4 histone acetyltransferase complex; Plc1p-regulated
PIS58788.1	23.6738983	18.7161973	23.7666584	24.59626689	18.10104909	24.60536222	0.857637423	0.978	0.382	PIS58788.1	orf19.2325	Ortholog(s) have RNA polymerase III general transcription initiation factor activity and RNA polymerase III type 1 promoter sequence-specific DNA binding, more
PIS52453.1	25.974696	26.5591173	25.5088668	26.57515903	26.13461205	26.47911834	0.318268943	0.966	0.382	PIS52453.1	orf19.4340.1	Ortholog(s) have poly(U) RNA binding, splicing factor binding activity and U1 snRNP, U2 snRNP, U4/U6 x U5 tri-snRNP complex, U5 snRNP, post-mRNA release spliceosomal complex, spliceosomal complex localization
PIS55594.1	30.4097022	29.8871324	30.0674469	30.19003819	30.64602473	30.67838369	0.332546014	0.967	0.383	PIS55594.1	orf19.6637	Predicted glycosyl hydrolase; hypoxia induced; flow model biofilm induced
PIS56634.1	31.4153229	31.046259	31.2756907	31.20789405	31.51347075	32.16953659	0.337008702	0.967	0.385	PIS56634.1	orf19.2582	Ortholog(s) have alpha-aminoacyl-tRNA binding, ribosomal large subunit binding, tRNA binding activity

PIS55551.1	30.5603778	30.6138264	29.7382717	30.67403976	30.51729095	30.87492151	0.235585462	0.961	0.385	PIS55551.1	SEC23	Putative GTPase-activating protein; regulated upon yeast-hypha switch; Spider biofilm repressed
PIS58152.1	27.3732343	27.9669901	27.8033277	28.22386405	27.79891904	28.27789575	0.201299861	0.959	0.386	PIS58152.1	orf19.1340	Putative aldose reductase; protein level decreases in stationary phase cultures; Spider biofilm repressed
PIS51489.1	26.7572067	27.7432499	27.2127406	28.56223498	27.11444493	27.19480212	0.439884777	0.97	0.386	PIS51489.1	orf19.5833	Protein involved in resistance to caspofungin and anidulafungin
PIS54855.1	26.9664269	26.3386176	26.6729289	27.67149116	26.97253259	26.49429503	0.32071275	0.966	0.387	PIS54855.1	HBR3	Essential protein; regulated by hemoglobin; S. cerevisiae ortholog is essential; Hap43p-induced gene
PIS58207.1	27.8755266	28.0813227	27.8951601	28.67250978	28.47076527	27.87008705	0.362447667	0.968	0.387	PIS58207.1	orf19.7485	Putative mitochondrial ribosomal protein of the large subunit; Hap43p-repressed gene; ortholog of S. cerevisiae MRPL9
PIS48520.1	24.82414	28.086622	28.7225643	25.75699285	28.37123892	28.66871521	0.70227082	0.976	0.388	PIS48520.1	orf19.3778	Protein with a predicted role in ribosome biogenesis; mutation confers hypersensitivity to 5-fluorocytosine (5-FC), 5-fluorouracil (5-FU); repressed in core stress response; repressed by prostaglandins; Hap43-induced
PIS50553.1	28.8265372	29.0750104	28.2218054	29.82072205	27.84192915	29.62338976	0.431507337	0.97	0.388	PIS50553.1	UAP1	UDP-N-acetylglucosamine pyrophosphorylase, catalyzes biosynthesis of UDP-N-acetylglucosamine from UTP and N-acetylglucosamine 1-phosphate; functional homolog of S. cerevisiae Qri1p; alkaline upregulated

PIS49563.1	31.2107102	30.7850495	30.5295853	31.72119683	31.26156581	30.70995671	0.242433143	0.962	0.389	PIS49563.1	ERG9	Putative farnesyl-diphosphate farnesyl transferase (squalene synthase); sterol biosynthesis pathway; likely essential for growth; regulated by fluconazole, lovastatin; amphotericin B, caspofungin repressed; Spider biofilm repressed
PIS51172.1	27.4074501	27.4227644	28.3729801	28.04408185	28.37469144	27.95282159	0.253470751	0.963	0.389	PIS51172.1	SIK1	Putative U3 snoRNP protein; Hap43p-induced gene; physically interacts with TAP-tagged Nop1p
PIS52381.1	28.5199105	27.2392937	25.9224333	28.12231562	27.73924186	26.99483582	0.561855675	0.973	0.392	PIS52381.1	orf19.326	Protein of unknown function; Spider biofilm repressed
PIS52232.1	22.3020521	20.2275955	23.0298839	23.64098795	20.9102461	22.18393033	0.789497871	0.977	0.392	PIS52232.1	orf19.6377	Ortholog(s) have protein C-terminal leucine carboxyl O-methyltransferase activity and role in C-terminal protein methylation, protein-containing complex assembly, regulation of autophagy
PIS55815.1	29.2720805	28.5745056	28.2085707	29.21754191	28.98419478	29.03416913	0.255911673	0.963	0.394	PIS55815.1	orf19.7378	Putative phosphopantothenoylcysteine decarboxylase, binds to protein phosphatase Ppz1p and regulates its activity
PIS56714.1	23.0686669	22.9281212	24.3774979	23.77861629	23.80278411	23.97829483	0.693179089	0.976	0.395	PIS56714.1	orf19.7670	Putative Ca2+/H+ antiporter; oral infection upregulated gene; mutants have reduced capacity to damage oral epithelial cells
PIS48292.1	29.4847558	29.0071176	29.4412052	29.71144079	29.70077839	29.70714137	0.1490625	0.953	0.395	PIS48292.1	YCK2	Plasma membrane protein similar to S. cerevisiae casein kinase I; mutation or inhibition impairs virulence and morphogenesis; transcription is activated in weak acid stress or on contact with with host cells

PIS58981.1	30.3091955	29.3573619	28.9306217	29.7858549	30.10910538	29.89414096	0.269971554	0.964	0.397	PIS58981.1	NAM7	Putative role in nonsense-mediated mRNA decay; similar to S. cerevisiae Nam7p; gene induced by ciclopirox olamine treatment
PIS54834.1	25.4682258	24.795942	25.4698187	25.38987901	25.44464705	26.0902602	0.40155866	0.969	0.397	PIS54834.1	orf19.6315	Ortholog of C. dubliniensis CD36 : Cd36_30140, C. parapsilosis CDC317 : CPAR2_204040, C. auris B8441 : B9J08_001978 and Candida tenuis NRRL Y-1498 : CANTEDRAFT_114703
PIS54659.1	24.6030722	24.1332016	23.5487618	25.2926214	24.27992886	23.90765556	0.49636207	0.972	0.398	PIS54659.1	orf19.6604	Ortholog of S. cerevisiae Pba1 that is involved in 20S proteasome assembly; upregulated in a cyr1 null mutant; contains a 5' UTR intron
PIS58965.1	29.6593437	29.5930916	29.4639675	29.97432459	29.87171604	30.06686575	0.303543903	0.965	0.399	PIS58965.1	orf19.1956	Ortholog(s) have role in mitochondrial translational initiation, reciprocal meiotic recombination and mitochondrial matrix, mitochondrion localization
PIS56884.1	24.328382	24.2767728	25.0494596	25.3784927	24.73686744	24.73832709	0.394995993	0.969	0.4	PIS56884.1	SPB1	Putative AdoMet-dependent methyltransferase; Hap43-induced; repressed by prostaglandins; possibly essential gene, disruptants not obtained by UAU1 method; Spider biofilm induced
PIS51282.1	24.6868108	24.2429339	24.7923873	24.99982144	24.97362546	24.95242391	0.296162724	0.965	0.401	PIS51282.1	VPS8	Putative vacuolar sorting protein; downregulated upon adherence to polystyrene
PIS49745.1	26.9372899	28.0776258	27.0391958	27.86106386	27.3855148	28.01704932	0.482651036	0.971	0.403	PIS49745.1	orf19.4905	Putative MFS transporter; Hap43p-induced gene; also regulated by regulated by Sef1p and Sfu1p; repressed in a ssr1 null mutant

PIS51497.1	25.2758549	23.9846893	24.9362927	25.64183433	25.20523601	24.55897187	0.364397627	0.968	0.403	PIS51497.1	PDR17	Fungal-specific protein (no human or murine homolog); role in sensitivity to fluconazole, specifically
PIS51496.1	27.5743892	26.9976103	25.0964121	27.85603089	27.46769698	25.553926	0.605849456	0.974	0.403	PIS51496.1	SER2	Ortholog(s) have L-phosphoserine phosphatase activity and role in L-serine biosynthetic process
PIS58353.1	31.2099324	30.4299599	29.7405163	31.25648806	30.77248493	30.56689057	0.4762127	0.971	0.405	PIS58353.1	ASR2	Adenylyl cyclase and stress responsive protein; induced in cyr1 or ras1 mutant; stationary phase enriched protein; Spider biofilm induced
PIS51963.1	25.4443353	27.7761426	27.0469271	26.75189469	27.27912792	27.45259161	0.530486469	0.973	0.405	PIS51963.1	orf19.6740	Ortholog(s) have ubiquitin-protein transferase activity and role in generation of catalytic spliceosome for first transesterification step
PIS55056.1	29.3079312	27.9942442	27.6659597	30.01189835	28.67538604	27.5007065	0.573468947	0.974	0.407	PIS55056.1	CLC1	Clathrin light chain; subunit of the major coat protein involved in intracellular protein transport and endocytosis; rat cathetr and Spider biofilm repressed
PIS52301.1	28.5871868	28.9658353	28.6088021	29.14032905	28.9592382	29.28259769	0.12791112	0.95	0.407	PIS52301.1	orf19.5229	Ortholog(s) have 3'-5'-RNA exonuclease activity, RNA endonuclease activity, RNA exonuclease activity, RNA nuclease activity, small GTPase binding, tRNA binding activity
PIS51590.1	23.7240656	24.4389481	23.4016027	24.92865527	24.12640014	23.73403844	0.589176725	0.974	0.408	PIS51590.1	VPS33	Ortholog(s) have ATP binding, phosphatidylinositol binding activity

PIS50608.1	27.9200624	27.6142591	27.4277388	30.62816416	27.15193173	26.4083195	0.632209885	0.975	0.409	PIS50608.1	HGT5	Putative glucose transporter of the major facilitator superfamily; the C. albicans glucose transporter family comprises 20 members; 12 probable membrane-spanning segments, extended N terminus; expressed in rich medium; Hap43p-repressed
PIS51788.1	24.5132339	24.7227255	24.7936491	23.95573216	25.00625156	26.29443625	0.661908641	0.975	0.409	PIS51788.1	orf19.676	Microtubule-binding protein of the cortical microtubule; delays exit from mitosis when the spindle is abnormally oriented; Spider biofilm repressed
PIS58352.1	29.2790819	29.0634044	29.2445303	29.60350088	29.44678456	29.76237056	0.180080488	0.957	0.409	PIS58352.1	PEX13	Protein required for peroxisomal protein import mediated by PTS1 and PTS2 targeting sequences; transcript induced in an RHE model of oral candidiasis; Hap43-repressed gene
PIS51516.1	28.4834351	28.0590106	27.7596531	28.75872507	28.46924829	28.30439152	0.292468867	0.965	0.41	PIS51516.1	orf19.2180	Ortholog(s) have calcium ion binding, zinc ion binding activity and role in axial cellular bud site selection, bipolar cellular bud site selection, regulation of COPII vesicle coating
PIS49551.1	24.1710956	24.922733	26.2373762	25.40891633	25.61448249	25.53667312	0.771302351	0.977	0.41	PIS49551.1	orf19.2478	Ortholog of C. dubliniensis CD36 : Cd36_05410, C. parapsilosis CDC317 : CPAR2_107480, C. auris B8441 : B9J08_004575 and Candida tenuis NRRL Y-1498 : CANTEDRAFT_94695
PIS58533.1	31.6842022	31.6425167	31.9433699	32.45415	32.01539381	32.03052456	0.123397162	0.95	0.41	PIS58533.1	TOM70	Ortholog(s) have mitochondrion targeting sequence binding, protein transmembrane transporter activity
PIS48784.1	29.5155965	28.6958311	26.8087112	27.39249717	29.29943815	29.56249251	0.574914676	0.974	0.411	PIS48784.1	orf19.511	Ortholog(s) have ribosylnicotinamide kinase activity and role in NAD biosynthesis via nicotinamide riboside salvage pathway, NAD biosynthetic process, nicotinamide riboside metabolic process

PIS52408.1	24.5932499	25.4530939	25.1670716	25.27797235	25.538656	25.63324293	0.493748778	0.972	0.412	PIS52408.1	NTF2	Putative nuclear envelope protein; regulated by Ssn6; rat catheter biofilm repressed
PIS50484.1	30.4721171	30.7698627	31.546193	31.06741294	31.2607255	31.69806014	0.215172001	0.96	0.413	PIS50484.1	PFK1	Phosphofructokinase alpha subinit; activated by fructose 2,6-bisphosphate, AMP, ATP inhibited; activity reduced on hyphal induction; phagocytosis-repressed; fluconazole, flow model biofilm induced; rat catheter and Spider biofilm repressed
PIS52001.1	28.9148187	29.8060623	29.5634168	29.14976957	30.14252618	30.23486556	0.464084032	0.971	0.414	PIS52001.1	OLE1	Fatty acid desaturase, essential protein involved in oleic acid synthesis; required for aerobic hyphal growth and chlamydospore formation; subject to hypoxic regulation; fluconazole-induced; caspofungin repressed; Hap43p-induced
PIS56719.1	27.7206204	26.6950607	25.4836893	28.18812031	27.13878609	25.81477276	0.544944994	0.973	0.414	PIS56719.1	VPS52	Subunit of GARP (Golgi-associated retrograde protein) complex, which has roles in Golgi to vacuole transport, ascospore wall assembly, cellular sphingolipid homeostasis; required for filamentous growth
PIS58160.1	27.478182	28.2199329	28.3763733	28.27350451	28.24550563	28.79926207	0.286142138	0.964	0.415	PIS58160.1	orf19.3357	Ortholog(s) have structural constituent of ribosome activity and mitochondrial small ribosomal subunit, mitochondrion localization
PIS55033.1	26.985406	25.500207	25.3036201	26.01690429	26.44022798	26.58305674	0.503619114	0.972	0.417	PIS55033.1	CHT4	Chitinase; similar to S. cerevisiae sporulation-specific Cts2p; functionally complements A. gossypii cts2 mutant sporulation defect; homozygous null mutation causes no obvious defects; transcription decreases upon yeast-to-hyphal switch
PIS48700.1	28.3543527	28.0792942	28.3974295	29.20541851	28.38581766	28.49095641	0.305779924	0.965	0.417	PIS48700.1	EXO84	Predicted subunit of the exocyst complex, involved in exocytosis; localizes to a crescent on the surface of the hyphal tip

PIS49816.1	26.7289365	27.2876995	27.993891	28.03228033	27.79629649	27.43646082	0.38272567	0.968	0.418	PIS49816.1	orf19.4283	Ortholog(s) have role in cytoplasmic translational initiation
PIS55689.1	26.4651692	26.0580226	26.6822772	27.31208716	25.88549329	27.26953988	0.445135666	0.97	0.421	PIS55689.1	ALG6	Putative glucosyltransferase involved in cell wall mannan biosynthesis; transcription is elevated in chk1, nik1, and sln1 homozygous null mutants; repressed by nitric oxide; possibly essential gene, disruptants not obtained by UAU1 method
PIS48237.1	23.6511264	23.9660898	25.2272475	25.02918866	24.3601903	24.7291565	0.375355937	0.968	0.425	PIS48237.1	orf19.7163	Ortholog of C. dubliniensis CD36 : Cd36_73790, C. parapsilosis CDC317 : CPAR2_702880, C. auris B8441 : B9J08_004922 and Candida tenuis NRRL Y-1498 : CANTEDRAFT_92070
PIS52100.1	29.2778704	29.1185587	28.5723687	29.84251327	29.56547005	28.85073693	0.215101397	0.96	0.43	PIS52100.1	orf19.439	Ortholog(s) have DNA binding, DNA strand exchange activity, single-stranded DNA binding, structural constituent of ribosome activity
PIS58599.1	26.6962512	28.4327989	30.2100092	28.89898985	27.96363658	29.7701826	0.732743869	0.976	0.431	PIS58599.1	ISW2	Ortholog of S. cerevisiae Isw2; an ATPase involved in chromatin remodeling; required for chlamydospore formation; Hap43-induced gene; repressed by high-level peroxide stress
PIS56726.1	27.9834949	28.7172457	29.0553008	28.66607406	29.04759336	29.3347145	0.186837625	0.958	0.431	PIS56726.1	MVD	Mevalonate diphosphate decarboxylase; functional homolog of S. cerevisiae Erg19; possible drug target; regulated by carbon source, yeast-hypha switch, growth phase, antifungals; gene has intron; rat catheter, Spider biofilm repressed
PIS54588.1	29.3255828	29.5176715	29.0559694	29.55407743	29.87913819	29.75904089	0.162399451	0.955	0.431	PIS54588.1	PAM17	Predicted component of the presequence translocase-associated import motor (PAM complex) involved in protein import into mitochondrial matrix

PIS54813.1	22.1695097	25.9134633	26.1047446	23.13050489	25.97172616	26.38119009	0.719983777	0.976	0.432	PIS54813.1	HLJ1	Putative HSP40 co-chaperone; repressed during the mating process
PIS48712.1	29.3704608	28.5614361	27.9744958	28.71248613	29.15360232	29.33787556	0.430345283	0.97	0.433	PIS48712.1	DPP3	Protein similar to S. cerevisiae pyrophosphate phosphatase Dpp1; required for farnesol biosynthesis; repressed by 17-beta-estradiol, ethynyl estradiol; Spider biofilm induced
PIS56746.1	30.4368652	30.1578789	30.6596377	30.3595666	31.03982306	31.15665034	0.178955609	0.957	0.434	PIS56746.1	orf19.2057	Ortholog(s) have ATP binding, ATPase, metallopeptidase activity, role in protein-containing complex assembly, signal peptide processing and m-AAA complex, mitochondrial inner boundary membrane, mitochondrial inner membrane [illegible]
PIS48796.1	24.5388752	24.1307296	23.7583446	26.30880867	25.28215694	22.13950233	0.73887388	0.976	0.434	PIS48796.1	orf19.686	Protein of unknown function; regulated by Nrg1
PIS54910.1	26.3125802	24.4790194	26.0958131	26.26621609	25.63857721	26.28894548	0.546418054	0.973	0.435	PIS54910.1	HST3	Histone H3K56 deacetylase; reduced copy number increases opaque cell formation; repressed by MMS, hydroxyurea and high-levels of hydrogen peroxide; Hap43p-induced; ectopic expression blocks genotoxin-induced switching; nicotinamide target [illegible]
PIS58867.1	23.6782056	23.0799592	24.0569925	24.88814389	23.40628697	23.82919144	0.675749276	0.975	0.436	PIS58867.1	LCB4	Putative sphingosine kinase; Tac1p-regulated expression; rat catheter biofilm induced
PIS58247.1	24.695191	23.8972001	24.1240127	25.17947711	24.8666945	23.97733079	0.428121734	0.97	0.436	PIS58247.1	orf19.5165	Ortholog(s) have GTPase activity, enzyme activator activity, zinc chaperone activity and role in cellular response to zinc ion starvation, protein maturation

PIS51420.1	28.3749303	27.9131543	27.9311371	28.29955725	28.33649499	28.89249836	0.283344226	0.964	0.436	PIS51420.1	SEC5	Predicted exocyst component; ortholog of S. cerevisiae Sec5p; merged with orf19.75 in Assembly 21
PIS58750.1	25.6385138	24.945822	24.8566378	27.58973545	24.83832734	24.32512292	0.584914192	0.974	0.437	PIS58750.1	NAM2	Mitochondrial leucyl-tRNA synthetase
PIS58526.1	27.325559	26.9960633	26.846513	28.36397754	26.94743371	27.16719522	0.380266719	0.968	0.437	PIS58526.1	orf19.6458.1	Ortholog(s) have U2 snRNA binding, U6 snRNA binding activity and role in P-body assembly, mRNA splicing, via spliceosome
PIS51408.1	30.5881497	30.5786082	31.0863403	31.32999069	31.11253197	31.12770249	0.15314701	0.954	0.439	PIS51408.1	orf19.4396	Mitochondrial inner membrane protein; mammalian mitofilin domain; Spider biofilm repressed
PIS48575.1	27.5386364	28.1163748	26.5361771	29.25681729	27.23692636	27.01791585	0.511184424	0.972	0.44	PIS48575.1	orf19.5618	Ortholog(s) have SNARE binding, unfolded protein binding activity
PIS54761.1	26.6430637	25.5047504	25.9790238	26.02466307	26.39260892	27.03782039	0.307752408	0.965	0.443	PIS54761.1	orf19.2459	Protein of unknown function; mRNA binds to She3; Hap43 repressed gene; Spider biofilm induced
PIS50485.1	24.6201256	25.9421083	24.5489381	25.02138385	25.64544999	25.77401883	0.340229684	0.967	0.443	PIS50485.1	SFL2	Transcription factor involved in regulation of morphogenesis; regulates transcription in response to carbon dioxide levels; required for filamentous growth, for virulence in RHE model but not in mice; Spider biofilm induced

PIS55080.1	25.0291367	24.3914192	25.0112297	26.74443755	23.99152691	25.02776511	0.566309526	0.973	0.444	PIS55080.1	CBF1	Transcription factor; binds ribosomal protein gene promoters and rDNA locus with Tbf1; regulates sulfur starvation-response, respiratory, glycolytic genes; does not bind to centromeres as does S. cerevisiae Cbf1; Spider biofilm repressed
PIS48216.1	24.4669054	24.2890577	23.6092857	25.37859953	24.550007	23.76979203	0.330284481	0.966	0.444	PIS48216.1	orf19.1430	Ortholog of C. dubliniensis CD36 : Cd36_43870, C. parapsilosis CDC317 : CPAR2_402120, Candida tropicalis MYA-3404 : CTRG_05742 and Candida albicans WO-1 : CAWG_03407
PIS48569.1	23.5925225	23.6214786	23.8230053	24.56463888	23.76413039	24.04286349	0.637181856	0.975	0.445	PIS48569.1	orf19.7029	Putative guanine deaminase; mutation confers hypersensitivity to toxic ergosterol analog; Spider biofilm induced
PIS55694.1	30.3922724	30.7213549	31.3525655	31.99888841	30.83583335	30.9689467	0.232207545	0.961	0.446	PIS55694.1	GUT2	Glycerol-3-phosphate dehydrogenase; Plc1p-regulated; rat catheter biofilm induced; Spider biofilm induced
PIS51576.1	25.6468228	25.3679984	25.6124063	25.36196577	25.9983116	26.60372699	0.451117766	0.971	0.446	PIS51576.1	orf19.6062	Putative TIM23 translocase complex subunit; membrane-localized; Hap43-repressed
PIS48482.1	29.1011063	29.327293	29.8909428	29.39852743	29.89884608	30.36223716	0.199119207	0.959	0.447	PIS48482.1	orf19.2200	Protein of unknown function; rat catheter biofilm repressed
PIS55537.1	27.5900076	28.55424	28.61828	28.73878706	28.26420588	29.10133944	0.469264435	0.971	0.447	PIS55537.1	orf19.5522	Ortholog of C. dubliniensis CD36 : Cd36_62760, C. parapsilosis CDC317 : CPAR2_601700, C. auris B8441 : B9J08_001639 and Candida tenuis NRRL Y-1498 : CANTEDRAFT_115220

PIS55742.1	26.4708418	25.1941904	28.1025706	27.50026095	26.63718926	26.97347924	0.611423196	0.974	0.448	PIS55742.1	DAP1	Similar to mammalian membrane-associated progesterone receptors involved in DNA damage response; induced in core stress response; Hog1 regulated; clade-associated expression; Hap43-repressed
PIS51380.1	24.0825468	24.0465179	25.653767	25.12368852	25.16272803	24.84027568	0.497090298	0.972	0.448	PIS51380.1	orf19.6874	Putative helix-loop-helix (HLH) transcription factor with a role in filamentous growth
PIS56615.1	28.5770099	27.5776853	27.3181292	27.53867656	28.75210933	28.53309035	0.30122512	0.965	0.45	PIS56615.1	orf19.1864	Ortholog(s) have protein-macromolecule adaptor activity and role in proteasome-mediated ubiquitin-dependent protein catabolic process, rescue of stalled ribosome, ribosome-associated ubiquitin-dependent protein catabolic process
PIS50534.1	24.6335569	23.4693151	23.0063363	22.44141842	25.20041378	24.81706593	0.644472349	0.975	0.45	PIS50534.1	orf19.3919.1	Putative adhesin-like protein
PIS55752.1	29.7274104	29.0424079	28.9113878	29.84372774	29.61382564	29.57463081	0.126677045	0.95	0.45	PIS55752.1	SEC8	Predicted subunit of the exocyst complex, involved in exocytosis; localizes to a crescent on the surface of the hyphal tip
PIS58370.1	24.4059896	24.8824074	23.564418	25.05283602	24.57666398	24.5785617	0.472093468	0.971	0.452	PIS58370.1	VMA7	Putative subunit of the V-ATPase complex, which is involved in control of vacuolar pH; highly similar to S. cerevisiae Vma7p; interacts with phosphatidylinositol 3-kinase Vps34p
PIS51367.1	23.9223164	23.9880603	23.8423154	24.48353192	24.17293295	24.47099458	0.548560867	0.973	0.458	PIS51367.1	orf19.190	Ortholog(s) have role in chromatin remodeling, endoplasmic reticulum organization and Swr1 complex localization

PIS49570.1	28.465573	28.7648747	28.6555589	28.9023392	28.83343982	29.5245901	0.364944295	0.968	0.458	PIS49570.1	orf19.4203	Ortholog(s) have structural constituent of nuclear pore activity, role in nuclear pore organization, spindle pole body duplication and nuclear envelope, nuclear pore, nuclear pore transmembrane ring, spindle pole body localization
PIS48432.1	24.3583661	24.3401981	24.1061683	25.6072414	24.17399431	24.40075494	0.46556486	0.971	0.459	PIS48432.1	orf19.2363	Ortholog(s) have thiosulfate sulfurtransferase activity and role in sulfur compound metabolic process
PIS58037.1	26.9369952	25.4899066	26.1058965	26.86499828	26.77211452	26.27436115	0.427432163	0.97	0.46	PIS58037.1	orf19.1195	Putative metalloendopeptidase; orthologs are involved in cellular iron ion homeostasis, protein processing involved in protein targeting to mitochondrion, protein stabilization, and mitochondrial matrix localization
PIS48307.1	30.268476	30.313984	30.88907	31.19542208	30.85469364	30.80566003	0.103453435	0.946	0.461	PIS48307.1	DPS1-1	Putative tRNA-Asp synthetase; genes encoding ribosomal subunits, translation factors, tRNA synthetases are downregulated upon phagocytosis by murine macrophage; protein enriched in stationary phase yeast cultures
PIS52175.1	28.2634627	28.3185816	28.3358089	27.8208122	28.71000245	29.77476047	0.401648771	0.969	0.463	PIS52175.1	orf19.6705	Putative guanyl nucleotide exchange factor with Sec7 domain; required for normal filamentous growth; regulated by yeast-hyphal switch; filament induced; regulated by Nrg1, Tup1, Mob2, Hap43; mRNA binds She3; Spider biofilm induced
PIS51992.1	24.8479281	24.2954875	24.5410025	26.86110351	24.34585126	23.87091779	0.567176899	0.973	0.464	PIS51992.1	orf19.6233	Ortholog of S. cerevisiae : PBR1, C. glabrata CBS138 : CAGL0J11264g, C. dubliniensis CD36 : Cd36_06340, C. parapsilosis CDC317 : CPAR2_206960 and C. auris B8441 : B9J08_003603
PIS56573.1	26.1976402	27.7102974	26.7060037	27.80422023	27.69581879	26.51143504	0.406012302	0.969	0.466	PIS56573.1	orf19.6246	Ortholog(s) have peptide alpha-N-acetyltransferase activity and role in N-terminal peptidyl-methionine acetylation, mitochondrion inheritance, regulation of actin cytoskeleton organization, ubiquitin-dependent ERAD pathway

PIS55477.1	29.2199786	28.3661399	27.2802807	29.40271674	28.5009228	28.36194593	0.320538563	0.966	0.466	PIS55477.1	PLD1	Phospholipase D1; required for phosphatidic acid and for most diacylglycerol production; required for wild-type mouse virulence, but not rat oral virulence; mutant defect in hyphal growth on solid substrates; similar to S. cerevisiae Spo14p
PIS58174.1	27.1798901	26.1843338	25.2950965	26.62927801	27.12709794	26.31016825	0.383847455	0.969	0.469	PIS58174.1	orf19.3848	Ortholog of C. dubliniensis CD36 : Cd36_31420, C. parapsilosis CDC317 : CPAR2_205100, C. auris B8441 : B9J08_000664, Debaryomyces hansenii CBS767 : DEHA2G10648g and Pichia stipitis Pignal : PICST_32997
PIS51710.1	26.7487543	26.8293598	27.0701806	28.21326852	26.94537649	26.89797719	0.547642834	0.973	0.469	PIS51710.1	PEP8	Protein similar to S. cerevisiae Pep8p, which is involved in retrograde transport; transposon mutation affects filamentous growth
PIS51858.1	28.7654536	28.0792343	27.8782751	29.45208519	28.53808194	28.14682711	0.476505239	0.971	0.471	PIS51858.1	SWI1	Protein involved in transcription regulation; ortholog of S. cerevisiae Swi1p, which is a subunit of the SWI/SNF chromatin remodeling complex; interacts with Snf2p; SWI/SNF complex is essential for hyphal growth and virulence
PIS51065.1	25.1329033	26.9858589	26.3810398	26.5807401	26.19193165	27.142199	0.462365512	0.971	0.472	PIS51065.1	orf19.1630	Putative RNA-binding protein involved in translational regulation; null mutant shows reduced autophagy in response to DNA damage and nitrogen starvation, possibly due to effects on protein levels of Atg1p and Atg13p
PIS52390.1	28.951069	28.9513466	28.2707912	29.81838869	28.9898435	28.7825881	0.290035873	0.965	0.473	PIS52390.1	orf19.5420	Ortholog(s) have structural constituent of ribosome activity, role in mitochondrial translation and mitochondrial large ribosomal subunit localization
PIS55456.1	26.5024064	26.373379	24.3698246	26.2440329	25.70221862	26.72267123	0.342911107	0.967	0.474	PIS55456.1	orf19.3737	Protein with a Vps9 vacuolar protein sorting protein domain; Hap43-repressed; repressed by ciclopirox olamine; Spider biofilm induced

PIS55576.1	26.375595	26.586979	27.3424756	27.21247773	27.2645587	27.25109781	0.393369492	0.969	0.474	PIS55576.1	orf19.6943	Predicted ORF from Assembly 19; removed from Assembly 20; subsequently reinstated in Assembly 21 based on comparative genome analysis
PIS51345.1	24.8510108	27.1848821	26.0661594	26.86639995	26.19048645	26.47110454	0.369566646	0.968	0.475	PIS51345.1	SMD3	Putative core snRNP protein; induced upon adherence to polystyrene
PIS51316.1	24.1261186	23.6191462	23.7025238	25.32615241	24.52346141	23.02554211	0.498092382	0.972	0.476	PIS51316.1	orf19.4904	Ortholog(s) have nucleosome binding activity, role in chromatin remodeling and Swr1 complex, cytoplasm, nuclear periphery localization
PIS49671.1	31.8688803	31.2383374	31.3764073	32.24951495	31.87310198	31.78827163	0.326982075	0.966	0.476	PIS49671.1	THR4	Putative threonine synthase; protein present in exponential and stationary growth phase yeast cultures; Gcn4p-regulated; sumoylation target
PIS49650.1	24.0896719	25.0701404	24.2260277	25.04734823	24.61599108	25.15223194	0.651812863	0.975	0.477	PIS49650.1	orf19.6492	Predicted protein serine/threonine kinase and/or protein tyrosine kinase; Spider biofilm induced
PIS51838.1	26.0453622	25.0574949	25.4292921	26.33196959	26.07586717	25.55624929	0.204637137	0.959	0.477	PIS51838.1	orf19.90	Ortholog(s) have sterol transfer activity and endoplasmic reticulum-plasma membrane contact site, perinuclear endoplasmic reticulum membrane localization
PIS55650.1	29.7022613	29.6967201	29.8636858	29.81759684	30.05630729	30.81846758	0.248447128	0.962	0.477	PIS55650.1	TIP120	Protein similar to human CAND1 (Cullin-Associated Nedd8-Dissociated) protein involved in regulation of SCF complexes; binds unnedddylated cullin Cdc53; mutants are viable

PIS56871.1	27.035596	27.5523721	25.2843499	27.41785955	27.00737263	26.88411315	0.43273543	0.97	0.479	PIS56871.1	orf19.2703	Specificity factor required for ubiquitination and sorting of specific cargo proteins at the multivesicular body; ortholog of S. cerevisiae Ear1; transcript regulated by iron; Ssr1-represssed; Spider biofilm induced
PIS55466.1	27.6762734	27.8097347	28.3767133	28.33263715	28.61369022	28.35461869	0.369362916	0.968	0.479	PIS55466.1	orf19.92	Protein that stimulates actin assembly; interacts with polarisome components Bni1p and Bud6p; Hap43-repressed; induced by prostaglandins
PIS48625.1	26.9723618	28.3956692	26.0887972	28.44084486	27.24532673	27.2113294	0.38463436	0.969	0.48	PIS48625.1	orf19.835	Ortholog(s) have role in chromatin remodeling and Ino80 complex, cytosol, nucleus localization
PIS49790.1	26.8714588	26.7085311	26.8178021	26.90748888	27.36707304	27.56743477	0.220348924	0.96	0.481	PIS49790.1	orf19.3183	Protein with a role in insertion of tail-anchored proteins into the ER membrane; required for efficient mating, in shmoo formation and nuclear migration in the pre-zygote of S. cerevisiae; Hap43-repressed
PIS48817.1	24.334462	26.0557497	24.5910052	25.76073348	26.09414877	24.56954484	0.379950082	0.968	0.481	PIS48817.1	orf19.4807	Ortholog(s) have inorganic diphosphate phosphatase activity, role in aerobic respiration and mitochondrion localization
PIS52191.1	30.6269151	30.6097988	30.8032768	31.54928867	30.9587281	30.97967132	0.183135001	0.957	0.483	PIS52191.1	CYC1	Cytochrome c; complements defects of S. cerevisiae cyc1 cyc7 double mutant; induced in high iron; alkaline repressed; repressed by nitric oxide; Hap43-dependent repression in low iron; regulated by Sef1, Sfu1
PIS55462.1	24.7341945	24.4865827	24.8157549	24.31923896	23.27170902	27.89593009	0.772189421	0.977	0.483	PIS55462.1	IFM1	Putative mitochondrial translation initiation factor; transcript regulated by Nrg1, Mig1, and Tup1

PIS49758.1	26.1129893	27.8157738	27.7006637	26.95420785	27.68790695	28.43891418	0.508601312	0.972	0.484	PIS49758.1	orf19.223	Putative serine/threonine protein kinase; Hap43-repressed; induced by prostaglandins; appears to be a downstream target of Ypk1; flow model biofilm induced; Spider biofilm induced
PIS55683.1	25.5860236	23.9238273	24.6605275	25.14975155	24.75706679	25.71935941	0.371801353	0.968	0.485	PIS55683.1	RGT1	Zn(II)2Cys6 transcription factor; transcriptional repressor involved in the regulation of glucose transporter genes; ortholog of S. cerevisiae Rgt1; mutants display decreased colonization of mouse kidneys
PIS58242.1	25.2893313	25.2870794	25.4412119	26.61165966	25.33729886	25.52630118	0.467485458	0.971	0.486	PIS58242.1	orf19.5160	Regulatory subunit of PP2A-like protein phosphatase Sit4p, involved in cell wall maintenance, regulation of hyphal growth, and virulence
PIS48356.1	29.3934396	29.5600894	30.1967573	30.11868231	30.1252386	30.36477943	0.124421322	0.95	0.486	PIS48356.1	orf19.585	Ortholog(s) have structural constituent of ribosome activity and mitochondrial large ribosomal subunit localization
PIS55836.1	23.8175477	23.4974448	23.5611637	24.45557028	24.18760163	23.69513641	0.370020263	0.968	0.487	PIS55836.1	orf19.3163	Ortholog of C. dubliniensis CD36 : Cd36_51910, C. parapsilosis CDC317 : CPAR2_101650, C. auris B8441 : B9J08_001942 and Candida tenuis NRRL Y-1498 : CANTEDRAFT_94797
PIS55674.1	27.714681	26.6289472	25.8132324	27.52012984	27.5428319	26.55468769	0.349944801	0.967	0.487	PIS55674.1	orf19.5457	Ortholog(s) have lipid droplet localization
PIS55500.1	29.9049577	29.3544567	28.6833084	29.95984497	29.75230965	29.69476571	0.225371381	0.961	0.488	PIS55500.1	orf19.5052	Ortholog of S. cerevisiae : MRX20, C. glabrata CBS138 : CAGL0C02013g, C. dubliniensis CD36 : Cd36_07340, C. parapsilosis CDC317 : CPAR2_208340 and C. auris B8441 : B9J08_001600

PIS48323.1	25.094698	24.5543451	24.0302338	24.75653833	24.96265229	25.43422922	0.24663914	0.962	0.491	PIS48323.1	CDC7	Catalytic subunit of Dbf4p-regulated serine/threonine protein kinase; negative regulator of hyphal development; cell-cycle regulated periodic mRNA expression; S. cerevisiae ortholog is not cell-cycle regulated
PIS49595.1	28.7093674	29.1159293	28.5274576	29.03705298	29.15128705	29.63597635	0.210299175	0.96	0.491	PIS49595.1	orf19.4537	Ortholog(s) have RNA polymerase II complex binding, RNA polymerase II complex recruiting activity, RNA polymerase II transcription regulatory region sequence-specific DNA binding activity
PIS56527.1	26.6963928	26.9614704	26.3453576	27.3065217	27.28520772	26.8875697	0.227408966	0.961	0.492	PIS56527.1	orf19.3482	Ortholog(s) have NAD+ diphosphatase activity, role in NAD-cap decapping, NADH metabolic process, RNA decapping and peroxisome localization
PIS49574.1	26.7636448	25.474316	24.5764488	27.34708451	25.9887995	24.95541153	0.499065941	0.972	0.492	PIS49574.1	PRP3	Predicted splicing factor, component of the U4/U6-U5 snRNP complex; Hap43-induced gene; rat catheter biofilm induced
PIS54479.1	26.9331519	24.3839094	26.4755171	27.18700227	26.27685127	25.80688016	0.686656744	0.976	0.493	PIS54479.1	orf19.7097	Putative cytoplasmic RNA-binding protein; heterozygous null mutant exhibits hypersensitivity to parnafungin and cordycepin in C. albicans fitness test; Spider biofilm repressed
PIS58832.1	27.1951383	26.737221	27.662986	27.43461494	27.29261945	28.34955416	0.27394743	0.964	0.494	PIS58832.1	MAK21	Putative 66S pre-ribosomal particle subunit; mutation confers hypersensitivity to tubercidin (7-deazaadenosine)
PIS52052.1	30.2593986	29.7951555	29.7285846	30.06221432	30.31496977	30.88703448	0.137803438	0.952	0.494	PIS52052.1	MDL2	Putative mitochondrial, half-size MDR-subfamily ABC transporter

PIS51140.1	28.3028558	28.023746	28.2783683	27.87172798	28.85856303	29.35808652	0.313386299	0.966	0.494	PIS51140.1	orf19.5129	Ortholog of C. dubliniensis CD36 : Cd36_72910, C. parapsilosis CDC317 : CPAR2_704100, C. auris B8441 : B9J08_002713 and Candida tenuis NRRL Y-1498 : CANTEDRAFT_113193
PIS58543.1	28.8679493	28.1179097	28.5263983	28.8810486	29.18729794	28.93823814	0.202430338	0.959	0.498	PIS58543.1	orf19.3351	Protein of unknown function; Hap43-induced; Spider biofilm induced
PIS48551.1	29.6325046	28.7709753	28.189821	29.81518859	29.14235078	29.13769926	0.363047606	0.968	0.501	PIS48551.1	PGA4	GPI-anchored cell surface protein; beta-1,3-glucanosyltransferase with similarity to the A. fumigatus GEL family; transcript induced in RHE model of oral candidiasis; fluconazol-induced
PIS51560.1	29.9786647	28.6325129	29.1680423	30.45386114	29.5565305	29.27461217	0.328213538	0.966	0.502	PIS51560.1	BBC1	Putative SH3-domain-containing protein
PIS58405.1	25.3166278	22.7277959	25.3952896	25.30068902	25.07451327	24.57114518	0.451755796	0.971	0.502	PIS58405.1	orf19.6984	Ortholog of C. dubliniensis CD36 : Cd36_85460, C. parapsilosis CDC317 : CPAR2_405760, C. auris B8441 : B9J08_000903 and Candida tenuis NRRL Y-1498 : CANTEDRAFT_116052
PIS51437.1	30.1869488	29.2306056	25.1637234	29.98660012	29.09644559	27.00698151	0.705342445	0.976	0.503	PIS51437.1	orf19.4701	Ortholog(s) have ATPase, tRNA binding activity, role in protein urmylation, regulation of transcription by RNA polymerase II, tRNA wobble uridine modification and elongator holoenzyme complex localization
PIS58475.1	26.4970513	25.8043769	24.9221915	26.41907281	25.87910029	26.44125344	0.565209517	0.973	0.505	PIS58475.1	orf19.2961	Putative transcription factor with zinc finger DNA-binding motif

PIS52425.1	30.1834183	29.3816125	27.6538965	29.94248313	29.17760855	29.6151286	0.512297741	0.972	0.505	PIS52425.1	orf19.4888	Has domain(s) with predicted RNA binding, nucleic acid binding activity
PIS58638.1	25.7730882	25.8758252	25.2409312	28.33545491	25.00863417	25.06259341	0.613399334	0.974	0.506	PIS58638.1	ATP14	Putative mitochondrial F1F0 ATP synthase subunit; macrophage/pseudohyphal-induced
PIS54647.1	28.5642293	27.8281749	28.8736854	29.23660954	28.46813445	29.07962671	0.26998661	0.964	0.506	PIS54647.1	orf19.200	Putative nuclear RNA-binding protein; Spider biofilm repressed
PIS58679.1	24.8850502	25.9575927	25.8269123	27.29153983	25.62321719	25.27506872	0.432859585	0.97	0.507	PIS58679.1	ARH2	Putative adrenodoxin-NADPH oxidoreductase; role in heme biosynthesis
PIS58251.1	24.2804289	24.3749689	24.7702552	25.97581479	24.1478092	24.82571667	0.337788508	0.967	0.508	PIS58251.1	orf19.4409	Ortholog(s) have nucleoside triphosphate diphosphatase activity, ribonucleoside triphosphate phosphatase activity and role in cellular response to phosphate starvation, nucleoside triphosphate metabolic process
PIS48518.1	26.1799035	25.8855599	25.4111171	26.08108295	26.16637789	26.75263357	0.34387395	0.967	0.508	PIS48518.1	SRB8	Putative RNA polymerase II mediator complex subunit; early-stage flow model biofilm induced
PIS51433.1	26.6274633	25.2720928	25.3773042	27.27248645	25.02695101	26.5013407	0.459229312	0.971	0.508	PIS51433.1	SRP40	Putative chaperone of small nucleolar ribonucleoprotein particles; macrophage/pseudohyphal-induced; rat catheter biofilm induced

PIS51578.1	31.3282785	31.6400957	31.3839783	32.2996664	31.95023474	31.63343473	0.314238251	0.966	0.51	PIS51578.1	GCN20	YEF3-subfamily ABC family protein, predicted not to be a transporter
PIS51018.1	28.0240082	27.4460251	26.7196296	27.93893262	27.97950623	27.80222985	0.35774418	0.968	0.51	PIS51018.1	orf19.6077	Putative protein of unknown function; shows colony morphology-related gene regulation by Ssn6p
PIS51159.1	28.6630615	27.4865319	26.3132774	28.09187751	27.91977218	27.9807845	0.328706311	0.966	0.51	PIS51159.1	orf19.6328	Putative protein of the mitochondrial intermembrane space; predicted role in acetate utilization and gluconeogenesis; Spider biofilm repressed
PIS56671.1	23.4842033	23.9669681	24.4623052	24.98886131	24.66604024	23.78973155	0.429728963	0.97	0.51	PIS56671.1	orf19.6722	Protein similar to S. cerevisiae Rad4p; down-regulation associated with azole resistance
PIS58320.1	25.8792993	25.0131905	24.4146582	25.50466824	25.60630873	25.73235096	0.323187361	0.966	0.512	PIS58320.1	GYP8	Ortholog(s) have GTPase activator activity, role in vesicle-mediated transport and cytoplasm, peroxisome localization
PIS54717.1	26.541116	22.9336654	25.7883203	28.30011483	24.86003469	23.64554679	0.642479999	0.975	0.514	PIS54717.1	orf19.3854	Ortholog of S. cerevisiae Sat4; amphotericin B induced; clade-associated gene expression; Spider biofilm induced
PIS51125.1	27.512474	27.0773414	26.605716	27.56220235	27.7869774	27.38965527	0.23315194	0.961	0.514	PIS51125.1	orf19.642	Regulatory subunit of PP2A-like protein phosphatase Sit4p, involved in cell wall maintenance, regulation of hyphal growth, and virulence

PIS50309.1	29.6215737	28.8336167	28.0547629	29.15473266	29.3301213	29.56644338	0.237000124	0.962	0.514	PIS50309.1	orf19.7643	Ortholog(s) have 4-hydroxybenzoate octaprenyltransferase activity, role in ubiquinone biosynthetic process and mitochondrial inner membrane, mitochondrial membrane, mitochondrion localization
PIS48650.1	28.2795501	28.191145	27.8394498	28.2755523	28.735233	28.84413482	0.097242497	0.945	0.515	PIS48650.1	CWH43	Putative sensor/transporter protein with a predicted role in cell wall biogenesis; possibly an essential gene, disruptants not obtained by UAU1 method; flow model and rat catheter biofilm repressed
PIS51265.1	21.2630716	22.4260823	20.9725134	22.17478694	21.63677628	22.39585368	0.322690657	0.966	0.515	PIS51265.1	MSN4	Zinc finger transcription factor; similar to S. cerevisiae Msn4, but not a significant stress response regulator in C. albicans; partly complements STRE-activation defect of S. cerevisiae msn2 msn4 double mutant; flow model biofilm induced
PIS55821.1	26.452453	27.7022154	27.103179	28.04928719	27.63920939	27.11403274	0.372631952	0.968	0.515	PIS55821.1	orf19.1214	Ortholog(s) have metalloaminopeptidase activity and role in protein maturation
PIS54529.1	28.424081	28.7583275	29.1686658	28.9826032	29.56964483	29.35412566	0.156990732	0.954	0.518	PIS54529.1	ARG5%2C6	activity; Gcn4 regulated; alkaline repressed; Spider biofilm induced
PIS54484.1	30.5297153	29.9944728	29.2804938	30.76792392	30.40879828	30.18657439	0.156661921	0.954	0.52	PIS54484.1	CCC1	Manganese transporter; required for normal filamentous growth; mRNA binds She3, localized to hyphal tips; repressed by NO, alkaline pH; colony morphology-related regulation by Ssn6; regulated by Sef1, Sfu1, Hap43; Spider biofilm induced
PIS58723.1	26.0349005	25.5985962	24.8780225	25.97057425	26.01926679	26.08241143	0.262389646	0.963	0.52	PIS58723.1	ITR1	MFS inositol transporter; uptake of exogenous inositol; 12 transmembrane motifs; expressed in rich medium; fluconazole, caspofungin repressed; possibly essential (UAU1 method); flow model, rat catheter and Spider biofilm induced

PIS50408.1	25.2065611	23.1927456	24.8935185	25.27687443	25.22473458	24.35986191	0.435890066	0.97	0.523	PIS50408.1	YPD1	Phosphohistidine intermediate protein in a phosphorelay signal transduction pathway; residue His69 is the phosphoacceptor histidine; predicted to be soluble and cytosolic; functional homolog of S. cerevisiae Ypd1p
PIS51348.1	26.7397479	27.1630592	27.8422493	28.83829841	27.40305283	27.07501597	0.396391823	0.969	0.524	PIS51348.1	orf19.4144	Predicted protein kinase; clade-associated gene expression
PIS56961.1	29.8664626	29.692844	29.0304614	29.85469523	30.21577276	30.09105729	0.119225376	0.949	0.524	PIS56961.1	STE24	Prenyl-dependent protease
PIS58842.1	27.3785973	27.1561431	27.1726188	27.4833567	27.62277823	28.17873778	0.189832116	0.958	0.526	PIS58842.1	SEC15	Exocyst subunit, involved in polarized growth; cellular bud tip-associated protein; interacts with Ras-related GTPase Rsr1p; ortholog of S. cerevisiae SEC15
PIS49474.1	29.0788336	27.6959902	27.9883673	28.99022699	28.62321553	28.73034546	0.271364076	0.964	0.527	PIS49474.1	orf19.7307	Putative oxidoreductase; similar to S. cerevisiae Pga3p; possible Kex2p substrate
PIS54506.1	24.263408	24.4505705	25.9577024	25.58674318	25.52517386	25.14401073	0.526838126	0.973	0.528	PIS54506.1	GAP4	High-affinity S-adenosylmethionine permease; required for SAM-induced morphogenesis; hyphal induced; regulated by Hap43, Gcn2 and Gcn4; colony morphology-related gene regulation by Ssnp
PIS58312.1	27.0674168	27.7183512	27.8199943	28.51095047	28.19742573	27.48147092	0.205526787	0.959	0.528	PIS58312.1	orf19.3881	Ortholog of C. dubliniensis CD36 : Cd36_31790, C. parapsilosis CDC317 : CPAR2_204900, C. auris B8441 : B9J08_000806 and Candida tenuis NRRL Y-1498 : CANTEDRAFT_136864

PIS49827.1	29.5773893	28.1869047	29.3745241	30.80042878	29.13586588	28.78536944	0.514504028	0.972	0.528	PIS49827.1	orf19.4368	Ortholog of C. dubliniensis CD36 : Cd36_28890, C. parapsilosis CDC317 : CPAR2_801690, C. auris B8441 : B9J08_004854 and Candida tenuis NRRL Y-1498 : CANTEDRAFT_106471
PIS55604.1	26.6327662	27.61029	28.8292824	28.03115688	28.38029975	28.24739484	0.29867837	0.965	0.529	PIS55604.1	orf19.5698	Putative mitochondrial ribosomal protein of the large subunit; transcript is upregulated in clinical isolates from HIV+ patients with oral candidiasis; Spider biofilm repressed
PIS58061.1	26.5588306	25.9443515	26.2250302	25.52413725	27.21064184	27.5841345	0.545112191	0.973	0.53	PIS58061.1	orf19.3453	Ortholog(s) have GTPase activator activity, role in establishment or maintenance of cell polarity, positive regulation of GTPase activity and cytoplasmic vesicle membrane, plasma membrane localization
PIS51158.1	30.2902091	29.7890364	29.8094	30.7455082	30.42845854	30.31640613	0.076183119	0.939	0.534	PIS51158.1	HET1	Putative sphingolipid transfer protein; involved in localization of glucosylceramide which is important for virulence; Spider biofilm repressed
PIS49749.1	25.1801577	24.4286587	25.9052259	25.6383268	25.638085	25.84082741	0.219907426	0.96	0.534	PIS49749.1	orf19.4736	Ortholog(s) have alkaline phosphatase activity, zinc ion sensor activity, role in nicotinamide nucleotide metabolic process and fungal-type vacuole, fungal-type vacuole membrane localization
PIS58117.1	32.5663648	32.5188862	32.9385541	33.59533575	32.86583374	33.16618953	0.072470671	0.937	0.535	PIS58117.1	RPS14B	Putative ribosomal protein; repressed upon phagocytosis by murine macrophage; transcript positively regulated by Tbf1; Spider biofilm repressed
PIS49779.1	25.5172552	26.0653976	25.8538975	25.90823532	27.21757805	25.91424195	0.546396122	0.973	0.535	PIS49779.1	TFC4	Putative RNA polymerase III transcription initiation factor complex (TFIIIC) subunit; induced by Mnl1 under weak acid stress; possibly essential gene, disruptants not obtained by UAU1 method

PIS58591.1	29.3989606	29.2810481	29.2583656	30.08653681	29.82138243	29.64286049	0.241371934	0.962	0.537	PIS58591.1	CYM1	Putative metalloprotease of the mitochondrial intermembrane space; rat catheter biofilm induced
PIS58420.1	24.1433709	24.0801492	23.9922091	24.58958091	24.92623502	24.31617682	0.282585659	0.964	0.539	PIS58420.1	PHR1	Cell surface glycosidase; may act on cell-wall beta-1,3-glucan prior to beta-1,6-glucan linkage; role in systemic, not vaginal virulence (neutral, not low pH); high pH or filamentation induced; Bcr1-repressed in RPMI a/a biofilm
PIS48710.1	24.3828539	24.2408493	22.6474164	25.47121113	23.64699038	23.77247125	0.349899383	0.967	0.54	PIS48710.1	GCN2	Translation initiation factor 2-alpha (eIF2alpha) kinase; has nonessential role in amino acid starvation response, in contrast to S. cerevisiae homolog; similar to S. cerevisiae Gcn2p
PIS48241.1	25.0026821	24.5490582	24.5591951	25.13402511	25.35900678	25.23774704	0.307692969	0.965	0.54	PIS48241.1	orf19.6525	S. cerevisiae ortholog Inp1 is a peripheral membrane protein of peroxisomes involved in peroxisomal inheritance; induced by Mnl1 under weak acid stress
PIS52222.1	26.1139101	25.2447092	25.346094	25.86150134	26.10857951	26.36289961	0.171198289	0.956	0.543	PIS52222.1	HXK1	N-acetylglucosamine (GlcNAc) kinase; involved in GlcNAc utilization; required for wild-type hyphal growth and mouse virulence; GlcNAc-induced transcript; induced by alpha pheromone in SpiderM medium
PIS52238.1	24.6945732	26.2794302	25.4075087	26.40487825	25.80912791	25.79868523	0.298748555	0.965	0.544	PIS52238.1	DCW1	Protein with predicted GPI modification; dfg5 dcw1 double mutant is inviable; not required for wild-type hyphal growth; upregulated in cyr1 mutant (yeast or hyphal form); Hap43-induced
PIS50338.1	25.3717319	24.4602158	24.8809959	25.19237442	25.5777206	25.57467663	0.302479048	0.965	0.544	PIS50338.1	HBR1	Essential protein involved in regulation of MTL gene expression; hemoglobin-regulated inhibitor of white-opaque switching, may affect survival in host; activator of MTLalpha1 and MTLalpha2; transcript activated by [illegible]

PIS48612.1	28.805045	28.6872622	29.101988	29.13869748	29.34171362	29.74911553	0.442393452	0.97	0.545	PIS48612.1	MSH2	Putative DNA mismatch repair factor; transcript regulated by Nrg1; flucytosine repressed; transcript regulated by tyrosol and cell density
PIS56551.1	28.3343514	28.7058077	28.6205443	29.82512873	29.03029844	28.44146035	0.130478238	0.951	0.545	PIS56551.1	orf19.3547	Ortholog(s) have large ribosomal subunit rRNA binding, mRNA 3'-UTR binding, mRNA 5'-UTR binding, mRNA regulatory element binding translation repressor activity
PIS52018.1	25.7793809	27.445299	27.2558539	27.17036042	27.54654695	27.40102643	0.236906845	0.962	0.546	PIS52018.1	GCD7	Putative translation initiator; downregulated in the presence of human whole blood or polymorphonuclear (PMN) cells
PIS58549.1	32.3119439	31.468043	31.7430562	32.69239259	32.13400043	32.33629781	0.092000186	0.943	0.547	PIS58549.1	orf19.3341	Putative tRNA-Arg synthetase; essential; genes encoding ribosomal subunits, translation factors, and tRNA synthetases are downregulated upon phagocytosis by murine macrophage; downregulated by growth in the mouse cecum
PIS51430.1	28.1842176	28.1868201	28.0770482	28.66921454	29.24878532	28.17561552	0.131921322	0.951	0.549	PIS51430.1	orf19.2852	Subunit of the mitochondrial ribosome; identified in a mutant screen as necessary for filamentation inside phagocytes
PIS56859.1	27.8538191	27.2159816	26.686793	27.10998541	28.12911419	28.16590912	0.285888432	0.964	0.549	PIS56859.1	orf19.4574	Ortholog(s) have lysophospholipase activity, role in lipid homeostasis and lipid droplet localization
PIS51589.1	29.262682	29.5390672	23.6040047	27.38926939	28.06034938	28.60653815	0.747723362	0.977	0.55	PIS51589.1	COX8	Putative cytochrome c oxidase; flucytosine induced; caspofungin repressed

PIS58868.1	22.1213962	18.7838793	21.7613077	23.9205802	18.68153922	21.71319208	0.712673989	0.976	0.55	PIS58868.1	ECM21	Predicted regulator of endcytosis of plasma membrane proteins; fluconazole induced, alkaline induced by Rim101; repressed by caspofungin and in azole-resistant strain overexpressing MDR1; flow model, rat catheter and Spider biofilm induced
PIS52310.1	29.7124863	29.3786218	29.2319892	30.44893605	29.73183626	29.79284236	0.171612572	0.956	0.55	PIS52310.1	IRA2	GTPase-activating protein; negatively regulates RAS by converting it from the GTP- to the GDP-bound inactive form; Spider biofilm induced; flow model biofilm repressed
PIS58539.1	31.1371724	31.3632681	31.0988311	31.91951453	31.46340981	31.86764535	0.058965393	0.932	0.55	PIS58539.1	orf19.4246	Phosphatase involved in stress response; Tn mutation affects filamentation; Hog1-repressed; colony morphology-related gene regulation by Ssn6p; induced during cell wall regeneration
PIS49643.1	25.6028812	25.5891355	26.7943559	26.312524	26.49288126	26.83497566	0.372050073	0.968	0.551	PIS49643.1	MRPL27	Putative 60S ribosomal protein L27, mitochondrial precursor
PIS50352.1	22.3443871	24.4993486	24.365476	24.29034213	23.68910016	24.8829534	0.626876874	0.975	0.551	PIS50352.1	orf19.173	C2H2 transcription factor; induced by Mnl1 under weak acid stress
PIS49536.1	29.9265476	30.2381644	30.6814251	29.88080289	31.05659186	31.56253752	0.242393637	0.962	0.551	PIS49536.1	orf19.5158	Protein with similarity to a human gene associated with colon cancer and to orf19.5158; regulated by Gcn4, Cyr1; induced by amino acid starvation; macrophage-induced protein, macrophage-repressed; Spider biofilm induced
PIS50569.1	25.953276	26.8958872	27.4330102	26.76108982	27.22553457	27.95508652	0.423046315	0.97	0.553	PIS50569.1	TIP41	Protein involved in TOR signaling pathway; regulates protein phosphatase 2A (PP2A) activity; regulates Rad53p during recovery from DNA damage

PIS48698.1	25.5488893	24.1396056	23.3135996	24.93592566	24.9475742	24.78303345	0.416113855	0.97	0.555	PIS48698.1	orf19.131.2	Has domain(s) with predicted protein folding chaperone activity, role in protein insertion into ER membrane and endoplasmic reticulum membrane localization
PIS58541.1	27.4647018	26.9188593	25.7156492	27.15152377	27.39063571	27.22186014	0.292614859	0.965	0.555	PIS58541.1	orf19.178	Has domain(s) with predicted role in peroxisome fission and peroxisomal membrane localization
PIS48599.1	30.0363498	29.4037221	29.3776392	30.5400842	30.33875104	29.60285827	0.202668484	0.959	0.555	PIS48599.1	TIM50	Predicted component of the Translocase of the Inner Mitochondrial membrane (TIM23 complex); involved in mitochondrial protein import; Spider biofilm repressed
PIS51040.1	32.2392289	32.4051427	32.6153115	33.16337791	32.64993286	33.11530582	0.054204256	0.929	0.556	PIS51040.1	ATP17	Mitochondrial ATPase complex subunit; downregulated by Efg1p; flucytosine induced; caspofungin repressed
PIS48771.1	24.6887642	26.8178603	26.5236411	27.23058597	26.77168258	25.69739706	0.399228592	0.969	0.556	PIS48771.1	MCM2	Phosphorylated protein of unknown function; transcription is periodic with a peak at M/G1 phase of the cell cycle
PIS50419.1	27.4037323	28.256668	29.8459998	29.21621593	29.05464586	28.9020632	0.37680005	0.968	0.556	PIS50419.1	orf19.5747	Ortholog(s) have structural constituent of ribosome activity and mitochondrial small ribosomal subunit localization
PIS58229.1	26.8919958	24.0614644	25.1668162	26.23573929	25.99563647	25.55626341	0.432686245	0.97	0.556	PIS58229.1	orf19.6901	Ortholog(s) have 5'-(N(7)-methyl 5'-triphosphoguanosine)-[mRNA] diphosphatase activity, RNA 7-methylguanosine cap binding, exoribonuclease activator activity, hydrolase activity and acting on acid anhydrides, more

PIS51318.1	27.9731018	27.646092	26.253979	27.1491008	28.34788443	28.04875806	0.438468119	0.97	0.558	PIS51318.1	FUN31	Putative PAS kinase involved in cell wall damage response; similar to S. cerevisiae Psk1p, a putative serine/threonine protein kinase; induced by Mnl1 under weak acid stress; rat catheter and Spider biofilm induced
PIS49761.1	27.2726095	26.7983394	26.5811558	27.59849688	27.88204402	26.85400697	0.267432906	0.963	0.561	PIS49761.1	orf19.216	Protein with a metallo-dependent phosphatase domain; ketoconazole-induced; upregulation correlates with clinical development of fluconazole resistance
PIS56763.1	29.6946651	29.1662858	30.1282559	30.68719728	30.12434945	29.86651747	0.13278021	0.951	0.563	PIS56763.1	BDF1	Essential chromatin-binding bromodomain protein; repressed upon adherence to polystyrene; reduced mRNA abundance detected in null mutant; macrophage/pseudohyphal-repressed
PIS52427.1	29.1420552	28.6715884	28.0775811	29.38582031	29.64726229	28.56088471	0.202061847	0.959	0.568	PIS52427.1	DOA1	WD repeat protein; required for ubiquitin-mediated protein degradation; role in control of cellular ubiquitin levels; may promote efficient NHEJ in postdiauxic/stationary phase; Spider biofilm repressed
PIS56937.1	27.6394794	27.3171067	27.9148374	28.41594915	27.99073812	28.16858824	0.165394962	0.955	0.568	PIS56937.1	orf19.849	Ortholog(s) have enzyme activator activity, role in protein N-linked glycosylation, protein O-linked glycosylation and Golgi apparatus localization
PIS48437.1	25.0110289	24.9395624	24.0282744	24.9182769	24.95944288	25.80985873	0.114928431	0.948	0.57	PIS48437.1	orf19.708	Ortholog(s) have gamma-tubulin binding activity
PIS56784.1	24.1674891	23.8147485	22.9806424	24.10133618	24.00156238	24.56867501	0.373512396	0.968	0.57	PIS56784.1	orf19.732	Possible dehydrogenase; flow model biofilm induced; rat catheter biofilm induced; Spider biofilm induced

PIS48540.1	29.2079709	29.3545378	28.8480615	29.63820511	29.57977965	29.9047927	0.103025428	0.946	0.571	PIS48540.1	orf19.4627	Ortholog(s) have structural constituent of nuclear pore activity
PIS51826.1	21.9907254	24.1403001	25.3762386	24.20796633	24.40100363	24.61337061	0.725608417	0.976	0.572	PIS51826.1	orf19.6012	Ortholog(s) have phosphatidylinositol-4,5-bisphosphate binding, phosphatidylserine binding activity and role in endocytosis, site selection
PIS55682.1	24.8106379	24.294887	23.1725401	25.02937779	24.52840784	24.43875602	0.426875776	0.97	0.573	PIS55682.1	ARG83	GAL4-like Zn(II)2Cys6 transcription factor; clade-associated expression; null shows abnormal regulation of invasive colony growth, is unable to utilize proline as a nitrogen source; flow model biofilm induced
PIS48707.1	26.9551555	26.4040551	24.6870865	26.53493151	26.46900319	26.76054504	0.550855838	0.973	0.573	PIS48707.1	orf19.6917	Putative heat shock protein with a zinc finger motif; required for protein import into mitochondria in S. cerevisiae; Spider biofilm induced
PIS58859.1	27.2274527	26.9992954	26.6888995	27.37631123	27.53500854	27.72816159	0.133907689	0.951	0.575	PIS58859.1	orf19.2302	Ortholog(s) have calcium-release channel activity, enzyme regulator activity
PIS48355.1	24.7272904	24.703833	26.5524076	25.2500892	25.93840026	26.51894464	0.269521521	0.964	0.575	PIS48355.1	orf19.581	Putative RNA-binding protein; transcript is upregulated in an RHE model of oral candidiasis
PIS54944.1	26.465471	26.5252107	26.5684063	27.32766178	26.93628809	27.02068782	0.557742932	0.973	0.575	PIS54944.1	UBP1	Ortholog(s) have cysteine-type deubiquitinase activity, role in protein deubiquitination and cytoplasm, endoplasmic reticulum, endoplasmic reticulum membrane localization

PIS50318.1	30.8358815	30.5565739	30.4907633	31.40191024	31.05294086	31.15777183	0.353862693	0.967	0.576	PIS50318.1	BLM3	Putative proteasome activator; binds core proteasome and stimulates proteasome-mediated protein degradation by inducing gate opening; ortholog of S. cerevisiae Blm10; transcript regulated by Nrg1 and Mig1
PIS51611.1	32.2466775	31.6788663	31.4102923	32.73032679	32.01486986	32.31902751	0.487625144	0.972	0.576	PIS51611.1	orf19.1791	Putative protein with a predicted role in 60S ribosomal subunit biogenesis; Hap43p-induced gene; ortholog of S. cerevisiae MAK11
PIS56841.1	26.1639886	25.3616531	24.6476891	27.65989762	25.71784369	24.52209941	0.409922137	0.969	0.576	PIS56841.1	orf19.2710	Ortholog(s) have enzyme activator activity and role in chromosome segregation
PIS51879.1	27.2360463	26.4186973	25.9526469	27.4200452	26.79034179	27.12707943	0.460631384	0.971	0.577	PIS51879.1	orf19.3266	Ortholog of C. dubliniensis CD36 : Cd36_25930, C. parapsilosis CDC317 : CPAR2_800910, C. auris B8441 : B9J08_003483 and Candida tenuis NRRL Y-1498 : CANTEDRAFT_132053
PIS51322.1	30.1911267	30.3337183	30.8330741	30.97397365	31.22717456	30.88737331	0.068627283	0.936	0.577	PIS51322.1	orf19.7459	Putative mitochondrial protein with a predicted role in respiratory growth; fluconazole-induced; ketoconazole-repressed; mutants display a strong defect in flow model biofilm formation; Spider biofilm induced
PIS52163.1	25.4611568	25.3355203	25.6813497	25.93978652	26.37532477	25.89732164	0.23624225	0.962	0.578	PIS52163.1	orf19.3132	Predicted endoplasmic reticulum zinc transporter; induced by nitric oxide
PIS49692.1	30.0627806	30.1878896	30.0464137	30.71376687	30.38605227	30.93373046	0.056769069	0.93	0.579	PIS49692.1	orf19.5095	Putative oxysterol-binding protein; caspofungin induced; possibly an essential gene, disruptants not obtained by UAU1 method

PIS58527.1	26.0907438	25.1869477	25.5291392	27.06350537	25.95918404	25.52161455	0.61154146	0.974	0.579	PIS58527.1	orf19.6457	Ortholog of S. cerevisiae : YBL086C, C. glabrata CBS138 : CAGL0C01815g, C. dubliniensis CD36 : Cd36_72270, C. parapsilosis CDC317 : CPAR2_702850 and C. auris B8441 : B9J08_001027
PIS55784.1	27.3913649	27.5639166	26.0053682	27.78649246	27.17590039	27.73515798	0.191506906	0.958	0.579	PIS55784.1	PPH21	Catalytic subunit of protein phosphatase of the Type 2A-related family (serine/threonine-specific), involved in dephosphorylation of septin Sep7p; caspofungin repressed; possibly an essential gene, disruptants not obtained by UAU1 method
PIS48497.1	23.9992868	24.3033384	25.8655117	24.81579787	25.47153483	25.62139955	0.307996813	0.965	0.58	PIS48497.1	MFG1	Regulator of filamentous growth; required for biofilm formation, virulence; interacts with Flo8 and Mss11
PIS55830.1	24.9755124	24.9718667	25.1592844	26.83636301	25.74167808	24.26885746	0.288242522	0.965	0.58	PIS55830.1	orf19.2350	Protein similar to S. cerevisiae Yor378w; MFS family transporter; transposon mutation affects filamentous growth; null mutants are viable; fungal-specific (no human or murine homolog)
PIS50632.1	23.314555	24.1387753	24.2088345	24.88805914	23.63817577	24.87509913	0.220452946	0.96	0.58	PIS50632.1	PIN3	Putative SH3-domain-containing protein; predicted role in actin cytoskeleton organization; Spider biofilm repressed
PIS54985.1	24.0150006	24.8293682	24.2528668	26.83861174	24.37600713	23.6229188	0.406819797	0.969	0.58	PIS54985.1	UTP13	Putative U3 snoRNA-associated protein; Hap43-induced; repressed in core stress response; physically interacts with TAP-tagged Nop1
PIS51336.1	26.9536134	26.8200093	26.2826265	27.04342399	27.73677167	27.01761952	0.256547352	0.963	0.581	PIS51336.1	FAD1	Ortholog(s) have FMN adenylyltransferase activity, role in FAD biosynthetic process and cytoplasm localization

PIS51027.1	29.7036839	29.2678973	29.6848828	30.18050403	30.20517506	30.01338669	0.166462207	0.956	0.581	PIS51027.1	orf19.2940	Putative v-SNARE of the endoplasmic reticulum membrane; possibly an essential gene, disruptants not obtained by UAU1 method
PIS59032.1	27.0256829	26.226668	26.6213194	27.49696106	26.99900515	27.12258101	0.332587223	0.967	0.582	PIS59032.1	MAF1	Putative negative regulator of RNA polymerase III; decreased expression in hyphae vs yeast cells; caspofungin repressed; Spider biofilm repressed
PIS56552.1	26.2921707	26.8135965	25.9448441	26.78824549	26.86156932	27.14966139	0.106671719	0.947	0.583	PIS56552.1	orf19.4324	Has domain(s) with predicted role in mitochondrial respiratory chain complex I assembly and membrane localization
PIS48381.1	31.1925822	30.3277033	30.3432273	31.67475475	31.40887436	30.52756773	0.148411744	0.953	0.583	PIS48381.1	RSP5	Putative NEDD4 family E3 ubiquitin ligase; induced during infection of murine kidney, compared to growth in vitro; has murine homolog; possibly an essential gene, disruptants not obtained by UAU1 method
PIS58735.1	28.2892483	25.5778283	26.100682	27.63616181	26.90676909	27.17824667	0.326660894	0.966	0.584	PIS58735.1	BZZ1	Protein similar to S. cerevisiae Bzz1p, which is an SH3 domain protein involved in the regulation of actin polymerization
PIS58376.1	24.9644499	24.968789	25.6330067	25.51383216	26.25319001	25.55049824	0.154840322	0.954	0.584	PIS58376.1	YTH1	Putative mRNA cleavage and polyadenylation specificity factor; transcription is regulated upon yeast-hyphal switch; decreased expression in hyphae compared to yeast-form cells; fluconazole or flucytosine induced
PIS58167.1	31.1170723	30.2306859	29.5659426	31.06114957	30.90878477	30.69949988	0.164094993	0.955	0.585	PIS58167.1	ADP1	Putative PDR-subfamily ABC transporter; similar to WHITE subfamily proteins; gene used for strain identification by multilocus sequence typing

PIS51622.1	27.4349116	25.9324768	26.9994058	27.53993307	27.38444496	27.20331086	0.202919423	0.959	0.587	PIS51622.1	HOM3	Putative L-aspartate 4-P-transferase; fungal-specific (no human or murine homolog); regulated by Gcn2 and Gcn4; early-stage flow model biofilm induced
PIS51714.1	28.0362572	28.3398645	28.1980901	28.68976342	28.97830594	28.6686099	0.182960942	0.957	0.587	PIS51714.1	orf19.2500	Mitochondrial protein involved in assembly of NADH ubiquinone oxidoreductase Complex I; required for biofilm formation, respiration and virulence
PIS58697.1	27.1254068	26.3881167	27.5318307	27.83017402	27.54365201	27.43181265	0.333538143	0.967	0.587	PIS58697.1	SLA1	Protein required for assembly of the cortical actin cytoskeleton; contains three SH3 domains; transcription is regulated by Nrg1 and Mig1; flow model biofilm repressed
PIS54614.1	27.0708595	27.5471935	27.2674406	28.14306052	27.68866915	27.81848824	0.132384469	0.951	0.588	PIS54614.1	orf19.1392	Ortholog(s) have protein disulfide isomerase activity, protein-disulfide reductase (glutathione) activity, unfolded protein binding activity and role in protein retention in ER lumen, ubiquitin-dependent ERAD pathway
PIS56637.1	26.6322389	26.2069746	26.492452	26.57511513	27.95392737	26.56699644	0.267194672	0.963	0.588	PIS56637.1	orf19.2574	Ortholog(s) have AP-1 adaptor complex binding activity, role in clathrin-coated vesicle cargo loading and membrane localization
PIS58134.1	27.2791112	27.9464069	28.5331601	29.34266639	28.30749191	27.87164948	0.210465471	0.96	0.588	PIS58134.1	orf19.3010	Putative lipoyl ligase; role in modification of mitochondrial enzymes by attachment of lipoic acid groups; rat catheter biofilm induced
PIS48476.1	28.6251163	28.3489664	27.8643961	28.69803813	29.03637293	28.8740056	0.401301729	0.969	0.59	PIS48476.1	orf19.1267	Ortholog(s) have cytosol, plasma membrane localization

PIS50586.1	25.0679656	24.7715623	24.571548	25.30143964	25.57426801	25.30871187	0.285701455	0.964	0.591	PIS50586.1	orf19.4168	Ortholog(s) have RNA binding, ribonuclease MRP activity, ribonuclease P activity and role in intronic box C/D RNA processing, nuclear-transcribed mRNA catabolic process, RNase MRP-dependent, rRNA processing, tRNA processing
PIS56711.1	29.3803965	27.8379234	28.2658434	29.36499992	29.70642431	28.18437389	0.340450949	0.967	0.591	PIS56711.1	orf19.7675	Mitochondrial ribosomal protein of the large subunit; Spider biofilm repressed
PIS52444.1	27.0774649	27.1527875	26.7649717	27.49756242	27.51391314	27.75847962	0.221915102	0.961	0.592	PIS52444.1	orf19.7370	Possible G-protein coupled receptor; vacuolar membrane transporter for cationic amino acids; PQ-loop motif; rat catheter and Spider biofilm induced
PIS50452.1	26.6847571	25.6600738	27.1370621	26.81089107	28.02790867	26.42173971	0.441568282	0.97	0.593	PIS50452.1	orf19.874	Predicted mitochondrial i-AAA protease supercomplex; degrades misfolded mitochondrial proteins; Hap43-repressed gene
PIS50370.1	28.4080902	28.9917252	28.0316935	30.16634334	28.43942701	28.60663489	0.235603297	0.961	0.594	PIS50370.1	orf19.4751	Ortholog(s) have structural constituent of ribosome activity and mitochondrial small ribosomal subunit localization
PIS48597.1	28.2218865	28.4008288	28.6569245	29.1114501	29.02631927	28.92949505	0.0568599	0.931	0.596	PIS48597.1	CHO1	Phosphatidylserine synthase; binds diacylglycerol and serine; appears to form hexamers comprising three dimers; transposon mutation affects filamentous growth; regulated by Nrg1, Tup1
PIS48280.1	29.0428793	28.9564588	28.5128136	29.366373	29.47134236	29.46283205	0.10296721	0.946	0.596	PIS48280.1	RNR21	Ribonucleoside-diphosphate reductase; regulated by tyrosol and cell density; ciclopirox olamine, fluconazole or flucytosine induced; regulated by Sef1, Sfu1, and Hap43

PIS52462.1	29.052642	28.3676057	27.1012052	28.51406688	28.93743566	28.87126156	0.338792727	0.967	0.6	PIS52462.1	orf19.2829	Ortholog(s) have role in protein transport
PIS51029.1	26.4617217	27.1289916	27.0784313	27.15669417	27.25162064	28.06318735	0.204019105	0.959	0.601	PIS51029.1	orf19.2938	Putative mitochondrial inner membrane protein with a predicted role in the assembly of respiratory complex III; Hap43p-repressed gene; S. cerevisiae ortholog FMP25 localizes to mitochondrion
PIS49587.1	27.1648722	27.5596307	27.6332282	28.45064908	27.67095835	28.04290046	0.306242454	0.965	0.602	PIS49587.1	orf19.748	Ortholog(s) have RNA binding, structural constituent of nuclear pore activity
PIS48732.1	26.5000666	27.0314784	27.1200426	29.05104318	26.97489175	26.43364664	0.314677843	0.966	0.603	PIS48732.1	orf19.2778	Protein of unknown function; transcript is upregulated in clinical isolates from HIV+ patients with oral candidiasis
PIS58378.1	28.2072775	24.7265518	26.1699507	26.75112697	27.19525291	26.96520839	0.503588299	0.972	0.603	PIS58378.1	SPO7	Putative regulatory subunit of Nem1-Spo7 phosphatase holoenzyme that regulates nuclear growth by controling phospholipid biosynthesis; induced by alpha pheromone in SpiderM medium
PIS55709.1	26.253462	26.6382332	25.179515	27.12720877	26.60134711	26.15925559	0.498878362	0.972	0.606	PIS55709.1	GPX3	Putative glutathione peroxidase involved in Cap1p-dependent oxidative stress response, required for Cap1p oxidation in response to H2O2; planktonic growth-induced
PIS54771.1	25.1464587	23.9574023	24.4455064	26.22858568	24.61436699	24.52442711	0.401162948	0.969	0.606	PIS54771.1	orf19.4225.1	Ortholog(s) have oxoglutarate dehydrogenase (succinyl-transferring) activity, structural constituent of ribosome activity and role in 2-oxoglutarate metabolic process, tricarboxylic acid cycle

PIS49569.1	27.8200782	27.4025243	26.0981339	28.32889921	27.82860946	26.99190732	0.195407656	0.958	0.61	PIS49569.1	NHX1	Protein similar to S. cerevisiae Nhx1p, which is an Na+/H+ exchanger required for intracellular sequestration of Na+
PIS50571.1	28.515783	28.2308905	27.5506315	28.74373591	28.61082808	28.7732535	0.074938302	0.938	0.61	PIS50571.1	TIP1	Protein with a predicted role in biogenesis of ER-derived COPII transport vesicles; mutation confers hypersensitivity to toxic ergosterol analog
PIS56869.1	26.7587642	26.0790163	25.385552	27.30882858	26.01507455	26.73477971	0.321545869	0.966	0.612	PIS56869.1	orf19.2697	Ortholog(s) have ubiquitin protein ligase activity, ubiquitin-protein transferase activity
PIS58268.1	29.5960611	29.1265349	28.5777365	29.67593546	29.95236963	29.51233322	0.184180874	0.957	0.613	PIS58268.1	DPM3	Dolichol-phosphate mannose synthase subunit, essential for enzyme activity; flow model biofilm repressed
PIS48492.1	31.8652299	31.8021559	31.3389991	32.83895275	32.21183187	31.79723349	0.368104284	0.968	0.614	PIS48492.1	RPT6	Putative ATPase of the 19S regulatory particle of the 26S proteasome; transcript regulated by Mig1; regulated by Gcn2 and Gcn4
PIS50381.1	30.4744577	29.9280582	29.873464	30.36956111	30.72085479	31.02970213	0.192323381	0.958	0.615	PIS50381.1	APM4	Cargo-binding subunit of the clathrin associated protein complex (AP-2), involved in endocytosis; regulates polarized growth through endocytic recycling of chitin synthase Chs3p
PIS55564.1	29.2286323	28.7362217	27.8330664	30.0261714	28.64328215	28.9759655	0.218516678	0.96	0.616	PIS55564.1	BET2	Putative Type II geranylgeranyltransferase beta subunit; transcript regulated by Mig1

PIS50446.1	24.7248019	25.7898587	24.3721054	25.74572201	26.2312783	24.75750823	0.264260843	0.963	0.616	PIS50446.1	orf19.864	Ortholog(s) have role in DNA recombination, nuclear-transcribed mRNA catabolic process, 3'-5' exonucleolytic nonsense-mediated decay, nuclear-transcribed mRNA catabolic process, nonsense-mediated decay and cytoplasm localization
PIS56557.1	26.521318	26.0176848	26.6908824	27.34237879	26.74561436	26.99737055	0.365699033	0.968	0.618	PIS56557.1	orf19.4295	Component of the HIR complex, a nucleosome assembly factor involved in chromatin formation; involved in regulation of white-opaque switching frequency, hyphal initiation, and azole sensitivity
PIS58643.1	29.6941947	28.5432092	26.5885094	29.73489039	28.56027996	28.38329195	0.406024376	0.969	0.618	PIS58643.1	orf19.5984	Putative protein of unknown function; homozygous transposon insertion causes decreased colony wrinkling under filamentous growth-inducing conditions, but does not block true hyphal formation in liquid media
PIS58668.1	27.9370811	27.2035031	27.6957313	28.76494372	28.21304127	27.71680637	0.162063077	0.955	0.619	PIS58668.1	orf19.6423	Ortholog(s) have fructose-2,6-bisphosphate 2-phosphatase activity and role in glucose metabolic process
PIS48477.1	24.5099932	24.3926134	25.0869854	24.89903037	25.71100492	25.23816211	0.118655599	0.949	0.62	PIS48477.1	orf19.1267.1	Ortholog(s) have cysteine desulfurase activity, role in iron-sulfur cluster assembly and L-cysteine desulfurase complex, mitochondrial matrix localization
PIS58873.1	28.6954479	28.4559633	27.5854471	29.27100328	28.76870226	28.55756646	0.108890767	0.947	0.62	PIS58873.1	orf19.1300	Putative mitochondrial membrane protein; homozygous transposon insertion causes decreased colony wrinkling under filamentous growth-inducing conditions, but does not block true hyphal formation in liquid media
PIS49622.1	28.2410416	28.0474438	27.7195202	28.41285425	28.54834151	28.90546032	0.145009334	0.953	0.62	PIS49622.1	orf19.7194	Protein required for virulence in reconstituted human epithelium (RHE) model of ex vivo infection; decreased transcription is observed upon fluphenazine treatment; induced upon adherence to polystyrene

PIS51982.1	22.9468215	23.580468	24.7155152	23.88256443	24.30489067	24.92167782	0.416827452	0.97	0.622	PIS51982.1	JAB1	Component of the COP9 signalosome (CSN) complex, provides catalytic activity for deneddylation that cleaves the ubiquitin-like protein tag Rub1p off the cullin (Cdc53p) molecule
PIS48288.1	24.065053	23.9594418	23.901541	24.87283271	24.36687936	24.55204049	0.074769852	0.938	0.622	PIS48288.1	orf19.7347	Ortholog(s) have ubiquitin-protein transferase activity, role in protein monoubiquitination, protein polyubiquitination, ubiquitin-dependent ERAD pathway and endoplasmic reticulum membrane localization
PIS52024.1	28.1358202	27.3202669	27.6510965	28.41129655	27.94368057	28.61988033	0.14261421	0.953	0.623	PIS52024.1	orf19.3572	Ortholog of C. dubliniensis CD36 : Cd36_19880, C. auris B8441 : B9J08_003635, Candida tenuis NRRL Y-1498 : CANTEDRAFT_117672 and Debaryomyces hansenii CBS767 : DEHA2D11154g
PIS54792.1	26.2276075	26.0452185	26.5666428	26.99463245	27.44492565	26.26839681	0.221980924	0.961	0.623	PIS54792.1	orf19.7102	Has domain(s) with predicted nucleic acid binding activity
PIS50391.1	23.6258425	22.7836344	18.1213971	23.9205802	20.29813956	22.18516208	0.71481063	0.976	0.624	PIS50391.1	orf19.3826	Predicted transmembrane protein with a role in cell wall polymer composition; Plc1-regulated; Spider biofilm induced
PIS50544.1	28.6494361	28.3298069	27.3563488	28.83458522	28.61510137	28.76334209	0.115966117	0.948	0.626	PIS50544.1	HEM2	Putative porphobilinogen synthase; induced in high iron; protein level decrease in stationary phase cultures; Spider biofilm repressed
PIS49680.1	28.5740598	28.1918838	27.6653177	28.80791883	28.76867343	28.73331446	0.151644273	0.954	0.626	PIS49680.1	RPN8	Putative regulatory subunit of the 26S proteasome; mutation confers hypersensitivity to amphotericin B; regulated by Mig1, Gcn2 and Gcn4; Spider biofilm repressed

PIS55465.1	24.6809136	24.6593591	23.8315438	24.25264628	24.86432187	25.94127408	0.231651627	0.961	0.629	PIS55465.1	CDC34	Putative ubiquitin-protein ligase; transcript regulated by Nrg1 and Tup1, and by Gcn2 and Gcn4; rat catheter biofilm induced
PIS51783.1	27.4336306	23.8285662	27.492122	27.43789861	25.76917187	27.44709598	0.482452559	0.971	0.633	PIS51783.1	NOP4	Putative nucleolar protein; Hap43-induced; mutation confers hypersensitivity to 5-fluorocytosine (5-FC), 5-fluorouracil (5-FU), and tubercidin (7-deazaadenosine); represses in core stress response
PIS55506.1	31.3115301	30.2505848	29.4644751	31.28009466	30.746806	30.90500803	0.210028892	0.96	0.635	PIS55506.1	ADE5,7	Phosphoribosylamine-glycine ligase and phosphoribosylformylglycinamidine cyclo-ligase; interacts with Vps34p; required for hyphal growth and virulence; flucytosine induced; not induced in GCN response, in contrast
PIS54531.1	28.7386689	28.8105669	29.1137563	28.72748155	29.75225302	30.09859663	0.373068727	0.968	0.638	PIS54531.1	orf19.4795	Protein of unknown function; Sef1-, Sfu1-, and Hap43 regulated; Spider biofilm induced
PIS56957.1	28.6915247	29.1251383	28.8632431	29.61363408	29.40820023	29.57143494	0.025635452	0.902	0.638	PIS56957.1	orf19.5370	Ortholog(s) have fungal-type vacuole membrane localization
PIS55668.1	29.8911555	29.2803992	29.0390339	30.53688641	29.55070586	30.03966875	0.131986613	0.951	0.639	PIS55668.1	PRS1	Phosphoribosylpyrophosphate synthetase; enzyme of purine, pyrimidine, histidine, and tryptophan biosynthesis; essential; flucytosine induced; macrophage/pseudohyphal-induced
PIS55085.1	28.8208332	27.9806531	28.0863004	28.78750343	29.09430623	28.9280802	0.064858232	0.934	0.641	PIS55085.1	HRR25	Predicted protein serine/threonine kinase, involved in regulation of response to cell wall and membrane stress; Spider biofilm induced

PIS58621.1	26.0271777	25.4991498	25.620349	26.06489683	25.58979546	27.41571492	0.278140223	0.964	0.641	PIS58621.1	orf19.2228	Ortholog(s) have actin filament binding, actin monomer binding activity
PIS52005.1	24.8288611	24.300306	27.8461904	26.31096641	25.35666234	27.2365615	0.426686807	0.97	0.643	PIS52005.1	OPY2	Predicted transmembrane protein; role in cell wall biogenesis; required for Cek1 phosphorylation; Spider biofilm induced
PIS52319.1	24.5723224	25.2343711	24.1026866	25.43378668	25.6542618	24.75036475	0.125078374	0.95	0.643	PIS52319.1	VPH2	Protein required for for proper vacuolar ATPase assembly and vacuolar functions
PIS55758.1	28.4389334	28.8767238	28.4843632	28.88225729	29.36308139	29.48728116	0.174235322	0.956	0.644	PIS55758.1	COX15	Cytochrome oxidase assembly protein; transcript regulated by Nrg1 and Tup1; alkaline repressed; Hap43-repressed; early-stage flow model biofilm induced; Spider biofilm repressed
PIS58817.1	28.1132029	28.3435551	28.6225486	29.37558997	29.18443938	28.45446775	0.061558175	0.933	0.645	PIS58817.1	orf19.392	Ortholog(s) have role in mitochondrial respiratory chain complex III assembly and mitochondrial membrane, mitochondrion localization
PIS56600.1	30.2074989	30.5230649	30.7155627	31.42433469	30.76374184	31.19747396	0.037643459	0.917	0.646	PIS56600.1	ARP3	Protein with Myo5p-dependent localization to cortical actin patches at hyphal tip; mutation confers hypersensitivity to cytochalasin D; Spider biofilm repressed
PIS58718.1	24.5813719	25.0190763	23.8307598	25.2017942	24.66249284	25.51435367	0.331779524	0.967	0.649	PIS58718.1	orf19.332	Member of a complex that contains Prp19; stabilizes U6 snRNA in catalytic forms of the spliceosome containing U2, U5, and U6 snRNAs; Spider biofilm induced

PIS50399.1	28.0350608	26.8057233	27.3437293	29.33182606	27.85411465	26.94646027	0.291369305	0.965	0.649	PIS50399.1	SSZ1	Putative HSP70 chaperone; protein level decreases in stationary phase cultures; Spider biofilm repressed
PIS51294.1	29.2517828	29.1153187	29.1098149	29.45835209	29.87459263	30.09407043	0.182887604	0.957	0.65	PIS51294.1	MUQ1	Putative choline phosphate cytidylyltransferase/phosphoethanolam ine cytidylyltransferase; repressed in hyphae compared vs yeast; Hap43-repressed; flow model biofilm induced; Spider biofilm repressed
PIS50497.1	23.5822761	27.1893358	26.6714798	27.74815455	26.64171689	25.00726264	0.558554879	0.973	0.651	PIS50497.1	orf19.2445	Putative dicarboxylic amino acid permease; fungal-specific (no human or murine homolog); induced by alpha pheromone in SpiderM medium
PIS51956.1	24.0123734	23.7644601	24.6705529	25.55470546	24.49299872	24.35650861	0.528955777	0.973	0.652	PIS51956.1	MDS3	[illegible] required for growth and hyphal formation at alkaline pH, for full virulence in a mouse model of systemic infection and for SD and Spider medium biofilm formation; role [illegible]
PIS56915.1	30.8185039	30.9380413	30.9811923	32.17689318	31.41575129	31.10715545	0.059977511	0.932	0.654	PIS56915.1	CCT7	Cytosolic chaperonin Cct ring complex; protein is present in exponential and stationary growth phase yeast cultures; sumoylation target
PIS49701.1	27.5236908	27.179961	27.6261632	28.59596819	28.22105443	27.48365505	0.351580398	0.967	0.657	PIS49701.1	orf19.6565	Conserved mitochondrial inner membrane insertase; mediates insertion of mitochondrial- and nuclear-encoded proteins from the matrix into the inner membrane; Spider biofilm repressed
PIS51574.1	30.4322444	29.9372519	29.522445	31.17037083	30.89407978	29.80447626	0.130526139	0.951	0.659	PIS51574.1	UBP6	Putative ubiquitin-specific protease of the 26S proteasome; oxidative stress-induced via Cap1p

PIS51255.1	30.3642446	30.3510386	30.3954977	30.7235673	31.05641364	31.31202163	0.032542577	0.912	0.66	PIS51255.1	AYR2	Putative NADPH-dependent 1-acyl dihydroxyacetone phosphate reductase; shows colony morphology-related gene regulation by Ssn6p
PIS55750.1	23.3687622	23.3553562	23.6759583	24.15447868	24.04722904	24.17859913	0.118741982	0.949	0.66	PIS55750.1	orf19.5247	Has domain(s) with predicted phosphatase activity
PIS52333.1	26.269737	26.1078554	26.1607555	26.73320196	26.59813794	27.19211831	0.090290188	0.943	0.662	PIS52333.1	RVS162	Protein containing a BAR domain, which is found in proteins involved in membrane curvature; null mutant does not display the endocytic, hyphal growth, virulence, or cell wall defects exhibited by mutants in related genes RVS161 and RVS167 [illegible]
PIS48673.1	26.8463918	26.5880994	25.563979	27.49425249	26.55036057	26.95420224	0.188450182	0.958	0.667	PIS48673.1	orf19.3406	Predicted chloride transporter; member of conserved Mcm1 regulon; Spider biofilm repressed
PIS58327.1	24.3499435	24.5819333	24.2985728	24.16267342	25.01302187	26.05465053	0.219051029	0.96	0.667	PIS58327.1	orf19.4907	Putative protein of unknown function; Hap43p-repressed gene; increased transcription is observed upon fluphenazine treatment; possibly transcriptionally regulated by Tac1p; induced by nitric oxide; fungal-specific (no human/murine homolog [illegible]
PIS54916.1	25.4003505	23.5584416	25.4076935	26.65888597	24.65065728	25.06348934	0.410466048	0.969	0.669	PIS54916.1	SGE1	Putative multidrug resistance factor; induced in low iron; regulated by Sef1, Sfu1, and Hap43
PIS56962.1	29.8610017	29.200094	28.6985014	30.4209749	29.72843246	29.62154828	0.097971149	0.945	0.67	PIS56962.1	LMO1	Protein involved together with Dck1p and Rac1p in invasive filamentous growth and cell wall integrity; acts upstream of MAP kinase Cek1p; has similarity to human ELMO1

PIS58434.1	28.7294536	27.6778461	28.1663125	28.9778863	28.69769783	28.90969184	0.07007037	0.936	0.671	PIS58434.1	CDC50	Predicted non-catalytic subunit of phospholipid flippase; involved in endocytosis, hyphal development, drug resistance; mutants show attenuated virulence in mouse model; induced by Mnl1p under weak acid stress
PIS51313.1	22.0579001	23.3523672	22.8650575	23.27423712	24.14955064	22.86823642	0.520290309	0.972	0.672	PIS51313.1	CAT8	Zn(II)2Cys6 transcription factor; similar to S. cerevisiae Cat8 but mutant phenotype suggests different target genes; mutant displays increased filamentous/invasive growth; flucytosine repressed; rat catheter biofilm induced
PIS58780.1	27.080676	26.1696652	26.8370465	27.86949376	27.23850631	26.99871224	0.08812601	0.942	0.673	PIS58780.1	RPL7	Ribosomal protein L7; repressed upon phagocytosis by murine macrophages; Hap43-induced; rat catheter and Spider biofilm induced
PIS56766.1	23.7343987	24.0612722	25.8336384	25.82357432	24.17794269	25.65359211	0.375728328	0.968	0.675	PIS56766.1	orf19.962	Protein with a fungal RNA polymerase I subunit RPA14 domain; proposed to play a role in the recruitment of pol I to the promoter; Hap43-induced gene
PIS54538.1	27.6361417	27.5451084	27.8824255	28.84631241	28.4045302	27.83757895	0.106367074	0.947	0.675	PIS54538.1	UTP8	Essential nucleolar protein; involved in tRNA export from the nucleus and ribosomal small subunit biogenesis; physically interacts with TAP-tagged Nop1; Spider biofilm induced
PIS51677.1	28.9687975	28.7908369	28.7446495	29.45822266	29.71393764	29.35735062	0.047666977	0.925	0.675	PIS51677.1	YML6	Putative mitochondrial ribosomal protein; induced upon adherence to polystyrene
PIS51844.1	26.5004555	26.4208633	23.197827	25.71121266	26.09626712	26.33890792	0.377670673	0.968	0.676	PIS51844.1	orf19.867	Ortholog(s) have superoxide-generating NAD(P)H oxidase activity, role in apoptotic process, regulation of actin cytoskeleton organization and perinuclear endoplasmic reticulum localization

PIS54563.1	24.38016	23.8387725	24.9053327	25.29524848	25.06277524	24.79536805	0.09622456	0.944	0.676	PIS54563.1	SPB4	Putative ATP-dependent RNA helicase; flucytosine repressed; Spider biofilm induced
PIS55608.1	28.1729304	28.4006881	27.7781453	28.16616263	28.83390681	29.38254822	0.081828109	0.94	0.677	PIS55608.1	orf19.5693	Subunit of the GPI (glycosylphosphatidylinositol):protein transamidase complex; removes the GPI-anchor signal and attaches GPI to proteins in the ER; Spider biofilm repressed
PIS50316.1	28.0628199	28.3471918	27.7311537	29.05954058	28.66496661	28.45173578	0.11165059	0.948	0.678	PIS50316.1	HPT11	Putative hypoxanthine-guanine phosphoribosyltransferase; protein abundance affected by URA3 expression in the CAI4 strain background; protein level decreases in stationary phase; Spider biofilm induced
PIS59031.1	27.3572785	27.3996361	25.4833123	27.5728738	27.22626202	27.47494445	0.252534634	0.963	0.678	PIS59031.1	orf19.4523	Ortholog(s) have 5-formyltetrahydrofolate cyclo-ligase activity and role in folic acid-containing compound biosynthetic process
PIS48721.1	29.2147886	27.8203218	26.4660443	28.63424851	28.40330104	28.50003604	0.243276599	0.962	0.679	PIS48721.1	orf19.7041	Putative pre-tRNA processing protein; heterozygous null mutant exhibits hypersensitivity to parnafungin and cordycepin in the C. albicans fitness test
PIS51762.1	29.8788324	29.4987471	28.7674129	30.89909756	29.79895049	29.48596032	0.276156112	0.964	0.68	PIS51762.1	CDC3	Septin; essential for viability; functional homolog of S. cerevisiae Cdc3p; down-regulation associated with azole resistance; macrophage/pseudohyphal-repressed; virulence-group-correlated expression; gene has intron
PIS55839.1	25.7176676	25.1423529	24.2034032	24.70438583	26.95273065	25.4490149	0.324230284	0.966	0.681	PIS55839.1	orf19.1404	Predicted tRNA dihydrouridine synthase; Spider biofilm induced

PIS51918.1	26.7190198	26.1953899	24.5833051	25.5616799	27.32134802	26.66148324	0.322028033	0.966	0.682	PIS51918.1	orf19.4173	Ortholog(s) have role in protein histidyl modification to diphthamide
PIS58440.1	29.6924465	29.7686294	28.8960811	29.96070232	30.27922995	30.16870677	0.05818702	0.931	0.684	PIS58440.1	HGT10	Glycerol permease involved in glycerol uptake; member of the major facilitator superfamily; induced by osmotic stress, at low glucose in rich media, during cell wall regeneration; 12 membrane spans; Hap43p-induced gene
PIS51731.1	27.5450684	28.4782701	27.7011179	28.58047301	28.0558657	29.14444831	0.217293895	0.96	0.685	PIS51731.1	orf19.2041	Component of the RSC chromatin remodeling complex
PIS58099.1	25.2119576	24.5321545	26.7305195	26.78558808	25.36827133	26.37825325	0.440474115	0.97	0.686	PIS58099.1	PFY1	Profilin; functional homolog of S. cerevisiae Pfy1; hyphae, macrophage/pseudohyphal-induced; regulated by Nrg1, Tup1; gene lacks intron (unlike S. cerevisiae PFY1); complements growth of S. cerevisiae srv2 mutant; nonessential
PIS52137.1	22.7250315	23.3385327	24.7324215	23.99960804	24.4793777	24.37886208	0.230762222	0.961	0.687	PIS52137.1	CHR1	Predicted DEAD-box ATP-dependent RNA helicase; functional homolog of S. cerevisiae Rok1; Hap43-induced; Spider biofilm induced
PIS50414.1	23.4596526	24.0642875	23.2667372	24.30689006	23.98695394	24.56085243	0.305148858	0.965	0.688	PIS50414.1	BUD6	Protein required for Spitzenkorper formation in hyphal cells (wild-type localization of Mlc1p to the Spitzenkorper); localizes to polarisome
PIS51218.1	27.0838679	27.1681677	26.937595	28.47343559	27.84538604	26.94065392	0.117546602	0.949	0.69	PIS51218.1	RCK2	Predicted MAP kinase-activated protein kinase, similar to S. cerevisiae serine/threonine protein kinase Rck2p; induced by osmotic stress via Hog1p; macrophage/pseudohyphal-repressed; mutants are sensitive to rapamycin

PIS58347.1	21.9935628	22.7192368	21.6988138	24.63508196	23.96949322	19.88200005	0.546198227	0.973	0.692	PIS58347.1	orf19.1338	Protein of unknown function; possible COPI-coated vesicle, Golgi apparatus, ribosome localization; rat catheter biofilm repressed
PIS58884.1	20.0919576	20.4995638	20.4020962	21.73739389	20.83734806	20.49379098	0.221734451	0.96	0.692	PIS58884.1	SFU1	GATA-type transcription factor; regulator of iron-responsive genes; represses iron utilization genes if iron is present; Hap43-repressed; promotes gastrointestinal commensalism in mice; Spider biofilm induced
PIS58582.1	28.3877311	27.5385517	27.1607146	29.15902523	28.05844218	27.95411352	0.091329236	0.943	0.695	PIS58582.1	PAN3	Ortholog(s) have RNA binding, poly(A) binding activity, role in DNA repair, nuclear-transcribed mRNA poly(A) tail shortening, postreplication repair and PAN complex localization
PIS48681.1	29.4672866	28.459613	28.1226768	30.94823557	28.69779151	28.4960585	0.318253663	0.966	0.698	PIS48681.1	FMP27	Putative mitochondrial protein; mRNA binds She3
PIS52111.1	24.5932053	24.0718892	22.4396746	24.38756733	24.42064992	24.38997009	0.169022019	0.956	0.698	PIS52111.1	LYS22	Homocitrate synthase, minor isoform; repressed by nitric oxide and by hypoxia; protein level decreases in stationary phase cultures; induced by ketoconazole, Spider biofilm induced; flow model biofilm repressed
PIS54793.1	23.4679332	24.2506727	24.5481448	24.2681909	24.17083911	25.92890187	0.169110114	0.956	0.7	PIS54793.1	orf19.7101	Ortholog(s) have telomeric DNA binding activity, role in protein localization to chromosome, telomere maintenance, telomere maintenance via telomerase and chromosome, telomeric region localization
PIS55813.1	30.416968	29.4887119	29.9759956	30.74447292	30.39542102	30.8461696	0.1437506	0.953	0.701	PIS55813.1	orf19.7380	Has domain(s) with predicted nucleic acid binding activity

PIS50349.1	27.4726892	26.8962638	26.8768271	28.30703466	27.4708706	27.57386652	0.278632778	0.964	0.702	PIS50349.1	orf19.1646	Ortholog(s) have rRNA primary transcript binding activity
PIS58622.1	28.8953513	28.8946573	29.3102785	29.35590681	29.33747959	30.51701147	0.514454498	0.972	0.703	PIS58622.1	orf19.2227	Protein of unknown function; flow model biofilm induced; nitric oxide-repressed
PIS55645.1	26.18055	25.8195837	25.6052706	26.71491997	26.96528442	26.03581811	0.13861151	0.952	0.704	PIS55645.1	orf19.6732	Ortholog(s) have thiamine phosphate phosphatase activity and role in phosphate ion transport
PIS58226.1	26.0696546	24.8824074	22.975076	26.32802314	24.27436885	25.44032793	0.365496693	0.968	0.705	PIS58226.1	orf19.6898	Protein similar to S. pombe SPBC1709.16c a predicted aromatic ring-opening dioxygenase; induced by benomyl treatment or in azole-resistant strain that overexpresses MDR1; Spider biofilm induced
PIS48438.1	26.8800115	26.6336171	25.8847499	26.94551538	27.30181773	27.26975943	0.311077736	0.966	0.706	PIS48438.1	APG7	Ortholog(s) have Atg12 activating enzyme activity, Atg8 activating enzyme activity
PIS52196.1	29.160761	28.6066006	28.5906313	29.36263903	29.49331503	29.61907514	0.063576374	0.934	0.706	PIS52196.1	orf19.1355	Putative protein of unknown function; stationary phase enriched protein
PIS48348.1	25.9710693	25.4362087	24.8651357	25.55009236	26.737623	26.11831582	0.485319121	0.972	0.711	PIS48348.1	orf19.1248	Putative RNA polymerase II subunit B44; heterozygous null mutant exhibits resistance to parnafungin in the C. albicans fitness test; Spider biofilm repressed

PIS49538.1	29.2819588	29.6211184	30.0835487	29.62520424	30.65209183	30.8471399	0.055996263	0.93	0.713	PIS49538.1	GLN1	Putative glutamate synthase; regulated by Tsa1, Tsa1B under H2O2 stress conditions; Spider biofilm induced
PIS51563.1	26.5436415	27.4488957	28.0674905	27.87390042	27.82794234	28.49986782	0.305152859	0.965	0.714	PIS51563.1	LHP1	Ortholog(s) have RNA binding, RNA folding chaperone, tRNA binding activity, role in tRNA 3'-end processing, tRNA 5'-leader removal, tRNA folding, tRNA processing and cytoplasm, nucleolus, nucleoplasm, nucleus localization
PIS58855.1	22.5094684	26.108992	26.0600569	25.08965531	26.48974435	25.24776829	0.4663454	0.971	0.716	PIS58855.1	RRP42	Putative exosome non-catalytic core component; involved in 3'-5' RNA processing; rat catheter biofilm induced
PIS58740.1	23.8445279	25.9134662	25.9968448	25.57084451	26.1859833	26.14988555	0.443247178	0.97	0.717	PIS58740.1	ABC1	Putative ubiquinol-cytochrome-c reductase; induced upon adherence to polystyrene; flow model biofilm induced; Spider biofilm induced
PIS51369.1	27.9200461	28.8847622	28.2598251	30.10133995	28.53945755	28.57576084	0.148392787	0.953	0.717	PIS51369.1	MDM10	Predicted component of the mitochondrial sorting and assembly machinery (SAM complex) and ER-mitochondria encounter structure (ERMES) complex with a role in protein import into mitochondria
PIS50382.1	26.0616195	25.1263713	25.3628991	26.54048302	25.44829488	26.71454301	0.33634745	0.967	0.717	PIS50382.1	PRS5	Putative 5-phospho-ribosyl-1(alpha)-pyrophosphate synthetase; Hap43-induced; repressed during core stress response
PIS56566.1	32.7762932	32.2605151	31.9336346	33.14785738	32.82814183	33.15019574	0.029313248	0.908	0.719	PIS56566.1	ECM4	Cytoplasmic glutathione S-transferase; regulated by Nrg1, Tup1; induced in core stress response, in cyr1 or ras1 mutant (yeast or hyphal cells); Tn mutation affects filamentous growth; stationary phase enriched; Spider biofilm induced

PIS58047.1	25.3853994	24.8256152	24.6316483	26.1706794	25.84276007	24.99408041	0.15184047	0.954	0.722	PIS58047.1	SPT3	Functional homolog of S. cerevisiae Spt3p; required for virulence in mouse systemic infection; homozygous null mutant is hyperfilamentous
PIS56882.1	30.0211639	29.4176357	29.3336581	30.44907865	30.38709524	30.11245476	0.070568851	0.937	0.725	PIS56882.1	orf19.5628	Mitochondrial dicarboxylate transporter; possibly an essential gene, disruptants not obtained by UAU1 method
PIS54850.1	23.8731954	24.7696682	23.9607126	25.39223509	23.68738934	25.70268653	0.374905302	0.968	0.726	PIS54850.1	KTR4	Mannosyltransferase; induced during cell wall regeneration; fungal-specific (no human or murine homolog); Bcr1-repressed in RPMI a/a biofilms
PIS54979.1	26.8850913	26.1878633	26.3483942	26.88727194	27.64391437	27.07118385	0.071226353	0.937	0.727	PIS54979.1	orf19.4278	Ortholog(s) have chromatin binding, methylated histone binding activity and role in negative regulation of transcription by RNA polymerase II
PIS51652.1	34.7091836	34.1681534	35.2825802	34.84812762	35.73210737	35.76435798	0.306466827	0.965	0.728	PIS51652.1	RPL13	Putative ribosomal subunit; antigenic during murine infection; downregulated upon phagocytosis by murine macrophage; Spider biofilm repressed
PIS58808.1	31.1635411	30.979779	30.6066412	31.21075224	32.04480161	31.68073651	0.027547594	0.905	0.729	PIS58808.1	ERG1	Squalene epoxidase, epoxidation of squalene to 2,3(S)-oxidosqualene; ergosterol biosynthesis; allylamine antifungal drug target; NADH reducing cofactor but S. cerevisiae Erg1 uses NADPH; flow model biofilm induced; Spider biofilm repressed
PIS58849.1	24.545694	24.4054701	24.8112485	24.27343641	26.01389306	25.6633532	0.137629302	0.952	0.729	PIS58849.1	orf19.1595	Ortholog(s) have clathrin binding activity, role in SREBP signaling pathway, clathrin-dependent endocytosis and Dsc E3 ubiquitin ligase complex, clathrin-coated vesicle localization

PIS49678.1	19.6042749	21.83888	21.729785	21.87317577	21.47316788	22.012536	0.24099291	0.962	0.729	PIS49678.1	orf19.3170	Ortholog(s) have mRNA binding activity
PIS51471.1	22.8230143	23.8068671	23.7659714	24.60028427	24.18485867	23.7965364	0.156628118	0.954	0.729	PIS51471.1	orf19.7237	Ortholog(s) have arylformamidase activity and role in NAD biosynthetic process
PIS49755.1	22.5309812	25.0442683	20.7083849	23.52532655	24.48244108	22.46139779	0.565137084	0.973	0.729	PIS49755.1	orf19.7497	Ortholog(s) have ubiquitin-protein transferase activity, role in ubiquitin-dependent protein catabolic process and Cul3-RING ubiquitin ligase complex localization
PIS58435.1	26.0989428	26.4192745	25.8078193	26.54986345	26.75016384	27.2172516	0.180213584	0.957	0.73	PIS58435.1	POP2	Component of the Ccr4-Pop2 mRNA deadenylase; heterozygous null mutant exhibits resistance to parnafungin and cordycepin in the C. albicans fitness test
PIS56628.1	24.7410084	24.9850482	24.5649974	25.90863743	25.38415924	25.19271734	0.612547387	0.974	0.731	PIS56628.1	orf19.6866	Putative U1-70K component of the U1 snRNP, involved in splicing; ortholog of S. cerevisiae SNP1; downregulated upon adherence to polystyrene
PIS48675.1	26.2753349	27.5058499	26.2586035	27.33188814	27.18564448	27.71644737	0.061671365	0.933	0.731	PIS48675.1	SEC12	Putative guanyl-nucleotide exchange factor; induced in high iron; Hap43-repressed
PIS51760.1	23.3982254	25.0493365	25.936853	25.24470521	25.77218108	25.56724386	0.375118489	0.968	0.733	PIS51760.1	orf19.1058	Subunit of the 19S regulatory base of the proteasome

PIS49646.1	26.8575519	27.9813792	28.4335493	28.22551024	28.54374396	28.70372303	0.095352346	0.944	0.733	PIS49646.1	orf19.4294	Ortholog(s) have oxidoreductase activity, role in cytochrome c-heme linkage, mitochondrial membrane organization and mitochondrial inner membrane localization
PIS52129.1	22.7324392	23.1567603	23.0247514	24.41282892	23.38273191	23.31881916	0.392476458	0.969	0.733	PIS52129.1	RAD1	Putative single-stranded DNA endonuclease; transcript regulated by Nrg1; macrophage-induced gene
PIS54887.1	28.5079742	28.623101	29.1364269	29.37486195	29.55165143	29.5439187	0.028659967	0.907	0.734	PIS54887.1	AKR1	Ankyrin-repeat protein; induced by fluphenazine
PIS51966.1	23.8939646	25.6789411	24.79402	26.27795018	25.28356931	25.01466323	0.283158204	0.964	0.736	PIS51966.1	orf19.5346	Ortholog(s) have role in transcription by RNA polymerase II and transcription factor TFIID complex localization
PIS48345.1	28.8478091	28.1410736	27.9146712	29.36890146	28.85708493	28.88910123	0.042109651	0.921	0.737	PIS48345.1	GYP2	Ortholog(s) have GTPase activator activity, role in activation of GTPase activity and cytoplasm, mating projection tip localization
PIS51472.1	29.3812132	28.9253997	29.7804892	30.01359927	29.81004115	30.47365743	0.027589881	0.905	0.737	PIS51472.1	TIF35	Putative translation initiation factor; repressed upon phagocytosis by murine macrophage; Spider biofilm repressed
PIS54909.1	27.5825623	27.6468034	28.2861485	28.60330721	28.69603353	28.43104868	0.35928285	0.968	0.738	PIS54909.1	orf19.1933	Ortholog(s) have role in ER-dependent peroxisome organization, peroxisome organization and endoplasmic reticulum, peroxisomal membrane, peroxisome localization

PIS58523.1	21.9111178	21.8628387	22.4835056	21.38694012	22.49525115	24.59334429	0.569925207	0.973	0.739	PIS58523.1	orf19.3275	Ortholog of C. dubliniensis CD36 : Cd36_25870, C. parapsilosis CDC317 : CPAR2_804000, C. auris B8441 : B9J08_001023 and Candida tenuis NRRL Y-1498 : CANTEDRAFT_115661
PIS58196.1	28.9864876	29.8140735	31.0031028	29.79634775	31.07650493	31.15173286	0.206429658	0.959	0.74	PIS58196.1	ARG1	Argininosuccinate synthase; arginine synthesis; Gcn4, Rim101 regulated; induced by amino acid starvation (3-AT), benomyl treatment; stationary phase enriched protein; repressed in alkalinizing medium; rat catheter, Spider biofilm induced
PIS51124.1	28.240919	28.9024168	28.1457629	30.16073234	28.58288703	28.76680042	0.145995565	0.953	0.74	PIS51124.1	orf19.641	Ortholog(s) have role in protein folding and endoplasmic reticulum localization
PIS52189.1	29.3920805	28.1141041	28.8743998	29.97535337	29.20108996	29.43263668	0.186926456	0.958	0.743	PIS52189.1	orf19.1772	Ortholog of S. cerevisiae : MRX1, C. glabrata CBS138 : CAGL0J03278g, C. dubliniensis CD36 : Cd36_24130, C. parapsilosis CDC317 : CPAR2_407430 and C. auris B8441 : B9J08_003802
PIS51759.1	26.9308052	25.9817854	25.5717546	24.87001598	28.04976043	27.79611655	0.473379983	0.971	0.744	PIS51759.1	orf19.3840	Serine/threonine protein kinase, acts as an upstream activating factor for the SNF1 complex that regulates responses to nutrient stress; Spider biofilm induced
PIS59019.1	27.4851361	26.9356842	26.541522	29.02783558	27.53468499	26.63708506	0.313202382	0.966	0.746	PIS59019.1	GCV3	Glycine decarboxylase, subunit H; protein level decrease in stationary phase cultures
PIS51391.1	25.483699	25.6307434	25.4451527	25.60829894	26.35861936	26.83157247	0.089614864	0.943	0.746	PIS51391.1	orf19.3292	Ortholog(s) have peptide-methionine (R)-S-oxide reductase activity, role in cellular response to oxidative stress and mitochondrion localization

PIS49798.1	31.3466596	31.0312457	31.0635817	31.82750462	31.78565617	32.07005849	0.013949464	0.872	0.747	PIS49798.1	orf19.1395	Ortholog(s) have copper ion transmembrane transporter activity, inorganic phosphate transmembrane transporter activity
PIS48401.1	28.5469626	29.1091969	28.4102946	29.88366734	29.28277904	29.14367925	0.023969198	0.899	0.748	PIS48401.1	orf19.6552	Flavin-linked sulfhydryl oxidase; predicted localization to endoplasmic reticulum lumen; involved in disulfide bond formation within the ER; Spider biofilm induced
PIS52436.1	24.7326367	23.8953369	24.4050526	25.25267633	25.36030199	24.67072173	0.097513002	0.945	0.75	PIS52436.1	orf19.4843	Putative iron/copper reductas; involved in iron homeostasis; rat catheter and Spider biofilm induced
PIS58465.1	32.0285645	31.6509232	31.0829724	32.17167076	32.53180014	32.32623565	0.035730742	0.915	0.756	PIS58465.1	GSC1	Essential beta-1,3-glucan synthase subunit; gsc1 allele determines resistance/sensitivity to echinocandins; 16 predicted membrane-spanning regions; mRNA abundance declines after yeast-to-hypha transition; Spider biofilm induced
PIS49765.1	22.17021	24.5953308	24.5974806	24.72437866	23.00837885	25.89850835	0.338572531	0.967	0.756	PIS49765.1	orf19.3607	Ortholog of C. dubliniensis CD36 : Cd36_22860, C. parapsilosis CDC317 : CPAR2_806540, C. auris B8441 : B9J08_004792 and Candida tenuis NRRL Y-1498 : CANTEDRAFT_108823
PIS51045.1	26.1362525	26.01364	25.8685239	28.64303637	27.53107503	24.11975241	0.451222957	0.971	0.758	PIS51045.1	BMS1	Putative GTPase; Hap43-induced gene; mutation confers resistance to 5-fluorocytosine (5-FC); flucytosine induced; repressed by prostaglandins; Spider biofilm induced
PIS52203.1	27.8560169	26.8611712	26.9117894	28.28985295	28.30673273	27.30545737	0.241565728	0.962	0.758	PIS52203.1	orf19.6933	Ortholog(s) have peptidyl-prolyl cis-trans isomerase activity, protein phosphatase regulator activity, role in mitotic spindle organization, response to osmotic stress and protein phosphatase type 2A complex localization

PIS54525.1	28.8845836	28.4939241	28.3238199	29.103111	29.59603904	29.28355708	0.130771006	0.951	0.76	PIS54525.1	orf19.6230	Ortholog(s) have 5'-hydroxyl dinucleotide hydrolase activity, GDP binding, RNA NAD-cap (NAD-forming) hydrolase activity, enzyme regulator activity, mRNA 5'-diphosphatase activity, phosphodiesterase decapping endonuclease activity
PIS49506.1	28.7501159	27.6798166	28.1698667	28.79656116	28.76840077	29.32831865	0.104251068	0.946	0.764	PIS49506.1	orf19.5682	Ortholog(s) have nuclear import signal receptor activity, nuclear localization sequence binding, protein-containing complex binding activity
PIS48766.1	27.876235	24.399763	25.0360485	26.06974095	25.73865014	27.79489565	0.341961895	0.967	0.764	PIS48766.1	orf19.6918	Mitochondrial protein required for expression of respiratory chain complex III (coenzyme Q:cytochrome c oxidoreductase); Hap43-induced gene; Spider biofilm induced
PIS48807.1	23.8778028	22.8376531	25.0034368	23.60626128	25.35885758	25.04957708	0.193608081	0.958	0.765	PIS48807.1	ATF1	Putative alcohol acetyltransferase; caspofungin repressed; expression depends on Tac1p
PIS52331.1	28.414647	27.9311756	28.0430631	28.75942771	29.08195508	28.84267967	0.021287862	0.894	0.765	PIS52331.1	orf19.6346	Putative nuclear export protein; Hap43p-induced gene; decreased transcription is observed in an azole-resistant strain that overexpresses MDR1
PIS48790.1	26.9018672	26.1326516	25.5382458	26.74564544	27.05179136	27.07393467	0.052749349	0.928	0.766	PIS48790.1	FPG1	Formamidopyrimidine DNA glycosylase, involved in repair of gamma-irradiated DNA; Hap43p-repressed gene
PIS54766.1	25.1804147	25.4103003	24.4766608	25.30994313	25.85220776	26.20525505	0.149887206	0.954	0.767	PIS54766.1	orf19.4358	Putative protein of unknown function; Hap43p-repressed gene; S. cerevisiae ortholog YDL157C localizes to mitochondria

PIS49461.1	28.2675331	26.4934284	28.1103749	28.50188807	27.71046151	28.96819823	0.137337156	0.952	0.77	PIS49461.1	orf19.827	Ortholog of C. dubliniensis CD36 : Cd36_18620, C. parapsilosis CDC317 : CPAR2_212480, C. auris B8441 : B9J08_004484 and Candida tenuis NRRL Y-1498 : CANTEDRAFT_115371
PIS55017.1	29.1907254	29.228692	28.3988044	29.89048361	29.53777869	29.70619864	0.17712938	0.957	0.772	PIS55017.1	orf19.1185	Ortholog(s) have ubiquitin protein ligase activity, ubiquitin-protein transferase activity
PIS48436.1	27.3607488	27.1282468	26.405093	28.40207061	27.4491958	27.3596073	0.247062548	0.962	0.772	PIS48436.1	PUP2	Alpha5 subunit of the 20S proteasome; macrophage/pseudohyphal-repressed; regulated by Gcn2p and Gcn4p; protein present in exponential and stationary growth phase yeast cultures
PIS48461.1	26.6848696	26.8716376	26.4178891	27.28407903	27.42450235	27.58407485	0.25810229	0.963	0.773	PIS48461.1	orf19.1364	Ortholog of S. pombe Stm1 G-protein coupled receptor; PQ-loop domains; constitutive expression independent of MTL or white-opaque status; Hap43-repressed
PIS55733.1	30.4304963	30.2555158	29.8532883	29.43249762	31.77241047	31.65762201	0.566037573	0.973	0.774	PIS55733.1	orf19.478	Protein required for fusion of cvt-vesicles and autophagosomes with the vacuole; plays a role in autophagy, protein targeting to vacuole and vesicle docking; flow model biofilm induced
PIS58827.1	23.6259844	22.9768414	24.109207	24.34522455	24.94225417	23.74509018	0.241987241	0.962	0.774	PIS58827.1	UME1	Ortholog of C. dubliniensis CD36 : Cd36_73630, C. parapsilosis CDC317 : CPAR2_805330, C. auris B8441 : B9J08_000281 and Candida tenuis NRRL Y-1498 : CANTEDRAFT_119330
PIS54637.1	30.0385715	29.1907433	28.5313106	30.12801736	30.11846178	29.84408442	0.052533755	0.928	0.777	PIS54637.1	DNM1	Putative dynamin-related GTPase involved in mitochondrial fission; transcript upregulated inbRHE model of oral candidiasis; transcript regulated by Nrg1, Mig1, and Tup1

PIS56752.1	23.8394315	24.1954806	24.2069206	25.12510999	25.03790414	24.41393233	0.05829391	0.931	0.778	PIS56752.1	SOD4	Cu-containing superoxide dismutase; role in response to host innate immune ROS; regulated on white-opaque switch; induced under iron starvation; ciclopirox olamine induced; caspofungin repressed; SOD1,4,5,6 gene family
PIS51361.1	25.3742663	24.12887	25.568708	26.12758544	25.29900861	25.98135383	0.302941895	0.965	0.779	PIS51361.1	orf19.4347	Putative serine/threonine protein kinase; Hog1p-induced
PIS54709.1	26.9015395	26.757031	24.8906915	26.73545416	27.23855185	26.91495431	0.314394382	0.966	0.78	PIS54709.1	PEX2	Ortholog(s) have ubiquitin protein ligase activity
PIS48735.1	24.5715167	23.6900578	22.7127364	24.35257086	24.27360742	24.68874053	0.409746127	0.969	0.78	PIS48735.1	RBT4	Pry family protein; required for virulence in mouse systemic/rabbit corneal infections; not filamentation; mRNA binds She3, is localized to hyphal tips; Hap43-induced; in both yeast and hyphal culture supernatants; Spider biofilm induced
PIS56879.1	24.2403629	25.5757867	24.8146725	25.92383672	26.46681785	24.58620926	0.137046603	0.952	0.782	PIS56879.1	orf19.2682	Ortholog(s) have TBP-class protein binding, transcription coregulator activity and role in RNA polymerase II preinitiation complex assembly, transcription initiation at RNA polymerase II promoter
PIS58216.1	25.252029	24.9487908	25.6360343	25.14944463	26.48734922	26.55277059	0.064396445	0.934	0.784	PIS58216.1	VID21	Subunit of the NuA4 histone acetyltransferase complex; soluble protein in hyphae; Spider biofilm repressed
PIS54500.1	27.1865379	27.3245741	24.7719138	27.76404505	26.99765457	26.87944223	0.193554325	0.958	0.786	PIS54500.1	orf19.1308	Predicted membrane transporter, member of the drug:proton antiporter (14 spanner) (DHA2) family, major facilitator superfamily (MFS)

PIS54570.1	29.3559191	30.6672002	31.205206	31.98864199	30.60440761	30.9925541	0.234805628	0.961	0.786	PIS54570.1	orf19.323	Aminophospholipid translocase (flippase), involved in phospholipid and sphingolipid translocation; type 4 P-type ATPase, localized to plasma membrane
PIS56605.1	29.0020543	29.1386795	28.5403958	29.75656111	29.66302532	29.62167277	0.060840231	0.932	0.787	PIS56605.1	LPT1	Lysophospholipid acyltransferase, involved in phospholipid remodeling; role in glycerophospholipid biosynthesis; rat catheter biofilm repressed
PIS48791.1	29.8869223	28.8840732	28.7565141	29.72980349	30.12329888	30.03764421	0.086321757	0.942	0.788	PIS48791.1	MAE1	Malic enzyme, mitochondrial; transcription regulated by Mig1, Tup1; colony morphology-related gene regulation by Ssn6; Hap43-repressed; Spider biofilm repressed
PIS58180.1	22.7324392	22.8773764	25.5071714	24.3889778	24.77591619	24.32021677	0.240607619	0.962	0.789	PIS58180.1	HAM1	Putative deoxyribonucleoside triphosphate pyrophosphohydrolase; caspofungin repressed; regulated by Gcn2p and Gcn4p
PIS51147.1	26.1509925	25.243199	25.4055002	26.60354467	26.20665774	26.35838725	0.104031421	0.946	0.79	PIS51147.1	orf19.3787	Ortholog(s) have chromatin DNA binding, nucleotidase activity
PIS56790.1	27.8024995	27.6344203	27.1409859	28.65543152	28.27409479	28.01982345	0.095153587	0.944	0.79	PIS56790.1	orf19.445	Protein of unknown function; repressed by prostaglandins
PIS52208.1	24.5971558	25.8942572	25.2823702	25.47875582	26.89307309	25.77292307	0.459354763	0.971	0.79	PIS52208.1	SOF1	Putative protein with a predicted role in 40S ribosomal subunit biogenesis; rat catheter biofilm induced

PIS54644.1	24.838143	26.2184853	22.9744332	25.57550651	26.17345644	24.65262018	0.447468498	0.971	0.79	PIS54644.1	STB3	Putative SIN3-binding protein 3 homolog; caspofungin induced; macrophage/pseudohyphal-repressed; rat catheter biofilm induced
PIS51064.1	28.3718882	27.85031	28.5908042	28.60602388	29.11470242	29.46380866	0.090030208	0.943	0.791	PIS51064.1	GPA2	G-protein alpha subunit; regulates filamentous growth, copper resistance; involved in cAMP-mediated glucose signaling; reports differ on role in cAMP-PKA pathway, MAP kinase cascade; Gpr1 C terminus binds Gpa2; regulates HWP1 and ECE1
PIS51943.1	25.780304	26.6731961	26.6651221	27.90170804	26.93195619	26.65847768	0.096817053	0.944	0.791	PIS51943.1	MET10	Sulfite reductase; role in sulfur amino acid metabolism; induced by human whole blood or PMNs; Hog1-induced; possibly adherence-induced; flow model, Spider model, F-12/CO2 biofilm induced
PIS58507.1	24.377087	24.3556707	24.678219	25.5412686	24.92316071	25.32377682	0.1120338	0.948	0.792	PIS58507.1	orf19.2594	Ortholog(s) have RNA polymerase I activity, role in nucleolar large rRNA transcription by RNA polymerase I, transcription by RNA polymerase I, transcription initiation at RNA polymerase I promoter and RNA polymerase I complex localization
PIS48715.1	25.0005229	24.18091	25.4781613	26.31230016	25.59037857	25.13337896	0.361000059	0.968	0.792	PIS48715.1	orf19.7036	Ortholog(s) have protein phosphatase binding activity
PIS49767.1	27.6978837	27.0660715	24.5199638	27.03045075	26.89972681	27.73248415	0.374778591	0.968	0.793	PIS49767.1	orf19.1359	Ortholog(s) have protein-containing complex binding activity, role in sporulation resulting in formation of a cellular spore, ubiquitin-dependent ERAD pathway and nucleus localization
PIS51063.1	31.9332117	30.9680992	30.5602971	32.12912369	31.68362517	32.027717	0.430631074	0.97	0.793	PIS51063.1	orf19.1619	Putative kinase subunit of RNA polymerase II carboxy-terminal domain kinase I; possibly an essential gene, disruptants not obtained by UAU1 method

PIS58530.1	28.9596968	27.9147176	24.8176963	27.89728939	28.16809401	28.00645521	0.310873787	0.966	0.793	PIS58530.1	orf19.6453	Ortholog of C. dubliniensis CD36 : Cd36_72300, C. parapsilosis CDC317 : CPAR2_703060, C. auris B8441 : B9J08_001030 and Candida tenuis NRRL Y-1498 : CANTEDRAFT_92002
PIS50342.1	29.66817	28.6293332	28.1338956	29.81232998	29.4438722	29.55500439	0.091154826	0.943	0.793	PIS50342.1	SSH1	Protein with a role in protein translocation across membranes
PIS48818.1	29.2057223	29.1507427	28.7191882	29.78179078	29.53483108	30.15156743	0.022253159	0.896	0.798	PIS48818.1	NUP188	Putative nuclear pore complex subunit; transcript regulated by Nrg1 and Mig1
PIS51328.1	31.7471526	31.126429	30.8280336	32.55941887	31.86959362	31.67317331	0.037798683	0.917	0.8	PIS51328.1	orf19.6828.1	Ortholog(s) have protein transmembrane transporter activity, protein-transporting ATPase activity, structural molecule activity
PIS56813.1	27.5470566	27.1207064	27.986675	28.48730889	27.94756678	28.62520724	0.134131531	0.951	0.802	PIS56813.1	orf19.1833	Ortholog(s) have pseudouridine synthase activity, role in box H/ACA RNA 3'-end processing, mRNA pseudouridine synthesis, rRNA processing, rRNA pseudouridine synthesis, snRNA pseudouridine synthesis and box H/ACA snoRNP
PIS52124.1	22.6732703	23.4707986	25.4892563	24.8702818	24.84445492	24.33551531	0.641713387	0.975	0.806	PIS52124.1	CAK1	Monomeric CDK-activating kinase; functional homolog of S. cerevisiae Cak1p; phosphorylates cyclin-free human CDK2; lacks glycine loop motif; conserved lysine (K36) not required for activity; possibly essential gene (by UAU1 method)
PIS55593.1	26.6942934	27.4971086	27.6662177	27.55417716	28.01106912	28.71037186	0.080019383	0.94	0.806	PIS55593.1	PTC4	Type PP2C serine/threonine phosphatase; localized to mitochondria; mutation causes sensitivity to sodium, potassium and azole drugs; decreased expression in hyphae compared to yeast-form cells

PIS49640.1	24.5212122	24.1548541	24.2955456	25.07533275	25.03007204	25.2892583	0.044331591	0.923	0.808	PIS49640.1	orf19.71	Ortholog(s) have protein-containing complex binding activity, role in endoplasmic reticulum to Golgi vesicle-mediated transport, protein secretion and Golgi membrane, cis-Golgi network, cytoplasm localization
PIS54835.1	25.2517074	24.327195	24.6473188	25.49410686	25.35837285	25.79887274	0.070488349	0.937	0.808	PIS54835.1	RPB8	Putative subunit of RNA polymerases I, II, and III; regulated by Gcn4p; repressed in response to amino acid starvation (3-aminotriazole treatment); heterozygous null mutant exhibits resistance to parnafungin
PIS55556.1	24.5326229	24.623138	24.8512827	25.93386452	25.80818191	24.69193579	0.126478334	0.95	0.809	PIS55556.1	AXL1	Putative endoprotease; induced by alpha factor; transcript is upregulated in an RHE model of oral candidiasis and in clinical isolates from HIV+ patients with oral candidiasis
PIS49741.1	27.8144051	27.5386348	27.3549462	28.6170886	28.76789251	27.75089601	0.044691289	0.923	0.809	PIS49741.1	ECM29	Putative scaffold protein; assists in association of the proteasome core particle with the regulatory particle; ortholog of S. cerevisiae Ecm29; transposon mutation affects filamentous growth; flow model biofilm repressed
PIS55083.1	28.5720865	28.4231566	28.1663411	29.19287848	29.27277345	29.12364308	0.221609101	0.96	0.809	PIS55083.1	orf19.73	Putative metalloprotease; associates with ribosomes and is involved in ribosome biogenesis; Spider biofilm induced
PIS52363.1	27.100956	26.2734261	24.9654854	27.33617685	26.30522929	27.12792657	0.233735024	0.961	0.81	PIS52363.1	SDH4	Succinate dehydrogenase, membrane subunit; induced in high iron
PIS56612.1	27.0203699	26.9208422	26.1618437	28.47685376	27.64233382	26.41430098	0.276743702	0.964	0.81	PIS56612.1	SWR1	Component of the SWR1 complex, which has a role in exchanging histone variant H2AZ for histone H2A in chromatin; required for proper nucleosome positioning on WOR1 promoter

PIS50552.1	29.5865224	29.2990347	28.017438	30.16914697	29.32463973	29.85078907	0.110810289	0.947	0.814	PIS50552.1	GUT1	Putative glycerol kinase; downregulated upon adherence to polystyrene; greater mRNA abundance observed in a cyr1 homozygous null mutant than in wild type
PIS51393.1	23.808905	23.424515	22.2076048	24.41539872	23.98761439	23.4790886	0.206263093	0.959	0.814	PIS51393.1	orf19.2397	Predicted membrane transporter, involved in biotin import; member of the anion:cation symporter (ACS) family, major facilitator superfamily (MFS); biotin-dependent transcription regulated by Vhr1p; amphotericin B, caspofungin repressed
PIS58547.1	25.3363625	25.6371832	25.4382541	26.65165285	25.8969178	26.30664555	0.138745044	0.952	0.814	PIS58547.1	RPB7	Functional homolog of S. cerevisiae Rpb7; essential subunit of RNA Polymerase II; enhances hyperfilamentation of an S. cerevisiae rpb4 mutant; suppresses defects of S. cerevisiae rpb4 and ess1 mutants; Spider biofilm repressed
PIS50563.1	30.6824359	30.0177744	30.9964471	31.48914247	31.05350211	31.59576548	0.04119014	0.92	0.814	PIS50563.1	RPL43A	Putative ribosomal protein, large subunit; repressed by human whole blood or PMNs; colony morphology-related gene regulation by Ssn6; Spider biofilm repressed
PIS48550.1	28.4378822	28.0258625	27.4415151	28.98346204	28.19070248	29.17707597	0.048668622	0.926	0.815	PIS48550.1	APM1	Ortholog of S. cerevisiae/S. pombe Apm1; a clathrin-associated protein complex (AP-1) subunit; phosphorylated protein; Tn mutation affects filamentous growth; Spider biofilm repressed
PIS48263.1	25.7172683	25.9356636	24.2081712	24.68045061	27.00783239	26.61893937	0.27373777	0.964	0.815	PIS48263.1	VPS27	Putative ESCRT-0 complex protein with a role in multivesicular body (MVB) trafficking
PIS50577.1	25.5073174	25.1533034	25.557012	28.1410673	25.13429422	25.38885982	0.303033416	0.965	0.816	PIS50577.1	orf19.4253	Ortholog(s) have role in negative regulation of gluconeogenesis, proteasome-mediated ubiquitin-dependent protein catabolic process, regulation of nitrogen utilization and GID complex localization

PIS51596.1	26.0522726	25.5284925	24.0670166	25.99025184	26.240338	25.86713886	0.130107053	0.951	0.817	PIS51596.1	orf19.28	Putative thiamine transmembrane transporter; Spider biofilm induced
PIS58215.1	31.8632903	31.0772931	30.7070698	32.49745994	31.66119036	31.94320771	0.042691057	0.922	0.818	PIS58215.1	RLI1	Member of RNase L inhibitor (RLI) subfamily of ABC family; predicted not to be a transporter; regulated by Sef1p, Sfu1p, and Hap43p
PIS50420.1	29.7704629	28.995369	29.0062774	30.1730532	30.09243142	29.96358668	0.068871834	0.936	0.819	PIS50420.1	LAG1	Putative ceramide synthase component; Hap43p-repressed gene; amphotericin B repressed
PIS55618.1	25.9104767	25.1672168	25.0431079	26.17745368	26.13386125	26.26718837	0.213249968	0.96	0.819	PIS55618.1	orf19.5249	Ortholog(s) have role in autophagosome assembly, macroautophagy and Atg1/ULK1 kinase complex, phagophore assembly site localization
PIS52099.1	27.5388406	27.0863966	26.4611246	28.0571285	28.00960914	27.48039191	0.03496249	0.914	0.82	PIS52099.1	orf19.438	Has domain(s) with predicted membrane localization
PIS48665.1	27.4253484	27.4651686	26.651077	28.17582866	28.01533424	27.81105178	0.141851058	0.953	0.82	PIS48665.1	orf19.504	Putative nuclear actin-related protein, component of INO80 complex, involved in chromatin remodeling
PIS51264.1	24.3373763	24.6864099	24.0395204	25.3379287	24.90953601	25.27485347	0.046011876	0.924	0.82	PIS51264.1	RCH1	Plasma membrane protein; involved in regulation of cytosolic calcium homeostasis; null mutation confers sensitivity to calcium and resistance to azoles and terbinafine; rat catheter biofilm induced

PIS49834.1	27.7680225	27.6756417	28.0769297	28.87791492	28.26082005	28.8519637	0.036025185	0.916	0.823	PIS49834.1	CTF1	Putative zinc-finger transcription factor, similar to A. nidulans FarA and FarB; activates genes required for fatty acid degradation; induced by oleate; null mutant displays carbon source utilization defects and slightly reduced virulence
PIS49597.1	25.1765845	24.341352	24.1972834	25.70723272	25.34127726	25.13980582	0.281449715	0.964	0.824	PIS49597.1	orf19.7285	Ortholog(s) have role in co-transcriptional mRNA 3'-end processing, cleavage and polyadenylation pathway, mRNA processing, sno(s)RNA 3'-end processing, termination of RNA polymerase II transcription, exosome- [illegible]
PIS54654.1	28.9764833	28.8280464	29.5423635	29.61622677	30.19412851	30.01159359	0.022576635	0.896	0.825	PIS54654.1	DFG10	Predicted polyprenol reductase with role in dolichol biosynthesis and dolichol-linked oligosaccharide biosynthetic process
PIS55021.1	25.4734332	26.2621619	25.8106641	26.68422931	26.69159015	26.64575393	0.189011869	0.958	0.825	PIS55021.1	HPA2	Ortholog(s) have D-amino-acid N-acetyltransferase activity, N-acetyltransferase activity and role in D-amino acid metabolic process, cellular detoxification of nitrogen compound, protein acetylation
PIS51041.1	27.6729279	27.9334621	27.6571089	29.21601345	28.24131708	28.28345764	0.066117615	0.935	0.826	PIS51041.1	KIN2	Protein with similarity to S. cerevisiae Kin2p, transcription is positively regulated by Tbf1
PIS48539.1	23.2958514	23.0813398	23.0683592	23.65545181	24.07323164	24.19397026	0.04846024	0.926	0.826	PIS48539.1	orf19.4628	Putative cleavage and polyadenylation factor; heterozygous null mutant exhibits hypersensitivity to parnafungin and cordycepin in the C. albicans fitness test; possibly an essential gene, disruptants not obtained by UAU1 method
PIS48733.1	28.1034132	28.0395899	27.2741943	29.17343957	28.59312728	28.13455019	0.106950622	0.947	0.828	PIS48733.1	orf19.6198.1	Ortholog(s) have lipid binding activity, role in mitochondrial fission, mitophagy and mitochondrial intermembrane space localization

PIS55492.1	29.0534441	28.6006964	28.2567493	29.5325059	29.26836769	29.59899199	0.039417283	0.919	0.83	PIS55492.1	VRG4	GDP-mannose transporter; essential; required for glycosylation, hyphal growth; functional homolog of S. cerevisiae Vrg4p, which imports GDP-mannose from cytoplasm to Golgi for protein and lipid mannosylation; no mammalian homolog
PIS58751.1	24.9046241	24.7533864	24.4708793	25.33617362	25.59319279	25.69393696	0.122248792	0.95	0.831	PIS58751.1	orf19.5704	Ortholog(s) have rRNA binding activity, role in RNA splicing, mitochondrial RNA processing, mitochondrial genome maintenance, positive regulation of rRNA processing, rRNA metabolic process and mitochondrion localization
PIS55754.1	25.0225859	24.5046176	24.2992279	26.04400377	25.15555014	25.12577586	0.123544671	0.95	0.833	PIS55754.1	orf19.3649	Ortholog(s) have adenyl-nucleotide exchange factor activity, role in cytoplasm protein quality control by the ubiquitin-proteasome system and cytosol localization
PIS56624.1	26.9684232	26.7657541	26.0817268	27.56892189	27.34866823	27.41838185	0.343582852	0.967	0.84	PIS56624.1	ALR1	Putative transporter of divalent cations; hyphal-induced expression; rat catheter biofilm induced
PIS48763.1	26.395149	27.3514938	25.9834741	27.42758636	27.08861084	27.734043	0.038449802	0.918	0.84	PIS48763.1	orf19.6462	Ortholog(s) have EMC complex localization
PIS55049.1	28.1668443	27.6448519	26.431845	28.54663482	28.15792598	28.06778999	0.157809239	0.955	0.843	PIS55049.1	SMF3	Putative vacuolar iron transporter; alkaline upregulated; caspofungin repressed; induced by Mnl1 under weak acid stress; Hap43-repressed
PIS58111.1	24.1414908	24.0621784	25.9274345	25.93604242	25.04901801	25.68121926	0.187169301	0.958	0.845	PIS58111.1	orf19.6271	Ortholog(s) have role in mRNA splicing, via spliceosome and euchromatin localization

PIS56910.1	23.9532155	24.1926335	23.9421326	24.86786167	24.80674508	24.95031106	0.573000599	0.973	0.846	PIS56910.1	orf19.3202	Ortholog of C. dubliniensis CD36 : Cd36_51590, C. parapsilosis CDC317 : CPAR2_303610, C. auris B8441 : B9J08_001456 and Candida tenuis NRRL Y-1498 : CANTEDRAFT_135127
PIS51062.1	23.8159759	27.2459637	27.8133408	27.05699863	27.27980838	27.08129077	0.347283433	0.967	0.848	PIS51062.1	orf19.1618.1	Ortholog(s) have role in mitochondrial fission and mitochondrial intermembrane space localization
PIS58670.1	26.2732466	25.6053198	25.1677585	26.82624225	25.52696621	27.23670222	0.084583371	0.941	0.848	PIS58670.1	PGA31	Cell wall protein; putative GPI anchor; expression regulated upon white-opaque switch; induced by Congo Red and cell wall regeneration; Bcr1-repressed in RPMI a/a biofilms
PIS49713.1	28.3845472	27.2268917	26.4124475	28.68622433	27.91620486	27.97165431	0.193860716	0.958	0.85	PIS49713.1	NOP15	Nucleolar ribosome biogenesis factor; hyphal-induced expression; Hap43-induced; rat catheter biofilm induced
PIS52183.1	30.137752	29.4883561	28.7661692	31.04378255	30.02583835	29.87980902	0.100724039	0.945	0.852	PIS52183.1	GYP7	Protein similar to S. cerevisiae Gyp7p (GTPase-activating protein for Ypt1p); caspofungin-induced
PIS52276.1	24.0423462	24.5890196	23.4752684	24.035602	25.51692745	25.11032712	0.075956811	0.939	0.852	PIS52276.1	orf19.25	Ortholog(s) have tRNA (guanine) methyltransferase activity, tRNA (guanosine(9)-N1)-methyltransferase activity and role in mRNA methylation, tRNA N1-guanine methylation, tRNA methylation
PIS49571.1	29.5674081	29.3326317	29.026993	30.11214942	30.12407463	30.24548245	0.007567996	0.83	0.852	PIS49571.1	orf19.4204	Ortholog(s) have structural constituent of ribosome activity and mitochondrial small ribosomal subunit localization

PIS52300.1	24.7363915	25.5424776	24.4779785	26.47646945	25.78278159	25.06175197	0.061647903	0.933	0.855	PIS52300.1	orf19.2726	Putative plasma membrane protein; Plc1-regulated; Spider biofilm induced
PIS54934.1	26.4529424	24.0951157	24.8490755	24.86316303	26.89111385	26.20836877	0.237950234	0.962	0.855	PIS54934.1	TSC11	Protein similar to S. cerevisiae Tsc11p which is involved in sphingolipid biosynthesis; transposon mutation affects filamentous growth
PIS51245.1	25.7894733	25.6695025	25.0896764	26.59437224	26.9815894	25.54353484	0.228267983	0.961	0.857	PIS51245.1	NOT3	Transcriptional regulator; not required for buccal epithelial cell adherence or virulence in mouse systemic infection; null mutant colonies exhibit slightly decreased filamentation ratio; required for yeast adherence to silicone substrate
PIS58500.1	27.9068299	26.9799255	27.9354945	28.64186411	28.54473875	28.21074681	0.027866406	0.906	0.858	PIS58500.1	orf19.3329	Ortholog(s) have sphingosine-1-phosphate phosphatase activity, role in calcium-mediated signaling and endoplasmic reticulum localization
PIS54833.1	27.8023675	26.7146037	27.4559355	28.38739396	28.12415451	28.03610633	0.286644226	0.964	0.858	PIS54833.1	orf19.6316	Predicted membrane transporter, member of the L-amino acid transporter-3 (LAT3) family, major facilitator superfamily (MFS)
PIS51490.1	27.0662368	26.5639319	26.4484679	28.23986563	26.92413833	27.49668075	0.048574022	0.926	0.861	PIS51490.1	HPT1	Putative hypoxanthine-guanine phosphoribosyltransferase; protein abundance affected by URA3 expression in the CAI4 strain background; protein level decreases in stationary phase; Spider biofilm induced
PIS54889.1	25.3270272	24.1641128	24.3638912	25.56246467	25.20782339	25.67089934	0.074971986	0.938	0.862	PIS54889.1	orf19.4951	Protein of unknown function; Spider biofilm induced

PIS52316.1	25.0517555	26.147502	26.145427	26.60762707	26.97721794	26.3461295	0.217773973	0.96	0.862	PIS52316.1	orf19.5235	Putative mitochondrial ribosomal protein of the large subunit; Hap43-induced; mutants are viable; protein level decreases in stationary phase
PIS51302.1	27.6499458	27.0772992	27.0993691	27.60776391	28.50068237	28.31423406	0.039256058	0.919	0.865	PIS51302.1	PMT6	Protein mannosyltransferase; required for virulence in mice, adhesion to endothelium; role in hyphal growth signaling, hygromycin B sensitivity; no major role in cellular PMT activity; Hap4-repressed; Bcr1-repressed in RPMI a/a biofilms
PIS51277.1	30.0712483	28.9885254	27.7650965	28.86808617	30.27197084	30.28420347	0.132336422	0.951	0.866	PIS51277.1	ERG4	Protein similar to sterol C-24 reductase; shows Mob2p-dependent hyphal regulation; fluconazole-induced; caspofungin repressed; rat catheter biofilm repressed
PIS55577.1	26.6622509	25.4736904	25.6344458	26.47960515	26.74412423	27.14904959	0.11269362	0.948	0.867	PIS55577.1	ORC3	Protein similar to S. cerevisiae Orc3p, which is a component of the origin recognition complex involved in DNA replication; mutation confers hypersensitivity to toxic ergosterol analog; induced under hydroxyurea treatment
PIS58162.1	25.6612121	24.1247612	24.7767646	26.13948481	24.99171773	26.03591616	0.190900194	0.958	0.868	PIS58162.1	ARP8	Putative chromatin-remodeling enzyme complex protein; mutation confers hypersensitivity to toxic ergosterol analog, and to amphotericin B; rat catheter biofilm induced
PIS52430.1	30.3839326	29.9951128	29.3155921	30.76241567	30.60178584	30.93397831	0.022683195	0.897	0.868	PIS52430.1	MCD4	Mannose-ethanolamine phosphotransferase, essential gene involved in GPI anchor biosynthesis
PIS50501.1	25.4375254	27.1671479	25.9790727	27.040833	27.18521379	26.96408558	0.198937632	0.959	0.869	PIS50501.1	orf19.3994	Dolichyl-diphosphooligosaccharide-protein glycotransferase; predicted role in protein N-linked glycosylation, protein O-linked mannosylation; Spider biofilm repressed

PIS58641.1	25.5483823	24.6976773	26.2151833	25.91186691	26.06917716	27.09051639	0.273219085	0.964	0.87	PIS58641.1	CDC46	Putative hexameric MCM complex subunit; predicted role in control of cell division; periodic mRNA expression. peak at cell-cycle M/G1 phase; regulated by tyrosol, cell density, Plc1; repressed by alpha pheromone in SpiderM medium
PIS54897.1	29.7761857	29.6110556	29.9512326	30.24330243	30.72208554	30.98365009	0.071944197	0.937	0.87	PIS54897.1	orf19.4705	Ortholog(s) have ATP binding, ATP:3'-cytidine-cytidine-tRNA adenylyltransferase activity, CCA tRNA nucleotidyltransferase activity, CTP:tRNA cytidylyltransferase activity, tRNA binding activity
PIS54546.1	28.2456854	29.6899263	27.0918184	29.34075527	29.31188847	29.00240883	0.1996631	0.959	0.876	PIS54546.1	ARX1	Putative ribosomal large subunit biogenesis protein; repressed in core stress response; repressed by prostaglandins
PIS55508.1	23.0827605	23.8239648	23.9239181	21.90836714	25.56658029	25.98233827	0.351262343	0.967	0.876	PIS55508.1	ERD1	Putative membrane protein required for the retention of lumenal endoplasmic reticulum proteins; rat catheter biofilm induced
PIS48473.1	24.7550725	21.7694449	26.1306219	24.59664915	24.86540656	25.82669154	0.315820165	0.966	0.878	PIS48473.1	YTA6	Protein similar to S. cerevisiae Yta6p ATPase but ortholog of S. cervisiae Sap1; transposon mutation affects filamentous growth; induced by Mnl1 under weak acid stress
PIS48283.1	27.5167565	25.3057273	24.3811818	26.95893967	27.00047398	25.88090866	0.304701209	0.965	0.879	PIS48283.1	orf19.5821	Ortholog of S. cerevisiae : VRL1, C. dubliniensis CD36 : Cd36_17530, C. parapsilosis CDC317 : CPAR2_212430, C. auris B8441 : B9J08_004968 and Candida tenuis NRRL Y-1498 : CANTEDRAFT_128807
PIS49714.1	24.9726644	25.6400991	25.5394002	27.12036811	25.54297312	26.12774493	0.248620521	0.962	0.88	PIS49714.1	orf19.1449	Protein of unknown function; induced in azole-resistant strain that overexpresses MDR1; protein present in exponential and stationary growth phase yeast cultures; Spider biofilm induced

PIS52418.1	25.1475409	27.6386449	28.2905736	27.66416399	27.61536927	28.43665962	0.183765869	0.957	0.88	PIS52418.1	orf19.4895	Ortholog of C. dubliniensis CD36 : Cd36_09650, C. parapsilosis CDC317 : CPAR2_805060, C. auris B8441 : B9J08_004034 and Candida tenuis NRRL Y-1498 : CANTEDRAFT_95780
PIS48225.1	30.4020425	29.8484928	28.6653534	30.89119323	30.54921926	30.11525214	0.142808255	0.953	0.88	PIS48225.1	orf19.6438	Ortholog(s) have serine C-palmitoyltransferase activity, role in sphingolipid biosynthetic process and SPOTS complex, endoplasmic reticulum localization
PIS52325.1	27.7408587	27.7631832	27.2645612	29.54923129	28.03924432	27.82283535	0.047466901	0.925	0.881	PIS52325.1	orf19.4913	Ortholog(s) have ubiquitin binding activity
PIS52475.1	26.8614332	26.9427611	27.3327993	28.68937468	28.26568253	26.82749494	0.258219215	0.963	0.882	PIS52475.1	orf19.2820	Ortholog(s) have structural constituent of nuclear pore activity and role in NLS-bearing protein import into nucleus, nuclear pore organization, protein import into nucleus, protein localization to nuclear inner membrane
PIS58575.1	28.863628	26.9200349	23.7624793	28.39917276	27.57321876	26.22062843	0.43228844	0.97	0.882	PIS58575.1	orf19.6855	Ortholog of C. dubliniensis CD36 : Cd36_04370, C. parapsilosis CDC317 : CPAR2_105410, C. auris B8441 : B9J08_000021 and Candida tenuis NRRL Y-1498 : CANTEDRAFT_114891
PIS54645.1	23.1306044	23.980112	23.0505446	24.1687292	25.10816101	23.53486112	0.19707159	0.959	0.883	PIS54645.1	CDC47	Phosphorylated protein described as having role in control of cell division; RNA abundance regulated by tyrosol and cell density; merged with orf19.201 in Assembly 20; unmerged from orf19.201 in a revision of Assembly 21
PIS54581.1	24.7574778	28.2106209	27.6775158	25.89262391	28.67812364	28.72606522	0.347646914	0.967	0.884	PIS54581.1	orf19.3154	Ortholog(s) have role in inner mitochondrial membrane organization and mitochondrial inner membrane localization

PIS50434.1	25.1475051	26.2850203	27.2462472	28.31968038	27.03183986	25.99171946	0.27287506	0.964	0.888	PIS50434.1	orf19.6627	Protein of unknown function; possibly transcriptionally regulated upon hyphal formation
PIS58586.1	29.5324529	29.1928522	28.4157911	30.31114936	29.786041	29.70794398	0.024734498	0.901	0.888	PIS58586.1	PXA1	Putative peroxisomal, half-size adrenoleukodystrophy protein (ALD or ALDp) subfamily ABC family transporter
PIS48724.1	24.6487496	23.8014258	23.8527647	25.00390119	24.98374583	24.97831641	0.330445677	0.966	0.888	PIS48724.1	UTP18	Putative U3 snoRNA-associated protein; Hap43-induced; repressed in core stress response; physically interacts with TAP-tagged Nop1
PIS52236.1	29.0785905	27.4845705	27.6091424	30.045127	28.63318724	28.16563805	0.092084291	0.943	0.891	PIS52236.1	orf19.1991	Has domain(s) with predicted membrane localization
PIS48663.1	30.0138365	28.4185192	28.0173194	30.09664187	29.36912565	29.66105791	0.070002857	0.936	0.892	PIS48663.1	orf19.500	Ortholog(s) have tRNA (adenine(58)-N1)-methyltransferase activity, role in tRNA methylation and nucleus, tRNA (m1A) methyltransferase complex localization
PIS49464.1	28.8581839	28.0905058	27.0783524	29.16729617	28.78696936	28.74980609	0.0644312	0.934	0.892	PIS49464.1	orf19.757	Ortholog of S. cerevisiae : YPR089W, C. glabrata CBS138 : CAGL0K08008g, C. dubliniensis CD36 : Cd36_04600, C. parapsilosis CDC317 : CPAR2_105630 and C. auris B8441 : B9J08_004487
PIS51300.1	25.522929	25.0042696	22.4396746	24.22012365	26.27072032	25.15756346	0.570069198	0.973	0.894	PIS51300.1	orf19.4715	Ortholog(s) have phosphatidylinositol-4,5-bisphosphate binding, tubulin binding activity

PIS51309.1	24.1789675	24.0537587	25.6731399	26.43234971	24.98809918	25.16798933	0.264049288	0.963	0.894	PIS51309.1	orf19.5103	Protein with a predicted phosphoglycerate mutase family domain; Hap43-repressed; clade-associated gene expression; induced by hypoxia
PIS52441.1	24.423451	25.8027924	25.6529956	26.151049	26.46906352	25.94289028	0.038448007	0.918	0.895	PIS52441.1	orf19.7361	Ortholog(s) have tRNA-intron endonuclease activity, role in tRNA-type intron splice site recognition and cleavage and mitochondrial outer membrane, tRNA-intron endonuclease complex localization
PIS58999.1	30.2977253	30.3521316	30.1821921	31.33119673	30.83963601	31.35272998	0.009168378	0.845	0.897	PIS58999.1	GLC7	Putative catalytic subunit of type 1 serine/threonine protein phosphatase; regulated by Shp1; induced in high iron; alternatively spliced intron in 5' UTR
PIS48529.1	25.0585609	26.0759342	26.8358071	27.69520478	26.66882455	26.29703232	0.126387382	0.95	0.897	PIS48529.1	orf19.6861	Ortholog(s) have ubiquitin protein ligase activity, role in anaphase-promoting complex-dependent catabolic process, chromatin organization, protein ubiquitination and anaphase-promoting complex localization
PIS51641.1	30.740531	28.3176313	29.6528763	31.94909497	29.97588825	29.48132426	0.260670813	0.963	0.898	PIS51641.1	TUF1	Translation elongation factor TU; macrophage/pseudohyphal-induced; repressed upon phagocytosis by murine macrophage; levels decrease in stationary phase cells; Spider biofilm repressed
PIS52177.1	26.7686948	26.3446509	25.4474353	26.82649885	27.4484677	26.982802	0.260909747	0.963	0.899	PIS52177.1	MED16	Putative RNA polymerase II mediator complex subunit; induced by nitric oxide
PIS52261.1	24.3313631	25.6559363	24.2377493	26.60904542	25.25176717	25.06682061	0.210535992	0.96	0.901	PIS52261.1	orf19.5504	Ortholog of C. dubliniensis CD36 : Cd36_73400, C. parapsilosis CDC317 : CPAR2_703180, C. auris B8441 : B9J08_003875 and Candida tenuis NRRL Y-1498 : CANTEDRAFT_104803

PIS56647.1	27.6955162	27.786161	27.04538	28.8898994	28.52809964	27.81508777	0.120301743	0.949	0.902	PIS56647.1	orf19.1534	Ortholog of S. cerevisiae Zrt3, vacuolar membrane zinc transporter; predicted Kex2 substrate; induced in oralpharyngeal candidasis; flow model biofilm induced; Spider biofilm induced
PIS55596.1	24.3879502	24.7013691	24.6098791	26.52551525	24.57418237	25.30532245	0.09363752	0.944	0.902	PIS55596.1	orf19.6635	Ortholog of C. dubliniensis CD36 : Cd36_31170, C. parapsilosis CDC317 : CPAR2_205340, C. auris B8441 : B9J08_001700 and Candida tenuis NRRL Y-1498 : CANTEDRAFT_133521
PIS49783.1	25.6397728	26.2927836	25.5845172	26.5297278	26.86576714	26.83063489	0.012976943	0.867	0.903	PIS49783.1	orf19.6717	Putative serine hydrolase; Spider biofilm repressed
PIS58653.1	29.8859003	30.3525459	30.1401106	30.44493917	31.25283865	31.39398613	0.038487289	0.918	0.904	PIS58653.1	orf19.590	Putative thiamine biosynthesis enzyme; decreased expression in an ssr1 null mutant; protein present in exponential and stationary growth phase yeast cultures
PIS52244.1	22.9116544	23.1857444	23.4804277	24.31333899	24.41397553	23.56561445	0.056016564	0.93	0.905	PIS52244.1	SRB9	Subunit of the RNA polymerase II mediator complex; transposon mutation affects filamentous growth; suppresses S. cerevisiae diploid filamentous (flo8, ste7, ste12, tec1) or haploid invasive (flo8) mutant growth defects
PIS52465.1	27.7233513	26.9953776	26.0852365	28.35884006	28.10675028	27.05635136	0.26694295	0.963	0.906	PIS52465.1	IPT1	Inositol phosphoryl transferase; catalyzes the synthesis of the most abundant sphingolipid, mannose-(inositol-P)2-ceramide, M(IP)2C, from MIPC; required for wild-type membrane localization of Cdr1; Spider biofilm induced
PIS56535.1	29.0494162	29.2859102	28.9695368	30.38357097	29.63502344	30.00346845	0.028462408	0.906	0.906	PIS56535.1	orf19.5516	Ortholog(s) have role in SRP-dependent cotranslational protein targeting to membrane and signal recognition particle, endoplasmic reticulum targeting localization

PIS56526.1	25.466991	25.1664953	26.4662648	25.68105094	26.51546297	27.62528587	0.306915392	0.965	0.907	PIS56526.1	orf19.3481	Putative mitochondrial ATP-dependent RNA helicase of the DEAD-box family, transcription is activated in the presence of elevated CO2
PIS52034.1	26.8397076	27.5506837	26.3472626	27.77946369	27.42246612	28.25785801	0.049822781	0.927	0.907	PIS52034.1	POB3	Protein involved in chromatin assembly and disassembly; ortholog of S. cerevisiae Pob3; transposon mutation affects filamentous growth; rat catheter biofilm repressed
PIS49639.1	24.4522426	24.9302089	24.5028157	26.14038289	25.29347913	25.17989995	0.023429081	0.898	0.909	PIS49639.1	MAK32	Putative protein involved in the structural stability of L-A double-stranded RNA-containing particles; downregulated upon adherence to polystyrene
PIS58082.1	26.1233721	27.0773792	24.4838613	26.09634013	27.01992149	27.29962573	0.139971529	0.952	0.91	PIS58082.1	orf19.6136	Ortholog of S. cerevisiae Mrpl4, a mitochondrial ribosomal protein of the large subunit; repressed by nitric oxide
PIS50543.1	26.6550801	26.514104	23.8442529	27.24069458	27.12070794	25.38293438	0.193039984	0.958	0.91	PIS50543.1	VPS20	ESCRT III complex protein; role in multivesicular body (MVB) trafficking; required for processing of Rim8; Bcr1-repressed in RPMI a/a biofilms
PIS49825.1	22.4338089	23.2799768	22.4341985	23.70472934	24.01551232	23.16337966	0.019962403	0.891	0.912	PIS49825.1	orf19.4365	Has domain(s) with predicted RNA methyltransferase activity and role in RNA processing
PIS58402.1	27.203473	27.7287571	26.4497728	28.26011396	27.87449478	27.98564032	0.106724826	0.947	0.913	PIS58402.1	ALG8	Putative glucosyltransferase involved in cell wall mannan biosynthesis; transcription is elevated in nik1 and sln1 homozygous null mutants, but not in the chk1 null mutant; possibly an essential gene, disruptants not obtained by UAU1 method

PIS58657.1	24.258798	24.2968574	24.9952834	26.0253422	25.27883503	24.98568163	0.085080934	0.941	0.913	PIS58657.1	RIO2	Putative serine kinase with a predicted role in the processing of the 20S pre-rRNA into mature 18S rRNA; null mutants are hypersensitive to caspofungin
PIS50589.1	26.5198693	25.3387524	24.2393455	26.67195558	26.67385448	25.49633489	0.271660832	0.964	0.915	PIS50589.1	orf19.4172	Ortholog of C. dubliniensis CD36 : Cd36_40750, C. parapsilosis CDC317 : CPAR2_402570, C. auris B8441 : B9J08_004417 and Candida tenuis NRRL Y-1498 : CANTEDRAFT_112981
PIS49489.1	30.3377421	30.3626175	30.4206198	31.30683214	31.15745167	31.40530526	0.002076759	0.694	0.916	PIS49489.1	SCO1	Putative copper transporter; Hap43p-repressed gene
PIS48317.1	29.6621276	29.6771502	28.9775727	30.88608448	30.08029746	30.10197758	0.078858261	0.939	0.917	PIS48317.1	ERG7	2,3-epoxysqualene-lanosterol cyclase (lanosterol synthase), conversion of 2,3-oxidosqualene to lanosterol in sterol biosynthesis; fluconazole-induced; possibly essential, disruptants not obtained by UAU1 method; rat catheter biofilm induced
PIS56704.1	26.4082037	25.9515915	26.2323199	27.21830494	26.86968671	27.25506724	0.022093062	0.895	0.917	PIS56704.1	orf19.7627	Ortholog of S. cerevisiae : YNL320W, C. glabrata CBS138 : CAGL0M04125g, C. dubliniensis CD36 : Cd36_35340, C. parapsilosis CDC317 : CPAR2_200350 and C. auris B8441 : B9J08_001244
PIS58600.1	26.2018887	25.3453062	25.243962	26.96846999	26.72311521	25.85879253	0.159348685	0.955	0.92	PIS58600.1	orf19.7425	Ortholog(s) have uracil DNA N-glycosylase activity, role in DNA repair, base-excision repair and cytoplasm, mitochondrion, nucleus localization
PIS51460.1	28.2110856	29.157605	29.5682457	29.07252078	30.00025451	30.63178911	0.141495389	0.952	0.923	PIS51460.1	PDX1	Pyruvate dehydrogenase complex protein X; essential component of the mitochondrial pyruvate dehydrogenase complex; role in the respiratory pathway; protein present in exponential and stationary growth phase yeast; Spider biofilm repressed

PIS51994.1	28.5112587	28.5941636	28.0412034	29.08779039	29.33160174	29.49897442	0.054544284	0.929	0.924	PIS51994.1	MRPL19	Putative ribosomal protein; induced upon adherence to polystyrene; Spider biofilm repressed
PIS48506.1	27.2595697	25.9534224	26.4214728	29.55370264	27.02304446	25.83268744	0.25305481	0.963	0.925	PIS48506.1	orf19.787.1	Protein of unknown function; ORF added to Assembly 21 based on comparative genome analysis; protein detected by mass spec in stationary phase cultures
PIS56583.1	27.5488619	27.0928668	27.5938293	29.36751114	27.54639257	28.09996664	0.146908894	0.953	0.926	PIS56583.1	CTA8	Essential transcription factor, mediates heat shock transcriptional induction; in the absence of heat stress, Cta8p levels are modulated by growth temperature to regulate basal expression of genes involved in protein folding
PIS51995.1	24.3477849	23.7629744	24.4981959	23.85953705	25.79570472	25.7459897	0.274848048	0.964	0.931	PIS51995.1	orf19.4906	Putative adhesin-like protein; positively regulated by Tbf1; Spider biofilm induced
PIS58727.1	26.8552004	27.2398861	27.1891045	28.14375697	28.15304418	27.78316433	0.075587739	0.938	0.932	PIS58727.1	orf19.5397	Putative conserved oligomeric Golgi complex subunit; decreased transcription is observed upon fluphenazine treatment
PIS56801.1	27.3844259	25.778053	24.9578169	27.03357687	27.30879977	26.57598935	0.185474246	0.957	0.933	PIS56801.1	orf19.7116	Protein of unknown function; transcript induced in RHE model of oral candidiasis; Spider biofilm repressed
PIS51195.1	30.9598233	30.7867404	30.6384457	31.32694761	31.89999583	31.9705166	0.01005785	0.851	0.937	PIS51195.1	FDH3	Glutathione-dependent formaldehyde dehydrogenase; glycine catabolism; repressed by Efg1 in yeast, not hyphal growth conditions; induced by Mnl1 under weak acid stress; Spider biofilm repressed

PIS55705.1	26.8095431	26.0854264	26.1376737	27.46423357	27.63684944	26.74443139	0.065752825	0.935	0.938	PIS55705.1	orf19.2208	Ortholog(s) have role in negative regulation of translation, response to salt stress and P-body localization
PIS51458.1	25.5069736	25.6805443	27.1144099	27.40016646	26.9288758	26.78834822	0.157891783	0.955	0.938	PIS51458.1	orf19.4529	Ortholog of Srp21, signal recognition particle subunit, functions in protein targeting to the endoplasmic reticulum membrane; predicted adhesin-like protein; mutants are viable
PIS58888.1	21.6921885	23.3176665	23.1951383	23.03885343	23.5172957	24.46692335	0.100878357	0.945	0.939	PIS58888.1	orf19.2426	Predicted HD domain metal dependent phosphohydrolase; Spider biofilm repressed
PIS50581.1	24.9118237	25.0535049	25.2410202	26.37885197	26.03073056	25.61460334	0.014234002	0.873	0.939	PIS50581.1	orf19.4185	Ortholog(s) have phosphatase activity, phosphoprotein phosphatase activity, thiosulfate sulfurtransferase activity and role in dephosphorylation
PIS52278.1	24.7426227	24.6303659	24.2952838	25.90922794	25.59762877	24.98977462	0.104261825	0.946	0.943	PIS52278.1	ORC4	Phosphorylated protein similar to S. cerevisiae Orc4, subunit of the origin recognition complex (ORC); induced by alpha pheromone in SpiderM medium
PIS56532.1	30.3885815	29.7647894	29.2077528	31.14313204	30.46925626	30.57656873	0.065186088	0.934	0.943	PIS56532.1	orf19.5515	Ortholog(s) have mRNA binding, ribosome binding activity
PIS58122.1	28.0434412	26.016354	26.3383901	27.44423708	28.92784677	26.85661249	0.325744147	0.966	0.944	PIS58122.1	BPH1	Ortholog of S. cerevisiae Bph1; a putative ortholog of human Chediak-Higashi syndrome protein and murine beige gene implicated in disease syndromes involving defective lysosomal trafficking; mutant is viable

PIS49813.1	28.6497508	27.823282	28.9178317	29.24450849	29.22477909	29.75736244	0.049397493	0.926	0.945	PIS49813.1	YCP4	Flavodoxin-like protein involved in oxidative stress protection and virulence; flow model, rat catheter and Spider biofilm repressed
PIS56829.1	29.8322515	29.3484907	29.3771033	30.77114338	30.29517308	30.32928671	0.005103013	0.797	0.946	PIS56829.1	PFK26	Putative 6-phosphofructo-2-kinase; protein repressed during the mating process
PIS49669.1	25.3148019	23.8963478	24.2437975	25.59132764	25.62448011	25.08027251	0.028616448	0.907	0.947	PIS49669.1	orf19.6667	Predicted histone deacetylase activity; Spider biofilm induced
PIS58075.1	26.3426458	25.7704921	25.9113505	26.75318264	26.54082617	27.5713699	0.255379249	0.963	0.947	PIS58075.1	WAL1	Protein required for hyphal growth and for wild-type cell morphology, polarized budding, endocytosis, vacuole morphology; similar to Wiskott-Aldrich syndrome protein; localizes to cortical actin patches and hyphal tips
PIS50521.1	28.6444434	27.5143317	26.9777178	29.037094	28.69451815	28.24796926	0.049875024	0.927	0.948	PIS50521.1	orf19.1323	Phosphorylated protein of unknown function; Hap43p-repressed gene
PIS52193.1	24.6086064	24.4349627	24.0932316	25.59161958	25.20701526	25.18202168	0.008326226	0.838	0.948	PIS52193.1	orf19.1769	Ortholog(s) have G-protein beta/gamma-subunit complex binding, actin binding activity, role in positive regulation of transcription by RNA polymerase II, protein folding and cytoplasm localization
PIS51170.1	26.6673948	26.8860059	27.0682115	27.65084454	27.22565715	28.59221688	0.109255128	0.947	0.949	PIS51170.1	orf19.4839	GET complex subunit; expression downregulated in an ssr1 null mutant

PIS55481.1	27.5661337	26.2254035	26.7946772	27.63678076	27.15926748	28.63793799	0.277152601	0.964	0.949	PIS55481.1	PTC1	Putative protein phosphatase of the Type 2C-related family (serine/threonine-specific), similar to S. cerevisiae Ptc1p; mutant shows virulence defect
PIS49524.1	25.6251681	25.908751	25.189095	25.68961352	26.54788583	27.33892888	0.117924306	0.949	0.951	PIS49524.1	orf19.4756	Ortholog of S. cerevisiae : YTP1, C. dubliniensis CD36 : Cd36_08490, C. parapsilosis CDC317 : CPAR2_801590, C. auris B8441 : B9J08_004547 and Candida tenuis NRRL Y-1498 : CANTEDRAFT_109732
PIS55693.1	24.8446077	25.9815784	23.3391679	25.27624708	25.95216104	25.79156726	0.1223839	0.95	0.952	PIS55693.1	HAL22	Putative phosphoadenosine-5'-phosphate or 3'-phosphoadenosine 5'-phosphosulfate phosphatase; possible role in sulfur recycling; Hap43-repressed; F-12/CO2 biofilm induced
PIS48227.1	23.6956378	23.4757525	25.7789555	24.91073108	25.74347047	25.16385865	0.248651394	0.962	0.956	PIS48227.1	orf19.6436	Ortholog of C. dubliniensis CD36 : Cd36_34210, C. parapsilosis CDC317 : CPAR2_205590, C. auris B8441 : B9J08_004911 and Candida tenuis NRRL Y-1498 : cten_CGOB_00133
PIS52332.1	24.4725067	25.5191331	25.8610112	25.39865495	26.4136541	26.91243793	0.241599761	0.962	0.957	PIS52332.1	orf19.6348	Predicted cysteine proteinase domain; mutants are viable
PIS52110.1	25.0632873	25.3382809	27.1600634	27.46646087	26.42774498	26.54203392	0.086131437	0.942	0.958	PIS52110.1	orf19.4503	Similar to HMG-box variant of S. pombe; Spider biofilm repressed
PIS52353.1	24.1663893	24.5016169	23.8307598	25.2017942	24.66249284	25.51435367	0.101937904	0.946	0.96	PIS52353.1	ECM25	Non-essential protein involved in cell morphogenesis

PIS49615.1	23.1052738	22.8472938	22.2662625	23.38660658	24.05795182	23.65297173	0.62172344	0.974	0.96	PIS49615.1	KNS1	Protein kinase involved in negative regulation of PolIII transcription; effector kinase of the TOR signaling pathway, phosphorylates Rpc53p to regulate ribosome and tRNA biosynthesis; Spider and flow model biofilm induced
PIS52250.1	24.7020661	24.284698	24.9739573	25.7023491	25.31993543	25.83108404	0.016691024	0.882	0.964	PIS52250.1	orf19.1458	Ortholog(s) have tRNA-specific adenosine deaminase activity and role in tRNA modification
PIS50470.1	24.7187905	23.8378716	23.9078348	25.39605341	24.59409597	25.37377057	0.204072529	0.959	0.966	PIS50470.1	PSD2	Ortholog(s) have phosphatidylserine decarboxylase activity, role in phosphatidylcholine biosynthetic process, phosphatidylethanolamine biosynthetic process and endosome localization
PIS58627.1	24.5214464	23.8301247	24.3087384	25.06496358	25.08305941	25.41266332	0.244636973	0.962	0.967	PIS58627.1	orf19.21	Ortholog(s) have role in ethanol metabolic process and mitochondrial inner membrane localization
PIS58902.1	25.329097	24.8129008	25.48732	26.02288593	26.01010955	26.4969653	0.057504745	0.931	0.967	PIS58902.1	orf19.2412	Ortholog(s) have role in late endosome to vacuole transport via multivesicular body sorting pathway and Vps55/Vps68 complex, late endosome localization
PIS50392.1	25.7515477	23.6524671	25.5334582	25.55569826	26.40651543	25.88098862	0.080070539	0.94	0.969	PIS50392.1	OMA1	Putative metalloendopeptidase of mitochondrial inner membrane, involved in TOR-mediated signaling
PIS52254.1	25.1296678	24.7602908	23.6147942	26.11443854	24.88271893	25.41372287	0.0510255	0.927	0.969	PIS52254.1	SOG2	Leucine-rich-repeat domain protein of RAM cell wall integrity signaling network; role in cell separation, azole sensitivity; required for hyphal growth; lacks orthologs in higher eukaryotes

PIS58521.1	24.9717768	24.0964426	23.2649135	25.70737751	25.08625304	24.45389357	0.137996387	0.952	0.971	PIS58521.1	orf19.3272	Ortholog(s) have protein-folding chaperone binding activity, role in box C/D snoRNP assembly, protein folding and R2TP complex localization
PIS58611.1	26.9553078	27.0502949	27.2403723	27.97885289	28.03048699	28.15236221	0.002902066	0.737	0.972	PIS58611.1	SNO1	Protein with a predicted role in pyridoxine metabolism; stationary phase protein; regulated by Tup1, Efg1; Spider biofilm induced
PIS51101.1	28.8809199	27.6040338	26.7253983	28.9226839	28.88857896	28.31820131	0.072798811	0.937	0.973	PIS51101.1	orf19.1217	Ortholog(s) have ribosomal large subunit binding, ubiquitin protein ligase activity, ubiquitin-protein transferase activity
PIS55688.1	25.5042042	26.2093482	25.6586795	26.88423335	27.06105543	26.35045215	0.040334706	0.92	0.975	PIS55688.1	orf19.4666	Protein of unknown function; hyphal-induced expression, regulated by Cyr1, Ras1, Efg1; Spider biofilm induced
PIS52356.1	28.2431122	26.6288499	27.7670037	28.82070731	28.51330326	28.23210871	0.047450947	0.925	0.976	PIS52356.1	orf19.4013	Putative protein of unknown function; Hap43p-repressed gene; S. cerevisiae ortholog YHR045W localizes to the endoplasmic reticulum
PIS49820.1	31.0436896	30.3672827	30.0500804	31.52007003	31.28524579	31.58893424	0.006446763	0.817	0.978	PIS49820.1	VTC3	Putative polyphosphate synthetase; downregulated in core caspofungin response; Hog1p-induced; transcription is opaque-specific; fungal-specific (no human or murine homolog)
PIS52059.1	26.1029716	24.7270003	24.1714206	25.40417333	25.91106327	26.63051311	0.148194089	0.953	0.981	PIS52059.1	MED17	Putative RNA polymerase II mediator complex subunit; possibly an essential gene, disruptants not obtained by UAU1 method

PIS49601.1	28.0182608	27.9706247	28.4447527	29.06350033	29.12174629	29.19553295	0.005359895	0.801	0.982	PIS49601.1	NSA2	Putative protein constituent of 66S pre-ribosomal particles; Hap43-induced; repressed by prostaglandins
PIS55084.1	32.5037248	32.1256166	30.2996526	32.95335576	32.41863455	32.50446358	0.12845612	0.951	0.982	PIS55084.1	orf19.3475	Described as a Gag-related protein; hyphal induced; downregulation correlates with clinical development of fluconazole resistance; repressed by nitric oxide, 17-beta-estradiol, ethynyl estradiol
PIS51180.1	26.747532	25.7652289	25.1401404	26.92431872	26.93295399	26.74405171	0.127328647	0.95	0.983	PIS51180.1	FGR34	Protein lacking an ortholog in S. cerevisiae; transposon mutation affects filamentous growth
PIS55834.1	32.4484848	30.3319557	30.4681289	34.15068211	31.43670586	30.61531538	0.406389935	0.969	0.985	PIS55834.1	orf19.4216	Putative heat shock protein; decreased expression in hyphae; transcription is increased in populations of cells exposed to fluconazole over multiple generations; overexpression increases resistance to farnesol and azoles
PIS58481.1	33.9840672	33.4369037	34.3132124	34.08253504	35.70969948	34.90124947	0.354823016	0.967	0.986	PIS58481.1	IDP2	Isocitrate dehydrogenase; white-opaque switch regulated; morphology-regulation by Ssn6; protein in exponential and stationary phase yeast; Hap43-repressed; Spider biofilm repressed by Bcr1, Tec1, Ndt80, Rob1, Brg1; Spider biofilm induced
PIS56708.1	25.6563772	24.7244338	25.5464039	27.4083695	25.44129304	26.05271784	0.089758041	0.943	0.992	PIS56708.1	MCD1	Alpha-kleisin cohesin complex subunit; for sister chromatid cohesion in mitosis and meiosis; repressed by alpha pheromone in SpiderM medium; periodic cell-cycle expression; Hap43-repressed; rat catheter and Spider biofilm repressed
PIS51457.1	27.8738827	27.8295147	28.0693113	29.30082178	29.01229818	28.43495055	0.031728882	0.911	0.992	PIS51457.1	orf19.4530.1	Protein of unknown function; regulated by Nrg1, Tup1; Spider and flow model biofilm induced

PIS52076.1	24.1426033	23.9974211	24.5455448	25.48029084	24.38044128	25.80232628	0.026421988	0.903	0.992	PIS52076.1	SSF1	Protein involved in ribosome biogenesis; ortholog of S. cerevisiae Ssf1; Hap43-induced; rat catheter and Spider biofilm induced
PIS49491.1	25.473732	23.8395748	23.0505016	25.69856575	25.2556929	24.38972608	0.077724654	0.939	0.993	PIS49491.1	orf19.7316	Putative phytanoyl-CoA dioxygenase family protein; mutation confers hypersensitivity to 5-fluorocytosine (5-FC), 5-fluorouracil (5-FU), and tubercidin (7-deazaadenosine); induced by nitric oxide
PIS48391.1	27.4361162	27.5737336	25.5273925	28.08783937	27.56058181	27.87382587	0.137992429	0.952	0.995	PIS48391.1	orf19.2494	Ortholog(s) have glutaminyl-tRNA synthase (glutamine-hydrolyzing) activity, role in glutaminyl-tRNAGln biosynthesis via transamidation, mitochondrial translation and glutamyl-tRNA(Gln) amidotransferase complex, mitochondrion localization
PIS51266.1	27.6515502	27.1911598	26.6499451	28.24875685	28.30838246	27.92025339	0.01376945	0.871	0.995	PIS51266.1	orf19.6156	Ortholog of S. cerevisiae : AIM11, C. glabrata CBS138 : CAGL0I04928g, C. dubliniensis CD36 : Cd36_80770, C. parapsilosis CDC317 : CPAR2_102260 and C. auris B8441 : B9J08_002841
PIS58368.1	29.0180098	27.0094569	26.2641485	29.25742866	28.00344539	28.01704651	0.148560597	0.953	0.995	PIS58368.1	orf19.804.1	Ortholog of S. cerevisiae : MIN8, C. dubliniensis CD36 : Cd36_02990, C. parapsilosis CDC317 : CPAR2_211020, C. auris B8441 : B9J08_000865 and Candida tenuis NRRL Y-1498 : CANTEDRAFT_116148
PIS58778.1	27.4626819	27.2314924	26.2619323	28.33547013	27.84323386	27.77167423	0.132243461	0.951	0.998	PIS58778.1	orf19.7082	S-adenosylmethionine transporter of the mitochondrial inner membrane; mitochondrial carrier family; predicted role in biotin biosynthesis and respiratory growth; Spider biofilm repressed
PIS51702.1	26.9475449	25.8264007	25.479397	27.58334753	26.56281643	27.1115413	0.061617985	0.933	1	PIS51702.1	END3	Protein involved in endocytosis, cell wall integrity and morphology; regulated by Gcn4p; induced in response to amino acid starvation (3-aminotriazole treatment)

PIS51221.1	28.4035229	27.6080131	27.9878235	29.22820974	28.77176616	29.00276858	0.056502121	0.93	1	PIS51221.1	FLU1	Multidrug efflux pump of the plasma membrane; MDR family member of the MFS (major facilitator superfamily) of transporters; involved in histatin 5 efflux; fungal-specific (no human/murine homolog)
PIS52210.1	26.9203567	26.5169238	26.2889643	27.69485782	27.49717241	27.53563291	0.270136439	0.964	1	PIS52210.1	IST1	Protein with a positive role in the multivesicular body sorting pathway; rat catheter biofilm repressed
PIS50366.1	27.6866339	26.4547673	26.2826144	28.27005517	27.62462276	27.5332089	0.041771374	0.921	1	PIS50366.1	orf19.168	Ortholog(s) have role in U4 snRNA 3'-end processing, exonucleolytic trimming to generate mature 3'-end of 5.8S rRNA from tricistronic rRNA transcript (SSU-rRNA, 5.8S rRNA and LSU-rRNA), more
PIS52401.1	23.8500611	24.0887246	24.635837	25.38202078	24.71030049	25.49058961	0.099149793	0.945	1	PIS52401.1	orf19.3627	Ortholog of C. dubliniensis CD36 : Cd36_22640, C. parapsilosis CDC317 : CPAR2_406910, C. auris B8441 : B9J08_004017 and Candida tenuis NRRL Y-1498 : CANTEDRAFT_104937
PIS51636.1	23.308801	23.0761783	24.807006	24.85237126	24.53075578	24.81526601	0.293422362	0.965	1	PIS51636.1	orf19.6054	Protein with a Bul1 domain; binds the ubiquitin ligase Rsp5 and is involved in intracellular trafficking of a general amino acid permease Gap1; repressed in an azole-resistant strain overexpressing MDR1; Spider biofilm induced
PIS56900.1	31.2483441	30.4011176	30.8708224	32.10737243	31.78049066	31.64172383	0.041112339	0.92	1	PIS56900.1	SAM51	Component of the SAM complex involved in mitochondrial protein import, involved in beta-barrel protein assembly; member of the Omp85 protein family
PIS55565.1	26.9104147	26.3869479	25.6409717	27.48078606	27.26856929	27.21722337	0.015882718	0.879	1.01	PIS55565.1	MNN14	Predicted alpha-1,3-mannosyltransferase activity with a role in protein glycosylation; Hap43-repressed; Spider biofilm induced

PIS48439.1	26.4213247	27.0652018	24.7459459	26.92741257	27.65440087	26.67239511	0.193362971	0.958	1.01	PIS48439.1	NMD3	Putative nonsense-mediated mRNA decay protein; repressed in core stress response; repressed by prostaglandins
PIS56837.1	24.4312731	23.1097692	24.498575	25.0300314	25.13902835	24.89696378	0.105985895	0.946	1.01	PIS56837.1	orf19.2769	Putative protease B inhibitor; hyphal-induced expression; Cyr1p- and Ras1p-repressed
PIS51149.1	26.873442	26.8520612	25.9966596	27.67988391	27.7060274	27.37934519	0.117607107	0.949	1.01	PIS51149.1	orf19.4245	Protein with a predicted pleckstrin domain; Hap43-repressed gene; mutation causes decreased interaction with macrophages
PIS48734.1	28.7538003	27.9720276	27.0754132	28.23702313	29.14750749	29.43556535	0.036884925	0.916	1.01	PIS48734.1	orf19.6199	Ortholog(s) have 5'-3' DNA helicase activity, role in postreplication repair and DNA helicase A complex, nuclear replisome localization
PIS51190.1	25.9905099	25.1691363	25.2306608	26.8348482	26.25944626	26.3337813	0.006045027	0.812	1.01	PIS51190.1	orf19.7593	Putative asparaginase; predicted role in asparagine catabolism; Spider biofilm induced
PIS50456.1	26.7964577	28.1727103	27.5124545	27.80141037	28.0509536	29.65861135	0.258578812	0.963	1.01	PIS50456.1	orf19.879	Ortholog(s) have myosin binding activity, role in intracellular mRNA localization, mating type switching and cytoplasm localization
PIS54754.1	27.1505959	27.0750871	27.0791969	27.51196253	28.1838605	28.63571098	0.02731969	0.905	1.01	PIS54754.1	SCT2	Putative glycerol-3-phosphate acyltransferase; fungal-specific (no human or murine homolog)

PIS52322.1	31.8874474	31.2232472	30.1631286	32.50891432	31.65862237	32.13060778	0.060262881	0.932	1.01	PIS52322.1	SNT1	Ortholog of S. cerevisiae Snt1; an NAD-independent histone deacetylase; heterozygous transposon mutation affects filamentous growth; null mutants are viable
PIS51881.1	26.916764	27.6889443	27.9227264	28.31234576	28.95940097	28.28715535	0.013248595	0.869	1.01	PIS51881.1	TRM1	Putative N2,N2-dimethylguanine tRNA methyltransferase; induced upon adherence to polystyrene
PIS49581.1	29.9273319	28.5768067	27.7231764	30.14778168	30.01052536	29.11599824	0.198738353	0.959	1.02	PIS49581.1	CDC11	Septin; cell and hyphal morphology, agar-invasive growth, full virulence and kidney tissue invasion in mouse, but not kidney colonization, immunogenicity; hyphal and cell-cycle-regulated phosphorylation; rat catheter biofilm repressed
PIS55038.1	28.2258927	27.934583	27.6655854	29.35049636	28.82692281	28.71727117	0.153891751	0.954	1.02	PIS55038.1	DFG5	N-linked mannoprotein of cell wall and membrane; possible signal transducer; role in hyphal growth and HWP1 induction in alkaline pH; GPI modification predicted; dfg5 dcw1 double mutant is inviable; caspofungin-induced, fungal-specific
PIS54845.1	28.2744229	25.9622186	27.0312452	28.05780545	27.99294851	28.27976808	0.133571868	0.951	1.02	PIS54845.1	KAP120	Putative karyopherin; mutation confers hypersensitivity to cytochalasin D
PIS50572.1	23.8089332	24.6489506	24.399519	25.36183232	25.30371272	25.26319265	0.006999288	0.824	1.02	PIS50572.1	MSM1	Mitochondrial methionyl-tRNA synthetase (MetRS); functionally complements methionine auxotrophy of an E. coli MetRS mutant; transcript regulated by Nrg1; flow model biofilm induced
PIS48449.1	23.7187692	24.7780754	24.3150858	25.22778349	25.13422809	25.49534822	0.566722083	0.973	1.02	PIS48449.1	orf19.1045	Putative U2 snRNP protein; Hap43p-induced gene; mutation confers hypersensitivity to 5-fluorocytosine (5-FC), 5-fluorouracil (5-FU), and tubercidin (7-deazaadenosine)

PIS55692.1	25.7516571	26.1378324	25.9112602	27.1561817	25.86841856	27.84454729	0.21491204	0.96	1.02	PIS55692.1	orf19.109	Probable mitochondrial tyrosyl-tRNA synthetase, based on conservation in other fungi
PIS55475.1	24.8284769	24.9410324	24.9357197	26.44444446	26.15111778	25.15707894	0.043236526	0.922	1.02	PIS55475.1	orf19.1163	Ortholog(s) have guanyl nucleotide binding activity, role in mitochondrial translational elongation, mitochondrial translational initiation and matrix side of mitochondrial inner membrane localization
PIS48514.1	23.9840418	22.4232489	25.3073338	24.38591451	25.16567642	25.23172587	0.474805449	0.971	1.02	PIS48514.1	orf19.3782	Predicted membrane transporter; member of the peptide-acetyl-coA transporter (PAT) family, major facilitator superfamily (MFS); induced by alpha pheromone in Spider medium
PIS56736.1	27.191021	26.6817999	28.7331805	28.12369529	28.11537736	29.41951363	0.09299974	0.943	1.02	PIS56736.1	orf19.6090	Putative nucleolar protein with a predicted role in pre-rRNA processing and ribosome biogenesis; repressed by nitric oxide; required for flow model biofilm formation; Spider biofilm repressed
PIS55052.1	23.9282893	25.0311944	23.0170846	26.22173087	24.2546044	24.55561669	0.17590679	0.957	1.02	PIS55052.1	PHO89	Putative phosphate permease; transcript regulated upon white-opaque switch; alkaline induced by Rim101; possibly adherence-induced; F-12/CO2 model, rat catheter and Spider biofilm induced
PIS52044.1	29.9207404	30.1892727	30.2024899	31.03485769	30.89501931	31.46444116	0.006978461	0.824	1.03	PIS52044.1	orf19.1545	Ortholog(s) have structural constituent of ribosome activity and mitochondrial small ribosomal subunit localization
PIS49808.1	28.1469071	27.5061246	26.8489841	29.14068373	28.32018514	28.12708121	0.05479832	0.929	1.03	PIS49808.1	orf19.549	Ortholog(s) have structural constituent of ribosome activity and mitochondrial large ribosomal subunit localization

PIS50611.1	23.2728271	24.6320165	23.5826777	23.9516147	22.78746943	27.83374256	0.307420298	0.965	1.03	PIS50611.1	PEX7	Ortholog(s) have peroxisome matrix targeting signal-2 binding activity, role in protein import into peroxisome matrix, docking and cytosol, peroxisome localization
PIS52010.1	25.2113793	21.7195363	24.1036775	24.30687092	25.05515772	24.75220133	0.528945337	0.973	1.03	PIS52010.1	PLB5	Putative GPI-linked phospholipase B, fungal-specific (no mammalian homolog); null mutation eliminates cell-associated phospholipase A2 activity and attenuates virulence; fluconazole-repressed; flow model biofilm repressed
PIS56847.1	24.5595304	23.3654454	26.6025095	24.57642769	26.29218852	26.74596936	0.159944633	0.955	1.03	PIS56847.1	TIM10	Predicted protein of the mitochondrial intermembrane space with role in protein import into mitochondrial inner membrane
PIS58027.1	29.650432	29.9028931	30.3137009	30.96978861	30.96461886	31.01548109	0.008492485	0.839	1.03	PIS58027.1	TOM1	Putative E3 ubiquitin ligase; transcript regulated by Nrg1 and Mig1
PIS58716.1	31.9403237	31.8986818	31.6789374	33.23840095	32.78843714	32.60628005	0.002196042	0.701	1.04	PIS58716.1	CDR1	Multidrug transporter of ABC superfamily; transports phospholipids in an in-to-out direction; induced by beta-estradiol, progesterone, corticosteroid, or cholesterol; Spider biofilm induced
PIS51664.1	26.945311	25.4235995	25.115315	28.82097529	27.2211574	24.56616267	0.337283414	0.967	1.04	PIS51664.1	MIA40	Predicted component of the mitochondrial intermembrane space import machinery; Hap43p-repressed gene
PIS49606.1	24.484382	24.0569159	25.0624498	25.3270914	25.97059811	25.41182192	0.049697469	0.926	1.04	PIS49606.1	NUP49	Nuclear pore protein

PIS51425.1	23.2403916	24.3412185	23.6893617	25.13947219	24.70302115	24.54586694	0.202622944	0.959	1.04	PIS51425.1	orf19.1083	Putative protein of unknown function; macrophage-induced gene
PIS48346.1	28.614671	27.3920552	28.1956172	29.37059741	28.85374726	29.10543604	0.080889419	0.94	1.04	PIS48346.1	orf19.1246	Putative eisosome component role in proper eisosome assembly; upregulated in cyr1 null mutant
PIS51747.1	26.7702847	25.7702207	26.0153303	28.27757765	26.46720422	26.93755963	0.173197522	0.956	1.04	PIS51747.1	orf19.2037	Has domain(s) with predicted role in cellular response to amino acid stimulus, cholesterol homeostasis, endosomal transport, lysosome organization and positive regulation of MAPK cascade, more
PIS54651.1	29.3523982	28.828589	28.5874892	30.22429774	29.91701822	29.75758558	0.003424946	0.756	1.04	PIS54651.1	orf19.213	Subunit of the 19S regulatory particle lid of the proteasome
PIS54579.1	25.5209209	25.1005329	25.1023218	26.29128618	26.19873675	26.35631723	0.150043279	0.954	1.04	PIS54579.1	orf19.3158	Ortholog of S. cerevisiae : RMD1, C. glabrata CBS138 : CAGL0M10483g, C. dubliniensis CD36 : Cd36_81090, C. parapsilosis CDC317 : CPAR2_101950 and C. auris B8441 : B9J08_002355
PIS49508.1	23.4590586	22.8270694	22.2494592	23.8283519	24.3354405	23.47993375	0.09796512	0.945	1.04	PIS49508.1	orf19.3418	Guanidinobutyrase (Gbase), enzyme involved in metabolism of guanidinobutyrate
PIS51121.1	28.3193536	28.532228	28.4991148	29.74477926	29.36613433	29.35268519	0.008936739	0.843	1.04	PIS51121.1	orf19.639.1	Ortholog(s) have structural constituent of ribosome activity and mitochondrial large ribosomal subunit localization

PIS52027.1	25.9081479	24.10328	24.9894465	26.1165757	25.15227374	26.85610888	0.182146548	0.957	1.04	PIS52027.1	RXT3	Putative transcriptional repressor
PIS49792.1	26.2753783	25.4713372	24.6255437	27.0429785	26.78673628	25.65691572	0.203612855	0.959	1.04	PIS49792.1	TAC1	Zn(2)-Cys(6) transcriptional activator of drug-responsive genes (CDR1 and CDR2); binds DRE element; gene in zinc cluster region near MTL locus; resequencing indicates that TAC1 spans orf19.3188 and orf19.3189; Spider biofilm induced
PIS51802.1	23.0908163	23.8316133	24.3102084	24.36437416	25.01267809	24.99835529	0.1279979	0.95	1.05	PIS51802.1	CCH1	Voltage-gated Ca2+ channel of the high affinity calcium uptake system; roles in thigmotropism, establishment of galvanotropism; transcript regulated by Nrg1 and Mig1; flow model biofilm repressed
PIS54755.1	27.3080076	27.7483585	27.0512881	28.36510583	28.72851115	28.15494909	0.004622999	0.787	1.05	PIS54755.1	orf19.5817	Ortholog(s) have guanyl-nucleotide exchange factor activity and role in cytoplasm to vacuole transport by the Cvt pathway, endoplasmic reticulum to Golgi vesicle-mediated transport, intra-Golgi vesicle-mediated transport, macroautophagy
PIS48740.1	25.4252591	25.0267773	24.8119775	26.28643682	26.06634878	26.09488876	0.049064109	0.926	1.06	PIS48740.1	HAT2	Putative Hat1-Hat2 histone acetyltransferase complex subunit; role in DNA damage repair and morphogenesis; mutations cause constitutive pseudohyphal growth, caspofungin sensitivity; rat catheter and Spider biofilm repressed
PIS55793.1	30.6361277	30.702613	30.3690008	32.1136344	31.27103029	31.50167903	0.006155636	0.813	1.06	PIS55793.1	MLT1	Vacuolar membrane transporter; MRP subfamily of ABC family; may transport organic anions conjugated to glutathione, glucuronate, or sulfate; needed for virulence in mouse peritonitis; Spider biofilm induced; flow model biofilm repressed
PIS58436.1	28.9547935	28.163973	26.8598092	29.58302019	29.30117502	28.28207111	0.050904058	0.927	1.06	PIS58436.1	NOG2	Putative nucleolar GTPase; repressed by prostaglandins; Hap43-induced, rat catheter and Spider biofilm induced

PIS55696.1	29.435526	28.8163831	29.1977336	30.15641705	29.97817188	30.48123875	0.142458833	0.953	1.06	PIS55696.1	orf19.3136	Ortholog(s) have transcription coactivator activity and role in carbon catabolite regulation of transcription from RNA polymerase II promoter
PIS58070.1	25.8456531	24.1236105	21.2873012	22.91523129	25.66909241	25.8592981	0.449260032	0.971	1.06	PIS58070.1	orf19.5534	Protein with a predicted role in mitotic spindle elongation, vesicle-mediated transport; flow model biofilm induced
PIS58934.1	28.8766317	28.0652022	28.1091183	29.30333931	29.42742488	29.50222738	0.009644785	0.848	1.06	PIS58934.1	orf19.7069	Putative AdoMet-dependent proline methyltransferase; Hap43-induced; required for normal flow model biofilm growth; Spider biofilm repressed
PIS54628.1	25.1124716	24.3736491	23.0365353	25.37840158	25.0998289	25.22508648	0.130485304	0.951	1.06	PIS54628.1	orf19.7345	Ortholog(s) have ATP binding activity
PIS50580.1	29.8884346	29.4048107	28.8478291	31.07042661	30.06410782	30.18854972	0.02322047	0.898	1.06	PIS50580.1	PCT1	Putative choline-phosphate cytidylyl transferase, antigenic during human oral infection
PIS58690.1	23.939576	24.8753299	25.8611522	25.71153985	25.91227988	26.26873438	0.139799722	0.952	1.07	PIS58690.1	orf19.2110	Ortholog(s) have arginyl-tRNA--protein transferase activity and role in protein arginylation
PIS58794.1	23.7577724	25.6287198	25.219059	25.43621976	25.98844967	26.38062929	0.028193731	0.906	1.07	PIS58794.1	orf19.2318.1	Ortholog(s) have inositol phosphoceramide synthase regulator activity, role in inositol phosphoceramide metabolic process and Golgi membrane, inositol phosphoceramide synthase complex localization

PIS54696.1	23.4426754	24.083308	23.6733465	24.42382348	26.6965196	23.27448298	0.113624724	0.948	1.07	PIS54696.1	orf19.2521	Ortholog of C. dubliniensis CD36 : Cd36_26390, Debaryomyces hansenii CBS767 : DEHA2C05302g, Pichia stipitis Pignal : PICST_68531 and Candida tropicalis NEW ASSEMBLY : CTRG1_00947
PIS51165.1	29.7341159	29.1919239	28.3983594	30.02264504	30.52223728	29.98250862	0.010643279	0.855	1.07	PIS51165.1	orf19.4850	Ortholog of S. cerevisiae : COB1, C. dubliniensis CD36 : Cd36_09270, C. parapsilosis CDC317 : CPAR2_803450, C. auris B8441 : B9J08_002739 and Candida tenuis NRRL Y-1498 : CANTEDRAFT_131760
PIS56574.1	28.9359114	28.709051	28.3039607	30.08045681	29.66260818	29.40742148	0.014015882	0.872	1.07	PIS56574.1	orf19.6245	Protein of unknown function; regulated by osmotic stress via Hog1 and oxidative stress (Hog1- and Cap1-independent); induced by alpha pheromone in SpiderM medium; Spider biofilm induced
PIS48762.1	27.3002781	27.5564322	27.2902928	28.33694055	28.20382706	28.82248857	0.105962723	0.946	1.07	PIS48762.1	orf19.6461	Ortholog(s) have unfolded protein binding activity, role in mitochondrial cytochrome c oxidase assembly, negative regulation of programmed cell death and mitochondrial inner membrane localization
PIS58769.1	29.8947443	28.8654086	27.4575071	30.4996888	29.99377753	28.94781631	0.093340402	0.944	1.07	PIS58769.1	PEL1	Predicted enzyme of mitochondrial phospholipid biosynthesis; rat catheter and flow model biofilm induced
PIS51655.1	24.4512964	24.0408893	23.8307598	25.29220841	25.00785624	25.26491055	0.058159365	0.931	1.08	PIS51655.1	orf19.2988	Predicted aminotransferase based on S. pombe ortholog SPBC660.12c; flow model biofilm induced
PIS48596.1	24.7523658	24.0917241	23.9225178	25.89780991	24.85061068	25.25517175	0.125817382	0.95	1.08	PIS48596.1	orf19.4043	Protein with a predicted pleckstrin homology domain; induced by alpha pheromone in SpiderM medium

PIS52068.1	23.7861981	24.575515	23.6426105	25.07679396	25.13445518	25.03742529	0.040423953	0.92	1.08	PIS52068.1	orf19.6255	Putative protein of unknown function; Hap43p-repressed gene
PIS48794.1	27.5836703	27.0538084	26.8975897	28.3058116	28.22234961	28.23524876	0.085407753	0.941	1.08	PIS48794.1	orf19.684	Putative transcription factor with zinc finger DNA-binding motif; heterozygous null mutant exhibits hypersensitivity to parnafungin and cordycepin in the C. albicans fitness test
PIS58592.1	28.8625951	28.6170229	28.7702173	28.75904193	30.27998066	30.44371496	0.074706732	0.938	1.08	PIS58592.1	orf19.7409.1	Ortholog(s) have structural constituent of ribosome activity and mitochondrial large ribosomal subunit localization
PIS51947.1	27.4153458	28.2563588	28.7744473	29.25324071	28.97354271	29.45001583	0.010346031	0.853	1.08	PIS51947.1	orf19.998	Putative adapter protein; links synaptojanins Inp52 and Inp53 to the cortical actin cytoskeleton in S. cerevisiae; mutants are viable
PIS48325.1	27.9179869	26.6251106	28.0834884	29.31101294	28.47536058	28.08429405	0.06087301	0.933	1.08	PIS48325.1	RPF2	Putative pre-rRNA processing protein; Hap43p-induced gene; mutation confers hypersensitivity to 5-fluorocytosine (5-FC), 5-fluorouracil (5-FU), and tubercidin (7-deazaadenosine)
PIS52170.1	26.6659264	26.4712218	26.4971215	28.47439599	27.15634046	27.23738776	0.019309712	0.889	1.08	PIS52170.1	TRP4	Predicted enzyme of amino acid biosynthesis; upregulated in biofilm; regulated by Gcn2p and Gcn4p; S. cerevisiae ortholog is Gcn4p regulated
PIS58211.1	25.7593948	26.078434	27.3826042	27.09973442	27.75205504	27.64253309	0.037907999	0.917	1.09	PIS58211.1	FRE10	Major cell-surface ferric reductase under low-iron conditions; 7 transmembrane regions and a secretion signal predicted; Tup1, Rim101, Ssn6, Hog1, caspofungin repressed; ciclopirox olamine induced; rat catheter biofilm induced

PIS51290.1	28.3107766	28.1159584	26.2821175	29.32148792	28.65445093	28.00125697	0.082812736	0.941	1.09	PIS51290.1	orf19.5395	Ortholog(s) have role in early endosome to late endosome transport, regulation of protein-containing complex assembly, vacuolar acidification and RAVE complex, cytoplasm localization
PIS51495.1	25.0470305	23.5342022	23.311038	26.42809565	24.5955171	24.12592644	0.168356582	0.956	1.09	PIS51495.1	orf19.5837	Ortholog(s) have 4-amino-4-deoxychorismate lyase activity and role in folic acid biosynthetic process
PIS52171.1	26.4089688	26.0490446	26.3184383	26.68101055	27.05454542	28.34145272	0.074553277	0.938	1.1	PIS52171.1	orf19.3098	Predicted RNA-dependent ATPase RNA helicase; Hap43-induced gene
PIS55512.1	28.0090266	27.0091716	27.2398041	28.80078659	28.22028522	28.54608956	0.027393592	0.905	1.1	PIS55512.1	orf19.4423	Putative glucosyltransferase; localized to the mitochondrial membrane
PIS52478.1	26.6544484	25.9939467	26.0410538	28.26087756	27.04782788	26.6873792	0.052044039	0.928	1.1	PIS52478.1	PAN6	Ortholog(s) have pantoate-beta-alanine ligase activity and role in pantothenate biosynthetic process
PIS50515.1	31.7688601	31.4514754	30.8993132	32.71501999	32.38667861	32.31935186	0.009351533	0.846	1.1	PIS50515.1	VTC4	Putative polyphosphate synthetase; decreased expression in hyphae compared to yeast-form cells; fungal-specific (no human or murine homolog); virulence-group-correlated expression
PIS54528.1	23.8151229	25.3636857	25.2514169	26.43391135	25.86085412	25.47662324	0.110406081	0.947	1.11	PIS54528.1	MSO1	Putative secretory protein involved in S. cerevisiae sporulation; repressed during pseudohyphal growth in the presence of lysed macrophages; Hap43-repressed; Spider biofilm induced

PIS56885.1	28.07327	27.7840661	27.7245265	30.29450289	28.98127742	27.63525622	0.059160722	0.932	1.11	PIS56885.1	orf19.5543	Has domain(s) with predicted role in intracellular protein transport, vesicle-mediated transport
PIS54706.1	27.1419676	27.6839184	27.3978647	28.31039582	30.13334366	27.15285488	0.081913576	0.94	1.12	PIS54706.1	LEM3	Putative membrane protein; mutation increases resistance to miltefosine; early-stage flow model biofilm induced
PIS51171.1	26.6529946	27.4824583	27.3356935	27.91944563	28.31476118	28.58485907	0.006812623	0.822	1.12	PIS51171.1	SHY1	Cytochrome c oxidase biosynthesis protein; transcript regulated by Nrg1 and Mig1
PIS50340.1	24.8567156	25.0420483	26.3474915	26.35286754	27.94654855	25.32686829	0.1688773	0.956	1.13	PIS50340.1	DTD2	Ortholog(s) have D-leucyl-tRNA(Leu) deacylase activity, D-tyrosyl-tRNA(Tyr) deacylase activity and role in D-leucine catabolic process, D-tyrosine catabolic process, tRNA metabolic process
PIS52038.1	27.8046748	28.438533	28.1129748	29.39317238	29.16794782	29.19192765	0.005342453	0.801	1.13	PIS52038.1	ENT3	Putative epsin; induced during the mating process; transcript is upregulated in an RHE model of oral candidiasis
PIS55494.1	25.1270978	23.510488	24.5826073	25.6025253	26.12539798	24.87735196	0.064841478	0.934	1.13	PIS55494.1	HAP2	CCAAT-binding transcription factor; regulates low-iron induction of FRP1; in these conditions CBF comprises Hap43 and probably Hap2 and Hap3; possibly essential, disruptants not obtained by UAU1 method; Cap1-dependent induction in low iron
PIS56697.1	25.2866163	24.1480516	24.1880157	25.61548909	26.06826661	25.32019906	0.027981576	0.906	1.13	PIS56697.1	orf19.1136	Ortholog(s) have AP-2 adaptor complex, clathrin-coated vesicle localization

PIS51368.1	31.8831052	31.01414	31.0881315	32.54069431	32.11396652	32.71262317	0.003790636	0.767	1.13	PIS51368.1	orf19.185	Ortholog(s) have structural constituent of ribosome activity, role in mitochondrial translation, mitochondrial translational initiation and mitochondrial small ribosomal subunit localization
PIS51208.1	26.1374083	25.3122493	25.554456	27.00475016	26.73028184	26.67281531	0.003172618	0.747	1.13	PIS51208.1	orf19.2284	Protein with an FMN-binding domain; Hap43-repressed; flow model biofilm induced
PIS56913.1	21.3147228	23.3680759	22.2416241	23.73418184	23.94893569	22.63405074	0.09154326	0.943	1.13	PIS56913.1	orf19.3204	Ortholog of C. dubliniensis CD36 : Cd36_51610, C. parapsilosis CDC317 : CPAR2_303630, C. auris B8441 : B9J08_001459 and Candida tenuis NRRL Y-1498 : CANTEDRAFT_135125
PIS48672.1	23.8212825	23.824589	26.306201	26.72769766	25.56339884	25.04872328	0.147150714	0.953	1.13	PIS48672.1	orf19.3393	Putative DEAD-box helicase; Hap43-induced; Spider biofilm induced
PIS50556.1	23.2096439	25.3717534	25.0280369	24.64632707	27.82527827	24.521515	0.373316361	0.968	1.13	PIS50556.1	orf19.4258	Puttaive pre-mRNA polyadenylation factor; heterozygous null mutant exhibits hypersensitivity to parnafungin and cordycepin in the C. albicans fitness test
PIS58273.1	25.0837832	26.168125	27.2362562	26.4477861	27.80991213	27.62995363	0.236736914	0.962	1.13	PIS58273.1	orf19.4656	Ortholog of C. dubliniensis CD36 : Cd36_41300, C. parapsilosis CDC317 : CPAR2_400390, C. auris B8441 : B9J08_000767 and Candida tenuis NRRL Y-1498 : CANTEDRAFT_94704
PIS56769.1	25.0740664	25.623011	24.5259641	26.39142247	27.06138843	25.16137534	0.210198659	0.96	1.13	PIS56769.1	orf19.954	Putative DnaJ-like chaperone; Hap43-repressed gene

PIS52121.1	29.2283783	28.7719416	28.3687819	29.80353545	29.77653519	30.17357356	0.002668253	0.726	1.13	PIS52121.1	RIM11	Ortholog of S. cerevisiae Rim11; a protein kinase involved in meiosis and sporulation in S. cerevisiae; mutant is viable
PIS48142.1	27.7042471	26.4948911	26.2951025	27.6787466	27.73858182	28.45640698	0.041489053	0.921	1.13	PIS48142.1	SHA3	Putative ser/thr kinase involved in glucose transport; Tn mutation affects filamentous growth; fluconazole-induced; ketoconazole-repressed; induced in by alpha pheromone in SpiderM; possibly essential; flow model biofilm induced
PIS58924.1	25.932736	25.0882017	25.74832	26.62225872	26.89546227	26.65356501	0.042102281	0.921	1.13	PIS58924.1	SHP1	Regulator of the type 1 protein phosphatase Glc7p activity, involved in control of morphogenesis, progression through the cell cycle and response to DNA damage
PIS51726.1	29.666113	29.6765229	28.46023	30.63277036	30.38195481	30.18714308	0.022220316	0.896	1.13	PIS51726.1	YOR1	Protein similar to S. cerevisiae Yor1; ABC-type plasma membrane transporter involved in resistance to aureobasidin A; white cell type-specific transcript; Spider biofilm induced
PIS58371.1	28.9037323	28.8920123	26.2055541	29.61128506	28.58859471	29.22845472	0.085356365	0.941	1.14	PIS58371.1	CHS5	Putative chitin biosynthesis protein; fungal-specific; repressed upon yeast-to-hypha switch; rat catheter biofilm repressed
PIS51987.1	29.4555092	29.2117038	29.61558	30.46665673	30.69280652	30.55425508	0.010082237	0.851	1.14	PIS51987.1	CSH3	Functional homolog of S. cerevisiae Shr3p, which is a chaperone specific for amino acid permeases; localized to ER; required for wild-type amino-acid responsive hyphal growth and for mouse systemic virulence; regulated by Gcn2p and Gcn4p
PIS51972.1	29.107497	30.2628955	26.7651368	29.67164992	30.05210585	29.82216032	0.173022902	0.956	1.14	PIS51972.1	GYP5	Putative Rab GTPase activator; role in ER to Golgi vesicle-mediated transport; Spider biofilm induced

PIS49559.1	31.9925915	31.430988	30.9044888	32.16361539	32.65369833	32.92827584	0.012305146	0.864	1.14	PIS49559.1	orf19.2244	Similar to oxidoreductases and to S. cerevisiae Yjr096wp; Sfu1 repressed; induced by benomyl treatment, Ssr1; Hap43-repressed; flow model biofilm repressed
PIS55071.1	25.9427781	25.6469536	26.2102655	29.79855042	26.51237056	24.9209118	0.398503213	0.969	1.14	PIS55071.1	orf19.6147	Putative histone chaperone; role in chromatin remodeling; rat catheter and Spider biofilm repressed
PIS58529.1	26.9757117	26.4296896	23.5460114	27.41298404	26.68856364	26.28159986	0.117236628	0.949	1.14	PIS58529.1	orf19.6455	Protein of unknown function, transcript is upregulated in clinical isolates from HIV+ patients with oral candidiasis
PIS54576.1	25.5100163	25.7208291	25.1805811	26.72804137	26.68556851	26.41933314	0.037630723	0.917	1.14	PIS54576.1	orf19.6929	Ortholog(s) have acid phosphatase activity, protein tyrosine phosphatase activity, metal-dependent activity
PIS54666.1	27.1671725	25.8639168	25.7716381	27.75186067	26.88939754	27.56942435	0.035556102	0.915	1.14	PIS54666.1	orf19.6980	Ortholog(s) have cell periphery, cellular bud neck localization
PIS52260.1	25.4535469	25.4220999	23.3139375	25.26846762	26.26008355	26.12173712	0.416145437	0.97	1.15	PIS52260.1	HIS7	Putative imidazole glycerol phosphate synthase; histidine biosynthesis; no human/murine homolog; transcription induced by histidine starvation; regulated by Gcn2p and Gcn4p; higher protein level in stationary phase
PIS51026.1	26.8327899	23.3824014	27.0723443	27.68074591	27.50587065	25.56317571	0.201862	0.959	1.15	PIS51026.1	SCW4	Putative cell wall protein; substrate for Kex2p processing in vitro; expression regulated by white-opaque switch; alkaline repressed; possibly essential (UAU1 method); flow model biofilm induced; Spider biofilm induced

PIS52246.1	29.5807755	29.4960216	29.35905	32.27396552	29.74088213	29.90040545	0.049604231	0.926	1.16	PIS52246.1	APT1	Adenine phosphoribosyltransferase; flucytosine induced; repressed by nitric oxide; protein level decreased in stationary phase yeast cultures
PIS49534.1	24.7972844	24.925347	22.6273952	25.82346248	24.68156084	25.33138078	0.060516119	0.932	1.16	PIS49534.1	orf19.4728	Ortholog(s) have role in chromatin remodeling and Set3 complex localization
PIS51506.1	24.5542747	23.7039386	24.3901936	25.56590798	25.72593026	24.82396089	0.01362025	0.87	1.16	PIS51506.1	orf19.5991	Ortholog(s) have role in assembly of large subunit precursor of preribosome, maturation of 5.8S rRNA from tricistronic rRNA transcript (SSU-rRNA, 5.8S rRNA and LSU-rRNA), more
PIS51790.1	27.6382105	27.0570465	26.419389	28.59566808	28.38588591	27.60413454	0.027528207	0.905	1.16	PIS51790.1	orf19.6225.1	Ortholog(s) have role in mitochondrial cytochrome c oxidase assembly
PIS51160.1	26.3086096	25.0272851	24.0258385	25.75198463	26.70057267	26.37803847	0.087107141	0.942	1.16	PIS51160.1	orf19.6329	Protein of unknown function; opaque-specific transcript; fluconazole-repressed; induced in cyr1 mutant and in oralpharyngeal candidasis; Spider biofilm induced
PIS49697.1	23.7312636	23.4523021	24.4079665	27.07211829	24.34513317	23.65621655	0.186537036	0.958	1.16	PIS49697.1	orf19.7199	Ortholog(s) have role in post-translational protein targeting to endoplasmic reticulum membrane and TRC complex, cytoplasm localization
PIS52037.1	23.1237109	24.4315034	24.0896871	25.79728962	23.76922363	25.5651864	0.377632318	0.968	1.16	PIS52037.1	SAC3	Putative nuclear pore-associated protein, required for small ribosomal subunit biogenesis; possibly an essential gene, disruptants not obtained by UAU1 method

PIS52346.1	26.5987659	27.7619131	25.5767782	29.62403059	27.12003371	26.67001789	0.155766747	0.954	1.16	PIS52346.1	SPA2	Protein involved in cell polarity, Spitzenkorper formation; required for mouse virulence; localizes to hyphal tip; cell-cycle regulated localization in yeast-form cells; functional domains conserved with S. cerevisiae ; Hap43p-induced gene
PIS58715.1	24.4577244	23.7585885	24.1673941	25.35772595	25.07241391	25.44378199	0.019586465	0.89	1.16	PIS58715.1	THG1	tRNA guanylyltransferase, with role in tRNA modification; catalyzes reverse (3'-5') nucleotide polymerization
PIS49817.1	25.2813292	24.8095852	25.38387	26.14001281	26.25080447	26.59280881	0.008313712	0.838	1.17	PIS49817.1	BUR2	Protein with similarity to S. cerevisiae Bur2p, contains a cyclin domain; not required for wild-type hyphal growth, adherence to buccal epithelial cells, or virulence in mouse systemic infection
PIS54568.1	22.8629364	22.9875514	23.8912869	24.88405696	23.8869344	24.49458063	0.102292168	0.946	1.17	PIS54568.1	MBP1	Putative component of the MBF transcription complex involved in G1/S cell-cycle progression; non-periodic mRNA expression; predicted, conserved MBF binding sites upstream of G1/S-regulated genes
PIS51522.1	26.544586	25.46504	24.5817038	25.42382928	27.50785756	27.16257489	0.074667	0.938	1.17	PIS51522.1	orf19.1285	Plasma membrane-localized protein of unknown function; Hap43p-repressed gene
PIS52269.1	29.6509919	28.0567058	25.1138303	30.13988722	29.07980811	27.11206314	0.283002214	0.964	1.17	PIS52269.1	orf19.4059	Ortholog(s) have role in arginine transmembrane transport, lysine transport, regulation of intracellular pH and cell division site, cell tip, cytoplasm, fungal-type vacuole, fungal-type vacuole membrane localization
PIS58120.1	27.6730446	27.0742061	27.889087	28.68959267	28.56242149	28.88714192	0.017849381	0.885	1.17	PIS58120.1	orf19.6264	Ortholog of C. dubliniensis CD36 : Cd36_06080, C. parapsilosis CDC317 : CPAR2_803360, C. auris B8441 : B9J08_000608 and Candida tenuis NRRL Y-1498 : CANTEDRAFT_112428

PIS58605.1	25.1640362	25.8181678	26.5059469	27.60218212	27.2056211	26.21790053	0.053702574	0.929	1.18	PIS58605.1	MGM101	Putative mitochondrial genome maintenance protein; fungal-specific (no human or murine homolog); mutation confers hypersensitivity to tubercidin (7-deazaadenosine)
PIS51162.1	24.0592095	26.3136974	24.3035405	25.47491703	27.01872536	25.72613413	0.215039293	0.96	1.18	PIS51162.1	orf19.2131	Ortholog(s) have ubiquitin protein ligase activity, ubiquitin-protein transferase activity
PIS52135.1	27.1627803	25.4588963	26.0411213	28.10313447	27.45290177	26.63586795	0.236998989	0.962	1.18	PIS52135.1	orf19.3758	Ortholog(s) have fungal-type vacuole membrane, membrane raft localization
PIS58041.1	28.5424469	27.3054659	27.7443637	30.71027279	28.70551523	27.71104214	0.140768936	0.952	1.18	PIS58041.1	orf19.4307	Ortholog(s) have role in ESCRT III complex assembly, late endosome to vacuole transport, late endosome to vacuole transport via multivesicular body sorting pathway, protein targeting to vacuole and cytoplasm, late endosome localization
PIS51786.1	26.0176315	27.0466709	27.0231461	28.360904	27.49269319	27.76985751	0.018945578	0.888	1.18	PIS51786.1	orf19.5201.1	Has domain(s) with predicted ATPase inhibitor activity, role in negative regulation of ATP-dependent activity and mitochondrion localization
PIS54995.1	26.6211306	27.6352883	27.2212978	29.58483305	28.03588114	27.3843717	0.041072	0.92	1.18	PIS54995.1	orf19.6477	Ortholog(s) have enzyme activator activity, role in tRNA (guanine-N7)-methylation and cytosol, nucleus, tRNA (m7G46) methyltransferase complex localization
PIS51540.1	24.6772195	24.6571944	24.5684796	26.29300879	25.26908246	25.86653284	0.068367275	0.936	1.18	PIS51540.1	RPB4	Protein similar to S. cerevisiae Rpb4p, which is a component of RNA polymerase II; transposon mutation affects filamentous growth

PIS55599.1	26.2617102	26.2645645	27.0720969	27.1985813	27.38063054	28.59203598	0.099444528	0.945	1.19	PIS55599.1	BUD5	Predicted GTP/GDP exchange factor for Rsr1; rat catheter biofilm induced
PIS54675.1	24.4919209	25.2240611	25.7123629	27.43476069	25.37079462	26.20095472	0.041244216	0.92	1.19	PIS54675.1	HYM1	Protein of RAM cell wall integrity signaling network; involved in regulation of Ace2 activity and cellular morphogenesis; role in cell separation, azole sensitivity; required for hyphal growth
PIS58975.1	24.6742884	24.6086175	24.7476801	25.92371727	25.81775426	25.86682812	0.046968926	0.925	1.19	PIS58975.1	orf19.7095	Ortholog(s) have fluoride transmembrane transporter activity, role in cellular detoxification of fluoride, fluoride export across plasma membrane, fluoride transmembrane transport and plasma membrane localization
PIS51450.1	26.4084063	26.1351793	27.5072749	27.89066671	27.48085881	28.24173027	0.009626984	0.848	1.19	PIS51450.1	PES1	Pescadillo homolog required for dispersal of biofilm cells into planktonic yeast cells; essential in yeast cells, not in hyphal cells; mutation confers hypersensitivity to 5-fluorocytosine, 5-fluorouracil, tubercidin
PIS55532.1	25.9401357	25.0959528	24.1761527	26.13917266	26.79381644	25.85423783	0.034397682	0.914	1.19	PIS55532.1	SEC20	Essential protein; similar to S. cerevisiae Sec20p; depletion causes membrane accumulation and drug sensitivity; expression regulated by growth phase; O-mannosylation regulates proteolysis; does not complement S. cerevisiae sec20-1
PIS55575.1	28.0841471	27.4330667	27.4746252	29.55573359	28.48808348	28.55352991	0.02749202	0.905	1.2	PIS55575.1	CPH2	Myc-bHLH transcription factor; promotes hyphal growth; directly regulates Tec1 to induce hypha-specific genes; probably homodimeric, phosphorylated; required for colonization of the mouse GI tract; rat catheter and Spider biofilm induced
PIS55505.1	22.9691161	23.3071331	23.0849989	23.68240335	24.24126848	25.02854258	0.098391939	0.945	1.2	PIS55505.1	GCS1	Gamma-glutamylcysteine synthetase; glutathione synthesis, required for virulence;induced in low iron, H2O2, Cd, or presence of human neutrophils; possibly adherence-induced; Spider and F-12/CO2 biofilm induced

PIS58358.1	32.9399575	31.2355368	29.4316643	32.01542974	32.95178468	32.25203345	0.348138625	0.967	1.2	PIS58358.1	HHO1	Putative histone H1; farnesol regulated; Hap43-induced; contains 5' UTR intron; Spider biofilm repressed
PIS52334.1	25.9101829	23.4853098	25.3412845	27.90874596	25.25616837	25.18559801	0.108438036	0.947	1.2	PIS52334.1	KIS2	Scaffold protein of Snf1p complex; similar to S. cerevisiae Gal83p and Sip2p; interacts with Snf4p; interaction with Snf1p complex is regulated by carbon source, decreased on ethanol; N-terminal myristoylation; Hog1p-downregulated
PIS54682.1	22.6138838	23.5308238	23.7977745	25.54656982	23.66892197	24.33049384	0.048837186	0.926	1.2	PIS54682.1	orf19.3226	Ortholog(s) have phosphatidylcholine binding, phosphatidylinositol binding, phosphatidylserine binding, sterol binding activity, role in intracellular sterol transport, sterol transport and fungal-type vacuole lumen localization
PIS50578.1	23.7583981	23.8275641	23.7606317	25.69521657	24.60002464	24.66212521	0.04801117	0.925	1.2	PIS50578.1	orf19.4252	Ortholog(s) have ATPase, protein serine/threonine kinase activity
PIS49663.1	25.3344823	24.7675597	24.3169806	26.26375901	26.31541918	25.44946103	0.057235403	0.931	1.2	PIS49663.1	orf19.527	Protein of unknown function; flow model biofilm induced
PIS50390.1	28.9398185	28.6363555	28.2235336	29.75645509	30.00229124	29.64899519	0.003309124	0.752	1.2	PIS50390.1	orf19.5296	Ortholog(s) have role in mitochondrial cytochrome c oxidase assembly and mitochondrial inner membrane, mitochondrial membrane localization
PIS56742.1	28.4197871	25.3013857	25.4811533	27.73351872	27.56780689	27.49086207	0.103698262	0.946	1.2	PIS56742.1	orf19.6625	NAP1 family histone chaperone

PIS50478.1	23.2004202	24.1387753	25.1315225	27.6863672	23.583781	24.80212782	0.17941142	0.957	1.2	PIS50478.1	PPR1	Transcription factor with zinc cluster DNA-binding motif involved in regulation of purine catabolism; has similarity to S. cerevisiae Ppr1p, which is a transcription factor involved in the regulation of uracil biosynthesis genes
PIS51646.1	26.8394155	26.847699	26.0971691	28.90084395	27.31874684	27.17085978	0.097834845	0.945	1.2	PIS51646.1	SNF7	ESCRT III complex protein; role in proteolytic activation of Rim101 and Rim8 processing/activation; separable roles in RIM101 pathway and in transport from MVB to vacuole; involved in echinocandin and azole sensitivity
PIS58142.1	28.0858341	27.7573893	29.1441478	30.05041941	29.00622886	29.56587972	0.166558213	0.956	1.21	PIS58142.1	orf19.3083	Putative lipid phosphatase of the endoplasmic reticulum; role in DNA repair, actin cytoskeleton organization, cellular manganese ion homeostasis; Spider biofilm repressed
PIS51835.1	25.2648817	24.6735995	23.78688	27.21350213	25.06581567	25.08258624	0.104963198	0.946	1.21	PIS51835.1	orf19.6581	Ortholog(s) have palmitoyltransferase activity, role in protein palmitoylation and endoplasmic reticulum localization
PIS51385.1	30.4273428	29.8171283	29.542681	31.26144891	31.00469901	31.1382005	0.012122871	0.863	1.21	PIS51385.1	orf19.7215	Nucleolar protein; component of the small subunit processome containing the U3 snoRNA; involved in pre-18S rRNA processing; flow model biofilm repressed
PIS54973.1	25.2890029	23.0171849	23.7174186	26.49928727	23.79634096	25.36854746	0.113686333	0.948	1.21	PIS54973.1	PNC1	Putative nicotinamidase, involved in NAD salvage pathway; decreased transcription is observed in an azole-resistant strain that overexpresses MDR1
PIS56964.1	27.1933037	27.1931213	25.3876643	27.50743618	28.1931973	27.69987664	0.03605626	0.916	1.21	PIS56964.1	SYS1	Putative Golgi integral membrane protein; transcript regulated by Mig1

PIS58681.1	25.7908287	25.4603029	24.7226446	26.1979443	26.44515158	26.98966251	0.069608844	0.936	1.22	PIS58681.1	orf19.4676	Protein with homology to mitochondrial intermembrane space proteins; regulated by Sef1p-, Sfu1p-, and Hap43p
PIS48481.1	27.2163025	26.573319	27.1001878	28.02073694	28.4753338	28.04909361	0.014934532	0.875	1.22	PIS48481.1	PHO86	Putative endoplasmic reticulum protein; possibly adherence-induced
PIS48226.1	21.2432023	18.3211302	22.3955958	23.24112336	20.92589405	21.4871729	0.380098092	0.968	1.23	PIS48226.1	CDC23	Similar to anaphase-promoting complex component; possibly transcriptionally regulated by Tac1p
PIS51912.1	23.9155861	24.1635088	23.9858293	25.74230139	24.743496	25.2819151	0.014215368	0.873	1.23	PIS51912.1	CNH1	Na+/H+ antiporter; required for wild-type growth, cell morphology, and virulence in a mouse model of systemic infection; not transcriptionally regulated by NaCl; fungal-specific (no human or murine homolog)
PIS51908.1	27.055651	27.148523	28.2217092	28.41874802	28.94601971	28.7479931	0.012256124	0.864	1.23	PIS51908.1	MET15	O-acetylhomoserine O-acetylserine sulfhydrylase; sulfur amino acid synthesis; immunogenic; Hog1, adherence-induced; brown color of mutant in Pb(2+) medium a visual selection; chlamydospore formation induced, F-12/CO2 biofilm induced
PIS56598.1	25.7077239	24.6093969	27.0721311	28.36097787	26.80854311	25.92408489	0.150294527	0.954	1.23	PIS56598.1	orf19.2286	Putative deoxyhypusine hydroxylase; ketoconazole-induced; protein level decreases in stationary phase cultures; required for biofilm formation; Spider biofilm repressed
PIS52415.1	27.508352	23.4875648	25.9567856	26.79547418	26.72227482	27.11856425	0.1636533	0.955	1.23	PIS52415.1	orf19.4893	Ortholog of C. dubliniensis CD36 : Cd36_09630, C. parapsilosis CDC317 : CPAR2_805080, C. auris B8441 : B9J08_004031 and Candida tenuis NRRL Y-1498 : CANTEDRAFT_136811

PIS55460.1	26.8139253	26.6363976	23.9993372	26.917785	27.20172389	27.00604099	0.132267474	0.951	1.23	PIS55460.1	orf19.6527	Pheromone-regulated protein (Prm10) of S. cerevisiae; colony morphology-related gene regulation by Ssn6; induced by Mnl1 under weak acid stress; possibly essential gene, disruptants not obtained by UAU1 method; Spider biofilm induced
PIS51363.1	26.1214925	25.1642257	25.0398955	25.90083745	27.45627267	26.66062914	0.026234627	0.903	1.23	PIS51363.1	orf19.7100	Putative vacuolar transporter of large neutral amino acids; possibly transcriptionally regulated upon hyphal formation; not the true Avt1 ortholog of S. cerevisiae
PIS55559.1	26.1369263	25.2509192	25.3629962	27.51356747	25.92931404	27.00309831	0.154062679	0.954	1.23	PIS55559.1	orf19.7341	Protein of unknown function; flow model biofilm induced; ketoconazole-repressed
PIS55826.1	27.4178959	27.854463	28.4229001	29.01404735	28.91259333	29.49676208	0.155504548	0.954	1.24	PIS55826.1	ASR1	Heat shock protein; transcript regulated by cAMP, osmotic stress, ciclopirox olamine, ketoconazole; repressed by Cyr1, Ras1; colony morphology-related regulated by Ssn6; stationary phase enriched; Hap43-induced; Spider biofilm induced
PIS51807.1	25.7368046	24.4665435	25.7156679	26.4418299	26.32928215	26.86471893	0.039537025	0.919	1.24	PIS51807.1	orf19.3304	Exosome non-catalytic core component; involved in 3'-5' RNA processing and degradation in the nucleus and cytoplasm; Spider biofilm induced
PIS51439.1	26.7010768	26.2407506	24.8365076	27.46395031	27.48695546	26.5386567	0.070785257	0.937	1.24	PIS51439.1	orf19.4780	Predicted MFS family membrane transporter, member of the drug:proton antiporter (12 spanner) (DHA1) family; Spider biofilm induced
PIS50375.1	27.0201186	24.6559331	27.2262583	27.47775512	27.21161111	27.92909338	0.129066493	0.951	1.24	PIS50375.1	VRP1	Verprolin-related protein involved in actin cytoskeleton organization and polarized morphogenesis; interacts with Wal1p and Myo5p; downregulated upon adherence to polystyrene

PIS58464.1	27.7946936	28.176432	27.2346961	28.91253673	28.7526693	29.28280721	0.004172394	0.777	1.25	PIS58464.1	MNN11	Ortholog(s) have alpha-1,6-mannosyltransferase activity, role in protein N-linked glycosylation, protein glycosylation and Golgi apparatus, mannan polymerase complex localization
PIS58766.1	30.6573207	30.1857793	30.691477	31.8058363	31.30817222	32.16907489	0.004099335	0.775	1.25	PIS58766.1	MRP7	Mitochondrial ribosomal protein of the large subunit; rat catheter biofilm induced
PIS50388.1	27.1997108	27.7544904	26.0333843	28.28396834	27.88833744	28.55261699	0.029727618	0.908	1.25	PIS50388.1	orf19.5300	Calnexin; integral membrane ER chaperone involved in folding and quality control of glycoproteins; caspofungin induced
PIS52336.1	23.7934466	19.4442682	23.8783752	24.05269982	23.34741141	23.47244691	0.490208428	0.972	1.25	PIS52336.1	TEC1	TEA/ATTS transcription factor; white cell pheromone response, hyphal gene regulation; required for Spider and RPMI biofilm formation; regulates BCR1; Cph2 regulated transcript; alkaline, rat catheter, Spider, flow model biofilm induced
PIS48608.1	25.7768665	25.3407792	24.7419437	27.0350291	26.45095922	26.14516588	0.009281947	0.846	1.26	PIS48608.1	orf19.2301	Ortholog(s) have role in proteasome regulatory particle assembly and cytoplasm, cytosol, nucleus localization
PIS56676.1	26.3745137	23.6446609	26.2940809	27.01147617	26.85660996	26.22806673	0.092194361	0.943	1.26	PIS56676.1	orf19.4110	Ortholog of C. dubliniensis CD36 : Cd36_20490, C. parapsilosis CDC317 : CPAR2_104180, C. auris B8441 : B9J08_001215 and Candida tenuis NRRL Y-1498 : CANTEDRAFT_116074
PIS55445.1	28.9114488	28.1135979	25.4458965	28.18633378	28.90316024	29.16063607	0.278773454	0.964	1.26	PIS55445.1	orf19.4240	Ortholog(s) have role in GPI anchor biosynthetic process, intracellular manganese ion homeostasis and endoplasmic reticulum, fungal-type vacuole membrane localization

PIS51619.1	28.3557884	29.5411835	29.4688148	29.82181379	30.55005109	30.7905509	0.004922513	0.793	1.27	PIS51619.1	orf19.1800	Protein of unknown function; Spider biofilm induced
PIS48603.1	19.0139101	25.4139218	25.7037002	24.38952013	24.46851313	25.08001267	0.39672003	0.969	1.27	PIS48603.1	orf19.2307	Ortholog(s) have role in co-transcriptional mRNA 3'-end processing, cleavage and polyadenylation pathway, mRNA processing and chromatin, mRNA cleavage and polyadenylation specificity factor complex, nucleolus
PIS51432.1	25.5544069	24.7287244	23.6954675	25.68618807	26.07002708	26.03100126	0.270479829	0.964	1.27	PIS51432.1	orf19.2857	Ortholog(s) have DNA helicase activity, DNA translocase activity, RNA polymerase II general transcription initiation factor activity
PIS54823.1	22.6607341	25.6176758	25.6662055	25.95027054	25.56485083	26.230336	0.167359294	0.956	1.27	PIS54823.1	orf19.6305	Hydroxytrimethyllysine aldolase, the second enzyme in the carnitine biosynthesis pathway; rat catheter biofilm repressed
PIS55030.1	26.2781692	23.8378622	26.7184842	26.64128809	27.19155809	26.79767723	0.071693227	0.937	1.27	PIS55030.1	SRD1	Sur7 family protein with PalI-like domains; not required for stress responses; mutant is viable
PIS50469.1	29.6987909	28.6145012	28.4208369	31.65245738	29.98985993	28.89863181	0.052552617	0.928	1.27	PIS50469.1	SRP101	Signal recognition particle (SRP) receptor alpha subunit; involved in SRP-dependent protein targeting; rat catheter biofilm repressed
PIS52258.1	24.146051	24.7260324	24.3794092	25.61994148	25.45952391	26.00105824	0.041827361	0.921	1.28	PIS52258.1	ENP1	Protein required for pre-rRNA processing and 40S ribosomal subunit synthesis; associated with U3 and U14 snoRNAs; transposon mutation affects filamentous growth; repressed by prostaglandins; Spider biofilm induced

PIS51060.1	26.3676217	25.2989293	26.2678399	27.04115199	26.79405386	27.93152672	0.083822237	0.941	1.28	PIS51060.1	orf19.1617	Protein similar to S. cerevisiae Ydr282cp; transposon mutation affects filamentous growth; Hap43p-repressed gene
PIS49546.1	27.3706798	25.8166796	26.2426919	27.10659041	27.95017912	28.22120277	0.008827941	0.842	1.28	PIS49546.1	orf19.2472.1	Ortholog of C. dubliniensis CD36 : Cd36_61610, C. parapsilosis CDC317 : CPAR2_107530, C. auris B8441 : B9J08_004570 and Candida tenuis NRRL Y-1498 : CANTEDRAFT_115252
PIS48579.1	25.8552314	25.9510006	25.8810405	27.2288606	26.85328522	27.44822187	0.01466628	0.874	1.28	PIS48579.1	orf19.5702	Ortholog(s) have role in diacylglycerol metabolic process, response to osmotic stress, vacuole fusion and vacuole-mitochondrion membrane contact site localization
PIS52126.1	30.3754693	30.1461097	30.5185848	31.84318731	31.55360878	31.51952889	0.001103433	0.597	1.29	PIS52126.1	ERG3	C-5 sterol desaturase; introduces C-5(6) double bond into episterol; some clinical isolates show increased azole resistance and defects in hyphal growth and virulence; Efg1p-repressed; fluconazole-induced
PIS48250.1	24.8991698	25.9652652	25.9416601	28.30251555	26.62463243	25.75587809	0.046603708	0.924	1.29	PIS48250.1	IQG1	Actomyosin ring component at bud neck; cell-cycle regulated ser phosphorylation at CDK sites regulate association with Bni1/Bnr1, Iqg1 degradation, and ring disassembly; mutation causes cytokinetic defects; [illegible]
PIS48725.1	27.2642444	26.3343563	26.0915013	27.854576	27.84994508	27.85641817	0.00304544	0.742	1.29	PIS48725.1	orf19.7153	Putative exportin, member of the Exportin-T family; flow model biofilm repressed
PIS59036.1	29.281081	29.8697545	27.2965299	30.39576262	29.93483991	30.00052005	0.05370839	0.929	1.29	PIS59036.1	PHM7	Putative transporter, possibly involved in ion homeostasis, drug tolerance, filamentous growth, virulence; fungal-specific; Hog1-repressed; repressed by 17-beta-estradiol, ethynyl estradiol; Hap43-induced; Spider biofilm induced

PIS56717.1	26.4643911	19.4472255	23.8307598	24.45828891	24.37296046	24.77691207	0.37411522	0.968	1.29	PIS56717.1	POP3	Putative RNase MRP and nuclear RNase P component; decreased repressed by prostaglandins; Spider biofilm induced
PIS58731.1	31.1336427	29.6347719	29.4532197	31.50052955	31.58650737	31.02179464	0.007557324	0.83	1.3	PIS58731.1	MET18	Putative protein with a predicted role in nucleotide excision repair (NER) and RNA polymerase II (RNAP II) transcription; Plc1p-regulated
PIS48444.1	26.5139507	24.6878282	24.9703246	26.76696408	27.19803742	26.10660414	0.127488061	0.95	1.3	PIS48444.1	orf19.1041	Ortholog(s) have cyclin-dependent protein serine/threonine kinase activator activity, cyclin-dependent protein serine/threonine kinase regulator activity
PIS58803.1	28.197751	27.1727571	26.6997205	28.48576879	28.50213888	28.99339318	0.0122565	0.864	1.3	PIS58803.1	orf19.2328	Ortholog of C. dubliniensis CD36 : Cd36_10220, C. parapsilosis CDC317 : CPAR2_210530, C. auris B8441 : B9J08_000256 and Candida tenuis NRRL Y-1498 : CANTEDRAFT_105553
PIS55662.1	24.8850454	24.5106217	22.3604514	25.07275814	25.39907596	25.17994543	0.339239425	0.967	1.3	PIS55662.1	orf19.2563	Ortholog(s) have U6 snRNA binding, snRNA binding activity, role in spliceosomal complex assembly, spliceosomal tri-snRNP complex assembly and U6 snRNP localization
PIS48779.1	28.3433987	27.6033817	26.765598	28.13280493	29.28116448	29.20585669	0.060287041	0.932	1.3	PIS48779.1	orf19.516	S. cerevisiae ortholog Rft1p has role in glycolipid translocation, protein N-linked glycosylation and localizes to endoplasmic reticulum membrane; regulated by Sef1p-, Sfu1p-, and Hap43p
PIS48717.1	22.8287335	24.1690844	24.4077016	24.53933793	24.84494654	25.92570522	0.017159923	0.883	1.3	PIS48717.1	orf19.7034	Putative eIF4E-associated protein;, accelerates mRNA degradation by promoting decapping; Spider biofilm repressed

PIS58486.1	28.8125513	28.5782426	28.1035225	30.02668226	29.33907045	30.01571533	0.025747608	0.902	1.3	PIS58486.1	orf19.7295	Ortholog(s) have protein phosphatase 1 binding activity, role in cortical actin cytoskeleton organization, positive regulation of clathrin-dependent endocytosis, protein secretion and actin cortical patch, nucleus localization
PIS51150.1	30.0626852	28.7338881	27.758164	30.43404433	29.83423794	30.19725887	0.060633168	0.932	1.3	PIS51150.1	RFC3	Putative heteropentameric replication factor C subunit; transcription is induced upon filamentous growth
PIS50530.1	21.768754	22.7725609	21.6484691	23.72008796	23.78529181	22.59955988	0.061372504	0.933	1.31	PIS50530.1	orf19.3916	Ortholog of C. dubliniensis CD36 : Cd36_53980, C. parapsilosis CDC317 : CPAR2_100980, C. auris B8441 : B9J08_004358 and Candida tenuis NRRL Y-1498 : CANTEDRAFT_116966
PIS55528.1	23.2435641	24.6892836	22.607227	24.662501	24.69962356	25.09872883	0.01375178	0.871	1.31	PIS55528.1	orf19.5532	Protein of unknown function; Spider biofilm induced
PIS51020.1	23.6925791	23.0217232	24.4688114	25.05684202	25.41680688	24.63841332	0.058242913	0.931	1.31	PIS51020.1	orf19.6075	Putative CCR4-Not complex transcription factor; ortholog of S. cerevisiae Cdc36; Hap43-repressed gene
PIS48448.1	26.9134684	27.2844096	26.4073756	28.42941024	27.77020479	28.35495368	0.19735936	0.959	1.32	PIS48448.1	ERB1	Protein with a predicted role in ribosomal large subunit biogenesis; mutation confers hypersensitivity to 5-fluorocytosine (5-FC), 5-fluorouracil (5-FU), and tubercidin (7-deazaadenosine); hyphal, macrophage repressed
PIS56844.1	28.5854562	28.572485	28.871384	30.34797738	29.40391389	30.23819965	0.003819712	0.768	1.32	PIS56844.1	ERG8	Putative phosphomevalonate kinase; enzyme of the mevalonate pathway of isoprenoid biosynthesis; transcript regulated by Nrg1; possible drug target; not conserved in H. sapiens

PIS52367.1	27.2528455	25.8285148	25.7324121	27.7720218	27.75930637	27.24455778	0.026764657	0.904	1.32	PIS52367.1	orf19.6152	Ortholog of C. dubliniensis CD36 : Cd36_32830, C. parapsilosis CDC317 : CPAR2_205930, C. auris B8441 : B9J08_003982 and Candida tenuis NRRL Y-1498 : CANTEDRAFT_115846
PIS51034.1	25.8276188	25.9851971	25.0211473	26.47718567	27.76975541	26.56149847	0.0305181	0.909	1.32	PIS51034.1	orf19.7624	Ortholog(s) have role in maturation of SSU-rRNA from tricistronic rRNA transcript (SSU-rRNA, 5.8S rRNA, LSU-rRNA), rRNA processing and nucleolus, small-subunit processome localization
PIS50351.1	26.5168422	25.1689752	24.0527839	26.6088033	26.37079243	26.72102695	0.024743288	0.901	1.32	PIS50351.1	RAD50	Putative DNA double-strand break repair factor; involved in response to oxidative stress and drug resistance; flow model biofilm repressed
PIS58248.1	29.0373797	28.9626146	29.1553789	30.06312058	30.19784221	30.83965733	0.000682483	0.512	1.32	PIS58248.1	TIM54	Predicted component of the mitochondrial TIM22 complex; involved in protein import into mitochondrial inner membrane; Spider biofilm repressed
PIS51401.1	25.2350208	22.9731662	24.8818638	26.25409164	25.39223695	25.42588894	0.096007706	0.944	1.33	PIS51401.1	KTI12	Protein similar to S. cerevisiae Kti12p, which associates with Elongator complex; has a role in resistance to killer toxin; predicted Kex2p substrate; Hap43p-induced gene
PIS52220.1	25.5272218	23.7813877	23.603558	24.86597168	25.2209082	26.81831617	0.160220722	0.955	1.33	PIS52220.1	MEF2	Putative mitochondrial translation elongation factor; caspofungin induced
PIS54982.1	23.0896521	22.3251925	24.1628381	24.55892518	24.48686838	24.5132412	0.085119093	0.941	1.33	PIS54982.1	orf19.4273	Putative mitochondrial membrane protein; ortholog of S. cerevisiae Sls1; coordinates expression of mitochondrially-encoded genes; Hap43-induced

PIS56886.1	32.0031884	31.6142524	30.9614171	33.0405017	32.68304454	32.83550751	0.005395694	0.802	1.33	PIS56886.1	SAC6	Fimbrin; actin filament bundling protein; involved in hyphal growth, oxidative stress response and virulence; transcript regulated by Nrg1 and Mig1; protein level decreases in stationary phase
PIS55676.1	28.6292176	28.6730993	27.7721964	28.93292421	30.72013	29.40857084	0.010296415	0.853	1.33	PIS55676.1	SEC6	Predicted subunit of the exocyst complex, involved in exocytosis; localizes to a crescent on the surface of the hyphal tip; Hap43p-repressed
PIS54741.1	28.0779036	26.5071459	25.7705507	29.09520242	27.0271305	28.21011732	0.22488904	0.961	1.33	PIS54741.1	TOP2	DNA topoisomerase II; catalyzes ATP-dependent DNA relaxation and decatenation in vitro; Y842 predicted to be catalytic; functional homolog of S. cerevisiae Top2p; sensitive to amsacrine or doxorubicin; farnesol-upregulated in biofilm
PIS55037.1	26.5748699	24.7234832	24.3284813	26.94780042	27.22150456	25.48372603	0.089450714	0.943	1.34	PIS55037.1	orf19.2073	Protein with a multidrug and toxin extrusion protein domain; induced by Mnl1 under weak acid stress
PIS49582.1	23.5399381	24.2569011	24.9715963	25.25161581	25.83224405	25.70147691	0.007930277	0.834	1.34	PIS49582.1	orf19.4128	Ortholog of C. dubliniensis CD36 : Cd36_19390, C. parapsilosis CDC317 : CPAR2_209600, C. auris B8441 : B9J08_004606 and Candida tenuis NRRL Y-1498 : CANTEDRAFT_134010
PIS54984.1	26.2783979	24.120627	24.5077297	26.66251992	25.89504466	26.36479639	0.054959549	0.93	1.34	PIS54984.1	orf19.4269	Ortholog of C. dubliniensis CD36 : Cd36_52330, C. parapsilosis CDC317 : CPAR2_101200, C. auris B8441 : B9J08_002133 and Candida tenuis NRRL Y-1498 : CANTEDRAFT_111562
PIS52204.1	27.5209343	26.7704156	25.56845	28.51095711	27.5985041	27.77740087	0.040624951	0.92	1.34	PIS52204.1	orf19.6931	Putative cleavage factor I subunit; heterozygous null mutant exhibits hypersensitivity to parnafungin and cordycepin in the C. albicans fitness test

PIS54945.1	23.081065	23.0650903	20.2929359	23.66384015	23.34159845	23.45965054	0.086021519	0.942	1.34	PIS54945.1	orf19.7368	Ortholog(s) have mRNA binding, poly(U) RNA binding, ribosome binding activity and role in nuclear-transcribed mRNA catabolic process, nonsense-mediated decay, regulation of mRNA stability, stress granule assembly, translational termination
PIS58200.1	26.7306302	25.0360653	22.4719592	27.41706937	25.59457768	25.25765753	0.253831587	0.963	1.34	PIS58200.1	orf19.7473	Ortholog(s) have role in endocytosis and actin cortical patch localization
PIS54718.1	25.2630255	24.4024596	22.7048625	25.78104004	25.80684759	24.84568359	0.047551324	0.925	1.35	PIS54718.1	CDC28	Cyclin-dependent protein kinase; interacts with regulatory subunit Cyb1; determination of cell morphology during the cell cycle; phosphorylated mostly by Swe1 and phosphorylation is regulated by Hsl1; 5'-UTR intron; Spider biofilm repressed
PIS54956.1	28.1350596	28.0369959	26.8440897	28.66670732	29.03810323	29.37490187	0.009045616	0.844	1.35	PIS54956.1	NCR1	Putative vacuolar membrane protein; predicted role in sphingolipid metabolism; transcript regulated by Nrg1 and Mig1; induced by prostaglandins
PIS49789.1	24.632799	24.6684355	22.9630921	25.54275659	25.57787756	25.20765151	0.097894766	0.945	1.35	PIS49789.1	orf19.578	Ortholog(s) have GTPase activator activity and role in endocytosis, exocytosis, formin-nucleated actin cable assembly, regulation of protein localization
PIS58848.1	27.9155246	26.6842567	26.7088856	28.78914668	27.83948056	28.72377172	0.03980303	0.919	1.35	PIS58848.1	orf19.789.1	Ortholog(s) have 7S RNA binding activity, role in SRP-dependent cotranslational protein targeting to membrane, translocation and signal recognition particle, endoplasmic reticulum targeting localization
PIS48611.1	27.0188553	26.2891497	26.6242969	26.96124675	28.27355777	28.75007618	0.014600688	0.874	1.35	PIS48611.1	YDC1	Alkaline dihydroceramidase; involved in sphingolipid metabolism; Mob2-dependent hyphal regulation; transcript is regulated by Nrg1 and Mig1; Hap43-repressed

PIS58444.1	26.2087622	24.4993935	24.6437479	27.5339052	25.92838437	25.97108658	0.242484654	0.962	1.36	PIS58444.1	ABP140	Ortholog of S. cerevisiae actin-binding protein Abp140; Hap43-induced; F-12/CO2 early biofilm induced
PIS56818.1	26.1175582	25.2035101	26.3766311	27.08692613	27.55069587	27.14712614	0.075716647	0.938	1.36	PIS56818.1	DBP3	Putative ATP-dependent DEAD-box RNA helicase; Hap43-induced; repressed by prostaglandins; Spider biofilm induced
PIS48750.1	30.9821264	29.5551215	28.9079716	31.57600902	31.11361622	30.83510449	0.010143835	0.852	1.36	PIS48750.1	LCB2	Putative serine palmitoyltransferase component; mutation confers hypersensitivity to aureobasidin A
PIS58175.1	28.2620743	28.5672272	28.4053026	30.42145506	29.7301043	29.15248973	0.003917656	0.77	1.36	PIS58175.1	orf19.1114	Cytochrome c oxidase subunit; membrane-localized protein; rat catheter biofilm induced
PIS55047.1	23.7044394	23.4467008	24.2370833	25.5821061	24.5693015	25.32311751	0.011422099	0.859	1.36	PIS55047.1	orf19.2097	Predicted DNA-dependent ATPase/helicase; Spider biofilm induced
PIS49786.1	25.7260045	25.0470901	25.2089121	25.99001688	26.88162106	27.19925823	0.002656194	0.726	1.36	PIS49786.1	orf19.3178	Ortholog(s) have RNA binding activity, role in mRNA 5'-splice site recognition, mRNA splicing, via spliceosome and U2-type prespliceosome localization
PIS54701.1	33.1025021	32.7530386	32.0787841	33.99389859	33.96612501	34.08518912	0.007666308	0.831	1.37	PIS54701.1	HGT7	Putative MFS glucose transporter; glucose, fluconazole, Snf3 induced, expressed at high glucose; 20 member C. albicans glucose transporter family; 12 TM regions predicted; flow model biofilm induced; Spider biofilm repressed

PIS49718.1	23.167298	23.590861	23.8055667	25.09630434	24.75349788	24.81604364	0.176853926	0.957	1.37	PIS49718.1	HUT1	Ortholog(s) have UDP-galactose transmembrane transporter activity and role in UDP-galactose transmembrane transport, UDP-glucose transmembrane transport, UDP-glucose transmembrane transport into endoplasmic reticulum
PIS48588.1	27.1120673	26.4977613	27.8625419	29.23422035	28.43696208	27.90144409	0.071534244	0.937	1.37	PIS48588.1	orf19.2167	Ortholog(s) have role in ribosomal large subunit biogenesis, ribosomal small subunit biogenesis and nucleolus localization
PIS51438.1	30.7220848	29.0569101	29.1505902	29.61023827	31.41756977	32.00688012	0.079040689	0.94	1.37	PIS51438.1	orf19.4699	Putative phospholipase of patatin family; similar to S. cerevisiae Tgl3p; predicted Kex2p substrate
PIS48678.1	32.7444644	32.3373751	31.957579	33.73424415	33.4171878	34.00442438	0.002548402	0.721	1.37	PIS48678.1	SUR7	Protein required for normal cell wall, plasma membrane, cytoskeletal organization, endocytosis; localizes to eisosome subdomains of plasma membrane; 4 transmembrane motifs; cytoplasmic C terminus regulates morphogenesis and stress response
PIS58809.1	27.3981727	24.5325373	25.4907382	28.73206196	24.68420593	28.12076331	0.19029305	0.958	1.37	PIS58809.1	VCX1	Putative H+/Ca2+ antiporter; Spider biofilm repressed
PIS48557.1	28.0526557	27.9545958	26.6608189	29.13246257	28.92826494	28.73741303	0.090859091	0.943	1.38	PIS48557.1	NEP1	Ortholog(s) have rRNA (pseudouridine) methyltransferase activity
PIS56807.1	27.6087996	27.2376274	25.512005	27.99065733	28.43240289	28.07703302	0.030274402	0.909	1.38	PIS56807.1	orf19.2313	Putative ortholog of S. cerevisiae Pex32 a peroxisomal integral membrane protein with a role in negative regulation of peroxisome size; Hap43-repressed gene

PIS52060.1	26.8978509	26.1222374	25.8116063	27.90210941	28.14070781	26.94178681	0.008330474	0.838	1.38	PIS52060.1	orf19.3057	Ortholog(s) have role in mitochondrial genome maintenance
PIS58726.1	28.4001588	28.5323799	27.864715	30.09102396	29.34463802	29.50236104	0.000366965	0.394	1.38	PIS58726.1	PMC1	Vacuolar calcium P-type ATPase; transcript regulated by calcineurin and fluconazole; mutant shows increased resistance to fluconazole, lithium; increased sensitivity to calcium; Spider biofilm induced
PIS51644.1	26.2605777	26.2922952	24.2991243	26.12480164	27.14301549	27.7443634	0.026657906	0.904	1.39	PIS51644.1	MOB2	Mob1/phocein domain protein of RAM signaling network; cell wall integrity; role in cell separation, cortical actin polarization; required for hyphal growth; phosphorylated by Cdc28 on hyphal induction; activates Cbk1; mRNA binds She3
PIS58946.1	24.4507563	22.4857023	24.3516867	26.06223695	23.95320147	25.44302191	0.353764273	0.967	1.39	PIS58946.1	orf19.1096	Has domain(s) with predicted voltage-gated chloride channel activity, role in chloride transport, transmembrane transport and membrane localization
PIS48526.1	27.9203672	27.5705959	27.0911389	28.9686415	28.72737305	29.05046785	0.002847739	0.734	1.39	PIS48526.1	orf19.2631	Subunit of Elongator complex; required for modification of wobble nucleosides in tRNA in S. cerevisiae; Hap43-induced gene
PIS58432.1	24.2746949	24.8624417	23.8972361	25.24698818	25.40388163	26.55550164	0.025859653	0.902	1.39	PIS58432.1	orf19.2851	Putative ornithine transport protein; localized to the mitochondrial membrane
PIS56588.1	31.3313294	30.9140899	30.6780893	32.3765568	32.55270896	32.16051755	0.017724919	0.885	1.39	PIS56588.1	SNQ2	Protein similar to S. cerevisiae Snq2p transporter; member of PDR subfamily of ABC family; transposon mutation affects filamentation; benomyl-induced transcription; detected at yeast-form cell plasma membrane by mass spec

PIS55789.1	28.8602182	27.7144328	27.896442	30.58221114	28.77878777	29.31719741	0.029297615	0.908	1.4	PIS55789.1	orf19.1676	Predicted potassium ion transporter; Spider biofilm induced
PIS51739.1	28.6167693	27.2240931	26.9942208	28.86121988	29.00164127	29.1594434	0.01481742	0.875	1.4	PIS51739.1	orf19.2030	Plasma membrane-associated protein; induced in cyr1 or ras1 mutant; induced by hypoxia, ketoconazole and during growth in the mouse cecum; induced in oralpharyngeal candidasis; Spider biofilm induced
PIS51663.1	29.0954512	28.4612243	28.1470393	29.51673865	30.2336395	30.14763398	0.000930158	0.568	1.4	PIS51663.1	orf19.2978	Protein of unknown function; Hap43-repressed gene
PIS55837.1	29.9609903	28.6522324	28.4588925	30.34064324	30.15829859	30.7602284	0.009658471	0.848	1.4	PIS55837.1	orf19.3166	Ortholog(s) have role in cell morphogenesis, retrograde transport, endosome to Golgi, retrograde transport, vesicle recycling within Golgi and endosome, trans-Golgi network localization
PIS50560.1	27.1671718	26.9357892	27.0589859	28.3350851	28.43347196	28.59019561	0.014028531	0.872	1.4	PIS50560.1	orf19.4262	Ortholog(s) have mRNA binding activity and role in establishment of mitochondrion localization, nuclear-transcribed mRNA catabolic process, deadenylation-dependent decay, protein-containing complex localization
PIS54480.1	26.561155	26.0451434	23.4726648	26.26640759	27.04300919	26.95590979	0.052786216	0.928	1.4	PIS54480.1	orf19.7098	Putative protein of unknown function; Hap43-repressed; repressed by nitric oxide; Spider biofilm induced
PIS58501.1	25.6421643	26.3501616	25.2965398	27.01789473	26.86577969	27.62852679	0.011809772	0.862	1.41	PIS58501.1	CRL1	Predicted GTPase of RHO family; CAAX motif geranylgeranylated; expression in S. cerevisiae causes dominant-negative inhibition of pheromone response

PIS48660.1	22.3174779	23.5820203	23.9464689	23.68416407	24.6321944	25.75886581	0.315405236	0.966	1.41	PIS48660.1	EAF7	Subunit of the NuA4 histone acetyltransferase complex
PIS48695.1	24.3707106	24.4695342	24.5628713	25.95210922	25.50690043	26.15954162	0.03788851	0.917	1.41	PIS48695.1	FCA1	Cytosine deaminase; enzyme of pyrimidine salvage; functional homolog of S. cerevisiae Fcy1p; mutation is associated with resistance to flucytosine (5-FC) in a clinical isolate; hyphal downregulated; gene has intron
PIS50417.1	24.129289	23.8690022	23.2106571	24.6962463	25.01465147	25.71490742	0.025521421	0.902	1.41	PIS50417.1	orf19.5587	Protein of unknown function; transcript is upregulated in clinical isolates from HIV+ patients with oral candidiasis
PIS54643.1	31.8887997	31.5453651	32.8980715	33.82633499	32.94904341	33.7923024	0.015027796	0.876	1.41	PIS54643.1	orf19.5943.1	Protein of unknown function; transcript upregulated in an RHE model of oral candidiasis; rat catheter and Spider biofilm repressed
PIS51722.1	23.3004885	22.7872753	23.7172849	24.75533283	24.31488202	24.96741252	0.11108342	0.947	1.41	PIS51722.1	orf19.7224	Ortholog(s) have ubiquitin protein ligase activity and role in histone catabolic process
PIS51104.1	25.3420486	24.4781645	24.0402082	26.33139017	26.73106011	25.05795109	0.022116169	0.895	1.42	PIS51104.1	BNR1	Formin; probable role in hyphal cytoskeletal polarity; synthetic lethality if Bnr1p and Bni1p are absent
PIS52105.1	28.0449075	27.1274762	27.3412629	29.23365675	28.61645386	28.93234705	0.006111635	0.813	1.42	PIS52105.1	GDT1	Golgi Ca2+/H+ exchanger, plays a compensatory role for the calcium pump Pmr1p in regulation of calcium homeostasis

PIS51339.1	22.6198218	23.7650414	23.0358819	24.35465011	24.37518555	24.9649016	0.002770032	0.731	1.42	PIS51339.1	HST1	Putative histone deacetylase, involved in regulation of white-opaque switching
PIS56928.1	23.7528157	22.1463955	22.9481539	24.28489598	24.53475783	24.28555497	0.020232698	0.891	1.42	PIS56928.1	orf19.4030	Ortholog(s) have DNA primase activity, single-stranded DNA binding activity and role in DNA replication, DNA replication, synthesis of RNA primer, mitotic DNA replication initiation
PIS51447.1	25.1548985	24.7315264	25.373314	26.56696905	26.07278792	26.87307904	0.011955105	0.862	1.42	PIS51447.1	orf19.4090.1	Ortholog(s) have role in vacuolar proton-transporting V-type ATPase complex assembly and endoplasmic reticulum membrane localization
PIS52108.1	26.9185493	27.5098675	26.8805951	28.65765863	28.35671703	28.54459198	0.135709053	0.952	1.42	PIS52108.1	RIM2	Putative mitochondrial carrier protein; induced by alpha pheromone in SpiderM medium; Spider biofilm induced
PIS54737.1	25.2378707	24.5493953	25.0812061	24.98136061	27.26686874	26.88070865	0.053777825	0.929	1.42	PIS54737.1	SHE3	mRNA-binding protein that localizes specific mRNAs to daughter yeast cells and to hyphal tips; required for normal filamentation and host epithelial cell damage; ortholog of S. cerevisiae She3 but target mRNAs differs
PIS55516.1	26.8043889	26.7055975	25.2198722	28.68781794	26.94885889	27.34974781	0.047869136	0.925	1.42	PIS55516.1	SKP1	Putative subunit D of kinetochore protein complex CBF3; regulated by Gcn4p; repressed in response to amino acid starvation (3-aminotriazole treatment)
PIS58777.1	28.0298945	28.2073787	27.2054601	29.17213205	29.31706357	29.22747343	0.002977681	0.74	1.42	PIS58777.1	SPL1	Protein similar to S. cerevisiae Spl1p, which is involved in tRNA splicing; member of pyridoxal-phosphate-dependent aminotransferase protein family; predicted to be essential

PIS52267.1	25.9907725	25.2554634	24.977897	27.43904234	26.84780832	26.23761244	0.02221119	0.896	1.43	PIS52267.1	ARG2	Putative enzyme of arginine biosynthesis; transcription of genes of arginine biosynthesis pathway, except for ARG2, is induced upon phagocytosis by macrophage
PIS54691.1	27.2787762	27.2077187	26.9396083	30.09206284	27.92295556	27.69184971	0.02190865	0.895	1.43	PIS54691.1	orf19.2518	Ortholog(s) have ATPase activator activity, soluble NSF attachment protein activity, role in SNARE complex disassembly, autophagy, vacuole fusion, non-autophagic, vesicle fusion with Golgi apparatus and SNARE complex, cytosol localization
PIS52157.1	26.925036	25.9740477	26.9862881	28.34265439	27.83155358	28.01013759	0.051268571	0.927	1.43	PIS52157.1	orf19.3124	Ortholog(s) have mRNA binding, metalloaminopeptidase activity, role in negative regulation of gene expression and cytosolic ribosome localization
PIS54572.1	25.9025839	25.4946482	24.629456	28.19556198	27.00337116	25.12641838	0.117793867	0.949	1.43	PIS54572.1	orf19.7197	Putative intranuclear transport and DNA replication mediator; heterozygous null mutant exhibits resistance to parnafungin in the C. albicans fitness test; Spider biofilm induced
PIS48811.1	28.5764043	27.2370254	26.0105842	28.89348302	28.76089272	28.44812326	0.105802149	0.946	1.43	PIS48811.1	SNF5	SWI/SNF chromatin remodeling complex subunit involved in transcriptional regulation; mutants have defects in silicone adherence, biofilm formation, hyphal morphogenesis, cell wall defects; increased cell aggregation during
PIS56836.1	24.4132283	25.7249725	24.958619	25.74868448	27.00039968	26.63249018	0.063284442	0.934	1.43	PIS56836.1	SOD1	Cytosolic copper- and zinc-containing superoxide dismutase; role in protection from oxidative stress; required for full virulence; alkaline induced by Rim101; induced by human blood; rat catheter, flow model and Spider biofilm repressed
PIS48408.1	28.3827485	27.257893	27.7034197	29.39426547	29.19521267	29.06308765	0.009081048	0.844	1.44	PIS48408.1	orf19.3516	Protein of unknown function; Hap43-repressed; Spider biofilm repressed

PIS55455.1	27.146523	26.0132535	25.4743355	27.66690111	27.65051037	27.64649861	0.003766952	0.766	1.44	PIS55455.1	orf19.4247	Protein of unknown function; rat catheter and Spider biofilm repressed
PIS49630.1	23.4044713	24.7547315	25.2024619	24.86320075	26.24751826	26.57320726	0.0701648	0.936	1.44	PIS49630.1	orf19.7291	Ortholog(s) have tRNA (adenine(58)-N1)-methyltransferase activity, role in tRNA methylation and nucleus, tRNA (m1A) methyltransferase complex localization
PIS58890.1	27.8949898	25.7644038	25.5815971	27.78174131	28.16338043	27.64216164	0.104672717	0.946	1.45	PIS58890.1	HGT18	Putative glucose transporter of the major facilitator superfamily; the C. albicans glucose transporter family comprises 20 members; 12 probable membrane-spanning segments; expressed in rich medium with 2% glucose
PIS48447.1	24.5385068	25.1235645	25.0374831	27.2907221	25.71156071	26.05523095	0.076287247	0.939	1.45	PIS48447.1	orf19.1049	Predicted NUDIX hydrolase domain; Hap43-induced
PIS52266.1	24.6147607	25.993689	24.360867	24.64225935	26.68377357	27.99994089	0.054665412	0.929	1.45	PIS52266.1	PSF2	Ortholog(s) have role in DNA-templated DNA replication, double-strand break repair via break-induced replication, mitotic DNA replication
PIS58797.1	25.6119549	24.8434961	24.9310751	26.82201622	25.92350621	26.98770188	0.026249925	0.903	1.45	PIS58797.1	RTG3	Transcription factor with bZIP motif involved in regulation of galactose catabolism genes; performs role analogous to Gal4p in S. cerevisiae; regulates carbon source-dependent stress response; regulates sphingolipid homeostasis
PIS55729.1	25.9492736	27.4702575	28.9811136	28.79630916	28.97798423	28.96283921	0.067228055	0.935	1.45	PIS55729.1	TPO4	Putative sperimidine transporter; fungal-specific (no human or murine homolog); Spider biofilm induced; promoter bound by Tec1 and Ndt80; Bcr1-repressed in RPMI a/a biofilms

PIS58276.1	27.2542703	25.426904	24.6017309	27.62656279	27.6842388	26.34238688	0.130363529	0.951	1.46	PIS58276.1	orf19.4659	Ortholog(s) have RNA binding activity, role in mRNA splicing, via spliceosome and U2 snRNP, U2-type prespliceosome localization
PIS49624.1	24.5939099	26.7616653	28.6173879	27.73414795	28.00449081	28.6208497	0.077000852	0.939	1.46	PIS49624.1	orf19.7196	Putative vacuolar protease; upregulated in the presence of human neutrophils; Spider biofilm induced
PIS54759.1	23.5370534	23.3297834	22.3586683	24.58458501	24.68368707	24.33835678	0.002748713	0.73	1.46	PIS54759.1	POL32	Subunit of DNA polymerase delta, involved in chromosomal DNA replication; required for pathogenesis; null mutant is viable but defective in resistance to DNA damage, has abnormal cells and reduced hyphal growth, and appears avirulent
PIS58967.1	27.0501088	26.7564357	26.354293	28.30676634	28.18888032	28.04475508	0.169243737	0.956	1.46	PIS58967.1	PUS4	Putative pseudouridine synthase; transcript regulated by Nrg1, Mig1, and Tup1
PIS48364.1	24.7963875	24.982877	25.4524713	27.04346094	25.63644298	26.92810919	0.015639814	0.878	1.46	PIS48364.1	SLD1	Sphingolipid delta-8 desaturase; catalyzes desaturation at C8 in the long-chain base moiety of ceramides in glucosylceramide synthesis, important for virulence; ketoconazole and hypoxia induced; Hap43-repressed; Spider biofilm induced
PIS54970.1	18.7847149	27.1549877	27.4699626	26.14054627	25.95917176	25.71852343	0.375593017	0.968	1.47	PIS54970.1	orf19.6687	Protein of unknown function; rat catheter biofilm repressed
PIS51134.1	23.8705627	23.4809073	23.6825198	25.25749516	25.09436239	25.10270986	0.032231658	0.911	1.47	PIS51134.1	UGT51C1	UDP-glucose:sterol glucosyltransferase; enzyme of sterol glucoside (membrane-bound lipid) biosynthesis; has UDP-sugar binding domain; activity is UDP-glucose-specific in vitro; enzyme does not use UDP-mannose; Mig1-regulated

PIS48456.1	24.3020846	24.113379	24.39654	25.75912009	25.47700389	26.01735147	0.000312674	0.366	1.48	PIS48456.1	MRP8	Mitochondrial ribosomal protein; ortholog of S. cerevisiae Mrp8; transcript induced in hyphal form; mutant is viable; flow model and rat catheter biofilm repressed
PIS51533.1	25.3705571	23.7934199	22.8296399	23.87482956	26.05230395	26.51267159	0.067208527	0.935	1.48	PIS51533.1	orf19.4492	Ortholog(s) have role in nuclear division, rRNA processing, ribosomal large subunit biogenesis and nuclear periphery, nucleolus, preribosome, large subunit precursor localization
PIS50605.1	25.3298119	23.8654471	24.3734389	26.74270907	25.74382935	25.52716842	0.130770167	0.951	1.48	PIS50605.1	orf19.913.2	Putative subunit 6 of the ubiquinol cytochrome-c reductase complex, a component of the mitochondrial inner membrane electron transport chain; null mutant is viable
PIS48157.1	26.9928497	26.4763128	27.8993582	28.06364339	28.10425668	29.64669862	0.017560471	0.884	1.48	PIS48157.1	RBT5	GPI-linked cell wall protein; hemoglobin utilization; Rfg1, Rim101, Tbf1, Fe regulated; Sfu1, Hog1, Tup1, serum, alkaline pH, antifungal drugs, geldamycin repressed; Hap43 induced; required for RPMI biofilms; Spider biofilm induced
PIS56718.1	27.8331708	26.0675529	25.8215908	28.82218712	27.64553879	27.6947088	0.081465941	0.94	1.48	PIS56718.1	RFC4	Putative heteropentameric replication factor C subunit; flucytosine induced; periodic mRNA expression, peak at cell-cycle G1/S phase
PIS55061.1	23.4991116	23.8604932	23.3000289	25.83789784	25.4141464	23.86245526	0.02168997	0.895	1.48	PIS55061.1	SAS10	Putative U3-containing small subunit processome complex subunit; Hap43p-induced gene; mutation confers resistance to 5-fluorocytosine (5-FC); repressed upon high-level peroxide stress
PIS49764.1	19.9259294	18.4421497	21.4544515	22.82808378	21.77525153	19.68274259	0.225556329	0.961	1.49	PIS49764.1	MSH3	Mismatch repair protein; predicted role in repair of insertion or deletion mutations and removal of nonhomologous DNA ends; rat catheter biofilm repressed

PIS54604.1	22.1347178	22.7608201	21.8865592	24.22165921	23.74718353	23.29634048	0.047921866	0.925	1.49	PIS54604.1	OPI3	Phosphatidylethanolamine N-methyltransferase; acts in phosphatidylcholine biosynthesis; downregulation correlates with clinical development of fluconazole resistance; amphotericin B, caspofungin repressed; Hap43-induced
PIS51424.1	26.2340233	24.9636351	28.399154	27.90041499	27.83141684	28.32784665	0.144072868	0.953	1.49	PIS51424.1	orf19.1082.1	Ortholog(s) have cytochrome-c oxidase activity and role in mitochondrial cytochrome c oxidase assembly, mitochondrial electron transport, cytochrome c to oxygen
PIS58308.1	25.1087063	22.5410521	25.2224991	25.75802917	25.76510811	25.82962307	0.037275846	0.917	1.49	PIS58308.1	orf19.3872	Protein of unknown function; oral infection induced; mutants have reduced capacity to damage oral epithelial cells; rat catheter biofilm repressed
PIS52251.1	25.0381831	24.4107951	24.6142065	25.65284639	26.38573346	26.50480165	0.023547589	0.898	1.49	PIS52251.1	PPE1	Protein similar to S. cerevisiae Ppe1p; shows genetic interaction with some genes involved in diploid filamentous growth and haploid invasive growth in S. cerevisiae
PIS55706.1	29.8823731	29.7378436	30.1632574	31.76225807	31.14521097	31.34380429	0.001991839	0.688	1.49	PIS55706.1	YVC1	Putative vacuolar cation channel shock; repressed by alpha pheromone in SpiderM medium; flow model biofilm repressed
PIS58932.1	27.0698564	29.0841819	28.6072356	28.73130644	30.1107843	30.43253954	0.065036453	0.934	1.5	PIS58932.1	NAT2	Putative N-terminal acetyltransferase; Hap43p-repressed gene; mutation confers hypersensitivity to toxic ergosterol analog
PIS52006.1	26.8525606	26.9290158	26.4467262	28.86120381	28.20349965	27.67047391	0.007238248	0.827	1.5	PIS52006.1	NOT5	Protein with similarity to S. cerevisiae Not5p, a member of the transcription regulatory CCR4-NOT complex; required for hyphal growth; antigenic during human oral infection; greater expression in yeast-form cells than hyphae

PIS50372.1	25.832665	24.7038411	24.5412512	26.57176152	25.30590845	27.70916308	0.027758262	0.905	1.5	PIS50372.1	orf19.1412	Ortholog of C. dubliniensis CD36 : Cd36_44020, C. parapsilosis CDC317 : CPAR2_302330, C. auris B8441 : B9J08_004187 and Candida tenuis NRRL Y-1498 : CANTEDRAFT_105448
PIS51035.1	26.9114945	26.4202493	26.750466	28.53348465	27.65808705	28.39955249	0.002078229	0.694	1.5	PIS51035.1	orf19.6406	Ortholog(s) have structural constituent of ribosome activity and mitochondrial small ribosomal subunit, peroxisome localization
PIS50451.1	26.2187276	24.5252287	25.13652	27.42415042	27.32961587	25.6663589	0.021544435	0.894	1.51	PIS50451.1	COX6	Putative cytochrome c oxidase; flucytosine induced
PIS55012.1	25.0221024	24.8004926	22.3868095	25.57587414	25.46538613	25.71177494	0.048630421	0.926	1.51	PIS55012.1	orf19.1177	Ortholog of S. cerevisiae Rtt106; histone chaperone that regulates chromatin structure in transcribed and silenced chromosomal regions; affects transcriptional elongation; Hap43-repressed; Spider biofilm repressed
PIS50330.1	31.7421012	29.9981041	29.744791	32.54817996	31.56621917	31.89869235	0.012258085	0.864	1.51	PIS50330.1	orf19.4931	Putative tRNA-Cys synthetase; induced by alpha pheromone in SpiderM medium; ribosomal subunits, translation factors, tRNA synthetases are downregulated upon phagocytosis by murine macrophage
PIS58228.1	23.9848243	23.3537328	23.2004818	25.20694955	24.50903213	25.35609941	0.000378017	0.399	1.51	PIS58228.1	orf19.7010	Ortholog(s) have ubiquitin ligase complex localization
PIS49687.1	28.3391214	28.0426574	28.1894448	29.70529907	29.0507885	30.34622239	0.002696895	0.728	1.51	PIS49687.1	OSH3	Protein required for wild-type filamentation; has oxysterol binding protein domain and pleckstrin homology motif; overproducton suppresses cph1 homozygous null filamnetation defect; similar to S. cerevisiae Osh3p

PIS48747.1	26.7140234	24.4487336	24.3075583	26.70673468	26.54297403	26.75677659	0.070451397	0.937	1.51	PIS48747.1	SSK1	Response regulator of two-component system; role in oxidative stress response, cell wall biosynthesis, virulence, hyphal growth on solid media; expressed in hyphae and yeast; peroxisomal targeting sequence (PTS1); Spider biofilm induced
PIS54800.1	25.9383174	25.3969609	24.8111364	27.00322995	26.32878486	27.35248487	0.125291336	0.95	1.51	PIS54800.1	SUA71	Transcription factor TFIIB; required for transcription initiation and start site selection by RNA polymerase II; downregulated during planktonic growth, whereas related SUA72 is induced; induced by Tbf1; rat catheter, Spider biofilm induced
PIS55670.1	24.6639509	24.4154808	24.4155699	25.21819648	26.22627604	26.60717825	0.046905191	0.925	1.52	PIS55670.1	orf19.1573	Ortholog(s) have fungal-type vacuole membrane, vacuole-mitochondrion membrane contact site localization
PIS51599.1	23.7613615	25.5823734	24.1678907	24.23906078	26.66764343	27.15911433	0.192506465	0.958	1.52	PIS51599.1	orf19.2007	Subunit of GARP (Golgi-associated retrograde protein) complex, which has roles in Golgi to vacuole transport, ascospore wall assembly, cellular sphingolipid homeostasis, and filamentous growth
PIS56542.1	25.2789437	25.3721948	26.4267893	27.83038972	26.78386174	27.03459305	0.005926993	0.81	1.52	PIS56542.1	orf19.5539	Ortholog(s) have SNAP receptor activity, role in retrograde vesicle-mediated transport, Golgi to endoplasmic reticulum and SNARE complex, cytoplasmic side of endoplasmic reticulum membrane localization
PIS58850.1	27.8100372	23.2949161	25.4965053	27.26294405	26.79168057	27.09459183	0.108153502	0.947	1.52	PIS58850.1	RAM1	Protein that acts in prenylation; transcription is alpha-factor induced; regulated in response to lovastatin and fluconazole; Hap43p-repressed gene
PIS51435.1	27.0410813	26.4387649	26.5321622	28.00596927	27.82632399	28.78452303	0.00050483	0.455	1.53	PIS51435.1	BMT8	Putative beta-mannosyltransferase, member of a 9-gene family including characterized BMT genes with roles in beta-1,2-mannosylation of cell wall phosphopeptidomannan; transposon insertion in promoter region causes decreased colony wrinkling

PIS49754.1	23.9428726	25.8332484	24.6719181	26.11235242	25.88914323	27.03492555	0.009591739	0.848	1.53	PIS49754.1	LEU1	3-isopropylmalate dehydratase; antigenic in humans; repressed in hyphae; alkaline repressed; upregulated by human whole blood or PMNs; regulated by Sef1, Sfu1, and Hap43; rat catheter biofilm induced, Spider biofilm repressed
PIS54582.1	24.5505195	25.1166335	26.160222	26.8662474	26.58566371	26.95615584	0.010184523	0.852	1.53	PIS54582.1	MSS4	Phosphatidylinositol-4-phosphate 5-kinase; activity induced by phosphatidic acid (Pld1 product); macrophage/pseudohyphal-repressed; mRNA binds to She3, localized to yeast cell buds and hyphal tips; Hap43-induced; Spider biofilm induced
PIS51729.1	23.3569204	24.1805638	23.1436356	26.29617519	24.68609427	24.29627229	0.026811197	0.904	1.53	PIS51729.1	orf19.2043	Has domain(s) with predicted ATP binding, ATP-dependent FeS chaperone activity, iron-sulfur cluster binding activity and role in iron-sulfur cluster assembly
PIS59028.1	26.6781625	26.0098321	26.8054013	27.6710608	28.04305237	28.37192083	0.126300719	0.95	1.53	PIS59028.1	orf19.3663.1	Predicted plasma membrane protein; gene has intron
PIS54987.1	29.0882568	27.4372148	27.8749303	30.82180284	29.64105799	28.52446987	0.014782405	0.875	1.53	PIS54987.1	orf19.5569	Ortholog(s) have role in establishment of mitotic sister chromatid cohesion, maintenance of rDNA, mitotic sister chromatid segregation and chromosome, telomeric region, nuclear envelope localization
PIS49839.1	26.9704576	25.574192	27.469083	28.43540947	28.2284989	27.93285781	0.055760745	0.93	1.53	PIS49839.1	orf19.5576	Putative pantothenate kinase; ortholog of S. cerevisiae Cab1; transposon mutation affects filamentous growth; repressed in core stress response
PIS49579.1	26.3054422	25.6660107	26.7217409	27.87392027	27.57109141	27.83601935	0.004868122	0.792	1.53	PIS49579.1	orf19.5780	Putative protein of unknown function; Hap43p-repressed gene

PIS51488.1	27.945384	28.8562378	27.7119193	29.87540847	28.91818891	30.30927176	0.005674744	0.806	1.53	PIS51488.1	orf19.5834	Ortholog(s) have nuclear import signal receptor activity, role in mRNA export from nucleus, protein import into nucleus and cytoplasm, nucleus localization
PIS56670.1	22.4892614	24.7208194	21.8056276	23.43092259	25.0328459	25.15659239	0.068080322	0.936	1.53	PIS56670.1	orf19.6723	Protein of unknown function; Spider biofilm induced
PIS56831.1	28.5760401	28.3188914	27.6212802	30.63958959	29.35461969	29.13669898	0.003253013	0.75	1.54	PIS56831.1	MSN5	Predicted karyopherin involved in nuclear import and export of proteins; flow model biofilm induced; Spider biofilm induced
PIS51659.1	24.7766458	24.499354	24.6048481	26.98459657	26.54132404	24.96569995	0.080752255	0.94	1.54	PIS51659.1	MST1	Ortholog(s) have threonine-tRNA ligase activity, role in mitochondrial threonyl-tRNA aminoacylation and mitochondrion localization
PIS51727.1	27.4912617	25.1011234	25.8555658	26.17751686	29.47724707	27.40963001	0.078766099	0.939	1.54	PIS51727.1	orf19.2045	Ortholog(s) have CTP-dependent diacylglycerol kinase activity and role in mitotic nuclear membrane biogenesis, phosphatidic acid biosynthetic process, regulation of isopentenyl diphosphate biosynthetic process, mevalonate pathway
PIS58978.1	20.4707933	23.9773162	24.2490824	24.6652525	23.7460067	24.90750891	0.357870237	0.968	1.54	PIS58978.1	orf19.934	Predicted ORF from Assembly 19; removed from Assembly 20; subsequently reinstated in Assembly 21 and merged with orf19.2224, based on comparative genome analysis; flow model biofilm repressed
PIS49774.1	26.2600201	26.3285365	25.8702485	26.56759174	28.21796308	28.28018609	0.012104391	0.863	1.54	PIS49774.1	POP4	Ortholog of S. cerevisiae Pop4; a subunit of both RNase MRP and nuclear RNase P; filament induced; regulated by Nrg1, Tup1; likely essential, based on UAU1 strategy; rat catheter and Spider biofilm induced

PIS51945.1	25.2942123	25.2346198	25.136507	27.11235852	25.68242652	27.50799573	0.010895245	0.856	1.55	PIS51945.1	orf19.2889	Ortholog(s) have role in CENP-A containing chromatin assembly, chromatin remodeling and Ino80 complex, chromatin localization
PIS54980.1	22.4514581	25.0232552	24.5996097	26.66716319	25.0566701	24.98845451	0.038343254	0.918	1.55	PIS54980.1	RAD9	DNA damage-dependent checkpoint protein; involved in regulation of DNA-damage-induced filamentous growth; induced by alpha pheromone in SpiderM medium
PIS52288.1	27.4301195	26.1692869	25.6582226	28.64529341	27.40543006	27.87315748	0.040424036	0.92	1.56	PIS52288.1	EMC9	Ortholog of S. cerevisiae Nnf2; possible role in chromosome segregation; mutants are viable, induced during the mating process
PIS52043.1	29.1362919	29.2827208	29.0460978	30.31419691	30.57364462	31.2559058	0.002731685	0.729	1.56	PIS52043.1	orf19.1546	Membrane-localized protein of unknown function
PIS51342.1	25.825107	26.7075993	26.2486236	29.08303034	27.47568076	26.90606504	0.065212134	0.934	1.56	PIS51342.1	orf19.4150	Putative glutaredoxin; induced by nitric oxide; Spider biofilm induced
PIS51459.1	29.2346402	26.8417764	26.190393	29.16698422	29.48077013	28.28745347	0.123576765	0.95	1.56	PIS51459.1	orf19.5022	Ortholog(s) have inorganic cation transmembrane transporter activity and role in cobalt ion transport, intracellular cobalt ion homeostasis, intracellular manganese ion homeostasis, manganese ion transport
PIS58365.1	25.6541977	24.7815743	25.0361322	26.95460848	26.39702814	26.78873974	0.03827754	0.918	1.56	PIS58365.1	TBF1	Essential transcription factor; induces ribosomal protein genes and the rDNA locus; acts with Cbf1 at subset of promoters; recruits Fhl1 and Ifh1 to promoters; role is analogous to that of S. cerevisiae Rap1; Spider biofilm induced

PIS56701.1	26.1500665	25.8602467	25.0420837	26.10310395	28.4034165	27.26834739	0.1847624	0.957	1.57	PIS56701.1	AVT4	Putative vacuolar transporter of large neutral amino acids; induced by alpha pheromone in SpiderM medium
PIS58313.1	23.6117388	23.7289929	23.959584	25.22847467	24.88735188	25.88483869	0.011184479	0.858	1.57	PIS58313.1	FGR50	Protein lacking an ortholog in S. cerevisiae; transposon mutation affects filamentous growth; Spider biofilm repressed
PIS51528.1	28.8424392	29.3206397	29.1958609	30.57816718	30.50389508	31.00290062	0.000872897	0.557	1.58	PIS51528.1	CDS1	Protein similar to S. cerevisiae Cds1p; transposon mutation affects filamentous growth
PIS54487.1	30.4687362	30.1331061	30.9380391	31.54208138	31.90210125	32.8417996	0.001857047	0.678	1.58	PIS54487.1	NCE102	Non classical protein export protein; localized to plasma membrane; Hap43-induced gene; flow model biofilm induced; Spider biofilm induced
PIS50315.1	25.0866921	26.802431	27.1563119	27.74364678	27.75949985	28.29551605	0.011409372	0.859	1.58	PIS50315.1	UTP9	Small-subunit processome protein; Ssr1-induced; repressed by prostaglandins; physically interacts with TAP-tagged Nop1
PIS58058.1	24.203031	24.1429077	24.1447702	25.8807604	25.36527089	26.01894408	0.005472184	0.803	1.59	PIS58058.1	CTM1	Lysine methyltransferase that trimethylates cytochrome c; involved in repression of hyphal growth via methylation activity; regulated by Gcn2 and Gcn4; transcript induced by Mnl1 under weak acid stress
PIS51092.1	26.8680733	26.9280302	24.9854971	28.16690521	28.45630418	26.94221977	0.02462271	0.9	1.59	PIS51092.1	MLP1	Ortholog(s) have promoter-terminator loop anchoring activity, ribonucleoprotein complex binding activity

PIS51332.1	26.8521965	27.3803532	26.404092	28.50629735	28.65828001	28.23757706	0.083653008	0.941	1.59	PIS51332.1	orf19.4727	Ortholog(s) have role in mitochondrial respiratory chain complex II assembly, protein flavinylation, tricarboxylic acid cycle and mitochondrial matrix localization
PIS54486.1	27.7091704	27.2313035	26.3444582	29.86974913	27.92563923	28.26521943	0.024778342	0.901	1.59	PIS54486.1	orf19.5961	Ortholog(s) have proteasome regulatory particle binding, protein folding chaperone activity, role in proteasome regulatory particle assembly and cytosol, nucleus localization
PIS56619.1	28.0219893	27.5188425	25.6722269	28.62907463	28.74032619	28.61016685	0.009604698	0.848	1.59	PIS56619.1	orf19.7058	Ortholog of S. cerevisiae : RMD8, C. glabrata CBS138 : CAGL0C01969g, C. dubliniensis CD36 : Cd36_70050, C. parapsilosis CDC317 : CPAR2_301100 and C. auris B8441 : B9J08_001157
PIS52049.1	28.6898107	27.8038176	28.3879835	29.76194009	29.31760722	30.57845589	0.005390372	0.802	1.59	PIS52049.1	PBP2	Putative RNA binding protein; transcript regulated by Nrg1, Mig1, and Tup1
PIS56665.1	29.4711297	29.0691994	28.4942195	30.57323988	30.53554511	30.75960712	0.001077878	0.593	1.61	PIS56665.1	orf19.273	Protein involved in regulation of C14-methylated sterol biosynthesis; mutations increase azole sensitivity
PIS55702.1	22.4372384	22.8270694	22.2494592	23.8283519	24.35433034	24.16492407	0.402186482	0.969	1.61	PIS55702.1	orf19.3142	Ortholog of C. dubliniensis CD36 : Cd36_46140, C. parapsilosis CDC317 : CPAR2_501210, C. auris B8441 : B9J08_001807 and Candida tenuis NRRL Y-1498 : CANTEDRAFT_97195
PIS58183.1	24.8471927	23.4273828	25.2139175	26.03494505	25.99036837	26.3024122	0.037876175	0.917	1.61	PIS58183.1	orf19.3220	Putative rRNA processing protein; Spider biofilm induced

PIS58630.1	27.3483449	25.2015938	24.1564706	26.50864609	27.66122814	27.36809406	0.038274366	0.918	1.61	PIS58630.1	SCP1	Putative cortical actin cytoskeleton protein; constitutive expression independent of MTL or white-opaque status
PIS54931.1	23.4598418	23.1758985	24.2257531	25.40485145	24.4613989	25.82566571	0.018773429	0.888	1.61	PIS54931.1	TRK1	Potassium transporter; mediates K+ and Cl- influx; role in sensitivity to cationic antimicrobial peptides, not by direct uptake, possibly related to membrane permeability; contains 5' UTR intron; Spider biofilm induced
PIS55545.1	24.395717	24.1943713	22.9723206	25.65075023	25.09728028	25.6639496	0.048500622	0.926	1.62	PIS55545.1	CDH1	Protein involved in regulation of mitosis; similar to S. cerevisiae Cdh1, which is an APC/C component; transcriptionally induced by Mnl1 under weak acid stress
PIS50488.1	25.6515534	24.300261	24.2034961	26.33442301	26.08355659	26.61155597	0.008477557	0.839	1.62	PIS50488.1	orf19.3972	Ortholog(s) have role in endoplasmic reticulum to Golgi vesicle-mediated transport, retrograde transport, endosome to Golgi
PIS52077.1	26.9053926	25.2784331	26.686218	27.57257901	28.32706459	27.83119697	0.074792649	0.938	1.62	PIS52077.1	orf19.7546	Protein involved in rRNA processing; required for maturation of the 35S primary transcript of pre-rRNA and for cleavage leading to mature 18S rRNA; Spider biofilm induced
PIS58818.1	27.7658802	27.1307602	24.1442872	28.50394222	27.53333544	27.89286618	0.059832512	0.932	1.63	PIS58818.1	APS3	Component of the adaptor complex AP-3, which is involved in vacuolar protein sorting
PIS51299.1	27.0292348	25.8991395	25.3660786	28.27850128	27.55846294	27.33789701	0.091787016	0.943	1.63	PIS51299.1	CMD1	Calmodulin; calmodulin inhibitors cause a defect in hyphal growth; transcript not regulated by yeast-hyphal transition; mutation confers hypersensitivity to cytochalasin D and high concentrations of tunicamycin; gene has intron

PIS50332.1	29.7977116	29.9621571	29.5247979	32.71979342	31.1429136	30.30426075	0.009808031	0.849	1.63	PIS50332.1	orf19.4932	Ortholog(s) have role in mitochondrial translation and mitochondrion localization
PIS55022.1	24.8324173	23.6619241	25.8136376	27.0994989	26.12914929	25.95663792	0.005127114	0.797	1.63	PIS55022.1	orf19.6056	Ortholog(s) have phosphatase activity and role in dephosphorylation
PIS56713.1	24.9938201	25.3826376	22.3806898	25.70442393	26.20474219	25.73378564	0.067337938	0.935	1.63	PIS56713.1	orf19.7672	Ortholog(s) have protein folding chaperone, protein-containing complex binding activity
PIS56650.1	27.0395155	28.0249269	26.3735026	29.81510449	28.10577322	28.43643096	0.068916236	0.936	1.64	PIS56650.1	orf19.1531	Ortholog(s) have RNA binding activity and role in co-transcriptional mRNA 3'-end processing, cleavage and polyadenylation pathway, mRNA processing, response to DNA damage checkpoint signaling
PIS56767.1	27.5521278	26.5375545	26.4959126	28.61465842	28.50123926	28.37875516	0.000177809	0.272	1.64	PIS56767.1	orf19.956	Ortholog(s) have role in fermentation, phospholipid homeostasis, protein maturation
PIS58955.1	24.6426285	25.0824558	26.6975703	27.07022424	26.96396965	27.29681703	0.145858967	0.953	1.64	PIS58955.1	ZCF1	Zn(II)2Cys6 transcription factor; transcript regulated during hypha formation; 5'-UTR intron; mutants show decreased colonization of mouse kidneys; flow model biofilm induced; Spider biofilm induced
PIS58895.1	26.0707974	26.7776533	25.4844408	29.329752	26.88351501	27.05844315	0.014132167	0.872	1.65	PIS58895.1	orf19.1900	Ortholog(s) have RNA methyltransferase activity and role in 7-methylguanosine cap hypermethylation, RNA methylation, meiotic cell cycle, nucleologenesis, regulation of telomere maintenance via telomerase, tRNA processing

PIS51116.1	27.1550594	27.0147525	25.78978	27.6758162	28.32094036	28.95722981	0.008305056	0.837	1.66	PIS51116.1	FDH1	Formate dehydrogenase; oxidizes formate to CO2; Mig1 regulated; induced by macrophages; fluconazole-repressed; repressed by Efg1 in yeast, not hyphal conditions; stationary phase enriched; rat catheter and Spider biofilm induced
PIS51387.1	26.6985969	27.4758871	27.0457859	28.85261173	28.04789371	29.29542416	0.005959563	0.811	1.66	PIS51387.1	MRV2	Protein of unknown function; repressed by fluphenazine treatment or in an azole-resistant strain that overexpresses CDR1 and CDR2; Spider biofilm induced
PIS51182.1	27.6136064	26.4198751	25.8869573	29.14738431	27.69737258	28.06711422	0.007110241	0.825	1.66	PIS51182.1	orf19.7578	Ortholog(s) have oligosaccharide binding activity and role in endoplasmic reticulum unfolded protein response, retrograde protein transport, ER to cytosol, ubiquitin-dependent ERAD pathway, ubiquitin-dependent glycoprotein ERAD
PIS51176.1	23.2009916	27.0901563	24.4654484	26.73615453	26.71505895	26.28542853	0.031830084	0.911	1.66	PIS51176.1	SPT7	Putative SAGA transcriptional regulatory complex subunit; mutation confers hypersensitivity to toxic ergosterol analog, and to amphotericin B
PIS51233.1	28.0632175	27.8251264	27.7769231	30.31375221	29.3098303	29.06105433	0.064588498	0.934	1.67	PIS51233.1	ARE2	Acyl CoA:sterol acyltransferase; uses cholesterol and oleoyl-CoA substrates; protoberberine derivative drug inhibits enzyme activity; ketoconazole-induced; Hap43-repressed; flow model biofilm induced; Spider biofilm induced
PIS54959.1	30.16575	29.9195264	29.2824066	31.85769274	31.47309967	31.04681242	0.000259113	0.333	1.67	PIS54959.1	FTR2	High-affinity iron permease; probably interacts with ferrous oxidase; regulated by iron level, ciclopirox olamine, amphotericin B, caspofungin; complements S. cerevisiae ftr1 iron transport defect; Hap43-repressed; Spider biofilm induced
PIS51333.1	27.7029531	26.8502062	23.7414687	27.56791607	27.90162341	27.82127308	0.049792423	0.927	1.67	PIS51333.1	orf19.4726	Ortholog(s) have 1-phosphatidylinositol 4-kinase activator activity, calcium ion binding, enzyme activator activity

PIS56946.1	21.3335752	24.3503728	26.0530191	26.48701311	25.45755612	24.8300802	0.235201058	0.961	1.68	PIS56946.1	AKL1	Putative serine/threonine protein kinase; induced during the mating process
PIS51135.1	25.5432313	22.971151	24.9061116	27.35947934	25.48046463	25.61225959	0.017775038	0.885	1.68	PIS51135.1	MET2	Homoserine acetyltransferase; Hap43p-, Gcn4p-regulated; macrophage/pseudohyphal-repressed; not highly biofilm induced, in contrast to many sulfur amino acid metabolic genes; no human or murine homolog; virulence-group-correlated expression
PIS58925.1	31.4306388	31.7793875	32.490662	33.31839409	33.88136623	33.55319287	0.000361903	0.391	1.68	PIS58925.1	MET6	Essential 5-methyltetrahydropteroyltriglutamate-homocysteine methyltransferase (cobalamin-independent methionine synthase); antigenic in murine/human systemic infection; heat shock, estrogen, GCN-induced; Spider biofilm
PIS54646.1	24.9815391	23.4007799	23.2474202	24.61799264	26.13200065	25.9179629	0.06177296	0.933	1.68	PIS54646.1	orf19.201	Protein of unknown function; unmerged from orf19.202 in a revision of Assembly 21
PIS55543.1	24.1969144	27.7203556	27.703271	28.51243642	28.11213359	28.03592598	0.078279535	0.939	1.68	PIS55543.1	orf19.2092	Putative peroxisomal cystathionine beta-lyase; Gcn4p-regulated
PIS51353.1	25.0544465	25.0318638	25.123754	27.10639962	26.7499215	26.38184892	0.03195876	0.911	1.68	PIS51353.1	ZCF14	Putative Zn(II)2Cys6 transcription factor; caspofungin induced
PIS51660.1	27.1734136	26.7535495	27.1388991	29.51362485	28.10131022	28.50879342	0.005256027	0.799	1.69	PIS51660.1	CDC73	Putative transcription elongation factor; cdc73 kap114 double transposon mutation affects filamentous growth; Spider biofilm repressed

PIS50314.1	31.3488885	31.8504615	31.9418762	33.80003094	33.00532067	33.4000225	0.006892243	0.823	1.69	PIS50314.1	KRE30	YEF3-subfamily ABC family protein; predicted not to be a transporter; repressed in core stress response; mutation confers hypersensitivity to amphotericin B
PIS55652.1	28.4254798	27.9500029	27.4867559	29.41099282	29.21162515	30.29472407	0.000422889	0.421	1.69	PIS55652.1	LYS4	Homoaconitase; regulated by Gcn4, Gcn2; induced in response to amino acid starvation (3-AT); induced by human whole blood or PMNs; Hap43-repressed; flow model and Spider biofilm repressed
PIS51434.1	24.5592691	24.7486554	22.2202691	24.9990727	25.69562474	25.90617258	0.012745018	0.866	1.69	PIS51434.1	orf19.1433	Protein of unknown function; Hap43-repressed; colony morphology-related gene regulation by Ssn6; Spider biofilm induced
PIS51929.1	25.6076698	24.8869259	25.2777851	27.01922778	28.10574965	25.72268661	0.018912685	0.888	1.69	PIS51929.1	orf19.36	Component of the conserved oligomeric Golgi complex; predicted to mediate fusion of transport vesicles to Golgi compartments; clade-associated gene expression
PIS54665.1	28.215803	27.6707436	24.9604591	28.88232856	28.4693397	28.55770434	0.029055093	0.907	1.69	PIS54665.1	orf19.6979	Ortholog(s) have role in intracellular magnesium ion homeostasis, intracellular manganese ion homeostasis, mitochondrion organization
PIS48387.1	27.9607594	28.385324	27.8808929	31.10550008	29.45001768	28.77810859	0.020502776	0.892	1.7	PIS48387.1	CHS2	Chitin synthase; nonessential; required for wild-type chitin deposition in hyphae; transcript regulated during dimorphic transition; Chs1 and Chs2, but not Chs3, are inhibited by the protoberberine HWY-289; flow model biofilm repressed
PIS58805.1	26.6941839	25.1297368	26.6590893	27.57417266	26.55872255	29.46288577	0.019959123	0.891	1.7	PIS58805.1	CTR2	Putative low-affinity copper transporter of the vacuolar membrane; induced by nitric oxide; clade-associated gene expression; rat catheter and flow model biofilm induced

PIS58795.1	24.1855181	23.9386949	23.1328857	26.62713952	25.48342347	24.25032958	0.042604384	0.921	1.7	PIS58795.1	orf19.2320	Putative serine/threonine-protein kinase; possibly an essential gene, disruptants not obtained by UAU1 method
PIS48400.1	25.9114115	27.6966633	27.8976607	27.56412535	29.5391008	29.49664874	0.024403991	0.9	1.7	PIS48400.1	orf19.6553	Membrane-localized protein of unknown function; possibly secreted; fluconazole-induced
PIS56730.1	24.8065895	23.6745683	23.9600452	26.03520089	26.2752764	25.23577868	0.016990574	0.882	1.7	PIS56730.1	RCA1	Protein involved in regulation of carbonic anhydrases; controls CO2 sensing; bZIP domain-containing transcription factor of the ATF/CREB family; null mutant displays slow growth, abnormal colony morphology and invasive growth
PIS58315.1	24.3097877	24.9194133	24.7200164	25.12433226	26.85509431	27.10614683	0.002984071	0.74	1.71	PIS58315.1	orf19.3887	Ortholog of S. cerevisiae : YML108W, C. glabrata CBS138 : CAGL0J06666g, C. dubliniensis CD36 : Cd36_31830, C. parapsilosis CDC317 : CPAR2_204870 and C. auris B8441 : B9J08_000809
PIS52009.1	27.2377015	25.4267367	25.7757857	28.01622188	27.78346508	27.8306412	0.011561623	0.86	1.73	PIS52009.1	DIP2	Putative small ribonucleoprotein complex; Tn mutation affects filamentous growth; physically interacts with TAP-tagged Nop1; heterozygous null mutant exhibits resistance to parnafungin; Hap43-induced gene; Spider biofilm induced
PIS54853.1	26.769377	26.130227	23.3199708	27.51424809	27.70342979	26.18411345	0.044538167	0.923	1.73	PIS54853.1	orf19.4479	Putative U3-containing 90S preribosome subunit; Hap43-induced; repressed in core stress response; Spider biofilm induced
PIS52344.1	27.2215908	27.4600892	27.1912484	28.90478376	29.05142647	29.11011266	0.000246808	0.325	1.73	PIS52344.1	orf19.5920	Ortholog of C. dubliniensis CD36 : Cd36_84570, C. parapsilosis CDC317 : CPAR2_404420, C. auris B8441 : B9J08_003958 and Candida tenuis NRRL Y-1498 : CANTEDRAFT_115339

PIS56822.1	23.423816	24.8678575	20.5458382	24.25146671	24.85900159	24.93614637	0.101301137	0.945	1.74	PIS56822.1	ARG81	Zn(II)2Cys6 transcription factor; required for utilization of ornithine as a nitrogen source and for wild-type resistance to caffeine; required for yeast cell adherence to silicone substrate
PIS50383.1	28.8937858	28.0919121	28.7871149	30.43129932	30.25870636	30.30150792	0.013856316	0.871	1.74	PIS50383.1	orf19.2196	Ortholog(s) have K63-linked polyubiquitin modification-dependent protein binding, ubiquitin binding activity, role in rescue of stalled ribosome and cytosolic ribosome localization
PIS49532.1	25.799862	25.2266754	26.6126606	27.83694975	27.14334582	27.87116109	0.012542966	0.865	1.74	PIS49532.1	orf19.4731	Ortholog(s) have role in cytoplasm to vacuole transport by the Cvt pathway, intra-Golgi vesicle-mediated transport and Golgi transport complex localization
PIS58673.1	26.7060689	25.5688996	26.725715	28.62733309	28.77013691	26.82296077	0.029234137	0.908	1.74	PIS58673.1	orf19.6419.1	Ortholog of C. parapsilosis CDC317 : CPAR2_205710, C. auris B8441 : B9J08_000119, Candida tenuis NRRL Y-1498 : CANTEDRAFT_117073 and Debaryomyces hansenii CBS767 : DEHA2A11462g
PIS51542.1	26.3858942	25.6379661	27.355592	28.00340971	27.93212297	28.67727868	0.005523856	0.804	1.74	PIS51542.1	TIM17	Predicted component of the Translocase of the Inner Mitochondrial membrane (TIM23 complex), involved in protein import into mitochondria
PIS49843.1	30.8858263	30.48638	29.4135674	31.9561788	31.69814662	32.42243001	0.006327651	0.816	1.76	PIS49843.1	CUP9	Transcription factor; represses SOK1 expression in response to farnesol inhibition; yeast-hypha switch repressed; ketoconazole-induced; Plc1-regulated; colony morphology-related Ssn6 regulation; Spider, flow model biofilm induced
PIS55511.1	26.3482086	25.5316573	23.6179318	27.38609246	27.29011129	26.11332485	0.04927575	0.926	1.76	PIS55511.1	IRE1	Protein kinase involved in regulation of unfolded protein response; role in cell wall regulation; mutant is hypersensitive to caspofungin; Spider biofilm induced

PIS51941.1	29.0448347	27.5762071	27.4391657	30.20837646	29.94309816	29.20263931	0.002976816	0.74	1.76	PIS51941.1	YWP1	Secreted yeast-cell wall protein; controls exposure of cell wall beta-glucan to host immune system; involved in adhesion and biofilm formation; growth phase, phosphate, Ssk1/Ssn6/Efg1/Efh1/Hap43 regulated
PIS52226.1	24.0347482	22.8505782	24.3980241	27.58427426	24.38573669	24.6213931	0.059196909	0.932	1.77	PIS52226.1	ARO7	Putative chorismate mutase; fungal-specific (no human or murine homolog); alkaline upregulated
PIS50398.1	25.1464939	22.3037968	24.3900691	25.38671793	26.12466368	25.66882727	0.150859529	0.954	1.78	PIS50398.1	GYP1	Putative Cis-golgi GTPase-activating protein; required for hyphen growth and virulence; transcript regulated by Nrg1, Mig1, and Tup1
PIS51467.1	25.5415363	24.8630116	25.4752633	26.85404894	25.42763692	28.95056523	0.036031413	0.916	1.78	PIS51467.1	HNM1	Putative choline/ethanolamine transporter; mutation confers hypersensitivity to toxic ergosterol analog; colony morphology-related gene regulation by Ssn6; clade-associated gene expression
PIS55490.1	31.4012337	31.2427409	30.2456771	32.14895684	32.69737712	33.39394157	0.005690266	0.807	1.78	PIS55490.1	orf19.1152	Protein of unknown function; induced in core stress response; Gcn2 and Gcn4 regulated; flow model biofilm induced; Spider biofilm induced
PIS54493.1	27.6634638	26.5682292	26.1447083	29.66374927	27.90651007	28.1536114	0.105447011	0.946	1.78	PIS54493.1	orf19.349	Ortholog(s) have role in aerobic respiration, mRNA metabolic process
PIS50389.1	23.7147258	23.7491786	23.3413616	25.481154	24.85099416	25.85806401	0.011238612	0.858	1.79	PIS50389.1	ECM1	Putative pre-ribosomal factor; decreased mRNA abundance observed in cyr1 homozygous mutant hyphae; induced by heavy metal (cadmium) stress; Hog1p regulated

PIS58721.1	27.0357332	25.180649	25.6324592	26.22900667	28.2263401	28.75521537	0.163237118	0.955	1.79	PIS58721.1	FTH1	Putative iron permease involved in the production of prostaglandin E2; mutants show decreased metabolic activity in biofilms
PIS55781.1	28.4881374	27.9258997	28.3299306	30.64369988	29.92705022	29.55135721	0.00050056	0.453	1.79	PIS55781.1	HEM1	Putative 5-aminolevulinate synthase; caspofungin repressed; induced by high iron, nitric oxide; regulated by Ssn6; Hap43-repressed; Spider biofilm induced
PIS51329.1	26.7294924	26.3500687	24.7385727	28.40066146	27.89894142	26.89154321	0.00385023	0.769	1.79	PIS51329.1	orf19.6828	Ortholog(s) have role in rRNA processing and preribosome, large subunit precursor localization
PIS54664.1	27.7127845	22.6302586	26.0225903	27.1197756	27.34253812	27.27722793	0.077355706	0.939	1.79	PIS54664.1	orf19.7566	High-specificity proline permease; transcript upregulated in clinical strains from HIV+ patients with oral candidiasis; alkaline upregulated by Rim101; rat catheter, Spider and flow model biofilm induced
PIS48296.1	26.4169054	26.4833488	26.5947392	28.60656695	28.22413506	28.02323598	0.013624012	0.87	1.79	PIS48296.1	PDR16	Phosphatidylinositol transfer protein; induction correlates with CDR1, CDR2 overexpression/azole resistance; fluphenazine, 17-beta-estradiol, ethynyl estradiol, NO induced; farnesol-downregulated in biofilm; rat catheter biofilm induced
PIS51701.1	24.6270257	25.0641661	26.2110457	27.5233295	26.65979649	27.10259666	0.002200498	0.701	1.79	PIS51701.1	PWP1	Putative rRNA processing protein; Hap43-induced; repressed in core stress response
PIS52032.1	26.4127055	24.2024316	24.8838049	28.14787698	25.78021739	26.98036847	0.025616745	0.902	1.8	PIS52032.1	ECM3	Has domain(s) with predicted role in transmembrane transport and membrane localization

PIS54879.1	22.7964364	23.1313635	25.7204944	25.37198403	25.89239865	25.78104594	0.017367828	0.884	1.8	PIS54879.1	GRX3	Putative glutaredoxin; flucytosine induced; regulated by Gcn4p; repressed in response to amino acid starvation (3-aminotriazole treatment)
PIS48510.1	25.0417993	24.097905	25.7295298	26.37888936	26.52214633	27.37048533	0.03636273	0.916	1.8	PIS48510.1	orf19.5291	Ortholog(s) have role in phospholipid biosynthetic process, phospholipid homeostasis, phospholipid metabolic process and endoplasmic reticulum localization
PIS51964.1	26.0572881	23.581988	27.1778353	28.42316471	27.3269503	26.45468372	0.042955315	0.922	1.8	PIS51964.1	orf19.6741	Putative plasma membrane protein; predicted role in cell wall integrity; regulated by Nrg1, Tup1; induced during chlamydospore formation in both C. albicans and C. dubliniensis
PIS51186.1	28.7300431	26.5499422	27.0519869	29.26459838	28.79249405	29.66578563	0.02294012	0.897	1.8	PIS51186.1	orf19.7589	Protein of unknown function; Hap43-repressed gene; transcript induced by elevated CO2
PIS56927.1	22.7542022	24.8353499	25.4319136	26.65543006	25.09787505	26.68548656	0.047717611	0.925	1.81	PIS56927.1	orf19.4029	Ortholog(s) have unfolded protein binding activity, role in ribosomal large subunit assembly, ribosomal large subunit biogenesis and cytosol localization
PIS48224.1	23.9666303	25.303478	24.8886536	26.54484659	26.39951791	26.65388971	0.000769472	0.534	1.81	PIS48224.1	orf19.6440	Ortholog(s) have ubiquitin-protein transferase activity and role in cellular response to amino acid stimulus, proteasome-mediated ubiquitin-dependent protein catabolic process, ubiquitin-dependent protein catabolic process
PIS51556.1	26.1572133	24.6513206	24.1215538	27.08873259	27.00263215	26.29769466	0.018284803	0.886	1.82	PIS51556.1	orf19.2786	Ortholog(s) have AP-2 adaptor complex, cellular bud neck localization

PIS54734.1	20.4707933	22.5563487	25.1838367	24.14990235	24.46466256	25.04352559	0.057306252	0.931	1.82	PIS54734.1	orf19.5014	Ortholog of S. cerevisiae : LCL3, C. glabrata CBS138 : CAGL0H03201g, C. dubliniensis CD36 : Cd36_12760, C. parapsilosis CDC317 : CPAR2_201640 and C. auris B8441 : B9J08_002513
PIS51287.1	27.3570462	26.7474401	26.0495458	28.61559046	28.85787867	28.13839755	0.001233561	0.616	1.82	PIS51287.1	orf19.5394.1	Ortholog(s) have role in mitochondrial cytochrome c oxidase assembly and mitochondrial inner membrane, mitochondrial intermembrane space localization
PIS58124.1	26.4239251	25.9902091	25.0604156	27.37990167	27.48360648	28.05763891	0.00759366	0.831	1.82	PIS58124.1	orf19.6259	Exosome non-catalytic core component; involved in 3'-5' RNA processing and degradation in the nucleus and cytoplasm; Spider biofilm induced
PIS54960.1	26.1730967	26.3993165	23.4311701	27.48150454	27.48145269	26.52081477	0.080908494	0.94	1.83	PIS54960.1	ENG1	Endo-1,3-beta-glucanase; controls exposure of cell wall beta-glucan to host immune system; caspofungin, fluconazole repressed; repressed by alpha pheromone in SpiderM medium; flow model biofilm induced; rat catheter biofilm repressed
PIS51594.1	23.1862218	24.4467319	23.4656439	25.66795822	24.89259839	26.02211255	0.267473954	0.963	1.83	PIS51594.1	orf19.194	Ortholog of C. dubliniensis CD36 : Cd36_19300, C. parapsilosis CDC317 : CPAR2_209720, C. auris B8441 : B9J08_003188 and Candida tenuis NRRL Y-1498 : CANTEDRAFT_114035
PIS49690.1	26.3830812	25.6621797	24.2057427	27.17561007	27.35772173	27.24004933	0.006075107	0.812	1.84	PIS49690.1	orf19.1304	Ortholog(s) have role in U4 snRNA 3'-end processing, exonucleolytic trimming to generate mature 3'-end of 5.8S rRNA from tricistronic rRNA transcript (SSU-rRNA, 5.8S rRNA and LSU-rRNA), more
PIS52299.1	25.3505908	24.6126144	24.0258385	26.17032404	26.53275276	26.79158721	0.016395012	0.881	1.84	PIS52299.1	orf19.7091	Protein of unknown function; induced by nitric oxide; Spider biofilm repressed

PIS48223.1	26.0018049	24.5059425	25.3225052	28.61787204	26.23474363	26.51298496	0.026713038	0.904	1.85	PIS48223.1	PRP8	Protein similar to S. cerevisiae Prp8, a component of the U4/U6-U5 snRNP complex; repressed by alpha pheromone in SpiderM medium
PIS55454.1	25.1695528	25.5077398	19.848897	24.95262457	25.55604347	25.59383578	0.179445845	0.957	1.86	PIS55454.1	BTS1	Putative geranylgeranyl diphosphate synthase; repressed by benomyl treatment; Spider biofilm induced
PIS48410.1	24.315607	24.2411834	22.8996982	25.91279328	25.37672755	25.75765228	0.020008687	0.891	1.86	PIS48410.1	orf19.3517	Ortholog(s) have role in SREBP signaling pathway and Dsc E3 ubiquitin ligase complex, endoplasmic reticulum, fungal-type vacuole lumen localization
PIS52379.1	26.7623614	26.3507471	23.5132982	27.7442143	28.63727291	25.85629951	0.051656356	0.928	1.87	PIS52379.1	NPR2	Putative urea transporter; induced during infection of murine kidney, compared to growth in vitro; has murine homolog
PIS52120.1	25.3101117	26.3337995	26.7164892	27.04369428	28.47563683	28.4389014	0.009510443	0.847	1.87	PIS52120.1	orf19.1592	Protein of unknown function; Spider biofilm induced
PIS58257.1	23.0084902	22.5844895	21.6735682	23.94716868	24.69998971	24.22723428	0.00140338	0.636	1.87	PIS58257.1	orf19.2528	Putative RNA polymerase III transcription factor (TFIIIB) subunit; flucytosine repressed
PIS54725.1	23.2570244	23.9853113	23.8552845	25.89823401	25.25183528	25.55918997	0.013410639	0.869	1.87	PIS54725.1	orf19.6356	Ortholog(s) have role in mRNA splicing, via spliceosome and U4/U6 snRNP, U4/U6 x U5 tri-snRNP complex localization

PIS50409.1	27.6187695	27.0012903	25.9735438	29.50758874	28.45599856	28.27606714	0.000928227	0.567	1.88	PIS50409.1	PHO15	HAD-family 2-phosphoglycolate phosphatase, likely involved in a metabolic repair system, not in protein dephosphorylation; involved in regulation of white-opaque switch; hyphal repressed; induced in core stress response
PIS54539.1	25.245572	23.4418968	23.7122482	25.94206878	25.9949155	26.11112026	0.005662511	0.806	1.88	PIS54539.1	VPS11	Protein involved in protein trafficking; putative role in vesicle-target membrane fusion; mutant lacks vacuole; role in hyphal growth, possibly via vacuole expansion into hypha; role in killing of and survival within macrophage
PIS51077.1	27.3352235	27.1178863	26.4752535	28.80966812	28.70656978	29.06733231	0.000293163	0.355	1.89	PIS51077.1	orf19.2378	Ortholog(s) have role in vacuolar proton-transporting V-type ATPase complex assembly and endoplasmic reticulum membrane localization
PIS58566.1	27.1531494	24.5854989	26.1608513	27.90211191	28.1653235	27.50466724	0.009645418	0.848	1.89	PIS58566.1	orf19.4611	Ortholog(s) have ribose phosphate diphosphokinase activity, role in 5-phosphoribose 1-diphosphate biosynthetic process, fungal-type cell wall organization and ribose phosphate diphosphokinase complex localization
PIS55484.1	28.7499116	27.4617385	25.5483006	28.99999079	28.9629478	29.48149168	0.04625153	0.924	1.89	PIS55484.1	PET100	Chaperone that facilitates the assembly of cytochrome c oxidase; plasma membrane protein
PIS52407.1	23.13523	23.99219	22.4564421	25.05310398	24.82602599	25.41028902	0.005933614	0.81	1.9	PIS52407.1	orf19.4878	Protein of unknown function; Hap43-repressed; rat catheter biofilm repressed
PIS49809.1	25.0561892	25.7486028	25.1257267	26.10973264	28.02077627	27.49077423	0.018062204	0.886	1.9	PIS49809.1	orf19.551	Ortholog of C. dubliniensis CD36 : Cd36_29980, C. parapsilosis CDC317 : CPAR2_204170, C. auris B8441 : B9J08_004836 and Candida tenuis NRRL Y-1498 : CANTEDRAFT_110235

PIS48739.1	22.4962776	26.3389246	22.1328443	25.1893689	25.59249978	25.92366687	0.040857131	0.92	1.91	PIS48739.1	orf19.2149	Putative sulfate permease; S. cerevisiae ortholog YPR003C localizes to the endoplasmic reticulum; regulated by Sef1p-, Sfu1p-, and Hap43p
PIS51133.1	27.1727221	26.5766134	25.6999858	28.1161034	28.44784929	28.6479043	0.012035168	0.863	1.92	PIS51133.1	MDL1	Putative mitochondrial, half-size MDR-subfamily ABC transporter
PIS48619.1	21.5533036	25.2096365	26.2974149	26.99106248	26.30310446	25.53500219	0.071217482	0.937	1.92	PIS48619.1	orf19.7499	Putative nicotinic acid mononucleotide adenylyltransferase, involved in NAD salvage pathway; Spider biofilm repressed
PIS58053.1	26.7432516	26.1435406	25.6163976	28.63208722	28.09747283	27.52101707	0.009086802	0.844	1.92	PIS58053.1	orf19.7615	Protein involved in endoplasmic reticulum (ER) to Golgi vesicle-mediated transport; putative subunit of the transport protein particle (TRAPP) complex of the cis-Golgi; Spider biofilm induced
PIS48299.1	24.8990666	24.4795946	24.352434	26.45147886	26.5018057	26.58108906	0.00221335	0.702	1.93	PIS48299.1	BNI4	Protein required for wild-type cell wall chitin distribution, morphology, hyphal growth; not essential; similar to S. cerevisiae Bni4p (targeting subunit for Glc7p phosphatase, involved in bud-neck localization of chitin synthase III)
PIS51281.1	23.302017	23.095198	22.9831141	27.24034318	23.88843807	24.035114	0.018963924	0.888	1.93	PIS51281.1	CHS8	Chitin synthase required for synthesis of long-chitin fibrils; nonessential; 8 or 9 membrane spanning regions; mRNA present in yeast and hyphae; induced during cell wall regeneration; flow model biofilm repressed
PIS50579.1	27.4333202	25.6868732	23.4915578	26.83618179	28.1638792	27.39528644	0.029985962	0.909	1.93	PIS50579.1	MMM1	Subunit of ER-mitochondrion tether ERMES complex, required for evasion of host immune response

PIS58905.1	25.4989191	24.4393425	23.1030097	27.13299366	25.19774673	26.48857112	0.224074754	0.961	1.93	PIS58905.1	orf19.1723	Ortholog(s) have role in response to purine-containing compound
PIS48267.1	28.4945255	27.2353608	26.487743	29.08105684	29.77374742	29.154469	0.018433412	0.887	1.93	PIS48267.1	orf19.2996	Ortholog of S. cerevisiae : ECM30, C. glabrata CBS138 : CAGL0M00924g, C. dubliniensis CD36 : Cd36_02850, C. parapsilosis CDC317 : CPAR2_110240 and C. auris B8441 : B9J08_004952
PIS49824.1	26.5556688	25.708128	25.6610887	29.25876514	26.68446024	27.75753721	0.017926399	0.885	1.93	PIS49824.1	SGD1	Predicted small ribosomal subunit biogenesis protein; repressed in core stress response; transcript increases in populations of cells exposed to fluconazole over multiple generations; Spider biofilm induced
PIS54690.1	27.9093519	26.1469783	26.7132719	28.9926966	28.90982132	28.70034891	0.00274276	0.73	1.94	PIS54690.1	CPA2	Putative arginine-specific carbamoylphosphate synthetase; protein enriched in stationary phase yeast cultures; rat catheter biofilm induced; Spider biofilm induced
PIS48737.1	22.3343674	23.3834954	22.4383806	23.96128176	24.20061943	25.80872812	0.012691585	0.866	1.94	PIS48737.1	orf19.6205	Ortholog of C. dubliniensis CD36 : Cd36_06550, C. parapsilosis CDC317 : CPAR2_209030, C. auris B8441 : B9J08_005439 and Candida tenuis NRRL Y-1498 : CANTEDRAFT_111035
PIS51875.1	25.726574	24.9382206	20.1741828	26.07923381	24.90647912	25.7230308	0.102053322	0.946	1.96	PIS51875.1	DAL81	Zn(II)2Cys6 transcription factor; ortholog of S. cerevisiae Dal81, involved in the regulation of nitrogen-degradation genes; required for yeast cell adherence to silicone substrate; Spider biofilm induced
PIS49849.1	25.4664734	26.5320695	27.2216238	29.16602177	27.82953389	28.10581417	0.000635803	0.499	1.96	PIS49849.1	LEA1	Predicted component of U2 snRNP; induced by alpha pheromone in SpiderM medium

PIS58472.1	22.6987469	23.5820203	23.5357172	25.39734515	24.97638722	25.31259197	0.053559106	0.929	1.96	PIS58472.1	NUP159	FG-nucleoporin component of central core of the nuclear pore complex (NPC); contributes to nucleocytoplasmic transport; rat catheter biofilm repressed
PIS55570.1	31.3957312	29.0778735	28.9826566	32.35492254	31.38458926	31.63418269	0.008086076	0.835	1.97	PIS55570.1	NOP5	Ortholog of S. cerevisiae Nop58; involved in pre-rRNA process; Tn mutation affects filamentous growth; macrophage/pseudohyphal-induced; physically interacts with TAP-tagged Nop1; Spider biofilm repressed
PIS55805.1	26.7790804	25.8431006	26.8840033	29.14624549	28.12651794	28.15318911	0.002619081	0.724	1.97	PIS55805.1	orf19.2436	SR-like protein kinase involved in osmotic stress and polyamine resistance; expression analysis suggests roles in mRNA processing and mitochondrial function; Spider biofilm induced; null mutant shows resistance to hygromycin B
PIS51657.1	24.2414055	23.9836289	23.8902117	26.54680025	25.77742948	25.68944359	0.000103783	0.211	1.97	PIS51657.1	orf19.2986	Ortholog of C. dubliniensis CD36 : Cd36_02750, C. parapsilosis CDC317 : CPAR2_106015, C. auris B8441 : B9J08_003254 and Candida tenuis NRRL Y-1498 : CANTEDRAFT_107500
PIS54684.1	25.3539599	24.5078218	24.7419899	26.57175805	26.9317976	27.02622012	0.000729229	0.524	1.98	PIS54684.1	orf19.775	Ortholog of C. dubliniensis CD36 : Cd36_04450, C. parapsilosis CDC317 : CPAR2_105460, C. auris B8441 : B9J08_002463, Debaryomyces hansenii CBS767 : DEHA2D07128g and Pichia stipitis Pignal : PICST_80203
PIS48633.1	24.5358236	24.3887509	26.3481077	26.98284768	27.41591242	26.90918569	0.00215425	0.699	2.01	PIS48633.1	ASR3	Adenylyl cyclase and stress responsive protein; induced in cyr1 or ras1 mutant; Spider biofilm induced
PIS48643.1	26.5050418	24.506285	26.0996299	27.43331639	27.27524033	28.43256107	0.006710411	0.821	2.01	PIS48643.1	CAP1	AP-1 bZIP transcription factor; apoptotic, oxidative stress response/resistance, multidrug resistance; nuclear in oxidative stress; complements S. cerevisiae yap1 mutant; oralpharyngeal candidasis-, human neutrophil, Spider biofilm [illegible]

PIS51492.1	29.9189385	24.988569	25.2675521	28.98676901	28.41438556	28.79576144	0.109212002	0.947	2.01	PIS51492.1	orf19.5828	Ortholog(s) have pre-mRNA branch point binding activity and role in mRNA branch site recognition, mRNA cis splicing, via spliceosome, spliceosomal complex assembly
PIS52045.1	24.3199179	24.6890837	25.8998485	27.51621422	26.52389972	26.9568685	0.014954578	0.876	2.03	PIS52045.1	orf19.1544	Putative cis-golgi localized protein involved in ER to Golgi transport; Spider biofilm repressed
PIS51796.1	25.3915644	23.7646696	23.2426796	27.16007268	26.84881013	24.48716587	0.01629195	0.88	2.03	PIS51796.1	orf19.6220	Ortholog(s) have role in positive regulation of transcription elongation by RNA polymerase II and CCR4-NOT core complex localization
PIS51414.1	25.5022467	24.7438486	26.1126477	27.56642966	27.8928569	27.02080755	0.002856107	0.735	2.04	PIS51414.1	MET14	Putative adenylylsulfate kinase; predicted role in sulfur metabolism; possibly adherence-induced; protein present in exponential and stationary growth phase yeast; F-12/CO2 biofilm induced
PIS56723.1	24.3892779	24.6842366	25.2516513	27.05648756	26.56934596	26.83002002	0.012256676	0.864	2.04	PIS56723.1	orf19.7664	Has domain(s) with predicted DNA binding activity
PIS58367.1	23.5239409	24.4521279	25.091732	26.17792976	27.12054678	25.87755948	0.01744151	0.884	2.04	PIS58367.1	orf19.804	Putative mitochondrial carrier family transporter; rat catheter biofilm induced
PIS54791.1	24.4086169	24.0554139	23.6661615	26.69240241	25.64206454	25.95639495	0.003558988	0.76	2.05	PIS54791.1	APN1	Ortholog of S. cerevisiae Apn1; an AP endonuclease; transcript induced by interaction with macrophages; fungal-specific (no human or murine homolog)

PIS55795.1	24.1157988	24.8025179	25.3550248	26.85546654	26.85905888	26.70235708	0.000151969	0.254	2.05	PIS55795.1	UTR2	Putative GPI anchored cell wall glycosidase; role in adhesion, hyphal growth on Spider (not serum) medium; chitin-binding, glycosyl hydrolase domains; induced during cell wall regeneration; mRNA in yeast-form cells; Spider biofilm induced
PIS58243.1	20.648574	25.471739	26.2085166	26.43263573	26.7906127	25.29341212	0.164851827	0.955	2.06	PIS58243.1	orf19.5161	Ortholog(s) have structural constituent of ribosome activity and mitochondrial large ribosomal subunit localization
PIS48792.1	26.5326577	25.503059	24.2215613	27.92749956	27.94010579	26.57841959	0.014586963	0.874	2.06	PIS48792.1	orf19.6681	Protein of unknown function; Spider biofilm induced
PIS54631.1	29.1100674	29.6293815	27.9728152	30.22526176	31.10745528	31.57049758	0.014172765	0.873	2.06	PIS54631.1	OYE2	Putative NAPDH dehydrogenase; induced by nitric oxide; Spider biofilm induced
PIS54996.1	30.7923124	29.8849983	29.6643823	32.2054927	32.09401582	32.21015878	8.28E-05	0.187	2.06	PIS54996.1	YCF1	Putative glutathione S-conjugate transporter; MRP/CFTR-subfamily, ABC type transporter; human neutrophil-induced; oxidative stress-induced via Cap1; possible association with multidrug resistance; possibly essential; Spider biofilm induced
PIS52423.1	27.4700183	27.2060411	26.6652427	29.25733112	29.04619267	29.25001764	9.89E-05	0.206	2.07	PIS52423.1	CDL1	Putative RNAse III, ortholog of S. cerevisiae RNT1; merged with orf19.3772 in Assembly 21
PIS58851.1	23.8066747	24.609122	24.4167974	25.65917933	25.98466307	27.40923394	0.000475469	0.444	2.07	PIS58851.1	PTP2	Predicted protein tyrosine phosphatase; involved in regulation of MAP kinase Hog1 activity; induced by Mnl1 under weak acid stress; rat catheter and Spider biofilm induced

PIS55655.1	28.8966345	28.6067878	28.168984	30.66863092	30.56895053	30.62988945	0.000348038	0.384	2.07	PIS55655.1	SCS7	Putative ceramide hydroxylase; regulated by Nrg1; induced in high iron; fluconazole-induced; Hap43-repressed; Spider biofilm induced
PIS56963.1	26.9819869	27.5616696	26.7229838	30.28313371	28.41011544	28.84543358	0.006381354	0.817	2.09	PIS56963.1	DRS1	Putative nucleolar DEAD-box protein; Hap43-induced; mutation confers hypersensitivity to 5-fluorouracil (5-FU), tubercidin (7-deazaadenosine); Tbf1-induced; repressed in core stress response
PIS50423.1	22.6634964	24.2269916	24.0571443	25.85907846	25.93742006	25.46008566	0.001240652	0.616	2.1	PIS50423.1	orf19.3237	Ortholog(s) have proteasome regulatory particle binding, ubiquitin protein ligase activity and role in free ubiquitin chain polymerization, protein K29-linked ubiquitination, ubiquitin-dependent protein catabolic process
PIS54773.1	27.2085713	28.1530185	27.7539443	30.00023411	30.17967821	29.24481781	0.00968248	0.848	2.1	PIS54773.1	orf19.4228	Protein with a role in insertion of tail-anchored proteins into the ER membrane; Spider biofilm repressed
PIS58732.1	27.28857	25.0857687	26.0589952	28.56022237	28.09697169	28.14871529	0.008357255	0.838	2.12	PIS58732.1	CAS4	RAM cell wall integrity signaling network protein; cell separation, azole sensitivity; needed for hyphal growth; insertion mutation near 3' end of gene increases caspofungin sensitivity; pheromone/hyphal induced; flow biofilm repressed
PIS51836.1	27.595381	28.4164556	29.1398036	29.52064658	30.90448203	31.07723662	0.00765686	0.831	2.12	PIS51836.1	PRX1	Thioredoxin peroxidase; transcriptionally induced by interaction with macrophage; fluconazole induced; Fkh2p-downregulated; caspofungin repressed; protein present in exponential and stationary growth phase yeast cultures
PIS55640.1	19.7886306	23.7373108	25.4395888	25.20531138	24.71647601	25.48808159	0.079850221	0.94	2.15	PIS55640.1	orf19.1662	Ortholog(s) have structural constituent of ribosome activity and mitochondrial small ribosomal subunit localization

PIS49661.1	24.8124849	22.0693211	23.7444163	25.07229409	26.54580678	25.4612815	0.02151376	0.894	2.15	PIS49661.1	orf19.529	Ortholog of C. dubliniensis CD36 : Cd36_29750, C. parapsilosis CDC317 : CPAR2_205260, C. auris B8441 : B9J08_005543 and Candida tenuis NRRL Y-1498 : CANTEDRAFT_91782
PIS55699.1	26.1209571	26.3394668	25.3896854	28.04985288	28.03061803	28.2593265	0.002136312	0.697	2.16	PIS55699.1	orf19.2890	Has domain(s) with predicted pyrimidine nucleotide-sugar transmembrane transporter activity, role in pyrimidine nucleotide-sugar transmembrane transport and Golgi membrane, membrane localization
PIS48601.1	22.5755111	23.5080617	23.7803736	26.09261477	25.14074174	25.10155086	0.000291752	0.354	2.16	PIS48601.1	PET127	Protein involved in maturation of mitochondrial RNA with a predicted 5'-to-3' exoribonuclease activity; member of the PD-(D/E)XK superfamily; ortholog of S. cerevisiae Pet127; Hap43-induced; rat catheter and Spider biofilm induced
PIS52414.1	26.8392405	26.1855959	26.0598827	28.4992145	28.67082638	28.43744587	0.02818012	0.906	2.17	PIS52414.1	TPK1	cAMP-dependent protein kinase (PKA) catalytic subunit; isoform of Tpk2; involved in regulation of filamentation, phenotypic switching and mating; WT nuclear localization requires Bcy1; produced during stationary, not exponential growth [illegible]
PIS51953.1	27.3690491	28.1366212	26.3563921	31.16535433	28.5354558	28.71574312	0.007974351	0.834	2.18	PIS51953.1	NOP13	Ortholog of S. cerevisiae Nop13; a nucleolar protein found in preribosomal complexes; Hap43-induced gene; rat catheter biofilm induced
PIS58414.1	25.1126961	24.2369412	24.2712216	26.72366058	26.46642511	26.99272206	0.002891993	0.736	2.19	PIS58414.1	orf19.2915	[illegible] Ortholog(s) have ADP phosphatase activity, ATPase, CDP phosphatase activity, GDP phosphatase activity, GTPase activity, UDP phosphatase activity, nucleoside diphosphate phosphatase activity, ribonucleoside triphosphate phosphatase activity [illegible]
PIS48706.1	23.3107462	23.1927678	23.5198848	25.06183318	25.29984206	26.23848055	0.011185961	0.858	2.19	PIS48706.1	orf19.3449	Ortholog(s) have stalled ribosome sensor activity, ubiquitin protein ligase activity

PIS58338.1	21.5015842	20.301153	21.7069186	24.81273979	22.96547916	22.3081202	0.133023538	0.951	2.19	PIS58338.1	orf19.7085	Protein of unknown function; induced in core stress response; induced by cadmium stress via Hog1; oxidative stress-induced via Cap1; induced by Mnl1 under weak acid stress; macrophage-repressed; rat catheter and Spider biofilm induced
PIS58190.1	26.4642729	25.3862761	23.2334559	27.82242635	26.59562828	27.27270273	0.01163963	0.861	2.2	PIS58190.1	orf19.5175	Putative ubiquitin protein ligase with ubiquitin-protein transferase activity; null mutant is sensitive to proteotoxic stress and shows differential expression of other proteins likely involved in ER-associated protein catabolism
PIS51668.1	27.3544459	25.9031644	23.4989727	27.77348652	27.68425928	27.90851762	0.012294252	0.864	2.2	PIS51668.1	PDE2	High affinity cyclic nucleotide phosphodiesterase; moderates signaling by cAMP; required for virulence, switching, cell wall, hyphal, not pseudohyphal growth; expressed shortly after hyphal induction; rat catheter and Spider biofilm induced
PIS48736.1	26.3948285	25.0141314	23.4572038	27.06719854	27.03786272	27.38066811	0.049472646	0.926	2.21	PIS48736.1	PUT3	Zn(II)2Cys6 transcription factor; has similarity to S. cerevisiae Put3, a transcription factor involved in the regulation of proline utilization genes
PIS48490.1	26.7703649	27.9182792	25.6472489	28.9097897	28.89633356	29.20375119	0.003236262	0.749	2.22	PIS48490.1	APE3	Putative vacuolar aminopeptidase Y,; regulated by Gcn2 and Gcn4; rat catheter and Spider biofilm repressed
PIS55669.1	23.8129348	23.479573	24.1202315	26.39564566	26.76046343	24.93349993	0.00044699	0.432	2.23	PIS55669.1	orf19.1574	Putative TFIID subunit involved in RNA polymerase II transcription initiation; possibly an essential gene, disruptants not obtained by UAU1 method
PIS54492.1	21.6615259	21.190996	21.0453646	23.73898505	22.78688952	24.07759729	0.094632612	0.944	2.24	PIS54492.1	TRY4	C2H2 transcription factor; fluconazole-repressed; induced in ssr1 mutant; required for yeast cell adherence to silicone substrate; Spider biofilm induced

PIS56577.1	25.7474968	25.569603	23.8912739	27.90356778	27.10883371	26.95058071	0.005784615	0.808	2.25	PIS56577.1	orf19.102	Protein required for expression of mitochondrial ATP synthase and cytochrome c oxidase (respiratory chain complex IV)
PIS58733.1	23.5560077	26.6653289	27.4189329	26.1887979	28.98084878	29.21798327	0.035458215	0.915	2.25	PIS58733.1	orf19.1697	Ortholog(s) have role in cytoplasmic translation, poly(A)+ mRNA export from nucleus and cytoplasm localization
PIS58305.1	31.488914	29.0029071	28.2872921	32.38842237	31.5024207	31.67148362	0.011758737	0.861	2.26	PIS58305.1	orf19.3869	Protein of unknown function; regulated by Tsa1, Tsa1B in minimal media at 37 degrees C; shows colony morphology-related gene regulation by Ssn6; Spider biofilm induced
PIS48764.1	24.6605974	23.7910673	23.1746994	25.36696211	25.97537064	27.07106674	0.001398979	0.636	2.26	PIS48764.1	orf19.6463	Putative ortholog of S. cerevisiae Npa3p; possibly essential for growth (however, depletion mutant is viable)
PIS49611.1	20.8706481	22.1537838	23.5236185	22.88540286	25.29035771	25.17699185	0.148068539	0.953	2.27	PIS49611.1	COX19	Putative cytochrome c oxidase assembly protein; Plc1-regulated; rat catheter biofilm induced
PIS54685.1	26.7379645	25.329361	23.4764023	28.03080259	26.92882609	27.38710131	0.006081711	0.812	2.27	PIS54685.1	FTH2	Putative iron permease involved in virulence and production of prostaglandin E2; mutants show decreased hyphal growth against C. elegans and abnormal biofilm metabolism and morphology
PIS51042.1	25.4368986	24.4220565	23.898869	26.84072574	26.79584135	26.94262572	0.001438198	0.64	2.27	PIS51042.1	orf19.7506	Ortholog(s) have ATPase, DNA binding, nucleosome binding activity, role in chromatin remodeling and Isw1b complex localization

PIS55825.1	22.6267915	22.606585	21.1178815	24.17136461	23.93272604	25.07208625	0.05831201	0.931	2.27	PIS55825.1	SSU81	Predicted adaptor protein involved in activation of MAP kinase-dependent signaling pathways; links response to oxidative stress to morphogenesis and cell wall biosynthesis; mediates formation of microcolonies; caspofungin repressed
PIS49505.1	28.9297688	26.8210994	27.3432311	30.8911733	29.90656056	29.12874258	0.001906404	0.682	2.28	PIS49505.1	orf19.5681	Ortholog of C. dubliniensis CD36 : Cd36_50190, C. parapsilosis CDC317 : CPAR2_302680, C. auris B8441 : B9J08_004528 and Candida tenuis NRRL Y-1498 : CANTEDRAFT_109949
PIS54953.1	24.8890483	25.5459978	24.9241966	27.21941106	27.55896041	27.45478001	5.11E-05	0.142	2.29	PIS54953.1	AVT7	Ortholog of S. cerevisiae Avt7 transporter; repressed upon adherence to polystyrene; constitutive expression independent of MTL or white-opaque status; Spider biofilm induced
PIS51417.1	28.6920711	28.5144487	27.5038857	31.60115519	29.98087204	30.01675788	0.000914798	0.565	2.3	PIS51417.1	orf19.3247	Putative ortholog of S. cerevisiae Laa1p; likely to be essential for growth, based on an insertional mutagenesis strategy
PIS55806.1	28.9365577	27.5910887	25.1249245	29.27506414	28.80467241	30.49407169	0.013304	0.869	2.31	PIS55806.1	ELP3	Predicted histone acetyltransferase; role in regulation of transcription, tRNA wobble uridine modification; Spider biofilm induced
PIS54817.1	26.0924631	25.8371861	24.151157	28.36682032	27.46982288	27.16730195	0.005463004	0.803	2.31	PIS54817.1	MED5	RNA polymerase II mediator complex subunit; transcription positively regulated by Tbf1p
PIS51640.1	24.3924119	24.7194064	25.8271841	26.58461992	26.65324821	28.70673149	0.000923705	0.567	2.34	PIS51640.1	orf19.6048	Protein of unknown function; Spider biofilm induced

PIS56724.1	24.6641062	24.2836819	24.127272	26.89756692	25.99495827	27.19103372	0.00035762	0.389	2.34	PIS56724.1	orf19.7665	Ortholog(s) have role in mitochondrial cytochrome c oxidase assembly and mitochondrial inner membrane localization
PIS48503.1	24.4602705	23.783283	24.6739651	26.37086241	27.05771241	26.54700323	0.000260354	0.334	2.35	PIS48503.1	orf19.6325.1	Ortholog of S. cerevisiae : MRX7, C. glabrata CBS138 : CAGL0K04785g, C. dubliniensis CD36 : Cd36_19820, C. parapsilosis CDC317 : CPAR2_603375 and C. auris B8441 : B9J08_005197
PIS49576.1	28.2102415	27.6204012	27.1579188	30.44218411	29.9975317	29.72651358	5.39E-05	0.146	2.39	PIS49576.1	orf19.4504	Has domain(s) with predicted oxidoreductase activity
PIS49471.1	24.4417654	25.6898776	26.151122	27.71416136	27.79778241	27.95279203	0.005932471	0.81	2.39	PIS49471.1	orf19.52	Ortholog(s) have role in intracellular iron ion homeostasis and mitochondrion localization
PIS54550.1	23.5013131	23.9451758	24.4176346	26.74262436	26.05150139	26.3121551	1.55E-05	0.0672	2.41	PIS54550.1	PHM5	Endo/exopolyphosphatase, hydrolyzes inorganic polyphosphate (poly P) into Pi; involved in polyphosphate catabolic process; redundant with exopolyphosphatase Ppx1p
PIS54756.1	25.3870865	24.8107937	22.9735566	24.06523007	28.05344105	28.29098153	0.044767785	0.923	2.41	PIS54756.1	SUR2	Putative ceramide hydroxylase; predicted enzyme of sphingolipid biosynthesis; regulated by Tsa1, Tsa1B under H2O2 stress conditions; Spider and flow model biofilm induced
PIS55738.1	26.7439224	26.4483394	25.2414824	28.78272259	28.56128303	28.35944506	0.004299503	0.78	2.42	PIS55738.1	MRPL40	Putative mitochondrial ribosomal protein; Spider biofilm repressed

PIS49500.1	25.6475445	25.4726118	24.2015292	28.63194046	27.35254638	26.67070109	0.000590069	0.485	2.44	PIS49500.1	orf19.429	Putative non-canonical poly(A) polymerase; repressed by nitric oxide; Spider biofilm induced
PIS58031.1	29.7590847	29.5417931	30.8910328	32.55131564	32.39470182	32.62746566	2.23E-05	0.0821	2.46	PIS58031.1	FET31	Putative multicopper oxidase; ketoconazole/caspofungin/amphotericin B repressed; Sef1/Sfu1/Hap43 regulated; reports differ if functional homolog of ScFet3; rat catheter and Spider biofilm induced
PIS58293.1	30.3493168	28.4597834	28.057219	31.54840422	30.92977401	31.8223709	0.000635583	0.499	2.48	PIS58293.1	DES1	Putative delta-4 sphingolipid desaturase; planktonic growth-induced gene
PIS58304.1	24.8960415	25.7724366	26.4817404	28.29998988	28.17441685	28.1059464	5.34E-05	0.145	2.48	PIS58304.1	DFI1	Cell-surface associated glycoprotein; promotes activation of Cek1 in a matrix-dependent manner; N-glycosylated; Spider biofilm induced
PIS51421.1	25.214121	25.6833333	27.18102	28.96557399	28.54639713	27.99642097	0.000433602	0.426	2.48	PIS51421.1	FTR1	High-affinity iron permease; required for mouse virulence, low-iron growth; iron, amphotericin B, caspofungin, ciclopirox, Hog1p, Sef1p, Sfu1p, and Hap43p regulated; complements S. cerevisiae ftr1 iron transport; Hap43p-repressed
PIS58259.1	28.5099355	25.4996339	23.7865054	29.13153713	28.03624325	28.09119359	0.013722703	0.871	2.49	PIS58259.1	HDA1	Histone deacetylase; inducer of filamentation; conserved deacetylation motif; regulates white-to-opaque switch frequency but not opaque-to-white switch; greater expression in white cells than opaque cells; inhibited by trichostatin-A;
PIS55572.1	24.2547243	23.6854646	22.9016839	26.11801344	25.40306171	26.78081829	0.008043789	0.835	2.49	PIS55572.1	orf19.1191	Ortholog(s) have ubiquitin-protein transferase activity and role in negative regulation of protein autoubiquitination, retrograde protein transport, ER to cytosol, ubiquitin-dependent ERAD pathway

PIS49459.1	24.2943	20.2661587	23.1924407	24.68909824	25.10575982	25.41431139	0.038469757	0.918	2.49	PIS49459.1	orf19.811	Protein of unknown function; mutant is viable; Hap43-repressed
PIS51791.1	24.3464913	24.6907546	24.0793971	27.41314539	26.24120354	26.94760772	9.05E-06	0.0443	2.5	PIS51791.1	PCL7	Putative cyclin-like protein; possible Pho85 cyclin; hyphal repressed; induced by Mnl1 under weak acid stress
PIS51870.1	29.3464471	28.6274165	29.2511983	32.69546607	31.517358	30.506063	0.000257695	0.332	2.5	PIS51870.1	PEP1	Type I transmembrane sorting receptor for multiple vacuolar hydrolases; cycles between late-Golgi and prevacuolar endosome-like compartments; rat catheter biofilm repressed
PIS58988.1	24.4375637	24.0478256	24.168748	27.29011726	26.87762998	26.09124916	0.000117916	0.224	2.53	PIS58988.1	GAP6	Broad-specificity amino acid permease; Plc1, Gcn4 regulated; rat catheter biofilm induced
PIS48642.1	27.0609342	25.5759733	23.140085	29.08154027	27.53957197	26.74966289	0.010620575	0.855	2.53	PIS48642.1	orf19.1624	Non-catalytic subunit of N-terminal acetyltransferase of the NatC type; flow model biofilm repressed
PIS58548.1	19.8366752	22.4134519	22.5354029	24.03679269	24.22429086	24.14877084	0.045867715	0.924	2.54	PIS58548.1	SIZ1	Possible SUMO/Smt3 ligase; Rim101-repressed
PIS54963.1	22.3655895	26.7345923	27.1247846	29.69935095	27.17379533	26.97063215	0.027441227	0.905	2.54	PIS54963.1	TIM9	Predicted protein of the mitochondrial intermembrane space; rat catheter biofilm induced; Spider biofilm repressed

PIS51365.1	25.5528206	26.5247528	26.084945	29.24033716	28.25152528	28.32931863	0.00086372	0.555	2.55	PIS51365.1	HIS3	Imidazoleglycerol-phosphate dehydratase, enzyme of histidine biosynthesis; functionally complements S. cerevisiae his3-1 mutation; hyphal-induced expression; regulated by Gcn2p and Gcn4p; fungal-specific (no human or murine homolog)
PIS52022.1	29.8864941	30.0482168	26.9318824	34.01003715	30.49119728	30.02323283	0.024490786	0.9	2.55	PIS52022.1	PEX6	Ortholog(s) have ATPase activity, role in protein import into peroxisome matrix, receptor recycling, protein unfolding and cytosol, peroxisome localization
PIS58674.1	27.6746287	25.3413425	26.3203563	29.75217329	28.99997161	28.31690544	0.007538649	0.83	2.58	PIS58674.1	TSR1	Component of 20S pre-rRNA processing unit; repressed by prostaglandins
PIS48560.1	27.1332233	25.4008858	25.0872848	28.38609311	28.03347793	28.95859937	0.000691589	0.514	2.59	PIS48560.1	TOS4	Putative fork-head transcription factor; rat catheter and Spider biofilm repressed
PIS58249.1	28.9438433	28.2084359	28.7345788	31.6013491	31.26563264	30.8864049	9.54E-05	0.202	2.62	PIS58249.1	GDH3	NADP-glutamate dehydrogenase; Nrg1, Plc1 regulated; hypha, hypoxia, Efg1-repressed; Rim101-induced at pH 8; GlcNAc, ciclopirox, ketoconazole induced; exp and stationary phase protein; Spider biofilm repressed; rat catheter biofilm induced
PIS58806.1	31.6261029	30.8900941	30.6873171	33.42935206	33.83123971	33.81625029	3.35E-06	0.0153	2.62	PIS58806.1	orf19.409	Ortholog of S. cerevisiae Aim38/Rcf2, cytochrome c oxidase subunit; plasma membrane localized; Hap43-repressed; induced in oralpharyngeal candidasis; flow model biofilm induced; Spider biofilm repressed
PIS55527.1	28.0126248	28.1175722	23.6085959	30.97655547	28.2565802	28.37999667	0.067962993	0.936	2.62	PIS55527.1	orf19.5533	Ortholog(s) have guanyl-nucleotide exchange factor activity, ubiquitin binding activity

PIS55833.1	25.5705342	25.1619858	20.8771489	26.7858571	26.2669198	26.43748448	0.031811585	0.911	2.63	PIS55833.1	RIX7	Putative ATPase of the AAA family; role in ribosomal subunit export from the nucleus; mutation impairs hyphal growth and biofilm formation
PIS56699.1	30.3908816	28.7877847	26.6009314	32.10722831	30.97308325	30.65386355	0.01894358	0.888	2.65	PIS56699.1	orf19.1139	Protein of unknown funtion; S. cerevisiae ortholog Svl3 plays a role in endocytosis and is localized to the bud neck; Spider biofilm induced
PIS51828.1	23.5214739	23.6188615	24.1435794	27.20322796	25.92815796	26.09074712	0.006859148	0.823	2.65	PIS51828.1	RRS1	Putative ribosome biogenesis and nuclear export protein; Hap43p-induced gene; mutation confers hypersensitivity to 5-fluorocytosine (5-FC), 5-fluorouracil (5-FU), and tubercidin (7-deazaadenosine)
PIS48469.1	23.0740043	22.4926158	22.7596758	25.52052222	25.36865531	25.42601563	3.41E-06	0.0156	2.66	PIS48469.1	orf19.6180	Protein similar to Pichia anomala YDL054c and S. cerevisiae Ydl054cp; transmembrane regions predicted; Hap43p-repressed gene
PIS56673.1	26.1175966	24.2964131	23.797781	27.3186942	27.20247341	27.67799113	0.009786808	0.849	2.66	PIS56673.1	orf19.6719	Component of the HIR complex, a nucleosome assembly factor involved in chromatin formation; involved in regulation of white-opaque switching, hyphal initiation, nitrogen utilization, and azole sensitivity
PIS49681.1	24.6128904	25.9202144	25.2389162	27.93978434	27.76032664	28.07720493	1.02E-05	0.0492	2.67	PIS49681.1	orf19.3167	Heme A:farnesyltransferase; catalyzes the 1st step in conversion of protoheme to the heme A prosthetic group required for cytochrome c oxidase activity; Spider biofilm repressed
PIS51362.1	25.8352468	26.939555	23.6070322	28.82324871	27.3957963	28.21876854	0.002736992	0.729	2.69	PIS51362.1	MED14	RNA polymerase II mediator complex subunit

PIS58423.1	26.4846884	24.5379208	19.5633718	26.78563863	26.34284325	25.54466522	0.052563552	0.928	2.7	PIS58423.1	NUP60	Ortholog of S. cerevisiae Nup60p; a subunit of the nuclear pore complex; mutants are viable
PIS48412.1	29.4914037	26.831515	25.7527181	30.026154	30.24714564	29.90994645	0.002713673	0.728	2.7	PIS48412.1	orf19.5852	Protein of unknown function; rat catheter biofilm repressed
PIS51806.1	19.8366752	21.5246522	21.8263855	23.65333274	23.92936808	23.72203693	0.010276844	0.853	2.71	PIS51806.1	orf19.3303	Ortholog(s) have tRNA methyltransferase activity and role in tRNA methylation, wybutosine biosynthetic process
PIS56674.1	26.8361877	26.7742782	26.3703153	29.46329819	29.33231046	29.33692892	0.000627465	0.496	2.72	PIS56674.1	NAG4	Putative transporter; fungal-specific; similar to Nag3p and to S. cerevisiae Ypr156Cp and Ygr138Cp; required for wild-type mouse virulence and wild-type cycloheximide resistance; gene cluster encodes enzymes of GlcNAc
PIS49730.1	24.981252	24.1136371	25.1485705	27.54157782	26.8741949	28.01646284	2.41E-05	0.0849	2.73	PIS49730.1	orf19.6789	S. cerevisiae ortholog Nud1 is a spindle pole body outer plaque component; it acts through the mitotic exit network to specify asymmetric spindle pole body inheritance; Hap43-induced gene
PIS48397.1	26.0538141	24.9231555	24.8844493	28.30610351	28.44014771	27.39045591	2.09E-05	0.0795	2.76	PIS48397.1	orf19.2484	Has domain(s) with predicted metalloexopeptidase activity and role in proteolysis
PIS48634.1	24.3902643	22.4989311	23.0294133	27.38017575	26.79223511	24.08445654	0.01456851	0.874	2.78	PIS48634.1	STE50	Protein with sterile alpha motif (SAM) and Ras-associated domain (RAD); similar to S. cerevisiae Rad50p, which is involved in signal transduction via interaction with and regulation of MAPKKK

PIS50503.1	26.3467197	23.8628262	23.3201251	27.61895374	27.2107532	27.27896668	0.00460147	0.787	2.86	PIS50503.1	orf19.3996	Putative mannosyltransferase; similar to S. cerevisiae Gpi10p; has HKEXRF motif
PIS58789.1	23.7485535	24.5748614	23.789519	28.73821386	26.01856663	25.97205406	0.001558042	0.652	2.87	PIS58789.1	UBA4	Putative ubiquitin activating protein; Hap43-repressed; induced by prostaglandins; clade-associated gene expression
PIS48537.1	25.318717	25.651244	26.0111769	28.81258309	28.4931865	28.30609774	2.02E-05	0.0781	2.88	PIS48537.1	CPA1	Putative carbamoyl-phosphate synthase subunit; alkaline repressed; rat catheter, Spider and flow model biofilm induced
PIS52385.1	24.7843027	22.6045885	22.9496515	26.69245663	25.91443265	26.49753762	0.015131882	0.876	2.92	PIS52385.1	SLR1	Protein similar to mammalian SR-like RNA splicing factor; involved in filamentous growth and virulence; gene has intron; Spider biofilm repressed
PIS48528.1	30.106578	28.1422187	26.0276688	31.9959164	31.12297312	29.93332788	0.004096445	0.775	2.93	PIS48528.1	orf19.6862	Hap43-induced gene; mutation confers hypersensitivity to 5-fluorocytosine (5-FC), 5-fluorouracil (5-FU), and tubercidin (7-deazaadenosine)
PIS51470.1	28.7699644	27.1978955	27.5125002	31.07798011	29.97523564	31.25087067	0.000497661	0.452	2.94	PIS51470.1	NPL3	Putative RNA-binding protein; required for normal flow model biofilm growth; nuclear export is facilitated by Hmt1p; transcript upregulated in RHE model of oralcandidiasis; Spider biofilm repressed
PIS49793.1	22.5064307	25.9478513	26.47858	27.44467719	27.30068417	29.09164978	0.002954794	0.739	2.97	PIS49793.1	HAL9	Putative Zn(II)2Cys6 transcription factor; gene in zinc cluster region of Chr. 5; induced by Mnl1 in weak acid; similar to S. cerevisiae Hal9, a putative transcription factor involved in salt tolerance

PIS58310.1	27.8792799	26.702476	26.4943489	30.62360638	29.6971892	29.65640162	0.000819237	0.545	2.97	PIS58310.1	orf19.3874	Predicted cation transmembrane transporter; Spider biofilm induced
PIS58383.1	26.8023638	26.4744106	25.8322	29.43643132	29.17241998	29.50315339	1.76E-06	0.00839	3	PIS58383.1	RFC1	Putative DNA replication factor C subunit; ortholog of S. cerviaie Rfc1; likely essential, based on an insertional mutagenesis strategy; rat catheter biofilm repressed
PIS58278.1	24.5979543	23.9726604	25.8888199	28.68757284	27.94667102	26.8156477	0.050253505	0.927	3	PIS58278.1	RLM1	Transcription factor required for wild-type resistance to cell wall perturbation caused by caspofungin treatment; regulates caspofungin-induced transcription of SKO1
PIS51445.1	29.282714	28.8852663	28.4781003	31.96633717	31.89711555	32.0918049	2.14E-05	0.0803	3.1	PIS51445.1	ERG24	C-14 sterol reductase, has a role in ergosterol biosynthesis; mutation confers increased sensitivity to dyclonine; rat catheter and Spider biofilm repressed
PIS48336.1	22.5144271	23.3396579	23.8582116	26.72343578	25.84318717	26.47462296	0.017842646	0.885	3.11	PIS48336.1	orf19.5449	Predicted integral membrane protein; Spider biofilm induced
PIS58062.1	23.2305047	18.5021598	19.9835621	24.13497417	23.91246081	23.46440123	0.005438288	0.803	3.27	PIS58062.1	orf19.3455	Putative mitochondrial inner membrane magnesium transporter; possibly an essential gene, disruptants not obtained by UAU1 method
PIS50344.1	22.6761305	21.5312169	18.589984	24.2623846	24.2640118	24.08545015	0.052387326	0.928	3.27	PIS50344.1	orf19.6554	Regulator of calcineurin; regulated by calcineurin-Crz1 pathway; feedback regulator of calcineurin-dependent signaling; Hap43-repressed; induced by ketoconazole, hypoxia, during growth in the mouse cecum; Spider biofilm induced

PIS52192.1	21.4553208	22.3304773	22.3755784	25.54912515	25.10853356	25.4157466	0.00468586	0.789	3.3	PIS52192.1	orf19.1394	Putative protein of unknown function; mutant is viable; protein level decreases in stationary phase cultures; Spider biofilm induced
PIS51224.1	25.4980775	20.5202968	20.149311	25.82692776	24.86975969	25.61743768	0.006227918	0.814	3.38	PIS51224.1	OLE2	Protein with similarity to fatty acid desaturase (stearoyl-CoA desaturase); homozygous null mutant shows decreased production of prostaglandin E2
PIS51732.1	26.2309804	23.9898495	25.4604867	28.09295301	28.21642979	29.51283208	4.91E-05	0.138	3.38	PIS51732.1	orf19.2040	Ortholog(s) have oxidoreductase activity, acting on NAD(P)H activity, role in iron-sulfur cluster assembly, nitric oxide biosynthetic process, positive regulation of nitric oxide biosynthetic process and mitochondrion localization
PIS58910.1	27.8799904	25.5268713	25.1259829	29.56809313	29.77243592	29.49846755	0.001303648	0.624	3.44	PIS58910.1	ERG25	Putative C-4 methyl sterol oxidase; C4-demethylation of ergosterol biosynthesis intermediates, based on similarity to S. cerevisiae Erg25; fluconazole-induced; induced in azole-resistant strain; rat catheter and Spider biofilm induced
PIS54623.1	25.64038	24.6534087	18.7171749	26.56631172	25.94767593	26.86455926	0.045558542	0.924	3.46	PIS54623.1	IPK2	Putative inositol polyphosphate multikinase; involved in regulation of calcium homeostasis, secretion and hyphal growth
PIS55823.1	27.2284579	25.8748949	20.5272328	28.59070674	28.77795921	26.63335202	0.053814106	0.929	3.46	PIS55823.1	orf19.1210	Ortholog(s) have L-arginine transmembrane transporter activity, L-aspartate transmembrane transporter activity and L-glutamate transmembrane transporter activity, more
PIS55824.1	30.9537814	30.6668108	31.2522641	34.89892682	34.66947429	33.80449368	4.96E-06	0.0239	3.5	PIS55824.1	AOX2	Alternative oxidase; cyanide-resistant respiration; induced by antimycin A, oxidants; growth; Hap43, chlamydospore formation repressed; rat catheter, Spider biofilm induced; regulated in Spider biofilms by Bcr1, Tec1, Ndt80, Brg1

PIS56659.1	23.0831968	24.0921819	24.9079069	28.28815021	27.31567554	27.17433543	0.015968078	0.879	3.56	PIS56659.1	orf19.1519	Protein of unknown function; Hap43-repressed gene
PIS55474.1	22.708951	23.4782041	17.4539314	24.9645055	24.59586053	25.10341499	0.014873247	0.875	3.67	PIS55474.1	GAR1	Putative H/ACA snoRNP pseudouridylase complex protein; mutation confers hypersensitivity to tubercidin (7-deazaadenosine); macrophage/pseudohyphal-induced; Spider biofilm repressed
PIS52256.1	28.0585815	23.7547217	26.6135292	30.24611569	29.05494809	30.26132567	0.000420604	0.42	3.71	PIS52256.1	orf19.7198	CCR4-NOT complex component; involved in controlling mRNA initiation, elongation and degradation; rat catheter biofilm induced
PIS48274.1	25.3002812	27.2376118	24.7710191	29.89337767	29.08937033	29.90617118	6.95E-05	0.169	3.86	PIS48274.1	orf19.1030	Putative peptidyl-prolyl cis-trans isomerase
PIS55835.1	22.7450854	21.2225007	22.5008856	26.23867471	25.33294542	26.53044911	0.008593951	0.84	3.88	PIS55835.1	orf19.3161	Ortholog(s) have ATPase activator activity, RNA binding activity
PIS58822.1	24.3602072	23.9342423	24.1744266	28.23805933	28.04018804	28.43670757	1.97E-06	0.00935	4.08	PIS58822.1	SSQ1	Protein involved in intracellular sequestering of iron ion and mitochondrial iron-sulfur cluster assembly; repression leads to defects in respiratory growth, activation of autophagy, attenuated virulence
PIS56786.1	27.3534827	24.7296197	26.0297199	30.01611268	30.04713767	30.49183126	0.000111407	0.219	4.15	PIS56786.1	GIS2	Translational activator for mRNAs with internal ribosome entry sites; induced in high iron; repressed by yeast-hypha switch; null exhibits sensitivity to sorbitol, 5-fluorocytosine, and cold temperatures; Spider biofilm repressed

PIS58799.1	24.7533555	21.8036214	19.1354574	26.88921035	26.64117574	25.97467782	0.001128851	0.601	4.6	PIS58799.1	BIG1	Endoplasmic reticulum (ER) protein; ortholog of S. cerevisiae Big1; required for beta-1,6-glucan synthesis, filamentation, adhesion, and virulence; rat catheter biofilm repressed
PIS54851.1	27.4590856	24.2720323	25.3783981	30.45991921	30.21847686	30.34423701	4.95E-05	0.139	4.64	PIS54851.1	OPT2	Oligopeptide transporter; induced upon phagocytosis by macrophage; macrophage/pseudohyphal-repressed after 16h; fluconazole-induced; virulence-group-correlated expression; Hap43-repressed
PIS50348.1	24.6248058	17.8291071	18.5790715	24.34832967	25.77371624	25.08662512	0.060337061	0.932	4.73	PIS50348.1	MAM33	Putative mitochondrial acidic matrix protein; regulated by Ssn6p; protein present in exponential and stationary growth phase yeast cultures
PIS52366.1	24.8866564	24.1546504	24.3299798	29.75287945	29.58138257	28.67553388	9.56E-09	3.85E-08	4.88	PIS52366.1	MDR1	Plasma membrane MDR/MFS multidrug efflux pump; methotrexate is preferred substrate; overexpression in drug-resistant clinical isolates confers fluconazole resistance; repressed in young biofilms; rat catheter biofilm [illegible]
PIS51654.1	26.5410233	24.9535358	18.1804836	28.19026619	29.52031129	28.17215461	0.004635008	0.787	5.4	PIS51654.1	XOG1	[illegible] hydrolase family member; affects sensitivity to chitin and glucan synthesis inhibitors; not required for yeast-to-hypha transition or for virulence in mice; Hap43-induced; [illegible]
PIS49748.1	24.7947532	22.5437132	20.4740971	27.83795528	28.08351167	28.89268751	2.50E-05	0.0864	5.67	PIS49748.1	TPO3	Putative polyamine transporter; MFS-MDR family; induced by Sfu1, regulated upon white-opaque; decreased expression in hyphae vs yeast-form cells; regulated by Nrg1; Spider biofilm repressed

GO enrichment analysis

Protein_IDs	Gene_names	logFC(*gfc1*Δ vs WT in YPG)	Description
**protein metabolic process(GO:0019538**			
PIS58836.1	HSL1	2.54	Probable protein kinase involved in determination of morphology during the cell cycle of both yeast-form and hyphal cells via regulation of Swe1p and Cdc28p; required for full virulence and kidney colonization in mouse systemic infection
PIS48694.1	VPS15	1.73	Protein involved in retrograde endosome-to-Golgi protein transport; required for normal virulence
PIS58654.1	PTP1	1.39	Phosphotyrosine-specific protein phosphatase; rat catheter biofilm induced
PIS48719.1	SAP3	1.33	Secreted aspartyl proteinase, acts in utilization of protein as nitrogen source; assessment of virulence role complicated by URA3 effects; regulated by growth phase; produced by opaque phase cells; alpha-pheromone repressed
PIS56967.1	MNN12	1.26	Predicted alpha-1,3-mannosyltransferase activity with a role in protein glycosylation
PIS52412.1	CLA4	1.24	Ste20p family Ser/Thr kinase required for wild-type filamentous growth, organ colonization and virulence in mouse systemic infection; role in chlamydospore formation; functional homolog of S. cerevisiae Cla4p; mutant caspofungin sensitive
PIS56786.1	GIS2	1.23	Translational activator for mRNAs with internal ribosome entry sites; induced in high iron; repressed by yeast-hypha switch; null exhibits sensitivity to sorbitol, 5-fluorocytosine, and cold temperatures; Spider biofilm repressed
PIS50348.1	MAM33	1.22	Putative mitochondrial acidic matrix protein; regulated by Ssn6p; protein present in exponential and stationary growth phase yeast cultures
PIS54764.1	HST7	1.19	MAP kinase kinase involved in mating and hyphal growth signal transduction pathways; phosphorylates Cek1p; wild-type virulence in mouse systemic infection; functional homolog of S. cerevisiae Ste7p; mutants are hypersensitive to caspofungin
PIS51245.1	NOT3	1.19	Transcriptional regulator; not required for buccal epithelial cell adherence or virulence in mouse systemic infection; null mutant colonies exhibit slightly decreased filamentation ratio; required for yeast adherence to silicone substrate
PIS51541.1	YAK1	1.16	Putative dual-specificity tyrosine-phosphorylation regulated kinase; involved in hyphal growth regulation and biofilm formation; appears to act either downstream or in parallel with the RAS/cAMP/PKA pathway
PIS54821.1	CDC43	1.06	Beta subunit of heterodimeric protein geranylgeranyltransferase type I; GGTase I enzyme binds zinc, is Mg-dependent; Cdc42p is GGTase I substrate
PIS54847.1	BET4	1.04	Ortholog(s) have Rab geranylgeranyltransferase activity and role in endoplasmic reticulum to Golgi vesicle-mediated transport, protein geranylgeranylation, protein targeting to membrane
PIS56905.1	RMT2	1.02	Minor protein arginine methyltransferases (PRMT) involved in methylation of arginine residues
PIS58949.1	KRE5	0.967	UDP-glucose:glycoprotein glucosyltransferase; 1,6-beta-D-glucan biosynthesis, hyphal growth, virulence in mouse IV model; partially complements S. cerevisiae kre5 mutant defects; flow biofilm repressed, Bcr1-repressed in RPMI a/a biofilms
PIS54813.1	HLJ1	0.956	Putative HSP40 co-chaperone; repressed during the mating process
PIS48631.1	STE11	0.931	Protein similar to S. cerevisiae Ste11p; mutants are sensitive to growth on H2O2 medium
PIS55709.1	GPX3	0.912	Putative glutathione peroxidase involved in Cap1p-dependent oxidative stress response, required for Cap1p oxidation in response to H2O2; planktonic growth-induced
PIS51107.1	EPL1	0.904	Subunit of the NuA4 histone acetyltransferase complex
PIS51802.1	CCH1	0.88	Voltage-gated Ca2+ channel of the high affinity calcium uptake system; roles in thigmotropism, establishment of galvanotropism; transcript regulated by Nrg1 and Mig1; flow model biofilm repressed
PIS48342.1	SMP3	0.85	Mannosyltransferase of glycosylphosphatidylinositol (GPI) biosynthesis; catalyzes mannosylation of Man3-GPI precursor; essential for viability; 8-9 transmembrane regions predicted; has HQEXRF motif; functional homolog of S. cerevisiae Smp3p
PIS55829.1	MNN2	0.815	Alpha-1,2-mannosyltransferase; similar to S. cerevisiae Mnn2; role in cell wall integrity, temperature sensitivity; iron utilization in low iron; Tn mutation affects filamentous growth; filament induced; increased chitin exposes beta-glucan

PIS58684.1	ANP1	0.806	Putative mannosyltransferase of Golgi; member of Mnn9p family; similar to S. cerevisiae Anp1p; fungal-specific (no human or murine homolog)
PIS58579.1	ESS1	0.805	Prolyl isomerase (parvulin class); essential; involved in yeast-hyphal switching, Cph1p pathway; has inflexible linker between WW and isomerase domains, unlike human homolog; functional homolog of S. cerevisiae Ess1p
PIS49782.1	RIT1	0.805	Putative initiator tRNA methionine ribosyltransferase; fungal-specific (no human or murine homolog)
PIS52124.1	CAK1	0.798	Monomeric CDK-activating kinase; functional homolog of S. cerevisiae Cak1p; phosphorylates cyclin-free human CDK2; lacks glycine loop motif; conserved lysine (K36) not required for activity; possibly essential gene (by UAU1 method)
PIS55714.1	RTT101	0.736	Putative cullin subunit of E3 ubiquitin ligase complex, involved in response to DNA damage; induced by alpha pheromone in SpiderM medium
PIS51318.1	FUN31	0.718	Putative PAS kinase involved in cell wall damage response; similar to S. cerevisiae Psk1p, a putative serine/threonine protein kinase; induced by Mnl1 under weak acid stress; rat catheter and Spider biofilm induced
PIS58544.1	MRP20	0.716	Component of mitochondrial ribosome; decreased expression in hyphae compared to yeast-form cells
PIS51846.1	PHO23	0.705	Ortholog(s) have methylated histone binding activity
PIS55564.1	BET2	0.702	Putative Type II geranylgeranyltransferase beta subunit; transcript regulated by Mig1
PIS55737.1	RPT4	0.686	26S proteasome regulatory subunit; regulated by Gcn2p and Gcn4p; protein level decreases in stationary phase cultures
PIS51110.1	MKC1	0.681	MAP kinase; role in biofilm formation, contact-induced invasive filamentation, systemic virulence in mouse, cell wall structure/maintenance, caspofungin response; phosphorylated on surface contact, membrane perturbation, or cell wall stress
PIS48313.1	CKA2	0.675	Catalytic alpha-subunit of protein kinase CK2; interaction with calcineurin pathway affects fluconazole sensitivity; synthetically lethal with CKA1; attenuated virulence in a mouse oropharyngeal candidiasis but not in a systemic mouse model
PIS51983.1	DOT4	0.668	Protein similar to ubiquitin C-terminal hydrolase; localizes to cell surface of hyphal cells, but not yeast-form cells; repressed upon high-level peroxide; Hap43p-induced; rat catheter biofilm induced
PIS51143.1	FGR10	0.65	Putative asparaginase; lacks ortholog in S. cerevisiae; transposon mutation affects filamentous growth; Spider biofilm induced
PIS55516.1	SKP1	0.631	Putative subunit D of kinetochore protein complex CBF3; regulated by Gcn4p; repressed in response to amino acid starvation (3-aminotriazole treatment)
PIS52430.1	MCD4	0.62	Mannose-ethanolamine phosphotransferase, essential gene involved in GPI anchor biosynthesis
PIS48685.1	ANB1	0.588	Translation initiation factor eIF-5A; repressed in hyphae vs yeast cells; downregulated upon phagocytosis by murine macrophage; Hap43-induced; GlcNAc-induced protein; Spider biofilm repressed
PIS55803.1	NPL4	-0.599	Putative ubiquitin-binding protein; regulated by Gcn2p and Gcn4p
PIS50563.1	RPL43A	-0.605	Putative ribosomal protein, large subunit; repressed by human whole blood or PMNs; colony morphology-related gene regulation by Ssn6; Spider biofilm repressed
PIS49720.1	RPD31	-0.608	Putative histone deacetylase; involved in regulation of white-opaque switch; Spider biofilm repressed
PIS51843.1	OCA1	-0.622	Putative protein phosphatase of the PTP family (tyrosine-specific); ortholog of S. cerevisiae Oca1; mutant is viable; mutant shows virulence defect
PIS55698.1	NOP1	-0.625	Nucleolar protein; flucytosine induced; Hap43-induced; Spider biofilm repressed
PIS55816.1	RPL10A	-0.626	Predicted ribosomal protein; downregulated upon phagocytosis by murine macrophages; Hap43-induced; Spider biofilm repressed
PIS51716.1	RPL4B	-0.626	Ribosomal protein 4B; repressed upon phagocytosis by murine macrophage; Spider biofilm repressed
PIS48636.1	RPL12	-0.654	Ribosomal protein L12, 60S ribosomal subunit; downregulated by human whole blood or polymorphonuclear cells; genes encoding cytoplasmic ribosomal subunits are downregulated upon phagocytosis by macrophage; Tbf1p-activated; Hap43p-induced

PIS51357.1	RMS1	-0.655	Putative lysine methyltransferase; Hap43-induced; protein induced during mating; possibly essential, disruptants not obtained by UAU1 method; rat catheter and Spider biofilm induced
PIS51641.1	TUF1	-0.669	Translation elongation factor TU; macrophage/pseudohyphal-induced; repressed upon phagocytosis by murine macrophage; levels decrease in stationary phase cells; Spider biofilm repressed
PIS51193.1	PUP1	-0.676	Putative beta 2 subunit of the 20S proteasome; macrophage/pseudohyphal-repressed; Spider biofilm repressed
PIS56703.1	EIF4E	-0.691	Translation initiation factor eIF4E; genes encoding ribosomal subunits, translation factors, tRNA synthetases downregulated by phagocytosis by macrophage; alternatively spliced intron in 5' UTR; protein levels decrease in stationary phase
PIS58479.1	ALG5	-0.697	Putative glucosyltransferase involved in cell wall mannan biosynthesis; possibly an essential gene, disruptants not obtained by UAU1 method
PIS49835.1	NBN1	-0.701	Subunit of the NuA4 histone acetyltransferase complex; Plc1p-regulated
PIS50541.1	HOG1	-0.717	MAP kinase of osmotic-, heavy metal-, and core stress response; role in regulation of response to stress; phosphorylated in response to H2O2 or NaCl; acts as repressor of START; mutant induces protective mouse immune response
PIS48407.1	RPL5	-0.722	Ribosomal protein; repressed upon phagocytosis by murine macrophages; Hap43-induced; Spider biofilm repressed
PIS48606.1	WBP1	-0.724	Putative oligosaccharyltransferase subunit; Spider biofilm repressed
PIS54788.1	PRE6	-0.726	Putative alpha-4 subunit of the proteasome; reported as macrophage-induced protein and macrophage/pseudohyphal-repressed gene; regulated by Gcn2p and Gcn4p; removed from/reinstated in Assembly 20 (see Locus History)
PIS51784.1	SIT4	-0.729	Serine/threonine protein phosphatase catalytic subunit; has a role in cell wall maintenance, hyphal growth, and virulence in a mouse systemic infection model
PIS54503.1	RPL35	-0.735	Ribosomal protein; downregulation correlates with clinical development of fluconazole resistance; colony morphology-related gene regulation by Ssn6; Hap43-induced; Spider biofilm repressed
PIS50399.1	SSZ1	-0.739	Putative HSP70 chaperone; protein level decreases in stationary phase cultures; Spider biofilm repressed
PIS48755.1	ASC1	-0.743	40S ribosomal subunit similar to G-beta subunits; glucose or N starvation induced filamentation; required for virulence in mice; snoRNA snR24 encoded in ASC1 intron; repressed in stationary phase; GlcNAc-induced; Spider biofilm repressed
PIS58068.1	RPL25	-0.744	Putative rRNA-binding ribosomal protein component of the 60S ribosomal subunit; Hap43-induced; colony morphology-related gene regulation by Ssn6
PIS59021.1	ACT1	-0.756	Actin; gene has intron; transcript regulated by growth phase, starvation; at polarized growth site in budding and hyphal cells; required for wild-type Cdc42 localization; unprocessed N terminus; Hap43-induced; Spider biofilm repressed
PIS52251.1	PPE1	-0.777	Protein similar to S. cerevisiae Ppe1p; shows genetic interaction with some genes involved in diploid filamentous growth and haploid invasive growth in S. cerevisiae
PIS58425.1	SWC4	-0.779	Subunit of the NuA4 histone acetyltransferase complex
PIS49829.1	RAD23	-0.786	S. cerevisiae Rad23 ortholog; binds damaged DNA; Spider biofilm repressed
PIS48226.1	CDC23	-0.788	Similar to anaphase-promoting complex component; possibly transcriptionally regulated by Tac1p
PIS52018.1	GCD7	-0.795	Putative translation initiator; downregulated in the presence of human whole blood or polymorphonuclear (PMN) cells
PIS50569.1	TIP41	-0.797	Protein involved in TOR signaling pathway; regulates protein phosphatase 2A (PP2A) activity; regulates Rad53p during recovery from DNA damage
PIS58221.1	RPP0	-0.805	Putative ribosomal protein; antigenic in mouse; repressed upon phagocytosis by murine macrophage; induced by Tbf1; overlaps orf19.7014; Spider biofilm repressed
PIS50411.1	FUN12	-0.825	Functional homolog of S. cerevisiae Fun12 translation initiation factor eIF5B; genes encoding ribosomal subunits, translation factors, and tRNA synthetases are downregulated upon phagocytosis by murine macrophage
PIS50470.1	PSD2	-0.868	Ortholog(s) have phosphatidylserine decarboxylase activity, role in phosphatidylcholine biosynthetic process, phosphatidylethanolamine biosynthetic process and endosome localization

PIS58802.1	ADA2	-0.868	Zinc finger and homeodomain transcriptional coactivator; role in cell wall integrity and in sensitivity to caspofungin; required for the normal transcriptional response to caspofungin; required for yeast cell adherence to silicone substrate
PIS54705.1	ERF1	-0.897	Putative translation release factor 1, which interacts with stop codons and promotes release of nascent peptides from ribosomes; Hap43p-induced gene
PIS49535.1	CPP1	-0.901	VH1 family MAPK phosphatase; regulates Cst20-Hst7-Cek1-Cph1 filamentation pathway; negatively regulates mating, represses yeast-hyphal switch; required for virulence in mice; yeast-enriched; induced by alpha pheromone in SpiderM medium;
PIS55719.1	PBS2	-0.924	MAPK kinase (MAPKK); role in osmotic and oxidative stress responses, oxidative stress adaptation; required for stress regulation of Hog1p localization and activity; functional homolog of S. cerevisiae Pbs2p
PIS51392.1	HMT1	-0.939	Major type I protein arginine methyltransferase (PRMT); involved in asymmetric dimethylation of arginine residues; involved in nuclear export of Npl3p; Spider biofilm repressed
PIS48438.1	APG7	-0.94	Ortholog(s) have Atg12 activating enzyme activity, Atg8 activating enzyme activity
PIS56762.1	BRE1	-0.96	Putative transcription factor with C3HC4 zinc finger DNA-binding motif; similar to S. cerevisiae Bre1p; transposon mutation affects filamentous growth
PIS54910.1	HST3	-0.97	Histone H3K56 deacetylase; reduced copy number increases opaque cell formation; repressed by MMS, hydroxyurea and high-levels of hydrogen peroxide; Hap43p-induced; ectopic expression blocks genotoxin-induced switching; nicotinamide target
PIS58402.1	ALG8	-0.972	Putative glucosyltransferase involved in cell wall mannan biosynthesis; transcription is elevated in nik1 and sln1 homozygous null mutants, but not in the chk1 null mutant; possibly an essential gene, disruptants not obtained by UAU1 method
PIS48655.1	SET1	-1.01	Lysine histone methyltransferase; methylates histone H3 K4; regulates of white-opaque switch, epithelial cell adhesion, agar-embedded filamentation, virulence in mice; unique N-terminus immunogenic in human; rat catheter biofilm repressed
PIS58355.1	RTT109	-1.02	Histone acetyltransferase, mutants are sensitive to DNA damage, show decreased virulence in mice, decreased white-to-opaque switching and increased susceptibility to killing by macrophages
PIS56545.1	RPL23A	-1.1	Ribosomal protein; downregulated upon phagocytosis by murine macrophage; Hap43-induced; sumoylation target; Spider biofilm repressed
PIS51916.1	SPC2	-1.14	Signal peptidase complex component; role in ER protein translocation; transcript is induced upon filamentous growth
PIS51246.1	PEX12	-1.18	Ortholog(s) have ubiquitin ligase activator activity, ubiquitin protein ligase activity and role in proteasome-mediated ubiquitin-dependent protein catabolic process, protein import into peroxisome matrix, protein polyubiquitination
PIS58059.1	HCR1	-1.27	Putative translation initiation factor; repressed upon phagocytosis by murine macrophage; Spider biofilm repressed
PIS58246.1	ECM39	-1.3	Putative mannosyltransferase similar to S. cerevisiae Ecm39p, which has a role in Calcofluor white resistance; predicted Kex2p substrate; has HKEXRF motif
PIS52220.1	MEF2	-1.37	Putative mitochondrial translation elongation factor; caspofungin induced
PIS58240.1	GPI13	-1.55	Major facilitator superfamily protein; has phosphodiesterase/nucleotide pyrophosphatase domain; similar to S. cerevisiae Gpi13p, which acts in GPI anchor biosynthesis; Hap43p-induced gene
PIS58100.1	UBA2	-1.57	Ortholog(s) have SUMO activating enzyme activity, role in protein sumoylation and SUMO activating enzyme complex, nucleus localization
PIS51078.1	NOT4	-1.58	Putative E3 ubiquitin-protein ligase; required for maintenance, but not induction, of hyphal development; homozygous null mutant is avirulent in mouse systemic infection despite persistence in host; repressed in rat oral candidiasis
PIS52174.1	PUP3	-1.68	Putative beta 3 subunit of the 20S proteasome; macrophage/pseudohyphal-repressed
PIS55462.1	IFM1	-2.14	Putative mitochondrial translation initiation factor; transcript regulated by Nrg1, Mig1, and Tup1
PIS56946.1	AKL1	-2.23	Putative serine/threonine protein kinase; induced during the mating process
PIS58722.1	CRK1	-3.76	Protein kinase of the Cdc2 subfamily involved in hyphal development, virulence; promotes hyphal development independently of Cph1 and Efg1; functionally complements pheromone hypersensitivity of S. cerevisiae sgv1 mutant; Hap43p-repressed

cellular amino acid metabolic

PIS49771.1	ARG3	2.44	Putative ornithine carbamoyltransferase; Gcn4-regulated; Hap43-induced; repressed in alkalinizing medium; rat catheter and Spider biofilm induced
PIS51017.1	AFP99	1.49	Agmatinase, involved in metabolism of agmatine; downregulated upon adherence to polystyrene; regulated by Gcn2p and Gcn4p
PIS54981.1	PUT1	1.45	Putative proline oxidase; regulated by Rim101p and Put3p; null mutant cannot grow on proline as a nitrogen source and shows reduced filamentation and reduced invasive growth both in vitro and in vivo; Spider biofilm induced
PIS52111.1	LYS22	1.13	Homocitrate synthase, minor isoform; repressed by nitric oxide and by hypoxia; protein level decreases in stationary phase cultures; induced by ketoconazole, Spider biofilm induced; flow model biofilm repressed
PIS55546.1	ARO8	1.09	Aromatic transaminase of the Ehrlich fusel oil pathway of aromatic alcohol biosynthesis; Rim101 independent alkaline induction; protein abundance affected by URA3 expression in CAI-4 strain; Gcn4-regulated; stationary phase enriched
PIS56685.1	ECM17	0.962	Putative sulfite reductase beta subunit; role in cell wall biogenesis; regulated by Tsa1/Tsa1B in H2O2 stress; Gcn4-regulated; Tbf1-activated; Hap43-repressed; Spider, flow, F-12/CO2 model biofilm induced
PIS51365.1	HIS3	0.945	Imidazoleglycerol-phosphate dehydratase, enzyme of histidine biosynthesis; functionally complements S. cerevisiae his3-1 mutation; hyphal-induced expression; regulated by Gcn2p and Gcn4p; fungal-specific (no human or murine homolog)
PIS52478.1	PAN6	0.889	Ortholog(s) have pantoate-beta-alanine ligase activity and role in pantothenate biosynthetic process
PIS49641.1	DAO1	0.877	Putative D-amino acid oxidase; transcription is regulated upon yeast-hyphal switch
PIS58249.1	GDH3	0.771	NADP-glutamate dehydrogenase; Nrg1, Plc1 regulated; hypha, hypoxia, Efg1-repressed; Rim101-induced at pH 8; GlcNAc, ciclopirox, ketoconazole induced; exp and stationary phase protein; Spider biofilm repressed; rat catheter biofilm induced
PIS51911.1	CAR2	0.722	Ornithine aminotransferase; arginine metabolism; alkaline induced; mutant sensitivite to toxic ergosterol analog, to amphotericin B; exponential and stationary phase yeast; flow model biofilm induced; rat catheter, Spider biofilm repressed
PIS49777.1	HPD1	0.687	3-hydroxypropionate dehydrogenase; involved in degradation of toxic propionyl-CoA; rat catheter and Spider biofilm induced
PIS51908.1	MET15	0.654	O-acetylhomoserine O-acetylserine sulfhydrylase; sulfur amino acid synthesis; immunogenic; Hog1, adherence-induced; brown color of mutant in Pb(2+) medium a visual selection; chlamydospore formation induced, F-12/CO2 biofilm induced
PIS51513.1	SER1	0.604	Putative 3-phosphoserine aminotransferase; predicted role in serine and glycine biosynthesis; protein present in exponential and stationary yeast growth phases; Spider biofilm repressed
PIS52226.1	ARO7	-0.59	Putative chorismate mutase; fungal-specific (no human or murine homolog); alkaline upregulated
PIS48736.1	PUT3	-0.621	Zn(II)2Cys6 transcription factor; has similarity to S. cerevisiae Put3, a transcription factor involved in the regulation of proline utilization genes
PIS51628.1	STR2	-0.639	Ortholog(s) have cystathionine gamma-synthase activity and role in sulfur compound metabolic process, transsulfuration
PIS52170.1	TRP4	-0.654	Predicted enzyme of amino acid biosynthesis; upregulated in biofilm; regulated by Gcn2p and Gcn4p; S. cerevisiae ortholog is Gcn4p regulated
PIS58196.1	ARG1	-0.667	Argininosuccinate synthase; arginine synthesis; Gcn4, Rim101 regulated; induced by amino acid starvation (3-AT), benomyl treatment; stationary phase enriched protein; repressed in alkalinizing medium; rat catheter, Spider biofilm induced
PIS54674.1	BAT21	-0.691	Putative branched chain amino acid aminotransferase; regulated by Gcn4, Gcn2; induced in response to amino acid starvation (3-aminotriazole treatment); early-stage flow model biofilm formation
PIS51624.1	ARO9	-0.711	Aromatic transaminase; Ehrlich fusel oil pathway of aromatic alcohol biosynthesis; Rim101-dependent pH-regulation (alkaline induced); Hap43-induced gene
PIS52260.1	HIS7	-0.715	Putative imidazole glycerol phosphate synthase; histidine biosynthesis; no human/murine homolog; transcription induced by histidine starvation; regulated by Gcn2p and Gcn4p; higher protein level in stationary phase
PIS51998.1	GCV2	-0.719	Glycine decarboxylase P subunit; protein of glycine catabolism; repressed by Efg1; Hog1-induced; induced by Rim101 at acid pH; transcript induced in elevated CO2; stationary phase enriched protein
PIS51116.1	FDH1	-0.813	Formate dehydrogenase; oxidizes formate to CO2; Mig1 regulated; induced by macrophages; fluconazole-repressed; repressed by Efg1 in yeast, not hyphal conditions; stationary phase enriched; rat catheter and Spider biofilm induced

PIS55682.1	ARG83	-0.864	GAL4-like Zn(II)2Cys6 transcription factor; clade-associated expression; null shows abnormal regulation of invasive colony growth, is unable to utilize proline as a nitrogen source; flow model biofilm induced
PIS52035.1	HOM2	-1.01	Aspartate-semialdehyde dehydrogenase; forms a homodimer; conserved in bacteria, archaea, and fungi but not in mammals; ketoconazole-repressed; protein present in exponential and stationary growth phase yeast cultures; GlcNAc-induced protein
PIS56901.1	THR1	-1.02	Putative homoserine kinase; regulated by Tup1; amphotericin B repressed; regulated by Gcn2 and Gcn4; Spider biofilm repressed
PIS49747.1	GLY1	-1.04	L-threonine aldolase; complements glycine auxotrophy of S. cerevisiae shm1 shm2 gly1-1 triple mutant; macrophage/pseudohyphal-induced; the GLY1 locus has an RFLP and is triploid in strain SGY269; flow model biofilm induced
PIS51135.1	MET2	-1.07	Homoserine acetyltransferase; Hap43p-, Gcn4p-regulated; macrophage/pseudohyphal-repressed; not highly biofilm induced, in contrast to many sulfur amino acid metabolic genes; no human or murine homolog; virulence-group-correlated expression
PIS54690.1	CPA2	-1.1	Putative arginine-specific carbamoylphosphate synthetase; protein enriched in stationary phase yeast cultures; rat catheter biofilm induced; Spider biofilm induced
PIS51875.1	DAL81	-1.11	Zn(II)2Cys6 transcription factor; ortholog of S. cerevisiae Dal81, involved in the regulation of nitrogen-degradation genes; required for yeast cell adherence to silicone substrate; Spider biofilm induced
PIS59002.1	ARO2	-1.15	Putative chorismate synthase; fungal-specific (no human or murine homolog); protein level decreased in stationary phase yeast cultures; GlcNAc-induced protein
PIS48531.1	SHM2	-1.17	Cytoplasmic serine hydroxymethyltransferase; complements glycine auxotrophy of S. cerevisiae shm1 shm2 gly1-1 mutant; antigenic; farnesol-upregulated in biofilm; stationary-phase enriched protein; rat catheter and Spider biofilm repressed
PIS56737.1	LEU4	-1.24	Putative 2-isopropylmalate synthase; involved in resistance to caspofungin and anidulafungin; regulated by NRG1, MIG1, TUP1, GCN4; induced by human whole blood or PMNs; macrophage/pseudohyphal-repressed after 16h; Spider biofilm repressed
PIS50631.1	ILV6	-1.27	Putative regulatory subunit of acetolacetate synthase; alkaline induced; regulated by Gcn2 and Gcn4; protein present in exponential and stationary growth phase yeast; Spider biofilm repressed
PIS54575.1	MET3	-1.65	ATP sulfurlyase; sulfate assimilation; repressed by Met, Cys, Sfu1, or in fluconazole-resistant isolate; Hog1, caspofungin, white phase-induced; induced on biofilm formation, even in presence of Met and Cys; Spider, F-12/CO2 biofilm induced
PIS52091.1	CDG1	-2.29	Putative cysteine dioxygenases; role in conversion of cysteine to sulfite; transcript regulated upon white-opaque switch; rat catheter, Spider and flow model biofilm induced
PIS54968.1	ARG4	-4.25	Argininosuccinate lyase, catalyzes the final step in the arginine biosynthesis pathway; alkaline downregulated; flow model biofilm induced; Spider biofilm induced
**carbohydrate metabolic**			
PIS51798.1	ATC1	2.17	Cell wall acid trehalase; catalyzes hydrolysis of the disaccharide trehalose; similar to S. cerevisiae vacuolar acid trehalase (Ath1p); Hap43p-repressed gene
PIS51654.1	XOG1	1.79	Exo-1,3-beta-glucanase; 5 glycosyl hydrolase family member; affects sensitivity to chitin and glucan synthesis inhibitors; not required for yeast-to-hypha transition or for virulence in mice; Hap43-induced; Spider biofilm induced
PIS48387.1	CHS2	1.57	Chitin synthase; nonessential; required for wild-type chitin deposition in hyphae; transcript regulated during dimorphic transition; Chs1 and Chs2, but not Chs3, are inhibited by the protoberberine HWY-289; flow model biofilm repressed
PIS52073.1	GPD2	1.4	Surface protein similar to glycerol 3-P dehydrogenase; binds host Factor H, FHL-1, plasminogen; regulated by Ssn6, Nrg1, Efg1; induced by cell wall regeneration, macrophage/pseudohyphal growth, core stress response; Spider biofilm induced
PIS48287.1	CHS4	1.36	Activator of Chs3p chitin synthase; required for wild-type wall chitin content, but not for hyphal growth; mutant resistant to Calcofluor white; prenylation and 2 transmembrane segments predicted; functional homolog of S. cerevisiae Chs4p
PIS58420.1	PHR1	1.21	Cell surface glycosidase; may act on cell-wall beta-1,3-glucan prior to beta-1,6-glucan linkage; role in systemic, not vaginal virulence (neutral, not low pH); high pH or filamentation induced; Bcr1-repressed in RPMI a/a biofilm
PIS50550.1	RHR2	1.18	Glycerol 3-phosphatase; roles in osmotic tolerance, glycerol accumulation in response to salt; Spider/flow model biofilm induced; regulated by macrophage, stress, yeast-hyphal switch, pheromone, Gcn4, Hog1, Nrg1, Tup1
PIS51188.1	INO1	1.15	Inositol-1-phosphate synthase; antigenic in human; repressed by farnesol in biofilm or by caspofungin; upstream inositol/choline regulatory element; glycosylation predicted; rat catheter, flow model induced; Spider biofilm repressed
PIS58949.1	KRE5	0.967	UDP-glucose:glycoprotein glucosyltransferase; 1,6-beta-D-glucan biosynthesis, hyphal growth, virulence in mouse IV model; partially complements S. cerevisiae kre5 mutant defects; flow biofilm repressed, Bcr1-repressed in RPMI a/a biofilms

PIS58799.1	BIG1	0.878	Endoplasmic reticulum (ER) protein; ortholog of S. cerevisiae Big1; required for beta-1,6-glucan synthesis, filamentation, adhesion, and virulence; rat catheter biofilm repressed
PIS52223.1	NAG1	0.843	Glucosamine-6-phosphate deaminase; required for normal hyphal growth and mouse virulence; converts glucosamine 6-P to fructose 6-P; reversible reaction in vitro; gene and protein is GlcNAc-induced; Spider biofilm induced
PIS55829.1	MNN2	0.815	Alpha-1,2-mannosyltransferase; similar to S. cerevisiae Mnn2; role in cell wall integrity, temperature sensitivity; iron utilization in low iron; Tn mutation affects filamentous growth; filament induced; increased chitin exposes beta-glucan
PIS51318.1	FUN31	0.718	Putative PAS kinase involved in cell wall damage response; similar to S. cerevisiae Psk1p, a putative serine/threonine protein kinase; induced by Mnl1 under weak acid stress; rat catheter and Spider biofilm induced
PIS51187.1	CHT3	0.679	Major chitinase; secreted; functional homolog of S. cerevisiae Cts1p; 4 N-glycosylation motifs; possible O-mannosylation; putative signal peptide; hyphal-repressed; farnesol upregulated in biofilm; regulated by Efg1p, Cyr1p, Ras1p
PIS51272.1	FOX2	0.652	3-hydroxyacyl-CoA epimerase; fatty acid beta-oxidation; induced by phagocytosis; regulated by Mig1, by white-opaque switch, by DNA methylation; transcriptional activation by oleate requires Ctf1; rat catheter and Spider biofilm induced
PIS55033.1	CHT4	0.618	Chitinase; similar to S. cerevisiae sporulation-specific Cts2p; functionally complements A. gossypii cts2 mutant sporulation defect; homozygous null mutation causes no obvious defects; transcription decreases upon yeast-to-hyphal switch
PIS54786.1	HXK2	0.589	Hexokinase II; antigenic in humans; repressed by human neutrophils; Efg1-regulated; fluconazole-induced; gene regulation by Ssn6; present in exponential and stationary growth phase; flow model biofilm induced; Spider biofilm repressed
PIS51679.1	GPH1	-0.587	Putative glycogen phosphorylase; role in glycogen metabolism; regulated by Ssk1, Mig1, Tup1, Hap43; fluconazole-induced; localizes to cell surface of hyphae, not yeast; stationary phase enriched protein; Spider biofilm induced
PIS52329.1	RBK1	-0.593	Has domain(s) with predicted kinase activity, ribokinase activity and role in D-ribose metabolic process
PIS56872.1	BGL2	-0.602	Cell wall 1,3-beta-glucosyltransferase; mutant has cell-wall and growth defects, but wild-type 1,3- or 1,6-beta-glucan content; antigenic; virulence role in mouse systemic infection; rat catheter biofilm induced
PIS51134.1	UGT51C1	-0.62	UDP-glucose:sterol glucosyltransferase; enzyme of sterol glucoside (membrane-bound lipid) biosynthesis; has UDP-sugar binding domain; activity is UDP-glucose-specific in vitro; enzyme does not use UDP-mannose; Mig1-regulated
PIS59039.1	INP51	-0.648	Putative phosphatidylinositol-4,5-bisphosphate phosphatase; involved in maintenance of phosphoinositide levels; affects hyphal growth, virulence, cell integrity; interacts with Irs4p
PIS51081.1	CIT1	-0.677	Citrate synthase; induced by phagocytosis; induced in high iron; Hog1-repressed; Efg1-regulated under yeast, not hyphal growth conditions; present in exponential and stationary phase; Spider biofilm repressed; rat catheter biofilm induced
PIS51848.1	GPD1	-0.685	Glycerol-3-phosphate dehydrogenase; glycerol biosynthesis; regulated by Efg1; regulated by Tsa1, Tsa1B under H2O2 stress conditions; Sflow model and Spider biofilm induced
PIS58820.1	ENO1	-0.692	Enolase, involved in glycolysis and gluconeogenesis; also has transglutaminase activity involved in assembly of cell wall polysaccharides; major cell-surface antigen; binds host plasmin/plasminogen; immunoprotective; may be essential
PIS55683.1	RGT1	-0.702	Zn(II)2Cys6 transcription factor; transcriptional repressor involved in the regulation of glucose transporter genes; ortholog of S. cerevisiae Rgt1; mutants display decreased colonization of mouse kidneys
PIS52399.1	GCA1	-0.736	Extracellular/plasma membrane-associated glucoamylase; expressed in rat oral infection; regulated by carbohydrates, pH, galactose; promotes biofilm matrix formation; flow model biofilm induced; Bcr1 repressed in RPMI a/a biofilms
PIS55064.1	CGR1	-0.837	Negative regulator of yeast-form growth; HSP70 family member; induced by growth cessation at yeast-hyphal transition or in planktonic growth; physically interacts with Msi3p; similar to rat anti-aging gene, SMP30, stationary phase enriched
PIS48243.1	PFK2	-0.837	Phosphofructokinase beta subunit; fructose 2,6-bisphosphate, AMP activated; ATP inhibited; phagocytosis, hyphal repressed; fluconazole-induced; stationary-phase enriched; flow model biofilm induced; rat catheter/Spider biofilm repressed
PIS58314.1	PGI1	-0.963	Glucose-6-phosphate isomerase; enzyme of glycolysis; antigenic; Efg1-regulated; induced upon adherence to polystyrene; repressed by phagocytosis, human neutrophils; flow model biofilm induced; rat catheter and Spider biofilm repressed
PIS58270.1	MDH1-1	-1.02	Predicted malate dehydrogenase precursor; macrophage-induced transcript; protein present in exponential and stationary growth phase yeast cultures; Spider biofilm repressed
PIS58927.1	PHR2	-1.89	Glycosidase; role in vaginal not systemic infection (low pH not neutral); low pH, high iron, fluconazole, Hap43-induced; Rim101-repressed at pH8; rat catheter biofilm induced; Bcr1-repressed in RPMI a/a biofilms

lipid metabolic process

PIS49566.1	FAD2	4.51	Delta-12 fatty acid desaturase, involved in production of linoleic acid, which is a major component of membranes
PIS48277.1	FGR22	2.52	Putative phosphatidylinositol-specific phospholipase C (PI-PLC); predicted type 2 membrane protein; no S. cerevisiae ortholog; role in, and regulated by, filamentation, Hap43p; almost identical to orf19.5797
PIS52465.1	IPT1	1.83	Inositol phosphoryl transferase; catalyzes the synthesis of the most abundant sphingolipid, mannose-(inositol-P)2-ceramide, M(IP)2C, from MIPC; required for wild-type membrane localization of Cdr1; Spider biofilm induced
PIS49670.1	PTH1	1.69	Putative gluconate transport protein; antigenic during human oral infection; possibly an essential gene, disruptants not obtained by UAU1 method
PIS54585.1	AYR1	1.56	Putative oxidoreductase; transcriptionally induced by interaction with macrophage; rat catheter biofilm repressed
PIS55756.1	FAT1	1.5	Predicted enzyme of sphingolipid biosynthesis; upregulated in biofilm
PIS58769.1	PEL1	1.42	Predicted enzyme of mitochondrial phospholipid biosynthesis; rat catheter and flow model biofilm induced
PIS48712.1	DPP3	1.2	Protein similar to S. cerevisiae pyrophosphate phosphatase Dpp1; required for farnesol biosynthesis; repressed by 17-beta-estradiol, ethynyl estradiol; Spider biofilm induced
PIS51188.1	INO1	1.15	Inositol-1-phosphate synthase; antigenic in human; repressed by farnesol in biofilm or by caspofungin; upstream inositol/choline regulatory element; glycosylation predicted; rat catheter, flow model induced; Spider biofilm repressed
PIS58293.1	DES1	1.15	Putative delta-4 sphingolipid desaturase; planktonic growth-induced gene
PIS58808.1	ERG1	1.12	Squalene epoxidase, epoxidation of squalene to 2,3(S)-oxidosqualene; ergosterol biosynthesis; allylamine antifungal drug target; NADH reducing cofactor but S. cerevisiae Erg1 uses NADPH; flow model biofilm induced; Spider biofilm repressed
PIS51435.1	BMT8	0.985	Putative beta-mannosyltransferase, member of a 9-gene family including characterized BMT genes with roles in beta-1,2-mannosylation of cell wall phosphopeptidomannan; transposon insertion in promoter region causes decreased colony wrinkling
PIS48317.1	ERG7	0.924	2,3-epoxysqualene-lanosterol cyclase (lanosterol synthase), conversion of 2,3-oxidosqualene to lanosterol in sterol biosynthesis; fluconazole-induced; possibly essential, disruptants not obtained by UAU1 method; rat catheter biofilm induced
PIS51255.1	AYR2	0.866	Putative NADPH-dependent 1-acyl dihydroxyacetone phosphate reductase; shows colony morphology-related gene regulation by Ssn6p
PIS48342.1	SMP3	0.85	Mannosyltransferase of glycosylphosphatidylinositol (GPI) biosynthesis; catalyzes mannosylation of Man3-GPI precursor; essential for viability; 8-9 transmembrane regions predicted; has HQEXRF motif; functional homolog of S. cerevisiae Smp3p
PIS52022.1	PEX6	0.793	Ortholog(s) have ATPase activity, role in protein import into peroxisome matrix, receptor recycling, protein unfolding and cytosol, peroxisome localization
PIS58282.1	EHT1	0.768	Putative acyl-coenzymeA:ethanol O-acyltransferase; regulated by Sef1, Sfu1, and Hap43; induced by alpha pheromone in SpiderM medium; Spider biofilm induced; promoter bound by Ndt80
PIS51451.1	TAZ1	0.711	Putative lyso-phosphatidylcholine acyltransferase, required for normal phospholipid content of mitochondrial membranes; rat catheter biofilm induced
PIS55454.1	BTS1	0.678	Putative geranylgeranyl diphosphate synthase; repressed by benomyl treatment; Spider biofilm induced
PIS51272.1	FOX2	0.652	3-hydroxyacyl-CoA epimerase; fatty acid beta-oxidation; induced by phagocytosis; regulated by Mig1, by white-opaque switch, by DNA methylation; transcriptional activation by oleate requires Ctf1; rat catheter and Spider biofilm induced
PIS52067.1	ANT1	0.622	Peroxisomal adenine nucleotide transporter; role in beta-oxidation of medium-chain fatty acid and peroxisome proliferation; rat catheter biofilm induced
PIS52430.1	MCD4	0.62	Mannose-ethanolamine phosphotransferase, essential gene involved in GPI anchor biosynthesis
PIS52070.1	MCR1	-0.598	NADH-cytochrome-b5 reductase; soluble in hyphae; alkaline downregulated; farnesol, ketoconazole or flucytosine induced; protein present in exponential and stationary growth phase yeast; YNB biofilm induced; rat catheter biofilm repressed
PIS51233.1	ARE2	-0.599	Acyl CoA:sterol acyltransferase; uses cholesterol and oleoyl-CoA substrates; protoberberine derivative drug inhibits enzyme activity; ketoconazole-induced; Hap43-repressed; flow model biofilm induced; Spider biofilm induced
PIS52119.1	ERG10	-0.619	Acetyl-CoA acetyltransferase; role in ergosterol biosynthesis; soluble in hyphae; changes in protein abundance associated with azole resistance; fluconazole or ketoconazole induced; macrophage-downregulated protein; GlcNAc-induced protein

PIS51134.1	UGT51C1	-0.62	UDP-glucose:sterol glucosyltransferase; enzyme of sterol glucoside (membrane-bound lipid) biosynthesis; has UDP-sugar binding domain; activity is UDP-glucose-specific in vitro; enzyme does not use UDP-mannose; Mig1-regulated
PIS59039.1	INP51	-0.648	Putative phosphatidylinositol-4,5-bisphosphate phosphatase; involved in maintenance of phosphoinositide levels; affects hyphal growth, virulence, cell integrity; interacts with Irs4p
PIS58983.1	SEC14	-0.715	Essential protein; functional homolog of S. cerevisiae Sec14p, a Golgi phosphatidylinositol/phosphatidylcholine transfer protein that regulates choline-phosphate cytidyltransferase and thereby affects secretion; biofilm-regulated
PIS48819.1	ERG12	-0.796	Ortholog(s) have mevalonate kinase activity and role in ergosterol biosynthetic process, farnesyl diphosphate biosynthetic process, mevalonate pathway, isopentenyl diphosphate biosynthetic process, mevalonate pathway
PIS52001.1	OLE1	-0.84	Fatty acid desaturase, essential protein involved in oleic acid synthesis; required for aerobic hyphal growth and chlamydospore formation; subject to hypoxic regulation; fluconazole-induced; caspofungin repressed; Hap43p-induced
PIS50470.1	PSD2	-0.868	Ortholog(s) have phosphatidylserine decarboxylase activity, role in phosphatidylcholine biosynthetic process, phosphatidylethanolamine biosynthetic process and endosome localization
PIS51592.1	TES1	-0.89	Putative acyl-CoA thioesterase
PIS58402.1	ALG8	-0.972	Putative glucosyltransferase involved in cell wall mannan biosynthesis; transcription is elevated in nik1 and sln1 homozygous null mutants, but not in the chk1 null mutant; possibly an essential gene, disruptants not obtained by UAU1 method
PIS58246.1	ECM39	-1.3	Putative mannosyltransferase similar to S. cerevisiae Ecm39p, which has a role in Calcofluor white resistance; predicted Kex2p substrate; has HKEXRF motif
PIS58240.1	GPI13	-1.55	Major facilitator superfamily protein; has phosphodiesterase/nucleotide pyrophosphatase domain; similar to S. cerevisiae Gpi13p, which acts in GPI anchor biosynthesis; Hap43p-induced gene
PIS58079.1	KSR1	-1.71	3-ketosphinganine reductase, catalyzes the second step in phytosphingosine synthesis
PIS51242.1	ERG28	-1.76	Ortholog(s) have protein-macromolecule adaptor activity, role in ergosterol biosynthetic process and endoplasmic reticulum membrane localization
PIS51299.1	CMD1	-2.26	Calmodulin; calmodulin inhibitors cause a defect in hyphal growth; transcript not regulated by yeast-hyphal transition; mutation confers hypersensitivity to cytochalasin D and high concentrations of tunicamycin; gene has intron
**cellular lipid metabolic process**			
PIS49566.1	FAD2	4.51	Delta-12 fatty acid desaturase, involved in production of linoleic acid, which is a major component of membranes
PIS52465.1	IPT1	1.83	Inositol phosphoryl transferase; catalyzes the synthesis of the most abundant sphingolipid, mannose-(inositol-P)2-ceramide, M(IP)2C, from MIPC; required for wild-type membrane localization of Cdr1; Spider biofilm induced
PIS49670.1	PTH1	1.69	Putative gluconate transport protein; antigenic during human oral infection; possibly an essential gene, disruptants not obtained by UAU1 method
PIS54585.1	AYR1	1.56	Putative oxidoreductase; transcriptionally induced by interaction with macrophage; rat catheter biofilm repressed
PIS55756.1	FAT1	1.5	Predicted enzyme of sphingolipid biosynthesis; upregulated in biofilm
PIS58769.1	PEL1	1.42	Predicted enzyme of mitochondrial phospholipid biosynthesis; rat catheter and flow model biofilm induced
PIS48712.1	DPP3	1.2	Protein similar to S. cerevisiae pyrophosphate phosphatase Dpp1; required for farnesol biosynthesis; repressed by 17-beta-estradiol, ethynyl estradiol; Spider biofilm induced
PIS51188.1	INO1	1.15	Inositol-1-phosphate synthase; antigenic in human; repressed by farnesol in biofilm or by caspofungin; upstream inositol/choline regulatory element; glycosylation predicted; rat catheter, flow model induced; Spider biofilm repressed
PIS58293.1	DES1	1.15	Putative delta-4 sphingolipid desaturase; planktonic growth-induced gene
PIS58808.1	ERG1	1.12	Squalene epoxidase, epoxidation of squalene to 2,3(S)-oxidosqualene; ergosterol biosynthesis; allylamine antifungal drug target; NADH reducing cofactor but S. cerevisiae Erg1 uses NADPH; flow model biofilm induced; Spider biofilm repressed

PIS51435.1	BMT8	0.985	Putative beta-mannosyltransferase, member of a 9-gene family including characterized BMT genes with roles in beta-1,2-mannosylation of cell wall phosphopeptidomannan; transposon insertion in promoter region causes decreased colony wrinkling
PIS48317.1	ERG7	0.924	2,3-epoxysqualene-lanosterol cyclase (lanosterol synthase), conversion of 2,3-oxidosqualene to lanosterol in sterol biosynthesis; fluconazole-induced; possibly essential, disruptants not obtained by UAU1 method; rat catheter biofilm induced
PIS51255.1	AYR2	0.866	Putative NADPH-dependent 1-acyl dihydroxyacetone phosphate reductase; shows colony morphology-related gene regulation by Ssn6p
PIS48342.1	SMP3	0.85	Mannosyltransferase of glycosylphosphatidylinositol (GPI) biosynthesis; catalyzes mannosylation of Man3-GPI precursor; essential for viability; 8-9 transmembrane regions predicted; has HQEXRF motif; functional homolog of S. cerevisiae Smp3p
PIS52022.1	PEX6	0.793	Ortholog(s) have ATPase activity, role in protein import into peroxisome matrix, receptor recycling, protein unfolding and cytosol, peroxisome localization
PIS58282.1	EHT1	0.768	Putative acyl-coenzymeA:ethanol O-acyltransferase; regulated by Sef1, Sfu1, and Hap43; induced by alpha pheromone in SpiderM medium; Spider biofilm induced; promoter bound by Ndt80
PIS51451.1	TAZ1	0.711	Putative lyso-phosphatidylcholine acyltransferase, required for normal phospholipid content of mitochondrial membranes; rat catheter biofilm induced
PIS55454.1	BTS1	0.678	Putative geranylgeranyl diphosphate synthase; repressed by benomyl treatment; Spider biofilm induced
PIS51272.1	FOX2	0.652	3-hydroxyacyl-CoA epimerase; fatty acid beta-oxidation; induced by phagocytosis; regulated by Mig1, by white-opaque switch, by DNA methylation; transcriptional activation by oleate requires Ctf1; rat catheter and Spider biofilm induced
PIS52067.1	ANT1	0.622	Peroxisomal adenine nucleotide transporter; role in beta-oxidation of medium-chain fatty acid and peroxisome proliferation; rat catheter biofilm induced
PIS52430.1	MCD4	0.62	Mannose-ethanolamine phosphotransferase, essential gene involved in GPI anchor biosynthesis
PIS52070.1	MCR1	-0.598	NADH-cytochrome-b5 reductase; soluble in hyphae; alkaline downregulated; farnesol, ketoconazole or flucytosine induced; protein present in exponential and stationary growth phase yeast; YNB biofilm induced; rat catheter biofilm repressed
PIS51233.1	ARE2	-0.599	Acyl CoA:sterol acyltransferase; uses cholesterol and oleoyl-CoA substrates; protoberberine derivative drug inhibits enzyme activity; ketoconazole-induced; Hap43-repressed; flow model biofilm induced; Spider biofilm induced
PIS52119.1	ERG10	-0.619	Acetyl-CoA acetyltransferase; role in ergosterol biosynthesis; soluble in hyphae; changes in protein abundance associated with azole resistance; fluconazole or ketoconazole induced; macrophage-downregulated protein; GlcNAc-induced protein
PIS51134.1	UGT51C1	-0.62	UDP-glucose:sterol glucosyltransferase; enzyme of sterol glucoside (membrane-bound lipid) biosynthesis; has UDP-sugar binding domain; activity is UDP-glucose-specific in vitro; enzyme does not use UDP-mannose; Mig1-regulated
PIS59039.1	INP51	-0.648	Putative phosphatidylinositol-4,5-bisphosphate phosphatase; involved in maintenance of phosphoinositide levels; affects hyphal growth, virulence, cell integrity; interacts with Irs4p
PIS58983.1	SEC14	-0.715	Essential protein; functional homolog of S. cerevisiae Sec14p, a Golgi phosphatidylinositol/phosphatidylcholine transfer protein that regulates choline-phosphate cytidylyltransferase and thereby affects secretion; biofilm-regulated
PIS48819.1	ERG12	-0.796	Ortholog(s) have mevalonate kinase activity and role in ergosterol biosynthetic process, farnesyl diphosphate biosynthetic process, mevalonate pathway, isopentenyl diphosphate biosynthetic process, mevalonate pathway
PIS52001.1	OLE1	-0.84	Fatty acid desaturase, essential protein involved in oleic acid synthesis; required for aerobic hyphal growth and chlamydospore formation; subject to hypoxic regulation; fluconazole-induced; caspofungin repressed; Hap43p-induced
PIS50470.1	PSD2	-0.868	Ortholog(s) have phosphatidylserine decarboxylase activity, role in phosphatidylcholine biosynthetic process, phosphatidylethanolamine biosynthetic process and endosome localization
PIS51592.1	TES1	-0.89	Putative acyl-CoA thioesterase
PIS58402.1	ALG8	-0.972	Putative glucosyltransferase involved in cell wall mannan biosynthesis; transcription is elevated in nik1 and sln1 homozygous null mutants, but not in the chk1 null mutant; possibly an essential gene, disruptants not obtained by UAU1 method
PIS58246.1	ECM39	-1.3	Putative mannosyltransferase similar to S. cerevisiae Ecm39p, which has a role in Calcofluor white resistance; predicted Kex2p substrate; has HKEXRF motif
PIS58240.1	GPI13	-1.55	Major facilitator superfamily protein; has phosphodiesterase/nucleotide pyrophosphatase domain; similar to S. cerevisiae Gpi13p, which acts in GPI anchor biosynthesis; Hap43p-induced gene
PIS58079.1	KSR1	-1.71	3-ketosphinganine reductase, catalyzes the second step in phytosphingosine synthesis

PIS51242.1	ERG28	-1.76	Ortholog(s) have protein-macromolecule adaptor activity, role in ergosterol biosynthetic process and endoplasmic reticulum membrane localization
PIS51299.1	CMD1	-2.26	Calmodulin; calmodulin inhibitors cause a defect in hyphal growth; transcript not regulated by yeast-hyphal transition; mutation confers hypersensitivity to cytochalasin D and high concentrations of tunicamycin; gene has intron
**lipid biosynthetic process**			
PIS49566.1	FAD2	4.51	Delta-12 fatty acid desaturase, involved in production of linoleic acid, which is a major component of membranes
PIS52465.1	IPT1	1.83	Inositol phosphoryl transferase; catalyzes the synthesis of the most abundant sphingolipid, mannose-(inositol-P)2-ceramide, M(IP)2C, from MIPC; required for wild-type membrane localization of Cdr1; Spider biofilm induced
PIS49670.1	PTH1	1.69	Putative gluconate transport protein; antigenic during human oral infection; possibly an essential gene, disruptants not obtained by UAU1 method
PIS54585.1	AYR1	1.56	Putative oxidoreductase; transcriptionally induced by interaction with macrophage; rat catheter biofilm repressed
PIS55756.1	FAT1	1.5	Predicted enzyme of sphingolipid biosynthesis; upregulated in biofilm
PIS58769.1	PEL1	1.42	Predicted enzyme of mitochondrial phospholipid biosynthesis; rat catheter and flow model biofilm induced
PIS48712.1	DPP3	1.2	Protein similar to S. cerevisiae pyrophosphate phosphatase Dpp1; required for farnesol biosynthesis; repressed by 17-beta-estradiol, ethynyl estradiol; Spider biofilm induced
PIS51188.1	INO1	1.15	Inositol-1-phosphate synthase; antigenic in human; repressed by farnesol in biofilm or by caspofungin; upstream inositol/choline regulatory element; glycosylation predicted; rat catheter, flow model induced; Spider biofilm repressed
PIS58293.1	DES1	1.15	Putative delta-4 sphingolipid desaturase; planktonic growth-induced gene
PIS58808.1	ERG1	1.12	Squalene epoxidase, epoxidation of squalene to 2,3(S)-oxidosqualene; ergosterol biosynthesis; allylamine antifungal drug target; NADH reducing cofactor but S. cerevisiae Erg1 uses NADPH; flow model biofilm induced; Spider biofilm repressed
PIS51435.1	BMT8	0.985	Putative beta-mannosyltransferase, member of a 9-gene family including characterized BMT genes with roles in beta-1,2-mannosylation of cell wall phosphopeptidomannan; transposon insertion in promoter region causes decreased colony wrinkling
PIS48317.1	ERG7	0.924	2,3-epoxysqualene-lanosterol cyclase (lanosterol synthase), conversion of 2,3-oxidosqualene to lanosterol in sterol biosynthesis; fluconazole-induced; possibly essential, disruptants not obtained by UAU1 method; rat catheter biofilm induced
PIS51255.1	AYR2	0.866	Putative NADPH-dependent 1-acyl dihydroxyacetone phosphate reductase; shows colony morphology-related gene regulation by Ssn6p
PIS48342.1	SMP3	0.85	Mannosyltransferase of glycosylphosphatidylinositol (GPI) biosynthesis; catalyzes mannosylation of Man3-GPI precursor; essential for viability; 8-9 transmembrane regions predicted; has HQEXRF motif; functional homolog of S. cerevisiae Smp3p
PIS55454.1	BTS1	0.678	Putative geranylgeranyl diphosphate synthase; repressed by benomyl treatment; Spider biofilm induced
PIS52430.1	MCD4	0.62	Mannose-ethanolamine phosphotransferase, essential gene involved in GPI anchor biosynthesis
PIS52070.1	MCR1	-0.598	NADH-cytochrome-b5 reductase; soluble in hyphae; alkaline downregulated; farnesol, ketoconazole or flucytosine induced; protein present in exponential and stationary growth phase yeast; YNB biofilm induced; rat catheter biofilm repressed
PIS52119.1	ERG10	-0.619	Acetyl-CoA acetyltransferase; role in ergosterol biosynthesis; soluble in hyphae; changes in protein abundance associated with azole resistance; fluconazole or ketoconazole induced; macrophage-downregulated protein; GlcNAc-induced protein
PIS51134.1	UGT51C1	-0.62	UDP-glucose:sterol glucosyltransferase; enzyme of sterol glucoside (membrane-bound lipid) biosynthesis; has UDP-sugar binding domain; activity is UDP-glucose-specific in vitro; enzyme does not use UDP-mannose; Mig1-regulated
PIS58983.1	SEC14	-0.715	Essential protein; functional homolog of S. cerevisiae Sec14p, a Golgi phosphatidylinositol/phosphatidylcholine transfer protein that regulates choline-phosphate cytidyltransferase and thereby affects secretion; biofilm-regulated
PIS48819.1	ERG12	-0.796	Ortholog(s) have mevalonate kinase activity and role in ergosterol biosynthetic process, farnesyl diphosphate biosynthetic process, mevalonate pathway, isopentenyl diphosphate biosynthetic process, mevalonate pathway

PIS52001.1	OLE1	-0.84	Fatty acid desaturase, essential protein involved in oleic acid synthesis; required for aerobic hyphal growth and chlamydospore formation; subject to hypoxic regulation; fluconazole-induced; caspofungin repressed; Hap43p-induced
PIS50470.1	PSD2	-0.868	Ortholog(s) have phosphatidylserine decarboxylase activity, role in phosphatidylcholine biosynthetic process, phosphatidylethanolamine biosynthetic process and endosome localization
PIS58240.1	GPI13	-1.55	Major facilitator superfamily protein; has phosphodiesterase/nucleotide pyrophosphatase domain; similar to S. cerevisiae Gpi13p, which acts in GPI anchor biosynthesis; Hap43p-induced gene
PIS58079.1	KSR1	-1.71	3-ketosphinganine reductase, catalyzes the second step in phytosphingosine synthesis
PIS51242.1	ERG28	-1.76	Ortholog(s) have protein-macromolecule adaptor activity, role in ergosterol biosynthetic process and endoplasmic reticulum membrane localization
**fatty acid metabolic process**			
PIS49566.1	FAD2	4.51	Delta-12 fatty acid desaturase, involved in production of linoleic acid, which is a major component of membranes
PIS55756.1	FAT1	1.5	Predicted enzyme of sphingolipid biosynthesis; upregulated in biofilm
PIS52022.1	PEX6	0.793	Ortholog(s) have ATPase activity, role in protein import into peroxisome matrix, receptor recycling, protein unfolding and cytosol, peroxisome localization
PIS51272.1	FOX2	0.652	3-hydroxyacyl-CoA epimerase; fatty acid beta-oxidation; induced by phagocytosis; regulated by Mig1, by white-opaque switch, by DNA methylation; transcriptional activation by oleate requires Ctf1; rat catheter and Spider biofilm induced
PIS52067.1	ANT1	0.622	Peroxisomal adenine nucleotide transporter; role in beta-oxidation of medium-chain fatty acid and peroxisome proliferation; rat catheter biofilm induced
PIS52119.1	ERG10	-0.619	Acetyl-CoA acetyltransferase; role in ergosterol biosynthesis; soluble in hyphae; changes in protein abundance associated with azole resistance; fluconazole or ketoconazole induced; macrophage-downregulated protein; GlcNAc-induced protein
PIS52001.1	OLE1	-0.84	Fatty acid desaturase, essential protein involved in oleic acid synthesis; required for aerobic hyphal growth and chlamydospore formation; subject to hypoxic regulation; fluconazole-induced; caspofungin repressed; Hap43p-induced
PIS51592.1	TES1	-0.89	Putative acyl-CoA thioesterase
**filamentous growth of a population of**			
PIS48277.1	FGR22	2.52	Putative phosphatidylinositol-specific phospholipase C (PI-PLC); predicted type 2 membrane protein; no S. cerevisiae ortholog; role in, and regulated by, filamentation, Hap43p; almost identical to orf19.5797
PIS48502.1	VID27	2.12	Protein similar to S. cerevisiae Vid27p; transposon mutation affects filamentous growth; mutation confers hypersensitivity to toxic ergosterol analog; fungal-specific (no human or murine homolog)
PIS52465.1	IPT1	1.83	Inositol phosphoryl transferase; catalyzes the synthesis of the most abundant sphingolipid, mannose-(inositol-P)2-ceramide, M(IP)2C, from MIPC; required for wild-type membrane localization of Cdr1; Spider biofilm induced
PIS52412.1	CLA4	1.24	Ste20p family Ser/Thr kinase required for wild-type filamentous growth, organ colonization and virulence in mouse systemic infection; role in chlamydospore formation; functional homolog of S. cerevisiae Cla4p; mutant caspofungin sensitive
PIS58296.1	RVS167	1.18	SH3-domain- and BAR domain-containing protein involved in endocytosis; null mutant exhibits defects in hyphal growth, virulence, cell wall integrity, and actin patch localization; cosediments with phosphorylated Myo5p
PIS51541.1	YAK1	1.16	Putative dual-specificity tyrosine-phosphorylation regulated kinase; involved in hyphal growth regulation and biofilm formation; appears to act either downstream or in parallel with the RAS/cAMP/PKA pathway
PIS55575.1	CPH2	1.13	Myc-bHLH transcription factor; promotes hyphal growth; directly regulates Tec1 to induce hypha-specific genes; probably homodimeric, phosphorylated; required for colonization of the mouse GI tract; rat catheter and Spider biofilm induced
PIS58808.1	ERG1	1.12	Squalene epoxidase, epoxidation of squalene to 2,3(S)-oxidosqualene; ergosterol biosynthesis; allylamine antifungal drug target; NADH reducing cofactor but S. cerevisiae Erg1 uses NADPH; flow model biofilm induced; Spider biofilm repressed

PIS55481.1	PTC1	1.09	Putative protein phosphatase of the Type 2C-related family (serine/threonine-specific), similar to S. cerevisiae Ptc1p; mutant shows virulence defect
PIS52013.1	DCK1	1.06	Putative guanine nucleotide exchange factor; required for embedded filamentous growth; activates Rac1; has a DOCKER domain; similar to adjacent DCK2 and to S. cerevisiae Ylr422wp; regulated by Nrg1; Spider biofilm induced
PIS56749.1	FGR15	0.968	Putative transcription factor with zinc finger DNA-binding motif; lacks an ortholog in S. cerevisiae; transposon mutation affects filamentous growth; caspofungin induced; mutation causes marginal increase in caspofungin sensitivity
PIS58949.1	KRE5	0.967	UDP-glucose:glycoprotein glucosyltransferase; 1,6-beta-D-glucan biosynthesis, hyphal growth, virulence in mouse IV model; partially complements S. cerevisiae kre5 mutant defects; flow biofilm repressed, Bcr1-repressed in RPMI a/a biofilms
PIS56854.1	TFG1	0.946	Protein similar to S. cerevisiae Tfg1p, which is part of transcription factor TFIIF; transposon mutation affects filamentous growth; possibly an essential gene, disruptants not obtained by UAU1 method
PIS51858.1	SWI1	0.892	Protein involved in transcription regulation; ortholog of S. cerevisiae Swi1p, which is a subunit of the SWI/SNF chromatin remodeling complex; interacts with Snf2p; SWI/SNF complex is essential for hyphal growth and virulence
PIS56654.1	SNF2	0.879	Protein involved in transcriptional regulation; ortholog of S. cerevisiae Snf2p, which is the catalytic subunit of the SWI/SNF chromatin remodeling complex; interacts with Swi1p; SWI/SNF complex is essential for hyphal growth and virulence
PIS52223.1	NAG1	0.843	Glucosamine-6-phosphate deaminase; required for normal hyphal growth and mouse virulence; converts glucosamine 6-P to fructose 6-P; reversible reaction in vitro; gene and protein is GlcNAc-induced; Spider biofilm induced
PIS58898.1	SMC5	0.822	Protein similar to S. cerevisiae Smc5p, which is involved in DNA repair; transposon mutation affects filamentous growth
PIS55829.1	MNN2	0.815	Alpha-1,2-mannosyltransferase; similar to S. cerevisiae Mnn2; role in cell wall integrity, temperature sensitivity; iron utilization in low iron; Tn mutation affects filamentous growth; filament induced; increased chitin exposes beta-glucan
PIS58579.1	ESS1	0.805	Prolyl isomerase (parvulin class); essential; involved in yeast-hyphal switching, Cph1p pathway; has inflexible linker between WW and isomerase domains, unlike human homolog; functional homolog of S. cerevisiae Ess1p
PIS58957.1	CDC24	0.791	GDP-GTP exchange factor for Cdc42p; phosphorylated; required for maintenance of hyphal growth; misexpression blocks hyphal growth and causes avirulence in a mouse model of systemic infection; antigenic during human oral infection
PIS49792.1	TAC1	0.787	Zn(2)-Cys(6) transcriptional activator of drug-responsive genes (CDR1 and CDR2); binds DRE element; gene in zinc cluster region near MTL locus; resequencing indicates that TAC1 spans orf19.3188 and orf19.3189; Spider biofilm induced
PIS51710.1	PEP8	0.773	Protein similar to S. cerevisiae Pep8p, which is involved in retrograde transport; transposon mutation affects filamentous growth
PIS48747.1	SSK1	0.701	Response regulator of two-component system; role in oxidative stress response, cell wall biosynthesis, virulence, hyphal growth on solid media; expressed in hyphae and yeast; peroxisomal targeting sequence (PTS1); Spider biofilm induced
PIS52322.1	SNT1	0.689	Ortholog of S. cerevisiae Snt1; an NAD-independent histone deacetylase; heterozygous transposon mutation affects filamentous growth; null mutants are viable
PIS58732.1	CAS4	0.669	RAM cell wall integrity signaling network protein; cell separation, azole sensitivity; needed for hyphal growth; insertion mutation near 3' end of gene increases caspofungin sensitivity; pheromone/hyphal induced; flow biofilm repressed
PIS51143.1	FGR10	0.65	Putative asparaginase; lacks ortholog in S. cerevisiae; transposon mutation affects filamentous growth; Spider biofilm induced
PIS51313.1	CAT8	0.645	Zn(II)2Cys6 transcription factor; similar to S. cerevisiae Cat8 but mutant phenotype suggests different target genes; mutant displays increased filamentous/invasive growth; flucytosine repressed; rat catheter biofilm induced
PIS58992.1	FGR32	-0.587	Protein similar to S. cerevisiae Swa2p; induced upon adherence to polystyrene; transposon mutation affects filamentous growth; Hap43p-repressed gene
PIS58815.1	CDC42	-0.663	Rho-type GTPase; required for budding and maintenance of hyphal growth; GGTase I geranylgeranylated; misexpression blocks hyphal growth, causes avirulence in mouse IV infection; shows actin-dependent localization to hyphal tip
PIS58820.1	ENO1	-0.692	Enolase, involved in glycolysis and gluconeogenesis; also has transglutaminase activity involved in assembly of cell wall polysaccharides; major cell-surface antigen; binds host plasmin/plasminogen; immunoprotective; may be essential
PIS49835.1	NBN1	-0.701	Subunit of the NuA4 histone acetyltransferase complex; Plc1p-regulated
PIS52353.1	ECM25	-0.713	Non-essential protein involved in cell morphogenesis
PIS50541.1	HOG1	-0.717	MAP kinase of osmotic-, heavy metal-, and core stress response; role in regulation of response to stress; phosphorylated in response to H2O2 or NaCl; acts as repressor of START; mutant induces protective mouse immune response

PIS51784.1	SIT4	-0.729	Serine/threonine protein phosphatase catalytic subunit; has a role in cell wall maintenance, hyphal growth, and virulence in a mouse systemic infection model
PIS48755.1	ASC1	-0.743	40S ribosomal subunit similar to G-beta subunits; glucose or N starvation induced filamentation; required for virulence in mice; snoRNA snR24 encoded in ASC1 intron; repressed in stationary phase; GlcNAc-induced; Spider biofilm repressed
PIS58370.1	VMA7	-0.761	Putative subunit of the V-ATPase complex, which is involved in control of vacuolar pH; highly similar to S. cerevisiae Vma7p; interacts with phosphatidylinositol 3-kinase Vps34p
PIS50478.1	PPR1	-0.8	Transcription factor with zinc cluster DNA-binding motif involved in regulation of purine catabolism; has similarity to S. cerevisiae Ppr1p, which is a transcription factor involved in the regulation of uracil biosynthesis genes
PIS58629.1	IMH3	-0.807	Inosine monophosphate (IMP) dehydrogenase; enzyme of GMP biosynthesis; target of mycophenolic acid and mizoribine monophosphate; antigenic during infection; repressed in core stress response; snoRNA snR54 encoded within IMH3 intron
PIS51180.1	FGR34	-0.821	Protein lacking an ortholog in S. cerevisiae; transposon mutation affects filamentous growth
PIS50470.1	PSD2	-0.868	Ortholog(s) have phosphatidylserine decarboxylase activity, role in phosphatidylcholine biosynthetic process, phosphatidylethanolamine biosynthetic process and endosome localization
PIS49535.1	CPP1	-0.901	VH1 family MAPK phosphatase; regulates Cst20-Hst7-Cek1-Cph1 filamentation pathway; negatively regulates mating, represses yeast-hyphal switch; required for virulence in mice; yeast-enriched; induced by alpha pheromone in SpiderM medium;
PIS56762.1	BRE1	-0.96	Putative transcription factor with C3HC4 zinc finger DNA-binding motif; similar to S. cerevisiae Bre1p; transposon mutation affects filamentous growth
PIS58126.1	ECM33	-1.49	GPI-anchored cell wall protein; mutants show cell-wall defects and reduced adhesion, host cell damage, and endocytosis; mutant infection is immunoprotective in murine model; fluconazole-induced; caspofungin repressed
PIS52258.1	ENP1	-1.5	Protein required for pre-rRNA processing and 40S ribosomal subunit synthesis; associated with U3 and U14 snoRNAs; transposon mutation affects filamentous growth; repressed by prostaglandins; Spider biofilm induced
PIS51078.1	NOT4	-1.58	Putative E3 ubiquitin-protein ligase; required for maintenance, but not induction, of hyphal development; homozygous null mutant is avirulent in mouse systemic infection despite persistence in host; repressed in rat oral candidiasis
PIS58646.1	DYN1	-1.61	Dynein heavy chain; motor protein that moves to microtubule minus end; required for yeast cell separation, spindle positioning, nuclear migration, hyphal growth; regulated by Mig1, Hap43; flow model and rat catheter biofilm repressed
PIS58075.1	WAL1	-1.73	Protein required for hyphal growth and for wild-type cell morphology, polarized budding, endocytosis, vacuole morphology; similar to Wiskott-Aldrich syndrome protein; localizes to cortical actin patches and hyphal tips
**positive regulation of filamentous growth**			
PIS52412.1	CLA4	1.24	Ste20p family Ser/Thr kinase required for wild-type filamentous growth, organ colonization and virulence in mouse systemic infection; role in chlamydospore formation; functional homolog of S. cerevisiae Cla4p; mutant caspofungin sensitive
PIS54764.1	HST7	1.19	MAP kinase kinase involved in mating and hyphal growth signal transduction pathways; phosphorylates Cek1p; wild-type virulence in mouse systemic infection; functional homolog of S. cerevisiae Ste7p; mutants are hypersensitive to caspofungin
PIS55575.1	CPH2	1.13	Myc-bHLH transcription factor; promotes hyphal growth; directly regulates Tec1 to induce hypha-specific genes; probably homodimeric, phosphorylated; required for colonization of the mouse GI tract; rat catheter and Spider biofilm induced
PIS51858.1	SWI1	0.892	Protein involved in transcription regulation; ortholog of S. cerevisiae Swi1p, which is a subunit of the SWI/SNF chromatin remodeling complex; interacts with Snf2p; SWI/SNF complex is essential for hyphal growth and virulence
PIS56654.1	SNF2	0.879	Protein involved in transcriptional regulation; ortholog of S. cerevisiae Snf2p, which is the catalytic subunit of the SWI/SNF chromatin remodeling complex; interacts with Swi1p; SWI/SNF complex is essential for hyphal growth and virulence
PIS58957.1	CDC24	0.791	GDP-GTP exchange factor for Cdc42p; phosphorylated; required for maintenance of hyphal growth; misexpression blocks hyphal growth and causes avirulence in a mouse model of systemic infection; antigenic during human oral infection
PIS49792.1	TAC1	0.787	Zn(2)-Cys(6) transcriptional activator of drug-responsive genes (CDR1 and CDR2); binds DRE element; gene in zinc cluster region near MTL locus; resequencing indicates that TAC1 spans orf19.3188 and orf19.3189; Spider biofilm induced
PIS48747.1	SSK1	0.701	Response regulator of two-component system; role in oxidative stress response, cell wall biosynthesis, virulence, hyphal growth on solid media; expressed in hyphae and yeast; peroxisomal targeting sequence (PTS1); Spider biofilm induced
PIS51110.1	MKC1	0.681	MAP kinase; role in biofilm formation, contact-induced invasive filamentation, systemic virulence in mouse, cell wall structure/maintenance, caspofungin response; phosphorylated on surface contact, membrane perturbation, or cell wall stress

PIS58815.1	CDC42	-0.663	Rho-type GTPase; required for budding and maintenance of hyphal growth; GGTase I geranylgeranylated; misexpression blocks hyphal growth, causes avirulence in mouse IV infection; shows actin-dependent localization to hyphal tip
PIS51784.1	SIT4	-0.729	Serine/threonine protein phosphatase catalytic subunit; has a role in cell wall maintenance, hyphal growth, and virulence in a mouse systemic infection model
PIS50630.1	ZCF27	-0.785	Putative Zn(II)2Cys6 transcription factor
PIS51956.1	MDS3	-0.858	TOR signaling pathway component; required for growth and hyphal formation at alkaline pH, for full virulence in a mouse model of systemic infection and for SD and Spider medium biofilm formation; role in chlamydospore formation
PIS51603.1	RIM20	-1.18	Protein involved in the pH response pathway; binds to the transcription factor Rim101 and may serve as a scaffold to facilitate the C-terminal proteolytic cleavage that activates Rim101; required for alkaline pH-induced hyphal growth
PIS58722.1	CRK1	-3.76	Protein kinase of the Cdc2 subfamily involved in hyphal development, virulence; promotes hyphal development independently of Cph1 and Efg1; functionally complements pheromone hypersensitivity of S. cerevisiae sgv1 mutant; Hap43p-repressed
**negative regulation of filamentous**			
PIS58836.1	HSL1	2.54	Probable protein kinase involved in determination of morphology during the cell cycle of both yeast-form and hyphal cells via regulation of Swe1p and Cdc28p; required for full virulence and kidney colonization in mouse systemic infection
PIS58171.1	SFL1	1.88	Transcription factor involved in negative regulation of morphogenesis, flocculation and virulence; induced in core caspofungin response; Spider biofilm induced
PIS52322.1	SNT1	0.689	Ortholog of S. cerevisiae Snt1; an NAD-independent histone deacetylase; heterozygous transposon mutation affects filamentous growth; null mutants are viable
PIS51313.1	CAT8	0.645	Zn(II)2Cys6 transcription factor; similar to S. cerevisiae Cat8 but mutant phenotype suggests different target genes; mutant displays increased filamentous/invasive growth; flucytosine repressed; rat catheter biofilm induced
PIS51285.1	FKH2	-0.63	Forkhead transcription factor; morphogenesis regulator; required for wild-type hyphal transcription, cell separation, and for virulence in cell culture; mutant lacks true hyphae, is constitutively pseudohyphal; upregulated in RHE model
PIS55683.1	RGT1	-0.702	Zn(II)2Cys6 transcription factor; transcriptional repressor involved in the regulation of glucose transporter genes; ortholog of S. cerevisiae Rgt1; mutants display decreased colonization of mouse kidneys
PIS50541.1	HOG1	-0.717	MAP kinase of osmotic-, heavy metal-, and core stress response; role in regulation of response to stress; phosphorylated in response to H2O2 or NaCl; acts as repressor of START; mutant induces protective mouse immune response
PIS49535.1	CPP1	-0.901	VH1 family MAPK phosphatase; regulates Cst20-Hst7-Cek1-Cph1 filamentation pathway; negatively regulates mating, represses yeast-hyphal switch; required for virulence in mice; yeast-enriched; induced by alpha pheromone in SpiderM medium;
PIS54910.1	HST3	-0.97	Histone H3K56 deacetylase; reduced copy number increases opaque cell formation; repressed by MMS, hydroxyurea and high-levels of hydrogen peroxide; Hap43p-induced; ectopic expression blocks genotoxin-induced switching; nicotinamide target
PIS58355.1	RTT109	-1.02	Histone acetyltransferase, mutants are sensitive to DNA damage, show decreased virulence in mice, decreased white-to-opaque switching and increased susceptibility to killing by macrophages
PIS58722.1	CRK1	-3.76	Protein kinase of the Cdc2 subfamily involved in hyphal development, virulence; promotes hyphal development independently of Cph1 and Efg1; functionally complements pheromone hypersensitivity of S. cerevisiae sgv1 mutant; Hap43p-repressed

Summary of GO enrichment analysis

ID	Term	sum	up	down	Category	Genes
GO:0019538	protein metabolic process	96	39	57	GO	HSL1,VPS15,PTP1,SAP3,MNN12,CLA4,GIS2,MAM33,HST7,NOT3,YAK1,CDC43,BET4,RMT2,KRE5,HLJ1,STE11,GPX3,EPL1,CCH1,SMP3,MNN2,ANP1,ESS1,RIT1,CAK1,RTT101,FUN31,MRP20,PHO23,BET2,RPT4,MKC1,CKA2,DOT4,FGR10,SKP1,MCD4,ANB1,NPL4,RPL43A,RPD31,OCA1,NOP1,RPL10A,RPL4B,RPL12,RMS1,TUF1,PUP1,EIF4E,ALG5,NBN1,HOG1,RPL5,WBP1,PRE6,SIT4,RPL35,SSZ1,ASC1,RPL25,ACT1,PPE1,SWC4,RAD23,CDC23,GCD7,TIP41,RPP0,FUN12,PSD2,ADA2,ERF1,CPP1,PBS2,HMT1,APG7,BRE1,HST3,ALG8,SET1,RTT109,RPL23A,SPC2,PEX12,HCR1,ECM39,MEF2,GPI13,UBA2,NOT4,PUP3,IEM1,AKL1,CRK1
GO:0006520	cellular amino acid metabolic process	38	14	24	GO	ARG3,AFP99,PUT1,LYS22,ARO8,ECM17,HIS3,PAN6,DAO1,GDH3,CAR2,HPD1,MET15,SER1,ARO7,PUT3,STR2,TRP4,ARG1,BAT21,ARO9,HIS7,GCV2,FDH1,ARG83,HOM2,THR1,GLY1,MET2,CPA2,DAL81,ARO2,SHM2,LEU4,ILV6,MET3,CDG1,ARG4
GO:0005975	carbohydrate metabolic process	32	17	15	GO	ATC1,XOG1,CHS2,GPD2,CHS4,PHR1,RHR2,INO1,KRE5,BIG1,NAG1,MNN2,FUN31,CHT3,FOX2,CHT4,HXK2,GPH1,RBK1,BGL2,UGT51C1,INP51,CIT1,GPD1,ENO1,RGT1,GCA1,CGR1,PFK2,PGI1,MDH1-1,PHR2
GO:0006629	lipid metabolic process	38	22	16	GO	FAD2,FGR22,IPT1,PTH1,AYR1,FAT1,PEL1,DPP3,INO1,DES1,ERG1,BMT8,ERG7,AYR2,SMP3,PEX6,EHT1,TAZ1,BTS1,FOX2,ANT1,MCD4,MCR1,ARE2,ERG10,UGT51C1,INP51,SEC14,ERG12,OLE1,PSD2,TES1,ALG8,ECM39,GPI13,KSR1,ERG28,CMD1
GO:0044255	cellular lipid metabolic process	37	21	16	GO	FAD2,IPT1,PTH1,AYR1,FAT1,PEL1,DPP3,INO1,DES1,ERG1,BMT8,ERG7,AYR2,SMP3,PEX6,EHT1,TAZ1,BTS1,FOX2,ANT1,MCD4,MCR1,ARE2,ERG10,UGT51C1,INP51,SEC14,ERG12,OLE1,PSD2,TES1,ALG8,ECM39,GPI13,KSR1,ERG28,CMD1
GO:0008610	lipid biosynthetic process	26	16	10	GO	FAD2,IPT1,PTH1,AYR1,FAT1,PEL1,DPP3,INO1,DES1,ERG1,BMT8,ERG7,AYR2,SMP3,BTS1,MCD4,MCR1,ERG10,UGT51C1,SEC14,ERG12,OLE1,PSD2,GPI13,KSR1,ERG28
GO:0006631	fatty acid metabolic process	8	5	3	GO	FAD2,FAT1,PEX6,FOX2,ANT1,ERG10,OLE1,TES1
GO:0036180	filamentous growth of a population of unicellular organisms in response to biotic stimulus	47	27	20	GO	FGR22,VID27,IPT1,CLA4,RVS167,YAK1,CPH2,ERG1,PTC1,DCK1,FGR15,KRE5,TFG1,SWI1,SNF2,NAG1,SMC5,MNN2,ESS1,CDC24,TAC1,PEP8,SSK1,SNT1,CAS4,FGR10,CAT8,FGR32,CDC42,ENO1,NBN1,ECM25,HOG1,SIT4,ASC1,VMA7,PPR1,IMH3,FGR34,PSD2,CPP1,BRE1,ECM33,ENP1,NOT4,DYN1,WAL1
GO:0090033	positive regulation of filamentous growth	15	9	6	GO	CLA4,HST7,CPH2,SWI1,SNF2,CDC24,TAC1,SSK1,MKC1,CDC42,SIT4,ZCF27,MDS3,RIM20,CRK1
GO:0060258	negative regulation of filamentous growth	11	4	7	GO	HSL1,SFL1,SNT1,CAT8,FKH2,RGT1,HOG1,CPP1,HST3,RTT109,CRK1

Notes:
1.Protein_IDs were according to the Uniprot database.
2.Gene_names and descriptions were according to the CGD database (http://www.candidagenome.org/).
3.Genes were classified according to Gene Ontology (https://www.geneontology.org/).
