# Supplementary material for: A single gene mutation underpins metabolic adaptation and acquisition of filamentous competence in the emerging fungal pathogen Candida auris
Source: PLoS Pathog. 2024 Jul 8;20(7):e1012362. doi: 10.1371/journal.ppat.1012362 (PMC11257696; doi:10.1371/journal.ppat.1012362)
Supplement: S2 Dataset — (PDF) [file ppat.1012362.s010.pdf]

Fatty acid profiles of the *gfc1*Δ mutant grown on YPG medium

Index	Compounds	Class	Molecular Weight	Formula	YPG_WT_1	YPG_WT_2	YPG_WT_3	YPG_*gfc1*Δ_1	YPG_*gfc1*Δ_2	YPG_*gfc1*Δ_3
C6-0	hexanoic acid	lipid	116.16	C6H12O2	0.420656	0.424688557	0.449548387	0.471533469	0.499662602	0.46522283
C8-0	octanoic acid	lipid	144.21	C8H16O2	0.528672	0.574344279	0.64171371	0.643959432	0.620178862	0.700027365
C9-0	nonanoic acid	lipid	158.24	C9H18O2	0.563972	0.592628856	0.91783871	0.802377282	0.722077236	0.864319781
C10-0	decanoic acid	lipid	172.26	C10H20O2	0.2860888	0.326147264	0.426262097	0.438235294	0.368732927	0.40539484
C11-0	hendecanoic acid	lipid	186.29	C11H22O2	0.1889652	0.192097512	0.246181048	0.20371927	0.206900407	0.201417514
C12-0	lauric acid	lipid	200.32	C12H24O2	1.006796	1.00070448	1.37215726	1.51761866	1.36891463	1.34695465
C13-0	tridecanoic acid	lipid	214.34	C13H26O2	8.4848	0.444919403	9.63967742	0.573415822	0.472634146	0.464640344
C14-1	myristoleic acid	lipid	226.35	C14H26O2	1.200476	1.07512836	1.25372581	1.31917647	1.12335366	1.32053557
C14-0	myristic acid	lipid	228.37	C14H28O2	5.6886	5.95160199	7.70947581	10.4821501	7.92227642	8.99882721
C15-0	pentadecanoic acid	lipid	242.4	C15H30O2	2.04576	1.90255522	2.42662903	4.67922921	5.92670732	5.92892885
C16-1	palmitoleic acid	lipid	254.41	C16H30O2	31.0888	27.479005	37.6581855	64.1959432	60.3406504	61.084441
C16-0	palmitic acid	lipid	257.43	C16H32O2	554.876	552.238806	764.322581	879.03854	903.792683	860.926505
C16-2	hexadecanedioic acid	lipid	266.41	C16H30O4	5.35116	5.28318408	5.44955645	5.38117647	5.395	5.34136044
C17-0	heptadecanoic acid	lipid	270.45	C17H34O2	18.32148	20.6593433	23.1376613	71.7732252	164.697154	130.477326
C18-3n3	α-linolenic acid	lipid	278.43	C18H30O2	38.43744	40.7156219	64.2681452	150.049493	111.136992	133.030493
C18-2n6	linoleic acid	lipid	280.45	C18H32O2	435.55	434.60	571.77	1130.87	1014.27	1089.65
C18-1n9	oleic acid	lipid	282.46	C18H34O2	333.01996	319.8948851	432.9272582	524.7361056	737.653049	613.7721661
C18-0	stearic acid	lipid	284.48	C18H36O2	322.392	369.306667	462.862903	539.476673	587.280488	538.659109
C19-1	carboenoic acid	lipid	296.49	C19H36O2	4.13872	5.34340299	5.83580645	6.8064503	16.8288211	11.7528147
C19-0	nonadecylic acid	lipid	298.5	C19H38O2	2.452952	2.58163582	2.96372581	6.28713996	10.3154878	10.5665364
C20-5n3	eicosapentaenoic acid	lipid	302.45	C20H30O2	26.52352	27.0439403	38.3260484	34.9029615	48.5764228	42.4339328
C20-3n3	eicosatrienoic acid	lipid	306.48	C20H34O2	5.64812	5.89508458	6.36112903	6.84965517	6.97853659	6.85813135
C20-2	eicosadienoic acid	lipid	308.5	C20H36O2	5.01112	5.29751244	5.40060484	6.55139959	6.91735772	6.78166536
C20-1	eicosenoic acid	lipid	310.51	C20H38O2	4.29248	4.5401791	5.29806452	6.73634888	7.05191057	7.53455825
C20-0	arachidic acid	lipid	312.53	C20H40O2	6.60284	6.16123383	7.87625	10.1882353	11.337439	11.7512119
C21-0	heneicosanoic acid	lipid	326.56	C21H42O2	2.687544	2.68882388	2.74685887	2.81058418	2.90639024	2.76644644
C22-1n9	erucic acid	lipid	338.57	C22H42O2	5.09392	5.19308903	5.29225806	5.4264503	5.83504065	6.92673964
C22-0	behenic acid	lipid	340.58	C22H44O2	5.6374	5.51267662	5.94129032	6.99983773	7.21833333	7.45238468
C23-0	tricosanoic acid	lipid	354.61	C23H46O2	10.35284	10.4068434	10.4608468	10.5446653	10.6417073	10.2295543
C24-0	lignoceric acid	lipid	368.64	C24H48O2	11.97768	11.6646766	12.5275403	15.5020284	15.9214634	16.1560203

Fatty acid profiles of the *mcu1*Δ mutant grown on YPD medium

Index	Compounds	Class	Molecular Weight	Formula	YPD_WT_1	YPD_WT_2	YPD_WT_3	YPD_*mcu1*Δ_1	YPD_*mcu1*Δ_2	YPD_*mcu1*Δ_3
C6-0	hexanoic acid	lipid	116.16	C6H12O2	0.314533443	0.369336519	0.430695297	0.294672957	0.29908303	0.298231828
C9-0	nonanoic acid	lipid	158.24	C9H18O2	0.348502477	0.211553963	0.234707157	0.187580406	0.400147833	0.39374685
C10-0	decanoic acid	lipid	172.26	C10H20O2	0.262061519	0.265981681	0.268166871	0.305129492	0.308123937	0.315246753
C11-0	hendecanoic acid	lipid	186.29	C11H22O2	0.203117671	0.21357507	0.203213497	0.216783377	0.232402187	0.212532274
C12-0	lauric acid	lipid	200.32	C12H24O2	0.971610239	1.05449223	0.995222904	2.35876732	1.87887404	2.1596666
C13-0	tridecanoic acid	lipid	214.34	C13H26O2	0.261010735	0.259930705	0.284448262	0.266176671	0.281872418	0.256476449
C14-1	myristoleic acid	lipid	226.35	C14H26O2	0.909066887	0.82731581	0.980519427	0.802706284	0.911603888	1.01003683
C14-0	myristic acid	lipid	228.37	C14H28O2	4.96085879	5.72532855	5.56396728	6.56534832	6.39959498	6.76557472
C15-0	pentadecanoic acid	lipid	242.4	C15H30O2	0.872981007	0.931596973	0.947231084	1.09543064	1.18607128	1.15293274
C16-1	palmitoleic acid	lipid	254.41	C16H30O2	10.7023947	15.2909598	13.1716564	11.1509737	10.2799109	9.91401434
C16-0	palmitic acid	lipid	257.43	C16H32O2	269.829067	257.779371	277.919836	265.654286	279.474281	270.597015
C17-0	heptadecanoic acid	lipid	270.45	C17H34O2	1.67604046	1.80649144	1.92787321	2.06314796	2.16838801	2.18812561
C18-3n3	α-linolenic acid	lipid	278.43	C18H30O2	6.86841453	10.1229391	9.03758691	11.1735796	11.8332118	11.4114363
C18-2n6	linoleic acid	lipid	280.45	C18H32O2	42.4546656	61.3226603	55.0082617	30.37362374	31.67878495	31.55666218
C18-1n9	oleic acid	lipid	282.46	C18H34O2	63.3503716	89.7099562	85.23227	33.20136521	36.49813687	34.468618
C18-0	stearic acid	lipid	284.48	C18H36O2	174.743187	170.376344	181.907975	175.119454	185.942082	177.324675
C19-1	carboenoic acid	lipid	296.49	C19H36O2	4.51403799	4.88422939	4.68846626	5.22613933	5.26132037	4.81957744
C19-0	nonadecylic acid	lipid	298.5	C19H38O2	1.84060281	1.77243329	1.82997137	1.78522787	1.82212637	1.7339213
C20-3n3	eicosatrienoic acid	lipid	306.48	C20H34O2	N/A	N/A	N/A	2.17088135	2.24256379	2.07349874
C20-2	eicosadienoic acid	lipid	308.5	C20H36O2	N/A	N/A	N/A	2.32241317	2.36209802	2.25349486
C20-1	eicosenoic acid	lipid	310.51	C20H38O2	2.11007845	2.26489048	2.1806544	2.22703473	2.37503443	2.33355301
C20-0	arachidic acid	lipid	312.53	C20H40O2	3.9126796	3.84101553	4.20331288	4.59080506	4.4163629	4.94316728
C22-1n9	erucic acid	lipid	338.57	C22H42O2	4.86936416	5.03209877	4.74813906	5.38956033	4.43406237	4.86729986
C22-0	behenic acid	lipid	340.58	C22H44O2	5.03418662	4.94747113	5.18895706	5.66464565	5.39874443	5.53405699
C24-0	lignoceric acid	lipid	368.64	C24H48O2	10.3864575	10.3856631	10.701227	11.0853242	10.6294451	10.6380306
